# ATF6 activation alters colonic lipid metabolism causing tumour-associated microbial adaptation

**DOI:** 10.1038/s42255-025-01350-6

**Published:** 2025-09-01

**Authors:** Olivia I. Coleman, Adam Sorbie, Alessandra Riva, Miriam von Stern, Stephanie Kuhls, Denise M. Selegato, Luisa Siegert, Isabel Keidel, Nikolai Köhler, Jakob Wirbel, Tim Kacprowski, Andreas Dunkel, Josch K. Pauling, Johannes Plagge, Diego Mediel-Cuadra, Sophia J. Wagner, Ines Chadly, Sandra Bierwith, Tingying Peng, Thomas Metzler, Xin Li, Mathias Heikenwälder, Clemens Schafmayer, Sebastian Hinz, Christian Röder, Christoph Röcken, Michael Zimmermann, Philip Rosenstiel, Katja Steiger, Moritz Jesinghaus, Gerhard Liebisch, Josef Ecker, Christina Schmidt, Georg Zeller, Klaus-Peter Janssen, Dirk Haller

**Affiliations:** 1 Chair of Nutrition and Immunology, School of Life Sciences, Technische Universität München, Freising-Weihenstephan, Munich, Germany; 2 Institute for Stroke and Dementia Research (ISD), University Hospital LMU Munich, Munich, Germany; 3 Molecular Systems Biology Unit, European Molecular Biology Laboratory, Heidelberg, Germany; 4 LipiTUM, Chair of Experimental Bioinformatics, School of Life Sciences Weihenstephan, Technische Universität München, Freising-Weihenstephan, Munich, Germany; 5 Structural and Computational Biology Unit, European Molecular Biology Laboratory, Heidelberg, Germany; 6 Division Data Science in Biomedicine, Peter L. Reichertz Institute for Medical Informatics of TU Braunschweig and Hannover Medical School, Braunschweig, Germany; 7 Braunschweig Integrated Centre of Systems Biology (BRICS), TU Braunschweig, Braunschweig, Germany; 8 Department of Computational Biology of Infection Research, Helmholtz Centre for Infection Research (HZI), Braunschweig, Germany; 9 Chair of Food Chemistry and Molecular Sensory Science, School of Life Sciences, Technische Universität München, München, Germany; 10 CIOBio, Institute of Clinical Chemistry and Laboratory Medicine, University Hospital and Faculty of Medicine Carl Gustav Carus, Dresden University of Technology, Dresden, Germany; 11 ZIEL Institute for Food & Health, Research Group Lipid Metabolism, Technische Universität München, Freising, Germany; 12 Helmholtz AI, Helmholtz Munich, Neuherberg, Germany; 13 Comparative Experimental Pathology (CEP), School of Medicine and Health, Technische Universität München, Munich, Germany; 14 Division of Chronic Inflammation and Cancer, German Cancer Research Center (DKFZ), Heidelberg, Germany; 15 Faculty of Biosciences, Heidelberg University, Heidelberg, Germany; 16 Faculty of Medicine, M3 Research Center, University Tübingen, Tübingen, Germany; 17 Cluster of Excellence iFIT (EXC 2180) ‘Image-Guided and Functionally Instructed Tumor Therapies’, University of Tübingen, Tübingen, Germany; 18 Department of General Surgery, University Hospital Rostock, Rostock, Germany; 19 Institute for Experimental Cancer Research, University Medical Center Schleswig-Holstein, Kiel, Germany; 20 Department of Pathology, Christian-Albrechts University, Kiel, Germany; 21 Institute of Clinical Molecular Biology, University hospital Schleswig-Holstein, Campus Kiel and Kiel University, Kiel, Germany; 22 Institute of Pathology, Universitätsklinikum Marburg, Baldingerstraße, Marburg, Germany; 23 Institute of Clinical Chemistry and Laboratory Medicine, University Hospital Regensburg, Regensburg, Germany; 24 Faculty of Medicine and Heidelberg University Hospital, Institute for Computational Biomedicine, Heidelberg University, Heidelberg, Germany; 25 Department of Surgery, Klinikum rechts der Isar, Technische Universität München, München, Germany; 26 ZIEL Institute for Food and Health, Technische Universität München, Freising, Germany

**Keywords:** Cancer metabolism, Endoplasmic reticulum, Microbiome, Lipids, Metabolism

## Abstract

Endoplasmic reticulum unfolded protein responses contribute to cancer development, with activating transcription factor 6 (ATF6) involved in microbiota-dependent tumorigenesis. Here we show the clinical relevance of ATF6 in individuals with early-onset and late colorectal cancer, and link ATF6 signalling to changes in lipid metabolism and intestinal microbiota. Transcriptional analysis in intestinal epithelial cells of ATF6 transgenic mice (nATF6^IEC^) identifies bacteria-specific changes in cellular metabolism enriched for fatty acid biosynthesis. Untargeted metabolomics and isotype labelling confirm ATF6-related enrichment of long-chain fatty acids in colonic tissue of humans, mice and organoids. FASN inhibition and microbiota transfer in germ-free nATF6^IEC^ mice confirm the causal involvement of ATF6-induced lipid alterations in tumorigenesis. The selective expansion of tumour-relevant microbial taxa, including *Desulfovibrio fairfieldensis*, is mechanistically linked to long-chain fatty acid exposure using bioorthogonal non-canonical amino acid tagging, and growth analysis of *Desulfovibrio* isolates. We postulate chronic ATF6 signalling to select for tumour-promoting microbiota by altering lipid metabolism.

## Abstract (web summary)

The transcription factor ATF6 causes an enrichment in long-chain fatty acids in the colonic epithelium, which leads to changes in the gut microbiota and contributes to the development of colorectal cancer in humans and mice, thereby linking endoplasmic reticulum stress responses to lipid metabolism and tumorigenesis.

## Main

The endoplasmic reticulum (ER) in mammalian cells forms an extensive tubular reticular network that acts as a gatekeeper controlling the synthesis and folding of proteins, and the synthesis of cellular lipids^1,2^. The accumulation of unfolded and misfolded ER proteins activates the unfolded protein response (UPR^ER^), a highly conserved group of intricately regulated signalling pathways that aim to restore ER homeostasis. PKR-like ER kinase, inositol-requiring enzyme 1 and ATF6 constitute the three main arms of the UPR^ER^. ER stress results in the dissociation of membrane-bound ATF6 complexes from the ER chaperone glucose-regulating protein 78 (GRP78), subsequent cleavage by the site-1 and site-2 proteases at the Golgi apparatus and translocation of the p50 active cytosolic N-terminal portion of the transcription factor (nATF6) into the nucleus to bind the ER stress response element.

Numerous studies recognize the UPR^ER^ as a fundamental mediator in cellular physiology and the pathogenesis of inflammatory disorders, metabolic diseases and cancer, including colorectal cancer (CRC)^3–5^. Enhanced expression of GRP78, a downstream target of nATF6, correlates with growth, invasion and metastasis of tumours^6^. Furthermore, *ATF6* mRNA expression and polymorphisms are associated with CRC metastasis and relapse^7,8^, and hepatocellular carcinoma^9^, respectively. We could show that chronic transgenic expression of nATF6 specifically in intestinal epithelial cells (IECs) induces spontaneous and microbiota-dependent colon adenoma formation in a mouse model of early-onset CRC (nATF6^IEC^)^10^. Accumulating evidence unarguably renders the intestinal microbiota as an important regulator of host health and a causal player in the development and progression of CRC. Our findings that dysbiosis precedes tumorigenesis in nATF6^IEC^ mice and that nATF6-expressing germ-free (GF) mice remain tumour-free^10^, clearly indicate a mechanistic link between ATF6 signalling and the intestinal microbiota in the context of colon tumorigenesis. What remains to be elucidated, is how ATF6 signalling induces dysbiosis at the pre-tumour stage.

The multifaceted possibilities of physiological outcomes in response to the UPR^ER^ underlines the necessity to understand the mechanistic contribution of UPR^ER^ signalling to disease pathology. Here we firstly set out to identify the relevance of ATF6 in CRC human populations, and secondly to understand the consequence of ATF6 signalling in the mouse. We characterize ATF6 expression in multiple independent German CRC cohorts and causally link chronic ATF6 activity to altered lipid metabolism and tumour-relevant microbiota changes.

## Results

### High ATF6 expression defines a subset of individuals with CRC

**Fig. 1:**
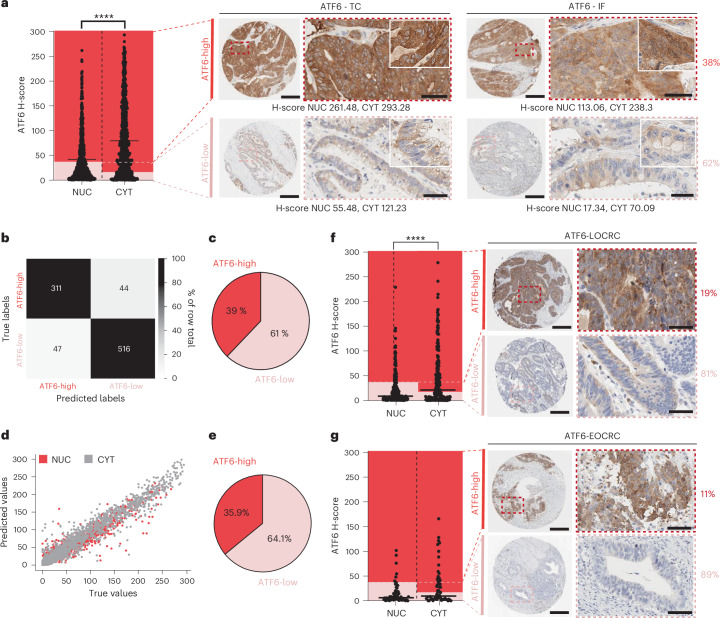
High ATF6 expression defines a subset of individuals with CRC. **a**, QuPath-quantified ATF6 H-score of NUC and CYT ATF6 expression in immunohistochemically stained CRC human tissue samples (cohort 1, *n* = 959 individuals). Significance was calculated using the two-tailed unpaired Wilcoxon test (*P* < 0.0001); indicated is the mean. Representative images of ATF6-high (*n* = 364 individuals) and ATF6-low (*n* = 595 individuals) staining in the tumour centre (TC) and the invasive front (IF). Percentage shows subpopulation of individuals with CRC in each ATF6-stained category (ATF6-low, light red; ATF6-high, dark red). Scale bars, 300 µm (overview) and 50 µm (zoom-in). **b**, Confusion matrix depicting the classification model of ATF6 H-scores quantified using QuPath (true labels) versus ATF6 H-scores predicted by the classification algorithm (predicted labels) for ATF6-high and ATF6-low individuals with CRC. **c**, Pie chart showing percentage of ATF6-high and ATF6-low individuals with CRC identified in CRC cohort 2 (*n* = 50 individuals) using the classification model. **d**, Regression model of ATF6 H-scores quantified using QuPath (true values) versus ATF6 H-scores predicted by the regression algorithm (predicted values) for NUC and CYT ATF6 expression. **e**, Pie chart showing percentage of ATF6-high and ATF6-low individuals with CRC identified in CRC cohort 2 (*n* = 50 individuals) using the regression model. **f**, QuPath-quantified ATF6 H-score of NUC and CYT ATF6 expression in immunohistochemically stained CRC human tissue samples (cohort 3, LOCRC, *n* = 256 individuals). Significance was calculated using the two-tailed unpaired Wilcoxon test (*P* < 0.0001); indicated is the mean. Representative images of ATF6-high (*n* = 49 individuals) and ATF6-low (*n* = 207 individuals) staining. Percentage shows subpopulation of individuals with CRC in each ATF6-stained category. Scale bars, 300 µm (overview) and 50 µm (zoom-in). **g**, QuPath-quantified ATF6 H-score of NUC and CYT ATF6 expression in immunohistochemically stained CRC human tissue samples (cohort 3, EOCRC, *n* = 55 individuals). Significance was calculated using the two-tailed unpaired Wilcoxon test (*P* = 0.0876), indicated is the mean. Representative images of ATF6-high (*n* = 6 individuals) and ATF6-low (*n* = 49 individuals) staining. Percentage shows subpopulation of individuals with CRC in each ATF6-stained category. Scale bars, 300 µm (overview), 50 µm (zoom-in). *P < 0.05, **P < 0.01, ***P < 0.001, ****P < 0.0001. Source data

**Extended Data Fig. 1:**
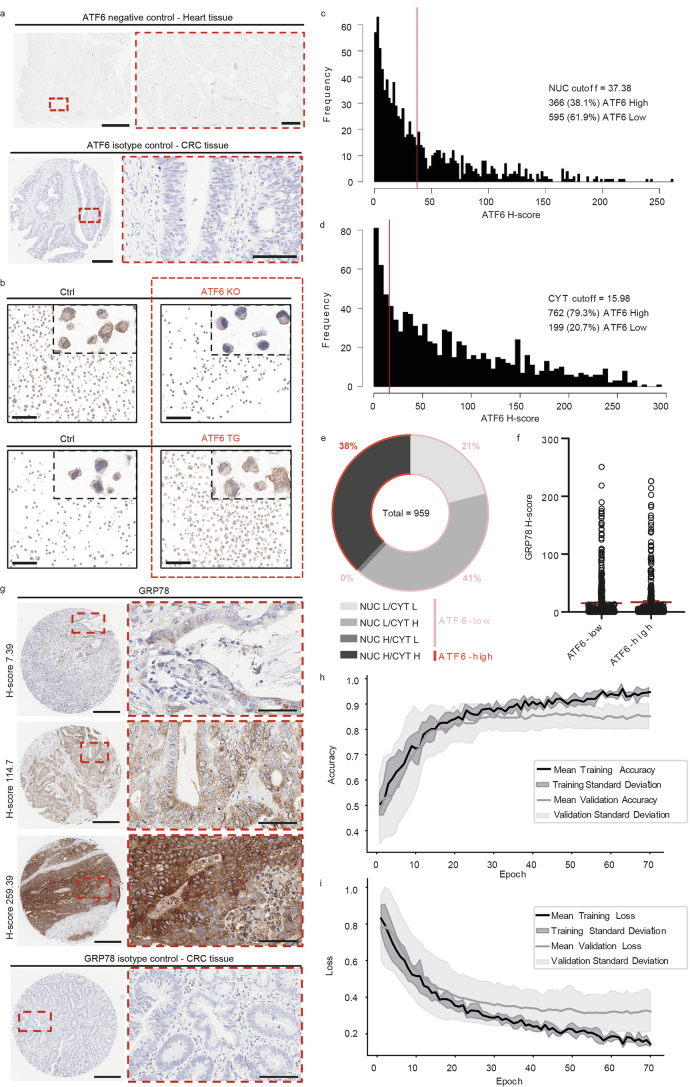
ATF6 and GRP78 analyses in human CRC patients. **a**, Representative images of ATF6 antibody staining in heart tissue (top, n = 1 patient) and the isotype control in CRC patient tissue (bottom, n = 170 patients). Scale bars: 300 µm overview, 50 µm zoom-in. **b**, Representative images of ATF6 antibody staining in the Colo800 cell lines, including plasmid-only controls (Ctrl, px459 for *Atf6* KO and MLG for *Atf6* TG), *Atf6* KO and *Atf6* TG. Scale bars: 100 µm. **c**, Nuclear (NUC) cutoff for the ATF6 H-score as determined using the online *Cutoff Finder* Web Application for Biomarker cutoff optimization, dividing patients into ATF6-high and ATF6-low. **d**, Cytoplasmic (CYT) cutoff for the ATF6 H-score as determined using the online *Cutoff Finder* Web Application for Biomarker cutoff optimization, dividing patients into ATF6-high and ATF6-low. **e**, Donut chart depicting the percentage of patients with all observed combinations of nuclear and cytoplasmic staining (NUC L/CYT L = low nuclear and low cytoplasmic staining, NUC L/CYT H = low nuclear and high cytoplasmic staining, NUC H/CYT L = high nuclear and low cytoplasmic staining, NUC H/CYT H = high nuclear and high cytoplasmic staining). **f**, QuPath quantified GRP78 H-score of ATF6-low and ATF6 high patients in immunohistochemically stained CRC patient tissue samples (cohort 1, n = 959), with group mean denoted by a horizontal red bar. Significance was calculated using the two-tailed unpaired Wilcoxon- test (*p* = 0.3524). **g**, Representative images of GRP78 antibody staining in CRC patient tissue samples (cohort 1, n = 959), stratified according to H-score and isotype control (cohort 1, n = 100). Scale bars: 300 µm overview, 50 µm zoom-in. **h**, Plot showing the classification model training accuracy, displaying the mean training and validation accuracy and their standard deviation. **i**, Plot showing the classification model training loss, displaying the mean training and validation loss and their standard deviation. A significant *p*-value < 0.05 is represented as an asterisk: *p < 0.05, **p < 0.01. ***p < 0.001, ****p < 0.0001. Source data

We previously identified increased *ATF6* expression to be associated with reduced disease-free survival in individuals with CRC^10^. To substantiate these findings, we quantified ATF6 protein expression via immunohistochemistry (IHC) staining in a German CRC human cohort (cohort 1; *n* = 959) using QuPath^11,12^. ATF6 antibody specificity was confirmed through negative staining in human heart muscle, and the appropriate isotype control staining in CRC tissue, as well as in Colo800 ATF6 knockout (KO) and transgenic overexpression (TG) human CRC cell lines (Extended Data Fig. 1a,b). ATF6 expression was compared between the tumour centre (ATF6-TC) and the tumour invasive front (ATF6-IF), showing similar expression levels between these two localizations (data not shown), and subsequently averaged to determine the ATF6 histochemical score (H-score; capturing both the intensity and the percentage of stained cells; Fig. 1a). Findings show that ATF6 upregulation occurs in 38% of individuals with CRC who can be classed as ATF6-high in terms of their nuclear (NUC) and cytoplasmic (CYT) expression (Fig. 1a and Extended Data Fig. 1c,d). Stratification into all possible combinations of NUC and CYT ATF6 staining into low (L) and high (H) shows that all NUC H individuals are also CYT H (38%), with 0% of NUC H individuals being CYT L (Extended Data Fig. 1d). The other combinations of NUC L/CYT L and NUC L/CYT H make up 21% and 41% of the individuals with CRC, respectively (Extended Data Fig. 1e). In the same cohort 1, we assessed the global levels of ER stress through IHC staining of GRP78, the master chaperone of the ER. Interestingly, quantification of GRP78 using QuPath showed that, despite the wide range of GRP78 H-scores among the CRC cohort, there is no difference in GRP78 H-scores between ATF6-high and ATF6-low CRC groups (Extended Data Fig. 1f,g). To validate the above findings, we trained a deep learning model to predict ATF6 expression levels of CRC human biopsy images. The model was trained on an independent CRC human cohort (cohort 2; *n* = 50) with classification and regression objectives using fivefold cross-validation. In line with our results, external testing results identified 39% (classification task) and 35.9% (regression task) of individuals with CRC in cohort 1 as ATF6-high (Fig. 1b–e and Extended Data Fig. 1h,i), confirming the robustness and applicability of our AI methodology. To further support our findings, we quantified ATF6 expression using QuPath in a third German CRC human cohort (cohort 3; *n* = 256). Here we identified a subpopulation of 19% of individuals with CRC to be ATF6-high (Fig. 1f). To investigate a potentially dissimilar effect of ATF6 expression in individuals with early-onset colorectal cancer (age < 50 years; EOCRC) versus late-onset colorectal cancer (age > 50 years; LOCRC), we quantified ATF6 expression in additional individuals from cohort 3 with EOCRC (cohort 3; *n* = 55 EOCRC). Findings show similar expression levels in EOCRC, with 11% of individuals classified as ATF6-high (Fig. 1g). Together, these results clearly demonstrate that high ATF6 expression defines a subpopulation of individuals with CRC (19–38% LOCRC; 11% EOCRC).

### ATF6 alters colonic FA metabolism in the presence of bacteria

**Fig. 2:**
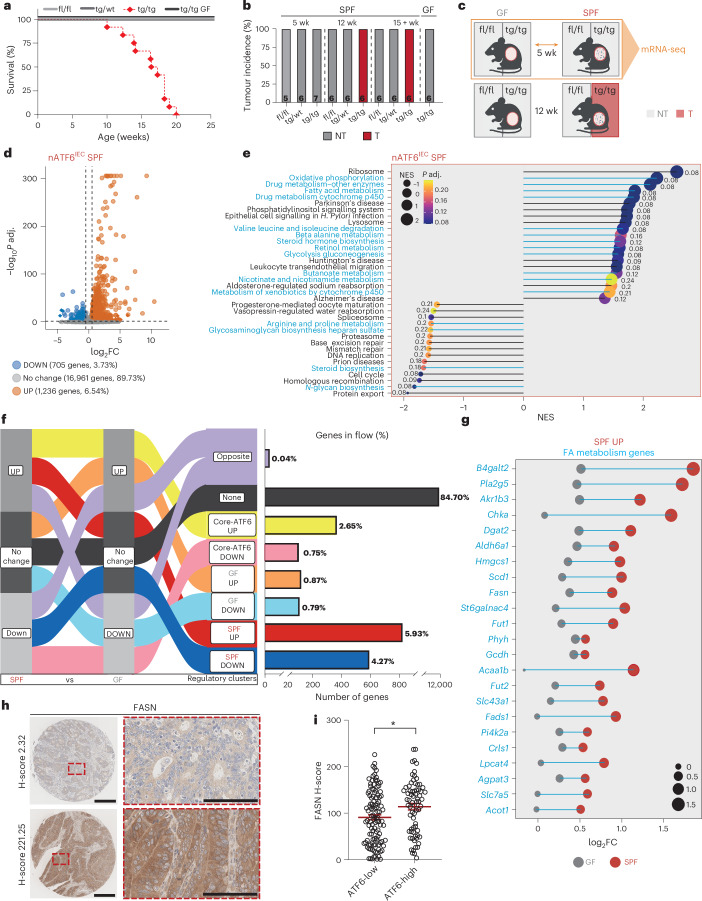
ATF6 alters colonic FA metabolism in the presence of bacteria. **a**, Survival curve showing the percentage survival of fl/fl, tg/wt, tg/tg SPF and tg/tg GF mice between 0 and 25 weeks of age (*n* = 6 fl/fl SPF mice, 7 tg/wt SPF mice, 12 tg/tg SPF mice and 10 GF tg/tg mice). **b**, Tumour incidence (percentage) of SPF fl/fl, tg/wt and tg/tg mice and GF tg/tg mice at the pre-tumour (5 week), tumour (12 week) and late-tumour (15+ week) time points. Grey represents no tumour, and red represents tumour. Number of mice is stated in each column. **c**, Schematic of non-tumour (NT) and tumour (T) phenotypes associated with GF and SPF fl/fl and tg/tg mice, highlighting the genotypes, age and colonization status used for the RNA-seq analyses (orange box and arrow; *n* = 6 mice per group). Created with BioRender.com. **d**, Volcano plot of detected genes in fl/fl versus tg/tg nATF6^IEC^ mice showing the number and percentage of genes that are unchanged (no change), upregulated in tg/tg mice (UP) or downregulated in tg/tg mice (DOWN). Threshold: −0.5 > log_2_FC > 0.5, *P* adj. < 0.05. **e**, Lollipop graph of the top and bottom regulated KEGG pathways in tg/tg versus fl/fl nATF6^IEC^ SPF mice (*P* adj. ≤ 0.25, −1 ≥ NES ≥ 1, with a minimum of 50% of genes detected in the pathway). Metabolic pathways are highlighted in blue. **f**, Alluvial plot depicting the flows used to define SiRCle clusters in a comparison between SPF and GF mice. Each data type (SPF and GF) has been labelled as a column, with one of three states (UP, no change, DOWN) defined for each column based on the results for differential analysis between fl/fl and tg/tg in that data type. Shown are the flows between each data type and state, which define the regulatory clusters (third column). The number and percentage of genes in the flow are depicted next to each regulatory cluster. **g**, Lollipop graph of the FA metabolism-related genes from the SPF UP cluster (blue) shown for GF (grey) and SPF (red) mice. **h**, Representative images of low H-score and high H-score FASN immunohistochemically stained in CRC human tissue samples (cohort 1, *n* = 181 individuals). Scale bars, 300 µm (overview), 50 µm (zoom-in). **i**, QuPath-quantified FASN H-score with individuals (cohort 1, *n* = 181 individuals) grouped into ATF6-low and ATF6-high (according to QuPath-quantified ATF6 H-score). Significance was calculated using the Student’s two-tailed unpaired *t*-test (*P* = 0.0105); indicated is the mean. *P < 0.05, **P < 0.01, ***P < 0.001, ****P < 0.0001. Source data

In our ATF6 transgenic mouse model (nATF6^IEC^), we previously established a causal role of the microbiota in adenoma formation^10^. In agreement with these findings, biallelic nATF6^IEC^ mice (tg/tg) show a reduced survival (Fig. 2a), and develop colonic tumours with an incidence of 100% under specific pathogen-free (SPF) conditions, while controls (fl/fl) and monoallelic (tg/wt) nATF6 expression, as well as GF tg/tg mice, remain tumour-free (Fig. 2b).

**Extended Data Fig. 2:**
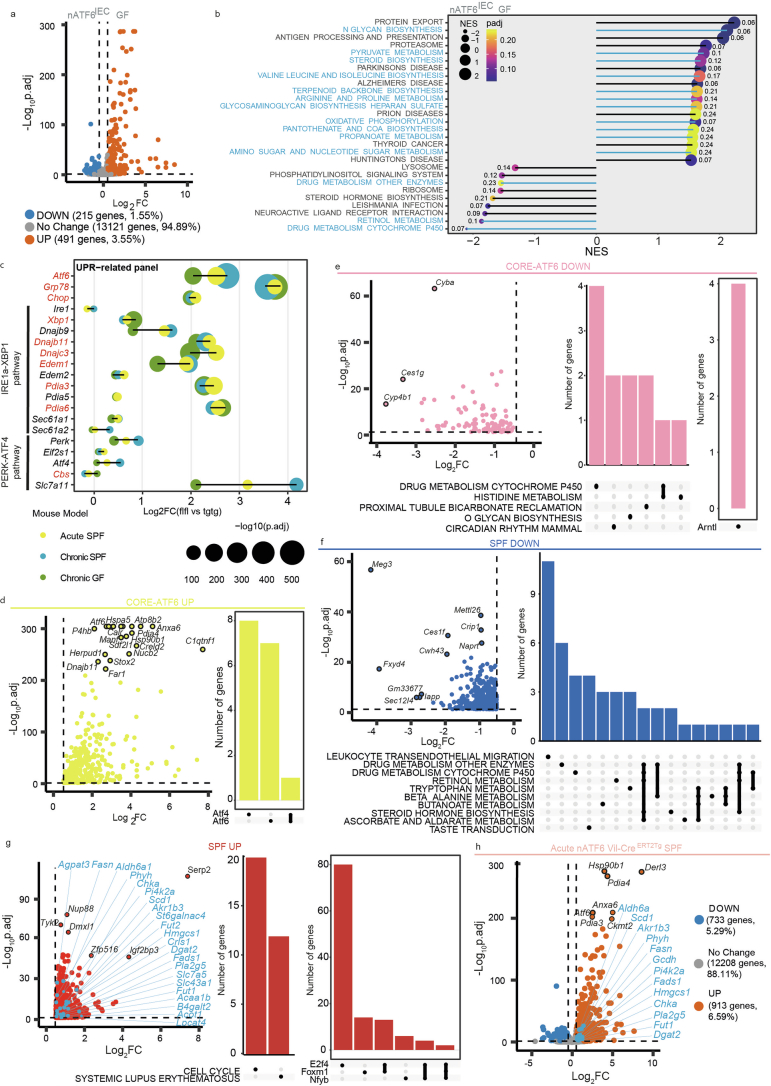
Gene expression and pathway analysis in response to ATF6 activation under GF and SPF conditions. **a**, Volcano plot of detected genes in fl/fl (n = 6 mice) versus tg/tg (n = 6 mice) nATF6^IEC^ mice showing the number and percentage of genes that are unchanged (No Change), upregulated in tg/tg mice (UP) or downregulated in tg/tg mice (DOWN). Threshold: −0.5 > log2FC > 0.5, *p* adj. <0.05. **b**, Lollipop graph of the top regulated KEGG pathways in tg/tg (n = 6 mice) versus fl/fl (n = 6 mice) nATF6^IEC^ GF mice (*p* adj. <= 0.25, −1 = > NES > = 1, with a minimum of 50% of genes detected in the pathway). Metabolic pathways are highlighted in blue. NES, normalized enrichment score. **c**, Lollipop graph of log2 FC of UPR-related genes. ATF6-pathway related genes are highlighted in red. Colour represents enrichment (yellow = Acute SPF, blue = Chronic SPF, green = Chronic GF) and size, -log10 adjusted p-value (n = 6 mice per genotype) **d**, Volcano Plot of genes in the regulatory cluster CORE-ATF6 UP. Upset plot representing the number of genes detected for the respective transcription factors driving the cluster (n = 6 mice per genotype). **e**, Volcano Plot of genes in the regulatory cluster CORE-ATF6 DOWN. Upset plot representing the number of genes detected in the cluster-associated KEGG pathways (threshold: >5% of pathway genes detected, *p* adj. < 0.2). Upset plot representing the number of genes detected and the respective transcription factors driving the cluster (n = 6 mice per genotype). **f**, Volcano Plot of genes in the regulatory cluster SPF DOWN. Upset plot representing the number of genes detected in the cluster-associated KEGG pathways (threshold: >5% of pathway genes detected, *p* adj. < 0.2, n = 6 mice per genotype). **g**, Volcano Plot of genes in the regulatory cluster SPF UP. Genes related to fatty acid metabolism are highlighted in blue. Upset plot representing the number of genes detected in the cluster-associated KEGG pathways (threshold: >5% of pathway genes detected, *p* adj. < 0.2). Upset plot representing the number of genes detected and the respective transcription factors driving the cluster (n = 6 mice per genotype). **h**, Volcano plot of detected genes in fl/fl versus tg/tg following acute activation of ATF6 in nATF6 Vil-Cre^ERT2^ SPF mice showing the number and percentage of genes that are unchanged (No Change), upregulated in tg/tg mice (UP) or downregulated in tg/tg mice (DOWN). Threshold: −0.5 > log2FC > 0.5, *p* adj. <0.05. Genes related to fatty acid metabolism are highlighted in blue (n = 6 mice per genotype). A significant *p*-value < 0.05 is represented as an asterisk: *p < 0.05, **p < 0.01. ***p < 0.001, ****p < 0.0001. Source data

To characterize the transcriptional response induced by chronic ATF6 activity and to further understand the contribution of bacteria, we performed bulk mRNA-sequencing (mRNA-seq) of colonic epithelial cells in fl/fl and tg/tg mice at the pre-tumour time point (5 week) under SPF and GF conditions (Fig. 2c). Transgenic chronic activation of nATF6 in SPF tg/tg mice results in a total of 1,941 differentially expressed genes (DEGs; threshold: −0.5 > log_2_fold change (FC) > 0.5, adjusted *P* value (*P* adj.) < 0.05) compared to fl/fl controls, of which 1,236 DEGs are upregulated and 705 DEGs are downregulated (Fig. 2d and Supplementary Table 1). Functional analysis using the Kyoto Encyclopedia of Genes and Genomes (KEGG) pathways showed that almost half (16/36) of the top- and bottom-ranked pathways, according to the normalized enrichment score (NES), are related to metabolic pathways in SPF tg/tg mice (Fig. 2e and Supplementary Table 2). Under GF conditions, tg/tg mice showed a total of 706 DEGs, of which 491 DEGs are upregulated and 215 DEGs are downregulated (Extended Data Fig. 2a and Supplementary Table 1). Again, half of the top- and bottom-ranked KEGG pathways (14/28) in GF tg/tg mice are linked to metabolic pathways (Extended Data Fig. 2b and Supplementary Table 2). Metabolic KEGG pathways associated with SPF tg/tg mice (tumour development) but not with GF tg/tg mice (no tumour development) are beta alanine metabolism, butanoate metabolism, fatty acid (FA) metabolism, glycolysis gluconeogenesis, metabolism of xenobiotics by cytochrome P450 and nicotinate and nicotinamide metabolism (Fig. 2e). Of these, FA metabolism represents the highest-ranked KEGG pathway upregulated in SPF tg/tg mice. To characterize the specificity of ATF6-related activation in the context of all UPR arms, we specifically looked at UPR-related genes, including the chronic SPF model, GF model and our acute (tamoxifen-inducible transgenic model at 4 days after ATF6 activation; nATF6 Vil-Cre^ERT2Tg^) SPF model (Extended Data Fig. 2c). We see that eight of the nine top regulated genes are also described to be ATF6 targets, with considerable overlap in the other UPR^ER^ arms. The panel reveals that the expression level of target genes is amplified by chronicity of ATF6 activation and bacterial presence.

To disentangle the transcriptional regulation between chronic tg/tg signalling under SPF and GF conditions, differentiating between the tumour-developing and non-tumour-developing phenotype, we applied Signature Regulatory Clustering (SiRCle)^13^. We defined three states of genes in tg/tg mice compared to fl/fl mice for each colonization status group, namely ‘no change’ (not regulated, −0.5 < log_2_FC < 0.5, *P* adj. > 0.05), UP (upregulated, log_2_FC > 0.5 and *P* adj. < 0.05) and DOWN (downregulated, log_2_FC < −0.5 and *P* adj. < 0.05; Fig. 2f and Supplementary Table 3). Following the flow of genes between SPF and GF conditions identified eight regulatory clusters named after the biological phenotype they include, for instance, Core-ATF6 UP (Fig. 2f and Supplementary Table 3). Depicted next to the Alluvial plot are the number and percentage of genes found in each of the clusters. After the regulatory cluster ‘none’ (84.70%), the highest percentages of DEGs were found in the clusters ‘SPF UP’ (5.93 %), ‘SPF DOWN’ (4.27%) and ‘Core-ATF6 UP’ (2.65%; Fig. 2f). On each of those clusters we performed over-representation analysis using KEGG pathways to identify pathways that each cluster of genes is enriched for (threshold: >5% of pathway genes detected, *P* adj. < 0.2; Supplementary Table 4) and DoRothEA transcription factor regulons^14,15^ to identify transcription factors that drive gene expression in the individual clusters (Supplementary Table 5). As anticipated, more than half (*P4hb*, *Atf6*, *Calr*, *Hspa5*, *Pdia4*, *Anxa6*, *Manf*, *Sdf2I1*, *Hsp90b1*, *CreId2*, *Nucb2*, *C1qtnf1*, *Herpud1* and *Dnajb11*) of the highly DEGs in the Core-ATF6 UP cluster (24 DEGs) are classically associated with ER protein folding and calcium homeostasis, and localized within the ER (Extended Data Fig. 2d). Transcription factors *Atf4* and *Atf6* were identified as the drivers of the CORE-ATF6 UP cluster (Extended Data Fig. 2d and Supplementary Table 5). Highly DEGs in the Core-ATF6 DOWN cluster include *Cyba*, *Ces1g* and *Cyp4b1*, which are associated with intestinal reactive oxygen species production and defence against bacteria at the colonic epithelium, hydrolysis of lipids and xenobiotics, and the hydroxylation of FAs and alcohols, respectively (Extended Data Fig. 2e). Functionally, the CORE-ATF6 DOWN cluster associates with five KEGG pathways, including the drug metabolism cytochrome p450 pathway, associated with cellular metabolism, homeostasis and the detoxification of drugs (Extended Data Fig. 2e and Supplementary Table 4). *Arntl* is the identified transcription factor driving the CORE-ATF6 DOWN cluster, responsible for maintenance of the circadian rhythm and has been shown to be downregulated in cancers with an association to anti-cancer roles (Extended Data Fig. 2e and Supplementary Table 5). Highly regulated DEGs in the SPF DOWN cluster include genes that have been associated with tumour suppression and CRC (*Meg3*, *Cwh43*, *Naprt)*, lipid metabolism and detoxification (*Ces1f*) and bactericidal/antimicrobial activity (*Iapp*; Extended Data Fig. 2f). The majority of KEGG pathways associated with the SPF DOWN cluster are metabolic pathways, including the drug metabolism cytochrome p450 pathway also identified in the CORE-ATF6 DOWN cluster (Extended Data Fig. 2f and Supplementary Table 4).

Most relevant to our mouse model and the role of ATF6 signalling in colon adenoma development in tg/tg mice is the SPF UP cluster. The highest DEG in this cluster is ER-localized *Serp2*, classically involved in the UPR and protein glycosylation (Extended Data Fig. 2g). Among the other highly DEGs in this cluster, *Nup88*, *Dmxl1* and *Igf2bp3* are associated with dysplasia and cancer, including CRC. Importantly, and in line with our findings above (Fig. 2e), the SPF UP cluster contains 23 differentially expressed FA metabolism-related genes (Fig. 2g and Extended Data Fig. 2g). Of note, additional analysis of the pre-tumour time point using our inducible nATF6 Vil-Cre^ERT2Tg^ mouse model that addresses the transcriptional response induced by acute (4 day) activation of nATF6, demonstrates that the upregulation of the majority (13/23) of these FA metabolism-related genes occurs already early on after nATF6 activation (Extended Data Fig. 2h and Supplementary Table 1). In accordance with our tumour phenotype, one of the two identified KEGG pathways associated with the SPF UP cluster is the cell cycle (Extended Data Fig. 2g and Supplementary Table 4). Three transcription factors were identified as drivers of the SPF UP cluster (Extended Data Fig. 2g and Supplementary Table 5), namely *E2f4* (control of cell cycle, increased expression in cancers), *Foxm1* (oncogenic transcription factor, control of cell cycle, regulation of CRC metastasis and progression) and *Nfyb* (promotes invasion, metastasis and differentiation, associated with multiple cancers, activator or repressor in a context-dependent manner).

### ATF6 positively correlates with FASN in individuals with CRC

**Extended Data Fig. 3:**
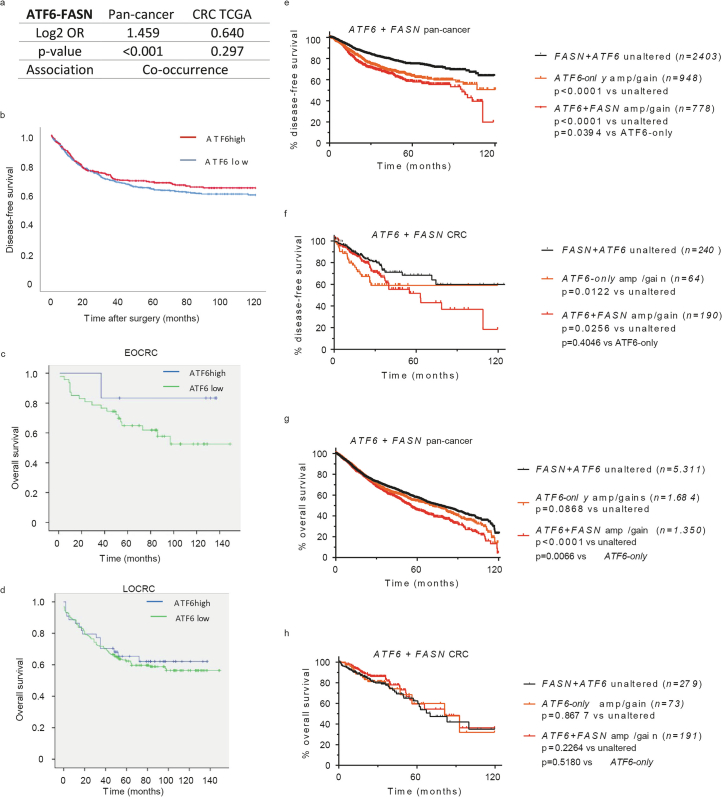
ATF6 and FASN co-occurrence in CRC patient cohorts. **a**, Table showing the analysis of *ATF6* amplifications/gain and *FASN* amplifications/gain in the pan-cancer and TCGA Datasets, indicating the log2 odds ratio (OR), *p* value and observed association/tendency. **b**, Kaplan-Meier analysis of disease-free survival shown for ATF6-low (blue) and ATF6-high (red) at different months after surgery in CRC cohort 1 (n = 959 patients, *p* = 0.284). **c**, Kaplan-Meier analysis of overall survival shown for ATF6-low (green) and ATF6-high (blue) at different months after surgery in CRC cohort 3 EOCRC patients. **d**, Kaplan-Meier analysis of overall survival shown for ATF6-low (green) and ATF6-high (blue) at different months after surgery in CRC cohort 3 LOCRC patients. **e**, Kaplan-Meier analysis of disease-free survival of patients in the Pan-Cancer Atlas database (n = 4129/10967 cases including approx. 600 CRC cases) with patients grouped into FASN + ATF6 unaltered, ATF6-only amp/gain or ATF6 + FASN amp/gain. **f**, Kaplan-Meier analysis of disease-free survival of patients in The Cancer Genome Atlas database (TCGA; n = 494/640 CRC cases) with patients grouped into FASN + ATF6 unaltered, ATF6-only amp/gain or ATF6 + FASN amp/gain. **g**, Kaplan-Meier analysis of overall survival of patients in the Pan-Cancer Atlas database (n = 8.345/10.967 cases including approx. 600 CRC cases) with patients grouped into FASN + ATF6 unaltered, ATF6-only amp/gain or ATF6 + FASN amp/gain. **h**, Kaplan-Meier analysis of overall survival of patients in The Cancer Genome Atlas database (TCGA; n = 543/640 CRC cases) with patients grouped into FASN + ATF6 unaltered, ATF6-only amp/gain or ATF6 + FASN amp/gain. Source data

Our findings from the bulk mRNA-seq identified fatty acid synthase (*Fasn*), encoding a key multi-enzyme complex of de novo lipid biosynthesis^16^, to be upregulated in response to both chronic and acute activation of nATF6 (Fig. 2g and Extended Data Fig. 2g,h). Indeed, alterations in the lipidomic profile have been observed in several cancer types^17–19^, including CRC^20^, which marks lipid metabolic rewiring as a phenotypic hallmark of cancer. Upregulation of FASN is associated with aggressive disease and poor prognosis in CRC^21,22^. We recently described a robust CRC-specific lipid signature based on quantitative lipidomics in a multi-cohort and cross-centre study, identifying triacylglycerol species and *FASN* expression as differential markers of cancerous tissue and bad prognosis, respectively^23^. To investigate the association between FASN and ATF6, we stained a random subset (*n* = 181) of tissue cores from our human CRC human cohort 1 for FASN and correlated the QuPath-quantified FASN H-score with our ATF6 classification (from Fig. 1a) in the same individuals. Exemplary images show that individuals clearly differed in their FASN expression levels (Fig. 2h). Strikingly, FASN H-scores were significantly higher in individuals with CRC classified as ATF6-high compared to those classified as ATF6-low (Fig. 2i), identifying a positive correlation between ATF6 and FASN expression in CRC human tissue. In a further approach, the publicly available Pan-Cancer Atlas (*n* = 10,967 cases including approximately 600 CRC cases) and The Cancer Genome Atlas (TCGA; *n* = 640 CRC cases) databases were screened for genomic amplifications/gains (amp/gain) in either *ATF6* alone (*ATF6*-only), *FASN* alone (*FASN*-only) or in both genes simultaneously (*ATF6* + *FASN*). The relationship between *ATF6*-high and *FASN*-high individuals is evident through their association classified as co-occurrence, despite not reaching significance in the TCGA database (*P* = 0.297), likely due to lower case numbers compared to the Pan-Cancer Atlas database (*P* < 0.001; Extended Data Fig. 3a). While the IHC-quantified ATF6 expression did not associate with CRC human disease-free survival in our human cohorts (Extended Data Fig. 3b–d), Kaplan–Meier analysis in both databases showed a significantly reduced disease-free survival in cases that were classed as *ATF6*-only amp/gain (*P* < 0.001 pan-cancer, *P* = 0.0122 TCGA) and those classed as combined *ATF6* and *FASN* amp/gain (*P* < 0.0001 pan-cancer, *P* < 0.0001 TCGA), compared to those cases unaltered in either of the two genes (Extended Data Fig. 3e,f). Importantly, disease-free survival was significantly reduced in cases of combined *ATF6* and *FASN* amp/gain compared to those showing *ATF6*-only amp/gain in the Pan-Cancer Atlas (*P* = 0.0394), with a trend observed in the TCGA database (*P* = 0.4046; Extended Data Fig. 3e,f). Amp/gain modifications were not associated with overall survival in the two databases (Extended Data Fig. 3g,h).

### ATF6 drives FA elongation and LCFA accumulate in mice and individuals with CRC

**Fig. 3:**
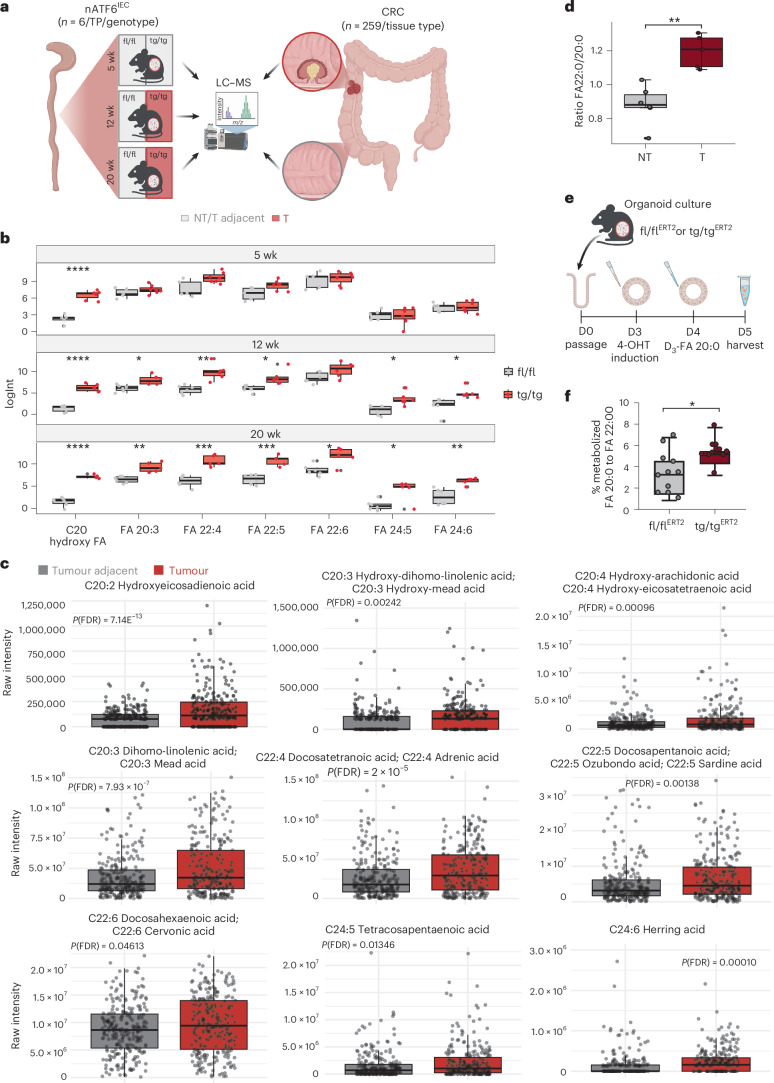
ATF6 drives FA elongation and LCFAs accumulate in mice and individuals with CRC. **a**, Schematic depicting metabolomic analysis strategy in nATF6^IEC^ mice and individuals with CRC (cohort 4). Created with BioRender.com. **b**, Box plots comparison of log-transformed FA intensities in caecal content, comparing fl/fl to tg/tg mice, stratified by time point (5 weeks *n* = 5 fl/fl mice, *n* = 7 tg/tg mice; 12 weeks *n* = 6 fl/fl mice, *n* = 5 tg/tg mice; 20 weeks *n* = 6 fl/fl mice, *n* = 5 tg/tg mice). Boxes depict the interquartile range (IQR), whiskers extend to furthest non-outlier values (no more than 1.5 times the IQR), and the centre lines indicate the median value. Only FA metabolites that were significant in mouse data and overlapped with CRC human data are shown. Statistical significance was calculated using pairwise *t*-tests and adjusted for multiple comparisons using the Benjamini–Hochberg procedure (week 5 C20 hydroxy FA *P* < 0.0001, week 12 C20 hydroxy FA *P* < 0.0001, FA 20:3 *P* = 0.003, FA 22:6 *P* = 0.02 FA 22:5 *P* = 0.0005, FA 22:4 *P* = 0.005, FA 24:6 *P* = 0.0005, FA 24:5 *P* = 0.01, week 20 C20 hydroxy FA *P* < 0.0001, FA 20:3 *P* = 0.01, FA 22:5 *P* = 0.01, FA 22:4 *P* = 0.005, FA 24:6 *P* = 0.01, FA 24:5 *P* = 0.03). **c**, Box plot comparison of FA intensities in tumour tissue and tumour-adjacent tissue of samples from cohort 4 (*n* = 259 individuals). The plot displays the intensity differences for nine FAs that showed significant abundance in tumour samples (left to right): hydroxyeicosadenoic acid (FA 20:2), hydroxy-dihomo-linolenic acid (FA 20:3), hydroxy-arachidonic acid (FA 20:4), dihomo-linoleic acid (FA 20:3), docosatetranoic acid (FA 22:4), docosapentanoic acid (FA 22:5), docosahexanoic acid (FA 22:6), tetracosapentanoic acid (FA 24:5) and herring acid (FA 24:6). Boxes depict the IQR, whiskers extend to the furthest non-outlier values (no more than 1.5 times the IQR), and the centre lines indicate the median value. Statistical significance was calculated using pairwise Wilcoxon tests and adjusted for multiple comparisons using the Benjamini–Hochberg procedure. A paired *t*-test was used to calculate *P* values (and corrected for false discovery rate (FDR)). Only FAs with *P* values (FDR corrected) < 0.05 are shown. **d**, Percentage of SAFAs in NT (*n* = 6 mice) and T (*n* = 5 mice) tissue after quantification of total FA using GC–MS. Box depicts IQR, whiskers extend to furthest non-outlier values (no more than 1.5 times the IQR), and the centre lines indicate the median value. Statistical analysis was performed using an unpaired Wilcoxon test (*P* = 0.0043). **e**, Scheme showing the treatment of intestinal organoids from nATF6 Vil-CreERT2 (fl/fl^ERT2^ and tg/tg^ERT2^) mice cultured ex vivo (*n* = 11 fl/fl and *n* = 12 tg/tg biological replicates). Organoids were passaged at D0, ATF6 expression in all wells was induced at D3 with 500 nM (Z)-4-hydroxytamoxifen (4-OHT) and treated with D3-FA 20:0 at D4, before harvesting at D5 (see also Supplementary Fig. 1). Created with BioRender.com. **f**, Percentage of metabolized D_3_-FA 20:0 to D_3_-FA: 22:0 in organoids of tg/tg^ERT2^, compared to fl/fl^ERT2^ controls using GC–MS. Significance was calculated using the Student’s two-tailed unpaired *t*-test (*P* = 0.0184). *P < 0.05, **P < 0.01, ***P < 0.001, ****P < 0.0001. Source data

**Extended Data Fig. 4:**
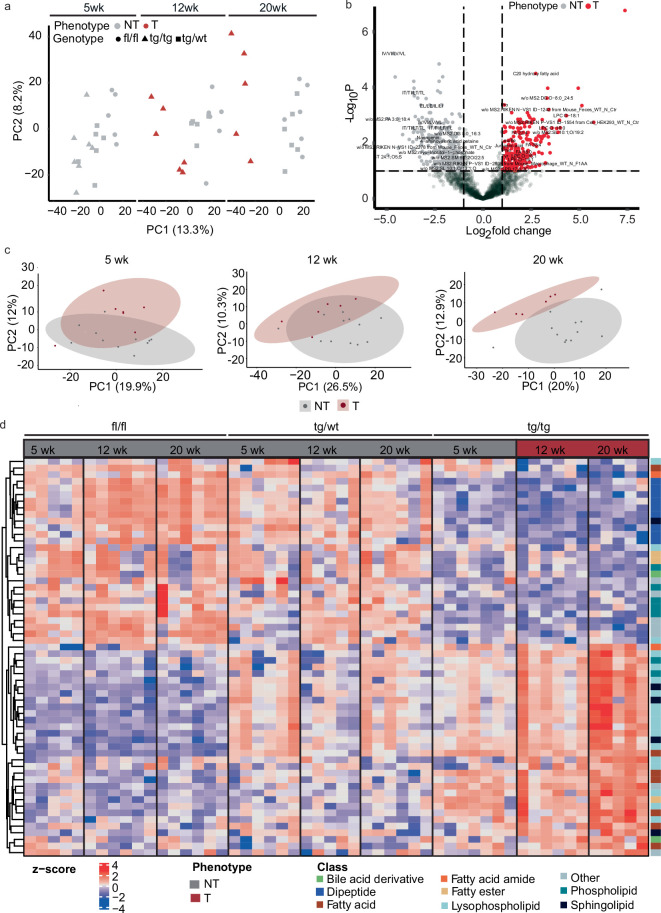
Metabolite profiles in nATF6^IEC^ mice. **a**, PCA of metabolite profiles comparing fl/fl (circles), tg/wt (squares) and tg/tg (triangles) mice at 5wk, 12wk and 20wk. Grey indicates non-tumour (NT) and red tumour (T) (5wk n = 5 fl/fl mice, n = 6 tg/wt mice, n = 7 tg/tg mice; 12wk n = 6 fl/fl mice, n = 6 tg/wt mice, n = 6 tg/tg mice, 20wk n = 6 fl/fl mice, n = 6 tg/wt mice, n = 5 tg/tg mice). **b**, Volcano plot of differentially enriched metabolites between fl/fl and tg/tg mice across all time points (Log2 fold change threshold = 1.0, FDR threshold = 0.1). Significance was calculated using paired T-tests on log-transformed data (5wk n = 5 fl/fl mice, n = 6 tg/wt mice, n = 7 tg/tg mice; 12wk n = 6 fl/fl mice, n = 6 tg/wt mice, n = 6 tg/tg mice, 20wk n = 6 fl/fl mice, n = 6 tg/wt mice, n = 5 tg/tg mice). **c**, PCA of luminal lipid profiles between tumor-susceptible/tumor (T) and non-tumor (NT) mice at 5wk, 12wk and 20wk (5wk n = 5 fl/fl mice, n = 6 tg/wt mice, n = 7 tg/tg mice; 12wk n = 6 fl/fl mice, n = 6 tg/wt mice, n = 6 tg/tg mice, 20wk n = 6 fl/fl mice, n = 6 tg/wt mice, n = 5 tg/tg mice). **d**, Heatmap of scaled (z-score) differentially enriched metabolites, sorted by genotype and age. Rows are clustered using Ward’s method (5wk n = 5 fl/fl mice, n = 6 tg/wt mice, n = 7 tg/tg mice; 12wk n = 6 fl/fl mice, n = 6 tg/wt mice, n = 6 tg/tg mice, 20wk n = 6 fl/fl mice, n = 6 tg/wt mice, n = 5 tg/tg mice). Source data

**Extended Data Fig. 5:**
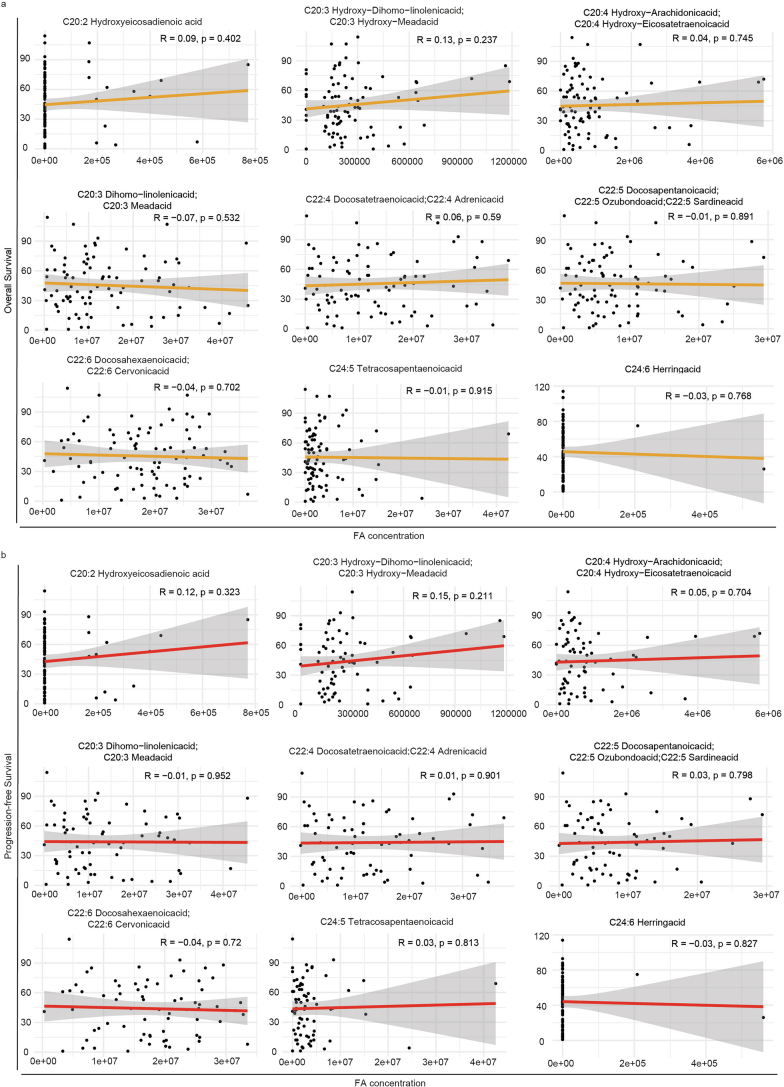
Correlations of LCFAs with clinical prognosis. **a**, Correlation plot, with 95% confidence intervals overlaid, of LCFA concentration and overall survival in CRC patients (n = 88) for the nine fatty acids that showed significant abundance in tumor samples: hydroxyeicosadenoic acid (FA 20:2), hydroxy-dihomo-linolenic acid (FA 20:3), hydroxy-arachidonic acid (FA 20:4), dihomo-linoleic acid (FA 20:3), docosatetranoic acid (FA 22:4), docosapentanoic acid (FA 22:5), docosahexanoic acid (FA 22:6), tetracosapentanoic acid (FA 24:5) and herring acid (FA 24:6). **b**, Correlation plot, with 95% confidence intervals overlaid, of LCFA concentration and progression-free survival in CRC patients (n = 77) for the nine fatty acids that showed significant abundance in tumor samples: hydroxyeicosadenoic acid (FA 20:2), hydroxy-dihomo-linolenic acid (FA 20:3), hydroxy-arachidonic acid (FA 20:4), dihomo-linoleic acid (FA 20:3), docosatetranoic acid (FA 22:4), docosapentanoic acid (FA 22:5), docosahexanoic acid (FA 22:6), tetracosapentanoic acid (FA 24:5) and herring acid (FA 24:6). Correlations were performed using pairwise Pearson correlation coefficients for all nine fatty acids. Significant correlations were defined as those with R > 0.6 and FDR-adjusted p < 0.05. Source data

**Extended Data Fig. 6:**
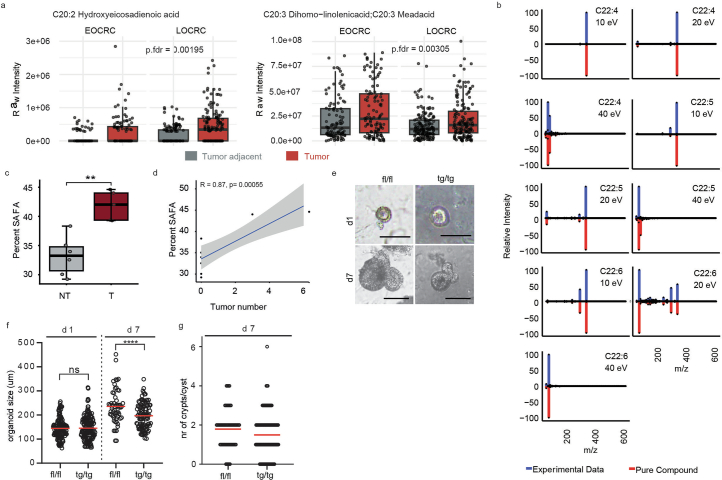
Metabolite profiles in CRC patients and nATF6^IEC^ mice. **a**, Boxplot comparison of fatty acid intensities in tumor and tumor-adjacent tissue stratified for EOCRC (n = 99 patients) and LOCRC (n = 152 patients). Displayed are intensity differences for two fatty acids that show significant abundance in tumor tissue for EOCRC in addition to EOCRC. Boxes depict interquartile range (IQR), whiskers extend to furthest non-outlier values (no more than 1.5 x IQR), and the centre lines indicate the median value. Only fatty acids with p-values (FDR corrected) <0.05 are shown. Statistical significance was determined using a paired T-test. **b**, Mirror plots of MS/MS spectra for the annotation of fatty acids at 10, 20 and 40 eV. The plots compare the experimental MS/MS spectra (top, blue) with the standard compound spectra (bottom, red) for selected fatty acids. Correct annotation occurs when retention time, high-resolution mass, precursor and diagnostic fragment ions are similar between the analyte and the CRC-sample. **c**, Percentage of saturated fatty acids (SAFA) in NT (n = 6 mice) and T (n = 5 mice) tissue after quantification of total FA using GC-MS. Boxes depict interquartile range (IQR), whiskers extend to furthest non-outlier values (no more than 1.5 x IQR), and the centre lines indicate the median value. Statistical significance was calculated using pairwise Wilcoxon-tests and adjusted for multiple comparisons using the Benjamini-Hochberg procedure. **d**, Spearman’s rank correlation of percentage SAFA and tumor number, with 95% confidence intervals, after quantification of total FA using GC-MS. **e**, Representative images of intestinal organoids on day 1 (d1) and day 7 (d7) after culture, for the fl/fl and tg/tg genotype. Scale bars: 50 µm (n = 4 replicates, with n = 149 cysts for fl/fl d1, n = 170 cysts for tg/tg d1, n = 57 cysts for fl/fl d7 and n = 111 cysts for tg/tg d7). **f**, Quantification of organoid size as the sum of two dimensions in µm at d1 and d7 for the fl/fl and tg/tg genotype, with group mean denoted by a horizontal red bar (n = 4 replicates, with n = 149 cysts for fl/fl d1, n = 170 cysts for tg/tg d1, n = 57 cysts for fl/fl d7 and n = 111 cysts for tg/tg d7). Statistical significance was determined by a one-way ANOVA and Sidak’s test for multiple comparison (p < 0.0001). **g**, Quantification of the number of crypts per cyst at d7 for the fl/fl and the tg/tg genotype, with group mean denoted by a horizontal red bar (n = 4 replicates, with n = 149 cysts for fl/fl d1, n = 170 cysts for tg/tg d1, n = 57 cysts for fl/fl d7 and n = 111 cysts for tg/tg d7). Statistical significance was determined by a one-way ANOVA and Sidak’s test for multiple comparison. A significant *p*-value < 0.05 is represented as an asterisk: *p < 0.05, **p < 0.01. ***p < 0.001, ****p < 0.0001. Source data

ATF6 signalling clearly alters epithelial metabolism. To understand its effects on the metabolome, we performed untargeted metabolomics of nATF6^IEC^ caecal content at pre-tumour (5 week), tumour (12 week) and late-tumour (20 week) time points using liquid chromatography–time-of-flight mass spectrometry (LC–TOF-MS; Fig. 3a). In parallel, we performed untargeted metabolomics of CRC human tumour tissue (T) and human-matched tumour adjacent (T adjacent) tissue in a cohort of 259 individuals with EOCRC and LOCRC (cohort 4; Fig. 3a). In our nATF6^IEC^ mouse model, principal-component analysis revealed a drastic difference in the metabolome between tumour-bearing mice (red) and non-tumour mice (grey), while metabolite profiles clustered in closer proximity for all three genotypes at the pre-tumour time point (Extended Data Fig. 4a). Differential enrichment analysis at the tumour time point demonstrated a depletion of peptide metabolites and sphingolipids, while long-chain fatty acids (LCFAs) and other complex lipid species were enriched in T mice (Extended Data Fig. 4b). Principal-component analysis showed that lipid profiles of non-tumour (NT) mice separate from tumour (T) mice already at the pre-tumour time point, with increasing diversity over time (Extended Data Fig. 4c). Differential metabolite analysis of the significantly regulated metabolites, shown between all genotypes and time points, demonstrated that tg/tg-associated metabolites mostly comprise lysophospholipids and LCFAs (Extended Data Fig. 4d). In the CRC human cohort 4, we identified 56 FAs that were significantly regulated in T tissue compared to T adjacent tissue (Supplementary Table 6). We next examined the overlap between LCFAs (≥C20) that were significantly regulated in CRC human T tissue and in T mice. Multiple FAs were significantly regulated in both, including docosatetranoic acid (FA 22:4), docosapentanoic acid (FA 22:5), docosahexanoic acid (FA 22:6) and putative FAs: FA 20:3, C20 hydroxy FAs, FA 24:5 and FA 24:6 (Fig. 3b,c). No correlation was observed between tumour-associated LCFA concentrations and overall survival or progression-free survival in the individuals with CRC (Extended Data Fig. 5a,b). Stratifying by time point, an unknown C20 hydroxy FA was the only LCFA enriched in tg/tg mice at the pre-tumour time point; however, the number of significant shared LCFAs increased at the tumour and late-tumour time points, indicating that tumour onset increases the LCFA pool (Fig. 3b). When further stratifying significantly regulated FAs in CRC human cohort 4 into EOCRC and LOCRC, we identified two LCFAs (C20:2 hydroxy FA and C20:3 FA) to also be significantly regulated in EOCRC T tissue compared to T adjacent tissue (Extended Data Fig. 6a). FAs 22:4, 22:5 and 22:6 have been validated by comparison with pure standards (Extended Data Fig. 6b).

To further characterize the altered lipid environment in tg/tg mice, we quantified total FAs in colonic tissue using gas chromatography–mass spectrometry (GC–MS). Analyses revealed an increase in saturated fatty acids (SAFAs) in T compared to NT mice, which also correlated with tumour number (Extended Data Fig. 6c,d). Furthermore, T mice show a clear elongation of SAFAs including FA 20:0 and FA 22:0 compared to NT mice (Fig. 3d). To determine whether elongation is a direct consequence of ATF6 signalling, we assessed ATF6-mediated elongation of FAs in ex vivo colon organoid cultures. We initially phenotypically characterized colon organoids derived from fl/fl controls and tg/tg mice at the late-tumour time point (20 week). Both fl/fl and tg/tg crypts formed viable organoids in culture, including cyst formation (day 1) and budding (day 7) of cysts (Extended Data Fig. 6e). Strikingly, tg/tg organoids in culture did not show any significant increase in organoid size (Extended Data Fig. 6f) or the number of budding crypts per cyst (Extended Data Fig. 6g) compared to fl/fl controls, indicating that the hyper-proliferative phenotype observed in vivo in the presence of the microbiota is lost in this ex vivo culture system. In a separate experiment, intestinal organoids from nATF6 Vil-Cre^ERT2^ (fl/fl^ERT2^ and tg/tg^ERT2^) mice were cultured, and *ATF6* expression induced by adding 500 nM (Z)-4-hydroxytamoxifen (4-OHT) to the culture medium for 24 h. This was followed by a 24 h exposure of organoids to deuterated eicoseinoic acid (D_3_-FA 20:0; Fig. 3e and Supplementary Fig. 1). Importantly, tg/tg^ERT2^ intestinal organoids showed a significantly increased potential of FA elongation, quantified as the percentage of metabolized D_3_-FA 20:0 to D_3_-FA: 22:0, compared to fl/fl^ERT2^ controls (Fig. 3f). The above findings demonstrate that ATF6 activation is the driver of FA elongation in the absence of microbial and immune signalling.

### ATF6-related lipid profiles are causally linked to tumour-associated microbiota

**Fig. 4:**
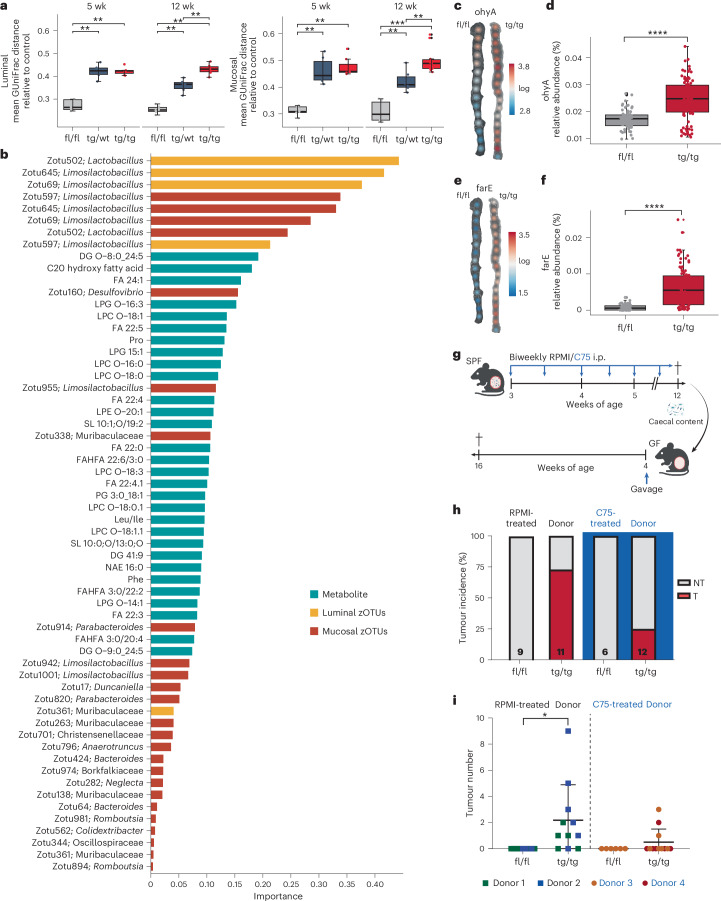
ATF6-related lipid profiles are causally linked to tumour-associated microbiota. **a**, Mean generalized UniFrac distance relative to control across fl/fl, tg/wt and tg/tg samples, at the pre-tumour time point (5 week) and at tumour onset (12 week) in luminal and mucosal communities. *P* values were calculated using pairwise Wilcoxon tests and adjusted for multiple comparisons using the Benjamini–Hochberg procedure (luminal: 5 weeks fl/fl versus tg/wt *P* = 0.0043, 5 weeks fl/fl versus tg/tg *P* = 0.0025, 12 weeks fl/fl versus tg/wt *P* = 0.0022, 12 weeks fl/fl versus tg/tg *P* = 0.0022, 12 weeks tg/wt versus tg/tg *P* = 0.0022; mucosal: 5 weeks fl/fl versus tg/wt *P* = 0.0043, 5 weeks fl/fl versus tg/tg *P* = 0.0025, 12 weeks fl/fl versus tg/wt *P* = 0.0022, 12 weeks fl/fl versus tg/tg *P* = 0.00016 12 weeks tg/wt versus tg/tg *P* = 0.0048). Boxes depict the IQR, whiskers extend to the furthest non-outlier values (no more than 1.5 times the IQR), and the centre lines indicate the median value (5 weeks *n* = 5 fl/fl mice, *n* = 6 tg/wt mice, *n* = 7 tg/tg mice; 12 weeks *n* = 6 fl/fl mice, *n* = 6 tg/wt mice, *n* = 6 tg/tg mice, *n* = 11 tissue samples). **b**, Loadings plots of omic features selected by the sPLS-DA model, discriminating tg/tg mice from other genotypes. Luminal zOTUs are shown in yellow, mucosal zOTUs in red and metabolites in blue. Features are sorted by importance (*n* = 18 tg/tg mice, *n* = 34 tg/wt and fl/fl mice). **c**, Spatial maps of log_10_-transformed predicted relative abundance of bacterial *ohyA*-positive zOTUs along the entire colon in fl/fl and tg/tg mice (*n* = 6 mice per group). **d**, Predicted relative abundance of bacterial *ohyA* in tg/tg and fl/fl mice (*n* = 6 mice per group, 15–17 tissue samples per mouse, *P* = 4.7 × 10^−10^). Boxes depict the IQR, whiskers extend to the furthest non-outlier values (no more than 1.5 times the IQR), and the centre lines indicate the median value. Statistical significance was calculated using pairwise *t*-tests and adjusted for multiple comparisons using the Benjamini–Hochberg procedure. **e**, Spatial maps of log_10_-transformed predicted relative abundance of bacterial FA efflux pump (farE)-positive zOTUs along the entire colon in fl/fl and tg/tg mice (*n* = 6 mice per group). **f**, Predicted relative abundance of FA efflux pump, farE in tg/tg and fl/fl mice (*n* = 6 mice per group, 15–17 tissue samples per mouse, *P* = <2.26 × 10^−16^). Boxes depict the IQR, whiskers extend to the furthest non-outlier values (no more than 1.5 times the IQR), and the centre lines indicate the median value. Statistical significance was calculated using pairwise *t*-tests and adjusted for multiple comparisons using the Benjamini–Hochberg procedure. **g**, Schematic showing the experimental design for in vivo C75 FASN inhibitor intervention and transfer experiment. SPF mice were i.p. injected with either the RPMI control or the C57 FASN inhibitor biweekly from the age of 3 weeks. After an intervention period of 9 weeks, mice were euthanized at 12 weeks of age. Arrows indicate i.p. injections. Created with BioRender.com. **h**, Tumour incidence (percentage) of GF fl/fl and tg/tg mice after gavage with donor material from either RPMI-treated mice (controls, *n* = 9 fl/fl mice and *n* = 11 tg/tg mice) or C75 FASN inhibitor-treated mice (*n* = 6 fl/fl mice and *n* = 12 tg/tg mice). **i**, Tumour number of GF fl/fl and tg/tg mice after gavage with donor material from either RPMI-treated mice (controls, *n* = *n* = 9 fl/fl mice and *n* = 11 tg/tg mice) or C75 FASN inhibitor-treated mice (*n* = 6 fl/fl mice and *n* = 12 tg/tg mice). Points are coloured by donor. Data are represented as the mean ± s.d. Statistical significance was calculated using an ordinary one-way ANOVA with Tukey’s multiple-comparison test (*P* = 0.0205). *P < 0.05, **P < 0.01, ***P < 0.001, ****P < 0.0001. Source data

**Extended Data Fig. 7:**
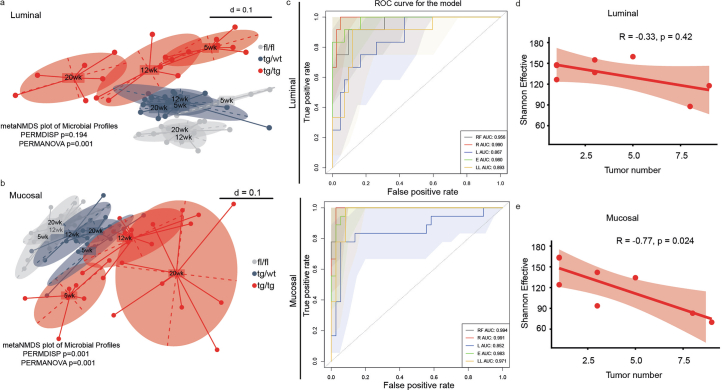
Luminal and mucosal microbiota profiles in nATF6^IEC^ mice. **a**, Non-metric multidimensional scaling (NMDS) plot of Beta diversity profiles based on generalized UniFrac distance comparing genotypes (fl/fl, tg/wt and tg/tg) at each timepoint (5, 12 and 20-weeks) in luminal (PERMANOVA, 5wk n = 5 fl/fl mice, n = 6 tg/wt mice, n = 7 tg/tg mice; 12wk n = 6 fl/fl mice, n = 6 tg/wt mice, n = 6 tg/tg mice, 20wk n = 6 fl/fl mice, n = 6 tg/wt mice, n = 6 tg/tg mice) and **b**, mucosal data (PERMANOVA, 5wk n = 5 fl/fl mice, n = 6 tg/wt mice, n = 7 tg/tg mice; 12wk n = 6 fl/fl mice, n = 6 tg/wt mice, n = 11 tg/tg mice, 20wk n = 6 fl/fl mice, n = 6 tg/wt mice, n = 9 tg/tg mice). **c**, ROC curves comparing classification accuracy of Random Forest (RF), Ridge regression (RR), LASSO (L), Elastic-net (E) and L1-penalized regression (LL) models built on luminal and mucosal data, respectively. Solid line represents mean accuracy, with shaded areas representing range. Mucosal data was randomly subsampled (5wk n = 5 fl/fl mice, n = 4 tg/wt mice, n = 5 tg/tg mice; 12wk n = 6 fl/fl mice, n = 6 tg/wt mice, n = 10 tg/tg mice, 20wk n = 5 fl/fl mice, n = 5 tg/wt mice, n = 8 tg/tg mice) to match luminal (5wk n = 5 fl/fl mice, n = 6 tg/wt mice, n = 7 tg/tg mice; 12wk n = 6 fl/fl mice, n = 6 tg/wt mice, n = 6 tg/tg mice, 20wk n = 6 fl/fl mice, n = 6 tg/wt mice, n = 6 tg/tg mice) sample size. Models were trained using repeated 5-fold cross-validation (CV). **d**, Spearman’s rank correlation between Shannon Effective and tumor number in mucosal and e, luminal data, with 95% confidence intervals (luminal n = 7 tg/tg mice, mucosal n = 7 tg/tg mice). Source data

We next sought to investigate whether chronic ATF6 activation would alter the luminal and mucosa-associated microbiota. To isolate the impact of tumour formation, we examined changes in microbiota composition at 5 weeks, before tumour onset. We observed augmented modulation of the mucosa-associated microbiota with increasing *Atf6* gene dose as shown by mean GUniFrac dissimilarity relative to control, between genotypes, an effect comparable to changes observed in the luminal environment (Fig. 4a). Global microbiota shifts were observed between all time points and genotypes, in both luminal and mucosal datasets, and appeared more distinct at later time points, particularly in tg/tg mice (Extended Data Fig. 7a,b; luminal permutational multivariate analysis of variance (PERMANOVA), *P* = 0.001, mucosal PERMANOVA *P* = 0.001). We applied machine learning models (random forest (RF), ridge regression (RR), LASSO (L), elastic-net (E) and L1-penalized regression (LL)) to both sample types, demonstrating that the mucosal microbiota can better classify the tumorigenic phenotype compared to the luminal microbiota. Supporting increased utility of mucosal over luminal microbiota, four of five models trained on mucosal microbiota data showed higher area under the curve values across all leave-one-out cross-validation repetitions (Extended Data Fig. 7c). Moreover, we observed a significant correlation between tumour number and Shannon effective diversity in mucosal data, which was absent in luminal data (Extended Data Fig. 7d,e), further supporting this finding and suggesting a link between tumour burden and microbial diversity.

**Extended Data Fig. 8:**
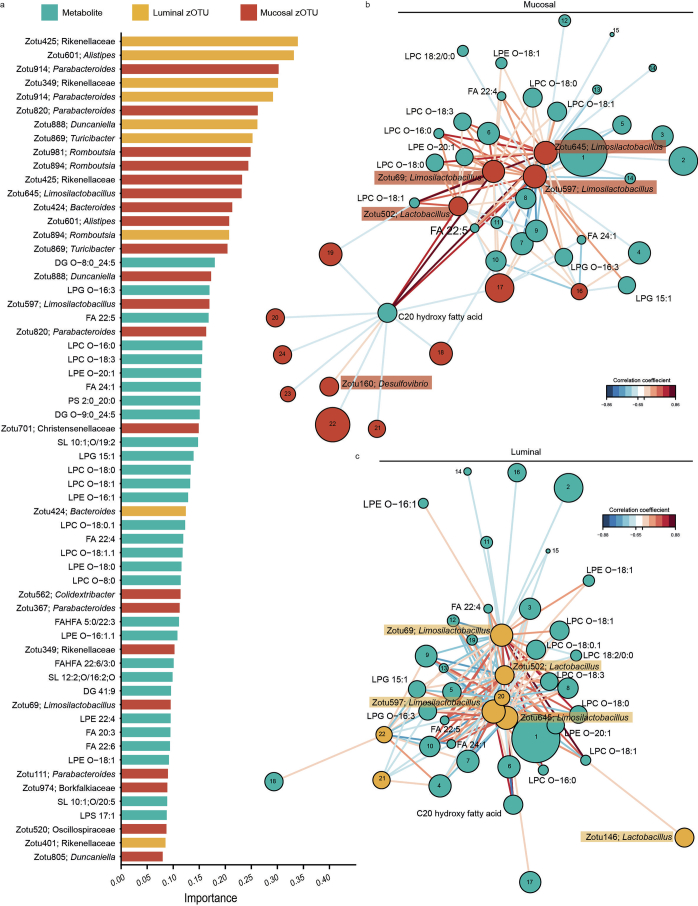
Multi-omics data integration of metabolite and microbial profiles. **a**, Loadings plots of omic features selected by the sPLS-DA model, discriminating T from NT mice. Luminal zOTUs are shown in yellow, mucosal zOTUs in red and metabolites in blue. Features are sorted by importance (n = 41 NT mice, n = 11 T mice). **b**, Relevance network of highly correlated (Absolute correlation >0.65) metabolites and mucosal zOTUs for the genotype sPLS-DA model (n = 41 NT mice, n = 11 T mice). **c**, Relevance network of highly correlated (Absolute correlation >0.65) metabolites and luminal zOTUs for the genotype sPLS-DA model (n = 41 NT mice, n = 11 T mice). Source data

**Extended Data Fig. 9:**
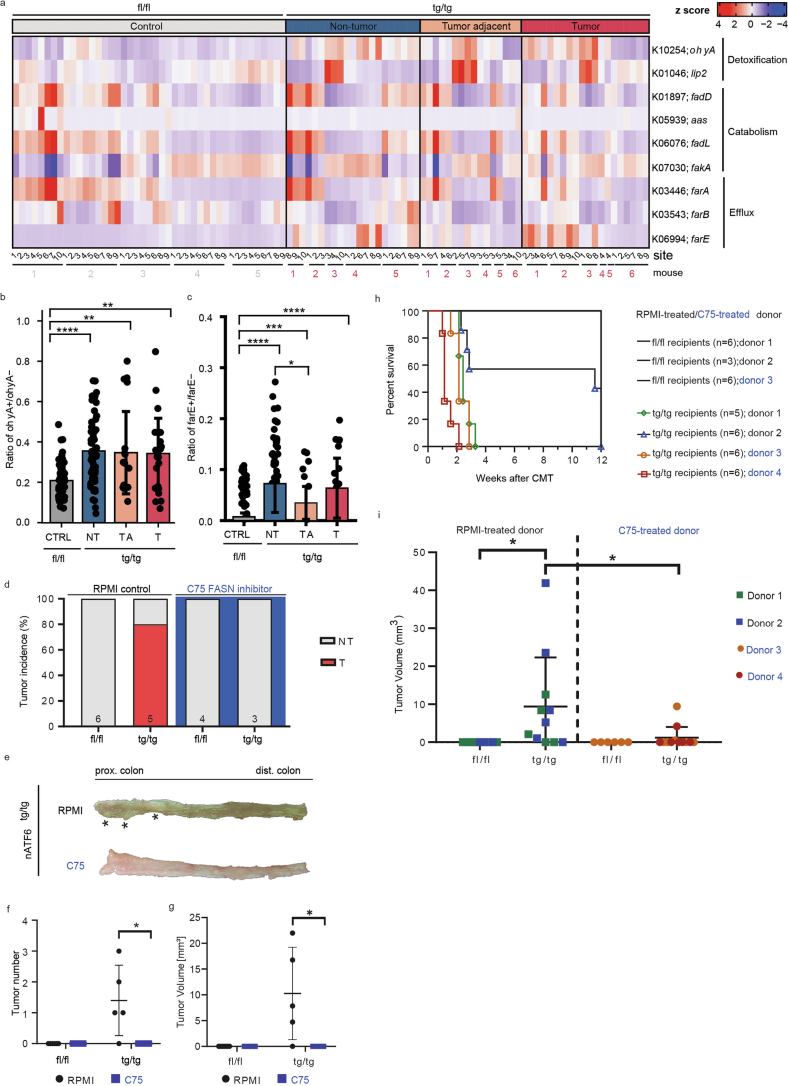
Bacterial lipid stress response and FASN inhibition. **a**, Heatmap of bacterial FA-response genes predicted by PICRUSt2 for fl/fl controls and non-tumour (NT), tumour-adjacent (TA) and tumour (T) mucosal phenotypes of tg/tg mice. Spatial sites along the colon and biological replicates (mouse) are numerically labelled underneath. Only tumour-susceptible sites (sites 1–10) are shown (n = 6 mice per group and 15–17 colonic sites per mouse). **b**, Ratio of ohyA positive to ohyA negative taxa across various mucosal phenotypes in fl/fl and tg/tg mice (n = 6 mice per group and 15–17 colonic sites per mouse). *P*-values were calculated by one-way ANOVA and Tukey’s test for multiple comparisons. Data are represented as mean ± s.d. **c**, Ratio of farE positive to farE negative taxa across various mucosal phenotypes in fl/fl and tg/tg mice (n = 6 mice per group and 15–17 colonic sites per mouse). *P*-values were calculated by one-way ANOVA and Tukey’s test for multiple comparisons. Data are represented as mean ± s.d. **d**, Tumour incidence (percentage) of SPF fl/fl and tg/tg mice after termination of the intervention with either RPMI (controls) or C75 FASN inhibitor. Grey represents no tumor, red represents tumor. Each bar shows the number of mice for each genotype. **e**, Representative macroscopic images of the colon of the tg/tg genotype on RPMI or C75. Marked with circles and asterisks are tumors identified in the control group. **f**, Tumour number in SPF fl/fl and tg/tg mice after intervention with RPMI (control, n = 6 fl/fl mice and n = 5 tg/tg mice) or C75 (n = 4 fl/fl mice and n = 3 tg/tg mice). Statistical significance was determined using a two-way ANOVA. Data are represented as mean ± s.d. **g**, Tumour volume in SPF fl/fl and tg/tg mice after intervention with RPMI (control, n = 6 fl/fl mice and n = 5 tg/tg mice) or C75 (n = 4 fl/fl mice and n = 3 tg/tg mice). Statistical significance was determined using a two-way ANOVA. Data are represented as mean ± s.d. **h**, Survival curve showing percent survival of fl/fl and tg/tg GF mice colonised with either RPMI-treated or C75-treated microbiota between 0–12 weeks of age. i, Tumor volume (mm^3^) in GF fl/fl and tg/tg mice after gavage with donor material from either RPMI (controls, n = 9 fl/fl mice and n = 11 tg/tg mice) or C75 (n = 6 fl/fl mice and n = 12 tg/tg mice) treated mice. Points are colored by donor. Statistical significance was determined using a two-way ANOVA. Data are represented as mean ± s.d. Statistical significance was calculated using pairwise T-tests and adjusted for multiple comparisons using the Benjamini-Hochberg procedure (p = 0.0317). A significant *p*-value < 0.05 is represented as an asterisk: *p < 0.05, **p < 0.01. ***p < 0.001, ****p < 0.0001. Source data

To examine whether these changes in microbiota community structure were associated with shifts in metabolite profiles, we performed a multi-omics data integration using sparse partial least squares discriminant analysis (sPLS-DA) of luminal untargeted metabolomic profiles and luminal and mucosal 16S data^24^. To delineate between genotype-driven and tumour-driven alterations, we performed two integrations that model genotype and phenotype separately. By sorting tg/tg discriminating features according to importance, we identified *Lactobacillus/Limosilactobacillus* and *Desulfovibrio* zero-radius OTUs (zOTUs) as well as multiple LCFAs and lysophospholipids as microbial and metabolite contributions, respectively (Fig. 4b). Metabolite contributions to phenotype discrimination were similar and comprised mostly LCFAs and lysophospholipids. However, microbial contributions differed greatly, with mostly zOTUs classified as *Parabacteroides* and *Romboutsia*, indicating the presence of tumours differentially modulates microbiota, compared to *Atf6* activation alone (Extended Data Fig. 8a). Next, to identify microbiota-correlated metabolites, we generated relevance networks using the network function of mixOmics DIABLO R package^24^. Importantly, several FAs, including an unidentified C20 hydroxy FA, nervonic acid (FA 24:1), docosapentanoic acid (FA 22:5) and docosatetranoic acid (FA 22:4), correlated positively with tg/tg discriminative zOTUs, particularly those classified as *Lactobacillus/Limosilactobacillus*, and additionally with *Desulfovibrio* in the mucosal dataset (Extended Data Fig. 8b,c and Supplementary Table 7). LCFA compounds are known to be toxic to bacteria, requiring adaptation mechanisms to allow growth in FA-rich milieus. Using PICRUSt2, we generated predictions of genomic content^25,26^, following a spatially resolved analysis of 16S rRNA amplicon sequencing-based bacterial profiles using individually defined 0.5 × 0.5-cm sites along the longitudinal colonic axis in fl/fl controls and tg/tg mice at the tumour (12 week) time point. We identified genes involved in bacterial responses to FAs in the proximal tumour-susceptible region of the colon, which are known to be involved in FA detoxification, catabolism and efflux (Extended Data Fig. 9a). Of this FA-response signature, bacterial oleate hydratase (*ohyA*) and the bacterial FA efflux pump (*farE*) were observed to be upregulated in tumour mice compared to controls (Extended Data Fig. 9a). *OhyA*-mediated FA hydroxylation enables growth of certain bacteria in lipid-rich environments^27^. Spatial mapping of log_10_-transformed relative abundance showed clear enrichment of *ohyA* across all sites in tg/tg mice compared to fl/fl controls (Fig. 4c). The relative abundance of *ohyA* was increased in tumour-bearing tg/tg mice compared to fl/fl controls (Fig. 4d). Furthermore, the ratio of *ohyA*-positive to *ohyA*-negative taxa was increased in all three mucosal phenotypes (non-tumour (NT), tumour adjacent (TA) and tumour (T)) in tg/tg mice, compared to fl/fl controls (Extended Data Fig. 9b). *FarE* constitutes a lipid efflux pump associated with antimicrobial FA resistance to limit the cellular accumulation of toxic FAs^28^. Similarly to *ohyA*, we observed an increased relative abundance across all tg/tg sites (Fig. 4e), and increased relative abundance of *farE* in tumour-bearing tg/tg mice (Fig. 4f). Furthermore, the ratio of *farE*-positive to *farE*-negative taxa was increased in all tg/tg mucosal phenotypes, compared to fl/fl controls (Extended Data Fig. 9c). Taken together, our findings clearly indicate an early pre-tumour microbial adaptation to the ATF6-altered FA-rich intestinal environment, to shape a tumour-associated bacterial lipid-response signature.

To understand the causal role played by FASN in colonic tumorigenesis in our nATF6^IEC^ mouse model, we exposed fl/fl and tg/tg SPF mice to biweekly intraperitoneal (i.p.) injections of the FASN inhibitor C75 from the age of 3 weeks (pre-tumour time point) until 12 weeks (tumour time point; Fig. 4g). Interestingly, FASN inhibition reduced tumour incidence in tg/tg mice from 80% to 0% with no tumours observed in the tg/tg colon (Extended Data Fig. 9d,e). In line with this, FASN inhibition was able to reduce tumour number and tumour volume in the colon of tg/tg mice (Extended Data Fig. 9f,g). To validate the link between FA synthesis and microbiota-dependent tumorigenesis in nATF6^IEC^ mice, we performed microbiota transfer experiments from C75-treated mice and RPMI-treated controls into GF tg/tg mice and fl/fl controls (Fig. 4g). Compared to fl/fl recipient mice, all tg/tg recipient mice showed reduced survival (Extended Data Fig. 9h). Strikingly, the tumour-promoting capacity of the C75-treatment-derived microbiota was reduced from a tumour incidence of 73% to 25% (Fig. 4h). In line with this, the microbiota from C75-treated mice reduced both tumour number and volume to levels comparable to tumour-free fl/fl controls (Fig. 4i and Extended Data Fig. 9i). Taken together, these findings clearly demonstrate that microbiota changes associated with ATF6-induced lipid alterations are causally involved in inducing a more aggressive tumorigenic phenotype in nATF6^IEC^ mice.

### LCFAs selectively activate the growth of tumour-related bacteria

**Fig. 5:**
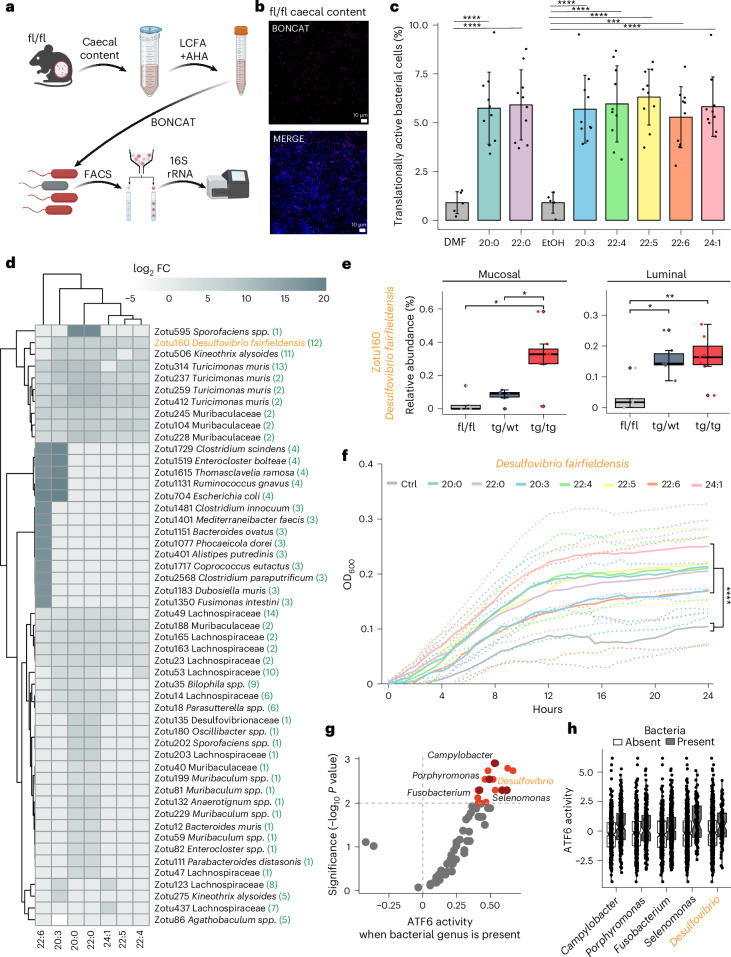
LCFAs selectively activate the growth of tumour-related bacteria. **a**, Schematic overview of BONCAT. Mouse caecal content was incubated in anaerobic conditions and stimulated with seven LCFAs, including arachidic acid (20:0), homo-γ-linolenic acid (20:3), docosanoic acid (22:0), docosatetraenoic acid (22:4), docosapentaenoic acid (22:5), docosahexaenoic acid (22:6) and nervonic acid (24:1) and along with the cellular activity marker l-azidohomoalanine (AHA). Translationally active bacterial cells were labelled by azide-alkyne click chemistry, sorted with FACS and sequenced by 16S rRNA gene amplicon sequencing. Created with BioRender.com. **b**, Representative confocal microscopic images of mouse caecal content stimulated with nervonic acid (24:1; *n* = 3 independent experiments and total of 5 biological replicates). Pink indicates active cells (BONCAT-Cy5); blue indicates all cells (DAPI); merge image. Scale bar, 10 µm. **c**, Percentage of translationally active bacteria after LCFA amendment. LCFAs were compared to their respective solvent control: 20:0 and 22:0 were compared to DMF, while 20:3, 22:4, 22:5, 22:6 and 24:1 were compared to ethanol (EtOh). *P* values were calculated by ANOVA and Tukey’s test for multiple comparisons (mean ± s.d.: 5.8% ± 1.6% for LCFAs in total; 0.9% ± 0.51% for the controls; ANOVA, *P* < 0.0001 for all comparisons, *n* = 80 total samples including seven LCFAs and two controls (DMF and EtOh), five biological replicates and two technical replicates). Error bars represent the s.d. of the mean. **d**, Heat map displaying log_2_-fold changes of significantly enriched or depleted zOTUs in the translationally active fraction compared to the unsorted group for each LCFA as calculated with the Wald test (*P* < 0.05, *n* = 160). All the zOTUs (51) listed in the heat map are significantly enriched or depleted. zOTUs were identified using the 16S-based ID tool of EzBioCloud. Numbers in parentheses correspond to the bar plots of Extended Data Fig. 10c where each barplot illustrates the shared and unique zOTUs between all LCFAs. **e**, Relative abundance of a *D. fairfieldensis* zOTUs, in luminal and mucosal communities comparing fl/fl, tg/wt and tg/tg mice (luminal *n* = 5 fl/fl mice, *n* = 6 tg/wt mice, *n* = 7 tg/tg mice; mucosal *n* = 5 fl/fl mice, *n* = 6 tg/wt mice, *n* = 7 tg/tg mice) at the 5-week time point. Boxes depict the IQR, whiskers extend to furthest non-outlier values (no more than 1.5 times the IQR), and the centre lines indicate the median. Statistical significance was calculated using pairwise *t*-tests and adjusted for multiple comparisons using the Benjamini–Hochberg procedure (mucosal: fl/fl versus tg/tg *P* = 0.014, tg/wt versus tg/tg *P* = 0.022; luminal fl/fl versus tg/wt *P* = 0.013, fl/fl versus tg/tg *P* = 0.0092). **f**, Growth curve of *D. fairfieldensis* in postgate medium, supplemented with different LCFAs. Each curve represents averaged values of *n* = 5 biological replicates in three technical replicates. LCFAs (20:0, 22:0, 20:3, 22:4, 22:5, 22:6 and 24:1) were added to postgate medium to a concentration of 10 µM each. The control (grey) contains only postgate medium with bacteria. All LCFA supplementations show significant increases in growth compared to the control (two-way ANOVA, Bonferroni test for multiple comparisons, *P* < 0,0001 for all com*p*arisons, *n* = 392 total samples for each LCFA and control at all time points as the mean of five biological replicates). Error bars shown as dashed lines represent the s.d. of the mean. **g**, Plot of ATF6 activity in individuals with CRC (TCGA database, *n* = 610 CRC cases) when bacterial genus is present (*x* axis) and the significance (*y* axis). Marked in orange/red are those genera significantly associated with ATF6 activity, with red dots representing classical CRC-associated genera. **h**, Plot of ATF6 activity in individuals with CRC (TCGA database, *n* = 610 CRC cases) when bacteria are absent (white) or present (grey) for the CRC-associated genera marked red in **g** (*P* values*:* Campylobacter = 0.00123, Desulfovibrio = 0.00507, Porphyromonas = 0.00287, Fusobacterium = 0.00507, Selenomonas = 0.00507). *P < 0.05, **P < 0.01, ***P < 0.001, ****P < 0.0001. Source data

**Extended Data Fig. 10:**
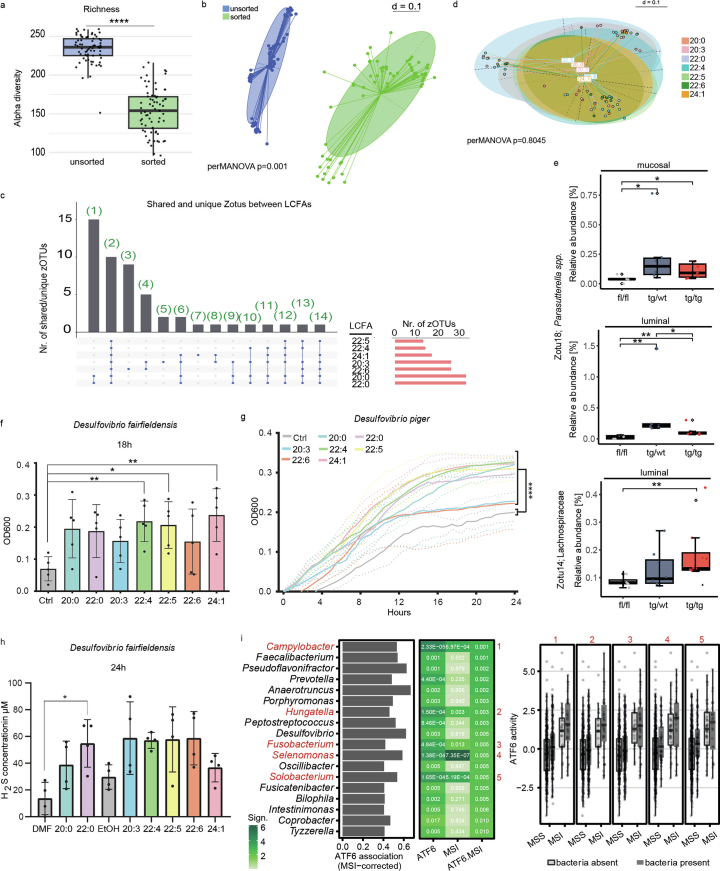
Bacterial responses to LCFAs. **a**, Alpha diversity richness showing significant difference between sorted and unsorted fraction (Wilcoxon test: p < 0.0001, n = 140 total samples including seven LCFAs sorted and unsorted fractions, 5 biological replicates and 2 technical replicates). Boxplot: boxplot medians (center lines), interquartile ranges (box ranges), whisker ranges. **b**, Beta-diversity MDS plot shows clustering of samples based on sorted and unsorted fractions (PERMANOVA, p = 0.001, n = 140 total samples including seven LCFAs sorted and unsorted fractions, 5 biological replicates and 2 technical replicates). Unsorted fraction= blue, sorted fraction= green. **c**, Venn diagram intersections shows 10 zOTUS shared between all LCFAs. 15 are shared between the saturated LCFAs (20:0 and 22:0), 9 are unique for 22:6 and 5 are shared between 22:6 and 20:3. The total size of each set is represented on the right barplot. The bottom plot represents all possible intersections, while their occurrence are shown in the top barplot. Each row corresponds to a set, and each column corresponds to one segment in a Venn diagram. A set is part of the intersection only when its cell is filled. Numbers in parenthesis on the top of each barplot represents which zOTUs belongs to each intersection. The corresponding zOTUS groups are highlighted in green in Fig. 5d. **d**, Beta-diversity MDS plot displayed the active fraction across the different LCFAs. No significant difference was found between LCFAs (PERMANOVA, p > 0.05, n = 70 total samples including seven LCFAs only sorted fraction, 5 biological replicates and 2 technical replicates). **e**, Relative abundance of zOTUs classified as *Parasutterella* (mucosal: fl/fl vs tg/tg p = 0.0017, fl/fl vs tg/tg p = 0.018, and luminal fl/fl vs tg/wt p = 0.008, fl/fl vs tg/tg p = 0.0057, tg/wt vs tg/tg p = 0.022) and Lachnospiraceae (luminal: fl/fl vs tg/tg p = 0.0025 s, in mucosal (top), mucosal (middle) and luminal (bottom) communities respectively, comparing fl/fl, tg/wt and tg/tg mice at the 5-week timepoint (n = 5 fl/fl, n = 6 tg/wt and n = 7 tg/tg mice). Boxes depict interquartile range (IQR), whiskers extend to furthest non-outlier values (no more than 1.5 x IQR), and the centre lines indicate the median value. Statistical significance was calculated using pairwise Wilcoxon-tests and adjusted for multiple comparisons using the Benjamini-Hochberg procedure. **f**, *Desulfovibrio fairfieldensis* growth in postgate medium, supplemented with different LCFAs, at 18 hours. Barplots represent averaged values of n = 5 biological replicates in three technical replicates. *P*-values were calculated by one-way ANOVA and Dunnet’s test for multiple comparisons (n = 40 total samples including seven LCFAs and controls, 5 biological replicates). Error bars represent standard deviation of the mean. **g**, Growth curve of *Desulfovibrio piger* in postgate medium, supplemented with different LCFAs. Each curve represents averaged values of n = 3 biological replicates in three technical replicates. Long-chain fatty acids (20:0, 22:0, 20:3, 22:4, 22:5, 22:6 and 24:1) were added to postgate medium to a concentration of 10 µM each. The control (gray) contains only postgate medium with bacteria. All LCFA supplementations show significant increase of growth compared to the control (two-way ANOVA, Bonferroni test for multiple comparisons, p < 0,0001 for all comparisons, n = 392 total samples for each LCFA and control at all timepoints as the mean of 3 biological replicates). Error bars shown as dotted line represent standard deviation of the mean. **h**, H_2_S concentration after 24-hour exposure of *Desulfovibrio fairfieldensis* in postgate medium to different LCFAs (10 µM). DMF and EtOH represent the respective solvent controls. Barplots represent averaged values of n = 4 biological replicates in three technical triplicates each. *P*-values were calculated by one-way ANOVA and Dunnet’s test for multiple comparison. Error bars represent standard deviation of the mean. **i**, Association of ATF6 activity with CRC-enriched genera (TCGA Database, n = 640 CRC cases) after correcting for MSI status, and correlation of the CRC-enriched genera (highlighted in red, 1–5) with ATF6 activity for MSS and MSI in the absence and presence of the respective bacteria. Boxes depict interquartile range (IQR), whiskers extend to furthest non-outlier values (no more than 1.5 x IQR), and the centre lines indicate the median value. A significant *p*-value < 0.05 is represented as an asterisk: *p < 0.05, **p < 0.01. ***p < 0.001, ****p < 0.0001. Source data

To assess whether LCFAs directly impact bacteria, we exposed the complex microbiota of 5-week-old fl/fl mice to previously identified (Fig. 3b and Supplementary Table 6) ATF6-associated LCFAs (24:1, 22:0, 20:0, 22:6, 22:5, 20:3 and 22:4). Our approach applied bioorthogonal non-canonical amino acid tagging (BONCAT) combined with FACS to identify translationally active bacteria in response to LCFA exposure (Fig. 5a). Confocal microscopy images displayed a significantly higher proportion of translationally active cells after LCFA supplementation compared to the controls (Fig. 5b and Supplementary Fig. 2). Microscopy images were confirmed with quantitative measure of translationally active bacterial cells exhibiting a higher percentage of translational activity compared to the controls (analysis of variance (ANOVA), *P* < 0.0001 for all comparisons; Fig. 5c). A distinct separation between sorted and unsorted communities along with a significant decrease of zOTUs in the sorted fraction, demonstrates a specific microbial response to LCFA exposure (PERMANOVA, *P* < 0.0001; Wilcoxon test: *P* < 0.0001; Extended Data Fig. 10a,b). In total, we identified 50 zOTUs that were significantly enriched in response to one or multiple LCFAs, while only one zOTU was depleted (Wald test, *P* < 0.05; Fig. 5d and Extended Data Fig. 10c). Strikingly, LCFAs selectively activated tumour-associated bacteria including those of the Desulfovibrionaceae family (for example, *Desulfovibrio fairfieldensis* and *Bilophila* spp.; Fig. 5d), while the overall microbiota profiles were similar in response to the different LCFAs (Extended Data Fig. 10d; PERMANOVA, *P* = 0.8045). Supporting the in vivo relevance of these findings, we examined the overlap between LCFA-enriched zOTUs and differentially abundant zOTUs at the pre-tumour time point, identifying *D. fairfieldensis* as one of three significantly enriched zOTUs in tg/tg mice at the pre-tumour time point in both the mucosal and luminal environments (Fig. 5e and Extended Data Fig. 10e). The higher translational activity in Desulfovibrionaceae was accompanied by a significant enhancement in bacterial growth when exposed to individual LCFAs, as indicated by the increased expansion of *D. fairfieldensis* (two-way ANOVA, *P* < 0.0001 for all comparisons; Fig. 5f and Extended Data Fig. 10f). A similar growth-promoting effect was observed for another *Desulfovibrio* isolate, *D. piger*, further confirming the ability of *Desulfovibrio* spp. to selectively grow in the presence of LCFAs (*P* < 0.0001 for all comparisons; Extended Data Fig. 10g). Additional zOTUs responding to the LCFA-rich environment included *Sutterella* spp., Lachnospiraceae, Muribaculaceae, *Turicimonas muris*, *Ruminococcus gnavus* and *Oscillibacter* spp. (Fig. 5d). The above findings demonstrate that LCFAs translationally activate specific bacteria, including tumour-associated taxa, providing a direct link between tumour-associated FA profiles and the selective expansion of bacterial taxa in mice.

As a sulfate-reducing bacterium, *Desulfovibrio* is capable of using inorganic sulfate as a terminal electron acceptor for anaerobic respiration. Using different substrates, such as lactate, and H_2_ as an energy source, sulfate-reducing bacteria are able to produce hydrogen sulfide (H_2_S). While H_2_S acts as an energy source in the healthy colonic epithelium, it becomes toxic to mammalian cells at high concentrations by inhibiting mitochondrial cytochrome c oxidase. Therefore, we measured the direct effect of LCFAs on *D. fairfieldensis*-related H_2_S production after 24-h exposure. We show a clear trend towards increased H_2_S production of *D. fairfieldensis* in response to nearly all of the tested FAs, reaching significance for the LCFA 22:0 (Extended Data Fig. 10h). Recent developments in identifying microbial signatures in the publicly available TCGA dataset^29^, allowed us to compare computationally inferred transcription factor activity using previously validated transcription factor–target gene relationships (RNA-seq data) and microbial profiles (identified in whole-exome sequencing data from the same samples) in individuals with CRC. In a hypothesis-free approach, ATF6 was identified among the most significant transcription factors, as calculated using a linear mixed-effects model, whose activities correlated positively with the presence of CRC-enriched genera, including Campylobacter, Porphyromonas, Fusobacterium, Selonomonas and Desulfovibrio (Fig. 5g,h). Both *ATF6* activity and bacterial profiles are strongly associated with microsatellite instability (MSI) status of the cancer, which could be a potential confounding factor, but we observed a strong correlation of the CRC-enriched genera with ATF6 activity even after correcting our association models for MSI status (Extended Data Fig. 10i). Of note is the observed overlap in specific tumour-associated and ATF6-associated taxa between mouse and human microbiota (Fig. 5d,e,g,h and Extended Data Fig. 10i), namely *D. fairfieldensis*, *Oscillibacter* spp., *Desulfovibrionaceae* spp. and *Bilophila* spp., supporting a causal link between ATF6-mediated LCFA synthesis and tumour-relevant changes in the microbiota.

## Discussion

CRC is the second leading cause of death worldwide^30^. Early and accurate diagnosis as well as additional treatment strategies are imperative in the management of this disease. As a fundamental mediator in the pathogenesis of CRC, the UPR^ER^ represents a promising host cellular mechanism for preventive and therapeutic intervention. While our mechanistic understanding of the role of ATF6 signalling in CRC remains limited, its expression has been associated with poor prognosis in individuals with CRC^7,8^. In the present study, we demonstrate the relevance of ATF6 expression in individuals with CRC across multiple independent German cohorts by identifying a clear subpopulation (19–38% LOCRC; 11% EOCRC) of individuals that present with high ATF6 expression, underlining the importance of ATF6 in CRC. Most importantly, we identified a mechanism through which ATF6-mediated changes in cellular lipid metabolism of the intestinal epithelium contribute to the development of tumour-promoting microbiota changes at an early disease stage.

We previously showed that chronic ATF6 activity specifically in mouse IECs (nATF6^IEC^) results in microbiota-dependent early-onset colonic adenomas^10^. The necessity of microbial triggers in tumour induction was shown via GF mice, which remained tumour-free^10^. Furthermore, the transfer of pre-tumour mouse caecal content into GF mice revealed the dysbiotic microbiota from tumour-susceptible mice to have an increased capacity to induce tumours^10^. Importantly, the development of dysbiosis in nATF6^IEC^ mice precedes tumour formation^10^, suggesting that tumour-independent mechanisms at early stages of the ATF6-driven pathogenesis initiate microbial changes. To ascertain these mechanisms, we set out to characterize the transcriptional response induced by chronic ATF6 activation in the presence (SPF) and absence (GF) of bacteria at the pre-tumour stage. We identify the FA metabolism KEGG pathway as one of the top-ranked metabolic pathways altered in tumour-susceptible SPF mice when compared to GF mice, and detect 23 upregulated FA metabolism-related genes. The majority are upregulated already in response to acute ATF6 activation, confirming FA alterations to be an early event occurring in direct response to ATF6 signalling in the presence of the microbiota. While the specific contribution of ATF6 to lipid metabolism is less well understood, the UPR^ER^ is known to play an important role in lipid metabolism^31–34^. Lipid metabolic rewiring forms a phenotypic hallmark of cancer, with altered lipidome profiles identified in tumours of numerous organs^18,35^. Ecker et al. recently identified a robust CRC tumour-specific triglyceride species signature that correlates with postoperative disease-free survival in a multi-cohort study spanning two clinical centres^23^. Moreover, gene expression of *FASN*, which forms a multi-enzyme complex responsible for catalysing FA synthesis, was prognostically detrimental^23^. Strikingly, the in vivo inhibition of FASN in nATF6^IEC^ mice results in the prevention of colon adenoma development. We show that our tumour-associated caecal metabolite signature mostly comprises lysophospholipids and LCFAs, with an overlap of several significantly altered LCFAs between tg/tg mice and CRC human tumour tissue, confirming the human relevance of the observations in our transgenic mouse tumour model. The absence of a significant correlation between the altered ATF6-associated LCFAs and clinical prognosis in our CRC cohort is likely attributed to the small sample size, and the stage at which LCFA profiling was conducted. Analyses were performed on human samples with advanced CRC, while our model proposes that ATF6 functions as an early driver that initiates metabolic restructuring and ultimately leads to tumorigenesis and therefore making it likely that alterations in LCFAs may not directly reflect disease prognosis at later stages. Analyses of mouse colonic tissue demonstrated increases in SAFAs and LCFAs. These findings are in line with a study profiling FAs in tissue and plasma of individuals with CRC and healthy controls, which identified a CRC-associated FA panel and found that LCFAs were the most increased FAs^36^. Our experimental approach in ex vivo organoids demonstrates that FA elongation is a direct consequence of ATF6 signalling, occurring in the absence of microbial signals.

A handful of studies have identified pro-oncogenic bacteria enriched in adenoma samples; however, questions remain over whether these taxa initiate disease, because adenoma formation induces metabolic alterations that likely impact the local microenvironment and alter microbial composition^37–39^. Our knowledge on which triggers may enable taxa to expand early on, and potentially become tumour-promoting, is sparse. We hypothesize that ATF6-driven lipid alterations impact the intestinal microbiota as an early event of tumorigenesis in nATF6^IEC^ mice. Our multi-omics data integration of 16S rRNA microbiota (luminal and mucosal) profiling and caecal metabolites identified several zOTUs, including those classified as *Lactobacillus/Limosilactobacillus* and *Desulfovibrio*, to be correlated with several LCFAs associated with our tumour-susceptible mice. Importantly, and in support of our hypothesis, PICRUSt2 predictions of genomic content confirm that the ATF6-driven FA-rich intestinal environment results in microbial adaptation in the form of a lipid-response signature that is associated with genes involved in FA detoxification, catabolism and efflux. Our data suggest a clear link between ATF6-induced lipid alterations and changes in the intestinal microbiota preceding tumour formation. Strikingly, faecal microbiota transplantation in GF mice using caecal content of FASN inhibitor-treated mice clearly demonstrated that the microbiota associated with ATF6-induced lipid alterations is causally involved in inducing a more aggressive tumorigenic phenotype in nATF6^IEC^ mice. To identify selective taxa of the intestinal microbiota responding to LCFAs, we here applied an approach to directly expose the microbiota of fl/fl control mice ex vivo to our identified LCFAs, showing that tumour-associated taxa are translationally activated in response to the LCFAs. Among these was *D. fairfieldensis*, which was significantly enriched in tg/tg mice at the pre-tumour time point in both the mucosal and luminal environments. In addition to CRC-associated lipid signatures, CRC-associated microbial signatures have also been identified^40,41^. In a hypothesis-free approach analysing transcription factor activity and microbial profiles in individuals with CRC, we show *ATF6* activity to positively associate with the presence of such a risk profile-associated bacterial genera, identifying a clear link between ATF6 signalling and the microbiota in CRC pathogenesis. Interestingly, and in line with our mouse data, we identified *Desulfovibrio* among the ATF6-associated CRC genera.

*Desulfovibrio* spp. have been implicated in disease onset through multiple mechanisms, including weakening of the gut barrier by downregulating tight junction proteins^42,43^, promoting inflammation via increased cytokine secretion^44^, and reducing sensitivity of CRC cells to chemotherapy by elevating serum *S*-adenosyl methionine levels^45^. These pathogenic effects are closely linked to their capability to produce the metabolite H_2_S, associated with mucus barrier disruption through cleavage of disulfide bonds^46,47^ and pro-inflammatory signalling^48^. Furthermore, in high concentrations H_2_S can exhibit effects directly linked to tumorigenesis, such as maintaining colon cancer cellular bioenergetics through protein persulfidation, supporting tumour growth and proliferation^48,49^. In addition, H_2_S was shown to promote angiogenesis and vasorelaxation, allowing increased nutrient and blood flow to the tumour site^50^. Taken together, these findings clearly support a mechanistic link for the tumour-promoting role of *D. fairfieldensis* in our transgenic model. We demonstrated that LCFAs not only trigger growth of *D. fairfieldensis* but also induce the functionally relevant metabolite H_2_S. Our findings provide clear evidence that LCFAs selectively activate bacterial members of the intestinal microbiota, including *D. fairfieldensis*, and likely due to the induction of bacterial anti-FA responses, such as FA detoxification and catabolism, *D. fairfieldensis* is able to sustain relevant energy metabolism and H_2_S release. The molecular mechanisms of *D. fairfieldensis* to take advantage of the tumour-related lipid milieu in the gut is under investigation.

**Fig. 6:**
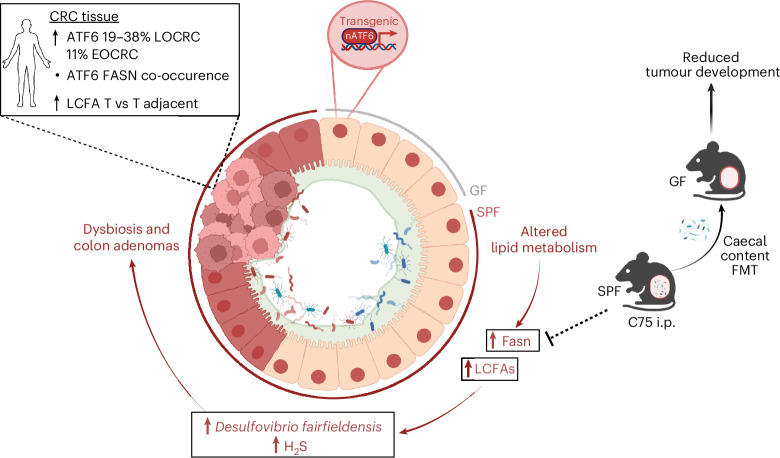
Chronic ATF6 signalling in the colonic epithelium alters lipid metabolism to select a tumour-promoting microbiota. Biallelic expression of activated ATF6 (p50 nuclear fragment) in IECs (nATF6^IEC^) induces spontaneous colon tumours in SPF but not GF mice. Mechanistically, biallelic SPF nATF6^IEC^ mice alter colonic lipid metabolism, including the upregulation of LCFAs and *Fasn*. Inhibition of FASN (C75 i.p.) prevents colon tumour formation in mice, and reduces the tumour-promoting potential of the intestinal microbiota (caecal content faecal microbiota transplantation (FMT)). Exposure of fl/fl control mouse microbiota to LCFAs ex vivo translationally activates tumour-associated bacteria, including *D. fairfieldensis.* Ex vivo exposure of *D. fairfieldensis* increases growth and H_2_S production. Humans with CRC show ATF6 upregulation, FASN co-occurrence and increased LCFAs in tumour (T) tissue. ATF6 activity links with CRC-associated microbiota in humans, including *Desulfovibrio*. nATF6, activated activating transcription factor 6; C75 i.p., intraperitoneal injection of the Fasn inhibitor C75. Created with BioRender.com.

In conclusion, we identify a clear mechanistic sequence of events in the initiation of colon tumorigenesis whereby ATF6 signalling drives a clinically relevant pathological response by altering lipid metabolism to induce microbial adaptation, ultimately leading to a tumour-relevant bacterial dysbiosis (Fig. 6). Although beyond the scope of this study, future work should focus on single-cell transcriptomics to characterize the ATF6 transcriptional response at the cellular level, as well as extending gnotobiotic mouse experiments with individual bacterial candidates, to identify causal tumour drivers and elucidate mechanisms of tumour initiation. We have not utilized an ATF6-KO model to further evaluate the role of ATF6 in carcinogenesis, because loss-of-function and gain-of-function models are not reciprocal in ATF6 biology. Complete loss of ATF6 disrupts both basal and stress-induced transcriptional programmes, often leading to compensatory activation of other UPR arms. Moreover, existing mouse models predominantly display small intestinal phenotypes and typically require chemical induction methods that directly alter the intestinal microbiota, rendering them unsuitable for studying the microbial contributions to CRC. Together, the above findings highlight new mechanistic findings in an exciting avenue of research bringing together cellular stress responses, lipid metabolism and the intestinal microbiota in the context of CRC. This evident triangular interplay presents potential targetable vulnerabilities in CRC that warrant future research efforts to identify potential future prevention and treatment strategies.

## Methods

### Ethics statement regarding animal experiments

All animal experiments, maintenance and breeding of mouse lines were approved by the Committee on Animal Health Care and Use of the state of Upper Bavaria (Regierung von Oberbayern; AZ ROB-55.2-1-54-2532-217-2014, AZ ROB-55.2-2532.Vet_02-20-58, AZ TVA 55.2-2532.Vet_02-18-149, AZ TVA 55.2-2532.Vet_02-18-121 and AZ TVA 55.2-2532.Vet_02-19-006) and performed in strict compliance with the EEC recommendations for the care and use of laboratory animals (Directive 2010/63/EU of the European Parliament and of the Council of 22 September 2010 on the protection of animals used for scientific purposes). The maximal burden (calculated as a score for the criteria: body weight, general body condition/appearance, behaviour and stool consistency) permitted by the authorities was never exceeded, with mice taken out of experiments and immediately euthanized after reaching predefined abortion criteria.

### Ethics statement regarding human samples

IHC staining and quantification of ATF6 were performed in three independent human cohorts: (1) a German large single-centre cohort comprising 1,004 individuals with CRC (median age, 69 years) who underwent surgical resection spanning two decades, for which 959 tissue samples could be quantified (cohort 1)^11^; (2) a German cohort consisting of 104 individuals with CRC followed up over 20 years (mean age, 66 years), for which 50 human samples could be quantified (cohort 2); and (3) a German single-centre cohort comprising more than 1,500 resected CRC human samples spanning approximately 25 years (mean age 70 years), for which 55 EOCRC (age < 50 years) and 256 LOCRC (age > 50 years) samples were obtained and could be quantified (cohort 3). The use of surgically resected human tissue samples was approved by the local ethics committee of the Technical University of Munich (TUM; ref. no. 252/16s, cohort 1), by the ethics committee of the Medical Faculty of TUM (ref. no. 1926/7, 375/16S and 2022-169-S-KH, cohort 2) and by the local ethics committee of the University Hospital Schleswig-Holstein (ref. no. A 110/99, cohort 3). CRC cohort 3 tissue specimens were supplied by the biobank TRIBanK (Translational Interdisciplinary Biobank Kiel) together with the Institute of Pathology, University Hospital Schleswig-Holstein, Kiel, Germany. Liquid chromatography–mass spectrometry (LC–MS) analyses were performed in a fourth German human cohort consisting of 259 individuals (mean age, 60 years) diagnosed with EOCRC (age < 50 years) and LOCRC (age >50 years) and included one tumour tissue (T) and one adjacent tissue (T adjacent) for each individual (cohort 4). The use of surgically resected human tissue samples was approved by the local ethics committee of the Medical School – The Christian-Albrechts-University of Kiel (ref. no. A 156/03), the ethics committee of the Medical School of the University of Rostock (ref. no. A 2019-0187) and the ethics committee of the EMBL (ref. no. 2024/HE000062 - Mi-EOCRC). For all human cohorts in this study, both sexes were deliberately included, with slightly more male cases, corresponding to the incidence in the overall population. Sex-dependent differences were not observed in our data. Human samples from all cohorts were obtained after prior informed written consent. Information on sex was acquired by self-reporting. Individuals did not receive any financial compensation for providing samples in this study.

### Generation of ATF6 overexpressing (TG) and KO Colo800 cell lines

To generate cells overexpressing the activated form of human ATF6 (nATF6, AA 1-386), its coding sequence (CDS), along with an HA-tag, was cloned into the retroviral vector MSCV-linker-IRES-GFP, creating MSCV-nATF6-IRES-GFP. Colo800^TG^ cells (ATF6-TG) and Colo800^WT^ control cells (Ctrl) were produced by transducing parental Colo800 cells with retroviral particles derived from the MSCV-nATF6-IRES-GFP and the parental MSCV-linker-IRES-GFP vector, respectively. Viral particles were generated by transfecting Phoenix GP cells (American Type Culture Collection, CRL-3215) with either the MSCV-nATF6-IRES-GFP or the MSCV-linker-IRES-GFP plasmid together with VSV-G (Clontech) vector. Transduced cells were expanded and sorted for GFP expression using a FACS Aria II flow cytometer (BD Biosciences). Colo800 ATF6-KO cells were generated by transfecting Colo800 cells with vectors derived from pSpCas9(BB)-2A-Puro (PX459) V2.0, using Lipofectamine 3000 (Thermo Fisher), followed by puromycin selection (10 μg ml^−1^ for 3 days). Single guide RNA sequences and primers for verifying indel formation were designed using the CRISPOR.org webtool. Control cells for the ATF6-KO line were transfected with the PX459 V2.0 vector lacking any single guide RNA insert. Successful indel formation was confirmed by TIDE assay analysis.

### IHC staining and quantification of ATF6

TMA cores and Colo800 cells were immunohistochemically stained with an anti-ATF6 antibody (Sigma-Aldrich, HPA-005935, lot number D179431, polyclonal, host rabbit) diluted at a 1:100 ratio, following antigen retrieval in an EDTA buffer (30 min, pH 9), or an anti-FASN antibody (LS Bio, LS-B3636; lot number, 27052/200 μl, polyclonal, host rabbit) or anti-GRP78 (Cell Signaling, 3177 s, lot number 10, clone C50B12, host rabbit) diluted at a 1:200 ratio following antigen retrieval in a citrate buffer (20 min, pH 6). Nuclei were counterstained with haematoxylin (Waldeck). Stainings were performed using the Leica Bond RXm (Leica) and scanned using the Aperio AT2 (Leica Biosystems). ATF6-, GRP78- and FASN-stained TMA cores were quantified using the QuPath Software^12^. Cells were identified through positive cell detection of the haematoxylin-positive nucleus. ATF6 staining intensities for nuclear and cytoplasmic regions were classified according to defined thresholds of the DAB OD maximum value and DAB OD mean, respectively. FASN and GRP78 staining was quantified based on cytoplasmic regions only. The object classifier was trained and applied to distinguish between epithelial cells and stromal cells. Exported epithelial H-scores (capturing both the intensity and the percentage of stained cells) for the nucleus and the cytoplasm were used for further human cohort analyses.

### ATF6 expression analysis using AI

Whole-slide images were segmented using QuPath into individual cores and scaled down to 312 × 312 pixels. This preprocessing step yielded a dataset of 176 TMA cores from a cohort of 50 individuals (cohort 2). For our deep learning model, a ResNet18 was trained (cs.CV)^51^, freezing all layers except the final convolutional and fully connected layers for the classification task and freezing the first layer for the regression task. This allowed leveraging pretrained features while fine-tuning the model’s output layers for our specific task. To improve model generalization, data augmentation techniques were applied, including random horizontal and vertical flips and random rotations. In the final layer, dropout regularization was incorporated to prevent overfitting. A fivefold cross-validation was conducted to evaluate our model’s performance. Each fold used data from 40 individuals for training and 10 individuals for validation. Throughout cross-validation, the best models were recorded based on their accuracy (classification task) or loss (regression task) in the validation dataset. Their weights were preserved for subsequent use. In our final prediction task of classifying samples as ATF6-high or ATF6-low, a weighted average ensemble strategy was used by combining the predictions of the five best models. In a final evaluation step, our ensemble’s predictions were tested on an entirely independent cohort comprising 918 individuals (cohort 1).

### Transgenic nATF6^IEC^ mice (SPF and GF)

nATF6^IEC^ mice (C57BL/6N ATF6 mice crossed with C57BL6/J VillinCre mice) were generated as previously described^10^ and housed under SPF and GF conditions (12-h light–dark cycles, 24–26 °C and 50% humidity) at the Technical University of Munich (Weihenstephan, Germany). All mice were fed a standard chow diet (autoclaved V1124-300; Ssniff, Soest, Germany) ad libitum. Mice were cage-separated according to sex but not according to genotype. Both sexes were used for all experiments. For the determination of the tumour phenotype, mice were euthanized and the colon excised. Presence and number of tumours was recorded for each mouse as macroscopically observed. Tumour size was measured using a centimetre ruler, recording the length, height and depth. No statistical methods were used to predetermine sample sizes, but our sample sizes are similar to those reported in previous publications^10^. Animals were assigned to their respective experimental groups according to genotype and age, independently of sex and without randomization. Data collection and analysis were not performed blind to the conditions of the experiments.

### Isolation of mouse primary colonic IECs

Colonic tissue was longitudinally opened, and transferred to 20 ml DMEM (Gibco) containing 10% fetal calf serum, 1% l-glutamine and 0.8% antibiotics/antimycotics (IEC isolation medium) supplemented with 1 mmol l^−1^ dithiothreitol (Roth). The tissue was vigorously vortexed for 1 min, incubated under continuous shaking (37 °C, 15 min) and vortexed again for 1 min. The cell suspension was centrifuged (7 min, 300*g*) and the cell pellet was resuspended in 5 ml IEC isolation medium. After vortexing for 1 min, the remaining tissue pieces were incubated in 20 ml phosphate-buffered saline (PBS; 10 min, 37 °C) containing 1.5 mM EDTA under continuous shaking. The cell suspension was vortexed again followed by another centrifugation to pellet the cells. Both cell suspension fractions were combined and purified by centrifugation through a 20%/40% (in medium/PBS) discontinuous Percoll gradient (GE Healthcare Life Sciences) at 600*g* for 30 min. IEC cells at the interface between the two Percoll phases were collected and washed twice (with medium/PBS). Isolated IECs were lysed in RA1 RNA lysis buffer (Macherey-Nagel).

### RNA isolation and library construction

RNA of colonic IECs was isolated according to the manufacturer’s instructions (NucleoSpin RNAII kit, Macherey-Nagel) and measured by NanoDrop ND-1000 spectrophotometer (Thermo Fisher Scientific). TruSeq stranded Total RNA kit (Illumina) was used for the construction of strand-specific sequencing libraries from the isolated colonic IEC RNA. Sequencing was performed on an Illumina HiSeq 2500 (75-nucleotide paired-end reads for each run, 1/10 lanes per sample).

### RNA-seq preprocessing

After initial trimming of reads using trim_galore (version 0.6.4) to satisfy a Phred score of at least 30, RNA-seq data were processed with the ‘nf-core/rnaseq‘ pipeline (version 1.4.2) and aligned to the GRCm38 genome using the ‘STAR‘ aligner (version 2.6.1 d). Quantification of transcripts was performed using Salmon (version 0.14.1). All sequence properties were inspected in MultiQC and passed the quality thresholds.

### RNA-seq functional analysis

Gene-set enrichment analysis (GSEA) was performed using the differential expression results ranked by *t*-value^52^. The KEGG gene set was downloaded from the Molecular Signatures Database^52–54^. Gene names were translated into mouse gene names before the analysis using scibiomart (v1.0.2), a wrapper around the API from BioMart. GSEA was performed using the packages fgsea^55^ (v1.24.0) and GSEABase^56^ (v1.60.0). Next, we performed regulatory clustering based on regulatory rules (Supplementary Table 3) using the sircleR package (https://github.com/ArianeMora/SiRCleR/) function sircleRCM_2Cond^13^. In brief, each gene was assigned to ‘UP’ (log_2_FC > 0.5, *P* adj. < 0.05), ‘DOWN’ (log_2_FC < −0.5, *P* adj. < 0.05) or ‘no change’ (0.5 > log_2_FC > −0.5 and/or *P* adj. > 0.05) based on its expression change between fl/fl and tg/tg. The background method chosen was ‘C1 & C2’, which means a gene had to be detected in both conditions to be included in the clustering. Over-representation analysis was performed on each cluster using clusterProfiler (v4.6.2)^57^ with the KEGG gene set as well as the Dorothea TF-regulon confidence ‘A, B, C’^14,15^. Plots were generated using the EnhancedVolcano package^58^ (v1.16.0), ggplot2 (ref. ^59^; v3.4.2), ggupset^60^ (v0.3.0) and enrichplot^61,62^ (v1.18.4).

### Untargeted metabolomics of mouse caecal content or tissue

Sample preparation was performed as previously described^63^. Briefly, mouse caecal content (~20 mg) or tissue (~25 mg) was mixed with 1 ml methanol-based extraction solvent in a 2 ml bead beater tube (CKMix 2 ml, Bertin Technologies) filled with ceramic beads (1.4 mm and 2.8 mm ceramic beads). Samples were homogenized using a bead beater (Precellys Evolution, Bertin Technologies) supplied with a Cryolys cooling module (Bertin Technologies, cooled with liquid nitrogen) three times for 20 s, at 7,322*g*. The resulting suspension was centrifuged for 10 min, at 6,010*g*. Finally, 100 μl of supernatant was mixed with 20 μl internal standard solution (7 µmol l^−1^) and injected into the LC–TOF-MS system for untargeted analysis. Untargeted LC–TOF-MS analysis was carried out as described in Metwaly et al.^63^. Briefly, untargeted analysis was performed on an ExionLC ultrahigh performance liquid chromatography (UHPLC) system (Sciex), connected to a 6600 TripleTOF instrument (Sciex) operating in positive and negative electrospray mode and calibrated using ESI positive and negative calibration solutions (Sciex). UHPLC phase separation was performed in reverse as well as hydrophilic interaction stationary phase (HILIC), on a Kinetex C18 column (Phenomenex) and ACQUITY BEH Amide column (Water), respectively. Mass spectrometry was performed using SWATH mode, with fragment spectra recorded in high-sensitivity mode. Metabolomics data processing was performed as described previously^63^. Briefly, raw data files were converted into Reifycs Abf (analysis base file) files and subsequent untargeted peak picking was performed using MS-DIAL software (v3.5274)^64^. Alignment was performed across all samples, and the peak areas of individual features were exported for further analysis using the R statistical software environment. Peak normalization was based on quality-control samples, using the method described in Wehrens et al.^65^. All features were combined into a single table and further normalized according to sample weight, before analysis.

### FA quantification

For FA quantification in mouse tissues and investigation of eicosanoic acid elongation in organoids, FA methyl esters were generated by acetyl chloride and methanol treatment, and extracted with hexan^66^. Total FA analysis was carried out using a Shimadzu 2010 GC–MS system (Duisburg, Germany). FA methyl esters were separated on a BPX70 column (SGE, 10-m length, 0.10-mm diameter, 0.20-μm film thickness) using helium as the carrier gas. The initial oven temperature was 50 °C and was increased at 40 °C min^−1^ to 155 °C, 6 °C min^−1^ to 210 °C, and finally 15 °C min^−1^ to 250 °C. D3-eicosanoic acid and its elongation products in organoids were quantified by single-ion monitoring of specific fragment ions (D3-eicosanoic acid, 329 *m/z*; D3-docosanoic acid, 357 *m/z*). The internal standard was C21:0-iso.

### Untargeted metabolomics of CRC human tissue—sample preparation

Solid tissues were prepared for LC–MS analysis by organic solvent extraction. In brief, 200 μl of 0.1 mm zirconia/silica beads (BioSpec Products) and 500 µl of organic solvent (acetonitrile:methanol, 1:1) supplemented with an internal standard mixture were added to 70–100 mg of pre-weighed solid material. Internal standard mixture consisted of phenylalanine-*d5*, tryptophan-*d5*, ibuprofen-*d4*, tolfenamic acid-*d4*, estriol-*d3*, diclofenac-*d4*, warfarin-*d5*, oxfendazole-*d3*, chloramphenicol-*d5*, nafcillin-*d5* and caffeine-*d9*, each to a final concentration of 80 nM. The material was homogenized by mechanical disruption with a bead beater (BioSpec Products) set for 2 min and 30 s on the highest setting at room temperature. After incubation for at least 1 h at −20 °C, samples were centrifuged (3,220*g*, 4 °C) for 15 min. For C18 LC–MS acquisition, 15 µl of supernatant was diluted with 15 µl H_2_O.

### Untargeted metabolomics of CRC human tissue—LC-MS data acquisition

Chromatographic separation was performed by reversed-phase chromatography using an Agilent 1200 Infinity UHPLC system. The qTOF instrument (Agilent 6550) was operated in negative scanning mode (50–1,700 *m/z*). Online mass calibration was performed using a second ionization source and a constant flow of reference solution (*m/z* 112.9857 and 1033.9881). InfinityLab Poroshell HPH-C18 column (Agilent, 2.1 × 100 mm, 1.9-µm particle size) was used for chromatography. LC solvent A was water with 0.1% formic acid (vol/vol) and solvent B was methanol with 0.1% formic acid (vol/vol). The column operated at 45 °C. In total, 5 µl of sample was injected at the following gradients: 0 min 5% B; 0.1 min 5% B; 5.50 min 95% B; 6.49 min 95% B; 6.5 min 5% B. The time between injections was 0.5 min. The qTOF was operated in negative scanning mode (50–1,700 *m/z*) using the following source parameters: VCap, 3,500 V; nozzle voltage, 2,000 V; gas temperature, 225 °C; drying gas, 13 l min^−1^; nebulizer, 20 psig; sheath gas temperature, 225 °C; sheath gas flow, 12 l min^−1^. Tandem mass spectrometry analysis (LC–MS/MS) was performed for FAs using the chromatographic separation and source parameters described above and the targeted-MS/MS mode of the instrument with a preferred inclusion list for parent ion with 20 ppm tolerance, iso width set to ‘narrow width’ and collision energy to 10, 20 or 40 eV.

### Semi-targeted metabolomics of CRC human tissue LC–MS data processing

An in silico library of 134 FAs was mined against C18-MS negative data for the extraction of area values of FA-putative features (Supplementary Table 8). The area under the curve from the targeted analysis was integrated using the MassHunter Quantitative Analysis Software (Agilent, version 7.0) based the accurate high-resolution mass of these reference analytes and the following parameters: signal threshold, 50,000; mass tolerances, 0.002 amu or 20 ppm; retention time tolerance, 0.2 min. The resulting table was further normalized in R 4.4.1 using RStudio/2023.06.1 according to established protocols^67,68^.

### Validation of significant FAs of CRC human tissue

To ensure the annotation of the significant FAs, we performed level-1 metabolite annotation, which is the direct comparison of an authentic chemical standard analysed under identical analytical conditions. For this, we used standards of docotetranoic acid, docopentanoic acid and docohexanoic acid, as well as human CRC samples that have shown high abundance of these metabolites. Correct annotation occurs when all three orthogonal properties of the metabolite of interest (retention time, high-resolution mass and fragmentation mass spectrum) are similar between the analyte and the CRC sample.

### Primary crypt isolation and intestinal organoid culture from mice

Intestinal crypts from mice were isolated following digestion with EDTA (30 min; 4 °C) and mechanical dissociation. The supernatant was filtered through a 70-μm cell strainer, pelleted by centrifugation and resuspended in Matrigel (BD Biosciences). Matrigel-organoid domes (25 µl) were plated in 48-well plates (Eppendorf) together with 250 µl IntestiCult Organoid Growth Medium (STEMCELL Technologies) and maintained in a humidified 5% CO_2_ atmosphere at 37 °C. Organoids were passaged every 7 days by mechanical disruption using a 1-ml syringe with a 20-gauge needle, pelleted by centrifugation, and embedded in fresh Matrigel.

### Induction of ex vivo recombination and FA stimulation of intestinal organoids

Intestinal organoids from nATF6 Vil-Cre^ERT2^ (fl/fl^ERT2^ and tg/tg^ERT2^) mice^10^ were passaged 3 days before the start of the experiment and cultivated in self-made crypt culture medium (CCM; advanced DMEM/F12 medium (Gibco), 2 mM GlutaMax (Gibco), 10 mM HEPES, penicillin, streptomycin and amphotericin (all Sigma-Aldrich) supplemented with N2, B27 (both Gibco), 1 mM *N*-acetylcysteine (Sigma-Aldrich), 50 ng ml^−1^ EGF (ImmunoTools), 100 ng ml^−1^ noggin and 0.5 µg ml^−1^ R-spondin 1 (both PeproTech)). *ATF6* overexpression in intestinal organoids was induced by adding 500 nM (Z)-4-hydroxytamoxifen (4-OHT; LKT) to the culture medium for 24 h. After induction, organoids were exposed to eicosanoic 20,20,20-D3 acid (30 µM; Larodan) in CCM for 24 h. Organoids were subsequently collected in 0.2% SDS in double-distilled H_2_0.

### Sampling of mouse caecal content and colonic mucosa-associated microbiota

Caecal content was collected immediately after euthanization and stored at −80 °C before DNA extraction. The mouse colon was excised and opened longitudinally before clearing of colonic content using a sterile needle and sterile PBS washes. Tissue sections were excised using a sterile scalpel pretreated with DNA Away (Fisher Scientific) to destroy contaminating DNA fragments. For spatial microbiota analyses, the entire colon was laid on a grid and dissected into individual 0.5 cm × 0.5 cm sections and subsequently frozen at −80 °C before DNA extraction. All steps were carried out in a laminar flow cabinet.

### Microbial DNA extraction from mouse caecal content and colonic tissue

DNA extraction from frozen caecal content was carried out using a modified version of the protocol described by Godon et al.^69^. Briefly, caecal samples were homogenized by vortexing and transferred to autoclaved screw-cap microcentrifuge tubes containing 500 mg silica beads (0.1 mm Carl Roth) and kept on ice. To this, 600 μl of DNA stabilizer (Macherey-Nagel), 250 μl 4 M guanidine thiocyanate and 500 μl 5% *N*-laurolyl-sarcosine was added. Samples were incubated under moderate shaking (700 rpm) for 1 h at 70 °C. Lysis was achieved via mechanical disruption with a FastPrep 24-bead beater (MP Biomedicals) using three 40-s cycles at a speed of 6.5 m s^−1^. Around 15 mg polyvinylpolypyrrolidone (PVPP; Sigma-Aldrich) was added to the homogenate to remove phenol contamination, before centrifugation at 15,000*g* for 3 min at 4 °C. The resulting supernatant was recovered, and centrifuged again under the same conditions, resulting in a clear supernatant containing lysed bacterial cells. To this mixture, 10 mg ml^−1^ RNase was added to degrade bacterial RNA and incubated at 37 °C for 30 min under constant shaking (700 rpm). DNA extraction from colonic tissue was performed using enzymatic digestion. Where possible samples were extracted using the same kit batch, to limit differences in inherent kit contamination between different batches^70^. Tissues were placed in 180 μl sterile lysis buffer (20 Mm Tris/HCL, 2 Mm EDTA, 1% Triton X-100; pH 8 supplemented with 20 mg ml^−1^ lysozyme) and incubated for 1 h in a shaking incubator (950 rpm) at 37 °C. Around 10 mg ml^−1^ Proteinase K (Macherey-Nagel) was then added, and samples were incubated for 1–3 h (until complete lysis of the tissue was obtained) at 56 °C under moderate shaking (950 rpm). The resulting genomic DNA was then purified using the NucleoSpin gDNA clean-up kit (Macherey-Nagel), according to the manufacturer’s instructions. The concentration and purity of extracted genomic DNA were determined using a Nanodrop ND-1000 Spectrophotometer. Samples were stored at −20 °C before sequencing.

### 16S rRNA gene amplicon sequencing

High-throughput amplicon sequencing of the 16S rRNA gene was carried out as previously described^63^. In brief, the V3 and V4 hypervariable regions were amplified via a two-step protocol, using the 341f and 785r primer pair (341f 5′-CCTACGGGNGGCWGCAG-3′, 785r 5′-GACTACHVGGGTATCTAATCC-3′), with 10 × 15 cycles for caecal content samples and 15 × 15 for mucosal samples. Samples were barcoded with a double index, according to Kozich et al.^71^. The resulting amplicons were purified using AGENCOURT AMPure XP Beads (Beckman Coulter), pooled in equimolar ratios and then sequenced on an Illumina MiSeq system (Illumina), in paired-end mode (2 × 275 bp). Raw reads were processed using IMNGS, which wraps the UPARSE/USEARCH software pipeline^72,73^. Sequences were demultiplexed, trimmed to first base with a quality score <3 and merged. To remove spurious sequences, a length filter was applied based on the expected amplicon size of 444 bp, removing those smaller than 300 bp and larger than 600 bp. The resulting paired sequences were then dereplicated and denoised using UNOISE3, to generate zOTUs, reflecting true biological sequences^74^. Taxonomy was assigned using the RDP classifier version (2.11)^75^. Samples with less than 5,000 total reads were excluded from further analysis. Since low biomass samples are susceptible to contamination, which can lead to spurious conclusions, all mucosal samples were further processed with the R package ‘decontam’ to remove putative contaminants^76^.

### Supervised classification of 16S rRNA-seq

Supervised classification of mucosal and luminal microbial profiles in non-tumour (fl/fl and tg/wt) and T (tg/tg) mice was performed using the SIAMCAT R package, utilizing random forest (RF), ridge regression (RR), LASSO (L), elastic-net (E) and L1-penalized regression (LL), which were chosen based on the ease of interpretation of model output^77,78^. To account for differences in sample size between mucosal and luminal data, mucosal data were randomly subsampled to match luminal data. Before model training, low prevalent features—defined as those present in less than one-third of samples—were removed, and the data were transformed using a centred log-ratio transform. All models were trained using fivefold cross-validation, or in case of smaller sample size (<50 total samples), leave-one-out cross-validation, each with five rounds of resampling.

### Multi-omics integration

Integration of metabolomic and microbiota data was performed on log-transformed, scaled and centred log-ratio-transformed metabolite and zOTU matrices, respectively. General associations between the microbiota (luminal and mucosal) and metabolites were identified using a multi-block sPLS-DA model with seven components and tenfold cross-validation, as implemented in the DIABLO framework of the mixOmics R package^24,79^.

### Functional potential prediction

To calculate predicted functional profiles based on 16S rRNA-seq data, we utilized PICRUSt2 (version 2.3)^25^. FASTA files of representative sequences and minimum sum scaled zOTU tables were used as input for the command: picrust2_pipeline.py, which runs the full PICRUSt2 pipeline, aligning and placing the sequences into a reference tree, calculation of 16S copy number, Enzyme Commission and KEGG orthologue abundances, adjustment of these by 16S abundance and finally infers MetaCyc pathways by collapsing Enzyme Commission numbers according to their associated metabolic pathway. All PICRUSt2 data were generated in a high-performance computing environment, utilizing the Linux-cluster system at the Leibniz Rechenzentrum, Garching.

### Intraperitoneal injections of mice with FASN inhibitor C75

The FASN inhibitor C75 (Biomol) was dissolved in RPMI 1640 (Sigma-Aldrich) to a final concentration of 1.5 mg ml^−1^, aliquoted and stored at −20 °C until used. C75 or RPMI was injected i.p. (10 ml per kg body weight) biweekly, starting at the age of 3 weeks until 12 weeks of age (tumour time point). When necessary, mice were given a subcutaneous depot (1:1 mixture (vol/vol) of 5% glucose and Ringer’s lactate (B. Braun)) to alleviate potential side-effects of C75. Mice were euthanized with CO_2_ at the age of 12 weeks or when abortion criteria were met.

### Faecal microbiota transfer into GF mice

Caecal content from C75-treated mice and RPMI-treated controls was instantly suspended at a 1:10 weight/volume ratio in filter-sterilized PBS/40% glycerol and stored at –80 °C. For gavage, caecal content solutions were centrifuged (3 min, 300*g*, 4 °C) to pellet debris, followed by centrifugation (10 min, 8,000*g*, 4 °C) to collect micro-organisms. This fraction was resuspended in an equal volume of sterile PBS. Each recipient mouse (tg/tg or fl/fl) was gavaged with 100 μl of the bacterial suspension at the age of 4 weeks. Recipient mice were housed in microbiota-specific isolators and euthanized after 12 weeks, or when abortion criteria (as defined in ethical proposals) were fulfilled.

### Ex vivo anaerobic incubations with LCFAs in caecal content of fl/fl mice

Caecal content was collected from ten wild-type mice at 5 weeks of age and immediately transferred to an anaerobic chamber (10% H_2_, 90% N_2_). To ensure sufficient material, caecal contents of two mice of the same genotype and litter were combined. Caecal content (200–300 mg each) was homogenized in 1× PBS and filtered through a 40-μm filter (Corning, Germany) to remove larger particles. The samples were then transferred to sterile Falcon tubes containing a final concentration of 50 µM of the cellular activity marker l-azidohomoalanine (AHA; Baseclick) and between 400 pM and 50 nM of seven LCFAs, including FAs 20:0, 20:3, 22:0, 22:4, 22:5, 22:6 and 24:1, dissolved in either ethanol or DMF at a final concentration of 1%. Specifically, ethanol and DMF were included as solvent controls for FAs 20:3, 22:4, 22:5, 22:6, 24:1, 20:0 and 22:0, respectively, while 2 mg ml^−1^ glucose was the positive control for each experiment. Samples were incubated under anaerobic conditions for 6 h. After incubation, a fraction of the samples was frozen for DNA extraction, and other aliquots were washed twice with PBS and fixed in 1:1 ethanol for further analysis by FACS^80^.

### BONCAT and bacterial cell quantification

To assess the ability of gut microbiota to utilize LCFAs, we applied BONCAT, a fluorescence-based single-cell labelling technique. BONCAT is based on the incorporation of the non-canonical amino acid AHA followed by fluorescence labelling of AHA-containing proteins via azide-alkyne click chemistry^81^. The click chemistry reaction was carried out on microscopy slides, as previously described^81^. Samples were counterstained with DAPI. Microscopy pictures were captured using a confocal microscope (Olympus, Fluoview, FV10i) and processed with Fiji software^82^ (Supplementary Fig. 1). Translationally active cells were quantified with flow cytometry using absolute counting beads (CountBright, Invitrogen, Thermo Fisher Scientific) according to the manufacturer’s instructions^80^.

### BONCAT-FACS and DNA extraction

BONCAT combined with FACS was used to identify bacteria capable of utilizing LCFAs. Samples were processed according to ref. ^81^. Briefly, ethanol-fixed samples were initially washed with 1× sterile PBS, resuspended in 96% ethanol, and centrifuged. The BONCAT dye solution was then added, and the samples were incubated for 30 min in the dark at room temperature^81^. Following incubation, the samples were washed three times with 1× sterile PBS and filtered through a 35-µm nylon mesh using BD tubes (12 × 75 mm, Corning) before sorting. Cy5-positive bacterial cells were sorted using a FACS Melody instrument (BD), collected in sterile tubes, and stored at −80 °C until DNA extraction. The gating strategy is depicted in Supplementary Fig. 2 for fl/fl mice. DNA from both sorted and unsorted bacterial cells was extracted using the QIAamp DNA Mini Kit (Qiagen), following the manufacturer’s instructions and according to ref. ^80^.

### *D. fairfieldensis* and *D. piger* cultivation in LCFA-supplemented media

*D. fairfieldensis* (CSUR P7329, from Collection de Souches de l’Unité des Rickettsies) and *D. piger* (CLA-AA-H201, from Human intestinal bacterial collection) were grown in postgate medium (PG; DSMZ 63). Strains were initially grown in PG medium broth at 37 °C in an anaerobic chamber (5% CO_2_, 5% H_2_, 90% N_2_). Once the cultures reached their maximum OD_600_, they were diluted to 0.05 OD_600_ into fresh PG medium broth supplemented with 10 µM of each of the seven LCFAs, including arachidic acid (20:0), homo-γ-linolenic acid (20:3), docosanoic acid (22:0), docosatetraenoic acid (22:4), docosapentaenoic acid (22:5), docosahexaenoic acid (22:6) and nervonic acid (24:1). PG medium with *D. fairfieldensis* or *D. piger* was used as the control. Bacterial suspensions were aliquoted into a sterile 96-well flat-bottom plate (BRANDplates, BRAND) and incubated for 24 h at 37 °C in a microplate reader (Cerillo) under anaerobic conditions. Growth data were measured every 30 min. Every experiment was conducted five times for *D. fairfieldensis* and three times for *D. piger*, with three technical replicates. LCFAs added to postgate medium in the same amount as that added to bacteria-inoculated tubes, served as blanks for each condition. The average OD values of the blanks were subtracted from each technical replicate of the corresponding sample incubated with each LCFA. Of these, the average and standard deviation were calculated and plotted.

### H_2_S production by *D. fairfieldensis* cultivated in LCFA-supplemented media

*D. fairfieldensis* was initially cultured on blood agar from a glycerol stock. A single colony was transferred to postgate + lactate medium and incubated for 24 h to generate a preculture. The optical density at 600 nm (OD_600_) was adjusted to 0.05 before treatment with 10 µM LCFAs or solvent control. Cultures were incubated for 24 h at 37 °C under anaerobic conditions. H_2_S production was quantified using a colorimetric assay as described by Gong^83^. Briefly, samples were stabilized in 0.05 M zinc acetate, and 15 µl of diamine reagent was added to 900 µl of diluted sample. After 20 min of incubation in the dark, absorbance was measured at 670 nm using a microplate reader (200 µl per well in triplicates). H_2_S concentrations were determined by interpolation from a standard curve (0–500 µM Na_2_S).

### TCGA dataset

For both CRC-related TCGA projects (TCGA-COAD and TCGA-READ), gene expression profiles were downloaded from the FireBrowse.org website (RSEM normalized gene expression). Using those tables, transcription factor activities were inferred using the DoRothEA R package (version 1.10.0)^14^ with default parameters (using transcription factor–target interactions of medium or higher confidence (A-C) and using the viper algorithm for statistical inference). Bacterial profiles derived from whole-exome sequencing of the same samples were extracted from the supplementary material of Dohlman et al.^29^. For all samples present in both data types (610 samples overlap: 451 COAD, 159 READ), the inferred activity of ATF6 was associated with bacterial presence, using the project information as the random effect in a random effect model implemented in the lmerTest R package^84^. Since we observed MSI status to be very strongly associated with both bacterial profiles and ATF6 activity, we additionally included MSI status as another random effect in a second association model. Bacterial genera were tested for CRC enrichment with a random effects model for all individuals with paired tumour and normal tissue samples and significantly CRC-enriched genera highlighted. All *P* values were corrected for multiple-hypothesis testing using the Benjamini–Hochberg procedure^85^.

### Statistical analysis

No animals or data points were excluded from any analyses in this study. For data analysis, unless otherwise stated below, statistical analyses were carried out using R (R software foundation) or GraphPad Prism (version 9.00; GraphPad software). For flow cytometry analyses, data processing and analysis were performed with FlowJo v10.10.0 software (BD Life Sciences). For ATF6 quantification of IHC staining, the H-score cut-off was determined using the online ‘Cutoff Finder’ web application for biomarker cut-off optimization^86^. For comparing the mean of two groups, an unpaired student’s *t*-test or a Wilcoxon test was used where appropriate. Data distribution was assumed to be normal and not formally tested, or known to deviate from a normal distribution. Differences between multiple groups were tested using ANOVA or Kruskal–Wallis tests. For testing differences in frequency, Fisher’s exact test was used. To test for differences in beta diversity, multivariate statistical testing was conducted using PERMANOVA^87^. For hierarchical clustering of metabolite intensities, Ward’s method was used^88^. Multiple comparisons were controlled using the method of Benjamini–Hochberg^89^.

For semi-targeted metabolomics of CRC human tissue, statistical analysis and plotting were performed in R 4.4.1 using RStudio 2023.06.1. The statistical significance of molecular feature in T versus T adjacent was assessed with paired *t*-tests (t.test function in R), and *P* values were FDR corrected for multiple-hypotheses testing using the Benjamini–Hochberg procedure (p.adjust function in R with ‘fdr’ parameter). Significant metabolites were selected when their intensity had a corrected *P* value < 0.05.

For 16S rRNA gene amplicon sequencing, the decontam package utilizes the relationship between pooled DNA concentration before sequencing and the prevalence and abundance of taxa in negative controls to identify putative contaminants. Identification of contaminants was performed with the ‘isContaminant’ function using default parameters. Downstream analyses were performed using Rhea and phyloseq^90,91^. Briefly, zOTU tables were normalized using minimum sum scaling or relative abundance. Alpha diversity was measured using Shannon effective and diversity indices. To assess differences between groups, beta diversity was calculated based on generalized UniFrac distance (GUniFrac)^92^. Mean GUniFrac dissimilarity for a given sample was calculated as the dissimilarity to the respective control mean. Differentially abundant taxa were identified using the linear discriminant analysis effect size algorithm, with a linear discriminant analysis threshold threshold of 3.0, to limit false positives^93,94^.

For BONCAT-FACS experiments, statistical analysis was performed using R statistical software (https://www.r-project.org/). Raw sequencing data were preprocessed using the web platform IMNGS2 (https://www.imngs2.org/). Contaminants originating from PCR and kit reagents were detected and removed using the R package decontam^76^. zOTU counts were subsampled to a number of reads smaller than the smallest library (1,000 reads). Beta diversity was calculated using generalized UniFrac distances^92^ and visualized using multidimensional scaling^90^. Permutational multivariate analysis of variance was performed to determine the significance of sample grouping. Alpha diversity was evaluated based on taxa richness^90^ and group comparison was calculated using the Wilcoxon test. Significantly enriched and depleted zOTUs between sorted and unsorted fractions were performed using DEseq2 (ref. ^95^).

Significant zOTU sequences were identified using the 16S-based ID tool of EzBioCloud (https://www.microbiologyresearch.org/content/journal/ijsem/10.1099/ijsem.0.001755/).

The variation in microbial communities across the seven LCFAs was further analysed using the UpSetR package in R, which uses a matrix-based layout to represent set intersections and their sizes^96^.

For quantifications of the percentage of translationally active cells, ANOVA and Tukey’s test for multiple comparisons were applied.

For growth curves, two-way ANOVA was used to compare *D. fairfieldensis* or *D. piger* stimulated with each LCFA with the control without LCFAs. Variables were expressed as the mean ± s.d., a probability value (*P* value) less than 0.05 was considered statistically significant and the Bonferroni method was used for multiple comparisons. For comparison of single time points, one-way ANOVA was used, and Dunnet’s test was used for multiple comparisons.

Unless otherwise specified, all data are presented as the mean ± s.d. *P* values < 0.05 are considered statistically significant (*P < 0.05 , **P < 0.01, ***P < 0.001, ****P < 0.0001).

### Reporting summary

Further information on research design is available in the Nature Portfolio Reporting Summary linked to this article.

## Supplementary information

Supplementary Information Supplementary Figs. 1 and 2 and Supplementary Tables 1–8.

Reporting Summary

## Source data

Source Data Fig. 1 Statistical source data.

Source Data Fig. 2 Statistical source data.

Source Data Fig. 3 Statistical source data.

Source Data Fig. 4 Statistical source data.

Source Data Fig. 5 Statistical source data.

Source Data Extended Data Fig. 1 Statistical source data.

Source Data Extended Data Fig. 2 Statistical source data.

Source Data Extended Data Fig. 3 Statistical source data.

Source Data Extended Data Fig. 4 Statistical source data.

Source Data Extended Data Fig. 5 Statistical source data.

Source Data Extended Data Fig. 6 Statistical source data.

Source Data Extended Data Fig. 7 Statistical source data.

Source Data Extended Data Fig. 8 Statistical source data.

Source Data Extended Data Fig. 9 Statistical source data.

Source Data Extended Data Fig. 10 Statistical source data.

## Data Availability

Raw and processed RNA-seq data are available at the Gene Expression Omnibus under accession GSE247122. Human CRC metabolomics data are available in the MetaboLights Database^97^ under accession number MTBLS7387. 16S rRNA gene amplicon sequence data have been deposited in the NCBI Short Read Archive under PRJNA1227744. Source data are provided with this paper.

## Code availability

Custom codes have been uploaded into the GitHub repository and are available under https://github.com/adamsorbie/Coleman_Sorbie_et_al_2025/.
