# Supplementary material for: ATF6 activation alters colonic lipid metabolism causing tumour-associated microbial adaptation
Source: Nat Metab. 2025 Sep 1;7(9):1830–50. doi: 10.1038/s42255-025-01350-6 (PMC12460170; doi:10.1038/s42255-025-01350-6)
Supplement: Supplementary file 2 — Reporting Summary [file 42255_2025_1350_MOESM2_ESM.pdf]

nature portfolio

Corresponding author(s): Prof. Dirk Haller

Last updated by author(s): Jul 2, 2025

Reporting Summary

Nature Portfolio wishes to improve the reproducibility of the work that we publish. This form provides structure for consistency and transparency in reporting. For further information on Nature Portfolio policies, see our Editorial Policies and the Editorial Policy Checklist.

Statistics

For all statistical analyses, confirm that the following items are present in the figure legend, table legend, main text, or Methods section.

n/a	Confirmed	
☐	☒	The exact sample size (*n*) for each experimental group/condition, given as a discrete number and unit of measurement
☐	☒	A statement on whether measurements were taken from distinct samples or whether the same sample was measured repeatedly
☐	☒	The statistical test(s) used AND whether they are one- or two-sided<br>*Only common tests should be described solely by name; describe more complex techniques in the Methods section.*
☐	☒	A description of all covariates tested
☐	☒	A description of any assumptions or corrections, such as tests of normality and adjustment for multiple comparisons
☐	☒	A full description of the statistical parameters including central tendency (e.g. means) or other basic estimates (e.g. regression coefficient) AND variation (e.g. standard deviation) or associated estimates of uncertainty (e.g. confidence intervals)
☐	☒	For null hypothesis testing, the test statistic (e.g. *F*, *t*, *r*) with confidence intervals, effect sizes, degrees of freedom and *P* value noted<br>*Give P values as exact values whenever suitable.*
☒	☐	For Bayesian analysis, information on the choice of priors and Markov chain Monte Carlo settings
☒	☐	For hierarchical and complex designs, identification of the appropriate level for tests and full reporting of outcomes
☐	☒	Estimates of effect sizes (e.g. Cohen's *d*, Pearson's *r*), indicating how they were calculated

Our web collection on statistics for biologists contains articles on many of the points above.

Software and code

Policy information about availability of computer code

Data collection	no software was used for data collection. Data analysis software detailed below.
Data analysis	16S rRNA sequencing was processed using NGSToolkit v3.5 an in-house pipeline, wrapping UNOISE3. Predicted metagenomes were generated using PICRUSt2 (version 2.3). Processed data were analysed in R version 4.2.2 using Rhea version 2.0, and Phyloseq 1.42.0. Analysis of untargeted metabolomic data was also performed in R (4.2.2). Multi-omic integration of luminal metabolites with mucosal 16S data was performed using mixOmics (version 6.22). Statistical analysis and plotting were carried out using the ggplot2 (3.4.4), ggpubr (0.6.0), ComplexHeatmap (2.14.0), EnhancedVolcano (1.16.0) and rstatix (0.7.2) packages. RNAseq functional data analysis was performed using R version 4.2.2 on x86_64-w64-mingw32/x64 (64-bit) platform running under Windows 10 x64. For the analysis and visualisation open source R packages were used. In detail, we used biomaRt v2.54.1, fgsea v1.24.0, GSEABase v 1.60.0, SiRCleR v0.0.0.9000, EnhancedVolcano v1.16.0, ggplot2 v3.4.2 and ggalluvial v0.12.5. For data wrangling we used base R packages and dplyr v1.1.2, tidyverse v2.0.0, tidyR v 1.3.0 and tibble v3.2.1. See also methods section "RNAseq functional analysis" for additional information.

For manuscripts utilizing custom algorithms or software that are central to the research but not yet described in published literature, software must be made available to editors and reviewers. We strongly encourage code deposition in a community repository (e.g. GitHub). See the Nature Portfolio guidelines for submitting code & software for further information.

Data

Policy information about availability of data

All manuscripts must include a data availability statement. This statement should provide the following information, where applicable:

- Accession codes, unique identifiers, or web links for publicly available datasets
- A description of any restrictions on data availability
- For clinical datasets or third party data, please ensure that the statement adheres to our policy

Raw and processed RNASeq data are available at GEO under accession GSE247122. Human CRC metabolomics data is available in the MetaboLights Database (https://www.ebi.ac.uk/metabolights/) under accession number MTBLS7387. 16S rRNA gene amplicon sequence data have been deposited in the NCBI Short Read Archive under PRJNA1227744. Source data are provided with this paper.

Research involving human participants, their data, or biological material

Policy information about studies with human participants or human data. See also policy information about sex, gender (identity/presentation), and sexual orientation and race, ethnicity and racism.

Reporting on sex and gender	In all our experiments we strive to have a balance between male and female mice. While this is not always achievable in a 50/50% ratio while also following the 3Rs concept, none of our experiments are performed with only one sex, and therefore our data is representative for both sexes. In case of our human cohorts, both sexes were deliberately included, with slightly more male cases, corresponding to the incidence in the overall population. We do not see any sex-dependent differences in our data.
Reporting on race, ethnicity, or other socially relevant groupings	Classification of patients into different groups was not relevant for this study and was not performed.
Population characteristics	Cohort 1: CRC cohort comprising 1004 patients from all stages, equally distributed between sites from the colorectal system, male/female 57%/43%, median age 69 years. Cohort 2: A retrospective cohort of patients diagnosed with primary colorectal cancer, undergoing surgical tumor resection at Dept. of Surgery, Klinikum rechts der Isar, TUM (n=104 patients), in Munich, Germany, with a mean age of 66 years, ratio male/female 64%/36%. Cohort 3: Cohort 3: A retrospective cohort of cases from Northern Germany (mean age 70 years) diagnosed with resectable colorectal carcinoma (n=311 patients) with male/female 53%/47% and with available tissue samples in the biobank was selected on the basis of age at the time of diagnosis comprising samples of n = 55 individuals with early onset of disease (EOCRC group, age ≤ 50 y) and n = 256 individuals with late onset of disease (LOCRC group, age > 50 y). Cohort 4: A retrospective cohort of surgically resected tissue in CRC patients (n=259 patients, mean age 60 years), male/female 54%/46%, with matching Tumor and Tumor adjacent tissue per patient.
Recruitment	Cohort 1: The patients were identified by searching the hospital's internal information system and included in the cohort. Exclusion criteria were either missing tissue or the presence of a tumor disease other than colorectal carcinoma (WDNET, appendiceal tumor, mesenchymal tumor, lymphoma). Cohort 2: Only patients with informed, written consent prior to surgery in Dept. of Surgery, Munich, Germany, were included. No further inclusion/exclusion criteria were applied regarding, e.g., socio-economic, sex/gender, ethnical or other parameters, in accordance with the approval by the Ethics committee of the Faculty of Medicine of TUM. Cohort 3: Cohort 3: Patients with colorectal carcinoma of all age groups who underwent surgical resection in the Dept. of General Surgery, University Hospital Kiel and who gave informed written consent for biosample collection in the biobank were included. Whole surgical resectates were processed for routine histological diagnostics by the pathology department, which provided tissue samples to the biobank and for the study. Cohort 4: Only patients with informed, written consent prior to surgery were included. No further inclusion/exclusion criteria were applied regarding, e.g., socio-economic, sex/gender, ethnical or other parameters, in accordance with the approval by the Ethics committee of the Medical School – The Christian-Albrechts-University of Kiel (ref.-no. A 156/03), the Ethics Committee of the Medical School of the University of Rostock (ref.-no. A 2019-0187) and the Ethics Committee of the EMBL (ref.-no. 2024/ HE000062 - MiEOCRC).
Ethics oversight	The use of surgically resected human tissue samples was approved by the local Ethics Committee of the Technical University of Munich (TUM) (ref.-no. 252/16 s, cohort 1), by the Ethics Committee of the Medical Faculty of TUM (ref.-no. 1926/7, 375/16S and 2022-169-S-KH, cohort 2), and by the local Ethics Committee of the University Hospital Schleswig-Holstein (ref.-no. A 110/99, cohort 3). CRC patient cohort 3 tissue specimens were supplied by the biobank TRIBanK (Translational Interdisciplinary Biobank Kiel) together with the Institute of Pathology, University Hospital Schleswig-Holstein, Kiel, Germany. Patient samples from all three cohorts were obtained after prior informed written consent. Cohort 4 was approved by the Medical School – The Christian-Albrechts-University of Kiel (ref.-no. A 156/03), the Ethics Committee of the Medical School of the University of Rostock (ref.-no. A 2019-0187) and the Ethics Committee of the EMBL (ref.-no. 2024/HE000062 - MiEOCRC).

Note that full information on the approval of the study protocol must also be provided in the manuscript.

Field-specific reporting

Please select the one below that is the best fit for your research. If you are not sure, read the appropriate sections before making your selection.

☒ Life sciences ☐ Behavioural & social sciences ☐ Ecological, evolutionary & environmental sciences

For a reference copy of the document with all sections, see nature.com/documents/nr-reporting-summary-flat.pdf

Life sciences study design

All studies must disclose on these points even when the disclosure is negative.

Sample size	No sample size calculations were performed for this analysis. Based on standards of biological replicates used in life sciences research and based on our previous experiments and published studies, we included, unless otherwise stated, 6 animals per group, in order to comply with "3R" rules limiting the number of animals used in research, while reaching an adequate number of animals to avoid underpowering the study and being able to draw statistically significant conclusions. For organoid experiments, a mouse cohort of 3 mice per genotype was used, with a biological replicate number (following transgene activation using 4-OHT) of 12 per genotype. For CRC patient analyses, the sample size was based on the available participants from the respective existing cohort studies, and no additional subjects were recruited for the purposes of this analysis.
Data exclusions	No data were excluded from the analyses.
Replication	Experimental replicates were necessary and performed for the intestinal organoid experiment and the exposure of desulfovibrio to LCFAs for growth analysis and H2S measurements. The organoid experiment was replicated in a total of three independent experiments, and the LCFA exposure of Desulfovibrio was replicated in a total of 5 independent experiments for growth analysis and 4 independent experiments for H2S measurements. All attempts at replication were successful.
Randomization	For murine experiments, mice were allocated into experimental groups according to their genotype. Mice were age matched and did not show and signs of illness or injury prior to the start of each study. Both male and female mice were used for each study, wherever possible in equal ratios. Mice from different litters and housing cages were included in experimental groups, to minimize potential litter or cage-specific effects. For CRC patient analyses, randomization was not applicable as participants were selected based on the presence of a confirmed colorectal cancer diagnosis.
Blinding	Human cohort data was anonymous. Histological scoring was performed in a blinded manner. For the murine experimental analyses, experimental procedures and sampling were not blinded as defined genotypes were taken into experiments.

Reporting for specific materials, systems and methods

We require information from authors about some types of materials, experimental systems and methods used in many studies. Here, indicate whether each material, system or method listed is relevant to your study. If you are not sure if a list item applies to your research, read the appropriate section before selecting a response.

Materials & experimental systems

n/a	Involved in the study	
☐	☒	Antibodies
☒	☐	Eukaryotic cell lines
☒	☐	Palaeontology and archaeology
☐	☒	Animals and other organisms
☒	☐	Clinical data
☒	☐	Dual use research of concern
☒	☐	Plants

Methods

n/a	Involved in the study	
☒	☐	ChIP-seq
☐	☒	Flow cytometry
☒	☐	MRI-based neuroimaging

Antibodies

Antibodies used	anti-ATF6 antibody (Sigma-Aldrich, HPA-005935, Lot-Number D179431, polyclonal, host rabbit, dilution 1:100), anti-FASN antibody (LS Bio, LS-B3636, Lot-Number #27052/200ul, polyclonal, host rabbit, dilution 1:200), anti-GRP78 (Cell Signalling, 3177s, Lot-Number 10, clone C50B12, host rabbit, dilution 1:200).
Validation	All primary antibodies were optimized for dilutions and type of fixation and/or for a given antigen-retrieval method, prior to experiment analysis. In general, different dilutions were tested based on manufacter's recommendations or previously published studies. Antibodies were validated by performing appropriate negative and isotype control staining using tissue sections or transgenic cell lines (Extended Data Fig. 1.a,b,g).

Animals and other research organisms

Policy information about studies involving animals; ARRIVE guidelines recommended for reporting animal research, and Sex and Gender in Research

Laboratory animals	Species Mus Musculus on BL6 genetic background with J and N crossing (C57BL/6JN). Mice were housed under SPF and GF conditions (12 h light/dark cycles, and 24-26°C and 50% humidity). All mice were of ages between 3-20 weeks. Specifically: 5 weeks for RNAseq experiments; 5,12 and 20 weeks for untargeted metabolimics experiments; 5 and 12 weeks for 16S rRNA sequencing analyses; 5 weeks for BONCAT experiments; 3 weeks for C57 inhibitor experiments; 4 weeks for FMT experiments.

Wild animals	The study did not involve wild animals.
Reporting on sex	We previously identified that tumor development is not sex-based in our ATF6 mouse models. Grouping into different sexes was therefore not necessary, and data in all cohorts is shown for both male and female mice. We did not see any sex differences in our data.
Field-collected samples	The study did not involve samples collected from the field.
Ethics oversight	All animal experiments, as well as maintenance and breeding of mouse lines, were approved by the Committee on Animal Health Care and Use of the state of Upper Bavaria (Regierung von Oberbayern; AZ ROB-55.2-1-54-2532-217-2014, AZ ROB-55.2-2532.Vet_02-20-58, AZ TVA 55.2-2532.Vet_02-18-149, AZ TVA 55.2-2532.Vet_02-18-121, AZ TVA 55.2-2532.Vet_02-19-006) and performed in strict compliance with the EEC recommendations for the care and use of laboratory animals (Directive 2010/63/EU of the European Parliament and of the Council of 22 September 2010 on the protection of animals used for scientific purposes (2010/63/EU).

Note that full information on the approval of the study protocol must also be provided in the manuscript.

Plants

Seed stocks	n/a
Novel plant genotypes	n/a
Authentication	n/a

Flow Cytometry

Plots

Confirm that:

☒ The axis labels state the marker and fluorochrome used (e.g. CD4-FITC).

☒ The axis scales are clearly visible. Include numbers along axes only for bottom left plot of group (a 'group' is an analysis of identical markers).

☒ All plots are contour plots with outliers or pseudocolor plots.

☒ A numerical value for number of cells or percentage (with statistics) is provided.

Methodology

Sample preparation	For ex-vivo experiments, mouse cecal contents were incubated in the presence of selected long chain fatty acids and the activity marker L-azidohomoalanine (AHA). Samples were incubated in an anaerobic chamber at 37°C for 6 h. After incubation samples were fixed in ethanol : PBS (1:1). Cu(I)-catalyzed click labelling of chemically fixed microbial cells was performed in solution immeditely before FACS.
Instrument	BD FACS Melody (BD, Germany).
Software	BD FACSChorus software (BD, Germany) and FlowJo™ v10.10.0 software
Cell population abundance	Translationally active cells were quantified with flow cytometry using absolute counting beads (CountBrightTM, Invitrogen, ThermoFisher Scientific, Germany) according to the manufacturer's instructions. mean ± sd: 5.8 ± 1.6% for LCFAs in total; 0.9 ± 0.51 for the controls
Gating strategy	Background noise of the machine and of PBS was detected using the parameters forward scatter (FSC) and side scatter (SSC). Bacteria were then displayed using the same settings in a scatter plot using the forward scatter (FSC) and side scatter (SSC) and pre-gated. Singlets discrimination was performed. The negative control no AHA, was used to set the gate position for BONCAT-positive cells. Gated Cy5 positive bacteria were then sorted.

☒ Tick this box to confirm that a figure exemplifying the gating strategy is provided in the Supplementary Information.
