# Supplementary material for: ATF6 activation alters colonic lipid metabolism causing tumour-associated microbial adaptation
Source: Nat Metab. 2025 Sep 1;7(9):1830–50. doi: 10.1038/s42255-025-01350-6 (PMC12460170; doi:10.1038/s42255-025-01350-6)
Supplement: Supplementary file 1 — Supplementary Figs. 1 and 2 and Supplementary Tables 1–8. [file 42255_2025_1350_MOESM1_ESM.pdf]

nature metabolism

Supplementary information

https://doi.org/10.1038/s42255-025-01350-6

ATF6 activation alters colonic lipid metabolism causing tumour-associated microbial adaptation

In the format provided by the authors and unedited

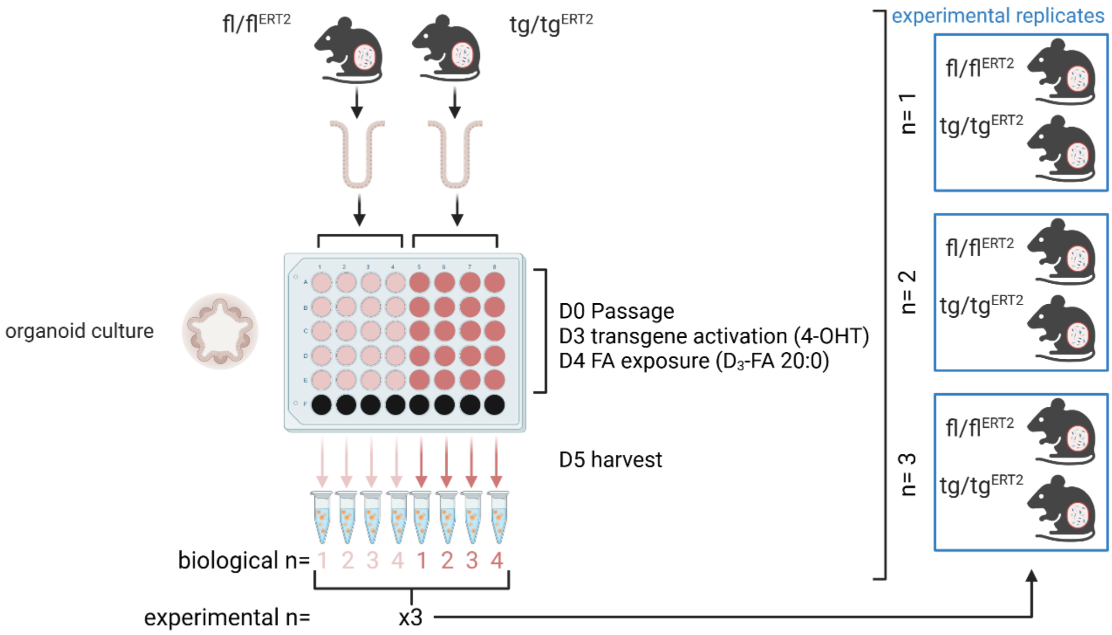

Supplementary Figure 1. Scheme depicting organoid experiment. Crypts from donor mice were isolated to generate 4 x 5 organoid cultures. The biological induction of the nATF6 transgene was performed after organoid formation through the application of tamoxifen to the respective 20 wells. After tamoxifen-induced nATF6 expression, all 20 wells were exposed to the isotope labelled fatty acid. Five wells were pooled to allow for enough material to determine fatty acid elongation in 4 different (biological) replicates per mouse. The setup was performed in three replicates with different ATF6fl/fl;Vil-CreERT2 and ATF6tg/tg;Vil-CreERT2 donor mice for organoid cultivation. Created in BioRender. Coleman, O. (2025) https://BioRender.com/cx0aoau.

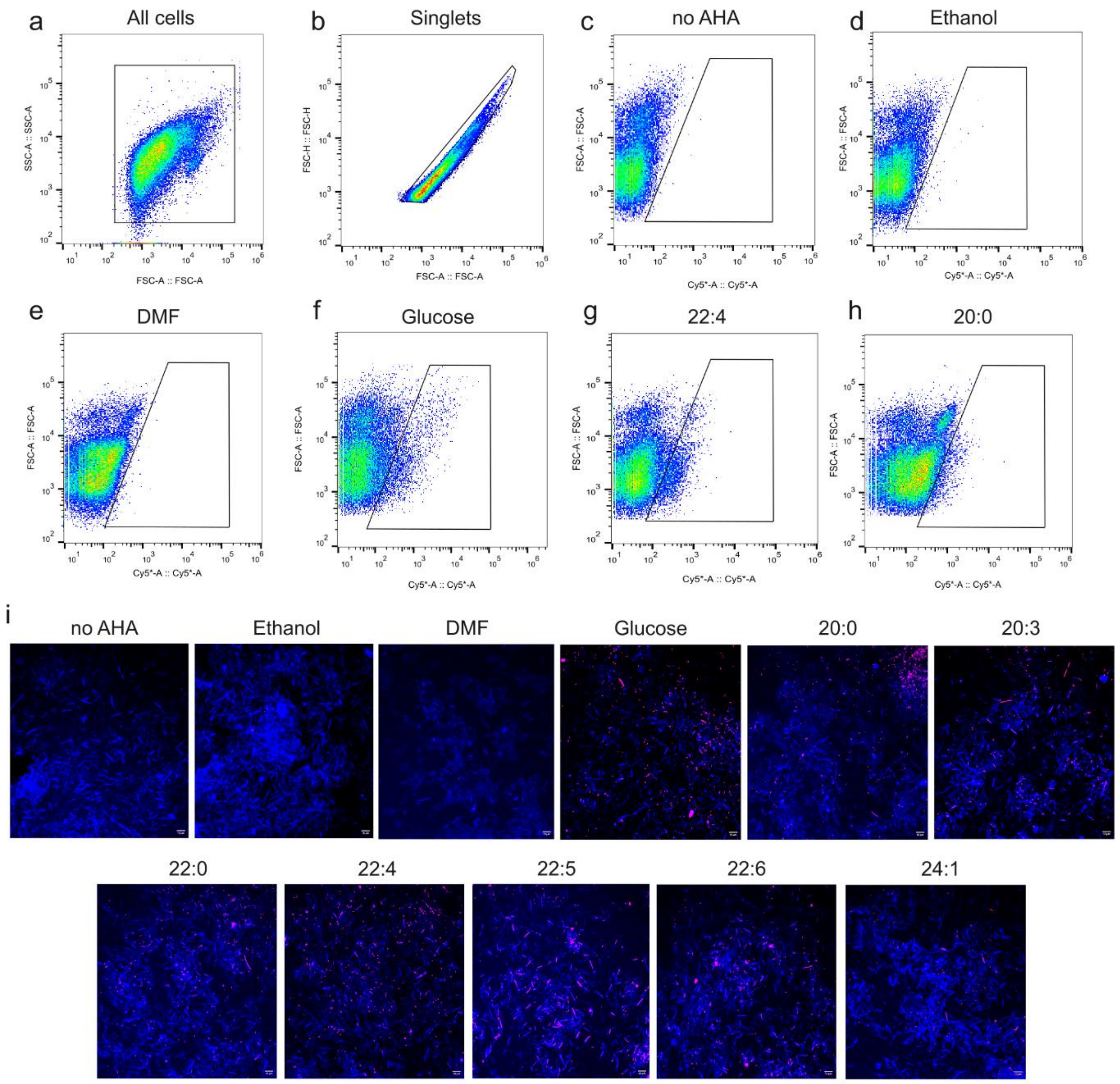

Supplementary Figure 2. Gating strategy and representative microscopic images of the cecal microbiota of fl/fl mice. Bacteria were sorted with FACS Melody cell sorter. The sorting gate was utilized to collect the active fraction after incubation with different long chain fatty acids (LCFAs). **a,** Bacteria were visualized using the forward scatter (FSC) and side scatter (SSC). **b,** Doublet discrimination was conducted to exclude doublets from analysis, preventing false positives in the sorted fraction. **c,** The negative control no AHA, which did not contain AHA, was used to set the gate position for BONCAT-positive cells. **d, e,** Ethanol and DMF were included as solvent controls for 20:3, 22:4, 22:5, 22:6, 24:1 and 20:0, 22:0, respectively. **f,** Positive control with glucose was included in every experiment to verify the accuracy of the click reaction and active cell labeling. All four controls were included in every sorting experiment. Example of Cy5-labeled cells in the presence of **g,** 22:4 and h, 20:0. Translationally active (BONCAT-positive) cells are represented as density plots. i, The images display BONCAT-positive (Cy5-labeled bacterial cells) following 6 hours of exposure to long-

chain fatty acids (LCFAs) in fl/fl mice. Glucose was used as positive control to confirm the success of the BONCAT and click reaction, no AHA condition was used as a negative control to ensure that no background noise was present in the Cy5 channel. Ethanol and DMF were used as solvent controls for LCFAs 20:3, 22:4, 22:5, 22:6, 24:1, and 20:0, 22:0, respectively. Translationally active bacterial cells are shown in pink (BONCAT positive signal), while all cells are shown in blue (DAPI). The images are displayed as merged channels, with a scale bar of 10 μm.

ensembl_gene_id	external_gene_name	entrezgene_id	gene_biotype	baseMean	log2FoldChange	lfcSE	stat	pvalue	padj	absLog2FC
ENSMUSG00000100573	1700081H04Rik	73477	lncRNA	1.277052062	0	2.58771768	0	1	1	0
ENSMUSG00000113630	4930404H11Rik	73815	lncRNA	1.60246229	0	3.03662027	0	1	1	0
ENSMUSG00000031534	Smim19	102032	protein_coding	104.3481346	-1.24E-05	0.124624165	9.95E-05	0.999920582	0.999920582	1.24E-05
ENSMUSG00000033712	Ccar2	219158	protein_coding	862.6957216	-3.23E-05	0.054208561	0.000595297	0.999525021	0.999654528	3.23E-05
ENSMUSG00000004897	Hdgf	15191	protein_coding	1601.02326	-7.70E-05	0.072677345	0.001059134	0.999154934	0.999457543	7.70E-05
ENSMUSG00000044573	Acp1	11431	protein_coding	478.8640721	8.73E-05	0.100417308	-0.00086891	0.99930671	0.999548819	8.73E-05
ENSMUSG00000010154	Spire2	234857	protein_coding	707.3039452	9.66E-05	0.086247259	-0.001120518	0.999105957	0.999457543	9.66E-05
ENSMUSG00000004771	Rab11a	53869	protein_coding	1283.591876	0.00010618	0.079163399	-0.001341274	0.998929819	0.999353427	0.00010618
ENSMUSG00000020133	2310011J03Rik	66374	protein_coding	402.9630236	0.000119126	0.077588698	-0.001535348	0.99877497	0.999319591	0.000119126
ENSMUSG00000026867	Gapvd1	66691	protein_coding	1087.836205	-0.000151106	0.053754663	0.002811026	0.997757128	0.998483602	0.000151106
ENSMUSG00000006050	Sra1	24068	protein_coding	841.7403856	0.000208183	0.070988323	-0.002932641	0.997660094	0.998483602	0.000208183
ENSMUSG00000041992	Rapgef5	217944	protein_coding	468.4509915	0.000212954	0.078204684	-0.002723034	0.997827336	0.998492433	0.000212954
ENSMUSG00000028016	Ints12	71793	protein_coding	208.5232331	-0.00022356	0.077504707	0.00288447	0.997698529	0.998483602	0.00022356
ENSMUSG00000018707	Dync1h1	13424	protein_coding	6159.674525	-0.000236844	0.084289837	0.002809874	0.997758048	0.998483602	0.000236844
ENSMUSG00000031609	Sap30	60406	protein_coding	86.00155943	0.000239647	0.126403354	-0.001895894	0.998487296	0.999092293	0.000239647
ENSMUSG00000073591	Pcdhb22	93893	protein_coding	7.710326395	-0.000245759	0.508163879	0.000483622	0.999614125	0.99967466	0.000245759
ENSMUSG00000022507	1810013L24Rik	69053	protein_coding	774.3756822	-0.000280278	0.07795149	0.003595538	0.997131182	0.998249138	0.000280278
ENSMUSG00000074340	Ovgp1	12659	protein_coding	4.585169625	0.000378842	0.647902123	-0.00058472	0.999533461	0.999654528	0.000378842
ENSMUSG00000056612	Ppp1r14b	18938	protein_coding	380.959695	0.000381748	0.123818738	-0.003083122	0.997540028	0.998483602	0.000381748
ENSMUSG00000000276	Dgke	56077	protein_coding	108.0632377	0.000402938	0.109297656	-0.003686612	0.997058516	0.998249138	0.000402938
ENSMUSG00000033124	Atg9a	245860	protein_coding	467.9146325	0.000450962	0.073374621	-0.006146027	0.995096211	0.997171234	0.000450962
ENSMUSG00000019818	Cd164	53599	protein_coding	2851.899185	0.00045761	0.110580739	-0.004138242	0.99669817	0.99808463	0.00045761
ENSMUSG00000022469	Rapgef3	223864	protein_coding	271.2134217	0.000520859	0.156439441	-0.003329464	0.997343477	0.99837123	0.000520859
ENSMUSG00000036698	Ago2	239528	protein_coding	1035.355603	0.000562776	0.102133777	-0.00551018	0.995603535	0.99741548	0.000562776
ENSMUSG00000036940	Kdm1a	99982	protein_coding	762.1568297	-0.000564145	0.056022474	0.010069974	0.991965459	0.995389267	0.000564145
ENSMUSG00000035829	Ppp1r26	241289	protein_coding	84.42778239	-0.000585851	0.143714117	0.004076505	0.996747428	0.99808463	0.000585851
ENSMUSG00000005907	Pex1	71382	protein_coding	186.4458389	-0.000588582	0.103141592	0.005706548	0.995446858	0.997384323	0.000588582
ENSMUSG00000040592	Cd79b	15985	protein_coding	2.593884132	-0.00061115	0.873853767	0.000699374	0.999441981	1	0.00061115
ENSMUSG00000030515	Tarsl2	272396	protein_coding	111.4302521	-0.000637517	0.170730076	0.003734064	0.997020655	0.998249138	0.000637517
ENSMUSG00000033237	Arid2	77044	protein_coding	594.575476	0.000672467	0.056968338	-0.011804222	0.990581813	0.994433839	0.000672467
ENSMUSG00000000134	Tfe3	209446	protein_coding	259.9004679	-0.000688296	0.078320251	0.008788222	0.992988103	0.995778769	0.000688296
ENSMUSG00000022946	Dop1b	70028	protein_coding	1196.033632	-0.000727246	0.10800482	0.006733462	0.994627515	0.996939463	0.000727246
ENSMUSG00000053838	Nudcd3	209586	protein_coding	567.3308542	0.000753768	0.060887952	-0.012379584	0.990122773	0.994337599	0.000753768
ENSMUSG00000050866	Clrn3	212070	protein_coding	1185.907672	-0.000761269	0.063606951	0.01196833	0.990450882	0.994433839	0.000761269
ENSMUSG00000028811	Yars	107271	protein_coding	752.4639051	-0.000764958	0.071234074	0.010738653	0.991431959	0.995047245	0.000764958
ENSMUSG00000063019	Manbal	69161	protein_coding	149.7060017	-0.00077066	0.100037235	0.007703728	0.993853375	0.996446603	0.00077066
ENSMUSG00000035517	Tdrd7	100121	protein_coding	770.2718511	-0.000857793	0.071845862	0.011939353	0.990474001	0.994433839	0.000857793
ENSMUSG00000004865	Srpk1	20815	protein_coding	1148.339418	0.000922545	0.071661815	-0.012873599	0.989728638	0.994183621	0.000922545
ENSMUSG00000050174	Nudt6	229228	protein_coding	23.39731417	0.000965991	0.211642943	-0.004564248	0.99635827	0.998050496	0.000965991

ensembl_gene_id	external_gene_name	entrezgene_id	gene_biotype	baseMean	log2FoldChange	lfcSE	stat	pvalue	padj	absLog2FC
ENSMUSG00000021892	Sh3bp5	24056	protein_coding	69.2198607	0.000982382	0.13488677	-0.007283011	0.99418905	0.996641735	0.000982382
ENSMUSG00000038546	Ranbp9	56705	protein_coding	448.7591511	0.001117371	0.062618174	-0.017844197	0.985763147	0.99195007	0.001117371
ENSMUSG00000069895	Atxn1l	52335	protein_coding	487.5596863	-0.001134663	0.080133234	0.014159701	0.988702571	0.993636457	0.001134663
ENSMUSG00000041012	Cmtm8	70031	protein_coding	60.84010339	-0.001139013	0.202496345	0.005624856	0.995512038	0.997384323	0.001139013
ENSMUSG00000006740	Kif5b	16573	protein_coding	3338.247337	-0.001154204	0.080724504	0.014298057	0.98859219	0.993636457	0.001154204
ENSMUSG00000032508	Myd88	17874	protein_coding	318.1733686	-0.00117501	0.08901355	0.013200346	0.989467953	0.994042693	0.00117501
ENSMUSG00000022792	Yars2	70120	protein_coding	184.098991	-0.001197818	0.119670116	0.010009334	0.99201384	0.995389267	0.001197818
ENSMUSG00000037493	Cib2	56506	protein_coding	22.87459745	-0.001201816	0.273151742	0.004399813	0.996489469	0.998060837	0.001201816
ENSMUSG00000029227	Fip1l1	66899	protein_coding	573.6716705	-0.001225636	0.056102932	0.021846203	0.982570638	0.990608762	0.001225636
ENSMUSG00000022913	Psmg1	56088	protein_coding	149.269976	0.001263429	0.105507519	-0.011974779	0.990445737	0.994433839	0.001263429
ENSMUSG00000028990	Lzic	69151	protein_coding	278.2900611	-0.001291746	0.082913684	0.015579405	0.987569936	0.99304207	0.001291746
ENSMUSG00000077714	Snord17	100313519	snoRNA	1196.118181	-0.001302503	0.212880605	0.006118469	0.995118199	0.997171234	0.001302503
ENSMUSG00000024799	Tm7sf2	73166	protein_coding	304.5683476	0.001329402	0.114056403	-0.011655651	0.990700346	0.994433839	0.001329402
ENSMUSG00000025047	Pdcd11	18572	protein_coding	449.8452708	-0.001336953	0.101081037	0.013226547	0.98944705	0.994042693	0.001336953
ENSMUSG00000047921	Trappc9	76510	protein_coding	548.5564266	-0.001342385	0.066322407	0.020240298	0.983851681	0.991234621	0.001342385
ENSMUSG00000020361	Hspa4	15525	protein_coding	2420.20498	0.001356884	0.085419272	-0.015884992	0.987326143	0.992978315	0.001356884
ENSMUSG00000030291	Med21	108098	protein_coding	185.6956275	-0.001364518	0.116997893	0.011662759	0.990694675	0.994433839	0.001364518
ENSMUSG00000041324	Inhba	16323	protein_coding	2.295440781	0.001378207	2.01518495	-0.000683911	0.999454318	1	0.001378207
ENSMUSG00000091649	Phf11b	236451	protein_coding	64.34463178	-0.001382427	0.320365157	0.004315161	0.99655701	0.998067952	0.001382427
ENSMUSG00000030051	Aplf	72103	protein_coding	119.6536241	0.001403937	0.101829949	-0.013787071	0.988999857	0.993753796	0.001403937
ENSMUSG00000028233	Tgs1	116940	protein_coding	137.943418	0.001434609	0.112175392	-0.01278898	0.989796149	0.994190961	0.001434609
ENSMUSG00000024943	Smc5	226026	protein_coding	304.0877152	-0.001498156	0.063954853	0.023425218	0.981311089	0.98994327	0.001498156
ENSMUSG00000033983	Coil	12812	protein_coding	96.6557696	0.001517499	0.116369794	-0.013040313	0.989595631	0.994110484	0.001517499
ENSMUSG00000037533	Rapgef6	192786	protein_coding	943.0214332	0.001574733	0.07533366	-0.02090344	0.983322682	0.990883011	0.001574733
ENSMUSG00000024206	Rfx2	19725	protein_coding	32.16477095	0.001582597	0.38912724	-0.004067042	0.996754979	0.99808463	0.001582597
ENSMUSG00000027942	4933434E20Rik	99650	protein_coding	102.6758371	0.001594678	0.135967163	-0.011728409	0.990642298	0.994433839	0.001594678
ENSMUSG00000030979	Uros	22276	protein_coding	200.0758897	0.00163292	0.096274837	-0.016961026	0.986467708	0.992356709	0.00163292
ENSMUSG00000005417	Mprip	26936	protein_coding	1847.156355	-0.001689492	0.098066393	0.017228039	0.986254694	0.992263312	0.001689492
ENSMUSG00000027646	Src	20779	protein_coding	2146.887716	0.001730339	0.089822291	-0.019264025	0.984630483	0.99157486	0.001730339
ENSMUSG00000029826	Zc3hav1	78781	protein_coding	1268.417383	0.001746693	0.068215835	-0.025605393	0.979572084	0.989055615	0.001746693
ENSMUSG00000035726	Supt16	114741	protein_coding	996.2198014	-0.001753737	0.101506634	0.017277067	0.98621558	0.992263312	0.001753737
ENSMUSG00000071172	Srsf3	20383	protein_coding	1783.902145	-0.001799904	0.072637075	0.024779415	0.980230911	0.989337077	0.001799904
ENSMUSG00000032020	Ubash3b	72828	protein_coding	27.54898273	-0.001811379	0.224496848	0.008068618	0.993562244	0.99621657	0.001811379
ENSMUSG00000026280	Atg4b	66615	protein_coding	564.5497199	-0.001825019	0.054597863	0.033426556	0.973334433	0.98611318	0.001825019
ENSMUSG00000039987	Phtf2	68770	protein_coding	48.95507312	0.00182588	0.184357333	-0.009904029	0.992097857	0.995401911	0.00182588
ENSMUSG00000050549	Fam241a	70617	protein_coding	149.5614365	-0.001839138	0.12718414	0.014460435	0.988462644	0.99357673	0.001839138
ENSMUSG00000021607	Mrpl36	94066	protein_coding	404.4919914	-0.001846181	0.083942246	0.021993466	0.982453168	0.990550804	0.001846181
ENSMUSG00000031364	Grpr	14829	protein_coding	30.25963417	0.001888063	0.332558804	-0.005677381	0.995470129	0.997384323	0.001888063
ENSMUSG00000033364	Usp37	319651	protein_coding	462.7823755	0.001891098	0.055002314	-0.03438215	0.972572417	0.985703995	0.001891098

ensembl_gene_id	external_gene_name	entrezgene_id	gene_biotype	baseMean	log2FoldChange	lfcSE	stat	pvalue	padj	absLog2FC
ENSMUSG00000032185	Carm1	59035	protein_coding	271.5097885	-0.001898206	0.085777163	0.0221295	0.982344654	0.99050187	0.001898206
ENSMUSG00000041390	Mdfic	16543	protein_coding	50.40902921	0.001907998	0.170086422	-0.011217817	0.991049664	0.994724011	0.001907998
ENSMUSG00000019978	Epb41l2	13822	protein_coding	420.9506095	0.001912214	0.099923638	-0.019136754	0.984732012	0.99157486	0.001912214
ENSMUSG00000035451	Foxa1	15375	protein_coding	566.9495321	-0.001931471	0.10131365	0.019064274	0.984789832	0.99157486	0.001931471
ENSMUSG00000071531	Gprin2	432839	protein_coding	3.095350147	-0.001993175	1.035042229	0.001925694	0.998463519	1	0.001993175
ENSMUSG00000044350	Lacc1	210808	protein_coding	107.8792558	-0.0020721	0.115767833	0.017898753	0.985719624	0.99195007	0.0020721
ENSMUSG00000022013	Dnajc15	66148	protein_coding	331.7192118	0.002082908	0.074640486	-0.027905876	0.977737222	0.988088396	0.002082908
ENSMUSG00000070426	Rnf121	75212	protein_coding	389.410576	0.00210336	0.063675807	-0.033032318	0.973648815	0.98618968	0.00210336
ENSMUSG00000035930	Chst4	26887	protein_coding	755.3702886	-0.002106814	0.238543778	0.00883198	0.992953191	0.995778769	0.002106814
ENSMUSG00000030225	Dera	232449	protein_coding	538.3981588	0.002121259	0.082574204	-0.025689129	0.979505295	0.989055615	0.002121259
ENSMUSG00000089872	Rps6kc1	320119	protein_coding	327.9857132	-0.002123631	0.067276705	0.031565618	0.974818462	0.986890148	0.002123631
ENSMUSG00000059475	Zfp426	235028	protein_coding	221.8442611	0.002162356	0.094205547	-0.0229536	0.981687285	0.990146635	0.002162356
ENSMUSG00000096992	Gm26788	108167617	lncRNA	17.89695839	-0.002166188	0.331928649	0.006526066	0.99479299	0.996966345	0.002166188
ENSMUSG00000066640	Fbxl18	231863	protein_coding	360.0502406	-0.00221628	0.085199146	0.026012935	0.979247022	0.989008948	0.00221628
ENSMUSG00000028191	Bcl10	12042	protein_coding	724.5456993	0.002230816	0.063947397	-0.034885175	0.972171302	0.985568894	0.002230816
ENSMUSG00000072872	Rybp	56353	protein_coding	225.3435194	0.002248374	0.105885983	-0.021233915	0.98305906	0.990798738	0.002248374
ENSMUSG00000024219	Anks1	224650	protein_coding	621.6085874	0.002260288	0.059289564	-0.038122863	0.969589723	0.984433119	0.002260288
ENSMUSG00000026784	Pdss1	56075	protein_coding	385.040132	-0.002273654	0.071306789	0.03188552	0.974563346	0.986813361	0.002273654
ENSMUSG00000031393	Mecp2	17257	protein_coding	330.4321051	0.002321396	0.069423892	-0.033438001	0.973325306	0.98611318	0.002321396
ENSMUSG00000034973	Dop1a	320615	protein_coding	726.4698021	-0.002335981	0.052051906	0.044877924	0.964204613	0.982347769	0.002335981
ENSMUSG00000031913	Vps4a	116733	protein_coding	602.663135	0.002373671	0.08476247	-0.028003796	0.977659124	0.988088396	0.002373671
ENSMUSG00000043085	Tmem82	213989	protein_coding	176.7582885	-0.002429696	0.128998821	0.018835027	0.984972712	0.991590016	0.002429696
ENSMUSG00000029594	Rbm19	74111	protein_coding	204.724593	0.002482288	0.114497644	-0.02167982	0.982703361	0.990621616	0.002482288
ENSMUSG00000022849	Hspbap1	66667	protein_coding	79.11399134	-0.002490832	0.137500282	0.018115108	0.985547025	0.991913919	0.002490832
ENSMUSG00000039714	Cplx3	235415	protein_coding	2.511315346	0.002520554	0.965566033	-0.002610442	0.997917171	1	0.002520554
ENSMUSG00000039021	Ttc16	338348	protein_coding	7.646279742	-0.002559799	0.719430907	0.003558089	0.997161062	0.998249138	0.002559799
ENSMUSG00000025872	Thoc3	73666	protein_coding	282.6253644	0.002567986	0.115173649	-0.022296642	0.982211327	0.990427908	0.002567986
ENSMUSG00000058351	Smim4	66487	protein_coding	70.48864374	0.002623413	0.124606275	-0.021053618	0.983202884	0.990883011	0.002623413
ENSMUSG00000021392	Nol8	70930	protein_coding	209.2336982	0.002628763	0.140727023	-0.018679875	0.985096483	0.991590016	0.002628763
ENSMUSG00000110185	Igip	109169	protein_coding	130.994716	0.002638975	0.189326069	-0.013938785	0.988878819	0.993692638	0.002638975
ENSMUSG00000059796	Eif4a1	13681	protein_coding	4015.611126	-0.002639155	0.10918166	0.024172148	0.980715295	0.989584039	0.002639155
ENSMUSG00000077217	Gm23951	115485840	snoRNA	0.637227717	-0.002661005	1.441072238	0.001846545	0.998526671	1	0.002661005
ENSMUSG00000020124	Usp15	14479	protein_coding	1004.507682	0.002662281	0.061563878	-0.04324421	0.965506864	0.982706396	0.002662281
ENSMUSG00000039063	Echdc3	67856	protein_coding	105.7596826	-0.00267418	0.125315717	0.021339539	0.982974803	0.990774286	0.00267418
ENSMUSG00000020715	Ern1	78943	protein_coding	525.2089985	-0.002734335	0.072581673	0.037672529	0.969948779	0.984509162	0.002734335
ENSMUSG00000021592	Arsk	77041	protein_coding	106.2054746	-0.002758806	0.123492434	0.022339881	0.982176836	0.990427908	0.002758806
ENSMUSG00000028224	Nbn	27354	protein_coding	333.469505	-0.002773398	0.108422429	0.025579562	0.979592688	0.989055615	0.002773398
ENSMUSG00000021967	Mrpl57	67840	protein_coding	394.8801979	-0.002825627	0.097413166	0.029006628	0.976859304	0.987811867	0.002825627
ENSMUSG00000028597	Gpx7	67305	protein_coding	25.12664132	0.002855732	0.204058144	-0.013994697	0.988834212	0.993692638	0.002855732

ensembl_gene_id	external_gene_name	entrezgene_id	gene_biotype	baseMean	log2FoldChange	lfcSE	stat	pvalue	padj	absLog2FC
ENSMUSG00000028843	Sh3bgrl3	73723	protein_coding	692.8798783	0.002896364	0.083866948	-0.034535222	0.972450356	0.985701275	0.002896364
ENSMUSG00000002031	Ift46	76568	protein_coding	289.4240698	0.002926048	0.097644162	-0.029966443	0.976093815	0.987575865	0.002926048
ENSMUSG00000028973	Abcb8	74610	protein_coding	813.9932726	-0.002956157	0.095507505	0.030952089	0.975307749	0.987234441	0.002956157
ENSMUSG00000036398	Ppp1r11	76497	protein_coding	483.4509429	0.002957935	0.062716144	-0.047163856	0.962382634	0.981474584	0.002957935
ENSMUSG00000063659	Zbtb18	30928	protein_coding	429.3822104	0.002964641	0.057794855	-0.051295937	0.959089706	0.980158095	0.002964641
ENSMUSG00000002812	Flii	14248	protein_coding	2660.862153	0.003044211	0.065898347	-0.046195565	0.963154378	0.981701728	0.003044211
ENSMUSG00000005034	Prkacb	18749	protein_coding	1104.098423	0.003055641	0.206336045	-0.014809053	0.988184517	0.993418099	0.003055641
ENSMUSG00000034820	Cpsf7	269061	protein_coding	448.0469124	-0.003057727	0.060375707	0.050644983	0.959608418	0.980447529	0.003057727
ENSMUSG00000030002	Dusp11	72102	protein_coding	1283.125366	0.003089395	0.066839213	-0.046221293	0.963133871	0.981701728	0.003089395
ENSMUSG00000040264	Gbp2b	14468	protein_coding	0.99786071	-0.003126751	2.222433082	0.001406905	0.998877453	1	0.003126751
ENSMUSG00000041439	Mfsd6	98682	protein_coding	2885.96381	-0.003228963	0.094793369	0.034063175	0.972826774	0.985840779	0.003228963
ENSMUSG00000061032	Rrp1	18114	protein_coding	1134.614073	0.003239203	0.066021169	-0.0490631	0.96086901	0.980946516	0.003239203
ENSMUSG00000050812	Ecpas	230249	protein_coding	1760.177073	0.003253207	0.092953	-0.034998413	0.972081006	0.985568894	0.003253207
ENSMUSG00000041674	BC006965	217294	lncRNA	6.70043436	-0.003290501	0.707546529	0.004650579	0.996289388	0.998042036	0.003290501
ENSMUSG00000039886	Tmem120a	215210	protein_coding	396.1376531	-0.00329616	0.105373943	0.031280597	0.975045764	0.987059753	0.00329616
ENSMUSG00000037415	Ranbp10	74334	protein_coding	349.3132716	0.003303517	0.08652944	-0.038177957	0.969545796	0.984433119	0.003303517
ENSMUSG00000043671	Dpy19l3	233115	protein_coding	68.67367529	0.003323706	0.13232717	-0.025117335	0.979961373	0.989246416	0.003323706
ENSMUSG00000041272	Tox	252838	protein_coding	187.0924109	-0.003330158	0.113571095	0.029322235	0.976607594	0.987811867	0.003330158
ENSMUSG00000025208	Mrpl43	94067	protein_coding	320.8006508	-0.003338138	0.085788234	0.038911374	0.968961048	0.98433949	0.003338138
ENSMUSG00000044518	Foxe3	30923	protein_coding	3.661887614	-0.003344915	0.858920935	0.003894322	0.996892788	1	0.003344915
ENSMUSG00000034601	2700049A03Rik	76967	protein_coding	142.1892166	0.003347456	0.098834581	-0.033869281	0.972981389	0.985876467	0.003347456
ENSMUSG00000022089	Bin3	57784	protein_coding	292.8845026	-0.00340044	0.083020366	0.0409591	0.967328502	0.983635967	0.00340044
ENSMUSG00000029462	Vps29	56433	protein_coding	387.2708906	0.003438003	0.064694788	-0.053141879	0.957618864	0.979202348	0.003438003
ENSMUSG00000052419	Smim30	66520	protein_coding	247.3876158	-0.003464967	0.10393977	0.033336299	0.973406407	0.9861256	0.003464967
ENSMUSG00000020056	Washc3	67282	protein_coding	272.9817775	0.003543076	0.081970585	-0.043223744	0.965523178	0.982706396	0.003543076
ENSMUSG00000021339	Mrs2	380836	protein_coding	906.7144092	0.003586162	0.07736787	-0.046352085	0.963029626	0.981695756	0.003586162
ENSMUSG00000024068	Spast	50850	protein_coding	444.9912384	-0.003591132	0.083853356	0.042826335	0.965839971	0.982740683	0.003591132
ENSMUSG00000069456	Rdh16	19683	protein_coding	351.4552562	-0.003654015	0.168491846	0.021686599	0.982697954	0.990621616	0.003654015
ENSMUSG00000031781	Ciapin1	109006	protein_coding	268.6508332	0.003662839	0.081907709	-0.0447191	0.964331209	0.982416138	0.003662839
ENSMUSG00000003199	Mpnd	68047	protein_coding	878.8970071	0.003682289	0.058710929	-0.062718977	0.949990286	0.975869522	0.003682289
ENSMUSG00000042742	Bmt2	101148	protein_coding	280.9763168	-0.003734192	0.12821368	0.029124752	0.976765095	0.987811867	0.003734192
ENSMUSG00000030753	Thap12	72981	protein_coding	564.7360381	-0.00373743	0.10187173	0.036687611	0.970734087	0.984868386	0.00373743
ENSMUSG00000031634	Ufsp2	192169	protein_coding	471.5930355	0.003755315	0.11309464	-0.033205069	0.973511056	0.986171119	0.003755315
ENSMUSG00000049307	Fut4	14345	protein_coding	1351.452686	-0.003802459	0.245911824	0.015462695	0.987663046	0.993075228	0.003802459
ENSMUSG00000056770	Setd3	52690	protein_coding	1448.032221	-0.003851792	0.074765375	0.051518397	0.958912443	0.980067227	0.003851792
ENSMUSG00000099843	Gm7160	635504	lncRNA	19.94306795	0.003890691	0.239751844	-0.016227993	0.987052503	0.992763569	0.003890691
ENSMUSG00000034343	Ube2f	67921	protein_coding	414.6869368	0.003909755	0.064808321	-0.060327978	0.951894419	0.976016991	0.003909755
ENSMUSG00000022246	Rai14	75646	protein_coding	425.9068381	0.00391071	0.126594087	-0.03089173	0.975355885	0.987234441	0.00391071
ENSMUSG00000046108	Il17c	234836	protein_coding	6.85799542	0.003960856	0.469234478	-0.008441101	0.993265056	0.995979059	0.003960856

ensembl_gene_id	external_gene_name	entrezgene_id	gene_biotype	baseMean	log2FoldChange	lfcSE	stat	pvalue	padj	absLog2FC
ENSMUSG00000027868	Tbx15	21384	protein_coding	1.7612301	-0.003963924	1.570418688	0.002524119	0.997986047	1	0.003963924
ENSMUSG00000031979	Cog2	76332	protein_coding	737.7728719	-0.00397506	0.094706569	0.041972376	0.96652072	0.983251596	0.00397506
ENSMUSG00000114797	Gm49336	71844	protein_coding	613.4960048	0.003976889	0.08803822	-0.045172307	0.963969968	0.982229903	0.003976889
ENSMUSG00000027428	Rbbp9	26450	protein_coding	121.7880144	0.004015954	0.124369084	-0.032290613	0.974240295	0.986657316	0.004015954
ENSMUSG00000020721	Helz	78455	protein_coding	412.3686085	-0.004095255	0.123719095	0.033101236	0.973593858	0.98618968	0.004095255
ENSMUSG00000031577	Tti2	234138	protein_coding	147.2515944	0.004141045	0.089996316	-0.046013497	0.963299492	0.98178904	0.004141045
ENSMUSG00000042179	Pnliprp1	18946	protein_coding	4.730888262	-0.004141616	0.617137378	0.006711011	0.994645428	0.996939463	0.004141616
ENSMUSG00000026147	Col9a1	12839	protein_coding	1.679628758	-0.004142447	1.917922116	0.002159862	0.998276681	1	0.004142447
ENSMUSG00000020359	Phykpl	72947	protein_coding	261.4111307	-0.00414275	0.110803493	0.037388265	0.970175429	0.984664559	0.00414275
ENSMUSG00000038084	Opa1	74143	protein_coding	1410.670368	-0.004149199	0.061429848	0.067543694	0.946148878	0.973795719	0.004149199
ENSMUSG00000028890	Mtf1	17764	protein_coding	328.1556375	-0.004185849	0.064204123	0.065195946	0.948017989	0.974999631	0.004185849
ENSMUSG00000009741	Ubp1	22221	protein_coding	604.2559899	0.004215528	0.051913535	-0.081202873	0.935280615	0.969552109	0.004215528
ENSMUSG00000024841	Eif1ad	69860	protein_coding	341.2458367	-0.004222943	0.102681321	0.041126696	0.967194892	0.983635967	0.004222943
ENSMUSG00000055717	Slain1	105439	protein_coding	63.57609454	-0.004228524	0.151112747	0.027982573	0.97767605	0.988088396	0.004228524
ENSMUSG00000056458	Mok	26448	protein_coding	8.319862078	0.004230274	0.441155846	-0.009589069	0.992349147	0.995423402	0.004230274
ENSMUSG00000014104	Sval2	84543	protein_coding	1.01033778	0.004242161	3.004388395	-0.001411988	0.998873397	1	0.004242161
ENSMUSG00000085234	Gm15614	102640951	lncRNA	2.917262422	-0.004257881	0.925438214	0.004600935	0.996328998	1	0.004257881
ENSMUSG00000095301	Or5p55	258926	protein_coding	0.922296796	0.004260532	2.989954025	-0.001424949	0.998863056	1	0.004260532
ENSMUSG00000114538	Gm31517	102633770	lncRNA	1.057898392	0.004278778	2.974199488	-0.001438632	0.998852138	1	0.004278778
ENSMUSG00000043727	F830045P16Rik	228592	protein_coding	1.148892156	0.004283669	2.969308392	-0.001442649	0.998848933	1	0.004283669
ENSMUSG00000073931	Or52d1	259058	protein_coding	1.148892156	0.004283669	2.969308392	-0.001442649	0.998848933	1	0.004283669
ENSMUSG00000113056	6030440G07Rik	77878	lncRNA	2.065391108	-0.004292326	0.796202852	0.005390996	0.995698628	1	0.004292326
ENSMUSG00000029595	Lhx5	16873	protein_coding	1.244013381	0.004293923	2.960373856	-0.001450466	0.998842696	1	0.004293923
ENSMUSG00000011349	Dmrtc2	71241	protein_coding	1.196452768	0.00429568	2.959386379	-0.001451544	0.998841836	1	0.00429568
ENSMUSG00000020168	Or14c43	257929	protein_coding	1.196452768	0.00429568	2.959386379	-0.001451544	0.998841836	1	0.00429568
ENSMUSG00000050504	Or5bh3	258385	protein_coding	1.382567757	0.004301634	2.953214332	-0.001456594	0.998837807	1	0.004301634
ENSMUSG00000087038	2900079G21Rik	620760	lncRNA	1.382567757	0.004301634	2.953214332	-0.001456594	0.998837807	1	0.004301634
ENSMUSG00000114807	Gm32090	102634531	lncRNA	1.382567757	0.004301634	2.953214332	-0.001456594	0.998837807	1	0.004301634
ENSMUSG00000086171	Pcsk2os1	378776	lncRNA	0.867408709	0.004303933	2.956591485	-0.001455708	0.998838514	1	0.004303933
ENSMUSG00000092162	Vmn2r86	625109	protein_coding	1.287446533	0.004304003	2.952051874	-0.00145797	0.998836709	1	0.004304003
ENSMUSG00000067522	Or5a1	258677	protein_coding	1.335007145	0.004307336	2.949098648	-0.00146056	0.998834642	1	0.004307336
ENSMUSG00000056019	Zfp709	236193	protein_coding	72.28737292	-0.004337408	0.16442543	0.026379177	0.978954903	0.988867032	0.004337408
ENSMUSG00000071000	Or13c7	29845	protein_coding	1.65554905	0.00433769	2.923652944	-0.001483654	0.998816216	1	0.00433769
ENSMUSG00000027088	Phospho2	73373	protein_coding	268.2992684	0.004337741	0.111131118	-0.039032639	0.968864366	0.984301824	0.004337741
ENSMUSG00000024790	Sac3d1	66406	protein_coding	131.547206	0.004342356	0.146567738	-0.02962696	0.976364564	0.98778928	0.004342356
ENSMUSG00000001280	Sp1	20683	protein_coding	1497.095104	0.004346101	0.079225539	-0.054857324	0.956252132	0.978107631	0.004346101
ENSMUSG00000037788	Vopp1	232023	protein_coding	221.9648527	0.004352234	0.109189743	-0.039859366	0.968205247	0.984116541	0.004352234
ENSMUSG00000038888	Ctu1	233189	protein_coding	162.1095651	-0.004391502	0.105982214	0.041436212	0.966948144	0.983565239	0.004391502
ENSMUSG00000020070	Rufy2	70432	protein_coding	100.7477828	-0.004422659	0.124998567	0.035381681	0.971775392	0.985501033	0.004422659

ensembl_gene_id	external_gene_name	entrezgene_id	gene_biotype	baseMean	log2FoldChange	lfcSE	stat	pvalue	padj	absLog2FC
ENSMUSG00000001445	Mrpl10	107732	protein_coding	621.5498773	-0.004424286	0.093448471	0.047344658	0.962238536	0.981434574	0.004424286
ENSMUSG00000035852	Misp	78906	protein_coding	7448.958347	0.004452934	0.127947274	-0.034802879	0.972236925	0.985568894	0.004452934
ENSMUSG00000057134	Ado	211488	protein_coding	395.1343538	-0.004472139	0.113851649	0.039280407	0.968666827	0.984161682	0.004472139
ENSMUSG00000051359	Ncald	52589	protein_coding	342.5184785	-0.00447297	0.154245909	0.028998956	0.976865424	0.987811867	0.00447297
ENSMUSG00000028101	Pias3	229615	protein_coding	164.8290924	0.004481135	0.139042156	-0.032228604	0.974289745	0.986657316	0.004481135
ENSMUSG00000020491	2810021J22Rik	69944	protein_coding	87.5773785	-0.004513938	0.128978573	0.034997581	0.972081669	0.985568894	0.004513938
ENSMUSG00000041769	Ppp2r2d	52432	protein_coding	358.9074714	0.004536676	0.073189828	-0.061985062	0.950574728	0.975869522	0.004536676
ENSMUSG00000035273	Hpse	15442	protein_coding	180.8848147	-0.004541391	0.110999686	0.040913549	0.967364816	0.983635967	0.004541391
ENSMUSG00000023873	1700010I14Rik	66931	protein_coding	44.39201065	0.004551056	0.192773203	-0.023608344	0.981165016	0.989916855	0.004551056
ENSMUSG00000020935	Dcakd	68087	protein_coding	147.4528786	0.00457489	0.123476937	-0.037050562	0.970444691	0.984756276	0.00457489
ENSMUSG00000009112	Bcl2l13	94044	protein_coding	703.1074778	0.00458516	0.05536503	-0.082816903	0.933997129	0.968724317	0.00458516
ENSMUSG00000041057	Wdr43	72515	protein_coding	558.0983811	0.004596564	0.102384343	-0.044895187	0.964190853	0.982347769	0.004596564
ENSMUSG00000032777	Gtf3c1	233863	protein_coding	1595.800602	0.004598477	0.039792057	-0.115562679	0.907999142	0.955211953	0.004598477
ENSMUSG00000020949	Fkbp3	30795	protein_coding	182.2044994	-0.004672805	0.110641409	0.042233778	0.966312336	0.983100167	0.004672805
ENSMUSG00000022020	Naa16	66897	protein_coding	124.6593066	0.004683871	0.114405066	-0.040941114	0.96734284	0.983635967	0.004683871
ENSMUSG00000031626	Sorbs2	234214	protein_coding	535.9549179	0.004701927	0.152465266	-0.030839335	0.975397671	0.987234441	0.004701927
ENSMUSG00000024370	Cdc23	52563	protein_coding	290.9827796	0.004706802	0.126570691	-0.037187136	0.970335796	0.984706282	0.004706802
ENSMUSG00000024424	Ttc39c	72747	protein_coding	17.68270885	0.004744183	0.313607601	-0.015127767	0.987930248	0.993283417	0.004744183
ENSMUSG00000042032	Mat2b	108645	protein_coding	993.1326032	0.004771693	0.074770689	-0.063817701	0.949115384	0.975520722	0.004771693
ENSMUSG00000043962	Thrap3	230753	protein_coding	1391.323634	0.004776233	0.084724529	-0.056373675	0.955044128	0.977538039	0.004776233
ENSMUSG00000006304	Arpc2	76709	protein_coding	2521.668858	-0.004776629	0.051092545	0.09348975	0.925514492	0.964363534	0.004776629
ENSMUSG00000115726	Gm30083	102631856	protein_coding	1.655795596	0.004777584	2.700886196	-0.001768895	0.998588627	1	0.004777584
ENSMUSG00000033845	Mrpl15	27395	protein_coding	561.2052347	-0.004784392	0.06443225	0.074254623	0.940807783	0.971638507	0.004784392
ENSMUSG00000029647	Pan3	72587	protein_coding	974.8481309	-0.004838029	0.049367732	0.097999821	0.921932435	0.962436702	0.004838029
ENSMUSG00000097665	Gm16853	330902	lncRNA	2.736898181	-0.004852414	0.76206592	0.006367447	0.994919547	1	0.004852414
ENSMUSG00000095567	Noc2l	57741	protein_coding	522.1849011	-0.004853522	0.094271546	0.05148449	0.958939461	0.980067227	0.004853522
ENSMUSG00000056912	1700017N19Rik	66605	protein_coding	1.104594524	0.004870992	2.583258494	-0.0018856	0.99849551	1	0.004870992
ENSMUSG00000035953	Pip4p1	219024	protein_coding	345.7412088	0.004871196	0.070596957	-0.069000089	0.944989549	0.97354273	0.004871196
ENSMUSG00000051190	Or7c70	258305	protein_coding	1.242838701	0.004889462	2.572240383	-0.001900857	0.998483336	1	0.004889462
ENSMUSG00000029992	Gfpt1	14583	protein_coding	11180.13193	-0.00489726	0.144412537	0.033911596	0.972947647	0.985876467	0.00489726
ENSMUSG00000094488	Or1e33	259010	protein_coding	1.151844937	0.004897699	2.568206842	-0.00190705	0.998478395	1	0.004897699
ENSMUSG00000078190	Dnm3os	474332	lncRNA	1.151528249	0.004898909	2.567555423	-0.001908005	0.998477633	1	0.004898909
ENSMUSG00000048015	Neurod4	11923	protein_coding	1.473437904	0.004903035	2.56428655	-0.001912046	0.998474409	1	0.004903035
ENSMUSG00000027981	Rnpc3	67225	protein_coding	127.9954263	0.004907738	0.092932139	-0.052809911	0.957883365	0.979351528	0.004907738
ENSMUSG00000017740	Slc12a5	57138	protein_coding	9.403661977	0.004912187	0.749822304	-0.006551134	0.994772988	0.996966345	0.004912187
ENSMUSG00000112331	Gm33035	102635787	lncRNA	0.96265355	0.004913941	2.560772104	-0.00191893	0.998468917	1	0.004913941
ENSMUSG00000022791	Tnk2	51789	protein_coding	657.4431438	-0.004916283	0.068632981	0.07163149	0.942895179	0.972316965	0.004916283
ENSMUSG00000074254	Cyp2a4	13086	protein_coding	1.196329151	0.004923469	2.554489413	-0.001927379	0.998462175	1	0.004923469
ENSMUSG00000074628	Tldc2	383766	protein_coding	92.10558334	-0.004934899	0.219148867	0.022518476	0.982034374	0.990370421	0.004934899

ensembl_gene_id	external_gene_name	entrezgene_id	gene_biotype	baseMean	log2FoldChange	lfcSE	stat	pvalue	padj	absLog2FC
ENSMUSG00000111869	Or1j14	258950	protein_coding	1.425877292	0.004935419	2.54723809	-0.001937557	0.998454054	1	0.004935419
ENSMUSG00000029526	1700123K08Rik	76658	protein_coding	1.057774775	0.004939593	2.546657721	-0.001939638	0.998452394	1	0.004939593
ENSMUSG00000039032	Tsga13	116732	protein_coding	1.043535072	0.004940645	2.546149598	-0.001940438	0.998451756	1	0.004940645
ENSMUSG00000086022	Rad51ap2	209550	protein_coding	0.847460503	0.004954383	2.539606907	-0.001950846	0.998443451	1	0.004954383
ENSMUSG00000005370	Msh6	17688	protein_coding	435.4233794	-0.004956253	0.133082432	0.037241979	0.970292068	0.984706282	0.004956253
ENSMUSG00000095932	Vmn1r49	24112	protein_coding	1.16500064	0.004960812	2.534622756	-0.001957219	0.998438366	1	0.004960812
ENSMUSG00000028958	Tmub1	64295	protein_coding	259.4297212	0.004962895	0.07205475	-0.068876717	0.945087752	0.97354273	0.004962895
ENSMUSG00000075208	Or5ar1	259017	protein_coding	0.874295879	0.004963162	2.535051721	-0.001957815	0.998437891	1	0.004963162
ENSMUSG00000080717	B230307C23Rik	245305	protein_coding	74.35730743	-0.004967796	0.128932413	0.038530233	0.969264927	0.984403993	0.004967796
ENSMUSG00000091468	Vmn2r82	624845	protein_coding	1.066270333	0.004983316	2.523397126	-0.001974844	0.998424304	1	0.004983316
ENSMUSG00000090304	Vmn2r99	665376	protein_coding	1.844616819	0.004984744	2.520335945	-0.001977809	0.998421938	1	0.004984744
ENSMUSG00000034623	Prss55	71037	protein_coding	1.022837181	0.004995012	2.517651072	-0.001983997	0.998417	1	0.004995012
ENSMUSG00000060335	Or1e25	193053	protein_coding	0.973436112	0.004997391	2.516646827	-0.001985734	0.998415615	1	0.004997391
ENSMUSG00000026271	Gpr35	64095	protein_coding	361.3985963	-0.005006522	0.076651362	0.065315505	0.947922797	0.974962449	0.005006522
ENSMUSG00000086844	B230206H07Rik	320871	lncRNA	17.19204084	-0.005020063	0.510042407	0.009842442	0.992146994	0.995401911	0.005020063
ENSMUSG00000032594	Ip6k1	27399	protein_coding	849.2192546	0.00502864	0.074594268	-0.067413219	0.946252746	0.973795719	0.00502864
ENSMUSG00000033972	Zfp944	319615	protein_coding	136.5038676	0.005056968	0.127442369	-0.039680427	0.968347906	0.984122985	0.005056968
ENSMUSG00000050765	Gm5084	100503759	lncRNA	1.69448389	0.00507117	2.477536774	-0.002046859	0.998366844	1	0.00507117
ENSMUSG00000091504	Vmn2r118	383258	protein_coding	1.69448389	0.00507117	2.477536774	-0.002046859	0.998366844	1	0.00507117
ENSMUSG00000070855	Or10ag54	257875	protein_coding	2.244326701	0.005094936	2.464912278	-0.002066985	0.998350786	1	0.005094936
ENSMUSG00000020950	Foxg1	15228	protein_coding	1.040606745	0.005112629	2.458965964	-0.002079179	0.998341057	1	0.005112629
ENSMUSG00000029726	Mepce	231803	protein_coding	452.6238051	0.005114043	0.082631536	-0.061889719	0.950650655	0.975869522	0.005114043
ENSMUSG00000005893	Nr2c2	22026	protein_coding	566.6827522	-0.00511806	0.076534602	0.066872496	0.946683209	0.973990811	0.00511806
ENSMUSG00000042198	Chchd7	66433	protein_coding	322.2298501	0.005138394	0.080983426	-0.063449949	0.949408214	0.97563953	0.005138394
ENSMUSG00000031310	Zmym3	56364	protein_coding	260.5321973	-0.005163266	0.102853979	0.050199963	0.959963041	0.980506535	0.005163266
ENSMUSG00000026675	Hsd17b7	15490	protein_coding	306.047576	-0.005163934	0.134361735	0.038433069	0.969342395	0.984403993	0.005163934
ENSMUSG00000070420	Zscan25	666311	protein_coding	139.3103244	-0.005173196	0.10685669	0.048412465	0.961387525	0.981111951	0.005173196
ENSMUSG00000037017	Zscan21	22697	protein_coding	212.0206503	-0.005181551	0.103095303	0.050259817	0.959915345	0.980506535	0.005181551
ENSMUSG00000036339	Tmem260	218989	protein_coding	390.399798	0.005212578	0.121172277	-0.043017908	0.965687259	0.982706396	0.005212578
ENSMUSG00000045180	Shroom2	110380	protein_coding	607.6456158	0.005268167	0.075151065	-0.070101029	0.944113247	0.973044134	0.005268167
ENSMUSG00000035086	Becn1	56208	protein_coding	1063.185592	0.005277878	0.045144643	-0.116910384	0.906931069	0.954378365	0.005277878
ENSMUSG00000033883	Zfp267	241944	protein_coding	170.6332006	0.005278593	0.100841988	-0.052345188	0.958253648	0.979508488	0.005278593
ENSMUSG00000077497	Gm23593	115485971	snoRNA	1.226548998	0.005310503	1.09477287	-0.00485078	0.996129653	1	0.005310503
ENSMUSG00000061559	Wdr61	66317	protein_coding	482.4422483	-0.005315311	0.09219848	0.057650744	0.954026829	0.977047797	0.005315311
ENSMUSG00000045053	Kcng3	225030	protein_coding	2.747548653	-0.005321584	0.849408797	0.006265044	0.995001251	1	0.005321584
ENSMUSG00000045671	Spred2	114716	protein_coding	328.4361693	-0.005354374	0.113010919	0.047379261	0.962210958	0.981434574	0.005354374
ENSMUSG00000032309	Fbxo22	71999	protein_coding	341.3702807	-0.005363454	0.073891955	0.072585093	0.94213629	0.971883559	0.005363454
ENSMUSG00000035439	Haus8	76478	protein_coding	191.5829763	-0.005395122	0.143277449	0.037655066	0.969962702	0.984509162	0.005395122
ENSMUSG00000024985	Tcf7l2	21416	protein_coding	558.5046978	0.005450128	0.208658745	-0.026119818	0.97916177	0.988983332	0.005450128

ensembl_gene_id	external_gene_name	entrezgene_id	gene_biotype	baseMean	log2FoldChange	lfcSE	stat	pvalue	padj	absLog2FC
ENSMUSG00000044224	Dnajc21	78244	protein_coding	301.3581676	0.005455805	0.090786953	-0.060094595	0.952080295	0.976016991	0.005455805
ENSMUSG00000048355	Arxes1	76219	protein_coding	0.818351343	-0.005457427	1.67365432	0.003260785	0.997398275	1	0.005457427
ENSMUSG00000037110	Ralgapa2	241694	protein_coding	1902.774412	0.00546321	0.078443901	-0.069644799	0.944476379	0.973296906	0.00546321
ENSMUSG00000028309	Rnf20	109331	protein_coding	802.1291369	0.005476486	0.073568068	-0.074441077	0.940659424	0.971606814	0.005476486
ENSMUSG00000027860	Vangl1	229658	protein_coding	169.4605644	-0.00552574	0.113142273	0.048838864	0.96104771	0.981007658	0.00552574
ENSMUSG00000033306	Lpp	210126	protein_coding	1851.766542	0.00560402	0.10940236	-0.05122394	0.959147076	0.980158095	0.00560402
ENSMUSG00000016510	Mtif3	76366	protein_coding	293.3454113	0.005610402	0.076495467	-0.073342937	0.941533225	0.971840719	0.005610402
ENSMUSG00000043911	Or8b55	258777	protein_coding	1.272959596	0.005618964	2.243096768	-0.002505003	0.998001299	1	0.005618964
ENSMUSG00000094651	Gal3st2	381334	protein_coding	159.6967969	-0.00563163	0.232725273	0.02419862	0.980694179	0.989584039	0.00563163
ENSMUSG00000061626	Or52a5	18369	protein_coding	1.294086469	0.005639144	2.235361958	-0.002522698	0.99798718	1	0.005639144
ENSMUSG00000043314	Or2z2	18329	protein_coding	1.234770362	0.005661454	2.227189119	-0.002541973	0.997971801	1	0.005661454
ENSMUSG00000027788	Otol1	229389	protein_coding	1.566326898	0.005663178	2.225868208	-0.002544256	0.99796998	1	0.005663178
ENSMUSG00000020436	Gabrg2	14406	protein_coding	1.580189068	0.005665099	2.225075121	-0.002546026	0.997968567	1	0.005665099
ENSMUSG00000041730	Prrxl1	107751	protein_coding	1.913919788	0.005685681	2.217126182	-0.002564437	0.997953877	1	0.005685681
ENSMUSG00000033931	Rbm34	52202	protein_coding	171.5399331	-0.005686418	0.129499593	0.043910703	0.964975583	0.982587667	0.005686418
ENSMUSG00000092239	1700031A10Rik	73286	lncRNA	1.336778757	0.005690334	2.216239225	-0.002567563	0.997951383	1	0.005690334
ENSMUSG00000049902	4921517D22Rik	70900	protein_coding	1.246101681	0.005691297	2.21607866	-0.002568184	0.997950888	1	0.005691297
ENSMUSG00000040650	Cyp2b23	243881	protein_coding	1.245784993	0.005693067	2.215428361	-0.002569737	0.997949649	1	0.005693067
ENSMUSG00000021792	Prxl2a	70564	protein_coding	92.13329467	-0.00569601	0.159042823	0.03581432	0.971430415	0.985415068	0.00569601
ENSMUSG00000024044	Epb41l3	13823	protein_coding	1178.556983	0.005703089	0.076003114	-0.075037569	0.94018482	0.971417829	0.005703089
ENSMUSG00000045381	Or10aa1	258712	protein_coding	1.15172132	0.00571835	2.206371872	-0.002591743	0.99793209	1	0.00571835
ENSMUSG00000044542	Prop1	19127	protein_coding	1.632683575	0.005722508	2.204043965	-0.002596368	0.997928401	1	0.005722508
ENSMUSG00000051367	Six1	20471	protein_coding	1.202860636	0.005727023	2.203046845	-0.002599592	0.997925828	1	0.005727023
ENSMUSG00000031241	Tbx22	245572	protein_coding	1.222565954	0.005729717	2.201977055	-0.002602078	0.997923844	1	0.005729717
ENSMUSG00000063011	Msln	56047	protein_coding	1.976206453	-0.005733806	1.285055999	0.004461912	0.996439921	1	0.005733806
ENSMUSG00000024593	Megf10	70417	protein_coding	2.956303408	0.005749286	2.193130518	-0.002621498	0.99790835	1	0.005749286
ENSMUSG00000029685	Asb15	78910	protein_coding	1.792681512	0.005755629	2.191874229	-0.002625894	0.997904842	1	0.005755629
ENSMUSG00000031358	Msl3	17692	protein_coding	526.0892	-0.005781457	0.101094143	0.057188843	0.954394765	0.977236803	0.005781457
ENSMUSG00000110970	Or13c7b	29846	protein_coding	1.199158315	0.005781725	2.183562073	-0.002647841	0.997887331	1	0.005781725
ENSMUSG00000029387	Gtf2h3	209357	protein_coding	377.6166657	0.005783371	0.0911347	-0.063459593	0.949400534	0.97563953	0.005783371
ENSMUSG00000020430	Pes1	64934	protein_coding	575.7868989	-0.005803929	0.07974034	0.072785349	0.941976929	0.971883559	0.005803929
ENSMUSG00000005982	Naa60	74763	protein_coding	332.7992821	-0.005831284	0.084873637	0.06870548	0.945224056	0.97354273	0.005831284
ENSMUSG00000090315	Vmn2r104	22313	protein_coding	1.060603938	0.005834303	2.165447358	-0.002694271	0.997850285	1	0.005834303
ENSMUSG00000052525	Spdya	70891	protein_coding	3.810493772	0.00586697	0.577087972	-0.01016651	0.991888439	1	0.00586697
ENSMUSG00000028842	Ago3	214150	protein_coding	219.0560777	-0.005874035	0.128522764	0.045704238	0.963545986	0.981940857	0.005874035
ENSMUSG00000024976	Shoc2	56392	protein_coding	1044.208139	-0.005876413	0.06095315	0.09640869	0.92319599	0.962738158	0.005876413
ENSMUSG00000052616	Terb1	320022	protein_coding	2.90037548	0.005879139	0.776932749	-0.007567114	0.993962374	1	0.005879139
ENSMUSG00000036980	Taf6	21343	protein_coding	222.2063311	0.005886869	0.096030076	-0.061302347	0.951118422	0.97587882	0.005886869
ENSMUSG00000062604	Srpk2	20817	protein_coding	219.5714223	0.005893837	0.097882297	-0.060213514	0.951985582	0.976016991	0.005893837

Supplementary Table 1

ensembl_gene_id	external_gene_name	entrezgene_id	gene_biotype	baseMean	log2FoldChange	lfcSE	stat	pvalue	padj	absLog2FC
ENSMUSG00000026883	Dab2ip	69601	protein_coding	1981.554412	0.005907888	0.053768054	-0.109877283	0.912506699	0.957266079	0.005907888
ENSMUSG00000062116	Zfp954	232853	protein_coding	196.7074958	-0.00592169	0.088978124	0.066552204	0.946938197	0.974163449	0.00592169
ENSMUSG00000036781	Rps27l	67941	protein_coding	1089.435257	-0.00595958	0.104555782	0.056999048	0.954545954	0.977331011	0.00595958
ENSMUSG00000021913	Ogdhl	239017	protein_coding	22.24098862	0.005969809	0.22665063	-0.026339255	0.978986745	0.988867032	0.005969809
ENSMUSG00000003500	Impdh1	23917	protein_coding	250.5606192	-0.005981102	0.114318849	0.052319471	0.958274139	0.979508488	0.005981102
ENSMUSG00000062007	Hsh2d	209488	protein_coding	39.27345323	0.005989992	0.211490261	-0.028322781	0.977404711	0.988088396	0.005989992
ENSMUSG00000021133	Susd6	217684	protein_coding	1182.388743	0.006042569	0.097723774	-0.061833149	0.950695705	0.975869522	0.006042569
ENSMUSG00000042118	Bhmt2	64918	protein_coding	8.154100691	-0.006069778	0.386301032	0.015712559	0.987463708	0.992998437	0.006069778
ENSMUSG00000046139	Patl1	225929	protein_coding	170.0591364	0.006077563	0.098148759	-0.061921952	0.950624986	0.975869522	0.006077563
ENSMUSG00000038957	Edc3	353190	protein_coding	203.5281583	-0.006120192	0.09888302	0.061893254	0.95064784	0.975869522	0.006120192
ENSMUSG00000024912	Fosl1	14283	protein_coding	31.28143243	0.006135872	0.433211706	-0.014163679	0.988699397	0.993636457	0.006135872
ENSMUSG00000029505	Ep400	75560	protein_coding	1086.666967	-0.006139831	0.104415501	0.058801916	0.953109883	0.976710073	0.006139831
ENSMUSG00000028937	Acot7	70025	protein_coding	944.0467129	0.006145378	0.105158721	-0.058439067	0.953398897	0.976822768	0.006145378
ENSMUSG00000020686	Gas2l2	237891	protein_coding	1.752291821	0.00621527	2.041670889	-0.003044207	0.997571078	1	0.00621527
ENSMUSG00000049482	Ctu2	66965	protein_coding	154.7043841	0.006235135	0.110384558	-0.056485574	0.954954988	0.977515248	0.006235135
ENSMUSG00000004364	Cul3	26554	protein_coding	1096.898828	0.006237504	0.08399716	-0.074258513	0.940804688	0.971638507	0.006237504
ENSMUSG00000030888	Rrp8	101867	protein_coding	139.7143289	0.006243016	0.108406559	-0.057588914	0.95407608	0.977047797	0.006243016
ENSMUSG00000042305	Tmem183a	57439	protein_coding	1756.387409	0.006277025	0.063269957	-0.099210194	0.920971381	0.961855464	0.006277025
ENSMUSG00000021978	Extl3	54616	protein_coding	525.8374457	0.006300884	0.088698153	-0.071037374	0.94336801	0.972595566	0.006300884
ENSMUSG00000048939	Atp13a5	268878	protein_coding	1.987524132	0.006315814	2.011858903	-0.003139292	0.997495211	1	0.006315814
ENSMUSG00000031162	Gata1	14460	protein_coding	1.809424736	0.006340521	2.004906666	-0.003162502	0.997476693	1	0.006340521
ENSMUSG00000020317	Theg	21830	protein_coding	1.085980126	0.006367339	1.998169094	-0.003186587	0.997457476	1	0.006367339
ENSMUSG00000001998	Ap4e1	108011	protein_coding	153.6991173	0.006378778	0.091237166	-0.069914252	0.94426191	0.973136619	0.006378778
ENSMUSG00000085399	Foxd2os	100040736	lncRNA	386.7154176	0.006439132	0.102841241	-0.062612354	0.950075192	0.975869522	0.006439132
ENSMUSG00000030898	Cckbr	12426	protein_coding	1.643574589	0.00644893	1.974563613	-0.003266003	0.997394111	1	0.00644893
ENSMUSG00000039316	Rftn1	76438	protein_coding	6.096606166	0.006475057	0.675262854	-0.009588944	0.992349247	0.995423402	0.006475057
ENSMUSG00000057541	Pus7	78697	protein_coding	239.2222074	0.006486605	0.111375972	-0.058240616	0.953556968	0.97686351	0.006486605
ENSMUSG00000079625	Tm4sf19	277203	protein_coding	1.09360816	0.006492409	1.963246436	-0.003306976	0.99736142	1	0.006492409
ENSMUSG00000009894	Snap47	67826	protein_coding	217.4726997	0.006495774	0.090576475	-0.071715907	0.942827997	0.972316965	0.006495774
ENSMUSG00000007950	Abhd8	64296	protein_coding	117.6948641	-0.006511022	0.182283333	0.035719238	0.971506231	0.985415068	0.006511022
ENSMUSG00000063549	Or2w3	404346	protein_coding	1.150540151	0.006521709	1.955202427	-0.003335567	0.997338607	1	0.006521709
ENSMUSG00000036023	Parp2	11546	protein_coding	265.9954174	0.006529404	0.08173507	-0.079884977	0.936328738	0.969802608	0.006529404
ENSMUSG00000002799	Jag2	16450	protein_coding	64.74631642	-0.006545777	0.222145561	0.029466163	0.976492805	0.987811867	0.006545777
ENSMUSG00000049719	Prss46	74306	protein_coding	1.025458165	0.006548678	1.948087511	-0.003361593	0.997317842	1	0.006548678
ENSMUSG00000033147	Slc22a15	242126	protein_coding	174.5050995	0.006565754	0.143741831	-0.045677406	0.963567372	0.981940857	0.006565754
ENSMUSG00000028221	Pip4p2	72519	protein_coding	244.1166455	0.006583543	0.090702094	-0.072584245	0.942136964	0.971883559	0.006583543
ENSMUSG00000032480	Dhx30	72831	protein_coding	572.2411215	-0.006598053	0.069368692	0.095115712	0.924222919	0.963320302	0.006598053
ENSMUSG00000029822	Osbpl3	71720	protein_coding	249.5851792	-0.006621462	0.217397668	0.030457834	0.975701921	0.987300339	0.006621462
ENSMUSG00000045232	Rln3	212108	protein_coding	1.15530581	0.006624921	1.927763578	-0.003436584	0.997258008	1	0.006624921

ensembl_gene_id	external_gene_name	entrezgene_id	gene_biotype	baseMean	log2FoldChange	lfcSE	stat	pvalue	padj	absLog2FC
ENSMUSG00000032497	Lrrfip2	71268	protein_coding	1199.983501	-0.006634509	0.098945848	0.067051922	0.946540368	0.973904526	0.006634509
ENSMUSG00000029090	Adgra3	70693	protein_coding	1450.907578	0.006635848	0.100517312	-0.066016966	0.947364318	0.974509428	0.006635848
ENSMUSG00000039117	Taf4	228980	protein_coding	436.194727	0.006641053	0.097863235	-0.06786055	0.945896643	0.973795719	0.006641053
ENSMUSG00000027206	Cops2	12848	protein_coding	742.0104914	-0.006758467	0.11095885	0.060909673	0.951431146	0.975912221	0.006758467
ENSMUSG00000026273	Mterf4	69821	protein_coding	153.1161409	-0.006782039	0.100690994	0.067354969	0.946299117	0.973795719	0.006782039
ENSMUSG00000014550	Rbsn	78287	protein_coding	404.7583959	-0.006786905	0.084354431	0.080457006	0.93587379	0.96968875	0.006786905
ENSMUSG00000026824	Kcnj3	16519	protein_coding	0.944921598	0.006802836	1.882700574	-0.003613339	0.997116979	1	0.006802836
ENSMUSG00000000384	Tbrg4	21379	protein_coding	906.4806778	-0.006804982	0.052270479	0.130187861	0.896417798	0.948996349	0.006804982
ENSMUSG00000059518	Znhit1	70103	protein_coding	294.015806	0.006826791	0.099291014	-0.068755372	0.945184342	0.97354273	0.006826791
ENSMUSG00000049305	Ccdc71	72454	protein_coding	415.9844361	-0.006868352	0.070137548	0.097926888	0.921990349	0.962436702	0.006868352
ENSMUSG00000034211	Mrps17	66258	protein_coding	474.8547191	0.006879508	0.090439513	-0.0760675	0.939365396	0.971181378	0.006879508
ENSMUSG00000100315	1700031P21Rik	73278	lncRNA	3.856869726	0.006901674	0.640443362	-0.0107764	0.991401843	1	0.006901674
ENSMUSG00000029657	Hsph1	15505	protein_coding	754.2506714	-0.006907372	0.172868423	0.039957395	0.968127093	0.984116541	0.006907372
ENSMUSG00000046785	Epm2aip1	77781	protein_coding	297.1929578	-0.006922837	0.103097007	0.067148768	0.94646327	0.973885877	0.006922837
ENSMUSG00000025135	Anapc11	66156	protein_coding	245.9474447	-0.006958912	0.096545918	0.072078781	0.942539213	0.972163488	0.006958912
ENSMUSG00000041570	Camsap2	67886	protein_coding	132.2481809	-0.006963586	0.163984122	0.042465002	0.966128011	0.982973198	0.006963586
ENSMUSG00000042520	Ubap2l	74383	protein_coding	842.7205024	0.006992239	0.061019258	-0.114590697	0.908769554	0.955501531	0.006992239
ENSMUSG00000038009	Dnajc22	72778	protein_coding	779.8779283	0.006994416	0.053737597	-0.130158708	0.896440863	0.948996349	0.006994416
ENSMUSG00000033671	Cep350	74081	protein_coding	1576.080099	-0.007012866	0.083244174	0.084244524	0.932862019	0.968109605	0.007012866
ENSMUSG00000020385	Clk4	12750	protein_coding	330.2708626	-0.007045781	0.114453522	0.061560196	0.950913076	0.97587882	0.007045781
ENSMUSG00000063785	Utp14a	72554	protein_coding	289.3843583	0.007056744	0.120533074	-0.058546122	0.953313625	0.976796005	0.007056744
ENSMUSG00000030091	Nup210	54563	protein_coding	382.4162076	-0.007079155	0.115043475	0.061534607	0.950933454	0.97587882	0.007079155
ENSMUSG00000020476	Dbnl	13169	protein_coding	1323.534683	-0.007079984	0.05724227	0.12368455	0.901565045	0.951341404	0.007079984
ENSMUSG00000024866	Acy3	71670	protein_coding	184.4799706	0.007117378	0.141216887	-0.050400331	0.959803373	0.980506535	0.007117378
ENSMUSG00000003228	Grk5	14773	protein_coding	119.6704512	0.00711781	0.102436804	-0.069484894	0.944603657	0.973367327	0.00711781
ENSMUSG00000021975	Ints9	210925	protein_coding	182.3392182	-0.00715615	0.090802087	0.078810415	0.93718342	0.970043107	0.00715615
ENSMUSG00000017176	Nt5c3b	68106	protein_coding	99.27375164	-0.007201982	0.127523025	0.056475933	0.954962668	0.977515248	0.007201982
ENSMUSG00000078974	Sec61g	20335	protein_coding	311.5139324	0.007234121	0.167955783	-0.04307158	0.965644474	0.982706396	0.007234121
ENSMUSG00000061755	Bod1l	665775	protein_coding	783.475028	0.007269901	0.064729345	-0.112312293	0.910575795	0.956641558	0.007269901
ENSMUSG00000028793	Rnf19b	75234	protein_coding	651.4443124	-0.007348579	0.136936313	0.053664208	0.9572027	0.978898029	0.007348579
ENSMUSG00000027342	Pcna	18538	protein_coding	808.611401	0.007350089	0.118270375	-0.062146495	0.950446171	0.975869522	0.007350089
ENSMUSG00000003154	Foxj2	60611	protein_coding	235.1554533	0.007352731	0.133835831	-0.054938436	0.956187511	0.978107631	0.007352731
ENSMUSG00000042558	Adprs	100206	protein_coding	253.1493821	0.007367214	0.098376482	-0.074887957	0.940303859	0.971417829	0.007367214
ENSMUSG00000031591	Asah1	11886	protein_coding	1637.262148	-0.007422949	0.101808308	0.072911037	0.941876911	0.971883559	0.007422949
ENSMUSG00000092131	BC050972	407808	lncRNA	0.839860736	0.007452894	1.736295048	-0.004292412	0.996575161	1	0.007452894
ENSMUSG00000020882	Cacnb1	12295	protein_coding	39.43291226	0.007467805	0.172707977	-0.043239493	0.965510624	0.982706396	0.007467805
ENSMUSG00000030539	Sema4b	20352	protein_coding	1460.368242	-0.007509132	0.120896313	0.062112168	0.950473507	0.975869522	0.007509132
ENSMUSG00000044595	Dnd1	213236	protein_coding	5.098127557	-0.00751441	0.602118463	0.012479952	0.990042697	0.994317649	0.00751441
ENSMUSG00000022911	Arl13b	68146	protein_coding	184.7486948	0.007548511	0.090743303	-0.083185325	0.933704181	0.968634984	0.007548511

ensembl_gene_id	external_gene_name	entrezgene_id	gene_biotype	baseMean	log2FoldChange	lfcSE	stat	pvalue	padj	absLog2FC
ENSMUSG00000040616	Tmem51	214359	protein_coding	328.6020681	0.007604762	0.079967461	-0.095098209	0.924236821	0.963320302	0.007604762
ENSMUSG00000031792	Usb1	101985	protein_coding	189.7154228	0.007614772	0.072902915	-0.104450856	0.916811566	0.959274295	0.007614772
ENSMUSG00000101892	9130401M01Rik	75758	protein_coding	67.200789	0.007631306	0.158876303	-0.048033002	0.961689941	0.981238658	0.007631306
ENSMUSG00000045215	Asxl3	211961	protein_coding	32.15789152	0.007634212	0.287025399	-0.026597688	0.978780617	0.988831409	0.007634212
ENSMUSG00000035891	Cerk	223753	protein_coding	382.239555	0.007645375	0.088378404	-0.086507275	0.931063174	0.966840628	0.007645375
ENSMUSG00000034875	Nudt19	110959	protein_coding	919.0254062	0.007703094	0.104534644	-0.073689392	0.94125754	0.971762583	0.007703094
ENSMUSG00000037600	Kdf1	69073	protein_coding	365.0036389	-0.007706598	0.07411925	0.103975666	0.91718866	0.959486477	0.007706598
ENSMUSG00000021830	Txndc16	70561	protein_coding	334.7793098	-0.00773743	0.205171412	0.037712026	0.969917288	0.984509162	0.00773743
ENSMUSG00000033623	Pcgf3	69587	protein_coding	1048.315396	0.007737592	0.08068315	-0.09590097	0.923599224	0.962971397	0.007737592
ENSMUSG00000047884	Klk9	101533	protein_coding	1.659587662	0.007799139	1.667452674	-0.004677278	0.996268086	1	0.007799139
ENSMUSG00000022570	Gfus	22122	protein_coding	1221.227912	0.007799894	0.086939274	-0.08971658	0.92851244	0.965886893	0.007799894
ENSMUSG00000057181	5730455P16Rik	70591	protein_coding	132.0121879	-0.00780972	0.120637356	0.064737165	0.948383271	0.975173098	0.00780972
ENSMUSG00000047671	Dynlt4	242646	protein_coding	31.34866226	-0.00781399	0.302345755	0.025844549	0.979381329	0.989031323	0.00781399
ENSMUSG00000070056	Mfhas1	52065	protein_coding	606.1597773	0.007835577	0.086931326	-0.090135256	0.928179733	0.965818434	0.007835577
ENSMUSG00000078572	Ndufaf8	208501	protein_coding	256.5653784	0.007847581	0.092231638	-0.085085562	0.932193368	0.967643553	0.007847581
ENSMUSG00000029048	Rer1	67830	protein_coding	1753.557343	-0.007849546	0.073958246	0.106134829	0.915475378	0.958483509	0.007849546
ENSMUSG00000053730	Tmem39b	230770	protein_coding	64.22735266	-0.007885625	0.14288971	0.055186791	0.955989652	0.978020884	0.007885625
ENSMUSG00000079487	Med12	59024	protein_coding	535.8692453	0.007946554	0.089427209	-0.088860589	0.929192705	0.966128972	0.007946554
ENSMUSG00000020878	Lrrc46	69297	protein_coding	4.212473996	-0.007998212	0.715306576	0.011181516	0.991078627	1	0.007998212
ENSMUSG00000021228	Acot3	171281	protein_coding	0.990589206	0.008011155	1.628630152	-0.004918953	0.996075259	1	0.008011155
ENSMUSG00000023882	Zfp54	22712	protein_coding	141.9419506	-0.008016706	0.124214685	0.064539114	0.948540964	0.975173098	0.008016706
ENSMUSG00000006717	Acot13	66834	protein_coding	304.5659411	-0.008028876	0.097950134	0.08196901	0.934671357	0.969180133	0.008028876
ENSMUSG00000075229	Ccdc58	381045	protein_coding	220.7892173	-0.008032453	0.081848765	0.098137747	0.921822915	0.962383589	0.008032453
ENSMUSG00000032743	Katnip	233865	protein_coding	602.9110334	0.008035023	0.050482642	-0.159164075	0.873539607	0.937764615	0.008035023
ENSMUSG00000000827	Tpd52l2	66314	protein_coding	560.1399664	-0.008082954	0.065146197	0.124074068	0.901256631	0.95132126	0.008082954
ENSMUSG00000029110	Rnf4	19822	protein_coding	1521.099862	-0.008089478	0.05047531	0.160266044	0.872671506	0.937225143	0.008089478
ENSMUSG00000020608	Smc6	67241	protein_coding	693.5444421	-0.008094068	0.087112076	0.092915567	0.925970639	0.964583514	0.008094068
ENSMUSG00000050908	Tvp23a	383103	protein_coding	5.026772231	0.008104718	0.465001216	-0.017429455	0.986094011	0.99222255	0.008104718
ENSMUSG00000044026	Slc35g1	240660	protein_coding	2181.405145	-0.008153281	0.092273127	0.088360298	0.929590315	0.966128972	0.008153281
ENSMUSG00000005936	Kctd20	66989	protein_coding	490.2031704	0.008159648	0.055697441	-0.146499505	0.883527081	0.942360409	0.008159648
ENSMUSG00000034771	Tle2	21886	protein_coding	43.83823658	-0.008161934	0.332030892	0.024581851	0.980388496	0.989375191	0.008161934
ENSMUSG00000027420	Bfsp1	12075	protein_coding	80.36863025	-0.008183653	0.183684243	0.044552833	0.964463739	0.982490542	0.008183653
ENSMUSG00000031337	Mtm1	17772	protein_coding	827.9720499	0.008187114	0.074419393	-0.110013179	0.912398923	0.957266079	0.008187114
ENSMUSG00000006699	Cdc42	12540	protein_coding	4200.393197	-0.008225363	0.07860573	0.104640758	0.916660872	0.959274295	0.008225363
ENSMUSG00000022372	Sla	20491	protein_coding	14.26540232	0.008228919	0.429141072	-0.019175324	0.984701243	0.99157486	0.008228919
ENSMUSG00000006134	Crkl	12929	protein_coding	513.8858672	0.008260695	0.056261831	-0.14682591	0.883269433	0.942211125	0.008260695
ENSMUSG00000022476	Polr3h	78929	protein_coding	96.59048031	0.008282849	0.146638045	-0.05648499	0.954955452	0.977515248	0.008282849
ENSMUSG00000028180	Zranb2	53861	protein_coding	606.6898468	-0.008287956	0.092870269	0.089242298	0.928889351	0.966049475	0.008287956
ENSMUSG00000027514	Zbp1	58203	protein_coding	190.090626	-0.008289982	0.293606482	0.02823501	0.977474714	0.988088396	0.008289982

Supplementary Table 1

ensembl_gene_id	external_gene_name	entrezgene_id	gene_biotype	baseMean	log2FoldChange	lfcSE	stat	pvalue	padj	absLog2FC
ENSMUSG00000034006	Slc66a2	66943	protein_coding	632.3857677	-0.008358132	0.064046479	0.130501038	0.896170033	0.948996349	0.008358132
ENSMUSG00000038279	Nop2	110109	protein_coding	430.7712611	0.00838017	0.095656717	-0.087606702	0.930189275	0.966352899	0.00838017
ENSMUSG00000049532	Sall2	50524	protein_coding	19.85570014	-0.008400054	0.36113853	0.023259922	0.981442941	0.990015804	0.008400054
ENSMUSG00000024242	Map4k3	225028	protein_coding	1180.88525	0.00840102	0.04764649	-0.176319807	0.860042702	0.931327553	0.00840102
ENSMUSG00000021461	Fancc	14088	protein_coding	120.8214734	-0.008435259	0.115380341	0.073108284	0.941719949	0.971883559	0.008435259
ENSMUSG00000056941	Commd7	99311	protein_coding	363.6700361	-0.008438923	0.075777159	0.111365001	0.911326912	0.956997404	0.008438923
ENSMUSG00000027508	Pag1	94212	protein_coding	401.4728419	0.008448826	0.124697279	-0.067754695	0.945980909	0.973795719	0.008448826
ENSMUSG00000020527	Myo19	66196	protein_coding	748.8571903	0.008448878	0.180058017	-0.046923087	0.962574528	0.98153379	0.008448878
ENSMUSG00000110902	Gm33104	102635880	lncRNA	1.458210017	0.008449226	1.181774053	-0.007149612	0.994295484	1	0.008449226
ENSMUSG00000048731	Ggnbp1	70772	protein_coding	17.06244312	-0.008486421	0.372981713	0.022752915	0.981847367	0.9902423	0.008486421
ENSMUSG00000073910	Mob3b	214944	protein_coding	356.1405204	0.008552435	0.102772228	-0.08321737	0.933678701	0.968634984	0.008552435
ENSMUSG00000097156	Gm3764	100042277	lncRNA	3.722814714	-0.00856217	2.485382133	0.003445012	0.997251284	1	0.00856217
ENSMUSG00000034893	Cog3	338337	protein_coding	1006.19759	-0.008591453	0.08805464	0.097569562	0.922274095	0.962478474	0.008591453
ENSMUSG00000040860	Crocc	230872	protein_coding	134.5599128	-0.008597398	0.177071965	0.048553128	0.961275424	0.981095512	0.008597398
ENSMUSG00000020841	Cpd	12874	protein_coding	4922.415684	-0.008623041	0.15447904	0.055820138	0.955485092	0.977832194	0.008623041
ENSMUSG00000028096	Gpr89	67549	protein_coding	501.4979689	-0.008633798	0.13800115	0.062563236	0.950114305	0.975869522	0.008633798
ENSMUSG00000041605	Inava	67313	protein_coding	1456.510761	-0.008664318	0.097799665	0.088592515	0.929405757	0.966128972	0.008664318
ENSMUSG00000000794	Kcnn3	140493	protein_coding	3.392877007	0.008668059	2.004069919	-0.004325228	0.996548978	1	0.008668059
ENSMUSG00000028397	Kdm4c	76804	protein_coding	375.5730967	-0.008731003	0.066974699	0.130362713	0.896279466	0.948996349	0.008731003
ENSMUSG00000001569	Nom1	433864	protein_coding	388.9805868	-0.008759657	0.096792114	0.090499699	0.927890133	0.965665343	0.008759657
ENSMUSG00000021615	Xrcc4	108138	protein_coding	99.6666059	0.00876372	0.119143263	-0.073556153	0.941363562	0.971787076	0.00876372
ENSMUSG00000001029	Icam2	15896	protein_coding	1.992939436	-0.008776091	1.23336237	0.007115582	0.994322635	1	0.008776091
ENSMUSG00000096995	2810029C07Rik	100504687	lncRNA	3.696688395	0.008788029	0.626796275	-0.014020551	0.988813585	1	0.008788029
ENSMUSG00000032666	1700025G04Rik	69399	protein_coding	200.5153835	-0.008807771	0.20016779	0.044001938	0.964902859	0.982587667	0.008807771
ENSMUSG00000077416	Gm23747	115485979	snoRNA	0.636004718	-0.008876334	1.360341896	0.006525076	0.994793779	1	0.008876334
ENSMUSG00000052926	Rnaseh2a	69724	protein_coding	267.2111017	-0.008887917	0.099334324	0.089474777	0.928704598	0.966007248	0.008887917
ENSMUSG00000005069	Pex5	19305	protein_coding	508.6938507	0.00889231	0.069698755	-0.127582048	0.898479739	0.950146927	0.00889231
ENSMUSG00000075272	Ttc30a2	620631	protein_coding	1.201385977	0.008898676	1.54841237	-0.005746968	0.995414609	1	0.008898676
ENSMUSG00000019470	Xab2	67439	protein_coding	536.9825064	-0.008914498	0.051245391	0.173957058	0.86189921	0.931842966	0.008914498
ENSMUSG00000046380	Jrk	16469	protein_coding	68.82618931	0.008922725	0.159041539	-0.056103112	0.955259665	0.977698054	0.008922725
ENSMUSG00000063849	Ppcdc	66812	protein_coding	181.1365283	-0.008925205	0.10909585	0.081810674	0.934797268	0.969249832	0.008925205
ENSMUSG00000048379	Socs4	67296	protein_coding	462.5769241	-0.008933254	0.066968319	0.133395226	0.89388082	0.947705869	0.008933254
ENSMUSG00000038347	Tcte2	21646	protein_coding	19.58984813	-0.008933736	0.380992045	0.023448615	0.981292426	0.98994327	0.008933736
ENSMUSG00000095325	Zfp870	240066	protein_coding	82.91614451	-0.008950311	0.114891911	0.077902012	0.937905999	0.970517013	0.008950311
ENSMUSG00000036437	Npy1r	18166	protein_coding	47.4759346	-0.008957873	0.33688795	0.026590067	0.978786696	0.988831409	0.008957873
ENSMUSG00000020230	Prmt2	15468	protein_coding	168.77251	0.008971286	0.138807184	-0.064631278	0.94846758	0.975173098	0.008971286
ENSMUSG00000039599	Fam149b	105428	protein_coding	366.7437944	0.008974215	0.060497053	-0.148341367	0.882073371	0.941960269	0.008974215
ENSMUSG00000051000	Fhip1a	229488	protein_coding	354.0945245	0.008981453	0.119264705	-0.075306878	0.939970549	0.971381329	0.008981453
ENSMUSG00000039578	Ccser1	232035	protein_coding	347.5532824	0.009062499	0.108994317	-0.083146526	0.933735032	0.968634984	0.009062499

ensembl_gene_id	external_gene_name	entrezgene_id	gene_biotype	baseMean	log2FoldChange	lfcSE	stat	pvalue	padj	absLog2FC
ENSMUSG00000035936	Aldh5a1	214579	protein_coding	231.7828807	-0.009074105	0.115147586	0.078804129	0.93718842	0.970043107	0.009074105
ENSMUSG00000049620	Prss33	353130	protein_coding	1.454541405	-0.009172701	1.527196402	0.006006235	0.995207747	1	0.009172701
ENSMUSG00000020544	Cox11	69802	protein_coding	84.69299495	0.009178265	0.125078877	-0.073379816	0.941503879	0.971840719	0.009178265
ENSMUSG00000005674	Tomm40l	641376	protein_coding	130.8454661	-0.009198506	0.116531896	0.078935517	0.937083913	0.970043107	0.009198506
ENSMUSG00000026805	Barhl1	54422	protein_coding	1.510755249	-0.009268455	1.136153484	0.008157749	0.99349113	1	0.009268455
ENSMUSG00000020386	Sar1b	66397	protein_coding	618.6740408	0.00933838	0.069215921	-0.134916642	0.892677781	0.947202684	0.00933838
ENSMUSG00000042814	Mcts2	66405	protein_coding	67.81762597	0.009354106	0.15543151	-0.06018153	0.952011056	0.976016991	0.009354106
ENSMUSG00000047996	Prrg1	546336	protein_coding	27.09399471	0.009354425	0.26193705	-0.035712492	0.97151161	0.985415068	0.009354425
ENSMUSG00000040213	Kyat3	229905	protein_coding	92.04096636	0.009355696	0.135164194	-0.069217265	0.94481668	0.973465352	0.009355696
ENSMUSG00000027852	Nras	18176	protein_coding	643.3402371	-0.009369624	0.068822925	0.13614103	0.891709792	0.947050968	0.009369624
ENSMUSG00000022159	Rab2b	76338	protein_coding	10.7732856	-0.009378499	0.369000046	0.025415984	0.979723162	0.989126875	0.009378499
ENSMUSG00000058318	Phf21a	192285	protein_coding	202.5891137	0.009378697	0.128913814	-0.072751685	0.942003718	0.971883559	0.009378697
ENSMUSG00000018554	Ybx2	53422	protein_coding	119.0188919	0.009385832	0.116589126	-0.080503497	0.935836815	0.96968875	0.009385832
ENSMUSG00000035765	Dym	69190	protein_coding	512.696312	0.009389227	0.087378563	-0.107454578	0.914428358	0.95811623	0.009389227
ENSMUSG00000021338	Carmil1	68732	protein_coding	538.9739354	-0.0094165	0.05498454	0.171257224	0.864021514	0.932456625	0.0094165
ENSMUSG00000048787	Dcun1d3	233805	protein_coding	144.387298	0.009436228	0.090901361	-0.103807337	0.917322244	0.959501353	0.009436228
ENSMUSG00000026803	Ttf1	22130	protein_coding	342.748089	0.009440753	0.065855941	-0.143354621	0.88601012	0.94348537	0.009440753
ENSMUSG00000026036	Nif3l1	65102	protein_coding	230.3399605	0.009479147	0.116630205	-0.08127523	0.935223072	0.969552109	0.009479147
ENSMUSG00000032897	Nfyc	18046	protein_coding	350.2025112	-0.009483748	0.091382237	0.103781086	0.917343076	0.959501353	0.009483748
ENSMUSG00000020453	Patz1	56218	protein_coding	106.39046	0.009489831	0.128678664	-0.073748285	0.941210677	0.971762583	0.009489831
ENSMUSG00000051435	Fhad1	329977	protein_coding	43.56735579	0.009495463	0.201404221	-0.047146296	0.96239663	0.981474584	0.009495463
ENSMUSG00000036109	Mbnl3	171170	protein_coding	348.1156329	0.00949969	0.119743567	-0.079333615	0.93676727	0.969910941	0.00949969
ENSMUSG00000029260	Ugt2b34	100727	protein_coding	6973.445709	0.009502066	0.085250703	-0.111460262	0.911251375	0.956997404	0.009502066
ENSMUSG00000028416	Bag1	12017	protein_coding	2074.95436	0.009512225	0.080552302	-0.11808756	0.90599828	0.953990085	0.009512225
ENSMUSG00000005802	Slc30a4	22785	protein_coding	1683.268306	-0.009568772	0.095579894	0.10011281	0.920254765	0.961475844	0.009568772
ENSMUSG00000031776	Arl2bp	107566	protein_coding	773.8108721	-0.009589563	0.057376053	0.167135288	0.8672636	0.934002289	0.009589563
ENSMUSG00000016833	Mrps18c	68735	protein_coding	217.917945	0.009603499	0.075536053	-0.127137956	0.898831211	0.950352959	0.009603499
ENSMUSG00000037112	Sik2	235344	protein_coding	210.98679	0.009610915	0.086513717	-0.11109123	0.911544003	0.956997404	0.009610915
ENSMUSG00000027552	E2f5	13559	protein_coding	330.6323299	0.009657573	0.073606918	-0.131204698	0.89561338	0.948731562	0.009657573
ENSMUSG00000063808	Gpatch1	67471	protein_coding	225.8791205	0.009721343	0.090323953	-0.107627519	0.914291167	0.95803327	0.009721343
ENSMUSG00000020705	Ddx42	72047	protein_coding	840.3238963	0.009728521	0.070495604	-0.138001806	0.89023899	0.946097348	0.009728521
ENSMUSG00000015112	Slc25a13	50799	protein_coding	626.7551711	0.009741484	0.067620471	-0.144061168	0.885452168	0.943195324	0.009741484
ENSMUSG00000038374	Rbm8a	60365	protein_coding	459.2819631	-0.009769357	0.072406041	0.134924615	0.892671478	0.947202684	0.009769357
ENSMUSG00000021298	Gpr132	56696	protein_coding	3.972508303	0.009802164	0.862069397	-0.011370504	0.990927846	1	0.009802164
ENSMUSG00000039164	Naif1	71254	protein_coding	113.9431011	0.009817848	0.13326835	-0.073669765	0.941273157	0.971762583	0.009817848
ENSMUSG00000002771	Grin2d	14814	protein_coding	32.23219669	-0.009844181	0.266899591	0.036883461	0.970577927	0.984770701	0.009844181
ENSMUSG00000041977	Arhgef11	213498	protein_coding	1145.033729	0.009862416	0.047147526	-0.209182038	0.834306134	0.916804065	0.009862416
ENSMUSG00000029014	Dnajc2	22791	protein_coding	397.459667	-0.009864897	0.089817776	0.109832348	0.912542336	0.957266079	0.009864897
ENSMUSG00000045350	Fam186a	72277	protein_coding	3.76491478	-0.009888454	2.379920457	0.004154951	0.996684838	1	0.009888454

ensembl_gene_id	external_gene_name	entrezgene_id	gene_biotype	baseMean	log2FoldChange	lfcSE	stat	pvalue	padj	absLog2FC
ENSMUSG00000024137	E4f1	13560	protein_coding	263.5657342	-0.009944488	0.110175287	0.0902606	0.928080129	0.965802209	0.009944488
ENSMUSG00000032030	Cul5	75717	protein_coding	425.8412887	-0.00995262	0.109427858	0.090951433	0.927531182	0.965445424	0.00995262
ENSMUSG00000038456	Dennd2a	209773	protein_coding	4.693304854	-0.00995431	0.991806042	0.010036549	0.991992127	0.995389267	0.00995431
ENSMUSG00000026822	Lcn2	16819	protein_coding	2.010435989	-0.009978936	2.163927302	0.004611493	0.996320574	1	0.009978936
ENSMUSG00000032643	Fhl3	14201	protein_coding	5.796376185	0.009996746	0.464647701	-0.021514678	0.982835095	0.990693936	0.009996746
ENSMUSG00000039176	Polg	18975	protein_coding	883.165952	-0.010000317	0.046617144	0.214520161	0.830141447	0.914175515	0.010000317
ENSMUSG00000031585	Gtf2e2	68153	protein_coding	230.7441799	0.010000963	0.089017762	-0.112347949	0.910547525	0.956641558	0.010000963
ENSMUSG00000054770	Kctd18	51960	protein_coding	95.61795713	-0.010049174	0.148775782	0.067545766	0.946147229	0.973795719	0.010049174
ENSMUSG00000002455	Prpf6	68879	protein_coding	744.3488081	0.010088513	0.082018384	-0.123003069	0.902104668	0.951530343	0.010088513
ENSMUSG00000090925	1810064F22Rik	69862	lncRNA	4.760376233	-0.010115539	0.50517436	0.020023857	0.984024341	0.991348095	0.010115539
ENSMUSG00000032112	Trappc4	60409	protein_coding	328.602505	0.010131836	0.075869833	-0.133542351	0.893764473	0.947692153	0.010131836
ENSMUSG00000021900	Btd	26363	protein_coding	140.2679393	0.010136004	0.133387966	-0.075988896	0.939427933	0.971185231	0.010136004
ENSMUSG00000024283	Wac	225131	protein_coding	864.3090866	0.010141492	0.058612639	-0.173025687	0.862631237	0.932166328	0.010141492
ENSMUSG00000047822	Angptl8	624219	protein_coding	17.46117535	-0.010201645	0.265867387	0.038371177	0.969391741	0.984403993	0.010201645
ENSMUSG00000003166	Dgcr2	13356	protein_coding	894.7944471	-0.010207429	0.116114411	0.087908372	0.929949502	0.966286169	0.010207429
ENSMUSG00000030330	Ing4	28019	protein_coding	17.15423099	0.010230163	0.365547396	-0.027985873	0.977673418	0.988088396	0.010230163
ENSMUSG00000026275	Ppp1r7	66385	protein_coding	400.9615479	0.010252637	0.102334536	-0.100187461	0.920195501	0.961475844	0.010252637
ENSMUSG00000036202	Rif1	51869	protein_coding	594.6160475	-0.010298591	0.094694966	0.108755419	0.913396485	0.957864571	0.010298591
ENSMUSG00000030380	Mzf1	109889	protein_coding	14.96875606	0.010300221	0.417009948	-0.024700179	0.980294112	0.9893404	0.010300221
ENSMUSG00000022895	Ets2	23872	protein_coding	2899.056956	0.010307638	0.06601214	-0.156147614	0.875916668	0.938630585	0.010307638
ENSMUSG00000042492	Tbc1d10b	68449	protein_coding	672.8799773	0.010327065	0.076928509	-0.134242364	0.893210927	0.947365784	0.010327065
ENSMUSG00000021067	Sav1	64010	protein_coding	267.375535	0.010334551	0.091492881	-0.112954702	0.910066468	0.956337108	0.010334551
ENSMUSG00000022019	Tdrd3	219249	protein_coding	409.8904133	0.010356888	0.065434707	-0.1582782	0.874237585	0.93820896	0.010356888
ENSMUSG00000015994	Fnta	14272	protein_coding	543.2609857	0.010370724	0.081093775	-0.12788558	0.898239523	0.949998977	0.010370724
ENSMUSG00000029192	Tbc1d14	100855	protein_coding	407.283084	-0.010378651	0.077842484	0.133328881	0.893933288	0.947705869	0.010378651
ENSMUSG00000058638	Zfp110	65020	protein_coding	548.2514821	-0.01045217	0.08143559	0.128348925	0.897872848	0.949809251	0.01045217
ENSMUSG00000030838	Ush1c	72088	protein_coding	2531.551252	0.01045261	0.083411025	-0.125314488	0.900274583	0.95096289	0.01045261
ENSMUSG00000003051	Elf3	13710	protein_coding	5854.295706	-0.010470057	0.083325748	0.12565212	0.900007304	0.950848993	0.010470057
ENSMUSG00000053536	Cstf2t	83410	protein_coding	750.0759245	0.010504451	0.082824764	-0.126827418	0.899076995	0.950413359	0.010504451
ENSMUSG00000060275	Nrg2	100042150	protein_coding	3.471468667	-0.010523041	0.852705403	0.012340769	0.990153741	1	0.010523041
ENSMUSG00000034848	Ttc21b	73668	protein_coding	159.1250609	-0.010534006	0.120422229	0.087475595	0.930293483	0.966400364	0.010534006
ENSMUSG00000005774	Rfx5	53970	protein_coding	124.1583742	-0.010538784	0.098679119	0.106798519	0.914948822	0.958296749	0.010538784
ENSMUSG00000001420	Tmem79	71913	protein_coding	275.1308966	-0.010570764	0.096619911	0.109405651	0.912880751	0.957419485	0.010570764
ENSMUSG00000028653	Trit1	66966	protein_coding	126.2777233	0.010574981	0.124563701	-0.084896169	0.932343937	0.967739018	0.010574981
ENSMUSG00000021028	Mbip	217588	protein_coding	92.57577092	0.010594604	0.137350237	-0.077135679	0.93851561	0.970606637	0.010594604
ENSMUSG00000023089	Ndufa5	68202	protein_coding	850.4439848	-0.01061535	0.085828538	0.123680893	0.901567941	0.951341404	0.01061535
ENSMUSG00000021621	Zcchc9	69085	protein_coding	287.7584964	0.010615756	0.066153636	-0.160471243	0.872509872	0.937146539	0.010615756
ENSMUSG00000064289	Tank	21353	protein_coding	349.1398749	0.010641928	0.090681175	-0.117355428	0.906578404	0.954311305	0.010641928
ENSMUSG00000016559	H3f3b	15081	protein_coding	8355.17645	0.010653336	0.103098112	-0.103332021	0.917699463	0.959656087	0.010653336

ensembl_gene_id	external_gene_name	entrezgene_id	gene_biotype	baseMean	log2FoldChange	lfcSE	stat	pvalue	padj	absLog2FC
ENSMUSG00000074305	Peak1	244895	protein_coding	267.549827	0.010655563	0.093146567	-0.114395655	0.908924158	0.955501531	0.010655563
ENSMUSG00000003379	Cd79a	12518	protein_coding	2.619882015	0.010669654	1.118778056	-0.009536882	0.992390785	1	0.010669654
ENSMUSG00000060288	Ppih	66101	protein_coding	169.0213923	-0.010759792	0.153417359	0.070134124	0.944086907	0.973044134	0.010759792
ENSMUSG00000003134	Tbc1d8	54610	protein_coding	186.2825174	-0.010793067	0.128485499	0.084002217	0.933054669	0.968172466	0.010793067
ENSMUSG00000008690	Ncaph2	52683	protein_coding	1069.225707	0.010803882	0.072258561	-0.149516983	0.88114571	0.941406719	0.010803882
ENSMUSG00000021895	Arhgef3	71704	protein_coding	510.03322	-0.010829743	0.086882139	0.124648672	0.900801694	0.951246263	0.010829743
ENSMUSG00000058624	Gda	14544	protein_coding	1153.46087	-0.010865467	0.139860515	0.077687883	0.938076333	0.970517013	0.010865467
ENSMUSG00000025155	Dus1l	68730	protein_coding	513.0951666	-0.01088832	0.080240276	0.135696447	0.892061257	0.947202684	0.01088832
ENSMUSG00000037104	Socs5	56468	protein_coding	146.6028721	0.010920228	0.144934162	-0.075346126	0.939939323	0.971381329	0.010920228
ENSMUSG00000040029	Ipo8	320727	protein_coding	879.6077946	-0.010924571	0.07029391	0.155412762	0.876495924	0.938630585	0.010924571
ENSMUSG00000039725	Trp53rka	381406	protein_coding	103.6473654	0.010928475	0.103850588	-0.105232678	0.916191181	0.959151705	0.010928475
ENSMUSG00000027079	Clp1	98985	protein_coding	158.176021	-0.01094104	0.100646173	0.108707955	0.913434132	0.957864571	0.01094104
ENSMUSG00000054484	Tmem62	96957	protein_coding	198.9971922	-0.010951323	0.09088738	0.12049333	0.904092363	0.952605199	0.010951323
ENSMUSG00000022412	Mief1	239555	protein_coding	607.0434697	0.010952647	0.064127664	-0.170794417	0.864385419	0.932569867	0.010952647
ENSMUSG00000028792	Ak2	11637	protein_coding	2578.085973	0.01096474	0.069687657	-0.157341209	0.874975945	0.938415991	0.01096474
ENSMUSG00000055065	Ddx17	67040	protein_coding	2704.206293	-0.010994062	0.074280328	0.148007723	0.882336673	0.941973978	0.010994062
ENSMUSG00000078716	Tmem8b	242409	protein_coding	38.21706924	-0.011001281	0.179963803	0.061130523	0.95125526	0.975912221	0.011001281
ENSMUSG00000106219	5830416I19Rik	74757	lncRNA	2.320500007	0.011025299	1.06542769	-0.010348238	0.991743448	1	0.011025299
ENSMUSG00000033004	Mycbp2	105689	protein_coding	900.9162999	0.011048938	0.081811681	-0.135053306	0.892569728	0.947202684	0.011048938
ENSMUSG00000032839	Trpc1	22063	protein_coding	11.23289426	0.011053501	0.383701876	-0.028807523	0.977018101	0.987905762	0.011053501
ENSMUSG00000048874	Phf3	213109	protein_coding	1004.503263	-0.01105393	0.062850163	0.175877514	0.860390171	0.931471303	0.01105393
ENSMUSG00000028719	Cmpk1	66588	protein_coding	5375.843685	0.011069918	0.09444353	-0.117212032	0.906692033	0.954353007	0.011069918
ENSMUSG00000075700	Selenot	69227	protein_coding	1443.96345	0.011107272	0.078491446	-0.141509332	0.887467588	0.944306685	0.011107272
ENSMUSG00000021681	Aggf1	66549	protein_coding	618.6985321	-0.011121304	0.075619904	0.147068474	0.883077973	0.942185661	0.011121304
ENSMUSG00000043079	Synpo	104027	protein_coding	510.0196713	0.011149315	0.180563729	-0.061747257	0.950764106	0.97587882	0.011149315
ENSMUSG00000026490	Cdc42bpa	226751	protein_coding	758.5877131	0.011162061	0.132641433	-0.084152148	0.932935464	0.968109605	0.011162061
ENSMUSG00000048000	Gigyf2	227331	protein_coding	1118.041789	-0.011178753	0.060142121	0.185872276	0.852544924	0.927761944	0.011178753
ENSMUSG00000013833	Med16	216154	protein_coding	306.3650478	0.011206505	0.100674889	-0.111313807	0.911367507	0.956997404	0.011206505
ENSMUSG00000052387	Trpm3	226025	protein_coding	6.524496426	0.011209403	2.536055899	-0.004420014	0.99647335	0.998060837	0.011209403
ENSMUSG00000090564	A430057M04Rik	319486	lncRNA	8.496378312	-0.011216587	0.450397007	0.024903778	0.980131714	0.989302036	0.011216587
ENSMUSG00000011179	Odc1	18263	protein_coding	2462.948111	0.01122357	0.069848993	-0.160683345	0.872342808	0.937028043	0.01122357
ENSMUSG00000025217	Btrc	12234	protein_coding	214.110568	-0.011224434	0.090426424	0.124127811	0.901214078	0.95132126	0.011224434
ENSMUSG00000038181	Chpf2	100910	protein_coding	250.8384886	-0.011226413	0.101102238	0.111040199	0.91158447	0.956997404	0.011226413
ENSMUSG00000024472	Dcp2	70640	protein_coding	752.9985476	0.011271539	0.104003765	-0.108376259	0.913697233	0.957921881	0.011271539
ENSMUSG00000033014	Trim33	94093	protein_coding	616.634426	0.011272042	0.052575478	-0.214397333	0.830237221	0.914187552	0.011272042
ENSMUSG00000041438	Utp4	21771	protein_coding	215.804727	-0.011292575	0.122946924	0.091849185	0.926817865	0.965096805	0.011292575
ENSMUSG00000068742	Cry2	12953	protein_coding	241.2815847	-0.011304959	0.213060035	0.053059971	0.957684125	0.979208448	0.011304959
ENSMUSG00000014426	Map3k4	26407	protein_coding	775.7514412	-0.011323132	0.056266235	0.20124205	0.840509313	0.920924283	0.011323132
ENSMUSG00000112352	Gm40617	105245123	lncRNA	1.042732304	-0.011331091	1.629269024	0.006954709	0.99445099	1	0.011331091

ensembl_gene_id	external_gene_name	entrezgene_id	gene_biotype	baseMean	log2FoldChange	lfcSE	stat	pvalue	padj	absLog2FC
ENSMUSG00000074071	Fam169b	434197	protein_coding	4.123447684	0.011369419	1.09455405	-0.010387262	0.991712313	1	0.011369419
ENSMUSG00000044034	Npb	208990	protein_coding	2.67898881	-0.011374784	0.670709946	0.016959319	0.986469069	1	0.011374784
ENSMUSG00000045294	Insig1	231070	protein_coding	1310.999947	0.011391345	0.176441721	-0.064561516	0.948523127	0.975173098	0.011391345
ENSMUSG00000020925	Ccdc43	52715	protein_coding	253.4470209	-0.011406851	0.081448831	0.140049286	0.888621049	0.94517522	0.011406851
ENSMUSG00000020109	Dnajb12	56709	protein_coding	641.1710569	0.011419138	0.072669693	-0.157137558	0.875136438	0.938415991	0.011419138
ENSMUSG00000046168	Kcnrg	328424	protein_coding	2.776361337	0.011449021	0.709068775	-0.016146559	0.98711747	1	0.011449021
ENSMUSG00000029313	Aff1	17355	protein_coding	1129.378958	-0.011465708	0.092697075	0.12369007	0.901560674	0.951341404	0.011465708
ENSMUSG00000032512	Wdr48	67561	protein_coding	787.9940572	0.011473579	0.089169751	-0.128671197	0.897617827	0.949716026	0.011473579
ENSMUSG00000051251	Nhlh1	18071	protein_coding	1.461124368	-0.01147461	2.083152036	0.005508292	0.995605041	1	0.01147461
ENSMUSG00000027167	Elp4	77766	protein_coding	141.4407399	0.011493877	0.118396247	-0.097079744	0.922663066	0.962530504	0.011493877
ENSMUSG00000058921	Slc10a5	241877	protein_coding	724.6548025	-0.011507391	0.085378315	0.134781193	0.892784876	0.947205958	0.011507391
ENSMUSG00000079033	Mef2b	17259	protein_coding	0.644317546	-0.011509836	1.590380883	0.007237157	0.994225634	1	0.011509836
ENSMUSG00000043289	Mei4	75033	protein_coding	1.20532104	0.011525254	1.005734511	-0.01145954	0.99085681	1	0.011525254
ENSMUSG00000042111	Ccdc115	69668	protein_coding	193.7637046	0.011535229	0.126083331	-0.091488928	0.927104104	0.965151432	0.011535229
ENSMUSG00000020143	Dock2	94176	protein_coding	25.03219337	0.011563189	0.413276496	-0.027979305	0.977678657	0.988088396	0.011563189
ENSMUSG00000038375	Trp53inp2	68728	protein_coding	1171.893606	0.011569362	0.139459849	-0.082958374	0.933884638	0.968697092	0.011569362
ENSMUSG00000039043	Arpin	70420	protein_coding	765.6526823	0.011590682	0.07848448	-0.147681204	0.882594366	0.941973978	0.011590682
ENSMUSG00000006574	Slc4a1	20533	protein_coding	7.440871256	-0.011594929	0.919993543	0.012603272	0.98994431	0.994279305	0.011594929
ENSMUSG00000108030	9530062K07Rik	78739	lncRNA	4.217707595	-0.011620401	0.680284635	0.017081676	0.986371458	1	0.011620401
ENSMUSG00000021646	Mccc2	78038	protein_coding	315.6190122	-0.011629262	0.129079524	0.090093782	0.92821269	0.965818434	0.011629262
ENSMUSG00000046020	Pofut1	140484	protein_coding	696.6322376	0.011629759	0.093510495	-0.124368488	0.901023522	0.95132126	0.011629759
ENSMUSG00000074211	Sdhaf1	68332	protein_coding	77.86100818	0.011641221	0.157581313	-0.073874377	0.941110345	0.971762583	0.011641221
ENSMUSG00000021785	Ngly1	59007	protein_coding	815.3799671	-0.011651673	0.079736461	0.14612729	0.883820903	0.942430322	0.011651673
ENSMUSG00000035620	Ric8b	237422	protein_coding	234.6549374	0.011660412	0.091870289	-0.126922558	0.899001692	0.950394593	0.011660412
ENSMUSG00000002103	Acp2	11432	protein_coding	330.5189153	-0.011731494	0.079332507	0.147877517	0.882439432	0.941973978	0.011731494
ENSMUSG00000051246	Msantd1	403174	protein_coding	18.63968727	-0.011739617	0.265070368	0.044288682	0.964674293	0.982587667	0.011739617
ENSMUSG00000021062	Rab15	104886	protein_coding	1200.151933	-0.011751116	0.19158962	0.061334826	0.951092556	0.97587882	0.011751116
ENSMUSG00000027613	Eif6	16418	protein_coding	2695.659702	-0.011768204	0.071870484	0.163741822	0.869934393	0.935658236	0.011768204
ENSMUSG00000063358	Mapk1	26413	protein_coding	1667.886836	0.01176868	0.069978768	-0.168175009	0.866445601	0.933791206	0.01176868
ENSMUSG00000036769	Wdr44	72404	protein_coding	398.2544898	0.011828659	0.099249744	-0.11918075	0.905132158	0.953251304	0.011828659
ENSMUSG00000007659	Bcl2l1	12048	protein_coding	498.2058197	0.011847126	0.090134548	-0.131438237	0.895428643	0.948680909	0.011847126
ENSMUSG00000039765	Cc2d2a	231214	protein_coding	247.705684	0.011849771	0.106888378	-0.110861169	0.911726438	0.956997404	0.011849771
ENSMUSG00000025371	Chmp6	208092	protein_coding	905.792454	0.01189594	0.082247517	-0.144635856	0.884998386	0.943016089	0.01189594
ENSMUSG00000032046	Abhd12	76192	protein_coding	412.1978032	-0.011912247	0.097467194	0.122218019	0.902726356	0.95200802	0.011912247
ENSMUSG00000026203	Dnajb2	56812	protein_coding	271.1336459	-0.01193962	0.107231785	0.11134404	0.911343533	0.956997404	0.01193962
ENSMUSG00000034940	Synrg	217030	protein_coding	581.6215687	0.011946745	0.077236928	-0.1546766	0.877076279	0.938936708	0.011946745
ENSMUSG00000039480	Nt5dc1	319638	protein_coding	241.1999973	0.011979355	0.08956918	-0.133744166	0.89360488	0.947601039	0.011979355
ENSMUSG00000064345	mt-Nd2	17717	protein_coding	13660.23877	0.011992535	0.419465109	-0.028590066	0.977191535	0.988020633	0.011992535
ENSMUSG00000029676	Pot1a	101185	protein_coding	144.8832799	-0.01200693	0.104913165	0.114446367	0.90888396	0.955501531	0.01200693

ensembl_gene_id	external_gene_name	entrezgene_id	gene_biotype	baseMean	log2FoldChange	lfcSE	stat	pvalue	padj	absLog2FC
ENSMUSG00000052139	Babam2	107976	protein_coding	334.1012628	0.012021489	0.058990629	-0.203786419	0.838520406	0.919582966	0.012021489
ENSMUSG00000028701	Lurap1	68075	protein_coding	29.9416668	0.012076745	0.209778181	-0.057569119	0.954091848	0.977047797	0.012076745
ENSMUSG00000031605	Klhl2	77113	protein_coding	536.0524713	0.012077255	0.052922375	-0.228206966	0.819485346	0.908985828	0.012077255
ENSMUSG00000028622	Mrpl37	56280	protein_coding	438.9917312	-0.012083889	0.095840662	0.126083108	0.899666138	0.950671014	0.012083889
ENSMUSG00000033009	Ogfod1	270086	protein_coding	307.8644066	0.012098457	0.10914587	-0.110846679	0.911737929	0.956997404	0.012098457
ENSMUSG00000032890	Rims3	242662	protein_coding	3.787188464	-0.012122079	1.048114905	0.011565601	0.990772191	1	0.012122079
ENSMUSG00000030327	Necap1	67602	protein_coding	420.1771001	0.012134177	0.080344287	-0.151027253	0.879954218	0.940676072	0.012134177
ENSMUSG00000057133	Chd6	71389	protein_coding	651.3093249	0.0121375	0.102275441	-0.118674633	0.905533134	0.953575703	0.0121375
ENSMUSG00000064994	Gm22422	115486004	snoRNA	101.5692616	-0.012150737	0.283218032	0.042902413	0.965779325	0.982739526	0.012150737
ENSMUSG00000042190	Cmklr1	14747	protein_coding	1.709466073	-0.012170081	1.700768002	0.007155639	0.994290675	1	0.012170081
ENSMUSG00000037149	Ddx1	104721	protein_coding	1343.195508	0.012190208	0.057450511	-0.212186236	0.831961744	0.915295615	0.012190208
ENSMUSG00000043931	Gimap7	231932	protein_coding	7.158845744	0.012199285	0.515009193	-0.023687509	0.98110187	0.989913623	0.012199285
ENSMUSG00000051498	Tlr6	21899	protein_coding	12.05174064	-0.012245484	0.34148159	0.035859867	0.971394097	0.985415068	0.012245484
ENSMUSG00000022529	Zfp263	74120	protein_coding	367.6305684	-0.012253144	0.071682011	0.170937508	0.864272903	0.932569867	0.012253144
ENSMUSG00000028559	Osbpl9	100273	protein_coding	822.1042299	-0.012255649	0.073043506	0.167785602	0.86675195	0.933837244	0.012255649
ENSMUSG00000089824	Rbm12	75710	protein_coding	139.0799931	-0.012285136	0.106389741	0.115472937	0.90807027	0.955211953	0.012285136
ENSMUSG00000010517	Faf1	14084	protein_coding	786.1381523	0.012285373	0.084013692	-0.14623061	0.883739342	0.942424291	0.012285373
ENSMUSG00000026064	Ptp4a1	19243	protein_coding	206.3697609	0.012285901	0.116803825	-0.105184067	0.916229754	0.959151705	0.012285901
ENSMUSG00000021254	Gpatch2l	70373	protein_coding	256.5887878	0.012289539	0.094252349	-0.130389737	0.896258086	0.948996349	0.012289539
ENSMUSG00000020307	Cdc34	216150	protein_coding	568.2422057	0.012289872	0.086357376	-0.142314092	0.886831916	0.94399288	0.012289872
ENSMUSG00000022330	Osr2	107587	protein_coding	455.5944004	-0.01231549	0.312552673	0.039402928	0.968569145	0.984122985	0.01231549
ENSMUSG00000060397	Zfp128	243833	protein_coding	21.12053501	0.012332229	0.228685995	-0.053926474	0.956993744	0.978805568	0.012332229
ENSMUSG00000036617	Etl4	208618	protein_coding	1473.072987	0.012355725	0.129880794	-0.095131269	0.924210562	0.963320302	0.012355725
ENSMUSG00000000787	Ddx3x	13205	protein_coding	4356.106278	-0.012388067	0.084942991	0.145839777	0.884047874	0.942489774	0.012388067
ENSMUSG00000059897	Zfp930	234358	protein_coding	106.4358003	-0.012420403	0.154181496	0.080557026	0.935794244	0.96968875	0.012420403
ENSMUSG00000030551	Nr2f2	11819	protein_coding	131.1809016	0.012433848	0.129672212	-0.09588676	0.92361051	0.962971397	0.012433848
ENSMUSG00000038695	Josd2	66124	protein_coding	227.921247	0.012437641	0.104240567	-0.119316702	0.905024452	0.953222387	0.012437641
ENSMUSG00000041765	Ubac2	68889	protein_coding	418.1486938	-0.012440761	0.054298002	0.229120055	0.818775607	0.908812719	0.012440761
ENSMUSG00000044678	Ly6k	76486	protein_coding	0.767144529	0.01245404	1.465317058	-0.008499212	0.993218692	1	0.01245404
ENSMUSG00000032899	Styk1	243659	protein_coding	1483.075253	0.012461972	0.097487478	-0.127831518	0.898282307	0.949998977	0.012461972
ENSMUSG00000020926	Adam11	11488	protein_coding	4.708587363	0.012480511	0.931843851	-0.013393351	0.989313972	0.99400894	0.012480511
ENSMUSG00000026596	Abl2	11352	protein_coding	346.3525722	0.012493361	0.086667301	-0.144153105	0.885379571	0.943195324	0.012493361
ENSMUSG00000056214	Pard6g	93737	protein_coding	11.91660895	-0.012585939	0.291700957	0.043146718	0.965584578	0.982706396	0.012585939
ENSMUSG00000055371	Stam2	56324	protein_coding	952.5603293	0.012586483	0.065965136	-0.190805075	0.848678312	0.925637253	0.012586483
ENSMUSG00000021870	Slmap	83997	protein_coding	1221.459757	0.012598253	0.069559118	-0.181115767	0.856276709	0.930174321	0.012598253
ENSMUSG00000025326	Ube3a	22215	protein_coding	760.263516	0.012635455	0.109701051	-0.115180797	0.908301819	0.955333816	0.012635455
ENSMUSG00000026812	Tsc1	64930	protein_coding	442.0451754	0.012660113	0.068542724	-0.184703967	0.853461235	0.92805494	0.012660113
ENSMUSG00000027472	Pdrg1	68559	protein_coding	211.0248283	-0.01267436	0.088781966	0.142758268	0.886481097	0.943865173	0.01267436
ENSMUSG00000040410	Fbxl4	269514	protein_coding	173.1393668	0.012677035	0.114644179	-0.110577225	0.911951608	0.957100023	0.012677035

ensembl_gene_id	external_gene_name	entrezgene_id	gene_biotype	baseMean	log2FoldChange	lfcSE	stat	pvalue	padj	absLog2FC
ENSMUSG00000042133	Ppig	228005	protein_coding	563.1150185	0.0126772	0.06633441	-0.191110467	0.848439047	0.925637253	0.0126772
ENSMUSG00000033684	Qsox1	104009	protein_coding	1499.281415	0.012688605	0.211503345	-0.059992456	0.952161643	0.976016991	0.012688605
ENSMUSG00000022200	Golph3	66629	protein_coding	2056.407873	0.012702154	0.074065441	-0.171499065	0.863831366	0.932373279	0.012702154
ENSMUSG00000097690	4930505N22Rik	100174921	lncRNA	3.853838291	-0.012707235	0.615118635	0.020658185	0.983518325	1	0.012707235
ENSMUSG00000031553	Adam3	11497	protein_coding	1.634193641	0.012755832	2.126965908	-0.005997196	0.995214958	1	0.012755832
ENSMUSG00000022858	Tra2b	20462	protein_coding	1032.782985	0.012803723	0.084652942	-0.151249595	0.879778829	0.940676072	0.012803723
ENSMUSG00000039068	Zzz3	108946	protein_coding	709.3471847	-0.012832101	0.081304141	0.157828387	0.87459203	0.938406345	0.012832101
ENSMUSG00000032300	1700017B05Rik	74211	protein_coding	139.0539572	-0.012899634	0.158364378	0.081455399	0.935079793	0.9694819	0.012899634
ENSMUSG00000017119	Nbr1	17966	protein_coding	2987.962052	-0.012946497	0.049682337	0.260585519	0.794412158	0.897695523	0.012946497
ENSMUSG00000018040	Rrp7a	74778	protein_coding	589.8311815	0.012998938	0.075241697	-0.172762429	0.86283817	0.932166328	0.012998938
ENSMUSG00000029684	Wasl	73178	protein_coding	821.3664904	0.013017536	0.074492075	-0.174750615	0.861275595	0.931649419	0.013017536
ENSMUSG00000041836	Ptpre	19267	protein_coding	151.7560572	-0.013017718	0.107805548	0.120751837	0.9038876	0.952450218	0.013017718
ENSMUSG00000079554	Aox2	213043	protein_coding	9.45613553	0.013034423	0.631550621	-0.020638762	0.98353382	0.990974832	0.013034423
ENSMUSG00000044477	Zfand3	21769	protein_coding	791.2260901	-0.013047082	0.074668159	0.174734219	0.861288478	0.931649419	0.013047082
ENSMUSG00000032905	Atg12	67526	protein_coding	530.130965	-0.013093908	0.06948659	0.188437906	0.850533387	0.927048271	0.013093908
ENSMUSG00000020752	Recql5	170472	protein_coding	596.0024789	0.013163109	0.142326614	-0.092485224	0.926312531	0.964753099	0.013163109
ENSMUSG00000026088	Mitd1	69028	protein_coding	180.1034309	0.013199815	0.095276131	-0.138542724	0.889811506	0.945840699	0.013199815
ENSMUSG00000100017	2410022M11Rik	100503842	lncRNA	38.13878163	0.013225859	0.220483875	-0.059985605	0.9521671	0.976016991	0.013225859
ENSMUSG00000025393	Atp5b	11947	protein_coding	25849.47995	0.013252596	0.074599926	-0.177648917	0.858998706	0.931099354	0.013252596
ENSMUSG00000030846	Tial1	21843	protein_coding	430.7337551	-0.01325432	0.085885169	0.154326059	0.877352651	0.939157889	0.01325432
ENSMUSG00000062619	2310039H08Rik	67101	protein_coding	220.96019	-0.013260125	0.085294635	0.155462594	0.876456641	0.938630585	0.013260125
ENSMUSG00000091896	Ube2d2a	56550	protein_coding	459.5165296	-0.013285755	0.073475196	0.180819588	0.856509188	0.930184975	0.013285755
ENSMUSG00000028894	Inpp5b	16330	protein_coding	345.7042876	0.013336644	0.071128728	-0.18750011	0.851268538	0.927421073	0.013336644
ENSMUSG00000055128	Cgrrf1	68755	protein_coding	193.0279761	0.013391021	0.086161874	-0.155417015	0.876492571	0.938630585	0.013391021
ENSMUSG00000033808	Tmem87a	211499	protein_coding	865.3422952	-0.013419428	0.087306592	0.153704636	0.877842629	0.9393553	0.013419428
ENSMUSG00000016308	Ube2a	22209	protein_coding	398.5669924	0.013509571	0.087719428	-0.15400888	0.877602734	0.93927028	0.013509571
ENSMUSG00000034361	Cpne2	234577	protein_coding	282.6125162	-0.013582262	0.114006051	0.119136322	0.905167355	0.953251304	0.013582262
ENSMUSG00000064030	Pym1	78428	protein_coding	186.0286792	0.013583248	0.080338828	-0.169074516	0.865738029	0.933333191	0.013583248
ENSMUSG00000021457	Syk	20963	protein_coding	1050.287954	-0.013589613	0.119692243	0.113537962	0.909604069	0.956044724	0.013589613
ENSMUSG00000027996	Sfrp2	20319	protein_coding	0.840895792	0.013612184	1.858973685	-0.007322419	0.994157607	1	0.013612184
ENSMUSG00000055239	Kcmf1	74287	protein_coding	648.7647878	0.013754272	0.060924393	-0.225759692	0.821388329	0.909752305	0.013754272
ENSMUSG00000045095	Magi1	14924	protein_coding	1030.148274	-0.013818769	0.06912502	0.199909803	0.841551124	0.92158767	0.013818769
ENSMUSG00000031095	Cul4b	72584	protein_coding	326.7166549	-0.013836281	0.077968468	0.177459963	0.859147111	0.931099354	0.013836281
ENSMUSG00000049396	Gemin4	276919	protein_coding	8.281882771	0.013874443	0.390506081	-0.035529391	0.97165761	0.985442104	0.013874443
ENSMUSG00000006673	Qrich1	69232	protein_coding	1073.185919	-0.013875186	0.046583419	0.297856758	0.765812493	0.882651278	0.013875186
ENSMUSG00000073079	Srp54a	24067	protein_coding	59.59895515	-0.013947643	0.20499298	0.068039613	0.9457541	0.973795719	0.013947643
ENSMUSG00000038459	Abhd17c	70178	protein_coding	1220.519893	0.013952095	0.109614167	-0.127283686	0.898715872	0.950335782	0.013952095
ENSMUSG00000024571	Naa12	27366	protein_coding	515.9380068	0.013962667	0.094421859	-0.147875364	0.882441131	0.941973978	0.013962667
ENSMUSG00000025939	Ube2w	66799	protein_coding	244.1483062	0.013963667	0.1065207	-0.131088769	0.895705086	0.948731562	0.013963667

ensembl_gene_id	external_gene_name	entrezgene_id	gene_biotype	baseMean	log2FoldChange	lfcSE	stat	pvalue	padj	absLog2FC
ENSMUSG00000020694	Tlk2	24086	protein_coding	430.3904274	-0.013983682	0.058183834	0.240336204	0.810069631	0.906149826	0.013983682
ENSMUSG00000042726	Trafd1	231712	protein_coding	404.7229248	-0.014039949	0.123167974	0.113990257	0.909245517	0.955778515	0.014039949
ENSMUSG00000019883	Echdc1	52665	protein_coding	175.1063838	-0.014075545	0.10377942	0.135629448	0.892114224	0.947202684	0.014075545
ENSMUSG00000005501	Usp40	227334	protein_coding	397.6856995	0.014112116	0.070904043	-0.199031188	0.842238348	0.921900136	0.014112116
ENSMUSG00000026755	Arpc5l	74192	protein_coding	433.6306594	-0.014123324	0.09070889	0.155699443	0.876269937	0.938630585	0.014123324
ENSMUSG00000030774	Pak1	18479	protein_coding	957.8264183	-0.014132872	0.062206055	0.227194481	0.820272519	0.9092783	0.014132872
ENSMUSG00000021552	Gkap1	56278	protein_coding	26.73281413	-0.014137623	0.233818265	0.060464154	0.951785965	0.976016991	0.014137623
ENSMUSG00000019689	Fmc1	66117	protein_coding	290.3046189	0.014164972	0.116834615	-0.121239515	0.903501327	0.952286247	0.014164972
ENSMUSG00000030374	Strn4	97387	protein_coding	1084.00476	0.014191447	0.050767558	-0.279537704	0.779832207	0.890125936	0.014191447
ENSMUSG00000024493	Lars	107045	protein_coding	674.5675802	0.014281483	0.113030251	-0.126350978	0.899454105	0.950621521	0.014281483
ENSMUSG00000079022	Col22a1	69700	protein_coding	5.079403721	0.014340056	1.879475145	-0.007629819	0.993912344	0.996446603	0.014340056
ENSMUSG00000027367	Stard7	99138	protein_coding	1939.735597	0.014413072	0.076143005	-0.189289501	0.849865923	0.926810134	0.014413072
ENSMUSG00000027598	Itch	16396	protein_coding	1421.355536	0.014443171	0.077215552	-0.187050031	0.851621406	0.927499565	0.014443171
ENSMUSG00000027519	Rab22a	19334	protein_coding	770.6433578	-0.014465097	0.073107691	0.197860123	0.843154505	0.922414938	0.014465097
ENSMUSG00000021619	Atg10	66795	protein_coding	45.91046229	-0.01451792	0.16012536	0.090665965	0.927758015	0.965588697	0.01451792
ENSMUSG00000024962	Vegfb	22340	protein_coding	85.51200619	-0.014519983	0.158631036	0.091533056	0.927069042	0.965151432	0.014519983
ENSMUSG00000018102	H2bc4	68024	protein_coding	2182.446874	-0.014543961	0.071015643	0.204799399	0.837728856	0.919158483	0.014543961
ENSMUSG00000034308	Sdr42e1	74032	protein_coding	550.8229917	0.014545815	0.14192852	-0.10248691	0.918370203	0.960053524	0.014545815
ENSMUSG00000036845	Lin37	75660	protein_coding	123.5907519	0.01456435	0.109648948	-0.132827081	0.894330136	0.947822351	0.01456435
ENSMUSG00000030598	Fbxo17	50760	protein_coding	13.75992051	0.014603176	0.306140893	-0.047700833	0.96195467	0.981387498	0.014603176
ENSMUSG00000014932	Yes1	22612	protein_coding	786.8443246	-0.014672519	0.099298981	0.147761019	0.882531374	0.941973978	0.014672519
ENSMUSG00000000581	C1d	57316	protein_coding	392.0780266	-0.01468401	0.08246343	0.178066932	0.858670411	0.930879221	0.01468401
ENSMUSG00000027952	Pmvk	68603	protein_coding	307.2651352	0.014732778	0.079649511	-0.184970105	0.853252484	0.927990748	0.014732778
ENSMUSG00000024614	Tmx3	67988	protein_coding	529.6782414	-0.014736298	0.092153057	0.159911114	0.872951094	0.937394946	0.014736298
ENSMUSG00000029415	Sdad1	231452	protein_coding	252.004233	-0.014736683	0.118671825	0.124180133	0.901172652	0.95132126	0.014736683
ENSMUSG00000072692	Rpl37rt	100502825	transcribed_processed_pseudogene	90.86948823	0.014792855	0.151706788	-0.097509514	0.922321779	0.962478474	0.014792855
ENSMUSG00000029708	Gcc1	74375	protein_coding	363.6904521	-0.014806022	0.068164452	0.217210309	0.828044451	0.913297021	0.014806022
ENSMUSG00000023104	Rfc2	19718	protein_coding	437.7878416	0.014810812	0.10594542	-0.13979662	0.888820684	0.945259194	0.014810812
ENSMUSG00000070777	Ceacam20	71601	protein_coding	1288.142015	-0.014827299	0.236243253	0.062762846	0.949955352	0.975869522	0.014827299
ENSMUSG00000001383	Zmat2	66492	protein_coding	516.7085864	0.014834619	0.050359418	-0.29457488	0.768318654	0.883975099	0.014834619
ENSMUSG00000039849	Pcif1	228866	protein_coding	545.2520912	0.014836494	0.055893215	-0.265443556	0.790667786	0.895609289	0.014836494
ENSMUSG00000038880	Mrps34	79044	protein_coding	147.7618195	-0.014846338	0.129701509	0.114465423	0.908868854	0.955501531	0.014846338
ENSMUSG00000030400	Ercc2	13871	protein_coding	245.2604769	0.014880085	0.076253902	-0.195138669	0.845284391	0.923801556	0.014880085
ENSMUSG00000022557	Bop1	12181	protein_coding	559.7687859	0.014929909	0.084142868	-0.177435226	0.85916654	0.931099354	0.014929909
ENSMUSG00000019808	Adat2	66757	protein_coding	45.17246423	0.014932681	0.177326103	-0.084210279	0.932889246	0.968109605	0.014932681
ENSMUSG00000046727	Cystm1	66060	protein_coding	3074.971537	0.015085864	0.105906795	-0.142444722	0.88672874	0.94399288	0.015085864
ENSMUSG00000033083	Tbc1d4	210789	protein_coding	365.9932362	0.015131838	0.098529916	-0.153576077	0.877944001	0.939378465	0.015131838
ENSMUSG00000024844	Banf1	23825	protein_coding	586.3792076	-0.015141907	0.092258262	0.164125216	0.869632572	0.935638301	0.015141907
ENSMUSG00000054648	Zfp869	66869	protein_coding	385.935471	0.01514961	0.071991935	-0.210434818	0.83332832	0.91603434	0.01514961

ensembl_gene_id	external_gene_name	entrezgene_id	gene_biotype	baseMean	log2FoldChange	lfcSE	stat	pvalue	padj	absLog2FC
ENSMUSG00000092305	Prps1l1	75456	protein_coding	2.055605934	0.015155022	1.466906111	-0.010331283	0.991756975	1	0.015155022
ENSMUSG00000029617	Ccz1	231874	protein_coding	770.9742414	0.015159948	0.089328487	-0.16971012	0.865238115	0.932932188	0.015159948
ENSMUSG00000027365	Trpm7	58800	protein_coding	1008.154812	-0.015165258	0.086423878	0.17547532	0.860706162	0.931474719	0.015165258
ENSMUSG00000054523	Ms4a5	269063	protein_coding	5.535614438	-0.015166508	0.440895272	0.034399343	0.972558708	0.985703995	0.015166508
ENSMUSG00000025784	Clec3b	21922	protein_coding	1.293241095	-0.015179713	1.630025525	0.009312562	0.992569758	1	0.015179713
ENSMUSG00000064302	Clasp1	76707	protein_coding	563.8180019	0.015218581	0.079743313	-0.190844608	0.848647339	0.925637253	0.015218581
ENSMUSG00000087128	Gm12655	115489886	lncRNA	3.88615255	-0.015221604	0.658678118	0.023109322	0.98156307	1	0.015221604
ENSMUSG00000029097	Trmt44	78890	protein_coding	120.2870202	0.015258087	0.137018385	-0.111357954	0.911332501	0.956997404	0.015258087
ENSMUSG00000066839	Ecsit	26940	protein_coding	327.3597475	-0.015286207	0.097942215	0.15607373	0.875974905	0.938630585	0.015286207
ENSMUSG00000025283	Sat1	20229	protein_coding	2202.827664	-0.015318237	0.097553574	0.157023844	0.875226057	0.938415991	0.015318237
ENSMUSG00000005148	Klf5	12224	protein_coding	4320.015984	-0.015324527	0.048258285	0.317552249	0.7508246	0.875049502	0.015324527
ENSMUSG00000022234	Cct5	12465	protein_coding	2802.304816	0.015347276	0.089345161	-0.171775123	0.863614323	0.932314788	0.015347276
ENSMUSG00000028986	Klhl7	52323	protein_coding	220.0475109	0.015357832	0.131214383	-0.117043816	0.906825332	0.954353007	0.015357832
ENSMUSG00000042148	Cox10	70383	protein_coding	534.0203504	-0.015359128	0.073220532	0.20976532	0.833850841	0.916410806	0.015359128
ENSMUSG00000018378	Cuedc1	70393	protein_coding	69.36655545	0.015372014	0.164530233	-0.093429721	0.92556218	0.964363534	0.015372014
ENSMUSG00000054079	Utp18	217109	protein_coding	225.8077873	0.015376197	0.106695733	-0.14411258	0.885411571	0.943195324	0.015376197
ENSMUSG00000022150	Dab2	13132	protein_coding	7.416907053	0.015389577	0.795988958	-0.019333908	0.984574735	0.99157486	0.015389577
ENSMUSG00000038914	Dido1	23856	protein_coding	1143.004386	-0.015403445	0.069941211	0.220234183	0.825688776	0.91190529	0.015403445
ENSMUSG00000036918	Ttc7	225049	protein_coding	1233.867092	0.015457046	0.058994226	-0.262009475	0.793314137	0.897130018	0.015457046
ENSMUSG00000030397	Mark4	232944	protein_coding	257.6057516	-0.01549284	0.105302541	0.147126934	0.883031831	0.942185661	0.01549284
ENSMUSG00000026417	Pigr	18703	protein_coding	52021.40112	-0.015534351	0.115057928	0.135013303	0.892601356	0.947202684	0.015534351
ENSMUSG00000027276	Jag1	16449	protein_coding	327.2789961	0.015616142	0.10502873	-0.148684479	0.881802609	0.941920329	0.015616142
ENSMUSG00000042745	Id1	15901	protein_coding	3257.893242	-0.01564499	0.125834503	0.124329887	0.901054084	0.95132126	0.01564499
ENSMUSG00000024902	Mrpl11	66419	protein_coding	350.2820424	-0.015655312	0.070003031	0.223637635	0.823039277	0.910297568	0.015655312
ENSMUSG00000021711	Trappc13	66975	protein_coding	470.5956646	0.015675958	0.071900792	-0.218022046	0.827411934	0.913026208	0.015675958
ENSMUSG00000046079	Lrrc8d	231549	protein_coding	372.7656508	0.015697787	0.092455024	-0.16978836	0.865176581	0.932932188	0.015697787
ENSMUSG00000021377	Dek	110052	protein_coding	615.1975044	-0.015716336	0.147059272	0.10687076	0.914891511	0.958296749	0.015716336
ENSMUSG00000034673	Pbx2	18515	protein_coding	436.350144	-0.015733789	0.085047079	0.185000935	0.853228303	0.927990748	0.015733789
ENSMUSG00000038705	Gmeb2	229004	protein_coding	373.6077898	-0.01573431	0.068564225	0.2294828	0.818493687	0.908812719	0.01573431
ENSMUSG00000017718	Afmid	71562	protein_coding	69.168046	0.015741816	0.170769954	-0.092181414	0.926553905	0.964893154	0.015741816
ENSMUSG00000047260	Emc6	66048	protein_coding	501.5145056	0.015804618	0.113613772	-0.139108298	0.889364571	0.945659633	0.015804618
ENSMUSG00000046947	Adck2	57869	protein_coding	234.9679623	-0.015848172	0.086156162	0.183947054	0.854054991	0.928522799	0.015848172
ENSMUSG00000055817	Mta3	116871	protein_coding	396.3511064	0.015853673	0.061655239	-0.25713424	0.797075147	0.899227966	0.015853673
ENSMUSG00000019917	Septin10	103080	protein_coding	194.9070346	0.015875536	0.144033349	-0.11022125	0.91223391	0.957266079	0.015875536
ENSMUSG00000011658	Fuz	70300	protein_coding	68.18011315	-0.015893478	0.149390959	0.10638849	0.915274125	0.95833356	0.015893478
ENSMUSG00000042997	Nhlrc3	212114	protein_coding	847.2497336	-0.015936325	0.082619907	0.192887225	0.84704729	0.924713357	0.015936325
ENSMUSG00000032584	Mst1r	19882	protein_coding	849.2034151	-0.015939615	0.092827827	0.171711604	0.863664262	0.932314788	0.015939615
ENSMUSG00000039308	Ndst2	17423	protein_coding	252.697605	0.01594823	0.065715993	-0.242684146	0.80825009	0.905463808	0.01594823
ENSMUSG00000000530	Acvrl1	11482	protein_coding	74.4878905	0.015958066	0.169509624	-0.094142535	0.924995932	0.964050667	0.015958066

Supplementary Table 1

ensembl_gene_id	external_gene_name	entrezgene_id	gene_biotype	baseMean	log2FoldChange	lfcSE	stat	pvalue	padj	absLog2FC
ENSMUSG00000046070	Igfals	16005	protein_coding	21.33285162	0.015989234	0.263462459	-0.06068885	0.951607012	0.976016284	0.015989234
ENSMUSG00000038822	Hace1	209462	protein_coding	229.4684836	-0.015998138	0.07974938	0.200605166	0.841007318	0.92126689	0.015998138
ENSMUSG00000029127	Zbtb49	75079	protein_coding	76.97864907	0.016035188	0.119023299	-0.134723103	0.892830806	0.947205958	0.016035188
ENSMUSG00000037070	Rbmxl1	19656	protein_coding	127.2006671	-0.016068035	0.155981471	0.103012459	0.917953083	0.959738982	0.016068035
ENSMUSG00000001419	Mef2d	17261	protein_coding	734.676898	0.016068125	0.133954342	-0.119952252	0.904520972	0.952874415	0.016068125
ENSMUSG00000019774	Mtrf1l	108853	protein_coding	106.7648344	0.016076243	0.109187097	-0.147235741	0.882945951	0.942166544	0.016076243
ENSMUSG00000027905	Ddx20	53975	protein_coding	191.7496645	-0.016096504	0.082390185	0.1953692	0.845103927	0.923798341	0.016096504
ENSMUSG00000022340	Sybu	319613	protein_coding	123.8591174	0.016151706	0.189061708	-0.085430871	0.931918851	0.967478151	0.016151706
ENSMUSG00000030534	Vps33b	233405	protein_coding	232.5759556	-0.016195859	0.079764406	0.203046193	0.839098927	0.919794636	0.016195859
ENSMUSG00000030303	Far2	330450	protein_coding	148.3282458	-0.016215803	0.149992939	0.108110444	0.913908084	0.957979661	0.016215803
ENSMUSG00000033577	Myo6	17920	protein_coding	3929.935051	0.016218638	0.069797414	-0.232367326	0.816252725	0.908409285	0.016218638
ENSMUSG00000022353	Mtss1	211401	protein_coding	685.0649789	0.01623178	0.067408981	-0.240795511	0.809713609	0.905997055	0.01623178
ENSMUSG00000020134	Peli1	67245	protein_coding	658.0306252	-0.016258591	0.073786493	0.22034644	0.825601355	0.91190529	0.016258591
ENSMUSG00000024673	Ms4a1	12482	protein_coding	4.650119365	0.016265968	0.65330297	-0.024898048	0.980136285	0.989302036	0.016265968
ENSMUSG00000041130	Zfp598	213753	protein_coding	901.933276	0.016305221	0.079363855	-0.205448954	0.837221376	0.91884587	0.016305221
ENSMUSG00000032349	Elovl5	68801	protein_coding	593.1708763	-0.016305238	0.123050449	0.132508562	0.894582051	0.947906833	0.016305238
ENSMUSG00000022801	Lrch3	70144	protein_coding	269.49943	-0.016309124	0.08826239	0.18477999	0.853401603	0.92805494	0.016309124
ENSMUSG00000040747	Cd53	12508	protein_coding	11.60564032	0.016344329	0.37751363	-0.043294673	0.965466637	0.982706396	0.016344329
ENSMUSG00000028975	Pex14	56273	protein_coding	311.2471487	-0.016366453	0.078329923	0.208942544	0.834493092	0.916826487	0.016366453
ENSMUSG00000026074	Map4k4	26921	protein_coding	253.0988704	0.016367473	0.093374244	-0.175288945	0.860852598	0.931532652	0.016367473
ENSMUSG00000066306	Numa1	101706	protein_coding	2651.612112	-0.016376356	0.078802688	0.20781469	0.835373662	0.917244724	0.016376356
ENSMUSG00000078234	Klhdc7a	242721	protein_coding	228.0811618	0.016376416	0.233308227	-0.070192191	0.94404069	0.973044134	0.016376416
ENSMUSG00000014956	Ppp1cb	19046	protein_coding	3474.045682	0.016377351	0.07220153	-0.226828307	0.820557251	0.9092783	0.016377351
ENSMUSG00000029275	Gfi1	14581	protein_coding	108.567098	0.016519843	0.190589083	-0.086677804	0.93092762	0.966815843	0.016519843
ENSMUSG00000044475	Ascc1	69090	protein_coding	234.8795043	-0.016538155	0.132272147	0.125031273	0.900498793	0.951064023	0.016538155
ENSMUSG00000024028	Tff2	21785	protein_coding	6.892245893	0.016538551	0.618339691	-0.026746708	0.978661759	0.988831409	0.016538551
ENSMUSG00000025050	Pcgf6	71041	protein_coding	110.6307327	0.016549263	0.157973223	-0.104759922	0.916566313	0.959274295	0.016549263
ENSMUSG00000025041	Nt5c2	76952	protein_coding	302.3856909	0.016571372	0.099092559	-0.167231249	0.867188098	0.933981885	0.016571372
ENSMUSG00000030127	Cops7a	26894	protein_coding	886.8610149	-0.01661746	0.073360666	0.226517301	0.820799105	0.909404657	0.01661746
ENSMUSG00000045691	Thtpa	105663	protein_coding	92.86672598	0.016628122	0.124197443	-0.133884576	0.893493847	0.947544143	0.016628122
ENSMUSG00000018858	Mrpl58	68572	protein_coding	345.4457971	-0.016651966	0.101387829	0.164240284	0.86954199	0.935632034	0.016651966
ENSMUSG00000009073	Nf2	18016	protein_coding	1370.0355	0.016671686	0.050009549	-0.333370049	0.738854969	0.867566194	0.016671686
ENSMUSG00000040407	Akap9	100986	protein_coding	3017.535284	-0.016739074	0.093325121	0.179363003	0.85765268	0.930374388	0.016739074
ENSMUSG00000028967	Errfi1	74155	protein_coding	1778.21864	-0.01675059	0.202943284	0.082538281	0.934218679	0.968832408	0.01675059
ENSMUSG00000079509	Zfx	22764	protein_coding	512.3693457	0.016752962	0.084770079	-0.197628248	0.843335932	0.922491196	0.016752962
ENSMUSG00000041278	Ttc1	66827	protein_coding	294.5769086	-0.016761612	0.072044769	0.232655504	0.816028923	0.908409285	0.016761612
ENSMUSG00000074886	Grk6	26385	protein_coding	1027.134303	-0.016868766	0.095448596	0.176731425	0.859719356	0.931236324	0.016868766
ENSMUSG00000077677	Gm24468	115490018	snRNA	0.775551077	-0.016884576	1.137347828	0.01484557	0.988155384	1	0.016884576
ENSMUSG00000022090	Pdlim2	213019	protein_coding	339.6611115	0.016902929	0.277015483	-0.061017993	0.951344879	0.975912221	0.016902929

Supplementary Table 1

ensembl_gene_id	external_gene_name	entrezgene_id	gene_biotype	baseMean	log2FoldChange	lfcSE	stat	pvalue	padj	absLog2FC
ENSMUSG00000036275	9530068E07Rik	213673	protein_coding	1130.018705	-0.016948443	0.073369458	0.231001338	0.817313762	0.908714429	0.016948443
ENSMUSG00000024560	Cxxc1	74322	protein_coding	524.1868969	-0.016992625	0.058215614	0.291891197	0.770369814	0.884816185	0.016992625
ENSMUSG00000041771	Slc24a4	238384	protein_coding	2.001657045	-0.016996014	2.339767042	0.007263977	0.994204236	1	0.016996014
ENSMUSG00000022961	Son	20658	protein_coding	3920.002183	0.017121281	0.056630915	-0.30233099	0.762399756	0.88093126	0.017121281
ENSMUSG00000111375	Btbd8	100503185	protein_coding	114.6337604	0.017168504	0.125989292	-0.136269548	0.891608196	0.947050968	0.017168504
ENSMUSG00000039988	Ankrd13c	433667	protein_coding	990.4967372	-0.017232772	0.073251394	0.235255209	0.814010658	0.907710402	0.017232772
ENSMUSG00000071014	Ndufb6	230075	protein_coding	513.9447013	0.017300259	0.105648525	-0.163752965	0.86992562	0.935658236	0.017300259
ENSMUSG00000031217	Efnb1	13641	protein_coding	1924.033483	0.017311088	0.095825152	-0.180652866	0.856640058	0.930204755	0.017311088
ENSMUSG00000035770	Dync1li2	234663	protein_coding	1231.386543	0.017335951	0.082665674	-0.209711603	0.833892769	0.916410806	0.017335951
ENSMUSG00000012114	Med15	94112	protein_coding	569.9550158	0.01735429	0.096992004	-0.178924961	0.857996623	0.930446023	0.01735429
ENSMUSG00000074746	Pdzd8	107368	protein_coding	1115.291601	-0.017357557	0.066419461	0.261332391	0.79383619	0.897263004	0.017357557
ENSMUSG00000031521	Aga	11593	protein_coding	256.9650657	-0.017371971	0.115393187	0.150545902	0.880333939	0.941010727	0.017371971
ENSMUSG00000080797	Gm15760	100042948	transcribed_processed_pseudogene	14.57319614	0.017403314	0.325435838	-0.053476945	0.9573519	0.978989985	0.017403314
ENSMUSG00000021951	Eef1akmt1	68043	protein_coding	149.7932179	-0.017421873	0.106832335	0.16307678	0.870457982	0.935980063	0.017421873
ENSMUSG00000021301	Hecw1	94253	protein_coding	4.166704329	0.01744059	1.872543386	-0.009313851	0.992568729	1	0.01744059
ENSMUSG00000020257	Wdr82	77305	protein_coding	978.8072758	-0.017467607	0.060091331	0.2906843	0.77129278	0.885199039	0.017467607
ENSMUSG00000022761	Lztr1	66863	protein_coding	697.2844069	-0.017469807	0.053220574	0.328252889	0.742720461	0.870063538	0.017469807
ENSMUSG00000016253	Nelfcd	57314	protein_coding	510.594096	0.017497389	0.066867809	-0.261671338	0.79357484	0.897240511	0.017497389
ENSMUSG00000009563	Tor2a	30933	protein_coding	409.6422281	0.017519695	0.085403924	-0.20513923	0.837463347	0.918959391	0.017519695
ENSMUSG00000053286	Trmt1l	98685	protein_coding	387.8784329	-0.017523958	0.058251197	0.300834307	0.763540846	0.881386379	0.017523958
ENSMUSG00000097145	9230114K14Rik	414108	lncRNA	11.20332696	-0.017538295	0.345842543	0.050711793	0.959555179	0.980447529	0.017538295
ENSMUSG00000019809	Pex3	56535	protein_coding	179.8465295	0.017542137	0.116719056	-0.150293687	0.880532914	0.941067509	0.017542137
ENSMUSG00000027242	Wdr76	241627	protein_coding	132.7376792	-0.017547219	0.145002383	0.121013316	0.903680488	0.952353515	0.017547219
ENSMUSG00000029038	Ssu72	68991	protein_coding	673.8437288	-0.017556003	0.062304543	0.281777253	0.778114314	0.889135053	0.017556003
ENSMUSG00000028522	Mier1	71148	protein_coding	1673.265418	-0.017564331	0.114111175	0.153922968	0.877670474	0.93927028	0.017564331
ENSMUSG00000047731	Wbp1l	226178	protein_coding	912.2921443	0.017657055	0.05747504	-0.307212576	0.758681574	0.878912418	0.017657055
ENSMUSG00000022221	Ripk3	56532	protein_coding	218.1419131	0.017678988	0.134576998	-0.131367089	0.895484922	0.948680909	0.017678988
ENSMUSG00000041957	Pkp2	67451	protein_coding	745.3446082	-0.017693643	0.066099772	0.267680843	0.788944997	0.894394019	0.017693643
ENSMUSG00000068917	Clk2	12748	protein_coding	272.9013151	-0.017700546	0.088671553	0.199619213	0.8417784	0.921642147	0.017700546
ENSMUSG00000034663	Bmp2k	140780	protein_coding	315.4500851	-0.017712003	0.094511993	0.187404817	0.851343246	0.92744128	0.017712003
ENSMUSG00000028496	Mllt3	70122	protein_coding	558.007186	0.017717716	0.102643432	-0.172614226	0.862954669	0.932166328	0.017717716
ENSMUSG00000032469	Dbr1	83703	protein_coding	162.8266324	0.017767619	0.113070554	-0.157137454	0.87513652	0.938415991	0.017767619
ENSMUSG00000039568	Ubald1	207740	protein_coding	335.7443364	-0.017857415	0.132402596	0.134872091	0.892713006	0.947202684	0.017857415
ENSMUSG00000111063	Zfp660	666257	lncRNA	1.777368946	-0.017858784	1.928408944	0.00926089	0.992610984	1	0.017858784
ENSMUSG00000027593	Raly	19383	protein_coding	1427.99255	0.017881542	0.063210354	-0.282889453	0.77726158	0.888775635	0.017881542
ENSMUSG00000028954	Nub1	53312	protein_coding	479.6949741	0.01788478	0.059079067	-0.302726175	0.762098549	0.880874346	0.01788478
ENSMUSG00000020794	Ube2g1	67128	protein_coding	497.6815876	0.01788768	0.057683536	-0.31010027	0.756484707	0.877843331	0.01788768
ENSMUSG00000071266	Zfp946	74149	protein_coding	252.0376236	-0.017935942	0.093980738	0.190846995	0.848645468	0.925637253	0.017935942
ENSMUSG00000070934	Rraga	68441	protein_coding	336.2071035	0.01794139	0.0949977	-0.18886131	0.850201518	0.926816617	0.01794139

ensembl_gene_id	external_gene_name	entrezgene_id	gene_biotype	baseMean	log2FoldChange	lfcSE	stat	pvalue	padj	absLog2FC
ENSMUSG00000013622	Atraid	381629	protein_coding	561.7100797	-0.017944344	0.087898416	0.204148667	0.838237324	0.919410944	0.017944344
ENSMUSG00000037151	Lrrc20	216011	protein_coding	113.9561715	0.018065019	0.127025042	-0.142216201	0.886909235	0.94399288	0.018065019
ENSMUSG00000027177	Hipk3	15259	protein_coding	1237.763669	-0.018085311	0.069559145	0.259999035	0.794864518	0.897960915	0.018085311
ENSMUSG00000040331	Nsmce4a	67872	protein_coding	522.3059995	0.018089231	0.088090222	-0.205348913	0.837299531	0.918870578	0.018089231
ENSMUSG00000022129	Dct	13190	protein_coding	10.27179372	0.018116298	0.43888025	-0.041278453	0.967073911	0.983632579	0.018116298
ENSMUSG00000030469	Zfp719	210105	protein_coding	122.687168	-0.018121257	0.129945788	0.139452442	0.889092634	0.945487524	0.018121257
ENSMUSG00000078920	Ifi47	15953	protein_coding	87.03949495	-0.018129222	0.292102778	0.06206453	0.950511444	0.975869522	0.018129222
ENSMUSG00000039208	Metrnl	210029	protein_coding	848.5904282	0.018171448	0.138589846	-0.131116731	0.895682966	0.948731562	0.018171448
ENSMUSG00000062248	Cks2	66197	protein_coding	276.9574786	-0.018177314	0.157354051	0.115518563	0.908034108	0.955211953	0.018177314
ENSMUSG00000029233	Srd5a3	57357	protein_coding	170.3143399	-0.018178256	0.104186503	0.174478031	0.861489795	0.931722347	0.018178256
ENSMUSG00000038733	Wdr26	226757	protein_coding	1400.837794	0.018179134	0.050836323	-0.357601281	0.72064172	0.855240917	0.018179134
ENSMUSG00000106380	Gm3519	100041805	lncRNA	1.83741441	0.018195364	0.954506589	-0.019062586	0.984791178	1	0.018195364
ENSMUSG00000028069	Gpatch4	66614	protein_coding	119.4556532	0.018211593	0.143926728	-0.126533783	0.899309409	0.950598193	0.018211593
ENSMUSG00000038533	Cbfa2t2	12396	protein_coding	337.472033	-0.018215001	0.059580215	0.305722312	0.759816084	0.879422681	0.018215001
ENSMUSG00000020253	Ppm1m	67905	protein_coding	48.4403481	-0.018310488	0.161290734	0.113524738	0.909614552	0.956044724	0.018310488
ENSMUSG00000022300	Dcaf13	223499	protein_coding	485.3927743	0.018367011	0.076107501	-0.241329837	0.809299487	0.905779176	0.018367011
ENSMUSG00000055761	Nkain3	269513	protein_coding	1.948487245	-0.018392149	2.323870799	0.007914445	0.993685252	1	0.018392149
ENSMUSG00000030530	Furin	18550	protein_coding	715.3213755	0.018410527	0.082044644	-0.224396457	0.822448827	0.910171686	0.018410527
ENSMUSG00000114469	C730002L08Rik	320155	lncRNA	4.338382363	0.018421344	0.672139094	-0.027407042	0.978135082	1	0.018421344
ENSMUSG00000082101	Slfn14	237890	protein_coding	0.862133039	-0.018422881	1.726028038	0.01067357	0.991483885	1	0.018422881
ENSMUSG00000001785	Pwp1	103136	protein_coding	319.7897648	0.018433123	0.104848134	-0.175807828	0.86044492	0.931471303	0.018433123
ENSMUSG00000045287	Rtn4rl1	237847	protein_coding	69.73973205	-0.018435402	0.138659022	0.132954941	0.894229016	0.947795386	0.018435402
ENSMUSG00000019471	Cdc37	12539	protein_coding	1213.040388	0.01844402	0.068270385	-0.270161364	0.787036115	0.893375436	0.01844402
ENSMUSG00000027430	Dtd1	66044	protein_coding	105.5882166	0.018446517	0.113476297	-0.16255833	0.870866199	0.936355518	0.018446517
ENSMUSG00000027303	Ptpra	19262	protein_coding	516.2957257	0.018453983	0.061993694	-0.297675163	0.765951102	0.882687823	0.018453983
ENSMUSG00000033318	Gstt2	14872	protein_coding	439.13518	0.018488171	0.072028806	-0.256677457	0.797427775	0.899441451	0.018488171
ENSMUSG00000048047	Zbtb33	56805	protein_coding	310.062824	-0.018521603	0.088964991	0.208189787	0.835080783	0.917066002	0.018521603
ENSMUSG00000038555	Reep2	225362	protein_coding	3.84141833	0.018543014	0.768045725	-0.024143112	0.980738455	1	0.018543014
ENSMUSG00000025133	Ints4	101861	protein_coding	480.1599165	-0.018591758	0.090914403	0.204497386	0.837964835	0.919173117	0.018591758
ENSMUSG00000028540	Dph2	67728	protein_coding	93.69296818	0.018604741	0.131772884	-0.141187931	0.88772148	0.94451598	0.018604741
ENSMUSG00000038203	Hoxa13	15398	protein_coding	470.6927732	-0.018610167	0.130792439	0.14228779	0.88685269	0.94399288	0.018610167
ENSMUSG00000038174	Hycc2	213056	protein_coding	380.6835199	0.01862464	0.108052117	-0.172367192	0.863148863	0.932166328	0.01862464
ENSMUSG00000047379	B4gat1	108902	protein_coding	2.800389224	0.018710865	0.642772102	-0.029109641	0.976777146	1	0.018710865
ENSMUSG00000101856	1700096K18Rik	73571	lncRNA	42.2189779	-0.018775455	0.190131269	0.098749961	0.9213368	0.962004133	0.018775455
ENSMUSG00000098280	Mir7687	102466849	miRNA	1.854227492	-0.018782232	0.918562092	0.020447427	0.98368645	1	0.018782232
ENSMUSG00000033228	Scaf11	72193	protein_coding	1327.854413	-0.018820556	0.072039952	0.261251648	0.793898451	0.897263004	0.018820556
ENSMUSG00000045438	Cox19	68033	protein_coding	282.1175846	-0.018843576	0.108747442	0.173278341	0.862432647	0.932036168	0.018843576
ENSMUSG00000020171	Yeats4	64050	protein_coding	296.2940537	0.018865777	0.136877281	-0.137829866	0.89037488	0.946160016	0.018865777
ENSMUSG00000021958	Pinx1	72400	protein_coding	73.40019041	0.018871174	0.190156185	-0.099240389	0.920947407	0.961855464	0.018871174

ensembl_gene_id	external_gene_name	entrezgene_id	gene_biotype	baseMean	log2FoldChange	lfcSE	stat	pvalue	padj	absLog2FC
ENSMUSG00000038022	Mindy4	330323	protein_coding	11.13512738	0.018886975	0.453873646	-0.041612849	0.96680733	0.983482583	0.018886975
ENSMUSG00000049191	Rtl5	331474	protein_coding	5.375238463	0.018932825	0.621517771	-0.030462243	0.975698405	0.987300339	0.018932825
ENSMUSG00000074063	Osgin1	71839	protein_coding	159.7224408	-0.018961722	0.183952979	0.10307918	0.917900129	0.959738982	0.018961722
ENSMUSG00000042854	Trp53rkb	76367	protein_coding	34.08736295	0.018993881	0.191583312	-0.099141624	0.921025824	0.961855464	0.018993881
ENSMUSG00000027162	Lin7c	22343	protein_coding	2719.402178	-0.018996194	0.096112441	0.197645525	0.843322414	0.922491196	0.018996194
ENSMUSG00000054612	Mgmt	17314	protein_coding	34.00882706	-0.018997988	0.222525201	0.085374544	0.93196363	0.967478151	0.018997988
ENSMUSG00000029291	Rufy3	52822	protein_coding	301.1981025	-0.019003437	0.075352486	0.252193901	0.800891192	0.901439282	0.019003437
ENSMUSG00000053565	Eif3k	73830	protein_coding	1326.460092	0.019010282	0.077577478	-0.245048986	0.806418502	0.904795436	0.019010282
ENSMUSG00000087968	Gm25395	115485773	scaRNA	535.0798835	0.019094175	0.261327756	-0.073066004	0.941753594	0.971883559	0.019094175
ENSMUSG00000026175	Vil1	22349	protein_coding	9369.642966	0.019108408	0.081984623	-0.233073068	0.815704667	0.908392129	0.019108408
ENSMUSG00000028121	Bcar3	29815	protein_coding	889.8409529	-0.019111428	0.109761081	0.174118436	0.861772385	0.931842966	0.019111428
ENSMUSG00000025369	Smarcc2	68094	protein_coding	1047.480611	-0.019209235	0.070812914	0.271267404	0.786185374	0.893161222	0.019209235
ENSMUSG00000024169	Ift140	106633	protein_coding	266.4201372	-0.019230488	0.078762987	0.244156407	0.807109687	0.904987729	0.019230488
ENSMUSG00000047466	8030462N17Rik	212163	protein_coding	332.0022407	-0.019247588	0.08979248	0.214356343	0.830269184	0.914187552	0.019247588
ENSMUSG00000027455	Nsfl1c	386649	protein_coding	515.4017401	0.019250321	0.060737556	-0.316942632	0.751287133	0.875081807	0.019250321
ENSMUSG00000007617	Homer1	26556	protein_coding	379.7080892	0.019252799	0.089018203	-0.21627935	0.828770005	0.913504709	0.019252799
ENSMUSG00000026917	Wdr5	140858	protein_coding	454.1855312	-0.019274755	0.096921513	0.198869724	0.842364653	0.921917283	0.019274755
ENSMUSG00000038485	Socs7	192157	protein_coding	323.9704978	-0.019278244	0.101980336	0.18903884	0.850062376	0.926816617	0.019278244
ENSMUSG00000003444	Med29	67224	protein_coding	88.61864346	0.019285778	0.131268891	-0.146918114	0.883196654	0.942211125	0.019285778
ENSMUSG00000028458	Tesk1	21754	protein_coding	297.8545401	-0.019287735	0.098642596	0.195531506	0.844976875	0.923798341	0.019287735
ENSMUSG00000041126	H2az2	77605	protein_coding	515.6106798	-0.019302725	0.094373018	0.204536482	0.837934286	0.919173117	0.019302725
ENSMUSG00000025404	R3hdm2	71750	protein_coding	340.9762829	-0.019337212	0.138224435	0.139897207	0.888741208	0.945235545	0.019337212
ENSMUSG00000022552	Sharpin	106025	protein_coding	404.9786408	-0.019346215	0.079961029	0.241945552	0.808822353	0.905678041	0.019346215
ENSMUSG00000097471	5830432E09Rik	67765	lncRNA	6.979710673	-0.01936973	0.490058897	0.039525309	0.968471575	0.984122985	0.01936973
ENSMUSG00000025809	Itgb1	16412	protein_coding	2859.043315	0.019451086	0.064461803	-0.301745928	0.762845753	0.881056435	0.019451086
ENSMUSG00000030738	Eif3c	56347	protein_coding	3331.404726	-0.019489041	0.061348655	0.317676741	0.750730156	0.87503937	0.019489041
ENSMUSG00000031153	Gripap1	54645	protein_coding	348.3881312	0.019510153	0.110166418	-0.177097101	0.859432119	0.931154836	0.019510153
ENSMUSG00000039449	Prpf18	67229	protein_coding	470.9013564	0.019513658	0.088810763	-0.219721773	0.826087847	0.912143267	0.019513658
ENSMUSG00000039244	Ints15	231868	protein_coding	201.4709446	0.01951705	0.083534705	-0.233640023	0.815264453	0.908146859	0.01951705
ENSMUSG00000027937	Jtb	23922	protein_coding	474.1992707	0.019543598	0.108995981	-0.179305674	0.857697692	0.930374388	0.019543598
ENSMUSG00000029089	5730480H06Rik	66768	protein_coding	84.44769508	0.019543931	0.116816078	-0.167305145	0.867129956	0.933980178	0.019543931
ENSMUSG00000021906	Oxnad1	218885	protein_coding	323.6506954	-0.019579342	0.067038215	0.292062393	0.77023892	0.884771487	0.019579342
ENSMUSG00000029389	Ddx55	67848	protein_coding	161.5454734	-0.019600975	0.105278265	0.186182539	0.852301616	0.927761944	0.019600975
ENSMUSG00000022401	Xpnpep3	321003	protein_coding	184.1290557	0.01960946	0.112426904	-0.174419639	0.861535681	0.931722347	0.01960946
ENSMUSG00000021668	Polk	27015	protein_coding	152.870442	-0.019616253	0.158403531	0.123837222	0.90144416	0.951341404	0.019616253
ENSMUSG00000078486	Perm1	74183	protein_coding	17.06738782	0.019636243	0.374141709	-0.052483438	0.958143492	0.979508488	0.019636243
ENSMUSG00000059423	Zfp933	242747	protein_coding	99.20090171	-0.019665093	0.113872746	0.172693588	0.862892284	0.932166328	0.019665093
ENSMUSG00000040548	Tex2	21763	protein_coding	678.4670315	-0.019691637	0.065410508	0.301047001	0.763378654	0.881383982	0.019691637
ENSMUSG00000001441	Npepps	19155	protein_coding	858.043543	0.019720117	0.048637892	-0.405447614	0.685148518	0.832151567	0.019720117

ensembl_gene_id	external_gene_name	entrezgene_id	gene_biotype	baseMean	log2FoldChange	lfcSE	stat	pvalue	padj	absLog2FC
ENSMUSG00000049493	Pls1	102502	protein_coding	3199.590583	-0.019785213	0.087085864	0.227192013	0.820274438	0.9092783	0.019785213
ENSMUSG00000029417	Cxcl9	17329	protein_coding	2.534476602	0.019806434	0.819913495	-0.024156737	0.980727587	1	0.019806434
ENSMUSG00000030057	Cnbp	12785	protein_coding	2209.837835	0.019814758	0.10221298	-0.193857552	0.846287422	0.924222269	0.019814758
ENSMUSG00000050244	Heatr1	217995	protein_coding	475.2564054	0.019830254	0.147404024	-0.134529936	0.892983541	0.947307136	0.019830254
ENSMUSG00000071180	Smim15	75616	protein_coding	859.6706893	-0.019834591	0.067540818	0.293668204	0.769011453	0.884245488	0.019834591
ENSMUSG00000042744	Hectd4	269700	protein_coding	1265.933277	0.019860354	0.157102629	-0.126416431	0.899402296	0.950621521	0.019860354
ENSMUSG00000002052	Supt6	20926	protein_coding	1181.586085	0.019861008	0.041552381	-0.477975207	0.632667852	0.799623213	0.019861008
ENSMUSG00000029152	Ociad1	68095	protein_coding	1121.666161	0.019960478	0.082566331	-0.241750812	0.808973255	0.905720972	0.019960478
ENSMUSG00000026587	Astn1	11899	protein_coding	4.858387377	0.019960686	1.34357495	-0.014856399	0.988146744	0.993418099	0.019960686
ENSMUSG00000029004	Kmt2e	69188	protein_coding	514.3547522	-0.019967125	0.157075839	0.127117738	0.898847212	0.950352959	0.019967125
ENSMUSG00000046032	Snx12	55988	protein_coding	287.850514	-0.01998145	0.084337926	0.236921292	0.812717852	0.907147741	0.01998145
ENSMUSG00000024941	Scyl1	78891	protein_coding	763.9354563	0.019998772	0.064846909	-0.308399766	0.75777816	0.87863969	0.019998772
ENSMUSG00000074384	AI429214	621080	protein_coding	6.465123833	0.019998973	0.440886514	-0.045360818	0.963819711	0.982137401	0.019998973
ENSMUSG00000038664	Herc1	235439	protein_coding	1384.702428	-0.020000051	0.070831002	0.282362952	0.77766522	0.888933284	0.020000051
ENSMUSG00000036733	Rbm42	68035	protein_coding	1.443338995	-0.020004843	1.058485255	0.018899501	0.984921278	1	0.020004843
ENSMUSG00000039230	Tbcd	108903	protein_coding	855.8850502	0.020052876	0.086932555	-0.230671648	0.817569901	0.908714429	0.020052876
ENSMUSG00000018470	Kcnab3	16499	protein_coding	6.811425686	-0.020054486	0.618093902	0.032445695	0.974116622	0.986602999	0.020054486
ENSMUSG00000028468	Rgp1	242406	protein_coding	875.9009975	-0.020090829	0.08714472	0.230545567	0.81766786	0.908714429	0.020090829
ENSMUSG00000112489	9230116L04Rik	321017	lncRNA	57.50768725	-0.020098286	0.186357722	0.107847883	0.914116359	0.958030863	0.020098286
ENSMUSG00000031429	Psmd10	53380	protein_coding	242.3725844	0.020103837	0.085594898	-0.23487191	0.814308152	0.907710402	0.020103837
ENSMUSG00000032567	Aste1	66595	protein_coding	158.0581095	0.020115746	0.087060682	-0.231054316	0.817272606	0.908714429	0.020115746
ENSMUSG00000034868	Myl12b	67938	protein_coding	462.4443232	-0.020125991	0.111236483	0.180929768	0.856422703	0.930184975	0.020125991
ENSMUSG00000021241	Isca2	74316	protein_coding	185.5759542	-0.020141769	0.087350008	0.230586916	0.817635734	0.908714429	0.020141769
ENSMUSG00000021983	Atp8a2	50769	protein_coding	523.5551485	0.020191664	0.07184066	-0.281061788	0.778663009	0.889454308	0.020191664
ENSMUSG00000015533	Itga2	16398	protein_coding	1043.140288	0.020216641	0.128151059	-0.157756331	0.874648811	0.938406345	0.020216641
ENSMUSG00000065637	Gm26397	115487043	snoRNA	246.6811701	-0.020220335	0.25773885	0.078452801	0.937467874	0.970191419	0.020220335
ENSMUSG00000026213	Stk11ip	71728	protein_coding	258.8874745	-0.020220892	0.075706609	0.267095462	0.789395662	0.894720656	0.020220892
ENSMUSG00000046613	Vwa5b2	328643	protein_coding	21.69914907	-0.020222688	0.315417783	0.064113975	0.948879474	0.975399666	0.020222688
ENSMUSG00000021771	Vdac2	22334	protein_coding	4853.067908	-0.02025008	0.077013559	0.262941749	0.792595477	0.896761253	0.02025008
ENSMUSG00000057982	Zfp809	235047	protein_coding	223.5133783	-0.020292289	0.103804257	0.195486095	0.845012422	0.923798341	0.020292289
ENSMUSG00000034928	Rnf44	105239	protein_coding	1032.101311	-0.020354283	0.085795821	0.237240958	0.812469864	0.907147741	0.020354283
ENSMUSG00000034518	Hmgxb4	70823	protein_coding	261.4082692	-0.020366307	0.092118801	0.221087406	0.825024376	0.911456552	0.020366307
ENSMUSG00000059689	Zfp637	232337	protein_coding	136.0000682	-0.020399088	0.095092401	0.214518594	0.830142669	0.914175515	0.020399088
ENSMUSG00000027131	Emc4	68032	protein_coding	292.5877738	-0.020404139	0.071931184	0.283661936	0.776669467	0.888506311	0.020404139
ENSMUSG00000020220	Vps13d	230895	protein_coding	3055.842085	0.020404811	0.107967651	-0.18899004	0.850100623	0.926816617	0.020404811
ENSMUSG00000003226	Ranbp2	19386	protein_coding	2322.805956	0.020411815	0.084755328	-0.240832231	0.809685148	0.905997055	0.020411815
ENSMUSG00000048930	Tada3	101206	protein_coding	297.5306447	0.020424733	0.079599374	-0.256594138	0.797492101	0.899452571	0.020424733
ENSMUSG00000005102	Eif2ak4	27103	protein_coding	356.2623977	-0.02043919	0.097073375	0.210554022	0.833235293	0.915993052	0.02043919
ENSMUSG00000005958	Ephb3	13845	protein_coding	356.170077	-0.020439559	0.134588587	0.151866953	0.879291874	0.940455052	0.020439559

ensembl_gene_id	external_gene_name	entrezgene_id	gene_biotype	baseMean	log2FoldChange	lfcSE	stat	pvalue	padj	absLog2FC
ENSMUSG00000018287	Spag7	216873	protein_coding	408.6789057	0.02044203	0.090784649	-0.225170561	0.82184659	0.91001573	0.02044203
ENSMUSG00000020166	Cnot2	72068	protein_coding	634.9309497	0.020475391	0.075226339	-0.272183809	0.78548069	0.892918574	0.020475391
ENSMUSG00000059273	Zc3h4	330474	protein_coding	812.7433577	0.020502672	0.098429016	-0.208299061	0.834995465	0.917066002	0.020502672
ENSMUSG00000078179	Rnf148	71300	protein_coding	2.943734535	0.020507883	0.645992793	-0.031746303	0.974674369	1	0.020507883
ENSMUSG00000040586	Ofd1	237222	protein_coding	116.0143619	-0.020518126	0.134850745	0.152154341	0.879065205	0.940340033	0.020518126
ENSMUSG00000035772	Mrps2	118451	protein_coding	348.6948722	0.02052324	0.101099195	-0.203001025	0.839134231	0.919794636	0.02052324
ENSMUSG00000085437	1700047A11Rik	76604	lncRNA	1.672788782	0.020560349	1.050193685	-0.019577673	0.984380275	1	0.020560349
ENSMUSG00000039000	Ube3c	100763	protein_coding	1123.302214	0.02057508	0.07003412	-0.293786518	0.768921038	0.884245488	0.02057508
ENSMUSG00000035614	Togaram1	328108	protein_coding	505.279921	-0.020675644	0.082563305	0.250421709	0.802261243	0.90234117	0.020675644
ENSMUSG00000037204	Atg101	68118	protein_coding	408.4332117	-0.020677745	0.080177219	0.257900503	0.796483697	0.898891871	0.020677745
ENSMUSG00000040481	Bptf	207165	protein_coding	1069.330002	0.020706206	0.074872707	-0.276552122	0.782124033	0.890758364	0.020706206
ENSMUSG00000028920	Fbxo42	213499	protein_coding	414.8071715	0.0207134	0.057693393	-0.359025509	0.719576006	0.854899358	0.0207134
ENSMUSG00000054309	Cpsf3	54451	protein_coding	574.331252	0.02073004	0.094669916	-0.218971778	0.826672032	0.912523774	0.02073004
ENSMUSG00000022760	Thap7	69009	protein_coding	163.7189817	0.020734925	0.092082094	-0.225178688	0.821840269	0.91001573	0.020734925
ENSMUSG00000035104	Eva1a	232146	protein_coding	27.65313407	-0.02076387	0.233570966	0.08889748	0.929163386	0.966128972	0.02076387
ENSMUSG00000027938	Creb3l4	78284	protein_coding	454.1285521	-0.020801755	0.237240569	0.087682115	0.930129335	0.966352899	0.020801755
ENSMUSG00000010025	Aldh3a2	11671	protein_coding	1656.789148	0.020830015	0.074645373	-0.279053001	0.780204151	0.890125936	0.020830015
ENSMUSG00000105387	Gm29681	545809	lncRNA	0.877474797	-0.020845445	1.731284462	0.01204045	0.990393343	1	0.020845445
ENSMUSG00000034579	Pla2g3	237625	protein_coding	231.8522878	-0.020854421	0.160078799	0.130275974	0.896348088	0.948996349	0.020854421
ENSMUSG00000018796	Acsl1	14081	protein_coding	293.5978578	0.020936564	0.179648219	-0.11654201	0.907222994	0.95457824	0.020936564
ENSMUSG00000051185	Fam174a	67698	protein_coding	153.7962866	-0.020940173	0.114680426	0.182595878	0.855115119	0.929465614	0.020940173
ENSMUSG00000036257	Pnpla8	67452	protein_coding	882.6644761	-0.020956132	0.107293959	0.195315121	0.84514626	0.923798341	0.020956132
ENSMUSG00000039804	Ncoa5	228869	protein_coding	339.9128393	0.020961241	0.082964371	-0.252653533	0.800535959	0.901189824	0.020961241
ENSMUSG00000025870	Arl10	56795	protein_coding	138.7139689	0.021029462	0.116863263	-0.179949295	0.857192375	0.930374388	0.021029462
ENSMUSG00000060240	Cend1	57754	protein_coding	3.469343665	0.021061847	0.694291464	-0.030335743	0.975799291	1	0.021061847
ENSMUSG00000074582	Arfgef2	99371	protein_coding	1319.996218	0.02106218	0.052151527	-0.403865068	0.686311945	0.832935655	0.02106218
ENSMUSG00000020340	Cyfip2	76884	protein_coding	17.3379801	-0.021073963	0.309311642	0.068131812	0.945680707	0.973795719	0.021073963
ENSMUSG00000024668	Sdhaf2	66072	protein_coding	426.0073436	0.021074307	0.071547334	-0.29455056	0.768337235	0.883975099	0.021074307
ENSMUSG00000005609	Ctr9	22083	protein_coding	829.6526447	-0.021169154	0.062127903	0.340735053	0.733303047	0.863385162	0.021169154
ENSMUSG00000040435	Ppp1r15a	17872	protein_coding	549.9063937	0.02127365	0.240309692	-0.088525975	0.92945864	0.966128972	0.02127365
ENSMUSG00000010047	Hyal2	15587	protein_coding	526.5150596	-0.021279212	0.102119748	0.208375094	0.834936102	0.917066002	0.021279212
ENSMUSG00000042364	Snx18	170625	protein_coding	215.2708519	-0.021315632	0.103429539	0.206088435	0.836721833	0.918419697	0.021315632
ENSMUSG00000036112	Metap2	56307	protein_coding	1437.723734	0.021326789	0.072509072	-0.294125803	0.768661774	0.884145751	0.021326789
ENSMUSG00000085876	Gm12409	100040567	lncRNA	3.129753829	-0.021335004	0.593024918	0.035976573	0.971301039	1	0.021335004
ENSMUSG00000021843	Ktn1	16709	protein_coding	746.4898419	-0.021453397	0.077432342	0.277059897	0.781734114	0.890621398	0.021453397
ENSMUSG00000036372	Tmem258	69038	protein_coding	575.7129316	0.021566016	0.112637165	-0.191464474	0.848161712	0.925501685	0.021566016
ENSMUSG00000027787	Nmd3	97112	protein_coding	217.9345312	0.021569559	0.093174881	-0.231495423	0.816929941	0.908700419	0.021569559
ENSMUSG00000035696	Rnf38	73469	protein_coding	413.2699619	0.021573641	0.072551636	-0.297355676	0.766194979	0.882690208	0.021573641
ENSMUSG00000030156	Cd69	12515	protein_coding	15.68593322	0.021606761	0.564021891	-0.038308373	0.969441815	0.984403993	0.021606761

ensembl_gene_id	external_gene_name	entrezgene_id	gene_biotype	baseMean	log2FoldChange	lfcSE	stat	pvalue	padj	absLog2FC
ENSMUSG00000037049	Smpd1	20597	protein_coding	345.1177398	-0.021641978	0.102475247	0.211192254	0.832737255	0.915689375	0.021641978
ENSMUSG00000044150	Bclaf3	382252	protein_coding	102.6755828	0.021652919	0.136155955	-0.159030275	0.873645022	0.937816815	0.021652919
ENSMUSG00000033739	Fkbpl	56299	protein_coding	65.7147216	0.021658001	0.144123268	-0.150274144	0.880548332	0.941067509	0.021658001
ENSMUSG00000093080	Mir3060	100526466	miRNA	1.047837915	-0.021662145	1.198550775	0.018073615	0.985580127	1	0.021662145
ENSMUSG00000057363	Uxs1	67883	protein_coding	212.5396505	-0.021678146	0.075427992	0.287401867	0.773804632	0.88672609	0.021678146
ENSMUSG00000025903	Lypla1	18777	protein_coding	959.5240635	0.021696983	0.079777444	-0.271968888	0.785645941	0.892925964	0.021696983
ENSMUSG00000038085	Cnbd2	70873	protein_coding	22.49029239	0.021736509	0.270302036	-0.080415631	0.935906696	0.96968875	0.021736509
ENSMUSG00000077744	Gm26343	115486206	snoRNA	0.617806996	-0.021756029	1.844020761	0.011798147	0.990586659	1	0.021756029
ENSMUSG00000057229	Dmac2	66349	protein_coding	527.4202969	-0.021792468	0.070755181	0.307998203	0.7580837	0.878750865	0.021792468
ENSMUSG00000027900	Dram2	67171	protein_coding	1628.64894	0.021806856	0.10279053	-0.212148494	0.831991188	0.915295615	0.021806856
ENSMUSG00000029066	Mrpl20	66448	protein_coding	447.6462885	0.021817882	0.071714787	-0.304231281	0.760951695	0.880144002	0.021817882
ENSMUSG00000036860	Mrpl55	67212	protein_coding	266.6085474	-0.021825514	0.093833487	0.232598353	0.816073306	0.908409285	0.021825514
ENSMUSG00000026516	Nvl	67459	protein_coding	398.6904197	-0.021827287	0.060690053	0.359651796	0.719107545	0.854587075	0.021827287
ENSMUSG00000005312	Ubqln1	56085	protein_coding	1651.432274	0.021852604	0.069779909	-0.313164697	0.754155538	0.876856731	0.021852604
ENSMUSG00000031379	Pir	69656	protein_coding	77.11770028	0.021854234	0.15439298	-0.141549401	0.887435937	0.944306685	0.021854234
ENSMUSG00000046562	Unc119b	106840	protein_coding	581.0162301	0.021889828	0.095918522	-0.228212734	0.819480862	0.908985828	0.021889828
ENSMUSG00000021772	Nkiras1	69721	protein_coding	92.83251593	0.021902842	0.121195134	-0.180723772	0.856584399	0.930204755	0.021902842
ENSMUSG00000098274	Rpl24	68193	protein_coding	144.2202308	0.022043859	0.151877513	-0.145142347	0.884598484	0.942955032	0.022043859
ENSMUSG00000023075	Akirin1	68050	protein_coding	667.7667488	-0.022083974	0.084788753	0.260458762	0.794509921	0.897715376	0.022083974
ENSMUSG00000043929	Klhl15	236904	protein_coding	124.2970006	0.022099092	0.126192061	-0.175122684	0.860983234	0.931580268	0.022099092
ENSMUSG00000060950	Trmt61a	328162	protein_coding	85.02265853	-0.022143405	0.195843935	0.113066584	0.909977767	0.95630475	0.022143405
ENSMUSG00000022035	Ccdc25	67179	protein_coding	525.2613602	-0.02216458	0.102687794	0.215844345	0.829109081	0.913524643	0.02216458
ENSMUSG00000046962	Zbtb21	114565	protein_coding	268.880694	-0.022179271	0.0781331	0.283865242	0.776513654	0.888506311	0.022179271
ENSMUSG00000021590	Spata9	75571	protein_coding	3.452038104	0.022180583	0.840476937	-0.026390472	0.978945894	1	0.022180583
ENSMUSG00000034105	Meak7	74347	protein_coding	253.0630914	-0.022199311	0.118653643	0.18709338	0.851587419	0.927499565	0.022199311
ENSMUSG00000032869	Psmf1	228769	protein_coding	316.2964239	0.022283897	0.066534058	-0.334924672	0.73768191	0.866866795	0.022283897
ENSMUSG00000021025	Nfkbia	18035	protein_coding	1365.832937	-0.022290147	0.23865999	0.093397083	0.925588107	0.964363534	0.022290147
ENSMUSG00000042700	Sipa1l1	217692	protein_coding	1443.128359	0.022308718	0.075384795	-0.295931276	0.767282565	0.883364555	0.022308718
ENSMUSG00000027651	Rprd1b	70470	protein_coding	406.2014306	-0.022315367	0.055583684	0.401473345	0.688071659	0.833786242	0.022315367
ENSMUSG00000001751	Naglu	27419	protein_coding	376.0621458	0.022328734	0.098364693	-0.226999477	0.820424149	0.9092783	0.022328734
ENSMUSG00000003346	Abhd17a	216169	protein_coding	936.1278651	-0.022335931	0.098211706	0.227426357	0.820092227	0.909231489	0.022335931
ENSMUSG00000021962	Dcp1a	75901	protein_coding	330.7767239	0.022349482	0.06844019	-0.32655493	0.744004537	0.870923726	0.022349482
ENSMUSG00000044982	Sft2d3	67158	protein_coding	72.0861204	-0.022354416	0.12057786	0.185394032	0.852919989	0.92788168	0.022354416
ENSMUSG00000024036	Slc37a1	224674	protein_coding	2369.972576	-0.022381918	0.062765221	0.356597454	0.721393184	0.855640813	0.022381918
ENSMUSG00000060121	Gemin2	66603	protein_coding	72.70527017	-0.022406353	0.138983713	0.161215671	0.871923538	0.936943344	0.022406353
ENSMUSG00000004931	Apba3	57267	protein_coding	451.2274431	-0.022429974	0.095273669	0.235426789	0.813877496	0.907703989	0.022429974
ENSMUSG00000027312	Atrn	11990	protein_coding	780.6632974	-0.022446365	0.078771343	0.284955973	0.775677872	0.88791795	0.022446365
ENSMUSG00000042599	Kdm7a	338523	protein_coding	1083.957843	0.022447067	0.087047049	-0.257872808	0.796505071	0.898891871	0.022447067
ENSMUSG00000018583	G3bp1	27041	protein_coding	2038.528828	0.022477136	0.114540489	-0.19623747	0.844424302	0.923559369	0.022477136

ensembl_gene_id	external_gene_name	entrezgene_id	gene_biotype	baseMean	log2FoldChange	lfcSE	stat	pvalue	padj	absLog2FC
ENSMUSG00000021576	Pdcd6	18570	protein_coding	823.4713197	-0.022507771	0.056373116	0.399264275	0.689698487	0.834370416	0.022507771
ENSMUSG00000001988	Npas1	18142	protein_coding	7.849728765	0.022516297	0.510847161	-0.044076387	0.964843515	0.982587667	0.022516297
ENSMUSG00000060373	Hnrnpc	15381	protein_coding	1708.710343	-0.022546667	0.097674299	0.230835207	0.817442828	0.908714429	0.022546667
ENSMUSG00000050043	Tmx2	66958	protein_coding	407.6303472	0.02255415	0.104000422	-0.216865952	0.828312812	0.913317606	0.02255415
ENSMUSG00000023987	Pgc	109820	protein_coding	1.929045266	0.022594638	1.957629537	-0.011541835	0.990791152	1	0.022594638
ENSMUSG00000029635	Cdk8	264064	protein_coding	478.9562782	0.0226503	0.069437663	-0.326196177	0.744275934	0.870990647	0.0226503
ENSMUSG00000029384	2010109A12Rik	75610	protein_coding	4.769351291	0.022757241	0.576617418	-0.039466794	0.968518227	0.984122985	0.022757241
ENSMUSG00000026014	Raph1	77300	protein_coding	620.1006443	0.022789905	0.17117394	-0.133138871	0.894083554	0.947786691	0.022789905
ENSMUSG00000021494	Ddx41	72935	protein_coding	393.822221	0.02279054	0.073753284	-0.309010507	0.757313534	0.878311377	0.02279054
ENSMUSG00000117648	Gm32282	102634780	lncRNA	2.258762352	-0.022795634	0.767067117	0.029717914	0.976292025	1	0.022795634
ENSMUSG00000033352	Map2k4	26398	protein_coding	590.2213792	0.02281479	0.082257008	-0.277359827	0.781503825	0.890612766	0.02281479
ENSMUSG00000019969	Psen1	19164	protein_coding	2121.944964	-0.022825967	0.052726637	0.43291149	0.665079072	0.819390913	0.022825967
ENSMUSG00000029054	Gabrd	14403	protein_coding	9.870224868	-0.022826165	0.529357605	0.043120501	0.965605477	0.982706396	0.022826165
ENSMUSG00000070327	Rnf213	672511	protein_coding	2399.813876	0.022838147	0.186993267	-0.122133527	0.90279327	0.95200802	0.022838147
ENSMUSG00000006205	Htra1	56213	protein_coding	1.526988781	-0.022848356	1.111123467	0.020563292	0.983594023	1	0.022848356
ENSMUSG00000004591	Pkn2	109333	protein_coding	1488.802224	0.022852622	0.056366477	-0.405429303	0.685161975	0.832151567	0.022852622
ENSMUSG00000029561	Oasl2	23962	protein_coding	378.9761745	-0.022853082	0.460802192	0.049594125	0.960445828	0.980697519	0.022853082
ENSMUSG00000043621	Ubxn10	212190	protein_coding	120.1361099	0.022899585	0.1468951	-0.155890734	0.876119149	0.938630585	0.022899585
ENSMUSG00000027502	Rtf2	66404	protein_coding	817.1982904	0.022908481	0.05970656	-0.383684488	0.701212311	0.842046256	0.022908481
ENSMUSG00000004945	Tmem242	70544	protein_coding	424.2974948	-0.022918071	0.101125509	0.22662997	0.820711486	0.909368591	0.022918071
ENSMUSG00000028517	Plpp3	67916	protein_coding	65.39564912	-0.022926999	0.167112857	0.137194704	0.890876897	0.946592528	0.022926999
ENSMUSG00000029599	Ddx54	71990	protein_coding	992.8838063	-0.022938897	0.05107176	0.449150319	0.653323224	0.811872956	0.022938897
ENSMUSG00000055408	Hottip	791364	lncRNA	188.298668	-0.02298329	0.207520281	0.110752021	0.911812993	0.957015366	0.02298329
ENSMUSG00000041712	Ubr7	66622	protein_coding	311.8382255	-0.022987788	0.089760269	0.25610204	0.797872047	0.899696756	0.022987788
ENSMUSG00000026809	Spaca9	69987	protein_coding	1.794774418	0.023038858	0.898219441	-0.025649476	0.979536923	1	0.023038858
ENSMUSG00000040842	Szrd1	213491	protein_coding	1426.485452	0.023044213	0.064304519	-0.358360701	0.720073397	0.855153117	0.023044213
ENSMUSG00000010045	Tmem115	56395	protein_coding	591.2177238	0.023130235	0.06145808	-0.376357922	0.706650823	0.846208704	0.023130235
ENSMUSG00000039536	Stau1	20853	protein_coding	487.2401182	-0.023195197	0.071872777	0.322725768	0.746902935	0.872440598	0.023195197
ENSMUSG00000094091	Ankrd40cl	102639132	protein_coding	430.4136744	-0.023197839	0.249396305	0.093015969	0.925890875	0.964583514	0.023197839
ENSMUSG00000027219	Slc28a2	269346	protein_coding	94.07853793	-0.023255107	0.186645517	0.124595045	0.900844151	0.951246263	0.023255107
ENSMUSG00000036376	Abt1	30946	protein_coding	149.3202402	-0.023271642	0.104197367	0.223341943	0.823269386	0.910297568	0.023271642
ENSMUSG00000031497	Tnfsf13b	24099	protein_coding	51.60539284	0.023278894	0.200498436	-0.116105116	0.907569235	0.954867376	0.023278894
ENSMUSG00000022350	Washc5	223593	protein_coding	560.9633071	0.023284061	0.070733802	-0.329178692	0.742020628	0.869666246	0.023284061
ENSMUSG00000043541	Casc1	320662	protein_coding	10.12888402	-0.023298723	0.572513563	0.040695496	0.967538653	0.983741738	0.023298723
ENSMUSG00000025915	Sgk3	170755	protein_coding	61.23617362	-0.023300443	0.157142707	0.14827569	0.8821252	0.941960269	0.023300443
ENSMUSG00000071019	Sdr16c6	242286	protein_coding	8.152936471	-0.023318047	0.576843466	0.040423525	0.967755475	0.983901614	0.023318047
ENSMUSG00000024425	Ndfip1	65113	protein_coding	1128.279776	-0.023350186	0.081787807	0.285497156	0.775263283	0.887843124	0.023350186
ENSMUSG00000039148	Sart1	20227	protein_coding	699.9519779	0.023359414	0.052368501	-0.446058476	0.655555007	0.81317775	0.023359414
ENSMUSG00000042389	Tsen2	381802	protein_coding	67.46692245	0.02337026	0.198427941	-0.117777061	0.906244306	0.954081249	0.02337026

ensembl_gene_id	external_gene_name	entrezgene_id	gene_biotype	baseMean	log2FoldChange	lfcSE	stat	pvalue	padj	absLog2FC
ENSMUSG00000090176	Cd200r2	271375	protein_coding	67.10904905	-0.023373302	0.219437612	0.106514568	0.915174098	0.95833356	0.023373302
ENSMUSG00000043587	Pxylp1	235534	protein_coding	561.0073743	-0.023380032	0.149927839	0.1559419	0.876078818	0.938630585	0.023380032
ENSMUSG00000100727	1700003P14Rik	75890	lncRNA	0.767302246	0.02338233	1.702249992	-0.013736132	0.989040497	1	0.02338233
ENSMUSG00000027207	Galk2	69976	protein_coding	591.7131392	-0.023410196	0.102569284	0.228237883	0.819461312	0.908985828	0.023410196
ENSMUSG00000048486	Fitm2	228859	protein_coding	250.5893746	-0.023412304	0.110708897	0.211476266	0.83251565	0.915629096	0.023412304
ENSMUSG00000035125	Gcfc2	330361	protein_coding	70.6160768	0.023415172	0.144942452	-0.161548062	0.87166176	0.936825965	0.023415172
ENSMUSG00000036885	Arhgef26	622434	protein_coding	95.97030486	0.023441679	0.137939199	-0.169942114	0.865055661	0.932932188	0.023441679
ENSMUSG00000031449	Atp4b	11945	protein_coding	1.287689153	-0.02344889	2.052601201	0.011423987	0.990885175	1	0.02344889
ENSMUSG00000026584	Scyl3	240880	protein_coding	327.9850113	-0.023452992	0.07244851	0.323719459	0.746150437	0.872287153	0.023452992
ENSMUSG00000029047	Pex10	668173	protein_coding	102.4749671	0.023484124	0.12184457	-0.192738372	0.847163871	0.924722861	0.023484124
ENSMUSG00000022779	Top3b	21976	protein_coding	474.8050027	0.023567597	0.05204183	-0.452858722	0.650650476	0.810197705	0.023567597
ENSMUSG00000030986	Dhx32	101437	protein_coding	1731.987154	-0.023631275	0.079218769	0.298303989	0.76547116	0.882510888	0.023631275
ENSMUSG00000021156	Zmynd11	66505	protein_coding	572.5057062	0.023636953	0.04701495	-0.502753962	0.615137265	0.787654245	0.023636953
ENSMUSG00000019173	Rab5c	19345	protein_coding	1724.214462	-0.023660149	0.068355783	0.346132369	0.729243238	0.86030736	0.023660149
ENSMUSG00000046873	Mbtps2	270669	protein_coding	150.3522418	0.02366344	0.119512173	-0.19800025	0.843044868	0.922356748	0.02366344
ENSMUSG00000024736	Tmem132a	98170	protein_coding	51.42025214	-0.023671079	0.151263426	0.156489111	0.875647502	0.938630585	0.023671079
ENSMUSG00000025218	Poll	56626	protein_coding	131.1007463	0.023675479	0.104333442	-0.226921287	0.820484949	0.9092783	0.023675479
ENSMUSG00000030019	Fbxl14	101358	protein_coding	531.3006424	0.023705135	0.078607681	-0.301562585	0.762985534	0.881056435	0.023705135
ENSMUSG00000056153	Socs6	54607	protein_coding	630.946538	-0.023707059	0.071787304	0.33024028	0.741218413	0.869043726	0.023707059
ENSMUSG00000104960	Snhg8	69895	lncRNA	145.0502993	-0.023738594	0.167215122	0.141964399	0.887108126	0.944106696	0.023738594
ENSMUSG00000008429	Herpud2	80517	protein_coding	324.1871457	0.023764623	0.095799522	-0.248066198	0.804083188	0.903431064	0.023764623
ENSMUSG00000086544	Chn1os3	329427	lncRNA	2.627049784	0.023767254	0.93667094	-0.025374177	0.979756508	1	0.023767254
ENSMUSG00000026718	Stam	20844	protein_coding	549.9711771	-0.023782587	0.077851482	0.305486632	0.759995549	0.879445484	0.023782587
ENSMUSG00000031681	Smad1	17125	protein_coding	692.3280126	-0.023899163	0.066884485	0.357319984	0.720852271	0.855367853	0.023899163
ENSMUSG00000092981	Mir5125	100628593	miRNA	14.41343606	-0.023913029	0.349671024	0.06838722	0.945477395	0.973720842	0.023913029
ENSMUSG00000023932	Cdc5l	71702	protein_coding	797.9172009	-0.023929167	0.108195761	0.221165476	0.82496359	0.911456552	0.023929167
ENSMUSG00000029788	Cpa5	74649	protein_coding	1.127913505	-0.023936136	1.935120322	0.012369327	0.990130956	1	0.023936136
ENSMUSG00000044308	Ubr3	68795	protein_coding	1134.920819	0.023960158	0.075960417	-0.315429523	0.752435554	0.875793979	0.023960158
ENSMUSG00000030657	Xylt1	233781	protein_coding	142.1495528	-0.023978244	0.237869193	0.100804326	0.919705792	0.961084696	0.023978244
ENSMUSG00000022620	Arsa	11883	protein_coding	257.9916799	0.023992338	0.068054023	-0.352548417	0.724427015	0.857268916	0.023992338
ENSMUSG00000025885	Myo5b	17919	protein_coding	3528.546107	-0.024001274	0.083199653	0.288478058	0.772980824	0.886212533	0.024001274
ENSMUSG00000027968	Larp7	28036	protein_coding	238.6832536	0.024029282	0.106183466	-0.226299657	0.820968365	0.909531167	0.024029282
ENSMUSG00000060450	Rnf14	56736	protein_coding	691.4625202	-0.024073602	0.085284021	0.28227564	0.777732163	0.888933284	0.024073602
ENSMUSG00000020441	2310033P09Rik	67862	protein_coding	103.7293549	0.024078543	0.114342895	-0.210581888	0.833213546	0.915993052	0.024078543
ENSMUSG00000021731	Mrps30	59054	protein_coding	244.1009014	0.024094087	0.106232345	-0.226805565	0.820574936	0.9092783	0.024094087
ENSMUSG00000030816	Rnf40	233900	protein_coding	840.9840696	0.024135836	0.056402003	-0.42792516	0.668705608	0.822256556	0.024135836
ENSMUSG00000060317	Acnat2	209186	protein_coding	0.77354633	-0.024195822	1.94693547	0.012427645	0.990084429	1	0.024195822
ENSMUSG00000022853	Ehhadh	74147	protein_coding	703.6897829	0.024202596	0.078871906	-0.306859523	0.7589503	0.878912418	0.024202596
ENSMUSG00000042705	Commd10	69456	protein_coding	203.045889	-0.024219601	0.091979344	0.263315654	0.792307295	0.896636371	0.024219601

ensembl_gene_id	external_gene_name	entrezgene_id	gene_biotype	baseMean	log2FoldChange	lfcSE	stat	pvalue	padj	absLog2FC
ENSMUSG00000015714	Cers2	76893	protein_coding	693.2637983	0.024304618	0.072120141	-0.337001811	0.736115535	0.865765398	0.024304618
ENSMUSG00000031584	Gsr	14782	protein_coding	1652.319842	0.024329711	0.097470089	-0.249612073	0.802887361	0.902682351	0.024329711
ENSMUSG00000038859	Baiap2l1	66898	protein_coding	1701.702209	-0.024338443	0.100031688	0.243307335	0.807767323	0.905233898	0.024338443
ENSMUSG00000058748	Zfp958	233987	protein_coding	114.5590951	-0.024347293	0.150982375	0.161259172	0.871889279	0.936943344	0.024347293
ENSMUSG00000029550	Sppl3	74585	protein_coding	563.0516496	-0.024353007	0.057807972	0.421274199	0.673554868	0.824971081	0.024353007
ENSMUSG00000044730	9930104L06Rik	194268	protein_coding	93.41051303	-0.024364311	0.106812787	0.228102939	0.819566215	0.909014472	0.024364311
ENSMUSG00000052557	Gan	209239	protein_coding	459.3403553	-0.024397566	0.103149678	0.236525858	0.813024644	0.907217398	0.024397566
ENSMUSG00000032263	Bckdhb	12040	protein_coding	89.94772499	-0.024435906	0.150834859	0.162004365	0.871302416	0.936580622	0.024435906
ENSMUSG00000021277	Traf3	22031	protein_coding	349.6321721	0.024443397	0.109556082	-0.223113095	0.823447487	0.910384401	0.024443397
ENSMUSG00000027796	Smad9	55994	protein_coding	4.826303239	-0.024443702	0.515571786	0.04741086	0.962185773	0.981434574	0.024443702
ENSMUSG00000027463	Slc52a3	69698	protein_coding	493.0956375	-0.024453735	0.09308978	0.262689791	0.792789686	0.896829747	0.024453735
ENSMUSG00000062470	Fbxl12os	66662	lncRNA	9.547693362	-0.024478013	0.390190785	0.062733447	0.949978763	0.975869522	0.024478013
ENSMUSG00000054814	Usp46	69727	protein_coding	330.4638833	-0.024479097	0.106754342	0.229303054	0.81863338	0.908812719	0.024479097
ENSMUSG00000047281	Sfn	55948	protein_coding	3686.64763	-0.024486508	0.139501578	0.175528538	0.860664348	0.931474719	0.024486508
ENSMUSG00000054894	Dmac2l	68055	protein_coding	96.13079775	-0.024492227	0.125517586	0.195129843	0.845291301	0.923801556	0.024492227
ENSMUSG00000048307	Ankrd46	68839	protein_coding	255.2132908	-0.024499205	0.08092599	0.302735928	0.762091115	0.880874346	0.024499205
ENSMUSG00000086877	A230072C01Rik	320742	lncRNA	7.91996384	0.024514094	0.431525819	-0.056807942	0.954698187	0.977426278	0.024514094
ENSMUSG00000025036	Sfxn2	94279	protein_coding	188.0910849	0.024598774	0.130759232	-0.188122658	0.8507805	0.927198731	0.024598774
ENSMUSG00000030909	Anks4b	72074	protein_coding	1690.25835	-0.024638401	0.098365164	0.250478935	0.802216993	0.90234117	0.024638401
ENSMUSG00000019826	Zbtb24	268294	protein_coding	199.5242096	0.024655183	0.101935229	-0.24187107	0.808880067	0.905678041	0.024655183
ENSMUSG00000038417	Fig4	103199	protein_coding	317.9947148	-0.024676458	0.081771489	0.301773374	0.762824829	0.881056435	0.024676458
ENSMUSG00000118249	Gm36608	102640578	lncRNA	1.390703487	0.024717413	0.952711182	-0.025944288	0.979301775	1	0.024717413
ENSMUSG00000056310	Tyw1	100929	protein_coding	279.783445	0.02476003	0.085170251	-0.290712188	0.77127145	0.885199039	0.02476003
ENSMUSG00000024381	Bin1	30948	protein_coding	167.2717602	0.024769901	0.0985788	-0.251270058	0.801605323	0.901751383	0.024769901
ENSMUSG00000037351	Actr1b	226977	protein_coding	1988.520245	-0.024774675	0.043999432	0.563068058	0.573388541	0.758644032	0.024774675
ENSMUSG00000025340	Rabgef1	56715	protein_coding	370.7417339	-0.02487062	0.070729312	0.351631021	0.725114997	0.857652848	0.02487062
ENSMUSG00000024764	Naa40	70999	protein_coding	447.3510272	-0.024874275	0.108034665	0.230243462	0.817902591	0.908812719	0.024874275
ENSMUSG00000091803	Cox16	66272	protein_coding	103.9265304	-0.024908542	0.127514749	0.19533852	0.845127943	0.923798341	0.024908542
ENSMUSG00000024384	Iws1	73473	protein_coding	766.3319381	-0.024934443	0.070599722	0.353180475	0.723953144	0.857076652	0.024934443
ENSMUSG00000070644	Etnk2	214253	protein_coding	15.49521301	-0.024939959	0.370397797	0.067332904	0.946316683	0.973795719	0.024939959
ENSMUSG00000021263	Degs2	70059	protein_coding	2217.608908	0.024954827	0.084683484	-0.294683518	0.768235655	0.883975099	0.024954827
ENSMUSG00000030205	Gprc5d	93746	protein_coding	0.584102615	-0.024958935	1.633164761	0.015282558	0.987806758	1	0.024958935
ENSMUSG00000024218	Taf11	68776	protein_coding	157.9914705	-0.024964718	0.083743458	0.298109474	0.765619611	0.882510888	0.024964718
ENSMUSG00000052428	Tmco1	68944	protein_coding	532.4164214	0.024990846	0.113522353	-0.220140311	0.825761882	0.91190529	0.024990846
ENSMUSG00000040128	Pnrc1	108767	protein_coding	1156.070224	0.025134395	0.185984149	-0.135142671	0.892499073	0.947202684	0.025134395
ENSMUSG00000026142	Rhbdd1	76867	protein_coding	315.8517257	0.025187809	0.067897308	-0.370969182	0.710660485	0.849070124	0.025187809
ENSMUSG00000036667	Tcaf1	77574	protein_coding	240.3176082	0.02519433	0.089672084	-0.280960687	0.778740553	0.889481359	0.02519433
ENSMUSG00000029621	Arpc1a	56443	protein_coding	926.287345	-0.025243508	0.074585042	0.338452684	0.735022078	0.864910546	0.025243508
ENSMUSG00000042535	Gtpbp1	14904	protein_coding	519.9626541	0.025272472	0.059820242	-0.422473591	0.672679373	0.824518194	0.025272472

ensembl_gene_id	external_gene_name	entrezgene_id	gene_biotype	baseMean	log2FoldChange	lfcSE	stat	pvalue	padj	absLog2FC
ENSMUSG00000042410	Agps	228061	protein_coding	1105.740173	-0.025283286	0.076524907	0.330392901	0.741103105	0.868970227	0.025283286
ENSMUSG00000024400	Wdr33	74320	protein_coding	709.0310368	0.025293348	0.051349552	-0.492571923	0.622315091	0.792910378	0.025293348
ENSMUSG00000028076	Cd1d1	12479	protein_coding	57.53808719	0.025306402	0.208896807	-0.121143079	0.903577708	0.952305972	0.025306402
ENSMUSG00000034190	Chmp7	105513	protein_coding	525.8498826	0.02533432	0.050162983	-0.505040145	0.61353064	0.786448489	0.02533432
ENSMUSG00000102923	Gm31728	102634049	lncRNA	1.876452905	0.025335847	0.909188658	-0.027866435	0.977768679	1	0.025335847
ENSMUSG00000021549	Rasa1	218397	protein_coding	571.1916931	0.0253641	0.076413315	-0.331932988	0.73993986	0.868161055	0.0253641
ENSMUSG00000043542	Zc2hc1a	67306	protein_coding	89.828691	-0.025373964	0.144509539	0.175586772	0.860618595	0.931474719	0.025373964
ENSMUSG00000045211	Nudt18	213484	protein_coding	65.1896984	-0.025374562	0.142929189	0.177532401	0.859090217	0.931099354	0.025374562
ENSMUSG00000018446	C1qbp	12261	protein_coding	659.08764	-0.025385037	0.101886407	0.249150379	0.803244461	0.902857271	0.025385037
ENSMUSG00000029470	P2rx4	18438	protein_coding	1492.732949	0.025385804	0.156563874	-0.16214343	0.871192906	0.936523867	0.025385804
ENSMUSG00000020130	Tbc1d15	66687	protein_coding	546.4920126	-0.02540319	0.146933616	0.172888895	0.862738761	0.932166328	0.02540319
ENSMUSG00000032624	Eml4	78798	protein_coding	1098.984992	-0.025412526	0.109330563	0.232437533	0.8161982	0.908409285	0.025412526
ENSMUSG00000024234	Mtpap	67440	protein_coding	266.1735047	-0.025424452	0.094628901	0.268675335	0.788179534	0.894017228	0.025424452
ENSMUSG00000022898	Vps26c	13185	protein_coding	490.1219003	-0.02543624	0.055046448	0.462086858	0.644019034	0.80597323	0.02543624
ENSMUSG00000046756	Mrps7	50529	protein_coding	482.3950551	-0.025444986	0.080198866	0.317273643	0.751035975	0.875081807	0.025444986
ENSMUSG00000041328	Pcf11	74737	protein_coding	1019.938773	-0.025453101	0.08507407	0.299187528	0.764796965	0.882219689	0.025453101
ENSMUSG00000027663	Zmat3	22401	protein_coding	207.815639	0.025465434	0.111960651	-0.227449858	0.820073955	0.909231489	0.025465434
ENSMUSG00000005823	Gpr108	78308	protein_coding	1154.099185	0.025467101	0.048101052	-0.529449982	0.596493333	0.774662172	0.025467101
ENSMUSG00000033382	Trappc8	75964	protein_coding	1073.980405	0.025556398	0.065576542	-0.389718595	0.696744645	0.839060823	0.025556398
ENSMUSG00000098022	Zfp82	330502	protein_coding	3.295089213	-0.025604036	0.579985312	0.044146007	0.96478802	1	0.025604036
ENSMUSG00000020114	Cand1	71902	protein_coding	1318.162028	0.025620584	0.077936961	-0.32873471	0.742356216	0.869945398	0.025620584
ENSMUSG00000040543	Pitpnm3	327958	protein_coding	178.9029784	-0.025625957	0.145853329	0.175696757	0.860532183	0.931474719	0.025625957
ENSMUSG00000094870	Zfp131	72465	protein_coding	322.0304232	0.025659682	0.080981008	-0.316860497	0.751349458	0.875081807	0.025659682
ENSMUSG00000074896	Ifit3	15959	protein_coding	48.67569434	0.025698077	0.437042705	-0.058799922	0.953111471	0.976710073	0.025698077
ENSMUSG00000029071	Dvl1	13542	protein_coding	741.5323333	-0.025735671	0.098510688	0.261247497	0.793901652	0.897263004	0.025735671
ENSMUSG00000034863	Ano8	382014	protein_coding	182.0174952	0.025772377	0.119081864	-0.216425708	0.828655929	0.913504709	0.025772377
ENSMUSG00000025358	Cdk2	12566	protein_coding	228.5310284	0.025774013	0.141668249	-0.18193218	0.85563595	0.929786937	0.025774013
ENSMUSG00000052549	Arl13a	74448	protein_coding	1.62745793	-0.025794406	1.121467747	0.023000577	0.981649812	1	0.025794406
ENSMUSG00000041741	Pde3a	54611	protein_coding	1375.498335	-0.025845399	0.090470079	0.285678966	0.775124016	0.88783793	0.025845399
ENSMUSG00000036323	Srp72	66661	protein_coding	1122.909158	0.025863753	0.10278369	-0.251632853	0.801324862	0.901569434	0.025863753
ENSMUSG00000029802	Abcg2	26357	protein_coding	989.3474481	0.025885196	0.120649273	-0.214549124	0.830118864	0.914175515	0.025885196
ENSMUSG00000026289	Atg16l1	77040	protein_coding	392.1685866	-0.025970647	0.077351913	0.33574667	0.737061909	0.866569868	0.025970647
ENSMUSG00000067365	Tmem128	66309	protein_coding	329.7400322	0.02599852	0.087524646	-0.297042279	0.766434231	0.882690208	0.02599852
ENSMUSG00000025019	Lcor	212391	protein_coding	934.378249	0.025999358	0.05805942	-0.447806027	0.654293193	0.812407823	0.025999358
ENSMUSG00000029190	D5Ertd579e	320661	protein_coding	550.9171374	-0.026040617	0.107857038	0.241436418	0.80921689	0.905779176	0.026040617
ENSMUSG00000035212	Leprot	230514	protein_coding	831.3905088	-0.0260487	0.07068912	0.368496595	0.712502983	0.850598485	0.0260487
ENSMUSG00000009907	Vps4b	20479	protein_coding	1618.563379	0.026052428	0.052869346	-0.492770014	0.6221751	0.792833975	0.026052428
ENSMUSG00000035629	Rubcn	100502698	protein_coding	437.3279173	0.026057749	0.06605506	-0.39448528	0.693222793	0.837078181	0.026057749
ENSMUSG00000029480	Dhx37	208144	protein_coding	186.3498753	0.026067189	0.091418504	-0.285141277	0.775535907	0.88791795	0.026067189

ensembl_gene_id	external_gene_name	entrezgene_id	gene_biotype	baseMean	log2FoldChange	lfcSE	stat	pvalue	padj	absLog2FC
ENSMUSG00000023004	Tuba1b	22143	protein_coding	395.485161	0.026131731	0.241186594	-0.108346534	0.913720811	0.957921881	0.026131731
ENSMUSG00000045193	Cirbp	12696	protein_coding	251.0645325	0.026144322	0.136969397	-0.190877108	0.848621876	0.925637253	0.026144322
ENSMUSG00000027465	Tbc1d20	67231	protein_coding	1007.34846	0.026183089	0.067460967	-0.388122059	0.697925709	0.839976943	0.026183089
ENSMUSG00000100084	2310068J16Rik	70281	lncRNA	9.689084562	0.026229054	0.371998407	-0.070508512	0.943788926	0.97289203	0.026229054
ENSMUSG00000026563	Tada1	27878	protein_coding	102.7396401	-0.026230709	0.109289667	0.240010875	0.810321826	0.906158059	0.026230709
ENSMUSG00000042380	Smim12	80284	protein_coding	186.844924	-0.026254346	0.086357512	0.304019257	0.761113221	0.880245376	0.026254346
ENSMUSG00000037296	Lsm1	67207	protein_coding	188.9399079	0.026296129	0.073330714	-0.35859639	0.719897048	0.855153117	0.026296129
ENSMUSG00000050714	Zbtb26	320633	protein_coding	87.58788839	-0.026303387	0.152476899	0.17250736	0.863038675	0.932166328	0.026303387
ENSMUSG00000116564	Riok2	67045	protein_coding	229.7384512	-0.026359277	0.114767794	0.229674859	0.818344433	0.908812719	0.026359277
ENSMUSG00000002635	Pdcd2l	68079	protein_coding	132.1380092	0.02636089	0.107132846	-0.246057964	0.805637363	0.904254429	0.02636089
ENSMUSG00000029681	Bcl7b	12054	protein_coding	217.0827428	-0.026402473	0.090663877	0.29121271	0.770888642	0.885199039	0.026402473
ENSMUSG00000003363	Pld3	18807	protein_coding	339.570721	-0.026411003	0.08338828	0.316723198	0.751453645	0.875092959	0.026411003
ENSMUSG00000026519	Tmem63a	208795	protein_coding	1442.579499	-0.026416692	0.113591991	0.232557694	0.816104882	0.908409285	0.026416692
ENSMUSG00000079523	Tmsb10	19240	protein_coding	2004.191944	0.026449728	0.094469334	-0.279982162	0.77949119	0.890072723	0.026449728
ENSMUSG00000028959	Fastk	66587	protein_coding	816.276798	-0.026461362	0.073157806	0.361702503	0.717574356	0.853563561	0.026461362
ENSMUSG00000027405	Nop56	67134	protein_coding	486.0083344	0.026462379	0.111696272	-0.236913719	0.812723727	0.907147741	0.026462379
ENSMUSG00000013736	Trnt1	70047	protein_coding	286.4460552	0.026489297	0.101051022	-0.262137845	0.793215171	0.897079532	0.026489297
ENSMUSG00000042851	Zc3h6	78751	protein_coding	69.59675575	0.02652958	0.195415718	-0.135759704	0.892011248	0.947202684	0.02652958
ENSMUSG00000019590	Cyb561	13056	protein_coding	1222.706352	-0.026538283	0.118554863	0.223848118	0.822875487	0.910240646	0.026538283
ENSMUSG00000034321	Exosc1	66583	protein_coding	108.5767924	0.026545764	0.131284365	-0.2022005	0.839759982	0.920280009	0.026545764
ENSMUSG00000021094	Dhrs7	66375	protein_coding	294.3031538	-0.02656098	0.140515398	0.189025403	0.850072907	0.926816617	0.02656098
ENSMUSG00000033060	Lmo7	380928	protein_coding	3213.51187	0.026562066	0.11306737	-0.234922476	0.814268905	0.907710402	0.026562066
ENSMUSG00000034258	Flvcr2	217721	protein_coding	3.830669675	0.026574321	0.847378034	-0.031360644	0.974981927	1	0.026574321
ENSMUSG00000002210	Smg9	71997	protein_coding	288.7587467	0.026580454	0.094184074	-0.282218137	0.777776252	0.888933284	0.026580454
ENSMUSG00000003380	Rabac1	14470	protein_coding	447.3826873	0.026589143	0.069582706	-0.382122874	0.702370226	0.84294636	0.026589143
ENSMUSG00000045817	Zfp36l2	12193	protein_coding	2015.553475	0.026604543	0.099917705	-0.266264553	0.790035471	0.895242955	0.026604543
ENSMUSG00000042429	Adora1	11539	protein_coding	20.47639689	-0.026621779	0.342411291	0.07774796	0.938028543	0.970517013	0.026621779
ENSMUSG00000027281	Slx4ip	74243	protein_coding	161.5268643	0.026632597	0.113272384	-0.235119951	0.814115634	0.907710402	0.026632597
ENSMUSG00000004934	Pias4	59004	protein_coding	384.084751	0.026680563	0.076756915	-0.347598163	0.728141986	0.859565127	0.026680563
ENSMUSG00000018509	Cenpv	73139	protein_coding	344.5544413	-0.02670446	0.084266953	0.316903115	0.751317119	0.875081807	0.02670446
ENSMUSG00000055204	Ankrd17	81702	protein_coding	1775.077928	0.026742591	0.06317487	-0.423310581	0.672068675	0.824189967	0.026742591
ENSMUSG00000010048	Ifrd2	15983	protein_coding	352.5350191	-0.02675173	0.109541699	0.244215038	0.807064281	0.904987729	0.02675173
ENSMUSG00000004846	Plod3	26433	protein_coding	302.6770271	0.026757184	0.099440165	-0.269078236	0.787869478	0.893788319	0.026757184
ENSMUSG00000036172	Cd200r3	74603	protein_coding	3.304358162	-0.026798009	0.839588998	0.031918008	0.974537437	1	0.026798009
ENSMUSG00000025877	Hk3	212032	protein_coding	2.063447716	-0.026798679	1.911409304	0.014020377	0.988813724	1	0.026798679
ENSMUSG00000028382	Ptbp3	230257	protein_coding	3433.702149	-0.026840095	0.067325703	0.398660458	0.690143408	0.834763659	0.026840095
ENSMUSG00000040463	Mybbp1a	18432	protein_coding	754.3354521	0.026845184	0.124182828	-0.216174692	0.82885158	0.913504709	0.026845184
ENSMUSG00000037029	Zfp146	26465	protein_coding	348.6647308	0.02687483	0.119181357	-0.225495252	0.821594019	0.909919095	0.02687483
ENSMUSG00000034793	G6pc3	68401	protein_coding	373.4205932	-0.026883338	0.114913861	0.233943386	0.815028931	0.908068261	0.026883338

ensembl_gene_id	external_gene_name	entrezgene_id	gene_biotype	baseMean	log2FoldChange	lfcSE	stat	pvalue	padj	absLog2FC
ENSMUSG00000019984	Med23	70208	protein_coding	443.2069647	0.026913402	0.075089865	-0.358415902	0.720032093	0.855153117	0.026913402
ENSMUSG00000005131	4930550C14Rik	75311	protein_coding	3.757173847	0.026937139	0.536715363	-0.050188873	0.959971879	1	0.026937139
ENSMUSG00000032482	Cspg5	29873	protein_coding	14.38678507	-0.026972572	0.573354154	0.047043475	0.962478578	0.981497544	0.026972572
ENSMUSG00000051390	Zbtb22	81630	protein_coding	493.1156869	-0.026981613	0.070842745	0.380866278	0.703302476	0.843758597	0.026981613
ENSMUSG00000029263	Pigg	433931	protein_coding	305.5888855	0.026988559	0.087085735	-0.309907921	0.756630979	0.877889692	0.026988559
ENSMUSG00000034926	Dhcr24	74754	protein_coding	2077.258113	0.027043337	0.07812619	-0.346149442	0.729230408	0.86030736	0.027043337
ENSMUSG00000098449	Gm7467	665055	lncRNA	18.60101864	-0.027045576	0.284028017	0.095221509	0.924138886	0.963320302	0.027045576
ENSMUSG00000039774	Galnt12	230145	protein_coding	1453.212179	-0.027061741	0.106646778	0.253751137	0.799687825	0.900821606	0.027061741
ENSMUSG00000007035	Msh5	17687	protein_coding	26.20037404	0.027072637	0.24366753	-0.111104818	0.911533228	0.956997404	0.027072637
ENSMUSG00000029670	Ing3	71777	protein_coding	158.6952338	-0.027091206	0.130651597	0.207354574	0.835732956	0.917517221	0.027091206
ENSMUSG00000032312	Csk	12988	protein_coding	890.7028015	0.027103422	0.065131423	-0.416134335	0.677311707	0.827106157	0.027103422
ENSMUSG00000033720	Sfxn5	94282	protein_coding	322.6708872	-0.027123568	0.130225533	0.208281487	0.835009186	0.917066002	0.027123568
ENSMUSG00000066829	Zfp810	235050	protein_coding	166.6600495	-0.027157223	0.123360354	0.220145465	0.825757867	0.91190529	0.027157223
ENSMUSG00000024896	Minpp1	17330	protein_coding	366.4530955	-0.027209297	0.118735834	0.229158261	0.818745913	0.908812719	0.027209297
ENSMUSG00000041396	Mettl18	69962	protein_coding	43.52192248	0.027248547	0.178314638	-0.152811609	0.878546843	0.939901695	0.027248547
ENSMUSG00000039004	Bmp6	12161	protein_coding	8.757105468	-0.027262305	0.562025667	0.048507225	0.961312007	0.981095512	0.027262305
ENSMUSG00000038777	Sema6c	20360	protein_coding	7.040356022	0.027274053	0.710684006	-0.038377187	0.96938695	0.984403993	0.027274053
ENSMUSG00000020572	Nampt	59027	protein_coding	862.0700147	0.027286382	0.084275868	-0.323774556	0.74610872	0.872287153	0.027286382
ENSMUSG00000024576	Csnk1a1	93687	protein_coding	5000.668383	0.027344422	0.050986674	-0.536305261	0.591747595	0.771818402	0.027344422
ENSMUSG00000013858	Tmem259	216157	protein_coding	1039.662488	0.02738981	0.071895604	-0.380966404	0.703228178	0.843730756	0.02738981
ENSMUSG00000063364	3300002I08Rik	69277	protein_coding	14.04859083	0.027409734	0.313656674	-0.087387697	0.930363348	0.966412148	0.027409734
ENSMUSG00000031012	Cask	12361	protein_coding	886.6680215	0.027410019	0.076742494	-0.357168727	0.720965496	0.855440739	0.027410019
ENSMUSG00000038446	Cdc40	71713	protein_coding	262.7338866	0.027435932	0.089894935	-0.305199983	0.760213845	0.879513202	0.027435932
ENSMUSG00000007411	Mark3	17169	protein_coding	794.7288035	-0.027493974	0.05776282	0.475980475	0.634088288	0.800507769	0.027493974
ENSMUSG00000053580	Tanc2	77097	protein_coding	107.1931513	0.027509125	0.106520842	-0.258251104	0.79621312	0.898808084	0.027509125
ENSMUSG00000036249	Rbm43	71684	protein_coding	88.59528472	-0.027532121	0.164185186	0.167689435	0.866827608	0.933837244	0.027532121
ENSMUSG00000114828	AI463229	100042802	lncRNA	22.77517513	-0.027573621	0.300737541	0.091686662	0.926946995	0.965151432	0.027573621
ENSMUSG00000027254	Map1a	17754	protein_coding	28.5173678	0.027575103	0.388284053	-0.071017861	0.94338354	0.972595566	0.027575103
ENSMUSG00000090751	Vmn2r63	435975	protein_coding	2.979752546	-0.027577402	1.973207236	0.013975928	0.988849186	1	0.027577402
ENSMUSG00000021905	Dph3	105638	protein_coding	327.8810703	-0.027610296	0.092844337	0.297382657	0.766174383	0.882690208	0.027610296
ENSMUSG00000030688	Stard10	56018	protein_coding	6196.068577	-0.027705043	0.061993557	0.446901975	0.654945838	0.812734264	0.027705043
ENSMUSG00000039952	Dag1	13138	protein_coding	2232.536582	-0.027715113	0.059065059	0.469230268	0.638905052	0.80314212	0.027715113
ENSMUSG00000035173	Ccdc186	213993	protein_coding	882.7504261	0.027722065	0.084426464	-0.328357528	0.742641352	0.870032583	0.027722065
ENSMUSG00000040312	Cchcr1	240084	protein_coding	175.9538727	0.027749132	0.107664065	-0.257738107	0.796609035	0.898947765	0.027749132
ENSMUSG00000041870	Ankrd13a	68420	protein_coding	2850.742552	-0.027835401	0.040374118	0.689436756	0.49054846	0.69793377	0.027835401
ENSMUSG00000020692	Nle1	217011	protein_coding	85.90620313	0.027841453	0.130373044	-0.213552219	0.830896264	0.914497068	0.027841453
ENSMUSG00000057230	Aak1	269774	protein_coding	515.0837067	-0.0278767	0.088020136	0.316708216	0.751465014	0.875092959	0.0278767
ENSMUSG00000069223	4930451E10Rik	78111	lncRNA	1.515389536	0.027887773	0.834278847	-0.033427401	0.973333759	1	0.027887773
ENSMUSG00000017858	Ift52	245866	protein_coding	328.5696524	0.027888474	0.076932869	-0.362504014	0.716975425	0.853197357	0.027888474

ensembl_gene_id	external_gene_name	entrezgene_id	gene_biotype	baseMean	log2FoldChange	lfcSE	stat	pvalue	padj	absLog2FC
ENSMUSG00000036208	Nepro	212547	protein_coding	174.7937779	-0.027901499	0.098120941	0.284358246	0.776135853	0.888226437	0.027901499
ENSMUSG00000044626	Liph	239759	protein_coding	690.688751	-0.027920044	0.114779047	0.243250355	0.807811461	0.905233898	0.027920044
ENSMUSG00000033444	Specc1l	74392	protein_coding	1975.60469	-0.027944287	0.136245227	0.20510287	0.837491754	0.918959391	0.027944287
ENSMUSG00000020097	Sgpl1	20397	protein_coding	1672.288111	0.027996448	0.089814018	-0.311715795	0.755256523	0.877032235	0.027996448
ENSMUSG00000028977	Casz1	69743	protein_coding	1151.544645	0.028046567	0.164268973	-0.17073563	0.864431645	0.932569867	0.028046567
ENSMUSG00000028988	Ctnnbip1	67087	protein_coding	222.243717	-0.028056429	0.103393502	0.27135583	0.786117371	0.893150482	0.028056429
ENSMUSG00000027274	Mkks	59030	protein_coding	248.4332971	0.028107364	0.163181956	-0.172245537	0.863244499	0.932166328	0.028107364
ENSMUSG00000017781	Pitpna	18738	protein_coding	1534.071778	0.028121098	0.054505873	-0.515927863	0.605904793	0.780796859	0.028121098
ENSMUSG00000068270	Shroom4	208431	protein_coding	36.5027473	0.028138602	0.292618078	-0.096161531	0.923392283	0.962865452	0.028138602
ENSMUSG00000037373	Ctbp1	13016	protein_coding	1998.830201	0.028142367	0.070157989	-0.401128482	0.688325532	0.833786242	0.028142367
ENSMUSG00000021929	Kpna3	16648	protein_coding	413.3639955	0.028148486	0.094589327	-0.297586278	0.766018949	0.882690208	0.028148486
ENSMUSG00000007837	Prrg2	65116	protein_coding	387.9328808	-0.028237607	0.099684601	0.283269494	0.77697026	0.888627112	0.028237607
ENSMUSG00000042228	Lyn	17096	protein_coding	313.0568662	-0.028258807	0.083480517	0.338507811	0.734980542	0.864910546	0.028258807
ENSMUSG00000037119	Fam91a1	210998	protein_coding	1315.670431	0.02826489	0.108977658	-0.259364085	0.795354338	0.898299072	0.02826489
ENSMUSG00000030697	Ppp4c	56420	protein_coding	1237.403828	-0.028268208	0.048229985	0.586112715	0.557799772	0.746959571	0.028268208
ENSMUSG00000031807	Pgls	66171	protein_coding	708.4788464	0.02827082	0.081805894	-0.345584149	0.72965526	0.860514985	0.02827082
ENSMUSG00000016503	Gtf3a	66596	protein_coding	152.3782087	0.028306623	0.121605214	-0.232774744	0.815936325	0.908409285	0.028306623
ENSMUSG00000034442	Trmt5	76357	protein_coding	101.8643898	0.028382547	0.137315027	-0.206696581	0.836246828	0.917959327	0.028382547
ENSMUSG00000058715	Fcer1g	14127	protein_coding	8.645181549	-0.028405548	0.396839956	0.071579354	0.942936671	0.972316965	0.028405548
ENSMUSG00000022568	Scrib	105782	protein_coding	1194.248862	0.028451294	0.053470442	-0.532093862	0.594660988	0.77336837	0.028451294
ENSMUSG00000028850	Gpatch3	242691	protein_coding	76.56250364	0.028467374	0.154144395	-0.184679915	0.853480101	0.92805494	0.028467374
ENSMUSG00000034203	Chchd4	72170	protein_coding	322.8664127	0.028489091	0.070753979	-0.402650025	0.687205703	0.833389267	0.028489091
ENSMUSG00000026573	Xcl1	16963	protein_coding	13.91269909	-0.028496729	0.493814645	0.057707338	0.953981748	0.977047797	0.028496729
ENSMUSG00000048755	Mcat	223722	protein_coding	192.4600739	-0.028498825	0.157410882	0.181047365	0.856330398	0.930174321	0.028498825
ENSMUSG00000037487	Ubr5	70790	protein_coding	2828.794598	0.028516507	0.061917335	-0.460557716	0.645115954	0.80668897	0.028516507
ENSMUSG00000039456	Morc3	338467	protein_coding	529.7794746	-0.028518169	0.067095552	0.425038153	0.670808862	0.823378739	0.028518169
ENSMUSG00000067577	A430093F15Rik	403202	lncRNA	1.107032597	-0.028579537	2.183377368	0.0130896	0.989556308	1	0.028579537
ENSMUSG00000019920	Lims1	110829	protein_coding	1160.69259	0.028610992	0.167408724	-0.170905022	0.864298448	0.932569867	0.028610992
ENSMUSG00000023051	Tarbp2	21357	protein_coding	234.0376885	0.028624042	0.121954315	-0.234711183	0.814432907	0.907710402	0.028624042
ENSMUSG00000013089	Etv5	104156	protein_coding	330.2747838	-0.028626152	0.205791513	0.139102686	0.889369006	0.945659633	0.028626152
ENSMUSG00000036452	Arhgap26	71302	protein_coding	418.3124529	0.028647924	0.091199782	-0.314122721	0.753427834	0.876451623	0.028647924
ENSMUSG00000022245	Skor1	207667	protein_coding	2.259739784	0.028693105	1.269513002	-0.022601663	0.981968017	1	0.028693105
ENSMUSG00000020305	Asb3	65257	protein_coding	111.8442959	-0.028708403	0.124800424	0.230034495	0.818064965	0.908812719	0.028708403
ENSMUSG00000091514	Gm17484	100503003	lncRNA	2.496641518	-0.028712276	0.682877201	0.042046031	0.966462003	1	0.028712276
ENSMUSG00000097021	4933433G15Rik	71274	lncRNA	9.197129396	0.028726402	0.416848869	-0.06891323	0.945058688	0.97354273	0.028726402
ENSMUSG00000029669	Tspan12	269831	protein_coding	526.1909569	0.028749978	0.108530855	-0.264901419	0.791085405	0.895862069	0.028749978
ENSMUSG00000020234	Tektip1	432479	protein_coding	10.26914474	0.028781523	0.372656433	-0.077233399	0.938437872	0.970587024	0.028781523
ENSMUSG00000024423	Impact	16210	protein_coding	87.98747125	-0.028785306	0.154022391	0.186890397	0.851746568	0.927574705	0.028785306
ENSMUSG00000033436	Armcx2	67416	protein_coding	4.787585489	0.028809611	0.781104876	-0.036883154	0.970578172	0.984770701	0.028809611

ensembl_gene_id	external_gene_name	entrezgene_id	gene_biotype	baseMean	log2FoldChange	lfcSE	stat	pvalue	padj	absLog2FC
ENSMUSG00000037773	Pced1a	319513	protein_coding	94.31679933	-0.028835112	0.122571265	0.235251811	0.814013295	0.907710402	0.028835112
ENSMUSG00000020993	Trappc6b	78232	protein_coding	293.0651559	0.028892651	0.07579472	-0.381196083	0.703057755	0.843587573	0.028892651
ENSMUSG00000025027	Xpnpep1	170750	protein_coding	905.1091454	0.028948817	0.069006745	-0.419507068	0.67484559	0.825633433	0.028948817
ENSMUSG00000030980	Knop1	66356	protein_coding	395.6497464	-0.028996154	0.110842012	0.261598951	0.793630653	0.897242186	0.028996154
ENSMUSG00000035575	Utp6	216987	protein_coding	472.6999581	0.029030912	0.117158376	-0.247792035	0.804295318	0.903546454	0.029030912
ENSMUSG00000026239	Pde6d	18582	protein_coding	105.1907647	0.029048925	0.107166994	-0.271062239	0.786343164	0.893161222	0.029048925
ENSMUSG00000059851	Kmt5c	232811	protein_coding	538.4007117	0.029063737	0.072444988	-0.401183545	0.688284995	0.833786242	0.029063737
ENSMUSG00000043391	2510009E07Rik	72190	protein_coding	356.2078157	-0.029074643	0.155975167	0.186405591	0.852126708	0.927743965	0.029074643
ENSMUSG00000062234	Gak	231580	protein_coding	2480.915234	-0.029106287	0.05217445	0.557864763	0.576936735	0.761228287	0.029106287
ENSMUSG00000024924	Vldlr	22359	protein_coding	244.1153256	0.029152207	0.124154982	-0.234804972	0.814360109	0.907710402	0.029152207
ENSMUSG00000049791	Fzd4	14366	protein_coding	85.23903084	-0.029188957	0.142704269	0.204541584	0.8379303	0.919173117	0.029188957
ENSMUSG00000020679	Hnf1b	21410	protein_coding	267.7460609	-0.029199673	0.125794433	0.23212214	0.816443149	0.908418149	0.029199673
ENSMUSG00000047767	Atg16l2	73683	protein_coding	132.2639703	-0.029213262	0.20344449	0.143593279	0.885821648	0.943345502	0.029213262
ENSMUSG00000029458	Brap	72399	protein_coding	634.8668499	-0.029296812	0.055342531	0.529372456	0.596547102	0.774662172	0.029296812
ENSMUSG00000068039	Tcp1	21454	protein_coding	2026.855519	0.029301821	0.106566582	-0.274962572	0.783344994	0.891264005	0.029301821
ENSMUSG00000078185	Chml	12663	protein_coding	255.26129	0.029312836	0.121849562	-0.240565785	0.809891671	0.906073508	0.029312836
ENSMUSG00000003438	Timm50	66525	protein_coding	376.8673482	-0.029319459	0.095516821	0.306955982	0.758876877	0.878912418	0.029319459
ENSMUSG00000000766	Oprm1	18390	protein_coding	2.8144158	0.02936544	1.65597877	-0.017732981	0.98585187	1	0.02936544
ENSMUSG00000031575	Ash2l	23808	protein_coding	582.8734968	-0.029383797	0.076666118	0.383269665	0.701519829	0.842293038	0.029383797
ENSMUSG00000056267	Cep70	68121	protein_coding	109.8539129	-0.029385487	0.131127825	0.22409803	0.822681026	0.91020732	0.029385487
ENSMUSG00000022770	Dlg1	13383	protein_coding	980.4266157	-0.029389265	0.048621627	0.604448399	0.545545566	0.737366138	0.029389265
ENSMUSG00000000804	Usp32	237898	protein_coding	494.1788967	-0.029405792	0.100398049	0.292892063	0.769604656	0.884510137	0.029405792
ENSMUSG00000016252	Atp5e	67126	protein_coding	1733.34122	0.029424917	0.09112431	-0.322909628	0.746763684	0.872440598	0.029424917
ENSMUSG00000041351	Rap1gap	110351	protein_coding	1610.303866	-0.029453944	0.120031165	0.245385801	0.806157722	0.904600714	0.029453944
ENSMUSG00000024659	Anxa1	16952	protein_coding	1826.852775	-0.029477614	0.127241489	0.231666686	0.816796908	0.908628099	0.029477614
ENSMUSG00000026355	Mcm6	17219	protein_coding	671.8078104	0.029504568	0.160091535	-0.184298115	0.853779593	0.928319475	0.029504568
ENSMUSG00000045822	Zswim3	67538	protein_coding	77.55914867	-0.02951894	0.136947711	0.215548984	0.829339326	0.913717368	0.02951894
ENSMUSG00000031901	Dus2	66369	protein_coding	89.2006143	0.029537336	0.135049616	-0.218714699	0.826872298	0.912640622	0.029537336
ENSMUSG00000032077	Bud13	215051	protein_coding	114.2296765	0.029551605	0.103389504	-0.285827905	0.775009934	0.887804402	0.029551605
ENSMUSG00000039968	Rsbn1l	242860	protein_coding	265.8045187	0.02966752	0.095849036	-0.309523399	0.756923415	0.878075079	0.02966752
ENSMUSG00000024537	Psmg2	107047	protein_coding	120.7250568	0.029686351	0.152162016	-0.195096985	0.845317023	0.923801556	0.029686351
ENSMUSG00000064647	Gm23301	115488997	snoRNA	6.45158088	-0.029695604	0.458539263	0.064761312	0.948364045	0.975173098	0.029695604
ENSMUSG00000041773	Enc1	13803	protein_coding	186.3338271	-0.029723132	0.118808433	0.250176953	0.802450508	0.902394803	0.029723132
ENSMUSG00000085502	Gm12320	102635935	lncRNA	34.77997628	0.02977079	0.242077625	-0.122980346	0.902122662	0.951530343	0.02977079
ENSMUSG00000037344	Slc12a9	83704	protein_coding	167.1876316	-0.029786827	0.122548624	0.243061294	0.807957915	0.905320736	0.029786827
ENSMUSG00000115902	D730005E14Rik	109361	lncRNA	28.75777974	0.029852925	0.345290851	-0.086457331	0.931102874	0.966840628	0.029852925
ENSMUSG00000020590	Snx13	217463	protein_coding	1631.237816	0.02985685	0.067605036	-0.441636477	0.658752284	0.815856848	0.02985685
ENSMUSG00000022765	Snap29	67474	protein_coding	453.0733939	0.029869816	0.081626178	-0.365934267	0.714414126	0.851829233	0.029869816
ENSMUSG00000006390	Elovl1	54325	protein_coding	717.9877106	-0.029968959	0.071417772	0.41962887	0.674756595	0.825585716	0.029968959

ensembl_gene_id	external_gene_name	entrezgene_id	gene_biotype	baseMean	log2FoldChange	lfcSE	stat	pvalue	padj	absLog2FC
ENSMUSG00000069539	Scyl2	213326	protein_coding	810.6403533	0.029975812	0.051833343	-0.578311375	0.563053916	0.750768863	0.029975812
ENSMUSG00000070720	Tmem200b	623230	protein_coding	4.519332246	0.029986143	0.613033403	-0.048914371	0.960987536	0.981006872	0.029986143
ENSMUSG00000026927	Entr1	68112	protein_coding	412.2007474	0.029986556	0.073382617	-0.408632967	0.682809034	0.830576634	0.029986556
ENSMUSG00000037085	Trmt12	68260	protein_coding	82.62532241	-0.029994987	0.155629647	0.192733114	0.847167988	0.924722861	0.029994987
ENSMUSG00000048652	Samd13	75015	protein_coding	10.51041307	-0.030014434	0.339839092	0.088319544	0.929622706	0.966128972	0.030014434
ENSMUSG00000034709	Ppp1r21	73825	protein_coding	307.1307181	0.030052218	0.070916396	-0.423769674	0.671733796	0.823993339	0.030052218
ENSMUSG00000097119	B230354K17Rik	105246278	lncRNA	144.1508011	0.030054566	0.125989815	-0.238547588	0.81145641	0.906704483	0.030054566
ENSMUSG00000051469	Zfp24	59057	protein_coding	451.040638	0.030060759	0.095321434	-0.315362006	0.752486811	0.875793979	0.030060759
ENSMUSG00000039981	Zc3h12d	237256	protein_coding	223.0869687	0.030091192	0.130803872	-0.230048173	0.818054337	0.908812719	0.030091192
ENSMUSG00000032363	Adamts7	108153	protein_coding	8.747976147	0.030091325	0.516192896	-0.058294729	0.953513866	0.97686351	0.030091325
ENSMUSG00000020547	Bzw2	66912	protein_coding	549.3890021	-0.030096289	0.113716477	0.26466076	0.791270809	0.89588517	0.030096289
ENSMUSG00000008976	Gabpa	14390	protein_coding	538.1918428	0.030113369	0.119166158	-0.252700681	0.800499522	0.901189824	0.030113369
ENSMUSG00000015757	Ppil4	67418	protein_coding	354.3280749	0.030140672	0.077557637	-0.388622872	0.697555145	0.839669485	0.030140672
ENSMUSG00000047539	Fbxo28	67948	protein_coding	383.5469783	0.030190042	0.093424549	-0.323148916	0.746582466	0.872440598	0.030190042
ENSMUSG00000058291	Zfp68	24135	protein_coding	356.004324	-0.030213846	0.118273752	0.255456904	0.798370225	0.899929685	0.030213846
ENSMUSG00000049122	Frmd3	242506	protein_coding	18.92785007	0.030251006	0.354394523	-0.085359688	0.931975441	0.967478151	0.030251006
ENSMUSG00000026321	Tnfrsf11a	21934	protein_coding	1859.811047	0.030256876	0.102934652	-0.293942568	0.768801789	0.884245212	0.030256876
ENSMUSG00000048012	Zfp473	243963	protein_coding	24.92353343	0.030316821	0.263046847	-0.115252554	0.908244944	0.955333816	0.030316821
ENSMUSG00000024217	Snrpc	20630	protein_coding	243.9712253	-0.030330513	0.126186781	0.240362048	0.810049597	0.906149826	0.030330513
ENSMUSG00000027782	Kpna4	16649	protein_coding	838.1856728	-0.030340025	0.055750275	0.544213017	0.586294895	0.767930988	0.030340025
ENSMUSG00000035666	Gtf3c4	269252	protein_coding	424.1029059	-0.030341239	0.082649357	0.367107984	0.713538472	0.851092403	0.030341239
ENSMUSG00000018572	Phf23	78246	protein_coding	377.3954941	-0.030370139	0.094078699	0.322816312	0.746834359	0.872440598	0.030370139
ENSMUSG00000015766	Eps8	13860	protein_coding	1993.207767	0.030376273	0.13255831	-0.229154044	0.81874919	0.908812719	0.030376273
ENSMUSG00000038319	Kcnh2	16511	protein_coding	39.1016121	-0.03043773	0.233922232	0.130119012	0.896472269	0.948996349	0.03043773
ENSMUSG00000049960	Mrps16	66242	protein_coding	420.0186482	-0.030440229	0.08200522	0.371198669	0.710489565	0.84898876	0.030440229
ENSMUSG00000032860	P2ry2	18442	protein_coding	213.9799813	-0.030485525	0.193862988	0.15725294	0.875045507	0.938415991	0.030485525
ENSMUSG00000074657	Kif5a	16572	protein_coding	6.316081593	0.030517113	1.150130162	-0.026533616	0.978831721	0.988831409	0.030517113
ENSMUSG00000006276	Eps15l1	13859	protein_coding	627.7380668	-0.030542375	0.060279674	0.506677843	0.612380879	0.785464687	0.030542375
ENSMUSG00000014668	Chfr	231600	protein_coding	644.2019802	0.030547792	0.066093975	-0.462187232	0.643947058	0.80597323	0.030547792
ENSMUSG00000031072	LTO1	72284	protein_coding	176.5798583	0.030563314	0.086304667	-0.354132805	0.723239358	0.856549354	0.030563314
ENSMUSG00000047583	Tyw3	209584	protein_coding	29.60763766	-0.030563584	0.248654533	0.122915851	0.902173733	0.951530343	0.030563584
ENSMUSG00000025795	Rassf3	192678	protein_coding	729.7293615	-0.03059208	0.079271938	0.385913113	0.699561018	0.84101582	0.03059208
ENSMUSG00000037461	Ints7	77065	protein_coding	423.7242079	0.030598111	0.100248873	-0.305221498	0.76019746	0.879513202	0.030598111
ENSMUSG00000035206	Sppl2b	73218	protein_coding	481.4058302	0.030604855	0.0649409	-0.471272411	0.637446215	0.802408631	0.030604855
ENSMUSG00000025702	Marchf8	71779	protein_coding	1468.52362	-0.030633386	0.074574551	0.410775334	0.681237282	0.829503357	0.030633386
ENSMUSG00000031565	Fgfr1	14182	protein_coding	10.23204615	-0.030644073	0.895245058	0.034229815	0.972693891	0.985766611	0.030644073
ENSMUSG00000031021	Tmem9b	56786	protein_coding	648.4689245	-0.030690095	0.093992959	0.326514825	0.744034875	0.870923726	0.030690095
ENSMUSG00000037426	Depdc5	277854	protein_coding	325.8445273	-0.03071494	0.076933752	0.399238818	0.689717243	0.834370416	0.03071494
ENSMUSG00000014074	Rnf168	70238	protein_coding	329.6890739	-0.030738698	0.111402539	0.275924569	0.782606003	0.891061468	0.030738698

ensembl_gene_id	external_gene_name	entrezgene_id	gene_biotype	baseMean	log2FoldChange	lfcSE	stat	pvalue	padj	absLog2FC
ENSMUSG00000035851	Ythdc1	231386	protein_coding	657.4636788	0.030777187	0.075409544	-0.408133835	0.683175421	0.830769833	0.030777187
ENSMUSG00000032580	Rbm5	83486	protein_coding	1163.622775	-0.030779711	0.060228042	0.511052835	0.609314061	0.783356356	0.030779711
ENSMUSG00000020440	Arf5	11844	protein_coding	1168.90788	0.030801041	0.074248233	-0.414838705	0.678259983	0.82760347	0.030801041
ENSMUSG00000021993	Mipep	70478	protein_coding	311.765382	0.030843846	0.09588494	-0.321675609	0.747698458	0.873111243	0.030843846
ENSMUSG00000029196	Tada2b	231151	protein_coding	201.6438262	0.030902571	0.102098009	-0.302675546	0.762137135	0.880874346	0.030902571
ENSMUSG00000028664	Ephb2	13844	protein_coding	1261.735467	-0.030921328	0.098717227	0.313231326	0.754104921	0.876856731	0.030921328
ENSMUSG00000041261	Car8	12319	protein_coding	697.4405106	-0.030938537	0.169547481	0.182477122	0.855208307	0.929505724	0.030938537
ENSMUSG00000037646	Vps13b	666173	protein_coding	1640.295709	-0.0309433	0.074689308	0.414293564	0.678659127	0.827845828	0.0309433
ENSMUSG00000021279	Cdc42bpb	217866	protein_coding	1627.949956	-0.030960034	0.072867009	0.424884104	0.670921163	0.82339418	0.030960034
ENSMUSG00000026753	Ppp6c	67857	protein_coding	759.5604352	0.031004551	0.076332116	-0.406179637	0.684610615	0.831787794	0.031004551
ENSMUSG00000025759	Mfsd8	72175	protein_coding	163.5038478	0.031018764	0.112726915	-0.275167331	0.783187684	0.891264005	0.031018764
ENSMUSG00000051007	Gatd1	213350	protein_coding	342.7967197	-0.031031429	0.089189863	0.347925511	0.727896126	0.859382261	0.031031429
ENSMUSG00000034152	Exoc3	211446	protein_coding	848.442134	-0.031083932	0.072811995	0.42690675	0.669447249	0.822609935	0.031083932
ENSMUSG00000028010	Gar1	68147	protein_coding	99.46474271	0.03110629	0.131073026	-0.237320303	0.812408313	0.907147741	0.03110629
ENSMUSG00000009079	Ewsr1	14030	protein_coding	1908.382367	0.031107711	0.066229318	-0.469696982	0.638571525	0.80301103	0.031107711
ENSMUSG00000037643	Prkci	18759	protein_coding	1269.266364	-0.03111156	0.087555455	0.355335479	0.722338279	0.856331252	0.03111156
ENSMUSG00000024870	Rab1b	76308	protein_coding	1426.874461	0.03111494	0.059526397	-0.522708278	0.601177277	0.777615016	0.03111494
ENSMUSG00000079144	A130010J15Rik	319266	protein_coding	146.545141	0.031133076	0.117342095	-0.265318907	0.7907638	0.895656612	0.031133076
ENSMUSG00000036006	Ripor2	193385	protein_coding	29.42514533	0.031135071	0.222274334	-0.140074972	0.888600755	0.94517522	0.031135071
ENSMUSG00000078578	Ube2d3	66105	protein_coding	3164.330952	0.031144826	0.048966003	-0.636049998	0.524743835	0.723223689	0.031144826
ENSMUSG00000045205	Dpy19l4	381510	protein_coding	332.3742949	0.031145823	0.086129701	-0.361615366	0.717639481	0.853579543	0.031145823
ENSMUSG00000029510	Gpc2	71951	protein_coding	3.108183131	0.031159394	0.828036076	-0.037630478	0.969982307	1	0.031159394
ENSMUSG00000041238	Rbbp8	225182	protein_coding	398.9579886	0.031161954	0.07302705	-0.426717968	0.669584762	0.822609935	0.031161954
ENSMUSG00000031105	Slc25a14	20523	protein_coding	53.2358648	0.031180867	0.140347404	-0.222169174	0.82418219	0.910891761	0.031180867
ENSMUSG00000028581	Laptm5	16792	protein_coding	27.46849862	-0.031195684	0.388954414	0.080203959	0.936075041	0.969722303	0.031195684
ENSMUSG00000032855	Pkd1	18763	protein_coding	701.2962289	-0.031226144	0.100676886	0.310161999	0.756437767	0.877843331	0.031226144
ENSMUSG00000020513	Tubd1	56427	protein_coding	50.30012727	0.031248598	0.16817374	-0.1858114	0.852592665	0.927761944	0.031248598
ENSMUSG00000036644	Tbc1d9b	76795	protein_coding	908.365506	-0.031253524	0.070757989	0.441696041	0.658709175	0.815856848	0.031253524
ENSMUSG00000042505	Sdhaf3	71238	protein_coding	62.11396972	0.031255428	0.140463972	-0.22251562	0.823912515	0.91065466	0.031255428
ENSMUSG00000022672	Prkdc	19090	protein_coding	358.097704	-0.031276672	0.089460539	0.349614165	0.726628279	0.858643248	0.031276672
ENSMUSG00000054387	Mdm4	17248	protein_coding	745.2782543	-0.031294017	0.080406746	0.389196409	0.69713086	0.839403501	0.031294017
ENSMUSG00000034636	Zyg11b	414872	protein_coding	965.9856194	-0.031317745	0.067732098	0.462376716	0.643811194	0.805935268	0.031317745
ENSMUSG00000088015	Mir1932	100316690	miRNA	2.679904738	0.031321947	0.720716567	-0.043459451	0.965335287	1	0.031321947
ENSMUSG00000029405	G3bp2	23881	protein_coding	1610.178834	0.031325158	0.084369257	-0.371286402	0.710424225	0.848979902	0.031325158
ENSMUSG00000020848	Doc2b	13447	protein_coding	3.027158117	0.031356704	1.018547892	-0.030785694	0.97544045	1	0.031356704
ENSMUSG00000096887	Gm20594	100463512	protein_coding	3.622079905	-0.03136418	0.714690929	0.043884956	0.964996107	1	0.03136418
ENSMUSG00000097354	2310001H17Rik	76432	lncRNA	78.00279963	-0.031397545	0.182792879	0.171765693	0.863621738	0.932314788	0.031397545
ENSMUSG00000032563	Mrpl3	94062	protein_coding	385.0820845	0.031408059	0.077053308	-0.40761468	0.683556585	0.830935108	0.031408059
ENSMUSG00000007777	0610009B22Rik	66050	protein_coding	170.8093752	-0.031420495	0.111380025	0.282101709	0.777865523	0.888973789	0.031420495

ensembl_gene_id	external_gene_name	entrezgene_id	gene_biotype	baseMean	log2FoldChange	lfcSE	stat	pvalue	padj	absLog2FC
ENSMUSG00000038002	Cramp1	57354	protein_coding	492.6497258	0.031432357	0.149239286	-0.210617173	0.833186011	0.915993052	0.031432357
ENSMUSG00000050029	Rap2c	72065	protein_coding	498.4702012	0.031504161	0.073687595	-0.427536835	0.668988362	0.82238042	0.031504161
ENSMUSG00000037361	Sf3b6	66055	protein_coding	462.7243084	0.031574825	0.075355185	-0.419013303	0.67520641	0.825971257	0.031574825
ENSMUSG00000036955	Kifbp	72320	protein_coding	229.913825	0.031606639	0.102945905	-0.307021819	0.758826764	0.878912418	0.031606639
ENSMUSG00000055835	Zfp1	22640	protein_coding	65.26397877	0.031635832	0.131505341	-0.240566895	0.809890811	0.906073508	0.031635832
ENSMUSG00000035184	Fam124a	629059	protein_coding	101.0158815	-0.031657568	0.181300749	0.174613552	0.861383299	0.931679578	0.031657568
ENSMUSG00000005698	Ctcf	13018	protein_coding	895.236324	0.031658704	0.074667766	-0.423994257	0.671570002	0.823884333	0.031658704
ENSMUSG00000087424	5730405O15Rik	70489	lncRNA	8.99928649	-0.031712022	0.423850478	0.07481889	0.940358812	0.971417829	0.031712022
ENSMUSG00000039194	Rlbp1	19771	protein_coding	1.831823386	-0.031716168	1.371497675	0.023125207	0.981550399	1	0.031716168
ENSMUSG00000039910	Cited2	17684	protein_coding	451.8622993	0.031727834	0.189122753	-0.167763175	0.866769594	0.933837244	0.031727834
ENSMUSG00000113770	1700019C18Rik	75543	lncRNA	2.259578759	0.031802248	0.925610458	-0.034358133	0.972591568	1	0.031802248
ENSMUSG00000032231	Anxa2	12306	protein_coding	6546.765741	0.031829773	0.082452243	-0.386038898	0.699467861	0.840991064	0.031829773
ENSMUSG00000026315	Serpinb8	20725	protein_coding	5.549787561	-0.031833968	0.571365307	0.055715612	0.955568363	0.977832194	0.031833968
ENSMUSG00000087368	BC065397	436230	lncRNA	9.592484986	-0.031847266	0.372489053	0.085498528	0.931865065	0.967478151	0.031847266
ENSMUSG00000028214	Gem	14579	protein_coding	12.5434369	-0.03185669	0.519603248	0.061309644	0.95111261	0.97587882	0.03185669
ENSMUSG00000002346	Slc25a42	73095	protein_coding	87.8798524	-0.031893137	0.247077295	0.129081618	0.897293067	0.949500045	0.031893137
ENSMUSG00000027628	Aar2	68295	protein_coding	371.3809736	-0.031912379	0.072194474	0.442033538	0.658464937	0.815684492	0.031912379
ENSMUSG00000037355	Uvssa	71101	protein_coding	199.5829437	-0.03192507	0.105510679	0.302576673	0.762212494	0.880899792	0.03192507
ENSMUSG00000088901	Mir1943	100316695	miRNA	0.809390907	0.031959333	1.270066014	-0.025163521	0.979924533	1	0.031959333
ENSMUSG00000000202	Btbd17	72014	protein_coding	1.378714198	0.031969009	1.069382416	-0.029894834	0.976150926	1	0.031969009
ENSMUSG00000041915	Ammecr1l	225339	protein_coding	371.2493527	0.031971293	0.075797799	-0.421797111	0.673173115	0.824897384	0.031971293
ENSMUSG00000016520	Lnx2	140887	protein_coding	428.3493711	-0.031980142	0.06616644	0.483328733	0.628862341	0.797437818	0.031980142
ENSMUSG00000033902	Mapkbp1	26390	protein_coding	117.7782669	0.031982177	0.138033224	-0.23169912	0.816771715	0.908628099	0.031982177
ENSMUSG00000031392	Irak1	16179	protein_coding	548.3783236	-0.032007269	0.07681167	0.416698002	0.676899316	0.826943172	0.032007269
ENSMUSG00000001786	Fbxo7	69754	protein_coding	172.085621	0.032008134	0.113390379	-0.282282625	0.777726807	0.888933284	0.032008134
ENSMUSG00000044167	Foxo1	56458	protein_coding	200.1939317	-0.032026609	0.129877254	0.246591359	0.805224492	0.904098264	0.032026609
ENSMUSG00000003345	Csnk1g2	103236	protein_coding	1319.643898	0.032045671	0.058786987	-0.545115032	0.585674405	0.767483504	0.032045671
ENSMUSG00000024395	Lims2	225341	protein_coding	6.667299217	-0.032061663	0.68878766	0.046547964	0.962873506	0.981615003	0.032061663
ENSMUSG00000026432	Avpr1b	26361	protein_coding	7.865342352	0.032076551	0.447678293	-0.071650896	0.942879735	0.972316965	0.032076551
ENSMUSG00000064941	Gm23238	115489085	snRNA	1.325466561	-0.032102841	1.123883289	0.028564212	0.977212155	1	0.032102841
ENSMUSG00000003458	Ncstn	59287	protein_coding	1544.545156	-0.032104959	0.070750957	0.4537742	0.649991355	0.809917796	0.032104959
ENSMUSG00000046722	Cdc42se1	57912	protein_coding	1087.608143	0.032125114	0.09574914	-0.335513344	0.73723788	0.866653357	0.032125114
ENSMUSG00000054263	Lifr	16880	protein_coding	63.02343558	0.032126577	0.264961534	-0.121249966	0.903493049	0.952286247	0.032126577
ENSMUSG00000035047	Kri1	215194	protein_coding	148.6716752	0.032178704	0.117111403	-0.274770035	0.783492921	0.891333845	0.032178704
ENSMUSG00000099083	Atf7	223922	protein_coding	162.6735136	0.03221506	0.127869976	-0.251936076	0.801090474	0.901479257	0.03221506
ENSMUSG00000032657	Entrep3	68521	protein_coding	24.35371786	0.032215498	0.35179179	-0.091575469	0.927035343	0.965151432	0.032215498
ENSMUSG00000022602	Arc	11838	protein_coding	416.6671842	-0.032224572	0.406309488	0.079310409	0.936785727	0.969910941	0.032224572
ENSMUSG00000035112	Wnk4	69847	protein_coding	262.6549	0.032236575	0.165984042	-0.194214906	0.846007612	0.924189304	0.032236575
ENSMUSG00000039183	Nubp2	26426	protein_coding	265.5870797	-0.032246658	0.081731729	0.394542716	0.693180397	0.837078181	0.032246658

ensembl_gene_id	external_gene_name	entrezgene_id	gene_biotype	baseMean	log2FoldChange	lfcSE	stat	pvalue	padj	absLog2FC
ENSMUSG00000036450	Hif1an	319594	protein_coding	884.8527848	-0.032299062	0.065298261	0.494638933	0.620855015	0.791721985	0.032299062
ENSMUSG00000031065	Cdk16	18555	protein_coding	1454.814391	0.032310606	0.051358389	-0.629120316	0.529270291	0.727020313	0.032310606
ENSMUSG00000024533	Spire1	68166	protein_coding	39.99318008	-0.032360539	0.219026665	0.147747027	0.882542417	0.941973978	0.032360539
ENSMUSG00000037519	Ppfia1	233977	protein_coding	884.7975857	0.032373197	0.072440395	-0.446894258	0.65495141	0.812734264	0.032373197
ENSMUSG00000015733	Capza2	12343	protein_coding	1697.129156	-0.032480797	0.055884549	0.581212479	0.561097258	0.749612501	0.032480797
ENSMUSG00000074218	Cox7a1	12865	protein_coding	460.943088	-0.032485062	0.12674568	0.256301139	0.797718318	0.899646268	0.032485062
ENSMUSG00000003604	Aven	74268	protein_coding	268.1686161	0.032502468	0.08479752	-0.383295031	0.701501023	0.842293038	0.032502468
ENSMUSG00000040731	Eif4h	22384	protein_coding	3323.63161	-0.032504999	0.057543229	0.564879649	0.572155627	0.757643976	0.032504999
ENSMUSG00000050428	Fbxo46	243867	protein_coding	231.2410091	-0.032511182	0.072683143	0.447300169	0.654658345	0.812675931	0.032511182
ENSMUSG00000026585	Kifap3	16579	protein_coding	217.5500837	0.032579899	0.101673791	-0.320435567	0.748638167	0.873772754	0.032579899
ENSMUSG00000034796	Cpne7	102278	protein_coding	3.303019233	-0.032641091	1.252984267	0.026050679	0.979216916	1	0.032641091
ENSMUSG00000037523	Mavs	228607	protein_coding	610.7260518	0.032657937	0.098816771	-0.330489815	0.741029887	0.868956809	0.032657937
ENSMUSG00000064427	Gm22748	115488064	snoRNA	101.1570093	0.032662317	0.210144656	-0.155427776	0.876484088	0.938630585	0.032662317
ENSMUSG00000031819	Emc8	18117	protein_coding	233.9503345	0.032667027	0.091306695	-0.357772523	0.720513555	0.855204229	0.032667027
ENSMUSG00000028629	Exo5	73172	protein_coding	143.5116503	-0.032683113	0.090356908	0.361711279	0.717567798	0.853563561	0.032683113
ENSMUSG00000034254	Agpat1	55979	protein_coding	1309.009409	0.032698415	0.079070601	-0.413534421	0.679215109	0.827973596	0.032698415
ENSMUSG00000027650	Tti1	75425	protein_coding	217.4487858	-0.032707053	0.123593339	0.264634433	0.791291092	0.89588517	0.032707053
ENSMUSG00000029050	Ski	20481	protein_coding	788.3764415	-0.0327459	0.06998082	0.467926783	0.639836949	0.803640659	0.0327459
ENSMUSG00000047547	Cltb	74325	protein_coding	1780.800572	0.032747195	0.052280189	-0.626378661	0.531066603	0.728048657	0.032747195
ENSMUSG00000043733	Ptpn11	19247	protein_coding	1069.936852	0.032754707	0.071415466	-0.458650051	0.646485488	0.807324664	0.032754707
ENSMUSG00000018923	Med11	66172	protein_coding	144.8556266	-0.032759723	0.113704754	0.28811217	0.773260877	0.886362513	0.032759723
ENSMUSG00000011958	Bnip2	12175	protein_coding	158.8131653	0.032786664	0.126428858	-0.259328961	0.795381436	0.898299072	0.032786664
ENSMUSG00000039048	Foxred1	235169	protein_coding	372.3471462	-0.032794367	0.077821982	0.421402357	0.673461298	0.824917659	0.032794367
ENSMUSG00000039840	Epg5	100502841	protein_coding	552.6386319	0.032887557	0.083930058	-0.391844805	0.695172887	0.838326567	0.032887557
ENSMUSG00000021149	Gtpbp4	69237	protein_coding	398.9262663	-0.032896589	0.105235309	0.312600295	0.754584354	0.876856731	0.032896589
ENSMUSG00000051166	Eml5	319670	protein_coding	25.97901467	0.032921365	0.317591887	-0.103659338	0.917439697	0.959505963	0.032921365
ENSMUSG00000037579	Kcnh3	16512	protein_coding	257.1305457	-0.032926179	0.145147649	0.226846105	0.820543412	0.9092783	0.032926179
ENSMUSG00000075486	Commd6	66200	protein_coding	301.987055	-0.032985197	0.076894594	0.428966406	0.667947673	0.821576632	0.032985197
ENSMUSG00000048078	Tenm4	23966	protein_coding	156.7247463	-0.033034674	0.156685861	0.210833792	0.833016969	0.915935963	0.033034674
ENSMUSG00000054517	Trim65	338364	protein_coding	177.7001396	-0.033056967	0.114949302	0.287578666	0.773669277	0.88672609	0.033056967
ENSMUSG00000030382	Slc27a5	26459	protein_coding	1.122127694	-0.033061762	2.540284497	0.013014984	0.989615838	1	0.033061762
ENSMUSG00000038467	Chmp4b	75608	protein_coding	2798.384126	-0.0330623	0.066950222	0.493834058	0.621423378	0.792202074	0.0330623
ENSMUSG00000030546	Plin1	103968	protein_coding	2.24861595	-0.0330824	2.135404097	0.015492337	0.987639398	1	0.0330824
ENSMUSG00000037710	Cisd1	52637	protein_coding	807.0651137	-0.033098591	0.092141833	0.359213505	0.719435374	0.854853631	0.033098591
ENSMUSG00000032246	Calml4	75600	protein_coding	1344.489793	-0.033145132	0.080561177	0.411428098	0.680758654	0.829059617	0.033145132
ENSMUSG00000086392	Mccc1os	654424	lncRNA	3.296867628	0.033160601	0.771177879	-0.042999938	0.965701583	1	0.033160601
ENSMUSG00000019782	Rwdd1	66521	protein_coding	164.2804645	-0.033163124	0.166681435	0.198961117	0.84229316	0.921900136	0.033163124
ENSMUSG00000007415	Gatad1	67210	protein_coding	717.6710778	-0.03316914	0.069812834	0.475115218	0.634704852	0.800822518	0.03316914
ENSMUSG00000050786	Ccdc126	57895	protein_coding	32.45863937	0.033173133	0.213121595	-0.155653552	0.876306111	0.938630585	0.033173133

ensembl_gene_id	external_gene_name	entrezgene_id	gene_biotype	baseMean	log2FoldChange	lfcSE	stat	pvalue	padj	absLog2FC
ENSMUSG00000078905	Gm14393	664987	protein_coding	1.469785856	0.03317613	1.154726783	-0.028730718	0.977079357	1	0.03317613
ENSMUSG00000025612	Bach1	12013	protein_coding	859.3836958	0.03317724	0.095060328	-0.349012471	0.727079947	0.858992577	0.03317724
ENSMUSG00000004393	Ddx56	52513	protein_coding	326.8369824	-0.033205966	0.107686183	0.308358652	0.757809441	0.87863969	0.033205966
ENSMUSG00000032097	Ddx6	13209	protein_coding	1922.566536	-0.033206768	0.091414417	0.363255268	0.716414205	0.852797822	0.033206768
ENSMUSG00000001910	Nacc1	66830	protein_coding	1241.63056	-0.033276826	0.065758911	0.506042833	0.612826581	0.785880315	0.033276826
ENSMUSG00000043190	Rfesd	218341	protein_coding	95.67391462	-0.033297298	0.153012378	0.217611792	0.827731596	0.913225519	0.033297298
ENSMUSG00000042532	Golga7b	71146	protein_coding	3.172997258	-0.033320622	0.613868866	0.054279707	0.956712317	1	0.033320622
ENSMUSG00000027394	Ttl	69737	protein_coding	95.49664183	-0.033327432	0.122439747	0.272194552	0.785472431	0.892918574	0.033327432
ENSMUSG00000041187	Prkd2	101540	protein_coding	209.3480263	-0.033332311	0.12027947	0.277123857	0.781685004	0.890621398	0.033332311
ENSMUSG00000020228	Helb	117599	protein_coding	335.2702203	0.033353674	0.090611483	-0.368095443	0.712802069	0.850705598	0.033353674
ENSMUSG00000059119	Nap1l4	17955	protein_coding	838.2488502	-0.033367028	0.084761527	0.393657703	0.693833773	0.837509753	0.033367028
ENSMUSG00000057969	Sema3b	20347	protein_coding	1051.546386	-0.033399405	0.069166466	0.482884369	0.629177841	0.797446101	0.033399405
ENSMUSG00000052031	Tagap1	380608	protein_coding	81.4237217	-0.033428158	0.154686205	0.216103034	0.828907435	0.913504709	0.033428158
ENSMUSG00000031921	Terf2	21750	protein_coding	186.6295612	0.033437942	0.084905603	-0.393824924	0.693710302	0.837421924	0.033437942
ENSMUSG00000046441	Cmtr2	234728	protein_coding	131.8845981	0.033451135	0.107059297	-0.312454277	0.754695306	0.876856731	0.033451135
ENSMUSG00000034785	Dio1	13370	protein_coding	682.3516825	-0.033457262	0.107224522	0.312029947	0.755017765	0.877003824	0.033457262
ENSMUSG00000067206	Lrrc66	231296	protein_coding	826.7385403	0.033463953	0.095802993	-0.349299658	0.726864355	0.85879931	0.033463953
ENSMUSG00000030339	Ltbr	17000	protein_coding	1392.656195	0.033481874	0.074521862	-0.449289289	0.653222984	0.811870577	0.033481874
ENSMUSG00000079507	H2-Q1	15006	protein_coding	214.5147542	-0.033527393	0.215476202	0.155596733	0.8763509	0.938630585	0.033527393
ENSMUSG00000040918	Slc19a2	116914	protein_coding	111.0741671	-0.033532372	0.124407416	0.269536763	0.787516656	0.893615254	0.033532372
ENSMUSG00000042284	Itga1	109700	protein_coding	189.760487	0.03353828	0.139820714	-0.239866318	0.810433893	0.906179443	0.03353828
ENSMUSG00000027843	Ptpn22	19260	protein_coding	14.97932535	0.033538347	0.42515085	-0.078885757	0.937123492	0.970043107	0.033538347
ENSMUSG00000031198	Fundc2	67391	protein_coding	183.8132229	0.033539784	0.136224434	-0.246209751	0.805519868	0.904245469	0.033539784
ENSMUSG00000046671	Mtfr1l	76824	protein_coding	458.1322023	-0.033594858	0.076510819	0.43908637	0.660598955	0.81673513	0.033594858
ENSMUSG00000022867	Usp25	30940	protein_coding	830.0848764	-0.033619745	0.058598968	0.573725887	0.566153308	0.752653012	0.033619745
ENSMUSG00000098912	1500004A13Rik	319830	lncRNA	26.65056609	0.033644247	0.225053326	-0.149494557	0.881163404	0.941406719	0.033644247
ENSMUSG00000028333	Anp32b	67628	protein_coding	375.038587	0.03366714	0.114273258	-0.294619586	0.768284498	0.883975099	0.03366714
ENSMUSG00000042182	Bend6	320705	protein_coding	1.412359531	-0.033668619	1.751520807	0.019222506	0.984663604	1	0.033668619
ENSMUSG00000002319	Ipo4	75751	protein_coding	134.3401761	0.033673705	0.130014631	-0.258999348	0.795635743	0.898463393	0.033673705
ENSMUSG00000029221	Slc30a9	109108	protein_coding	753.9144217	-0.033754858	0.071607097	0.471389836	0.637362373	0.802408631	0.033754858
ENSMUSG00000022992	Kansl2	69612	protein_coding	199.9678977	-0.033778157	0.116526278	0.289875881	0.771911194	0.885478012	0.033778157
ENSMUSG00000039461	Tcta	102791	protein_coding	423.9356025	0.033813907	0.086198109	-0.39228131	0.694850372	0.838124245	0.033813907
ENSMUSG00000057177	Gsk3a	606496	protein_coding	421.2772019	0.033844575	0.063088687	-0.536460283	0.591640478	0.771785126	0.033844575
ENSMUSG00000030868	Dctn5	59288	protein_coding	455.2974559	0.033870392	0.073419037	-0.461329837	0.644561981	0.806325018	0.033870392
ENSMUSG00000024513	Mbd2	17191	protein_coding	1295.339469	-0.033877873	0.059946308	0.565136939	0.571980625	0.757533728	0.033877873
ENSMUSG00000026614	Slc30a10	226781	protein_coding	1208.93023	-0.033882558	0.221717805	0.152818388	0.878541496	0.939901695	0.033882558
ENSMUSG00000022471	Xrcc6	14375	protein_coding	297.1039015	-0.03396037	0.11568634	0.293555577	0.769097525	0.884277416	0.03396037
ENSMUSG00000025571	Tnrc6c	217351	protein_coding	595.3098105	0.033991502	0.070816349	-0.479995117	0.631230865	0.799065123	0.033991502
ENSMUSG00000038861	Pi4kb	107650	protein_coding	542.8230119	-0.033993763	0.048165928	0.705763684	0.48033511	0.691324212	0.033993763

ensembl_gene_id	external_gene_name	entrezgene_id	gene_biotype	baseMean	log2FoldChange	lfcSE	stat	pvalue	padj	absLog2FC
ENSMUSG00000041945	Mfsd9	211798	protein_coding	134.9284141	-0.034002693	0.11612933	0.292800218	0.769674862	0.884510137	0.034002693
ENSMUSG00000031813	Mvb12a	73711	protein_coding	751.2837275	-0.034008544	0.073794653	0.460853776	0.644903517	0.80663006	0.034008544
ENSMUSG00000029687	Ezh2	14056	protein_coding	365.1070791	-0.034010117	0.095791787	0.355042101	0.722558051	0.856468824	0.034010117
ENSMUSG00000060149	BC002059	213811	lncRNA	68.94001385	-0.034045883	0.133967992	0.25413446	0.799391682	0.90054944	0.034045883
ENSMUSG00000030757	Zkscan2	210162	protein_coding	2.228054327	0.034076653	2.429485077	-0.014026286	0.98880901	1	0.034076653
ENSMUSG00000010914	Pdhx	27402	protein_coding	355.5053074	0.034098736	0.073282568	-0.465304869	0.641713149	0.804528617	0.034098736
ENSMUSG00000054364	Rhob	11852	protein_coding	5073.205047	0.034109265	0.137437835	-0.248179589	0.803995458	0.903393958	0.034109265
ENSMUSG00000029730	Mcm7	17220	protein_coding	496.7902965	0.034120856	0.170874356	-0.199683891	0.841727813	0.921642147	0.034120856
ENSMUSG00000078622	Ccdc47	67163	protein_coding	955.8667786	-0.034138204	0.09192258	0.371379962	0.710354548	0.848979902	0.034138204
ENSMUSG00000100594	2810414N06Rik	102640772	lncRNA	11.23564056	0.034145185	0.310287294	-0.110043775	0.912374658	0.957266079	0.034145185
ENSMUSG00000018567	Gabarap	56486	protein_coding	2382.832443	-0.03418287	0.046161838	0.740500631	0.458996278	0.673388737	0.03418287
ENSMUSG00000049969	Plekhf2	71801	protein_coding	843.9126404	-0.034196606	0.053867963	0.634822691	0.525544063	0.724020576	0.034196606
ENSMUSG00000062397	Zfp706	68036	protein_coding	1299.842648	-0.034277726	0.077184172	0.444103047	0.656968084	0.814465699	0.034277726
ENSMUSG00000038780	Smurf1	75788	protein_coding	410.4652388	0.034321688	0.090856895	-0.377755458	0.705612266	0.845753813	0.034321688
ENSMUSG00000093081	Mir3075	100526533	miRNA	0.587822831	-0.034383874	1.586981276	0.021666213	0.982714216	1	0.034383874
ENSMUSG00000035790	Cep19	66994	protein_coding	277.1533057	0.034436265	0.08190763	-0.420428045	0.674172784	0.825194181	0.034436265
ENSMUSG00000024867	Pip5k1b	18719	protein_coding	804.234879	-0.034437815	0.067587449	0.509529737	0.61038096	0.784117555	0.034437815
ENSMUSG00000035954	Dock4	238130	protein_coding	23.78515086	-0.03444417	0.321169017	0.107246242	0.914593631	0.958199105	0.03444417
ENSMUSG00000034028	Cd226	225825	protein_coding	7.328228876	-0.034457624	0.518054365	0.066513529	0.946968987	0.974163449	0.034457624
ENSMUSG00000034931	Dhx8	217207	protein_coding	608.8919922	0.034514563	0.066784492	-0.516805056	0.605292251	0.780311961	0.034514563
ENSMUSG00000020064	Herc4	67345	protein_coding	649.7763553	0.034548751	0.081065712	-0.426182043	0.669975201	0.822722373	0.034548751
ENSMUSG00000029474	Rnf34	80751	protein_coding	272.0260329	-0.034561919	0.106154821	0.325580308	0.744741913	0.8712288	0.034561919
ENSMUSG00000034341	Wbp2	22378	protein_coding	656.9308231	0.034608522	0.058125348	-0.595411875	0.55156819	0.742355007	0.034608522
ENSMUSG00000036572	Upf3b	68134	protein_coding	204.8191292	0.034732584	0.082214158	-0.422464757	0.67268582	0.824518194	0.034732584
ENSMUSG00000046519	Golph3l	229593	protein_coding	4145.26386	-0.03483414	0.154086962	0.226068056	0.821148489	0.909608677	0.03483414
ENSMUSG00000053436	Mapk14	26416	protein_coding	765.9905329	-0.034897639	0.071980634	0.484819829	0.627804156	0.796376973	0.034897639
ENSMUSG00000065258	Gm23969	115489598	snoRNA	7.174507202	-0.034905137	0.465615394	0.074965599	0.940242083	0.971417829	0.034905137
ENSMUSG00000056216	Cebpg	12611	protein_coding	1061.817003	0.0349375	0.071764295	-0.486836809	0.626373983	0.795381772	0.0349375
ENSMUSG00000083649	Rasl2-9	19428	protein_coding	6.436345765	-0.03504077	0.475710127	0.073659921	0.94128099	0.971762583	0.03504077
ENSMUSG00000040701	Ap1g2	11766	protein_coding	514.8732753	-0.035065235	0.073887731	0.474574526	0.635090267	0.801105392	0.035065235
ENSMUSG00000038876	Rnf146	68031	protein_coding	143.0146489	0.035068555	0.112457226	-0.311839052	0.755162843	0.877032235	0.035068555
ENSMUSG00000063889	Crem	12916	protein_coding	53.10885537	0.035102353	0.174315743	-0.201372246	0.840407515	0.920873844	0.035102353
ENSMUSG00000023170	Gps2	56310	protein_coding	269.8012375	-0.035109582	0.073151638	0.479956192	0.631258544	0.799065123	0.035109582
ENSMUSG00000070417	Or6a2	18317	protein_coding	2.968386822	0.035137418	2.453842009	-0.014319348	0.988575204	1	0.035137418
ENSMUSG00000034435	Tmem30b	238257	protein_coding	2877.910642	0.035151261	0.093407234	-0.376322686	0.706677016	0.846208704	0.035151261
ENSMUSG00000059495	Arhgef12	69632	protein_coding	1102.365509	-0.035164637	0.055964187	0.62834177	0.529780074	0.72724756	0.035164637
ENSMUSG00000005897	Nr2c1	22025	protein_coding	146.899595	0.035216372	0.091963994	-0.382936526	0.701766828	0.842528346	0.035216372
ENSMUSG00000047819	Tigd4	403175	protein_coding	4.274011072	-0.035227161	0.672703615	0.052366541	0.958236634	1	0.035227161
ENSMUSG00000024515	Smad4	17128	protein_coding	1274.892756	0.035295516	0.127828697	-0.276115743	0.782459169	0.890955714	0.035295516

ensembl_gene_id	external_gene_name	entrezgene_id	gene_biotype	baseMean	log2FoldChange	lfcSE	stat	pvalue	padj	absLog2FC
ENSMUSG00000039159	Ube2h	22214	protein_coding	746.2375354	0.035295936	0.082230037	-0.429234095	0.667752873	0.821398208	0.035295936
ENSMUSG00000029701	Rbm28	68272	protein_coding	352.2724992	0.035330781	0.075357727	-0.468840845	0.639183402	0.803282676	0.035330781
ENSMUSG00000028327	Stra6l	74152	protein_coding	269.6177593	0.035353514	0.117485024	-0.300919319	0.763476018	0.881386379	0.035353514
ENSMUSG00000041846	Ppp4r3a	68734	protein_coding	1067.591956	0.035353576	0.071245518	-0.496221764	0.619737958	0.790880748	0.035353576
ENSMUSG00000025102	3110040N11Rik	67290	protein_coding	142.2909643	-0.035405226	0.118256748	0.299392857	0.764640312	0.882132403	0.035405226
ENSMUSG00000024382	Ercc3	13872	protein_coding	421.829526	0.035434447	0.07706492	-0.459799953	0.645659817	0.806964824	0.035434447
ENSMUSG00000026484	Rnf2	19821	protein_coding	288.2043309	-0.03544895	0.088318603	0.401375803	0.688143462	0.833786242	0.03544895
ENSMUSG00000017776	Crk	12928	protein_coding	2274.035314	0.035483488	0.07084483	-0.50086207	0.616468199	0.788502504	0.035483488
ENSMUSG00000040479	Dgkz	104418	protein_coding	1918.499743	0.035503631	0.056311598	-0.630485243	0.528377155	0.726359126	0.035503631
ENSMUSG00000036721	Zscan12	22758	protein_coding	74.02585578	-0.035544558	0.189226891	0.187840944	0.851001338	0.927281893	0.035544558
ENSMUSG00000026095	Asnsd1	70396	protein_coding	100.7332503	0.035551362	0.131947407	-0.269435856	0.787594297	0.893615254	0.035551362
ENSMUSG00000073599	Ecscr	68545	protein_coding	40.73092259	0.035553138	0.199330128	-0.178363094	0.858437831	0.930749284	0.035553138
ENSMUSG00000020128	Vps54	245944	protein_coding	1246.139095	-0.035569578	0.079129407	0.449511503	0.653062712	0.811818066	0.035569578
ENSMUSG00000015120	Ube2i	22196	protein_coding	599.3902778	0.035613905	0.092391062	-0.385469161	0.69988985	0.841086565	0.035613905
ENSMUSG00000020246	Hcfc2	67933	protein_coding	213.0286832	0.035689795	0.097529488	-0.365938508	0.714410961	0.851829233	0.035689795
ENSMUSG00000030134	Rasgef1a	70727	protein_coding	4.188339838	0.03571214	0.643904351	-0.055461871	0.955770506	1	0.03571214
ENSMUSG00000032217	Rnf111	93836	protein_coding	467.8855333	0.035742675	0.064565806	-0.553585206	0.579862753	0.763259485	0.035742675
ENSMUSG00000015957	Wnt11	22411	protein_coding	14.70304944	-0.035745174	0.400616269	0.089225468	0.928902725	0.966049475	0.035745174
ENSMUSG00000039789	Zfp597	71063	protein_coding	123.2774803	-0.03575064	0.140149774	0.255088816	0.798654502	0.900025962	0.03575064
ENSMUSG00000025787	Tgm4	331046	protein_coding	6.017114624	-0.035782645	0.408129128	0.087674813	0.930135139	0.966352899	0.035782645
ENSMUSG00000021585	Cast	12380	protein_coding	1835.944952	-0.03592669	0.094859085	0.378737474	0.704882822	0.845163358	0.03592669
ENSMUSG00000049792	Bag5	70369	protein_coding	187.7407246	-0.035940248	0.114381354	0.314214223	0.753358341	0.876432521	0.035940248
ENSMUSG00000021712	Trim23	81003	protein_coding	132.9894365	0.035948594	0.103445049	-0.347513917	0.728205265	0.859565127	0.035948594
ENSMUSG00000097047	1110020A21Rik	68531	lncRNA	20.51568029	0.035965804	0.256822596	-0.140041433	0.888627254	0.94517522	0.035965804
ENSMUSG00000070923	Klhl9	242521	protein_coding	1306.506231	0.035973012	0.078470556	-0.458426876	0.646645787	0.807443873	0.035973012
ENSMUSG00000020464	Pnpt1	71701	protein_coding	461.9427711	0.035973597	0.103653694	-0.347055616	0.728549536	0.859744679	0.035973597
ENSMUSG00000024695	Zfp91	109910	protein_coding	986.2411488	0.035989039	0.111692059	-0.322216629	0.747288587	0.872752739	0.035989039
ENSMUSG00000022698	Naa50	72117	protein_coding	915.9558108	-0.035989854	0.104148504	0.345562851	0.729671269	0.860514985	0.035989854
ENSMUSG00000074300	Smim31	407795	protein_coding	1243.82924	-0.036046764	0.14943918	0.24121361	0.809389563	0.905818599	0.036046764
ENSMUSG00000002325	Irf9	16391	protein_coding	400.6960847	0.036091197	0.153202996	-0.235577618	0.813760443	0.907696046	0.036091197
ENSMUSG00000093805	Gal3st2b	100041596	protein_coding	92.00109841	0.036110292	0.469117672	-0.076974913	0.938643503	0.970660753	0.036110292
ENSMUSG00000030983	Bccip	66165	protein_coding	429.1934178	-0.036146441	0.100158238	0.360893341	0.718179182	0.853791016	0.036146441
ENSMUSG00000087366	Junos	230451	lncRNA	31.68335001	0.036147875	0.36976863	-0.097758089	0.922124388	0.9624373	0.036147875
ENSMUSG00000029596	Sdsl	257635	protein_coding	202.1355249	0.036156091	0.093818944	-0.385381556	0.699954745	0.841086565	0.036156091
ENSMUSG00000042292	Mrtfa	223701	protein_coding	328.0160912	0.036203629	0.086617994	-0.417968915	0.675969845	0.826457838	0.036203629
ENSMUSG00000087938	Gm26049	115490020	snRNA	2.468360153	0.036218233	0.953608787	-0.03798018	0.969703485	1	0.036218233
ENSMUSG00000033618	Map3k13	71751	protein_coding	291.5219037	-0.036267027	0.131679358	0.275419229	0.782994172	0.891196207	0.036267027
ENSMUSG00000063524	Eno1	13806	protein_coding	2814.790772	-0.036273618	0.117499603	0.308712687	0.757540092	0.878512436	0.036273618
ENSMUSG00000005846	Rsl1d1	66409	protein_coding	601.5452053	0.036341808	0.099935131	-0.363653973	0.716116418	0.852650725	0.036341808

ensembl_gene_id	external_gene_name	entrezgene_id	gene_biotype	baseMean	log2FoldChange	lfcSE	stat	pvalue	padj	absLog2FC
ENSMUSG00000020455	Trim11	94091	protein_coding	296.6819996	-0.03636857	0.092848978	0.391695961	0.695282874	0.838339763	0.03636857
ENSMUSG00000025439	Clns1a	12729	protein_coding	469.6005279	0.036381845	0.108566478	-0.33511122	0.737541189	0.866806221	0.036381845
ENSMUSG00000021519	Mterf3	66410	protein_coding	209.0082901	0.036443367	0.092834082	-0.392564523	0.694641147	0.8379481	0.036443367
ENSMUSG00000042015	Wdr41	218460	protein_coding	314.3618874	-0.036466624	0.099093766	0.368001196	0.712872343	0.850727986	0.036466624
ENSMUSG00000039771	Polr2j	20022	protein_coding	241.1033609	-0.036484432	0.072400524	0.503924971	0.614314102	0.78673795	0.036484432
ENSMUSG00000041203	Trir	68544	protein_coding	859.3931811	0.036561438	0.077845306	-0.469667851	0.63859234	0.80301103	0.036561438
ENSMUSG00000024991	Eif3a	13669	protein_coding	2741.284954	0.036588535	0.088091533	-0.415346781	0.67788806	0.827372024	0.036588535
ENSMUSG00000056211	R3hdm1	226412	protein_coding	317.0983168	0.036589149	0.106389619	-0.343916533	0.730909058	0.861359607	0.036589149
ENSMUSG00000004748	Mtfp1	67900	protein_coding	150.1404937	0.036604208	0.114345428	-0.320119556	0.748877701	0.87389673	0.036604208
ENSMUSG00000051351	Zfp46	22704	protein_coding	206.9641432	-0.036656984	0.101433548	0.361389152	0.717808556	0.853596205	0.036656984
ENSMUSG00000004980	Hnrnpa2b1	53379	protein_coding	4108.370311	0.036657159	0.077448078	-0.473312696	0.635990106	0.801613541	0.036657159
ENSMUSG00000041926	Rnpep	215615	protein_coding	1024.559863	-0.036671933	0.089300603	0.410657166	0.68132394	0.829503357	0.036671933
ENSMUSG00000052291	5330438D12Rik	102633868	lncRNA	16.61470006	-0.036736391	0.269703756	0.136210159	0.891655145	0.947050968	0.036736391
ENSMUSG00000024259	Slc25a46	67453	protein_coding	669.841404	0.036748252	0.075788089	-0.484881623	0.627760319	0.796376973	0.036748252
ENSMUSG00000032281	Acsbg1	94180	protein_coding	5.409771198	0.036758853	0.741905448	-0.049546547	0.960483744	0.980697519	0.036758853
ENSMUSG00000022553	Maf1	68877	protein_coding	730.0962625	-0.036764645	0.060090747	0.611818735	0.540657693	0.734843633	0.036764645
ENSMUSG00000024981	Acsl5	433256	protein_coding	1371.93623	-0.036807802	0.116575334	0.315742628	0.752197868	0.875699372	0.036807802
ENSMUSG00000022466	Rpap3	71919	protein_coding	134.3974547	0.03684929	0.123597367	-0.298139766	0.765596492	0.882510888	0.03684929
ENSMUSG00000023967	Mrps18a	68565	protein_coding	394.1276613	-0.036871571	0.073496303	0.501679266	0.615893152	0.788072176	0.036871571
ENSMUSG00000015522	Arnt	11863	protein_coding	487.9069061	-0.036881358	0.067194087	0.548878032	0.583089155	0.765756487	0.036881358
ENSMUSG00000088958	Scarna8	100217448	scaRNA	1.108339289	0.036883194	1.116991331	-0.033020125	0.973658539	1	0.036883194
ENSMUSG00000061981	Flot2	14252	protein_coding	150.2268157	-0.036921611	0.119207142	0.309726502	0.756768947	0.877988085	0.036921611
ENSMUSG00000007038	Neu1	18010	protein_coding	2008.022453	-0.03692206	0.182439722	0.202379502	0.839620052	0.920255229	0.03692206
ENSMUSG00000048285	Frmd6	319710	protein_coding	39.36221791	0.036922643	0.181808045	-0.203085858	0.839067925	0.919794636	0.036922643
ENSMUSG00000031487	Brf2	66653	protein_coding	81.65625327	-0.036952361	0.132971384	0.277897094	0.781091355	0.890541867	0.036952361
ENSMUSG00000086370	Ftx	78878	lncRNA	65.96581796	-0.036993011	0.188801373	0.195936134	0.844660154	0.923599766	0.036993011
ENSMUSG00000068264	Ap5s1	69596	protein_coding	182.549863	-0.037016436	0.12730924	0.290760006	0.771234875	0.885199039	0.037016436
ENSMUSG00000039656	Rxrb	20182	protein_coding	554.9876103	0.037019186	0.069349601	-0.533805324	0.59347623	0.772683055	0.037019186
ENSMUSG00000040472	Rabggta	56187	protein_coding	238.5210526	-0.037058488	0.081724993	0.453453549	0.650222185	0.809917796	0.037058488
ENSMUSG00000054434	Tmem120b	330189	protein_coding	31.4089334	0.037134963	0.224037385	-0.165753422	0.868351003	0.934807592	0.037134963
ENSMUSG00000041293	Adgrf1	77596	protein_coding	46.67352637	0.037146392	0.382095583	-0.097217537	0.922553641	0.962530504	0.037146392
ENSMUSG00000029815	Malsu1	75593	protein_coding	164.0933818	0.037146804	0.089185976	-0.416509469	0.67703724	0.826943172	0.037146804
ENSMUSG00000031902	Nfatc3	18021	protein_coding	762.2869193	-0.037149118	0.050526918	0.735234198	0.462196875	0.675880563	0.037149118
ENSMUSG00000025979	Mob4	19070	protein_coding	708.3215347	0.037215156	0.087148204	-0.427032968	0.669355315	0.822609935	0.037215156
ENSMUSG00000044145	1810024B03Rik	329509	protein_coding	13.55893557	-0.037225524	0.349240923	0.106589812	0.915114401	0.95833356	0.037225524
ENSMUSG00000035596	Mboat7	77582	protein_coding	486.5674141	-0.037234837	0.074639504	0.498862334	0.617876371	0.789468115	0.037234837
ENSMUSG00000024566	Atp9b	50771	protein_coding	642.1483617	0.037262973	0.060958142	-0.611287868	0.541009021	0.735049182	0.037262973
ENSMUSG00000074909	Ranbp6	240614	protein_coding	217.8213493	-0.037305766	0.08613246	0.433120866	0.664926965	0.819267308	0.037305766
ENSMUSG00000038618	Rassf7	66985	protein_coding	1072.703907	-0.037325893	0.096388465	0.387244394	0.698575287	0.840298483	0.037325893

ensembl_gene_id	external_gene_name	entrezgene_id	gene_biotype	baseMean	log2FoldChange	lfcSE	stat	pvalue	padj	absLog2FC
ENSMUSG00000026792	Lrsam1	227738	protein_coding	245.7141234	-0.037356609	0.092625572	0.403307725	0.68672186	0.833127005	0.037356609
ENSMUSG00000056383	AI987944	233168	protein_coding	137.7857914	-0.037394237	0.122061745	0.306355093	0.759334296	0.879038683	0.037394237
ENSMUSG00000013646	Sh3bp5l	79566	protein_coding	428.9945128	-0.03742073	0.063366107	0.590548046	0.554823284	0.744703672	0.03742073
ENSMUSG00000061013	Mkx	210719	protein_coding	160.6123841	-0.037429829	0.131996579	0.283566661	0.77674249	0.888506311	0.037429829
ENSMUSG00000026065	Slc9a4	110895	protein_coding	5.86196662	0.037439697	0.50150563	-0.07465459	0.940489539	0.971492103	0.037439697
ENSMUSG00000006423	Steep1	77644	protein_coding	342.2605622	0.037450363	0.106669178	-0.351088883	0.725521668	0.857956591	0.037450363
ENSMUSG00000049086	Bmyc	107771	protein_coding	17.18315258	0.037455388	0.262552212	-0.142658816	0.886559644	0.943887948	0.037455388
ENSMUSG00000069874	Irgm2	54396	protein_coding	175.3532193	-0.03751705	0.222923218	0.168295839	0.866350547	0.933791206	0.03751705
ENSMUSG00000025373	Rnf41	67588	protein_coding	342.0705477	-0.037532683	0.075241137	0.498831955	0.617897774	0.789468115	0.037532683
ENSMUSG00000036537	Rnf113a1	69942	protein_coding	26.28009886	0.037543065	0.208829483	-0.179778567	0.857326411	0.930374388	0.037543065
ENSMUSG00000006471	Ndor1	78797	protein_coding	65.50198746	0.03757272	0.163798273	-0.229384102	0.818570391	0.908812719	0.03757272
ENSMUSG00000063801	Ap3s2	11778	protein_coding	310.1390708	0.037597386	0.072404543	-0.51926832	0.603573642	0.779177919	0.037597386
ENSMUSG00000027652	Ralgapb	228850	protein_coding	1538.59055	-0.037604375	0.059481961	0.632197971	0.527257523	0.725715181	0.037604375
ENSMUSG00000002980	Bcam	57278	protein_coding	56.95408451	0.037646522	0.163955723	-0.229613958	0.81839176	0.908812719	0.037646522
ENSMUSG00000021675	F2rl2	14064	protein_coding	19.1215894	0.037652298	0.276441214	-0.136203635	0.891660302	0.947050968	0.037652298
ENSMUSG00000021595	Nsun2	28114	protein_coding	959.5752043	0.037660883	0.096628991	-0.389747246	0.696723457	0.839060823	0.037660883
ENSMUSG00000019578	Ubxn6	66530	protein_coding	208.4422178	0.037718968	0.134840118	-0.279731052	0.779683853	0.890125936	0.037718968
ENSMUSG00000042724	Map3k9	338372	protein_coding	252.2027264	-0.037741239	0.103098376	0.366070159	0.714312724	0.851829233	0.037741239
ENSMUSG00000037938	Chchd5	66170	protein_coding	135.6819797	-0.037770208	0.094510818	0.399638995	0.68942243	0.834319362	0.037770208
ENSMUSG00000018924	Alox15	11687	protein_coding	5.651957746	0.037782131	0.677812268	-0.055741291	0.955547905	0.977832194	0.037782131
ENSMUSG00000042306	S100a14	66166	protein_coding	1308.369798	-0.037799075	0.163984246	0.230504308	0.817699916	0.908714429	0.037799075
ENSMUSG00000029432	Nipsnap2	14467	protein_coding	983.1906584	-0.037873196	0.097006606	0.390418736	0.696226935	0.838743187	0.037873196
ENSMUSG00000097254	C430042M11Rik	320021	lncRNA	30.14714013	0.037882324	0.227169974	-0.166757618	0.867560767	0.934200477	0.037882324
ENSMUSG00000023249	Parp3	235587	protein_coding	112.7434322	-0.037913736	0.135766844	0.279256225	0.780048198	0.890125936	0.037913736
ENSMUSG00000052137	Rbm12b2	77604	protein_coding	95.44078377	0.037941789	0.15566862	-0.243734345	0.807436572	0.90502442	0.037941789
ENSMUSG00000028329	Xpa	22590	protein_coding	112.4650907	-0.037942207	0.134583599	0.281922959	0.778002585	0.889068901	0.037942207
ENSMUSG00000049739	Zfp646	233905	protein_coding	814.9213813	0.037953364	0.038924981	-0.975038721	0.329541053	0.551855356	0.037953364
ENSMUSG00000026177	Slc11a1	18173	protein_coding	4.536060121	-0.037968171	1.005638461	0.037755289	0.969882793	0.984509162	0.037968171
ENSMUSG00000006288	Ttc5	219022	protein_coding	321.5649251	-0.037987029	0.089371153	0.425047986	0.670801694	0.823378739	0.037987029
ENSMUSG00000014767	Tbp	21374	protein_coding	150.3709788	0.037987586	0.100478066	-0.37806845	0.705379745	0.845574994	0.037987586
ENSMUSG00000037896	Rcor1	217864	protein_coding	1015.378361	0.038002382	0.087586847	-0.433882289	0.664373922	0.818950015	0.038002382
ENSMUSG00000061759	Armt1	73419	protein_coding	174.3880616	0.038006572	0.116748319	-0.325542775	0.744770314	0.8712288	0.038006572
ENSMUSG00000025917	Cops5	26754	protein_coding	593.4415913	0.038014512	0.074776003	-0.508378498	0.611187929	0.784646895	0.038014512
ENSMUSG00000006599	Gtf2h1	14884	protein_coding	574.7943877	0.038040133	0.070331387	-0.540869942	0.58859723	0.769577776	0.038040133
ENSMUSG00000030315	Vgll4	232334	protein_coding	571.390717	-0.038070931	0.10767631	0.353568306	0.72366243	0.856795141	0.038070931
ENSMUSG00000040321	Zfp770	228491	protein_coding	81.89772367	0.038073237	0.154880239	-0.245823721	0.805818693	0.904335026	0.038073237
ENSMUSG00000034751	Mast4	328329	protein_coding	306.5120436	0.038076261	0.113779487	-0.334649611	0.737889416	0.866982505	0.038076261
ENSMUSG00000089235	Gm23119	115486069	scaRNA	89.37322216	-0.038079192	0.245040136	0.155399814	0.876506131	0.938630585	0.038079192
ENSMUSG00000054196	Cthrc1	68588	protein_coding	4.787686734	0.038091756	0.499491579	-0.076261057	0.939211408	0.97108297	0.038091756

ensembl_gene_id	external_gene_name	entrezgene_id	gene_biotype	baseMean	log2FoldChange	lfcSE	stat	pvalue	padj	absLog2FC
ENSMUSG00000002329	Mdp1	67881	protein_coding	943.9921465	-0.038114162	0.099001137	0.384987121	0.700246958	0.841315261	0.038114162
ENSMUSG00000014349	Ube2z	268470	protein_coding	1279.828942	0.038120192	0.044865552	-0.849653923	0.395517523	0.617841913	0.038120192
ENSMUSG00000032966	Fkbp1a	14225	protein_coding	461.8704569	0.038126608	0.09150247	-0.41667299	0.676917613	0.826943172	0.038126608
ENSMUSG00000040506	Ambra1	228361	protein_coding	601.3264084	0.038156116	0.066272316	-0.575747439	0.564785905	0.751588072	0.038156116
ENSMUSG00000021832	Psmc6	67089	protein_coding	1000.992988	0.038157726	0.083149106	-0.458907224	0.646300791	0.807226927	0.038157726
ENSMUSG00000038708	Golga4	54214	protein_coding	3773.399457	0.038165661	0.063391172	-0.60206587	0.547130292	0.738779548	0.038165661
ENSMUSG00000048921	Zfp689	71131	protein_coding	31.65405392	-0.038184082	0.182715656	0.208980899	0.83446315	0.916826487	0.038184082
ENSMUSG00000028433	Ubap2	68926	protein_coding	374.704412	0.038228781	0.090483367	-0.422495123	0.67266366	0.824518194	0.038228781
ENSMUSG00000009535	Rnmt	67897	protein_coding	252.7742681	-0.038241026	0.112840336	0.338895	0.734688831	0.864656729	0.038241026
ENSMUSG00000037661	Gpr160	71862	protein_coding	382.7976112	-0.038264923	0.134246997	0.285033734	0.775618297	0.88791795	0.038264923
ENSMUSG00000038010	Ccdc138	76138	protein_coding	50.59785374	-0.038320542	0.173246458	0.221190912	0.824943785	0.911456552	0.038320542
ENSMUSG00000045281	Gpr20	239530	protein_coding	212.3629767	-0.03833711	0.249467661	0.153675671	0.877865469	0.9393553	0.03833711
ENSMUSG00000030095	Tmem43	74122	protein_coding	491.6061233	-0.038353083	0.110012851	0.348623663	0.727371861	0.859261888	0.038353083
ENSMUSG00000033430	Terf2ip	57321	protein_coding	254.6123832	0.03838255	0.113818639	-0.337225519	0.735946901	0.865628713	0.03838255
ENSMUSG00000110909	B020014A21Rik	619314	lncRNA	1.092188399	0.038419811	2.29981321	-0.016705622	0.986671462	1	0.038419811
ENSMUSG00000025646	Atrip	235610	protein_coding	135.9673752	0.038484841	0.126622441	-0.303933808	0.761178321	0.880259019	0.038484841
ENSMUSG00000065864	Gm24031	115488396	snoRNA	0.769468905	-0.03849408	1.121922123	0.034310831	0.972629288	1	0.03849408
ENSMUSG00000006412	Pfdn2	18637	protein_coding	133.1162265	-0.038542505	0.09409741	0.40960219	0.68209779	0.830017162	0.038542505
ENSMUSG00000000605	Clcn4	12727	protein_coding	1124.302622	-0.038612373	0.083678603	0.461436629	0.644485377	0.806290266	0.038612373
ENSMUSG00000001962	Fam50a	108160	protein_coding	192.5687404	-0.038652073	0.104762231	0.368950458	0.712164653	0.85037506	0.038652073
ENSMUSG00000009470	Tnpo1	238799	protein_coding	1713.415294	0.03866323	0.081369408	-0.475156832	0.634675193	0.800822518	0.03866323
ENSMUSG00000022109	Med4	67381	protein_coding	157.2741223	0.038686139	0.097467163	-0.396914584	0.69143045	0.835769466	0.038686139
ENSMUSG00000033327	Tnxb	81877	protein_coding	8.97222531	0.03872054	0.781945739	-0.049518193	0.960506339	0.980697519	0.03872054
ENSMUSG00000032673	Prorsd1	67939	protein_coding	106.5104771	-0.0387402	0.129874799	0.298288816	0.76548274	0.882510888	0.0387402
ENSMUSG00000000938	Hoxa10	15395	protein_coding	377.2528266	-0.038809497	0.138992014	0.279221058	0.780075184	0.890125936	0.038809497
ENSMUSG00000048329	Mfsd6l	215723	protein_coding	168.4053621	-0.038823255	0.094903803	0.409080078	0.682480896	0.830405926	0.038823255
ENSMUSG00000020121	Srgap1	117600	protein_coding	246.2531101	0.038893656	0.138845052	-0.280122738	0.77938334	0.890030875	0.038893656
ENSMUSG00000024999	Noc3l	57753	protein_coding	176.7363687	0.03890479	0.130090141	-0.299060248	0.764894076	0.882255147	0.03890479
ENSMUSG00000015214	Mtmr1	53332	protein_coding	391.6805975	0.038910263	0.100367764	-0.387676889	0.698255161	0.840144679	0.038910263
ENSMUSG00000035392	Dennd1a	227801	protein_coding	159.3726411	-0.039021584	0.100126156	0.389724177	0.696740517	0.839060823	0.039021584
ENSMUSG00000022661	Cd200	17470	protein_coding	24.76322006	-0.039034219	0.215057983	0.181505555	0.855970773	0.93002838	0.039034219
ENSMUSG00000042541	Sem1	20422	protein_coding	1183.951142	0.039040554	0.067809788	-0.57573626	0.564793463	0.751588072	0.039040554
ENSMUSG00000024309	Pfdn6	14976	protein_coding	349.1596209	-0.039079299	0.072532232	0.53878529	0.590035015	0.770846581	0.039079299
ENSMUSG00000039005	Tlr4	21898	protein_coding	385.8979073	0.039111478	0.12233283	-0.319713671	0.749185395	0.874014884	0.039111478
ENSMUSG00000108129	4930417O13Rik	73870	lncRNA	2.330756465	-0.039134584	0.945279735	0.041400003	0.96697701	1	0.039134584
ENSMUSG00000028565	Nfia	18027	protein_coding	331.7212265	0.039206193	0.096463032	-0.406437497	0.684421173	0.831662311	0.039206193
ENSMUSG00000050229	Pigm	67556	protein_coding	355.7625997	-0.039209929	0.080205616	0.488867619	0.624935422	0.794593745	0.039209929
ENSMUSG00000036552	Ermard	381062	protein_coding	50.84057706	-0.039283033	0.170249895	0.230737489	0.817518747	0.908714429	0.039283033
ENSMUSG00000030591	Psmd8	57296	protein_coding	1521.092148	0.039288085	0.061671576	-0.637053368	0.524090082	0.722741011	0.039288085

Supplementary Table 1

ensembl_gene_id	external_gene_name	entrezgene_id	gene_biotype	baseMean	log2FoldChange	lfcSE	stat	pvalue	padj	absLog2FC
ENSMUSG00000038965	Ube2l3	22195	protein_coding	868.9568896	-0.039313043	0.072860129	0.539568665	0.589494531	0.770506411	0.039313043
ENSMUSG00000036779	Tent4b	214627	protein_coding	411.5000018	-0.039332791	0.064872298	0.606311058	0.544308217	0.736796542	0.039332791
ENSMUSG00000066235	Pomgnt2	215494	protein_coding	126.833415	-0.039357131	0.168599398	0.233435775	0.815423036	0.908262243	0.039357131
ENSMUSG00000066798	Zbtb6	241322	protein_coding	345.8318255	-0.039392146	0.08265482	0.476586187	0.633656821	0.800362194	0.039392146
ENSMUSG00000000776	Polr3d	67065	protein_coding	232.9622134	0.039414412	0.085301103	-0.462062165	0.644036741	0.80597323	0.039414412
ENSMUSG00000028098	Rnf115	67845	protein_coding	541.6062068	0.039455656	0.064548029	-0.611260438	0.541027178	0.735049182	0.039455656
ENSMUSG00000018569	Cldn7	53624	protein_coding	6571.834732	-0.039494916	0.076344646	0.517323984	0.604930014	0.780088572	0.039494916
ENSMUSG00000031365	Zfp275	27081	protein_coding	188.358204	0.039502106	0.118161153	-0.334307043	0.738147876	0.866982505	0.039502106
ENSMUSG00000000441	Raf1	110157	protein_coding	731.5476738	0.039522429	0.059289318	-0.666602859	0.505025842	0.709123099	0.039522429
ENSMUSG00000049225	Pdp1	381511	protein_coding	269.0199049	0.039639209	0.162531811	-0.243885853	0.807319226	0.904987729	0.039639209
ENSMUSG00000015837	Sqstm1	18412	protein_coding	2595.756012	0.039642848	0.122138378	-0.324573232	0.745504089	0.871840133	0.039642848
ENSMUSG00000038949	Cnst	226744	protein_coding	637.157925	-0.039670303	0.075279386	0.526974318	0.598211423	0.775969165	0.039670303
ENSMUSG00000107006	Gm5553	433874	processed_pseudogene	0.607579554	-0.039732755	1.860530643	0.021355604	0.982961989	1	0.039732755
ENSMUSG00000020755	Sap30bp	57230	protein_coding	375.2724246	-0.039783882	0.062600268	0.635522557	0.52508766	0.723512525	0.039783882
ENSMUSG00000073775	Kti12	100087	protein_coding	177.3892844	-0.039785859	0.149590614	0.265964943	0.790266208	0.895392916	0.039785859
ENSMUSG00000065400	Mir200a	387242	miRNA	20.66459916	-0.039785955	0.309764762	0.128439253	0.897801369	0.949794479	0.039785955
ENSMUSG00000040563	Plppr2	235044	protein_coding	15.43158002	-0.039810783	0.294478433	0.135190829	0.892460998	0.947202684	0.039810783
ENSMUSG00000040242	Fgfr1op2	67529	protein_coding	866.204485	0.039845287	0.071537685	-0.556983182	0.57753892	0.761475268	0.039845287
ENSMUSG00000040688	Tbl3	213773	protein_coding	394.1816926	-0.039909113	0.089553325	0.445646361	0.655852716	0.813424841	0.039909113
ENSMUSG00000036330	Slc18a1	110877	protein_coding	55.02963601	-0.039968845	0.187152303	0.213563201	0.830887699	0.914497068	0.039968845
ENSMUSG00000016028	Celsr1	12614	protein_coding	1754.411007	-0.04001813	0.09225563	0.433774395	0.664452277	0.818950015	0.04001813
ENSMUSG00000040969	Arhgef38	77669	protein_coding	794.8629897	-0.040060477	0.155695225	0.257300612	0.79694672	0.899144506	0.040060477
ENSMUSG00000032481	Smarcc1	20588	protein_coding	642.3469086	0.040071288	0.084778393	-0.472659209	0.636456333	0.801895161	0.040071288
ENSMUSG00000010660	Plcd1	18799	protein_coding	534.0587554	-0.040129547	0.138434102	0.289881943	0.771906556	0.885478012	0.040129547
ENSMUSG00000043487	Acot6	217700	protein_coding	27.18880892	-0.04018921	0.198821916	0.202136719	0.839809842	0.920280009	0.04018921
ENSMUSG00000060261	Gtf2i	14886	protein_coding	760.5775839	0.040196434	0.074087295	-0.542555017	0.587436218	0.768755187	0.040196434
ENSMUSG00000046138	9930021J03Rik	240613	protein_coding	576.1918315	0.040233696	0.088185961	-0.456236979	0.648219577	0.808756278	0.040233696
ENSMUSG00000063870	Chd4	107932	protein_coding	3194.539178	0.040238538	0.075582233	-0.532380916	0.5944622	0.77323163	0.040238538
ENSMUSG00000061322	Dnai1	68922	protein_coding	2.115843828	-0.040247029	1.852426748	0.021726651	0.982666004	1	0.040247029
ENSMUSG00000068854	H2bc21	319190	protein_coding	119.8048083	0.040267814	0.134790391	-0.298743953	0.765135418	0.882363568	0.040267814
ENSMUSG00000020669	Sh3yl1	24057	protein_coding	352.804777	-0.040282664	0.078896819	0.510573999	0.609649386	0.783482488	0.040282664
ENSMUSG00000079164	Tlr5	53791	protein_coding	68.0372835	0.040285004	0.278299215	-0.144754287	0.884904876	0.943016089	0.040285004
ENSMUSG00000036061	Smug1	71726	protein_coding	228.3211143	-0.040295407	0.095591595	0.421537129	0.673362905	0.824897384	0.040295407
ENSMUSG00000067825	Pex26	74043	protein_coding	330.3867817	0.040304527	0.067723571	-0.595132926	0.551754621	0.742476843	0.040304527
ENSMUSG00000089847	Timm10b	14356	protein_coding	93.58132037	-0.040310175	0.180079293	0.22384681	0.822876505	0.910240646	0.040310175
ENSMUSG00000029518	Rab35	77407	protein_coding	725.7809227	0.0403834	0.075341432	-0.536005215	0.591954946	0.771976939	0.0403834
ENSMUSG00000022528	Hes1	15205	protein_coding	977.2623035	0.040505206	0.071309249	-0.568021766	0.570020189	0.755664558	0.040505206
ENSMUSG00000070565	Rasal2	226525	protein_coding	135.1537411	-0.04053187	0.10325337	0.392547671	0.694653596	0.8379481	0.04053187
ENSMUSG00000045629	Sh3tc2	225608	protein_coding	29.66374938	-0.040557383	0.254053651	0.159641017	0.873163867	0.937483135	0.040557383

ensembl_gene_id	external_gene_name	entrezgene_id	gene_biotype	baseMean	log2FoldChange	lfcSE	stat	pvalue	padj	absLog2FC
ENSMUSG00000022261	Sdc2	15529	protein_coding	451.862126	-0.040558437	0.090496484	0.448176937	0.654025506	0.81231093	0.040558437
ENSMUSG00000028948	Nol9	74035	protein_coding	332.0089935	0.040559146	0.102511423	-0.395654897	0.692359633	0.836513744	0.040559146
ENSMUSG00000023892	Zfp51	22709	protein_coding	159.2934514	-0.040561321	0.11810914	0.343422374	0.73128073	0.861613154	0.040561321
ENSMUSG00000066149	Cdc26	66440	protein_coding	217.8797284	-0.040610067	0.086830338	0.467694454	0.640003108	0.8036518	0.040610067
ENSMUSG00000025138	Sirt7	209011	protein_coding	728.2683722	-0.040620417	0.089352666	0.454607775	0.64939144	0.809667817	0.040620417
ENSMUSG00000033149	Phldb2	208177	protein_coding	106.1895724	0.040635919	0.12420191	-0.327176284	0.743534558	0.870461483	0.040635919
ENSMUSG00000027679	Dnajc19	67713	protein_coding	252.0431548	-0.040643835	0.077558988	0.524037721	0.600252304	0.77680602	0.040643835
ENSMUSG00000031431	Tsc22d3	14605	protein_coding	773.1679406	-0.04064785	0.116759161	0.348134144	0.727739443	0.859322531	0.04064785
ENSMUSG00000029191	Rfc1	19687	protein_coding	547.3247749	-0.040668887	0.106464976	0.381993106	0.702466478	0.843000612	0.040668887
ENSMUSG00000030213	Atf7ip	54343	protein_coding	752.5508647	0.040680017	0.073757017	-0.551540974	0.581262887	0.764339772	0.040680017
ENSMUSG00000026343	Gpr39	71111	protein_coding	106.7933073	0.040685779	0.153646187	-0.264801748	0.79116219	0.895862069	0.040685779
ENSMUSG00000029238	Clock	12753	protein_coding	1508.642235	-0.040685837	0.11224912	0.362460187	0.71700817	0.853197357	0.040685837
ENSMUSG00000028161	Ppp3ca	19055	protein_coding	886.4617117	-0.040703809	0.099680378	0.408343249	0.683021692	0.830651757	0.040703809
ENSMUSG00000023156	Rpp14	67053	protein_coding	224.1607357	-0.040734364	0.138323878	0.294485409	0.768387011	0.883975099	0.040734364
ENSMUSG00000046822	Slc39a3	106947	protein_coding	396.0290589	0.040736839	0.091006069	-0.447627721	0.654421894	0.812494814	0.040736839
ENSMUSG00000042499	Hoxd11	15431	protein_coding	10.38919938	-0.040739057	0.411826474	0.09892287	0.92119951	0.961976016	0.040739057
ENSMUSG00000024422	Dhx16	69192	protein_coding	715.3513868	0.04076287	0.058095117	-0.701657426	0.48289283	0.693654751	0.04076287
ENSMUSG00000041757	Plekha6	240753	protein_coding	4665.171911	-0.040764323	0.061113954	0.667021524	0.504758385	0.70880782	0.040764323
ENSMUSG00000058835	Abi1	11308	protein_coding	1498.261271	-0.040773616	0.058870965	0.692592951	0.488565035	0.696497169	0.040773616
ENSMUSG00000052794	1700030K09Rik	72254	protein_coding	59.8882899	-0.040775204	0.145497443	0.280246878	0.779288104	0.889983662	0.040775204
ENSMUSG00000028582	Cc2d1b	319965	protein_coding	507.345905	0.040816689	0.059960271	-0.680728899	0.496043047	0.702242166	0.040816689
ENSMUSG00000084826	AI847159	100048701	lncRNA	1.534471555	-0.040858339	1.05403564	0.038763717	0.969078773	1	0.040858339
ENSMUSG00000056537	Rlim	19820	protein_coding	1374.53376	0.040870562	0.074019324	-0.552160703	0.580838255	0.764064128	0.040870562
ENSMUSG00000036893	Ehmt1	77683	protein_coding	775.0473659	-0.040925912	0.070199523	0.582994158	0.559897233	0.748554316	0.040925912
ENSMUSG00000093044	Snord8	100217445	snoRNA	3.327967407	-0.040926452	0.551080613	0.074265817	0.940798876	1	0.040926452
ENSMUSG00000025484	Bet1l	54399	protein_coding	244.6068901	-0.04093148	0.07837532	0.522249607	0.601496551	0.777725809	0.04093148
ENSMUSG00000052373	Mpp3	13384	protein_coding	106.5723709	0.04093945	0.178266424	-0.229653177	0.818361282	0.908812719	0.04093945
ENSMUSG00000027424	Mgme1	74528	protein_coding	141.0405667	-0.040941574	0.148616873	0.275484026	0.782944397	0.891196207	0.040941574
ENSMUSG00000053470	Kdm3a	104263	protein_coding	682.1946227	-0.040945869	0.073022125	0.560732364	0.574980001	0.760164897	0.040945869
ENSMUSG00000025436	Atp23	68876	protein_coding	47.58138411	0.040962702	0.171114854	-0.239387175	0.810805374	0.906297546	0.040962702
ENSMUSG00000016831	Tox4	268741	protein_coding	816.5118391	0.04097033	0.057472659	-0.71286644	0.475928408	0.687930892	0.04097033
ENSMUSG00000050323	Ndufaf6	76947	protein_coding	73.86264982	-0.040994405	0.155595332	0.263468089	0.792189816	0.896636371	0.040994405
ENSMUSG00000027522	Stx16	228960	protein_coding	530.0125725	-0.040995987	0.060272417	0.680178249	0.496391603	0.702494724	0.040995987
ENSMUSG00000036282	Naa30	70646	protein_coding	461.7903256	0.040996796	0.063282642	-0.647836353	0.5170908	0.717711842	0.040996796
ENSMUSG00000019726	Lyst	17101	protein_coding	298.9354274	-0.041005409	0.092076427	0.445341013	0.656073333	0.813637356	0.041005409
ENSMUSG00000022472	Desi1	28075	protein_coding	214.8826824	-0.04100962	0.090848444	0.451406959	0.651696269	0.810888501	0.04100962
ENSMUSG00000034543	Morc2a	74522	protein_coding	416.6655941	0.041019985	0.070889303	-0.578648447	0.562826408	0.75067522	0.041019985
ENSMUSG00000026436	Elk4	13714	protein_coding	553.1714474	-0.0410376	0.069033311	0.594460839	0.552203927	0.742839333	0.0410376
ENSMUSG00000024972	Lgals12	56072	protein_coding	244.4386253	0.041070066	0.221289839	-0.185594	0.852763159	0.92788168	0.041070066

ensembl_gene_id	external_gene_name	entrezgene_id	gene_biotype	baseMean	log2FoldChange	lfcSE	stat	pvalue	padj	absLog2FC
ENSMUSG00000033658	Ddx19b	234733	protein_coding	429.9602003	-0.041089899	0.090537261	0.453845176	0.649940266	0.809917796	0.041089899
ENSMUSG00000038346	Zfp384	269800	protein_coding	333.8183899	-0.04111091	0.09465812	0.43430939	0.664063786	0.818817302	0.04111091
ENSMUSG00000030788	Rnf141	67150	protein_coding	881.4482722	0.041114149	0.099696389	-0.412393561	0.680050982	0.828381052	0.041114149
ENSMUSG00000086386	Gm12108	104692	lncRNA	1.464638804	-0.041119632	2.359632589	0.017426286	0.986096539	1	0.041119632
ENSMUSG00000032534	Cep63	28135	protein_coding	307.8618181	-0.041135971	0.084855036	0.484779371	0.627832857	0.796376973	0.041135971
ENSMUSG00000029208	Guf1	231279	protein_coding	329.6280776	0.041161352	0.067901088	-0.606195773	0.544384759	0.736796542	0.041161352
ENSMUSG00000015189	Casd1	213819	protein_coding	1151.818215	0.04116221	0.194752578	-0.211356433	0.832609151	0.915629096	0.04116221
ENSMUSG00000006705	Pknox1	18771	protein_coding	263.647121	-0.041172028	0.106717248	0.385804808	0.699641234	0.84101582	0.041172028
ENSMUSG00000021244	Ylpm1	56531	protein_coding	334.233419	-0.041187607	0.12811465	0.321490222	0.747838921	0.873148468	0.041187607
ENSMUSG00000012350	Ehf	13661	protein_coding	6098.003229	-0.041211903	0.125000191	0.329694724	0.741630641	0.869465313	0.041211903
ENSMUSG00000028944	Prkag2	108099	protein_coding	426.5156573	-0.041217747	0.103574322	0.397953335	0.690664584	0.835178936	0.041217747
ENSMUSG00000035007	Rundc1	217201	protein_coding	247.4381947	0.041230156	0.088128814	-0.467839679	0.639899243	0.803650332	0.041230156
ENSMUSG00000034957	Cebpa	12606	protein_coding	664.2369576	0.041243233	0.090105983	-0.457719139	0.647154236	0.807915411	0.041243233
ENSMUSG00000029075	Tnfrsf4	22163	protein_coding	5.691792291	0.041252848	0.532884818	-0.077414192	0.938294051	0.970559847	0.041252848
ENSMUSG00000029249	Rest	19712	protein_coding	713.3274774	0.041255038	0.08979368	-0.459442554	0.645916396	0.807082912	0.041255038
ENSMUSG00000008226	Scrn3	74616	protein_coding	147.6038897	-0.041256468	0.14010886	0.294460091	0.768406355	0.883975099	0.041256468
ENSMUSG00000070469	Adamtsl3	269959	protein_coding	2.065521336	0.041257398	1.800163532	-0.022918694	0.981715128	1	0.041257398
ENSMUSG00000043140	Tmem186	66690	protein_coding	279.847645	-0.04125922	0.124562245	0.33123375	0.740467929	0.868595495	0.04125922
ENSMUSG00000070939	Tgfbrap1	73122	protein_coding	363.8857476	-0.041259513	0.070893434	0.581993439	0.5605711	0.749146509	0.041259513
ENSMUSG00000090258	Churc1	211151	protein_coding	178.7246763	0.041271331	0.109406127	-0.377230524	0.706002298	0.845943387	0.041271331
ENSMUSG00000000409	Lck	16818	protein_coding	59.44306172	0.041288365	0.178477822	-0.231336109	0.817053697	0.908714429	0.041288365
ENSMUSG00000016918	Sulf1	240725	protein_coding	9.009965816	-0.041344831	0.821690988	0.050316763	0.959869966	0.980506535	0.041344831
ENSMUSG00000047213	Ythdf3	229096	protein_coding	1534.500626	0.041368567	0.058472491	-0.707487684	0.479263464	0.690564248	0.041368567
ENSMUSG00000040225	Prrc2c	226562	protein_coding	1681.050734	0.041389812	0.109081711	-0.379438602	0.704362189	0.844600435	0.041389812
ENSMUSG00000027763	Mbnl1	56758	protein_coding	1423.605178	-0.041406756	0.07032992	0.588750225	0.55602884	0.745551704	0.041406756
ENSMUSG00000036594	H2-Aa	14960	protein_coding	21.78448967	0.041414017	0.399217022	-0.103738105	0.917377187	0.959501353	0.041414017
ENSMUSG00000087687	Pet100	100503890	protein_coding	356.812572	-0.04142131	0.088882737	0.466021991	0.64119976	0.804106371	0.04142131
ENSMUSG00000019842	Traf3ip2	103213	protein_coding	333.0693412	0.0414376	0.08161961	-0.507691713	0.611669561	0.784979105	0.0414376
ENSMUSG00000047037	Nipa1	233280	protein_coding	47.81072901	-0.041442386	0.178474637	0.232203223	0.816380174	0.908409285	0.041442386
ENSMUSG00000038544	Inip	66209	protein_coding	197.2549459	0.041449739	0.08449238	-0.490573701	0.623727991	0.793440482	0.041449739
ENSMUSG00000036529	Sbf1	77980	protein_coding	1475.932471	0.041488273	0.068988448	-0.601379998	0.547586918	0.739145316	0.041488273
ENSMUSG00000021540	Smad5	17129	protein_coding	518.9970626	-0.041533426	0.132034121	0.314565856	0.753091307	0.876245321	0.041533426
ENSMUSG00000020482	Ccdc117	104479	protein_coding	322.1895079	-0.041536268	0.088971251	0.466850442	0.640606884	0.803690806	0.041536268
ENSMUSG00000022390	Zc3h7b	20286	protein_coding	525.9521777	0.041577674	0.062120078	-0.669311359	0.503296883	0.707899218	0.041577674
ENSMUSG00000026219	Trip12	14897	protein_coding	2751.211867	-0.041606352	0.051280263	0.811352146	0.417163472	0.637283772	0.041606352
ENSMUSG00000039233	Tbce	70430	protein_coding	242.0789388	-0.0416114	0.108304391	0.38420788	0.700824378	0.841825268	0.0416114
ENSMUSG00000103421	Golt1a	68338	protein_coding	43.41842828	0.041624257	0.169887471	-0.245010753	0.806448106	0.904795436	0.041624257
ENSMUSG00000020380	Rad50	19360	protein_coding	391.5765062	0.041636223	0.099085807	-0.4202037	0.674336652	0.825194181	0.041636223
ENSMUSG00000041073	Nacad	192950	protein_coding	73.23832666	-0.041679551	0.259036406	0.16090229	0.872170359	0.937028043	0.041679551

ensembl_gene_id	external_gene_name	entrezgene_id	gene_biotype	baseMean	log2FoldChange	lfcSE	stat	pvalue	padj	absLog2FC
ENSMUSG00000022656	Nectin3	58998	protein_coding	1380.410809	-0.041702626	0.096189893	0.433544784	0.664619039	0.819068567	0.041702626
ENSMUSG00000056035	Cyp3a11	13112	protein_coding	6.622515776	0.041702782	0.957557381	-0.04355121	0.965262144	0.982706396	0.041702782
ENSMUSG00000033721	Vav3	57257	protein_coding	218.1135615	0.041729755	0.097082261	-0.429839132	0.667312665	0.821223739	0.041729755
ENSMUSG00000062300	Nectin2	19294	protein_coding	1539.913486	-0.041733045	0.098488409	0.423735601	0.671758649	0.823993339	0.041733045
ENSMUSG00000102869	Norad	347740	lncRNA	1233.322402	0.041790136	0.122486502	-0.341181558	0.732966906	0.863168757	0.041790136
ENSMUSG00000027030	Stk39	53416	protein_coding	465.088394	-0.0418334	0.130225657	0.321237771	0.748030211	0.873310067	0.0418334
ENSMUSG00000034382	AI661453	224833	protein_coding	417.5778101	0.04188815	0.144945776	-0.288991864	0.772587604	0.885884716	0.04188815
ENSMUSG00000040236	Trappc5	66682	protein_coding	283.4208203	0.041922706	0.074715723	-0.561096167	0.574731981	0.760117839	0.041922706
ENSMUSG00000057236	Rbbp4	19646	protein_coding	1144.388345	0.041942589	0.1027991	-0.408005411	0.683269703	0.830769833	0.041942589
ENSMUSG00000038369	Ncoa6	56406	protein_coding	366.7004528	0.041943345	0.149994707	-0.279632169	0.779759724	0.890125936	0.041943345
ENSMUSG00000036167	Pphln1	223828	protein_coding	227.492626	0.041949591	0.085426534	-0.491060437	0.623383703	0.793353766	0.041949591
ENSMUSG00000059406	Tmprss9	432478	protein_coding	3.881028166	-0.041978167	0.809757949	0.051840389	0.958655873	1	0.041978167
ENSMUSG00000090110	Cmc4	105886298	protein_coding	17.20737053	-0.041987745	0.230270121	0.182341266	0.855314916	0.929560416	0.041987745
ENSMUSG00000040040	Ift88	21821	protein_coding	134.4653147	0.041990118	0.093660739	-0.448321447	0.653921224	0.812249349	0.041990118
ENSMUSG00000026648	Dclre1c	227525	protein_coding	95.8733748	-0.042004155	0.121200869	0.346566452	0.728917053	0.860116914	0.042004155
ENSMUSG00000041734	Kirrel	170643	protein_coding	6.441008468	0.04203288	0.711894991	-0.059043652	0.952917339	0.976632343	0.04203288
ENSMUSG00000029207	Apbb2	11787	protein_coding	254.7053901	-0.042037191	0.087738732	0.479117831	0.631854806	0.799444652	0.042037191
ENSMUSG00000041702	Btbd7	238386	protein_coding	943.8978678	-0.042080322	0.06065353	0.693781917	0.487818984	0.696186289	0.042080322
ENSMUSG00000017999	Ddx27	228889	protein_coding	446.4966693	-0.042119556	0.082342651	0.511515664	0.608990023	0.783067117	0.042119556
ENSMUSG00000029528	Pxn	19303	protein_coding	1771.562019	0.0421598	0.070900489	-0.594633422	0.552088534	0.742744607	0.0421598
ENSMUSG00000024442	Dele1	66839	protein_coding	519.3851828	-0.042224831	0.096663627	0.436822332	0.662240202	0.81729577	0.042224831
ENSMUSG00000049866	Arl4c	320982	protein_coding	13.03593617	0.042236506	0.350759834	-0.120414318	0.90415495	0.952610364	0.042236506
ENSMUSG00000050088	1600012H06Rik	67912	protein_coding	395.6213538	0.042265537	0.083770533	-0.50453943	0.613882361	0.78655252	0.042265537
ENSMUSG00000049672	Zbtb14	22666	protein_coding	169.0633892	-0.042293498	0.114750091	0.368570502	0.712447886	0.850598485	0.042293498
ENSMUSG00000041444	Arhgap32	330914	protein_coding	1742.38265	-0.042332408	0.068639637	0.616734139	0.5374101	0.732906636	0.042332408
ENSMUSG00000000142	Axin2	12006	protein_coding	371.478686	0.042348738	0.139211515	-0.304204273	0.76097227	0.880144002	0.042348738
ENSMUSG00000089874	9230117E06Rik	78082	lncRNA	1.371531985	0.042350893	1.284481848	-0.032971188	0.973697563	1	0.042350893
ENSMUSG00000026618	Iars2	381314	protein_coding	1538.983752	-0.042393791	0.091409595	0.463778344	0.64280656	0.805165923	0.042393791
ENSMUSG00000039756	Dnttip2	99480	protein_coding	450.3347881	-0.042430381	0.089661979	0.473226017	0.636051938	0.801630291	0.042430381
ENSMUSG00000096956	Snhg18	100616095	lncRNA	369.6357112	-0.04243755	0.122634189	0.346049908	0.729305208	0.86030736	0.04243755
ENSMUSG00000029843	Slc13a4	243755	protein_coding	2.698179346	0.042470677	1.666504354	-0.025484888	0.979668202	1	0.042470677
ENSMUSG00000020640	Itsn2	20403	protein_coding	1271.038871	-0.042480054	0.056378513	0.753479501	0.451161825	0.667666133	0.042480054
ENSMUSG00000031174	Rpgr	19893	protein_coding	77.2902948	0.042483263	0.127255828	-0.333841397	0.738499243	0.867333511	0.042483263
ENSMUSG00000026082	Rev1	56210	protein_coding	102.5416283	-0.04251491	0.132565784	0.320708018	0.748431671	0.873593484	0.04251491
ENSMUSG00000052296	Ppp6r1	243819	protein_coding	1827.344075	-0.042519152	0.049562474	0.857890024	0.390953193	0.613299234	0.042519152
ENSMUSG00000038721	Hoxb7	15415	protein_coding	91.88997522	0.042522516	0.246069838	-0.172806697	0.862803373	0.932166328	0.042522516
ENSMUSG00000087138	Gm15545	100502630	lncRNA	16.75115688	0.042537816	0.275735646	-0.154270283	0.877396628	0.939157889	0.042537816
ENSMUSG00000048251	Bcl11b	58208	protein_coding	294.3284053	-0.042553678	0.116604193	0.364941236	0.715155272	0.852123921	0.042553678
ENSMUSG00000044006	Cilp2	68709	protein_coding	3.281935893	0.042557359	1.281894189	-0.033198808	0.973516049	1	0.042557359

ensembl_gene_id	external_gene_name	entrezgene_id	gene_biotype	baseMean	log2FoldChange	lfcSE	stat	pvalue	padj	absLog2FC
ENSMUSG00000028786	Tmem54	66260	protein_coding	1597.344639	0.042579149	0.090826625	-0.468795897	0.639215533	0.803282676	0.042579149
ENSMUSG00000110086	Smim32	115488204	protein_coding	11.28324883	-0.042612387	0.307429303	0.138608735	0.88975934	0.945840699	0.042612387
ENSMUSG00000072571	Tmem253	619301	protein_coding	117.6058135	-0.042621982	0.150816892	0.282607479	0.777477747	0.888933284	0.042621982
ENSMUSG00000053684	Kics2	270802	protein_coding	32.67400262	-0.042626835	0.179389833	0.237621244	0.812174872	0.907147741	0.042626835
ENSMUSG00000015971	Actr8	56249	protein_coding	340.8433058	-0.042635034	0.067919803	0.627726116	0.530183374	0.727438372	0.042635034
ENSMUSG00000024853	Sf3b2	319322	protein_coding	2138.938131	-0.042636674	0.06563043	0.649647942	0.515919658	0.716883239	0.042636674
ENSMUSG00000045934	Mtmr11	194126	protein_coding	501.2978706	-0.04265778	0.161908845	0.263467876	0.79218998	0.896636371	0.04265778
ENSMUSG00000039936	Pik3cd	18707	protein_coding	40.04192858	0.042668937	0.34655062	-0.123124688	0.902008362	0.951530343	0.042668937
ENSMUSG00000036186	Dipk1b	56279	protein_coding	47.9995376	0.042676433	0.196688214	-0.216975039	0.828227798	0.913297021	0.042676433
ENSMUSG00000028668	Eloa	27224	protein_coding	961.0917449	-0.042701079	0.061554882	0.693707434	0.487865703	0.696186289	0.042701079
ENSMUSG00000020397	Med7	66213	protein_coding	155.2412162	0.042702409	0.114625541	-0.372538343	0.70949207	0.84834929	0.042702409
ENSMUSG00000024072	Yipf4	67864	protein_coding	583.2814623	0.042712894	0.111741455	-0.382247524	0.702277774	0.84294636	0.042712894
ENSMUSG00000020219	Timm13	30055	protein_coding	754.5271526	0.042729472	0.087104068	-0.490556556	0.62374012	0.793440482	0.042729472
ENSMUSG00000030347	D6Wsu163e	28040	protein_coding	164.7560529	0.04273884	0.119428889	-0.357860149	0.720447974	0.855204229	0.04273884
ENSMUSG00000040990	Sh3kbp1	58194	protein_coding	161.721298	0.042791168	0.181055362	-0.236343002	0.813166521	0.907217398	0.042791168
ENSMUSG00000086316	Nbdy	70123	protein_coding	60.01764469	0.042796584	0.19074394	-0.224366675	0.822471999	0.910171686	0.042796584
ENSMUSG00000089542	Gm25835	115487914	snoRNA	246.1698507	0.042816834	0.187084821	-0.228863217	0.81897523	0.90889719	0.042816834
ENSMUSG00000054843	Atrnl1	226255	protein_coding	1986.452605	-0.042902399	0.075678726	0.566901703	0.570780967	0.756249943	0.042902399
ENSMUSG00000004768	Rab23	19335	protein_coding	70.50102242	0.04290368	0.161570207	-0.265542026	0.790591939	0.895584805	0.04290368
ENSMUSG00000040843	Tiprl	226591	protein_coding	296.9076699	0.042917766	0.084023803	-0.510781047	0.609504382	0.783477444	0.042917766
ENSMUSG00000026798	Coq4	227683	protein_coding	151.2016363	-0.042991592	0.105238011	0.40851772	0.682893624	0.830618347	0.042991592
ENSMUSG00000108084	Gm7932	666105	lncRNA	13.43988509	0.043024715	0.290527304	-0.148091811	0.882270312	0.941973978	0.043024715
ENSMUSG00000091827	Speer4f2	100041749	protein_coding	0.914559436	0.043036239	2.287070191	-0.018817192	0.984986939	1	0.043036239
ENSMUSG00000026047	Poglut2	72050	protein_coding	91.44322005	0.043079423	0.124530139	-0.345935721	0.729391023	0.86030736	0.043079423
ENSMUSG00000032109	Nlrx1	270151	protein_coding	371.9870974	0.043086199	0.140087613	-0.307566084	0.758412531	0.878845311	0.043086199
ENSMUSG00000006262	Mob1b	68473	protein_coding	1096.299405	0.04308973	0.090434712	-0.476473341	0.633737196	0.800362194	0.04308973
ENSMUSG00000038250	Usp38	74841	protein_coding	499.4588503	0.043099135	0.092849557	-0.464182454	0.642517031	0.80495599	0.043099135
ENSMUSG00000028851	Nudc	18221	protein_coding	662.4261716	0.043102275	0.103591349	-0.416079869	0.67735156	0.827106157	0.043102275
ENSMUSG00000020435	Osbp2	74309	protein_coding	5.823101018	0.043115041	0.846426173	-0.050937745	0.959375128	0.980330479	0.043115041
ENSMUSG00000021796	Bmpr1a	12166	protein_coding	1252.688138	-0.043149081	0.085336725	0.505633202	0.613114169	0.786099952	0.043149081
ENSMUSG00000015092	Edf1	59022	protein_coding	2177.94877	-0.043169704	0.078913213	0.547052925	0.584342372	0.766711421	0.043169704
ENSMUSG00000068580	Zfyve19	72008	protein_coding	292.6248758	-0.043181297	0.070862143	0.609370465	0.54227891	0.735940424	0.043181297
ENSMUSG00000032612	Usp4	22258	protein_coding	1340.642495	-0.043182968	0.063681442	0.678109144	0.497702493	0.703115827	0.043182968
ENSMUSG00000062580	Timm17a	21854	protein_coding	613.1877017	0.043195278	0.081088911	-0.532690322	0.594247967	0.77323163	0.043195278
ENSMUSG00000029924	Slc37a3	72144	protein_coding	536.2576432	0.043218245	0.098550652	-0.438538394	0.660996044	0.816884691	0.043218245
ENSMUSG00000025423	Pias2	17344	protein_coding	404.5180564	0.043219029	0.08433661	-0.512458695	0.608330022	0.782626881	0.043219029
ENSMUSG00000022974	Paxbp1	67367	protein_coding	396.2719077	0.043262737	0.115363906	-0.375011025	0.707652267	0.846946626	0.043262737
ENSMUSG00000026781	Acbd5	74159	protein_coding	928.6399199	-0.043282933	0.078906258	0.548536124	0.583323832	0.765922697	0.043282933
ENSMUSG00000022371	Col14a1	12818	protein_coding	5.78303575	-0.043482801	0.90011214	0.048308205	0.961470616	0.981136115	0.043482801

ensembl_gene_id	external_gene_name	entrezgene_id	gene_biotype	baseMean	log2FoldChange	lfcSE	stat	pvalue	padj	absLog2FC
ENSMUSG00000068391	Chrac1	93696	protein_coding	179.4573957	-0.043499528	0.101811933	0.427253727	0.669194532	0.822558876	0.043499528
ENSMUSG00000057691	Zfp746	69228	protein_coding	381.3481077	-0.043499627	0.062074815	0.700761286	0.483452002	0.693875647	0.043499627
ENSMUSG00000038095	Sbno1	243272	protein_coding	1507.974933	-0.043525207	0.071724728	0.606836842	0.543959196	0.73666903	0.043525207
ENSMUSG00000035152	Ap2b1	71770	protein_coding	2105.976682	-0.043526025	0.083057676	0.524045787	0.600246694	0.77680602	0.043526025
ENSMUSG00000036114	Rpp25l	69961	protein_coding	204.9060122	-0.043548025	0.094794724	0.459392919	0.645952032	0.807082912	0.043548025
ENSMUSG00000014245	Pigl	327942	protein_coding	74.88668916	-0.043643278	0.132490422	0.329407042	0.741848046	0.869560398	0.043643278
ENSMUSG00000026749	Nek6	59126	protein_coding	821.4336718	0.043647733	0.084563951	-0.516150586	0.605749239	0.780657323	0.043647733
ENSMUSG00000032475	Nck1	17973	protein_coding	346.2868404	-0.043667239	0.08964703	0.487101898	0.626186121	0.795381772	0.043667239
ENSMUSG00000030431	Tmem238	664968	protein_coding	276.5064705	0.043735732	0.151566435	-0.288558163	0.772919515	0.886203768	0.043735732
ENSMUSG00000033933	Vhl	22346	protein_coding	206.4146817	-0.043774025	0.081411392	0.537689185	0.590791647	0.771232643	0.043774025
ENSMUSG00000042229	Rabif	98710	protein_coding	332.1493859	0.043777291	0.108336158	-0.404087533	0.686148353	0.832872319	0.043777291
ENSMUSG00000021557	Agtpbp1	67269	protein_coding	63.29970036	0.043789079	0.155109632	-0.28231051	0.777705428	0.888933284	0.043789079
ENSMUSG00000043333	Rhbdl2	230726	protein_coding	510.3034724	-0.043807791	0.105155553	0.416599886	0.676971093	0.826943172	0.043807791
ENSMUSG00000022370	Mrpl13	68537	protein_coding	311.2963923	-0.043830918	0.087970069	0.498248078	0.618309199	0.789606655	0.043830918
ENSMUSG00000028030	Tbck	271981	protein_coding	489.7233539	-0.043889696	0.07173615	0.611821177	0.540656077	0.734843633	0.043889696
ENSMUSG00000028431	Elp1	230233	protein_coding	302.4024951	0.04390138	0.110735764	-0.396451681	0.691771847	0.835998557	0.04390138
ENSMUSG00000024033	Rsph1	22092	protein_coding	15.08614931	-0.043912336	0.299086791	0.146821383	0.883273006	0.942211125	0.043912336
ENSMUSG00000086714	0610009E02Rik	100125929	lncRNA	7.583474848	0.043932232	0.412831978	-0.106416738	0.915251714	0.95833356	0.043932232
ENSMUSG00000034101	Ctnnd1	12388	protein_coding	831.9623403	0.043935492	0.082465173	-0.53277632	0.594188428	0.77323163	0.043935492
ENSMUSG00000053134	Supt7l	72195	protein_coding	201.9525074	0.043990668	0.10321372	-0.426209503	0.669955194	0.822722373	0.043990668
ENSMUSG00000000078	Klf6	23849	protein_coding	7142.565263	-0.04401657	0.172495988	0.255174456	0.798588359	0.900012841	0.04401657
ENSMUSG00000086391	1700042O10Rik	73321	lncRNA	2.57431512	0.044085524	0.742283079	-0.059391795	0.952640048	1	0.044085524
ENSMUSG00000004996	Mri1	67873	protein_coding	198.5004799	-0.044109369	0.100271775	0.439898154	0.660010873	0.816323585	0.044109369
ENSMUSG00000025008	Tctn3	67590	protein_coding	54.21534624	0.0441477	0.153239131	-0.288096775	0.773272661	0.886362513	0.0441477
ENSMUSG00000001482	Def8	23854	protein_coding	244.3035699	0.044150999	0.073259978	-0.602661925	0.546733616	0.738518372	0.044150999
ENSMUSG00000001100	Poldip2	67811	protein_coding	655.6451439	0.04419288	0.088672232	-0.498384651	0.618212953	0.789572213	0.04419288
ENSMUSG00000027907	S100a11	20195	protein_coding	307.8448088	0.044243037	0.123472538	-0.358322895	0.720101686	0.855153117	0.044243037
ENSMUSG00000002608	Ccdc97	52132	protein_coding	233.6375721	-0.044280732	0.106551061	0.415582276	0.677715699	0.827306109	0.044280732
ENSMUSG00000032328	Tmem30a	69981	protein_coding	1584.013196	0.044295052	0.076360104	-0.580081078	0.561859943	0.750140815	0.044295052
ENSMUSG00000057278	Snrpg	68011	protein_coding	220.577199	-0.044297035	0.141091382	0.313959888	0.753551504	0.876528151	0.044297035
ENSMUSG00000017801	Mlx	21428	protein_coding	1204.162923	-0.044321434	0.110220045	0.402117728	0.687597386	0.833638003	0.044321434
ENSMUSG00000034227	Foxj1	15223	protein_coding	3.005990548	0.044324184	0.626821262	-0.070712637	0.943626463	1	0.044324184
ENSMUSG00000044317	Gpr4	319197	protein_coding	8.920763787	0.044331964	0.429496065	-0.103218558	0.917789512	0.959689482	0.044331964
ENSMUSG00000071647	Eml3	225898	protein_coding	283.9416586	0.044332856	0.06875219	-0.644821004	0.519043189	0.719334577	0.044332856
ENSMUSG00000024027	Glp1r	14652	protein_coding	14.50486651	0.044339225	0.423549649	-0.104684835	0.916625896	0.959274295	0.044339225
ENSMUSG00000024132	Eci1	13177	protein_coding	618.0241444	-0.044353019	0.103156111	0.429960172	0.667224613	0.821176574	0.044353019
ENSMUSG00000071477	Zfp777	72306	protein_coding	244.9605466	-0.044381637	0.073443163	0.604299098	0.545644806	0.737439915	0.044381637
ENSMUSG00000001552	Jup	16480	protein_coding	3801.723192	0.04446186	0.103548182	-0.429383298	0.667644307	0.821350533	0.04446186
ENSMUSG00000020982	Nemf	66244	protein_coding	625.5727049	0.044485422	0.06320254	-0.703854964	0.481523101	0.692354022	0.044485422

ensembl_gene_id	external_gene_name	entrezgene_id	gene_biotype	baseMean	log2FoldChange	lfcSE	stat	pvalue	padj	absLog2FC
ENSMUSG00000024481	Lvrn	74574	protein_coding	1.212300783	0.044517408	1.949632944	-0.022833738	0.981782896	1	0.044517408
ENSMUSG00000085385	Snhg17	68108	lncRNA	116.4128262	-0.044531457	0.147256267	0.302407891	0.76234114	0.88093126	0.044531457
ENSMUSG00000085408	C530005A16Rik	654318	lncRNA	9.007585209	0.044569538	0.365077703	-0.122082332	0.902833814	0.95200802	0.044569538
ENSMUSG00000024897	Apba1	319924	protein_coding	8.516515362	0.044597914	0.677022864	-0.065873571	0.947478482	0.974566157	0.044597914
ENSMUSG00000010406	Mrpl52	68836	protein_coding	507.6556446	0.044666641	0.104759858	-0.426371721	0.669837004	0.822722373	0.044666641
ENSMUSG00000018651	Tada2a	217031	protein_coding	103.2184097	0.044670688	0.1175001	-0.380175747	0.703814961	0.844104331	0.044670688
ENSMUSG00000038132	Rbm24	666794	protein_coding	3.029333524	-0.04468744	0.816077708	0.054758804	0.95633062	1	0.04468744
ENSMUSG00000029135	Fosl2	14284	protein_coding	3196.581954	0.044721417	0.133768252	-0.334320115	0.738138013	0.866982505	0.044721417
ENSMUSG00000016319	Slc25a5	11740	protein_coding	13756.28303	0.044776799	0.096891911	-0.462131442	0.643987064	0.80597323	0.044776799
ENSMUSG00000024293	Esco1	77805	protein_coding	494.1642558	0.044784593	0.091165221	-0.491246471	0.623252135	0.793308598	0.044784593
ENSMUSG00000041303	Gtf3c3	98488	protein_coding	311.9224918	0.04480071	0.11455063	-0.391099635	0.69572359	0.83856979	0.04480071
ENSMUSG00000025451	Paip1	218693	protein_coding	566.6489282	0.044856845	0.084243832	-0.532464437	0.594404366	0.77323163	0.044856845
ENSMUSG00000037210	Fam193a	231128	protein_coding	441.1765218	0.044877696	0.091769601	-0.489025726	0.624823484	0.79451259	0.044877696
ENSMUSG00000030583	Sipa1l3	74206	protein_coding	1161.898356	-0.044886511	0.186903495	0.240158757	0.810207185	0.906158059	0.044886511
ENSMUSG00000015597	Zfp318	57908	protein_coding	232.6466497	0.044934582	0.084310587	-0.532964886	0.594057888	0.773192935	0.044934582
ENSMUSG00000035601	Trmt10b	69934	protein_coding	115.6779227	0.044939354	0.106796142	-0.420795671	0.673904293	0.825096143	0.044939354
ENSMUSG00000040322	Slc25a24	229731	protein_coding	4125.357335	-0.04494494	0.099893233	0.449929779	0.652761075	0.811601896	0.04494494
ENSMUSG00000031592	Pcm1	18536	protein_coding	897.5454437	-0.044955719	0.068710822	0.654274207	0.51293516	0.714956306	0.044955719
ENSMUSG00000027770	Dhx36	72162	protein_coding	582.2647813	-0.045010142	0.094558143	0.476004927	0.634070868	0.800507769	0.045010142
ENSMUSG00000090330	9130221H12Rik	77124	lncRNA	32.12441167	0.04502611	0.190402257	-0.236478867	0.813061104	0.907217398	0.04502611
ENSMUSG00000087259	2610035D17Rik	72386	lncRNA	280.3508375	0.04504154	0.169417406	-0.265861348	0.790345994	0.895392916	0.04504154
ENSMUSG00000028114	Mettl14	210529	protein_coding	173.0047697	-0.045072227	0.112557715	0.400436583	0.688834984	0.833904116	0.045072227
ENSMUSG00000025538	Sumf2	67902	protein_coding	62.68645906	-0.045086016	0.158823038	0.283875793	0.776505568	0.888506311	0.045086016
ENSMUSG00000024908	Ppp6r3	52036	protein_coding	2261.466381	-0.04509059	0.066503635	0.678016929	0.49776096	0.703115827	0.04509059
ENSMUSG00000044452	Zfp507	668501	protein_coding	417.8848172	-0.04509484	0.102945608	0.438045302	0.661353444	0.816921981	0.04509484
ENSMUSG00000061666	Gdpd1	66569	protein_coding	1173.649685	-0.045173766	0.113353783	0.398520148	0.69024681	0.834766446	0.045173766
ENSMUSG00000043648	Pld6	194908	protein_coding	2.868979415	-0.045173834	0.644638692	0.070076207	0.944133005	1	0.045173834
ENSMUSG00000030849	Fgfr2	14183	protein_coding	691.5289067	-0.045193244	0.127248054	0.355158628	0.722470757	0.856426823	0.045193244
ENSMUSG00000038902	Pogz	229584	protein_coding	188.9148655	0.045244576	0.112418629	-0.402465112	0.687341759	0.833389267	0.045244576
ENSMUSG00000033257	Ttll4	67534	protein_coding	355.7589674	0.045266841	0.089713047	-0.50457367	0.613858307	0.78655252	0.045266841
ENSMUSG00000020238	Ncln	103425	protein_coding	1130.261013	0.045291045	0.062609232	-0.72339244	0.469438815	0.683156904	0.045291045
ENSMUSG00000021288	Klc1	16593	protein_coding	879.0531191	-0.045307723	0.103557467	0.437512857	0.661739454	0.816921981	0.045307723
ENSMUSG00000028405	Aco1	11428	protein_coding	1492.284116	-0.045312223	0.088972206	0.509285151	0.610552365	0.784161879	0.045312223
ENSMUSG00000059305	Vpreb1	22362	protein_coding	2.868567947	0.045328285	0.772878665	-0.058648644	0.953231965	1	0.045328285
ENSMUSG00000106718	Gm20052	102637176	lncRNA	1.121769154	0.04533231	1.99335447	-0.02274172	0.981856296	1	0.04533231
ENSMUSG00000030615	Tmem126a	66271	protein_coding	279.1545126	0.045339122	0.081295169	-0.557709915	0.577042486	0.761298159	0.045339122
ENSMUSG00000035245	Eogt	101351	protein_coding	174.9575124	-0.045353385	0.093351158	0.48583634	0.627083207	0.795976331	0.045353385
ENSMUSG00000060260	Pwwp2b	101631	protein_coding	219.7169153	-0.045469352	0.093174016	0.488004636	0.625546557	0.794916857	0.045469352
ENSMUSG00000061762	Tac1	21333	protein_coding	39.94273795	-0.045491951	0.209983565	0.216645292	0.828484786	0.913446239	0.045491951

Supplementary Table 1

ensembl_gene_id	external_gene_name	entrezgene_id	gene_biotype	baseMean	log2FoldChange	lfcSE	stat	pvalue	padj	absLog2FC
ENSMUSG00000028700	Pomgnt1	68273	protein_coding	515.3519249	-0.045524929	0.069737762	0.652801688	0.513884135	0.715629739	0.045524929
ENSMUSG00000022450	Ndufa6	67130	protein_coding	2149.131824	0.045548285	0.091860804	-0.495840258	0.620007119	0.791007228	0.045548285
ENSMUSG00000028741	Mrto4	69902	protein_coding	166.1042923	-0.045605692	0.16443329	0.277350724	0.781510814	0.890612766	0.045605692
ENSMUSG00000030335	Mrpl51	66493	protein_coding	877.8613607	0.045613747	0.088852371	-0.513365556	0.607695635	0.782250036	0.045613747
ENSMUSG00000039628	Hs3st6	328779	protein_coding	0.73548046	-0.045708479	1.717482718	0.026613647	0.978767888	1	0.045708479
ENSMUSG00000019731	Slc35e1	270066	protein_coding	1512.596385	-0.045712295	0.045379545	1.00733258	0.313774978	0.535013399	0.045712295
ENSMUSG00000024436	Mrps18b	66973	protein_coding	184.5457018	0.045729328	0.095528892	-0.478696306	0.632154694	0.799512805	0.045729328
ENSMUSG00000028403	Zdhhc21	68268	protein_coding	894.8452555	-0.045741334	0.081960523	0.558089834	0.576783042	0.761222618	0.045741334
ENSMUSG00000021504	B4galt7	218271	protein_coding	199.6490538	0.045793305	0.081859307	-0.559414766	0.575878686	0.760926598	0.045793305
ENSMUSG00000003135	Cnot11	52846	protein_coding	311.812095	0.045796223	0.077561074	-0.590453701	0.554886517	0.744703672	0.045796223
ENSMUSG00000014606	Slc25a11	67863	protein_coding	1304.307442	0.045816181	0.089511008	-0.511849686	0.608756214	0.782882962	0.045816181
ENSMUSG00000024430	Cabyr	71132	protein_coding	2.532361036	-0.045821259	1.026794361	0.044625546	0.96440578	1	0.045821259
ENSMUSG00000022329	Stk3	56274	protein_coding	295.9587441	-0.045874478	0.072175509	0.635596192	0.525039652	0.723506743	0.045874478
ENSMUSG00000058388	Phtf1	18685	protein_coding	154.2405022	0.045875341	0.117360542	-0.390892376	0.695876789	0.838607402	0.045875341
ENSMUSG00000002524	Puf60	67959	protein_coding	1285.879775	0.045878457	0.087179981	-0.526249914	0.598714578	0.776186935	0.045878457
ENSMUSG00000045098	Kmt5b	225888	protein_coding	805.4033878	-0.045884171	0.08851597	0.518371669	0.604198978	0.779660446	0.045884171
ENSMUSG00000037062	Sh3glb1	54673	protein_coding	1353.226895	-0.045894203	0.076484688	0.600044327	0.548476694	0.739637131	0.045894203
ENSMUSG00000029571	Tmem106b	71900	protein_coding	830.7058683	-0.045896755	0.118250775	0.388130691	0.697919321	0.839976943	0.045896755
ENSMUSG00000005656	Snx6	72183	protein_coding	613.4937303	-0.045959911	0.085195705	0.53946277	0.589567579	0.770506411	0.045959911
ENSMUSG00000025337	Sbds	66711	protein_coding	929.4541527	-0.04598212	0.073932284	0.621949126	0.533975321	0.730224807	0.04598212
ENSMUSG00000034781	Gna11	14672	protein_coding	3849.034124	-0.046009622	0.126393198	0.364019763	0.715843251	0.852575063	0.046009622
ENSMUSG00000042502	Cd2bp2	70233	protein_coding	579.6943179	-0.046014757	0.064131132	0.717510444	0.473059194	0.685332006	0.046014757
ENSMUSG00000024081	Cebpz	12607	protein_coding	408.8416885	-0.046046933	0.126552378	0.363856721	0.715965004	0.852575063	0.046046933
ENSMUSG00000005871	Apc	11789	protein_coding	1700.168744	0.046103368	0.06824224	-0.675584031	0.499304783	0.704201299	0.046103368
ENSMUSG00000042396	Rbm7	67010	protein_coding	615.035543	0.046115593	0.077238111	-0.597057488	0.550469	0.741471864	0.046115593
ENSMUSG00000038145	Snrk	20623	protein_coding	718.945101	-0.046117388	0.139152837	0.331415364	0.740330762	0.868496285	0.046117388
ENSMUSG00000042189	Tekt3	71062	protein_coding	1.834224334	-0.046119424	1.30501923	0.035340034	0.971808601	1	0.046119424
ENSMUSG00000089876	Tmem102	380705	protein_coding	390.0597871	-0.046145453	0.0999614	0.46163272	0.644344727	0.806175378	0.046145453
ENSMUSG00000100199	Gm20324	100504637	lncRNA	13.64636787	0.046161522	0.337085225	-0.136943178	0.891075709	0.946708891	0.046161522
ENSMUSG00000032178	Ilf3	16201	protein_coding	402.3168446	-0.046164298	0.104694446	0.440943147	0.659254156	0.816110122	0.046164298
ENSMUSG00000085486	Ccdc200	667306	protein_coding	0.699327368	-0.04616548	1.494063146	0.030899283	0.975349862	1	0.04616548
ENSMUSG00000022111	Uchl3	50933	protein_coding	220.0731081	-0.046190048	0.102422685	0.450974786	0.652007721	0.811153798	0.046190048
ENSMUSG00000019951	Uhrf1bp1l	75089	protein_coding	570.3516433	0.046192479	0.087598353	-0.527321317	0.597970473	0.77577849	0.046192479
ENSMUSG00000071234	Syndig1l	627191	protein_coding	3.022144569	0.04622482	0.892825036	-0.05177366	0.958709044	1	0.04622482
ENSMUSG00000029613	Eif2ak1	15467	protein_coding	531.2195083	-0.046241246	0.071876513	0.643342918	0.520001614	0.720115363	0.046241246
ENSMUSG00000049300	Prmt6	99890	protein_coding	97.90918897	-0.046300311	0.117811487	0.393003369	0.694316993	0.837885199	0.046300311
ENSMUSG00000058655	Eif4b	75705	protein_coding	4476.274837	-0.046322659	0.087261713	0.530847459	0.59552449	0.773864504	0.046322659
ENSMUSG00000002320	Tm9sf1	74140	protein_coding	1046.192045	0.046329938	0.096744887	-0.478887719	0.632018508	0.799444652	0.046329938
ENSMUSG00000039285	Azi2	27215	protein_coding	1384.778526	-0.046336836	0.062293256	0.743849959	0.456967252	0.671862287	0.046336836

Supplementary Table 1

ensembl_gene_id	external_gene_name	entrezgene_id	gene_biotype	baseMean	log2FoldChange	lfcSE	stat	pvalue	padj	absLog2FC
ENSMUSG00000041997	Tlk1	228012	protein_coding	1092.081191	-0.046372767	0.105419718	0.439887039	0.660018924	0.816323585	0.046372767
ENSMUSG00000021111	Papola	18789	protein_coding	1643.447509	0.046396586	0.078235099	-0.593040553	0.553154013	0.743572273	0.046396586
ENSMUSG00000036188	Ankmy2	217473	protein_coding	385.6970401	-0.046400416	0.103494931	0.448335161	0.653911329	0.812249349	0.046400416
ENSMUSG00000117713	Gm46637	108168397	lncRNA	1.814432718	-0.046418157	1.097467588	0.042295697	0.966262975	1	0.046418157
ENSMUSG00000020758	Itgb4	192897	protein_coding	2307.714954	-0.046460253	0.081611024	0.569288938	0.569160072	0.754948549	0.046460253
ENSMUSG00000021000	Mia2	338320	protein_coding	1257.758648	-0.046492238	0.05818617	0.799025585	0.424275577	0.643978573	0.046492238
ENSMUSG00000034621	Gpatch8	237943	protein_coding	651.3406688	0.046502312	0.131326731	-0.35409632	0.7232667	0.856549354	0.046502312
ENSMUSG00000047638	Nr1h4	20186	protein_coding	1471.675918	0.046544324	0.160243334	-0.290460282	0.771464132	0.885228942	0.046544324
ENSMUSG00000030101	Sumf1	58911	protein_coding	395.6543987	0.046648536	0.068955496	-0.676502071	0.498721932	0.703739933	0.046648536
ENSMUSG00000020778	Ten1	69535	protein_coding	205.9616663	0.0466692	0.110231586	-0.423374111	0.67202233	0.824189967	0.0466692
ENSMUSG00000038696	Mapkap1	227743	protein_coding	903.0722465	0.046699412	0.071141974	-0.65642559	0.51155033	0.71433639	0.046699412
ENSMUSG00000029229	Chic2	74277	protein_coding	211.9800798	-0.046708172	0.112865906	0.413837741	0.678992942	0.827951097	0.046708172
ENSMUSG00000097537	2610020C07Rik	69918	lncRNA	12.66306697	0.046748972	0.299870659	-0.155897119	0.876114116	0.938630585	0.046748972
ENSMUSG00000033653	Vps8	209018	protein_coding	292.6589925	0.046756465	0.07462318	-0.626567574	0.530942729	0.728048657	0.046756465
ENSMUSG00000028636	Ppcs	106564	protein_coding	268.9091024	-0.046787368	0.09371762	0.499237688	0.617611947	0.789415146	0.046787368
ENSMUSG00000020415	Pttg1	30939	protein_coding	267.6518431	0.046791849	0.084622508	-0.552948024	0.580299001	0.76356297	0.046791849
ENSMUSG00000028106	Rprd2	75137	protein_coding	496.5423049	0.046792797	0.093803073	-0.498840766	0.617891566	0.789468115	0.046792797
ENSMUSG00000039195	Bbln	73737	protein_coding	136.5140402	0.046804991	0.09770805	-0.479029015	0.631917987	0.799444652	0.046804991
ENSMUSG00000022214	Dcaf11	28199	protein_coding	1230.547606	0.046843559	0.056608773	-0.827496461	0.407955727	0.628624818	0.046843559
ENSMUSG00000027668	Mfn1	67414	protein_coding	773.8224423	0.046851619	0.065974371	-0.710148779	0.47761188	0.68930064	0.046851619
ENSMUSG00000033295	Ptprf	19268	protein_coding	5339.622355	0.046865271	0.051077789	-0.9175274	0.358866342	0.581581822	0.046865271
ENSMUSG00000022272	Myo10	17909	protein_coding	1717.865139	0.04688266	0.061842235	-0.758100997	0.448390523	0.665277305	0.04688266
ENSMUSG00000019054	Fis1	66437	protein_coding	861.1189941	-0.046911992	0.072978184	0.642822136	0.520339518	0.720291626	0.046911992
ENSMUSG00000020115	Tbk1	56480	protein_coding	509.4932014	-0.046944077	0.066417109	0.706806987	0.479686432	0.690909666	0.046944077
ENSMUSG00000027479	Mapre1	13589	protein_coding	1099.860583	0.046950346	0.078286647	-0.599723551	0.54869049	0.739800355	0.046950346
ENSMUSG00000001847	Rac1	19353	protein_coding	3106.654676	-0.046965088	0.062873424	0.746978369	0.455076618	0.670515281	0.046965088
ENSMUSG00000033916	Chmp2a	68953	protein_coding	1542.473883	-0.046969971	0.066981267	0.701240408	0.483152996	0.693738201	0.046969971
ENSMUSG00000064370	mt-Cytb	17711	protein_coding	78985.31774	0.046981274	0.465238557	-0.100983191	0.919563804	0.96099713	0.046981274
ENSMUSG00000059974	Ntm	235106	protein_coding	3.659531686	-0.046988221	1.023927474	0.045890184	0.963397778	1	0.046988221
ENSMUSG00000054237	Fra10ac1	70567	protein_coding	108.6580335	0.047016808	0.122438481	-0.384003524	0.700975836	0.841852283	0.047016808
ENSMUSG00000027575	Arfgap1	228998	protein_coding	762.3291882	0.047054219	0.060325396	-0.7800068	0.435386872	0.653693864	0.047054219
ENSMUSG00000021240	Abcd4	19300	protein_coding	130.8221782	0.047068275	0.125887222	-0.373892399	0.708484372	0.847573958	0.047068275
ENSMUSG00000024079	Eif2ak2	19106	protein_coding	603.5542509	0.047081549	0.10854648	-0.433745516	0.66447325	0.818950015	0.047081549
ENSMUSG00000070473	Cldn3	12739	protein_coding	7191.229362	-0.047091336	0.100520267	0.468476035	0.639444205	0.803370142	0.047091336
ENSMUSG00000033826	Dnah8	13417	protein_coding	379.9825071	-0.0471231	0.189340607	0.248880053	0.803453565	0.903013746	0.0471231
ENSMUSG00000048756	Foxo3	56484	protein_coding	2028.738699	-0.047124385	0.140902787	0.334446081	0.738042972	0.866982505	0.047124385
ENSMUSG00000030942	Thumpd1	233802	protein_coding	347.9742012	0.04717634	0.114165543	-0.41322748	0.679439956	0.828120806	0.04717634
ENSMUSG00000041362	Shtn1	71653	protein_coding	610.7565979	-0.047202744	0.068916335	0.684928243	0.493389203	0.699749347	0.047202744
ENSMUSG00000070332	Trim80	432613	protein_coding	2.828269179	0.047217835	0.951188288	-0.049640892	0.96040856	1	0.047217835

ensembl_gene_id	external_gene_name	entrezgene_id	gene_biotype	baseMean	log2FoldChange	lfcSE	stat	pvalue	padj	absLog2FC
ENSMUSG00000106918	Mrpl33	66845	protein_coding	276.115692	0.047222569	0.06221633	-0.759006024	0.447848953	0.664930223	0.047222569
ENSMUSG00000042508	Dmtf1	23857	protein_coding	312.548522	-0.047225954	0.074666458	0.632492217	0.527065293	0.725511065	0.047225954
ENSMUSG00000024387	Csnk2b	13001	protein_coding	824.6334087	-0.047242881	0.056245542	0.839939996	0.400942031	0.622737045	0.047242881
ENSMUSG00000020152	Actr2	66713	protein_coding	2725.701425	0.047256835	0.076886386	-0.614632018	0.538797764	0.733468492	0.047256835
ENSMUSG00000073616	Cops9	66915	protein_coding	546.9661948	-0.047266071	0.099600136	0.474558292	0.63510184	0.801105392	0.047266071
ENSMUSG00000007817	Zmiz1	328365	protein_coding	825.7428542	-0.047269506	0.298233177	0.158498483	0.874064016	0.938083652	0.047269506
ENSMUSG00000028385	Snx30	209131	protein_coding	210.6678967	-0.047276657	0.126022756	0.375143811	0.707553516	0.846946626	0.047276657
ENSMUSG00000033272	Slc35a4	67843	protein_coding	695.34763	0.047291452	0.09024819	-0.524015513	0.60026775	0.77680602	0.047291452
ENSMUSG00000045664	Cdc42ep2	104252	protein_coding	161.0442645	0.047293877	0.238401883	-0.198378789	0.842748711	0.922154269	0.047293877
ENSMUSG00000075592	Nynrin	277154	protein_coding	34.07726207	0.047313562	0.210928168	-0.224311253	0.822515121	0.910171686	0.047313562
ENSMUSG00000031988	Vps26b	69091	protein_coding	608.7298483	0.047328472	0.075569781	-0.626288333	0.531125837	0.728066081	0.047328472
ENSMUSG00000040365	Trim41	211007	protein_coding	539.0201825	0.047344629	0.059397091	-0.797086663	0.425400702	0.644974953	0.047344629
ENSMUSG00000063904	Dpp3	75221	protein_coding	1819.678253	-0.047368559	0.06888248	0.687672093	0.491659297	0.698491194	0.047368559
ENSMUSG00000044715	Gskip	66787	protein_coding	175.0804953	-0.047381531	0.102133535	0.463917464	0.64270688	0.805102133	0.047381531
ENSMUSG00000047459	Dynlrb1	67068	protein_coding	568.1729339	-0.047396376	0.068501278	0.69190499	0.488996997	0.696686775	0.047396376
ENSMUSG00000027425	Kat14	228714	protein_coding	367.405923	-0.047428779	0.063255964	0.749791413	0.453380341	0.669150322	0.047428779
ENSMUSG00000086712	Mexis	381524	lncRNA	16.95192891	-0.047478988	0.256187489	0.185329066	0.852970941	0.92788168	0.047478988
ENSMUSG00000026705	Klhl20	226541	protein_coding	131.6826506	-0.047506375	0.091050303	0.521759659	0.601837681	0.77804505	0.047506375
ENSMUSG00000038838	Vars2	68915	protein_coding	413.874439	0.047511513	0.086573469	-0.548799924	0.583142763	0.765756487	0.047511513
ENSMUSG00000024240	Epc1	13831	protein_coding	370.5920365	-0.047516328	0.095964679	0.495143926	0.620498529	0.791450739	0.047516328
ENSMUSG00000052151	Plpp2	50784	protein_coding	2257.524969	-0.047518007	0.102903351	0.461773173	0.644243991	0.806110415	0.047518007
ENSMUSG00000017686	Rhot1	59040	protein_coding	438.2298768	0.047519663	0.071174723	-0.667648019	0.504358298	0.708596728	0.047519663
ENSMUSG00000042354	Gnl3	30877	protein_coding	284.1088853	0.047521426	0.13556444	-0.350544923	0.725929782	0.858172551	0.047521426
ENSMUSG00000031513	Leprotl1	68192	protein_coding	490.885223	-0.047525192	0.077375597	0.614214222	0.539073777	0.733662794	0.047525192
ENSMUSG00000024858	Grk2	110355	protein_coding	1180.37282	0.0475318	0.03937735	-1.207084794	0.22739949	0.43599843	0.0475318
ENSMUSG00000035142	Nubpl	76826	protein_coding	89.54622824	-0.04754577	0.1396337	0.340503543	0.733477355	0.863523565	0.04754577
ENSMUSG00000044894	Uqcrq	22272	protein_coding	1838.756021	0.047548839	0.109755295	-0.433225926	0.664850646	0.819267308	0.047548839
ENSMUSG00000024477	Pggt1b	225467	protein_coding	615.3453384	-0.047554787	0.09692363	0.490641829	0.623679796	0.793440482	0.047554787
ENSMUSG00000032501	Trib1	211770	protein_coding	1772.429127	0.047563414	0.169496104	-0.28061656	0.779004513	0.889721317	0.047563414
ENSMUSG00000025779	Ly96	17087	protein_coding	37.3801427	-0.047572634	0.202783001	0.234598728	0.814520197	0.907746426	0.047572634
ENSMUSG00000044857	Lemd2	224640	protein_coding	512.7588254	0.047582818	0.07529911	-0.631917404	0.52744085	0.725788994	0.047582818
ENSMUSG00000004626	Stxbp2	20911	protein_coding	1123.155784	-0.047605443	0.05134158	0.927229811	0.353807228	0.576210312	0.047605443
ENSMUSG00000024619	Cdx1	12590	protein_coding	3646.628501	-0.047606128	0.088012941	0.540899181	0.588577075	0.769577776	0.047606128
ENSMUSG00000052142	Rasal3	320484	protein_coding	12.94421837	-0.047650937	0.429755421	0.110879199	0.911712141	0.956997404	0.047650937
ENSMUSG00000044221	Grsf1	231413	protein_coding	685.5788848	0.047695216	0.092614448	-0.514986772	0.606562264	0.781400205	0.047695216
ENSMUSG00000087475	4933406I18Rik	71045	lncRNA	1.537798909	0.04771295	1.032737219	-0.046200475	0.963150464	1	0.04771295
ENSMUSG00000061288	Taok3	330177	protein_coding	872.8916586	0.047738536	0.109351348	-0.43656102	0.662429737	0.817346435	0.047738536
ENSMUSG00000055413	H2-Q5	15016	protein_coding	6.054764816	0.04777577	0.613898161	-0.077823608	0.937968367	0.970517013	0.04777577
ENSMUSG00000028848	Gpn2	100210	protein_coding	165.4536528	-0.047849465	0.084226503	0.568104613	0.569963936	0.755650645	0.047849465

ensembl_gene_id	external_gene_name	entrezgene_id	gene_biotype	baseMean	log2FoldChange	lfcSE	stat	pvalue	padj	absLog2FC
ENSMUSG00000041341	Atg2b	76559	protein_coding	561.4035487	-0.047854781	0.0564652	0.847509275	0.396711319	0.618855346	0.047854781
ENSMUSG00000029715	Pop7	74097	protein_coding	183.2419054	0.047858231	0.096339724	-0.49676529	0.619354575	0.790541154	0.047858231
ENSMUSG00000030059	Tmf1	232286	protein_coding	1552.40419	-0.047891953	0.07101713	0.674371836	0.500074944	0.704748047	0.047891953
ENSMUSG00000022193	Psmb5	19173	protein_coding	605.938988	0.047901874	0.110114455	-0.435018943	0.663548678	0.818482437	0.047901874
ENSMUSG00000023994	Nfya	18044	protein_coding	342.8471929	-0.047920122	0.082855064	0.578360811	0.563020546	0.750768863	0.047920122
ENSMUSG00000054000	Tusc1	69136	protein_coding	46.05526213	0.047938046	0.216902991	-0.221011458	0.825083512	0.911460908	0.047938046
ENSMUSG00000019518	Ap4m1	11781	protein_coding	264.9355203	-0.047939875	0.075945214	0.631242877	0.527881731	0.726026078	0.047939875
ENSMUSG00000030364	Clec2h	94071	protein_coding	3635.819034	0.047974793	0.129669836	-0.369976506	0.711399996	0.849682329	0.047974793
ENSMUSG00000031949	Adat1	30947	protein_coding	41.73104028	0.047995952	0.186740954	-0.257018888	0.797164193	0.899266991	0.047995952
ENSMUSG00000094002	Gm9866	636791	lncRNA	5.525183168	0.047997993	0.954304755	-0.050296295	0.959886277	0.980506535	0.047997993
ENSMUSG00000005687	Bcas2	68183	protein_coding	377.3671205	0.048004974	0.105176401	-0.456423428	0.648085522	0.808650118	0.048004974
ENSMUSG00000030822	Prr14	233895	protein_coding	389.5537016	0.048007443	0.109529247	-0.438307067	0.661163704	0.816884691	0.048007443
ENSMUSG00000021948	Prkcd	18753	protein_coding	2582.568836	-0.048011112	0.083048691	0.578107991	0.563191212	0.750770075	0.048011112
ENSMUSG00000006676	Usp19	71472	protein_coding	1235.725008	-0.048062252	0.046519464	1.033164347	0.301526995	0.521787361	0.048062252
ENSMUSG00000038982	Bloc1s5	17828	protein_coding	152.9268728	-0.048081644	0.102168246	0.470612406	0.637917546	0.802698942	0.048081644
ENSMUSG00000088008	Gm25492	115490291	scaRNA	32.30073512	-0.048086371	0.248124916	0.19379904	0.846333239	0.924222269	0.048086371
ENSMUSG00000073608	Gal3st2c	619597	protein_coding	423.4594127	0.048103757	0.458740886	-0.10486041	0.916486574	0.959274295	0.048103757
ENSMUSG00000037885	Stk35	67333	protein_coding	393.0125496	0.048134155	0.073680278	-0.653284108	0.513573136	0.715485319	0.048134155
ENSMUSG00000024127	Prepl	213760	protein_coding	458.21874	0.0481348	0.061503554	-0.782634438	0.433841812	0.652382416	0.0481348
ENSMUSG00000031995	St14	19143	protein_coding	4321.850461	0.048136601	0.070857194	-0.679346704	0.496918211	0.702914252	0.048136601
ENSMUSG00000021039	Snw1	66354	protein_coding	520.4535032	-0.048153245	0.092700956	0.519447122	0.603448978	0.779177919	0.048153245
ENSMUSG00000039329	Tex19.1	73679	protein_coding	1.009463784	0.048201861	1.386177284	-0.03477323	0.972260567	1	0.048201861
ENSMUSG00000029269	Sult1b1	56362	protein_coding	1841.29105	-0.048215664	0.108809053	0.443121804	0.657677635	0.815005268	0.048215664
ENSMUSG00000029557	Mrm2	68017	protein_coding	84.93593561	-0.048217584	0.125008559	0.38571426	0.6997083	0.841035219	0.048217584
ENSMUSG00000028709	Mob3c	100465	protein_coding	207.1867882	0.048231634	0.099068864	-0.486849571	0.626364938	0.795381772	0.048231634
ENSMUSG00000046556	Zfp319	79233	protein_coding	216.8318076	0.048247413	0.081178147	-0.59433992	0.552284784	0.742887588	0.048247413
ENSMUSG00000029600	Rita1	100764	protein_coding	153.6895203	-0.048301087	0.112496668	0.429355712	0.667664379	0.821350533	0.048301087
ENSMUSG00000045598	Zfp553	233887	protein_coding	212.0012115	-0.048301856	0.088685933	0.544639433	0.586001528	0.767729412	0.048301856
ENSMUSG00000029655	N4bp2l2	381695	protein_coding	679.5250793	-0.048331813	0.067066287	0.720657349	0.47112036	0.684193826	0.048331813
ENSMUSG00000031701	Dnaja2	56445	protein_coding	1887.318486	-0.048343849	0.078377536	0.616807466	0.537361727	0.732906636	0.048343849
ENSMUSG00000015653	Steap2	74051	protein_coding	242.4230108	0.048347362	0.102223389	-0.472957925	0.636243198	0.801687789	0.048347362
ENSMUSG00000042444	Mindy2	235461	protein_coding	319.9414456	-0.048405449	0.11997355	0.403467668	0.686604215	0.83310074	0.048405449
ENSMUSG00000032369	Plscr1	22038	protein_coding	1593.878051	-0.048432352	0.064310214	0.753105128	0.451386743	0.667939129	0.048432352
ENSMUSG00000024983	Vti1a	53611	protein_coding	355.6216008	0.048452385	0.073365361	-0.660425903	0.508980554	0.712495114	0.048452385
ENSMUSG00000040865	Ino80d	227195	protein_coding	635.9744273	0.048466959	0.090076953	-0.53806171	0.590534446	0.77103778	0.048466959
ENSMUSG00000026162	Nhej1	75570	protein_coding	43.41829067	0.048484036	0.152807748	-0.317287811	0.751025225	0.875081807	0.048484036
ENSMUSG00000107623	Gm10415	100038574	lncRNA	0.916159086	0.048513294	2.198166724	-0.022069888	0.982392206	1	0.048513294
ENSMUSG00000026796	Niban2	227737	protein_coding	1469.178001	-0.048526086	0.071203843	0.681509364	0.495549244	0.701603242	0.048526086
ENSMUSG00000019302	Atp6v0a1	11975	protein_coding	665.8219351	0.048549246	0.077407679	-0.627189012	0.530535345	0.72786082	0.048549246

ensembl_gene_id	external_gene_name	entrezgene_id	gene_biotype	baseMean	log2FoldChange	lfcSE	stat	pvalue	padj	absLog2FC
ENSMUSG00000041440	Gk5	235533	protein_coding	254.6984928	-0.048567045	0.132868047	0.365528405	0.714717008	0.851944324	0.048567045
ENSMUSG00000009681	Bcr	110279	protein_coding	506.7205755	-0.048579325	0.090894454	0.534458629	0.593024265	0.772538316	0.048579325
ENSMUSG00000085664	Atxn7l1os2	75836	lncRNA	4.468521523	-0.048607013	0.523158167	0.092910741	0.925974473	0.964583514	0.048607013
ENSMUSG00000016940	Kctd2	70382	protein_coding	258.0037563	-0.048639268	0.085798717	0.566899707	0.570782323	0.756249943	0.048639268
ENSMUSG00000028712	Cyp4a31	666168	protein_coding	1.149834687	0.048650852	2.2086355	-0.022027561	0.982425971	1	0.048650852
ENSMUSG00000040188	Scamp2	24044	protein_coding	2291.616956	0.048653997	0.066402015	-0.732718675	0.463730032	0.677223005	0.048653997
ENSMUSG00000015217	Hmgb3	15354	protein_coding	203.4057761	-0.048667171	0.129912579	0.374614774	0.707946984	0.847201146	0.048667171
ENSMUSG00000028447	Dctn3	53598	protein_coding	540.7918818	-0.048694579	0.081784247	0.595402918	0.551574175	0.742355007	0.048694579
ENSMUSG00000027739	Rab33b	19338	protein_coding	184.3075534	0.048704502	0.109258437	-0.445773378	0.655760954	0.813372119	0.048704502
ENSMUSG00000024290	Rock1	19877	protein_coding	1396.35145	0.048708981	0.068869847	-0.70726135	0.479404079	0.690650055	0.048708981
ENSMUSG00000038253	Hoxa5	15402	protein_coding	84.58990411	0.048837625	0.11921904	-0.409646183	0.682065514	0.830017162	0.048837625
ENSMUSG00000051184	Zfp524	66056	protein_coding	61.92287201	-0.048851594	0.142976796	0.341674977	0.732595505	0.862854445	0.048851594
ENSMUSG00000015290	Ubl4a	27643	protein_coding	474.3453339	0.048876523	0.055385007	-0.88248653	0.377513761	0.599649878	0.048876523
ENSMUSG00000017132	Cyth1	19157	protein_coding	668.8086033	-0.048885926	0.080272293	0.609001242	0.542523616	0.736113301	0.048885926
ENSMUSG00000038496	Slc19a3	80721	protein_coding	2.434640418	-0.048924273	0.683765647	0.07155123	0.942959054	1	0.048924273
ENSMUSG00000032712	Resf1	67246	protein_coding	569.4000688	0.048958114	0.068056224	-0.71937747	0.471908374	0.684625749	0.048958114
ENSMUSG00000029020	Mfn2	170731	protein_coding	1369.714301	0.048967107	0.075091636	-0.652098021	0.514337941	0.715988936	0.048967107
ENSMUSG00000099045	Mir6374	102465194	miRNA	1.129438093	0.048971346	0.994435212	-0.049245386	0.960723742	1	0.048971346
ENSMUSG00000021009	Ptpn21	24000	protein_coding	133.5032855	0.048973918	0.112051114	-0.437067655	0.662062284	0.817198338	0.048973918
ENSMUSG00000074364	Ehd2	259300	protein_coding	7.111448387	0.048993582	0.790049546	-0.062013303	0.950552239	0.975869522	0.048993582
ENSMUSG00000048497	Mmgt2	216829	protein_coding	218.2901072	0.049019681	0.093078879	-0.526646668	0.598438978	0.776081458	0.049019681
ENSMUSG00000024350	Dnajc18	76594	protein_coding	90.07851999	0.049033284	0.164065271	-0.298864495	0.765043439	0.882319111	0.049033284
ENSMUSG00000113450	Zfp935	71508	protein_coding	31.02981101	0.04903469	0.198574903	-0.246932966	0.804960103	0.903862864	0.04903469
ENSMUSG00000035595	Fam174c	69770	protein_coding	87.33849133	-0.049157255	0.125612374	0.391340866	0.695545296	0.838533729	0.049157255
ENSMUSG00000018501	Ncor1	20185	protein_coding	2917.33458	-0.049197669	0.055492641	0.886562038	0.37531474	0.597565331	0.049197669
ENSMUSG00000031864	Ints10	70885	protein_coding	476.6047163	0.049234435	0.087308956	-0.563910476	0.57281506	0.758091673	0.049234435
ENSMUSG00000021488	Nsd1	18193	protein_coding	2877.124776	0.049273017	0.060103803	-0.819798656	0.412330897	0.632329199	0.049273017
ENSMUSG00000021302	Ggps1	14593	protein_coding	328.0285971	-0.049291147	0.071653136	0.687913322	0.491507367	0.698491194	0.049291147
ENSMUSG00000017707	Serinc3	26943	protein_coding	4379.961868	-0.049294461	0.144548816	0.341022934	0.733086316	0.86324782	0.049294461
ENSMUSG00000039671	Zmynd8	228880	protein_coding	1037.212499	-0.049357596	0.071101014	0.694189759	0.487563214	0.696047947	0.049357596
ENSMUSG00000034544	Rsrc1	66880	protein_coding	226.4029052	-0.049370804	0.083248465	0.593053632	0.55314526	0.743572273	0.049370804
ENSMUSG00000027714	Exosc9	50911	protein_coding	231.8620596	-0.04939278	0.094985533	0.520003183	0.603061357	0.778956218	0.04939278
ENSMUSG00000084786	Ubl5	66177	protein_coding	982.2858235	-0.049398381	0.057797433	0.854681222	0.392727649	0.615439779	0.049398381
ENSMUSG00000053214	Gm9899	100502829	lncRNA	2.710655565	0.049421649	1.282283997	-0.03854189	0.969255633	1	0.049421649
ENSMUSG00000021972	Hmbox1	219150	protein_coding	262.7617553	-0.049437431	0.11386734	0.434166914	0.664167237	0.818817302	0.049437431
ENSMUSG00000033819	Ppp1r16a	73062	protein_coding	739.9406805	0.049467639	0.084643864	-0.584420844	0.558937206	0.747694534	0.049467639
ENSMUSG00000050471	Fam118b	109229	protein_coding	334.9936252	-0.049495307	0.073423898	0.674103504	0.500245512	0.704868121	0.049495307
ENSMUSG00000034620	Rxylt1	216395	protein_coding	226.5558125	-0.049503841	0.100677895	0.49170517	0.622927783	0.793281643	0.049503841
ENSMUSG00000070814	Zswim9	321008	protein_coding	59.71020104	-0.049512787	0.161145928	0.307254349	0.758649781	0.878912418	0.049512787

ensembl_gene_id	external_gene_name	entrezgene_id	gene_biotype	baseMean	log2FoldChange	lfcSE	stat	pvalue	padj	absLog2FC
ENSMUSG00000024830	Rps6kb2	58988	protein_coding	189.9348894	0.049541332	0.115306751	-0.429648149	0.667451607	0.821333519	0.049541332
ENSMUSG00000050394	Armcx6	278097	protein_coding	16.62218778	0.049614294	0.263969584	-0.187954585	0.850912252	0.927277612	0.049614294
ENSMUSG00000037996	Slc24a2	76376	protein_coding	2.901800251	0.049679532	2.17800628	-0.022809637	0.981802121	1	0.049679532
ENSMUSG00000008999	Bmp7	12162	protein_coding	28.36711592	-0.049705675	0.309074267	0.160821136	0.872234278	0.937028043	0.049705675
ENSMUSG00000087819	Gm25117	115487522	snoRNA	235.3715276	-0.049743055	0.210146398	0.236706674	0.812884357	0.907147741	0.049743055
ENSMUSG00000010290	AI597479	98404	protein_coding	214.3396101	0.049754835	0.122247546	-0.407000681	0.684007488	0.831360834	0.049754835
ENSMUSG00000025077	Dclre1a	55947	protein_coding	210.0849668	0.049756818	0.081785332	-0.608383144	0.542933389	0.736235501	0.049756818
ENSMUSG00000059316	Slc27a4	26569	protein_coding	1489.337256	0.049761193	0.12624924	-0.39415044	0.693469974	0.837254214	0.049761193
ENSMUSG00000035305	Ror1	26563	protein_coding	111.9186615	-0.049783223	0.163842644	0.303847774	0.761243868	0.880273177	0.049783223
ENSMUSG00000006906	Stambp	70527	protein_coding	132.016982	0.049829363	0.116554059	-0.427521471	0.66899955	0.82238042	0.049829363
ENSMUSG00000020090	Npffr1	237362	protein_coding	4.396552229	-0.049839244	0.662718111	0.075204289	0.940052172	0.971404891	0.049839244
ENSMUSG00000031540	Kat6a	244349	protein_coding	968.3435318	0.049874132	0.09184231	-0.543040912	0.587101634	0.768458962	0.049874132
ENSMUSG00000026608	Kctd3	226823	protein_coding	664.6157538	0.049878576	0.060575661	-0.82340951	0.410275157	0.630552252	0.049878576
ENSMUSG00000020413	Hus1	15574	protein_coding	94.30768451	-0.049886318	0.144966352	0.344123427	0.730753465	0.861267215	0.049886318
ENSMUSG00000051506	Wdfy4	545030	protein_coding	10.2830043	0.049891097	0.79278456	-0.062931469	0.949821076	0.975869522	0.049891097
ENSMUSG00000051355	Commd1	17846	protein_coding	290.6588638	-0.049934288	0.097288245	0.513261268	0.607768574	0.782282948	0.049934288
ENSMUSG00000027349	Fam98b	68215	protein_coding	129.2771557	0.049942596	0.114688056	-0.435464662	0.663225184	0.81814452	0.049942596
ENSMUSG00000040661	Rad54l2	81000	protein_coding	446.1354644	-0.049953671	0.054580207	0.915234182	0.360068704	0.5825209	0.049953671
ENSMUSG00000047180	Neurl3	214854	protein_coding	1669.371538	-0.050039541	0.11373894	0.439950824	0.659972724	0.816323585	0.050039541
ENSMUSG00000029204	Rhoh	74734	protein_coding	11.16337664	0.050042837	0.686994465	-0.072843145	0.941930937	0.971883559	0.050042837
ENSMUSG00000024013	Fgd2	26382	protein_coding	22.50262092	-0.05004539	0.283199611	0.176714192	0.859732893	0.931236324	0.05004539
ENSMUSG00000056492	Adgrf5	224792	protein_coding	28.0662815	-0.050063025	0.285375063	0.175428872	0.860742656	0.931474719	0.050063025
ENSMUSG00000022841	Ap2m1	11773	protein_coding	1383.215582	-0.050083211	0.084787626	0.5906901	0.554728083	0.744703672	0.050083211
ENSMUSG00000027082	Tfpi	21788	protein_coding	53.03424009	0.05021247	0.224589447	-0.223574487	0.823088417	0.910297568	0.05021247
ENSMUSG00000041740	Rnf10	50849	protein_coding	1616.161243	0.050269601	0.085135906	-0.590462988	0.554880293	0.744703672	0.050269601
ENSMUSG00000019362	D8Ertd738e	101966	protein_coding	741.5329195	-0.050349732	0.04875775	1.032650852	0.301767321	0.522023104	0.050349732
ENSMUSG00000003527	Ess2	27886	protein_coding	173.0817792	0.05037754	0.088650465	-0.568271584	0.569850571	0.755621674	0.05037754
ENSMUSG00000062432	Cyp26c1	546726	protein_coding	0.899227057	-0.050403301	1.25065973	0.04030137	0.967852861	1	0.050403301
ENSMUSG00000030660	Pik3c2a	18704	protein_coding	1443.56607	0.050436341	0.060611807	-0.832120736	0.405340799	0.62645534	0.050436341
ENSMUSG00000025785	Exosc7	66446	protein_coding	268.9503236	0.050447249	0.118353738	-0.426241286	0.669932036	0.822722373	0.050447249
ENSMUSG00000027439	Gzf1	74533	protein_coding	348.1795133	-0.050469366	0.078905327	0.639619256	0.522420161	0.721840784	0.050469366
ENSMUSG00000055334	Snupn	66069	protein_coding	102.2881572	-0.050512862	0.114321921	0.44184756	0.65859952	0.815790015	0.050512862
ENSMUSG00000058301	Upf1	19704	protein_coding	1315.148323	0.050519352	0.072441536	-0.697381017	0.485564378	0.695380179	0.050519352
ENSMUSG00000063800	Prpf38a	230596	protein_coding	429.3935735	0.050528735	0.078683043	-0.642180746	0.520755833	0.720610174	0.050528735
ENSMUSG00000016946	Kctd5	69259	protein_coding	804.6743852	0.050565555	0.07122144	-0.709976591	0.477718653	0.68930064	0.050565555
ENSMUSG00000002064	Sdf2	20316	protein_coding	590.5449718	-0.050578465	0.083403298	0.606432432	0.544227638	0.736796542	0.050578465
ENSMUSG00000024816	Frmd8	67457	protein_coding	518.7423883	-0.0505801	0.085560386	0.591162601	0.554411479	0.744656458	0.0505801
ENSMUSG00000058239	Usf2	22282	protein_coding	243.0113582	-0.050624549	0.106165172	0.476847043	0.633471044	0.800270871	0.050624549
ENSMUSG00000030313	Dennd5b	320560	protein_coding	255.8938271	0.050652071	0.102537378	-0.493986406	0.62131578	0.792160606	0.050652071

ensembl_gene_id	external_gene_name	entrezgene_id	gene_biotype	baseMean	log2FoldChange	lfcSE	stat	pvalue	padj	absLog2FC
ENSMUSG00000042672	Dcst1	77772	protein_coding	24.96985472	-0.050674431	0.319448359	0.158631057	0.873959559	0.938083652	0.050674431
ENSMUSG00000071862	Lrrtm2	107065	protein_coding	43.79545557	0.05072639	0.300963306	-0.168546759	0.866153161	0.93371978	0.05072639
ENSMUSG00000023845	Lnpep	240028	protein_coding	2331.272301	-0.050753731	0.095486656	0.531526952	0.595053669	0.773528957	0.050753731
ENSMUSG00000018537	Pcgf2	22658	protein_coding	107.572164	0.050762978	0.226030454	-0.224584686	0.82230238	0.910171686	0.050762978
ENSMUSG00000021866	Anxa11	11744	protein_coding	1329.199054	0.050788497	0.057109274	-0.889321357	0.373830397	0.595844868	0.050788497
ENSMUSG00000018334	Ksr1	16706	protein_coding	356.9210958	0.050798661	0.183343821	-0.277067753	0.781728082	0.890621398	0.050798661
ENSMUSG00000000631	Myo18a	360013	protein_coding	3327.289707	0.050827413	0.145493298	-0.349345391	0.726830025	0.85879931	0.050827413
ENSMUSG00000027215	Cd82	12521	protein_coding	1373.168635	-0.050829774	0.070544599	0.72053389	0.471196341	0.684193826	0.050829774
ENSMUSG00000021786	Oxsm	71147	protein_coding	355.5899188	-0.050834415	0.105671892	0.481059003	0.630474565	0.79831751	0.050834415
ENSMUSG00000089911	Mfsd14a	15247	protein_coding	508.559947	0.050849037	0.08650516	-0.58781507	0.556656427	0.745972916	0.050849037
ENSMUSG00000036977	Anapc10	68999	protein_coding	92.0936508	0.050857918	0.121620163	-0.418170119	0.675822741	0.826400352	0.050857918
ENSMUSG00000011831	Evi5	14020	protein_coding	405.7268598	-0.050918729	0.076273948	0.667576945	0.504403679	0.708596728	0.050918729
ENSMUSG00000005886	Ncoa2	17978	protein_coding	837.3431736	0.050928396	0.071569439	-0.711594176	0.476716115	0.68833522	0.050928396
ENSMUSG00000017843	Ppp2r5c	26931	protein_coding	1406.423931	0.050960305	0.054350986	-0.937615096	0.348442251	0.570284968	0.050960305
ENSMUSG00000053110	Yap1	22601	protein_coding	686.0032134	-0.050967116	0.075047066	0.679135355	0.497052103	0.702914252	0.050967116
ENSMUSG00000006005	Tpr	108989	protein_coding	2013.334769	-0.051009976	0.092084009	0.553950423	0.579612775	0.762991261	0.051009976
ENSMUSG00000026080	Chst10	98388	protein_coding	7.787626272	-0.051023811	0.527235681	0.096776095	0.922904208	0.96266044	0.051023811
ENSMUSG00000018752	Tnfsfm13	619441	protein_coding	9.20502018	0.051049192	0.317454192	-0.160808058	0.872244578	0.937028043	0.051049192
ENSMUSG00000072770	Acrbp	54137	protein_coding	32.18799446	-0.051060815	0.307658733	0.16596576	0.868183896	0.934688628	0.051060815
ENSMUSG00000058173	Smco4	170748	protein_coding	158.824437	0.051110077	0.163553	-0.312498559	0.754661658	0.876856731	0.051110077
ENSMUSG00000024014	Pim1	18712	protein_coding	181.494518	-0.051136138	0.160034076	0.319532809	0.749322516	0.874044129	0.051136138
ENSMUSG00000043015	Nemp2	227094	protein_coding	49.53947012	-0.051151355	0.189970695	0.269259189	0.787730235	0.89369175	0.051151355
ENSMUSG00000029084	Cd38	12494	protein_coding	2088.509137	-0.051182396	0.139010393	0.368191149	0.71273071	0.850681912	0.051182396
ENSMUSG00000074794	Arrdc3	105171	protein_coding	893.8034142	-0.05118285	0.137572203	0.372043543	0.70986043	0.84860541	0.05118285
ENSMUSG00000029569	Tmem168	101118	protein_coding	958.3111973	-0.051224065	0.154884521	0.330724234	0.740852795	0.868861804	0.051224065
ENSMUSG00000041235	Chd7	320790	protein_coding	1233.397455	-0.051239152	0.113479386	0.451528281	0.651608847	0.810851996	0.051239152
ENSMUSG00000059588	Calcrl	54598	protein_coding	31.13093386	0.05130132	0.261626924	-0.19608578	0.844543026	0.923599766	0.05130132
ENSMUSG00000020349	Ppp2ca	19052	protein_coding	3190.806009	0.051309242	0.054699161	-0.938026122	0.348230986	0.570165229	0.051309242
ENSMUSG00000045538	Ddx28	71986	protein_coding	110.7986286	-0.05132228	0.109055238	0.47060812	0.637920608	0.802698942	0.05132228
ENSMUSG00000091491	Vmn2r97	627367	protein_coding	3.072694531	0.051360728	1.502732605	-0.034178221	0.972735033	1	0.051360728
ENSMUSG00000046605	B3gntl1	210004	protein_coding	90.86506362	0.051363241	0.120654052	-0.425706727	0.67032156	0.82296411	0.051363241
ENSMUSG00000021911	Parg	26430	protein_coding	651.8892626	0.051424391	0.071517548	-0.719045785	0.47211271	0.684706793	0.051424391
ENSMUSG00000027677	Ttc14	67120	protein_coding	396.3077821	0.051436245	0.091356547	-0.563027467	0.57341618	0.758644032	0.051436245
ENSMUSG00000059714	Flot1	14251	protein_coding	366.4544497	-0.05144612	0.101042782	0.509151854	0.610645788	0.784213744	0.05144612
ENSMUSG00000019132	BC005537	79555	protein_coding	1472.474649	-0.051463547	0.06572427	0.783021969	0.433614211	0.652196761	0.051463547
ENSMUSG00000096094	A630095N17Rik	381272	protein_coding	2.750910852	0.051543989	0.843127826	-0.061134253	0.951252291	1	0.051543989
ENSMUSG00000022696	Sidt1	320007	protein_coding	459.499791	-0.051640054	0.138015894	0.374160193	0.708285138	0.847451753	0.051640054
ENSMUSG00000014791	Elmo3	234683	protein_coding	881.8542105	-0.051734674	0.064530998	0.80170267	0.422724971	0.642569972	0.051734674
ENSMUSG00000049878	Rlf	109263	protein_coding	461.0060998	0.051741763	0.070202619	-0.737034656	0.461101275	0.674995696	0.051741763

ensembl_gene_id	external_gene_name	entrezgene_id	gene_biotype	baseMean	log2FoldChange	lfcSE	stat	pvalue	padj	absLog2FC
ENSMUSG00000033735	Spr	20751	protein_coding	704.6207135	-0.051781055	0.079930353	0.647827176	0.517096736	0.717711842	0.051781055
ENSMUSG00000003731	Kpna6	16650	protein_coding	822.9195696	-0.051805423	0.073580776	0.704061934	0.481394205	0.692354022	0.051805423
ENSMUSG00000005681	Apoa2	11807	protein_coding	6.008385744	-0.051807693	0.477423468	0.108515179	0.91358704	0.957921881	0.051807693
ENSMUSG00000027678	Ncoa3	17979	protein_coding	898.9334698	0.051851135	0.060013976	-0.863984323	0.387596502	0.610061822	0.051851135
ENSMUSG00000024588	Fech	14151	protein_coding	569.1961052	-0.051868586	0.079192628	0.654967354	0.512488773	0.714893745	0.051868586
ENSMUSG00000021096	Ppm1a	19042	protein_coding	1290.19638	-0.051908366	0.050408545	1.029753298	0.303125829	0.523653558	0.051908366
ENSMUSG00000027004	Frzb	20378	protein_coding	1.05896454	0.051950003	2.513108405	-0.020671613	0.983507614	1	0.051950003
ENSMUSG00000032388	Spg21	27965	protein_coding	383.8469005	-0.051953249	0.090000048	0.57725802	0.563765168	0.751050176	0.051953249
ENSMUSG00000037447	Arid5a	214855	protein_coding	90.20094355	-0.051968271	0.222790545	0.233260667	0.815559	0.908352419	0.051968271
ENSMUSG00000050813	Or2av9	257932	protein_coding	1.478565516	0.051989979	2.51790858	-0.020648081	0.983526386	1	0.051989979
ENSMUSG00000070867	Trabd2b	666048	protein_coding	18.74676971	-0.052010479	0.322049436	0.161498432	0.871700846	0.936825965	0.052010479
ENSMUSG00000028141	Oaz3	53814	protein_coding	1.717763374	-0.052026135	0.858866634	0.060575336	0.951697416	1	0.052026135
ENSMUSG00000027551	Zfp64	22722	protein_coding	269.1510409	-0.052080748	0.097668981	0.533237342	0.593869295	0.773008398	0.052080748
ENSMUSG00000052363	Zdhhc19	245308	protein_coding	3.924516379	-0.052095614	1.097116509	0.047484122	0.962127385	1	0.052095614
ENSMUSG00000058216	Gstp3	225884	protein_coding	42.16899432	-0.052112435	0.215018435	0.242362636	0.808499183	0.905558567	0.052112435
ENSMUSG00000019849	Prep	19072	protein_coding	581.0961888	0.052114771	0.087357368	-0.596569846	0.550794608	0.741668474	0.052114771
ENSMUSG00000068130	Zfp442	668923	protein_coding	39.59916297	-0.052176704	0.164538305	0.317109773	0.751160309	0.875081807	0.052176704
ENSMUSG00000004815	Dgkq	110524	protein_coding	466.345769	-0.052187879	0.169750151	0.307439369	0.758508966	0.878895387	0.052187879
ENSMUSG00000023027	Atf1	11908	protein_coding	244.49034	0.052196441	0.084395253	-0.618476033	0.536261592	0.732309926	0.052196441
ENSMUSG00000035547	Capn5	12337	protein_coding	1552.556781	0.052210473	0.094206873	-0.554210868	0.579434542	0.76287826	0.052210473
ENSMUSG00000024109	Nrxn1	18189	protein_coding	8.614002926	-0.05221601	0.691191659	0.075544908	0.939781169	0.97136798	0.05221601
ENSMUSG00000099687	Or51b6b	259126	protein_coding	0.881797363	-0.052227058	1.430423923	0.036511595	0.970874434	1	0.052227058
ENSMUSG00000034932	Mrpl54	66047	protein_coding	502.5721588	-0.052246809	0.091264602	0.572476166	0.566999428	0.753307251	0.052246809
ENSMUSG00000090035	Galnt4	14426	protein_coding	3428.352042	-0.0522641	0.087659017	0.59622047	0.551027952	0.741922186	0.0522641
ENSMUSG00000035764	Fbxo45	268882	protein_coding	264.0342665	0.052315278	0.126880526	-0.412319206	0.680105473	0.828386324	0.052315278
ENSMUSG00000044149	Nkrf	77286	protein_coding	79.88296582	0.052359824	0.168907223	-0.309991622	0.756567328	0.877877519	0.052359824
ENSMUSG00000032232	Cgnl1	68178	protein_coding	608.7705343	-0.052398453	0.116718879	0.448928684	0.653483103	0.812010532	0.052398453
ENSMUSG00000054021	Sirt5	68346	protein_coding	119.0970619	-0.052436012	0.133835938	0.391793209	0.695211013	0.838326567	0.052436012
ENSMUSG00000022223	Sdr39u1	654795	protein_coding	74.95106045	0.052449785	0.155155131	-0.338047378	0.735327484	0.865203089	0.052449785
ENSMUSG00000025813	Homer2	26557	protein_coding	382.7662762	-0.052475194	0.102131787	0.513798843	0.607392637	0.781981914	0.052475194
ENSMUSG00000078451	Ppil6	73075	protein_coding	9.486774504	-0.052541295	0.349144195	0.150485949	0.880381236	0.941010727	0.052541295
ENSMUSG00000054582	Pabpc1l	381404	protein_coding	5.978271588	0.05255087	0.765171572	-0.068678545	0.945245497	0.97354273	0.05255087
ENSMUSG00000028563	Tm2d1	94043	protein_coding	165.1264175	-0.052551165	0.098447045	0.53380135	0.59347898	0.772683055	0.052551165
ENSMUSG00000045659	Plekha7	233765	protein_coding	1094.297527	0.05258218	0.116060367	-0.45305888	0.650506344	0.810140405	0.05258218
ENSMUSG00000024969	Mark2	13728	protein_coding	1181.813416	0.052589553	0.078935438	-0.666235022	0.505260891	0.709332513	0.052589553
ENSMUSG00000074006	Omp	18378	protein_coding	96.66974073	-0.052596494	0.167571352	0.313875212	0.753615818	0.876528151	0.052596494
ENSMUSG00000033439	Trmt13	229780	protein_coding	51.79583712	0.052608674	0.148404474	-0.3544952	0.722967801	0.856549354	0.052608674
ENSMUSG00000023353	Agap3	213990	protein_coding	459.7700054	0.05264194	0.084698333	-0.621522738	0.534255738	0.730364909	0.05264194
ENSMUSG00000022286	Grhl2	252973	protein_coding	858.7456566	0.052750351	0.064511071	-0.817694543	0.413531627	0.633669972	0.052750351

ensembl_gene_id	external_gene_name	entrezgene_id	gene_biotype	baseMean	log2FoldChange	lfcSE	stat	pvalue	padj	absLog2FC
ENSMUSG00000036707	Cab39	12283	protein_coding	3787.699087	0.052775941	0.060166226	-0.877168878	0.380394918	0.60257474	0.052775941
ENSMUSG00000032350	Gclc	14629	protein_coding	1021.596245	0.052777617	0.100461112	-0.525353703	0.599337331	0.77652216	0.052777617
ENSMUSG00000038866	Zcchc2	227449	protein_coding	408.3779774	0.052850138	0.101634139	-0.520003797	0.603060929	0.778956218	0.052850138
ENSMUSG00000038007	Acer2	230379	protein_coding	88.43414376	0.052852237	0.114083069	-0.463278532	0.643164732	0.80530915	0.052852237
ENSMUSG00000017288	Vps53	68299	protein_coding	687.6329507	0.052866334	0.05888638	-0.897768443	0.369309012	0.591883639	0.052866334
ENSMUSG00000027456	Sdcbp2	228765	protein_coding	2405.05019	0.052874382	0.137161612	-0.385489656	0.699874669	0.841086565	0.052874382
ENSMUSG00000030905	Crym	12971	protein_coding	47.86465347	0.052881732	0.180660824	-0.292712778	0.769741702	0.884525396	0.052881732
ENSMUSG00000077167	Gm24119	115487534	snoRNA	27.7203354	-0.052895328	0.264614777	0.199895594	0.841562237	0.92158767	0.052895328
ENSMUSG00000067336	Bmpr2	12168	protein_coding	646.0065238	-0.05291299	0.090864848	0.582326291	0.56034692	0.748973617	0.05291299
ENSMUSG00000037111	Setd7	73251	protein_coding	446.4293261	-0.052971379	0.124651623	0.424955392	0.670869193	0.823391591	0.052971379
ENSMUSG00000032766	Gng11	66066	protein_coding	200.0483134	0.052986986	0.091568379	-0.578660306	0.562818404	0.75067522	0.052986986
ENSMUSG00000036867	Smad6	17130	protein_coding	342.3636243	-0.052997976	0.187278963	0.282989477	0.777184903	0.888773967	0.052997976
ENSMUSG00000026096	Osgepl1	72085	protein_coding	73.34148271	0.053003231	0.145647246	-0.363915092	0.715921414	0.852575063	0.053003231
ENSMUSG00000015016	Acsf3	257633	protein_coding	295.4306026	-0.053005182	0.091225864	0.581032395	0.56121862	0.749713986	0.053005182
ENSMUSG00000046707	Csnk2a2	13000	protein_coding	292.3057116	-0.053029854	0.073863384	0.717945083	0.472791149	0.685206817	0.053029854
ENSMUSG00000020863	Luc7l3	67684	protein_coding	690.7410512	0.053031482	0.075227323	-0.704949743	0.480841513	0.691751611	0.053031482
ENSMUSG00000028436	Dcaf12	68970	protein_coding	680.365107	-0.053059492	0.061048374	0.869138503	0.384771379	0.607042377	0.053059492
ENSMUSG00000038416	Cdc16	69957	protein_coding	466.2773484	0.0530955	0.090171994	-0.588824725	0.555978858	0.745548913	0.0530955
ENSMUSG00000001829	Clpb	20480	protein_coding	441.7761713	0.053099824	0.070768562	-0.750330688	0.453055564	0.669122175	0.053099824
ENSMUSG00000027951	Adar	56417	protein_coding	763.9929948	-0.053101282	0.088447672	0.600369466	0.548260032	0.739498445	0.053101282
ENSMUSG00000053695	Defb37	353320	protein_coding	51.64239128	0.053138109	0.207964142	-0.255515726	0.798324799	0.899929685	0.053138109
ENSMUSG00000031312	Itgb1bp2	26549	protein_coding	4.503546455	0.053156053	0.54404091	-0.097705986	0.922165762	0.9624373	0.053156053
ENSMUSG00000024480	Ap3s1	11777	protein_coding	363.8025901	-0.053191151	0.096477803	0.551330457	0.581407164	0.764378465	0.053191151
ENSMUSG00000014039	Prdm15	114604	protein_coding	272.6019784	-0.053196554	0.083841613	0.634488676	0.525761955	0.724199927	0.053196554
ENSMUSG00000025157	Zdhhc16	74168	protein_coding	168.080131	0.053215338	0.093613654	-0.568457013	0.569724687	0.755515416	0.053215338
ENSMUSG00000091476	Catspere2	545391	protein_coding	19.83493917	0.053246542	0.29689739	-0.179343248	0.857668191	0.930374388	0.053246542
ENSMUSG00000041684	Bivm	246229	protein_coding	194.5308304	0.053251406	0.106373009	-0.500610132	0.61664553	0.788668238	0.053251406
ENSMUSG00000038605	Samd10	229011	protein_coding	137.770031	-0.05326527	0.103978971	0.512269646	0.608462306	0.782626881	0.05326527
ENSMUSG00000029840	Mtpn	14489	protein_coding	1910.132463	0.053372672	0.076045192	-0.701854652	0.482769812	0.693558997	0.053372672
ENSMUSG00000035168	Tanc1	66860	protein_coding	485.025639	-0.053414515	0.064008153	0.834495487	0.404001829	0.625216588	0.053414515
ENSMUSG00000003099	Ppp5c	19060	protein_coding	1139.887943	0.053429876	0.072553824	-0.736417093	0.461476905	0.675186464	0.053429876
ENSMUSG00000028744	Slc66a1	212555	protein_coding	80.02297489	0.053477986	0.135673942	-0.394165489	0.693458863	0.837254214	0.053477986
ENSMUSG00000067058	Rps15a-ps5	100042335	processed_pseudogene	2.611215804	-0.053485821	0.635956718	0.084102926	0.932974599	1	0.053485821
ENSMUSG00000039361	Picalm	233489	protein_coding	2691.786791	0.053500216	0.043974999	-1.216605277	0.223754434	0.43083733	0.053500216
ENSMUSG00000031985	Gnpat	14712	protein_coding	443.9711774	0.053525193	0.076863381	-0.696367927	0.486198444	0.695624496	0.053525193
ENSMUSG00000025280	Polr3a	218832	protein_coding	293.4893006	-0.053562399	0.071029456	0.754087136	0.450796901	0.667604701	0.053562399
ENSMUSG00000028953	Abcf2	27407	protein_coding	502.9074116	0.053610205	0.093707174	-0.572103529	0.567251839	0.753386941	0.053610205
ENSMUSG00000031529	Tnks	21951	protein_coding	608.622695	0.053615906	0.080598873	-0.665219046	0.5059104	0.709931167	0.053615906
ENSMUSG00000030614	Tmem126b	68472	protein_coding	210.855299	0.053633004	0.096944134	-0.553236192	0.580101687	0.763391447	0.053633004

ensembl_gene_id	external_gene_name	entrezgene_id	gene_biotype	baseMean	log2FoldChange	lfcSE	stat	pvalue	padj	absLog2FC
ENSMUSG00000056148	Rdh9	103142	protein_coding	37.94146053	0.053644954	0.310919533	-0.172536454	0.863015805	0.932166328	0.053644954
ENSMUSG00000034462	Pkd2	18764	protein_coding	183.8363801	0.053648959	0.126455261	-0.424252488	0.671381685	0.823836911	0.053648959
ENSMUSG00000037379	Spon2	100689	protein_coding	18.39375529	-0.053651654	0.253871901	0.211333567	0.832626992	0.915629096	0.053651654
ENSMUSG00000025793	Hgs	15239	protein_coding	938.9182425	0.053654723	0.079327447	-0.676370229	0.498805614	0.703739933	0.053654723
ENSMUSG00000001017	Chtop	66511	protein_coding	1111.814973	0.053663297	0.077241616	-0.694745906	0.487214554	0.695767999	0.053663297
ENSMUSG00000054509	Parp4	328417	protein_coding	369.8639777	0.053776561	0.295414587	-0.182037593	0.855553223	0.929758221	0.053776561
ENSMUSG00000026319	Relch	227446	protein_coding	1355.328722	0.053793303	0.063501603	-0.847117242	0.396929775	0.618912124	0.053793303
ENSMUSG00000026150	Mff	75734	protein_coding	636.8211995	-0.053820558	0.072379183	0.743591671	0.457123544	0.671972423	0.053820558
ENSMUSG00000098132	Rassf10	78748	protein_coding	47.26370974	-0.053846649	0.254658087	0.211446845	0.832538606	0.915629096	0.053846649
ENSMUSG00000009647	Mcu	215999	protein_coding	1539.40124	0.053853093	0.062858143	-0.85674012	0.391588525	0.614062569	0.053853093
ENSMUSG00000004843	Chmp2b	68942	protein_coding	1651.75741	-0.053902054	0.078492756	0.686713735	0.49226314	0.698928166	0.053902054
ENSMUSG00000021969	Zdhhc20	75965	protein_coding	921.5298508	0.053927186	0.110354878	-0.488670618	0.625074909	0.794593769	0.053927186
ENSMUSG00000001323	Srr	27364	protein_coding	188.2132717	-0.053934066	0.090178041	0.598084253	0.549783717	0.740730056	0.053934066
ENSMUSG00000045039	Megf8	269878	protein_coding	549.2885661	-0.053967189	0.096822498	0.557382743	0.577265953	0.761298159	0.053967189
ENSMUSG00000038342	Mlxip	208104	protein_coding	847.8383414	0.053975657	0.089107652	-0.605735367	0.544690495	0.736813469	0.053975657
ENSMUSG00000022337	Emc2	66736	protein_coding	489.9532964	0.053978908	0.084779571	-0.63669711	0.524322156	0.72294031	0.053978908
ENSMUSG00000001729	Akt1	11651	protein_coding	2120.411924	0.053985814	0.065443258	-0.824925528	0.409413872	0.630049453	0.053985814
ENSMUSG00000118117	Gm30961	102632967	lncRNA	4.277422171	0.054036263	0.514605078	-0.105005305	0.916371599	1	0.054036263
ENSMUSG00000031112	Stk26	70415	protein_coding	128.8569229	0.054066297	0.342506055	-0.15785501	0.874571051	0.938406345	0.054066297
ENSMUSG00000072969	Armcx5	494468	protein_coding	45.65546027	-0.054079092	0.181662082	0.297690589	0.765939327	0.882687823	0.054079092
ENSMUSG00000026227	2810459M11Rik	72792	protein_coding	441.2579602	-0.054083295	0.106425298	0.508180822	0.611326539	0.784660848	0.054083295
ENSMUSG00000056536	Pign	27392	protein_coding	554.1545413	-0.054084415	0.073739355	0.733453866	0.463281656	0.676807614	0.054084415
ENSMUSG00000020184	Mdm2	17246	protein_coding	478.7893984	0.054105887	0.089125557	-0.60707488	0.54380122	0.736636318	0.054105887
ENSMUSG00000071203	Naip5	17951	protein_coding	632.0577579	-0.054182619	0.088260023	0.613897628	0.539282978	0.733766095	0.054182619
ENSMUSG00000056481	Cd248	70445	protein_coding	2.021936778	-0.054192345	1.923882093	0.028168225	0.97752798	1	0.054192345
ENSMUSG00000023307	Marchf5	69104	protein_coding	399.8556117	-0.054196826	0.067584579	0.801911123	0.422604371	0.642504934	0.054196826
ENSMUSG00000021957	Tkt	21881	protein_coding	6066.757133	0.054241723	0.081151584	-0.668400057	0.503878259	0.708294652	0.054241723
ENSMUSG00000031652	N4bp1	80750	protein_coding	514.173585	0.054316843	0.076756862	-0.707648039	0.479163853	0.690541222	0.054316843
ENSMUSG00000046516	Cox17	12856	protein_coding	108.2471873	0.054344525	0.123561995	-0.439815863	0.660070478	0.816326209	0.054344525
ENSMUSG00000034194	R3hcc1	71843	protein_coding	151.3490368	0.054400137	0.094509409	-0.575605517	0.564881851	0.751588072	0.054400137
ENSMUSG00000095125	Vmn1r198	171254	protein_coding	2.393546296	-0.054438051	1.932758811	0.028165983	0.977529768	1	0.054438051
ENSMUSG00000003200	Sh3gl1	20405	protein_coding	438.6154944	0.054452555	0.071419804	-0.762429351	0.445803794	0.662983366	0.054452555
ENSMUSG00000039747	Orai2	269717	protein_coding	309.6367658	-0.054505592	0.141253912	0.385869614	0.699593236	0.84101582	0.054505592
ENSMUSG00000028059	Arhgef2	16800	protein_coding	719.364388	-0.054574101	0.117867142	0.463013693	0.643354552	0.805485753	0.054574101
ENSMUSG00000035495	Tstd2	272027	protein_coding	325.6746644	0.054603527	0.078497844	-0.695605436	0.486675961	0.695624496	0.054603527
ENSMUSG00000057093	Zfp607b	112415	protein_coding	11.4509136	0.054608505	0.350036181	-0.156008172	0.876026579	0.938630585	0.054608505
ENSMUSG00000000486	Septin1	54204	protein_coding	17.17041654	0.054627264	0.434122024	-0.125833892	0.899863413	0.9507578	0.054627264
ENSMUSG00000051495	Irf2bp2	270110	protein_coding	2286.022553	0.054695016	0.111978579	-0.488441771	0.625236961	0.794610064	0.054695016
ENSMUSG00000041115	Iqsec2	245666	protein_coding	251.6417703	0.054701005	0.184922303	-0.295805343	0.767378742	0.883408334	0.054701005

Supplementary Table 1

ensembl_gene_id	external_gene_name	entrezgene_id	gene_biotype	baseMean	log2FoldChange	lfcSE	stat	pvalue	padj	absLog2FC
ENSMUSG00000038039	Gcc2	70297	protein_coding	1681.6507	-0.054766271	0.098116764	0.558174454	0.576725263	0.761222618	0.054766271
ENSMUSG00000026739	Bmi1	12151	protein_coding	174.840088	-0.054832705	0.103898253	0.527753867	0.59767018	0.775625491	0.054832705
ENSMUSG00000026494	Kif26b	269152	protein_coding	12.9990139	-0.054861531	0.422591757	0.129821584	0.896707586	0.94900212	0.054861531
ENSMUSG00000029465	Arpc3	56378	protein_coding	1918.109696	-0.054927054	0.076442324	0.718542442	0.472422889	0.684950973	0.054927054
ENSMUSG00000027566	Psma7	26444	protein_coding	1367.485168	0.05492925	0.07899049	-0.695390679	0.4868105	0.695624496	0.05492925
ENSMUSG00000055799	Tcf7l1	21415	protein_coding	136.4244752	-0.054929635	0.196444905	0.279618526	0.779770192	0.890125936	0.054929635
ENSMUSG00000037541	Shank2	210274	protein_coding	362.0286094	-0.054941614	0.140484032	0.391087963	0.695732218	0.83856979	0.054941614
ENSMUSG00000067219	Nipal1	70701	protein_coding	607.5150295	-0.054953255	0.103494188	0.530979139	0.595433235	0.773824226	0.054953255
ENSMUSG00000073664	Nbeal1	269198	protein_coding	2082.49584	-0.054985646	0.086113614	0.638524432	0.523132355	0.722040793	0.054985646
ENSMUSG00000004221	Ikbkg	16151	protein_coding	658.3508421	-0.055000016	0.073406106	0.749256692	0.453702505	0.669388475	0.055000016
ENSMUSG00000021340	Gpld1	14756	protein_coding	105.3380546	0.055010251	0.155883262	-0.352893894	0.724167989	0.857084875	0.055010251
ENSMUSG00000033106	Slc7a6os	66432	protein_coding	214.1096019	-0.055019765	0.079232657	0.694407674	0.487426582	0.696010599	0.055019765
ENSMUSG00000035203	Epn1	13854	protein_coding	2450.622276	0.055049933	0.056155948	-0.980304576	0.326935797	0.548792208	0.055049933
ENSMUSG00000013787	Ehmt2	110147	protein_coding	1920.462319	0.055060149	0.033385011	-1.649247612	0.099096917	0.245940261	0.055060149
ENSMUSG00000036819	Jmjd4	194952	protein_coding	162.8341734	0.055119834	0.098519752	-0.559480036	0.575834152	0.760926598	0.055119834
ENSMUSG00000047193	Dync2h1	110350	protein_coding	110.9581192	0.05514615	0.131805915	-0.418389039	0.675662699	0.826265833	0.05514615
ENSMUSG00000042625	Safb2	224902	protein_coding	721.0116071	0.055171175	0.144248927	-0.382471996	0.702111297	0.842880631	0.055171175
ENSMUSG00000079215	Zfp664	269704	protein_coding	697.9429906	-0.055173087	0.07981554	0.691257456	0.489403763	0.697206154	0.055173087
ENSMUSG00000026806	Ddx31	227674	protein_coding	103.913994	0.055175322	0.154074446	-0.358108194	0.720262348	0.855204229	0.055175322
ENSMUSG00000027300	Ubox5	140629	protein_coding	133.4013872	-0.055197944	0.121304872	0.455034844	0.649084172	0.809468057	0.055197944
ENSMUSG00000015672	Mrpl32	75398	protein_coding	267.4910084	-0.055230924	0.086178221	0.640891907	0.521592914	0.721043477	0.055230924
ENSMUSG00000026878	Rab14	68365	protein_coding	1429.570078	-0.055241753	0.049986872	1.105125218	0.269105335	0.484149199	0.055241753
ENSMUSG00000030361	Klrb1a	17057	protein_coding	4.919144578	0.055250317	0.623852118	-0.088563164	0.929429084	0.966128972	0.055250317
ENSMUSG00000025730	Rab40c	224624	protein_coding	311.2157269	-0.055286397	0.070893789	0.77984824	0.435480208	0.653742472	0.055286397
ENSMUSG00000054728	Phactr1	218194	protein_coding	4.485917552	-0.055291844	2.075864657	0.026635573	0.9787504	0.988831409	0.055291844
ENSMUSG00000021285	Ppp1r13b	21981	protein_coding	574.3530382	0.055310026	0.072884853	-0.7588686	0.447931164	0.664930223	0.055310026
ENSMUSG00000028927	Padi2	18600	protein_coding	163.04075	0.055327217	0.188781393	-0.293075584	0.769464379	0.884482916	0.055327217
ENSMUSG00000037824	Tspan14	52588	protein_coding	496.5329324	0.055367825	0.138640111	-0.39936368	0.689625252	0.834370416	0.055367825
ENSMUSG00000052310	Slc39a1	30791	protein_coding	1451.271777	-0.055391839	0.07151659	0.774531322	0.438616653	0.656764359	0.055391839
ENSMUSG00000020898	Ctc1	68964	protein_coding	243.5905799	0.055431904	0.118067534	-0.469493201	0.638717143	0.80301103	0.055431904
ENSMUSG00000032485	Scap	235623	protein_coding	776.4028261	-0.055459263	0.054208612	1.023071075	0.306274242	0.526745764	0.055459263
ENSMUSG00000034729	Mrps10	64657	protein_coding	206.8942947	0.055466275	0.082189607	-0.674857527	0.499766288	0.704451599	0.055466275
ENSMUSG00000118537	Shld3	113002583	protein_coding	23.23702822	0.055483869	0.285118165	-0.194599558	0.845706449	0.924043688	0.055483869
ENSMUSG00000037262	Kin	16588	protein_coding	154.816442	0.055493116	0.099152028	-0.559677066	0.575699728	0.760811885	0.055493116
ENSMUSG00000028910	Mecr	26922	protein_coding	743.187993	-0.055533145	0.134140259	0.413993125	0.678879142	0.827931507	0.055533145
ENSMUSG00000037410	Tbc1d2b	67016	protein_coding	387.314174	-0.055550977	0.133115583	0.41731385	0.676448859	0.826798642	0.055550977
ENSMUSG00000031077	Fadd	14082	protein_coding	178.2217693	-0.055569472	0.106763183	0.520492831	0.602720123	0.778759104	0.055569472
ENSMUSG00000046573	Lyrm4	380840	protein_coding	84.14603203	-0.055597655	0.120915525	0.459805765	0.645655645	0.806964824	0.055597655
ENSMUSG00000047084	Ngrn	83485	protein_coding	236.7423489	-0.055619525	0.101926737	0.5456814	0.58528496	0.767282764	0.055619525

Supplementary Table 1

ensembl_gene_id	external_gene_name	entrezgene_id	gene_biotype	baseMean	log2FoldChange	lfcSE	stat	pvalue	padj	absLog2FC
ENSMUSG00000040929	Rfx3	19726	protein_coding	105.7599651	-0.055619553	0.126201552	0.440720039	0.659415688	0.816133095	0.055619553
ENSMUSG00000064145	Arih2	23807	protein_coding	723.8823985	0.055719893	0.065739122	-0.847591083	0.396665742	0.618855346	0.055719893
ENSMUSG00000056692	Ilrun	224647	protein_coding	1622.240605	-0.055739553	0.043315378	1.286830577	0.198153347	0.398418975	0.055739553
ENSMUSG00000085421	4732490B19Rik	319871	lncRNA	20.52674784	0.055771267	0.260724839	-0.213908531	0.830618387	0.91440345	0.055771267
ENSMUSG00000032384	Csnk1g1	214897	protein_coding	383.6384088	0.055802864	0.087379847	-0.638623957	0.523067592	0.722040793	0.055802864
ENSMUSG00000047694	Yipf6	77929	protein_coding	1482.42088	0.055930669	0.133521102	-0.418890108	0.675296447	0.825971257	0.055930669
ENSMUSG00000038242	Aox4	71872	protein_coding	3.895445327	-0.055947985	1.953273305	0.028643193	0.977149163	1	0.055947985
ENSMUSG00000006715	Gmnn	57441	protein_coding	182.8118387	0.05596374	0.111349589	-0.502594943	0.615249086	0.78770474	0.05596374
ENSMUSG00000030447	Cyfip1	20430	protein_coding	1600.028643	-0.055966913	0.070024951	0.799242447	0.424149843	0.643957486	0.055966913
ENSMUSG00000025491	Ifitm1	68713	protein_coding	1.101263219	-0.055995054	0.95524994	0.058618223	0.953256195	1	0.055995054
ENSMUSG00000074182	Znhit6	229937	protein_coding	64.53388172	-0.056159059	0.170500143	0.329378371	0.741869714	0.869560398	0.056159059
ENSMUSG00000019320	Noxo1	71893	protein_coding	34.67061316	-0.056166049	0.187461093	0.299614432	0.764471275	0.882132403	0.056166049
ENSMUSG00000037275	Gemin5	216766	protein_coding	304.6508661	-0.056181658	0.125744757	0.446791255	0.655025786	0.812765484	0.056181658
ENSMUSG00000034255	Arhgap27	544817	protein_coding	1268.875679	-0.056229717	0.169587428	0.331567723	0.740215695	0.868422989	0.056229717
ENSMUSG00000006728	Cdk4	12567	protein_coding	818.5757048	0.056241416	0.117914625	-0.476967264	0.633385433	0.800270871	0.056241416
ENSMUSG00000002820	Atg4d	235040	protein_coding	1062.996459	-0.056272004	0.062771294	0.896460779	0.370006719	0.59260115	0.056272004
ENSMUSG00000069729	Arid1b	239985	protein_coding	922.9902519	0.056278873	0.056000391	-1.004972853	0.314909922	0.535990818	0.056278873
ENSMUSG00000034247	Plekhm1	353047	protein_coding	561.9895551	-0.056285209	0.16903237	0.332984794	0.739145761	0.867716116	0.056285209
ENSMUSG00000037375	Hhat	226861	protein_coding	41.25574929	0.056340337	0.184402354	-0.305529379	0.759962998	0.879445484	0.056340337
ENSMUSG00000040451	Sgms1	208449	protein_coding	523.6650687	0.056345394	0.072025247	-0.782300596	0.434037937	0.652558493	0.056345394
ENSMUSG00000035401	Emsy	233545	protein_coding	290.0621738	0.056358827	0.093880007	-0.600328316	0.548287451	0.739498445	0.056358827
ENSMUSG00000015002	Efr3a	76740	protein_coding	1075.438988	0.056387225	0.084881622	-0.664304285	0.506495579	0.71028091	0.056387225
ENSMUSG00000019794	Katna1	23924	protein_coding	413.8993854	-0.056398034	0.070273209	0.802553849	0.422232653	0.64211714	0.056398034
ENSMUSG00000032436	Cmtm7	102545	protein_coding	75.05868472	-0.05640198	0.264395544	0.213324245	0.831074064	0.914530359	0.05640198
ENSMUSG00000025034	Trim8	93679	protein_coding	444.3672675	-0.056436427	0.096069463	0.587454389	0.556898575	0.746166522	0.056436427
ENSMUSG00000034681	Rnps1	19826	protein_coding	445.1479628	0.056437525	0.08195646	-0.688628139	0.491057309	0.69829677	0.056437525
ENSMUSG00000021636	Marveld2	218518	protein_coding	515.799178	-0.056444911	0.064951891	0.869026446	0.384832666	0.607042377	0.056444911
ENSMUSG00000032393	Dpp8	74388	protein_coding	1666.9145	-0.056454357	0.079239759	0.712449883	0.476186235	0.687965835	0.056454357
ENSMUSG00000001157	Gmcl1	23885	protein_coding	389.5082801	-0.05646964	0.07290948	0.774517113	0.438625052	0.656764359	0.05646964
ENSMUSG00000034164	Emid1	140703	protein_coding	6.630223952	0.056476664	0.71688483	-0.078780665	0.937207083	0.970043107	0.056476664
ENSMUSG00000044502	Bod1	69556	protein_coding	377.0111451	-0.056506291	0.071663382	0.788496008	0.430406633	0.648871201	0.056506291
ENSMUSG00000030016	Zfp638	18139	protein_coding	984.4249458	0.056507038	0.091128555	-0.620080481	0.535204802	0.731167175	0.056507038
ENSMUSG00000004085	Map3k20	65964	protein_coding	311.0441484	0.056526002	0.140349254	-0.402752422	0.687130365	0.833389267	0.056526002
ENSMUSG00000027748	Trpc4	22066	protein_coding	2.350551478	0.056526052	1.062993028	-0.053176315	0.957591427	1	0.056526052
ENSMUSG00000035585	Tsen34	66078	protein_coding	394.0749782	0.056544466	0.086363131	-0.654729225	0.512642106	0.714893745	0.056544466
ENSMUSG00000048039	Isg20l2	229504	protein_coding	337.5566942	0.056545018	0.091858035	-0.615569647	0.538178589	0.733377497	0.056545018
ENSMUSG00000003198	Zfp959	224893	protein_coding	59.44447192	0.056589668	0.131392987	-0.430690175	0.666693664	0.820645435	0.056589668
ENSMUSG00000032264	Zw10	26951	protein_coding	345.8537793	0.056599846	0.079867209	-0.708674402	0.478526554	0.690104577	0.056599846
ENSMUSG00000018800	Abca5	217265	protein_coding	21.0641141	-0.056603231	0.236407281	0.239430998	0.810771396	0.906297546	0.056603231

ensembl_gene_id	external_gene_name	entrezgene_id	gene_biotype	baseMean	log2FoldChange	lfcSE	stat	pvalue	padj	absLog2FC
ENSMUSG00000058153	Sez6l	56747	protein_coding	4.942110659	-0.05664013	0.840769266	0.067367032	0.946289514	0.973795719	0.05664013
ENSMUSG00000063694	Cycs	13063	protein_coding	1816.087837	0.056655075	0.109471891	-0.517530796	0.604785677	0.779964063	0.056655075
ENSMUSG00000117048	Gm41584	105246280	lncRNA	3.65356735	-0.056681818	0.622598126	0.091040778	0.92746019	1	0.056681818
ENSMUSG00000026117	Zap70	22637	protein_coding	13.71382398	-0.056738393	0.43629812	0.13004501	0.896530816	0.948997494	0.056738393
ENSMUSG00000056220	Pla2g4a	18783	protein_coding	695.5582492	0.056749219	0.167177951	-0.339453968	0.734267769	0.864269275	0.056749219
ENSMUSG00000028423	Nfx1	74164	protein_coding	888.3720165	0.056758308	0.090864375	-0.624648638	0.53220169	0.728866518	0.056758308
ENSMUSG00000034525	Ice1	218333	protein_coding	582.5288514	0.056823839	0.08862444	-0.641175721	0.521408522	0.720922137	0.056823839
ENSMUSG00000036185	Sapcd1	78376	protein_coding	0.622570152	-0.056823894	1.417370169	0.040091075	0.968020517	1	0.056823894
ENSMUSG00000017307	Acot8	170789	protein_coding	442.9423308	0.056837418	0.077346142	-0.734844907	0.462433954	0.675987813	0.056837418
ENSMUSG00000023977	Ubr2	224826	protein_coding	1005.783937	0.056901954	0.055278343	-1.029371557	0.30330511	0.523766661	0.056901954
ENSMUSG00000089669	Tnfsf13	69583	protein_coding	121.4931398	0.056916211	0.116461566	-0.488712398	0.625045325	0.794593769	0.056916211
ENSMUSG00000026558	Uck2	80914	protein_coding	789.9579402	-0.056949184	0.145640558	0.391025584	0.695778325	0.83856979	0.056949184
ENSMUSG00000029624	Ptcd1	71799	protein_coding	275.2641663	-0.056992393	0.078889336	0.722434683	0.47002727	0.683555242	0.056992393
ENSMUSG00000000711	Rab5b	19344	protein_coding	830.2480502	0.056994421	0.095969477	-0.593880705	0.552591905	0.743179634	0.056994421
ENSMUSG00000044864	Ankrd50	99696	protein_coding	633.9345785	-0.057001414	0.113109161	0.503950459	0.614296191	0.78673795	0.057001414
ENSMUSG00000060591	Ifitm2	80876	protein_coding	305.0563423	0.057016738	0.160784501	-0.354615885	0.722877374	0.856549354	0.057016738
ENSMUSG00000004947	Dtx2	74198	protein_coding	102.7436778	0.057029675	0.114412735	-0.498455654	0.618162918	0.789569374	0.057029675
ENSMUSG00000081769	Gm12216	622459	lncRNA	16.65513802	-0.057039889	0.394000542	0.144771093	0.884891607	0.943016089	0.057039889
ENSMUSG00000057346	Apol9a	223672	protein_coding	76.64459762	0.057048048	0.483625038	-0.117959252	0.906099945	0.953990085	0.057048048
ENSMUSG00000021112	Pals1	56217	protein_coding	885.8978065	-0.057054123	0.071140528	0.801991849	0.422557673	0.642504934	0.057054123
ENSMUSG00000029486	Mrpl1	94061	protein_coding	257.6834778	-0.057055291	0.098877781	0.577028432	0.563920249	0.751086942	0.057055291
ENSMUSG00000047228	A2ml1	232400	protein_coding	10.45402249	-0.057078456	0.591171272	0.096551472	0.923082596	0.962730128	0.057078456
ENSMUSG00000037103	Dcaf15	212123	protein_coding	506.2195272	-0.057081304	0.076109035	0.749993804	0.453258436	0.669122175	0.057081304
ENSMUSG00000062031	Pgghg	212974	protein_coding	374.4409313	-0.05711895	0.087792769	0.650611103	0.515297561	0.716335759	0.05711895
ENSMUSG00000021337	Scgn	214189	protein_coding	12.57146849	0.057136579	0.306989513	-0.186118991	0.852351449	0.927761944	0.057136579
ENSMUSG00000062691	Cebpzos	68554	protein_coding	118.6268358	0.05715031	0.118313445	-0.483041553	0.629066232	0.797446101	0.05715031
ENSMUSG00000031370	Zrsr2	22184	protein_coding	242.5704049	0.057151045	0.072685253	-0.786281159	0.431702788	0.650053783	0.057151045
ENSMUSG00000027193	Api5	11800	protein_coding	1363.922969	-0.057159168	0.0998409	0.572502537	0.566981568	0.753307251	0.057159168
ENSMUSG00000039086	Ss18l1	269397	protein_coding	95.35596766	-0.057199303	0.109127207	0.524152544	0.600172445	0.77680602	0.057199303
ENSMUSG00000059995	Atxn7l3	217218	protein_coding	729.2087119	0.057208424	0.086949212	-0.657952177	0.510568862	0.71375046	0.057208424
ENSMUSG00000020284	Cfap410	67884	protein_coding	96.03045317	-0.057211447	0.127487654	0.448760686	0.653604302	0.812100026	0.057211447
ENSMUSG00000021546	Hnrnpk	15387	protein_coding	3988.668425	0.057246163	0.081783778	-0.69996965	0.483946258	0.694394109	0.057246163
ENSMUSG00000028923	Necap2	66147	protein_coding	791.6068424	-0.057250236	0.063470635	0.901995644	0.367059181	0.589852079	0.057250236
ENSMUSG00000042289	Hsd3b7	101502	protein_coding	0.54520931	0.057299085	1.865106005	-0.030721624	0.975491546	1	0.057299085
ENSMUSG00000035403	Crb2	241324	protein_coding	4.000812904	0.057313272	0.797447251	-0.071870926	0.942704628	1	0.057313272
ENSMUSG00000024276	Zfp397	69256	protein_coding	365.9737917	-0.057326455	0.119648421	0.479124211	0.631850267	0.799444652	0.057326455
ENSMUSG00000043257	Pigv	230801	protein_coding	267.3263692	0.057334736	0.078154308	-0.733609417	0.46318682	0.676807614	0.057334736
ENSMUSG00000060301	2610008E11Rik	72128	protein_coding	150.3547196	-0.057385417	0.124933426	0.459327972	0.645998664	0.807082912	0.057385417
ENSMUSG00000042605	Atxn2	20239	protein_coding	434.253721	0.057469107	0.088541786	-0.649061982	0.516298314	0.717206457	0.057469107

Supplementary Table 1

ensembl_gene_id	external_gene_name	entrezgene_id	gene_biotype	baseMean	log2FoldChange	lfcSE	stat	pvalue	padj	absLog2FC
ENSMUSG00000033970	Rfc3	69263	protein_coding	162.3140363	-0.057491289	0.138220918	0.415937685	0.677455602	0.827172038	0.057491289
ENSMUSG00000037349	Nudt22	68323	protein_coding	288.3946355	-0.057534978	0.176272358	0.326398189	0.744123107	0.87096527	0.057534978
ENSMUSG00000044881	Coa4	68185	protein_coding	204.4549474	0.057540488	0.097430336	-0.590580819	0.55480132	0.744703672	0.057540488
ENSMUSG00000024974	Smc3	13006	protein_coding	867.6231607	0.057544422	0.082994916	-0.693348759	0.488090711	0.696186289	0.057544422
ENSMUSG00000070427	Il18bp	16068	protein_coding	58.63539734	0.057610149	0.222610237	-0.258793801	0.795794341	0.898576349	0.057610149
ENSMUSG00000018167	Stard3	59045	protein_coding	641.9301441	0.057624068	0.073075012	-0.7885605	0.430368925	0.648871201	0.057624068
ENSMUSG00000024639	Gnaq	14682	protein_coding	591.1139821	0.057634367	0.066012062	-0.873088428	0.382614881	0.604944412	0.057634367
ENSMUSG00000090100	Ttbk2	140810	protein_coding	83.64902468	-0.057683385	0.161866736	0.356363425	0.721568417	0.855725733	0.057683385
ENSMUSG00000058440	Nrf1	18181	protein_coding	393.713823	0.057683385	0.062880021	-0.917356336	0.358955945	0.581669952	0.057683385
ENSMUSG00000028478	Clta	12757	protein_coding	1651.883584	-0.057686135	0.060066239	0.960375339	0.336866345	0.559233675	0.057686135
ENSMUSG00000039515	Ptpa	110854	protein_coding	1054.85505	0.057704985	0.077400204	-0.745540471	0.455945055	0.671255829	0.057704985
ENSMUSG00000030595	Nfkbib	18036	protein_coding	223.3587594	0.057722597	0.088471235	-0.652444796	0.514114274	0.715798257	0.057722597
ENSMUSG00000060681	Slc9a6	236794	protein_coding	268.9704449	0.057726479	0.075554077	-0.764041888	0.444842281	0.662290428	0.057726479
ENSMUSG00000015095	Fbxw5	30839	protein_coding	510.2491016	0.05780426	0.068220821	-0.847311109	0.396821736	0.618855346	0.05780426
ENSMUSG00000040945	Rcc2	108911	protein_coding	934.2887112	0.057819851	0.16604767	-0.348212358	0.727680708	0.859322531	0.057819851
ENSMUSG00000066232	Ipo7	233726	protein_coding	1988.879048	0.057835365	0.114450621	-0.505330285	0.613326875	0.786311623	0.057835365
ENSMUSG00000021027	Ralgapa1	56784	protein_coding	896.777353	0.057858639	0.095285808	-0.607211512	0.543710553	0.736573919	0.057858639
ENSMUSG00000051147	Nat2	17961	protein_coding	212.4514293	-0.057916479	0.103149428	0.561481342	0.574469446	0.7598549	0.057916479
ENSMUSG00000031502	Col4a1	12826	protein_coding	17.60117868	-0.057926217	0.518633577	0.111690063	0.911069158	0.956997404	0.057926217
ENSMUSG00000024914	Drap1	66556	protein_coding	772.0048318	0.057944721	0.077420489	-0.748441672	0.454193795	0.669812138	0.057944721
ENSMUSG00000004994	Ccdc130	67736	protein_coding	91.12203088	0.057945349	0.123640611	-0.468659519	0.639313027	0.803288086	0.057945349
ENSMUSG00000030491	Tdrd12	71981	protein_coding	13.77284155	0.057948541	0.333612793	-0.173699997	0.86210124	0.931908663	0.057948541
ENSMUSG00000075307	Klhl41	228003	protein_coding	2.864591295	-0.057988634	0.726434436	0.079826383	0.93637534	1	0.057988634
ENSMUSG00000020098	Pcbd1	13180	protein_coding	307.514167	0.057996055	0.128847511	-0.4501139	0.652628315	0.811601896	0.057996055
ENSMUSG00000037339	Fam53a	74504	protein_coding	302.5111735	-0.058022654	0.094166701	0.616169552	0.537782622	0.733113936	0.058022654
ENSMUSG00000021643	Serf1	20365	protein_coding	66.04851685	-0.058030523	0.16163426	0.359023655	0.719577393	0.854899358	0.058030523
ENSMUSG00000024105	Themis3	74556	protein_coding	652.1502432	-0.058034106	0.120575138	0.481310713	0.630295684	0.79831751	0.058034106
ENSMUSG00000018740	Slc25a35	71998	protein_coding	193.4874031	0.058050199	0.115038177	-0.504616823	0.613827991	0.78655252	0.058050199
ENSMUSG00000026048	Ercc5	22592	protein_coding	206.8289576	-0.058061519	0.090250625	0.643336472	0.520005795	0.720115363	0.058061519
ENSMUSG00000021598	Med10	28077	protein_coding	246.2721117	0.058080722	0.073934077	-0.785574445	0.432116841	0.65055862	0.058080722
ENSMUSG00000035299	Mid1	17318	protein_coding	81.20401498	0.058095514	0.245201943	-0.236929255	0.812711674	0.907147741	0.058095514
ENSMUSG00000049288	Lix1l	280411	protein_coding	6.56769069	0.058098962	0.556007281	-0.104493167	0.91677799	0.959274295	0.058098962
ENSMUSG00000061461	Smim20	66278	protein_coding	396.4474027	0.05812298	0.083792822	-0.693651066	0.487901061	0.696186289	0.05812298
ENSMUSG00000035382	Pcsk7	18554	protein_coding	1871.252149	0.05814392	0.049064404	-1.185053027	0.235996482	0.446089228	0.05814392
ENSMUSG00000063047	Zfp780b	338354	protein_coding	128.9575993	-0.058162163	0.105348586	0.552092484	0.580884991	0.764064128	0.058162163
ENSMUSG00000039357	Fut11	73068	protein_coding	117.199384	-0.058178874	0.10071719	0.577645923	0.563503195	0.750868206	0.058178874
ENSMUSG00000034121	Mks1	380718	protein_coding	97.37700836	-0.058187892	0.114043565	0.510225127	0.60989375	0.783613584	0.058187892
ENSMUSG00000055447	Cd47	16423	protein_coding	377.8569283	-0.058189662	0.101707011	0.5721303	0.567233703	0.753386941	0.058189662
ENSMUSG00000035049	Rrp12	107094	protein_coding	195.2773882	-0.058205101	0.131402494	0.442952789	0.657799883	0.815044063	0.058205101

ensembl_gene_id	external_gene_name	entrezgene_id	gene_biotype	baseMean	log2FoldChange	lfcSE	stat	pvalue	padj	absLog2FC
ENSMUSG00000015342	Xk	22439	protein_coding	240.7235836	-0.058304224	0.097167296	0.600039586	0.548479854	0.739637131	0.058304224
ENSMUSG00000050213	Snip1	76793	protein_coding	266.4318448	-0.058379754	0.088357198	0.66072437	0.508789092	0.712306522	0.058379754
ENSMUSG00000079243	Xirp1	22437	protein_coding	10.75330478	-0.058407224	0.499148511	0.117013719	0.906849182	0.954353007	0.058407224
ENSMUSG00000085958	A930019D19Rik	77806	lncRNA	8.324259878	-0.058425128	0.386606511	0.15112298	0.879878706	0.940676072	0.058425128
ENSMUSG00000033688	Inhca	71775	protein_coding	2.678958123	0.058434591	1.207217104	-0.048404376	0.961393972	1	0.058434591
ENSMUSG00000066037	Hnrnpr	74326	protein_coding	594.531797	0.05846417	0.075681934	-0.77249836	0.439819318	0.657836809	0.05846417
ENSMUSG00000003299	Mrpl4	66163	protein_coding	440.3913454	0.058519788	0.079784462	-0.733473498	0.463269686	0.676807614	0.058519788
ENSMUSG00000026836	Acvr1	11477	protein_coding	127.9398337	0.058528695	0.125850258	-0.465066153	0.641884084	0.804573016	0.058528695
ENSMUSG00000031390	Avpr2	12000	protein_coding	1.528653045	-0.058586729	1.385351167	0.042290165	0.966267386	1	0.058586729
ENSMUSG00000040511	Pvr	52118	protein_coding	159.5834414	-0.058599884	0.139904794	0.418855443	0.675321783	0.825971257	0.058599884
ENSMUSG00000031154	Otud5	54644	protein_coding	878.2017388	0.05861439	0.06822845	-0.859090156	0.390290778	0.612497263	0.05861439
ENSMUSG00000031820	Babam1	68251	protein_coding	614.9760069	-0.058616945	0.068339135	0.857736127	0.391038186	0.613316042	0.058616945
ENSMUSG00000001095	Slc13a2	20500	protein_coding	858.574303	0.058629955	0.202762705	-0.28915552	0.772462369	0.885864136	0.058629955
ENSMUSG00000063522	Ly6m	67038	protein_coding	1009.146903	0.058696855	0.483281016	-0.121454915	0.903330723	0.952286247	0.058696855
ENSMUSG00000020781	Tsen54	76265	protein_coding	242.553	-0.058700559	0.08675417	0.676630982	0.498640117	0.703739933	0.058700559
ENSMUSG00000037243	Zfp692	103836	protein_coding	195.8978844	-0.058715701	0.10699157	0.548788102	0.583150877	0.765756487	0.058715701
ENSMUSG00000021103	Mnat1	17420	protein_coding	136.7850454	-0.058726681	0.121278464	0.484230089	0.628222585	0.796810121	0.058726681
ENSMUSG00000041815	Poldip3	73826	protein_coding	1117.286957	-0.058771807	0.053668732	1.095084693	0.273479534	0.489835334	0.058771807
ENSMUSG00000016495	Plgrkt	67759	protein_coding	815.9143735	0.058776888	0.090356171	-0.650502203	0.515367879	0.716335759	0.058776888
ENSMUSG00000031948	Kars	85305	protein_coding	1444.090208	-0.058813531	0.078180823	0.752275668	0.451885297	0.668257706	0.058813531
ENSMUSG00000024269	Tpgs2	66648	protein_coding	83.5255598	0.058820115	0.171180539	-0.3436145	0.731136218	0.861504353	0.058820115
ENSMUSG00000106464	C130083M11Rik	414111	lncRNA	10.74303857	0.058831254	0.314294711	-0.187184994	0.85151559	0.927499565	0.058831254
ENSMUSG00000022295	Atp6v1c1	66335	protein_coding	419.9322619	-0.058885387	0.070121551	0.839761626	0.401042053	0.622737045	0.058885387
ENSMUSG00000026439	Rbbp5	213464	protein_coding	372.1020827	0.05890086	0.074717837	-0.788310565	0.430515069	0.648975432	0.05890086
ENSMUSG00000010376	Nedd8	18002	protein_coding	810.615637	-0.058937782	0.060602109	0.972536807	0.330783563	0.553114597	0.058937782
ENSMUSG00000039047	Pigk	329777	protein_coding	505.3824965	-0.058943045	0.091824573	0.641909278	0.520932089	0.720614094	0.058943045
ENSMUSG00000049303	Syt12	171180	protein_coding	12.37763451	-0.058945688	0.367871996	0.160234235	0.872696562	0.937225143	0.058945688
ENSMUSG00000021707	Dhfr	13361	protein_coding	184.6434921	0.05894921	0.108010815	-0.545771371	0.585223105	0.767282764	0.05894921
ENSMUSG00000043088	Il17re	57890	protein_coding	298.3563706	-0.058974973	0.108878979	0.541656192	0.588055374	0.769309474	0.058974973
ENSMUSG00000017264	Exosc10	50912	protein_coding	446.5303307	-0.059051944	0.073522457	0.803182408	0.421869312	0.641714691	0.059051944
ENSMUSG00000044367	Slc16a13	69309	protein_coding	81.84054082	-0.059058069	0.120119157	0.491662363	0.622958049	0.793281643	0.059058069
ENSMUSG00000011148	Adssl1	11565	protein_coding	4.061033187	0.059060448	0.528475531	-0.111756258	0.911016672	1	0.059060448
ENSMUSG00000042303	Sgsm3	105835	protein_coding	1531.056034	0.059096091	0.105140498	-0.562067831	0.574069805	0.759387116	0.059096091
ENSMUSG00000000194	Gpr107	277463	protein_coding	1049.89801	-0.05912774	0.052068546	1.135575021	0.256134468	0.468895375	0.05912774
ENSMUSG00000038784	Cnot4	53621	protein_coding	198.9181572	-0.059151401	0.080247895	0.737108434	0.461056411	0.674995696	0.059151401
ENSMUSG00000034538	Zfp418	232854	protein_coding	39.54956756	-0.059178904	0.183212918	0.323006176	0.746690565	0.872440598	0.059178904
ENSMUSG00000103711	Tstd1	226654	protein_coding	1445.58105	-0.059182376	0.115701392	0.511509631	0.608994246	0.783067117	0.059182376
ENSMUSG00000040848	Sft2d2	108735	protein_coding	2626.181642	-0.059215131	0.056881554	1.041025207	0.297863849	0.517488122	0.059215131
ENSMUSG00000034738	Nostrin	329416	protein_coding	802.6478818	0.059216936	0.087088676	-0.679961377	0.496528917	0.702568634	0.059216936

Supplementary Table 1

ensembl_gene_id	external_gene_name	entrezgene_id	gene_biotype	baseMean	log2FoldChange	lfcSE	stat	pvalue	padj	absLog2FC
ENSMUSG00000091512	Lamtor3	56692	protein_coding	315.5351406	-0.059217509	0.080399566	0.736540157	0.461402038	0.675184655	0.059217509
ENSMUSG00000052369	Tmem106c	380967	protein_coding	171.2943808	-0.059222325	0.126758949	0.467204291	0.640353724	0.803678477	0.059222325
ENSMUSG00000006638	Abhd1	57742	protein_coding	6.178501974	-0.059253962	0.614740058	0.096388646	0.923211909	0.962738158	0.059253962
ENSMUSG00000035529	Prdm4	72843	protein_coding	374.4402374	0.059262894	0.066313618	-0.893676073	0.371495231	0.593888891	0.059262894
ENSMUSG00000020707	Rnf135	71956	protein_coding	78.5694516	-0.059266943	0.127479031	0.464915227	0.641992166	0.804573016	0.059266943
ENSMUSG00000019927	Ube2d1	216080	protein_coding	113.3980404	-0.059281539	0.125222958	0.473407916	0.635922183	0.801613541	0.059281539
ENSMUSG00000001783	Rtcb	28088	protein_coding	1753.385115	-0.059308757	0.102759187	0.577162575	0.563829637	0.751075471	0.059308757
ENSMUSG00000055882	Abhd16b	241850	protein_coding	2.56245946	0.059363746	0.67441636	-0.088022399	0.929858874	1	0.059363746
ENSMUSG00000059920	4930453N24Rik	67609	protein_coding	337.1834794	-0.059367844	0.089150896	0.665925374	0.505458801	0.709429427	0.059367844
ENSMUSG00000022055	Nefl	18039	protein_coding	3.368687167	0.059484739	0.664312351	-0.089543328	0.928650121	1	0.059484739
ENSMUSG00000030538	Cib1	23991	protein_coding	1889.924488	0.059526833	0.065375373	-0.910539089	0.362538277	0.584928009	0.059526833
ENSMUSG00000024273	2700062C07Rik	68046	protein_coding	96.32911124	-0.059536749	0.124765434	0.477189449	0.633227223	0.800207711	0.059536749
ENSMUSG00000014769	Psmb1	19170	protein_coding	1661.638129	0.059545846	0.091965757	-0.647478453	0.517322335	0.717843966	0.059545846
ENSMUSG00000047867	Gimap6	231931	protein_coding	26.5092813	0.059555951	0.337590962	-0.176414532	0.859968289	0.931308041	0.059555951
ENSMUSG00000041673	Lrrc18	67580	protein_coding	1.367741268	-0.059556979	1.282539305	0.046436767	0.962962132	1	0.059556979
ENSMUSG00000031576	Kcnu1	16532	protein_coding	4.264300142	-0.059571653	0.502570972	0.118533812	0.905644705	1	0.059571653
ENSMUSG00000028099	Polr3c	74414	protein_coding	448.8417663	-0.059572485	0.081198667	0.733663328	0.463153954	0.676807614	0.059572485
ENSMUSG00000002957	Ap2a2	11772	protein_coding	1403.244422	0.059578067	0.057529002	-1.035617948	0.300380415	0.520512296	0.059578067
ENSMUSG00000057637	Prdm2	110593	protein_coding	482.5717085	0.05958457	0.110323639	-0.540088878	0.589135741	0.770185847	0.05958457
ENSMUSG00000030741	Spns1	73658	protein_coding	405.5596154	0.059595994	0.085692118	-0.69546646	0.486763024	0.695624496	0.059595994
ENSMUSG00000116648	Rpl31-ps12	665562	processed_pseudogene	1.16345761	0.05962217	1.031870377	-0.057780678	0.95392333	1	0.05962217
ENSMUSG00000049421	Zfp260	26466	protein_coding	428.5618219	0.059627729	0.132658126	-0.449484183	0.653082415	0.811818066	0.059627729
ENSMUSG00000074269	Rec114	73673	protein_coding	2.937927397	0.059680115	0.809538556	-0.073721152	0.941232268	1	0.059680115
ENSMUSG00000031796	Cfap20	14894	protein_coding	253.0089725	0.059707864	0.097129237	-0.614725962	0.538735711	0.733468492	0.059707864
ENSMUSG00000026988	Wdsub1	72137	protein_coding	186.4479868	0.059735629	0.110930629	-0.538495362	0.590235106	0.770846581	0.059735629
ENSMUSG00000022671	Mzt2	72083	protein_coding	144.0271302	-0.059803919	0.116729032	0.512331152	0.608419266	0.782626881	0.059803919
ENSMUSG00000019982	Myb	17863	protein_coding	1250.196708	0.059873021	0.146378163	-0.409029733	0.682517842	0.830405926	0.059873021
ENSMUSG00000025735	Rhbdl1	214951	protein_coding	11.26657583	-0.059940983	0.340977035	0.175791849	0.860457474	0.931471303	0.059940983
ENSMUSG00000052917	Senp7	66315	protein_coding	197.1584131	-0.05996583	0.107379816	0.558446008	0.576539863	0.761222618	0.05996583
ENSMUSG00000032812	Arap1	69710	protein_coding	1155.941913	0.059988863	0.057848384	-1.03700155	0.299735135	0.519697021	0.059988863
ENSMUSG00000051786	Tubgcp6	328580	protein_coding	295.5191081	0.06001989	0.098054065	-0.612110164	0.540464873	0.73470834	0.06001989
ENSMUSG00000034570	Inpp5j	170835	protein_coding	862.3470057	0.060069566	0.222961191	-0.269417141	0.787608697	0.893615254	0.060069566
ENSMUSG00000026578	Ccdc181	74895	protein_coding	115.5476576	-0.060088481	0.108015815	0.556293365	0.578010322	0.76182422	0.060088481
ENSMUSG00000004054	Map3k11	26403	protein_coding	1005.545006	0.060103976	0.072821742	-0.825357564	0.40916862	0.629730716	0.060103976
ENSMUSG00000000567	Sox9	20682	protein_coding	520.5531139	0.060118921	0.107166314	-0.560987116	0.57480632	0.760117839	0.060118921
ENSMUSG00000031333	Abcb7	11306	protein_coding	885.9039725	-0.060120583	0.09279499	0.64788609	0.517058628	0.717711842	0.060120583
ENSMUSG00000033615	Cplx1	12889	protein_coding	1.071992129	-0.060137987	1.814992504	0.033134014	0.973567719	1	0.060137987
ENSMUSG00000032726	Bmp8a	12163	protein_coding	79.37403579	-0.060165004	0.155363055	0.387254256	0.698567987	0.840298483	0.060165004
ENSMUSG00000084798	4930533B01Rik	75166	lncRNA	7.237681953	-0.060173634	0.476480458	0.126287727	0.899504171	0.950621521	0.060173634

ensembl_gene_id	external_gene_name	entrezgene_id	gene_biotype	baseMean	log2FoldChange	lfcSE	stat	pvalue	padj	absLog2FC
ENSMUSG00000051504	Siglech	233274	protein_coding	2.478526697	-0.060247903	1.786072951	0.03373205	0.973090821	1	0.060247903
ENSMUSG00000018849	Wwc1	211652	protein_coding	736.3357747	-0.06035137	0.079903322	0.755304885	0.450066068	0.666821375	0.06035137
ENSMUSG00000006526	Stimate	69179	protein_coding	156.8328039	0.060355174	0.105867972	-0.570098525	0.568610876	0.754583735	0.060355174
ENSMUSG00000064090	Vrk2	69922	protein_coding	83.47234383	0.060360134	0.118269102	-0.510362664	0.609797409	0.783550764	0.060360134
ENSMUSG00000025920	Stau2	29819	protein_coding	173.0622286	0.060375179	0.092096814	-0.655562076	0.512105933	0.714689206	0.060375179
ENSMUSG00000028060	Khdc4	74200	protein_coding	641.2375582	0.060384967	0.117372097	-0.514474634	0.606920191	0.781739336	0.060384967
ENSMUSG00000016921	Srsf6	67996	protein_coding	1530.817171	0.060420672	0.06781649	-0.890943657	0.372959398	0.595257447	0.060420672
ENSMUSG00000097177	9330159M07Rik	319673	lncRNA	19.47911415	0.060424378	0.267235111	-0.226109429	0.821116311	0.909608677	0.060424378
ENSMUSG00000024535	Snx24	69226	protein_coding	241.0129987	0.060425642	0.129666375	-0.46600857	0.641209366	0.804106371	0.060425642
ENSMUSG00000031845	Bco1	63857	protein_coding	27.50523309	0.060465097	0.247925522	-0.243884116	0.807320571	0.904987729	0.060465097
ENSMUSG00000041629	Fam104a	28081	protein_coding	176.4185183	-0.060477996	0.108516768	0.557314761	0.577312392	0.761298159	0.060477996
ENSMUSG00000035236	Scai	320271	protein_coding	95.66243056	0.060498086	0.137319896	-0.440563146	0.659529288	0.8161622	0.060498086
ENSMUSG00000074064	Mlycd	56690	protein_coding	452.9213898	0.060536959	0.063968927	-0.94634945	0.343970379	0.56579982	0.060536959
ENSMUSG00000042548	Asxl1	228790	protein_coding	679.0116527	-0.060542079	0.061848506	0.978876987	0.327640764	0.549807904	0.060542079
ENSMUSG00000032462	Pik3cb	74769	protein_coding	172.469929	0.06054469	0.091608643	-0.660905873	0.50867268	0.712245263	0.06054469
ENSMUSG00000043557	Mdga1	74762	protein_coding	13.13685766	0.060584822	0.579957088	-0.104464319	0.916800883	0.959274295	0.060584822
ENSMUSG00000042211	Fbxo38	107035	protein_coding	877.4665073	0.060597605	0.052276328	-1.159178681	0.246383359	0.458272537	0.060597605
ENSMUSG00000037997	Parp11	101187	protein_coding	116.4256213	-0.06062911	0.131654726	0.460516011	0.645145882	0.80668897	0.06062911
ENSMUSG00000035569	Ankrd11	77087	protein_coding	2434.147604	0.060642478	0.085414731	-0.709976809	0.477718517	0.68930064	0.060642478
ENSMUSG00000004071	Cdip1	66626	protein_coding	330.1607526	-0.060657373	0.087653757	0.692011105	0.488930355	0.696651931	0.060657373
ENSMUSG00000024776	Stambpl1	76630	protein_coding	212.6538638	0.060676547	0.09238615	-0.656771038	0.511328149	0.71414691	0.060676547
ENSMUSG00000062040	Zfp27	22689	protein_coding	124.4393726	0.060682221	0.094072654	-0.645056971	0.518890267	0.719334577	0.060682221
ENSMUSG00000006538	Ihh	16147	protein_coding	1115.852292	-0.060693169	0.092290631	0.657630883	0.510775345	0.713797415	0.060693169
ENSMUSG00000033985	Tesk2	230661	protein_coding	377.7315813	0.06073987	0.130705104	-0.464709244	0.642139688	0.804696828	0.06073987
ENSMUSG00000031622	Sin3b	20467	protein_coding	825.8050992	0.060741916	0.043182706	-1.406625985	0.159538324	0.343720271	0.060741916
ENSMUSG00000022387	Brd1	223770	protein_coding	1284.146688	-0.06080027	0.058097237	1.046526006	0.295318244	0.514433539	0.06080027
ENSMUSG00000021314	Amph	218038	protein_coding	29.39039281	0.060845096	0.255174592	-0.23844496	0.811535999	0.906704483	0.060845096
ENSMUSG00000030032	Wdr54	75659	protein_coding	20.14062541	0.060879373	0.376937695	-0.161510439	0.87169139	0.936825965	0.060879373
ENSMUSG00000068290	Ddrgk1	77006	protein_coding	799.0359983	-0.060889882	0.066770548	0.911927242	0.36180702	0.584169058	0.060889882
ENSMUSG00000044433	Camsap3	69697	protein_coding	832.1750987	0.060898674	0.082113805	-0.741637464	0.458307019	0.67278137	0.060898674
ENSMUSG00000002384	Bmp8b	12164	protein_coding	13.18293562	-0.060901687	0.453089911	0.134414132	0.893075108	0.947328174	0.060901687
ENSMUSG00000064165	Krt39	237934	protein_coding	0.880641899	-0.060901777	1.339613039	0.045462216	0.963738891	1	0.060901777
ENSMUSG00000006215	Zbtb17	22642	protein_coding	323.9627921	-0.060904489	0.065922031	0.923886719	0.355545298	0.578413462	0.060904489
ENSMUSG00000010277	Bltp2	72503	protein_coding	4626.661819	-0.060909872	0.056132627	1.085106382	0.277874541	0.494858209	0.060909872
ENSMUSG00000044024	Rell2	225392	protein_coding	8.302219111	-0.060931601	0.459193283	0.132692709	0.894436409	0.947833434	0.060931601
ENSMUSG00000028580	Pum1	80912	protein_coding	1063.33865	0.060950128	0.05278898	-1.154599461	0.248254488	0.460585845	0.060950128
ENSMUSG00000028886	Eya3	14050	protein_coding	480.1215137	0.06096203	0.068444888	-0.89067324	0.373104495	0.595366473	0.06096203
ENSMUSG00000034009	Rxfp1	381489	protein_coding	3.586262302	0.060966845	2.242687417	-0.027184727	0.978312397	1	0.060966845
ENSMUSG00000070661	Rnf186	66825	protein_coding	1679.074283	-0.061029613	0.118659847	0.514324052	0.607025449	0.781796866	0.061029613

ensembl_gene_id	external_gene_name	entrezgene_id	gene_biotype	baseMean	log2FoldChange	lfcSE	stat	pvalue	padj	absLog2FC
ENSMUSG00000028771	Ptpn12	19248	protein_coding	324.5785497	-0.061082831	0.081455684	0.749890342	0.453320752	0.669122175	0.061082831
ENSMUSG00000025316	Banp	53325	protein_coding	183.3974089	0.061128306	0.21471149	-0.284699742	0.775874189	0.888050066	0.061128306
ENSMUSG00000031016	Wee1	22390	protein_coding	163.8924021	0.061129666	0.154545554	-0.395544642	0.692440983	0.836513744	0.061129666
ENSMUSG00000019370	Calm3	12315	protein_coding	5825.809008	-0.061129972	0.072255077	0.846030123	0.39753594	0.619446591	0.061129972
ENSMUSG00000031145	Prickle3	54630	protein_coding	115.4786011	-0.061145528	0.117080693	0.522251158	0.601495471	0.777725809	0.061145528
ENSMUSG00000098678	Mroh6	223645	protein_coding	78.25381665	0.061150745	0.597267681	-0.102384152	0.918451763	0.960078011	0.061150745
ENSMUSG00000000600	Krit1	79264	protein_coding	322.6532954	-0.061152756	0.085467716	0.715507081	0.474295767	0.686291751	0.061152756
ENSMUSG00000035958	Tdp2	56196	protein_coding	273.2652868	-0.061161461	0.082427405	0.74200397	0.45808493	0.672740084	0.061161461
ENSMUSG00000038766	Gabpb2	213054	protein_coding	962.1262061	0.061165743	0.078706633	-0.777135813	0.437078646	0.655340182	0.061165743
ENSMUSG00000026404	Ddx59	67997	protein_coding	50.29384407	0.061195165	0.192110451	-0.31854157	0.750074167	0.874455706	0.061195165
ENSMUSG00000060407	Cyp2a12	13085	protein_coding	2.384692582	0.061320056	1.378485211	-0.044483652	0.964518883	1	0.061320056
ENSMUSG00000005882	Uqcc1	56046	protein_coding	642.8517209	-0.061336181	0.089203118	0.687601313	0.491703881	0.698494442	0.061336181
ENSMUSG00000058486	Wdr91	101240	protein_coding	50.62106656	-0.06134655	0.181742185	0.337547113	0.735704503	0.865466887	0.06134655
ENSMUSG00000032565	Nudt16	75686	protein_coding	229.6389046	0.061352647	0.083587073	-0.733996836	0.462950667	0.676623357	0.061352647
ENSMUSG00000039099	Wdr93	626359	protein_coding	5.342610216	-0.061352731	0.472133586	0.129947821	0.896607709	0.94900212	0.061352731
ENSMUSG00000019907	Ppp1r12a	17931	protein_coding	1271.924976	-0.06137262	0.068211371	0.899741774	0.368257687	0.5907413	0.06137262
ENSMUSG00000042167	Tent2	100715	protein_coding	279.4524173	0.061380938	0.092431119	-0.664072218	0.50664409	0.710428845	0.061380938
ENSMUSG00000022750	Klhl22	224023	protein_coding	411.2233039	0.06140126	0.07867276	-0.780464034	0.435117789	0.653468095	0.06140126
ENSMUSG00000057789	Bak1	12018	protein_coding	979.8344782	-0.061415208	0.079682826	0.770745859	0.440857584	0.658733455	0.061415208
ENSMUSG00000018846	Pank3	211347	protein_coding	4424.538117	0.061415995	0.093878611	-0.65420648	0.512978787	0.714956306	0.061415995
ENSMUSG00000004113	Cacna1b	12287	protein_coding	96.21697865	0.061426303	0.141976312	-0.432651771	0.665267772	0.819481407	0.061426303
ENSMUSG00000020745	Pafah1b1	18472	protein_coding	2167.125183	0.061429667	0.051980199	-1.181789755	0.237289134	0.44767168	0.061429667
ENSMUSG00000006299	Aamp	227290	protein_coding	1509.329689	0.061430623	0.051585786	-1.190843992	0.233714835	0.443169915	0.061430623
ENSMUSG00000062014	Gmfb	63985	protein_coding	944.1541707	-0.061433919	0.094870648	0.647554544	0.517273105	0.717835971	0.061433919
ENSMUSG00000026123	Plekhb2	226971	protein_coding	4353.610701	-0.061449204	0.051137646	1.2016432	0.229501797	0.438111017	0.061449204
ENSMUSG00000037157	Il22ra1	230828	protein_coding	399.7007517	0.061454037	0.169171004	-0.363265783	0.716406351	0.852797822	0.061454037
ENSMUSG00000071350	Setdb2	239122	protein_coding	131.0039703	0.061459596	0.10278957	-0.597916661	0.549895542	0.74082028	0.061459596
ENSMUSG00000028800	Hdac1	433759	protein_coding	925.6909707	-0.061465602	0.095812491	0.641519715	0.521185076	0.720841738	0.061465602
ENSMUSG00000025035	Arl3	56350	protein_coding	63.51519192	0.061491482	0.151282512	-0.406467879	0.684398854	0.831662311	0.061491482
ENSMUSG00000038506	Dcun1d2	102323	protein_coding	285.0660373	-0.061492623	0.083675509	0.734893921	0.462404101	0.675987813	0.061492623
ENSMUSG00000025592	Dach2	93837	protein_coding	7.424815713	0.061495585	0.42136168	-0.145944891	0.883964893	0.94246215	0.061495585
ENSMUSG00000015943	Bola1	69168	protein_coding	146.1559798	-0.061509539	0.115134172	0.534242246	0.593173944	0.772538316	0.061509539
ENSMUSG00000029466	Anapc7	56317	protein_coding	304.508052	-0.061536297	0.094823147	0.6489586	0.516365136	0.717238948	0.061536297
ENSMUSG00000038563	Efl1	101592	protein_coding	322.9297096	-0.061598343	0.070430852	0.874593181	0.381795299	0.604102573	0.061598343
ENSMUSG00000039512	Uhrf1bp1	224648	protein_coding	208.3966934	-0.061700116	0.132017059	0.467364725	0.640238956	0.803678477	0.061700116
ENSMUSG00000099145	Mir8116	102466887	miRNA	2.649642625	0.061735789	0.861004096	-0.071702085	0.942838997	1	0.061735789
ENSMUSG00000071856	Mcc	328949	protein_coding	329.3948469	-0.061740899	0.133207244	0.463495055	0.643009558	0.805274537	0.061740899
ENSMUSG00000056204	Pgpep1	66522	protein_coding	685.0482887	-0.061835205	0.089835714	0.688314286	0.491254887	0.698491194	0.061835205
ENSMUSG00000035933	Cog5	238123	protein_coding	575.6117785	-0.061858348	0.073523782	0.841337958	0.400158632	0.622301501	0.061858348

ensembl_gene_id	external_gene_name	entrezgene_id	gene_biotype	baseMean	log2FoldChange	lfcSE	stat	pvalue	padj	absLog2FC
ENSMUSG00000044156	Hepacam2	101202	protein_coding	1616.887732	-0.061873139	0.272669658	0.226916115	0.820488971	0.9092783	0.061873139
ENSMUSG00000054435	Gimap4	107526	protein_coding	32.80574215	-0.061901439	0.343514482	0.180200376	0.856995263	0.93035707	0.061901439
ENSMUSG00000026333	Gin1	252876	protein_coding	68.20994423	0.061916074	0.130905009	-0.47298476	0.636224052	0.801687789	0.061916074
ENSMUSG00000031917	Nip7	66164	protein_coding	203.7250508	-0.061919432	0.102029963	0.606874976	0.543933886	0.73666903	0.061919432
ENSMUSG00000052914	Cyp2j6	13110	protein_coding	834.3909343	0.061920104	0.093147629	-0.66475233	0.506208917	0.710135685	0.061920104
ENSMUSG00000052144	Ppp4r2	232314	protein_coding	525.6202662	-0.06193902	0.100703552	0.615062909	0.538513176	0.733377497	0.06193902
ENSMUSG00000017631	Abr	109934	protein_coding	2774.525899	-0.061975022	0.082204708	0.753910855	0.450902752	0.667636772	0.061975022
ENSMUSG00000031169	Porcn	53627	protein_coding	28.38268476	-0.061979962	0.239555833	0.25872867	0.795844597	0.898576349	0.061979962
ENSMUSG00000020900	Myh10	77579	protein_coding	20.2883211	0.062001891	0.45532795	-0.136169745	0.891687093	0.947050968	0.062001891
ENSMUSG00000053868	Gm5142	380907	lncRNA	1.833544745	0.062008962	0.995754509	-0.062273343	0.950345156	1	0.062008962
ENSMUSG00000052632	Asap2	211914	protein_coding	461.0613836	-0.062009271	0.115364505	0.537507365	0.590917199	0.771232643	0.062009271
ENSMUSG00000034377	Tulp4	68842	protein_coding	854.1876338	-0.062024402	0.065497836	0.946968731	0.343654713	0.56547568	0.062024402
ENSMUSG00000021820	Camk2g	12325	protein_coding	144.3587938	0.062061604	0.102098931	-0.60785753	0.543281969	0.736456016	0.062061604
ENSMUSG00000040717	Il17rd	171463	protein_coding	58.97202401	-0.062083096	0.184850121	0.335856398	0.736979158	0.86653427	0.062083096
ENSMUSG00000055493	Epm2a	13853	protein_coding	13.47981553	-0.062097238	0.360304729	0.172346441	0.863165176	0.932166328	0.062097238
ENSMUSG00000011837	Snapc2	102209	protein_coding	148.1569327	-0.062131293	0.093999579	0.660974154	0.508628889	0.712244337	0.062131293
ENSMUSG00000063884	Ptcd3	69956	protein_coding	520.0081868	-0.062151163	0.091830734	0.676801327	0.498532016	0.703739933	0.062151163
ENSMUSG00000038150	Ormdl3	66612	protein_coding	273.7271575	0.06219563	0.095479071	-0.651405898	0.514784504	0.716272317	0.06219563
ENSMUSG00000022489	Pde1b	18574	protein_coding	75.35047773	0.062223239	0.124893425	-0.498210689	0.618335549	0.789606655	0.062223239
ENSMUSG00000055943	Emc7	73024	protein_coding	501.0723743	0.062229735	0.095505558	-0.65158234	0.514670643	0.716210584	0.062229735
ENSMUSG00000022075	Rhobtb2	246710	protein_coding	192.9243397	-0.062271849	0.079162519	0.786632987	0.431496743	0.649802774	0.062271849
ENSMUSG00000072825	Cep170b	217882	protein_coding	2206.006064	-0.062341457	0.052783361	1.181081622	0.2375703	0.447960257	0.062341457
ENSMUSG00000029790	Cep41	83922	protein_coding	57.15421145	0.062348702	0.18083365	-0.344784843	0.730256129	0.861143224	0.062348702
ENSMUSG00000052957	Gas1	14451	protein_coding	0.765117643	-0.062364763	1.743161708	0.035776809	0.971460325	1	0.062364763
ENSMUSG00000014554	Dguok	27369	protein_coding	202.5056706	0.062403652	0.091415846	-0.682634956	0.49483754	0.700835947	0.062403652
ENSMUSG00000058076	Sdhc	66052	protein_coding	2220.521394	-0.062427277	0.093026025	0.671073245	0.50217387	0.706921771	0.062427277
ENSMUSG00000057130	Txnl4a	27366	protein_coding	242.7498073	0.062490468	0.108085242	-0.578159116	0.563156698	0.750770075	0.062490468
ENSMUSG00000034021	Pds5b	100710	protein_coding	255.6994972	0.06252291	0.127405013	-0.490741368	0.623609384	0.793440482	0.06252291
ENSMUSG00000008859	Rala	56044	protein_coding	781.4618439	0.062539738	0.06934238	-0.90189777	0.367111176	0.589852079	0.062539738
ENSMUSG00000028102	Pex11b	18632	protein_coding	122.9769866	-0.062553974	0.120248356	0.520206475	0.602919673	0.778895054	0.062553974
ENSMUSG00000041975	Mettl8	228019	protein_coding	98.69917926	0.062569794	0.119991431	-0.521452183	0.602051808	0.778258299	0.062569794
ENSMUSG00000032583	Mon1a	72825	protein_coding	503.5632156	0.062579735	0.090006032	-0.695283786	0.486877473	0.695624496	0.062579735
ENSMUSG00000047557	Lxn	17035	protein_coding	121.1771449	-0.06259451	0.11672663	0.536248755	0.591786642	0.771818402	0.06259451
ENSMUSG00000003423	Pih1d1	68845	protein_coding	366.8195032	0.062607605	0.083465942	-0.750097624	0.45319591	0.669122175	0.062607605
ENSMUSG00000022808	Snx4	69150	protein_coding	733.6582934	-0.06266725	0.076948599	0.814404047	0.415413521	0.635668911	0.06266725
ENSMUSG00000000531	Tamalin	56149	protein_coding	71.02418755	0.062735671	0.245182242	-0.255873632	0.798048416	0.899834189	0.062735671
ENSMUSG00000037594	Clba1	217887	protein_coding	27.57640884	0.062800947	0.194237475	-0.323320447	0.746452571	0.872440598	0.062800947
ENSMUSG00000044636	Csrnp2	207785	protein_coding	76.55076962	0.062816033	0.204740013	-0.306808777	0.758988927	0.878912418	0.062816033
ENSMUSG00000052812	Atad2b	320817	protein_coding	542.8856793	0.062853271	0.064572837	-0.973370135	0.330369377	0.552639068	0.062853271

ensembl_gene_id	external_gene_name	entrezgene_id	gene_biotype	baseMean	log2FoldChange	lfcSE	stat	pvalue	padj	absLog2FC
ENSMUSG00000024427	Spry4	24066	protein_coding	48.32217485	-0.062857895	0.259821009	0.241927684	0.808836199	0.905678041	0.062857895
ENSMUSG00000058690	Ccser2	72972	protein_coding	650.7097907	0.062898141	0.056587777	-1.111514616	0.266346902	0.481401448	0.062898141
ENSMUSG00000027556	Car1	12346	protein_coding	64053.33163	-0.062906335	0.178487572	0.35244098	0.724507573	0.857302814	0.062906335
ENSMUSG00000071076	Jund	16478	protein_coding	2533.569084	-0.062907297	0.149663282	0.420325522	0.674247668	0.825194181	0.062907297
ENSMUSG00000032412	Atp1b3	11933	protein_coding	454.2286601	-0.062919808	0.118356483	0.531612693	0.594994272	0.773528957	0.062919808
ENSMUSG00000021706	Zfyve16	218441	protein_coding	415.4504947	-0.06293298	0.078923585	0.7973913	0.42522381	0.644825161	0.06293298
ENSMUSG00000029055	Plch2	269615	protein_coding	847.6445335	0.062961947	0.093703582	-0.671926793	0.501630302	0.706397443	0.062961947
ENSMUSG00000022307	Oxr1	170719	protein_coding	522.0198206	0.062972618	0.079839179	-0.788743312	0.430262048	0.648871201	0.062972618
ENSMUSG00000070643	Sox13	20668	protein_coding	590.0932493	0.063016424	0.1249677	-0.504261695	0.614077492	0.786724259	0.063016424
ENSMUSG00000058407	Txndc9	98258	protein_coding	688.3861576	-0.063028424	0.074381273	0.847369529	0.396789183	0.618855346	0.063028424
ENSMUSG00000021694	Ercc8	71991	protein_coding	93.07374297	0.063042579	0.125526873	-0.50222377	0.615510124	0.787780401	0.063042579
ENSMUSG00000037652	Phc3	241915	protein_coding	467.9703507	-0.063047429	0.096340997	0.654419518	0.512841563	0.714956306	0.063047429
ENSMUSG00000046999	1110032F04Rik	68725	protein_coding	1.983058731	-0.06305595	1.287180339	0.048987658	0.960929132	1	0.06305595
ENSMUSG00000060098	Prmt7	214572	protein_coding	276.8985201	-0.063076619	0.138396747	0.455766634	0.6485578	0.809117134	0.063076619
ENSMUSG00000032737	Inppl1	16332	protein_coding	1270.805821	-0.063077663	0.066469695	0.948968747	0.342636508	0.564644176	0.063077663
ENSMUSG00000043279	Trim56	384309	protein_coding	766.1801347	-0.063087466	0.070705032	0.892262751	0.372252115	0.594533905	0.063087466
ENSMUSG00000062944	9130023H24Rik	100043133	protein_coding	96.30482037	-0.063114579	0.150781277	0.418583662	0.675520432	0.826153034	0.063114579
ENSMUSG00000047264	Zfp358	140482	protein_coding	131.9875309	0.063139125	0.117280743	-0.538358845	0.590329333	0.770846581	0.063139125
ENSMUSG00000027531	Impa1	55980	protein_coding	965.3801658	0.063160847	0.070969971	-0.8899658	0.37348425	0.59579974	0.063160847
ENSMUSG00000025231	Sufu	24069	protein_coding	98.91341521	-0.063177379	0.133708622	0.472500413	0.636569647	0.801948109	0.063177379
ENSMUSG00000024151	Msh2	17685	protein_coding	385.5560742	0.063204277	0.143207401	-0.441347838	0.658961199	0.815932012	0.063204277
ENSMUSG00000022022	Mtrf1	211253	protein_coding	66.51060607	-0.063254893	0.144393908	0.438071756	0.661334268	0.816921981	0.063254893
ENSMUSG00000023806	Rsph3b	100037282	protein_coding	165.1372348	0.063319883	0.127678931	-0.495930557	0.619943406	0.790987051	0.063319883
ENSMUSG00000028691	Prdx1	18477	protein_coding	4199.930351	-0.063349832	0.098314204	0.64436093	0.519341414	0.719557354	0.063349832
ENSMUSG00000019659	Ccdc12	72654	protein_coding	599.3843879	-0.063366826	0.096378217	0.657480789	0.51087182	0.713811425	0.063366826
ENSMUSG00000028182	Lrriq3	74435	protein_coding	20.52989199	-0.063433665	0.282643686	0.224429798	0.822422886	0.910171686	0.063433665
ENSMUSG00000051209	Gpr119	236781	protein_coding	4.406998855	0.063455466	0.559434421	-0.113427889	0.90969133	0.956064572	0.063455466
ENSMUSG00000020921	Tmem101	76547	protein_coding	121.268534	-0.063505803	0.108277979	0.586507094	0.557534795	0.74672584	0.063505803
ENSMUSG00000069089	Cdk7	12572	protein_coding	277.1983076	0.06350932	0.086606562	-0.733308405	0.46337035	0.676855263	0.06350932
ENSMUSG00000030246	Ldhb	16832	protein_coding	64.31383397	-0.063523439	0.15852737	0.400709597	0.688633944	0.83386713	0.063523439
ENSMUSG00000073411	H2-D1	14964	protein_coding	7161.649703	0.063526428	0.084616897	-0.750753465	0.45280104	0.669012828	0.063526428
ENSMUSG00000038170	Pde4dip	83679	protein_coding	1856.785113	0.063595446	0.067188181	-0.946527277	0.343879716	0.565733178	0.063595446
ENSMUSG00000028049	Scamp3	24045	protein_coding	145.4763278	0.063649089	0.119499359	-0.532631215	0.59428889	0.77323163	0.063649089
ENSMUSG00000041781	Cpsf2	51786	protein_coding	678.6042163	0.063674471	0.093130498	-0.683712338	0.494156831	0.700292277	0.063674471
ENSMUSG00000031286	Glt28d2	320302	protein_coding	104.3562161	-0.063679242	0.173715661	0.366571679	0.713938537	0.851508089	0.063679242
ENSMUSG00000022607	Ptk2	14083	protein_coding	925.6641341	0.063683502	0.055973053	-1.137752873	0.255223689	0.467839271	0.063683502
ENSMUSG00000062190	Lancl2	71835	protein_coding	158.5674588	-0.063695053	0.140773984	0.45246324	0.650935298	0.810260975	0.063695053
ENSMUSG00000032504	Pdcd6ip	18571	protein_coding	2706.283118	0.063722691	0.079138979	-0.80519981	0.420704386	0.640678	0.063722691
ENSMUSG00000018189	Uchl5	56207	protein_coding	202.0796214	0.063769874	0.145638544	-0.437863988	0.661484882	0.816921981	0.063769874

Supplementary Table 1

ensembl_gene_id	external_gene_name	entrezgene_id	gene_biotype	baseMean	log2FoldChange	lfcSE	stat	pvalue	padj	absLog2FC
ENSMUSG00000041645	Ddx24	27225	protein_coding	798.9114487	-0.063789929	0.06348228	1.004846219	0.314970905	0.535990818	0.063789929
ENSMUSG00000037089	Slc35b2	73836	protein_coding	664.4692759	0.063797209	0.091440588	-0.697690273	0.485370913	0.695380179	0.063797209
ENSMUSG00000040025	Ythdf2	213541	protein_coding	814.6121221	-0.063836101	0.09137976	0.698580313	0.484814351	0.695226137	0.063836101
ENSMUSG00000038366	Lasp1	16796	protein_coding	4134.065352	-0.063868814	0.057625947	1.108334312	0.267717462	0.482962166	0.063868814
ENSMUSG00000022855	Senp2	75826	protein_coding	460.3352808	0.063925753	0.071299849	-0.896576269	0.369945066	0.59260115	0.063925753
ENSMUSG00000025200	Cwf19l1	72502	protein_coding	126.6303118	0.063973174	0.108460839	-0.589827396	0.555306373	0.745058233	0.063973174
ENSMUSG00000044533	Rps2	16898	protein_coding	8938.095252	-0.063980306	0.102688345	0.62305324	0.533249536	0.729644779	0.063980306
ENSMUSG00000011158	Brf1	72308	protein_coding	336.7736962	-0.064024964	0.098557022	0.649623567	0.515935406	0.716883239	0.064024964
ENSMUSG00000029491	Pde6b	18587	protein_coding	4.616758124	0.064090933	0.793474925	-0.080772474	0.935622899	0.96968875	0.064090933
ENSMUSG00000028383	Hsdl2	72479	protein_coding	378.002759	-0.064101488	0.085614494	0.748722378	0.454024553	0.669801811	0.064101488
ENSMUSG00000072772	Grcc10	14790	protein_coding	185.8288976	-0.064109057	0.116257342	0.551440932	0.581331448	0.764339772	0.064109057
ENSMUSG00000028992	Nmnat1	66454	protein_coding	159.3185053	-0.064153076	0.105808623	0.606312358	0.544307354	0.736796542	0.064153076
ENSMUSG00000029650	Slc46a3	71706	protein_coding	80.90939854	0.06415446	0.265741767	-0.241416547	0.809232289	0.905779176	0.06415446
ENSMUSG00000112980	D430020J02Rik	319545	lncRNA	27.64794032	-0.064162828	0.236667772	0.271109273	0.78630699	0.893161222	0.064162828
ENSMUSG00000042608	Stk40	74178	protein_coding	544.4584298	0.064197434	0.092029766	-0.697572503	0.485444582	0.695380179	0.064197434
ENSMUSG00000049553	Polr1a	20019	protein_coding	380.420794	-0.064239978	0.104453018	0.615013133	0.538546047	0.733377497	0.064239978
ENSMUSG00000046826	Fam187b	76415	protein_coding	37.93587334	-0.064274354	0.230007324	0.279444815	0.779903483	0.890125936	0.064274354
ENSMUSG00000020790	Ankfy1	11736	protein_coding	1574.870498	0.064281168	0.052269365	-1.229805792	0.218769839	0.424931207	0.064281168
ENSMUSG00000021264	Yy1	22632	protein_coding	1347.484189	0.064360395	0.065991898	-0.975277226	0.329422764	0.551796561	0.064360395
ENSMUSG00000040043	Rbms2	56516	protein_coding	135.3639371	-0.064362498	0.08728559	0.737378279	0.460892341	0.674995696	0.064362498
ENSMUSG00000035248	Tut7	214290	protein_coding	1332.85998	0.064363657	0.052102156	-1.235335773	0.216705565	0.422162995	0.064363657
ENSMUSG00000031078	Cttn	13043	protein_coding	3702.805296	-0.064378775	0.076537675	0.841138367	0.400270424	0.622315002	0.064378775
ENSMUSG00000056666	Retsat	67442	protein_coding	1239.587024	0.064408483	0.063407628	-1.015784466	0.309732035	0.530813078	0.064408483
ENSMUSG00000051234	Rnf7	19823	protein_coding	458.0844	-0.064408548	0.091865138	0.701120675	0.483227709	0.693738201	0.064408548
ENSMUSG00000012296	Tjap1	74094	protein_coding	187.7393747	0.064433091	0.105154415	-0.612747368	0.540043397	0.734487099	0.064433091
ENSMUSG00000014198	Zfp385c	278304	protein_coding	5.025822988	-0.064456242	0.819835089	0.078620984	0.937334096	0.970113767	0.064456242
ENSMUSG00000026153	Fam135a	68187	protein_coding	892.8485813	0.06449214	0.07138507	-0.903440175	0.366292326	0.588935008	0.06449214
ENSMUSG00000074634	Tmem267	633640	protein_coding	42.82438194	0.064492294	0.374705244	-0.172114736	0.863347328	0.93221641	0.064492294
ENSMUSG00000025950	Idh1	15926	protein_coding	2420.069268	-0.064498939	0.06188387	1.042257677	0.297292229	0.51684218	0.064498939
ENSMUSG00000020459	Mtif2	76784	protein_coding	384.3409984	-0.064514127	0.093780752	0.687925033	0.491499992	0.698491194	0.064514127
ENSMUSG00000095677	Dynlt1f	100040531	protein_coding	66.06774957	0.064519648	0.185465156	-0.347880161	0.727930185	0.859382261	0.064519648
ENSMUSG00000026842	Abl1	11350	protein_coding	1188.428583	-0.064527082	0.080753712	0.799060255	0.424255474	0.643978573	0.064527082
ENSMUSG00000020564	Atxn7l1	380753	protein_coding	149.1687275	-0.064553385	0.160386202	0.402487147	0.687325546	0.833389267	0.064553385
ENSMUSG00000008301	Phax	56698	protein_coding	291.375526	-0.064596657	0.101517638	0.636309698	0.524574585	0.723107237	0.064596657
ENSMUSG00000031442	Mcf2l	17207	protein_coding	1125.007219	-0.064597491	0.156164491	0.413650314	0.67913022	0.827951097	0.064597491
ENSMUSG00000021928	Ebpl	68177	protein_coding	166.0458532	-0.0645994	0.134236605	0.481235351	0.630349239	0.79831751	0.0645994
ENSMUSG00000023262	Acy1	109652	protein_coding	470.0688601	0.06468491	0.107966192	-0.599121904	0.549091596	0.740220295	0.06468491
ENSMUSG00000034917	Tjp3	27375	protein_coding	4029.363309	-0.064691696	0.077025824	0.839870226	0.400981153	0.622737045	0.064691696
ENSMUSG00000022354	Ndufb9	66218	protein_coding	2185.99715	-0.064702311	0.088764493	0.728921094	0.466049931	0.679736277	0.064702311

Supplementary Table 1

ensembl_gene_id	external_gene_name	entrezgene_id	gene_biotype	baseMean	log2FoldChange	lfcSE	stat	pvalue	padj	absLog2FC
ENSMUSG00000085741	5430405H02Rik	74487	lncRNA	16.29935689	0.064705838	0.296187511	-0.218462413	0.827068841	0.912738228	0.064705838
ENSMUSG00000028832	Stmn1	16765	protein_coding	432.9105832	-0.064729844	0.18799083	0.344324478	0.730602278	0.861253255	0.064729844
ENSMUSG00000043940	Wdfy3	72145	protein_coding	683.1700445	0.064741398	0.112056766	-0.57775537	0.563429291	0.750868206	0.064741398
ENSMUSG00000091575	2010016I18Rik	69206	lncRNA	98.73580328	0.064744744	0.193579279	-0.33446113	0.738031617	0.866982505	0.064744744
ENSMUSG00000053929	Cyhr1	54151	protein_coding	997.1700655	-0.064764174	0.061676685	1.050059271	0.293690863	0.512903015	0.064764174
ENSMUSG00000074221	Zfp568	243905	protein_coding	262.3764332	-0.064806081	0.073200182	0.885326773	0.375980414	0.598238321	0.064806081
ENSMUSG00000028156	Eif4e	13684	protein_coding	573.5437982	0.064827018	0.110225275	-0.588132065	0.556443652	0.745748293	0.064827018
ENSMUSG00000027109	Sp3	20687	protein_coding	1627.139229	-0.064858456	0.065157376	0.995412333	0.319535763	0.540878801	0.064858456
ENSMUSG00000022890	Atp5j	11957	protein_coding	1352.576686	-0.064859737	0.062808652	1.032656087	0.301764871	0.522023104	0.064859737
ENSMUSG00000039210	Gpatch2	67769	protein_coding	656.0482092	0.064898587	0.100288592	-0.647118336	0.517555359	0.717985391	0.064898587
ENSMUSG00000026377	Nifk	67949	protein_coding	368.531189	-0.064900897	0.121599477	0.533726782	0.593530577	0.772689314	0.064900897
ENSMUSG00000061024	Rrs1	59014	protein_coding	209.6325132	0.064911675	0.126581888	-0.512803819	0.608088558	0.782450869	0.064911675
ENSMUSG00000026159	Agfg1	15463	protein_coding	604.5679391	0.064929785	0.067153622	-0.966884331	0.333601861	0.555969435	0.064929785
ENSMUSG00000074627	Mroh8	629499	protein_coding	13.04389898	0.064963963	0.36320559	-0.178862785	0.858045445	0.930446023	0.064963963
ENSMUSG00000041623	D11Wsu47e	276852	protein_coding	116.3223731	-0.064995143	0.122861776	0.529010286	0.596798315	0.774805611	0.064995143
ENSMUSG00000044447	Dock5	68813	protein_coding	1143.419835	-0.064996433	0.095349098	0.681668048	0.495448876	0.701521284	0.064996433
ENSMUSG00000038518	Jarid2	16468	protein_coding	794.9696561	-0.065017631	0.055931553	1.162449945	0.245052746	0.456749554	0.065017631
ENSMUSG00000005054	Cstb	13014	protein_coding	475.1805444	-0.065092338	0.082416556	0.789796869	0.429646408	0.648455238	0.065092338
ENSMUSG00000071265	1700086L19Rik	74284	transcribed_unitary_pseudogene	2.622014148	0.065134586	0.704414428	-0.092466286	0.926327576	1	0.065134586
ENSMUSG00000031601	Cnot7	18983	protein_coding	461.6119699	0.065161182	0.078734064	-0.827611062	0.407890801	0.628584238	0.065161182
ENSMUSG00000045103	Dmd	13405	protein_coding	263.0792773	-0.065163213	0.100057186	0.651259704	0.514878856	0.716272317	0.065163213
ENSMUSG00000027304	Rtf1	76246	protein_coding	344.1611472	-0.065313944	0.073540356	0.888137459	0.374466815	0.596502845	0.065313944
ENSMUSG00000018765	Fxr2	23879	protein_coding	625.6009825	0.065347375	0.06148656	-1.062791198	0.287876652	0.506822498	0.065347375
ENSMUSG00000026073	Il1r2	16178	protein_coding	0.505221152	-0.065361826	1.901156003	0.034380044	0.972574097	1	0.065361826
ENSMUSG00000003235	Eif2b5	224045	protein_coding	717.783089	0.065384126	0.086654322	-0.754539699	0.450525217	0.667322041	0.065384126
ENSMUSG00000084806	Gm15232	627268	lncRNA	1.252824341	-0.06545449	1.545042961	0.042364188	0.966208377	1	0.06545449
ENSMUSG00000067924	Rtl8b	553127	protein_coding	8.425670282	-0.06549989	0.358205532	0.182855608	0.854911315	0.929305257	0.06549989
ENSMUSG00000021763	Cspg4b	408066	protein_coding	3.019044354	-0.065506437	0.762671928	0.085890715	0.931553293	1	0.065506437
ENSMUSG00000000056	Narf	67608	protein_coding	690.9771543	-0.065519835	0.110550248	0.592670177	0.553401904	0.743731716	0.065519835
ENSMUSG00000092074	Dynlt1a	100310872	protein_coding	30.18889292	-0.065543253	0.202897442	0.32303637	0.746667698	0.872440598	0.065543253
ENSMUSG00000038331	Satb2	212712	protein_coding	2427.171748	0.065549824	0.110389917	-0.593802638	0.552644124	0.743189338	0.065549824
ENSMUSG00000021470	Ercc6l2	76251	protein_coding	331.6194376	0.065567318	0.119393174	-0.549171412	0.582887821	0.765654588	0.065567318
ENSMUSG00000063145	Bbs5	72569	protein_coding	39.05168485	0.06559827	0.169101588	-0.387922258	0.698073566	0.840048595	0.06559827
ENSMUSG00000024589	Nedd4l	83814	protein_coding	1125.37471	-0.065653972	0.111603802	0.588277198	0.556346248	0.745678268	0.065653972
ENSMUSG00000040385	Ppp1ca	19045	protein_coding	2268.012974	0.065715811	0.063826512	-1.029600534	0.303197564	0.523689253	0.065715811
ENSMUSG00000022982	Sod1	20655	protein_coding	2852.819923	-0.065742618	0.065460475	1.004310126	0.315229155	0.536227262	0.065742618
ENSMUSG00000031479	Vps36	70160	protein_coding	490.2114026	-0.065750776	0.073222388	0.897960007	0.369206871	0.591834816	0.065750776
ENSMUSG00000002416	Ndufb2	68198	protein_coding	407.5491078	-0.065755677	0.128293075	0.512542688	0.608271253	0.782624968	0.065755677
ENSMUSG00000005804	Bloc1s6	18457	protein_coding	317.9509451	-0.065791159	0.082754527	0.795015823	0.426604298	0.64620651	0.065791159

ensembl_gene_id	external_gene_name	entrezgene_id	gene_biotype	baseMean	log2FoldChange	lfcSE	stat	pvalue	padj	absLog2FC
ENSMUSG00000070780	Rbm47	245945	protein_coding	3598.285803	0.065825882	0.074060568	-0.888811463	0.374104414	0.59609806	0.065825882
ENSMUSG00000016528	Mapkapk2	17164	protein_coding	1086.866666	0.065832568	0.066006537	-0.997364368	0.31858768	0.540015186	0.065832568
ENSMUSG00000055202	Zfp811	240063	protein_coding	2.081172811	-0.065857838	1.23753518	0.053216942	0.957559057	1	0.065857838
ENSMUSG00000032739	Pram1	378460	protein_coding	1.200625976	-0.065863351	0.892744545	0.073776257	0.94118842	1	0.065863351
ENSMUSG00000051671	Coa6	67892	protein_coding	150.0339445	-0.065866341	0.092596005	0.711330276	0.476879594	0.68845088	0.065866341
ENSMUSG00000023495	Pcbp4	59092	protein_coding	36.27696522	0.0658705	0.213561508	-0.30843807	0.757749017	0.87863969	0.0658705
ENSMUSG00000055003	Lrtm2	211187	protein_coding	2.204320806	0.065894449	1.028103417	-0.064093211	0.948896008	1	0.065894449
ENSMUSG00000025410	Dctn2	69654	protein_coding	1478.18125	-0.065901413	0.04471788	1.473715042	0.140558331	0.314808425	0.065901413
ENSMUSG00000044795	Cyb5d1	327951	protein_coding	74.55850501	-0.065959185	0.137668166	0.47911719	0.631855261	0.799444652	0.065959185
ENSMUSG00000022773	Ypel1	106369	protein_coding	13.48139131	-0.065974512	0.338457407	0.194927074	0.845450038	0.923885782	0.065974512
ENSMUSG00000049327	Kmt5a	67956	protein_coding	1741.01081	0.065986779	0.050648112	-1.302847757	0.192626743	0.39045514	0.065986779
ENSMUSG00000025020	Slit1	20562	protein_coding	5.771477316	0.065987236	1.080253747	-0.061084941	0.951291562	0.975912221	0.065987236
ENSMUSG00000027375	Mal	17153	protein_coding	599.9101955	0.065998262	0.220458775	-0.299367816	0.764659416	0.882132403	0.065998262
ENSMUSG00000039929	Urb1	207932	protein_coding	185.8752693	0.066002756	0.137488338	-0.480060757	0.631184191	0.799065123	0.066002756
ENSMUSG00000039783	Kmo	98256	protein_coding	3.576975636	-0.066013614	0.798309523	0.082691753	0.934096643	1	0.066013614
ENSMUSG00000028645	Slc2a1	20525	protein_coding	1231.166972	-0.066049192	0.120140826	0.549764756	0.582480735	0.765241595	0.066049192
ENSMUSG00000024212	Mllt1	64144	protein_coding	621.8322326	0.066053869	0.075197643	-0.87840346	0.379724807	0.601975182	0.066053869
ENSMUSG00000025766	D3Ertd751e	73852	protein_coding	109.685901	-0.066081347	0.16545382	0.39939451	0.689602538	0.834370416	0.066081347
ENSMUSG00000031839	Hsbp1	68196	protein_coding	763.9332763	-0.066107773	0.074060734	0.892615684	0.372063017	0.594336299	0.066107773
ENSMUSG00000041241	Mul1	68350	protein_coding	877.6765487	0.066108239	0.060045998	-1.100959962	0.270914083	0.486449404	0.066108239
ENSMUSG00000066042	Med18	67219	protein_coding	75.30198101	-0.066206484	0.118452877	0.558926774	0.576211696	0.761183901	0.066206484
ENSMUSG00000062627	Mysm1	320713	protein_coding	383.0374956	-0.066255862	0.070089864	0.94529877	0.344506366	0.566265254	0.066255862
ENSMUSG00000027509	Rae1	66679	protein_coding	396.5610274	0.06628489	0.07497612	-0.884080023	0.376653016	0.598887725	0.06628489
ENSMUSG00000020530	Ggnbp2	217039	protein_coding	1182.873977	-0.066313716	0.05059649	1.310638666	0.189979869	0.387311023	0.066313716
ENSMUSG00000037075	Rnf139	75841	protein_coding	298.5705382	-0.066386984	0.09691329	0.685014247	0.493334931	0.699749347	0.066386984
ENSMUSG00000006335	Tfpt	69714	protein_coding	120.609035	-0.066398877	0.098435625	0.674541121	0.499967352	0.704656555	0.066398877
ENSMUSG00000028163	Nfkb1	18033	protein_coding	998.2156696	-0.066425969	0.046266041	1.435739212	0.151076563	0.331061353	0.066425969
ENSMUSG00000036111	Lmo1	109594	protein_coding	6.754120882	0.06646184	0.566305038	-0.117360496	0.906574387	0.954311305	0.06646184
ENSMUSG00000059540	Tcea2	21400	protein_coding	17.99091363	-0.066462149	0.249655536	0.266215403	0.790073321	0.895242955	0.066462149
ENSMUSG00000032745	Gpbp1	73274	protein_coding	774.9942926	0.066484137	0.063389779	-1.048814772	0.294263377	0.513414201	0.066484137
ENSMUSG00000089998	Phtf1os	100125931	lncRNA	2.906648543	-0.066503815	0.786730839	0.084531852	0.932633579	1	0.066503815
ENSMUSG00000018417	Myo1b	17912	protein_coding	824.973714	-0.066512338	0.103601837	0.641999605	0.52087344	0.720614094	0.066512338
ENSMUSG00000001105	Ift20	55978	protein_coding	376.2927683	0.066517273	0.085782121	-0.775421177	0.438090826	0.6563215	0.066517273
ENSMUSG00000026126	Ptpn18	19253	protein_coding	538.1380855	-0.066524021	0.070868497	0.93869665	0.347886514	0.569827206	0.066524021
ENSMUSG00000019804	Snx3	54198	protein_coding	924.6592829	0.066530456	0.065985697	-1.008255706	0.313331721	0.534596554	0.066530456
ENSMUSG00000029366	Dck	13178	protein_coding	135.9592643	0.066582173	0.147612386	-0.451060882	0.65194567	0.811137708	0.066582173
ENSMUSG00000033752	Mnd1	76915	protein_coding	16.63056592	-0.066603199	0.264659418	0.251656258	0.80130677	0.901569434	0.066603199
ENSMUSG00000020610	Amz2	13929	protein_coding	363.8499462	0.066603924	0.111333663	-0.598237062	0.549681765	0.740653122	0.066603924
ENSMUSG00000040651	Tasor	218850	protein_coding	550.6147054	-0.066610822	0.08185961	0.813720236	0.415805238	0.636091497	0.066610822

Supplementary Table 1

ensembl_gene_id	external_gene_name	entrezgene_id	gene_biotype	baseMean	log2FoldChange	lfcSE	stat	pvalue	padj	absLog2FC
ENSMUSG00000026767	Mindy3	66960	protein_coding	388.0117471	0.066611907	0.066160641	-1.006820756	0.314020918	0.5351691	0.066611907
ENSMUSG00000045690	Wdr89	72338	protein_coding	23.33271388	0.066628091	0.319439052	-0.208578415	0.834777362	0.917066002	0.066628091
ENSMUSG00000073144	4930599N23Rik	75379	lncRNA	7.478447814	0.066664559	0.446582103	-0.149277274	0.881334847	0.941481575	0.066664559
ENSMUSG00000086992	Gm15941	100503538	lncRNA	7.550658364	-0.066689411	0.407051275	0.163835407	0.869860717	0.935658236	0.066689411
ENSMUSG00000043061	Tmem18	211986	protein_coding	275.2259515	0.066754635	0.100792401	-0.662298297	0.507780068	0.711603273	0.066754635
ENSMUSG00000028868	Wasf2	242687	protein_coding	1230.209667	0.066778902	0.067974683	-0.982408437	0.325898671	0.54788666	0.066778902
ENSMUSG00000012777	Acp4	100503991	protein_coding	5.926359103	-0.066789642	0.533047597	0.125297708	0.900287867	0.95096289	0.066789642
ENSMUSG00000039354	Smarcal1	54380	protein_coding	247.8665457	0.066822456	0.095305776	-0.701137526	0.483217193	0.693738201	0.066822456
ENSMUSG00000032352	Lrrc1	214345	protein_coding	844.621941	0.066839376	0.073231382	-0.91271493	0.36139249	0.583801091	0.066839376
ENSMUSG00000033365	Ipo13	230673	protein_coding	204.6843821	0.066844711	0.09003501	-0.742430203	0.457826726	0.67261003	0.066844711
ENSMUSG00000008958	Vps72	21427	protein_coding	383.898735	-0.066879209	0.067264867	0.994266575	0.320093104	0.541378279	0.066879209
ENSMUSG00000002250	Ppard	19015	protein_coding	1365.93894	0.066900315	0.12712109	-0.52627235	0.598698991	0.776186935	0.066900315
ENSMUSG00000004530	Coro1c	23790	protein_coding	1913.291188	-0.066905209	0.083080411	0.805306666	0.420642736	0.640643193	0.066905209
ENSMUSG00000026626	Ppp2r5a	226849	protein_coding	1437.589895	-0.066938276	0.054914744	1.218949073	0.222863522	0.429893421	0.066938276
ENSMUSG00000031537	Ikbkb	16150	protein_coding	587.1183688	0.066949937	0.063416483	-1.055718223	0.291096969	0.510000436	0.066949937
ENSMUSG00000020868	Xylt2	217119	protein_coding	213.1859992	-0.066956935	0.116557127	0.574455947	0.565659303	0.752299084	0.066956935
ENSMUSG00000037514	Pank2	74450	protein_coding	522.2485661	0.066969393	0.064743204	-1.034384904	0.300956259	0.521072726	0.066969393
ENSMUSG00000047259	Mc4r	17202	protein_coding	0.931758449	0.066970233	1.715218282	-0.039044729	0.968854727	1	0.066970233
ENSMUSG00000022635	Zcrb1	67197	protein_coding	266.9532603	0.066985873	0.080003681	-0.837284897	0.402432446	0.624001556	0.066985873
ENSMUSG00000001844	Zdhhc4	72881	protein_coding	213.5326459	-0.066994524	0.084312405	0.794598661	0.426846997	0.646395058	0.066994524
ENSMUSG00000057666	Gapdh	14433	protein_coding	7129.863873	0.066996259	0.141545687	-0.473318971	0.635985629	0.801613541	0.066996259
ENSMUSG00000026112	Coa5	76178	protein_coding	1311.186608	-0.067040696	0.049391862	1.357322699	0.174678698	0.365932262	0.067040696
ENSMUSG00000035759	Bbs10	71769	protein_coding	24.73942644	-0.06705613	0.245916897	0.27267801	0.78510074	0.892732467	0.06705613
ENSMUSG00000052676	Zmat1	215693	protein_coding	14.26073181	0.067100169	0.297847318	-0.225283778	0.821758519	0.91001573	0.067100169
ENSMUSG00000001472	Tcf25	66855	protein_coding	2536.591484	0.067110102	0.033281592	-2.016433021	0.043754715	0.134681337	0.067110102
ENSMUSG00000005373	Mlxipl	58805	protein_coding	149.3616619	0.067136597	0.200205886	-0.335337778	0.737370299	0.866747322	0.067136597
ENSMUSG00000053353	2310001K24Rik	69517	lncRNA	0.910721045	0.067146517	1.554618792	-0.043191628	0.965548778	1	0.067146517
ENSMUSG00000020593	Lpin1	14245	protein_coding	126.3869156	0.067164075	0.188243928	-0.356792784	0.721246939	0.855640813	0.067164075
ENSMUSG00000028955	Vamp3	22319	protein_coding	1112.859941	-0.067212896	0.089622573	0.749954996	0.45328181	0.669122175	0.067212896
ENSMUSG00000000339	Rtca	66368	protein_coding	422.5162335	0.06727258	0.083656357	-0.804153837	0.421308135	0.641124452	0.06727258
ENSMUSG00000065431	Mir186	387181	miRNA	1.164450947	-0.067290061	0.905639556	0.07430115	0.940770762	1	0.067290061
ENSMUSG00000023972	Ptk7	71461	protein_coding	277.5398839	0.06734848	0.109608623	-0.614445088	0.538921249	0.733576122	0.06734848
ENSMUSG00000044288	Cnr1	12801	protein_coding	1.128127169	0.067350004	1.657543407	-0.040632422	0.967588936	1	0.067350004
ENSMUSG00000019295	Tmem129	68366	protein_coding	300.5066476	0.067361007	0.089151863	-0.755575985	0.449903459	0.666700082	0.067361007
ENSMUSG00000054455	Vapb	56491	protein_coding	951.9244188	0.067370178	0.068677333	-0.980966718	0.326609154	0.548411142	0.067370178
ENSMUSG00000019461	Plscr3	70310	protein_coding	118.0493701	-0.067403721	0.12459686	0.540974477	0.588525175	0.769577776	0.067403721
ENSMUSG00000016624	Phf21b	271305	protein_coding	94.70426459	0.067469343	0.148398237	-0.454650572	0.649360646	0.809667817	0.067469343
ENSMUSG00000008435	Rdh13	108841	protein_coding	465.862737	-0.067474218	0.10039085	0.672115224	0.501510345	0.706297653	0.067474218
ENSMUSG00000032598	Nckipsd	80987	protein_coding	84.76812467	0.067495331	0.127060109	-0.531207882	0.595274732	0.773679122	0.067495331

ensembl_gene_id	external_gene_name	entrezgene_id	gene_biotype	baseMean	log2FoldChange	lfcSE	stat	pvalue	padj	absLog2FC
ENSMUSG00000022453	Naga	17939	protein_coding	1572.105303	0.067500875	0.05746042	-1.174736895	0.240099994	0.450443439	0.067500875
ENSMUSG00000039356	Exosc2	227715	protein_coding	201.7249048	0.067566499	0.1285344	-0.525668607	0.599118479	0.776435029	0.067566499
ENSMUSG00000035967	Ints6l	236790	protein_coding	289.9311262	-0.06757028	0.09137235	0.739504679	0.459600599	0.673998628	0.06757028
ENSMUSG00000038212	Mfsd14b	66631	protein_coding	1095.09053	-0.067581404	0.055596892	1.21556081	0.224152271	0.431277012	0.067581404
ENSMUSG00000028455	Stoml2	66592	protein_coding	506.2256191	-0.067688858	0.085201507	0.794456111	0.426929949	0.646395058	0.067688858
ENSMUSG00000036093	Arl5a	75423	protein_coding	650.6679401	0.06772752	0.083680983	-0.809353777	0.418311681	0.638329247	0.06772752
ENSMUSG00000020448	Rnf185	193670	protein_coding	361.6879186	0.067780642	0.063358456	-1.069796289	0.284711013	0.503072723	0.067780642
ENSMUSG00000043153	Crppa	75847	protein_coding	29.91910271	0.067845313	0.230138096	-0.294802617	0.768144667	0.883975099	0.067845313
ENSMUSG00000027366	Sppl2a	66552	protein_coding	3568.611491	-0.067880437	0.112005501	0.606045561	0.544484499	0.736796542	0.067880437
ENSMUSG00000024050	Wiz	22404	protein_coding	262.7710879	-0.067892128	0.091517324	0.741850021	0.45817821	0.672744283	0.067892128
ENSMUSG00000020075	Ddx21	56200	protein_coding	999.6313137	-0.067901715	0.099099409	0.685187896	0.493225361	0.699692776	0.067901715
ENSMUSG00000044791	Setd2	235626	protein_coding	1121.412052	-0.067910878	0.067433448	1.007080015	0.313896324	0.535062335	0.067910878
ENSMUSG00000042178	Armc5	233912	protein_coding	309.5404149	0.067916445	0.122195863	-0.555799872	0.578347673	0.761994054	0.067916445
ENSMUSG00000030204	Ddx47	67755	protein_coding	471.8831773	-0.067958032	0.064741156	1.049688266	0.293861461	0.513092425	0.067958032
ENSMUSG00000005413	Hmox1	15368	protein_coding	47.40455991	0.068022881	0.225382879	-0.301810331	0.762796655	0.881056435	0.068022881
ENSMUSG00000026426	Arl8a	68724	protein_coding	130.4229553	-0.06804499	0.183272519	0.371277652	0.710430741	0.848979902	0.06804499
ENSMUSG00000030236	Slco1b2	28253	protein_coding	1.953765687	0.068046352	1.280825031	-0.053126969	0.957630743	1	0.068046352
ENSMUSG00000002767	Mrpl2	27398	protein_coding	550.2904757	-0.068054784	0.078883475	0.862725485	0.388288417	0.610859762	0.068054784
ENSMUSG00000051278	Zgrf1	71643	protein_coding	55.05004615	0.068065409	0.238787815	-0.285045572	0.775609228	0.88791795	0.068065409
ENSMUSG00000032434	Cmtm6	67213	protein_coding	1657.734761	-0.068092167	0.103319374	0.659045488	0.509866559	0.713191289	0.068092167
ENSMUSG00000027007	Itprid2	70599	protein_coding	1907.392445	0.068112348	0.150211365	-0.453443373	0.650229511	0.809917796	0.068112348
ENSMUSG00000061086	Myl4	17896	protein_coding	3.145448905	-0.068132319	0.879306284	0.077484171	0.938238383	1	0.068132319
ENSMUSG00000018326	Ywhab	54401	protein_coding	2206.723001	-0.068137776	0.082038784	0.830555651	0.406224697	0.627187233	0.068137776
ENSMUSG00000117679	Apbb3	225372	protein_coding	150.0323109	0.068153186	0.153639559	-0.44359139	0.657338032	0.814655179	0.068153186
ENSMUSG00000025898	Cwf19l2	244672	protein_coding	253.5435558	-0.068214014	0.079882952	0.853924544	0.393146799	0.615862857	0.068214014
ENSMUSG00000051232	Tmem199	195040	protein_coding	123.2651491	0.068236255	0.1249422	-0.546142579	0.584967933	0.767158627	0.068236255
ENSMUSG00000054426	A930005H10Rik	68161	lncRNA	17.20344683	-0.068259187	0.271527737	0.251389373	0.801513083	0.901709044	0.068259187
ENSMUSG00000037318	Traf3ip3	215243	protein_coding	9.926112178	0.068284394	0.55092734	-0.123944464	0.901359247	0.951341404	0.068284394
ENSMUSG00000030682	Cdipt	52858	protein_coding	807.8695068	0.068298517	0.065265886	-1.046465788	0.295346033	0.514433539	0.068298517
ENSMUSG00000042992	Borcs5	67774	protein_coding	105.3241392	0.068344066	0.123107895	-0.555155829	0.578788079	0.762380457	0.068344066
ENSMUSG00000016181	Utp25	215193	protein_coding	85.07091324	-0.068364721	0.150575263	0.454023589	0.649811849	0.809917796	0.068364721
ENSMUSG00000078144	Capns2	69543	protein_coding	3.315073958	-0.06836493	0.533827334	0.128065622	0.898097041	1	0.06836493
ENSMUSG00000050390	C77080	97130	protein_coding	2537.273468	-0.068366682	0.135404491	0.504907048	0.613624124	0.786448489	0.068366682
ENSMUSG00000003546	Klc4	74764	protein_coding	2099.753362	-0.068373152	0.104775956	0.65256529	0.514036569	0.715750413	0.068373152
ENSMUSG00000045140	Pigw	70325	protein_coding	89.41539518	0.06839503	0.127901283	-0.534748589	0.592823718	0.772538316	0.06839503
ENSMUSG00000048833	Slc39a9	328133	protein_coding	1829.84863	0.068443509	0.071461275	-0.957770602	0.338178446	0.559860817	0.068443509
ENSMUSG00000009630	Ppp2cb	19053	protein_coding	925.6465058	-0.068450587	0.127247403	0.53793308	0.59062325	0.771092762	0.068450587
ENSMUSG00000042828	Trim72	434246	protein_coding	83.68233834	0.068452154	0.193604526	-0.353566912	0.723663475	0.856795141	0.068452154
ENSMUSG00000075284	Wipf1	215280	protein_coding	162.2899364	-0.068455338	0.124108893	0.551574801	0.581239705	0.764339772	0.068455338

ensembl_gene_id	external_gene_name	entrezgene_id	gene_biotype	baseMean	log2FoldChange	lfcSE	stat	pvalue	padj	absLog2FC
ENSMUSG00000035868	Zfp983	73229	protein_coding	80.50408371	-0.068461467	0.130259507	0.525577511	0.599181786	0.776435029	0.068461467
ENSMUSG00000031833	Mast3	546071	protein_coding	216.9694872	0.068471712	0.118871915	-0.576012526	0.564606715	0.751588072	0.068471712
ENSMUSG00000054556	Gm4876	232599	lncRNA	47.3221993	0.068494697	0.229718583	-0.298167856	0.765575054	0.882510888	0.068494697
ENSMUSG00000022883	Robo1	19876	protein_coding	20.81640115	0.068526001	0.336117124	-0.203875364	0.838450897	0.919582966	0.068526001
ENSMUSG00000046229	Scand1	19018	protein_coding	264.5705353	0.068526147	0.152280564	-0.449999296	0.652710948	0.811601896	0.068526147
ENSMUSG00000031691	Tnpo2	212999	protein_coding	973.9095529	0.068527928	0.050750055	-1.350302541	0.176918958	0.369037513	0.068527928
ENSMUSG00000045160	Bola3	78653	protein_coding	274.375824	-0.068540804	0.112688387	0.608233071	0.543032904	0.736259883	0.068540804
ENSMUSG00000027282	Mtch2	56428	protein_coding	2721.88699	-0.068549875	0.079007824	0.867634004	0.385594725	0.607820554	0.068549875
ENSMUSG00000060794	Tssk5	73542	protein_coding	1.364849377	0.068603908	1.475939478	-0.046481518	0.962926464	1	0.068603908
ENSMUSG00000021065	Fut8	53618	protein_coding	1425.17357	0.06865002	0.175898959	-0.390280988	0.69632878	0.838747423	0.06865002
ENSMUSG00000021428	Riok1	71340	protein_coding	215.3313685	0.068686854	0.0912393	-0.752820925	0.451557531	0.668047177	0.068686854
ENSMUSG00000030189	Ybx3	56449	protein_coding	2172.674916	-0.068691146	0.099979102	0.687055038	0.492048046	0.698788598	0.068691146
ENSMUSG00000025496	Drd4	13491	protein_coding	2.658134025	-0.068691369	1.21735635	0.056426673	0.955001909	1	0.068691369
ENSMUSG00000016984	Etaa1	68145	protein_coding	127.1998353	0.06869181	0.161080531	-0.426443902	0.669784417	0.822722373	0.06869181
ENSMUSG00000031029	Eif3f	66085	protein_coding	2033.531463	-0.068725517	0.086882155	0.791019941	0.428932355	0.647921517	0.068725517
ENSMUSG00000053898	Ech1	51798	protein_coding	2546.545203	-0.068751519	0.066917944	1.027400344	0.30423199	0.524504007	0.068751519
ENSMUSG00000007570	Fance	72775	protein_coding	139.899566	-0.068752066	0.102972701	0.667672744	0.504342512	0.708596728	0.068752066
ENSMUSG00000039678	Tbc1d13	70296	protein_coding	1223.827048	-0.068770352	0.078629515	0.874612444	0.381784814	0.604102573	0.068770352
ENSMUSG00000021840	Mapk1ip1l	218975	protein_coding	1007.570325	0.068780928	0.059430356	-1.157336622	0.247134857	0.459127576	0.068780928
ENSMUSG00000051977	Prdm9	213389	protein_coding	74.66931531	-0.068787541	0.147272295	0.467077265	0.640444599	0.803690806	0.068787541
ENSMUSG00000024241	Sos1	20662	protein_coding	399.0003076	0.068817842	0.087886208	-0.783033472	0.433607457	0.652196761	0.068817842
ENSMUSG00000051721	Wdcp	238037	protein_coding	37.56614496	-0.068823127	0.175268716	0.392672056	0.694561712	0.8379481	0.068823127
ENSMUSG00000087691	Cd55os	102640368	lncRNA	3.290089576	0.068866993	0.710284648	-0.096956894	0.922760627	1	0.068866993
ENSMUSG00000032621	Srek1	218543	protein_coding	487.4957516	0.0689099	0.086933389	-0.792674731	0.427967356	0.647057325	0.0689099
ENSMUSG00000101268	2010310C07Rik	72131	lncRNA	2.874707482	-0.068911277	1.333757476	0.051667022	0.958794014	1	0.068911277
ENSMUSG00000040302	Rbm48	269623	protein_coding	61.32442785	-0.06900029	0.149757923	0.460745507	0.644981202	0.806666129	0.06900029
ENSMUSG00000070319	Eif3g	53356	protein_coding	900.2936309	0.069040236	0.086782451	-0.795555267	0.426290576	0.646027582	0.069040236
ENSMUSG00000031715	Smarca5	93762	protein_coding	1363.658735	0.069046768	0.118934173	-0.580546078	0.561546423	0.749969885	0.069046768
ENSMUSG00000032338	Hcn4	330953	protein_coding	5.899782784	0.069050852	0.759528523	-0.090912783	0.927561893	0.965445424	0.069050852
ENSMUSG00000017615	Tnfaip1	21927	protein_coding	1422.299688	0.069120306	0.075074502	-0.9206895	0.35721256	0.580211292	0.069120306
ENSMUSG00000018012	Rac3	170758	protein_coding	58.65399913	0.06918545	0.136591096	-0.506515081	0.612495105	0.785550184	0.06918545
ENSMUSG00000038582	Pptc7	320717	protein_coding	677.6166279	-0.069223161	0.100616631	0.687989261	0.491459544	0.698491194	0.069223161
ENSMUSG00000024098	Twsg1	65960	protein_coding	459.4848628	-0.069225681	0.124799692	0.554694323	0.57910376	0.762564348	0.069225681
ENSMUSG00000020077	Srgn	19073	protein_coding	16.41908765	0.069264202	0.538578256	-0.128605642	0.897669702	0.949716026	0.069264202
ENSMUSG00000031955	Bcar1	12927	protein_coding	827.6346153	-0.069264578	0.072449148	0.956044071	0.33904997	0.560532006	0.069264578
ENSMUSG00000021759	Plpp1	19012	protein_coding	188.2263753	0.069268624	0.258528322	-0.267934374	0.788749834	0.894265208	0.069268624
ENSMUSG00000002249	Tead3	21678	protein_coding	243.3977576	0.069282088	0.132065246	-0.524604995	0.599857813	0.7765798	0.069282088
ENSMUSG00000032540	Abhd5	67469	protein_coding	351.1418453	-0.069288783	0.082999759	0.83480704	0.403826361	0.625062193	0.069288783
ENSMUSG00000101970	Chaserr	69170	lncRNA	81.33333299	-0.069303635	0.140368822	0.493725276	0.621500212	0.792236609	0.069303635

Supplementary Table 1

ensembl_gene_id	external_gene_name	entrezgene_id	gene_biotype	baseMean	log2FoldChange	lfcSE	stat	pvalue	padj	absLog2FC
ENSMUSG00000032381	Ciao2a	68250	protein_coding	806.7451656	-0.069311136	0.087880742	0.788695391	0.430290062	0.648871201	0.069311136
ENSMUSG00000084910	C630043F03Rik	68285	lncRNA	23.65459368	0.069355483	0.206373945	-0.33606705	0.736820305	0.86640918	0.069355483
ENSMUSG00000031835	Mbtps1	56453	protein_coding	1675.173016	0.069411262	0.086111121	-0.806066176	0.420204693	0.640153165	0.069411262
ENSMUSG00000042215	Bag2	213539	protein_coding	31.98523335	-0.069413042	0.18841	0.368414851	0.712563926	0.850598485	0.069413042
ENSMUSG00000026456	Cyb5r1	72017	protein_coding	127.2714532	-0.069445679	0.13516018	0.513802798	0.607389871	0.781981914	0.069445679
ENSMUSG00000037531	Mrpl47	74600	protein_coding	245.5110236	0.069455857	0.084118474	-0.825690882	0.408979466	0.629556944	0.069455857
ENSMUSG00000011306	Sugp1	70616	protein_coding	250.2585933	0.069502463	0.081454206	-0.853270395	0.393509374	0.616138599	0.069502463
ENSMUSG00000041671	Pyroxd1	232491	protein_coding	221.7219353	-0.069504034	0.124376884	0.558817943	0.576285976	0.761221133	0.069504034
ENSMUSG00000049422	Chchd10	103172	protein_coding	2433.51141	0.069525336	0.110035107	-0.631846859	0.527486951	0.725788994	0.069525336
ENSMUSG00000066324	Bpnt2	242291	protein_coding	2087.365767	0.069535496	0.117617853	-0.591198479	0.554387442	0.744656458	0.069535496
ENSMUSG00000056752	Dnah9	237806	protein_coding	17.07850251	-0.069538975	0.43519727	0.15978725	0.873048668	0.937420397	0.069538975
ENSMUSG00000005506	Celf1	13046	protein_coding	1549.059469	0.069565635	0.069945508	-0.994569019	0.319945922	0.541253166	0.069565635
ENSMUSG00000020974	Pole2	18974	protein_coding	47.45773765	-0.069567315	0.259631548	0.267946307	0.788740649	0.894265208	0.069567315
ENSMUSG00000037315	Jade3	382207	protein_coding	132.3340814	-0.069580816	0.115644125	0.601680511	0.547386825	0.738947603	0.069580816
ENSMUSG00000050821	Fam131a	78408	protein_coding	5.486416865	-0.069591608	0.704780641	0.098742224	0.921342943	0.962004133	0.069591608
ENSMUSG00000047613	A430005L14Rik	97159	protein_coding	229.0303708	0.06959224	0.092616949	-0.751398536	0.452412844	0.668738427	0.06959224
ENSMUSG00000046985	Tapt1	231225	protein_coding	456.2937628	0.069603202	0.077122917	-0.902497014	0.366792907	0.589522317	0.069603202
ENSMUSG00000058709	Egln2	112406	protein_coding	922.570554	0.069607827	0.051390905	-1.354477525	0.175584079	0.367456023	0.069607827
ENSMUSG00000026577	Blzf1	66352	protein_coding	234.0888566	0.069626746	0.094049652	-0.74031902	0.459106443	0.673388737	0.069626746
ENSMUSG00000030232	Aebp2	11569	protein_coding	573.4951451	0.069649593	0.051956169	-1.340545199	0.18006816	0.373667454	0.069649593
ENSMUSG00000048429	Timm29	69773	protein_coding	324.6561147	0.06967999	0.087780063	-0.793802001	0.427310708	0.646801927	0.06967999
ENSMUSG00000031231	Cox7b	66142	protein_coding	4210.71067	-0.069681968	0.072157633	0.965690888	0.33419888	0.556515106	0.069681968
ENSMUSG00000022771	Ppil2	66053	protein_coding	801.0564228	-0.06968577	0.043357446	1.607238821	0.108001989	0.261594876	0.06968577
ENSMUSG00000042390	Gatad2b	229542	protein_coding	323.736406	-0.069692455	0.112236164	0.620944732	0.534635988	0.730517043	0.069692455
ENSMUSG00000084957	Bbip1	100503572	protein_coding	240.9502092	0.069721217	0.087901356	-0.793175676	0.427675477	0.647057325	0.069721217
ENSMUSG00000020850	Prpf8	192159	protein_coding	2879.78303	0.069759897	0.090332641	-0.772255702	0.439962996	0.657874003	0.069759897
ENSMUSG00000037503	Fam168b	214469	protein_coding	1374.744895	-0.069818167	0.083485003	0.836295912	0.402988453	0.624327666	0.069818167
ENSMUSG00000024926	Kat5	81601	protein_coding	322.0692931	-0.069826651	0.080445283	0.868001811	0.385393341	0.607636589	0.069826651
ENSMUSG00000021555	Naa35	78689	protein_coding	673.4273177	0.06984099	0.079338215	-0.880294441	0.378699823	0.601173216	0.06984099
ENSMUSG00000022952	Runx1	12394	protein_coding	157.0660506	-0.069900459	0.143463359	0.487235621	0.626091365	0.795381772	0.069900459
ENSMUSG00000038756	Ttll6	237930	protein_coding	365.3338347	-0.069980892	0.123985407	0.564428457	0.572462576	0.757928885	0.069980892
ENSMUSG00000029447	Cct6a	12466	protein_coding	1606.695493	0.070032574	0.086218638	-0.812267228	0.416638311	0.636776036	0.070032574
ENSMUSG00000078161	Erich3	209601	protein_coding	5.577721169	0.070079559	0.876488548	-0.079954905	0.936273121	0.969802608	0.070079559
ENSMUSG00000027327	1700037H04Rik	67326	protein_coding	203.744219	-0.070095555	0.10259845	0.683202868	0.494478661	0.700667401	0.070095555
ENSMUSG00000024664	Fads3	60527	protein_coding	323.8414458	0.070109933	0.084769162	-0.827068849	0.408198039	0.628822987	0.070109933
ENSMUSG00000055024	Ep300	328572	protein_coding	789.296829	-0.070133057	0.143532235	0.488622341	0.625109093	0.794593769	0.070133057
ENSMUSG00000002814	Top3a	21975	protein_coding	138.0332421	0.070157103	0.100246581	-0.699845343	0.484023894	0.694394109	0.070157103
ENSMUSG00000029148	Nrbp1	192292	protein_coding	906.6638531	-0.070174334	0.063658342	1.102358804	0.270305714	0.485621036	0.070174334
ENSMUSG00000024913	Lrp5	16973	protein_coding	981.8029703	-0.070180007	0.077039177	0.910965172	0.362313725	0.584758488	0.070180007

ensembl_gene_id	external_gene_name	entrezgene_id	gene_biotype	baseMean	log2FoldChange	lfcSE	stat	pvalue	padj	absLog2FC
ENSMUSG00000025878	Uimc1	20184	protein_coding	243.6989128	-0.07020706	0.092855007	0.756093427	0.449593183	0.666359883	0.07020706
ENSMUSG00000034403	Pja1	18744	protein_coding	759.8271443	-0.070252011	0.07698962	0.912486785	0.361512523	0.583920353	0.070252011
ENSMUSG00000025958	Creb1	12912	protein_coding	646.6146078	-0.070267291	0.104046204	0.675346993	0.499455334	0.704201299	0.070267291
ENSMUSG00000019795	Pcmt1	18537	protein_coding	457.274046	-0.070283542	0.057472271	1.222912219	0.221362853	0.428054585	0.070283542
ENSMUSG00000027845	Dclre1b	140917	protein_coding	119.6461029	0.070291785	0.100450395	-0.699766134	0.484073368	0.69440476	0.070291785
ENSMUSG00000059278	Naa38	78304	protein_coding	227.8996355	0.07031446	0.10740591	-0.65466099	0.512686046	0.714894653	0.07031446
ENSMUSG00000021770	Samd8	67630	protein_coding	475.6266307	0.070339615	0.109493849	-0.642407003	0.520608954	0.72058807	0.070339615
ENSMUSG00000106847	Peg13	353342	lncRNA	128.9803648	-0.070344017	0.121984846	0.576661933	0.564167853	0.751296404	0.070344017
ENSMUSG00000002280	Ciao3	67563	protein_coding	349.5834463	-0.070355689	0.062669145	1.122652772	0.261584993	0.475244062	0.070355689
ENSMUSG00000069227	Gprin1	26913	protein_coding	13.39858102	-0.070361882	0.430684974	0.163372039	0.870225517	0.935910399	0.070361882
ENSMUSG00000039166	Akap7	432442	protein_coding	540.7886955	0.070367977	0.089312051	-0.787888934	0.430761674	0.649228647	0.070367977
ENSMUSG00000022009	Nufip1	27275	protein_coding	141.7216202	-0.070385819	0.124384205	0.565874255	0.571479264	0.756991142	0.070385819
ENSMUSG00000032571	Pik3r4	75669	protein_coding	430.1078844	-0.07038589	0.084222608	0.835712541	0.403316638	0.624624492	0.07038589
ENSMUSG00000001632	Brpf1	78783	protein_coding	440.5951091	-0.070445577	0.095106006	0.740705871	0.458871799	0.673388737	0.070445577
ENSMUSG00000004936	Map2k1	26395	protein_coding	2706.144762	0.070452077	0.091210599	-0.772411078	0.439870995	0.65785452	0.070452077
ENSMUSG00000055733	Nap1l3	54561	protein_coding	8.645070279	-0.070470554	0.414177089	0.170145948	0.864895359	0.93291195	0.070470554
ENSMUSG00000013160	Atp6v0d1	11972	protein_coding	1085.36966	0.070551755	0.089598408	-0.787421978	0.431034885	0.649494967	0.070551755
ENSMUSG00000056124	B4galt6	56386	protein_coding	302.622827	-0.070572776	0.092020074	0.766928059	0.44312429	0.660786267	0.070572776
ENSMUSG00000034292	Traf3ip1	74019	protein_coding	118.4607734	0.070594894	0.104157336	-0.677771692	0.497916462	0.703146268	0.070594894
ENSMUSG00000067274	Rplp0	11837	protein_coding	16339.61871	-0.070613456	0.113472793	0.622294156	0.533748464	0.73008549	0.070613456
ENSMUSG00000028952	Zbtb48	100090	protein_coding	108.641675	0.070734755	0.11371726	-0.622023031	0.533926724	0.730224807	0.070734755
ENSMUSG00000025983	Ccdc150	78016	protein_coding	2.727865709	-0.070766411	1.087211555	0.065089826	0.948102481	1	0.070766411
ENSMUSG00000024528	Srfbp1	67222	protein_coding	127.6191079	-0.070781633	0.102664006	0.689449359	0.490540531	0.69793377	0.070781633
ENSMUSG00000047417	Rexo1	66932	protein_coding	504.6263062	0.070810711	0.086505587	-0.81856807	0.413032891	0.633140737	0.070810711
ENSMUSG00000046157	Tmem229b	268567	protein_coding	25.24685237	-0.070842518	0.215717657	0.328403891	0.742606301	0.870032583	0.070842518
ENSMUSG00000020308	Tpgs1	110012	protein_coding	187.8513955	-0.070851502	0.139802514	0.506797052	0.612297225	0.785464687	0.070851502
ENSMUSG00000022887	Masp1	17174	protein_coding	4.283993468	-0.070917537	1.794405229	0.039521472	0.968474634	1	0.070917537
ENSMUSG00000030545	Pex11a	18631	protein_coding	187.3347374	-0.071034757	0.089618166	0.792637923	0.427988807	0.647057325	0.071034757
ENSMUSG00000024122	Pdpk1	18607	protein_coding	1315.899722	0.071083007	0.062055281	-1.145478782	0.25201088	0.464375257	0.071083007
ENSMUSG00000039218	Srrm2	75956	protein_coding	3452.68944	0.071088608	0.164365187	-0.432504047	0.665375112	0.819511083	0.071088608
ENSMUSG00000007610	Gtpbp3	70359	protein_coding	241.2286097	-0.071167132	0.095542128	0.744876984	0.45634609	0.671470886	0.071167132
ENSMUSG00000022663	Atg3	67841	protein_coding	373.4466004	0.071222665	0.089808822	-0.79304754	0.427750126	0.647057325	0.071222665
ENSMUSG00000021486	Prelid1	66494	protein_coding	2287.833299	-0.071228562	0.077084423	0.92403314	0.355469062	0.578346413	0.071228562
ENSMUSG00000032249	Anp32a	11737	protein_coding	1112.254822	0.071245486	0.078083968	-0.912421443	0.361546906	0.583920353	0.071245486
ENSMUSG00000033799	Tasor2	105203	protein_coding	422.3488757	-0.07127605	0.090978161	0.783441319	0.433368	0.652026162	0.07127605
ENSMUSG00000000562	Adora3	11542	protein_coding	2.184868721	0.071293424	1.050683368	-0.067854337	0.945901588	1	0.071293424
ENSMUSG00000027314	Dll4	54485	protein_coding	124.2143631	0.071298255	0.125172592	-0.569599573	0.568949318	0.754850891	0.071298255
ENSMUSG00000034371	Tkfc	225913	protein_coding	167.739711	-0.071339385	0.102407997	0.696619282	0.486041086	0.695595302	0.071339385
ENSMUSG00000021023	Prorp	66132	protein_coding	185.5788494	-0.071354896	0.093922027	0.759724825	0.447419086	0.664688628	0.071354896

ensembl_gene_id	external_gene_name	entrezgene_id	gene_biotype	baseMean	log2FoldChange	lfcSE	stat	pvalue	padj	absLog2FC
ENSMUSG00000026617	Bpnt1	23827	protein_coding	1624.291107	-0.071365493	0.084470758	0.844854417	0.398192128	0.62019321	0.071365493
ENSMUSG00000069793	Slfn9	237886	protein_coding	371.9677368	0.071370511	0.206051704	-0.346371854	0.729063275	0.860227988	0.071370511
ENSMUSG00000024146	Cript	56724	protein_coding	422.4762476	-0.071379634	0.095368281	0.748463042	0.454180909	0.669812138	0.071379634
ENSMUSG00000046603	Tcaim	382117	protein_coding	121.7839963	0.071400061	0.118617617	-0.601934709	0.547217599	0.738779548	0.071400061
ENSMUSG00000056824	Zfp663	381405	protein_coding	1.18598668	-0.071450831	1.352754965	0.052818753	0.957876319	1	0.071450831
ENSMUSG00000097402	Gm17399	100502695	lncRNA	1.429694201	-0.071480803	0.855858668	0.0835194	0.933438552	1	0.071480803
ENSMUSG00000022443	Myh9	17886	protein_coding	13088.56029	0.07150555	0.065032734	-1.099531661	0.271536232	0.487195712	0.07150555
ENSMUSG00000020170	Frs2	327826	protein_coding	374.043006	0.071624042	0.088230124	-0.811786711	0.416914029	0.636961631	0.071624042
ENSMUSG00000081534	Slc48a1	67739	protein_coding	277.6326829	-0.071655155	0.104190545	0.687731836	0.491621668	0.698491194	0.071655155
ENSMUSG00000034789	Rab24	19336	protein_coding	123.1350826	0.071680861	0.112242868	-0.638622854	0.52306831	0.722040793	0.071680861
ENSMUSG00000068328	Aup1	11993	protein_coding	971.3266859	0.071704537	0.063087887	-1.136581683	0.255713201	0.468471767	0.071704537
ENSMUSG00000053483	Usp21	30941	protein_coding	218.6872546	-0.07170548	0.089698561	0.799405019	0.424055601	0.643940615	0.07170548
ENSMUSG00000039116	Adgrg6	215798	protein_coding	29.54077578	0.07170879	0.237133363	-0.30239857	0.762348245	0.88093126	0.07170879
ENSMUSG00000047434	Xxylt1	268880	protein_coding	161.8929539	0.07171652	0.120586714	-0.594729865	0.552024055	0.742718368	0.07171652
ENSMUSG00000026029	Casp8	12370	protein_coding	1302.936966	0.071736327	0.074064023	-0.968571838	0.33275886	0.555180826	0.071736327
ENSMUSG00000016087	Fli1	14247	protein_coding	8.295508489	-0.071818975	0.639544796	0.11229702	0.910587904	0.956641558	0.071818975
ENSMUSG00000074158	Zfp976	208111	protein_coding	63.19180948	-0.071823046	0.174871666	0.410718602	0.681278885	0.829503357	0.071823046
ENSMUSG00000028634	Hivep3	16656	protein_coding	80.14312156	-0.071841607	0.163305137	0.439922519	0.659993226	0.816323585	0.071841607
ENSMUSG00000074445	Sprr2a3	100042514	protein_coding	1819.801065	-0.071866267	0.266745472	0.269418881	0.787607358	0.893615254	0.071866267
ENSMUSG00000010097	Nxf1	53319	protein_coding	770.0948704	0.071928293	0.061889385	-1.162207272	0.245151281	0.456778547	0.071928293
ENSMUSG00000012889	Podnl1	244550	protein_coding	6.654473095	0.071997531	0.522545926	-0.137782207	0.890412547	0.946160016	0.071997531
ENSMUSG00000021495	Fam193b	212483	protein_coding	408.8402297	0.071998913	0.168173398	-0.428123079	0.668561515	0.822256556	0.071998913
ENSMUSG00000022556	Hsf1	15499	protein_coding	287.1582588	-0.07204896	0.08839347	0.81509369	0.415018683	0.635300198	0.07204896
ENSMUSG00000039427	Alg1	208211	protein_coding	144.7167449	-0.072087646	0.109005137	0.661323384	0.508404948	0.712111524	0.072087646
ENSMUSG00000026202	Tuba4a	22145	protein_coding	1531.051937	0.072095135	0.104638857	-0.68899008	0.49082951	0.698213329	0.072095135
ENSMUSG00000024925	Rnaseh2c	68209	protein_coding	191.683545	0.072099401	0.122474925	-0.588687041	0.556071233	0.745551704	0.072099401
ENSMUSG00000036315	Polr1h	66136	protein_coding	207.8424377	-0.072102807	0.090127785	0.800006429	0.423707072	0.643604486	0.072102807
ENSMUSG00000020647	Ncoa1	17977	protein_coding	707.3254314	-0.072103373	0.080932511	0.890907408	0.372978846	0.595257447	0.072103373
ENSMUSG00000020802	Ube2o	217342	protein_coding	252.419927	0.072132168	0.096690617	-0.746010002	0.455661373	0.671017649	0.072132168
ENSMUSG00000047518	Slfnl1	194219	protein_coding	2.394458992	-0.072150781	0.877661584	0.082207975	0.934481332	1	0.072150781
ENSMUSG00000003206	Ebi3	50498	protein_coding	1.907913679	-0.072183023	0.988410496	0.073029397	0.941782724	1	0.072183023
ENSMUSG00000019998	Stx7	53331	protein_coding	1048.852036	-0.072191555	0.06744786	1.070331291	0.284470214	0.502862462	0.072191555
ENSMUSG00000096768	Gm47283	170942	lncRNA	9.53692367	-0.072199419	0.902027698	0.080041244	0.936204454	0.96979553	0.072199419
ENSMUSG00000031516	Dctn6	22428	protein_coding	462.682918	-0.072203482	0.07276957	0.992220803	0.321089826	0.542231046	0.072203482
ENSMUSG00000055538	Zcchc24	71918	protein_coding	7.561834759	-0.072203619	0.50221133	0.143771386	0.885680999	0.943256547	0.072203619
ENSMUSG00000053641	Dennd4a	102442	protein_coding	152.5043923	-0.072204126	0.118291397	0.610392033	0.541602144	0.735467298	0.072204126
ENSMUSG00000031931	Ankrd49	56503	protein_coding	222.0559217	-0.072231568	0.095155962	0.759086106	0.44780105	0.664930223	0.072231568
ENSMUSG00000027324	Rpusd2	271842	protein_coding	57.63500509	0.072330901	0.148555137	-0.486895992	0.62633204	0.795381772	0.072330901
ENSMUSG00000027665	Pik3ca	18706	protein_coding	774.5748466	-0.072370836	0.097095344	0.745358455	0.456055052	0.671357918	0.072370836

ensembl_gene_id	external_gene_name	entrezgene_id	gene_biotype	baseMean	log2FoldChange	lfcSE	stat	pvalue	padj	absLog2FC
ENSMUSG00000036270	Edc4	234699	protein_coding	549.6862616	0.072372396	0.052743611	-1.372154752	0.170015268	0.359584034	0.072372396
ENSMUSG00000035561	Aldh1b1	72535	protein_coding	3834.574957	0.072417252	0.120478592	-0.601079833	0.547786815	0.739162904	0.072417252
ENSMUSG00000044916	1700029I15Rik	75641	protein_coding	10.81515042	0.072462769	0.458749334	-0.157957219	0.87449051	0.938406345	0.072462769
ENSMUSG00000026893	Gca	227960	protein_coding	148.4738171	-0.072479971	0.102365637	0.708049824	0.478914317	0.690541222	0.072479971
ENSMUSG00000091381	Vmn2r83	625029	protein_coding	0.667830234	-0.072485024	1.679189123	0.043166683	0.965568664	1	0.072485024
ENSMUSG00000037279	Ovol2	107586	protein_coding	205.0987239	-0.072502309	0.145155808	0.499479212	0.617441828	0.789258793	0.072502309
ENSMUSG00000041133	Smc1a	24061	protein_coding	1171.974711	0.07251915	0.106586295	-0.680379688	0.496264079	0.702459427	0.07251915
ENSMUSG00000029146	Snx17	266781	protein_coding	675.0808095	0.072538072	0.066690103	-1.087688708	0.276732555	0.493649227	0.072538072
ENSMUSG00000024193	Phf1	21652	protein_coding	142.8040974	0.072618682	0.16521337	-0.439544827	0.660266809	0.816494106	0.072618682
ENSMUSG00000029478	Ncor2	20602	protein_coding	331.1085108	0.072623916	0.235822093	-0.307960612	0.758112304	0.878750865	0.072623916
ENSMUSG00000019235	Rps6kl1	238323	protein_coding	22.05753863	0.072649886	0.232653508	-0.312266452	0.754838033	0.876856731	0.072649886
ENSMUSG00000037708	Spag6	381350	protein_coding	1.305640353	-0.072655037	1.181960973	0.061469912	0.950984976	1	0.072655037
ENSMUSG00000038178	Slc43a2	215113	protein_coding	154.6380287	0.072687287	0.190101289	-0.382360831	0.702193739	0.842918326	0.072687287
ENSMUSG00000053226	Dand5	23863	protein_coding	27.28363634	-0.072692751	0.238553042	0.304723637	0.760576646	0.879809661	0.072692751
ENSMUSG00000001674	Ddx18	66942	protein_coding	166.5279987	0.072702461	0.1185812	-0.613102756	0.539808398	0.734239015	0.072702461
ENSMUSG00000024871	Doc2g	60425	protein_coding	12.55454676	0.07272643	0.342386643	-0.212410242	0.831786996	0.915204382	0.07272643
ENSMUSG00000004096	Cwc15	66070	protein_coding	669.1558435	0.072731516	0.066240533	-1.0979911	0.272208376	0.488030519	0.072731516
ENSMUSG00000060032	H2aj	232440	protein_coding	744.9012931	-0.072734076	0.085220784	0.853478138	0.393394206	0.616016681	0.072734076
ENSMUSG00000056394	Lig1	16881	protein_coding	317.0370031	0.072735172	0.148202009	-0.490783981	0.623579241	0.793440482	0.072735172
ENSMUSG00000052997	Uba2	50995	protein_coding	906.9369156	0.07273834	0.078853587	-0.92244808	0.356294901	0.579347577	0.07273834
ENSMUSG00000029250	Polr2b	231329	protein_coding	867.9206337	0.072742602	0.11955229	-0.608458456	0.542883452	0.736235501	0.072742602
ENSMUSG00000022536	Glyr1	74022	protein_coding	1798.09112	0.072748963	0.054940601	-1.324138471	0.185457061	0.380783029	0.072748963
ENSMUSG00000033737	Fndc3c1	333564	protein_coding	2.254231156	0.072761562	2.748693799	-0.026471323	0.978881406	1	0.072761562
ENSMUSG00000033208	S100b	20203	protein_coding	3.48302778	-0.072840015	0.593663424	0.12269581	0.902347982	1	0.072840015
ENSMUSG00000033871	Ppargc1b	170826	protein_coding	697.4297624	-0.072841621	0.130423116	0.558502382	0.576501378	0.761222618	0.072841621
ENSMUSG00000078908	Mon1b	270096	protein_coding	456.5177364	-0.072863931	0.09206727	0.79142057	0.42869861	0.647656102	0.072863931
ENSMUSG00000059482	Cfap418	67157	protein_coding	47.49555018	0.072865906	0.14506139	-0.502310825	0.615448896	0.787780401	0.072865906
ENSMUSG00000059981	Taok2	381921	protein_coding	795.949316	0.072873564	0.077951967	-0.93485214	0.349864505	0.571706158	0.072873564
ENSMUSG00000033769	Exoc6b	75914	protein_coding	579.7788482	0.072901256	0.093950737	-0.775951931	0.437777368	0.656109195	0.072901256
ENSMUSG00000057897	Camk2b	12323	protein_coding	377.720462	-0.072901605	0.11898565	0.612692412	0.540079741	0.734487099	0.072901605
ENSMUSG00000092335	Zfp977	637776	protein_coding	3.441032652	-0.072930991	0.68483825	0.106493747	0.915190616	1	0.072930991
ENSMUSG00000039069	Mtg2	52856	protein_coding	166.8661459	-0.072937883	0.09465817	0.770539753	0.440979784	0.658856432	0.072937883
ENSMUSG00000063802	Hspbp1	66245	protein_coding	263.7071864	0.072962005	0.08925995	-0.817410335	0.413693972	0.633801118	0.072962005
ENSMUSG00000030970	Ctbp2	13017	protein_coding	912.5815397	0.072968638	0.054308083	-1.343605466	0.179076001	0.37212295	0.072968638
ENSMUSG00000005354	Txn2	56551	protein_coding	1103.372475	-0.073004039	0.062959632	1.159537258	0.246237259	0.458233276	0.073004039
ENSMUSG00000023852	Chd1	12648	protein_coding	950.2037711	0.073092502	0.064089598	-1.140473719	0.254088996	0.466691767	0.073092502
ENSMUSG00000022400	Rbx1	56438	protein_coding	541.3485084	-0.07312623	0.101271862	0.722078456	0.470246243	0.68365582	0.07312623
ENSMUSG00000027667	Zfp639	67778	protein_coding	156.4773481	-0.073156674	0.092564518	0.790331709	0.429334073	0.648260298	0.073156674
ENSMUSG00000011960	Ccnt1	12455	protein_coding	519.1692111	-0.073157468	0.081214846	0.90078935	0.367700331	0.590110273	0.073157468

ensembl_gene_id	external_gene_name	entrezgene_id	gene_biotype	baseMean	log2FoldChange	lfcSE	stat	pvalue	padj	absLog2FC
ENSMUSG00000032959	Pebp1	23980	protein_coding	922.1133449	0.073173395	0.124167724	-0.58931091	0.555652727	0.74529314	0.073173395
ENSMUSG00000045237	Eola1	68618	protein_coding	160.3678012	-0.073186747	0.091649879	0.798547115	0.424553064	0.644177805	0.073186747
ENSMUSG00000027706	Sec62	69276	protein_coding	867.512681	0.073245045	0.123064132	-0.595177852	0.551724593	0.742476843	0.073245045
ENSMUSG00000021113	Snapc1	75627	protein_coding	149.2677397	0.073279998	0.099592052	-0.735801664	0.461851409	0.675674536	0.073279998
ENSMUSG00000026353	Ubxn4	67812	protein_coding	984.6877432	0.073323104	0.065265197	-1.123464064	0.261240454	0.47491467	0.073323104
ENSMUSG00000026201	Stk16	20872	protein_coding	673.9597439	0.073332435	0.070718392	-1.036964116	0.299752581	0.519697021	0.073332435
ENSMUSG00000028243	Ubxn2b	68053	protein_coding	97.19988081	0.073341777	0.116476173	-0.62967193	0.528909251	0.726837595	0.073341777
ENSMUSG00000020716	Nf1	18015	protein_coding	718.20267	0.073385652	0.107216209	-0.684464157	0.493682115	0.699975951	0.073385652
ENSMUSG00000003452	Bicd1	12121	protein_coding	211.0520948	-0.073389255	0.110856381	0.662021026	0.507957746	0.711726983	0.073389255
ENSMUSG00000039041	Adrm1	56436	protein_coding	503.8593163	0.073421736	0.100483275	-0.730686137	0.464970885	0.678555072	0.073421736
ENSMUSG00000003119	Cdk12	69131	protein_coding	847.911418	0.073470601	0.074919903	-0.980655319	0.326762744	0.548613253	0.073470601
ENSMUSG00000073016	Uprt	331487	protein_coding	229.5660483	-0.073501791	0.104068154	0.706285137	0.480010834	0.691115487	0.073501791
ENSMUSG00000026526	Fh1	14194	protein_coding	1550.328137	-0.073511809	0.07733135	0.950608128	0.341803341	0.563829035	0.073511809
ENSMUSG00000027099	Mtx2	53375	protein_coding	603.0802267	-0.073532494	0.077546356	0.948239193	0.343007699	0.564917636	0.073532494
ENSMUSG00000021076	Actr10	56444	protein_coding	606.5491003	0.073540235	0.064171578	-1.145993868	0.251797689	0.464320478	0.073540235
ENSMUSG00000024312	Wdr46	57315	protein_coding	270.8799745	0.073558542	0.096084721	-0.765559191	0.443938634	0.661243132	0.073558542
ENSMUSG00000059288	Cdyl	12593	protein_coding	147.3746199	0.073575608	0.095850424	-0.767608578	0.442719765	0.660497294	0.073575608
ENSMUSG00000024078	Ttc27	74196	protein_coding	157.4281687	-0.073578972	0.156418249	0.470398898	0.638070051	0.802764652	0.073578972
ENSMUSG00000047036	Zfp445	235682	protein_coding	943.0709585	-0.073597225	0.103794108	0.709069385	0.47828142	0.68987155	0.073597225
ENSMUSG00000074578	Zfas1	68949	lncRNA	322.2735656	-0.073628468	0.124445015	0.591654623	0.554081888	0.744516542	0.073628468
ENSMUSG00000020986	Sec23a	20334	protein_coding	1312.684775	0.073653502	0.075060895	-0.981249987	0.326469479	0.548343875	0.073653502
ENSMUSG00000094328	Tdpoz9	668359	protein_coding	0.78622542	0.073729475	1.101899862	-0.06691123	0.946652373	1	0.073729475
ENSMUSG00000028833	Ncdn	26562	protein_coding	359.135346	0.073739184	0.061417603	-1.200619695	0.229898758	0.438451101	0.073739184
ENSMUSG00000020536	Llgl1	16897	protein_coding	455.4433636	-0.073768538	0.067523541	1.092486231	0.274619439	0.491020508	0.073768538
ENSMUSG00000022122	Ednrb	13618	protein_coding	0.855237627	0.073768977	1.312510146	-0.0562045	0.955178896	1	0.073768977
ENSMUSG00000029141	Slc4a1ap	20534	protein_coding	233.4924963	0.073842236	0.073952941	-0.998503038	0.318035492	0.539389762	0.073842236
ENSMUSG00000020956	Dtd2	328092	protein_coding	107.5770969	0.073881297	0.107673943	-0.686157623	0.492613717	0.699148013	0.073881297
ENSMUSG00000028369	Svep1	64817	protein_coding	11.06831908	-0.073906932	0.607959563	0.121565539	0.903243107	0.952286247	0.073906932
ENSMUSG00000038335	Tsr1	104662	protein_coding	444.4057602	-0.073911507	0.096009817	0.769832809	0.441399075	0.659125086	0.073911507
ENSMUSG00000039065	Atpsckmt	68073	protein_coding	159.4703557	0.07392467	0.107055601	-0.690525951	0.489863496	0.697627026	0.07392467
ENSMUSG00000018736	Ndel1	83431	protein_coding	726.9430645	0.073934542	0.090640161	-0.815692965	0.414675763	0.634951837	0.073934542
ENSMUSG00000023951	Vegfa	22339	protein_coding	660.1726819	-0.073947707	0.202693124	0.364825927	0.71524135	0.852138782	0.073947707
ENSMUSG00000030271	Ogg1	18294	protein_coding	83.39595566	-0.073956186	0.122482911	0.6038082	0.545971167	0.737699873	0.073956186
ENSMUSG00000073471	Rsph3a	66832	protein_coding	131.9807273	0.073966132	0.112721048	-0.656187401	0.511703555	0.714354347	0.073966132
ENSMUSG00000010609	Psen2	19165	protein_coding	177.4624009	-0.073974393	0.087972917	0.840876887	0.400416908	0.622382905	0.073974393
ENSMUSG00000020706	Ftsj3	56095	protein_coding	450.0591043	-0.074091066	0.101291523	0.731463641	0.464496005	0.677981883	0.074091066
ENSMUSG00000029402	Snrnp35	76167	protein_coding	87.29959937	0.074103994	0.191104733	-0.387766397	0.698188915	0.840126183	0.074103994
ENSMUSG00000024696	Lpxn	107321	protein_coding	13.42599148	-0.074105982	0.273559958	0.270894844	0.786471911	0.89324602	0.074105982
ENSMUSG00000005580	Adcy9	11515	protein_coding	240.8029088	-0.074110301	0.091016296	0.814253102	0.41549997	0.635742288	0.074110301

ensembl_gene_id	external_gene_name	entrezgene_id	gene_biotype	baseMean	log2FoldChange	lfcSE	stat	pvalue	padj	absLog2FC
ENSMUSG00000021901	Bap1	104416	protein_coding	497.6567757	0.074132354	0.055463962	-1.336585981	0.181357818	0.375447286	0.074132354
ENSMUSG00000032288	Imp3	102462	protein_coding	343.3363181	-0.074144673	0.122004658	0.607720017	0.543373185	0.736456016	0.074144673
ENSMUSG00000032053	Pou2af1	18985	protein_coding	2.840142479	-0.074152921	1.113276708	0.066607808	0.94689393	1	0.074152921
ENSMUSG00000041642	Kif21b	16565	protein_coding	738.8685292	0.074173502	0.215389361	-0.344369384	0.73056851	0.861253255	0.074173502
ENSMUSG00000022848	Slc49a4	224132	protein_coding	481.2973534	-0.074214473	0.087141073	0.851658934	0.39440342	0.616875898	0.074214473
ENSMUSG00000074569	Gcnt7	654821	protein_coding	16.73809033	-0.074234953	0.274639521	0.2702996	0.786929773	0.893375436	0.074234953
ENSMUSG00000019066	Rab3d	19340	protein_coding	1976.964593	0.074250145	0.156063107	-0.475770006	0.634238241	0.800627603	0.074250145
ENSMUSG00000040521	Tsfm	66399	protein_coding	261.4789656	-0.074273414	0.080825461	0.918935859	0.358129122	0.580790426	0.074273414
ENSMUSG00000044062	Plekhd1os	68354	lncRNA	12.27190352	-0.074288736	0.298056139	0.249244105	0.803171965	0.902837235	0.074288736
ENSMUSG00000068250	Amn1	232566	protein_coding	178.3719994	0.074306562	0.117853104	-0.630501524	0.528366506	0.726359126	0.074306562
ENSMUSG00000024238	Zeb1	21417	protein_coding	5.488968061	0.074332944	0.801267665	-0.09276918	0.926086936	0.964614821	0.074332944
ENSMUSG00000040389	Wdr47	99512	protein_coding	69.58829461	-0.074333599	0.142226257	0.52264329	0.601222509	0.777615016	0.074333599
ENSMUSG00000031980	Agt	11606	protein_coding	135.3727578	-0.074359722	0.142351925	0.522365411	0.601415934	0.777725809	0.074359722
ENSMUSG00000033557	Fam20b	215015	protein_coding	2414.706563	0.074404107	0.068432184	-1.08726776	0.276918492	0.493794621	0.074404107
ENSMUSG00000051910	Sox6	20679	protein_coding	215.9482222	0.074469665	0.202453536	-0.367835834	0.712995648	0.850752177	0.074469665
ENSMUSG00000032806	Slc10a3	214601	protein_coding	229.2486502	0.074511429	0.114536315	-0.650548506	0.51533798	0.716335759	0.074511429
ENSMUSG00000035171	1110059E24Rik	66206	protein_coding	544.6424862	0.074523929	0.111068212	-0.670974421	0.502236824	0.706950129	0.074523929
ENSMUSG00000029416	Slc15a4	100561	protein_coding	237.798877	0.074533763	0.098538216	-0.756394482	0.449412715	0.666174493	0.074533763
ENSMUSG00000059552	Trp53	22059	protein_coding	286.8677191	-0.074556251	0.089625467	0.831864575	0.405485389	0.62645577	0.074556251
ENSMUSG00000020612	Prkar1a	19084	protein_coding	883.178799	0.074568781	0.071840486	-1.03797713	0.299280703	0.519260509	0.074568781
ENSMUSG00000091945	Vmn2r114	666002	protein_coding	4.288916074	0.07460139	2.23324674	-0.033404903	0.9733517	1	0.07460139
ENSMUSG00000035437	Rabgap1	227800	protein_coding	523.3318927	-0.074629653	0.079079966	0.943723888	0.345310763	0.566901476	0.074629653
ENSMUSG00000047040	Prr15l	217138	protein_coding	3498.917221	0.074636867	0.100953902	-0.739316309	0.459714948	0.674101639	0.074636867
ENSMUSG00000028274	Rngtt	24018	protein_coding	239.3114651	-0.074652007	0.080375623	0.928789153	0.352998367	0.575403714	0.074652007
ENSMUSG00000073460	Pnldc1	240023	protein_coding	3.546418738	0.074702628	0.617607187	-0.120954921	0.903726741	1	0.074702628
ENSMUSG00000022957	Itsn1	16443	protein_coding	467.9399724	0.074732163	0.064761853	-1.153953436	0.24851926	0.460736668	0.074732163
ENSMUSG00000026895	Ndufa8	68375	protein_coding	1110.338218	-0.074736245	0.084261804	0.886952833	0.375104295	0.597345451	0.074736245
ENSMUSG00000069830	Nlrp1a	195046	protein_coding	1.83156864	0.074748784	1.7984264	-0.041563438	0.966846721	1	0.074748784
ENSMUSG00000031981	Capn9	73647	protein_coding	429.6776005	-0.074755613	0.224790723	0.332556488	0.739469093	0.867855348	0.074755613
ENSMUSG00000001098	Kctd10	330171	protein_coding	961.4025273	-0.074801162	0.083412019	0.896767188	0.36984316	0.592567182	0.074801162
ENSMUSG00000046792	Zfp787	67109	protein_coding	310.8505099	-0.074808543	0.064140501	1.166323023	0.243483868	0.454749219	0.074808543
ENSMUSG00000032936	Camkv	235604	protein_coding	1.525313563	-0.0748167	1.714758589	0.04363104	0.965198509	1	0.0748167
ENSMUSG00000032370	Lactb	80907	protein_coding	154.625219	-0.074819452	0.111683524	0.66992381	0.502906361	0.707530724	0.074819452
ENSMUSG00000035964	Tmem59l	67937	protein_coding	1.606433954	-0.07482141	1.267348308	0.059037764	0.952922029	1	0.07482141
ENSMUSG00000015340	Cybb	13058	protein_coding	2.987484453	-0.07484029	1.018826377	0.073457354	0.941442179	1	0.07484029
ENSMUSG00000050312	Nsun3	106338	protein_coding	191.3194327	-0.074851784	0.121862036	0.614233824	0.539060825	0.733662794	0.074851784
ENSMUSG00000019857	Asf1a	66403	protein_coding	106.1963009	0.074855708	0.137717163	-0.543546689	0.586753454	0.768287864	0.074855708
ENSMUSG00000031403	Dkc1	245474	protein_coding	299.2094204	0.074898008	0.137098165	-0.546309341	0.584853316	0.767077092	0.074898008
ENSMUSG00000050188	Lsm10	116748	protein_coding	96.27582219	0.074945543	0.122593339	-0.611334539	0.54097813	0.735049182	0.074945543

Supplementary Table 1

ensembl_gene_id	external_gene_name	entrezgene_id	gene_biotype	baseMean	log2FoldChange	lfcSE	stat	pvalue	padj	absLog2FC
ENSMUSG00000020671	Rab10	19325	protein_coding	1802.655222	0.074947715	0.080021144	-0.936598893	0.348964921	0.570683233	0.074947715
ENSMUSG00000023805	Synj2	20975	protein_coding	597.4651718	-0.075001108	0.085892124	0.873201229	0.382553405	0.604944412	0.075001108
ENSMUSG00000025528	2010106E10Rik	67715	protein_coding	22.51025251	0.075008105	0.413956208	-0.181198164	0.856212035	0.930174321	0.075008105
ENSMUSG00000097080	1700086O06Rik	73516	lncRNA	21.35540086	-0.075022623	0.234965372	0.319292252	0.749504907	0.87416654	0.075022623
ENSMUSG00000072501	Phf20l1	239510	protein_coding	554.8090196	0.075029504	0.063508626	-1.181406498	0.237441278	0.447819239	0.075029504
ENSMUSG00000058979	Hdhd5	214932	protein_coding	202.8695647	-0.075035311	0.093434734	0.803077265	0.421930078	0.64171609	0.075035311
ENSMUSG00000030402	Ppm1n	232941	protein_coding	3.115993931	-0.075039042	0.947357113	0.079208823	0.936866527	1	0.075039042
ENSMUSG00000049643	2310022A10Rik	66367	protein_coding	87.5253253	0.075086548	0.135677993	-0.553417299	0.579977696	0.763289104	0.075086548
ENSMUSG00000022403	St13	70356	protein_coding	1498.212399	-0.075104917	0.094746517	0.792693172	0.427956609	0.647057325	0.075104917
ENSMUSG00000059182	Skap2	54353	protein_coding	668.9908224	-0.075112319	0.127035598	0.591269847	0.55433963	0.744656458	0.075112319
ENSMUSG00000068259	Or9a7	258380	protein_coding	1.482554047	-0.07511817	1.576488152	0.047649054	0.961995937	1	0.07511817
ENSMUSG00000030064	Frmd4b	232288	protein_coding	722.9317977	-0.075125209	0.051399446	1.461595713	0.14385203	0.320287504	0.075125209
ENSMUSG00000020782	Llgl2	217325	protein_coding	2854.052633	-0.075175955	0.073256283	1.026204879	0.304795022	0.524967146	0.075175955
ENSMUSG00000044827	Tlr1	21897	protein_coding	872.7424452	0.075228301	0.122179372	-0.615720152	0.538079235	0.733336674	0.075228301
ENSMUSG00000016757	Ttll12	223723	protein_coding	339.7872756	0.075245009	0.104987486	-0.716704549	0.473556419	0.68565173	0.075245009
ENSMUSG00000034757	Tmub2	72053	protein_coding	534.4270945	0.075294381	0.052453524	-1.435449441	0.151159066	0.33115426	0.075294381
ENSMUSG00000086330	1700007J10Rik	69320	lncRNA	5.386302331	-0.075345912	0.442333	0.170337534	0.864744695	0.932810366	0.075345912
ENSMUSG00000038968	Klk1b16	16615	protein_coding	1.718220811	0.075355347	0.997381444	-0.075553187	0.939774581	1	0.075355347
ENSMUSG00000020549	Elac2	68626	protein_coding	332.9697058	0.075366903	0.121899146	-0.618272606	0.536395656	0.732371878	0.075366903
ENSMUSG00000025228	Actr1a	54130	protein_coding	2086.60869	0.075389501	0.04629529	-1.628448616	0.103429796	0.253681814	0.075389501
ENSMUSG00000079415	Cntf	12803	protein_coding	2.207754389	0.07539298	0.670366616	-0.112465297	0.910454484	1	0.07539298
ENSMUSG00000032570	Atp2c1	235574	protein_coding	969.9261378	-0.075426461	0.119721435	0.630016345	0.52868389	0.726648836	0.075426461
ENSMUSG00000048277	Syngr2	20973	protein_coding	1997.156939	0.075517649	0.095544956	-0.790388652	0.429300827	0.648260298	0.075517649
ENSMUSG00000031755	Bbs2	67378	protein_coding	109.4071676	-0.075553612	0.105458307	0.716431105	0.473725194	0.685755422	0.075553612
ENSMUSG00000028136	Snx27	76742	protein_coding	462.2750787	-0.075605554	0.094688867	0.798462963	0.424601878	0.644177805	0.075605554
ENSMUSG00000045876	Pcdhb8	93879	protein_coding	1.826113024	0.075607683	0.77753065	-0.097240775	0.922535187	1	0.075607683
ENSMUSG00000022827	Rabl3	67657	protein_coding	142.6938389	0.075640297	0.106143812	-0.712620887	0.476080383	0.687930892	0.075640297
ENSMUSG00000021764	Ndufs4	17993	protein_coding	945.1813423	-0.075643591	0.083234125	0.908805025	0.363453054	0.585795796	0.075643591
ENSMUSG00000023911	Flywch2	76917	protein_coding	82.27139462	0.075695223	0.12494354	-0.605835428	0.544624042	0.736796542	0.075695223
ENSMUSG00000067148	Polr1c	20016	protein_coding	519.3510667	-0.075700264	0.065902061	1.148678252	0.250688677	0.46291768	0.075700264
ENSMUSG00000032435	Dync1li1	235661	protein_coding	638.6775373	0.075731252	0.078514944	-0.964545711	0.334772403	0.557076931	0.075731252
ENSMUSG00000027641	Rbl1	19650	protein_coding	403.5683313	-0.075756947	0.095947136	0.789569649	0.429779139	0.64845799	0.075756947
ENSMUSG00000028869	Gnl2	230737	protein_coding	385.1463516	0.075791653	0.090201184	-0.8402512	0.400767557	0.622662097	0.075791653
ENSMUSG00000030866	Ern2	26918	protein_coding	1338.548802	-0.075816135	0.157072316	0.482682989	0.629320844	0.79746811	0.075816135
ENSMUSG00000041660	Bbox1	170442	protein_coding	2.231189267	-0.075817678	0.984697045	0.076995944	0.938626772	1	0.075817678
ENSMUSG00000035357	Pdzrn3	55983	protein_coding	81.0640561	-0.075825856	0.21608784	0.350902928	0.725661175	0.857991597	0.075825856
ENSMUSG00000030527	Crtc3	70461	protein_coding	150.7682416	-0.075838714	0.1062173	0.713995872	0.475229732	0.687150319	0.075838714
ENSMUSG00000024067	Dpy30	66310	protein_coding	416.2133222	-0.075882907	0.064257977	1.180910299	0.23763836	0.447999484	0.075882907
ENSMUSG00000085196	Gm14963	105246847	lncRNA	23.50274471	0.075908531	0.422926604	-0.179483936	0.85755773	0.930374388	0.075908531

ensembl_gene_id	external_gene_name	entrezgene_id	gene_biotype	baseMean	log2FoldChange	lfcSE	stat	pvalue	padj	absLog2FC
ENSMUSG00000038115	Ano2	243634	protein_coding	2.676016662	-0.076002207	3.09466081	0.02455914	0.980406611	1	0.076002207
ENSMUSG00000003581	Rnf215	71673	protein_coding	188.3011977	-0.0760867	0.082703741	0.91999103	0.357577447	0.580632641	0.0760867
ENSMUSG00000032316	Clk3	102414	protein_coding	534.2491955	-0.076103174	0.063678506	1.195115567	0.232041881	0.441111962	0.076103174
ENSMUSG00000039770	Ypel5	383295	protein_coding	458.3992333	-0.076131419	0.104781658	0.726572002	0.467488185	0.681117777	0.076131419
ENSMUSG00000008540	Mgst1	56615	protein_coding	1665.251476	0.076164051	0.09575481	-0.795407049	0.426376761	0.646068054	0.076164051
ENSMUSG00000033938	Ndufb7	66916	protein_coding	1084.944519	-0.076176419	0.080387753	0.947612234	0.343326896	0.56521786	0.076176419
ENSMUSG00000054931	Zkscan4	544922	protein_coding	7.231578324	-0.076189848	0.464695302	0.163956571	0.869765332	0.935658236	0.076189848
ENSMUSG00000031176	Dynlt3	67117	protein_coding	755.2625448	0.076247507	0.123915245	-0.61531983	0.538343524	0.733377497	0.076247507
ENSMUSG00000040687	Madd	228355	protein_coding	526.1434559	-0.076256626	0.067272112	1.133554815	0.256981336	0.470017696	0.076256626
ENSMUSG00000033960	Jcad	240185	protein_coding	6.191713569	0.076270808	0.69467843	-0.10979297	0.912573567	0.957266079	0.076270808
ENSMUSG00000039263	Npepl1	228961	protein_coding	654.3238295	-0.076293007	0.05569474	1.369842244	0.170736152	0.360508479	0.076293007
ENSMUSG00000034226	Rhov	228543	protein_coding	14.61597288	0.076293931	0.332264789	-0.229617862	0.818388726	0.908812719	0.076293931
ENSMUSG00000079658	Eloc	67923	protein_coding	370.6795997	0.076307059	0.060812467	-1.254793018	0.209553908	0.413054814	0.076307059
ENSMUSG00000020594	Pum2	80913	protein_coding	1717.042974	0.076308294	0.091086522	-0.837756154	0.402167668	0.623781054	0.076308294
ENSMUSG00000027499	Pkia	18767	protein_coding	72.04492766	-0.076317538	0.197466639	0.386483198	0.699138844	0.840656682	0.076317538
ENSMUSG00000020059	Sycp3	20962	protein_coding	1.722972562	0.076317688	0.736914289	-0.103563859	0.917515469	1	0.076317688
ENSMUSG00000005615	Pcyt1a	13026	protein_coding	832.5869308	-0.076384551	0.063556164	1.201843311	0.229424241	0.438111017	0.076384551
ENSMUSG00000091415	Ak9	633979	protein_coding	2.528132084	0.076421943	2.134111472	-0.035809724	0.971434079	1	0.076421943
ENSMUSG00000045967	Gpr158	241263	protein_coding	1.756285756	-0.076447155	1.318374878	0.057985901	0.953759859	1	0.076447155
ENSMUSG00000035172	Plekhh3	217198	protein_coding	333.3969353	-0.076501382	0.134258132	0.569808184	0.568807804	0.754784418	0.076501382
ENSMUSG00000028961	Pgd	110208	protein_coding	2121.539938	0.076550089	0.07980779	-0.95918066	0.337467741	0.559438733	0.076550089
ENSMUSG00000020843	Timm22	56322	protein_coding	248.2184145	0.076580416	0.104116241	-0.735528053	0.462017962	0.675841352	0.076580416
ENSMUSG00000032463	Faim	23873	protein_coding	112.380589	0.076665374	0.118219436	-0.648500591	0.516661229	0.717449153	0.076665374
ENSMUSG00000028294	Cfap206	69329	protein_coding	6.221725837	-0.076679789	0.452070062	0.169619259	0.865309576	0.932932188	0.076679789
ENSMUSG00000042549	Map2k3os	24082	lncRNA	16.93426453	-0.07668874	0.272377585	0.28155305	0.778286245	0.889208458	0.07668874
ENSMUSG00000000826	Dnajc5	13002	protein_coding	1623.639493	0.076733365	0.054024003	-1.420356895	0.155503805	0.337876038	0.076733365
ENSMUSG00000037890	Wdr19	213081	protein_coding	96.77594162	0.076792804	0.124871823	-0.61497303	0.538572532	0.733377497	0.076792804
ENSMUSG00000052962	Mrpl35	66223	protein_coding	298.7019004	-0.076840507	0.090221851	0.851684001	0.394389503	0.616875898	0.076840507
ENSMUSG00000040936	Ulk4	209012	protein_coding	30.66172417	0.076849384	0.311083736	-0.247037615	0.804879114	0.90383338	0.076849384
ENSMUSG00000059555	Tor4a	227612	protein_coding	688.1687155	0.076863667	0.088064341	-0.872812604	0.382765228	0.605068399	0.076863667
ENSMUSG00000036533	Cdc42ep3	260409	protein_coding	147.9129777	-0.076884892	0.16773141	0.458381003	0.646678737	0.807443873	0.076884892
ENSMUSG00000042734	Ttc9	69480	protein_coding	3.850690346	-0.076894186	0.978135964	0.078612983	0.937340461	1	0.076894186
ENSMUSG00000003062	Stard3nl	76205	protein_coding	328.7422013	0.076902322	0.068123392	-1.12886808	0.258953488	0.472341459	0.076902322
ENSMUSG00000038644	Pold1	18971	protein_coding	305.6445021	0.076992794	0.158528845	-0.485670564	0.627200757	0.796064353	0.076992794
ENSMUSG00000048170	Mcmbp	210711	protein_coding	902.399064	-0.07708786	0.059851214	1.287991581	0.197748894	0.398004294	0.07708786
ENSMUSG00000003032	Klf4	16600	protein_coding	8123.791838	0.077110614	0.138825982	-0.555447998	0.57858827	0.762250234	0.077110614
ENSMUSG00000027882	Stxbp3	20912	protein_coding	688.1629699	-0.077131955	0.069188564	1.114807858	0.264932768	0.479778455	0.077131955
ENSMUSG00000040345	Arhgap9	216445	protein_coding	64.74730205	0.077139304	0.193357758	-0.398945999	0.689932995	0.834570281	0.077139304
ENSMUSG00000037933	Bicd2	76895	protein_coding	238.8591342	-0.077159192	0.109280709	0.706064162	0.480148237	0.691115487	0.077159192

ensembl_gene_id	external_gene_name	entrezgene_id	gene_biotype	baseMean	log2FoldChange	lfcSE	stat	pvalue	padj	absLog2FC
ENSMUSG00000000346	Dazap2	23994	protein_coding	2093.364858	-0.077178556	0.055960169	1.379169466	0.16784251	0.356391801	0.077178556
ENSMUSG00000053754	Chd8	67772	protein_coding	808.7315227	0.077190685	0.073457435	-1.050821952	0.293340372	0.512561987	0.077190685
ENSMUSG00000044600	Smim7	66818	protein_coding	1180.695233	0.077214431	0.086894077	-0.88860408	0.374215898	0.59621817	0.077214431
ENSMUSG00000004070	Hmox2	15369	protein_coding	303.3825702	0.077232424	0.093404901	-0.826856226	0.408318557	0.628891312	0.077232424
ENSMUSG00000071722	Spin4	270624	protein_coding	12.62398837	0.07723982	0.276737812	-0.279108298	0.780161715	0.890125936	0.07723982
ENSMUSG00000047473	Zfp30	22693	protein_coding	12.00107267	0.077240097	0.399610699	-0.193288361	0.846733141	0.9245093	0.077240097
ENSMUSG00000039253	Fn3krp	238024	protein_coding	154.0156827	-0.077251799	0.118747887	0.650553041	0.515335052	0.716335759	0.077251799
ENSMUSG00000034994	Eef2	13629	protein_coding	30265.42393	-0.077284162	0.073340675	1.053769438	0.291988489	0.510848385	0.077284162
ENSMUSG00000000275	Trim25	217069	protein_coding	1223.768201	-0.077291639	0.123361552	0.626545612	0.530957129	0.728048657	0.077291639
ENSMUSG00000031266	Gla	11605	protein_coding	246.3581704	-0.077324265	0.093915201	0.823341313	0.410313926	0.630553153	0.077324265
ENSMUSG00000030965	Abraxas2	109359	protein_coding	363.7860396	0.077407415	0.080638256	-0.959934141	0.337088362	0.559327232	0.077407415
ENSMUSG00000020783	Ncbp3	66874	protein_coding	457.3041882	-0.077571713	0.053529282	1.449145414	0.147296978	0.325282468	0.077571713
ENSMUSG00000034652	Cd300a	217303	protein_coding	4.59938374	0.077590407	1.340972645	-0.05786129	0.953859118	0.977047797	0.077590407
ENSMUSG00000047067	Dusp28	67446	protein_coding	81.87106786	-0.077617243	0.136471966	0.568741297	0.569531718	0.755320172	0.077617243
ENSMUSG00000020585	Laptm4a	17775	protein_coding	2112.924896	0.077646541	0.091013248	-0.853134493	0.393584725	0.616198156	0.077646541
ENSMUSG00000031400	G6pdx	14381	protein_coding	754.2545782	-0.077682957	0.066399841	1.169926853	0.242030407	0.45280278	0.077682957
ENSMUSG00000003344	Btbd2	208198	protein_coding	415.6233392	-0.07768697	0.079800908	0.973509855	0.330299964	0.552585717	0.07768697
ENSMUSG00000045427	Hnrnph2	56258	protein_coding	626.0231367	-0.077697522	0.124043151	0.626374945	0.53106904	0.728048657	0.077697522
ENSMUSG00000019952	Poc1b	382406	protein_coding	280.7045699	0.077719483	0.089501795	-0.8683567	0.385199091	0.607446313	0.077719483
ENSMUSG00000042638	Gucy2c	14917	protein_coding	5198.336781	0.077724954	0.058307079	-1.333027748	0.182522698	0.37700811	0.077724954
ENSMUSG00000004069	Dnaja3	83945	protein_coding	988.339349	-0.0777377	0.079001527	0.984002495	0.325114286	0.546957754	0.0777377
ENSMUSG00000041161	Otud3	73162	protein_coding	238.7731989	0.077766676	0.089347386	-0.870385578	0.384089726	0.60650772	0.077766676
ENSMUSG00000020682	Mmp28	118453	protein_coding	176.658549	-0.077782535	0.105447715	0.737640781	0.460732767	0.674875015	0.077782535
ENSMUSG00000037455	Slc18b1	76306	protein_coding	321.0670197	-0.077798016	0.094318868	0.824840436	0.409462186	0.630052795	0.077798016
ENSMUSG00000054520	Sh3bp2	24055	protein_coding	83.24620012	-0.07781803	0.143863359	0.540916263	0.588565301	0.769577776	0.07781803
ENSMUSG00000061393	Acvr2b	11481	protein_coding	64.3080288	0.077834957	0.173284649	-0.449173991	0.653306148	0.811872956	0.077834957
ENSMUSG00000031865	Dctn1	13191	protein_coding	1687.350764	0.077893001	0.050354999	-1.546877221	0.121892855	0.284857762	0.077893001
ENSMUSG00000031600	Vps37a	52348	protein_coding	523.665299	-0.077910813	0.062772983	1.241152001	0.214549603	0.419497057	0.077910813
ENSMUSG00000039963	Ccdc40	207607	protein_coding	4.18132282	-0.077912531	0.899826743	0.086586147	0.931000477	1	0.077912531
ENSMUSG00000060512	0610040J01Rik	76261	protein_coding	854.3634461	-0.077925905	0.086008001	0.906030883	0.364919506	0.587471313	0.077925905
ENSMUSG00000024581	Napg	108123	protein_coding	413.4943011	-0.077927059	0.083367365	0.934742979	0.349920772	0.571741528	0.077927059
ENSMUSG00000058761	Rnf169	108937	protein_coding	323.0531693	-0.07792834	0.084892973	0.917959841	0.358639891	0.581328932	0.07792834
ENSMUSG00000051427	Ccdc157	216516	protein_coding	74.36784863	0.077995952	0.14282783	-0.546083717	0.585008392	0.767158627	0.077995952
ENSMUSG00000024829	Mrpl21	353242	protein_coding	409.1335298	-0.078007358	0.0842483	0.925922042	0.354486495	0.577145813	0.078007358
ENSMUSG00000025921	Rdh10	98711	protein_coding	268.4120197	-0.078015998	0.089485849	0.871824969	0.383303871	0.605788125	0.078015998
ENSMUSG00000053333	Dis3l2	208718	protein_coding	247.5716902	0.078027354	0.083869125	-0.930346586	0.352191665	0.574428953	0.078027354
ENSMUSG00000039529	Atp8b1	54670	protein_coding	7307.62984	-0.078110375	0.069406015	1.125412179	0.260414413	0.474068167	0.078110375
ENSMUSG00000022517	Mgrn1	17237	protein_coding	1265.725168	-0.078181093	0.059778295	1.307850831	0.190923909	0.388417088	0.078181093
ENSMUSG00000042082	Arsb	11881	protein_coding	204.3937251	-0.078195841	0.083726079	0.933948439	0.350330491	0.572354345	0.078195841

Supplementary Table 1

ensembl_gene_id	external_gene_name	entrezgene_id	gene_biotype	baseMean	log2FoldChange	lfcSE	stat	pvalue	padj	absLog2FC
ENSMUSG00000024247	Pkdcc	106522	protein_coding	1079.410992	0.078213045	0.078691117	-0.993924706	0.320259525	0.541493376	0.078213045
ENSMUSG00000066442	Mthfs	107885	protein_coding	22.11071805	0.078214456	0.267340863	-0.292564537	0.769855024	0.884532517	0.078214456
ENSMUSG00000009549	Srp14	20813	protein_coding	352.0434316	0.078226981	0.09788356	-0.799184057	0.424183695	0.643957486	0.078226981
ENSMUSG00000037991	Rmi2	223970	protein_coding	37.88534569	0.078238102	0.285523603	-0.274016234	0.784072146	0.891754761	0.078238102
ENSMUSG00000039473	Ubn1	170644	protein_coding	836.7246119	0.078239998	0.069062702	-1.132883532	0.257263166	0.470354227	0.078239998
ENSMUSG00000001305	Rrp15	67223	protein_coding	131.0982258	0.078250199	0.114324102	-0.684459331	0.493685162	0.699975951	0.078250199
ENSMUSG00000026933	Camsap1	227634	protein_coding	263.0049137	0.078264017	0.076469922	-1.023461452	0.306089717	0.52648324	0.078264017
ENSMUSG00000018585	Atox1	11927	protein_coding	619.6648236	0.078272906	0.088170977	-0.887740035	0.374680605	0.596728278	0.078272906
ENSMUSG00000036561	Ppp6r2	71474	protein_coding	459.3198868	0.078301509	0.06756412	-1.158921464	0.246488199	0.458287111	0.078301509
ENSMUSG00000052595	A1cf	69865	protein_coding	301.1905659	0.078314089	0.159974185	-0.489542039	0.624457999	0.794172689	0.078314089
ENSMUSG00000040785	Ttc3	22129	protein_coding	912.9624596	-0.078603092	0.094463425	0.832100806	0.405352047	0.62645534	0.078603092
ENSMUSG00000051747	Ttn	22138	protein_coding	43.33369643	0.07865088	0.864207513	-0.091009253	0.927485238	0.965445424	0.07865088
ENSMUSG00000040699	Limd2	67803	protein_coding	74.85182956	0.078676228	0.169506645	-0.464148339	0.642541471	0.80495599	0.078676228
ENSMUSG00000032562	Gnai2	14678	protein_coding	1692.666622	0.078707682	0.06630682	-1.187022423	0.235218776	0.445131871	0.078707682
ENSMUSG00000050732	Vamp8	22320	protein_coding	1282.993836	-0.078742945	0.062490362	1.260081435	0.207639987	0.410708835	0.078742945
ENSMUSG00000022811	Zfp148	22661	protein_coding	942.3363751	-0.078772486	0.076062061	1.035634387	0.300372743	0.520512296	0.078772486
ENSMUSG00000026427	Eif2d	16865	protein_coding	465.7609502	0.078776554	0.061807289	-1.274551201	0.202468139	0.404473864	0.078776554
ENSMUSG00000024517	Grp	225642	protein_coding	1.066562172	0.078788717	2.458431847	-0.032048363	0.974433482	1	0.078788717
ENSMUSG00000014355	Anapc1	17222	protein_coding	1381.470169	0.078825705	0.093635867	-0.841832377	0.399881786	0.622046705	0.078825705
ENSMUSG00000073982	Rhog	56212	protein_coding	241.8405984	-0.078860687	0.097582762	0.808141572	0.419009088	0.638921152	0.078860687
ENSMUSG00000019823	Mical1	171580	protein_coding	149.5207038	0.078862671	0.160172921	-0.492359575	0.622465172	0.792979237	0.078862671
ENSMUSG00000019988	Nedd1	17997	protein_coding	273.6782321	0.078870676	0.151380375	-0.521009912	0.602359866	0.778438024	0.078870676
ENSMUSG00000004264	Phb2	12034	protein_coding	2014.644426	-0.078879829	0.065412043	1.20589155	0.227859309	0.436477048	0.078879829
ENSMUSG00000002578	Ikzf4	22781	protein_coding	5.910760904	0.078882238	0.906595188	-0.087009328	0.930664097	0.966663747	0.078882238
ENSMUSG00000024767	Otub1	107260	protein_coding	755.2509353	-0.078888043	0.120935437	0.652315356	0.514197756	0.715854134	0.078888043
ENSMUSG00000085355	3010003L21Rik	109163	lncRNA	42.68671819	0.078897661	0.18833362	-0.418924995	0.675270949	0.825971257	0.078897661
ENSMUSG00000005078	Jkamp	104771	protein_coding	149.1062892	-0.078955845	0.089301606	0.884148104	0.376616268	0.598886958	0.078955845
ENSMUSG00000038812	Trmt112	67674	protein_coding	81.48726205	-0.078981785	0.126235284	0.625671223	0.531530612	0.728500002	0.078981785
ENSMUSG00000026104	Stat1	20846	protein_coding	236.2328057	0.079010309	0.14672699	-0.538485176	0.590242137	0.770846581	0.079010309
ENSMUSG00000011254	Thg1l	66628	protein_coding	98.94949089	0.079029534	0.152705222	-0.517530001	0.604786232	0.779964063	0.079029534
ENSMUSG00000037224	Zfyve28	231125	protein_coding	3.076241437	0.079076046	1.821232139	-0.043418983	0.965367546	1	0.079076046
ENSMUSG00000026342	Slc35f5	74150	protein_coding	1367.414973	-0.079076132	0.080810807	0.978534129	0.32781022	0.549974786	0.079076132
ENSMUSG00000028799	Zfp362	230761	protein_coding	68.41644334	0.079111557	0.141740669	-0.558142964	0.576746765	0.761222618	0.079111557
ENSMUSG00000041974	Spidr	224008	protein_coding	147.7990195	0.079129305	0.104887232	-0.754422659	0.45059547	0.667366241	0.079129305
ENSMUSG00000025782	Taf3	209361	protein_coding	84.95994785	-0.079141408	0.13169648	0.600937915	0.547881339	0.739192192	0.079141408
ENSMUSG00000019792	Trmt11	73681	protein_coding	145.6598703	0.079152254	0.103364354	-0.765759667	0.443819317	0.661197851	0.079152254
ENSMUSG00000026017	Carf	241066	protein_coding	82.1507569	-0.07915746	0.136804477	0.578617467	0.562847316	0.75067522	0.07915746
ENSMUSG00000036087	Slain2	75991	protein_coding	921.7263341	0.079164726	0.06869473	-1.152413375	0.249151247	0.461264989	0.079164726
ENSMUSG00000003660	Snrnp200	320632	protein_coding	1666.581472	0.079173056	0.069120484	-1.145435499	0.2520288	0.464375257	0.079173056

ensembl_gene_id	external_gene_name	entrezgene_id	gene_biotype	baseMean	log2FoldChange	lfcSE	stat	pvalue	padj	absLog2FC
ENSMUSG00000052906	Ubxn8	108159	protein_coding	267.9672243	-0.079181088	0.135601027	0.583926907	0.55926949	0.747948226	0.079181088
ENSMUSG00000078453	Abracl	73112	protein_coding	375.1839319	0.079209114	0.114175479	-0.693748909	0.487839688	0.696186289	0.079209114
ENSMUSG00000104011	Gm32391	102634922	lncRNA	14.51212114	0.079212713	0.320481838	-0.247167558	0.804778552	0.903803848	0.079212713
ENSMUSG00000029763	Exoc4	20336	protein_coding	660.4493929	0.079218182	0.109270822	-0.724971046	0.468469793	0.682033868	0.079218182
ENSMUSG00000088108	Snora47	100217450	snoRNA	10.57154881	0.079247055	0.452824516	-0.175006107	0.861074835	0.931590005	0.079247055
ENSMUSG00000032244	Fem1b	14155	protein_coding	735.2895706	-0.079279607	0.097727891	0.811228053	0.417234718	0.637333654	0.079279607
ENSMUSG00000050912	Tmem123	71929	protein_coding	283.885057	0.079299027	0.109811999	-0.722134447	0.470211821	0.68365582	0.079299027
ENSMUSG00000021327	Zkscan3	72739	protein_coding	635.4172376	0.079405655	0.059788539	-1.328108299	0.184142332	0.379181439	0.079405655
ENSMUSG00000034165	Ccnd3	12445	protein_coding	429.1339762	0.079419905	0.121988917	-0.651041969	0.515019395	0.716272317	0.079419905
ENSMUSG00000061718	Ppp1r1b	19049	protein_coding	2857.254842	-0.079435355	0.135632819	0.585664706	0.558100856	0.747120423	0.079435355
ENSMUSG00000029587	Zfp12	231866	protein_coding	191.7825272	-0.079471768	0.108732255	0.73089414	0.464843815	0.678429586	0.079471768
ENSMUSG00000028029	Aimp1	13722	protein_coding	480.0135598	-0.079488468	0.091462446	0.869083121	0.384801668	0.607042377	0.079488468
ENSMUSG00000042631	Xkr7	228787	protein_coding	2.463119853	-0.079515622	1.149787044	0.069156825	0.944864789	1	0.079515622
ENSMUSG00000024457	Trim26	22670	protein_coding	843.1000583	0.079543119	0.067034613	-1.186597721	0.235386335	0.445367775	0.079543119
ENSMUSG00000020973	Dnaaf2	109065	protein_coding	17.8031453	-0.079568161	0.251426906	0.316466372	0.751648546	0.875244964	0.079568161
ENSMUSG00000045410	Akr1e1	56043	protein_coding	431.5421654	-0.07957032	0.074357986	1.070097851	0.284575266	0.50295958	0.07957032
ENSMUSG00000085747	Slc13a2os	790911	lncRNA	75.72440877	-0.079616987	0.203905226	0.39046075	0.696195874	0.838743187	0.079616987
ENSMUSG00000056069	Otulinl	223433	protein_coding	15.97967558	0.079633405	0.32607748	-0.244216207	0.807063376	0.904987729	0.079633405
ENSMUSG00000048924	Ccdc125	76041	protein_coding	85.19516631	-0.079648607	0.155283899	0.512922506	0.60800553	0.782404996	0.079648607
ENSMUSG00000014232	Cluap1	76779	protein_coding	81.38410672	0.079669217	0.134735409	-0.591301262	0.554318584	0.744656458	0.079669217
ENSMUSG00000038740	Mvb12b	72543	protein_coding	77.69568181	0.079687035	0.233872101	-0.340729121	0.733307513	0.863385162	0.079687035
ENSMUSG00000042709	Atpaf2	246782	protein_coding	304.7610393	-0.079737567	0.094960884	0.839688551	0.401083035	0.622742125	0.079737567
ENSMUSG00000097073	9430037G07Rik	320692	lncRNA	11.39573895	0.079780151	0.357719607	-0.22302426	0.823516625	0.910399889	0.079780151
ENSMUSG00000027239	Mdk	17242	protein_coding	6.360402348	0.079792592	0.550993975	-0.144815725	0.884856366	0.943016089	0.079792592
ENSMUSG00000029577	Ube3b	117146	protein_coding	1554.807736	0.079867721	0.0611487	-1.30612295	0.19151075	0.389196224	0.079867721
ENSMUSG00000020664	Dld	13382	protein_coding	1632.108468	-0.079869824	0.078185829	1.021538368	0.306999441	0.527501415	0.079869824
ENSMUSG00000039239	Tgfb2	21808	protein_coding	3.507660226	0.07988608	1.154052173	-0.069222243	0.944812718	1	0.07988608
ENSMUSG00000020623	Map2k6	26399	protein_coding	237.632531	0.079906725	0.115407839	-0.692385595	0.48869521	0.696497169	0.079906725
ENSMUSG00000038206	Fbxo8	50753	protein_coding	389.2928006	0.079922805	0.073641596	-1.085294303	0.277791328	0.494763373	0.079922805
ENSMUSG00000028796	Phc2	54383	protein_coding	571.8248052	-0.079952463	0.053234155	1.501901603	0.133122523	0.302752759	0.079952463
ENSMUSG00000035545	Leng8	232798	protein_coding	918.0719852	0.079985909	0.189371225	-0.422376254	0.672750407	0.824536161	0.079985909
ENSMUSG00000004661	Arid3b	56380	protein_coding	63.07989816	-0.079988366	0.177824879	0.449815383	0.652843565	0.811643351	0.079988366
ENSMUSG00000039483	Asb6	72323	protein_coding	200.7363104	0.080011394	0.092710182	-0.863027049	0.388122595	0.610657064	0.080011394
ENSMUSG00000016619	Nup50	18141	protein_coding	828.3949253	0.080014252	0.073267504	-1.092083765	0.274796285	0.491230336	0.080014252
ENSMUSG00000104945	Gm35715	102639389	lncRNA	6.656568959	-0.080035356	0.484543821	0.165176714	0.868804893	0.935174293	0.080035356
ENSMUSG00000054939	Zfp174	385674	protein_coding	24.99359651	-0.0800538	0.223395249	0.358350505	0.720081027	0.855153117	0.0800538
ENSMUSG00000028878	Fam76a	230789	protein_coding	267.5034866	-0.080060024	0.078060019	1.025621374	0.305070089	0.525167043	0.080060024
ENSMUSG00000021594	Srd5a1	78925	protein_coding	104.0644324	0.080097728	0.152036147	-0.526833452	0.59830925	0.776035105	0.080097728
ENSMUSG00000004902	Slc25a18	71803	protein_coding	8.854901957	-0.080145895	0.387024411	0.207082274	0.835945606	0.917689672	0.080145895

ensembl_gene_id	external_gene_name	entrezgene_id	gene_biotype	baseMean	log2FoldChange	lfcSE	stat	pvalue	padj	absLog2FC
ENSMUSG00000040434	Large2	228366	protein_coding	111.7449363	0.080172437	0.107486733	-0.745882165	0.4557386	0.671071533	0.080172437
ENSMUSG00000066129	Kndc1	76484	protein_coding	3.606082647	0.080216381	2.091188128	-0.038359237	0.969401261	1	0.080216381
ENSMUSG00000022684	Bfar	67118	protein_coding	736.4801588	-0.080216454	0.085646656	0.93659761	0.348965582	0.570683233	0.080216454
ENSMUSG00000005873	Reep5	13476	protein_coding	489.1467418	-0.080232445	0.122412939	0.655424545	0.512194453	0.714728777	0.080232445
ENSMUSG00000026819	Slc25a25	227731	protein_coding	785.0815469	-0.0802749	0.152864852	0.525136413	0.599488364	0.776544903	0.0802749
ENSMUSG00000062931	Zfp938	237411	protein_coding	65.00570351	-0.08029432	0.171041234	0.469444225	0.638752143	0.80301103	0.08029432
ENSMUSG00000056493	Foxk1	17425	protein_coding	429.2700268	-0.080317304	0.092484604	0.868439722	0.385153658	0.607432671	0.080317304
ENSMUSG00000024070	Prkd3	75292	protein_coding	153.9630995	0.080319799	0.171527195	-0.468262766	0.639596692	0.803461112	0.080319799
ENSMUSG00000064363	mt-Nd4	17719	protein_coding	13676.37189	-0.08037642	0.414303649	0.19400365	0.846173024	0.924222269	0.08037642
ENSMUSG00000007603	Dus3l	224907	protein_coding	383.9464826	0.080428848	0.06548051	-1.228286824	0.219339315	0.425536825	0.080428848
ENSMUSG00000002100	Mybpc3	17868	protein_coding	3.872944523	0.080487706	1.424039478	-0.056520699	0.954927007	1	0.080487706
ENSMUSG00000034163	Zfc3h1	216345	protein_coding	732.5074732	-0.080500581	0.117458015	0.685356216	0.493119166	0.699602226	0.080500581
ENSMUSG00000021902	Phf7	71838	protein_coding	73.97855052	-0.080504081	0.116860831	0.688888488	0.490893445	0.698215786	0.080504081
ENSMUSG00000038462	Uqcrfs1	66694	protein_coding	3233.181284	-0.080514189	0.080842517	0.995938669	0.319279945	0.540704851	0.080514189
ENSMUSG00000092416	Zfp141	434178	protein_coding	115.6701353	-0.080529887	0.13216181	0.609327966	0.542307074	0.735940424	0.080529887
ENSMUSG00000054065	Pkp3	56460	protein_coding	1072.214996	-0.080568224	0.111875607	0.720158985	0.471427114	0.684288244	0.080568224
ENSMUSG00000055932	Fto	26383	protein_coding	440.5940799	0.080655092	0.058252891	-1.384568052	0.166184586	0.353565093	0.080655092
ENSMUSG00000028729	Ebna1bp2	69072	protein_coding	456.6647702	-0.08068387	0.088353503	0.913193786	0.361140632	0.583549549	0.08068387
ENSMUSG00000028557	Rnf11	29864	protein_coding	907.5097372	-0.080705496	0.10366212	0.77854376	0.436248519	0.654452039	0.080705496
ENSMUSG00000038542	Pcid2	234069	protein_coding	189.7837869	0.080759947	0.092500836	-0.873072624	0.382623494	0.604944412	0.080759947
ENSMUSG00000050675	Gp1ba	14723	protein_coding	7.845267709	0.080769924	0.540255294	-0.149503253	0.881156543	0.941406719	0.080769924
ENSMUSG00000028893	Sesn2	230784	protein_coding	226.9272739	-0.080771703	0.124830274	0.647052194	0.517598164	0.717985391	0.080771703
ENSMUSG00000075267	Pjvk	381375	protein_coding	1.107493418	-0.080825237	1.069833437	0.075549366	0.939777622	1	0.080825237
ENSMUSG00000019837	Gtf3c6	67371	protein_coding	342.9980447	0.080832829	0.067278037	-1.201474247	0.22956729	0.438122528	0.080832829
ENSMUSG00000037857	Nufip2	68564	protein_coding	1244.422367	0.080844611	0.05308975	-1.522791332	0.127810919	0.294806545	0.080844611
ENSMUSG00000022231	Sema5a	20356	protein_coding	1266.67659	-0.080875077	0.149420614	0.541257825	0.588329886	0.76949784	0.080875077
ENSMUSG00000000399	Ndufa9	66108	protein_coding	1775.628822	-0.080890894	0.0924202	0.875251236	0.381437221	0.60376251	0.080890894
ENSMUSG00000032547	Ryk	20187	protein_coding	648.1702737	-0.080921488	0.081269167	0.995721879	0.319385297	0.540748207	0.080921488
ENSMUSG00000041237	Pklr	18770	protein_coding	728.7347591	0.080970502	0.074429898	-1.087876032	0.276649838	0.493582047	0.080970502
ENSMUSG00000099474	1700097N02Rik	67522	lncRNA	8.107112003	-0.080974678	0.420695071	0.192478313	0.847367554	0.924757321	0.080974678
ENSMUSG00000022938	Fam3b	52793	protein_coding	1221.499069	-0.080998044	0.083919646	0.965185719	0.334451799	0.556711723	0.080998044
ENSMUSG00000073530	Pappa2	23850	protein_coding	66.45226844	-0.081088709	0.219392182	0.36960619	0.711675938	0.849914408	0.081088709
ENSMUSG00000110080	Gm6145	620376	lncRNA	5.298191743	0.081107601	0.461175498	-0.175871445	0.860394939	0.931471303	0.081107601
ENSMUSG00000039477	Tnrc18	231861	protein_coding	1524.410869	0.081135179	0.168204565	-0.48236015	0.629550125	0.79763624	0.081135179
ENSMUSG00000028252	Ccnc	51813	protein_coding	254.2909199	0.081206208	0.081669266	-0.994330084	0.320062194	0.541378279	0.081206208
ENSMUSG00000034826	Nup54	269113	protein_coding	188.4304367	-0.08121536	0.105099093	0.77275034	0.439670148	0.657673264	0.08121536
ENSMUSG00000057054	Inca1	103844	protein_coding	16.56657705	-0.081247231	0.340662746	0.238497553	0.811495213	0.906704483	0.081247231
ENSMUSG00000050271	Prag1	244418	protein_coding	531.4977982	0.081299947	0.147861322	-0.549839173	0.582429688	0.76523541	0.081299947
ENSMUSG00000026193	Fn1	14268	protein_coding	19.23437255	-0.081364919	0.357177248	0.227799837	0.819801853	0.909153704	0.081364919

ensembl_gene_id	external_gene_name	entrezgene_id	gene_biotype	baseMean	log2FoldChange	lfcSE	stat	pvalue	padj	absLog2FC
ENSMUSG00000055675	Kbtbd11	74901	protein_coding	1060.439902	-0.081374952	0.126063151	0.645509426	0.518597112	0.719129457	0.081374952
ENSMUSG00000054874	Pcnx3	104401	protein_coding	1057.762378	0.081422901	0.078004057	-1.043829057	0.296564485	0.515753304	0.081422901
ENSMUSG00000017861	Mybl2	17865	protein_coding	82.93424163	0.081427824	0.185745411	-0.438384045	0.66110791	0.816884691	0.081427824
ENSMUSG00000064367	mt-Nd5	17721	protein_coding	18023.65428	-0.081481599	0.431444625	0.188857607	0.850204421	0.926816617	0.081481599
ENSMUSG00000048368	Omd	27047	protein_coding	2.653625591	0.081484241	0.811325206	-0.100433514	0.920000164	1	0.081484241
ENSMUSG00000092607	Scnm1	69269	protein_coding	206.0651448	0.081489869	0.076294507	-1.068096148	0.285477143	0.503945908	0.081489869
ENSMUSG00000028378	Ptgr1	67103	protein_coding	248.0092734	-0.081500764	0.149510409	0.545117659	0.585672598	0.767483504	0.081500764
ENSMUSG00000024862	Klc2	16594	protein_coding	157.0792134	0.081525814	0.112043486	-0.727626535	0.466842233	0.680564322	0.081525814
ENSMUSG00000040459	Arglu1	234023	protein_coding	643.9211325	-0.081531246	0.105096124	0.775777859	0.437880159	0.656184478	0.081531246
ENSMUSG00000028414	Fktn	246179	protein_coding	259.8932964	0.081577299	0.147254164	-0.55398976	0.579585854	0.762991261	0.081577299
ENSMUSG00000068134	Zfp120	104348	protein_coding	193.560182	-0.081601253	0.10225911	0.79798517	0.424879096	0.644361593	0.081601253
ENSMUSG00000034607	Pof1b	69693	protein_coding	2420.48784	-0.081695219	0.134482793	0.607477109	0.54353433	0.736456016	0.081695219
ENSMUSG00000028847	Trappc3	27096	protein_coding	469.8684007	-0.081713428	0.061770404	1.322857278	0.185882845	0.381372755	0.081713428
ENSMUSG00000028809	Srrm1	51796	protein_coding	696.9474567	0.081720786	0.08436043	-0.968709927	0.332689938	0.55512192	0.081720786
ENSMUSG00000029203	Ube2k	53323	protein_coding	648.0185302	0.081733647	0.060388046	-1.353473938	0.175904271	0.367893	0.081733647
ENSMUSG00000068876	Cgn	70737	protein_coding	2399.76795	-0.08174684	0.082849778	0.9866875	0.32379586	0.545573394	0.08174684
ENSMUSG00000053347	Zfp943	74670	protein_coding	212.1605897	-0.081747151	0.110633879	0.738897999	0.459968938	0.674354318	0.081747151
ENSMUSG00000071653	1810009A15Rik	66276	protein_coding	3.941485077	0.081748274	0.546003166	-0.149721245	0.880984546	1	0.081748274
ENSMUSG00000051306	Usp42	76800	protein_coding	233.0502944	0.081773405	0.111777198	-0.731575015	0.464428003	0.677942548	0.081773405
ENSMUSG00000074030	Exoc8	102058	protein_coding	484.5393203	-0.081782213	0.068246867	1.198329189	0.230788888	0.439438221	0.081782213
ENSMUSG00000036225	Kctd1	106931	protein_coding	54.30876168	-0.081790374	0.169641098	0.482137733	0.629708107	0.797713998	0.081790374
ENSMUSG00000025607	Copg2	54160	protein_coding	739.5936038	-0.081795858	0.089206779	0.916924237	0.359182342	0.581865529	0.081795858
ENSMUSG00000039410	Prdm16	70673	protein_coding	95.13244527	-0.081807102	0.165979957	0.492873374	0.622102062	0.792822461	0.081807102
ENSMUSG00000037197	Rbm17	76938	protein_coding	595.4068448	0.081811758	0.084055915	-0.97330162	0.330403418	0.552639068	0.081811758
ENSMUSG00000090019	Gimap1	16205	protein_coding	25.46064451	-0.08182311	0.262552258	0.311645046	0.755310297	0.877032235	0.08182311
ENSMUSG00000029782	Tmem209	72649	protein_coding	270.4821444	0.081828055	0.108064865	-0.757212398	0.448922623	0.665664739	0.081828055
ENSMUSG00000020189	Osbpl8	237542	protein_coding	249.6747805	0.081844973	0.114384741	-0.715523523	0.474285611	0.686291751	0.081844973
ENSMUSG00000051343	Rab11fip5	52055	protein_coding	58.320776	-0.081868098	0.19610311	0.417474756	0.676331185	0.826766769	0.081868098
ENSMUSG00000052776	Oas1a	246730	protein_coding	543.7411761	-0.081868704	0.172175608	0.475495365	0.634433936	0.800714821	0.081868704
ENSMUSG00000039463	Slc9a8	77031	protein_coding	505.40761	-0.081869707	0.055044567	1.487334939	0.136926371	0.309203076	0.081869707
ENSMUSG00000017837	Nkiras2	71966	protein_coding	856.689835	-0.081876693	0.086628264	0.94514988	0.344582363	0.566268598	0.081876693
ENSMUSG00000023905	Tnfrsf12a	27279	protein_coding	90.00946823	0.081905157	0.195911586	-0.418072042	0.675894445	0.826426838	0.081905157
ENSMUSG00000045248	Med26	70625	protein_coding	135.6988479	0.081917136	0.102438936	-0.799667966	0.423903197	0.643827591	0.081917136
ENSMUSG00000034024	Cct2	12461	protein_coding	2149.269614	-0.081920156	0.094737375	0.864707896	0.387199134	0.60964446	0.081920156
ENSMUSG00000029536	Gatc	384281	protein_coding	144.936096	-0.081948097	0.113520713	0.721877923	0.470369536	0.683706314	0.081948097
ENSMUSG00000035863	Palm	18483	protein_coding	29.66210753	-0.081974743	0.323594137	0.253325797	0.800016463	0.900994567	0.081974743
ENSMUSG00000066415	Msl2	77853	protein_coding	835.7683303	0.081990475	0.090515064	-0.905821322	0.365030434	0.587561035	0.081990475
ENSMUSG00000036606	Plxnb2	140570	protein_coding	5971.56391	0.082025033	0.0732112	-1.120389129	0.262547975	0.476573341	0.082025033
ENSMUSG00000021417	Eci2	23986	protein_coding	459.6443063	-0.082033637	0.072601349	1.129918904	0.258510398	0.471988664	0.082033637

ensembl_gene_id	external_gene_name	entrezgene_id	gene_biotype	baseMean	log2FoldChange	lfcSE	stat	pvalue	padj	absLog2FC
ENSMUSG00000047090	Tmem198b	73827	protein_coding	27.81034831	0.082051344	0.209638509	-0.391394427	0.695505711	0.838533729	0.082051344
ENSMUSG00000036368	Rmdn2	381110	protein_coding	55.48248884	0.082052654	0.140142474	-0.585494545	0.558215234	0.747162726	0.082052654
ENSMUSG00000020894	Vamp2	22318	protein_coding	174.4743137	0.082094844	0.132644355	-0.618909445	0.535976016	0.732041017	0.082094844
ENSMUSG00000044847	Lsm11	72290	protein_coding	141.2657211	-0.082107958	0.135131208	0.607616542	0.543441827	0.736456016	0.082107958
ENSMUSG00000030917	Tmem159	233806	protein_coding	221.3857999	-0.082121767	0.149279828	0.550119654	0.582237307	0.765104392	0.082121767
ENSMUSG00000110231	Gm6329	622523	lncRNA	6.108389897	0.082172411	0.440738727	-0.186442457	0.8520978	0.927743965	0.082172411
ENSMUSG00000053799	Exoc6	107371	protein_coding	379.6808668	-0.082176162	0.118557688	0.693132287	0.488226538	0.696271072	0.082176162
ENSMUSG00000029706	Pax4	18506	protein_coding	6.05739431	-0.082180299	0.526544577	0.156074724	0.875974122	0.938630585	0.082180299
ENSMUSG00000029634	Rnf6	74132	protein_coding	841.7503041	0.082182997	0.058256476	-1.410710063	0.158330122	0.342188672	0.082182997
ENSMUSG00000018474	Chd3	216848	protein_coding	1068.309645	0.082184637	0.158731998	-0.517757216	0.604627673	0.779942456	0.082184637
ENSMUSG00000029836	Cbx3	12417	protein_coding	234.534599	0.082198381	0.144152467	-0.570218342	0.568529617	0.754536534	0.082198381
ENSMUSG00000023328	Ache	11423	protein_coding	35.77220164	-0.08220532	0.289988879	0.28347749	0.776810835	0.888506311	0.08220532
ENSMUSG00000038844	Kif16b	16558	protein_coding	1059.296738	-0.082234385	0.064267063	1.279572794	0.200695423	0.401975042	0.082234385
ENSMUSG00000102758	Naaladl2	635702	protein_coding	148.4376237	-0.082305518	0.183894	0.447570435	0.654463244	0.812494814	0.082305518
ENSMUSG00000002205	Vrk3	101568	protein_coding	398.7915335	-0.082358149	0.073360109	1.122655765	0.261583722	0.475244062	0.082358149
ENSMUSG00000087174	5530601H04Rik	71445	lncRNA	90.67551369	-0.082523604	0.138257394	0.596883838	0.550584939	0.741538599	0.082523604
ENSMUSG00000023020	Cox14	66379	protein_coding	768.9662218	-0.082527965	0.072020537	1.145894882	0.251838649	0.464320478	0.082527965
ENSMUSG00000025512	Chid1	68038	protein_coding	690.5544785	0.082529247	0.129338157	-0.638088937	0.523415787	0.722112641	0.082529247
ENSMUSG00000039242	B3galnt2	97884	protein_coding	343.5186353	0.082546348	0.102312354	-0.806807239	0.419777548	0.639797547	0.082546348
ENSMUSG00000032058	Ppp2r1b	73699	protein_coding	403.5104658	0.082562516	0.144594522	-0.570993383	0.568004128	0.754081531	0.082562516
ENSMUSG00000032228	Tcf12	21406	protein_coding	839.8638007	0.082617352	0.051414572	-1.606885933	0.108079393	0.261665898	0.082617352
ENSMUSG00000055730	Ces2a	102022	protein_coding	2071.791551	0.082620146	0.183594449	-0.450014401	0.652700057	0.811601896	0.082620146
ENSMUSG00000040482	Dxo	112403	protein_coding	272.2389135	0.082635177	0.075288989	-1.097573204	0.272390899	0.488304745	0.082635177
ENSMUSG00000054808	Actn4	60595	protein_coding	5271.025557	0.082645002	0.126510917	-0.653263796	0.513586228	0.715485319	0.082645002
ENSMUSG00000029440	Psmd9	67151	protein_coding	241.1995837	0.08266454	0.09295957	-0.889252604	0.373867338	0.595844868	0.08266454
ENSMUSG00000025971	Maip1	68115	protein_coding	117.7726876	0.082726463	0.091652877	-0.902606282	0.366734891	0.589522317	0.082726463
ENSMUSG00000024548	Setbp1	240427	protein_coding	464.815649	0.082729268	0.118896969	-0.695806362	0.486550104	0.695624496	0.082729268
ENSMUSG00000033323	Ctdp1	67655	protein_coding	336.0465754	-0.082730852	0.082297082	1.005270781	0.314766481	0.535937072	0.082730852
ENSMUSG00000022394	L3mbtl2	214669	protein_coding	221.4514775	0.082752993	0.123494078	-0.670096859	0.502796047	0.707478587	0.082752993
ENSMUSG00000035754	Wdr18	216156	protein_coding	410.2386945	-0.082809578	0.088224575	0.93862257	0.347924561	0.569833005	0.082809578
ENSMUSG00000026679	Enkur	71233	protein_coding	8.340947251	0.082816995	0.477991283	-0.173260471	0.862446693	0.932036168	0.082816995
ENSMUSG00000054611	Kdm2a	225876	protein_coding	1224.216544	-0.082828568	0.059317614	1.396357031	0.162607008	0.348151449	0.082828568
ENSMUSG00000039899	Fgl2	14190	protein_coding	681.4582921	-0.082852843	0.159612133	0.519088629	0.603698937	0.779177919	0.082852843
ENSMUSG00000015224	Cyp2j9	74519	protein_coding	27.80810996	-0.082975546	0.231962018	0.357711777	0.720559019	0.855204229	0.082975546
ENSMUSG00000045038	Prkce	18754	protein_coding	267.9625607	-0.082993064	0.187708428	0.442138187	0.658389213	0.815651873	0.082993064
ENSMUSG00000031993	Snx19	102607	protein_coding	536.4723763	0.083014614	0.108473489	-0.765298643	0.444093732	0.661295211	0.083014614
ENSMUSG00000074579	Lekr1	624866	protein_coding	28.91573691	0.083042608	0.259438432	-0.320085993	0.748903144	0.87389673	0.083042608
ENSMUSG00000032329	Hmg20a	66867	protein_coding	248.9452531	-0.083043465	0.087120332	0.953204179	0.340486629	0.561998621	0.083043465
ENSMUSG00000023707	Ogfod2	66627	protein_coding	280.2170006	0.083045701	0.079906215	-1.039289635	0.298670054	0.518527888	0.083045701

ensembl_gene_id	external_gene_name	entrezgene_id	gene_biotype	baseMean	log2FoldChange	lfcSE	stat	pvalue	padj	absLog2FC
ENSMUSG00000025428	Atp5a1	11946	protein_coding	20336.21103	-0.083046504	0.091794743	0.904697818	0.365625497	0.588321101	0.083046504
ENSMUSG00000015961	Adss	11566	protein_coding	828.9832117	-0.083054571	0.084896454	0.978304357	0.327923816	0.550003442	0.083054571
ENSMUSG00000034333	Zbed4	223773	protein_coding	151.2548337	-0.083075647	0.127945488	0.649305021	0.516141241	0.717108906	0.083075647
ENSMUSG00000036737	Oxsr1	108737	protein_coding	928.2978305	-0.083081024	0.054341048	1.528881503	0.126293822	0.292303079	0.083081024
ENSMUSG00000028454	Pigo	56703	protein_coding	380.9580023	0.083103799	0.080118624	-1.037259438	0.299614964	0.519676664	0.083103799
ENSMUSG00000021893	Capn7	12339	protein_coding	858.5342921	0.083132707	0.075555178	-1.100291324	0.271205211	0.486866274	0.083132707
ENSMUSG00000017664	Slc35c2	228875	protein_coding	239.5094485	0.083191077	0.101051387	-0.823255173	0.4103629	0.630569734	0.083191077
ENSMUSG00000000197	Nalcn	338370	protein_coding	3.055616801	0.083245831	1.779821974	-0.046771999	0.962694946	1	0.083245831
ENSMUSG00000000131	Xpo6	74204	protein_coding	695.946306	0.083250422	0.054758206	-1.520327767	0.128428619	0.295635135	0.083250422
ENSMUSG00000053801	Grwd1	101612	protein_coding	192.8949483	-0.08326652	0.149257105	0.557873072	0.576931061	0.761228287	0.08326652
ENSMUSG00000044393	Dsg2	13511	protein_coding	3365.925221	-0.083266654	0.119688634	0.695693915	0.486620537	0.695624496	0.083266654
ENSMUSG00000030850	Ate1	11907	protein_coding	450.2662285	0.083304447	0.070318853	-1.184667321	0.236149008	0.446273789	0.083304447
ENSMUSG00000044042	Fmn1	14260	protein_coding	634.7943512	0.083409446	0.126123509	-0.661331476	0.50839976	0.712111524	0.083409446
ENSMUSG00000007987	Ift22	67286	protein_coding	79.97152245	0.083412557	0.14382182	-0.579971503	0.561933835	0.750140815	0.083412557
ENSMUSG00000051154	Commd3	12238	protein_coding	340.3582797	0.083412935	0.062965151	-1.324747638	0.185254867	0.380604488	0.083412935
ENSMUSG00000045007	Tubg2	103768	protein_coding	10.80499974	0.083453624	0.37449224	-0.222844734	0.823656351	0.910493405	0.083453624
ENSMUSG00000015671	Psma2	19166	protein_coding	1270.740536	0.083459507	0.080404767	-1.037992031	0.299273765	0.519260509	0.083459507
ENSMUSG00000031125	3830403N18Rik	70691	protein_coding	2.378487937	0.08345999	1.397174329	-0.059734844	0.95236682	1	0.08345999
ENSMUSG00000031101	Sash3	74131	protein_coding	11.79917071	-0.08346793	0.417676195	0.199838849	0.841606617	0.92158767	0.08346793
ENSMUSG00000040123	Zmym5	219105	protein_coding	332.9204503	-0.083484068	0.081316382	1.026657432	0.3045818	0.524764095	0.083484068
ENSMUSG00000030231	Plekha5	109135	protein_coding	1295.800449	0.083499433	0.057286098	-1.457586334	0.144954599	0.322004337	0.083499433
ENSMUSG00000087020	Gm16283	102635853	lncRNA	3.533157523	0.083502453	0.755776669	-0.110485618	0.912024255	1	0.083502453
ENSMUSG00000040383	Aqr	11834	protein_coding	702.3621942	0.083503748	0.075285356	-1.109163229	0.267359772	0.482533255	0.083503748
ENSMUSG00000085704	4921531C22Rik	66730	lncRNA	57.8816305	0.083504658	0.254831906	-0.327685255	0.743149653	0.870239999	0.083504658
ENSMUSG00000005371	Fbxo11	225055	protein_coding	401.7987009	0.083509159	0.095354688	-0.875774027	0.381152892	0.603601732	0.083509159
ENSMUSG00000002396	Ocel1	77090	protein_coding	319.060215	-0.083528305	0.087042277	0.95962913	0.337241903	0.559393767	0.083528305
ENSMUSG00000039901	Armh3	71617	protein_coding	215.0153421	-0.083558086	0.107592982	0.776612798	0.437387249	0.65562431	0.083558086
ENSMUSG00000033128	Gga1	106039	protein_coding	744.9021485	0.083584228	0.058749046	-1.422733383	0.154813459	0.336792184	0.083584228
ENSMUSG00000048271	Rbm33	381626	protein_coding	585.1870221	0.083615209	0.102605527	-0.814919146	0.415118593	0.635394239	0.083615209
ENSMUSG00000036693	Nop14	75416	protein_coding	188.052456	0.08365503	0.112988532	-0.74038514	0.459066333	0.673388737	0.08365503
ENSMUSG00000029032	Arhgef16	230972	protein_coding	1654.107241	-0.083680106	0.062461544	1.33970601	0.180340943	0.374092492	0.083680106
ENSMUSG00000020659	Cbll1	104836	protein_coding	83.25620086	-0.083722358	0.156338743	0.535518944	0.592291062	0.772293296	0.083722358
ENSMUSG00000027091	Zc3h15	69082	protein_coding	851.5654738	0.083729551	0.068355598	-1.224911401	0.220608604	0.427096189	0.083729551
ENSMUSG00000071757	Zhx2	387609	protein_coding	188.4805382	-0.083739055	0.082776127	1.011632913	0.311713603	0.532495959	0.083739055
ENSMUSG00000052298	Cdc42se2	72729	protein_coding	883.2458195	0.083741491	0.095581333	-0.876128097	0.3809604	0.603354756	0.083741491
ENSMUSG00000040697	Dnajc16	214063	protein_coding	353.9336687	-0.083749131	0.06485853	1.291258544	0.196614041	0.396311693	0.083749131
ENSMUSG00000020287	Mpg	268395	protein_coding	68.82915167	0.083755843	0.127327559	-0.657798227	0.510667794	0.713786099	0.083755843
ENSMUSG00000030102	Itpr1	16438	protein_coding	414.8143605	-0.083771402	0.190609644	0.439491939	0.660305122	0.816494106	0.083771402
ENSMUSG00000055341	Zfp457	431706	protein_coding	3.771408617	-0.083806607	0.583444773	0.143641027	0.885783941	1	0.083806607

Supplementary Table 1

ensembl_gene_id	external_gene_name	entrezgene_id	gene_biotype	baseMean	log2FoldChange	lfcSE	stat	pvalue	padj	absLog2FC
ENSMUSG00000038214	Bend3	331623	protein_coding	135.1912546	0.083839938	0.103442595	-0.810497244	0.417654448	0.63773884	0.083839938
ENSMUSG00000066233	Tmem42	66079	protein_coding	60.68036362	0.083841264	0.154711543	-0.541919903	0.587873685	0.769205771	0.083841264
ENSMUSG00000031555	Adam9	11502	protein_coding	1108.191737	0.083876763	0.080659423	-1.039887959	0.298391957	0.51821109	0.083876763
ENSMUSG00000047117	Ankdd1b	271144	protein_coding	33.63362269	-0.083911854	0.236925672	0.354169529	0.723211837	0.856549354	0.083911854
ENSMUSG00000010607	Pigyl	66268	protein_coding	111.2358106	-0.083969951	0.133514035	0.628922276	0.529399941	0.727088376	0.083969951
ENSMUSG00000068184	Ndufaf2	75597	protein_coding	131.6499938	-0.083997547	0.097140019	0.864705892	0.387200235	0.60964446	0.083997547
ENSMUSG00000045210	Vcpip1	70675	protein_coding	614.1568775	0.083999471	0.084547712	-0.993515605	0.32045875	0.541719295	0.083999471
ENSMUSG00000035226	Rims4	241770	protein_coding	194.7520098	0.084020043	0.310498861	-0.270596943	0.786701047	0.893302015	0.084020043
ENSMUSG00000047797	Gjb1	14618	protein_coding	574.0310504	0.084031207	0.123872036	-0.678371076	0.497536443	0.703090606	0.084031207
ENSMUSG00000021483	Cdk20	105278	protein_coding	30.65604135	0.084040926	0.208685697	-0.402715314	0.687157667	0.833389267	0.084040926
ENSMUSG00000036948	Trappc14	231807	protein_coding	243.5670798	-0.084047818	0.084639899	0.993004708	0.32070766	0.541897747	0.084047818
ENSMUSG00000089661	Mia	12587	protein_coding	5.744699605	-0.08409649	0.422663872	0.198967775	0.842287953	0.921900136	0.08409649
ENSMUSG00000021413	Prpf4b	19134	protein_coding	1282.578097	0.084101066	0.056388032	-1.491470161	0.1358381	0.307249743	0.084101066
ENSMUSG00000072704	Smim10l1	381820	protein_coding	1136.870505	0.084108821	0.091190584	-0.922341076	0.356350695	0.579378227	0.084108821
ENSMUSG00000089781	Gm15756	102631993	lncRNA	0.776972506	0.084141501	1.282152531	-0.065625188	0.947676235	1	0.084141501
ENSMUSG00000073131	Vma21	67048	protein_coding	186.4280695	0.084174027	0.126703409	-0.664339092	0.506473306	0.71028091	0.084174027
ENSMUSG00000026457	Adipor1	72674	protein_coding	2638.029858	-0.084234605	0.063976688	1.316645296	0.187957548	0.38424498	0.084234605
ENSMUSG00000022521	Crebbp	12914	protein_coding	1011.501531	0.084267748	0.12161295	-0.692917558	0.48836129	0.696363286	0.084267748
ENSMUSG00000022949	Clic6	209195	protein_coding	710.4623773	-0.08427539	0.185687055	0.453857111	0.649931675	0.809917796	0.08427539
ENSMUSG00000048920	Fkrp	243853	protein_coding	209.4919459	0.084340561	0.093569633	-0.901366804	0.367393325	0.590040437	0.084340561
ENSMUSG00000002496	Tsc2	22084	protein_coding	753.4543215	-0.084403674	0.053901398	1.565890267	0.117374309	0.277378268	0.084403674
ENSMUSG00000097405	D630044L22Rik	240054	lncRNA	1.607990219	0.084439837	1.051730712	-0.080286556	0.93600935	1	0.084439837
ENSMUSG00000020224	Llph	66225	protein_coding	121.0579614	-0.084471881	0.133365532	0.633386152	0.526481506	0.724949186	0.084471881
ENSMUSG00000021704	Mtx3	382793	protein_coding	239.981147	-0.084503919	0.137419285	0.614934933	0.538597692	0.733377497	0.084503919
ENSMUSG00000029265	Dr1	13486	protein_coding	413.3455001	0.084562741	0.089801005	-0.94166809	0.346362597	0.568119977	0.084562741
ENSMUSG00000025858	Get4	67604	protein_coding	289.025544	0.084564145	0.096688711	-0.874602051	0.381790471	0.604102573	0.084564145
ENSMUSG00000036180	Gatad2a	234366	protein_coding	1130.68463	0.084615897	0.055358125	-1.528518121	0.126383948	0.292373241	0.084615897
ENSMUSG00000027956	Tmem144	70652	protein_coding	103.2534975	-0.084641179	0.162374869	0.521270194	0.602178562	0.778302948	0.084641179
ENSMUSG00000066861	Oas1g	23960	protein_coding	199.9957288	-0.084642546	0.195783546	0.43232717	0.665503643	0.819563591	0.084642546
ENSMUSG00000021534	1700001L19Rik	69315	protein_coding	4.122505672	0.084654693	0.560043654	-0.151157312	0.879851623	1	0.084654693
ENSMUSG00000016257	Prelid3b	66390	protein_coding	1700.464768	0.084655725	0.080784489	-1.047920542	0.294675217	0.513807046	0.084655725
ENSMUSG00000027014	Cwc22	80744	protein_coding	332.7712011	0.084673044	0.0923135	-0.917233605	0.359020241	0.581717059	0.084673044
ENSMUSG00000022756	Slc7a4	224022	protein_coding	213.8582661	0.084681737	0.145244085	-0.583030537	0.559872743	0.748554316	0.084681737
ENSMUSG00000018171	Vmp1	75909	protein_coding	1363.21093	0.084705934	0.097229943	-0.871191851	0.383649408	0.606155692	0.084705934
ENSMUSG00000035671	Zswim4	212168	protein_coding	228.9599354	0.084786377	0.162616833	-0.521387454	0.602096889	0.778258299	0.084786377
ENSMUSG00000018882	Mrpl45	67036	protein_coding	430.3129331	-0.084827987	0.092182184	0.920221052	0.357457255	0.580534441	0.084827987
ENSMUSG00000049858	Suox	211389	protein_coding	240.1947429	-0.084856108	0.143932669	0.589554185	0.555489575	0.745195357	0.084856108
ENSMUSG00000078201	Tmem203	227615	protein_coding	39.68713246	-0.084863328	0.177451474	0.478233997	0.632483668	0.799512805	0.084863328
ENSMUSG00000031731	Ap1g1	11765	protein_coding	1290.882232	0.084877314	0.06303284	-1.346557048	0.178122935	0.370562126	0.084877314

Supplementary Table 1

ensembl_gene_id	external_gene_name	entrezgene_id	gene_biotype	baseMean	log2FoldChange	lfcSE	stat	pvalue	padj	absLog2FC
ENSMUSG00000097290	1300002E11Rik	100043489	lncRNA	99.24224419	-0.08488835	0.12409893	0.684037725	0.493951344	0.700180271	0.08488835
ENSMUSG00000056648	Hoxb8	15416	protein_coding	49.44547246	-0.084901887	0.341205718	0.248829029	0.803493036	0.903013746	0.084901887
ENSMUSG00000018293	Pfn1	18643	protein_coding	5870.0814	-0.084922422	0.089605576	0.947735905	0.343263917	0.565170522	0.084922422
ENSMUSG00000027475	Kif3b	16569	protein_coding	744.0651421	0.084925827	0.068315697	-1.243137835	0.213817046	0.418758859	0.084925827
ENSMUSG00000043243	Niban3	100037278	protein_coding	10.75696494	0.084958069	0.387157528	-0.219440571	0.826306869	0.912263113	0.084958069
ENSMUSG00000047126	Cltc	67300	protein_coding	6589.824786	0.084985675	0.093587158	-0.908091198	0.36383004	0.586288962	0.084985675
ENSMUSG00000024181	Mrpl28	68611	protein_coding	627.8144038	0.085064893	0.076684585	-1.109282827	0.267308191	0.482492892	0.085064893
ENSMUSG00000046056	Sbsn	282619	protein_coding	4.410968919	-0.085099447	0.50080992	0.169923646	0.865070185	0.932932188	0.085099447
ENSMUSG00000022008	Gpalpp1	67467	protein_coding	493.0278922	0.085163916	0.103689563	-0.821335472	0.411455208	0.631719162	0.085163916
ENSMUSG00000058799	Nap1l1	53605	protein_coding	541.6312878	0.08517561	0.144747433	-0.588442974	0.556235	0.745631404	0.08517561
ENSMUSG00000051890	Klhdc1	271005	protein_coding	37.82838666	-0.085276132	0.204675672	0.416640294	0.676941532	0.826943172	0.085276132
ENSMUSG00000022377	Asap1	13196	protein_coding	251.9615957	0.085279526	0.258049261	-0.330477697	0.741039042	0.868956809	0.085279526
ENSMUSG00000007041	Clic1	114584	protein_coding	2243.990554	-0.08533874	0.064011978	1.333168307	0.182476577	0.377007156	0.08533874
ENSMUSG00000020697	Lig3	16882	protein_coding	441.532217	0.085377459	0.096031695	-0.889055012	0.373973515	0.595946987	0.085377459
ENSMUSG00000024958	Gpr137	107173	protein_coding	282.3076436	-0.085378271	0.11135125	0.766747306	0.443231772	0.660786267	0.085378271
ENSMUSG00000025728	Pigq	14755	protein_coding	716.5103422	-0.085411872	0.061932264	1.379117564	0.167858509	0.356391801	0.085411872
ENSMUSG00000072568	Lratd2	399603	protein_coding	517.5846693	0.085441291	0.096776458	-0.88287268	0.377305066	0.599520433	0.085441291
ENSMUSG00000033590	Myo5c	208943	protein_coding	2854.646692	0.085446586	0.096894096	-0.881855443	0.377854985	0.600047814	0.085446586
ENSMUSG00000057777	Mab21l2	23937	protein_coding	6.449792549	-0.085455187	0.483103397	0.176887987	0.859596375	0.93121061	0.085455187
ENSMUSG00000027380	Acoxl	74121	protein_coding	2.745458749	0.085509496	0.726821222	-0.117648595	0.906346099	1	0.085509496
ENSMUSG00000032301	Psma4	26441	protein_coding	1142.949208	0.08552117	0.083442307	-1.02491378	0.305403872	0.525632052	0.08552117
ENSMUSG00000025235	Bbs4	102774	protein_coding	58.48496626	-0.08553112	0.170191668	0.502557623	0.615275329	0.78770474	0.08553112
ENSMUSG00000059263	Usp47	74996	protein_coding	2184.258744	0.085533281	0.062372775	-1.371323957	0.170273991	0.359946836	0.085533281
ENSMUSG00000054764	Mtnr1a	17773	protein_coding	1.365097336	-0.085537984	1.17626515	0.072719985	0.942028945	1	0.085537984
ENSMUSG00000030042	Pole4	66979	protein_coding	254.4463464	0.085538382	0.103767602	-0.824326474	0.40975408	0.630279329	0.085538382
ENSMUSG00000036160	Surf6	20935	protein_coding	374.0021679	-0.08554724	0.07649802	1.118293516	0.263441655	0.477601877	0.08554724
ENSMUSG00000035623	Rsf1	233532	protein_coding	493.5247481	-0.085573049	0.076514612	1.118388322	0.26340118	0.477601877	0.085573049
ENSMUSG00000041415	Dicer1	192119	protein_coding	901.7598496	0.085588031	0.074024271	-1.156215794	0.247592901	0.459757614	0.085588031
ENSMUSG00000003528	Slc25a1	13358	protein_coding	988.037439	-0.085595312	0.075912034	1.127559197	0.259506127	0.472954995	0.085595312
ENSMUSG00000033581	Igf2bp2	319765	protein_coding	635.7329994	0.085611314	0.153536892	-0.557594419	0.577121368	0.761298159	0.085611314
ENSMUSG00000057531	Dtnbp1	94245	protein_coding	164.6894005	-0.085645151	0.110707492	0.773616576	0.439157567	0.657120605	0.085645151
ENSMUSG00000042699	Dhx9	13211	protein_coding	1002.806736	0.085650851	0.142504955	-0.601037708	0.547814871	0.739162904	0.085650851
ENSMUSG00000041638	Gcn1	231659	protein_coding	1224.23084	0.085754776	0.07842269	-1.093494451	0.274176764	0.490619315	0.085754776
ENSMUSG00000043702	Pde12	211948	protein_coding	401.3737513	0.085769598	0.07115307	-1.205423713	0.228039772	0.436620149	0.085769598
ENSMUSG00000042272	Sestd1	228071	protein_coding	509.4756121	0.085779607	0.073709495	-1.163752468	0.24452434	0.456022015	0.085779607
ENSMUSG00000025266	Gnl3l	237107	protein_coding	665.2886205	0.085797205	0.087138456	-0.984607818	0.324816748	0.546769252	0.085797205
ENSMUSG00000022884	Eif4a2	13682	protein_coding	3468.785449	-0.085814688	0.078882664	1.087877662	0.276649119	0.493582047	0.085814688
ENSMUSG00000047108	Dnajb7	57755	protein_coding	4.580369568	-0.085836778	0.494794829	0.173479537	0.862274511	0.932001143	0.085836778
ENSMUSG00000021221	Dpf3	70127	protein_coding	41.26573558	-0.085850713	0.293382162	0.292624174	0.769809434	0.884532517	0.085850713

ensembl_gene_id	external_gene_name	entrezgene_id	gene_biotype	baseMean	log2FoldChange	lfcSE	stat	pvalue	padj	absLog2FC
ENSMUSG00000070697	Utp3	65961	protein_coding	289.7086351	0.085877723	0.091841843	-0.935060973	0.349756878	0.571586846	0.085877723
ENSMUSG00000030798	Cd37	12493	protein_coding	10.85166666	0.085897477	0.35571215	-0.2414803	0.809182883	0.905779176	0.085897477
ENSMUSG00000032560	Dnajc13	235567	protein_coding	1568.426671	0.085971647	0.065919855	-1.304184403	0.192170716	0.38986575	0.085971647
ENSMUSG00000027810	Eif2a	229317	protein_coding	643.1805375	0.086003748	0.108571807	-0.792137027	0.428280783	0.647320963	0.086003748
ENSMUSG00000067370	B3galt4	54218	protein_coding	104.7652715	0.086039437	0.102694981	-0.837815402	0.402134387	0.623781054	0.086039437
ENSMUSG00000026918	Brd3	67382	protein_coding	429.5603543	-0.086125284	0.09499418	0.906637483	0.364598533	0.587183556	0.086125284
ENSMUSG00000006941	Eif1b	68969	protein_coding	132.9535484	0.0861439	0.124220385	-0.693476357	0.488010658	0.696186289	0.0861439
ENSMUSG00000030521	Mphosph10	67973	protein_coding	210.0242118	0.086168725	0.160063325	-0.538341465	0.590341329	0.770846581	0.086168725
ENSMUSG00000026341	Actr3	74117	protein_coding	3437.310809	-0.086171998	0.083827759	1.027964942	0.303966319	0.524360158	0.086171998
ENSMUSG00000024955	Esrra	26379	protein_coding	2221.328652	0.086206116	0.09083301	-0.94906154	0.342589314	0.564622747	0.086206116
ENSMUSG00000061898	Rbak	57782	protein_coding	104.3612038	-0.08621808	0.121820828	0.707744985	0.479103636	0.690541222	0.08621808
ENSMUSG00000021203	Otub2	68149	protein_coding	27.37900818	-0.086270681	0.226803805	0.38037581	0.703666469	0.844104331	0.086270681
ENSMUSG00000032468	Armc8	74125	protein_coding	500.1575359	-0.086273863	0.080381047	1.073311015	0.283131595	0.501193606	0.086273863
ENSMUSG00000031644	Nek1	18004	protein_coding	259.9087613	0.086334605	0.09135708	-0.945023689	0.344646782	0.566318105	0.086334605
ENSMUSG00000090659	Zfp493	72958	protein_coding	34.01283736	-0.086348796	0.214289495	0.402953939	0.68698211	0.8333203	0.086348796
ENSMUSG00000036686	Cc2d1a	212139	protein_coding	682.6734724	-0.086355597	0.093002682	0.928528037	0.353133731	0.57545396	0.086355597
ENSMUSG00000034674	Tdg	21665	protein_coding	264.728122	-0.0863714	0.102837004	0.839886391	0.400972089	0.622737045	0.0863714
ENSMUSG00000028398	Dmac1	66928	protein_coding	210.3406246	0.086382785	0.125242525	-0.689724083	0.490367718	0.69793377	0.086382785
ENSMUSG00000026083	Eif5b	226982	protein_coding	540.6758949	0.086399418	0.078757621	-1.097029304	0.272628582	0.488518708	0.086399418
ENSMUSG00000042185	Nfrkb	235134	protein_coding	284.5119275	0.086419484	0.07205065	-1.199426849	0.230362013	0.439030384	0.086419484
ENSMUSG00000094396	Vmn2r124	637021	protein_coding	1.553447458	-0.086488948	2.631079336	0.032872041	0.973776629	1	0.086488948
ENSMUSG00000017670	Elmo2	140579	protein_coding	194.7409318	0.086501559	0.08942775	-0.967278708	0.333404725	0.555809168	0.086501559
ENSMUSG00000034645	Zyg11a	230590	protein_coding	5.359491047	-0.086502189	0.703925806	0.122885378	0.902197865	0.951530343	0.086502189
ENSMUSG00000000216	Scnn1g	20278	protein_coding	25.60705508	-0.086551059	0.288819456	0.299671843	0.764427478	0.882132403	0.086551059
ENSMUSG00000021171	Esyt2	52635	protein_coding	2047.840396	-0.086655095	0.066837817	1.296497992	0.194803972	0.393901406	0.086655095
ENSMUSG00000026641	Usf1	22278	protein_coding	440.7036777	-0.086778545	0.060394881	1.436852653	0.150759866	0.330823349	0.086778545
ENSMUSG00000011877	Git1	216963	protein_coding	715.4736689	-0.086779416	0.095731437	0.906488182	0.364677517	0.587211164	0.086779416
ENSMUSG00000024610	Cd74	16149	protein_coding	61.81853981	0.08679968	0.324880106	-0.267174501	0.789334808	0.894720656	0.08679968
ENSMUSG00000030672	Mylpf	17907	protein_coding	15.38439179	0.086801538	0.466541409	-0.186053235	0.852403015	0.927761944	0.086801538
ENSMUSG00000033411	Ctdspl2	329506	protein_coding	340.966244	-0.086807802	0.098705664	0.879461196	0.379151264	0.60160505	0.086807802
ENSMUSG00000024491	Rbm27	225432	protein_coding	587.4703764	0.08681126	0.056029123	-1.549395284	0.121286727	0.284063113	0.08681126
ENSMUSG00000033033	Calhm2	72691	protein_coding	20.55098041	0.086813004	0.233096729	-0.372433385	0.709570201	0.848381285	0.086813004
ENSMUSG00000002372	Ranbp3	71810	protein_coding	676.5916014	0.086835264	0.053212145	-1.631869273	0.102707028	0.252397465	0.086835264
ENSMUSG00000028483	Snapc3	77634	protein_coding	158.4577113	-0.086838266	0.132608275	0.654848017	0.512565612	0.714893745	0.086838266
ENSMUSG00000004864	Mapk13	26415	protein_coding	2087.058654	0.086852496	0.077035874	-1.127429231	0.259561046	0.472954995	0.086852496
ENSMUSG00000042050	Dync2i1	217935	protein_coding	117.8703249	-0.086867136	0.126666134	0.685796065	0.492841718	0.699388858	0.086867136
ENSMUSG00000042298	Ttc19	72795	protein_coding	270.4744083	-0.086867349	0.084502541	1.027985045	0.303956862	0.524360158	0.086867349
ENSMUSG00000018677	Slc25a39	68066	protein_coding	1969.12372	-0.086885052	0.056224514	1.545323309	0.12226808	0.285349785	0.086885052
ENSMUSG00000029504	Ddx51	69663	protein_coding	138.2042885	-0.08692926	0.129338753	0.672105289	0.501516669	0.706297653	0.08692926

ensembl_gene_id	external_gene_name	entrezgene_id	gene_biotype	baseMean	log2FoldChange	lfcSE	stat	pvalue	padj	absLog2FC
ENSMUSG00000038510	Rpf2	67239	protein_coding	118.770027	-0.086935342	0.135970577	0.639368784	0.522583051	0.721840784	0.086935342
ENSMUSG00000021891	Mettl6	67011	protein_coding	169.4492327	0.086938513	0.080234296	-1.083558008	0.278560818	0.495777145	0.086938513
ENSMUSG00000021400	Wrnip1	78903	protein_coding	377.3311514	0.086948757	0.089215571	-0.974591721	0.329762822	0.552030063	0.086948757
ENSMUSG00000058488	Kl	16591	protein_coding	4.224595511	0.086975651	1.139615011	-0.076320205	0.939164352	1	0.086975651
ENSMUSG00000030401	Rtn2	20167	protein_coding	29.56129584	0.086982771	0.308815512	-0.28166581	0.778199773	0.889171179	0.086982771
ENSMUSG00000025162	Csnk1d	104318	protein_coding	1635.65125	0.08701654	0.057472363	-1.514058871	0.130010909	0.297863506	0.08701654
ENSMUSG00000046404	Yod1	226418	protein_coding	156.6423495	0.087052276	0.104938282	-0.829556897	0.406789355	0.627629974	0.087052276
ENSMUSG00000019494	Cops6	26893	protein_coding	500.6867127	0.087103422	0.076087247	-1.14478347	0.252298864	0.464617944	0.087103422
ENSMUSG00000001558	Klhl10	66720	protein_coding	2.140100407	0.087126778	0.741480773	-0.117503758	0.906460866	1	0.087126778
ENSMUSG00000061904	Slc25a3	18674	protein_coding	7224.770198	-0.087135293	0.070469285	1.236500306	0.216272652	0.421530084	0.087135293
ENSMUSG00000021395	Spin1	20729	protein_coding	718.7436703	-0.087139987	0.075676762	1.151476148	0.249536402	0.461770971	0.087139987
ENSMUSG00000004099	Dnmt1	13433	protein_coding	954.3335969	0.087229584	0.138046505	-0.631885499	0.527461699	0.725788994	0.087229584
ENSMUSG00000063108	Zfp26	22688	protein_coding	370.8039221	-0.087239471	0.070491092	1.237595687	0.215866014	0.421123611	0.087239471
ENSMUSG00000044674	Fzd1	14362	protein_coding	368.0297195	0.087243268	0.104868234	-0.831932269	0.405447176	0.62645534	0.087243268
ENSMUSG00000007036	Abhd16a	193742	protein_coding	790.063873	-0.087253526	0.080327388	1.086223867	0.27737996	0.494297255	0.087253526
ENSMUSG00000045314	Sowahb	78088	protein_coding	519.1512499	-0.087280416	0.121289345	0.719604975	0.471768248	0.684542733	0.087280416
ENSMUSG00000038806	Sde2	208768	protein_coding	269.8663414	-0.087304798	0.071070541	1.228424551	0.219287636	0.425536548	0.087304798
ENSMUSG00000034274	Thoc5	107829	protein_coding	380.2156107	-0.087321949	0.101388497	0.861260903	0.389094363	0.611719756	0.087321949
ENSMUSG00000020859	Spag9	70834	protein_coding	1172.069214	-0.08732851	0.092346497	0.945661316	0.344321359	0.56607413	0.08732851
ENSMUSG00000035686	Thrsp	21835	protein_coding	1.204472807	-0.087365452	2.016817188	0.043318479	0.965447661	1	0.087365452
ENSMUSG00000019897	Ccdc59	52713	protein_coding	226.7257678	0.087384268	0.071047589	-1.22993995	0.218719593	0.424883585	0.087384268
ENSMUSG00000019082	Slc25a22	68267	protein_coding	209.9047824	0.087390606	0.161988984	-0.539484873	0.589552332	0.770506411	0.087390606
ENSMUSG00000035834	Polr3g	67486	protein_coding	126.8418686	0.087397785	0.121583342	-0.718830258	0.472245512	0.684753919	0.087397785
ENSMUSG00000028217	Cdh17	12557	protein_coding	9368.265391	-0.087428268	0.086196269	1.014292956	0.310442984	0.53139398	0.087428268
ENSMUSG00000051022	Hs3st1	15476	protein_coding	135.8372902	0.087460763	0.105073137	-0.832379857	0.405194568	0.626416691	0.087460763
ENSMUSG00000037720	Tmem33	67878	protein_coding	732.3518865	-0.087518655	0.086317847	1.013911469	0.310624998	0.531626202	0.087518655
ENSMUSG00000010122	Slc47a1	67473	protein_coding	22.98406562	0.087561559	0.367254457	-0.238422046	0.81155377	0.906704483	0.087561559
ENSMUSG00000030752	Kdm8	77035	protein_coding	71.06086522	0.087563344	0.183753877	-0.476525153	0.633700292	0.800362194	0.087563344
ENSMUSG00000090210	Itga10	213119	protein_coding	14.11981055	0.08756749	0.469171803	-0.186642696	0.851940789	0.927725037	0.08756749
ENSMUSG00000043881	Kbtbd7	211255	protein_coding	173.139623	-0.087578661	0.101907953	0.859389854	0.390125465	0.612475705	0.087578661
ENSMUSG00000037640	Zfp60	22718	protein_coding	119.0250904	-0.087625557	0.136948689	0.639842248	0.522275163	0.721805342	0.087625557
ENSMUSG00000052299	Ltn1	78913	protein_coding	811.381982	0.087634669	0.092749417	-0.944854122	0.344733356	0.566362983	0.087634669
ENSMUSG00000027864	Ptgfrn	19221	protein_coding	2156.407059	0.087660114	0.055872758	-1.568924065	0.116665634	0.276098635	0.087660114
ENSMUSG00000039485	Tspyl4	72480	protein_coding	9.727491583	-0.087685452	0.333139137	0.2632097	0.792388955	0.896636371	0.087685452
ENSMUSG00000039414	Heatr5b	320473	protein_coding	397.9568725	0.087735563	0.074881817	-1.171653762	0.241336094	0.451964647	0.087735563
ENSMUSG00000021123	Rdh12	77974	protein_coding	8.261797029	-0.087743166	0.410240825	0.213882092	0.830639005	0.91440345	0.087743166
ENSMUSG00000024759	Atl3	109168	protein_coding	877.5037634	-0.087790499	0.09626023	0.912012148	0.361762322	0.584163582	0.087790499
ENSMUSG00000087630	Gm11513	102640343	lncRNA	1.930360315	-0.087819811	0.811508045	0.108218041	0.913822735	1	0.087819811
ENSMUSG00000041236	Vps41	218035	protein_coding	833.2065943	-0.087832254	0.073226221	1.199464522	0.230347372	0.439030384	0.087832254

Supplementary Table 1

ensembl_gene_id	external_gene_name	entrezgene_id	gene_biotype	baseMean	log2FoldChange	lfcSE	stat	pvalue	padj	absLog2FC
ENSMUSG00000061360	Phf5a	68479	protein_coding	443.7434928	0.087844644	0.108444828	-0.810039963	0.417917208	0.637944042	0.087844644
ENSMUSG00000026127	Imp4	27993	protein_coding	515.7899613	-0.087866425	0.085936468	1.022457953	0.306564203	0.527079777	0.087866425
ENSMUSG00000046782	Ttc6	70846	protein_coding	73.4206902	0.087931677	0.166006492	-0.529688185	0.596328141	0.774560557	0.087931677
ENSMUSG00000024251	Thada	240174	protein_coding	249.6851263	0.087949953	0.121871332	-0.721662357	0.470502091	0.683786987	0.087949953
ENSMUSG00000067150	Xpo5	72322	protein_coding	511.8374872	0.088017173	0.083990016	-1.047948043	0.294662546	0.513807046	0.088017173
ENSMUSG00000035284	Vps13c	320528	protein_coding	1045.858842	-0.088034521	0.144841662	0.607798335	0.543321234	0.736456016	0.088034521
ENSMUSG00000019718	L3hypdh	67217	protein_coding	9.409085421	-0.088071462	0.356009668	0.247385028	0.80461026	0.903777298	0.088071462
ENSMUSG00000026941	Mamdc4	381352	protein_coding	53.96107224	0.088094764	0.23944918	-0.367905893	0.712943406	0.850751312	0.088094764
ENSMUSG00000044566	Cage1	71213	protein_coding	28.0292417	0.08810175	0.221038492	-0.398581031	0.690201941	0.834766446	0.08810175
ENSMUSG00000030649	Anapc15	75430	protein_coding	61.5840391	0.088116759	0.139606456	-0.631179684	0.527923044	0.726026078	0.088116759
ENSMUSG00000073077	Cfap47	636104	protein_coding	30.39217275	-0.088121306	0.256919015	0.342992544	0.731604069	0.861932625	0.088121306
ENSMUSG00000036913	Trim67	330863	protein_coding	4.263515847	-0.088143735	1.266021426	0.069622625	0.944494028	1	0.088143735
ENSMUSG00000085241	Snhg3	399101	lncRNA	2.233972557	0.088154168	0.76924297	-0.1145986	0.908763289	1	0.088154168
ENSMUSG00000038291	Snx25	102141	protein_coding	346.6310805	-0.088155482	0.093877785	0.93904518	0.347707548	0.569590562	0.088155482
ENSMUSG00000020888	Dvl2	13543	protein_coding	102.610294	-0.088250779	0.127961164	0.689668458	0.490402706	0.69793377	0.088250779
ENSMUSG00000041565	L3mbtl4	320858	protein_coding	4.886827072	0.088286864	0.718523377	-0.12287264	0.902207952	0.951530343	0.088286864
ENSMUSG00000027620	Rbm39	170791	protein_coding	2632.99219	0.088299276	0.049347989	-1.789318625	0.073563516	0.197983688	0.088299276
ENSMUSG00000032101	Ddx25	30959	protein_coding	6.502323357	-0.088300684	0.603596625	0.146290884	0.883691762	0.942424291	0.088300684
ENSMUSG00000094936	Rbm4	19653	protein_coding	53.76943733	0.088365408	0.145078987	-0.609084812	0.542468224	0.736098624	0.088365408
ENSMUSG00000050447	Lypd6	320343	protein_coding	2.119288815	0.088386276	1.177679207	-0.075051232	0.940173949	1	0.088386276
ENSMUSG00000027001	Dusp19	68082	protein_coding	112.1927233	0.088399733	0.122301571	-0.722801288	0.469801976	0.683370901	0.088399733
ENSMUSG00000061544	Zfp229	381067	protein_coding	65.43624497	-0.088543631	0.165050475	0.536463955	0.591637941	0.771785126	0.088543631
ENSMUSG00000065847	Gm25188	115489822	snoRNA	0.979078393	0.088566162	1.186229521	-0.07466191	0.940483714	1	0.088566162
ENSMUSG00000043284	Tmem11	216821	protein_coding	365.1345699	-0.088596841	0.096925215	0.914074226	0.360677845	0.583005439	0.088596841
ENSMUSG00000025937	Lactb2	212442	protein_coding	284.34044	0.08864314	0.099045463	-0.894974262	0.370800847	0.593065878	0.08864314
ENSMUSG00000029330	Cds1	74596	protein_coding	2220.100154	-0.088647554	0.08833409	1.00354862	0.31559623	0.53655334	0.088647554
ENSMUSG00000021079	Timm9	30056	protein_coding	115.6158279	-0.088660999	0.144556253	0.613332159	0.539656733	0.734194205	0.088660999
ENSMUSG00000060475	Wtap	60532	protein_coding	627.6446884	-0.088680469	0.085802543	1.033541274	0.301350666	0.521536878	0.088680469
ENSMUSG00000009210	Prr29	75573	protein_coding	2.353087113	0.08870595	0.873998602	-0.101494384	0.919158015	1	0.08870595
ENSMUSG00000027108	Ola1	67059	protein_coding	842.1984811	0.088728563	0.101821795	-0.871410313	0.383530157	0.606087752	0.088728563
ENSMUSG00000019877	Serinc1	56442	protein_coding	1061.985152	-0.088761611	0.113136247	0.784555032	0.432714504	0.651280288	0.088761611
ENSMUSG00000042225	Ammecr1	56068	protein_coding	276.4674192	0.088773214	0.166744663	-0.532390138	0.594455814	0.77323163	0.088773214
ENSMUSG00000026966	Ssna1	68475	protein_coding	298.8328443	-0.088775865	0.11528665	0.770044626	0.441273422	0.659125086	0.088775865
ENSMUSG00000038299	Wdr36	225348	protein_coding	534.6067043	-0.088793859	0.089505226	0.99205224	0.321172043	0.542303804	0.088793859
ENSMUSG00000048696	Mex3d	237400	protein_coding	84.45859521	-0.088824355	0.134326617	0.661256551	0.5084478	0.712111524	0.088824355
ENSMUSG00000047388	Atmin	234776	protein_coding	405.8792573	0.088864952	0.096373832	-0.922085901	0.35648377	0.579378227	0.088864952
ENSMUSG00000039960	Rhou	69581	protein_coding	487.9694294	-0.088935174	0.107122786	0.830217155	0.406416018	0.627248048	0.088935174
ENSMUSG00000030079	Ruvbl1	56505	protein_coding	167.2010427	0.088954121	0.157358822	-0.565294785	0.571873275	0.757452299	0.088954121
ENSMUSG00000037366	Pafah2	100163	protein_coding	878.904703	-0.08896003	0.070562809	1.260721209	0.20740931	0.410345914	0.08896003

ensembl_gene_id	external_gene_name	entrezgene_id	gene_biotype	baseMean	log2FoldChange	lfcSE	stat	pvalue	padj	absLog2FC
ENSMUSG00000001767	Crnkl1	66877	protein_coding	378.3996272	0.088969499	0.074674511	-1.191430618	0.233484579	0.442885865	0.088969499
ENSMUSG00000032718	Mansc1	67729	protein_coding	759.3207405	0.089016089	0.142615624	-0.624167862	0.532517351	0.729065788	0.089016089
ENSMUSG00000021501	Caml	12328	protein_coding	250.4509871	0.089037896	0.084269999	-1.056578817	0.290703852	0.50962669	0.089037896
ENSMUSG00000030990	Pgap2	233575	protein_coding	622.2495058	-0.089074036	0.117602775	0.757414409	0.448801626	0.665664739	0.089074036
ENSMUSG00000001285	Myg1	60315	protein_coding	231.067875	0.089087044	0.095874198	-0.929207715	0.35278145	0.575163675	0.089087044
ENSMUSG00000076271	Mir697	735277	miRNA	0.905942072	0.089104948	1.081789121	-0.082368131	0.934353978	1	0.089104948
ENSMUSG00000026276	Septin2	18000	protein_coding	37.88971125	-0.08914291	0.264658406	0.336822516	0.736250699	0.8658627	0.08914291
ENSMUSG00000026205	Slc23a3	22626	protein_coding	50.42628245	0.089146135	0.155537325	-0.573149466	0.566543497	0.753111109	0.089146135
ENSMUSG00000040396	Abhd13	68904	protein_coding	1025.655518	0.089164857	0.09606676	-0.928155133	0.353327104	0.575712264	0.089164857
ENSMUSG00000021693	Kif2a	16563	protein_coding	317.0311523	-0.089172253	0.094297269	0.945650431	0.344326913	0.56607413	0.089172253
ENSMUSG00000021481	Zfp346	26919	protein_coding	95.02506379	0.08917513	0.152433922	-0.585008436	0.558542044	0.747347538	0.08917513
ENSMUSG00000025025	Mxi1	17859	protein_coding	637.6971302	-0.089228796	0.139167449	0.641161397	0.521417827	0.720922137	0.089228796
ENSMUSG00000026483	Niban1	63913	protein_coding	1164.926466	-0.08924032	0.086665188	1.029713568	0.303144484	0.523653558	0.08924032
ENSMUSG00000005204	Senp3	80886	protein_coding	542.2316589	-0.089329804	0.068072383	1.312276721	0.189426784	0.386626363	0.089329804
ENSMUSG00000025938	Slco5a1	240726	protein_coding	2.271420429	-0.089336811	1.762437285	0.050689356	0.959573059	1	0.089336811
ENSMUSG00000022945	Chaf1b	110749	protein_coding	114.6352875	0.089386956	0.178695695	-0.500218857	0.616920982	0.788881899	0.089386956
ENSMUSG00000055660	Mettl4	76781	protein_coding	272.1301617	-0.089420819	0.134434133	0.665164548	0.505945253	0.709931167	0.089420819
ENSMUSG00000034880	Mrpl34	94065	protein_coding	409.3700315	-0.089450532	0.101906672	0.877769139	0.380069015	0.60223179	0.089450532
ENSMUSG00000102976	Zc3h11a	70579	protein_coding	111.7819893	0.089470755	0.110538045	-0.80941141	0.418278541	0.638329247	0.089470755
ENSMUSG00000002059	Rab34	19376	protein_coding	2.946569567	-0.089472058	0.798383738	0.112066484	0.910770692	1	0.089472058
ENSMUSG00000071456	1110002L01Rik	100043040	lncRNA	16.97612085	-0.089473638	0.261327408	0.342381379	0.7320639	0.862351327	0.089473638
ENSMUSG00000043782	Bicdl2	212733	protein_coding	274.8778627	-0.089499341	0.089688192	0.997894353	0.318330591	0.539723961	0.089499341
ENSMUSG00000027341	Tmem230	70612	protein_coding	146.5393624	0.089529875	0.091759265	-0.975703922	0.329211208	0.551602465	0.089529875
ENSMUSG00000032913	Lrig2	269473	protein_coding	351.5835528	-0.089539329	0.085047399	1.052816784	0.292424971	0.511503652	0.089539329
ENSMUSG00000024084	Qpct	70536	protein_coding	7.144603626	0.089550597	0.457113634	-0.195904455	0.84468495	0.923599766	0.089550597
ENSMUSG00000003072	Atp5d	66043	protein_coding	4002.816721	-0.089569095	0.083806568	1.068759843	0.285177898	0.503574403	0.089569095
ENSMUSG00000022833	Ccdc14	239839	protein_coding	69.48795703	-0.089607611	0.154795443	0.578877582	0.562671778	0.75067522	0.089607611
ENSMUSG00000034218	Atm	11920	protein_coding	576.2834829	-0.089615418	0.156681992	0.571957353	0.567350867	0.753395965	0.089615418
ENSMUSG00000024319	Vps52	224705	protein_coding	497.7404191	0.089617978	0.076773712	-1.167300307	0.243089115	0.454166041	0.089617978
ENSMUSG00000033446	Lpar6	67168	protein_coding	171.2750344	0.089635477	0.099000041	-0.905408478	0.365249026	0.587772599	0.089635477
ENSMUSG00000026176	Ctdsp1	227292	protein_coding	712.9638435	-0.089636795	0.07126693	1.257761412	0.208478055	0.411572816	0.089636795
ENSMUSG00000028696	Ipp	16351	protein_coding	130.3827786	-0.089662617	0.12295285	0.729243912	0.465852474	0.679601393	0.089662617
ENSMUSG00000001247	Lsr	54135	protein_coding	621.9465722	0.089748263	0.216910682	-0.413756771	0.679052246	0.827951097	0.089748263
ENSMUSG00000071648	Rom1	19881	protein_coding	32.79686737	-0.089785096	0.283260027	0.316970584	0.751265923	0.875081807	0.089785096
ENSMUSG00000020978	Klhdc2	69554	protein_coding	295.3317119	-0.089788555	0.095853435	0.936727565	0.348898713	0.570683233	0.089788555
ENSMUSG00000029623	Pdap1	231887	protein_coding	680.8168822	0.089830196	0.086248878	-1.041523063	0.297632855	0.517311687	0.089830196
ENSMUSG00000043323	Fbrsl1	381668	protein_coding	368.286246	-0.089875403	0.141233895	0.636358598	0.524542719	0.723107237	0.089875403
ENSMUSG00000030034	Ino80b	70020	protein_coding	172.2200786	-0.089888508	0.105390158	0.852911785	0.393708226	0.616333078	0.089888508
ENSMUSG00000052572	Dlg2	23859	protein_coding	10.15676131	-0.089922424	0.580675886	0.154858203	0.876933105	0.938919382	0.089922424

Supplementary Table 1

ensembl_gene_id	external_gene_name	entrezgene_id	gene_biotype	baseMean	log2FoldChange	lfcSE	stat	pvalue	padj	absLog2FC
ENSMUSG00000043909	Trp53bp1	27223	protein_coding	412.0176718	0.089979206	0.08505024	-1.057953588	0.290076602	0.50901339	0.089979206
ENSMUSG00000077394	Gm24339	115487283	snoRNA	183.1854907	-0.089984546	0.148666121	0.60527944	0.54499334	0.736964526	0.089984546
ENSMUSG00000009035	Tmem184b	223693	protein_coding	301.7265981	0.090010238	0.106234451	-0.847279175	0.396839532	0.618855346	0.090010238
ENSMUSG00000018362	Kpna2	16647	protein_coding	1084.955951	-0.090030363	0.144053438	0.624978923	0.531984891	0.728820184	0.090030363
ENSMUSG00000045435	Tmem60	212090	protein_coding	114.3807168	0.090071035	0.114682294	-0.78539618	0.43222132	0.650656598	0.090071035
ENSMUSG00000037525	Bcdin3d	75284	protein_coding	60.27020649	-0.090088264	0.137795193	0.653783789	0.513251112	0.715139121	0.090088264
ENSMUSG00000060657	Marf1	223989	protein_coding	930.3898782	-0.090098275	0.106725547	0.844205328	0.398554678	0.620508339	0.090098275
ENSMUSG00000077505	Gm24233	115485895	snoRNA	13.31865458	-0.090106975	0.506469241	0.177912037	0.858792057	0.930949982	0.090106975
ENSMUSG00000045019	Acer1	171168	protein_coding	587.555246	-0.0901324	0.145816112	0.618123738	0.536493775	0.732400135	0.0901324
ENSMUSG00000079466	Prdm12	381359	protein_coding	0.978957204	-0.090160963	2.274192978	0.039645256	0.968375947	1	0.090160963
ENSMUSG00000000708	Kat2b	18519	protein_coding	559.2131352	-0.090176727	0.08986772	1.003438464	0.315649354	0.53655334	0.090176727
ENSMUSG00000013663	Pten	19211	protein_coding	1440.493439	-0.090188496	0.072354975	1.246472638	0.212590928	0.417180016	0.090188496
ENSMUSG00000032440	Tgfbr2	21813	protein_coding	786.8752464	-0.090217474	0.103563752	0.871129835	0.383683266	0.606155692	0.090217474
ENSMUSG00000034297	Med13	327987	protein_coding	1207.899547	0.090245199	0.068081342	-1.325549655	0.184988912	0.380389352	0.090245199
ENSMUSG00000029179	Zcchc4	78796	protein_coding	135.0003293	0.090271276	0.094115101	-0.959158254	0.337479027	0.559438733	0.090271276
ENSMUSG00000062373	Tmem65	74868	protein_coding	421.1174626	0.090287147	0.084738793	-1.065475957	0.2866606	0.50522539	0.090287147
ENSMUSG00000085595	Gm16090	102633478	lncRNA	5.94240911	0.09030837	0.573844958	-0.157374164	0.874949974	0.938415991	0.09030837
ENSMUSG00000029500	Pgam5	72542	protein_coding	361.8155275	0.09032613	0.105720462	-0.854386452	0.3928909	0.615578778	0.09032613
ENSMUSG00000043262	Uevld	54122	protein_coding	236.0878077	-0.090335706	0.115862122	0.779682816	0.435577596	0.653742472	0.090335706
ENSMUSG00000021147	Wdr37	207615	protein_coding	232.1176158	-0.090413519	0.082060211	1.101794868	0.270550862	0.485955725	0.090413519
ENSMUSG00000110012	Or51a43	259125	protein_coding	1.367230395	0.090433702	2.345153977	-0.038561946	0.969239642	1	0.090433702
ENSMUSG00000034714	Ttyh2	117160	protein_coding	328.4724258	-0.090448272	0.147318556	0.613963874	0.5392392	0.733766095	0.090448272
ENSMUSG00000040102	Klhl42	232539	protein_coding	295.4695803	-0.090454789	0.110689625	0.817193019	0.413818131	0.633873724	0.090454789
ENSMUSG00000050608	Micos10	433771	protein_coding	543.0706089	-0.090474771	0.098829056	0.915467325	0.359946349	0.582491838	0.090474771
ENSMUSG00000024026	Glo1	109801	protein_coding	868.3804269	-0.090515557	0.081734662	1.107431711	0.268107322	0.48334534	0.090515557
ENSMUSG00000058498	Rnf207	433809	protein_coding	3.705894836	-0.090531864	1.073822301	0.08430805	0.932811512	1	0.090531864
ENSMUSG00000003279	Dlgap1	224997	protein_coding	9.311617682	0.090538825	0.838683794	-0.107953469	0.914032603	0.958005484	0.090538825
ENSMUSG00000040888	Gfer	11692	protein_coding	463.1890007	0.090594195	0.088184233	-1.027328713	0.304265706	0.524504007	0.090594195
ENSMUSG00000020156	Pwwp3a	68114	protein_coding	327.2983054	-0.0906138	0.09362241	0.967864428	0.333112082	0.55548954	0.0906138
ENSMUSG00000001774	Chordc1	66917	protein_coding	601.1358465	0.090616373	0.115900739	-0.78184465	0.434305877	0.652783039	0.090616373
ENSMUSG00000038683	Pak1ip1	68083	protein_coding	473.449776	0.090632618	0.081902982	-1.106585087	0.268473358	0.483542311	0.090632618
ENSMUSG00000057506	Bloc1s2	73689	protein_coding	165.9683531	-0.090662602	0.143535902	0.631637108	0.527624033	0.725856654	0.090662602
ENSMUSG00000047514	Tspyl1	22110	protein_coding	483.5082896	0.090697981	0.072056496	-1.258706516	0.208136357	0.41124734	0.090697981
ENSMUSG00000022003	Slc25a30	67554	protein_coding	55.29017026	-0.09070042	0.175496899	0.516820641	0.60528137	0.780311961	0.09070042
ENSMUSG00000024359	Hspa9	15526	protein_coding	4096.984852	-0.090750127	0.090763694	0.999850521	0.317382852	0.538827241	0.090750127
ENSMUSG00000022100	Xpo7	65246	protein_coding	747.9754155	0.090763885	0.0729948	-1.24342946	0.21370962	0.418744149	0.090763885
ENSMUSG00000032285	Dnaja4	58233	protein_coding	39.77389029	-0.090769039	0.224659675	0.404029066	0.686191345	0.832872319	0.090769039
ENSMUSG00000005718	Tfap4	83383	protein_coding	187.9396536	-0.090814079	0.121333673	0.748465591	0.454179372	0.669812138	0.090814079
ENSMUSG00000037902	Sirpa	19261	protein_coding	31.0083744	0.090818599	0.28037495	-0.323918378	0.74599983	0.872287153	0.090818599

Supplementary Table 1

ensembl_gene_id	external_gene_name	entrezgene_id	gene_biotype	baseMean	log2FoldChange	lfcSE	stat	pvalue	padj	absLog2FC
ENSMUSG00000025764	Jade1	269424	protein_coding	538.5897295	0.09081907	0.072541791	-1.251955162	0.210586203	0.414644158	0.09081907
ENSMUSG00000037822	Smim14	68552	protein_coding	1559.01755	0.090836138	0.126647329	-0.717236908	0.47322793	0.685335967	0.090836138
ENSMUSG00000028394	Pole3	59001	protein_coding	306.8486804	0.09084653	0.123238012	-0.737163225	0.461023094	0.674995696	0.09084653
ENSMUSG00000114531	A530065N20Rik	328263	lncRNA	0.761390086	-0.090873239	2.21583847	0.041010769	0.967287311	1	0.090873239
ENSMUSG00000068115	Ninl	78177	protein_coding	24.25192258	0.090913909	0.226133051	-0.402037245	0.687656616	0.833648609	0.090913909
ENSMUSG00000046062	Ppp1r15b	108954	protein_coding	2453.875502	-0.090927435	0.064880889	1.401451739	0.16107903	0.345901869	0.090927435
ENSMUSG00000070798	Psg25	114868	protein_coding	2.077192107	-0.090938768	1.101029255	0.082594325	0.934174114	1	0.090938768
ENSMUSG00000040652	Oaz2	18247	protein_coding	232.2889813	-0.090973852	0.127109991	0.715709683	0.47417063	0.686291751	0.090973852
ENSMUSG00000029994	Anxa4	11746	protein_coding	3760.299753	-0.091081481	0.053875138	1.690603212	0.090912611	0.230406822	0.091081481
ENSMUSG00000037685	Atp8a1	11980	protein_coding	1415.028919	0.091148908	0.114345872	-0.797133344	0.425373593	0.644974953	0.091148908
ENSMUSG00000028437	Ubap1	67123	protein_coding	730.8447152	-0.091158433	0.059862768	1.522790141	0.127811217	0.294806545	0.091158433
ENSMUSG00000048310	Pskh1	244631	protein_coding	352.614403	-0.091181207	0.075592461	1.206220918	0.22773232	0.43633502	0.091181207
ENSMUSG00000021131	Erh	13877	protein_coding	229.2491588	-0.091196889	0.157841496	0.577775118	0.563415956	0.750868206	0.091196889
ENSMUSG00000027361	Gabpb1	14391	protein_coding	200.2355533	0.091200822	0.095164058	-0.95835364	0.337884464	0.559633454	0.091200822
ENSMUSG00000026502	Desi2	78825	protein_coding	401.7540063	0.091201844	0.071735032	-1.271371067	0.203596671	0.405906838	0.091201844
ENSMUSG00000000420	Galnt1	14423	protein_coding	1177.144389	0.091246691	0.086314966	-1.057136377	0.290449352	0.509468423	0.091246691
ENSMUSG00000074604	Mgst2	211666	protein_coding	976.5929977	-0.091247846	0.072703903	1.25506118	0.209456551	0.412914333	0.091247846
ENSMUSG00000057788	Ddx49	234374	protein_coding	294.5102007	0.091265471	0.087659796	-1.041132602	0.297814011	0.517476912	0.091265471
ENSMUSG00000041594	Tmtc4	70551	protein_coding	325.802073	0.091282593	0.095807281	-0.95277302	0.340705087	0.562302999	0.091282593
ENSMUSG00000038072	Galnt11	231050	protein_coding	236.8636733	-0.091291434	0.088288429	1.034013568	0.30112982	0.521287512	0.091291434
ENSMUSG00000048087	Ahcyl	11615	protein_coding	9.003623966	-0.091298584	0.35099371	0.260114587	0.794775386	0.897921648	0.091298584
ENSMUSG00000026192	Atic	108147	protein_coding	869.1200869	-0.091298591	0.113875957	0.801737201	0.422704992	0.642569972	0.091298591
ENSMUSG00000028111	Ctsk	13038	protein_coding	2.186025295	0.091327564	0.766117817	-0.11920825	0.905110371	1	0.091327564
ENSMUSG00000041058	Wwp1	107568	protein_coding	930.4823602	0.09141366	0.068761669	-1.329427595	0.183706939	0.378649075	0.09141366
ENSMUSG00000019889	Ptprk	19272	protein_coding	2077.456133	-0.091447142	0.088918487	1.028437899	0.303743889	0.524250269	0.091447142
ENSMUSG00000036523	Greb1	268527	protein_coding	143.0634574	-0.091479106	0.110155427	0.830454826	0.406281678	0.62720573	0.091479106
ENSMUSG00000028982	Slc25a33	70556	protein_coding	74.75868366	-0.091493942	0.150264477	0.608886036	0.54259998	0.736156431	0.091493942
ENSMUSG00000044018	Mrpl50	28028	protein_coding	447.4224522	0.091591616	0.103669255	-0.883498351	0.376967073	0.599156327	0.091591616
ENSMUSG00000097162	2310010J17Rik	78329	lncRNA	22.5970299	0.091604329	0.220470521	-0.415494681	0.677779808	0.827323214	0.091604329
ENSMUSG00000026940	Ccdc183	77058	protein_coding	2.158202852	-0.091623253	0.797940101	0.114824726	0.90858405	1	0.091623253
ENSMUSG00000032187	Smarca4	20586	protein_coding	1864.270814	0.091664664	0.06450989	-1.420939699	0.15533429	0.337697097	0.091664664
ENSMUSG00000033075	Senp1	223870	protein_coding	356.9392813	0.091683102	0.074133004	-1.236737976	0.216184375	0.421495722	0.091683102
ENSMUSG00000031983	2310022B05Rik	69551	protein_coding	224.4264115	-0.091694937	0.093661531	0.979003181	0.327578408	0.549759128	0.091694937
ENSMUSG00000037475	Thoc2	331401	protein_coding	815.4182992	0.091772245	0.065231564	-1.406868685	0.159466331	0.343609993	0.091772245
ENSMUSG00000023452	Pisd	320951	protein_coding	585.7610711	-0.091793861	0.087364871	1.050695318	0.293398547	0.512561987	0.091793861
ENSMUSG00000048581	E130311K13Rik	329659	protein_coding	37.54466638	0.091811691	0.237170428	-0.387112726	0.698672757	0.840340998	0.091811691
ENSMUSG00000020267	Hint1	15254	protein_coding	3289.977407	-0.091818325	0.095914638	0.957292097	0.338419843	0.560042619	0.091818325
ENSMUSG00000085105	Gm12758	100126229	lncRNA	4.895105878	-0.091860709	0.622390842	0.147593285	0.882663755	0.941987155	0.091860709
ENSMUSG00000026499	Acbd3	170760	protein_coding	1130.368925	0.091889589	0.063146465	-1.455181834	0.145618929	0.322785367	0.091889589

Supplementary Table 1

ensembl_gene_id	external_gene_name	entrezgene_id	gene_biotype	baseMean	log2FoldChange	lfcSE	stat	pvalue	padj	absLog2FC
ENSMUSG00000030265	Kras	16653	protein_coding	1260.398754	-0.092026371	0.058107507	1.583726019	0.113256081	0.270127107	0.092026371
ENSMUSG00000047909	Ankrd16	320816	protein_coding	203.0675355	-0.092043833	0.152080416	0.605231336	0.545025297	0.736964526	0.092043833
ENSMUSG00000022048	Dpysl2	12934	protein_coding	908.7598952	0.092049697	0.162021885	-0.568131255	0.569945846	0.755650645	0.092049697
ENSMUSG00000028134	Ptbp2	56195	protein_coding	117.1926193	-0.092135697	0.156948627	0.587043666	0.55717438	0.746364189	0.092135697
ENSMUSG00000005836	Gata6	14465	protein_coding	626.9015689	-0.092158831	0.063452024	1.45241752	0.146385548	0.32396287	0.092158831
ENSMUSG00000028690	Mmachc	67096	protein_coding	164.9900424	-0.092184279	0.121198185	0.760607747	0.446891391	0.664203441	0.092184279
ENSMUSG00000027012	Dync1i2	13427	protein_coding	1110.268729	0.092256529	0.064093351	-1.439408722	0.150034755	0.329724247	0.092256529
ENSMUSG00000047415	Gpr68	238377	protein_coding	14.92524598	-0.092273549	0.448083085	0.205929552	0.836845941	0.918494874	0.092273549
ENSMUSG00000060708	Bloc1s4	117197	protein_coding	87.68975658	-0.092306725	0.148016345	0.623625215	0.532873747	0.72936091	0.092306725
ENSMUSG00000057738	Sptan1	20740	protein_coding	6224.069403	-0.092311379	0.05088899	1.81397544	0.069681507	0.190422043	0.092311379
ENSMUSG00000046010	Zfp830	66983	protein_coding	158.9921388	0.09231494	0.100096392	-0.922260418	0.356392755	0.579378227	0.09231494
ENSMUSG00000097292	A230107N01Rik	320919	lncRNA	3.586617157	0.092365976	0.683781069	-0.135081213	0.892547664	1	0.092365976
ENSMUSG00000026358	Rgs1	50778	protein_coding	56.10332297	-0.092376724	0.265046668	0.348530033	0.727442163	0.859261888	0.092376724
ENSMUSG00000021782	Dlg5	71228	protein_coding	375.8630646	0.092386395	0.121040584	-0.763267924	0.445303627	0.662536791	0.092386395
ENSMUSG00000059013	Sh2d3c	27387	protein_coding	45.25823103	-0.092462208	0.159526334	0.579604671	0.562181242	0.750196731	0.092462208
ENSMUSG00000042446	Zmym4	67785	protein_coding	247.137144	-0.092505817	0.079586569	1.16232951	0.245101644	0.456768986	0.092505817
ENSMUSG00000034379	Wdr5b	69544	protein_coding	53.79782624	-0.092587041	0.182973725	0.506012768	0.612847687	0.785880315	0.092587041
ENSMUSG00000057841	Rpl32	19951	protein_coding	5424.702469	-0.092603847	0.097672678	0.948103899	0.343076564	0.564974708	0.092603847
ENSMUSG00000026356	Dars	226414	protein_coding	715.4848051	-0.092606183	0.097094265	0.953776034	0.340197023	0.561689026	0.092606183
ENSMUSG00000030726	Pold3	67967	protein_coding	373.2768737	-0.092667172	0.096475954	0.960520922	0.336793107	0.559233675	0.092667172
ENSMUSG00000027751	Supt20	56790	protein_coding	481.3795144	0.092693033	0.067509084	-1.373045333	0.169738254	0.359143126	0.092693033
ENSMUSG00000026116	Tmem131	56030	protein_coding	1670.119934	0.092719154	0.086923984	-1.066669403	0.286121149	0.50453867	0.092719154
ENSMUSG00000007097	Atp1a2	98660	protein_coding	5.598462455	-0.092819696	0.79312944	0.117029694	0.906836523	0.954353007	0.092819696
ENSMUSG00000049401	Ogfr	72075	protein_coding	720.9925613	-0.092835198	0.070051219	1.325247421	0.185089102	0.380500613	0.092835198
ENSMUSG00000009356	Lpo	76113	protein_coding	243.532443	0.09284753	0.22980771	-0.404022692	0.686196032	0.832872319	0.09284753
ENSMUSG00000079109	Pms2	18861	protein_coding	100.8493115	0.092871516	0.120637872	-0.76983715	0.441396499	0.659125086	0.092871516
ENSMUSG00000059325	Hopx	74318	protein_coding	204.2778739	0.092887444	0.176081542	-0.527525164	0.597828946	0.775716761	0.092887444
ENSMUSG00000022297	Fzd6	14368	protein_coding	130.0004297	-0.092931966	0.178839735	0.519638245	0.603315736	0.779139367	0.092931966
ENSMUSG00000025066	Sfr1	67788	protein_coding	394.4284715	0.092932261	0.087156282	-1.066271512	0.286300923	0.504747886	0.092932261
ENSMUSG00000028607	Cpt2	12896	protein_coding	1138.57578	-0.092945958	0.06978565	1.331877809	0.182900344	0.377599236	0.092945958
ENSMUSG00000085683	Tmem238l	71576	protein_coding	899.7247968	0.092950106	0.08818128	-1.054079804	0.291846382	0.510653862	0.092950106
ENSMUSG00000025981	Coq10b	67876	protein_coding	390.7919251	-0.092952642	0.108896144	0.853589837	0.393332291	0.615978138	0.092952642
ENSMUSG00000025470	Zfp511	69752	protein_coding	112.0648217	-0.092978395	0.122097996	0.761506316	0.446354708	0.663584952	0.092978395
ENSMUSG00000079283	2310009B15Rik	69549	protein_coding	74.65546005	0.093005324	0.115344525	-0.8063263	0.42005473	0.63998633	0.093005324
ENSMUSG00000015937	Macroh2a1	26914	protein_coding	1279.524277	0.093015369	0.053052409	-1.75327323	0.079555118	0.208999876	0.093015369
ENSMUSG00000030842	Lamtor1	66508	protein_coding	758.8787466	-0.093060685	0.070432103	1.321282222	0.186407279	0.3822113	0.093060685
ENSMUSG00000028782	Adgrb2	230775	protein_coding	4.879714499	-0.093094372	1.30857393	0.071141851	0.94328486	0.972595566	0.093094372
ENSMUSG00000005107	Slc2a9	117591	protein_coding	83.5984349	0.093112136	0.166038714	-0.560785695	0.57494364	0.760164897	0.093112136
ENSMUSG00000038827	Abitram	230234	protein_coding	101.6828616	0.093116051	0.123562813	-0.753592838	0.451093746	0.667666133	0.093116051

ensembl_gene_id	external_gene_name	entrezgene_id	gene_biotype	baseMean	log2FoldChange	lfcSE	stat	pvalue	padj	absLog2FC
ENSMUSG00000052544	St6galnac3	20447	protein_coding	2.121003391	0.093176513	1.033264382	-0.090176837	0.928146691	1	0.093176513
ENSMUSG00000052593	Adam17	11491	protein_coding	558.0451558	0.093181482	0.085175514	-1.093993766	0.273957712	0.490474594	0.093181482
ENSMUSG00000025060	Slk	20874	protein_coding	2173.74461	-0.09325568	0.0724723	1.286776891	0.198172064	0.398418975	0.09325568
ENSMUSG00000023079	Gtf2ird1	57080	protein_coding	900.9864121	0.093257303	0.099925819	-0.933265328	0.350682992	0.572816907	0.093257303
ENSMUSG00000078762	Haus5	71909	protein_coding	12.40532544	-0.093270804	0.384583813	0.242523998	0.808374164	0.905485545	0.093270804
ENSMUSG00000020875	Hoxb9	15417	protein_coding	499.254042	0.093278128	0.080460467	-1.159303836	0.246332359	0.458272537	0.093278128
ENSMUSG00000067194	Eif1ax	66235	protein_coding	815.8694392	-0.093296601	0.125933041	0.740842911	0.458788693	0.673341315	0.093296601
ENSMUSG00000024052	Lpin2	64898	protein_coding	724.2800779	-0.093349617	0.232522968	0.401464069	0.688078487	0.833786242	0.093349617
ENSMUSG00000071078	Nr2c2ap	75692	protein_coding	27.36724317	-0.093359538	0.212870721	0.438573882	0.660970326	0.816884691	0.093359538
ENSMUSG00000060227	Golm2	319996	protein_coding	233.903554	-0.093363359	0.198385357	0.470616182	0.63791485	0.802698942	0.093363359
ENSMUSG00000036435	Exoc1	69940	protein_coding	441.0878136	0.093364045	0.07343903	-1.27131371	0.203617067	0.405906838	0.093364045
ENSMUSG00000050890	Pdik1l	230809	protein_coding	363.3990395	0.09340545	0.097603786	-0.95698593	0.338574357	0.560242177	0.09340545
ENSMUSG00000039428	Tmem135	72759	protein_coding	552.8010373	0.09340854	0.104851186	-0.890867748	0.373000125	0.595257447	0.09340854
ENSMUSG00000003360	Ddx23	74351	protein_coding	966.0473989	0.093433642	0.044532193	-2.098114574	0.035895025	0.115594862	0.093433642
ENSMUSG00000089900	Tbc1d22bos	74092	lncRNA	4.248032864	0.093466233	0.5417496	-0.17252663	0.863023527	1	0.093466233
ENSMUSG00000037992	Rara	19401	protein_coding	302.0788982	-0.093472237	0.1539054	0.607335654	0.543628181	0.736522749	0.093472237
ENSMUSG00000026260	Ndufa10	67273	protein_coding	2077.738484	-0.0935175	0.08311199	1.125198673	0.260504855	0.474068167	0.0935175
ENSMUSG00000026603	Smyd2	226830	protein_coding	193.3625396	-0.093523672	0.104899856	0.891551957	0.372633131	0.594901732	0.093523672
ENSMUSG00000019966	Kitl	17311	protein_coding	1467.224304	-0.093557679	0.118164919	0.791755111	0.428503481	0.647539027	0.093557679
ENSMUSG00000030811	Fbxl19	233902	protein_coding	606.5415955	0.093584246	0.074934938	-1.248873328	0.211711408	0.416065952	0.093584246
ENSMUSG00000006585	Cdt1	67177	protein_coding	199.7967066	-0.093601819	0.140547484	0.665980041	0.505423858	0.709429427	0.093601819
ENSMUSG00000026240	Cops7b	26895	protein_coding	145.7033779	0.09370928	0.096153229	-0.974582772	0.329767263	0.552030063	0.09370928
ENSMUSG00000038535	Zfp280d	235469	protein_coding	244.4694919	-0.093720495	0.096552804	0.970665694	0.33171478	0.554166705	0.093720495
ENSMUSG00000066637	Ttc32	75516	protein_coding	89.60241738	0.093771418	0.131548139	-0.71282968	0.475951157	0.687930892	0.093771418
ENSMUSG00000079560	Hoxa3	15400	protein_coding	76.1928106	-0.093797113	0.16025992	0.585281165	0.558358678	0.747162726	0.093797113
ENSMUSG00000074766	Ism1	319909	protein_coding	21.46593545	0.093813026	0.751873405	-0.124772369	0.900703763	0.951219667	0.093813026
ENSMUSG00000063787	Chchd1	66121	protein_coding	258.7221668	-0.093852755	0.092942914	1.009789246	0.312596274	0.533617414	0.093852755
ENSMUSG00000036442	Thap11	59016	protein_coding	282.5939577	-0.093899262	0.095465589	0.983592756	0.325315788	0.547073822	0.093899262
ENSMUSG00000031851	Ntpcr	66566	protein_coding	156.442917	-0.093915214	0.111203024	0.844538306	0.398368668	0.620277218	0.093915214
ENSMUSG00000028328	Tmod1	21916	protein_coding	2.319459546	-0.093948985	1.044177952	0.089974112	0.928307787	1	0.093948985
ENSMUSG00000097487	Ptges3l	73635	protein_coding	19.66054709	-0.09395027	0.230389846	0.40778824	0.683429148	0.830902522	0.09395027
ENSMUSG00000057342	Sphk2	56632	protein_coding	1009.191439	-0.093965992	0.098371545	0.955215172	0.339468895	0.560935494	0.093965992
ENSMUSG00000047248	C2cd3	277939	protein_coding	392.0152112	0.093967873	0.090750565	-1.035452204	0.300457776	0.520523758	0.093967873
ENSMUSG00000040370	Etfrf1	67636	protein_coding	252.453004	0.093979141	0.098458888	-0.954501348	0.339829927	0.561307404	0.093979141
ENSMUSG00000046034	Otulin	432940	protein_coding	211.2440725	-0.09399677	0.097893978	0.960189501	0.33695985	0.559251755	0.09399677
ENSMUSG00000038848	Ythdf1	228994	protein_coding	448.1380149	0.094055118	0.068718274	-1.368706052	0.171091178	0.361027307	0.094055118
ENSMUSG00000071072	Ptges3	56351	protein_coding	974.5639108	-0.094095038	0.122566506	0.767705969	0.442661889	0.660473296	0.094095038
ENSMUSG00000071302	2610044O15Rik8	72139	protein_coding	93.91449155	-0.094100813	0.155314056	0.605874414	0.544598151	0.736796542	0.094100813
ENSMUSG00000031976	Urb2	382038	protein_coding	283.1867295	-0.094116681	0.113460483	0.829510664	0.406815505	0.627629974	0.094116681

ensembl_gene_id	external_gene_name	entrezgene_id	gene_biotype	baseMean	log2FoldChange	lfcSE	stat	pvalue	padj	absLog2FC
ENSMUSG00000018372	Cep95	320162	protein_coding	142.0176079	-0.094126294	0.099507632	0.945920348	0.344189214	0.566016798	0.094126294
ENSMUSG00000020734	Grin2c	14813	protein_coding	2.565877376	-0.094153306	1.06226862	0.088634178	0.929372644	1	0.094153306
ENSMUSG00000028901	Gmeb1	56809	protein_coding	216.8798913	-0.094200846	0.078568783	1.198960226	0.230543411	0.43912271	0.094200846
ENSMUSG00000046201	Scaf8	106583	protein_coding	661.0821937	-0.094216556	0.052754668	1.785937809	0.074109328	0.198998609	0.094216556
ENSMUSG00000026896	Ifih1	71586	protein_coding	896.1111371	-0.094228756	0.107343093	0.877827844	0.380037152	0.60223179	0.094228756
ENSMUSG00000074890	Lcmt2	329504	protein_coding	104.8531656	-0.094234284	0.130644159	0.721304991	0.470721887	0.683906563	0.094234284
ENSMUSG00000031483	Erlin2	244373	protein_coding	1600.535497	-0.094255673	0.078895972	1.194682962	0.232210922	0.441179379	0.094255673
ENSMUSG00000030652	Coq7	12850	protein_coding	259.5809379	-0.094261788	0.135536441	0.695471916	0.486759605	0.695624496	0.094261788
ENSMUSG00000089290	Gm23207	115488981	snRNA	1.198806553	0.094273203	1.507247034	-0.062546617	0.950127539	1	0.094273203
ENSMUSG00000050944	Efcab5	319634	protein_coding	54.79870693	0.094335114	0.163782904	-0.575976558	0.564631027	0.751588072	0.094335114
ENSMUSG00000021120	Pigh	110417	protein_coding	127.3475748	-0.094360595	0.111690934	0.844836652	0.398202047	0.62019321	0.094360595
ENSMUSG00000039458	Mtmr12	268783	protein_coding	460.6496001	-0.094366858	0.059590661	1.583584686	0.113288261	0.270127107	0.094366858
ENSMUSG00000039740	Alg2	56737	protein_coding	210.7378678	0.09442524	0.112723623	-0.837670379	0.402215854	0.623797203	0.09442524
ENSMUSG00000001524	Gtf2h4	14885	protein_coding	171.4065912	0.094434992	0.119130597	-0.792701413	0.427951807	0.647057325	0.094434992
ENSMUSG00000030200	Bcl2l14	66813	protein_coding	1019.285578	0.094493735	0.091319699	-1.034757409	0.300782218	0.520880615	0.094493735
ENSMUSG00000015647	Lama5	16776	protein_coding	220.6118158	0.094496581	0.263514974	-0.358600422	0.719894031	0.855153117	0.094496581
ENSMUSG00000020079	Supv3l1	338359	protein_coding	299.6652534	-0.094522555	0.101894275	0.927653245	0.353587469	0.575966015	0.094522555
ENSMUSG00000020650	Bcap29	12033	protein_coding	80.12767736	-0.094526413	0.14406658	0.656130054	0.511740448	0.714354347	0.094526413
ENSMUSG00000033707	Lrrc24	378937	protein_coding	2.749347438	-0.094536151	0.87339646	0.108239677	0.913805572	1	0.094536151
ENSMUSG00000015474	Ppt2	54397	protein_coding	124.7190533	-0.09454113	0.12628686	0.748622064	0.454085029	0.669812138	0.09454113
ENSMUSG00000045631	Tmprss12	75002	protein_coding	1.606814593	0.094644394	0.885553427	-0.106875984	0.914887366	1	0.094644394
ENSMUSG00000086359	9630013K17Rik	78883	lncRNA	4.45759765	-0.094722778	0.602462963	0.157225895	0.875066821	0.938415991	0.094722778
ENSMUSG00000037703	Lzts3	241638	protein_coding	1213.483171	0.094725775	0.066025833	-1.434677475	0.151379026	0.331436869	0.094725775
ENSMUSG00000020801	Med31	67279	protein_coding	59.58406693	0.094729703	0.147566252	-0.641946936	0.520907637	0.720614094	0.094729703
ENSMUSG00000027291	Vps39	269338	protein_coding	380.6155154	-0.094756948	0.076156203	1.244244654	0.213409534	0.418308017	0.094756948
ENSMUSG00000039050	Osbpl2	228983	protein_coding	1095.904788	-0.094768403	0.105747138	0.896179363	0.370156976	0.592724939	0.094768403
ENSMUSG00000042810	Krba1	77827	protein_coding	326.9760194	0.094772853	0.121873209	-0.77763484	0.436784316	0.654986223	0.094772853
ENSMUSG00000036990	Otud4	73945	protein_coding	1042.666477	-0.094788875	0.086992724	1.089618425	0.275881263	0.492718348	0.094788875
ENSMUSG00000019878	Hsf2	15500	protein_coding	87.67201104	-0.094824152	0.133285458	0.711436596	0.476813728	0.688415973	0.094824152
ENSMUSG00000028453	Fancg	60534	protein_coding	102.4198065	0.094853144	0.119548445	-0.793428505	0.427528209	0.647057325	0.094853144
ENSMUSG00000029920	Smarcad1	13990	protein_coding	357.4596729	-0.094916435	0.085252347	1.113358622	0.265554439	0.480535393	0.094916435
ENSMUSG00000032615	Nt5m	103850	protein_coding	146.5455911	-0.094950132	0.10028333	0.946818697	0.343731172	0.565545141	0.094950132
ENSMUSG00000025078	Nhlrc2	66866	protein_coding	1005.867775	-0.094979856	0.073495954	1.292314068	0.196248402	0.395804361	0.094979856
ENSMUSG00000085492	Trmt61b	68789	lncRNA	59.4461778	0.094988042	0.132872523	-0.714881002	0.474682577	0.686659782	0.094988042
ENSMUSG00000022304	Dpys	64705	protein_coding	1.987987616	0.094995913	1.337272027	-0.07103709	0.943368237	1	0.094995913
ENSMUSG00000001518	Itfg2	101142	protein_coding	135.5512881	0.095096243	0.107848678	-0.881756221	0.377908651	0.600075333	0.095096243
ENSMUSG00000047003	Zfp41	22701	protein_coding	80.52509336	0.095096447	0.198729502	-0.478522043	0.632278689	0.799512805	0.095096447
ENSMUSG00000041220	Elovl6	170439	protein_coding	1115.9085	-0.095098631	0.099314342	0.957551834	0.338288797	0.55988186	0.095098631
ENSMUSG00000044700	Tmem201	230917	protein_coding	182.8831733	0.095151444	0.095450409	-0.99686785	0.318828659	0.540266681	0.095151444

ensembl_gene_id	external_gene_name	entrezgene_id	gene_biotype	baseMean	log2FoldChange	lfcSE	stat	pvalue	padj	absLog2FC
ENSMUSG00000086728	Man2c1os	76794	lncRNA	12.83534159	-0.095283461	0.298970188	0.318705558	0.7499498	0.874376659	0.095283461
ENSMUSG00000022437	Samm50	68653	protein_coding	1575.537244	0.095306404	0.074729334	-1.275354648	0.202183743	0.404116133	0.095306404
ENSMUSG00000030609	Aen	68048	protein_coding	222.1027474	-0.095308512	0.152126056	0.626510108	0.530980409	0.728048657	0.095308512
ENSMUSG00000031309	Rps6ka3	110651	protein_coding	1731.852806	-0.09531119	0.115035119	0.828539934	0.407364789	0.628183969	0.09531119
ENSMUSG00000029482	Aacs	78894	protein_coding	1255.705574	-0.09534827	0.135475258	0.703805786	0.48155373	0.692354022	0.09534827
ENSMUSG00000024073	Birc6	12211	protein_coding	3018.825341	0.09535494	0.07209273	-1.322670676	0.185944919	0.38145272	0.09535494
ENSMUSG00000038112	AW551984	244810	protein_coding	19.94353953	-0.095396961	0.263617273	0.361876746	0.717444138	0.853563561	0.095396961
ENSMUSG00000002844	Adprh	11544	protein_coding	310.8894472	0.095411885	0.091198856	-1.046196082	0.295470511	0.514582498	0.095411885
ENSMUSG00000045854	Lyrm2	108755	protein_coding	115.6380785	-0.095429241	0.135025459	0.706749983	0.479721862	0.690909666	0.095429241
ENSMUSG00000027165	Iftap	68170	protein_coding	55.17319935	-0.095456102	0.173450188	0.550337263	0.58208807	0.765030035	0.095456102
ENSMUSG00000022141	Nipbl	71175	protein_coding	1174.539931	-0.095466018	0.086796819	1.099879235	0.271384743	0.48708267	0.095466018
ENSMUSG00000037958	Nsrp1	237859	protein_coding	235.9718144	0.095488452	0.094782942	-1.007443425	0.313721732	0.534985614	0.095488452
ENSMUSG00000097357	Gm16793	100504714	lncRNA	3.801329604	-0.095504279	0.640101629	0.149201744	0.881394444	1	0.095504279
ENSMUSG00000030061	Uba3	22200	protein_coding	579.4614206	-0.095504751	0.06269104	1.523419462	0.127653796	0.294670784	0.095504751
ENSMUSG00000043421	Hilpda	69573	protein_coding	89.08040033	-0.095511281	0.110100634	0.867490747	0.38567318	0.607820554	0.095511281
ENSMUSG00000010067	Rassf1	56289	protein_coding	198.7444279	0.095527919	0.142457249	-0.670572536	0.502492884	0.707250275	0.095527919
ENSMUSG00000024426	Atat1	73242	protein_coding	51.74323032	-0.095569594	0.204524244	0.467277579	0.640301296	0.803678477	0.095569594
ENSMUSG00000001507	Itga3	16400	protein_coding	1774.84768	0.095588798	0.07835121	-1.220004106	0.222463318	0.429533455	0.095588798
ENSMUSG00000002968	Med25	75613	protein_coding	862.2584561	0.095599878	0.073801827	-1.295359237	0.195196333	0.394331932	0.095599878
ENSMUSG00000063235	Ptpmt1	66461	protein_coding	292.5469103	-0.095626877	0.091500146	1.04510081	0.29597638	0.515205432	0.095626877
ENSMUSG00000046806	Cyren	78412	protein_coding	23.53843467	0.095670293	0.237013145	-0.403649736	0.686470306	0.833066625	0.095670293
ENSMUSG00000028295	Smim8	66291	protein_coding	139.1077029	0.095670924	0.133044861	-0.719087709	0.47208688	0.684706793	0.095670924
ENSMUSG00000027411	Vps16	80743	protein_coding	418.046946	-0.095694264	0.06148658	1.556343902	0.119626337	0.281411586	0.095694264
ENSMUSG00000033790	Tubgcp5	233276	protein_coding	152.6557495	0.095804183	0.108051768	-0.886650769	0.375266951	0.597546855	0.095804183
ENSMUSG00000015357	Clpx	270166	protein_coding	603.6437515	-0.095831487	0.067953853	1.410243624	0.158467759	0.342262172	0.095831487
ENSMUSG00000028647	Mycbp	56309	protein_coding	177.5250763	-0.09584154	0.099123047	0.966894607	0.333596723	0.555969435	0.09584154
ENSMUSG00000090326	Dthd1	100322896	protein_coding	3.279251972	0.095876308	0.589041374	-0.162766679	0.870702144	1	0.095876308
ENSMUSG00000035274	Tpbg	21983	protein_coding	45.49741922	-0.095879838	0.179101948	0.535336655	0.592417084	0.772328749	0.095879838
ENSMUSG00000048899	Rimkla	194237	protein_coding	24.89882453	0.095920559	0.344082041	-0.278772349	0.780419536	0.890186044	0.095920559
ENSMUSG00000028086	Fbxw7	50754	protein_coding	247.4532085	-0.095984172	0.073576858	1.304542949	0.192048526	0.389807821	0.095984172
ENSMUSG00000017734	Dbndd2	52840	protein_coding	36.28633515	-0.095993311	0.203263947	0.47225941	0.636741639	0.801948109	0.095993311
ENSMUSG00000028751	Pla2g2e	26970	protein_coding	2.279100459	-0.096019963	1.30839098	0.073387821	0.941497509	1	0.096019963
ENSMUSG00000028089	Chd1l	68058	protein_coding	213.4654384	-0.096070814	0.11345236	0.846794327	0.397109771	0.619075876	0.096070814
ENSMUSG00000082315	Gm16523	100042584	transcribed_processed_pseudogene	3.656768762	-0.09608863	0.77732303	0.123614798	0.901620275	1	0.09608863
ENSMUSG00000001467	Cyp51	13121	protein_coding	1493.539929	0.096155637	0.13556247	-0.709308679	0.478132944	0.689777883	0.096155637
ENSMUSG00000020962	Gtf2a1	83602	protein_coding	444.1433746	-0.096171082	0.086450088	1.112446313	0.265946301	0.480980968	0.096171082
ENSMUSG00000038056	Kmt2c	231051	protein_coding	1692.979842	0.096185689	0.139230925	-0.690835667	0.489668819	0.697463418	0.096185689
ENSMUSG00000073926	Or52d3	57250	protein_coding	3.352536062	0.096345071	0.711279421	-0.135453197	0.892253567	1	0.096345071
ENSMUSG00000033454	Zbtb1	268564	protein_coding	318.1200781	0.096347787	0.099401122	-0.969282688	0.332404164	0.554813258	0.096347787

ensembl_gene_id	external_gene_name	entrezgene_id	gene_biotype	baseMean	log2FoldChange	lfcSE	stat	pvalue	padj	absLog2FC
ENSMUSG00000022956	Atp5o	28080	protein_coding	2770.003752	-0.096354634	0.08696735	1.107940323	0.26788759	0.483116267	0.096354634
ENSMUSG00000037553	Zdhhc18	503610	protein_coding	296.3816779	0.096372468	0.102697495	-0.938411086	0.348033191	0.569954395	0.096372468
ENSMUSG00000022680	Pdxdc1	94184	protein_coding	7144.321256	-0.096390217	0.138250513	0.697214171	0.485668772	0.695380179	0.096390217
ENSMUSG00000090069	E430024P14Rik	100040822	lncRNA	12.23048543	0.096412519	0.313355241	-0.307678015	0.75832735	0.878808271	0.096412519
ENSMUSG00000044501	Zfp758	224598	protein_coding	160.5172214	0.096438162	0.146674356	-0.657498449	0.510860468	0.713811425	0.096438162
ENSMUSG00000000563	Atp5pb	11950	protein_coding	3963.396068	-0.096477089	0.08231809	1.172003487	0.241195656	0.451804114	0.096477089
ENSMUSG00000029804	Herc3	73998	protein_coding	233.9247998	-0.096480381	0.097919291	0.985305142	0.324474208	0.546493327	0.096480381
ENSMUSG00000035314	Gdpd5	233552	protein_coding	117.5064908	-0.096488854	0.197762327	0.487903109	0.625618472	0.794916857	0.096488854
ENSMUSG00000022515	Anks3	72615	protein_coding	259.4382235	0.096536076	0.084801416	-1.138378121	0.254962626	0.467516411	0.096536076
ENSMUSG00000074457	S100a16	67860	protein_coding	1428.508977	-0.096565076	0.149766047	0.644772816	0.519074421	0.719334577	0.096565076
ENSMUSG00000000282	Mnt	17428	protein_coding	192.266028	0.096594361	0.126461004	-0.763827244	0.444970199	0.662372535	0.096594361
ENSMUSG00000020515	Cnot8	69125	protein_coding	417.2630813	-0.096635826	0.079075373	1.222072337	0.221680273	0.42856802	0.096635826
ENSMUSG00000033728	Lrrc14	223664	protein_coding	203.4043011	0.096638218	0.105323179	-0.917539895	0.358859797	0.581581822	0.096638218
ENSMUSG00000039081	Zfp503	218820	protein_coding	24.52005863	-0.096642767	0.287282993	0.336402674	0.736567233	0.866173274	0.096642767
ENSMUSG00000028530	Jak1	16451	protein_coding	1421.880104	-0.09668242	0.077131855	1.253469405	0.210034927	0.413854764	0.09668242
ENSMUSG00000031511	Arhgef7	54126	protein_coding	482.7505443	0.096690459	0.055493767	-1.742366131	0.081444398	0.212471132	0.096690459
ENSMUSG00000028048	Gba	14466	protein_coding	666.3890191	0.096696508	0.075591146	-1.27920416	0.200825171	0.402137369	0.096696508
ENSMUSG00000073563	Csnk1g3	70425	protein_coding	565.5615583	-0.096742499	0.071273486	1.357342035	0.174672557	0.365932262	0.096742499
ENSMUSG00000074797	Itpa	16434	protein_coding	369.3641328	-0.096768417	0.130457333	0.741762954	0.458230969	0.672761934	0.096768417
ENSMUSG00000032002	Dcun1d5	76863	protein_coding	401.9225579	0.096775869	0.071888572	-1.346192675	0.178240387	0.37057407	0.096775869
ENSMUSG00000023048	Prr13	66151	protein_coding	1560.919312	-0.096780977	0.105422509	0.91802954	0.358603401	0.581328932	0.096780977
ENSMUSG00000008035	Mid1ip1	68041	protein_coding	816.4118149	0.096857153	0.110203353	-0.878894794	0.379458321	0.601768559	0.096857153
ENSMUSG00000026254	Eif4e2	26987	protein_coding	798.0777801	-0.096864931	0.076028443	1.274061755	0.202641531	0.404694914	0.096864931
ENSMUSG00000027610	Gss	14854	protein_coding	1873.027529	-0.096879107	0.079123185	1.224408588	0.220798131	0.427312825	0.096879107
ENSMUSG00000028017	Egf	13645	protein_coding	84.14889033	-0.096909638	0.216051279	0.448549245	0.653756854	0.812167358	0.096909638
ENSMUSG00000051978	Erich1	234086	protein_coding	88.4623656	-0.096919013	0.12511101	0.774664137	0.438538148	0.656764359	0.096919013
ENSMUSG00000072582	Ptrh2	217057	protein_coding	163.9294927	0.096932877	0.120418911	-0.804963912	0.420840505	0.640714813	0.096932877
ENSMUSG00000038227	Hoxa9	15405	protein_coding	306.3192661	-0.096970766	0.101481211	0.955553884	0.339297669	0.560708667	0.096970766
ENSMUSG00000035266	Helq	191578	protein_coding	96.99089434	-0.096977854	0.125965548	0.769876012	0.441373444	0.659125086	0.096977854
ENSMUSG00000068823	Csde1	229663	protein_coding	4033.627671	-0.0969835	0.088375014	1.097408592	0.272462819	0.488337435	0.0969835
ENSMUSG00000030924	Rexo5	434234	protein_coding	74.84881116	-0.097022951	0.148171975	0.654799606	0.512596784	0.714893745	0.097022951
ENSMUSG00000097585	E230029C05Rik	319711	lncRNA	3.0196279	-0.097033289	0.685583262	0.141533924	0.887448162	1	0.097033289
ENSMUSG00000020812	Snhg16	66293	lncRNA	65.36258322	-0.097119863	0.129935563	0.747446352	0.454794175	0.670218722	0.097119863
ENSMUSG00000004268	Emg1	14791	protein_coding	664.1098304	0.097154246	0.099634548	-0.975105997	0.329507684	0.551855356	0.097154246
ENSMUSG00000023118	Sympk	68188	protein_coding	1257.885784	0.097160802	0.055382998	-1.754343489	0.079371668	0.208654479	0.097160802
ENSMUSG00000038495	Otud7b	229603	protein_coding	561.1072242	0.097195866	0.08388111	-1.158733666	0.246564764	0.458326261	0.097195866
ENSMUSG00000033216	Eefsec	65967	protein_coding	210.293974	0.097199558	0.091695461	-1.060025837	0.289132845	0.508005086	0.097199558
ENSMUSG00000030201	Lrp6	16974	protein_coding	2094.149072	-0.097238143	0.061273232	1.586959589	0.112521797	0.269146142	0.097238143
ENSMUSG00000020840	Blmh	104184	protein_coding	754.7798093	0.097270548	0.064177705	-1.515643907	0.129609418	0.297320449	0.097270548

ensembl_gene_id	external_gene_name	entrezgene_id	gene_biotype	baseMean	log2FoldChange	lfcSE	stat	pvalue	padj	absLog2FC
ENSMUSG00000022361	Zhx1	22770	protein_coding	403.1193151	-0.097273246	0.086189616	1.128595888	0.259068346	0.472369953	0.097273246
ENSMUSG00000041164	Zmiz2	52915	protein_coding	482.1686083	-0.097286875	0.122229754	0.795934474	0.426070122	0.645752753	0.097286875
ENSMUSG00000015882	Lcorl	209707	protein_coding	568.9023919	-0.0973149	0.088158513	1.103862771	0.26965267	0.484817006	0.0973149
ENSMUSG00000025262	Fam120c	207375	protein_coding	106.1704831	-0.097327135	0.142207664	0.684401477	0.493721684	0.699975951	0.097327135
ENSMUSG00000033396	Spg11	214585	protein_coding	668.4212716	-0.09733751	0.051478999	1.890819789	0.058648405	0.167999957	0.09733751
ENSMUSG00000055313	Pgbd1	319207	protein_coding	14.23247548	0.097408769	0.31758949	-0.306712822	0.75906197	0.878912418	0.097408769
ENSMUSG00000021670	Hmgcr	15357	protein_coding	2649.025496	0.097416251	0.13532286	-0.719880226	0.471598744	0.684381431	0.097416251
ENSMUSG00000113621	2900060N12Rik	102641621	lncRNA	1.900169643	-0.097416817	2.165715112	0.044981363	0.964122164	1	0.097416817
ENSMUSG00000057551	Zfp317	244713	protein_coding	340.2104578	-0.09743063	0.099825718	0.976007313	0.329060841	0.551494032	0.09743063
ENSMUSG00000064068	Mtx1	17827	protein_coding	354.4106156	0.097479148	0.071787419	-1.357886238	0.174499785	0.365696631	0.097479148
ENSMUSG00000028911	Srsf4	57317	protein_coding	132.3113178	-0.097487416	0.09556569	1.020108957	0.307676789	0.52811293	0.097487416
ENSMUSG00000022052	Ppp2r2a	71978	protein_coding	229.8579573	0.097543934	0.195969005	-0.497751848	0.618658959	0.789897483	0.097543934
ENSMUSG00000020226	Slc5a4b	64454	protein_coding	9.386331724	0.097555095	0.600427605	-0.162476032	0.870931001	0.936356422	0.097555095
ENSMUSG00000032750	Gab3	210710	protein_coding	1.64261965	0.097568554	1.068805422	-0.091287481	0.927264165	1	0.097568554
ENSMUSG00000061950	Ppp4r1	70351	protein_coding	854.2728103	0.097569114	0.072194445	-1.351476752	0.176542762	0.36866808	0.097569114
ENSMUSG00000024286	Ccny	67974	protein_coding	1097.207222	-0.097604421	0.097774685	0.998258611	0.318153972	0.539479894	0.097604421
ENSMUSG00000023078	Cxcl13	55985	protein_coding	3.725476048	-0.097625161	0.518688028	0.188215565	0.850707671	1	0.097625161
ENSMUSG00000034390	Cmip	74440	protein_coding	787.2449189	-0.09765765	0.14089806	0.69310855	0.488241432	0.696271072	0.09765765
ENSMUSG00000030281	Il17rc	171095	protein_coding	1245.03069	-0.097676161	0.105958606	0.921833204	0.356615585	0.579526645	0.097676161
ENSMUSG00000104368	2010103J01Rik	69889	lncRNA	6.384529396	0.097677562	0.542305604	-0.180115347	0.857062014	0.93035707	0.097677562
ENSMUSG00000054589	Gm9949	225609	lncRNA	3.597948128	-0.097691717	0.531229097	0.183897527	0.854093845	1	0.097691717
ENSMUSG00000030619	Eed	13626	protein_coding	363.6695245	0.097725079	0.080194599	-1.21859926	0.22299633	0.429954629	0.097725079
ENSMUSG00000098702	1500015A07Rik	68982	lncRNA	27.82996088	0.097854321	0.293601173	-0.333289951	0.738915424	0.867575493	0.097854321
ENSMUSG00000044322	Dsc1	13505	protein_coding	1.598105152	0.097860256	2.666357085	-0.036701857	0.970722728	1	0.097860256
ENSMUSG00000032290	Ptpn9	56294	protein_coding	654.0680618	0.097919177	0.061528283	-1.591449838	0.111508376	0.267419303	0.097919177
ENSMUSG00000086427	Hoxa11os	15397	lncRNA	275.465696	-0.097953495	0.160461162	0.610449865	0.541563844	0.735467298	0.097953495
ENSMUSG00000074151	Nlrc5	434341	protein_coding	30.26315127	-0.097955839	0.374076222	0.261860641	0.793428885	0.897198344	0.097955839
ENSMUSG00000043592	Unc5cl	76589	protein_coding	133.0830562	0.097984731	0.136288604	-0.71895029	0.472171549	0.684706793	0.097984731
ENSMUSG00000038658	Ric1	226089	protein_coding	365.4118696	-0.098016529	0.091768028	1.068090183	0.285479834	0.503945908	0.098016529
ENSMUSG00000071253	Slc25a16	73132	protein_coding	386.3097794	0.098027916	0.095691257	-1.024418724	0.305637542	0.525979404	0.098027916
ENSMUSG00000036026	Tmem63b	224807	protein_coding	913.2597842	-0.098043743	0.076879274	1.275294868	0.202204893	0.404116133	0.098043743
ENSMUSG00000028358	Zfp618	72701	protein_coding	429.2888367	-0.098065834	0.124523871	0.787526386	0.430973788	0.649489062	0.098065834
ENSMUSG00000020477	Mrps24	64660	protein_coding	546.3330773	-0.09810415	0.068264319	1.437121926	0.150683352	0.330726326	0.09810415
ENSMUSG00000030714	Sgf29	75565	protein_coding	310.7734134	-0.098129227	0.073707511	1.331332788	0.183079534	0.377733062	0.098129227
ENSMUSG00000061207	Stk19	54402	protein_coding	179.1444467	-0.098192667	0.106892558	0.918610885	0.358299137	0.580947662	0.098192667
ENSMUSG00000024331	Dsc2	13506	protein_coding	3077.280873	-0.098262823	0.100682294	0.975969253	0.329079701	0.551494032	0.098262823
ENSMUSG00000017286	Glod4	67201	protein_coding	572.8588186	-0.098297037	0.109217957	0.900008023	0.368115981	0.59060209	0.098297037
ENSMUSG00000021806	Nid2	18074	protein_coding	94.11891529	-0.098309335	0.138867017	0.7079387	0.478983325	0.690541222	0.098309335
ENSMUSG00000027427	Polr3f	70408	protein_coding	212.5351216	0.098326987	0.077389422	-1.270548143	0.203889445	0.406017465	0.098326987

ensembl_gene_id	external_gene_name	entrezgene_id	gene_biotype	baseMean	log2FoldChange	lfcSE	stat	pvalue	padj	absLog2FC
ENSMUSG00000022551	Cyc1	66445	protein_coding	3374.269143	-0.098339805	0.070076005	1.403330652	0.160518262	0.345021291	0.098339805
ENSMUSG00000044134	Pheta1	231717	protein_coding	856.6406299	0.098348062	0.104889027	-0.937639193	0.348429863	0.570284968	0.098348062
ENSMUSG00000037098	Rab11fip3	215445	protein_coding	272.3149248	0.098381466	0.097238135	-1.011758051	0.311653752	0.532495959	0.098381466
ENSMUSG00000071037	Camkmt	73582	protein_coding	88.32981505	-0.098408935	0.133606899	0.736555787	0.46139253	0.675184655	0.098408935
ENSMUSG00000015747	Vps45	22365	protein_coding	219.6935938	0.098420604	0.082637347	-1.190994237	0.233655847	0.443108941	0.098420604
ENSMUSG00000040124	Gorab	98376	protein_coding	188.1480892	-0.098427428	0.117588527	0.837049585	0.402564697	0.624030976	0.098427428
ENSMUSG00000051224	Tceanc	245695	protein_coding	65.83440949	-0.098448193	0.17148889	0.574079131	0.565914255	0.752577549	0.098448193
ENSMUSG00000026005	Rpe	66646	protein_coding	535.2417745	0.098472815	0.111345937	-0.884386244	0.376487746	0.598740239	0.098472815
ENSMUSG00000102748	Pcdhgb2	93700	protein_coding	7.892418808	0.098495431	0.403786388	-0.243929549	0.807285384	0.904987729	0.098495431
ENSMUSG00000004056	Akt2	11652	protein_coding	430.2851353	-0.098506512	0.065099062	1.513178671	0.130234281	0.298143983	0.098506512
ENSMUSG00000068329	Htra2	64704	protein_coding	179.9089845	-0.09855077	0.095954862	1.027053436	0.304395303	0.524552231	0.09855077
ENSMUSG00000037740	Mrps26	99045	protein_coding	369.6257949	0.098620534	0.090245619	-1.092801343	0.274481032	0.490866888	0.098620534
ENSMUSG00000031631	Cfap97	66756	protein_coding	203.928897	-0.098671033	0.127968286	0.771058486	0.440672266	0.658605063	0.098671033
ENSMUSG00000022538	Lsg1	224092	protein_coding	265.3562693	0.098736864	0.072294575	-1.365757575	0.172015066	0.362441146	0.098736864
ENSMUSG00000001416	Cct3	12462	protein_coding	1826.016615	0.098758567	0.113038541	-0.873671631	0.382297104	0.604659935	0.098758567
ENSMUSG00000030744	Rps3	27050	protein_coding	8881.17455	-0.098769374	0.102231453	0.96613489	0.333976688	0.556313398	0.098769374
ENSMUSG00000028080	Lrba	80877	protein_coding	2828.716916	-0.098777694	0.094499123	1.045276303	0.295895287	0.515123206	0.098777694
ENSMUSG00000059923	Grb2	14784	protein_coding	613.4558992	-0.098781129	0.067872906	1.455383811	0.145563036	0.322749862	0.098781129
ENSMUSG00000030663	1110004F10Rik	56372	protein_coding	477.0475237	0.098810296	0.069615231	-1.419377564	0.15578897	0.338423974	0.098810296
ENSMUSG00000061313	Ddhd2	72108	protein_coding	206.6101282	-0.098815645	0.077462803	1.275652846	0.202078264	0.404039964	0.098815645
ENSMUSG00000035769	Xylb	102448	protein_coding	60.77354562	-0.098827827	0.141652954	0.697675721	0.485380015	0.695380179	0.098827827
ENSMUSG00000022335	Zfat	380993	protein_coding	83.57723578	0.098828226	0.154845509	-0.638237596	0.523319027	0.722098569	0.098828226
ENSMUSG00000078671	Chd2	244059	protein_coding	964.7212595	0.098837354	0.102600798	-0.963319544	0.335387188	0.557762742	0.098837354
ENSMUSG00000021550	2210016F16Rik	70153	protein_coding	896.8275867	-0.098867361	0.100701459	0.981786779	0.326204903	0.548178261	0.098867361
ENSMUSG00000116114	Gm35853	102639573	lncRNA	4.03156485	-0.098877487	0.603194123	0.16392316	0.869791635	1	0.098877487
ENSMUSG00000027649	Ctnnbl1	66642	protein_coding	342.8706471	0.098925557	0.073470652	-1.346463581	0.178153058	0.37057407	0.098925557
ENSMUSG00000073733	Cplane2	76166	protein_coding	6.872035166	0.098935843	0.402231534	-0.245967395	0.805707472	0.904271659	0.098935843
ENSMUSG00000051716	Apon	28194	protein_coding	1.317633634	-0.098936224	1.162926148	0.085075242	0.932201573	1	0.098936224
ENSMUSG00000097727	F630040K05Rik	433111	lncRNA	5.42108129	-0.098940547	0.918992986	0.107661917	0.91426388	0.95803327	0.098940547
ENSMUSG00000007833	Aldh16a1	69748	protein_coding	1073.283288	-0.09894586	0.063939259	1.547497763	0.121743264	0.28456734	0.09894586
ENSMUSG00000005683	Cs	12974	protein_coding	7001.289517	-0.099126372	0.078770859	1.258414259	0.208241978	0.41130117	0.099126372
ENSMUSG00000024399	Ltb	16994	protein_coding	10.49663326	0.09917413	0.551156303	-0.179938302	0.857201005	0.930374388	0.09917413
ENSMUSG00000020955	Ap4s1	11782	protein_coding	110.1639707	-0.099208014	0.108067254	0.918021046	0.358607847	0.581328932	0.099208014
ENSMUSG00000028470	Hint2	68917	protein_coding	324.3859735	-0.099208085	0.063107702	1.572043998	0.115940348	0.274894315	0.099208085
ENSMUSG00000026455	Klhl12	240756	protein_coding	255.0302668	-0.099216367	0.078541877	1.263228868	0.206506941	0.409247045	0.099216367
ENSMUSG00000067199	Frat1	14296	protein_coding	253.5171458	-0.0992342	0.084051636	1.180633774	0.237748241	0.447999484	0.0992342
ENSMUSG00000019814	Ltv1	353258	protein_coding	281.4381197	0.099236674	0.106083286	-0.93546003	0.349551273	0.571420483	0.099236674
ENSMUSG00000038895	Zfp653	319601	protein_coding	47.33391911	0.099307603	0.16964384	-0.585388792	0.558286323	0.747162726	0.099307603
ENSMUSG00000024750	Zfand5	22682	protein_coding	1548.089782	0.099331477	0.141111992	-0.703919459	0.481482933	0.692354022	0.099331477

ensembl_gene_id	external_gene_name	entrezgene_id	gene_biotype	baseMean	log2FoldChange	lfcSE	stat	pvalue	padj	absLog2FC
ENSMUSG00000038569	Rad9b	231724	protein_coding	30.16659814	-0.099349938	0.239308456	0.415154312	0.678028942	0.827422396	0.099349938
ENSMUSG00000031982	Arv1	68865	protein_coding	28.50481231	0.099370991	0.208486877	-0.476629477	0.633625989	0.800362194	0.099370991
ENSMUSG00000036748	Cuedc2	67116	protein_coding	228.7380023	-0.099377048	0.076794914	1.294057688	0.195645495	0.394964884	0.099377048
ENSMUSG00000015149	Sirt2	64383	protein_coding	395.4592805	-0.099390893	0.066649914	1.491238129	0.135898986	0.307285909	0.099390893
ENSMUSG00000050711	Scg2	20254	protein_coding	15.0753977	0.099424998	0.309108091	-0.321651231	0.747716928	0.873111243	0.099424998
ENSMUSG00000054256	Msi1	17690	protein_coding	17.537195	0.099439838	0.36682872	-0.271079751	0.786329695	0.893161222	0.099439838
ENSMUSG00000035285	Nat14	269854	protein_coding	15.86673525	0.099525931	0.366046721	-0.271894065	0.785703475	0.892925964	0.099525931
ENSMUSG00000035085	1700020L24Rik	66330	protein_coding	25.89045284	-0.09953166	0.255985853	0.388817033	0.6974115	0.839586229	0.09953166
ENSMUSG00000074715	Ccl28	56838	protein_coding	1687.535385	-0.099565611	0.178633009	0.557375211	0.577271099	0.761298159	0.099565611
ENSMUSG00000038241	Cep250	16328	protein_coding	251.9515672	0.099576157	0.120174873	-0.828593821	0.407334285	0.628183969	0.099576157
ENSMUSG00000029575	Mmab	77697	protein_coding	206.2814438	-0.099655163	0.133637404	0.745713101	0.455840743	0.671162093	0.099655163
ENSMUSG00000028037	Ifi44	99899	protein_coding	41.73573182	-0.099655356	0.557131804	0.178872137	0.858038101	0.930446023	0.099655356
ENSMUSG00000041429	Nthl1	18207	protein_coding	26.5466328	-0.099678024	0.1872879	0.53221817	0.5945749	0.773317311	0.099678024
ENSMUSG00000037608	Bclaf1	72567	protein_coding	1574.430206	0.099711735	0.082145798	-1.213838544	0.224809385	0.432138537	0.099711735
ENSMUSG00000046798	Cldn12	64945	protein_coding	942.0426725	0.099776418	0.13395902	-0.744827917	0.456375756	0.671470886	0.099776418
ENSMUSG00000038872	Zfhx3	11906	protein_coding	336.9483217	0.099804867	0.122126317	-0.817226539	0.413798979	0.633873724	0.099804867
ENSMUSG00000044229	Nxpe4	244853	protein_coding	625.9074155	0.099812006	0.299259978	-0.333529417	0.738734688	0.867548331	0.099812006
ENSMUSG00000024188	Luc7l	66978	protein_coding	477.6278202	0.099822191	0.08664082	-1.152138109	0.249264325	0.461422606	0.099822191
ENSMUSG00000022420	Dnal4	54152	protein_coding	174.4436091	0.099833088	0.08326396	-1.198995192	0.230529815	0.43912271	0.099833088
ENSMUSG00000071669	Snx29	74478	protein_coding	89.13755821	-0.099860303	0.242044834	0.412569447	0.67992209	0.828297243	0.099860303
ENSMUSG00000043866	Taf10	24075	protein_coding	734.0956585	0.099874568	0.092698174	-1.077416778	0.281294112	0.499023496	0.099874568
ENSMUSG00000026709	Dars2	226539	protein_coding	310.5499348	-0.09991662	0.124137146	0.804888973	0.420883752	0.640714813	0.09991662
ENSMUSG00000005625	Psmd4	19185	protein_coding	1084.441408	0.099933448	0.052462458	-1.904856368	0.056798744	0.164036653	0.099933448
ENSMUSG00000032216	Nedd4	17999	protein_coding	4438.321901	0.099940143	0.10720429	-0.93224015	0.35121243	0.573211901	0.099940143
ENSMUSG00000033373	Fntb	110606	protein_coding	192.4461214	0.099987792	0.081034118	-1.233897452	0.217241119	0.422906972	0.099987792
ENSMUSG00000024740	Ddb1	13194	protein_coding	4802.556638	0.099989581	0.107413286	-0.930886529	0.351912264	0.574143363	0.099989581
ENSMUSG00000112449	Srp54b	665155	protein_coding	0.559318665	-0.099995834	1.765006635	0.05665465	0.9548203	1	0.099995834
ENSMUSG00000027198	Ext2	14043	protein_coding	580.1368562	0.100006854	0.063734646	-1.569112874	0.116621641	0.276057789	0.100006854
ENSMUSG00000054199	Gon4l	76022	protein_coding	335.3622922	0.100007581	0.06967341	-1.435376577	0.151179817	0.33115579	0.100007581
ENSMUSG00000063077	Kif1b	16561	protein_coding	1115.183368	-0.100018059	0.063165619	1.583425616	0.113324488	0.270127107	0.100018059
ENSMUSG00000057191	AB124611	382062	protein_coding	2.161476846	0.100065706	0.74274581	-0.134724026	0.892830077	1	0.100065706
ENSMUSG00000022244	Amacr	17117	protein_coding	136.4631411	-0.100125107	0.107830388	0.928542576	0.353126193	0.57545396	0.100125107
ENSMUSG00000014470	Rnf166	68718	protein_coding	238.2362781	0.100151062	0.085958222	-1.165113237	0.243973159	0.4551483	0.100151062
ENSMUSG00000028964	Park7	57320	protein_coding	803.6597066	0.100154769	0.084747819	-1.181797594	0.237286023	0.44767168	0.100154769
ENSMUSG00000053897	Slc39a8	67547	protein_coding	528.3273163	0.100159145	0.138066891	-0.725439272	0.468182588	0.681791678	0.100159145
ENSMUSG00000058812	0610039K10Rik	68386	lncRNA	19.83594179	0.10017729	0.3476896	-0.288122767	0.773252766	0.886362513	0.10017729
ENSMUSG00000097248	Gm2694	100040294	lncRNA	2.890023421	-0.100267185	1.322454346	0.075819015	0.939563088	1	0.100267185
ENSMUSG00000050856	Atp5k	11958	protein_coding	1964.017933	-0.100293981	0.10672752	0.939719957	0.347361226	0.569192626	0.100293981
ENSMUSG00000019428	Fkbp8	14232	protein_coding	3293.1903	0.100312008	0.057978734	-1.730151736	0.083603169	0.216806333	0.100312008

ensembl_gene_id	external_gene_name	entrezgene_id	gene_biotype	baseMean	log2FoldChange	lfcSE	stat	pvalue	padj	absLog2FC
ENSMUSG00000028621	Cyb5rl	230582	protein_coding	66.67876503	-0.100325673	0.158832464	0.631644631	0.527619116	0.725856654	0.100325673
ENSMUSG00000028830	AU040320	100317	protein_coding	1386.402599	-0.100353293	0.044627792	2.248672623	0.024533332	0.086109128	0.100353293
ENSMUSG00000046442	Ppm1e	320472	protein_coding	9.441679398	0.100369596	0.464697827	-0.21598895	0.828996361	0.913504709	0.100369596
ENSMUSG00000026885	Ttll11	74410	protein_coding	14.6043498	0.100379667	0.341077898	-0.294301296	0.768527682	0.884053089	0.100379667
ENSMUSG00000038481	Cdk19	78334	protein_coding	375.2294911	-0.100414931	0.103549038	0.969733107	0.332179543	0.554606509	0.100414931
ENSMUSG00000031220	Awat2	245532	protein_coding	6.83322058	0.100445412	0.396711748	-0.253194951	0.800117568	0.900998399	0.100445412
ENSMUSG00000050621	Rps27rt	100043813	protein_coding	20.93454735	0.100493595	0.372504166	-0.269778447	0.787330705	0.893606822	0.100493595
ENSMUSG00000027259	Adal	75894	protein_coding	93.69636391	0.100546845	0.130234094	-0.772047025	0.440086576	0.657938595	0.100546845
ENSMUSG00000053553	3110082I17Rik	73212	protein_coding	53.97363731	0.100549361	0.182945903	-0.549612533	0.582585161	0.765317902	0.100549361
ENSMUSG00000035268	Pkig	18769	protein_coding	294.0699519	0.100553072	0.118253644	-0.850316902	0.395148922	0.617417854	0.100553072
ENSMUSG00000025485	Ric8a	101489	protein_coding	671.8936772	-0.100565581	0.062738096	1.602942835	0.108947276	0.26324771	0.100565581
ENSMUSG00000030762	Aqp8	11833	protein_coding	2018.274923	-0.100575707	0.406865031	0.247196735	0.804755972	0.903803848	0.100575707
ENSMUSG00000032551	1110059G10Rik	66202	protein_coding	128.9935714	-0.100580158	0.101015059	0.99569469	0.319398511	0.540748207	0.100580158
ENSMUSG00000024975	Pdcd4	18569	protein_coding	2012.089842	0.100585091	0.154607226	-0.650584668	0.51531463	0.716335759	0.100585091
ENSMUSG00000078923	Ube2v1	66589	protein_coding	14.30578298	-0.100588826	0.331620894	0.303324753	0.761642384	0.880610679	0.100588826
ENSMUSG00000002846	Timmdc1	76916	protein_coding	281.8916255	-0.100661874	0.067504098	1.491196502	0.135909912	0.307285909	0.100661874
ENSMUSG00000023965	Fbxl17	50758	protein_coding	270.7417162	-0.100688418	0.074547483	1.350661528	0.176803882	0.368933447	0.100688418
ENSMUSG00000079043	Fastkd5	380601	protein_coding	62.55686403	-0.10071539	0.168288385	0.598469048	0.549527006	0.740505016	0.10071539
ENSMUSG00000054051	Ercc6	319955	protein_coding	336.5859435	-0.10076437	0.073520667	1.370558428	0.17051265	0.360359086	0.10076437
ENSMUSG00000051341	Zfp52	22710	protein_coding	58.83800224	-0.100781574	0.171281712	0.588396582	0.556266132	0.745631404	0.100781574
ENSMUSG00000055013	Agap1	347722	protein_coding	826.5808074	-0.100786087	0.078059071	1.291151501	0.196651149	0.396326223	0.100786087
ENSMUSG00000062198	2700097O09Rik	72658	protein_coding	61.85161149	0.100823035	0.153451661	-0.657034495	0.511158736	0.714031075	0.100823035
ENSMUSG00000054951	9130008F23Rik	71583	protein_coding	200.3400827	0.100845033	0.14151809	-0.71259464	0.476096629	0.687930892	0.100845033
ENSMUSG00000033981	Gria2	14800	protein_coding	5.711257716	-0.100856517	1.61436547	0.062474402	0.950185046	0.975869522	0.100856517
ENSMUSG00000071645	Tut1	70044	protein_coding	249.5397104	0.100872213	0.082103802	-1.228593689	0.219224183	0.425500655	0.100872213
ENSMUSG00000025869	Nop16	28126	protein_coding	252.9412923	0.100878603	0.097758133	-1.031920313	0.302109449	0.522421155	0.100878603
ENSMUSG00000020628	Trappc12	217449	protein_coding	497.8466927	-0.100933818	0.067894043	1.486637302	0.13711063	0.309427758	0.100933818
ENSMUSG00000067656	Slc22a27	171405	protein_coding	2.222976911	-0.10099841	0.719400543	0.140392458	0.888349917	1	0.10099841
ENSMUSG00000062202	Btbd9	224671	protein_coding	459.8879082	-0.101010594	0.065096798	1.551698342	0.12073442	0.283211393	0.101010594
ENSMUSG00000078584	AU022252	230696	protein_coding	209.6983516	0.101110758	0.085233671	-1.186277166	0.235512861	0.445505084	0.101110758
ENSMUSG00000031667	Aktip	14339	protein_coding	508.1421673	-0.101125659	0.060311293	1.676728429	0.093595599	0.235436057	0.101125659
ENSMUSG00000052684	Jun	16476	protein_coding	10893.12234	-0.101203975	0.115077918	0.879438696	0.379163459	0.60160505	0.101203975
ENSMUSG00000030887	Pdzd9	67983	protein_coding	7.046305552	-0.101210839	0.462309012	0.218924651	0.826708743	0.912523774	0.101210839
ENSMUSG00000054474	Thnsl2	232078	protein_coding	269.2060895	0.101218754	0.133396762	-0.758779691	0.447984356	0.664930223	0.101218754
ENSMUSG00000031446	Cul4a	99375	protein_coding	1068.338099	0.101256667	0.077301888	-1.309886073	0.190234379	0.387604015	0.101256667
ENSMUSG00000002342	Tmem161a	234371	protein_coding	343.8457682	-0.101295018	0.07706008	1.314494055	0.188679997	0.385300595	0.101295018
ENSMUSG00000066720	Cldn9	56863	protein_coding	3.692596347	-0.101316685	0.71910321	0.140893107	0.887954388	1	0.101316685
ENSMUSG00000056167	Cnot10	78893	protein_coding	481.3768611	0.101367657	0.053766672	-1.885325107	0.059385958	0.169583213	0.101367657
ENSMUSG00000025899	Alkbh8	67667	protein_coding	204.1690826	0.101444402	0.128137957	-0.791681127	0.42854663	0.647544976	0.101444402

ensembl_gene_id	external_gene_name	entrezgene_id	gene_biotype	baseMean	log2FoldChange	lfcSE	stat	pvalue	padj	absLog2FC
ENSMUSG00000024095	Hnrnpll	72692	protein_coding	747.8826423	0.101453637	0.060849297	-1.667293497	0.095456059	0.238987471	0.101453637
ENSMUSG00000024773	Atg2a	329015	protein_coding	705.5939272	-0.101460874	0.121681066	0.83382631	0.404378867	0.62546825	0.101460874
ENSMUSG00000019797	Mtres1	67851	protein_coding	295.271451	-0.101565692	0.085753437	1.184392084	0.236257892	0.44640307	0.101565692
ENSMUSG00000102428	Pcdhga12	93724	protein_coding	6.017960096	-0.101596661	0.414898887	0.244870893	0.806556399	0.904855461	0.101596661
ENSMUSG00000014748	Tex261	21766	protein_coding	877.6581805	-0.101597236	0.066159871	1.535632319	0.124628583	0.289400593	0.101597236
ENSMUSG00000037580	Gch1	14528	protein_coding	264.9180874	-0.101646069	0.140528039	0.72331522	0.469486244	0.683156904	0.101646069
ENSMUSG00000018841	Rad51d	19364	protein_coding	263.2998261	-0.101662923	0.095961413	1.05941461	0.289410999	0.50827749	0.101662923
ENSMUSG00000037960	Card19	68480	protein_coding	387.1994243	-0.101672773	0.116321773	0.87406485	0.382082938	0.604440039	0.101672773
ENSMUSG00000058325	Dock1	330662	protein_coding	1451.907829	-0.101691332	0.045831768	2.21879573	0.026500625	0.091363531	0.101691332
ENSMUSG00000020289	Nprl3	17168	protein_coding	141.3475538	-0.101727108	0.119401351	0.851976185	0.39422731	0.616875898	0.101727108
ENSMUSG00000013973	Dedd	21945	protein_coding	103.0458816	-0.10179726	0.124704086	0.816310536	0.41432255	0.634528665	0.10179726
ENSMUSG00000049299	Trappc1	245828	protein_coding	332.487863	0.101828932	0.085984565	-1.1842699	0.23630624	0.446443342	0.101828932
ENSMUSG00000092417	Gpank1	81845	protein_coding	117.8317273	-0.101833678	0.10622241	0.958683554	0.337718186	0.559610488	0.101833678
ENSMUSG00000098766	Mir6386	102465203	miRNA	7.980301981	-0.101841474	0.402365684	0.25310676	0.800185715	0.901013699	0.101841474
ENSMUSG00000050106	Tmc8	217356	protein_coding	142.5382406	0.10184174	0.139896984	-0.727976665	0.466627871	0.68031188	0.10184174
ENSMUSG00000024997	Prdx3	11757	protein_coding	1370.966002	-0.10185792	0.067257074	1.514456598	0.129910074	0.29771794	0.10185792
ENSMUSG00000048027	Rgmb	68799	protein_coding	795.9202801	0.101865072	0.106241009	-0.958811224	0.337653854	0.559563944	0.101865072
ENSMUSG00000068860	Gm128	229588	protein_coding	3.57693077	-0.101869059	0.606077677	0.168079214	0.866520962	1	0.101869059
ENSMUSG00000043411	Usp48	170707	protein_coding	798.9166092	0.101886064	0.049336128	-2.065141057	0.038909656	0.123307245	0.101886064
ENSMUSG00000026171	Rnf25	57751	protein_coding	212.0772359	-0.101923733	0.080018425	1.273753302	0.20275086	0.404864292	0.101923733
ENSMUSG00000026848	Tor1b	30934	protein_coding	461.8046635	-0.10201792	0.067789699	1.504917744	0.132345226	0.301713259	0.10201792
ENSMUSG00000025264	Tsr2	69499	protein_coding	115.3163958	0.102020548	0.155528224	-0.655961631	0.511848812	0.714390744	0.102020548
ENSMUSG00000046897	Zfp740	68744	protein_coding	853.0021571	-0.102040634	0.077494054	1.316754366	0.187920974	0.384217774	0.102040634
ENSMUSG00000003923	Tfam	21780	protein_coding	453.3928952	-0.102042716	0.087742669	1.16297711	0.24483879	0.456505337	0.102042716
ENSMUSG00000051832	E230016K23Rik	100504464	lncRNA	2.122951362	-0.10204974	0.906372257	0.112591421	0.910354487	1	0.10204974
ENSMUSG00000026672	Optn	71648	protein_coding	493.352055	0.102084671	0.153883989	-0.663387219	0.50708259	0.710854454	0.102084671
ENSMUSG00000075419	Dolk	227697	protein_coding	194.7713663	0.102119786	0.077531649	-1.317136785	0.187792779	0.384098334	0.102119786
ENSMUSG00000028567	Txndc12	66073	protein_coding	356.2975786	-0.102127446	0.107075444	0.953789614	0.340190148	0.561689026	0.102127446
ENSMUSG00000064351	mt-Co1	17708	protein_coding	639120.1894	-0.102183676	0.436549577	0.234071183	0.814929717	0.908068261	0.102183676
ENSMUSG00000028187	Rpf1	70285	protein_coding	317.2765411	-0.102185402	0.096396667	1.060051195	0.289121309	0.508005086	0.102185402
ENSMUSG00000024392	Bag6	224727	protein_coding	1428.854144	-0.102197466	0.081621205	1.252094546	0.210535415	0.414593589	0.102197466
ENSMUSG00000039046	Usp6nl	98910	protein_coding	584.9452705	-0.102202882	0.068343461	1.495430283	0.134802182	0.3056602	0.102202882
ENSMUSG00000026383	Epb41l5	226352	protein_coding	220.010906	-0.102219515	0.101660236	1.005501448	0.314655453	0.535858528	0.102219515
ENSMUSG00000037957	Wdr20	69641	protein_coding	324.1455388	0.10224128	0.075614148	-1.352144831	0.176328988	0.368268232	0.10224128
ENSMUSG00000029176	Anapc4	52206	protein_coding	412.4802661	0.102258674	0.094138488	-1.086257878	0.277364917	0.494297255	0.102258674
ENSMUSG00000021066	Atl1	73991	protein_coding	24.87899126	-0.102269651	0.27372099	0.37362736	0.708681575	0.847687059	0.102269651
ENSMUSG00000026857	Ntmt1	66617	protein_coding	207.367212	0.102344118	0.073015583	-1.401675006	0.161012317	0.345858144	0.102344118
ENSMUSG00000030172	Erc1	111173	protein_coding	391.8836755	0.102368436	0.075932412	-1.348152033	0.177609489	0.370029503	0.102368436
ENSMUSG00000056185	Snx32	225861	protein_coding	39.63488868	-0.102368693	0.270733269	0.378116413	0.705344116	0.845574994	0.102368693

ensembl_gene_id	external_gene_name	entrezgene_id	gene_biotype	baseMean	log2FoldChange	lfcSE	stat	pvalue	padj	absLog2FC
ENSMUSG00000035027	Map2k2	26396	protein_coding	1352.38048	0.102404195	0.074364454	-1.377058384	0.168494198	0.357236254	0.102404195
ENSMUSG00000056851	Pcbp2	18521	protein_coding	755.7165134	0.102406927	0.150921871	-0.678542655	0.497427688	0.702997077	0.102406927
ENSMUSG00000022323	Rida	15473	protein_coding	198.9566846	-0.102408367	0.111672067	0.917045498	0.359118799	0.581819665	0.102408367
ENSMUSG00000000278	Scpep1	74617	protein_coding	361.358767	-0.1024139	0.083650213	1.22431129	0.22083482	0.427333749	0.1024139
ENSMUSG00000046791	Riox1	71952	protein_coding	148.5930353	-0.10247479	0.100049266	1.024243297	0.305720373	0.526067129	0.10247479
ENSMUSG00000003039	Fam32a	67922	protein_coding	1145.541053	-0.102547448	0.092952545	1.103223672	0.269930044	0.485210052	0.102547448
ENSMUSG00000040167	Ikzf5	67143	protein_coding	31.0312011	-0.102550694	0.197096216	0.520307776	0.602849077	0.778864783	0.102550694
ENSMUSG00000040618	Pck2	74551	protein_coding	447.598437	0.102563503	0.108137299	-0.948456301	0.342897209	0.564917636	0.102563503
ENSMUSG00000056632	Dsg3	13512	protein_coding	8.400734732	-0.102571717	0.467217662	0.219537327	0.826231506	0.912240897	0.102571717
ENSMUSG00000109186	Gm34821	102638204	lncRNA	8.573993317	0.102600288	0.389729649	-0.263260157	0.792350067	0.896636371	0.102600288
ENSMUSG00000036281	Snapc4	227644	protein_coding	155.0726039	0.102614407	0.099670683	-1.02953451	0.303228572	0.523689253	0.102614407
ENSMUSG00000031858	Mau2	74549	protein_coding	1226.329803	-0.102641446	0.051305204	2.000604997	0.045434975	0.138818348	0.102641446
ENSMUSG00000019528	Gyg	27357	protein_coding	234.6494006	-0.102658406	0.121945579	0.841837861	0.399878716	0.622046705	0.102658406
ENSMUSG00000036298	Slc2a13	239606	protein_coding	568.8871784	0.102665484	0.101423012	-1.012250391	0.311418348	0.53237708	0.102665484
ENSMUSG00000041515	Irf8	15900	protein_coding	1077.247636	0.102710968	0.101865175	-1.00830307	0.31330899	0.534596554	0.102710968
ENSMUSG00000034290	Nek9	217718	protein_coding	781.7891733	-0.102722942	0.051902531	1.979150907	0.047799018	0.144095106	0.102722942
ENSMUSG00000025658	Cnksr2	245684	protein_coding	2.280681024	0.102733846	2.097870843	-0.04897053	0.960942782	1	0.102733846
ENSMUSG00000028677	Rnf220	66743	protein_coding	500.2995915	0.102746836	0.079457004	-1.293112387	0.195972192	0.395392153	0.102746836
ENSMUSG00000024012	Mtch1	56462	protein_coding	1693.823553	-0.102757997	0.076858965	1.336968262	0.181232997	0.375235922	0.102757997
ENSMUSG00000058006	Mdn1	100019	protein_coding	496.0830249	0.102797169	0.12609278	-0.815250239	0.414929086	0.635253445	0.102797169
ENSMUSG00000083394	Gm11703	108167871	processed_pseudogene	0.641152516	-0.102873761	1.443491784	0.0712673	0.94318502	1	0.102873761
ENSMUSG00000030801	Kat8	67773	protein_coding	208.0402352	0.102874521	0.085596169	-1.201858938	0.229418186	0.438111017	0.102874521
ENSMUSG00000027710	Acad9	229211	protein_coding	516.6957296	-0.102935359	0.104300378	0.986912619	0.323685478	0.545498722	0.102935359
ENSMUSG00000038637	Lrrc56	70552	protein_coding	101.5752832	0.102938429	0.122007381	-0.843706571	0.398833395	0.620766699	0.102938429
ENSMUSG00000034832	Tet3	194388	protein_coding	1396.022975	-0.102941168	0.111999569	0.919121112	0.358032228	0.580790426	0.102941168
ENSMUSG00000029434	Vps33a	77573	protein_coding	388.3946719	0.102972337	0.073419588	-1.402518595	0.160760442	0.345496868	0.102972337
ENSMUSG00000033467	Crlf2	57914	protein_coding	38.62118195	-0.102987395	0.209601024	0.491349673	0.623179153	0.793304622	0.102987395
ENSMUSG00000042644	Itpr3	16440	protein_coding	2871.248831	-0.103016924	0.057286745	1.798268061	0.072134539	0.195389167	0.103016924
ENSMUSG00000022433	Csnk1e	27373	protein_coding	164.3559332	0.103046206	0.135935468	-0.758052382	0.448419625	0.665277305	0.103046206
ENSMUSG00000036813	Entpd8	72090	protein_coding	1379.528626	-0.103135126	0.097724602	1.05536501	0.291258419	0.510066755	0.103135126
ENSMUSG00000097404	Gm10814	100502985	lncRNA	7.960126162	-0.103177514	0.570580657	0.180828972	0.856501822	0.930184975	0.103177514
ENSMUSG00000073406	H2-Bl	14963	protein_coding	11.23352825	0.10319093	0.55106271	-0.187258053	0.85145831	0.927499565	0.10319093
ENSMUSG00000022500	Litaf	56722	protein_coding	1050.220678	-0.103205542	0.065650418	1.572046982	0.115939656	0.274894315	0.103205542
ENSMUSG00000043822	Adamtsl5	66548	protein_coding	70.51789367	-0.103216041	0.278466809	0.370658327	0.710892033	0.849223889	0.103216041
ENSMUSG00000064138	Fam172a	68675	protein_coding	174.0886253	-0.103222116	0.087655523	1.177588268	0.238960789	0.449146195	0.103222116
ENSMUSG00000030610	Det1	76375	protein_coding	150.1344388	0.103246041	0.100214318	-1.030252391	0.302891542	0.523380864	0.103246041
ENSMUSG00000041408	Wapl	218914	protein_coding	2069.195697	0.103252645	0.063066182	-1.637210955	0.101586399	0.250425106	0.103252645
ENSMUSG00000039232	Stx11	74732	protein_coding	3.709874668	-0.103268329	0.815885045	0.126572156	0.899279036	1	0.103268329
ENSMUSG00000024899	Papss2	23972	protein_coding	6410.762028	-0.103269891	0.135663573	0.761220484	0.446525385	0.663778937	0.103269891

ensembl_gene_id	external_gene_name	entrezgene_id	gene_biotype	baseMean	log2FoldChange	lfcSE	stat	pvalue	padj	absLog2FC
ENSMUSG00000075232	Amd1	11702	protein_coding	402.6814976	-0.103296923	0.153069422	0.674837088	0.499779274	0.704451599	0.103296923
ENSMUSG00000020454	Eif4enif1	74203	protein_coding	413.038094	0.103353276	0.058175032	-1.77659164	0.075635452	0.20160769	0.103353276
ENSMUSG00000030879	Mrpl17	27397	protein_coding	471.2054793	0.10337485	0.097959103	-1.055285802	0.291294633	0.510066755	0.10337485
ENSMUSG00000030911	Zp2	22787	protein_coding	5.418931895	-0.103404622	0.658441335	0.157044548	0.875209739	0.938415991	0.103404622
ENSMUSG00000046456	Tmem150b	330460	protein_coding	199.6146188	-0.103410617	0.123680629	0.836110053	0.403092994	0.624336681	0.103410617
ENSMUSG00000039997	Ifi203	15950	protein_coding	6.048377488	0.103437671	0.735304645	-0.140673218	0.888128104	0.94482687	0.103437671
ENSMUSG00000040048	Ndufb10	68342	protein_coding	1503.821282	-0.103524619	0.083658642	1.237464733	0.215914599	0.421168638	0.103524619
ENSMUSG00000057058	Skap1	78473	protein_coding	9.003785644	0.103527451	0.533825431	-0.19393503	0.846226754	0.924222269	0.103527451
ENSMUSG00000052459	Atp6v1a	11964	protein_coding	991.831285	-0.103531983	0.089905987	1.151558262	0.249502641	0.461760239	0.103531983
ENSMUSG00000015461	Atf6b	12915	protein_coding	455.1214415	0.103588122	0.080246683	-1.290871077	0.196748386	0.396473807	0.103588122
ENSMUSG00000030403	Vasp	22323	protein_coding	4728.198015	-0.103645905	0.079460269	1.304373957	0.19210611	0.389807821	0.103645905
ENSMUSG00000080365	Gm25776	115488078	snoRNA	122.5279008	-0.103657309	0.287670418	0.360333572	0.718597697	0.854165577	0.103657309
ENSMUSG00000031823	Zdhhc7	102193	protein_coding	933.3012635	0.103805042	0.069577009	-1.491944599	0.13571367	0.30705241	0.103805042
ENSMUSG00000038522	Mfsd4b1	215929	protein_coding	1.340139256	0.103830259	1.100828384	-0.094320114	0.924854872	1	0.103830259
ENSMUSG00000057554	Lgals8	56048	protein_coding	644.1824923	0.103838861	0.082433046	-1.259675172	0.207786565	0.410708835	0.103838861
ENSMUSG00000050796	B3galt6	117592	protein_coding	92.95029415	0.103858599	0.143944842	-0.721516644	0.470591704	0.683821668	0.103858599
ENSMUSG00000024789	Jak2	16452	protein_coding	916.3442459	-0.103872877	0.069926072	1.485467065	0.137420142	0.309858682	0.103872877
ENSMUSG00000037408	Cnnm4	94220	protein_coding	6137.144141	-0.103887441	0.088901785	1.168564175	0.242579269	0.45367543	0.103887441
ENSMUSG00000038975	Rabggtb	19352	protein_coding	440.8189274	-0.103933569	0.089181431	1.165417141	0.243850182	0.455021685	0.103933569
ENSMUSG00000038324	Trpc4ap	56407	protein_coding	1159.503051	0.103950863	0.056693883	-1.833546384	0.066721357	0.184376923	0.103950863
ENSMUSG00000098112	Bin2	668218	protein_coding	16.29595073	0.104052263	0.358430312	-0.290299843	0.771586859	0.885228942	0.104052263
ENSMUSG00000029551	Psmg3	66506	protein_coding	98.5049566	0.104070987	0.117949524	-0.882334951	0.377595701	0.599693731	0.104070987
ENSMUSG00000029580	Actb	11461	protein_coding	54482.32294	0.104087843	0.109089955	-0.954146903	0.340009287	0.561491337	0.104087843
ENSMUSG00000085049	Parvaos	102636183	lncRNA	2.01205628	0.104097177	1.091989858	-0.095327971	0.924054326	1	0.104097177
ENSMUSG00000033767	Tmem131l	229473	protein_coding	196.2909425	-0.104100214	0.101835227	1.02224168	0.306666528	0.527150555	0.104100214
ENSMUSG00000041308	Sntb2	20650	protein_coding	169.8832226	0.10412869	0.115171589	-0.904117854	0.365932912	0.588586355	0.10412869
ENSMUSG00000090150	Acad11	102632	protein_coding	1469.791732	-0.104148292	0.088212286	1.180655176	0.237739735	0.447999484	0.104148292
ENSMUSG00000011096	Akt1s1	67605	protein_coding	545.6327809	0.104157795	0.081076974	-1.284677868	0.198904875	0.39947392	0.104157795
ENSMUSG00000029605	Oas1b	23961	protein_coding	19.87747968	0.104181109	0.40149335	-0.25948402	0.79526181	0.898286835	0.104181109
ENSMUSG00000038871	Bpgm	12183	protein_coding	575.2319686	0.104217551	0.073977611	-1.408771526	0.158902737	0.342932541	0.104217551
ENSMUSG00000032705	Exd2	97827	protein_coding	246.3547716	0.104264937	0.099610447	-1.046726929	0.29522554	0.514332163	0.104264937
ENSMUSG00000030244	Gys2	232493	protein_coding	1.698799735	0.104299873	1.286980721	-0.081042296	0.935408316	1	0.104299873
ENSMUSG00000042572	Ube2q1	70093	protein_coding	828.7091037	-0.10432357	0.054946596	1.898635727	0.057612388	0.16577019	0.10432357
ENSMUSG00000028256	Odf2l	52184	protein_coding	157.840942	0.104334504	0.087883062	-1.187196957	0.235149941	0.445073481	0.104334504
ENSMUSG00000021810	Ecd	70601	protein_coding	492.5904637	0.104360966	0.077060007	-1.354281817	0.175646485	0.367540047	0.104360966
ENSMUSG00000025139	Tollip	54473	protein_coding	685.0697994	-0.104375461	0.052468478	1.989298434	0.046668272	0.141461059	0.104375461
ENSMUSG00000014980	Tsen15	66637	protein_coding	59.83425618	0.104392778	0.177758638	-0.587272604	0.557020637	0.746218772	0.104392778
ENSMUSG00000051934	Spats2	72572	protein_coding	154.4600214	0.104433152	0.108679578	-0.960927104	0.336588821	0.559203284	0.104433152
ENSMUSG00000039221	Rpl22l1	68028	protein_coding	565.2474978	0.104483562	0.128822268	-0.811067552	0.417326878	0.637415471	0.104483562

ensembl_gene_id	external_gene_name	entrezgene_id	gene_biotype	baseMean	log2FoldChange	lfcSE	stat	pvalue	padj	absLog2FC
ENSMUSG00000020023	Tmcc3	319880	protein_coding	1496.282863	0.104556564	0.173950968	-0.601069173	0.547793914	0.739162904	0.104556564
ENSMUSG00000029145	Eif2b4	13667	protein_coding	358.870762	-0.104578941	0.091736034	1.139998502	0.254286925	0.466951438	0.104578941
ENSMUSG00000028587	Orc1	18392	protein_coding	129.3687287	0.104580809	0.115627016	-0.904466903	0.365747876	0.588390676	0.104580809
ENSMUSG00000037217	Syn1	20964	protein_coding	5.550659735	-0.104615605	0.521213663	0.200715393	0.840921123	0.921253246	0.104615605
ENSMUSG00000047719	Ubiad1	71707	protein_coding	229.8652062	0.104618749	0.103551095	-1.010310408	0.312346596	0.533456622	0.104618749
ENSMUSG00000019088	Dnase1l1	69537	protein_coding	153.1493468	-0.104649825	0.119207939	0.877876302	0.380010851	0.60223179	0.104649825
ENSMUSG00000103409	Lsmem2	434436	protein_coding	9.973412545	-0.104708918	0.38702323	0.27054944	0.786737587	0.893302015	0.104708918
ENSMUSG00000009640	Dmap1	66233	protein_coding	209.3706332	-0.104754458	0.096312179	1.087655369	0.276747277	0.493649227	0.104754458
ENSMUSG00000045827	Serpinb9	20723	protein_coding	36.60481177	-0.104772317	0.177744906	0.589453277	0.555557246	0.7452256	0.104772317
ENSMUSG00000084803	5830444B04Rik	641454	lncRNA	44.05052523	-0.104915572	0.358304693	0.292811047	0.769666584	0.884510137	0.104915572
ENSMUSG00000051295	9630028B13Rik	319562	lncRNA	30.65846421	0.104929082	0.321710081	-0.326160379	0.744303017	0.870990647	0.104929082
ENSMUSG00000088951	Gm24940	115487682	snRNA	0.760793376	0.104947673	1.61978264	-0.064791208	0.948340242	1	0.104947673
ENSMUSG00000045767	B230219D22Rik	78521	protein_coding	587.3948086	0.10499056	0.094623896	-1.109556504	0.267190183	0.482454733	0.10499056
ENSMUSG00000020137	Thap2	66816	protein_coding	228.274071	0.104995771	0.127351816	-0.824454449	0.409681388	0.630279329	0.104995771
ENSMUSG00000026765	Lypd6b	71897	protein_coding	58.5044114	-0.10512821	0.276828551	0.379759275	0.704124115	0.844376272	0.10512821
ENSMUSG00000003970	Rpl8	26961	protein_coding	8930.751957	-0.105173886	0.088247823	1.191801487	0.233339093	0.442718029	0.105173886
ENSMUSG00000018196	Glrx2	69367	protein_coding	207.7533573	0.105193699	0.096489788	-1.090205512	0.275622626	0.492426209	0.105193699
ENSMUSG00000062960	Kdr	16542	protein_coding	3.086564233	-0.105236747	2.428591127	0.043332427	0.965436543	1	0.105236747
ENSMUSG00000049940	Pgrmc2	70804	protein_coding	375.9280373	0.105258194	0.081814893	-1.286540745	0.198254408	0.3984875	0.105258194
ENSMUSG00000024431	Nr3c1	14815	protein_coding	1489.699065	-0.105259843	0.05767575	1.825027735	0.067996844	0.186963018	0.105259843
ENSMUSG00000038803	Ost4	67695	protein_coding	393.2972045	0.105290383	0.05706754	-1.845013534	0.06503557	0.181112546	0.105290383
ENSMUSG00000028127	Abcd3	19299	protein_coding	2944.723536	0.105321402	0.064859821	-1.62383122	0.104411835	0.255483337	0.105321402
ENSMUSG00000025332	Kdm5c	20591	protein_coding	2456.467971	0.105344485	0.064919844	-1.622685436	0.104656666	0.255796319	0.105344485
ENSMUSG00000044496	2510039O18Rik	77034	protein_coding	1362.549504	0.105377236	0.065278769	-1.614265064	0.106469944	0.259060655	0.105377236
ENSMUSG00000042978	Sbk1	104175	protein_coding	263.7722422	-0.105403075	0.107033121	0.984770639	0.324736746	0.546768212	0.105403075
ENSMUSG00000098332	Pigbos1	66364	protein_coding	85.91415225	-0.105426074	0.128492733	0.820482774	0.411940945	0.632229811	0.105426074
ENSMUSG00000102440	Pcdhga9	93717	protein_coding	11.45871078	-0.105464497	0.3029474	0.348128082	0.727743996	0.859322531	0.105464497
ENSMUSG00000056820	Tsnax	53424	protein_coding	585.5018824	-0.105471187	0.095239921	1.107426225	0.268109692	0.48334534	0.105471187
ENSMUSG00000058297	Spock2	94214	protein_coding	14.09051112	-0.10548779	0.455786338	0.231441316	0.816971971	0.908700419	0.10548779
ENSMUSG00000021282	Eif5	217869	protein_coding	2591.574461	0.105506508	0.06805499	-1.550312591	0.121066507	0.283788828	0.105506508
ENSMUSG00000036504	Phpt1	75454	protein_coding	17.78360501	0.105522907	0.262108622	-0.402592278	0.687248191	0.833389267	0.105522907
ENSMUSG00000030650	Tmc5	74424	protein_coding	1653.523475	-0.105545448	0.115245646	0.915830245	0.359755938	0.582349713	0.105545448
ENSMUSG00000071291	Zfp58	238693	protein_coding	33.00017572	-0.105581036	0.177281881	0.59555458	0.551472827	0.742339604	0.105581036
ENSMUSG00000003882	Il7r	16197	protein_coding	2.494114433	-0.105611954	0.818417381	0.129044124	0.897322735	1	0.105611954
ENSMUSG00000042203	Tbc1d22b	381085	protein_coding	282.2710126	0.105614135	0.084339871	-1.252244435	0.210480809	0.414545055	0.105614135
ENSMUSG00000043183	Simc1	319719	protein_coding	55.46419195	0.105654242	0.19984997	-0.52866779	0.597035925	0.774998778	0.105654242
ENSMUSG00000071424	Grid2	14804	protein_coding	27.74865513	0.105707909	0.298401897	-0.354246773	0.723153953	0.856549354	0.105707909
ENSMUSG00000048779	P2ry6	233571	protein_coding	173.6679412	-0.105777513	0.10669613	0.991390344	0.321495014	0.542471509	0.105777513
ENSMUSG00000002033	Cd3g	12502	protein_coding	21.8240925	-0.105789813	0.373147012	0.283507063	0.776788168	0.888506311	0.105789813

ensembl_gene_id	external_gene_name	entrezgene_id	gene_biotype	baseMean	log2FoldChange	lfcSE	stat	pvalue	padj	absLog2FC
ENSMUSG00000089857	Zfp882	382019	protein_coding	24.14027483	0.105800009	0.267535322	-0.395461835	0.692502082	0.836513744	0.105800009
ENSMUSG00000073147	5031425E22Rik	269630	lncRNA	205.1424808	-0.105862805	0.227183466	0.465979356	0.641230277	0.804106371	0.105862805
ENSMUSG00000062761	Zfp512	269639	protein_coding	182.843183	-0.105891186	0.086508382	1.224056945	0.220930746	0.427441699	0.105891186
ENSMUSG00000044465	Fhip1b	74349	protein_coding	732.3457401	-0.105906149	0.08187546	1.293502953	0.195837163	0.395168001	0.105906149
ENSMUSG00000028683	Eif2b3	108067	protein_coding	105.8057144	-0.10592928	0.15418689	0.687018717	0.492070933	0.698788598	0.10592928
ENSMUSG00000058997	Vwa8	219189	protein_coding	2526.719602	-0.105933378	0.084668489	1.251154695	0.210878044	0.415010021	0.105933378
ENSMUSG00000029064	Gnb1	14688	protein_coding	3513.604006	0.105957494	0.060762593	-1.743794816	0.081194875	0.211992437	0.105957494
ENSMUSG00000022704	Qtrt2	106248	protein_coding	166.0286267	-0.105962013	0.102809905	1.030659578	0.302700487	0.523269741	0.105962013
ENSMUSG00000087177	E130307A14Rik	327744	lncRNA	31.78932449	0.105969401	0.250520506	-0.422996914	0.672297513	0.824348168	0.105969401
ENSMUSG00000031803	B3gnt3	72297	protein_coding	3052.958989	0.105995821	0.068163055	-1.555033305	0.119938137	0.281984397	0.105995821
ENSMUSG00000055302	Mrfap1	67568	protein_coding	1484.797484	-0.105999115	0.100010432	1.05988058	0.289198932	0.508065109	0.105999115
ENSMUSG00000038530	Rgs4	19736	protein_coding	6.238012696	0.10600377	0.597616281	-0.177377648	0.859211763	0.931099354	0.10600377
ENSMUSG00000031023	Akip1	57373	protein_coding	98.42193533	-0.106011516	0.165938378	0.638860746	0.522913526	0.722022907	0.106011516
ENSMUSG00000021037	Ahsa1	217737	protein_coding	848.1617248	-0.106036018	0.117418201	0.90306287	0.366492529	0.589199534	0.106036018
ENSMUSG00000047878	A4galt	239559	protein_coding	170.5537055	-0.10606364	0.256362298	0.413725578	0.679075093	0.827951097	0.10606364
ENSMUSG00000030228	Pik3c2g	18705	protein_coding	18.78719684	-0.106086921	0.304692726	0.348176741	0.727707454	0.859322531	0.106086921
ENSMUSG00000052033	Pfdn4	109054	protein_coding	95.68201487	0.10610949	0.123306393	-0.860535187	0.389494096	0.612056856	0.10610949
ENSMUSG00000030780	Rusf1	233913	protein_coding	334.9807159	0.106135561	0.072808209	-1.457741672	0.144911761	0.321952485	0.106135561
ENSMUSG00000022255	Mtdh	67154	protein_coding	1453.468859	0.106193626	0.08519825	-1.246429659	0.212606698	0.417180016	0.106193626
ENSMUSG00000030126	Tmcc1	330401	protein_coding	426.3760262	-0.106256142	0.068991812	1.540126852	0.123529436	0.287399986	0.106256142
ENSMUSG00000092341	Malat1	72289	lncRNA	45152.54829	-0.106279632	0.145800247	0.728939998	0.466038367	0.679736277	0.106279632
ENSMUSG00000022545	Ercc4	50505	protein_coding	164.3916374	-0.10628205	0.082396592	1.289884053	0.197090918	0.397106786	0.10628205
ENSMUSG00000037257	Aagab	66939	protein_coding	761.6564489	-0.106290111	0.10505401	1.011766333	0.311649791	0.532495959	0.106290111
ENSMUSG00000019916	P4ha1	18451	protein_coding	222.5658689	0.106296831	0.098635779	-1.07767011	0.281181002	0.498917274	0.106296831
ENSMUSG00000024556	Me2	107029	protein_coding	2078.302351	-0.106304017	0.100134054	1.061617023	0.288409583	0.50724071	0.106304017
ENSMUSG00000056014	A430033K04Rik	243308	protein_coding	140.2643715	0.106329086	0.090683941	-1.172523885	0.240986788	0.45166903	0.106329086
ENSMUSG00000022175	Lrp10	65107	protein_coding	3466.077545	0.106347724	0.07092091	-1.499525646	0.133737321	0.303828141	0.106347724
ENSMUSG00000008036	Ap2s1	232910	protein_coding	1052.019843	0.10636781	0.065547712	-1.622753973	0.104642009	0.255796319	0.10636781
ENSMUSG00000043683	Fem1a	14154	protein_coding	789.0453671	-0.106408165	0.08471132	1.256126862	0.209069979	0.412396276	0.106408165
ENSMUSG00000040390	Map3k10	269881	protein_coding	134.3249288	0.106420932	0.116131292	-0.916384637	0.35946519	0.582152413	0.106420932
ENSMUSG00000002550	Uck1	22245	protein_coding	341.0699447	-0.106424583	0.105221061	1.011438032	0.311806826	0.532600117	0.106424583
ENSMUSG00000021456	Fbp2	14120	protein_coding	1616.048715	-0.106529392	0.076237213	1.397341109	0.162311019	0.347734431	0.106529392
ENSMUSG00000001707	Eef1e1	66143	protein_coding	146.4284245	0.106536372	0.11979448	-0.88932622	0.373827784	0.595844868	0.106536372
ENSMUSG00000040464	Gtpbp10	207704	protein_coding	167.3405463	-0.106546148	0.128389089	0.829869183	0.40661275	0.627434401	0.106546148
ENSMUSG00000042308	Setd1a	233904	protein_coding	100.1811584	0.106581194	0.139762326	-0.762588868	0.445708626	0.662922836	0.106581194
ENSMUSG00000085169	Gm10785	100038392	lncRNA	13.51222303	-0.106586909	0.34748776	0.306735722	0.759044537	0.878912418	0.106586909
ENSMUSG00000078954	Arhgap8	73167	protein_coding	67.08843854	-0.106590161	0.159208234	0.669501558	0.503175587	0.707788896	0.106590161
ENSMUSG00000020231	Dip2a	64451	protein_coding	1124.506352	-0.106610291	0.049821494	2.139845304	0.032367271	0.10695305	0.106610291
ENSMUSG00000024725	Ostf1	20409	protein_coding	948.4176055	0.106620729	0.081860944	-1.302461512	0.192758667	0.390530809	0.106620729

ensembl_gene_id	external_gene_name	entrezgene_id	gene_biotype	baseMean	log2FoldChange	lfcSE	stat	pvalue	padj	absLog2FC
ENSMUSG00000019297	Nop9	67842	protein_coding	240.2118745	0.106631506	0.121813043	-0.875370192	0.381372513	0.603717953	0.106631506
ENSMUSG00000003721	Insig2	72999	protein_coding	305.4759099	0.106635889	0.08967483	-1.189139575	0.234384748	0.443879555	0.106635889
ENSMUSG00000025940	Tmem70	70397	protein_coding	405.2832558	-0.106641258	0.11015074	0.96813928	0.332974815	0.555316713	0.106641258
ENSMUSG00000042719	Naa25	231713	protein_coding	358.7958188	0.10666058	0.084152415	-1.267469032	0.20498763	0.407409522	0.10666058
ENSMUSG00000006273	Atp6v1b2	11966	protein_coding	775.5482221	-0.106660774	0.087389729	1.220518423	0.22226841	0.429396526	0.106660774
ENSMUSG00000100627	A830008E24Rik	108167736	lncRNA	2.678573221	-0.106679485	1.159010551	0.092043584	0.926663412	1	0.106679485
ENSMUSG00000018217	Pmp22	18858	protein_coding	2285.728431	0.106688928	0.183292986	-0.582067707	0.560521076	0.749145763	0.106688928
ENSMUSG00000028902	Sf3a3	75062	protein_coding	427.5666805	0.106737627	0.11946297	-0.893478763	0.37160084	0.593942729	0.106737627
ENSMUSG00000061458	Nol10	217431	protein_coding	79.50673311	0.106803025	0.146902398	-0.727033911	0.467205182	0.680853016	0.106803025
ENSMUSG00000039768	Dnajc11	230935	protein_coding	972.1653897	-0.106820343	0.073949143	1.444510912	0.148595313	0.327361658	0.106820343
ENSMUSG00000033900	Map9	213582	protein_coding	23.23895291	0.106856114	0.262335914	-0.407325527	0.683768917	0.83113204	0.106856114
ENSMUSG00000008167	Fbxw9	68628	protein_coding	218.3596666	0.106874896	0.091952161	-1.162288022	0.24511849	0.456768986	0.106874896
ENSMUSG00000039201	Tbc1d25	209815	protein_coding	96.9241324	-0.106900275	0.118271201	0.903857188	0.366071133	0.588694	0.106900275
ENSMUSG00000038023	Atp6v0a2	21871	protein_coding	2174.584519	0.10690178	0.069206898	-1.544669437	0.122426241	0.285476835	0.10690178
ENSMUSG00000023723	Mrps23	64656	protein_coding	592.9324733	-0.106948879	0.08498686	1.258416643	0.208241116	0.41130117	0.106948879
ENSMUSG00000005447	Pafah1b3	18476	protein_coding	690.1722408	0.107040492	0.100463222	-1.06546943	0.286663552	0.50522539	0.107040492
ENSMUSG00000118559	A930007A09Rik	432999	protein_coding	6.520009181	-0.107052009	0.621359675	0.172286702	0.863212139	0.932166328	0.107052009
ENSMUSG00000060038	Dhps	330817	protein_coding	190.9566499	0.107056447	0.091710129	-1.167335047	0.24307509	0.454166041	0.107056447
ENSMUSG00000021650	Ptcd2	68927	protein_coding	569.7018585	0.107060871	0.098867061	-1.082877045	0.278863002	0.496167516	0.107060871
ENSMUSG00000071662	Polr2g	67710	protein_coding	251.0951382	0.10707354	0.092903683	-1.152522019	0.249106627	0.461264989	0.10707354
ENSMUSG00000020419	Hormad2	75828	protein_coding	1.475333014	0.107088192	1.154310206	-0.092772456	0.926084334	1	0.107088192
ENSMUSG00000057894	Zfp329	67230	protein_coding	141.4898355	-0.10710373	0.137506397	0.778899982	0.436038635	0.654196603	0.10710373
ENSMUSG00000029310	Nudt9	74167	protein_coding	391.2299778	-0.10710905	0.073058756	1.466067257	0.142629965	0.318339132	0.10710905
ENSMUSG00000090000	Ier3ip1	66191	protein_coding	351.8918202	0.107175299	0.092918214	-1.153436927	0.248731092	0.460883095	0.107175299
ENSMUSG00000032403	2300009A05Rik	69478	protein_coding	41.41350372	-0.107217269	0.204115365	0.525277794	0.599390092	0.77652216	0.107217269
ENSMUSG00000006932	Ctnnb1	12387	protein_coding	10595.83715	0.107219554	0.042576335	-2.518289919	0.011792621	0.04843157	0.107219554
ENSMUSG00000049657	Zbtb5	230119	protein_coding	122.3670956	-0.107222751	0.131527079	0.815214266	0.414949674	0.635253445	0.107222751
ENSMUSG00000031766	Slc12a3	20497	protein_coding	8.452439679	0.107230702	0.689801239	-0.155451594	0.876465313	0.938630585	0.107230702
ENSMUSG00000007656	Arpp19	59046	protein_coding	867.517463	-0.107238624	0.060192718	1.781587985	0.074816445	0.19999509	0.107238624
ENSMUSG00000033632	AW554918	225289	protein_coding	91.50225169	0.107242396	0.112148085	-0.956257034	0.338942392	0.560514185	0.107242396
ENSMUSG00000024104	Washc2	28006	protein_coding	569.1278381	-0.107255342	0.081965993	1.308534653	0.190692029	0.388133006	0.107255342
ENSMUSG00000054514	Atad3aos	70448	lncRNA	5.010153085	-0.107310818	0.506015231	0.212070331	0.832052166	0.915301723	0.107310818
ENSMUSG00000069306	H4c17	100041230	protein_coding	0.773698078	-0.107329467	1.658733238	0.064705683	0.948408338	1	0.107329467
ENSMUSG00000021396	Nxnl2	75124	protein_coding	3.068097215	0.107383007	0.725716643	-0.14796823	0.882367841	1	0.107383007
ENSMUSG00000057396	Zfp759	268670	protein_coding	77.42661503	-0.107390658	0.134160646	0.800463187	0.423442484	0.643305352	0.107390658
ENSMUSG00000041729	Coro2b	235431	protein_coding	13.00555245	0.10739154	0.592872865	-0.181137552	0.85625961	0.930174321	0.10739154
ENSMUSG00000000958	Slc7a7	20540	protein_coding	420.9106072	0.10740681	0.078393103	-1.370105342	0.17065402	0.360508479	0.10740681
ENSMUSG00000032998	Foxj3	230700	protein_coding	401.53314	-0.107414385	0.094905823	1.13179973	0.257718641	0.47089684	0.107414385
ENSMUSG00000021819	Zswim8	268721	protein_coding	558.7977277	0.10743396	0.09313172	-1.153570021	0.248676495	0.460883095	0.10743396

ensembl_gene_id	external_gene_name	entrezgene_id	gene_biotype	baseMean	log2FoldChange	lfcSE	stat	pvalue	padj	absLog2FC
ENSMUSG00000018821	Avpi1	69534	protein_coding	123.5903826	0.107502194	0.1723511	-0.623739528	0.532798659	0.72936091	0.107502194
ENSMUSG00000031696	Vps35	65114	protein_coding	1407.355784	0.107551381	0.083214303	-1.292462668	0.196196966	0.395770858	0.107551381
ENSMUSG00000032846	Zswim6	67263	protein_coding	290.8133656	-0.107552582	0.127934594	0.840684123	0.400524917	0.622394781	0.107552582
ENSMUSG00000018199	Ro60	20822	protein_coding	301.8138463	0.107559139	0.108788349	-0.988700907	0.322809499	0.544244621	0.107559139
ENSMUSG00000041459	Tardbp	230908	protein_coding	1216.332788	0.107621515	0.088615786	-1.214473412	0.224566997	0.431773126	0.107621515
ENSMUSG00000001583	Tnk1	83813	protein_coding	355.7738152	-0.10767586	0.095110205	1.132116788	0.257585338	0.470794255	0.10767586
ENSMUSG00000031328	Flna	192176	protein_coding	339.285902	0.107757987	0.117612347	-0.916213221	0.359555073	0.582240877	0.107757987
ENSMUSG00000049728	Zfp668	244219	protein_coding	108.7536669	0.107789184	0.126640369	-0.851143949	0.394689394	0.617166997	0.107789184
ENSMUSG00000001739	Cldn15	60363	protein_coding	2021.985921	-0.107833568	0.109920414	0.981014939	0.326585374	0.548411142	0.107833568
ENSMUSG00000032489	Kif9	16578	protein_coding	24.33026664	-0.107841425	0.249173241	0.432796976	0.66516227	0.819432282	0.107841425
ENSMUSG00000025821	Zfp282	101095	protein_coding	317.2746568	-0.107874902	0.073342631	1.470834913	0.141335768	0.316005805	0.107874902
ENSMUSG00000027478	Dnmt3b	13436	protein_coding	43.96226413	-0.107899985	0.224938306	0.479687014	0.631449963	0.799123664	0.107899985
ENSMUSG00000038697	Taf5l	102162	protein_coding	425.0357075	-0.107961682	0.057107924	1.890485148	0.058693105	0.168040558	0.107961682
ENSMUSG00000041360	Pum3	52874	protein_coding	420.7868047	-0.108066435	0.102249262	1.056892083	0.290560842	0.509484153	0.108066435
ENSMUSG00000042590	Ipo11	76582	protein_coding	283.5944447	0.108071343	0.118378976	-0.912926833	0.361281024	0.583719287	0.108071343
ENSMUSG00000024982	Zdhhc6	66980	protein_coding	440.2541734	0.108071392	0.085250704	-1.26768915	0.204908981	0.407353559	0.108071392
ENSMUSG00000020250	Txnrd1	50493	protein_coding	3215.801385	-0.108097001	0.067394137	1.603952595	0.108724502	0.262880883	0.108097001
ENSMUSG00000030795	Fus	233908	protein_coding	431.9428224	0.108128774	0.164128858	-0.658804158	0.510021537	0.71331253	0.108128774
ENSMUSG00000031438	Rnf128	66889	protein_coding	2348.199402	-0.108143726	0.061765899	1.750864592	0.079969237	0.209820779	0.108143726
ENSMUSG00000019732	Calr3	73316	protein_coding	7.354353238	-0.10820249	0.414215417	0.26122275	0.793920735	0.897263004	0.10820249
ENSMUSG00000054312	Mrps21	66292	protein_coding	458.5677327	-0.108214728	0.084236718	1.284650339	0.198914499	0.39947392	0.108214728
ENSMUSG00000018377	Vezf1	22344	protein_coding	470.5724864	-0.108240815	0.088187104	1.227399586	0.219672442	0.425947586	0.108240815
ENSMUSG00000027983	Cyp2u1	71519	protein_coding	18.67955595	-0.108287819	0.363233879	0.298121474	0.765610453	0.882510888	0.108287819
ENSMUSG00000023939	Mrpl14	68463	protein_coding	316.0296976	-0.108294409	0.083230873	1.301132677	0.193213044	0.391355356	0.108294409
ENSMUSG00000039841	Zfp800	627049	protein_coding	314.7046692	0.108318894	0.086577084	-1.251126612	0.210888288	0.415010021	0.108318894
ENSMUSG00000032423	Syncrip	56403	protein_coding	903.8417648	0.108338535	0.103559723	-1.046145472	0.295493874	0.514582498	0.108338535
ENSMUSG00000031974	Abcb10	56199	protein_coding	376.1863104	-0.108349771	0.085104389	1.27313964	0.202968495	0.405213026	0.108349771
ENSMUSG00000007207	Stx1a	20907	protein_coding	21.91743188	0.108349855	0.41605528	-0.260421775	0.794538448	0.897715376	0.108349855
ENSMUSG00000024683	Mrpl16	94063	protein_coding	250.4964966	0.108351037	0.090089164	-1.202708876	0.229088998	0.438019649	0.108351037
ENSMUSG00000031897	Psmb10	19171	protein_coding	1309.538983	-0.108428406	0.0651897	1.66327511	0.096257379	0.240265168	0.108428406
ENSMUSG00000022571	Pycrl	66194	protein_coding	644.3923307	-0.108440908	0.086403091	1.255058197	0.209457634	0.412914333	0.108440908
ENSMUSG00000025995	Wdr75	73674	protein_coding	314.5104785	0.108446246	0.101553154	-1.067876693	0.285576137	0.504062027	0.108446246
ENSMUSG00000026235	Epha4	13838	protein_coding	260.7468617	0.108463443	0.147271495	-0.736486331	0.461434783	0.675184655	0.108463443
ENSMUSG00000024645	Timm21	67105	protein_coding	188.6113742	-0.108523541	0.127540953	0.850891718	0.394829506	0.617210759	0.108523541
ENSMUSG00000004565	Pnpla6	50767	protein_coding	706.1092591	-0.108554729	0.10004872	1.085018669	0.277913387	0.494874021	0.108554729
ENSMUSG00000057594	Arl16	70317	protein_coding	78.36348307	0.108565151	0.143425114	-0.756946591	0.449081861	0.665841071	0.108565151
ENSMUSG00000033419	Snap91	20616	protein_coding	3.251829716	-0.108598233	1.34161614	0.080945831	0.935485032	1	0.108598233
ENSMUSG00000061607	Mdc1	240087	protein_coding	248.3376121	0.108639744	0.123041776	-0.882950063	0.377263253	0.599511678	0.108639744
ENSMUSG00000091370	5730435O14Rik	70538	lncRNA	0.780683872	-0.108695826	1.5339423	0.07086044	0.943508829	1	0.108695826

ensembl_gene_id	external_gene_name	entrezgene_id	gene_biotype	baseMean	log2FoldChange	lfcSE	stat	pvalue	padj	absLog2FC
ENSMUSG00000024570	Rbfa	68731	protein_coding	110.2766775	-0.108698347	0.132641313	0.819490888	0.412506399	0.632451088	0.108698347
ENSMUSG00000049612	Omg	18377	protein_coding	5.007887367	0.108714692	0.513591721	-0.21167532	0.832360344	0.915579745	0.108714692
ENSMUSG00000024646	Cyb5a	109672	protein_coding	999.7410295	0.108781453	0.069075889	-1.574810759	0.115300132	0.273824144	0.108781453
ENSMUSG00000084880	Tomm6os	100415787	lncRNA	5.93748596	-0.108824458	0.531589342	0.204715274	0.837794586	0.919169532	0.108824458
ENSMUSG00000092260	Zfp963	620419	protein_coding	29.98041749	0.108825834	0.210165282	-0.517810712	0.604590344	0.779942456	0.108825834
ENSMUSG00000087260	Lamtor5	68576	protein_coding	360.4939305	-0.108916173	0.11904269	0.914933738	0.360226417	0.582642414	0.108916173
ENSMUSG00000090164	BC035044	232406	protein_coding	2.675241534	-0.108941103	0.741355171	0.146948599	0.883172592	1	0.108941103
ENSMUSG00000029178	Klf3	16599	protein_coding	2954.586206	-0.109008839	0.084683284	1.287253325	0.198006006	0.398279073	0.109008839
ENSMUSG00000004630	Pcp2	18545	protein_coding	1.498441324	-0.10901701	1.333665544	0.081742391	0.934851568	1	0.10901701
ENSMUSG00000046733	Gprc5a	232431	protein_coding	1239.746564	-0.109088388	0.22494434	0.484957249	0.627706671	0.796376973	0.109088388
ENSMUSG00000021738	Atxn7	246103	protein_coding	463.1648533	0.109133206	0.058195336	-1.875291266	0.060752664	0.172412699	0.109133206
ENSMUSG00000029153	Ociad2	433904	protein_coding	2149.281875	-0.109161253	0.072499908	1.505674357	0.13215079	0.3013667	0.109161253
ENSMUSG00000088835	Gm23547	115490496	snoRNA	4.742595552	-0.109174543	0.544357266	0.200556785	0.841045152	0.92126689	0.109174543
ENSMUSG00000020520	Galnt10	171212	protein_coding	2016.380478	-0.109202698	0.132508004	0.824121525	0.409870511	0.630340996	0.109202698
ENSMUSG00000021097	Clmn	94040	protein_coding	2578.882791	-0.109223692	0.125152169	0.872727115	0.382811834	0.605068399	0.109223692
ENSMUSG00000021803	Cdhr1	170677	protein_coding	87.45868768	0.109234846	0.213907263	-0.510664504	0.609586	0.783477444	0.109234846
ENSMUSG00000022283	Pabpc1	18458	protein_coding	3903.83026	-0.109339784	0.098733328	1.10742528	0.268110101	0.48334534	0.109339784
ENSMUSG00000022962	Gart	14450	protein_coding	533.733064	-0.109344088	0.130428147	0.838347318	0.401835668	0.623441772	0.109344088
ENSMUSG00000026657	Frmd4a	209630	protein_coding	155.0714926	0.109368854	0.091811809	-1.191228619	0.233563847	0.442985342	0.109368854
ENSMUSG00000020483	Dynll2	68097	protein_coding	866.2964104	0.109407329	0.091058864	-1.201501135	0.229556867	0.438122528	0.109407329
ENSMUSG00000025651	Uqcrc1	22273	protein_coding	5157.735676	-0.10941094	0.080408507	1.360688608	0.173612116	0.364391266	0.10941094
ENSMUSG00000038970	Lmtk2	231876	protein_coding	1503.861004	0.109506812	0.0719232	-1.522551995	0.127870828	0.294844608	0.109506812
ENSMUSG00000075010	AW112010	107350	lncRNA	367.0131301	0.109514569	0.146089336	-0.749641091	0.453470896	0.669164362	0.109514569
ENSMUSG00000024947	Men1	17283	protein_coding	405.627589	0.109526443	0.11012627	-0.994553281	0.319953579	0.541253166	0.109526443
ENSMUSG00000032405	Pias1	56469	protein_coding	282.0144843	-0.109526903	0.071856311	1.5242489	0.127446547	0.294356962	0.109526903
ENSMUSG00000045725	Prr15	78004	protein_coding	503.3092539	0.109540237	0.176817838	-0.619508971	0.535581114	0.73156216	0.109540237
ENSMUSG00000086995	Gm13544	100502932	lncRNA	1.751811137	-0.109568855	0.952497181	0.115033259	0.90841876	1	0.109568855
ENSMUSG00000028862	Map3k6	53608	protein_coding	129.5734996	-0.109629865	0.167946423	0.652766895	0.513906569	0.715629739	0.109629865
ENSMUSG00000029919	Hpgds	54486	protein_coding	76.46245632	0.109630084	0.148744458	-0.737036428	0.461100197	0.674995696	0.109630084
ENSMUSG00000039809	Gabbr2	242425	protein_coding	4.523670617	0.109660769	1.017481438	-0.107776677	0.914172844	0.958030863	0.109660769
ENSMUSG00000048799	Cep120	225523	protein_coding	196.4794581	-0.109663195	0.075418915	1.454054261	0.145931263	0.323295567	0.109663195
ENSMUSG00000032299	Commd4	66199	protein_coding	230.7066956	-0.109664079	0.07521555	1.45799743	0.144841251	0.321916639	0.109664079
ENSMUSG00000027433	Xrn2	24128	protein_coding	907.6336118	-0.10967475	0.053097663	2.065528783	0.038872995	0.123253915	0.10967475
ENSMUSG00000070501	Ifi214	545384	protein_coding	1.413395245	-0.109693985	2.054609328	0.053389218	0.957421796	1	0.109693985
ENSMUSG00000075271	Ttc30a1	78802	protein_coding	50.97136333	-0.109730398	0.19325422	0.567803373	0.570168489	0.755800484	0.109730398
ENSMUSG00000025613	Cct8	12469	protein_coding	1583.966873	0.109736869	0.09753411	-1.125112733	0.260541266	0.474068167	0.109736869
ENSMUSG00000041949	Tango6	272538	protein_coding	64.50478636	-0.109740156	0.143074437	0.767014415	0.443072945	0.660786267	0.109740156
ENSMUSG00000027374	Mrps5	77721	protein_coding	434.82836	-0.109779779	0.097109061	1.130479259	0.258274333	0.471755595	0.109779779
ENSMUSG00000028115	Bnipl	171388	protein_coding	89.38591824	-0.109797578	0.161707084	0.67899053	0.497143862	0.702914252	0.109797578

ensembl_gene_id	external_gene_name	entrezgene_id	gene_biotype	baseMean	log2FoldChange	lfcSE	stat	pvalue	padj	absLog2FC
ENSMUSG00000022438	Parvb	170736	protein_coding	3.454062976	-0.109804593	1.60840352	0.068269306	0.945571257	1	0.109804593
ENSMUSG00000033809	Alg3	208624	protein_coding	90.47625297	0.109832544	0.164563317	-0.667418144	0.50450508	0.708596728	0.109832544
ENSMUSG00000022706	Mrpl40	18100	protein_coding	340.0230489	-0.109858762	0.098311207	1.117459196	0.263798037	0.47814299	0.109858762
ENSMUSG00000075000	Nrbf2	641340	protein_coding	104.7938198	-0.109867695	0.106878505	1.027968108	0.30396483	0.524360158	0.109867695
ENSMUSG00000061306	Slc38a10	72055	protein_coding	1589.40058	0.10988507	0.077181891	-1.423715706	0.154528785	0.336447148	0.10988507
ENSMUSG00000053119	Chmp3	66700	protein_coding	1810.308388	-0.109923846	0.072181709	1.522876729	0.127789549	0.294806545	0.109923846
ENSMUSG00000017291	Taok1	216965	protein_coding	1669.588264	0.109935622	0.055289042	-1.988379928	0.046769687	0.141690444	0.109935622
ENSMUSG00000033088	Triobp	110253	protein_coding	805.3844684	0.10994406	0.048246836	-2.278782809	0.022679979	0.08105111	0.10994406
ENSMUSG00000068663	Clec16a	74374	protein_coding	411.9323834	0.110018766	0.069233373	-1.589100171	0.112037779	0.268218365	0.110018766
ENSMUSG00000032324	Tspan3	56434	protein_coding	3441.092879	0.110024268	0.120211068	-0.915259056	0.360055648	0.5825209	0.110024268
ENSMUSG00000037234	Hook3	320191	protein_coding	362.8608812	-0.110108377	0.080548941	1.36697487	0.171633183	0.361939775	0.110108377
ENSMUSG00000029320	1700016H13Rik	74218	protein_coding	7.02443844	0.110113476	0.412903926	-0.266680623	0.789715074	0.894959836	0.110113476
ENSMUSG00000066368	Actl11	67722	protein_coding	3.028851954	0.11015461	1.475571974	-0.074652143	0.940491486	1	0.11015461
ENSMUSG00000074166	AW146154	101835	protein_coding	94.40368696	-0.110188149	0.111640499	0.986990835	0.323647131	0.545489766	0.110188149
ENSMUSG00000032373	Car12	76459	protein_coding	845.9142005	0.110210495	0.129716347	-0.849626883	0.395532561	0.617841913	0.110210495
ENSMUSG00000040532	Abhd11	68758	protein_coding	530.1723571	0.110235222	0.072178884	-1.527250307	0.126698781	0.292957669	0.110235222
ENSMUSG00000006567	Atp7b	11979	protein_coding	25.50514424	0.110256476	0.322428473	-0.341956389	0.732383713	0.862666522	0.110256476
ENSMUSG00000054191	Klf1	16596	protein_coding	9.522377488	-0.110290175	0.412818857	0.267163608	0.789343195	0.894720656	0.110290175
ENSMUSG00000037458	Azin1	54375	protein_coding	2332.013389	-0.110290323	0.067205144	1.641099409	0.100776782	0.249173195	0.110290323
ENSMUSG00000047371	Zfp768	233890	protein_coding	429.6306211	-0.11032699	0.069010999	1.598687057	0.109890155	0.264768897	0.11032699
ENSMUSG00000072640	Lyrm9	66274	protein_coding	108.9574634	-0.110337859	0.139860371	0.788914391	0.430162044	0.648857873	0.110337859
ENSMUSG00000020021	Fgd6	13998	protein_coding	368.7037482	-0.110361324	0.201400477	0.547969525	0.583712828	0.766128886	0.110361324
ENSMUSG00000085078	C030013C21Rik	77417	lncRNA	9.287337574	0.110377278	0.387088727	-0.285147228	0.775531348	0.88791795	0.110377278
ENSMUSG00000019850	Tnfaip3	21929	protein_coding	538.0938343	-0.11044017	0.227288196	0.485903678	0.62703546	0.795976331	0.11044017
ENSMUSG00000067851	Arfgef1	211673	protein_coding	2759.553327	0.110486128	0.08998446	-1.227835648	0.219508671	0.42576535	0.110486128
ENSMUSG00000028268	Gbp3	55932	protein_coding	41.46117599	0.110497383	0.420261025	-0.262925601	0.792607924	0.896761253	0.110497383
ENSMUSG00000022674	Ube2v2	70620	protein_coding	92.32951449	-0.110530718	0.164111592	0.67350951	0.500623201	0.705219785	0.110530718
ENSMUSG00000025010	Ccnj	240665	protein_coding	63.33964729	-0.110564745	0.177837442	0.621718037	0.534127289	0.730361684	0.110564745
ENSMUSG00000034235	Usp54	78787	protein_coding	107.8276544	0.110567062	0.157736616	-0.700960026	0.483327962	0.693817626	0.110567062
ENSMUSG00000035621	Midn	59090	protein_coding	2123.847966	0.110570008	0.141871268	-0.779368578	0.435762628	0.653940181	0.110570008
ENSMUSG00000022463	Srebf2	20788	protein_coding	1725.959458	-0.1106203	0.057907537	1.9102919	0.05609564	0.162548412	0.1106203
ENSMUSG00000021807	Rtraf	68045	protein_coding	1074.166309	-0.110621133	0.101375154	1.091205574	0.275182436	0.49170772	0.110621133
ENSMUSG00000046840	Hnf4aos	68314	lncRNA	5.77961545	-0.110642867	0.473211634	0.233812651	0.815130428	0.908120076	0.110642867
ENSMUSG00000052675	Zfp112	57745	protein_coding	26.77296208	-0.110661266	0.253558033	0.43643368	0.662522107	0.817399333	0.110661266
ENSMUSG00000022788	Fgd4	224014	protein_coding	1029.285362	-0.11066316	0.081078099	1.364895841	0.172285789	0.36280925	0.11066316
ENSMUSG00000031156	Slc35a2	22232	protein_coding	1187.094006	0.110669591	0.141307662	-0.783181818	0.43352035	0.652195961	0.110669591
ENSMUSG00000032602	Slc25a20	57279	protein_coding	1778.61799	-0.110675044	0.136935252	0.80822902	0.418958755	0.638903396	0.110675044
ENSMUSG00000017478	Zc3h18	76014	protein_coding	1198.153698	0.110710125	0.06822933	-1.622617786	0.104671136	0.255796319	0.110710125
ENSMUSG00000025364	Pa2g4	18813	protein_coding	854.5705535	0.110723341	0.087348671	-1.267601902	0.204940153	0.407364189	0.110723341

Supplementary Table 1

ensembl_gene_id	external_gene_name	entrezgene_id	gene_biotype	baseMean	log2FoldChange	lfcSE	stat	pvalue	padj	absLog2FC
ENSMUSG00000028536	2610528J11Rik	66451	protein_coding	482.0731302	-0.110730221	0.08455737	1.309527727	0.190355652	0.387766717	0.110730221
ENSMUSG00000053253	Ndfip2	76273	protein_coding	1879.43666	0.110736408	0.091996237	-1.203705839	0.228703295	0.437687589	0.110736408
ENSMUSG00000027893	Ahcyl1	229709	protein_coding	1030.850838	0.11074014	0.102157754	-1.084011103	0.278359876	0.495562203	0.11074014
ENSMUSG00000032320	Rcn2	26611	protein_coding	268.4426736	0.110764216	0.082271518	-1.346325181	0.178197668	0.37057407	0.110764216
ENSMUSG00000100200	H2al1m	76383	protein_coding	1.282989417	-0.110773252	0.98042542	0.112984883	0.91004254	1	0.110773252
ENSMUSG00000030745	Il21r	60504	protein_coding	8.121029649	-0.110777557	0.503631599	0.219957519	0.82590424	0.912001512	0.110777557
ENSMUSG00000043091	Tuba1c	22146	protein_coding	1515.453147	0.11080861	0.116046441	-0.954864349	0.339646301	0.561116348	0.11080861
ENSMUSG00000020083	Fam241b	69894	protein_coding	166.9779528	-0.110812508	0.089566563	1.237208444	0.216009708	0.421254642	0.110812508
ENSMUSG00000014601	Strip1	229707	protein_coding	495.038811	0.110830979	0.066599066	-1.664152141	0.096082029	0.239900005	0.110830979
ENSMUSG00000074024	4632427E13Rik	666737	lncRNA	135.8535309	-0.110839329	0.176917735	0.626502078	0.530985674	0.728048657	0.110839329
ENSMUSG00000037139	Myom3	242702	protein_coding	39.73891553	-0.110858157	0.354633172	0.312599514	0.754584947	0.876856731	0.110858157
ENSMUSG00000025239	Limd1	29806	protein_coding	1066.455482	-0.110878435	0.069291811	1.600166508	0.10956165	0.264291499	0.110878435
ENSMUSG00000025888	Casp1	12362	protein_coding	1676.690301	-0.110879105	0.083875311	1.32195164	0.186184255	0.38181259	0.110879105
ENSMUSG00000032186	Tmod2	50876	protein_coding	9.389283513	0.110890363	0.617728866	-0.179513002	0.857534909	0.930374388	0.110890363
ENSMUSG00000063663	Brwd3	382236	protein_coding	425.2912314	0.110921861	0.090252659	-1.229014872	0.21906623	0.42530681	0.110921861
ENSMUSG00000116903	Gm19522	100503040	lncRNA	10.3700997	0.110923618	0.383513372	-0.289230118	0.772405285	0.885864136	0.110923618
ENSMUSG00000038236	Hoxa7	15404	protein_coding	277.9025816	0.110932344	0.089999549	-1.232587781	0.217729598	0.423608221	0.110932344
ENSMUSG00000024927	Rela	19697	protein_coding	389.0372732	-0.110959475	0.064012628	1.733399787	0.083024635	0.215780426	0.110959475
ENSMUSG00000026604	Ptpn14	19250	protein_coding	60.64017323	-0.110959643	0.14642227	0.757805781	0.448567261	0.665436557	0.110959643
ENSMUSG00000109813	Or4g16	258388	protein_coding	1.020055681	-0.110988143	1.759389925	0.063083311	0.949700163	1	0.110988143
ENSMUSG00000035367	Rmi1	74386	protein_coding	114.8636548	-0.111048941	0.108982884	1.018957629	0.308223082	0.528838189	0.111048941
ENSMUSG00000027579	Srms	20811	protein_coding	5.487685274	-0.111063911	0.502207342	0.221151507	0.824974466	0.911456552	0.111063911
ENSMUSG00000058258	Idi1	319554	protein_coding	209.9063587	-0.111099338	0.202668915	0.548181443	0.583567323	0.766020418	0.111099338
ENSMUSG00000041112	Elmo1	140580	protein_coding	208.6714144	0.111160457	0.164865251	-0.674250374	0.500152149	0.704796705	0.111160457
ENSMUSG00000026087	Mrpl30	107734	protein_coding	647.5118251	-0.111164362	0.066671373	1.667347717	0.095445284	0.238987471	0.111164362
ENSMUSG00000022905	Kpna1	16646	protein_coding	761.7622851	0.111182047	0.073356761	-1.515634618	0.129611768	0.297320449	0.111182047
ENSMUSG00000044017	Adgrd1	243277	protein_coding	7.094397557	0.11123815	0.830388685	-0.133959136	0.893434888	0.947542466	0.11123815
ENSMUSG00000069114	Zbtb10	229055	protein_coding	551.8484185	0.111323167	0.086475084	-1.287343846	0.197974467	0.398279073	0.111323167
ENSMUSG00000021518	Ptdss1	19210	protein_coding	1228.74279	-0.111348059	0.065257345	1.70629159	0.087953803	0.22515016	0.111348059
ENSMUSG00000033554	Dph5	69740	protein_coding	98.4881183	-0.111358207	0.169294517	0.657777986	0.510680802	0.713786099	0.111358207
ENSMUSG00000032018	Sc5d	235293	protein_coding	739.1845923	0.111361626	0.074107688	-1.502700044	0.132916411	0.30270362	0.111361626
ENSMUSG00000025949	Pikfyve	18711	protein_coding	517.9017231	-0.111374776	0.054831235	2.031228676	0.042231803	0.131176532	0.111374776
ENSMUSG00000025737	Wdr24	268933	protein_coding	400.7469501	0.111380828	0.06727042	-1.655717742	0.097778993	0.243290987	0.111380828
ENSMUSG00000001436	Slc19a1	20509	protein_coding	111.996956	0.111449673	0.13631875	-0.81756672	0.413604637	0.633723045	0.111449673
ENSMUSG00000018861	Fdxr	14149	protein_coding	199.5812501	-0.111454278	0.11161919	0.998522549	0.318026036	0.539389762	0.111454278
ENSMUSG00000090877	Hspa1b	15511	protein_coding	1185.438871	0.11147638	0.470115059	-0.237125737	0.812559246	0.907147741	0.11147638
ENSMUSG00000014496	Ankrd28	105522	protein_coding	584.4076719	0.111489575	0.101810176	-1.095073009	0.273484652	0.489835334	0.111489575
ENSMUSG00000028139	Riiad1	66353	protein_coding	14.46614412	-0.111519458	0.496893935	0.224433124	0.822420299	0.910171686	0.111519458
ENSMUSG00000058503	Fam133b	68152	protein_coding	194.6558142	0.111528484	0.09493285	-1.174814447	0.240068959	0.450443439	0.111528484

ensembl_gene_id	external_gene_name	entrezgene_id	gene_biotype	baseMean	log2FoldChange	lfcSE	stat	pvalue	padj	absLog2FC
ENSMUSG00000097781	9330136K24Rik	319870	lncRNA	1.622827424	-0.111581537	0.886344028	0.125889647	0.899819278	1	0.111581537
ENSMUSG00000000881	Dlg3	53310	protein_coding	751.5365116	-0.111650023	0.072516354	1.539653013	0.123644956	0.28762823	0.111650023
ENSMUSG00000031327	Chic1	12212	protein_coding	239.0571319	0.111661969	0.129101336	-0.864917222	0.387084224	0.609604127	0.111661969
ENSMUSG00000061540	Orm2	18406	protein_coding	1.171780014	-0.111686962	1.592154687	0.070148311	0.944075615	1	0.111686962
ENSMUSG00000041035	Dpcd	226162	protein_coding	185.9118463	0.1117364	0.088007453	-1.269624288	0.204218493	0.406467904	0.1117364
ENSMUSG00000062797	Hikeshi	67669	protein_coding	176.7357568	-0.111769038	0.123099703	0.90795538	0.363901796	0.586347377	0.111769038
ENSMUSG00000032596	Uba7	74153	protein_coding	510.5974075	-0.111817972	0.115848202	0.965211121	0.334439078	0.556711723	0.111817972
ENSMUSG00000036636	Clcn7	26373	protein_coding	347.8130011	-0.111843962	0.069443774	1.610568607	0.107273781	0.260339278	0.111843962
ENSMUSG00000036104	Rab3gap1	226407	protein_coding	955.8959493	-0.111849565	0.087995454	1.271083443	0.203698965	0.405918699	0.111849565
ENSMUSG00000054693	Adam10	11487	protein_coding	1382.500117	0.111941509	0.079143832	-1.414405982	0.157242735	0.340507018	0.111941509
ENSMUSG00000061143	Maml3	433586	protein_coding	314.9984307	0.111960158	0.088451362	-1.265782187	0.205591074	0.408255959	0.111960158
ENSMUSG00000026020	Nop58	55989	protein_coding	734.2137714	0.111967589	0.089161315	-1.25578666	0.209193329	0.412590307	0.111967589
ENSMUSG00000026971	Itgb6	16420	protein_coding	528.3669595	-0.111975807	0.119037592	0.94067601	0.346870917	0.568558443	0.111975807
ENSMUSG00000000759	Tubgcp3	259279	protein_coding	391.1456918	-0.111976306	0.070328601	1.592187318	0.111342622	0.267099369	0.111976306
ENSMUSG00000049354	Dcaf7	71833	protein_coding	1278.278334	0.112015514	0.10011094	-1.118913819	0.263176907	0.477488842	0.112015514
ENSMUSG00000021998	Lcp1	18826	protein_coding	146.6785962	0.112036296	0.169829544	-0.659698506	0.509447326	0.712785998	0.112036296
ENSMUSG00000020865	Abcc3	76408	protein_coding	4603.498921	-0.112038452	0.137889067	0.812526	0.416489874	0.636725956	0.112038452
ENSMUSG00000029199	Lias	79464	protein_coding	326.2443609	-0.112064557	0.076745208	1.460215676	0.144230808	0.320998127	0.112064557
ENSMUSG00000028882	Ppp1r8	100336	protein_coding	347.2255198	0.112100002	0.085751601	-1.307264239	0.191122985	0.388695194	0.112100002
ENSMUSG00000020922	Lsm12	268490	protein_coding	519.4260763	0.112109648	0.08897811	-1.259968856	0.207680598	0.410708835	0.112109648
ENSMUSG00000037787	Coa8	68020	protein_coding	196.0186938	-0.11212835	0.085496498	1.311496407	0.189690107	0.386972505	0.11212835
ENSMUSG00000022755	Adgrg7	239853	protein_coding	3829.312265	-0.112155717	0.112435174	0.997514508	0.318514835	0.539980904	0.112155717
ENSMUSG00000036099	Vezt	215008	protein_coding	222.7275296	-0.112164204	0.08861523	1.265744086	0.205604719	0.408255959	0.112164204
ENSMUSG00000024604	Rbm22	66810	protein_coding	417.5412993	-0.112166691	0.063861483	1.756405983	0.079019112	0.208221097	0.112166691
ENSMUSG00000047139	Cd24a	12484	protein_coding	3877.486936	0.112223957	0.153790722	-0.729718642	0.465562184	0.67923791	0.112223957
ENSMUSG00000089682	Bcl2l2	12050	protein_coding	598.7159045	0.11223478	0.097520481	-1.150884193	0.249779881	0.462014445	0.11223478
ENSMUSG00000024160	Spsb3	79043	protein_coding	84.0507282	0.112294181	0.116542839	-0.963544234	0.335274477	0.557631454	0.112294181
ENSMUSG00000038619	Ensa	56205	protein_coding	1173.359601	0.112298853	0.055025163	-2.040863636	0.041264383	0.128987322	0.112298853
ENSMUSG00000027680	Fxr1	14359	protein_coding	507.537419	0.112305695	0.083235809	-1.349247365	0.177257526	0.369553185	0.112305695
ENSMUSG00000050914	Ankrd37	654824	protein_coding	67.20030449	-0.112325719	0.30052778	0.373761516	0.708581754	0.847629053	0.112325719
ENSMUSG00000024732	Ccdc86	108673	protein_coding	121.3690289	-0.112338445	0.151742087	0.740324897	0.459102878	0.673388737	0.112338445
ENSMUSG00000071632	2510002D24Rik	72307	protein_coding	177.8050013	-0.112352711	0.083985149	1.337768786	0.18097182	0.374977244	0.112352711
ENSMUSG00000042447	Mios	252875	protein_coding	194.3466528	-0.112391783	0.087832895	1.279609231	0.200682602	0.401975042	0.112391783
ENSMUSG00000022817	Itgb5	16419	protein_coding	464.9284882	0.112427706	0.101746726	-1.104976157	0.26916992	0.484159903	0.112427706
ENSMUSG00000011008	Mcoln2	68279	protein_coding	373.1536352	0.112486818	0.145229872	-0.774543257	0.438609598	0.656764359	0.112486818
ENSMUSG00000022565	Plec	18810	protein_coding	11731.88422	-0.112577065	0.180113983	0.625032343	0.531949831	0.728820184	0.112577065
ENSMUSG00000090553	Snrpe	20643	protein_coding	383.5655275	-0.112602412	0.1284625	0.876539167	0.380736994	0.603058768	0.112602412
ENSMUSG00000020537	Drg2	13495	protein_coding	410.4384943	0.112639271	0.088564496	-1.271833251	0.203432372	0.405734564	0.112639271
ENSMUSG00000003184	Irf3	54131	protein_coding	759.0073551	-0.112646669	0.054398522	2.070767108	0.038380565	0.122005131	0.112646669

ensembl_gene_id	external_gene_name	entrezgene_id	gene_biotype	baseMean	log2FoldChange	lfcSE	stat	pvalue	padj	absLog2FC
ENSMUSG00000042323	Pbrm1	66923	protein_coding	1280.358423	0.112711464	0.087253494	-1.291770207	0.196436736	0.396067528	0.112711464
ENSMUSG00000059975	Zfp74	72723	protein_coding	49.78500075	-0.112748615	0.163928442	0.68779166	0.491583989	0.698491194	0.112748615
ENSMUSG00000027573	Gid8	76425	protein_coding	748.4144508	-0.112760915	0.070031743	1.610140067	0.107367282	0.260514736	0.112760915
ENSMUSG00000017950	Hnf4a	15378	protein_coding	9439.864415	-0.112770085	0.073720744	1.529692711	0.126092809	0.292047215	0.112770085
ENSMUSG00000039844	Rapgef1	107746	protein_coding	546.81881	0.112780096	0.062107102	-1.815896932	0.069386184	0.18970918	0.112780096
ENSMUSG00000063065	Mapk3	26417	protein_coding	2997.508925	-0.112801281	0.068168173	1.654749949	0.09797523	0.243645564	0.112801281
ENSMUSG00000067369	Trmt2b	215201	protein_coding	511.9423622	-0.112843963	0.074140402	1.522030628	0.128001407	0.295101944	0.112843963
ENSMUSG00000074250	Gm10653	677044	transcribed_processed_pseudogene	2.216509913	-0.112845829	0.915111964	0.123313686	0.901858704	1	0.112845829
ENSMUSG00000008200	Fnbp4	55935	protein_coding	448.5136126	0.11284666	0.111020671	-1.016447287	0.309416438	0.53032725	0.11284666
ENSMUSG00000044033	Ccdc141	545428	protein_coding	18.76409479	-0.112855343	0.354056488	0.31874954	0.749916445	0.874376659	0.112855343
ENSMUSG00000070394	Tmem256	69186	protein_coding	367.18172	-0.112882847	0.088015403	1.282535136	0.199654988	0.40075936	0.112882847
ENSMUSG00000005813	Metap1	75624	protein_coding	778.1805512	0.112915958	0.098776573	-1.143145132	0.252978338	0.465324602	0.112915958
ENSMUSG00000096199	Ptrhd1	69709	protein_coding	65.48513862	0.112947564	0.158621587	-0.712056702	0.476429664	0.688162291	0.112947564
ENSMUSG00000019254	Ppp1r12c	232807	protein_coding	403.9937018	0.112948118	0.0856636	-1.318507724	0.187333741	0.383501006	0.112948118
ENSMUSG00000091971	Hspa1a	193740	protein_coding	429.081421	0.112958486	0.565285547	-0.199825533	0.841617031	0.92158767	0.112958486
ENSMUSG00000026466	Tor1aip1	208263	protein_coding	266.9270102	-0.112962648	0.095794804	1.179214778	0.238312663	0.448589299	0.112962648
ENSMUSG00000023341	Mx2	17858	protein_coding	20.72591541	0.112965071	0.463219401	-0.243869472	0.807331914	0.904987729	0.112965071
ENSMUSG00000027245	Hypk	67693	protein_coding	19.06586084	0.112978758	0.34676582	-0.325807075	0.744570325	0.871118268	0.112978758
ENSMUSG00000018412	Kansl1	76719	protein_coding	687.5247721	-0.113013012	0.07556417	1.495589931	0.134760549	0.305611042	0.113013012
ENSMUSG00000035078	Mtmr9	210376	protein_coding	352.403288	-0.113052217	0.100485853	1.125056045	0.260565286	0.474068167	0.113052217
ENSMUSG00000069259	Prl6a1	19111	protein_coding	3.035506202	0.113110437	0.597776014	-0.189218761	0.849921364	1	0.113110437
ENSMUSG00000078812	Eif5a	276770	protein_coding	3379.974848	0.113114946	0.078007577	-1.450050756	0.147044366	0.324941878	0.113114946
ENSMUSG00000015341	Golga7	57437	protein_coding	418.4278076	-0.113116825	0.087963419	1.285953025	0.198459456	0.398802563	0.113116825
ENSMUSG00000092134	Gm17089	118027456	lncRNA	4.527573319	0.113116873	0.693345718	-0.163146422	0.87040315	0.935980063	0.113116873
ENSMUSG00000024644	Cndp2	66054	protein_coding	654.2474763	-0.113125767	0.085042993	1.330218556	0.183446272	0.378300667	0.113125767
ENSMUSG00000002307	Daxx	13163	protein_coding	317.9125459	-0.113130192	0.073334742	1.542654816	0.12291455	0.286211349	0.113130192
ENSMUSG00000097167	Gm16740	100504029	lncRNA	4.191643076	0.113140319	0.503380278	-0.224761128	0.822165108	1	0.113140319
ENSMUSG00000050114	Prdx6b	320769	protein_coding	0.920862446	0.11316769	1.567166285	-0.072211667	0.942433461	1	0.11316769
ENSMUSG00000020648	Dus4l	71916	protein_coding	41.17680244	0.113169026	0.18324657	-0.617577867	0.536853638	0.732676764	0.113169026
ENSMUSG00000019179	Mdh2	17448	protein_coding	2884.943669	0.113184491	0.07757605	-1.459013319	0.144561442	0.321507788	0.113184491
ENSMUSG00000022838	Eaf2	106389	protein_coding	6.326626064	0.113232521	0.488117448	-0.231978023	0.816555083	0.908481483	0.113232521
ENSMUSG00000073434	Wdr90	106618	protein_coding	171.0514665	0.113232958	0.138966915	-0.814819539	0.415175615	0.635422624	0.113232958
ENSMUSG00000021196	Pfkp	56421	protein_coding	2353.111196	-0.113283151	0.074848514	1.513498995	0.130152956	0.298087027	0.113283151
ENSMUSG00000087370	Tmem170b	621976	protein_coding	293.114318	0.113322522	0.121151919	-0.935375381	0.34959488	0.571435202	0.113322522
ENSMUSG00000041361	Myzap	102371	protein_coding	191.6829207	-0.113376109	0.247834175	0.457467616	0.647334974	0.808079957	0.113376109
ENSMUSG00000037868	Egr2	13654	protein_coding	76.93892788	-0.11343386	0.333746568	0.339880228	0.733946727	0.86401456	0.11343386
ENSMUSG00000079562	Maea	59003	protein_coding	1286.222438	0.113439989	0.049037974	-2.313309028	0.020705655	0.075334803	0.113439989
ENSMUSG00000038984	Tspyl5	239364	protein_coding	7.828824251	-0.113474161	0.422278605	0.268718708	0.788146154	0.894017228	0.113474161
ENSMUSG00000027347	Rasgrp1	19419	protein_coding	13.88361056	-0.113506371	0.4072895	0.278687201	0.780484885	0.890186044	0.113506371

ensembl_gene_id	external_gene_name	entrezgene_id	gene_biotype	baseMean	log2FoldChange	lfcSE	stat	pvalue	padj	absLog2FC
ENSMUSG00000033434	Gtpbp6	107999	protein_coding	94.45075298	-0.113547024	0.145309668	0.781414104	0.434558979	0.652884901	0.113547024
ENSMUSG00000018565	Elp5	54351	protein_coding	415.1402663	0.113549337	0.07221332	-1.572415401	0.115854245	0.274864513	0.113549337
ENSMUSG00000067714	Lpar5	381810	protein_coding	93.26879754	0.113568519	0.107892112	-1.052611881	0.29251891	0.511613776	0.113568519
ENSMUSG00000021749	Fam3d	18300	protein_coding	10609.73214	-0.113572816	0.181703794	0.625043724	0.531942361	0.728820184	0.113572816
ENSMUSG00000021794	Glud1	14661	protein_coding	2313.637158	-0.113577449	0.080185083	1.416441122	0.156646391	0.339527301	0.113577449
ENSMUSG00000002105	Slc39a13	68427	protein_coding	75.74068633	0.113584677	0.135044328	-0.841091801	0.400296508	0.622315002	0.113584677
ENSMUSG00000032333	Stoml1	69106	protein_coding	128.4566268	-0.113669354	0.118321317	0.960683641	0.336711259	0.559233675	0.113669354
ENSMUSG00000097403	9230116N13Rik	320054	lncRNA	3.565625583	-0.113767407	0.672198221	0.169246813	0.865602509	1	0.113767407
ENSMUSG00000013275	Slc41a1	98396	protein_coding	180.3469236	-0.113774259	0.134910327	0.843332465	0.399042531	0.621033677	0.113774259
ENSMUSG00000039100	Marchf6	223455	protein_coding	1420.184248	-0.113801951	0.064073343	1.776120087	0.075713126	0.201763202	0.113801951
ENSMUSG00000028861	Mrps15	66407	protein_coding	347.6677283	0.113838129	0.077458057	-1.469674472	0.14164994	0.316622511	0.113838129
ENSMUSG00000092558	Med20	56771	protein_coding	228.1130456	0.113863998	0.088287955	-1.289688926	0.197158686	0.397106786	0.113863998
ENSMUSG00000033307	Mif	17319	protein_coding	564.7395936	0.113891886	0.13887761	-0.820088173	0.412165844	0.632329199	0.113891886
ENSMUSG00000093661	Eif4e3	66892	protein_coding	66.25397919	0.113908239	0.250994496	-0.453827639	0.649952889	0.809917796	0.113908239
ENSMUSG00000080465	Snord94	115490493	snoRNA	145.4550709	0.113909207	0.266158246	-0.427975496	0.668668961	0.822256556	0.113909207
ENSMUSG00000025059	Gk	14933	protein_coding	736.5370709	-0.113919281	0.226923748	0.502015685	0.615656488	0.787858751	0.113919281
ENSMUSG00000023953	Polh	80905	protein_coding	164.1775412	0.114039097	0.130547561	-0.873544448	0.38236639	0.604711604	0.114039097
ENSMUSG00000030465	Psd3	234353	protein_coding	871.129074	-0.114063772	0.073543556	1.550968946	0.120909126	0.283500399	0.114063772
ENSMUSG00000029270	Dipk1a	67266	protein_coding	87.67492513	-0.114074408	0.241183012	0.47297862	0.636228433	0.801687789	0.114074408
ENSMUSG00000031708	Tecr	106529	protein_coding	1163.358896	0.114090558	0.062320764	-1.830698962	0.067145488	0.185177119	0.114090558
ENSMUSG00000046731	Kctd11	216858	protein_coding	95.40016898	-0.114094265	0.142822649	0.798852744	0.424375803	0.644012315	0.114094265
ENSMUSG00000025004	Cyp2c40	13099	protein_coding	2.154628473	0.114111188	0.948761118	-0.120273888	0.904266189	1	0.114111188
ENSMUSG00000030264	Thumpd3	14911	protein_coding	611.7116579	-0.114112816	0.075757161	1.506297407	0.131990843	0.301146282	0.114112816
ENSMUSG00000029642	Polr1d	20018	protein_coding	910.7901649	-0.114120605	0.083673596	1.363878334	0.172605861	0.363248781	0.114120605
ENSMUSG00000063382	Bcl9l	80288	protein_coding	173.8979565	-0.114123842	0.181530801	0.628674813	0.529561971	0.727166502	0.114123842
ENSMUSG00000039662	Icmt	57295	protein_coding	761.7155222	0.114265722	0.086300429	-1.324045814	0.18548783	0.380798859	0.114265722
ENSMUSG00000039686	Zer1	227693	protein_coding	255.2272495	-0.114276384	0.071704317	1.593716946	0.110999447	0.266624708	0.114276384
ENSMUSG00000031433	Rbm41	237073	protein_coding	70.60691746	-0.114287233	0.144389183	0.791522126	0.42863937	0.647625851	0.114287233
ENSMUSG00000053560	Ier2	15936	protein_coding	4317.336684	-0.114318763	0.113493143	1.007274624	0.313802821	0.535013399	0.114318763
ENSMUSG00000024797	Vps51	68505	protein_coding	441.7293096	-0.11433075	0.065131497	1.755383429	0.079193745	0.208527092	0.11433075
ENSMUSG00000048603	Gm9828	105246056	lncRNA	10.18873096	0.114351377	0.374142253	-0.305636094	0.759881735	0.87943703	0.114351377
ENSMUSG00000022824	Muc13	17063	protein_coding	15820.20597	-0.114360371	0.102898627	1.111388697	0.266401076	0.481401448	0.114360371
ENSMUSG00000034675	Dbn1	56320	protein_coding	12.64149407	-0.114365012	0.467219243	0.244778044	0.806628295	0.904874646	0.114365012
ENSMUSG00000021222	Dcaf4	73828	protein_coding	150.676184	-0.114392342	0.122143923	0.93653732	0.348996607	0.570683233	0.114392342
ENSMUSG00000021678	F2rl1	14063	protein_coding	1109.857099	0.114428297	0.074569262	-1.534523665	0.124900876	0.289855686	0.114428297
ENSMUSG00000086894	Gm15708	100504231	lncRNA	26.08976304	-0.114433063	0.265225117	0.431456359	0.666136578	0.820143104	0.114433063
ENSMUSG00000028514	Usp24	329908	protein_coding	1116.858959	0.114468715	0.098307058	-1.164399767	0.244262042	0.455584296	0.114468715
ENSMUSG00000009090	Ap1b1	11764	protein_coding	1548.938373	-0.114494244	0.063981463	1.789490886	0.073535794	0.197941337	0.114494244
ENSMUSG00000022667	Cd200r1	57781	protein_coding	5.961290507	-0.114557637	0.618171983	0.185316772	0.852980584	0.92788168	0.114557637

ensembl_gene_id	external_gene_name	entrezgene_id	gene_biotype	baseMean	log2FoldChange	lfcSE	stat	pvalue	padj	absLog2FC
ENSMUSG00000031059	Ndufb11	104130	protein_coding	1318.24369	0.114569112	0.085071082	-1.346745675	0.178062156	0.370529039	0.114569112
ENSMUSG00000040774	Cept1	99712	protein_coding	1387.327697	0.114645513	0.075016602	-1.528268535	0.126445879	0.292413842	0.114645513
ENSMUSG00000044469	Tnfaip8l1	66443	protein_coding	194.3668154	0.114711712	0.120766973	-0.949859958	0.342183415	0.564082441	0.114711712
ENSMUSG00000035506	Slc12a8	171286	protein_coding	1245.649984	0.114712342	0.206328342	-0.555969873	0.57823145	0.76196251	0.114712342
ENSMUSG00000108383	Gm6567	625243	lncRNA	1.861736789	-0.114761332	1.223286696	0.09381393	0.925256966	1	0.114761332
ENSMUSG00000004896	Rrnad1	229503	protein_coding	115.0814237	-0.114775717	0.130804386	0.877460765	0.380236421	0.602439245	0.114775717
ENSMUSG00000034216	Vps18	228545	protein_coding	297.1567281	-0.114780349	0.082350426	1.393803957	0.163376813	0.349482473	0.114780349
ENSMUSG00000073139	Tmem185a	236848	protein_coding	147.9911379	0.114795161	0.107107476	-1.071775432	0.28382091	0.502090896	0.114795161
ENSMUSG00000022805	Cfap91	320214	protein_coding	9.867286605	-0.114816954	0.458700333	0.250309289	0.802348173	0.90234117	0.114816954
ENSMUSG00000067931	Zfp948	381066	protein_coding	192.4283584	-0.114825162	0.098952685	1.160404709	0.24588407	0.457679163	0.114825162
ENSMUSG00000031314	Taf1	270627	protein_coding	834.5065612	0.114833907	0.094636799	-1.213417066	0.224970405	0.432209358	0.114833907
ENSMUSG00000047632	Fgfbp3	72514	protein_coding	10.58154737	-0.114985079	0.327546903	0.351049204	0.725551435	0.857956591	0.114985079
ENSMUSG00000027346	Gpcpd1	74182	protein_coding	438.2336428	-0.114987279	0.10159592	1.131810017	0.257714315	0.47089684	0.114987279
ENSMUSG00000014177	Tvp23b	67510	protein_coding	671.4679294	0.114997891	0.114328306	-1.005856682	0.314484516	0.535752227	0.114997891
ENSMUSG00000036863	Syde2	214804	protein_coding	201.0733423	0.11502852	0.083154984	-1.383302777	0.166572047	0.354161295	0.11502852
ENSMUSG00000001763	Tspan33	232670	protein_coding	202.2864137	0.115114444	0.183741297	-0.626502836	0.530985177	0.728048657	0.115114444
ENSMUSG00000000976	Heatr6	217026	protein_coding	295.0485245	0.115139903	0.061606986	-1.868942329	0.061630838	0.174007807	0.115139903
ENSMUSG00000024383	Map3k2	26405	protein_coding	828.4402793	-0.11516469	0.093399924	1.233027667	0.217565442	0.423388606	0.11516469
ENSMUSG00000031574	Star	20845	protein_coding	25.8581114	-0.115178679	0.218468482	0.527209591	0.598048049	0.775818184	0.115178679
ENSMUSG00000014195	Dnajc7	56354	protein_coding	882.2527227	-0.115263675	0.072151583	1.597521075	0.110149604	0.265200548	0.115263675
ENSMUSG00000020003	Pex7	18634	protein_coding	472.6262791	0.115365128	0.096394583	-1.196800938	0.231384153	0.440188363	0.115365128
ENSMUSG00000022636	Alcam	11658	protein_coding	571.9139148	0.115392704	0.165982849	-0.695208598	0.486924585	0.695624496	0.115392704
ENSMUSG00000034252	Senp6	215351	protein_coding	1198.325844	0.115410063	0.070870313	-1.628468374	0.10342561	0.253681814	0.115410063
ENSMUSG00000078429	Ctdsp2	52468	protein_coding	1552.978946	-0.115413094	0.07038142	1.639823335	0.101041905	0.249608668	0.115413094
ENSMUSG00000016942	Tmprss6	71753	protein_coding	4.911360699	0.115431761	0.717999424	-0.160768598	0.872275659	0.937028043	0.115431761
ENSMUSG00000004508	Gab2	14389	protein_coding	240.6721597	0.115462023	0.104616621	-1.103668062	0.269737155	0.484916108	0.115462023
ENSMUSG00000054823	Nsd3	234135	protein_coding	631.986976	0.115487159	0.096651878	-1.19487755	0.232134876	0.441179379	0.115487159
ENSMUSG00000031066	Usp11	236733	protein_coding	30.04660988	-0.115547236	0.21639164	0.533972737	0.593360396	0.772650496	0.115547236
ENSMUSG00000039294	Cybc1	217370	protein_coding	225.0308874	-0.115552216	0.078034219	1.480789035	0.138662799	0.311717339	0.115552216
ENSMUSG00000026270	Capn10	23830	protein_coding	297.0444992	0.115554644	0.072214275	-1.600163464	0.109562325	0.264291499	0.115554644
ENSMUSG00000027247	Arhgap1	228359	protein_coding	1469.622678	-0.115578096	0.09858117	1.172415538	0.241030264	0.451699249	0.115578096
ENSMUSG00000039114	Nrn1	68404	protein_coding	62.00897819	-0.115594917	0.190634555	0.60636917	0.544269636	0.736796542	0.115594917
ENSMUSG00000028779	Pef1	67898	protein_coding	890.0870871	-0.115597723	0.048745547	2.371451981	0.017718349	0.066683451	0.115597723
ENSMUSG00000074212	Dnajb14	70604	protein_coding	318.946203	0.115640608	0.092557623	-1.249390424	0.211522309	0.415941821	0.115640608
ENSMUSG00000025792	Slc25a10	27376	protein_coding	1972.588701	0.11565207	0.086708242	-1.333807111	0.18226708	0.376762869	0.11565207
ENSMUSG00000069744	Psmb3	26446	protein_coding	856.9587278	0.115730626	0.116874197	-0.99021537	0.322068861	0.543328754	0.115730626
ENSMUSG00000025616	Usp16	74112	protein_coding	583.9991483	0.115733642	0.074063138	-1.562634863	0.118138503	0.278825103	0.115733642
ENSMUSG00000049957	Ccdc137	67291	protein_coding	182.4960553	-0.115779276	0.088520239	1.307941289	0.190893224	0.388417088	0.115779276
ENSMUSG00000027387	Zc3h8	57432	protein_coding	30.61782197	-0.115784445	0.227937155	0.507966528	0.611476817	0.784792738	0.115784445

ensembl_gene_id	external_gene_name	entrezgene_id	gene_biotype	baseMean	log2FoldChange	lfcSE	stat	pvalue	padj	absLog2FC
ENSMUSG00000037916	Ndufv1	17995	protein_coding	2627.668385	-0.115785827	0.082568441	1.402301236	0.160825312	0.345591307	0.115785827
ENSMUSG00000027884	Clcc1	229725	protein_coding	363.1078127	0.115804554	0.139086531	-0.832607966	0.405065865	0.62627635	0.115804554
ENSMUSG00000028189	Ctbs	74245	protein_coding	105.8112336	-0.11581193	0.151220231	0.765849448	0.443765888	0.661197851	0.11581193
ENSMUSG00000039130	Zc3hc1	232679	protein_coding	112.1481715	-0.115835523	0.10178907	1.137995692	0.255122281	0.467705301	0.115835523
ENSMUSG00000013662	Atad1	67979	protein_coding	1148.638371	-0.1158555	0.091956841	1.259889952	0.207709064	0.410708835	0.1158555
ENSMUSG00000045730	Adrb2	11555	protein_coding	14.5949692	0.115864518	0.591396649	-0.195916765	0.844675315	0.923599766	0.115864518
ENSMUSG00000055917	Zfp277	246196	protein_coding	255.7402109	-0.115885444	0.083086998	1.394748233	0.163091774	0.349008366	0.115885444
ENSMUSG00000029169	Dhx15	13204	protein_coding	1713.059324	0.115947462	0.088372366	-1.312033026	0.189508991	0.386709513	0.115947462
ENSMUSG00000021945	Zmym2	76007	protein_coding	777.057143	-0.1159501	0.077328042	1.499457328	0.133755031	0.303828141	0.1159501
ENSMUSG00000015944	Castor2	80909	protein_coding	47.70126298	-0.115963604	0.368391492	0.31478361	0.752925957	0.876176397	0.115963604
ENSMUSG00000039735	Fnbp1l	214459	protein_coding	829.5247202	-0.115982013	0.069959091	1.657854783	0.097346781	0.242325103	0.115982013
ENSMUSG00000074344	Tmigd3	69296	protein_coding	1.278748165	-0.115986423	1.078347004	0.107559462	0.914345155	1	0.115986423
ENSMUSG00000050982	Apol10a	245282	protein_coding	2324.087725	-0.116012518	0.139307327	0.83278116	0.404968162	0.626183916	0.116012518
ENSMUSG00000021779	Thrb	21834	protein_coding	421.219062	-0.116073158	0.068133653	1.703609794	0.088454011	0.22586956	0.116073158
ENSMUSG00000034269	Setd5	72895	protein_coding	1717.700806	0.116073737	0.118632446	-0.978431624	0.327860894	0.549974786	0.116073737
ENSMUSG00000033192	Lpcat2	270084	protein_coding	186.8488295	0.116113613	0.186958493	-0.621066265	0.534556024	0.730517043	0.116113613
ENSMUSG00000045409	Trim39	79263	protein_coding	253.3546579	0.116114806	0.107075037	-1.08442461	0.278176577	0.495289271	0.116114806
ENSMUSG00000047098	Rnf31	268749	protein_coding	402.5241713	-0.11617086	0.148524927	0.78216406	0.434118163	0.652619695	0.11617086
ENSMUSG00000024575	Pde6a	225600	protein_coding	18.60275242	-0.116186329	0.396094897	0.293329526	0.769270287	0.884414475	0.116186329
ENSMUSG00000031730	Dhodh	56749	protein_coding	142.6438489	0.116202641	0.139303912	-0.834166388	0.404187229	0.625349723	0.116202641
ENSMUSG00000008384	Sertad1	55942	protein_coding	666.6342118	0.11621412	0.160226929	-0.725309541	0.468262154	0.681791678	0.11621412
ENSMUSG00000004535	Tax1bp1	52440	protein_coding	5914.090463	0.116229797	0.104051961	-1.117036104	0.263978889	0.47841828	0.116229797
ENSMUSG00000003038	Hmgn2	15331	protein_coding	542.1518034	0.116261581	0.112955261	-1.029271058	0.30335232	0.523793413	0.116261581
ENSMUSG00000045005	Fzd5	14367	protein_coding	2191.467994	-0.116268209	0.069748408	1.66696577	0.095521212	0.239041868	0.116268209
ENSMUSG00000027792	Bche	12038	protein_coding	553.0046021	-0.116289601	0.098613918	1.179241261	0.23830212	0.448589299	0.116289601
ENSMUSG00000036019	Tmtc2	278279	protein_coding	579.4166189	-0.11629577	0.085016099	1.367926436	0.171335104	0.361449656	0.11629577
ENSMUSG00000027284	Cdan1	68968	protein_coding	105.1097377	0.116305635	0.121301905	-0.958811285	0.337653823	0.559563944	0.116305635
ENSMUSG00000038593	Tctn1	654470	protein_coding	63.84356858	-0.116310211	0.184200523	0.631432581	0.527757719	0.725980089	0.116310211
ENSMUSG00000035234	Abraxas1	70681	protein_coding	91.51164593	0.116317973	0.108807399	-1.069026315	0.285057812	0.503470022	0.116317973
ENSMUSG00000018415	Gid4	66771	protein_coding	397.5020583	-0.116318888	0.098852018	1.176697154	0.239316404	0.449590928	0.116318888
ENSMUSG00000030689	Ino80e	233875	protein_coding	189.0051074	0.116375259	0.100714526	-1.155496274	0.247887258	0.460059583	0.116375259
ENSMUSG00000021748	Pdhb	68263	protein_coding	1143.439619	0.116428499	0.06214429	-1.873518841	0.060996774	0.172956854	0.116428499
ENSMUSG00000022024	Sugt1	67955	protein_coding	663.990884	-0.116444223	0.048639518	2.394025006	0.016664613	0.063600511	0.116444223
ENSMUSG00000094724	Rnaset2b	68195	protein_coding	8.024548272	0.116447158	0.450568929	-0.258444713	0.796063713	0.89873561	0.116447158
ENSMUSG00000037730	Mynn	80732	protein_coding	214.4702668	-0.116451578	0.082466151	1.412113649	0.157916501	0.341473496	0.116451578
ENSMUSG00000051375	Pcdh1	75599	protein_coding	1412.087919	-0.116452518	0.121819896	0.955940053	0.339102522	0.560532006	0.116452518
ENSMUSG00000024414	Mrpl27	94064	protein_coding	294.8358312	-0.116455745	0.087473034	1.331333085	0.183079437	0.377733062	0.116455745
ENSMUSG00000026965	Anapc2	99152	protein_coding	617.3986561	-0.116471856	0.062606122	1.860390855	0.062830248	0.176389198	0.116471856
ENSMUSG00000028670	Lypla2	26394	protein_coding	861.0476234	-0.11649178	0.082600136	1.410309787	0.15844823	0.342262172	0.11649178

ensembl_gene_id	external_gene_name	entrezgene_id	gene_biotype	baseMean	log2FoldChange	lfcSE	stat	pvalue	padj	absLog2FC
ENSMUSG00000008730	Hipk1	15257	protein_coding	1809.544795	-0.116502989	0.048688817	2.392807944	0.016719991	0.06376765	0.116502989
ENSMUSG00000031388	Naa10	56292	protein_coding	278.6029068	0.116512663	0.105989298	-1.09928705	0.271642879	0.487334112	0.116512663
ENSMUSG00000038013	Wipf2	68524	protein_coding	516.1103071	-0.116592734	0.130611111	0.892670875	0.372033452	0.594336299	0.116592734
ENSMUSG00000038607	Gng10	14700	protein_coding	7.810376033	0.116613596	0.365583681	-0.318979216	0.749742274	0.874258149	0.116613596
ENSMUSG00000024603	Dctn4	67665	protein_coding	898.4175777	-0.116671036	0.06915132	1.687184523	0.091567876	0.231605439	0.116671036
ENSMUSG00000022120	Obi1	72486	protein_coding	132.9310276	-0.116676029	0.123781641	0.942595589	0.345887797	0.567566681	0.116676029
ENSMUSG00000026730	Pter	19212	protein_coding	122.3086332	-0.116738343	0.179846349	0.649100432	0.516273462	0.717206457	0.116738343
ENSMUSG00000003131	Pafah1b2	18475	protein_coding	799.2895445	0.116760408	0.071109657	-1.641976817	0.100594809	0.248835031	0.116760408
ENSMUSG00000010392	Gosr1	53334	protein_coding	701.7600756	0.116766091	0.093911528	-1.243362705	0.213734207	0.418744149	0.116766091
ENSMUSG00000041921	Metap1d	66559	protein_coding	147.4012318	-0.116780087	0.099402294	1.174822854	0.240065595	0.450443439	0.116780087
ENSMUSG00000038622	Med30	69790	protein_coding	152.6073145	0.116797053	0.127647638	-0.914995802	0.360193834	0.582642414	0.116797053
ENSMUSG00000030982	Vps35l	71517	protein_coding	583.9463777	-0.116845196	0.054724801	2.135141565	0.032749448	0.108013659	0.116845196
ENSMUSG00000021686	Ap3b1	11774	protein_coding	1803.215302	-0.116868314	0.046646786	2.505388343	0.012231701	0.049808611	0.116868314
ENSMUSG00000033478	Fhip2a	226252	protein_coding	317.6267656	0.116869665	0.081985676	-1.425488832	0.154015946	0.335676301	0.116869665
ENSMUSG00000038152	5033430I15Rik	76000	lncRNA	19.58418481	0.116875952	0.282922738	-0.41310201	0.679531877	0.828120806	0.116875952
ENSMUSG00000080440	Gm25848	115488713	snoRNA	15.651527	0.116877118	0.388442298	-0.300886692	0.763500898	0.881386379	0.116877118
ENSMUSG00000032410	Xrn1	24127	protein_coding	587.0815179	-0.11691987	0.094088007	1.24266497	0.213991317	0.419000785	0.11691987
ENSMUSG00000029703	Lrwd1	71735	protein_coding	212.4697788	0.116938982	0.101325088	-1.154097012	0.248460398	0.460735958	0.116938982
ENSMUSG00000038650	Rnh1	107702	protein_coding	1399.213453	0.117007712	0.075252244	-1.554873378	0.119976229	0.2820338	0.117007712
ENSMUSG00000020456	Ogdh	18293	protein_coding	4470.084894	-0.117035974	0.059302786	1.973532488	0.048434927	0.145560397	0.117035974
ENSMUSG00000042680	Garem1	381126	protein_coding	340.7383349	-0.117049234	0.136944702	0.854718969	0.392706747	0.615439779	0.117049234
ENSMUSG00000015846	Rxra	20181	protein_coding	1391.706035	-0.117063044	0.083393063	1.403750391	0.160393191	0.344842229	0.117063044
ENSMUSG00000043131	Mob1a	232157	protein_coding	1566.708822	-0.117065781	0.109047234	1.073532784	0.283032138	0.501169049	0.117065781
ENSMUSG00000040146	Rgl3	71746	protein_coding	69.96124208	0.117066525	0.220930668	-0.529879016	0.596195816	0.774449596	0.117066525
ENSMUSG00000024002	Brd4	57261	protein_coding	440.0022091	0.117094102	0.189661309	-0.617385291	0.536980621	0.732746487	0.117094102
ENSMUSG00000032253	Phip	83946	protein_coding	837.0753281	0.117166738	0.084074491	-1.393606271	0.163436534	0.349519674	0.117166738
ENSMUSG00000063236	1110038F14Rik	117171	protein_coding	156.3978304	-0.117219621	0.08886809	1.319029375	0.187159292	0.383324885	0.117219621
ENSMUSG00000004100	Ppan	235036	protein_coding	239.966713	0.117230704	0.111712962	-1.049392135	0.293997677	0.513159947	0.117230704
ENSMUSG00000029128	Rab28	100972	protein_coding	301.8024362	0.117268377	0.075459412	-1.554058984	0.120170348	0.282329367	0.117268377
ENSMUSG00000028755	Cda	72269	protein_coding	190.8067967	0.117318272	0.169006493	-0.69416429	0.487579184	0.696047947	0.117318272
ENSMUSG00000026458	Ppfia4	68507	protein_coding	20.72674013	-0.11734245	0.437802158	0.268026202	0.78867915	0.894265208	0.11734245
ENSMUSG00000054400	Cklf	75458	protein_coding	59.49395383	0.117375929	0.145151217	-0.808645852	0.418718883	0.638714542	0.117375929
ENSMUSG00000031904	Slc7a6	330836	protein_coding	145.5871566	0.117412127	0.156613545	-0.749693314	0.453439435	0.669164362	0.117412127
ENSMUSG00000066191	Anks6	75691	protein_coding	121.596989	0.117446658	0.147013228	-0.79888497	0.424357115	0.644012315	0.117446658
ENSMUSG00000029781	Fkbp9	27055	protein_coding	888.5527658	0.117466263	0.15735523	-0.746503712	0.45536319	0.67085817	0.117466263
ENSMUSG00000031907	Zfp90	22751	protein_coding	59.4683702	-0.117482848	0.138761871	0.846650794	0.397189793	0.619083741	0.117482848
ENSMUSG00000038215	Cep44	382010	protein_coding	84.42305707	0.11749998	0.158961382	-0.739173117	0.459801883	0.674169253	0.11749998
ENSMUSG00000090038	Gm16573	102635191	lncRNA	4.696953068	-0.117531255	0.705392107	0.166618331	0.86767037	0.934257579	0.117531255
ENSMUSG00000068740	Celsr2	53883	protein_coding	58.76810813	-0.117587558	0.220090369	0.534269439	0.593155133	0.772538316	0.117587558

ensembl_gene_id	external_gene_name	entrezgene_id	gene_biotype	baseMean	log2FoldChange	lfcSE	stat	pvalue	padj	absLog2FC
ENSMUSG00000046230	Vps13a	271564	protein_coding	964.1414764	0.117614369	0.073939066	-1.590693195	0.111678639	0.26767214	0.117614369
ENSMUSG00000056763	Cspp1	211660	protein_coding	328.6503539	-0.11761941	0.108836965	1.080693588	0.279833436	0.49722072	0.11761941
ENSMUSG00000024780	Cdc37l1	67072	protein_coding	448.6978157	-0.117622086	0.09784758	1.202094992	0.229326726	0.438111017	0.117622086
ENSMUSG00000031827	Cotl1	72042	protein_coding	708.4557323	0.117632084	0.117335733	-1.002525664	0.316089778	0.536972183	0.117632084
ENSMUSG00000002660	Clpp	53895	protein_coding	356.6302607	-0.11763216	0.082946252	1.418173299	0.156140175	0.338874866	0.11763216
ENSMUSG00000058444	Map2k5	23938	protein_coding	161.1708463	0.117770691	0.098817107	-1.191804676	0.233337842	0.442718029	0.117770691
ENSMUSG00000033423	Eri3	140546	protein_coding	371.5135347	0.117799406	0.077235534	-1.525197021	0.127209963	0.293933865	0.117799406
ENSMUSG00000064341	mt-Nd1	17716	protein_coding	63758.75734	0.117803819	0.485753778	-0.242517555	0.808379156	0.905485545	0.117803819
ENSMUSG00000029198	Grpel1	17713	protein_coding	1025.287478	0.117816197	0.057687428	-2.042320151	0.041119785	0.128705861	0.117816197
ENSMUSG00000032582	Rbm6	19654	protein_coding	919.6861948	-0.117823004	0.054524689	2.160911061	0.03070221	0.102718051	0.117823004
ENSMUSG00000037949	Ano10	102566	protein_coding	869.1009443	-0.117844759	0.07504178	1.570388646	0.116324722	0.275528753	0.117844759
ENSMUSG00000041747	Utp15	105372	protein_coding	184.6651461	0.117862443	0.124773197	-0.944613475	0.344856245	0.566380511	0.117862443
ENSMUSG00000074405	Zfp865	319748	protein_coding	160.8252661	0.11789385	0.132393836	-0.890478388	0.373209068	0.595475802	0.11789385
ENSMUSG00000022174	Dad1	13135	protein_coding	828.5965287	0.117921429	0.123018612	-0.958565763	0.337777547	0.559613498	0.117921429
ENSMUSG00000055491	Pprc1	226169	protein_coding	220.3131817	0.117936481	0.1592947	-0.740366636	0.459077558	0.673388737	0.117936481
ENSMUSG00000027384	Ndufaf5	69487	protein_coding	145.3228924	0.117948536	0.09822441	-1.200806762	0.229826168	0.438404222	0.117948536
ENSMUSG00000024446	Rpp21	67676	protein_coding	205.3452701	-0.117961078	0.086538206	1.363109808	0.172847906	0.363480239	0.117961078
ENSMUSG00000018405	Mrm1	217038	protein_coding	140.4971331	-0.117980817	0.129030617	0.914362963	0.360526156	0.582866455	0.117980817
ENSMUSG00000020904	Cfap52	71860	protein_coding	17.76975557	-0.117987381	0.252994461	0.466363494	0.640955338	0.804026642	0.117987381
ENSMUSG00000054008	Ndst1	15531	protein_coding	715.4484963	-0.118005254	0.069785218	1.690977798	0.090841043	0.230331489	0.118005254
ENSMUSG00000028899	Taf12	66464	protein_coding	270.9071581	0.118007568	0.079725973	-1.480164659	0.138829309	0.31192207	0.118007568
ENSMUSG00000041347	Bdkrb1	12061	protein_coding	0.687309041	0.118021865	1.279262972	-0.092257704	0.926493292	1	0.118021865
ENSMUSG00000025521	Tmem192	73067	protein_coding	113.1960046	-0.118032662	0.15818115	0.746186651	0.455554671	0.670920346	0.118032662
ENSMUSG00000018809	Smyd4	319822	protein_coding	72.41245556	-0.118057828	0.166923474	0.707257197	0.47940666	0.690650055	0.118057828
ENSMUSG00000107002	0610012G03Rik	106264	protein_coding	416.943223	-0.118099356	0.078043327	1.513253727	0.130215222	0.298143983	0.118099356
ENSMUSG00000030522	Mtmr10	233315	protein_coding	206.1602176	-0.118101172	0.118304029	0.998285291	0.318141038	0.539479894	0.118101172
ENSMUSG00000097383	1500026H17Rik	69002	lncRNA	12.32945406	-0.118116826	0.400481309	0.294937176	0.768041872	0.883975099	0.118116826
ENSMUSG00000046523	Kctd4	67516	protein_coding	9.493713567	0.118120261	0.370891584	-0.318476519	0.750123503	0.874455706	0.118120261
ENSMUSG00000045302	Preb	50907	protein_coding	946.7595375	0.118122092	0.079783348	-1.480535667	0.13873035	0.311742141	0.118122092
ENSMUSG00000045625	Pigz	239827	protein_coding	2.52924997	-0.118132246	0.832262803	0.141941038	0.887126579	1	0.118132246
ENSMUSG00000005362	Crbn	58799	protein_coding	275.0400213	-0.11815933	0.091010512	1.298304193	0.194182826	0.392885958	0.11815933
ENSMUSG00000047907	Tshz2	228911	protein_coding	166.4024931	-0.118189928	0.096965327	1.21888857	0.222886489	0.429893421	0.118189928
ENSMUSG00000030067	Foxp1	108655	protein_coding	1295.992018	-0.118234223	0.101520213	1.164637268	0.244165852	0.455456329	0.118234223
ENSMUSG00000038260	Trpm4	68667	protein_coding	2943.158723	-0.118248885	0.12260455	0.964473873	0.334808401	0.557080699	0.118248885
ENSMUSG00000024963	Dnajc4	57431	protein_coding	247.0073784	-0.118255223	0.085018477	1.390935561	0.164244968	0.350538094	0.118255223
ENSMUSG00000032215	Rsl24d1	225215	protein_coding	163.2196902	-0.118272961	0.126262379	0.936723687	0.348900709	0.570683233	0.118272961
ENSMUSG00000028572	Hook1	77963	protein_coding	2309.312772	0.118286999	0.088847431	-1.331349689	0.183073976	0.377733062	0.118286999
ENSMUSG00000029490	Mfsd7a	243197	protein_coding	174.0801337	0.118345312	0.154312425	-0.766920173	0.443128979	0.660786267	0.118345312
ENSMUSG00000024177	Nme4	56520	protein_coding	52.31643567	0.118352001	0.15855138	-0.746458349	0.455390583	0.67085817	0.118352001

ensembl_gene_id	external_gene_name	entrezgene_id	gene_biotype	baseMean	log2FoldChange	lfcSE	stat	pvalue	padj	absLog2FC
ENSMUSG00000021218	Gdi2	14569	protein_coding	4521.497369	0.118373127	0.084091579	-1.407669222	0.159229039	0.343330529	0.118373127
ENSMUSG00000064368	mt-Nd6	17722	protein_coding	3380.479065	-0.118377272	0.377206165	0.313826452	0.753652853	0.876528151	0.118377272
ENSMUSG00000015787	Abo	80908	protein_coding	125.4478922	-0.118389905	0.292899417	0.404199866	0.686065753	0.832872319	0.118389905
ENSMUSG00000052533	Nup188	227699	protein_coding	343.9027707	0.118395703	0.085576255	-1.383511147	0.166508192	0.354116714	0.118395703
ENSMUSG00000033377	Palmd	114301	protein_coding	31.41619875	0.118444569	0.249348842	-0.475015517	0.634775913	0.800822518	0.118444569
ENSMUSG00000027630	Tbl1xr1	81004	protein_coding	1493.81903	0.118455264	0.077914494	-1.52032385	0.128429603	0.295635135	0.118455264
ENSMUSG00000020828	Pld2	18806	protein_coding	27.54689787	0.118481364	0.32583115	-0.363628105	0.716135737	0.852650725	0.118481364
ENSMUSG00000040621	Gemin8	237221	protein_coding	22.62203591	-0.118486233	0.244210855	0.485180045	0.627548636	0.796376973	0.118486233
ENSMUSG00000062082	Cd200r4	239849	protein_coding	4.777698601	-0.11850523	0.718501347	0.1649339	0.868996009	0.935258087	0.11850523
ENSMUSG00000062963	Ufc1	66155	protein_coding	851.9250555	0.118519353	0.097151374	-1.219945202	0.222485649	0.429533455	0.118519353
ENSMUSG00000051111	Sv2c	75209	protein_coding	61.14187876	0.118528846	0.178984264	-0.662230539	0.507823485	0.711603273	0.118528846
ENSMUSG00000037791	Phf12	268448	protein_coding	395.8222313	0.118560042	0.111980859	-1.058752747	0.289712399	0.508545065	0.118560042
ENSMUSG00000040351	Ankib1	70797	protein_coding	1219.921176	-0.118560419	0.078901033	1.502647225	0.132930038	0.30270362	0.118560419
ENSMUSG00000063765	Chadl	214685	protein_coding	6.697538576	0.118563965	0.398877232	-0.297244252	0.766280039	0.882690208	0.118563965
ENSMUSG00000039676	Capsl	75568	protein_coding	55.54902213	0.11857262	0.20712821	-0.572460022	0.567010363	0.753307251	0.11857262
ENSMUSG00000054892	Txk	22165	protein_coding	12.12931953	0.118604285	0.631785023	-0.187728865	0.851089202	0.927286869	0.118604285
ENSMUSG00000052656	Rnf103	22644	protein_coding	1274.858828	0.118628188	0.077747266	-1.52581812	0.127055167	0.293636687	0.118628188
ENSMUSG00000067942	Zfp160	224585	protein_coding	218.3979525	-0.118650409	0.091991179	1.289802025	0.197119405	0.397106786	0.118650409
ENSMUSG00000100632	9430060I03Rik	100037260	lncRNA	12.32560271	0.118676941	0.362389479	-0.32748451	0.743301457	0.870311987	0.118676941
ENSMUSG00000092203	1110038B12Rik	68763	lncRNA	572.1586308	-0.118687339	0.095018248	1.249100471	0.211628328	0.416065952	0.118687339
ENSMUSG00000042557	Sin3a	20466	protein_coding	658.1158104	0.118700745	0.072192442	-1.644226749	0.100129374	0.247943691	0.118700745
ENSMUSG00000001065	Zfp276	57247	protein_coding	291.906634	0.118799048	0.102571628	-1.158205731	0.24678009	0.458623273	0.118799048
ENSMUSG00000006732	Mettl1	17299	protein_coding	149.1692129	0.118805461	0.144968979	-0.819523332	0.412487896	0.632451088	0.118805461
ENSMUSG00000052512	Nav2	78286	protein_coding	241.3770995	-0.118814883	0.181621636	0.654189036	0.512990024	0.714956306	0.118814883
ENSMUSG00000048772	Tmem53	68777	protein_coding	56.47752859	-0.11883062	0.147373407	0.806323356	0.420056427	0.63998633	0.11883062
ENSMUSG00000031740	Mmp2	17390	protein_coding	7.797256383	-0.118846273	0.459922817	0.258404821	0.796094496	0.89873561	0.118846273
ENSMUSG00000037536	Fbxo34	78938	protein_coding	448.8205931	0.118860047	0.104505453	-1.137357362	0.255388924	0.468075728	0.118860047
ENSMUSG00000028789	Azin2	242669	protein_coding	11.0550222	-0.118968129	0.623489119	0.190810272	0.84867424	0.925637253	0.118968129
ENSMUSG00000023286	Ube2j2	140499	protein_coding	580.4621	-0.119096763	0.074014502	1.609100372	0.107594396	0.26091246	0.119096763
ENSMUSG00000024451	Arap3	106952	protein_coding	12.45013857	-0.119106285	0.634197434	0.187806318	0.851028483	0.927281893	0.119106285
ENSMUSG00000027848	Olfml3	99543	protein_coding	4.033768734	0.11915205	0.641218864	-0.185821186	0.85258499	1	0.11915205
ENSMUSG00000047714	Ppp1r2	66849	protein_coding	601.1592578	-0.119153807	0.085496128	1.393674901	0.163415799	0.349519674	0.119153807
ENSMUSG00000030835	Nomo1	211548	protein_coding	1342.110227	0.119157169	0.070876324	-1.681198503	0.092724364	0.233671624	0.119157169
ENSMUSG00000027438	Napb	17957	protein_coding	25.46144702	-0.119175787	0.203479344	0.585689852	0.558083955	0.747120423	0.119175787
ENSMUSG00000031353	Rbbp7	245688	protein_coding	1492.146113	0.11922535	0.097694419	-1.220390597	0.22231684	0.429396526	0.11922535
ENSMUSG00000043051	Disc1	244667	protein_coding	1.647001954	-0.119284033	1.171645512	0.101808979	0.918908299	1	0.119284033
ENSMUSG00000067713	Prkag1	19082	protein_coding	355.1301635	0.119293576	0.074649018	-1.598059541	0.110029727	0.265066507	0.119293576
ENSMUSG00000030881	Arfip2	76932	protein_coding	712.2689597	0.119326817	0.073651736	-1.620149417	0.105200184	0.256689693	0.119326817
ENSMUSG00000041471	Shld2	75698	protein_coding	67.45092635	0.119389968	0.135083199	-0.883825444	0.376790449	0.598990901	0.119389968

ensembl_gene_id	external_gene_name	entrezgene_id	gene_biotype	baseMean	log2FoldChange	lfcSE	stat	pvalue	padj	absLog2FC
ENSMUSG00000040952	Rps19	20085	protein_coding	5195.67392	0.119461886	0.113095763	-1.056289661	0.290835898	0.509749949	0.119461886
ENSMUSG00000010751	Tnfrsf22	79202	protein_coding	5.914618913	-0.119498811	0.662440323	0.180391814	0.856844981	0.93035707	0.119498811
ENSMUSG00000001794	Capns1	12336	protein_coding	2228.855101	-0.119510108	0.076405804	1.564149613	0.117782437	0.278183519	0.119510108
ENSMUSG00000027377	Mall	228576	protein_coding	1223.183997	-0.119540049	0.123089029	0.971167375	0.331464937	0.553861375	0.119540049
ENSMUSG00000021134	Srsf5	20384	protein_coding	3080.480499	-0.119557357	0.081651806	1.464234084	0.14313	0.319067066	0.119557357
ENSMUSG00000048200	Cracr2b	213573	protein_coding	192.2015774	-0.11961301	0.186498613	0.641361393	0.52128791	0.720922137	0.11961301
ENSMUSG00000022544	Eef2kmt	70511	protein_coding	167.7158871	-0.119614751	0.115550138	1.035176181	0.30058664	0.52065112	0.119614751
ENSMUSG00000049775	Tmsb4x	19241	protein_coding	3622.360051	-0.119621311	0.074000686	1.616489226	0.105988581	0.258193749	0.119621311
ENSMUSG00000029592	Usp30	100756	protein_coding	224.949513	0.119633671	0.085461433	-1.399855667	0.161556544	0.346538122	0.119633671
ENSMUSG00000048905	Bnip5	207819	protein_coding	1137.465037	-0.119815796	0.347984154	0.344313941	0.730610201	0.861253255	0.119815796
ENSMUSG00000037025	Foxa2	15376	protein_coding	325.9542937	0.119831409	0.233220712	-0.513811182	0.60738401	0.781981914	0.119831409
ENSMUSG00000021583	Erap1	80898	protein_coding	1249.550482	-0.119865215	0.09419163	1.272567577	0.20317153	0.40541018	0.119865215
ENSMUSG00000071550	Cfap44	212517	protein_coding	10.96760611	0.119942121	0.53478481	-0.224281092	0.822538588	0.910171686	0.119942121
ENSMUSG00000008348	Ubc	22190	protein_coding	1413.173933	-0.119945677	0.089448994	1.340939364	0.17994014	0.373495661	0.119945677
ENSMUSG00000032422	Snx14	244962	protein_coding	534.4327796	-0.119972533	0.08493576	1.412509094	0.157800115	0.341333371	0.119972533
ENSMUSG00000021559	Dapk1	69635	protein_coding	203.2765934	-0.11999038	0.085698218	1.400150225	0.161468338	0.346538122	0.11999038
ENSMUSG00000038507	Parp12	243771	protein_coding	874.2743617	-0.120017362	0.131216636	0.914650503	0.360375137	0.582711741	0.120017362
ENSMUSG00000046541	Zfp526	210172	protein_coding	159.9761532	0.120044788	0.098771458	-1.215379318	0.224221452	0.431309617	0.120044788
ENSMUSG00000039852	Rere	68703	protein_coding	748.2248829	-0.12011419	0.158937564	0.755731921	0.449809942	0.666621321	0.12011419
ENSMUSG00000040987	Mill2	243864	protein_coding	56.37309173	-0.120201425	0.198365487	0.605959369	0.544541734	0.736796542	0.120201425
ENSMUSG00000039115	Itga9	104099	protein_coding	4.771444485	0.12026515	0.976585302	-0.123148638	0.901989397	0.951530343	0.12026515
ENSMUSG00000057110	Cntrl	26920	protein_coding	817.6802486	-0.120297732	0.096940803	1.240940123	0.214627869	0.419600407	0.120297732
ENSMUSG00000004667	Polr2e	66420	protein_coding	464.7758358	-0.120314898	0.063756869	1.887089187	0.059148331	0.169109338	0.120314898
ENSMUSG00000056735	A930024E05Rik	109202	lncRNA	6.847511813	0.120328276	0.40499567	-0.297110032	0.766382506	0.882690208	0.120328276
ENSMUSG00000027896	Slc16a4	229699	protein_coding	11.60240637	0.120340998	0.333182965	-0.361185927	0.717960461	0.853653903	0.120340998
ENSMUSG00000014551	Mrps25	64658	protein_coding	272.3355759	-0.120344483	0.084053036	1.431768428	0.152210106	0.332794609	0.120344483
ENSMUSG00000055301	Adh7	11529	protein_coding	4.681506268	-0.120349597	0.512480856	0.234837254	0.814335051	0.907710402	0.120349597
ENSMUSG00000031399	Fam3a	66294	protein_coding	367.3257733	0.120371699	0.071480857	-1.683971116	0.092187251	0.232815456	0.120371699
ENSMUSG00000022744	Cldnd1	224250	protein_coding	274.2034426	-0.120434094	0.098015418	1.228726014	0.219174549	0.425467087	0.120434094
ENSMUSG00000032376	Usp3	235441	protein_coding	1010.131598	-0.120475591	0.071296141	1.689791198	0.091067908	0.23065881	0.120475591
ENSMUSG00000033596	Rfwd3	234736	protein_coding	563.8760748	0.120490053	0.104498237	-1.153034307	0.248896303	0.461051436	0.120490053
ENSMUSG00000028962	Slc4a2	20535	protein_coding	2677.040284	-0.120491158	0.132016723	0.912696169	0.361402359	0.583801091	0.120491158
ENSMUSG00000022000	Zc3h13	67302	protein_coding	316.7294425	0.120506834	0.079191311	-1.52171789	0.128079783	0.295198531	0.120506834
ENSMUSG00000052658	5830454E08Rik	76100	lncRNA	6.960215435	0.120569639	0.494775201	-0.243685696	0.807474253	0.90502442	0.120569639
ENSMUSG00000071337	Tia1	21841	protein_coding	335.1727928	0.120621319	0.089203226	-1.352208038	0.176308773	0.368268232	0.120621319
ENSMUSG00000003031	Cdkn1b	12576	protein_coding	780.1322636	-0.120642609	0.073560686	1.640041913	0.100996453	0.249566874	0.120642609
ENSMUSG00000032340	Neo1	18007	protein_coding	1167.688291	-0.120660439	0.069605616	1.733487128	0.083009123	0.215774069	0.120660439
ENSMUSG00000024957	Kcnk4	16528	protein_coding	6.670262305	0.120662689	0.509744232	-0.236712221	0.812880054	0.907147741	0.120662689
ENSMUSG00000040363	Bcor	71458	protein_coding	600.4378349	0.12071086	0.068800668	-1.754501284	0.07934465	0.208654479	0.12071086

ensembl_gene_id	external_gene_name	entrezgene_id	gene_biotype	baseMean	log2FoldChange	lfcSE	stat	pvalue	padj	absLog2FC
ENSMUSG00000051950	B3glct	381694	protein_coding	181.5499657	-0.120711225	0.105587731	1.143231542	0.252942469	0.465324602	0.120711225
ENSMUSG00000024125	Sbpl	638345	protein_coding	2.266870592	0.120754529	0.919508761	-0.131325045	0.89551818	1	0.120754529
ENSMUSG00000027102	Hoxd8	15437	protein_coding	28.29488974	-0.120769856	0.337600645	0.357729932	0.720545431	0.855204229	0.120769856
ENSMUSG00000056737	Capg	12332	protein_coding	499.9260518	0.12082473	0.16149399	-0.748168586	0.454358477	0.669875536	0.12082473
ENSMUSG00000006731	B4galnt1	14421	protein_coding	1147.660956	0.12084814	0.097823891	-1.235364278	0.216694961	0.422162995	0.12084814
ENSMUSG00000037808	Fam76b	72826	protein_coding	289.3586737	0.120955736	0.086025492	-1.406045273	0.159710682	0.343912139	0.120955736
ENSMUSG00000021116	Eif2s1	13665	protein_coding	710.1420045	0.120968618	0.082551804	-1.465366136	0.142821051	0.318636429	0.120968618
ENSMUSG00000020300	Cpeb4	67579	protein_coding	1115.494207	-0.120981286	0.080521586	1.50247024	0.132975708	0.302752759	0.120981286
ENSMUSG00000023266	Frs3	107971	protein_coding	24.9503182	-0.120983524	0.219997699	0.549930864	0.582366794	0.765213657	0.120983524
ENSMUSG00000028293	Slc35a1	24060	protein_coding	2225.243424	0.120984115	0.174801669	-0.692122196	0.488860593	0.696638145	0.120984115
ENSMUSG00000004677	Myo9b	17925	protein_coding	616.6139027	0.120989033	0.107309334	-1.127479109	0.259539968	0.472954995	0.120989033
ENSMUSG00000042659	Arrdc4	66412	protein_coding	988.0380608	0.12106909	0.101043745	-1.19818491	0.230845039	0.439494463	0.12106909
ENSMUSG00000038797	Zscan2	22691	protein_coding	33.10194188	-0.12115792	0.210436665	0.575745295	0.564787355	0.751588072	0.12115792
ENSMUSG00000033400	Agl	77559	protein_coding	357.8835194	0.121166637	0.109025228	-1.111363297	0.266412004	0.481401448	0.121166637
ENSMUSG00000051346	Spryd4	66701	protein_coding	196.3296636	-0.121172183	0.1528622	0.792688987	0.427959048	0.647057325	0.121172183
ENSMUSG00000024277	Mapre2	212307	protein_coding	832.7077104	-0.121181381	0.056827586	2.132439351	0.032970745	0.108526787	0.121181381
ENSMUSG00000013878	Rnf170	77733	protein_coding	192.6777183	0.121181835	0.102553572	-1.181644207	0.237346905	0.44769238	0.121181835
ENSMUSG00000078440	Dohh	102115	protein_coding	98.27667242	0.121196264	0.115987414	-1.044908746	0.296065147	0.515232502	0.121196264
ENSMUSG00000020271	Fbxw11	103583	protein_coding	874.9049676	-0.121203179	0.071157489	1.703308837	0.088510288	0.225948198	0.121203179
ENSMUSG00000036568	Bicral	210982	protein_coding	323.4636603	-0.121214157	0.079255765	1.529404911	0.126164096	0.292089426	0.121214157
ENSMUSG00000040325	Dcaf1	321006	protein_coding	700.3522082	0.121218801	0.071038747	-1.706375827	0.087938128	0.225148876	0.121218801
ENSMUSG00000048410	Zfp407	240476	protein_coding	400.5667338	0.121220315	0.099039643	-1.223957511	0.220968256	0.427441699	0.121220315
ENSMUSG00000049588	Ccdc69	52570	protein_coding	4.935166174	-0.121250271	0.488219569	0.248351929	0.803862124	0.903305601	0.121250271
ENSMUSG00000022889	Mrpl39	27393	protein_coding	404.7960814	-0.121257537	0.080214881	1.51165888	0.130620664	0.298680373	0.121257537
ENSMUSG00000022477	Aco2	11429	protein_coding	6383.475493	-0.121270551	0.080588691	1.504808542	0.132373307	0.301713259	0.121270551
ENSMUSG00000072572	Slc39a2	214922	protein_coding	5.398220294	0.121299615	0.609390043	-0.199050865	0.842222956	0.921900136	0.121299615
ENSMUSG00000039193	Nlrc4	268973	protein_coding	696.4504825	-0.121349191	0.074322985	1.632727643	0.102526291	0.252139861	0.121349191
ENSMUSG00000022404	Slc25a17	20524	protein_coding	528.7877429	-0.121354717	0.098325957	1.234208351	0.217125276	0.422731291	0.121354717
ENSMUSG00000021606	Ndufs6	407785	protein_coding	945.0829601	0.121360166	0.091709288	-1.323313795	0.185731046	0.381203392	0.121360166
ENSMUSG00000028798	Eif3i	54709	protein_coding	1555.300578	-0.121479156	0.106985611	1.135471902	0.256177648	0.468911293	0.121479156
ENSMUSG00000049658	Bdp1	544971	protein_coding	828.0313123	0.121528755	0.073012668	-1.66448861	0.096014825	0.239840996	0.121528755
ENSMUSG00000030876	Mettl9	59052	protein_coding	761.3448473	0.121541636	0.090112731	-1.348773205	0.177409824	0.36973067	0.121541636
ENSMUSG00000037337	Map4k1	26411	protein_coding	16.09114518	0.121541913	0.447374033	-0.271678515	0.785869223	0.892991423	0.121541913
ENSMUSG00000026790	Odf2	18286	protein_coding	396.2724986	0.121543239	0.088395194	-1.374998274	0.16913198	0.358358419	0.121543239
ENSMUSG00000021361	Tmem14c	66154	protein_coding	1033.824755	-0.121589799	0.090224873	1.347630589	0.177777227	0.370076028	0.121589799
ENSMUSG00000022440	C1qtnf6	72709	protein_coding	226.592101	0.121669318	0.131494071	-0.925283684	0.354818364	0.577401503	0.121669318
ENSMUSG00000014633	Cmc2	66531	protein_coding	155.4183076	0.121703439	0.109183334	-1.114670476	0.264991657	0.479832481	0.121703439
ENSMUSG00000014763	Fam120b	67544	protein_coding	639.8427545	0.121739307	0.047223197	-2.577955631	0.009938676	0.042377303	0.121739307
ENSMUSG00000043090	Zfp866	330788	protein_coding	169.2176307	-0.121813501	0.148089851	0.822564819	0.410755512	0.630971948	0.121813501

ensembl_gene_id	external_gene_name	entrezgene_id	gene_biotype	baseMean	log2FoldChange	lfcSE	stat	pvalue	padj	absLog2FC
ENSMUSG00000031378	Abcd1	11666	protein_coding	317.6248089	-0.121829149	0.071404649	1.706179499	0.087974665	0.22515016	0.121829149
ENSMUSG00000085112	A530072M11Rik	100415915	lncRNA	20.57573978	0.12187632	0.234746851	-0.519181917	0.603633888	0.779177919	0.12187632
ENSMUSG00000031467	Agpat5	52123	protein_coding	584.2269825	-0.121884186	0.108951483	1.11870148	0.263267514	0.477583041	0.121884186
ENSMUSG00000004568	Arhgef18	102098	protein_coding	721.9985317	-0.121912206	0.071083211	1.715063282	0.086333625	0.222142956	0.121912206
ENSMUSG00000008763	Man1a2	17156	protein_coding	1972.564438	0.121932418	0.081245985	-1.500780835	0.133412257	0.303216352	0.121932418
ENSMUSG00000019777	Hdac2	15182	protein_coding	531.090065	0.121951241	0.081180434	-1.502224554	0.133039126	0.302752759	0.121951241
ENSMUSG00000020463	Ppp4r3b	104570	protein_coding	1234.375528	0.121953317	0.06233527	-1.956409548	0.050416928	0.149799416	0.121953317
ENSMUSG00000079173	Zan	22635	protein_coding	1169.427704	-0.121984913	0.137997327	0.883965769	0.376714692	0.598928123	0.121984913
ENSMUSG00000040147	Maob	109731	protein_coding	356.5946709	0.121998628	0.151364656	-0.805991513	0.420247743	0.640159692	0.121998628
ENSMUSG00000068587	Mgam	232714	protein_coding	6147.07189	0.122116725	0.096327713	-1.26772163	0.204897378	0.407353559	0.122116725
ENSMUSG00000051124	Gimap9	317758	protein_coding	73.78849664	0.122201742	0.149759904	-0.815984378	0.41450907	0.634755452	0.122201742
ENSMUSG00000024404	Riok3	66878	protein_coding	1534.01495	0.122375271	0.049236253	-2.485470863	0.012938018	0.05194233	0.122375271
ENSMUSG00000039813	Tbc1d2	381605	protein_coding	809.961464	0.12237575	0.107423841	-1.139186132	0.254625529	0.467157647	0.12237575
ENSMUSG00000079610	Ankrd39	109346	protein_coding	47.64952645	0.122409833	0.169803273	-0.720892069	0.470975923	0.684066582	0.122409833
ENSMUSG00000010057	Nprl2	56032	protein_coding	189.844649	-0.122435425	0.101844889	1.202175446	0.22929556	0.438111017	0.122435425
ENSMUSG00000019987	Arg1	11846	protein_coding	10.52819663	0.122545771	0.324613173	-0.377513241	0.705792226	0.845823862	0.122545771
ENSMUSG00000022299	Slc25a32	69906	protein_coding	285.7645602	0.122573297	0.080812844	-1.516755148	0.129328515	0.29700057	0.122573297
ENSMUSG00000037683	Armc3	70882	protein_coding	10.16162001	-0.122600446	0.451201594	0.271719887	0.785837409	0.892991423	0.122600446
ENSMUSG00000044676	Zfp612	234725	protein_coding	135.1671635	0.122614267	0.130246586	-0.941401008	0.346499397	0.568175062	0.122614267
ENSMUSG00000028347	Tmeff1	230157	protein_coding	123.5380524	-0.122641723	0.169984215	0.721488893	0.470608772	0.683821668	0.122641723
ENSMUSG00000022561	Gpaa1	14731	protein_coding	538.2218699	-0.122758173	0.084663592	1.44995234	0.14707181	0.324959041	0.122758173
ENSMUSG00000031891	Hsd11b2	15484	protein_coding	2275.465186	-0.122818186	0.202389382	0.606841052	0.543956402	0.73666903	0.122818186
ENSMUSG00000028797	Tmem234	76799	protein_coding	940.7174323	-0.122849592	0.06986518	1.758380817	0.078682735	0.207566563	0.122849592
ENSMUSG00000022641	Bbx	70508	protein_coding	467.6579809	-0.122868724	0.103645338	1.185472756	0.23583058	0.445952845	0.122868724
ENSMUSG00000024054	Smchd1	74355	protein_coding	816.9455969	0.122886226	0.077729797	-1.580941033	0.113891521	0.271186338	0.122886226
ENSMUSG00000032293	Ireb2	64602	protein_coding	598.023779	-0.122889577	0.219537187	0.559766562	0.575638674	0.760792082	0.122889577
ENSMUSG00000000838	Fmr1	14265	protein_coding	690.0949544	0.122891754	0.081573146	-1.506522192	0.131933175	0.30108314	0.122891754
ENSMUSG00000022809	Nr1i2	18171	protein_coding	973.9203917	-0.122893928	0.073960425	1.661617394	0.096589513	0.240948524	0.122893928
ENSMUSG00000007815	Rhoa	11848	protein_coding	2563.817113	-0.122896041	0.08986591	1.367549064	0.17145327	0.361606551	0.122896041
ENSMUSG00000025199	Chuk	12675	protein_coding	1064.376508	0.122947246	0.068373493	-1.798171203	0.072149882	0.195389167	0.122947246
ENSMUSG00000033644	Piwil2	57746	protein_coding	8.089036415	0.122973564	0.453094625	-0.271408127	0.786077152	0.893150482	0.122973564
ENSMUSG00000056130	Ticam2	225471	protein_coding	4.09464322	-0.123072428	0.675032683	0.182320696	0.855331058	1	0.123072428
ENSMUSG00000018076	Med13l	76199	protein_coding	685.8742953	0.12311756	0.131385594	-0.937070463	0.348722314	0.570573694	0.12311756
ENSMUSG00000024413	Npc1	18145	protein_coding	451.3867505	-0.123126854	0.123578938	0.996341739	0.31908413	0.540502136	0.123126854
ENSMUSG00000028360	Slc44a5	242259	protein_coding	3.988389921	0.123137285	1.028455698	-0.119730276	0.904696816	1	0.123137285
ENSMUSG00000026858	Miga2	108958	protein_coding	655.4814443	0.123140639	0.08095351	-1.521127848	0.128227757	0.295417576	0.123140639
ENSMUSG00000099759	1700030C10Rik	69513	lncRNA	16.87164499	0.123267501	0.295222678	-0.417540758	0.676282918	0.826766769	0.123267501
ENSMUSG00000078515	Ddi2	68817	protein_coding	1399.41296	0.123297721	0.059252699	-2.08087941	0.037444945	0.119675987	0.123297721
ENSMUSG00000018547	Pip4k2b	108083	protein_coding	124.8194004	0.123300656	0.114344537	-1.078325729	0.280888419	0.498558829	0.123300656

Supplementary Table 1

ensembl_gene_id	external_gene_name	entrezgene_id	gene_biotype	baseMean	log2FoldChange	lfcSE	stat	pvalue	padj	absLog2FC
ENSMUSG00000019373	Cops3	26572	protein_coding	574.9497688	0.123320868	0.064724864	-1.905309041	0.056739911	0.163972315	0.123320868
ENSMUSG00000049625	Tifab	212937	protein_coding	2.632548469	-0.123353022	1.22815353	0.100437786	0.919996772	1	0.123353022
ENSMUSG00000030967	Zranb1	360216	protein_coding	628.0893306	-0.123402115	0.08902304	1.386181765	0.165691408	0.352762472	0.123402115
ENSMUSG00000013150	Gfod2	70575	protein_coding	210.927637	-0.123412521	0.119122444	1.036014012	0.300195606	0.520301242	0.123412521
ENSMUSG00000061758	Akr1b10	67861	protein_coding	66.39966856	-0.12348973	0.124115995	0.994954198	0.319758541	0.541034072	0.12348973
ENSMUSG00000024335	Brd2	14312	protein_coding	2456.713081	0.123490018	0.104950805	-1.1766467	0.23933655	0.449590928	0.123490018
ENSMUSG00000021973	Micu2	68514	protein_coding	767.5458014	-0.123499749	0.06884398	1.793907751	0.072827894	0.19654843	0.123499749
ENSMUSG00000028149	Rap1gds1	229877	protein_coding	607.6853407	-0.123585188	0.07356977	1.679836533	0.092989125	0.234124471	0.123585188
ENSMUSG00000015143	Actn1	109711	protein_coding	1269.54438	-0.123598939	0.108109828	1.143271996	0.252925678	0.465324602	0.123598939
ENSMUSG00000026632	Tatdn3	68972	protein_coding	58.87668535	0.123685226	0.150037208	-0.824363689	0.40973294	0.630279329	0.123685226
ENSMUSG00000090935	Synj2bp	24071	protein_coding	744.1883865	-0.123750917	0.103647982	1.193953954	0.232495981	0.441568559	0.123750917
ENSMUSG00000002617	Zfp40	22700	protein_coding	58.81674738	0.123761023	0.154703459	-0.799988726	0.423717329	0.643604486	0.123761023
ENSMUSG00000030549	Rhcg	56315	protein_coding	1.423525057	0.123811418	2.325279831	-0.053245814	0.957536053	1	0.123811418
ENSMUSG00000089862	Umad1	100036521	protein_coding	259.4510169	0.123929279	0.161240185	-0.768600447	0.44213054	0.659918993	0.123929279
ENSMUSG00000033499	Larp4b	217980	protein_coding	1915.027239	-0.123935258	0.066747456	1.856778744	0.063342639	0.177475457	0.123935258
ENSMUSG00000030557	Mef2a	17258	protein_coding	344.3861875	0.123936123	0.074205153	-1.670182157	0.094883329	0.237733773	0.123936123
ENSMUSG00000053332	Gas5	14455	lncRNA	969.2952358	-0.123945791	0.079958964	1.550117515	0.121113313	0.283858253	0.123945791
ENSMUSG00000057963	Itpk1	217837	protein_coding	2356.349752	0.123958487	0.12083528	-1.025846809	0.304963798	0.525093542	0.123958487
ENSMUSG00000024805	Pcgf5	76073	protein_coding	333.5506832	-0.123974174	0.149050204	0.831761184	0.405543757	0.626487335	0.123974174
ENSMUSG00000020899	Pfas	237823	protein_coding	440.8823239	-0.123984889	0.116921248	1.060413661	0.28895645	0.507895701	0.123984889
ENSMUSG00000091091	Kcnmb3	100502876	protein_coding	2.515263353	0.124025873	0.929261951	-0.133467073	0.893824003	1	0.124025873
ENSMUSG00000015501	Hivep2	15273	protein_coding	406.3929505	0.124051037	0.130760552	-0.948688536	0.342779046	0.564822707	0.124051037
ENSMUSG00000058174	Gm5148	381438	protein_coding	13.91959111	-0.124057468	0.324603421	0.382181642	0.702326638	0.84294636	0.124057468
ENSMUSG00000022897	Dyrk1a	13548	protein_coding	898.2351066	0.124065643	0.043496662	-2.852302602	0.004340376	0.021615492	0.124065643
ENSMUSG00000026926	Pmpca	66865	protein_coding	707.5510641	0.124067114	0.061925632	-2.003485619	0.04512519	0.137999515	0.124067114
ENSMUSG00000042063	Zfp386	56220	protein_coding	305.9727509	-0.12408917	0.106449519	1.16570907	0.243732092	0.454904132	0.12408917
ENSMUSG00000021597	Slf1	105377	protein_coding	179.8872853	0.124133808	0.096433017	-1.28725422	0.198005695	0.398279073	0.124133808
ENSMUSG00000001948	Spa17	20686	protein_coding	8.922361452	0.12415305	0.315941853	-0.392961709	0.694347763	0.837885199	0.12415305
ENSMUSG00000047617	Paxx	227622	protein_coding	109.7005956	-0.124189245	0.100371753	1.237292774	0.215978409	0.421243351	0.124189245
ENSMUSG00000027166	Dnajc24	99349	protein_coding	83.8747143	0.124214846	0.146609468	-0.847249825	0.396855887	0.618855346	0.124214846
ENSMUSG00000018507	Trpv2	22368	protein_coding	4.520845049	0.124246983	0.899544461	-0.138122115	0.890143908	0.946057182	0.124246983
ENSMUSG00000062115	Rai1	19377	protein_coding	269.6462434	0.124276318	0.143860218	-0.863868551	0.387660105	0.610103781	0.124276318
ENSMUSG00000024826	Dpf2	19708	protein_coding	439.9365561	-0.124304533	0.058902062	2.110359616	0.034827392	0.113038434	0.124304533
ENSMUSG00000013465	Nelfb	58202	protein_coding	802.7123969	-0.124437344	0.047231856	2.634606244	0.008423494	0.037065168	0.124437344
ENSMUSG00000029406	Pitpnm2	19679	protein_coding	58.36391149	-0.124438768	0.285363136	0.436071631	0.662784759	0.817662297	0.124438768
ENSMUSG00000028552	Eps15	13858	protein_coding	573.1905474	-0.124482976	0.09550139	1.303467685	0.192415142	0.390165244	0.124482976
ENSMUSG00000014498	Ankrd52	237615	protein_coding	596.7664929	0.124538692	0.102703429	-1.212604995	0.225280878	0.432685604	0.124538692
ENSMUSG00000022014	Epsti1	108670	protein_coding	498.0470268	-0.124540569	0.085653905	1.453997565	0.145946981	0.323295567	0.124540569
ENSMUSG00000067028	Cntnap5b	241175	protein_coding	2.620401536	0.124562745	2.358344032	-0.052817885	0.957877011	1	0.124562745

ensembl_gene_id	external_gene_name	entrezgene_id	gene_biotype	baseMean	log2FoldChange	lfcSE	stat	pvalue	padj	absLog2FC
ENSMUSG00000007216	Zfp775	243372	protein_coding	90.80748979	0.12457221	0.128987261	-0.965771422	0.334158572	0.5565041	0.12457221
ENSMUSG00000056919	Cep162	382090	protein_coding	200.064871	0.124579968	0.082016952	-1.518953892	0.128774102	0.29613919	0.124579968
ENSMUSG00000021144	Mta1	116870	protein_coding	226.0357485	-0.124600296	0.100511754	1.239658961	0.215101563	0.420139706	0.124600296
ENSMUSG00000036815	Dpp10	269109	protein_coding	3.714102894	-0.124615777	2.175237052	0.057288366	0.954315487	1	0.124615777
ENSMUSG00000043207	Zmpste24	230709	protein_coding	588.4330586	0.124663252	0.087288949	-1.42816763	0.153243615	0.334272057	0.124663252
ENSMUSG00000021635	Rad17	19356	protein_coding	246.7175769	0.124759208	0.080274055	-1.554166009	0.120144823	0.28232699	0.124759208
ENSMUSG00000038141	Tmem181a	77106	protein_coding	397.3048453	-0.124970532	0.12888988	0.969591495	0.332250154	0.554612255	0.124970532
ENSMUSG00000073771	Btbd19	78611	protein_coding	63.80694196	-0.124986058	0.179176695	0.697557558	0.485453932	0.695380179	0.124986058
ENSMUSG00000028392	Bspry	192120	protein_coding	2842.800909	-0.125005849	0.046997821	2.659822247	0.00781819	0.034960626	0.125005849
ENSMUSG00000026667	Uhmk1	16589	protein_coding	1453.529275	-0.125022563	0.079386131	1.574866572	0.115287246	0.273824144	0.125022563
ENSMUSG00000020132	Rab21	216344	protein_coding	568.8758004	0.125052294	0.074604706	-1.676198467	0.093699324	0.235661079	0.125052294
ENSMUSG00000024811	Tnks2	74493	protein_coding	1822.920889	0.125065092	0.075022173	-1.667041722	0.095506109	0.239040298	0.125065092
ENSMUSG00000047446	Arl4a	11861	protein_coding	1355.774172	-0.125096295	0.174114905	0.718469764	0.472467685	0.684955785	0.125096295
ENSMUSG00000026821	Ralgds	19730	protein_coding	1279.053183	0.125135883	0.127525313	-0.98126309	0.326463019	0.548343875	0.125135883
ENSMUSG00000042594	Sh2b3	16923	protein_coding	165.3305508	-0.125153364	0.088684994	1.411212413	0.158181991	0.341996258	0.125153364
ENSMUSG00000055296	Tmem245	242474	protein_coding	557.524107	-0.125200643	0.116733615	1.072532904	0.283480743	0.501585257	0.125200643
ENSMUSG00000037815	Ctnna1	12385	protein_coding	6087.065458	-0.125241774	0.082341782	1.520999073	0.12826007	0.295450802	0.125241774
ENSMUSG00000015176	Nolc1	70769	protein_coding	534.0002211	0.125328134	0.128637201	-0.974275974	0.329919531	0.552117058	0.125328134
ENSMUSG00000071451	Psmg4	69666	protein_coding	64.16319132	-0.125345998	0.145320779	0.862546975	0.388386595	0.610900332	0.125345998
ENSMUSG00000066036	Ubr4	69116	protein_coding	3298.129105	0.125358345	0.137927294	-0.908872647	0.363417354	0.585795796	0.125358345
ENSMUSG00000022634	Yaf2	67057	protein_coding	105.2669957	0.125359905	0.111009171	-1.129275211	0.258781755	0.472212364	0.125359905
ENSMUSG00000026166	Ccl20	20297	protein_coding	11.93398347	-0.125360955	0.409000391	0.306505708	0.759219634	0.87897876	0.125360955
ENSMUSG00000041040	Fam117b	72750	protein_coding	735.3023302	0.125385018	0.065988713	-1.900097952	0.057420266	0.165344954	0.125385018
ENSMUSG00000040423	Rc3h1	381305	protein_coding	1081.954256	0.125420112	0.084710769	-1.480568674	0.138721548	0.311742141	0.125420112
ENSMUSG00000078597	Cyp4a12b	13118	protein_coding	1.222943214	0.125460556	2.64261903	-0.047475839	0.962133986	1	0.125460556
ENSMUSG00000024485	Slc4a9	240215	protein_coding	5.911253614	-0.125462538	0.583849004	0.214888673	0.829854116	0.914162556	0.125462538
ENSMUSG00000069631	Strada	72149	protein_coding	68.73738379	-0.125506281	0.129846642	0.966573174	0.33375745	0.556110732	0.125506281
ENSMUSG00000026849	Tor1a	30931	protein_coding	325.6520304	0.125522773	0.07190841	-1.745592373	0.080881809	0.211442486	0.125522773
ENSMUSG00000044066	Cep68	216543	protein_coding	293.3842999	0.12553171	0.103071241	-1.217912083	0.223257384	0.430343308	0.12553171
ENSMUSG00000026303	Mlph	171531	protein_coding	801.1275227	0.125531824	0.106033687	-1.183886241	0.236458097	0.446602599	0.125531824
ENSMUSG00000020150	Gamt	14431	protein_coding	17.04155812	-0.125547897	0.284642275	0.441072559	0.659160469	0.816056375	0.125547897
ENSMUSG00000005103	Wdr1	22388	protein_coding	4534.050505	-0.125565441	0.061403719	2.044915912	0.040863149	0.128048205	0.125565441
ENSMUSG00000028776	Tinagl1	94242	protein_coding	799.9261926	0.125566324	0.160489753	-0.78239465	0.433982677	0.65253482	0.125566324
ENSMUSG00000031918	Mtmr2	77116	protein_coding	645.5744003	0.125622335	0.087948977	-1.428354706	0.153189789	0.334228588	0.125622335
ENSMUSG00000026435	Slc45a3	212980	protein_coding	428.4746985	-0.125626928	0.15254239	0.823554211	0.410192902	0.630552252	0.125626928
ENSMUSG00000061979	Rcc1l	94254	protein_coding	199.4339522	-0.12568036	0.141793857	0.886359691	0.375423733	0.597623629	0.12568036
ENSMUSG00000049470	Aff4	93736	protein_coding	1724.622974	-0.125715761	0.074694443	1.683067116	0.0923621	0.233078807	0.125715761
ENSMUSG00000054452	Tle5	14797	protein_coding	5086.158442	-0.125763056	0.082165027	1.530615412	0.12586447	0.291600149	0.125763056
ENSMUSG00000029328	Hnrnpdl	50926	protein_coding	1275.635719	0.125774845	0.058881669	-2.136061147	0.03267443	0.107787762	0.125774845

ensembl_gene_id	external_gene_name	entrezgene_id	gene_biotype	baseMean	log2FoldChange	lfcSE	stat	pvalue	padj	absLog2FC
ENSMUSG00000020867	Spata20	217116	protein_coding	2.447455494	0.125827252	1.013083393	-0.124202265	0.901155129	1	0.125827252
ENSMUSG00000060601	Nr1h2	22260	protein_coding	626.3491183	-0.125849079	0.085886628	1.465293058	0.14284098	0.318637841	0.125849079
ENSMUSG00000001855	Nup214	227720	protein_coding	314.9213827	0.125870121	0.090222927	-1.395101277	0.162985301	0.348916161	0.125870121
ENSMUSG00000025437	Usp33	170822	protein_coding	919.6892473	-0.125877043	0.073400185	1.714941761	0.086355905	0.222165667	0.125877043
ENSMUSG00000039917	Rhbdd2	215160	protein_coding	144.0658447	-0.125881057	0.108322711	1.162092941	0.245197714	0.456813522	0.125881057
ENSMUSG00000029501	Ankle2	71782	protein_coding	534.2463162	0.125896564	0.076686174	-1.641711385	0.100649831	0.248933849	0.125896564
ENSMUSG00000027395	Polr1b	20017	protein_coding	301.8966296	-0.125896865	0.125922793	0.999794101	0.317410161	0.538827241	0.125896865
ENSMUSG00000042439	Zfp532	328977	protein_coding	132.7755007	-0.125897705	0.105760433	1.190404595	0.233887408	0.443446229	0.125897705
ENSMUSG00000020248	Nfyb	18045	protein_coding	305.8483908	0.12591422	0.080387145	-1.56634771	0.117267237	0.27724426	0.12591422
ENSMUSG00000034586	Hid1	217310	protein_coding	998.6323538	0.125931446	0.197306348	-0.638253395	0.523308744	0.722098569	0.125931446
ENSMUSG00000020196	Cabin1	104248	protein_coding	917.2522083	0.125938486	0.080697648	-1.560621513	0.11861308	0.279625468	0.125938486
ENSMUSG00000064262	Gimap8	243374	protein_coding	27.02826674	-0.125962717	0.218843735	0.575582923	0.564897127	0.751588072	0.125962717
ENSMUSG00000042121	Ssh1	231637	protein_coding	141.3231193	0.125996664	0.110569472	-1.139524877	0.254484298	0.467106114	0.125996664
ENSMUSG00000023025	Larp4	207214	protein_coding	1537.026427	-0.126058108	0.087203539	1.445561844	0.148300135	0.326885802	0.126058108
ENSMUSG00000018126	Baiap2l2	207495	protein_coding	638.6510066	-0.126087469	0.167546565	0.7525518	0.451719291	0.668072037	0.126087469
ENSMUSG00000101225	1700008J07Rik	629159	lncRNA	25.11920886	-0.126146865	0.253248655	0.49811465	0.618403235	0.789632028	0.126146865
ENSMUSG00000097180	2700038G22Rik	67194	lncRNA	8.414676053	-0.126147937	0.405606653	0.311010522	0.755792623	0.877286805	0.126147937
ENSMUSG00000115219	Eef1akmt4	110599566	protein_coding	51.9799717	-0.126151102	0.191208538	0.659756638	0.509410014	0.712785998	0.126151102
ENSMUSG00000000861	Bcl11a	14025	protein_coding	55.53108243	-0.126154601	0.169957868	0.742269847	0.457923857	0.67261003	0.126154601
ENSMUSG00000024077	Strn	268980	protein_coding	840.5082035	-0.126234263	0.06627215	1.904785976	0.056807898	0.164036653	0.126234263
ENSMUSG00000026615	Eprs	107508	protein_coding	2193.020555	0.126237336	0.097039224	-1.300889784	0.193296183	0.391427734	0.126237336
ENSMUSG00000003316	Glg1	20340	protein_coding	1708.542241	-0.126244425	0.080223294	1.57366295	0.115565392	0.274320379	0.126244425
ENSMUSG00000022637	Cblb	208650	protein_coding	254.7499099	-0.126296637	0.112268398	1.124952691	0.260609082	0.474080016	0.126296637
ENSMUSG00000022023	Wbp4	22380	protein_coding	326.3596072	0.126298247	0.085174169	-1.482823365	0.138121347	0.310726208	0.126298247
ENSMUSG00000060427	Zfp868	234362	protein_coding	145.4520578	-0.126304039	0.103857156	1.216132274	0.223934538	0.431058976	0.126304039
ENSMUSG00000044726	Erich5	239368	protein_coding	7.615997893	0.126308052	0.406088176	-0.311036024	0.755773236	0.877286805	0.126308052
ENSMUSG00000072066	6720489N17Rik	211378	protein_coding	15.42648362	0.126350312	0.267985923	-0.471481154	0.637297174	0.802403594	0.126350312
ENSMUSG00000032114	Slc37a4	14385	protein_coding	412.1042178	-0.126358306	0.103106744	1.225509607	0.220383273	0.426788709	0.126358306
ENSMUSG00000043991	Pura	19290	protein_coding	748.8876873	-0.12643976	0.074131893	1.705605441	0.088081566	0.225215631	0.12643976
ENSMUSG00000019977	Hbs1l	56422	protein_coding	562.9253552	0.126487096	0.067548739	-1.872530821	0.061133202	0.1731653	0.126487096
ENSMUSG00000006289	Osgep	66246	protein_coding	313.3150836	0.126523678	0.107065566	-1.181740151	0.237308822	0.44767168	0.126523678
ENSMUSG00000074867	Zfp808	630579	protein_coding	21.06093209	0.126527544	0.253512811	-0.49909724	0.617710882	0.789468115	0.126527544
ENSMUSG00000026932	Nacc2	67991	protein_coding	400.3580114	-0.126534121	0.084797246	1.492196105	0.135647743	0.306987369	0.126534121
ENSMUSG00000027540	Ptpn1	19246	protein_coding	347.8962339	0.126554552	0.085129996	-1.48660353	0.137119555	0.309427758	0.126554552
ENSMUSG00000002129	Sf3a1	67465	protein_coding	834.0778207	0.126583057	0.078295023	-1.616744625	0.105933417	0.258135524	0.126583057
ENSMUSG00000037306	Man1c1	230815	protein_coding	18.05065698	-0.126588247	0.335561086	0.377243525	0.705992638	0.845943387	0.126588247
ENSMUSG00000035109	Shc4	271849	protein_coding	3.630532206	0.12659767	0.695432155	-0.182041727	0.85554998	1	0.12659767
ENSMUSG00000075576	Gm12359	100037262	lncRNA	8.389093063	-0.126608568	0.372684989	0.339720063	0.734067351	0.864094963	0.126608568
ENSMUSG00000021468	Sptlc1	268656	protein_coding	761.1247772	0.126697493	0.082506431	-1.535607495	0.124634675	0.289400593	0.126697493

Supplementary Table 1

ensembl_gene_id	external_gene_name	entrezgene_id	gene_biotype	baseMean	log2FoldChange	lfcSE	stat	pvalue	padj	absLog2FC
ENSMUSG00000058587	Tmod3	50875	protein_coding	1297.800695	-0.126703089	0.068116122	1.86010427	0.062870775	0.17645275	0.126703089
ENSMUSG00000031822	Gse1	382034	protein_coding	584.5893379	0.126704782	0.175454209	-0.72215299	0.470200422	0.68365582	0.126704782
ENSMUSG00000073008	Gpr174	213439	protein_coding	2.573285249	0.12674758	1.072801217	-0.11814638	0.905951675	1	0.12674758
ENSMUSG00000026754	Golga1	76899	protein_coding	505.8567937	0.12675344	0.073513061	-1.724230202	0.084666294	0.219013029	0.12675344
ENSMUSG00000033460	Armcx1	78248	protein_coding	32.71425468	0.126768776	0.215754226	-0.587561033	0.556826973	0.746140914	0.126768776
ENSMUSG00000062743	Zfp677	210503	protein_coding	44.0197094	-0.126797659	0.203984196	0.621605307	0.534201431	0.730364909	0.126797659
ENSMUSG00000018541	Cwc25	67480	protein_coding	209.5030988	-0.126868689	0.090769539	1.397701155	0.162202827	0.347646657	0.126868689
ENSMUSG00000023094	Msrb2	76467	protein_coding	66.92287338	-0.126896403	0.176764034	0.717885878	0.472827656	0.685206817	0.126896403
ENSMUSG00000024037	Wdr4	57773	protein_coding	207.2091162	0.126898505	0.084040758	-1.509963832	0.131052653	0.299543739	0.126898505
ENSMUSG00000025246	Tbl1x	21372	protein_coding	1012.300908	-0.126901547	0.073178082	1.73414693	0.082892016	0.215537515	0.126901547
ENSMUSG00000045576	St7l	229681	protein_coding	311.0057431	-0.126927442	0.062430289	2.033106748	0.042041739	0.130773645	0.126927442
ENSMUSG00000022054	Nefm	18040	protein_coding	8.286032868	0.126939464	0.54959192	-0.230970397	0.8173378	0.908714429	0.126939464
ENSMUSG00000028249	Sdcbp	53378	protein_coding	2651.544183	-0.12695233	0.084732907	1.498264776	0.134064472	0.304481573	0.12695233
ENSMUSG00000034903	Cobll1	319876	protein_coding	289.511799	0.126956501	0.08017816	-1.583429968	0.113323497	0.270127107	0.126956501
ENSMUSG00000027171	Prrg4	228413	protein_coding	15.53347481	0.127003099	0.38558391	-0.329378626	0.741869521	0.869560398	0.127003099
ENSMUSG00000021033	Gstz1	14874	protein_coding	359.9660706	-0.127028155	0.078253107	1.623298555	0.104525598	0.255685933	0.127028155
ENSMUSG00000003234	Abcf3	27406	protein_coding	589.0444762	0.127036762	0.066355077	-1.914499518	0.055556361	0.161179189	0.127036762
ENSMUSG00000040483	Xaf1	327959	protein_coding	77.46434433	-0.127053613	0.283726056	0.447803824	0.654294783	0.812407823	0.127053613
ENSMUSG00000066057	Gm1976	100038746	lncRNA	28.01576986	-0.127072234	0.20830415	0.610032174	0.541840495	0.735609498	0.127072234
ENSMUSG00000049717	Lig4	319583	protein_coding	189.1916665	0.127078846	0.084131114	-1.510485718	0.130919531	0.299280888	0.127078846
ENSMUSG00000036687	Tmem184a	231832	protein_coding	430.3316956	-0.127214842	0.071310178	1.783964717	0.074429399	0.199468856	0.127214842
ENSMUSG00000064125	Prr36	73072	protein_coding	12.99490529	-0.127260315	0.624638937	0.203734201	0.838561214	0.919582966	0.127260315
ENSMUSG00000022177	Haus4	219072	protein_coding	155.4182726	0.127267392	0.142802326	-0.891213714	0.372814529	0.595133797	0.127267392
ENSMUSG00000019478	Rab4a	19341	protein_coding	386.7084748	0.127271171	0.115247109	-1.104332872	0.269448768	0.484503153	0.127271171
ENSMUSG00000030766	Arhgap17	70497	protein_coding	407.5697076	0.127332759	0.179917418	-0.707728915	0.479113617	0.690541222	0.127332759
ENSMUSG00000070705	Eid2b	434156	protein_coding	28.0679257	-0.127345766	0.210395715	0.605267871	0.545001026	0.736964526	0.127345766
ENSMUSG00000015889	Lta4h	16993	protein_coding	1209.27889	-0.127349993	0.070896803	1.796272732	0.072451152	0.195920934	0.127349993
ENSMUSG00000026510	Trp53bp2	209456	protein_coding	584.314862	-0.127366449	0.053973572	2.359792851	0.018285142	0.068347858	0.127366449
ENSMUSG00000014226	Cacybp	12301	protein_coding	454.0041175	0.127372805	0.094269292	-1.351159028	0.176644496	0.36877688	0.127372805
ENSMUSG00000016933	Plcg1	18803	protein_coding	267.7134409	-0.127388738	0.094463755	1.348546205	0.17748277	0.36983602	0.127388738
ENSMUSG00000039831	Arhgap29	214137	protein_coding	166.7729657	-0.127390372	0.117235402	1.086620341	0.277204631	0.494198131	0.127390372
ENSMUSG00000047515	BC049715	320135	protein_coding	33.06041037	0.127398317	0.228516537	-0.557501522	0.577184819	0.761298159	0.127398317
ENSMUSG00000036461	Elf1	13709	protein_coding	1315.932882	-0.127399497	0.067494666	1.887549113	0.059086507	0.168985435	0.127399497
ENSMUSG00000044320	1700001O22Rik	73598	protein_coding	29.81158916	-0.127412708	0.218221157	0.583869641	0.55930802	0.747948226	0.127412708
ENSMUSG00000059326	Csf2ra	12982	protein_coding	173.0963835	0.127493769	0.206457591	-0.617530061	0.536885159	0.732676764	0.127493769
ENSMUSG00000035064	Eef2k	13631	protein_coding	1066.491226	-0.127532578	0.088223248	1.445566564	0.148298811	0.326885802	0.127532578
ENSMUSG00000063253	Scoc	56367	protein_coding	49.11445205	0.127534489	0.207377215	-0.61498795	0.538562678	0.733377497	0.127534489
ENSMUSG00000024101	Washc1	68767	protein_coding	279.9942724	-0.127546581	0.06616407	1.927731809	0.053888493	0.157814254	0.127546581
ENSMUSG00000020831	0610010K14Rik	104457	protein_coding	5.887441764	0.127571036	0.489431162	-0.260651642	0.794361161	0.897695523	0.127571036

ensembl_gene_id	external_gene_name	entrezgene_id	gene_biotype	baseMean	log2FoldChange	lfcSE	stat	pvalue	padj	absLog2FC
ENSMUSG00000084834	4930565N06Rik	68306	lncRNA	36.35492176	-0.127643205	0.209786955	0.608442049	0.54289433	0.736235501	0.127643205
ENSMUSG00000035835	Plppr3	216152	protein_coding	50.40882313	0.127722705	0.16986438	-0.751909879	0.452105257	0.66852312	0.127722705
ENSMUSG00000057092	Fxyd3	17178	protein_coding	259.2705776	-0.127730214	0.169718823	0.75259898	0.45169093	0.668072037	0.127730214
ENSMUSG00000028672	Hmgcl	15356	protein_coding	998.1519118	-0.127759666	0.065148566	1.961051083	0.049873064	0.148611292	0.127759666
ENSMUSG00000006850	Tmco6	71983	protein_coding	179.9565955	-0.127804343	0.087717891	1.456992884	0.145118344	0.322298818	0.127804343
ENSMUSG00000061132	Blnk	17060	protein_coding	1026.459518	-0.127804627	0.08694939	1.469873769	0.141595945	0.316544664	0.127804627
ENSMUSG00000115970	8430426J06Rik	78102	lncRNA	2.242083502	-0.127825736	1.091691392	0.117089626	0.906789031	1	0.127825736
ENSMUSG00000028248	Pnisr	66625	protein_coding	710.7415833	-0.127856644	0.092619274	1.380453963	0.167446915	0.355746604	0.127856644
ENSMUSG00000024769	Cdc42bpg	240505	protein_coding	1780.072349	0.127891327	0.099739176	-1.282257699	0.199752262	0.40082018	0.127891327
ENSMUSG00000036264	Fstl4	320027	protein_coding	36.67332891	-0.127953805	0.203260952	0.629505095	0.529018434	0.726903567	0.127953805
ENSMUSG00000057469	E2f6	50496	protein_coding	154.4987255	0.127975903	0.116988045	-1.093922917	0.273988787	0.490477055	0.127975903
ENSMUSG00000049339	Retreg2	227298	protein_coding	866.7240549	-0.128003453	0.064596089	1.981597554	0.047524301	0.143502708	0.128003453
ENSMUSG00000029427	Zcchc8	70650	protein_coding	441.3032106	-0.12806405	0.05293532	2.419255252	0.015552323	0.060119629	0.12806405
ENSMUSG00000025580	Eif4a3	192170	protein_coding	957.5209637	0.128105252	0.073589523	-1.740808285	0.081717189	0.213086636	0.128105252
ENSMUSG00000022983	Scaf4	224432	protein_coding	274.1670147	0.128129495	0.133429523	-0.960278451	0.336915092	0.559233675	0.128129495
ENSMUSG00000047879	Usp14	59025	protein_coding	589.9173727	0.128185122	0.09379351	-1.366673691	0.171727608	0.36209266	0.128185122
ENSMUSG00000022119	Rbm26	74213	protein_coding	482.0974359	0.1281971	0.074596174	-1.718547935	0.085696719	0.220951697	0.1281971
ENSMUSG00000031109	Enox2	209224	protein_coding	278.5351999	-0.128205667	0.090652331	1.414256703	0.157286544	0.34055723	0.128205667
ENSMUSG00000021385	Ippk	75678	protein_coding	454.0991197	0.128256942	0.13026176	-0.984609317	0.324816012	0.546769252	0.128256942
ENSMUSG00000038014	Fam120a	218236	protein_coding	6539.024078	0.128281167	0.048334351	-2.654037231	0.007953505	0.035440956	0.128281167
ENSMUSG00000051065	Mb21d2	239796	protein_coding	164.5820633	0.128306367	0.089371121	-1.435658029	0.151099674	0.331068065	0.128306367
ENSMUSG00000108504	Gm4265	100043150	lncRNA	1.703767149	0.128326409	0.86670071	-0.148063117	0.882292957	1	0.128326409
ENSMUSG00000037211	Spry1	24063	protein_coding	136.9784133	-0.128338896	0.121300496	1.058024497	0.290044274	0.509010748	0.128338896
ENSMUSG00000087362	Gm13710	672763	lncRNA	0.873141777	0.128342114	1.455359131	-0.088185872	0.929728946	1	0.128342114
ENSMUSG00000028532	Cachd1	320508	protein_coding	872.2975185	0.128364967	0.068522509	-1.873325556	0.061023443	0.173002023	0.128364967
ENSMUSG00000090626	Tex9	21778	protein_coding	107.8371029	0.128461956	0.186490577	-0.688838859	0.490924679	0.698215786	0.128461956
ENSMUSG00000021234	Fam161b	217705	protein_coding	35.04438247	0.12848565	0.200162234	-0.641907551	0.520933211	0.720614094	0.12848565
ENSMUSG00000020329	Polrmt	216151	protein_coding	359.6032937	-0.12857194	0.068766035	1.869701243	0.061525317	0.173816344	0.12857194
ENSMUSG00000059669	Taf1b	21340	protein_coding	57.56724226	0.128609813	0.170852278	-0.752754451	0.451597483	0.668047177	0.128609813
ENSMUSG00000030814	Bcl7c	12055	protein_coding	97.05556954	-0.128611203	0.146336993	0.878870068	0.379471729	0.601768559	0.128611203
ENSMUSG00000021276	Cinp	67236	protein_coding	160.2961646	0.128636043	0.144181193	-0.892183235	0.372294726	0.594533905	0.128636043
ENSMUSG00000024142	Mlst8	56716	protein_coding	273.5018638	0.128639658	0.071253971	-1.805368263	0.071017074	0.192986007	0.128639658
ENSMUSG00000034795	Ccdc122	108811	protein_coding	98.32966307	0.128658723	0.148302015	-0.867545347	0.385643277	0.607820554	0.128658723
ENSMUSG00000005161	Prdx2	21672	protein_coding	1643.461573	-0.128664774	0.087379851	1.472476473	0.140892256	0.315313011	0.128664774
ENSMUSG00000022359	Wdyhv1	76773	protein_coding	128.1128278	-0.128677131	0.108727507	1.183482768	0.236617872	0.446793087	0.128677131
ENSMUSG00000005779	Psmb4	19172	protein_coding	1677.892811	0.128678614	0.076079511	-1.691370152	0.090766129	0.230247597	0.128678614
ENSMUSG00000034055	Phka1	18679	protein_coding	214.558948	0.128682725	0.087991065	-1.462452183	0.143617338	0.319894352	0.128682725
ENSMUSG00000073609	D2hgdh	98314	protein_coding	211.2524378	-0.128692712	0.08261827	1.55767861	0.119309454	0.280906233	0.128692712
ENSMUSG00000058833	Rex1bd	66462	protein_coding	288.4281024	0.128718247	0.111257766	-1.156937196	0.24729802	0.459379022	0.128718247

ensembl_gene_id	external_gene_name	entrezgene_id	gene_biotype	baseMean	log2FoldChange	lfcSE	stat	pvalue	padj	absLog2FC
ENSMUSG00000034574	Daam1	208846	protein_coding	900.2476254	-0.128737188	0.067198254	1.915781735	0.055392884	0.160822447	0.128737188
ENSMUSG00000068394	Cep152	99100	protein_coding	157.8494312	0.128743451	0.112610806	-1.143260188	0.252930579	0.465324602	0.128743451
ENSMUSG00000046711	Hmga1	15361	protein_coding	333.8735469	-0.128801104	0.142247731	0.905470358	0.365216257	0.587772599	0.128801104
ENSMUSG00000028749	Pla2g2f	26971	protein_coding	517.2429252	0.128842355	0.398004634	-0.323720741	0.746149467	0.872287153	0.128842355
ENSMUSG00000046082	Tmem174	68344	protein_coding	23.90126583	-0.128850439	0.528261395	0.243914168	0.807297296	0.904987729	0.128850439
ENSMUSG00000017421	Zfp207	22680	protein_coding	1412.451618	0.128864973	0.04497139	-2.865487915	0.004163672	0.020854981	0.128864973
ENSMUSG00000037946	Fgd3	30938	protein_coding	197.7264452	-0.128869497	0.121922438	1.056979331	0.290521021	0.509468423	0.128869497
ENSMUSG00000025812	Pard3	93742	protein_coding	949.5614785	-0.128903026	0.046182798	2.791148042	0.005252144	0.025397924	0.128903026
ENSMUSG00000032399	Rpl4	67891	protein_coding	12120.75198	-0.128908991	0.090222589	1.428788425	0.153065053	0.334133019	0.128908991
ENSMUSG00000023018	Smarcd1	83797	protein_coding	150.45321	-0.128933374	0.09928391	1.298633127	0.194069864	0.392753643	0.128933374
ENSMUSG00000097915	A330009N23Rik	380977	lncRNA	6.768139305	-0.129040413	0.604568086	0.213442317	0.830981977	0.914497068	0.129040413
ENSMUSG00000021910	Nisch	64652	protein_coding	1484.991774	-0.129050616	0.069201093	1.864863848	0.062200491	0.17504753	0.129050616
ENSMUSG00000028107	Tars2	71807	protein_coding	365.5726028	-0.129056064	0.120052201	1.074999569	0.282374921	0.500444242	0.129056064
ENSMUSG00000031967	Afg3l1	114896	protein_coding	435.7180204	-0.129072279	0.063116736	2.044977094	0.040857116	0.128048205	0.129072279
ENSMUSG00000036879	Phkb	102093	protein_coding	598.4234219	-0.12909908	0.104070319	1.240498555	0.214791048	0.419775423	0.12909908
ENSMUSG00000044617	Zbtb39	320080	protein_coding	224.2881725	0.129122739	0.098654695	-1.30883522	0.190590174	0.388089535	0.129122739
ENSMUSG00000024833	Pola2	18969	protein_coding	233.8658238	0.129130681	0.119157766	-1.083695049	0.278500032	0.495758276	0.129130681
ENSMUSG00000036501	Fam13b	225358	protein_coding	598.3037126	-0.129149796	0.086952123	1.485297784	0.137464959	0.309868458	0.129149796
ENSMUSG00000051844	B230319C09Rik	320775	lncRNA	28.36220451	-0.129167085	0.246877046	0.523204111	0.600832221	0.777353752	0.129167085
ENSMUSG00000038074	Fkbp14	231997	protein_coding	43.35868784	0.129199114	0.167904734	-0.769478683	0.441609195	0.659260011	0.129199114
ENSMUSG00000052253	Zfp622	52521	protein_coding	682.2767959	-0.12921977	0.075670038	1.707674175	0.087696817	0.224854857	0.12921977
ENSMUSG00000046096	Mosmo	233812	protein_coding	78.90351952	-0.129271852	0.137407497	0.940791846	0.34681154	0.568538439	0.129271852
ENSMUSG00000074829	2010315B03Rik	630836	protein_coding	385.6370174	-0.129275634	0.085312002	1.515327621	0.129689457	0.297380166	0.129275634
ENSMUSG00000035378	Shq1	72171	protein_coding	96.68676732	-0.129292577	0.12115368	1.06717829	0.285891334	0.50434884	0.129292577
ENSMUSG00000076432	Ywhaq	22630	protein_coding	1117.109388	0.129305359	0.110894454	-1.166021867	0.243605604	0.454822267	0.129305359
ENSMUSG00000026305	Lrrfip1	16978	protein_coding	1378.652832	-0.129414219	0.078003225	1.659088065	0.097098048	0.241894213	0.129414219
ENSMUSG00000030612	Mrpl46	67308	protein_coding	339.3754319	-0.129533347	0.091003179	1.423393649	0.154622073	0.336553171	0.129533347
ENSMUSG00000021188	Trip11	109181	protein_coding	1188.868932	0.129547622	0.071119054	-1.821559975	0.068521779	0.188061933	0.129547622
ENSMUSG00000003208	Yju2	72886	protein_coding	111.3243278	0.129575606	0.108072378	-1.198970623	0.230539369	0.43912271	0.129575606
ENSMUSG00000085184	4933439K11Rik	71319	lncRNA	35.42683183	0.129616667	0.213032537	-0.608436009	0.542898335	0.736235501	0.129616667
ENSMUSG00000052026	Slc6a7	240332	protein_coding	54.92117695	-0.129634926	0.276457168	0.468915047	0.63913036	0.803282676	0.129634926
ENSMUSG00000076434	Wfdc3	71856	protein_coding	4.597358712	0.129683498	0.54869269	-0.236349964	0.813161119	0.907217398	0.129683498
ENSMUSG00000057440	Mpp7	75739	protein_coding	274.0268437	-0.129696672	0.095199618	1.362365477	0.173082573	0.363688435	0.129696672
ENSMUSG00000007721	Ccdc124	234388	protein_coding	480.7330138	0.129768613	0.098399943	-1.318787484	0.18724017	0.383442977	0.129768613
ENSMUSG00000024817	Uhrf2	109113	protein_coding	1267.936366	0.129792672	0.085391836	-1.519965822	0.128519566	0.29575977	0.129792672
ENSMUSG00000042873	Lhfpl4	269788	protein_coding	4.589798181	-0.129823941	0.837972825	0.154926195	0.876879503	0.938919382	0.129823941
ENSMUSG00000040669	Phc1	13619	protein_coding	107.796515	-0.129880725	0.132279289	0.98186743	0.326165163	0.548167261	0.129880725
ENSMUSG00000042417	Ccno	218630	protein_coding	5.188191461	0.129887485	0.755703582	-0.171876234	0.863534831	0.932314788	0.129887485
ENSMUSG00000020457	Drg1	13494	protein_coding	484.9991154	0.129891196	0.094215257	-1.378664137	0.167998332	0.356505326	0.129891196

ensembl_gene_id	external_gene_name	entrezgene_id	gene_biotype	baseMean	log2FoldChange	lfcSE	stat	pvalue	padj	absLog2FC
ENSMUSG00000035673	Sbno2	216161	protein_coding	1092.612557	0.12990104	0.113376199	-1.145752293	0.25189766	0.464371283	0.12990104
ENSMUSG00000028807	Zbtb8a	73680	protein_coding	200.9695165	-0.129907262	0.111942496	1.160482094	0.24585258	0.457679163	0.129907262
ENSMUSG00000015377	Dennd6b	69440	protein_coding	93.00451493	-0.129936162	0.14280142	0.909908052	0.362871003	0.585225632	0.129936162
ENSMUSG00000035949	Fbxw2	30050	protein_coding	491.8534557	0.129984703	0.060772581	-2.138870861	0.032446129	0.107141645	0.129984703
ENSMUSG00000030611	Mrps11	67994	protein_coding	178.0638643	0.130009236	0.087318933	-1.488900881	0.136513472	0.308481591	0.130009236
ENSMUSG00000020526	Znhit3	448850	protein_coding	63.69480418	0.130014742	0.151918568	-0.855818633	0.392098107	0.614686552	0.130014742
ENSMUSG00000022414	Tab1	66513	protein_coding	358.657703	-0.130031592	0.087409481	1.487614284	0.136852644	0.309078852	0.130031592
ENSMUSG00000055839	Elob	67673	protein_coding	1378.699015	-0.130036941	0.075815386	1.715178783	0.086312454	0.222126524	0.130036941
ENSMUSG00000095845	Gng14	100503710	protein_coding	1.260157496	0.130064266	1.156542885	-0.112459527	0.910459059	1	0.130064266
ENSMUSG00000022828	Gtf2e1	74197	protein_coding	135.6752349	0.13008191	0.096684052	-1.345432956	0.178485458	0.370989158	0.13008191
ENSMUSG00000063273	Naa15	74838	protein_coding	592.7965227	0.130108747	0.10496353	-1.239561462	0.215137642	0.420139706	0.130108747
ENSMUSG00000042694	Stn1	108689	protein_coding	106.601017	0.130119279	0.128482197	-1.012741704	0.311183552	0.532085855	0.130119279
ENSMUSG00000031278	Acsl4	50790	protein_coding	207.2236614	0.130213002	0.108988225	-1.194743763	0.232187158	0.441179379	0.130213002
ENSMUSG00000006058	Snf8	27681	protein_coding	492.4095448	-0.130230157	0.065078343	2.001128962	0.045378494	0.138710092	0.130230157
ENSMUSG00000068566	Myadm	50918	protein_coding	729.5271106	0.130238842	0.063921883	-2.037468791	0.041603086	0.129751344	0.130238842
ENSMUSG00000032741	Tpcn1	252972	protein_coding	1179.441028	0.130255164	0.060456676	-2.154520777	0.031199354	0.104013109	0.130255164
ENSMUSG00000043004	Gng2	14702	protein_coding	10.29380901	-0.130264722	0.346520124	0.375922532	0.706974489	0.846503532	0.130264722
ENSMUSG00000035504	Reep6	70335	protein_coding	174.5240926	-0.130285768	0.137065761	0.950534745	0.341840608	0.563829035	0.130285768
ENSMUSG00000020740	Gga3	260302	protein_coding	168.3214584	0.130308353	0.125119331	-1.041472585	0.29765627	0.517311687	0.130308353
ENSMUSG00000022095	Fhip2b	239170	protein_coding	269.6380338	-0.130329577	0.109588035	1.189268308	0.234334102	0.443834541	0.130329577
ENSMUSG00000027185	Nat10	98956	protein_coding	310.8481827	-0.130344504	0.07205049	1.809071722	0.070439862	0.191827816	0.130344504
ENSMUSG00000042650	Alkbh5	268420	protein_coding	2030.404804	0.130392134	0.056199314	-2.320173047	0.020331518	0.074331346	0.130392134
ENSMUSG00000031166	Wdr13	73447	protein_coding	825.0941752	-0.130473986	0.098241945	1.328088377	0.184148913	0.379181439	0.130473986
ENSMUSG00000022247	Brix1	67832	protein_coding	269.7309277	0.13048472	0.107374698	-1.21522782	0.224279213	0.431320242	0.13048472
ENSMUSG00000032067	Pts	19286	protein_coding	469.0749963	0.13054717	0.087903989	-1.485110888	0.137514452	0.309937719	0.13054717
ENSMUSG00000031916	Cog8	97484	protein_coding	428.497522	-0.130552071	0.076528965	1.705917116	0.088023512	0.22515016	0.130552071
ENSMUSG00000001918	Slc1a5	20514	protein_coding	1456.892046	-0.13055567	0.086297732	1.512851682	0.130317338	0.298275885	0.13055567
ENSMUSG00000025278	Flnb	286940	protein_coding	9435.182382	-0.130571323	0.093134382	1.401966923	0.160925124	0.345760799	0.130571323
ENSMUSG00000027122	Arl14ep	212772	protein_coding	168.1962635	0.130601548	0.122358936	-1.0673642	0.285807408	0.504312604	0.130601548
ENSMUSG00000033545	Znrf1	170737	protein_coding	190.064011	0.130642706	0.086368456	-1.512620606	0.130376057	0.298368931	0.130642706
ENSMUSG00000074282	Zfp94	22756	protein_coding	26.32235995	-0.130668179	0.216809147	0.602687576	0.546716548	0.738518372	0.130668179
ENSMUSG00000037605	Adgrl3	319387	protein_coding	3.679928652	0.130685933	1.394834872	-0.093692763	0.925353219	1	0.130685933
ENSMUSG00000033751	Gadd45gip1	102060	protein_coding	209.5034419	0.130734679	0.121537145	-1.075676734	0.282071856	0.499960785	0.130734679
ENSMUSG00000080316	Spaca6	75202	protein_coding	20.37794132	-0.130750905	0.457867753	0.285564782	0.775211481	0.887843124	0.130750905
ENSMUSG00000071652	Ints5	109077	protein_coding	260.3633168	0.13078074	0.107804105	-1.213133211	0.225078894	0.432354642	0.13078074
ENSMUSG00000042404	Dennd4b	229541	protein_coding	28.94609477	0.130787054	0.279673333	-0.467642203	0.640040479	0.8036518	0.130787054
ENSMUSG00000031299	Pdha1	18597	protein_coding	3288.160209	-0.130800858	0.082203896	1.591175909	0.111569993	0.267528222	0.130800858
ENSMUSG00000032336	Nptn	20320	protein_coding	1042.911922	-0.130805097	0.04058792	3.222759295	0.001269622	0.00775103	0.130805097
ENSMUSG00000046865	Fbl	14113	protein_coding	280.533028	-0.130829399	0.131347529	0.996055279	0.319223287	0.540682396	0.130829399

ensembl_gene_id	external_gene_name	entrezgene_id	gene_biotype	baseMean	log2FoldChange	lfcSE	stat	pvalue	padj	absLog2FC
ENSMUSG00000035200	Chrnb4	108015	protein_coding	52.8690522	-0.130858009	0.185489231	0.705474964	0.480514707	0.691465456	0.130858009
ENSMUSG00000001833	Septin7	235072	protein_coding	1085.006817	0.130860135	0.080521323	-1.625161277	0.104128199	0.254940412	0.130860135
ENSMUSG00000038406	Scaf1	233208	protein_coding	826.7846388	0.130872242	0.122267727	-1.070374379	0.284450827	0.502862462	0.130872242
ENSMUSG00000027823	Gmps	229363	protein_coding	670.9014105	0.130975801	0.111159765	-1.17826626	0.238690474	0.448874722	0.130975801
ENSMUSG00000006010	Odr4	226499	protein_coding	445.6288598	0.130994098	0.111264427	-1.177322367	0.239066863	0.449192192	0.130994098
ENSMUSG00000039202	Abhd2	54608	protein_coding	6667.120591	-0.131000946	0.075010201	1.746441733	0.080734224	0.211290803	0.131000946
ENSMUSG00000022822	Abcc5	27416	protein_coding	132.6428053	-0.131023627	0.235425545	0.556539551	0.577842065	0.761753322	0.131023627
ENSMUSG00000101031	Ms4a12	381213	protein_coding	257.8282058	-0.13104117	0.180577731	0.725677354	0.468036588	0.68170367	0.13104117
ENSMUSG00000051864	Tbc1d22a	223754	protein_coding	729.0061965	-0.131111521	0.093036536	1.409247662	0.158761949	0.342763083	0.131111521
ENSMUSG00000036898	Zfp157	72154	protein_coding	119.1959828	0.131161602	0.121356188	-1.080798631	0.279786697	0.49722072	0.131161602
ENSMUSG00000072487	Mroh5	268816	protein_coding	5.75787416	0.131186957	1.808197934	-0.072551215	0.942163249	0.971883559	0.131186957
ENSMUSG00000023460	Rab12	19328	protein_coding	85.78545312	0.131192375	0.138536138	-0.946990271	0.343643736	0.56547568	0.131192375
ENSMUSG00000031546	Gins4	109145	protein_coding	195.2876209	0.131208021	0.091994952	-1.426252407	0.153795497	0.335284335	0.131208021
ENSMUSG00000013653	1810065E05Rik	69864	protein_coding	10784.0731	0.131210633	0.190295456	-0.689510069	0.490502339	0.69793377	0.131210633
ENSMUSG00000029016	Clcn6	26372	protein_coding	35.62719059	-0.131222619	0.216556895	0.605949854	0.544548053	0.736796542	0.131222619
ENSMUSG00000025962	Fastkd2	75619	protein_coding	259.4936421	0.131250477	0.135201245	-0.970778612	0.331658535	0.554128799	0.131250477
ENSMUSG00000040177	2310057M21Rik	68277	protein_coding	76.79736757	0.131277836	0.14659906	-0.895488935	0.370525778	0.593022866	0.131277836
ENSMUSG00000020091	Eif4ebp2	13688	protein_coding	1257.811302	-0.131321922	0.089177572	1.472589124	0.140861859	0.315287712	0.131321922
ENSMUSG00000062646	Ganc	76051	protein_coding	203.7773796	-0.131331587	0.091993614	1.427616351	0.153402315	0.334559671	0.131331587
ENSMUSG00000046949	Nqo2	18105	protein_coding	359.4065381	-0.131371773	0.103398718	1.270535804	0.203893838	0.406017465	0.131371773
ENSMUSG00000036499	Eea1	216238	protein_coding	1455.90765	0.131389135	0.078433269	-1.675170974	0.093900691	0.235970255	0.131389135
ENSMUSG00000068798	Rap1a	109905	protein_coding	527.3019075	-0.131421529	0.107078339	1.227340002	0.219694827	0.425947586	0.131421529
ENSMUSG00000106943	Dancr	70036	lncRNA	32.6243551	-0.131470466	0.328162342	0.400626305	0.688695276	0.83386713	0.131470466
ENSMUSG00000028617	Lrrc42	77809	protein_coding	732.9766904	-0.131517116	0.105387429	1.247939324	0.212053278	0.41642117	0.131517116
ENSMUSG00000026078	Pdcl3	68833	protein_coding	442.2466726	-0.131574421	0.077275839	1.702659233	0.088631858	0.226153663	0.131574421
ENSMUSG00000102418	Sh2d1b1	26904	protein_coding	4.355743129	0.131588434	0.938796146	-0.140167207	0.888527881	1	0.131588434
ENSMUSG00000018666	Cbx1	12412	protein_coding	610.4485483	0.131589435	0.114671624	-1.147532668	0.25116154	0.463479905	0.131589435
ENSMUSG00000035011	Zbtb7a	16969	protein_coding	1965.712822	0.131691343	0.124589812	-1.056999295	0.290511909	0.509468423	0.131691343
ENSMUSG00000038534	Osbpl7	71240	protein_coding	775.9324625	0.131732455	0.143228741	-0.919734785	0.35771137	0.580678813	0.131732455
ENSMUSG00000026977	Marchf7	57438	protein_coding	1126.926087	-0.131813583	0.067292818	1.9588061	0.050135499	0.149231729	0.131813583
ENSMUSG00000057246	BC051142	407788	protein_coding	18.01994418	0.131840314	0.337812507	-0.390276591	0.696332032	0.838747423	0.131840314
ENSMUSG00000056999	Ide	15925	protein_coding	704.2452246	0.131911824	0.100815189	-1.308451891	0.190720082	0.388133006	0.131911824
ENSMUSG00000051517	Arhgef39	230098	protein_coding	209.448188	0.131977983	0.095761101	-1.378200343	0.168141441	0.356671517	0.131977983
ENSMUSG00000033020	Polr2f	69833	protein_coding	406.0119582	-0.132002401	0.075199774	1.755356356	0.079198372	0.208527092	0.132002401
ENSMUSG00000073728	Tmem51os1	100038693	lncRNA	20.74899355	-0.132024817	0.301112918	0.438456172	0.661055634	0.816884691	0.132024817
ENSMUSG00000025558	Dock9	105445	protein_coding	535.9716094	-0.132028323	0.081954856	1.610988406	0.107182249	0.26018046	0.132028323
ENSMUSG00000020280	Pus10	74467	protein_coding	328.3127441	0.132059834	0.066307011	-1.991642103	0.04641034	0.140963833	0.132059834
ENSMUSG00000000561	Wdr77	70465	protein_coding	143.0322137	0.132107956	0.145696545	-0.906733624	0.364547678	0.587158915	0.132107956
ENSMUSG00000028082	Sh3d19	27059	protein_coding	1482.28052	-0.132115188	0.053863892	2.452759757	0.014176498	0.05583573	0.132115188

ensembl_gene_id	external_gene_name	entrezgene_id	gene_biotype	baseMean	log2FoldChange	lfcSE	stat	pvalue	padj	absLog2FC
ENSMUSG00000085944	1700003D09Rik	69338	lncRNA	24.88163404	-0.132186574	0.291434109	0.453572763	0.650136361	0.809917796	0.132186574
ENSMUSG00000019810	Fuca2	66848	protein_coding	983.1473107	-0.132201924	0.09282046	1.424275679	0.154366685	0.336218865	0.132201924
ENSMUSG00000028601	Echdc2	52430	protein_coding	149.8874021	-0.132203556	0.089790332	1.472358476	0.1409241	0.315341543	0.132203556
ENSMUSG00000020611	Gna13	14674	protein_coding	2096.710386	-0.132206396	0.059410753	2.225294081	0.026061503	0.090207431	0.132206396
ENSMUSG00000059734	Ndufs8	225887	protein_coding	1216.933323	-0.132211397	0.076464916	1.729046517	0.08380077	0.217114201	0.132211397
ENSMUSG00000035824	Tk2	57813	protein_coding	158.1685014	-0.132220228	0.087429645	1.512304294	0.130456469	0.298470232	0.132220228
ENSMUSG00000102805	Gm37240	99889	protein_coding	10.19236841	0.132223698	0.424284408	-0.311639304	0.75531466	0.877032235	0.132223698
ENSMUSG00000059883	Irak4	266632	protein_coding	513.8082731	0.132247188	0.088391748	-1.49614857	0.134614943	0.305377683	0.132247188
ENSMUSG00000036661	Dennd3	105841	protein_coding	189.292885	-0.132251671	0.093047587	1.421333703	0.15521977	0.337542702	0.132251671
ENSMUSG00000029601	Iqcd	75732	protein_coding	17.40210234	0.132299014	0.341655561	-0.387229214	0.698586525	0.840298483	0.132299014
ENSMUSG00000039953	Clstn1	65945	protein_coding	3047.543092	-0.132369401	0.057647335	2.296192891	0.021664855	0.078201839	0.132369401
ENSMUSG00000066440	Zfyve26	211978	protein_coding	333.4367724	0.132450996	0.114100482	-1.160827658	0.245711993	0.45758404	0.132450996
ENSMUSG00000041117	Ccdc8	434130	protein_coding	1.967294293	0.132473234	1.109585018	-0.119389891	0.90496647	1	0.132473234
ENSMUSG00000056260	Lrif1	321000	protein_coding	161.2748825	0.132546374	0.105447813	-1.256985524	0.208758877	0.412028696	0.132546374
ENSMUSG00000062012	Zfp13	22654	protein_coding	40.79942229	0.132588839	0.174883347	-0.758155887	0.448357666	0.665277305	0.132588839
ENSMUSG00000037202	Prf1	18646	protein_coding	1.09479915	-0.132594253	1.377040576	0.096289286	0.92329082	1	0.132594253
ENSMUSG00000018899	Irf1	16362	protein_coding	1328.362785	-0.132602695	0.115317015	1.149897048	0.250186277	0.462507128	0.132602695
ENSMUSG00000100121	1700025N23Rik	69986	lncRNA	0.983285879	-0.132605229	1.352632698	0.098034913	0.92190457	1	0.132605229
ENSMUSG00000022722	Arl6	56297	protein_coding	22.28030652	0.132667147	0.227781328	-0.582432053	0.560275696	0.748973617	0.132667147
ENSMUSG00000036873	2410004B18Rik	66421	protein_coding	165.9535922	0.132677766	0.090205077	-1.470845875	0.141332803	0.316005805	0.132677766
ENSMUSG00000044453	Ffar1	233081	protein_coding	5.774044218	-0.132709041	0.637517297	0.208165396	0.835099827	0.917066002	0.132709041
ENSMUSG00000025867	Cplx2	12890	protein_coding	56.33984595	-0.132720122	0.179711297	0.738518522	0.460199416	0.674460666	0.132720122
ENSMUSG00000032609	Klhdc8b	78267	protein_coding	36.72062243	-0.132740124	0.219481916	0.604788434	0.54531958	0.737121023	0.132740124
ENSMUSG00000002870	Mcm2	17216	protein_coding	446.4823686	0.132745919	0.126611628	-1.048449662	0.294431483	0.513653234	0.132745919
ENSMUSG00000021143	Pacs2	217893	protein_coding	525.1120661	-0.132749499	0.053254091	2.492756845	0.012675562	0.051167007	0.132749499
ENSMUSG00000002010	Idh3g	15929	protein_coding	1547.092056	-0.132808792	0.078124674	1.699959635	0.089138518	0.227130611	0.132808792
ENSMUSG00000034473	Sec22a	317717	protein_coding	332.2584714	0.132829618	0.090685106	-1.464734657	0.142993326	0.318805424	0.132829618
ENSMUSG00000001036	Epn2	13855	protein_coding	190.6138576	-0.132857634	0.106532061	1.247114086	0.212355669	0.416836029	0.132857634
ENSMUSG00000005947	Itgae	16407	protein_coding	34.96667659	-0.13286126	0.331838647	0.400379103	0.688877313	0.833904116	0.13286126
ENSMUSG00000071074	Yipf3	28064	protein_coding	505.7984822	-0.132884631	0.064827335	2.049824051	0.040381604	0.126876294	0.132884631
ENSMUSG00000028232	Tmem68	72098	protein_coding	155.1992167	0.13288994	0.125242259	-1.061063101	0.288661225	0.507554459	0.13288994
ENSMUSG00000079036	Alkbh1	211064	protein_coding	179.0594048	-0.132893461	0.117150485	1.134382509	0.256634133	0.4695907	0.132893461
ENSMUSG00000029714	Gigyf1	57330	protein_coding	230.299878	-0.132923701	0.175072396	0.759249912	0.447703074	0.664871274	0.132923701
ENSMUSG00000009566	Fpgs	14287	protein_coding	225.7609451	0.132926294	0.10204319	-1.302647381	0.192695174	0.390530809	0.132926294
ENSMUSG00000050240	Hic2	58180	protein_coding	153.3479668	-0.132929216	0.101069063	1.315231512	0.188432108	0.384976843	0.132929216
ENSMUSG00000053007	Creb5	231991	protein_coding	7.872798789	0.132947593	2.300962454	-0.057779123	0.953924568	0.977047797	0.132947593
ENSMUSG00000033099	Nol12	97961	protein_coding	160.8394	0.132968882	0.103255287	-1.287768263	0.197826643	0.398112257	0.132968882
ENSMUSG00000024366	Gfra3	14587	protein_coding	18.94717527	0.132974263	0.27526514	-0.483077017	0.629041052	0.797446101	0.132974263
ENSMUSG00000046909	Tefm	68550	protein_coding	90.02804128	-0.13297915	0.151482545	0.877851303	0.380024419	0.60223179	0.13297915

Supplementary Table 1

ensembl_gene_id	external_gene_name	entrezgene_id	gene_biotype	baseMean	log2FoldChange	lfcSE	stat	pvalue	padj	absLog2FC
ENSMUSG00000097392	Thoc2l	100042165	protein_coding	75.90756144	-0.13299648	0.151399485	0.878447371	0.379700987	0.601975182	0.13299648
ENSMUSG00000030505	Prmt3	71974	protein_coding	170.584704	-0.133001107	0.14256682	0.932903657	0.350869714	0.572963936	0.133001107
ENSMUSG00000022338	Eny2	223527	protein_coding	478.1035033	0.133002856	0.078130473	-1.702317304	0.088695903	0.226282116	0.133002856
ENSMUSG00000029686	Cul1	26965	protein_coding	1250.234759	-0.133037951	0.059534471	2.234637322	0.025441169	0.088524117	0.133037951
ENSMUSG00000078348	Sf3b5	66125	protein_coding	336.3953393	0.133054808	0.108458627	-1.226779382	0.219905522	0.426134686	0.133054808
ENSMUSG00000047789	Slc38a9	268706	protein_coding	104.9599605	-0.133058807	0.142783359	0.93189296	0.351391846	0.573407604	0.133058807
ENSMUSG00000003575	Crtc1	382056	protein_coding	131.7731293	0.133076042	0.14217968	-0.935970891	0.349288175	0.571046919	0.133076042
ENSMUSG00000050668	Gpatch11	53951	protein_coding	159.7813347	-0.133135751	0.095196362	1.398538217	0.161951505	0.347172272	0.133135751
ENSMUSG00000024851	Pitpnm1	18739	protein_coding	366.6858031	-0.13313691	0.179244857	0.74276558	0.457623618	0.672408259	0.13313691
ENSMUSG00000061808	Ttr	22139	protein_coding	152.4335955	-0.133172329	0.400872001	0.332206611	0.739733252	0.867980313	0.133172329
ENSMUSG00000044811	Cd300c2	140497	protein_coding	0.796804571	-0.133173146	1.563577982	0.085172053	0.932124609	1	0.133173146
ENSMUSG00000024029	Tff3	21786	protein_coding	5983.949861	-0.133194113	0.316954814	0.420230605	0.674316999	0.825194181	0.133194113
ENSMUSG00000026374	Tsn	22099	protein_coding	607.516533	0.133207702	0.097807041	-1.361943885	0.173215595	0.363760214	0.133207702
ENSMUSG00000024735	Prpf19	28000	protein_coding	1138.866871	0.133235357	0.10949932	-1.216768804	0.223692192	0.43083733	0.133235357
ENSMUSG00000028553	Angptl3	30924	protein_coding	7.010377194	0.133238586	0.558550751	-0.238543383	0.811459672	0.906704483	0.133238586
ENSMUSG00000015980	Lrrc27	76612	protein_coding	7.061607266	-0.133385571	0.533807308	0.24987588	0.802683336	0.902595167	0.133385571
ENSMUSG00000029780	Nt5c3	107569	protein_coding	716.8673324	-0.13339822	0.093995473	1.419198354	0.155841196	0.338448383	0.13339822
ENSMUSG00000028676	Srsf10	14105	protein_coding	756.7466317	0.133427899	0.127784306	-1.044164992	0.296409061	0.515610058	0.133427899
ENSMUSG00000006191	Cdkal1	68916	protein_coding	200.6936062	-0.133461028	0.088459073	1.508731928	0.131367303	0.300075862	0.133461028
ENSMUSG00000046668	Cxxc5	67393	protein_coding	123.2444396	0.133481403	0.156695882	-0.851850096	0.394297297	0.616875898	0.133481403
ENSMUSG00000024771	Lipk	240633	protein_coding	4.681873987	0.13349536	0.563623417	-0.236852047	0.812771572	0.907147741	0.13349536
ENSMUSG00000072762	4930522L14Rik	100041734	protein_coding	38.25845462	-0.133566613	0.213202854	0.626476665	0.531002338	0.728048657	0.133566613
ENSMUSG00000006024	Napa	108124	protein_coding	1070.605921	0.133610619	0.047190118	-2.831326233	0.004635541	0.022844324	0.133610619
ENSMUSG00000002222	Rmnd5a	68477	protein_coding	2406.283831	0.133649867	0.066412105	-2.012432326	0.044174384	0.135669662	0.133649867
ENSMUSG00000065799	Gm25517	115488411	snoRNA	2.464038408	0.13368368	0.822414892	-0.162550169	0.870872624	1	0.13368368
ENSMUSG00000020018	Snrpf	69878	protein_coding	129.7247619	0.133690852	0.130133735	-1.027334323	0.304263066	0.524504007	0.133690852
ENSMUSG00000015023	Ddx19a	13680	protein_coding	283.9410931	0.133698864	0.109686419	-1.218919032	0.222874925	0.429893421	0.133698864
ENSMUSG00000052707	Tnrc6a	233833	protein_coding	649.5973494	-0.13370557	0.10135461	1.319185881	0.187106977	0.383265272	0.13370557
ENSMUSG00000071847	Apcdd1	494504	protein_coding	3.994677581	0.133765702	0.925520496	-0.144530243	0.885081777	1	0.133765702
ENSMUSG00000092212	Slc22a13b	109280	protein_coding	4.043778091	0.13378055	0.794116862	-0.168464562	0.86621782	1	0.13378055
ENSMUSG00000020523	Fam114a2	67726	protein_coding	828.4750121	-0.133804799	0.071082049	1.882399298	0.059781821	0.170333437	0.133804799
ENSMUSG00000074918	Inafm2	100043272	protein_coding	265.6418298	-0.133805619	0.106208963	1.259833584	0.207729402	0.410708835	0.133805619
ENSMUSG00000028525	Pde4b	18578	protein_coding	28.29420545	-0.133833435	0.299674782	0.446595585	0.655167084	0.812818663	0.133833435
ENSMUSG00000035443	Thyn1	77862	protein_coding	226.9185197	0.1338362	0.11367569	-1.177351113	0.239055394	0.449192192	0.1338362
ENSMUSG00000046027	Stard5	170460	protein_coding	513.6841835	-0.133863187	0.120890695	1.107307611	0.268160955	0.48334534	0.133863187
ENSMUSG00000034032	Rpap1	68925	protein_coding	368.0669027	0.133942796	0.0915112	-1.463676534	0.14328235	0.319339495	0.133942796
ENSMUSG00000039623	Ap5z1	231855	protein_coding	215.0415307	-0.134015087	0.11106222	1.206666738	0.227560514	0.436092673	0.134015087
ENSMUSG00000055015	Gm9961	791359	lncRNA	2.457962739	-0.134069603	0.637706234	0.210237246	0.833482511	1	0.134069603
ENSMUSG00000024360	Etf1	225363	protein_coding	1648.873343	0.134092839	0.07487208	-1.790959184	0.073299845	0.197442677	0.134092839

ensembl_gene_id	external_gene_name	entrezgene_id	gene_biotype	baseMean	log2FoldChange	lfcSE	stat	pvalue	padj	absLog2FC
ENSMUSG00000022503	Nubp1	26425	protein_coding	247.5933791	0.134110327	0.104360399	-1.285069131	0.198768127	0.399325652	0.134110327
ENSMUSG00000058594	Fbh1	50755	protein_coding	1368.485815	-0.134117749	0.060734615	2.208258824	0.027226236	0.093397187	0.134117749
ENSMUSG00000022781	Pak2	224105	protein_coding	1611.781312	-0.13412644	0.068228524	1.965841153	0.049316962	0.147459771	0.13412644
ENSMUSG00000086158	Pierce2	546143	protein_coding	54.81141277	-0.134128737	0.156036588	0.85959799	0.390010684	0.612460672	0.134128737
ENSMUSG00000022617	Chkb	12651	protein_coding	102.7680814	-0.134147132	0.202274787	0.66319255	0.507207242	0.710916686	0.134147132
ENSMUSG00000026740	Dnajc1	13418	protein_coding	384.4692246	0.134198479	0.089128608	-1.505672338	0.132151308	0.3013667	0.134198479
ENSMUSG00000015879	Fam184b	58227	protein_coding	12.07817338	0.134198893	0.454204965	-0.295458886	0.767643356	0.883589766	0.134198893
ENSMUSG00000053644	Aldh7a1	110695	protein_coding	253.5989402	0.134212973	0.077875088	-1.72343912	0.084809146	0.219348196	0.134212973
ENSMUSG00000000326	Comt	12846	protein_coding	698.2828375	-0.134255242	0.061496478	2.183137088	0.029025716	0.098364596	0.134255242
ENSMUSG00000000915	Hip1r	29816	protein_coding	1364.168767	0.134256759	0.050718345	-2.64710448	0.008118427	0.036020338	0.134256759
ENSMUSG00000059791	Nrm	106582	protein_coding	76.95679048	-0.134276027	0.19802358	0.678080999	0.497720338	0.703115827	0.134276027
ENSMUSG00000066406	Akap13	75547	protein_coding	2864.148041	-0.134281163	0.137368137	0.977527727	0.328307958	0.55048001	0.134281163
ENSMUSG00000034041	Lyl1	17095	protein_coding	5.155064776	-0.134357609	0.436680981	0.307679095	0.758326529	0.878808271	0.134357609
ENSMUSG00000028195	Ccn1	16007	protein_coding	429.4117269	-0.134371613	0.287253912	0.467779922	0.63994198	0.803650332	0.134371613
ENSMUSG00000032342	Mto1	68291	protein_coding	192.7281982	0.13439791	0.09022412	-1.489600681	0.136329263	0.308134243	0.13439791
ENSMUSG00000030922	Lyrm1	73919	protein_coding	22.58693813	0.134490871	0.233856595	-0.575099759	0.565223831	0.751901591	0.134490871
ENSMUSG00000091764	Zfp964	636741	protein_coding	25.93050097	0.134527291	0.251322911	-0.535276671	0.592458556	0.772328749	0.134527291
ENSMUSG00000050730	Arhgap42	71544	protein_coding	414.6566278	0.134532476	0.084803178	-1.58640842	0.112646692	0.269245209	0.134532476
ENSMUSG00000049882	Vcpkmt	207965	protein_coding	44.90295989	-0.134539136	0.15640765	0.860182584	0.389688403	0.612245675	0.134539136
ENSMUSG00000021500	Ddx46	212880	protein_coding	845.5158993	0.134544057	0.0605358	-2.22255357	0.026245918	0.09071266	0.134544057
ENSMUSG00000024287	Thoc1	225160	protein_coding	224.9615936	0.134555609	0.094698892	-1.420878391	0.155352116	0.337697097	0.134555609
ENSMUSG00000010080	Epn3	71889	protein_coding	618.0992612	0.134587517	0.083317277	-1.615361448	0.106232444	0.258635202	0.134587517
ENSMUSG00000037935	Smarce1	57376	protein_coding	649.2246245	-0.134591281	0.072933418	1.845399328	0.064979471	0.181047913	0.134591281
ENSMUSG00000044468	Tent5c	74645	protein_coding	144.4420188	-0.134603939	0.271591821	0.495611164	0.620168775	0.791152348	0.134603939
ENSMUSG00000055991	Zkscan5	22757	protein_coding	178.1272042	-0.134609502	0.095247317	1.41326292	0.157578432	0.340965701	0.134609502
ENSMUSG00000086549	Gm13648	100415901	lncRNA	122.0450129	0.134615101	0.238686427	-0.563983059	0.572765662	0.758091673	0.134615101
ENSMUSG00000003662	Ciao1	26371	protein_coding	437.1498273	-0.134626227	0.070660182	1.905262948	0.056745899	0.163972315	0.134626227
ENSMUSG00000033540	Idua	15932	protein_coding	114.5553171	-0.134633597	0.11094973	1.213464848	0.224952146	0.432209358	0.134633597
ENSMUSG00000026643	Nmt2	18108	protein_coding	437.032003	-0.134644982	0.088005492	1.529961133	0.12602635	0.291934232	0.134644982
ENSMUSG00000019432	Ddx39b	53817	protein_coding	1665.63617	0.134662622	0.069607315	-1.934604471	0.053038851	0.155998286	0.134662622
ENSMUSG00000031134	Rbmx	19655	protein_coding	367.9470669	0.134833744	0.09648789	-1.397416228	0.162288442	0.347734431	0.134833744
ENSMUSG00000007888	Crlf1	12931	protein_coding	9.123960002	-0.134841214	0.485103451	0.277963832	0.781040123	0.890541867	0.134841214
ENSMUSG00000025085	Ablim1	226251	protein_coding	1378.235025	-0.134864807	0.122737434	1.09880745	0.271852062	0.487629303	0.134864807
ENSMUSG00000079108	Srp54c	100101806	protein_coding	82.88430199	0.134867704	0.155238562	-0.868777076	0.384969074	0.60719955	0.134867704
ENSMUSG00000028857	Tmem222	52174	protein_coding	313.7052145	0.134868298	0.091507614	-1.47384782	0.14052257	0.314808425	0.134868298
ENSMUSG00000001056	Nhp2	52530	protein_coding	302.7219409	-0.134897981	0.103569126	1.302492216	0.192748177	0.390530809	0.134897981
ENSMUSG00000007021	Syngr3	20974	protein_coding	2.728499952	-0.134914549	0.715705783	0.188505601	0.850480325	1	0.134914549
ENSMUSG00000026028	Trak2	70827	protein_coding	786.8992732	-0.134983299	0.099346933	1.358706249	0.174239691	0.365337006	0.134983299
ENSMUSG00000004018	Fancl	67030	protein_coding	93.71346347	0.134988823	0.130205782	-1.036734473	0.299859622	0.519828028	0.134988823

Supplementary Table 1

ensembl_gene_id	external_gene_name	entrezgene_id	gene_biotype	baseMean	log2FoldChange	lfcSE	stat	pvalue	padj	absLog2FC
ENSMUSG00000048521	Cxcr6	80901	protein_coding	9.174290609	-0.134994136	0.40517402	0.333175695	0.739001664	0.867615063	0.134994136
ENSMUSG00000069495	Epc2	227867	protein_coding	400.1733828	0.135020704	0.102539792	-1.316763971	0.187917753	0.384217774	0.135020704
ENSMUSG00000020776	Fbf1	217335	protein_coding	202.5922164	0.135027057	0.114558461	-1.178673805	0.23852809	0.448702897	0.135027057
ENSMUSG00000059336	Slc14a1	108052	protein_coding	1.912013781	0.135057803	1.190384361	-0.113457306	0.909668009	1	0.135057803
ENSMUSG00000037236	Matr3	17184	protein_coding	748.5534765	-0.135060695	0.112242791	1.203290602	0.228863885	0.437741278	0.135060695
ENSMUSG00000054150	Syne3	212073	protein_coding	140.1294119	-0.135067676	0.18534626	0.728731595	0.466165862	0.679754841	0.135067676
ENSMUSG00000042369	Rbm45	241490	protein_coding	424.0458552	-0.135070311	0.090932861	1.485385032	0.137441858	0.309858682	0.135070311
ENSMUSG00000047284	Neurl4	216860	protein_coding	627.4043111	-0.135330493	0.060447123	2.238824405	0.025167344	0.087793307	0.135330493
ENSMUSG00000055471	Alk	11682	protein_coding	4.145192173	0.135331144	2.26314147	-0.059797916	0.952316585	1	0.135331144
ENSMUSG00000075420	Smim6	68528	protein_coding	897.3642135	-0.13539381	0.176838885	0.765633704	0.443894284	0.661236714	0.13539381
ENSMUSG00000053477	Tcf4	21413	protein_coding	346.35157	0.135397243	0.094424301	-1.433923702	0.151594037	0.331843045	0.135397243
ENSMUSG00000026354	Lct	226413	protein_coding	30.95146894	0.135429858	0.874644071	-0.154839966	0.876947483	0.938919382	0.135429858
ENSMUSG00000033319	Fem1c	240263	protein_coding	459.4611467	-0.135564924	0.077638362	1.746107468	0.080792279	0.21130879	0.135564924
ENSMUSG00000038217	Tlcd2	380712	protein_coding	58.41941336	-0.135611992	0.173214885	0.782911887	0.433678857	0.652196761	0.135611992
ENSMUSG00000101348	Gm7135	634346	transcribed_unprocessed_pseudogene	0.681628359	-0.135661762	1.584297475	0.085628971	0.931761368	1	0.135661762
ENSMUSG00000027840	Wnt2b	22414	protein_coding	9.642866508	-0.135675439	0.4719957	0.287450583	0.773767335	0.88672609	0.135675439
ENSMUSG00000055723	Rras2	66922	protein_coding	626.0289645	-0.135691158	0.070235914	1.931934123	0.053367637	0.156761502	0.135691158
ENSMUSG00000040723	Rcsd1	226594	protein_coding	12.88445651	-0.135699935	0.63462454	0.213827115	0.830681879	0.91440345	0.135699935
ENSMUSG00000040187	Arntl2	272322	protein_coding	14.58403305	-0.135761938	0.301432612	0.450389016	0.652429963	0.8114957	0.135761938
ENSMUSG00000004612	Nkg7	72310	protein_coding	12.57757806	0.135763359	0.466840979	-0.290812859	0.77119445	0.885199039	0.135763359
ENSMUSG00000027329	Spef1	70997	protein_coding	105.0135703	-0.135802938	0.166209923	0.817056741	0.413896003	0.633934205	0.135802938
ENSMUSG00000032239	Rp9	55934	protein_coding	407.267924	-0.135811735	0.078197322	1.736782445	0.082425579	0.214493541	0.135811735
ENSMUSG00000068267	Cenpb	12616	protein_coding	2721.108475	-0.135814784	0.049445492	2.746757661	0.006018759	0.02823687	0.135814784
ENSMUSG00000070738	Dgkd	227333	protein_coding	1608.338521	-0.135831722	0.060393033	2.249128989	0.024504291	0.08602548	0.135831722
ENSMUSG00000026384	Ptpn4	19258	protein_coding	300.3082407	-0.135850484	0.104795908	1.296333863	0.194860487	0.393919217	0.135850484
ENSMUSG00000008575	Nfib	18028	protein_coding	674.7439564	0.135911586	0.120840526	-1.124718588	0.260708303	0.474208273	0.135911586
ENSMUSG00000014859	E2f4	104394	protein_coding	451.2474567	0.135946588	0.062365487	-2.179836862	0.029269556	0.09897928	0.135946588
ENSMUSG00000033964	Zbtb41	226470	protein_coding	440.1438559	0.135949316	0.102652757	-1.324361078	0.185383155	0.380725956	0.135949316
ENSMUSG00000027363	Usp8	84092	protein_coding	769.9885913	0.136204868	0.0468612	-2.906559578	0.003654273	0.018770347	0.136204868
ENSMUSG00000025630	Hprt	15452	protein_coding	740.159001	0.136244781	0.104285905	-1.306454415	0.191398071	0.389062992	0.136244781
ENSMUSG00000033066	Gas7	14457	protein_coding	109.6808095	-0.136260659	0.127453218	1.069103329	0.285023112	0.503462581	0.136260659
ENSMUSG00000060206	Zfp462	242466	protein_coding	253.2242154	-0.136429204	0.113879927	1.198009239	0.23091342	0.439526427	0.136429204
ENSMUSG00000001366	Fbxo9	71538	protein_coding	540.0865011	0.136448193	0.07205285	-1.893723748	0.058261687	0.16718219	0.136448193
ENSMUSG00000056665	Them6	223626	protein_coding	18.83623671	0.136504276	0.270562512	-0.504520285	0.613895811	0.78655252	0.136504276
ENSMUSG00000050373	Snx21	101113	protein_coding	190.1166748	0.136517893	0.099904619	-1.366482295	0.171787634	0.362126754	0.136517893
ENSMUSG00000042766	Trim46	360213	protein_coding	157.5687987	-0.136527749	0.188764481	0.723270333	0.469513815	0.683156904	0.136527749
ENSMUSG00000071054	Safb	224903	protein_coding	802.6656485	0.136591378	0.112265645	-1.21668012	0.223725946	0.43083733	0.136591378
ENSMUSG00000086796	4932702P03Rik	73326	lncRNA	4.432403258	0.13660625	0.854379666	-0.159889397	0.872968201	0.937394946	0.13660625
ENSMUSG00000064390	Snord73b	19871	snoRNA	6.303939264	0.136606542	0.445992448	-0.306297881	0.759377852	0.879038683	0.136606542

Supplementary Table 1

ensembl_gene_id	external_gene_name	entrezgene_id	gene_biotype	baseMean	log2FoldChange	lfcSE	stat	pvalue	padj	absLog2FC
ENSMUSG00000024048	Myl12a	67268	protein_coding	565.4570145	-0.13660876	0.083467831	1.636663606	0.101700777	0.250669647	0.13660876
ENSMUSG00000037316	Bag4	67384	protein_coding	275.353017	-0.136661497	0.094750751	1.442326267	0.149210353	0.32840994	0.136661497
ENSMUSG00000031848	Lsm4	50783	protein_coding	554.9203043	0.136666636	0.094798094	-1.441660164	0.149398267	0.328779714	0.136666636
ENSMUSG00000019775	Rgs17	56533	protein_coding	167.8923628	-0.136684318	0.207820515	0.657703684	0.510728554	0.71379243	0.136684318
ENSMUSG00000024845	Tmem134	66990	protein_coding	708.0906157	-0.136696277	0.064981544	2.103616927	0.035411876	0.114552737	0.136696277
ENSMUSG00000030770	Parva	57342	protein_coding	1049.893741	-0.136703295	0.114415754	1.194794336	0.232167394	0.441179379	0.136703295
ENSMUSG00000028331	Trmo	74753	protein_coding	61.27883652	-0.136730783	0.169846654	0.805024884	0.42080532	0.640714813	0.136730783
ENSMUSG00000022248	Rad1	19355	protein_coding	76.49876496	0.136753108	0.128520629	-1.0640557	0.287303468	0.506201691	0.136753108
ENSMUSG00000029213	Commd8	27784	protein_coding	305.8896121	0.136764172	0.084867669	-1.611499095	0.107070984	0.260075004	0.136764172
ENSMUSG00000047824	Pygo2	68911	protein_coding	554.6380191	0.136764349	0.055759672	-2.452746664	0.014177014	0.05583573	0.136764349
ENSMUSG00000062929	Cfl2	12632	protein_coding	262.5608052	-0.136767271	0.199130008	0.68682401	0.492193638	0.698889573	0.136767271
ENSMUSG00000001150	Mcm3ap	54387	protein_coding	570.4286609	0.136812948	0.072208648	-1.89468924	0.058133584	0.166901601	0.136812948
ENSMUSG00000087305	A430035B10Rik	320312	lncRNA	22.21264256	-0.136828443	0.24721133	0.55348775	0.579929466	0.76328646	0.136828443
ENSMUSG00000020785	Camkk1	55984	protein_coding	34.18290483	0.136838146	0.185326576	-0.73836224	0.460294354	0.674531987	0.136838146
ENSMUSG00000087598	Zfp111	56707	protein_coding	138.9887755	-0.136846968	0.12011895	1.13926211	0.254593847	0.467151421	0.136846968
ENSMUSG00000026889	Rbm18	67889	protein_coding	227.3988285	0.136850286	0.090681378	-1.509133285	0.131264725	0.299903938	0.136850286
ENSMUSG00000023043	Krt18	16668	protein_coding	6615.610191	-0.136909412	0.083078967	1.647943125	0.099364343	0.246352764	0.136909412
ENSMUSG00000008393	Carhsp1	52502	protein_coding	1302.71217	-0.136929174	0.092884934	1.474180667	0.140432954	0.314711603	0.136929174
ENSMUSG00000027534	Snx16	74718	protein_coding	88.10294702	-0.136944335	0.104490705	1.310588679	0.189996766	0.387311023	0.136944335
ENSMUSG00000015932	Dstn	56431	protein_coding	12626.54385	0.136957725	0.099673521	-1.37406327	0.169422041	0.358767397	0.136957725
ENSMUSG00000058135	Gstm1	14862	protein_coding	2427.491274	0.136958051	0.142527938	-0.96092074	0.336592022	0.559203284	0.136958051
ENSMUSG00000036078	Sigmar1	18391	protein_coding	486.1848236	0.136969181	0.104507177	-1.310619846	0.189986231	0.387311023	0.136969181
ENSMUSG00000032094	Cd3d	12500	protein_coding	9.210008843	-0.136987282	0.494940188	0.276775427	0.781952551	0.890747356	0.136987282
ENSMUSG00000063450	Syne2	319565	protein_coding	3017.1959	-0.137023255	0.113067252	1.211873935	0.225560641	0.433049501	0.137023255
ENSMUSG00000010453	Kansl3	226976	protein_coding	818.009483	-0.13706659	0.079444673	1.725308756	0.084471845	0.218595521	0.13706659
ENSMUSG00000034930	Rtkn	20166	protein_coding	167.1817196	-0.137103837	0.114971116	1.192506799	0.233062586	0.442542894	0.137103837
ENSMUSG00000046982	Tshz1	110796	protein_coding	865.1781386	-0.13714312	0.064462708	2.127479948	0.033380226	0.109497021	0.13714312
ENSMUSG00000036513	Commd2	52245	protein_coding	165.1172451	0.137153678	0.104611961	-1.311070717	0.189833873	0.387122324	0.137153678
ENSMUSG00000028126	Pip5k1a	18720	protein_coding	233.2506158	-0.137156237	0.080645566	1.700728799	0.088993925	0.226867192	0.137156237
ENSMUSG00000052056	Zfp217	228913	protein_coding	814.976122	-0.137178636	0.067932743	2.019330145	0.043452917	0.133877139	0.137178636
ENSMUSG00000058392	Rrp1b	72462	protein_coding	135.8044974	0.137192223	0.1619028	-0.847373999	0.396786693	0.618855346	0.137192223
ENSMUSG00000049411	Tmem241	338363	protein_coding	115.713243	0.137204007	0.111164394	-1.234244182	0.217111929	0.422731291	0.137204007
ENSMUSG00000037686	Aspg	104816	protein_coding	670.927295	0.137206121	0.151034644	-0.908441382	0.36364507	0.586048082	0.137206121
ENSMUSG00000014850	Msh3	17686	protein_coding	292.8901036	-0.137212072	0.085726878	1.600572369	0.109471666	0.264184582	0.137212072
ENSMUSG00000044814	Or55b3	257947	protein_coding	1.131997778	0.137231877	0.967554186	-0.141833789	0.887211294	1	0.137231877
ENSMUSG00000034610	Tut4	230594	protein_coding	239.6838012	-0.137246971	0.09762278	1.405890824	0.159756547	0.343942789	0.137246971
ENSMUSG00000055833	1700034H15Rik	98736	lncRNA	6.846739016	-0.137255876	0.496440319	0.276480114	0.782179331	0.890759912	0.137255876
ENSMUSG00000017978	Cadps2	320405	protein_coding	905.3223004	-0.137264916	0.078123461	1.757025529	0.078913458	0.208009073	0.137264916
ENSMUSG00000025158	Rfng	19719	protein_coding	184.0891524	0.137269106	0.107913819	-1.272025287	0.203364135	0.405696463	0.137269106

Supplementary Table 1

ensembl_gene_id	external_gene_name	entrezgene_id	gene_biotype	baseMean	log2FoldChange	lfcSE	stat	pvalue	padj	absLog2FC
ENSMUSG00000026565	Pou2f1	18986	protein_coding	234.2035066	0.137285784	0.121156756	-1.13312529	0.257161642	0.470243313	0.137285784
ENSMUSG00000036864	Proser3	333193	protein_coding	38.53986395	0.137302194	0.213574329	-0.642877798	0.520303397	0.720291626	0.137302194
ENSMUSG00000029015	Slc26a5	80979	protein_coding	1.510887764	0.137307388	2.671289263	-0.051401168	0.959005854	1	0.137307388
ENSMUSG00000025147	Mob2	101513	protein_coding	654.1347338	0.137319925	0.051385521	-2.672346659	0.007532279	0.033883971	0.137319925
ENSMUSG00000002778	Kdelr1	68137	protein_coding	1267.817866	0.137326653	0.086747958	-1.583053447	0.113409283	0.270251212	0.137326653
ENSMUSG00000021932	Rnaseh2b	67153	protein_coding	148.5686196	0.13734676	0.120613375	-1.138735733	0.254813394	0.467346556	0.13734676
ENSMUSG00000024064	Galnt14	71685	protein_coding	3.496291461	-0.137396105	1.99844696	0.06875144	0.945187472	1	0.137396105
ENSMUSG00000072494	Ppp1r3e	105651	protein_coding	48.43480083	-0.137399029	0.252154015	0.544901211	0.585821463	0.767554399	0.137399029
ENSMUSG00000066043	Phactr4	100169	protein_coding	422.7290478	-0.137401817	0.12753324	1.077380431	0.281310343	0.499023496	0.137401817
ENSMUSG00000054792	Klhl18	270201	protein_coding	249.6503138	-0.137468516	0.076547095	1.795868483	0.072515436	0.196025521	0.137468516
ENSMUSG00000028430	Nol6	230082	protein_coding	364.8617309	0.137489578	0.106860645	-1.286625	0.198225026	0.398476941	0.137489578
ENSMUSG00000028657	Ppt1	19063	protein_coding	383.6484916	-0.137584844	0.083626782	1.645224653	0.099923491	0.247508105	0.137584844
ENSMUSG00000022560	Slc52a2	52710	protein_coding	239.673431	-0.13760615	0.141389217	0.973243595	0.330432249	0.552639068	0.13760615
ENSMUSG00000034109	Golim4	73124	protein_coding	1055.517088	0.137688874	0.060355836	-2.28128517	0.022531578	0.080642929	0.137688874
ENSMUSG00000030722	Nfatc2ip	18020	protein_coding	70.32055125	0.137715295	0.152725192	-0.901719573	0.367205853	0.589852079	0.137715295
ENSMUSG00000028007	Snx7	76561	protein_coding	694.0637143	-0.13772874	0.081441881	1.691129165	0.090812136	0.230328923	0.13772874
ENSMUSG00000021270	Hsp90aa1	15519	protein_coding	3121.300132	-0.137835439	0.159862453	0.86221271	0.388570477	0.611070646	0.137835439
ENSMUSG00000021022	Ppp2r3c	59032	protein_coding	119.8297512	-0.137842148	0.11689681	1.179178012	0.2383273	0.448589299	0.137842148
ENSMUSG00000028639	Ybx1	22608	protein_coding	5081.886756	-0.137842537	0.076303266	1.806509014	0.070838867	0.192691988	0.137842537
ENSMUSG00000032407	U2surp	67958	protein_coding	1078.919948	-0.137873643	0.067478801	2.043214164	0.041031242	0.128477424	0.137873643
ENSMUSG00000071064	Zfp827	622675	protein_coding	316.157415	-0.137894724	0.101371387	1.360292369	0.173737422	0.364468978	0.137894724
ENSMUSG00000038418	Egr1	13653	protein_coding	3255.422974	-0.137911946	0.198397089	0.695130896	0.486973274	0.695624496	0.137911946
ENSMUSG00000089695	Gm15946	108168333	processed_pseudogene	0.614287737	-0.137912922	1.441593439	0.095667002	0.92378505	1	0.137912922
ENSMUSG00000024847	Aip	11632	protein_coding	276.093547	-0.137932931	0.073161101	1.885331538	0.059385091	0.169583213	0.137932931
ENSMUSG00000031596	Slc7a2	11988	protein_coding	13.13805403	0.137970071	0.343909075	-0.401181826	0.68828626	0.833786242	0.137970071
ENSMUSG00000041245	Wnk3	279561	protein_coding	4.449226845	-0.138005888	0.94390916	0.146206747	0.883758179	0.942424291	0.138005888
ENSMUSG00000020775	Mrpl38	60441	protein_coding	432.3968237	-0.13806635	0.114333395	1.20757676	0.227210103	0.435789505	0.13806635
ENSMUSG00000033417	Cacul1	78832	protein_coding	691.8688026	0.138071963	0.052370547	-2.636443043	0.008378026	0.036904435	0.138071963
ENSMUSG00000028245	Nsmaf	18201	protein_coding	194.2933861	-0.138076355	0.083106001	1.661448669	0.09662337	0.240996576	0.138076355
ENSMUSG00000038290	Smg6	103677	protein_coding	459.5467225	-0.13808157	0.08781662	1.572385388	0.115861201	0.274864513	0.13808157
ENSMUSG00000103168	Gm30948	102633020	lncRNA	0.952893882	0.138082061	3.078699063	-0.044850782	0.964226248	1	0.138082061
ENSMUSG00000021666	Gfm2	320806	protein_coding	661.1070842	-0.138100581	0.07007648	1.970712306	0.048756792	0.14626152	0.138100581
ENSMUSG00000020630	Rnaseh1	19819	protein_coding	127.2281801	-0.138105601	0.107803143	1.281090672	0.20016182	0.40124694	0.138105601
ENSMUSG00000076431	Sox4	20677	protein_coding	582.0213123	0.138116618	0.084201914	-1.640302607	0.100942264	0.2494703	0.138116618
ENSMUSG00000004642	Slbp	20492	protein_coding	448.0132009	0.13812042	0.076667157	-1.801559182	0.071614788	0.194226738	0.13812042
ENSMUSG00000023960	Enpp5	83965	protein_coding	549.7231653	0.138218184	0.149332194	-0.925575261	0.354666755	0.577325459	0.138218184
ENSMUSG00000084799	Ino80dos	100380944	lncRNA	20.37349578	0.138259297	0.294963496	-0.468733584	0.639260078	0.803282676	0.138259297
ENSMUSG00000031527	Eri1	67276	protein_coding	377.5312863	0.138298828	0.106211361	-1.302109555	0.192878937	0.390726542	0.138298828
ENSMUSG00000040170	Fmo2	55990	protein_coding	6.682885309	-0.138316445	0.462045561	0.299356724	0.764667878	0.882132403	0.138316445

Supplementary Table 1

ensembl_gene_id	external_gene_name	entrezgene_id	gene_biotype	baseMean	log2FoldChange	lfcSE	stat	pvalue	padj	absLog2FC
ENSMUSG00000052738	Suclg1	56451	protein_coding	2791.888596	-0.138325348	0.087823903	1.575030757	0.115249345	0.273806314	0.138325348
ENSMUSG00000038954	Supt3	109115	protein_coding	35.09588863	0.138392882	0.216440197	-0.639404715	0.522559682	0.721840784	0.138392882
ENSMUSG00000062981	Mrpl42	67270	protein_coding	657.2712602	-0.138406779	0.077016354	1.797108942	0.072318326	0.195652825	0.138406779
ENSMUSG00000097099	Gm9917	100038755	lncRNA	75.87248311	-0.138419664	0.165329696	0.83723413	0.402460977	0.624001556	0.138419664
ENSMUSG00000019791	Hint3	66847	protein_coding	291.8754322	-0.138459229	0.071525465	1.935803252	0.052891803	0.155751645	0.138459229
ENSMUSG00000055320	Tead1	21676	protein_coding	200.8670039	-0.138488099	0.17432305	0.794433664	0.426943013	0.646395058	0.138488099
ENSMUSG00000085772	D630024D03Rik	414116	lncRNA	9.88362935	-0.138505122	0.395318865	0.350363048	0.726066254	0.858172551	0.138505122
ENSMUSG00000034487	Poglut3	68304	protein_coding	180.6178559	0.1385304	0.127307587	-1.0881551	0.276526642	0.493575548	0.1385304
ENSMUSG00000038267	Slc22a23	73102	protein_coding	1624.623001	-0.138575492	0.098996975	1.399795217	0.161574651	0.346538122	0.138575492
ENSMUSG00000031311	Nono	53610	protein_coding	1686.546673	-0.138575724	0.100694693	1.376196889	0.168760685	0.357709402	0.138575724
ENSMUSG00000024580	Grpel2	17714	protein_coding	699.9215511	-0.138581711	0.079937925	1.733616568	0.082986139	0.215748283	0.138581711
ENSMUSG00000118423	Lrrc70	113523645	transcribed_unitary_pseudogene	4.531903047	-0.138591338	0.544336168	0.254606153	0.799027306	0.900323208	0.138591338
ENSMUSG00000040712	Camta2	216874	protein_coding	665.2457163	0.138613189	0.158332295	-0.875457459	0.381325047	0.603700684	0.138613189
ENSMUSG00000030045	Mrpl19	56284	protein_coding	293.3041964	-0.138653223	0.08908313	1.55644759	0.119601696	0.281393704	0.138653223
ENSMUSG00000023169	Slc38a1	105727	protein_coding	673.7516263	-0.13872558	0.130561157	1.062533324	0.287993638	0.506866348	0.13872558
ENSMUSG00000026032	Ndufb3	66495	protein_coding	501.4098443	-0.138749685	0.094441535	1.469159574	0.141789512	0.316830918	0.138749685
ENSMUSG00000098789	Jmjd7	433466	protein_coding	16.17866178	-0.138777224	0.258709554	0.536420947	0.591667658	0.771785126	0.138777224
ENSMUSG00000057409	Zfp53	24132	protein_coding	189.6915033	0.138877252	0.103439057	-1.342599755	0.17940161	0.372658891	0.138877252
ENSMUSG00000028879	Stx12	100226	protein_coding	834.8270405	-0.138881809	0.073389597	1.892390937	0.058438913	0.167508599	0.138881809
ENSMUSG00000030802	Bckdk	12041	protein_coding	617.3560972	0.138898965	0.087700884	-1.583780668	0.11324364	0.270127107	0.138898965
ENSMUSG00000028053	Ash1l	192195	protein_coding	1157.755275	-0.138904572	0.080672662	1.721829544	0.085100401	0.219864981	0.138904572
ENSMUSG00000030386	Zfp606	67370	protein_coding	140.434808	-0.139003951	0.10858894	1.280093077	0.200512403	0.401803401	0.139003951
ENSMUSG00000032802	Srxn1	76650	protein_coding	1647.499408	-0.139009811	0.123827387	1.122609585	0.261603343	0.475244062	0.139009811
ENSMUSG00000017210	Med24	23989	protein_coding	483.6462172	0.139040917	0.069383904	-2.003936203	0.045076895	0.137877355	0.139040917
ENSMUSG00000029188	Slc34a2	20531	protein_coding	615.1368902	0.139080201	0.315579328	-0.440713915	0.659420121	0.816133095	0.139080201
ENSMUSG00000063239	Grm4	268934	protein_coding	7.165093042	-0.139110934	0.571926703	0.243232102	0.8078256	0.905233898	0.139110934
ENSMUSG00000030733	Sh2b1	20399	protein_coding	472.4766372	0.139134272	0.108870207	-1.277982981	0.201255427	0.402657151	0.139134272
ENSMUSG00000030536	Iqgap1	29875	protein_coding	5398.671723	-0.139148227	0.096991431	1.434644538	0.151388416	0.331436869	0.139148227
ENSMUSG00000032220	Myo1e	71602	protein_coding	1337.304645	-0.139149131	0.06905746	2.014976102	0.043907153	0.135006119	0.139149131
ENSMUSG00000026365	Cfh	12628	protein_coding	6.117805254	-0.139161465	0.509732365	0.273008886	0.784846385	0.892504697	0.139161465
ENSMUSG00000113836	Gm3325	100041420	transcribed_processed_pseudogene	36.59338682	-0.139190354	0.200189402	0.695293321	0.486871499	0.695624496	0.139190354
ENSMUSG00000086529	Acss2os	74945	lncRNA	2.143250408	-0.139267811	0.680522574	0.204648334	0.837846889	1	0.139267811
ENSMUSG00000052539	Magi3	99470	protein_coding	1464.067504	0.139281	0.046367349	-3.003859497	0.002665784	0.01440064	0.139281
ENSMUSG00000026313	Hdac4	208727	protein_coding	684.8505781	-0.139325935	0.119835654	1.162641755	0.244974882	0.456655966	0.139325935
ENSMUSG00000027067	Ssrp1	20833	protein_coding	1194.231544	0.13935266	0.101537873	-1.372420513	0.169932568	0.359501207	0.13935266
ENSMUSG00000026390	Marco	17167	protein_coding	1.485611066	-0.139363042	1.771317194	0.078677632	0.937289038	1	0.139363042
ENSMUSG00000028322	Exosc3	66362	protein_coding	232.892623	0.139393426	0.077992904	-1.78725781	0.07389583	0.198651429	0.139393426
ENSMUSG00000021715	Cwc27	67285	protein_coding	133.2553665	0.139412604	0.104343956	-1.336087005	0.181520837	0.375643497	0.139412604
ENSMUSG00000027582	Zgpat	229007	protein_coding	388.260569	-0.139426343	0.068280005	2.041979118	0.041153603	0.128762902	0.139426343

ensembl_gene_id	external_gene_name	entrezgene_id	gene_biotype	baseMean	log2FoldChange	lfcSE	stat	pvalue	padj	absLog2FC
ENSMUSG00000032042	Srpr	67398	protein_coding	1393.289148	0.139427072	0.091726791	-1.520025609	0.128504539	0.29575977	0.139427072
ENSMUSG00000043987	Cep164	214552	protein_coding	476.364828	-0.139454471	0.079937016	1.74455437	0.08106247	0.211747172	0.139454471
ENSMUSG00000056602	Fry	320365	protein_coding	229.3975717	-0.139492179	0.115911016	1.203441949	0.228805343	0.437741278	0.139492179
ENSMUSG00000022555	Dgat1	13350	protein_coding	415.4187623	-0.139520578	0.110631602	1.261127701	0.207262842	0.410105269	0.139520578
ENSMUSG00000024792	Zfpl1	81909	protein_coding	262.3323067	0.139533741	0.081925369	-1.703181112	0.08853418	0.225974259	0.139533741
ENSMUSG00000022056	Adam7	11500	protein_coding	2.606074123	0.139548937	0.787496613	-0.177205762	0.85934677	1	0.139548937
ENSMUSG00000024993	Dennd10	67894	protein_coding	269.248535	0.139564349	0.070992365	-1.965906446	0.049309418	0.147459771	0.139564349
ENSMUSG00000043036	Ccdc63	330188	protein_coding	3.495792971	-0.139580788	1.265736687	0.110276323	0.912190234	1	0.139580788
ENSMUSG00000025132	Arhgdia	192662	protein_coding	2931.661644	0.139584217	0.070137561	-1.99014929	0.046574492	0.141280523	0.139584217
ENSMUSG00000088183	Gm22215	115488890	snRNA	0.698573286	-0.139586877	1.393595094	0.100163152	0.9202148	1	0.139586877
ENSMUSG00000002395	Use1	67023	protein_coding	815.0658561	-0.139595577	0.065938042	2.117071922	0.034253744	0.111571269	0.139595577
ENSMUSG00000033732	Sf3b3	101943	protein_coding	1324.648794	0.139614548	0.092188182	-1.514451697	0.129911316	0.29771794	0.139614548
ENSMUSG00000022856	Tmem41a	66664	protein_coding	186.1213655	-0.139657132	0.095784601	1.458033244	0.14483138	0.321916639	0.139657132
ENSMUSG00000066148	Prpf4	70052	protein_coding	280.9187884	0.139659853	0.108118498	-1.291729502	0.196450837	0.396067528	0.139659853
ENSMUSG00000041037	Irgq	210146	protein_coding	457.8695003	0.139671534	0.085198763	-1.639361068	0.101138085	0.24962192	0.139671534
ENSMUSG00000017760	Ctsa	19025	protein_coding	1597.910186	-0.139713124	0.05096273	2.741476458	0.006116374	0.028605439	0.139713124
ENSMUSG00000040018	Cox15	226139	protein_coding	666.4446293	-0.139739064	0.094966871	1.471450658	0.141169282	0.315761821	0.139739064
ENSMUSG00000015755	Tab2	68652	protein_coding	961.9808755	0.139853493	0.059489126	-2.350908527	0.018727637	0.069749255	0.139853493
ENSMUSG00000022142	Nup155	170762	protein_coding	270.1099852	0.139879991	0.247121018	-0.566038423	0.571367661	0.756904024	0.139879991
ENSMUSG00000029649	Pomp	66537	protein_coding	713.8378287	0.139885155	0.073819138	-1.89497139	0.058096192	0.166823251	0.139885155
ENSMUSG00000078652	Psme3	19192	protein_coding	751.575389	0.139915814	0.070000509	-1.998782797	0.04563186	0.13918813	0.139915814
ENSMUSG00000028788	Ptp4a2	19244	protein_coding	5043.244296	0.139943567	0.088841205	-1.575210141	0.115207947	0.273747344	0.139943567
ENSMUSG00000031659	Adcy7	11513	protein_coding	25.02913141	0.139949517	0.308342939	-0.453876187	0.649917944	0.809917796	0.139949517
ENSMUSG00000030921	Trim30a	20128	protein_coding	80.66718771	-0.139957002	0.394690906	0.354599005	0.722890022	0.856549354	0.139957002
ENSMUSG00000058656	Samd12	320679	protein_coding	275.3088588	0.139988387	0.136367646	-1.026551321	0.304631786	0.524795464	0.139988387
ENSMUSG00000030685	Kctd13	233877	protein_coding	56.45497762	0.140077824	0.162001706	-0.864668825	0.387220585	0.60964446	0.140077824
ENSMUSG00000031445	Proz	66901	protein_coding	20.70397448	0.140105451	0.266263353	-0.526191268	0.598755321	0.776186935	0.140105451
ENSMUSG00000024182	Axin1	12005	protein_coding	710.4561102	0.140119047	0.064693516	-2.165890129	0.030319584	0.101747126	0.140119047
ENSMUSG00000039878	Slc39a5	72002	protein_coding	1179.049675	-0.140179494	0.080578096	1.739672452	0.081916549	0.213539052	0.140179494
ENSMUSG00000030509	Asb7	117589	protein_coding	390.0790262	0.140209834	0.087472401	-1.602903676	0.108955922	0.26324771	0.140209834
ENSMUSG00000038525	Armc10	67211	protein_coding	375.7459975	-0.140214552	0.117614965	1.19214891	0.233202862	0.442685693	0.140214552
ENSMUSG00000022160	Mettl3	56335	protein_coding	207.5742565	-0.14022034	0.125386668	1.118303425	0.263437425	0.477601877	0.14022034
ENSMUSG00000000753	Serpinf1	20317	protein_coding	3.142259092	0.140223366	1.343423424	-0.10437764	0.916869667	1	0.140223366
ENSMUSG00000017747	Ghdc	80860	protein_coding	148.1797926	-0.140225178	0.08639833	1.62300797	0.104587701	0.255796319	0.140225178
ENSMUSG00000042377	Fam83g	69640	protein_coding	565.1230476	-0.140242123	0.094410761	1.485446377	0.137425618	0.309858682	0.140242123
ENSMUSG00000038280	Ostm1	14628	protein_coding	162.591141	-0.140289796	0.114013794	1.230463357	0.21852364	0.424602824	0.140289796
ENSMUSG00000025420	Katnal2	71206	protein_coding	5.264833715	-0.140294461	0.660529759	0.212396881	0.831797419	0.915204382	0.140294461
ENSMUSG00000032786	Alas1	11655	protein_coding	2578.379321	-0.140343419	0.127750151	1.098577323	0.271952474	0.487730576	0.140343419
ENSMUSG00000090247	Bloc1s1	14533	protein_coding	96.52255526	-0.14034462	0.12746756	1.101022258	0.27088697	0.486449404	0.14034462

ensembl_gene_id	external_gene_name	entrezgene_id	gene_biotype	baseMean	log2FoldChange	lfcSE	stat	pvalue	padj	absLog2FC
ENSMUSG00000041837	Pdcd7	50996	protein_coding	156.8995055	0.140385396	0.095177596	-1.474983625	0.140216946	0.314312833	0.140385396
ENSMUSG00000075595	Zfp652	268469	protein_coding	839.4536825	0.140478469	0.087186861	-1.61123439	0.107128645	0.260126811	0.140478469
ENSMUSG00000046719	Nxph3	104079	protein_coding	4.356429966	0.140489111	0.774573933	-0.181375987	0.856072466	1	0.140489111
ENSMUSG00000034303	Ccdc15	245902	protein_coding	39.47211795	0.140493183	0.197122465	-0.712720303	0.47601885	0.687930892	0.140493183
ENSMUSG00000037022	Mmaa	109136	protein_coding	257.2953813	-0.140513448	0.076795734	1.829703822	0.067294239	0.185483473	0.140513448
ENSMUSG00000031723	Txnl4b	234723	protein_coding	94.67669286	0.140557058	0.128533668	-1.093542728	0.274155579	0.490619315	0.140557058
ENSMUSG00000022185	Acin1	56215	protein_coding	1511.198482	0.140602625	0.037841797	-3.715537716	0.000202772	0.001599893	0.140602625
ENSMUSG00000031935	Med17	234959	protein_coding	348.382282	-0.140626553	0.085391848	1.64683815	0.099591318	0.246796371	0.140626553
ENSMUSG00000097277	2900076A07Rik	100504421	lncRNA	32.10222618	-0.140641719	0.223916459	0.628099066	0.529939045	0.727284417	0.140641719
ENSMUSG00000036371	Serbp1	66870	protein_coding	3247.029824	0.140683366	0.083360671	-1.687646755	0.091479058	0.231451688	0.140683366
ENSMUSG00000021243	Fcf1	73736	protein_coding	253.1261959	-0.140703634	0.123557202	1.13877323	0.25479775	0.467346556	0.140703634
ENSMUSG00000029570	Lfng	16848	protein_coding	171.058869	0.140714232	0.164682145	-0.854459551	0.392850412	0.615573746	0.140714232
ENSMUSG00000000171	Sdhd	66925	protein_coding	3460.198097	-0.140730134	0.086742184	1.622395552	0.104718681	0.255833124	0.140730134
ENSMUSG00000009995	Tafazzin	66826	protein_coding	589.9714738	-0.140733863	0.061332508	2.294604713	0.021755788	0.078410101	0.140733863
ENSMUSG00000034083	Ccdc174	232236	protein_coding	215.554625	-0.140756955	0.075068015	1.875058969	0.060784611	0.172473724	0.140756955
ENSMUSG00000013495	Tmem175	72392	protein_coding	161.006676	-0.140793103	0.098933786	1.423104359	0.154705905	0.336646899	0.140793103
ENSMUSG00000022125	Cln5	211286	protein_coding	318.2476164	-0.140903338	0.078970991	1.78424173	0.074384394	0.19938276	0.140903338
ENSMUSG00000017831	Rab5a	271457	protein_coding	849.3327004	0.140944967	0.067329922	-2.093348147	0.036318088	0.116638837	0.140944967
ENSMUSG00000006392	Med8	80509	protein_coding	413.1369198	-0.140959726	0.070661628	1.994855346	0.046058659	0.140153435	0.140959726
ENSMUSG00000038412	Higd1a	56295	protein_coding	2563.934196	0.140982549	0.126545035	-1.114089929	0.265240609	0.480125333	0.140982549
ENSMUSG00000043602	Zfp3	193043	protein_coding	43.78835612	0.140985199	0.197850465	-0.712584619	0.476102832	0.687930892	0.140985199
ENSMUSG00000017299	Dnttip1	76233	protein_coding	230.3419712	0.140996855	0.078684754	-1.791920896	0.073145638	0.197148208	0.140996855
ENSMUSG00000038240	Pdss2	71365	protein_coding	407.2387427	-0.141035191	0.073309791	1.923824763	0.054376552	0.158736855	0.141035191
ENSMUSG00000055612	Cdca7	66953	protein_coding	432.6264585	-0.141036577	0.123178121	1.144980747	0.252217133	0.464617944	0.141036577
ENSMUSG00000044709	Gemin7	69731	protein_coding	227.4774152	-0.141037557	0.078928373	1.786905655	0.073952738	0.198739711	0.141037557
ENSMUSG00000026817	Ak1	11636	protein_coding	13.00955309	-0.141043282	0.369419328	0.381797246	0.702611761	0.843113692	0.141043282
ENSMUSG00000035847	Ids	15931	protein_coding	324.9353823	-0.141055641	0.113566731	1.242050732	0.214217845	0.419245495	0.141055641
ENSMUSG00000005699	Pard6a	56513	protein_coding	44.79860983	-0.141056481	0.164174225	0.859187734	0.39023695	0.612497263	0.141056481
ENSMUSG00000039238	Zfp750	319530	protein_coding	101.8290454	-0.141065027	0.170217859	0.828732236	0.407255941	0.628133427	0.141065027
ENSMUSG00000059149	Mfsd4a	213006	protein_coding	1756.262415	-0.141133389	0.180633196	0.78132587	0.434610858	0.652884901	0.141133389
ENSMUSG00000045078	Rnf216	108086	protein_coding	469.7915932	-0.141149551	0.072379179	1.95014026	0.051159404	0.151705224	0.141149551
ENSMUSG00000073725	Lmbrd1	68421	protein_coding	645.0474803	-0.141151239	0.076643389	1.841662281	0.065524565	0.181983293	0.141151239
ENSMUSG00000073062	Zxdb	668166	protein_coding	49.24936466	-0.141200252	0.187390343	0.753508688	0.451144292	0.667666133	0.141200252
ENSMUSG00000040725	Hnrnpul1	232989	protein_coding	2018.114763	0.141207473	0.044886277	-3.145894092	0.0016558	0.009689537	0.141207473
ENSMUSG00000065581	Mir194-1	387189	miRNA	2.322930858	0.141232963	0.693096651	-0.20377095	0.838532495	1	0.141232963
ENSMUSG00000022210	Dhrs4	28200	protein_coding	402.4074178	-0.141263478	0.097334115	1.45132545	0.146689258	0.324504542	0.141263478
ENSMUSG00000061371	Zfp873	408062	protein_coding	64.44908187	-0.141345295	0.136284883	1.037131136	0.299674746	0.519697021	0.141345295
ENSMUSG00000025786	Zdhhc3	69035	protein_coding	1483.44268	0.141354793	0.064588791	-2.188534423	0.028630697	0.097285458	0.141354793
ENSMUSG00000021572	Cep72	74470	protein_coding	77.22189387	0.141376907	0.134537834	-1.050833823	0.293334918	0.512561987	0.141376907

ensembl_gene_id	external_gene_name	entrezgene_id	gene_biotype	baseMean	log2FoldChange	lfcSE	stat	pvalue	padj	absLog2FC
ENSMUSG00000030796	Tead2	21677	protein_coding	45.57470188	0.141407164	0.182734916	-0.77383768	0.439026787	0.65700846	0.141407164
ENSMUSG00000042772	Smg7	226517	protein_coding	870.4462135	0.141418787	0.064592241	-2.189408261	0.02856718	0.097089608	0.141418787
ENSMUSG00000118522	Gm31160	102633301	protein_coding	20.51492307	0.141441557	0.316404943	-0.447027015	0.654855555	0.812734264	0.141441557
ENSMUSG00000024479	Mal2	105853	protein_coding	3444.332555	-0.141463849	0.077119061	1.834356479	0.066601095	0.184167865	0.141463849
ENSMUSG00000047443	Erfe	227358	protein_coding	92.94328302	0.141465272	0.128251508	-1.103030089	0.270014099	0.485271256	0.141465272
ENSMUSG00000032816	Igdcc4	56741	protein_coding	899.2614112	0.141467818	0.089213672	-1.585719045	0.112803057	0.269506609	0.141467818
ENSMUSG00000032782	Cntrob	216846	protein_coding	162.6974529	0.141473446	0.110445825	-1.280930682	0.200218015	0.401310875	0.141473446
ENSMUSG00000018347	Zkscan6	52712	protein_coding	224.7153379	-0.141489984	0.085149977	1.66165616	0.096581736	0.240948524	0.141489984
ENSMUSG00000050919	Zfp366	238803	protein_coding	2.465416625	-0.141519751	1.742673467	0.081208416	0.935276207	1	0.141519751
ENSMUSG00000029797	Sspo	243369	protein_coding	83.30183518	-0.141532377	0.243862574	0.580377605	0.561660004	0.750060917	0.141532377
ENSMUSG00000053158	Fes	14159	protein_coding	112.9389638	-0.141604108	0.111680895	1.267934935	0.204821187	0.40732383	0.141604108
ENSMUSG00000024621	Csf1r	12978	protein_coding	4.959883036	-0.141634131	0.615831419	0.229988477	0.818100723	0.908812719	0.141634131
ENSMUSG00000062028	Irgc1	210145	protein_coding	5.620827915	0.141659904	0.556567578	-0.254524175	0.79909063	0.900333128	0.141659904
ENSMUSG00000064181	Rab3ip	216363	protein_coding	393.05894	-0.1416793	0.08838432	1.60299134	0.108936566	0.26324771	0.1416793
ENSMUSG00000029513	Prkab1	19079	protein_coding	1376.859034	-0.141688938	0.088804556	1.595514279	0.11059728	0.265929452	0.141688938
ENSMUSG00000021068	Nin	18080	protein_coding	83.06623531	-0.141710199	0.149286281	0.949251317	0.342492807	0.564520034	0.141710199
ENSMUSG00000019852	Arfgef3	215821	protein_coding	948.7429017	0.141728017	0.176869343	-0.801314771	0.422949442	0.64285201	0.141728017
ENSMUSG00000040721	Zfhx2	239102	protein_coding	71.6515451	0.141730664	0.323616113	-0.437959231	0.661415838	0.816921981	0.141730664
ENSMUSG00000077042	Mir574	100124451	miRNA	1.675876616	0.141751176	1.112935752	-0.127366899	0.898650014	1	0.141751176
ENSMUSG00000020522	Mfap3	216760	protein_coding	288.7674094	0.141790006	0.076611873	-1.850757601	0.064204433	0.179403047	0.141790006
ENSMUSG00000038591	Colec10	239447	protein_coding	1.008547246	-0.141851686	2.073177533	0.068422354	0.945449428	1	0.141851686
ENSMUSG00000031683	Lsm6	78651	protein_coding	270.7683395	0.141894095	0.099761042	-1.422339742	0.154927647	0.336996201	0.141894095
ENSMUSG00000045624	Esf1	66580	protein_coding	244.5976547	0.141947683	0.135216016	-1.049784535	0.293817187	0.513069369	0.141947683
ENSMUSG00000033763	Mtss2	244654	protein_coding	10.02930454	0.141971894	0.86435432	-0.164251963	0.869532796	0.935632034	0.141971894
ENSMUSG00000030393	Zik1	22775	protein_coding	4.115317907	0.141982838	0.754099517	-0.188281301	0.850656143	1	0.141982838
ENSMUSG00000101942	Gm19582	100503175	lncRNA	3.09434699	0.141992706	0.5601784	-0.253477653	0.799899128	1	0.141992706
ENSMUSG00000068856	Sf3b4	107701	protein_coding	163.2557641	0.142005456	0.138020289	-1.028873776	0.303538993	0.52400616	0.142005456
ENSMUSG00000078815	Cacng6	54378	protein_coding	0.61496024	0.142065916	1.416521024	-0.100292134	0.920112402	1	0.142065916
ENSMUSG00000027998	Plrg1	53317	protein_coding	468.0823841	0.142101763	0.076195547	-1.864961507	0.0621868	0.17503883	0.142101763
ENSMUSG00000042258	Isl1	16392	protein_coding	8.982573981	0.142105515	0.48864653	-0.290814538	0.771193166	0.885199039	0.142105515
ENSMUSG00000006641	Slc5a6	330064	protein_coding	358.654102	-0.142106661	0.18346632	0.774565386	0.438596517	0.656764359	0.142106661
ENSMUSG00000036352	Ubac1	98766	protein_coding	432.8361834	-0.142113898	0.084908188	1.673736087	0.094182478	0.236336338	0.142113898
ENSMUSG00000036751	Cox6b1	110323	protein_coding	4449.02711	-0.142115531	0.066601503	2.133818674	0.032857627	0.10826234	0.142115531
ENSMUSG00000000402	Egfl6	54156	protein_coding	2.687023648	-0.142166392	0.775743431	0.183264706	0.854590326	1	0.142166392
ENSMUSG00000057219	Armc7	276905	protein_coding	72.83403963	0.14216663	0.140772545	-1.009903109	0.312541713	0.533579433	0.14216663
ENSMUSG00000047412	Zbtb44	235132	protein_coding	482.601323	-0.142193071	0.135777302	1.047252145	0.294983301	0.514127096	0.142193071
ENSMUSG00000031207	Msn	17698	protein_coding	27.04623497	0.142201588	0.322835685	-0.440476671	0.659591905	0.8161622	0.142201588
ENSMUSG00000041215	Yeats2	208146	protein_coding	421.0575941	0.142222496	0.081534814	-1.744316184	0.081103971	0.21178858	0.142222496
ENSMUSG00000030779	Rbbp6	19647	protein_coding	633.7332516	0.142265703	0.0678487	-2.096808082	0.036010568	0.115831422	0.142265703

ensembl_gene_id	external_gene_name	entrezgene_id	gene_biotype	baseMean	log2FoldChange	lfcSE	stat	pvalue	padj	absLog2FC
ENSMUSG00000045917	Tmem268	230279	protein_coding	379.6886918	0.142276882	0.10371607	-1.371792077	0.170128174	0.359776754	0.142276882
ENSMUSG00000072945	Ripply1	622473	protein_coding	8.667513059	-0.142283558	0.497804714	0.285822038	0.775014428	0.887804402	0.142283558
ENSMUSG00000039385	Cdh6	12563	protein_coding	2.304618159	0.142284816	1.118623878	-0.127196298	0.898785035	1	0.142284816
ENSMUSG00000028576	Ift74	67694	protein_coding	100.6256802	0.142297601	0.1331843	-1.068426235	0.285328288	0.503786095	0.142297601
ENSMUSG00000020780	Srp68	217337	protein_coding	640.884532	0.142314847	0.067712848	-2.101740677	0.035575998	0.114836207	0.142314847
ENSMUSG00000027318	Adam33	110751	protein_coding	2.199383616	-0.142347903	1.48833061	0.095642663	0.92380438	1	0.142347903
ENSMUSG00000020409	Slu7	193116	protein_coding	690.6729968	-0.142355965	0.073605159	1.934048728	0.053107137	0.156162974	0.142355965
ENSMUSG00000027164	Traf6	22034	protein_coding	399.9330407	0.142434844	0.079328628	-1.795503697	0.072573484	0.196079582	0.142434844
ENSMUSG00000036916	Zfp280c	208968	protein_coding	95.05042106	-0.142443001	0.13878245	1.026376177	0.304714303	0.524882862	0.142443001
ENSMUSG00000040822	1700123O20Rik	58248	protein_coding	319.2678475	0.142486438	0.082300405	-1.731296923	0.08339882	0.216412337	0.142486438
ENSMUSG00000026554	Dcaf8	98193	protein_coding	1267.788483	-0.1425366	0.06185844	2.30423852	0.021209258	0.076884085	0.1425366
ENSMUSG00000047786	Lix1	66643	protein_coding	7.89025562	0.14256569	0.474019563	-0.300759084	0.763598211	0.881390987	0.14256569
ENSMUSG00000058298	Mcm9	71567	protein_coding	175.086336	-0.142595973	0.09728278	1.46578843	0.142705934	0.318422618	0.142595973
ENSMUSG00000025956	Mettl21a	67099	protein_coding	92.11944108	0.142637372	0.142150783	-1.003423047	0.315656789	0.53655334	0.142637372
ENSMUSG00000055639	Dach1	13134	protein_coding	31.74633781	-0.142707267	0.225449822	0.632989044	0.526740798	0.725185288	0.142707267
ENSMUSG00000040044	Orc3	50793	protein_coding	310.357224	-0.142856973	0.075627205	1.888962756	0.058896819	0.168536142	0.142856973
ENSMUSG00000024082	Ndufaf7	73694	protein_coding	222.7307749	-0.142949252	0.101564498	1.407472649	0.159287282	0.343403417	0.142949252
ENSMUSG00000070371	Prss36	77613	protein_coding	26.06666559	0.142994366	0.205609389	-0.69546613	0.48676323	0.695624496	0.142994366
ENSMUSG00000030272	Camk1	52163	protein_coding	238.3051859	-0.143037327	0.107500965	1.330567847	0.183331248	0.37815791	0.143037327
ENSMUSG00000028599	Tnfrsf1b	21938	protein_coding	292.2581752	-0.143042196	0.13941355	1.026027933	0.304878418	0.525029915	0.143042196
ENSMUSG00000022752	Tomm70a	28185	protein_coding	1477.672194	0.143057634	0.081543007	-1.754382627	0.079364966	0.208654479	0.143057634
ENSMUSG00000026589	Sec16b	89867	protein_coding	458.8487622	-0.143068665	0.07352421	1.9458715	0.051670177	0.152917797	0.143068665
ENSMUSG00000032842	Abcc10	224814	protein_coding	352.9141666	0.143115349	0.133299885	-1.073634453	0.28298655	0.50115173	0.143115349
ENSMUSG00000036198	Arhgap36	75404	protein_coding	2.230688868	0.143120635	1.376718714	-0.10395779	0.917202846	1	0.143120635
ENSMUSG00000026131	Dst	13518	protein_coding	3941.365959	-0.143124414	0.160136193	0.893766806	0.371446673	0.593868753	0.143124414
ENSMUSG00000086186	Ccdc34os	75517	lncRNA	1.163777638	0.143220052	1.995832425	-0.071759558	0.942793258	1	0.143220052
ENSMUSG00000029735	Tpk1	29807	protein_coding	359.0778597	-0.143236148	0.074583186	1.920488469	0.054796228	0.15967971	0.143236148
ENSMUSG00000033773	Rpap2	231571	protein_coding	146.7359965	-0.143280586	0.097651847	1.467259367	0.142305513	0.31779121	0.143280586
ENSMUSG00000025324	Atp10a	11982	protein_coding	19.57432217	0.143440679	0.361937386	-0.39631352	0.691873755	0.836060529	0.143440679
ENSMUSG00000021374	Nup153	218210	protein_coding	708.6936044	0.143447551	0.061661759	-2.326361649	0.019999266	0.073392861	0.143447551
ENSMUSG00000000148	Brat1	231841	protein_coding	218.5106924	0.143462782	0.117505476	-1.220902953	0.222122766	0.429222486	0.143462782
ENSMUSG00000030805	Stx4a	20909	protein_coding	294.3675415	-0.14346539	0.117002914	1.226169374	0.220134944	0.426485613	0.14346539
ENSMUSG00000060166	Zdhhc8	27801	protein_coding	77.79322777	-0.143472067	0.158974831	0.902482902	0.3668004	0.589522317	0.143472067
ENSMUSG00000040009	Gnaz	14687	protein_coding	9.521500254	0.143576367	0.367412279	-0.39077727	0.695961878	0.838607402	0.143576367
ENSMUSG00000028693	Nasp	50927	protein_coding	428.0915598	-0.143610408	0.115335472	1.245153858	0.2130752	0.417801455	0.143610408
ENSMUSG00000039545	Flicr	78185	lncRNA	2.190747627	0.143624926	1.233363121	-0.11644983	0.907296045	1	0.143624926
ENSMUSG00000039156	Stim2	116873	protein_coding	548.971765	-0.143645678	0.126076021	1.139357643	0.254554015	0.467148794	0.143645678
ENSMUSG00000041797	Abca9	217262	protein_coding	3.530862016	-0.143669806	1.55725913	0.092258124	0.926492958	1	0.143669806
ENSMUSG00000026072	Il1r1	16177	protein_coding	304.532062	0.143679373	0.136405702	-1.053323809	0.292192611	0.511151353	0.143679373

ensembl_gene_id	external_gene_name	entrezgene_id	gene_biotype	baseMean	log2FoldChange	lfcSE	stat	pvalue	padj	absLog2FC
ENSMUSG00000034871	Fam151a	230579	protein_coding	0.787522863	-0.143795386	1.95826814	0.073429876	0.941464044	1	0.143795386
ENSMUSG00000038646	Ramac	67148	protein_coding	295.1300081	-0.143809191	0.118673416	1.211806279	0.225586544	0.433049501	0.143809191
ENSMUSG00000003555	Cyp17a1	13074	protein_coding	2.035742069	-0.14384171	1.219562726	0.117945315	0.906110987	1	0.14384171
ENSMUSG00000018604	Tbx3	21386	protein_coding	162.7735875	0.143844022	0.27359172	-0.525761605	0.599053854	0.776435029	0.143844022
ENSMUSG00000097006	9530082P21Rik	638247	lncRNA	101.3756101	0.143875865	0.175474822	-0.819923127	0.412259932	0.632329199	0.143875865
ENSMUSG00000038235	F11r	16456	protein_coding	2532.069226	-0.143913059	0.059369127	2.424038644	0.015348972	0.059503296	0.143913059
ENSMUSG00000038909	Kat7	217127	protein_coding	892.1738583	0.143916953	0.061274025	-2.34874326	0.01883689	0.070067307	0.143916953
ENSMUSG00000028479	Gne	50798	protein_coding	6891.926151	-0.143961874	0.137726902	1.045270541	0.295897949	0.515123206	0.143961874
ENSMUSG00000038034	Igsf8	140559	protein_coding	340.3466292	-0.144008091	0.096678554	1.489555694	0.136341099	0.308134243	0.144008091
ENSMUSG00000028409	Smu1	74255	protein_coding	969.6134858	0.144013782	0.082398229	-1.747777632	0.08050254	0.210915627	0.144013782
ENSMUSG00000020283	Pex13	72129	protein_coding	411.1283689	-0.14403403	0.061437597	2.344395567	0.019057947	0.070692483	0.14403403
ENSMUSG00000027550	Lrrcc1	71710	protein_coding	206.3337199	-0.144054457	0.15035799	0.958076499	0.338024185	0.559780524	0.144054457
ENSMUSG00000000154	Slc22a18	18400	protein_coding	934.0731162	-0.14407841	0.102138226	1.410621811	0.158356157	0.342200154	0.14407841
ENSMUSG00000047013	Fbxo41	330369	protein_coding	4.583202195	0.144108044	1.072553622	-0.134359757	0.893118102	0.947328174	0.144108044
ENSMUSG00000002379	Ndufa11	69875	protein_coding	868.9266312	0.144117667	0.090984844	-1.583974444	0.113199535	0.270127107	0.144117667
ENSMUSG00000036938	Try5	103964	protein_coding	0.688408279	0.144117814	2.165730331	-0.066544672	0.946944194	1	0.144117814
ENSMUSG00000003068	Stk11	20869	protein_coding	917.3606012	0.144135979	0.068535762	-2.103076922	0.035459046	0.114637957	0.144135979
ENSMUSG00000101174	Hoxd4	15436	protein_coding	24.44118061	-0.144141378	0.351213407	0.410409669	0.681505455	0.829602026	0.144141378
ENSMUSG00000031887	Tradd	71609	protein_coding	875.0650637	-0.144168096	0.084967061	1.69675278	0.089743409	0.228322063	0.144168096
ENSMUSG00000073043	Atoh1	11921	protein_coding	975.0809339	0.144182762	0.222347895	-0.648455708	0.516690249	0.717449153	0.144182762
ENSMUSG00000029559	2210016L21Rik	72357	protein_coding	279.1918441	-0.144208112	0.076123348	1.894400553	0.058171863	0.166982469	0.144208112
ENSMUSG00000002825	Qtrt1	60507	protein_coding	141.0349038	-0.14422675	0.123971931	1.163382305	0.244674425	0.456250391	0.14422675
ENSMUSG00000025354	Dnajc14	74330	protein_coding	1087.44961	-0.144247371	0.043248785	3.33529305	0.000852096	0.00556186	0.144247371
ENSMUSG00000023456	Tpi1	21991	protein_coding	5783.675939	-0.144261699	0.057728197	2.498981538	0.01245508	0.050511589	0.144261699
ENSMUSG00000020474	Polm	54125	protein_coding	123.1791818	-0.144300109	0.107111362	1.347197039	0.177916782	0.370319855	0.144300109
ENSMUSG00000031657	Heatr3	234549	protein_coding	235.2878471	-0.144328026	0.150973927	0.955979809	0.339082435	0.560532006	0.144328026
ENSMUSG00000034312	Iqsec1	232227	protein_coding	281.1373398	0.144330854	0.140075692	-1.030377593	0.302832788	0.5233341	0.144330854
ENSMUSG00000053574	4930563E22Rik	75304	protein_coding	4.053925356	-0.144348937	0.685159021	0.210679466	0.833137399	1	0.144348937
ENSMUSG00000005378	Bud23	66138	protein_coding	341.3918117	0.144365477	0.085893721	-1.680745404	0.092812377	0.233822059	0.144365477
ENSMUSG00000051391	Ywhag	22628	protein_coding	766.9862369	-0.144399607	0.08757981	1.648777355	0.099193256	0.246105382	0.144399607
ENSMUSG00000032080	Apoa4	11808	protein_coding	19.15411222	0.144449811	1.106416613	-0.130556437	0.896126206	0.948996349	0.144449811
ENSMUSG00000054720	Lrrc8c	100604	protein_coding	9.6039644	-0.144470543	0.463549431	0.311661569	0.755297738	0.877032235	0.144470543
ENSMUSG00000043059	Zfp513	101023	protein_coding	219.4775546	0.144512445	0.110764579	-1.304681028	0.192001484	0.389807821	0.144512445
ENSMUSG00000055652	Klhl25	207952	protein_coding	211.2225385	0.144607202	0.089739845	-1.611404623	0.10709156	0.260075004	0.144607202
ENSMUSG00000063646	Jakmip1	76071	protein_coding	117.2701958	0.14462592	0.107902402	-1.340340138	0.180134788	0.37375875	0.14462592
ENSMUSG00000033161	Atp1a1	11928	protein_coding	30040.49394	-0.144645557	0.128485172	1.125776271	0.26026023	0.473946662	0.144645557
ENSMUSG00000079477	Rab7	19349	protein_coding	1138.312815	-0.14464811	0.057066041	2.534749354	0.011252781	0.046796074	0.14464811
ENSMUSG00000007379	Dennd2c	329727	protein_coding	24.28135563	0.144681679	0.231642197	-0.624591205	0.532239393	0.728866518	0.144681679
ENSMUSG00000026743	Mllt10	17354	protein_coding	494.2928466	-0.144725416	0.057847741	2.501833499	0.012355202	0.050180472	0.144725416

ensembl_gene_id	external_gene_name	entrezgene_id	gene_biotype	baseMean	log2FoldChange	lfcSE	stat	pvalue	padj	absLog2FC
ENSMUSG00000041886	Macc1	238455	protein_coding	83.5106868	-0.144772081	0.204508427	0.707902765	0.479005642	0.690541222	0.144772081
ENSMUSG00000018160	Med1	19014	protein_coding	839.5223917	0.144795428	0.045905495	-3.154206894	0.001609349	0.009447848	0.144795428
ENSMUSG00000075543	Urad	231903	protein_coding	16.55649068	0.144860183	0.29932529	-0.483955709	0.628417303	0.796934675	0.144860183
ENSMUSG00000075040	Zfp408	381410	protein_coding	176.5478492	-0.144883171	0.118768815	1.219875528	0.222512064	0.429533455	0.144883171
ENSMUSG00000038425	Poli	26447	protein_coding	67.58444083	-0.144899832	0.14575136	0.994157672	0.320146112	0.541384622	0.144899832
ENSMUSG00000024006	Stk38	106504	protein_coding	956.1633139	-0.14490986	0.05486962	2.640985289	0.008266531	0.036559585	0.14490986
ENSMUSG00000025968	Ndufs1	227197	protein_coding	2111.139465	-0.145002567	0.075210849	1.927947477	0.05386166	0.157763648	0.145002567
ENSMUSG00000028991	Mtor	56717	protein_coding	963.0921367	0.145045602	0.053801653	-2.695932074	0.007019198	0.031967742	0.145045602
ENSMUSG00000078599	Skint8	639774	protein_coding	1.809730397	-0.14509077	1.423772282	0.101905882	0.918831381	1	0.14509077
ENSMUSG00000037486	Asxl2	75302	protein_coding	642.0545228	-0.145105092	0.060221892	2.409507356	0.015974074	0.061433595	0.145105092
ENSMUSG00000022789	Dnm1l	74006	protein_coding	1131.267453	0.145106225	0.065511489	-2.214973691	0.02676187	0.092148775	0.145106225
ENSMUSG00000035278	Plekhj1	78670	protein_coding	215.0580731	-0.1451095	0.07491124	1.937085803	0.052734856	0.155400323	0.1451095
ENSMUSG00000070733	Fryl	72313	protein_coding	3009.082645	0.145122104	0.128981337	-1.125140331	0.260529573	0.474068167	0.145122104
ENSMUSG00000015467	Egfl8	81701	protein_coding	10.17254917	-0.145140361	0.414269331	0.350352658	0.726074051	0.858172551	0.145140361
ENSMUSG00000001665	Gstt3	103140	protein_coding	587.8363315	0.14516053	0.114663595	-1.265968771	0.205524263	0.408230416	0.14516053
ENSMUSG00000032034	Kcnj5	16521	protein_coding	3.725660928	0.145207187	1.301002893	-0.111611733	0.911131269	1	0.145207187
ENSMUSG00000024561	Mbd1	17190	protein_coding	758.3660209	-0.145259016	0.070837271	2.050601515	0.040305769	0.126662125	0.145259016
ENSMUSG00000057193	Slc44a2	68682	protein_coding	345.6675785	-0.145434454	0.108034029	1.346191156	0.178240877	0.37057407	0.145434454
ENSMUSG00000047414	Flrt2	399558	protein_coding	22.19431058	0.145454923	0.24006527	-0.605897403	0.544582884	0.736796542	0.145454923
ENSMUSG00000026987	Baz2b	407823	protein_coding	728.7810802	-0.145466887	0.100935726	1.441183341	0.149532894	0.328900667	0.145466887
ENSMUSG00000039318	Rab3gap2	98732	protein_coding	700.2376524	0.145483117	0.059477557	-2.446017053	0.014444417	0.056578534	0.145483117
ENSMUSG00000096917	2500002B13Rik	78353	lncRNA	14.80205201	-0.145522167	0.49644003	0.293131412	0.769421707	0.884482916	0.145522167
ENSMUSG00000024672	Ms4a7	109225	protein_coding	11.5790626	-0.145547021	0.366630641	0.396985425	0.691378209	0.835769466	0.145547021
ENSMUSG00000021548	Ccnh	66671	protein_coding	154.3866665	0.145652888	0.116520996	-1.250014106	0.211294394	0.415642124	0.145652888
ENSMUSG00000049680	Urgcp	72046	protein_coding	997.8816612	0.145671134	0.077984032	-1.86796104	0.061767502	0.174214948	0.145671134
ENSMUSG00000001173	Ocrl	320634	protein_coding	280.1648003	-0.145677095	0.098986379	1.47168829	0.141105071	0.315703719	0.145677095
ENSMUSG00000020521	Rnft1	76892	protein_coding	500.9125185	0.145684672	0.066518939	-2.190123203	0.028515303	0.096986852	0.145684672
ENSMUSG00000040525	Cblc	80794	protein_coding	902.5919015	-0.145736187	0.079461944	1.834037535	0.066648423	0.184267881	0.145736187
ENSMUSG00000029538	Srsf9	108014	protein_coding	332.6248162	0.145772993	0.089717068	-1.624807817	0.104203515	0.255086991	0.145772993
ENSMUSG00000039481	Nrtn	18188	protein_coding	15.69840363	0.145798939	0.304865423	-0.478240325	0.632479164	0.799512805	0.145798939
ENSMUSG00000117011	E130008D07Rik	545207	lncRNA	5.663770056	-0.145818592	0.592449192	0.246128434	0.805582813	0.904254429	0.145818592
ENSMUSG00000009145	Dqx1	93838	protein_coding	512.9304682	-0.145964937	0.10257782	1.422967814	0.154745486	0.336688663	0.145964937
ENSMUSG00000066551	Hmgb1	15289	protein_coding	1435.588607	0.145968861	0.143890358	-1.01444505	0.310370437	0.53139398	0.145968861
ENSMUSG00000073927	Or52h7	259050	protein_coding	2.055307622	-0.146031743	1.298845839	0.112431929	0.91048094	1	0.146031743
ENSMUSG00000020708	Psmc5	19184	protein_coding	797.6732475	0.14603488	0.090325639	-1.616760006	0.105930096	0.258135524	0.14603488
ENSMUSG00000063281	Zfp35	22694	protein_coding	141.1866167	0.146047842	0.120572967	-1.211281812	0.225787417	0.433362785	0.146047842
ENSMUSG00000032199	Polr2m	28015	protein_coding	1141.664073	0.14604897	0.073344328	-1.991278318	0.046450298	0.141033318	0.14604897
ENSMUSG00000022774	Ncbp2	68092	protein_coding	178.869323	0.146093921	0.151537956	-0.964074776	0.335008438	0.557245099	0.146093921
ENSMUSG00000111356	Rpl32l	621697	processed_pseudogene	12.21977783	0.146134235	0.338335155	-0.431921521	0.665798452	0.819849053	0.146134235

ensembl_gene_id	external_gene_name	entrezgene_id	gene_biotype	baseMean	log2FoldChange	lfcSE	stat	pvalue	padj	absLog2FC
ENSMUSG00000024118	Tedc2	72016	protein_coding	55.25510727	-0.146147613	0.158062395	0.924619759	0.355163731	0.577906578	0.146147613
ENSMUSG00000014907	Naf1	234344	protein_coding	92.9100874	0.146149947	0.139735408	-1.045904899	0.295604942	0.514721638	0.146149947
ENSMUSG00000034602	Mon2	67074	protein_coding	1641.660205	0.146179206	0.037729176	-3.874434123	0.000106873	0.000913981	0.146179206
ENSMUSG00000024317	Rnf138	56515	protein_coding	193.4523752	-0.146196555	0.105332674	1.387950665	0.165152068	0.352113109	0.146196555
ENSMUSG00000059183	Mtfmt	69606	protein_coding	170.560391	-0.146245656	0.102722063	1.423702479	0.154532616	0.336447148	0.146245656
ENSMUSG00000024325	Ring1	19763	protein_coding	252.9407056	-0.146263334	0.08444657	1.732022197	0.08326961	0.216212948	0.146263334
ENSMUSG00000034430	Zxdc	80292	protein_coding	279.5139735	-0.146363094	0.091915664	1.592362904	0.111303186	0.26704356	0.146363094
ENSMUSG00000040761	Spen	56381	protein_coding	788.2303577	0.146443952	0.182818199	-0.801035964	0.423110826	0.642978943	0.146443952
ENSMUSG00000001865	Cpa3	12873	protein_coding	1.531213025	-0.14645552	1.316487145	0.111247208	0.911420317	1	0.14645552
ENSMUSG00000032330	Cox7a2	12866	protein_coding	2799.102643	-0.146467257	0.083531747	1.753432228	0.079527843	0.208994717	0.146467257
ENSMUSG00000050705	2310061I04Rik	69662	protein_coding	215.5701841	-0.146476616	0.124955405	1.172231137	0.241104271	0.451758428	0.146476616
ENSMUSG00000080574	Mir466i	100316665	miRNA	1.551329845	-0.146570712	0.839599343	0.174572209	0.861415787	1	0.146570712
ENSMUSG00000057497	Fam136a	66488	protein_coding	425.446747	-0.146623179	0.12230049	1.198876458	0.230575987	0.439134108	0.146623179
ENSMUSG00000048439	Nupl2	231042	protein_coding	109.9223268	0.146625546	0.121493592	-1.206858268	0.227486732	0.436052025	0.146625546
ENSMUSG00000033016	Nfatc1	18018	protein_coding	134.4118108	-0.146631426	0.094960895	1.54412431	0.122558221	0.285669361	0.146631426
ENSMUSG00000036957	Lrfn3	233067	protein_coding	146.7846539	0.146638729	0.21146133	-0.693454113	0.488024613	0.696186289	0.146638729
ENSMUSG00000074826	Gm10767	100038538	protein_coding	25.06724779	-0.146669899	0.198960214	0.737182054	0.461011645	0.674995696	0.146669899
ENSMUSG00000036941	Elac1	114615	protein_coding	150.1587693	0.146672168	0.127524571	-1.150148294	0.250082798	0.462419363	0.146672168
ENSMUSG00000025997	Ikzf2	22779	protein_coding	202.1234934	0.146679679	0.146856353	-0.998796961	0.317893059	0.539328021	0.146679679
ENSMUSG00000014075	Dynlt2b	66061	protein_coding	22.99217873	0.146697733	0.240284404	-0.610517083	0.541519331	0.735467298	0.146697733
ENSMUSG00000056342	Usp34	17847	protein_coding	1494.162943	0.146824655	0.128970796	-1.138433345	0.254939577	0.467516411	0.146824655
ENSMUSG00000031952	Chst5	56773	protein_coding	1.52594337	0.146826344	0.828065128	-0.177312556	0.859262889	1	0.146826344
ENSMUSG00000038175	Mylip	218203	protein_coding	218.6844744	-0.146904962	0.089967542	1.632866245	0.102497131	0.252105692	0.146904962
ENSMUSG00000002345	Borcs8	72368	protein_coding	190.4113525	-0.146945391	0.083115968	1.767956204	0.077068226	0.204680714	0.146945391
ENSMUSG00000028960	Ube4b	63958	protein_coding	641.0509984	0.146958218	0.079613883	-1.845886826	0.06490864	0.180947387	0.146958218
ENSMUSG00000044348	Slc25a53	67062	protein_coding	25.35956333	-0.146977991	0.205081269	0.716681694	0.473570524	0.68565173	0.146977991
ENSMUSG00000087714	Gm25827	115490026	snRNA	0.6877759	0.146979359	1.669689937	-0.088027936	0.929854473	1	0.146979359
ENSMUSG00000042790	Rnf214	235315	protein_coding	261.7405882	0.146983789	0.085643802	-1.716222131	0.086121394	0.221735064	0.146983789
ENSMUSG00000024960	Plcb3	18797	protein_coding	4777.978611	-0.147009188	0.056076533	2.621581249	0.00875229	0.038236855	0.147009188
ENSMUSG00000061613	U2af1	108121	protein_coding	426.0997156	-0.147076656	0.132288456	1.111787536	0.266229512	0.481307968	0.147076656
ENSMUSG00000020570	Sypl	19027	protein_coding	1930.627441	0.147107011	0.140173812	-1.049461443	0.293965792	0.513159947	0.147107011
ENSMUSG00000085227	6330418K02Rik	69004	lncRNA	8.501587164	-0.147155345	0.403262822	0.364911758	0.715177277	0.852123921	0.147155345
ENSMUSG00000037331	Larp1	73158	protein_coding	1958.681898	0.147158716	0.073576396	-2.000080513	0.045491571	0.138914164	0.147158716
ENSMUSG00000031505	Naxd	69225	protein_coding	530.0679456	-0.147194002	0.068322567	2.154398022	0.031208972	0.104013109	0.147194002
ENSMUSG00000090191	9230105E05Rik	320626	lncRNA	80.41281884	-0.147226743	0.146542973	1.004666009	0.315057701	0.535990818	0.147226743
ENSMUSG00000074406	Zfp628	232816	protein_coding	126.3487006	0.147244921	0.139519751	-1.055369722	0.291256265	0.510066755	0.147244921
ENSMUSG00000021890	Eaf1	74427	protein_coding	466.6377235	0.147253102	0.058423506	-2.520442764	0.01172073	0.048232278	0.147253102
ENSMUSG00000045519	Zfp560	434377	protein_coding	51.67393339	-0.147281636	0.164889125	0.893216185	0.371741412	0.594020826	0.147281636
ENSMUSG00000002910	Arrdc2	70807	protein_coding	220.7885124	0.147286896	0.181836916	-0.809994465	0.417943357	0.637944042	0.147286896

ensembl_gene_id	external_gene_name	entrezgene_id	gene_biotype	baseMean	log2FoldChange	lfcSE	stat	pvalue	padj	absLog2FC
ENSMUSG00000029352	Crybb3	12962	protein_coding	9.396343387	0.147288446	0.567267393	-0.259645535	0.795137209	0.898207529	0.147288446
ENSMUSG00000021639	Gtf2h2	23894	protein_coding	183.3134357	0.147357818	0.080124079	-1.839120267	0.065897499	0.182803846	0.147357818
ENSMUSG00000030880	Polr3e	26939	protein_coding	334.7993247	-0.147410012	0.109168599	1.350296814	0.176920794	0.369037513	0.147410012
ENSMUSG00000068921	Dap3	65111	protein_coding	683.7040482	-0.147483353	0.071072672	2.075106369	0.037976678	0.120977402	0.147483353
ENSMUSG00000038668	Lpar1	14745	protein_coding	489.6090911	-0.147490881	0.074683245	1.974885818	0.048281107	0.1452832	0.147490881
ENSMUSG00000003778	Brd8	78656	protein_coding	690.7837688	0.147545936	0.080012611	-1.844033508	0.065178257	0.181269925	0.147545936
ENSMUSG00000027580	Helz2	229003	protein_coding	1644.243965	0.147618105	0.18908173	-0.780710572	0.434972741	0.653309672	0.147618105
ENSMUSG00000041079	Rwdd2b	53858	protein_coding	196.2004387	-0.147633572	0.145775377	1.01274697	0.311181037	0.532085855	0.147633572
ENSMUSG00000038855	Itpkb	320404	protein_coding	157.3725608	0.147644813	0.128009245	-1.153391794	0.248749608	0.460883095	0.147644813
ENSMUSG00000035944	Ttc38	239570	protein_coding	1139.337446	0.147685263	0.09577314	-1.542032175	0.123065775	0.286482691	0.147685263
ENSMUSG00000034501	Pcnx4	67708	protein_coding	63.1862134	0.147739789	0.143775885	-1.027570026	0.30415213	0.524504007	0.147739789
ENSMUSG00000021209	Ppp4r4	74521	protein_coding	7.848079816	0.147783642	0.413345142	-0.357530855	0.720694432	0.855242013	0.147783642
ENSMUSG00000040850	Psme4	103554	protein_coding	2104.015171	0.147793898	0.083576979	-1.768356536	0.077001318	0.204535912	0.147793898
ENSMUSG00000024354	Slc23a1	20522	protein_coding	9.420136467	0.147885247	0.498072044	-0.296915374	0.766531118	0.882740229	0.147885247
ENSMUSG00000033542	Arhgef5	54324	protein_coding	1822.326907	-0.147912609	0.076131836	1.942848298	0.05203449	0.153913232	0.147912609
ENSMUSG00000021669	Cert1	68018	protein_coding	820.7787678	0.147919782	0.110784093	-1.335207767	0.181808356	0.376049998	0.147919782
ENSMUSG00000055963	Triqk	208820	protein_coding	75.60360982	0.147957296	0.17402477	-0.850208256	0.395209312	0.617453792	0.147957296
ENSMUSG00000001376	Vps50	73288	protein_coding	488.8429866	0.148030648	0.091045669	-1.625894455	0.10397211	0.254671525	0.148030648
ENSMUSG00000036817	Sun1	77053	protein_coding	1066.772875	-0.148034804	0.090836199	1.629689557	0.103167126	0.253263255	0.148034804
ENSMUSG00000035498	Cdcp1	109332	protein_coding	1515.50675	0.148036815	0.06919615	-2.139379357	0.032404957	0.107027093	0.148036815
ENSMUSG00000061959	Ces1e	13897	protein_coding	215.2919061	-0.14806101	0.116170705	1.274512459	0.20248186	0.404473864	0.14806101
ENSMUSG00000068252	Apol7b	278679	protein_coding	1.310323023	-0.148064162	1.31642155	0.112474733	0.910447003	1	0.148064162
ENSMUSG00000038736	Nudcd1	67429	protein_coding	418.0448302	0.148073233	0.114282302	-1.295679466	0.195085939	0.394262295	0.148073233
ENSMUSG00000000916	Nsun5	100609	protein_coding	103.7024453	0.14808885	0.138744096	-1.067352448	0.285812713	0.504312604	0.14808885
ENSMUSG00000040446	Rprd1a	225283	protein_coding	96.37218463	-0.148119612	0.118628695	1.248598508	0.211811958	0.416114999	0.148119612
ENSMUSG00000073678	Pgap1	241062	protein_coding	572.4578457	0.148131484	0.078553253	-1.885746014	0.059329189	0.169479714	0.148131484
ENSMUSG00000027010	Slc25a12	78830	protein_coding	326.8098605	0.148236071	0.174765385	-0.848200406	0.396326371	0.618730733	0.148236071
ENSMUSG00000024300	Myo1f	17916	protein_coding	17.11563977	0.148313366	0.395058252	-0.375421513	0.707347008	0.846814623	0.148313366
ENSMUSG00000079418	Atg4a	666468	protein_coding	173.4152957	-0.148319481	0.108859493	1.362485498	0.173044718	0.363688435	0.148319481
ENSMUSG00000030452	Nipa2	93790	protein_coding	536.1609971	0.148346943	0.066406469	-2.233923075	0.025488135	0.088668856	0.148346943
ENSMUSG00000034616	Ssh3	245857	protein_coding	1175.850803	-0.148401398	0.059436087	2.496823131	0.012531144	0.050745295	0.148401398
ENSMUSG00000047728	Ly6g2	223631	protein_coding	142.8527222	0.148406258	0.189870474	-0.781618411	0.434438864	0.652884901	0.148406258
ENSMUSG00000087230	Mroh3	76422	protein_coding	78.80372371	-0.148411194	0.209415167	0.708693628	0.47851462	0.690104577	0.148411194
ENSMUSG00000021811	Dnajc9	108671	protein_coding	193.2649202	0.148414171	0.184432935	-0.804705361	0.420989725	0.640805153	0.148414171
ENSMUSG00000037573	Tob1	22057	protein_coding	2138.375132	-0.148443941	0.115335874	1.287057841	0.198074128	0.398335908	0.148443941
ENSMUSG00000001741	Il16	16170	protein_coding	14.08138229	0.14845343	0.418693003	-0.354563914	0.722916314	0.856549354	0.14845343
ENSMUSG00000031878	Nae1	234664	protein_coding	277.1434762	-0.148489336	0.095459801	1.555516934	0.119823005	0.281753823	0.148489336
ENSMUSG00000028656	Cap1	12331	protein_coding	3002.012387	-0.148506912	0.060569013	2.451862829	0.014211883	0.055893077	0.148506912
ENSMUSG00000071252	2210408I21Rik	72371	protein_coding	42.66694025	-0.148524097	0.154795742	0.959484383	0.337314783	0.559393767	0.148524097

ensembl_gene_id	external_gene_name	entrezgene_id	gene_biotype	baseMean	log2FoldChange	lfcSE	stat	pvalue	padj	absLog2FC
ENSMUSG00000026727	Rsu1	20163	protein_coding	186.0108377	0.148538461	0.110217255	-1.34768791	0.177758782	0.370076028	0.148538461
ENSMUSG00000024053	Emilin2	246707	protein_coding	4.570408644	-0.148554024	0.887112512	0.167457929	0.867009748	0.933936484	0.148554024
ENSMUSG00000002748	Baz1b	22385	protein_coding	1482.053825	0.148559454	0.072306204	-2.054587929	0.039918823	0.125757237	0.148559454
ENSMUSG00000035093	Secisbp2l	70354	protein_coding	1257.879835	0.148561483	0.094085993	-1.578996815	0.114336788	0.272029637	0.148561483
ENSMUSG00000074922	Fam122a	68034	protein_coding	78.0497284	0.14860527	0.155135028	-0.957909197	0.338108548	0.559860817	0.14860527
ENSMUSG00000025185	Loxl4	67573	protein_coding	5.879074114	-0.148609345	0.748678474	0.198495549	0.842657367	0.922115416	0.148609345
ENSMUSG00000108659	Gm34121	102637256	lncRNA	9.349209097	-0.148611826	0.415156719	0.357965605	0.720369054	0.855204229	0.148611826
ENSMUSG00000033909	Usp36	72344	protein_coding	339.5713787	0.148639279	0.115047477	-1.291982089	0.196363347	0.395987827	0.148639279
ENSMUSG00000015659	Serac1	321007	protein_coding	75.06439237	-0.148646848	0.15606579	0.952462726	0.340862361	0.562506348	0.148646848
ENSMUSG00000001815	Evx2	14029	protein_coding	8.993358845	0.1486565	0.540726032	-0.274920184	0.78337756	0.891264005	0.1486565
ENSMUSG00000028898	Trnau1ap	71787	protein_coding	243.1865703	0.148677489	0.093496931	-1.590185779	0.111792935	0.26786833	0.148677489
ENSMUSG00000031583	Wrn	22427	protein_coding	330.3244311	-0.148680992	0.080551044	1.845798443	0.064921477	0.180947387	0.148680992
ENSMUSG00000090550	Prlh	623503	protein_coding	0.880501033	-0.14873706	1.344428498	0.110632184	0.911908025	1	0.14873706
ENSMUSG00000023348	Trip6	22051	protein_coding	115.055607	0.148747681	0.136547006	-1.089351466	0.275998925	0.49283969	0.148747681
ENSMUSG00000022971	Ifnar2	15976	protein_coding	35.12856088	-0.148803814	0.237981955	0.625273518	0.531791557	0.728736684	0.148803814
ENSMUSG00000111080	Gm20300	100504586	lncRNA	172.1510412	-0.148813043	0.122641417	1.213399576	0.224977089	0.432209358	0.148813043
ENSMUSG00000063568	Jazf1	231986	protein_coding	11.20033409	0.148829377	0.340643807	-0.43690616	0.662179405	0.817281815	0.148829377
ENSMUSG00000032601	Prkar2a	19087	protein_coding	1726.122523	0.148832258	0.080090756	-1.858295079	0.063127122	0.176991731	0.148832258
ENSMUSG00000066952	Myo1h	231646	protein_coding	10.88546173	0.148859218	0.395443174	-0.376436434	0.706592464	0.846208704	0.148859218
ENSMUSG00000020142	Slc1a4	55963	protein_coding	545.5931425	0.148875054	0.113609599	-1.310409122	0.190057469	0.387358014	0.148875054
ENSMUSG00000032417	Rwdd2a	69519	protein_coding	5.648116711	-0.148916697	0.454398979	0.32772234	0.74312161	0.870239999	0.148916697
ENSMUSG00000021275	Tecpr2	104859	protein_coding	264.1933498	-0.148921648	0.076303881	1.951691662	0.050974823	0.151293715	0.148921648
ENSMUSG00000025350	Rdh5	19682	protein_coding	33.56962929	-0.148934597	0.200445136	0.743019261	0.457470019	0.672242383	0.148934597
ENSMUSG00000008450	Nutf2	68051	protein_coding	109.9091208	-0.148939903	0.158934715	0.937113726	0.348700062	0.570573694	0.148939903
ENSMUSG00000079469	Pigb	55981	protein_coding	120.9704376	0.149014322	0.129476714	-1.150896689	0.24977474	0.462014445	0.149014322
ENSMUSG00000020116	Pno1	66249	protein_coding	289.4476729	0.149037793	0.115992711	-1.284889297	0.198830972	0.399403317	0.149037793
ENSMUSG00000029338	Antxr2	71914	protein_coding	529.3485051	-0.149124561	0.078786687	1.892763459	0.058389333	0.167403029	0.149124561
ENSMUSG00000023030	Slc11a2	18174	protein_coding	441.9655707	0.149263784	0.112443058	-1.327461085	0.184356205	0.379371759	0.149263784
ENSMUSG00000034748	Sirt6	50721	protein_coding	171.7348456	0.149288964	0.087989154	-1.696674613	0.089758195	0.228322063	0.149288964
ENSMUSG00000045362	Tnfrsf26	244237	protein_coding	2.036740981	-0.149297217	1.109195574	0.134599543	0.892928503	1	0.149297217
ENSMUSG00000045071	E130308A19Rik	230259	protein_coding	100.9316689	0.149300633	0.102925219	-1.450573874	0.146898555	0.324750031	0.149300633
ENSMUSG00000079426	Arpc4	68089	protein_coding	1155.549884	0.14931355	0.061219603	-2.438982662	0.014728675	0.057501025	0.14931355
ENSMUSG00000040565	Btaf1	107182	protein_coding	812.3548347	0.149398896	0.072468411	-2.061572671	0.039248438	0.124214011	0.149398896
ENSMUSG00000023791	Pigx	72084	protein_coding	251.8388068	-0.149408463	0.07438824	2.008495743	0.044590638	0.136567098	0.149408463
ENSMUSG00000026283	Ing5	66262	protein_coding	298.4357207	-0.149433585	0.101716592	1.469117099	0.141801031	0.316830918	0.149433585
ENSMUSG00000025982	Sf3b1	81898	protein_coding	3699.483714	0.149452312	0.037429668	-3.992883676	6.53E-05	0.000592929	0.149452312
ENSMUSG00000015291	Gdi1	14567	protein_coding	661.8729403	-0.149454096	0.061657846	2.423926628	0.015353707	0.059503296	0.149454096
ENSMUSG00000031970	Dbndd1	72185	protein_coding	12.80893886	0.149487867	0.360116395	-0.415109863	0.67806148	0.827422396	0.149487867
ENSMUSG00000024410	Rmc1	76482	protein_coding	439.8526301	-0.149489104	0.059678921	2.504889509	0.012248965	0.049834789	0.149489104

Supplementary Table 1

ensembl_gene_id	external_gene_name	entrezgene_id	gene_biotype	baseMean	log2FoldChange	lfcSE	stat	pvalue	padj	absLog2FC
ENSMUSG00000025880	Smad7	17131	protein_coding	901.5879665	-0.149518484	0.182633915	0.818678631	0.412969792	0.63310278	0.149518484
ENSMUSG00000107283	Mpv17	17527	protein_coding	137.1643973	-0.14953302	0.099041499	1.509801658	0.131094042	0.299596873	0.14953302
ENSMUSG00000020849	Ywhae	22627	protein_coding	1785.667618	0.149572391	0.067452273	-2.217455159	0.026592004	0.091602076	0.149572391
ENSMUSG00000115667	Gm10389	100038608	lncRNA	9.42329016	-0.149668163	0.599490516	0.249658934	0.802851119	0.902682351	0.149668163
ENSMUSG00000032443	Zcwpw2	100039681	protein_coding	2.760267322	-0.149694484	1.119209881	0.133750145	0.893600151	1	0.149694484
ENSMUSG00000033508	Asprv1	67855	protein_coding	6.408574903	0.149764411	0.64481047	-0.232261134	0.816335197	0.908409285	0.149764411
ENSMUSG00000003348	Mob3a	208228	protein_coding	364.9557869	0.149782392	0.059078729	-2.535301554	0.011235056	0.046746214	0.149782392
ENSMUSG00000022179	4931414P19Rik	74359	protein_coding	98.4914423	-0.149794724	0.137793693	1.087094197	0.276995182	0.493878043	0.149794724
ENSMUSG00000013418	B4galnt2	14422	protein_coding	4895.674482	-0.149824261	0.114434488	1.309257939	0.190446992	0.387893639	0.149824261
ENSMUSG00000087625	4930419G24Rik	73874	lncRNA	0.986827362	0.14983293	1.294788939	-0.115719964	0.907874484	1	0.14983293
ENSMUSG00000014402	Tsg101	22088	protein_coding	524.5910612	-0.149878845	0.0725885	2.064773966	0.038944392	0.123393648	0.149878845
ENSMUSG00000020917	Acly	104112	protein_coding	5124.893006	-0.149914517	0.179483646	0.835254465	0.40357445	0.62490656	0.149914517
ENSMUSG00000021451	Sema4d	20354	protein_coding	101.7713482	0.149950164	0.103595113	-1.447463679	0.147767102	0.326059049	0.149950164
ENSMUSG00000074480	Mex3a	72640	protein_coding	34.62614591	0.149998997	0.204859319	-0.732204898	0.464043515	0.677560974	0.149998997
ENSMUSG00000041827	Oasl1	231655	protein_coding	233.3759016	-0.150077717	0.293043338	0.512134889	0.608556608	0.782687214	0.150077717
ENSMUSG00000026872	Zeb2	24136	protein_coding	7.378744722	0.150087513	1.256374754	-0.119460784	0.904910308	0.953204491	0.150087513
ENSMUSG00000022051	Bnip3l	12177	protein_coding	617.3414146	-0.150125895	0.094085174	1.595638168	0.110569601	0.265929452	0.150125895
ENSMUSG00000030782	Tgfb1i1	21804	protein_coding	4.714040252	-0.150128869	0.99286946	0.151207057	0.879812384	0.940676072	0.150128869
ENSMUSG00000074892	B3galt5	93961	protein_coding	7152.506842	0.150210324	0.141504478	-1.061523472	0.288452072	0.50724071	0.150210324
ENSMUSG00000020639	Pfn4	382562	protein_coding	1.761904997	0.150221459	0.976113311	-0.153897562	0.877690506	1	0.150221459
ENSMUSG00000065778	Gm22154	115490050	snoRNA	292.5540673	0.150253357	0.209449086	-0.717374134	0.473143276	0.685335967	0.150253357
ENSMUSG00000038677	Scube3	268935	protein_coding	8.172535952	0.150277433	0.773375841	-0.194313586	0.845930349	0.924166034	0.150277433
ENSMUSG00000038528	Mfsd4b5	215928	protein_coding	11.38620434	0.150292162	0.504189989	-0.298086367	0.765637247	0.882510888	0.150292162
ENSMUSG00000026187	Xrcc5	22596	protein_coding	289.7646833	-0.150302657	0.116868923	1.286078911	0.198415523	0.398762803	0.150302657
ENSMUSG00000029185	Fam114a1	68303	protein_coding	1048.498491	0.150315233	0.092294513	-1.628647559	0.10338765	0.253653789	0.150315233
ENSMUSG00000028261	Ndufaf4	68493	protein_coding	353.1687524	0.150352954	0.095572163	-1.57318773	0.115675356	0.274502489	0.150352954
ENSMUSG00000039865	Slc44a3	213603	protein_coding	951.0497586	-0.150355741	0.130174916	1.15502852	0.248078747	0.460363236	0.150355741
ENSMUSG00000020107	Anapc16	52717	protein_coding	431.051953	0.150363189	0.066814528	-2.250456503	0.024419982	0.085802463	0.150363189
ENSMUSG00000024782	Ak3	56248	protein_coding	2644.987741	-0.150388703	0.106373399	1.41378112	0.157426179	0.340684159	0.150388703
ENSMUSG00000033813	Tcea1	21399	protein_coding	514.9973281	0.150453218	0.094057524	-1.599587275	0.109690173	0.264518622	0.150453218
ENSMUSG00000035476	Tab3	66724	protein_coding	162.2963182	0.15048345	0.095525319	-1.575325287	0.11518138	0.273724003	0.15048345
ENSMUSG00000029102	Hgfac	54426	protein_coding	2123.576893	-0.150526403	0.172821428	0.870993861	0.383757505	0.606204104	0.150526403
ENSMUSG00000004105	Angptl2	26360	protein_coding	16.92717173	0.150528804	0.435061328	-0.345994448	0.729346887	0.86030736	0.150528804
ENSMUSG00000047821	Trim16	94092	protein_coding	1508.441922	-0.150585904	0.095308388	1.579985843	0.114110109	0.271607716	0.150585904
ENSMUSG00000052302	Tbc1d30	74694	protein_coding	275.7739104	-0.150668286	0.082728752	1.821232424	0.068571534	0.188104702	0.150668286
ENSMUSG00000029775	Klhdc10	76788	protein_coding	692.962787	0.150762931	0.068324036	-2.206587006	0.027342926	0.093703879	0.150762931
ENSMUSG00000055818	A230083G16Rik	442825	lncRNA	15.22140655	0.150766756	0.319112256	-0.472456803	0.636600768	0.801948109	0.150766756
ENSMUSG00000022800	Fyttd1	69823	protein_coding	496.6851887	0.150790318	0.115501383	-1.305528251	0.191713034	0.389463531	0.150790318
ENSMUSG00000068749	Psma5	26442	protein_coding	585.155524	-0.150823881	0.148111196	1.018315193	0.30852819	0.529189296	0.150823881

ensembl_gene_id	external_gene_name	entrezgene_id	gene_biotype	baseMean	log2FoldChange	lfcSE	stat	pvalue	padj	absLog2FC
ENSMUSG00000078202	Nrarp	67122	protein_coding	79.01685135	0.150862568	0.158102738	-0.954205915	0.339979421	0.561491337	0.150862568
ENSMUSG00000050965	Prkca	18750	protein_coding	1625.190949	-0.150887234	0.064611772	2.335290127	0.01952827	0.07201649	0.150887234
ENSMUSG00000046836	Brox	71678	protein_coding	628.4200648	-0.150893215	0.09309345	1.620878974	0.105043597	0.256383382	0.150893215
ENSMUSG00000057375	Yipf1	230584	protein_coding	1238.380621	-0.150906221	0.116472933	1.295633386	0.195101821	0.394262295	0.150906221
ENSMUSG00000021987	Mtmr6	219135	protein_coding	1455.636617	-0.150946486	0.075694892	1.994143631	0.04613636	0.140312312	0.150946486
ENSMUSG00000024168	Tmem204	407831	protein_coding	5.094998447	-0.151014151	0.479631756	0.314854363	0.752872233	0.876175621	0.151014151
ENSMUSG00000026181	Ppm1f	68606	protein_coding	84.02915785	0.151021222	0.149167563	-1.012426687	0.311334084	0.532288131	0.151021222
ENSMUSG00000063714	Sp3os	100503849	lncRNA	24.37508724	0.151040136	0.210316034	-0.71815797	0.47265989	0.685174282	0.151040136
ENSMUSG00000061436	Hipk2	15258	protein_coding	445.6101467	-0.151172858	0.210962009	0.716588065	0.473628311	0.685675281	0.151172858
ENSMUSG00000025474	Tubgcp2	74237	protein_coding	199.3752751	0.151210126	0.105130931	-1.438302926	0.150348122	0.330166076	0.151210126
ENSMUSG00000040749	Siah1b	20438	protein_coding	55.34559573	0.151219682	0.195815858	-0.772254519	0.439963697	0.657874003	0.151219682
ENSMUSG00000002948	Map2k7	26400	protein_coding	213.9767761	-0.15122196	0.075002573	2.016223641	0.043776595	0.134723574	0.15122196
ENSMUSG00000032078	Zpr1	22687	protein_coding	323.6301268	-0.151262874	0.109213036	1.385025813	0.166044575	0.353403803	0.151262874
ENSMUSG00000002043	Trappc6a	67091	protein_coding	834.0377947	0.151315055	0.088531839	-1.709159733	0.087421365	0.224312683	0.151315055
ENSMUSG00000059146	Ntrk3	18213	protein_coding	10.71417191	0.151337418	2.00608059	-0.075439351	0.939865151	0.971381329	0.151337418
ENSMUSG00000023944	Hsp90ab1	15516	protein_coding	10969.34108	-0.151384748	0.109212236	1.386151896	0.165700527	0.352762472	0.151384748
ENSMUSG00000019647	Sema6a	20358	protein_coding	239.4680644	-0.15142593	0.217458411	0.69634432	0.486213224	0.695624496	0.15142593
ENSMUSG00000022723	Crybg3	224273	protein_coding	346.7037727	-0.151427048	0.074182059	2.041289365	0.041222074	0.128879463	0.151427048
ENSMUSG00000028413	B4galt1	14595	protein_coding	1284.480197	-0.151459495	0.13137012	1.152921957	0.248942419	0.46108514	0.151459495
ENSMUSG00000025159	Mms19	72199	protein_coding	325.073273	-0.151462642	0.077176827	1.962540398	0.049699602	0.148254918	0.151462642
ENSMUSG00000029062	Cdk11b	12537	protein_coding	566.8403893	0.151512323	0.097051534	-1.561153309	0.118487583	0.279437874	0.151512323
ENSMUSG00000021432	Slc35b3	108652	protein_coding	587.4095805	0.151524198	0.060764947	-2.493611945	0.01264507	0.051106386	0.151524198
ENSMUSG00000003421	Nosip	66394	protein_coding	387.375426	-0.151538811	0.06495597	2.332946618	0.019650947	0.072388075	0.151538811
ENSMUSG00000014444	Piezo1	234839	protein_coding	237.2644011	-0.151574627	0.102124742	1.484210624	0.137753052	0.310179152	0.151574627
ENSMUSG00000022766	Serpind1	15160	protein_coding	2.309397462	0.151582558	0.88112562	-0.172032857	0.863411698	1	0.151582558
ENSMUSG00000054200	Ffar4	107221	protein_coding	283.180685	0.151641968	0.23734619	-0.638906264	0.522883913	0.722022907	0.151641968
ENSMUSG00000004038	Gstm3	14864	protein_coding	578.5111532	-0.151696725	0.139435986	1.087930953	0.27662559	0.493582047	0.151696725
ENSMUSG00000045284	Dcaf12l1	245404	protein_coding	11.72648822	-0.151706967	0.310502524	0.488585295	0.625135326	0.794593769	0.151706967
ENSMUSG00000004043	Stat5a	20850	protein_coding	143.2243409	-0.151716931	0.097690182	1.553041741	0.120413163	0.282675077	0.151716931
ENSMUSG00000006763	Saal1	78935	protein_coding	149.2256565	0.151766876	0.121586084	-1.248225715	0.211948409	0.416333535	0.151766876
ENSMUSG00000038764	Ptpn3	545622	protein_coding	1230.480631	-0.151782832	0.087757164	1.729577678	0.083705757	0.216936107	0.151782832
ENSMUSG00000025374	Nabp2	69917	protein_coding	319.2694171	0.151787312	0.088353256	-1.717959456	0.085804011	0.22115998	0.151787312
ENSMUSG00000024228	Nudt12	67993	protein_coding	195.5921657	-0.151828367	0.102799143	1.476941959	0.139691196	0.313346973	0.151828367
ENSMUSG00000034007	Scaper	244891	protein_coding	276.4869789	-0.151873941	0.097714758	1.554257962	0.120122897	0.28232699	0.151873941
ENSMUSG00000040985	Sun3	194974	protein_coding	4.789634065	0.151915543	0.619112263	-0.245376408	0.806164994	0.904600714	0.151915543
ENSMUSG00000009569	Mrtfb	239719	protein_coding	694.243977	-0.151917429	0.091161508	1.666464626	0.095620908	0.239182623	0.151917429
ENSMUSG00000070699	Sars2	71984	protein_coding	162.9186859	0.151932978	0.126494548	-1.201102975	0.229711259	0.438295983	0.151932978
ENSMUSG00000028389	Zfp37	22696	protein_coding	95.9516319	-0.151933846	0.129826607	1.170282805	0.241887179	0.452586095	0.151933846
ENSMUSG00000025171	Ubtd1	226122	protein_coding	58.20730642	-0.151936894	0.158130419	0.960832805	0.336636241	0.55922049	0.151936894

Supplementary Table 1

ensembl_gene_id	external_gene_name	entrezgene_id	gene_biotype	baseMean	log2FoldChange	lfcSE	stat	pvalue	padj	absLog2FC
ENSMUSG00000002580	Mien1	103742	protein_coding	481.9702706	0.151942232	0.088927503	-1.708607885	0.087523607	0.224470391	0.151942232
ENSMUSG00000028229	Rmdn1	66302	protein_coding	112.418735	-0.151956063	0.108050921	1.406337514	0.159623926	0.343804463	0.151956063
ENSMUSG00000018428	Akap1	11640	protein_coding	1449.432005	-0.151956125	0.091173032	1.666678426	0.095578365	0.239112425	0.151956125
ENSMUSG00000027255	Arfgap2	77038	protein_coding	406.2449256	0.151990283	0.06217943	-2.444382044	0.014510052	0.056795212	0.151990283
ENSMUSG00000024085	Man2a1	17158	protein_coding	1649.70066	0.151994039	0.091327068	-1.664282475	0.096055993	0.239895137	0.151994039
ENSMUSG00000035133	Arhgap5	11855	protein_coding	3151.211937	-0.152000852	0.086976771	1.747602841	0.080532823	0.210915627	0.152000852
ENSMUSG00000029673	Auts2	319974	protein_coding	388.0644268	-0.152047087	0.147311486	1.03214685	0.302003329	0.522337974	0.152047087
ENSMUSG00000044876	Zfp444	72667	protein_coding	215.3105253	0.152064424	0.086200158	-1.764085207	0.077717635	0.205842665	0.152064424
ENSMUSG00000091530	Cldn20	621628	processed_pseudogene	2.439288402	0.152084204	0.672847236	-0.226030807	0.82117746	1	0.152084204
ENSMUSG00000003948	Mmd	67468	protein_coding	85.18995449	-0.152095721	0.160998704	0.944701527	0.344811277	0.566362983	0.152095721
ENSMUSG00000013822	Elof1	66126	protein_coding	671.9700388	0.152101069	0.07572672	-2.008552185	0.044584646	0.136567098	0.152101069
ENSMUSG00000024998	Plce1	74055	protein_coding	1575.495755	-0.152109621	0.082795517	1.837172177	0.066184482	0.183322799	0.152109621
ENSMUSG00000079104	Prps1l3	328099	protein_coding	289.1564668	0.15212059	0.13315461	-1.142435776	0.253272927	0.465710848	0.15212059
ENSMUSG00000022707	Gbe1	74185	protein_coding	295.3048401	-0.1521273	0.107227277	1.418736947	0.155975721	0.338606949	0.1521273
ENSMUSG00000043003	Rasef	242505	protein_coding	1739.600568	0.152181014	0.08458226	-1.799207224	0.071985907	0.195041062	0.152181014
ENSMUSG00000015536	Mocs2	17434	protein_coding	364.3859448	-0.152211721	0.077142045	1.973135674	0.048480107	0.145669666	0.152211721
ENSMUSG00000001158	Snrnp27	66618	protein_coding	250.8513462	0.152219365	0.083602918	-1.820742238	0.068646048	0.188215315	0.152219365
ENSMUSG00000033047	Eif3l	223691	protein_coding	1842.775235	-0.152230701	0.111599742	1.364077538	0.172543164	0.363163115	0.152230701
ENSMUSG00000027615	Hps3	12807	protein_coding	141.0155408	-0.152320069	0.125338812	1.215266575	0.224264436	0.431320242	0.152320069
ENSMUSG00000029106	Add1	11518	protein_coding	3152.639566	-0.152387581	0.061764464	2.467237167	0.013616014	0.054116692	0.152387581
ENSMUSG00000005251	Ripk4	72388	protein_coding	644.7540681	-0.152418298	0.103493324	1.472735546	0.140822358	0.315242025	0.152418298
ENSMUSG00000026111	Unc50	67387	protein_coding	494.8076963	0.152526495	0.090173244	-1.691482845	0.090744622	0.230228404	0.152526495
ENSMUSG00000044345	Marveld1	277010	protein_coding	54.87199256	-0.152629213	0.184700059	0.826362555	0.408598458	0.629263727	0.152629213
ENSMUSG00000020844	Nxn	18230	protein_coding	30.78198882	0.152635009	0.178772158	-0.85379631	0.393217859	0.615915748	0.152635009
ENSMUSG00000002428	Hltf	20585	protein_coding	566.4980161	0.152642701	0.100921637	-1.512487372	0.130409923	0.29840508	0.152642701
ENSMUSG00000022203	Efs	13644	protein_coding	4.591170666	-0.152643317	0.652434631	0.233959557	0.815016377	0.908068261	0.152643317
ENSMUSG00000047187	Rab2a	59021	protein_coding	1956.898274	0.152695905	0.103610312	-1.473752006	0.140548375	0.314808425	0.152695905
ENSMUSG00000068206	Pick1	18693	protein_coding	201.0547693	0.152727987	0.087901661	-1.73748692	0.082301261	0.214305112	0.152727987
ENSMUSG00000031879	Ciao2b	68523	protein_coding	281.1495088	-0.152738017	0.105089789	1.453404925	0.14611136	0.323442761	0.152738017
ENSMUSG00000026915	Strbp	20744	protein_coding	1857.814973	-0.152770843	0.061544404	2.482286497	0.013054227	0.05235005	0.152770843
ENSMUSG00000041488	Stx3	20908	protein_coding	700.6841598	-0.15280746	0.07706032	1.982959059	0.047372003	0.143173729	0.15280746
ENSMUSG00000040488	Ltbp4	108075	protein_coding	16.44956176	0.152813908	0.461826843	-0.330890052	0.740727536	0.868781842	0.152813908
ENSMUSG00000013766	Ly6g6e	70274	protein_coding	107.674528	-0.152819857	0.216620943	0.705471294	0.48051699	0.691465456	0.152819857
ENSMUSG00000070003	Ssbp4	76900	protein_coding	444.0025175	0.152833773	0.136303994	-1.121271422	0.262172346	0.47603234	0.152833773
ENSMUSG00000047368	Abhd17b	226016	protein_coding	695.9202747	0.152863935	0.092753189	-1.648072006	0.099337896	0.246352764	0.152863935
ENSMUSG00000056870	Gulp1	70676	protein_coding	64.70572429	0.15288093	0.184108929	-0.830383029	0.406322257	0.62720573	0.15288093
ENSMUSG00000031425	Plp1	18823	protein_coding	6.920297362	-0.15304047	0.570889455	0.268073737	0.788642561	0.894265208	0.15304047
ENSMUSG00000043290	Zfp784	654801	protein_coding	5.786559283	0.15307731	0.429476599	-0.356427591	0.72152037	0.855725733	0.15307731
ENSMUSG00000085826	Gm15638	100503378	lncRNA	14.09439051	-0.153081281	0.35860518	0.426879725	0.669466933	0.822609935	0.153081281

ensembl_gene_id	external_gene_name	entrezgene_id	gene_biotype	baseMean	log2FoldChange	lfcSE	stat	pvalue	padj	absLog2FC
ENSMUSG00000065715	Snord7	100302731	snoRNA	1.233024909	0.153174558	1.175925136	-0.130258767	0.896361701	1	0.153174558
ENSMUSG00000038611	Phrf1	101471	protein_coding	848.9383617	0.153264582	0.092542602	-1.656151632	0.097691116	0.243108963	0.153264582
ENSMUSG00000054280	Prr14l	215476	protein_coding	950.7701099	-0.153287546	0.065302526	2.347344814	0.018907748	0.07028111	0.153287546
ENSMUSG00000062352	Itgb1bp1	16413	protein_coding	86.64239956	0.153297333	0.108303269	-1.415445116	0.15693803	0.340070151	0.153297333
ENSMUSG00000031256	Cstf2	108062	protein_coding	795.8820411	0.153305313	0.083998989	-1.825085219	0.06798817	0.186963018	0.153305313
ENSMUSG00000002897	Il17ra	16172	protein_coding	266.9436012	-0.153343982	0.066479575	2.306633018	0.021075287	0.076491713	0.153343982
ENSMUSG00000006575	Rundc3a	51799	protein_coding	87.71933739	-0.153369473	0.110175728	1.392044116	0.163909039	0.350167383	0.153369473
ENSMUSG00000042102	Dmgdh	74129	protein_coding	10.22489634	0.153378031	0.362138719	-0.423533921	0.671905755	0.82411257	0.153378031
ENSMUSG00000032459	Mrps22	64655	protein_coding	253.8975596	-0.153424741	0.088865164	1.726489147	0.08425945	0.218131456	0.153424741
ENSMUSG00000002949	Timm44	21856	protein_coding	710.2846818	-0.153425596	0.060112905	2.552290482	0.010701725	0.044980477	0.153425596
ENSMUSG00000058230	Arhgap35	232906	protein_coding	987.2526262	-0.153438831	0.099241527	1.546115179	0.122076753	0.285167413	0.153438831
ENSMUSG00000088625	Gm23318	115489368	snRNA	0.753951854	0.153466152	1.440795007	-0.106514911	0.915173825	1	0.153466152
ENSMUSG00000026021	Sumo1	22218	protein_coding	542.0052041	-0.153510933	0.104947161	1.462744977	0.143537174	0.319845216	0.153510933
ENSMUSG00000026922	Agpat2	67512	protein_coding	1089.124154	-0.153541647	0.10226455	1.501416147	0.13324796	0.302968031	0.153541647
ENSMUSG00000025086	Trub1	72133	protein_coding	99.1890106	-0.153545974	0.134472868	1.141836091	0.253522157	0.465961592	0.153545974
ENSMUSG00000091455	Otogl	628870	protein_coding	2.768052669	0.153650272	2.025336005	-0.075864089	0.939527227	1	0.153650272
ENSMUSG00000062906	Hdac10	170787	protein_coding	207.3683616	-0.153659663	0.071787142	2.140490051	0.032315184	0.10685821	0.153659663
ENSMUSG00000032431	Crtap	56693	protein_coding	85.02002527	-0.15368164	0.12123006	1.267685913	0.204910138	0.407353559	0.15368164
ENSMUSG00000035735	Dagla	269060	protein_coding	175.7919717	-0.153711871	0.128168068	1.199299267	0.2304116	0.439074217	0.153711871
ENSMUSG00000028689	Ccdc163	68394	protein_coding	67.37163982	-0.153792249	0.131213296	1.172078236	0.241165647	0.451799148	0.153792249
ENSMUSG00000020032	Nuak1	77976	protein_coding	13.17647467	0.153832017	0.376198608	-0.408911711	0.682604454	0.830450122	0.153832017
ENSMUSG00000033855	Ston1	77057	protein_coding	57.27315809	-0.15384153	0.205089367	0.750119486	0.453182745	0.669122175	0.15384153
ENSMUSG00000015702	Anxa9	71790	protein_coding	22.18715301	0.153871454	0.328141171	-0.468918463	0.639127919	0.803282676	0.153871454
ENSMUSG00000040591	Cstpp1	228356	protein_coding	41.36288346	0.153939345	0.16926879	-0.909437264	0.363119359	0.585429376	0.153939345
ENSMUSG00000027806	Tsc22d2	72033	protein_coding	406.90775	0.153979681	0.147100991	-1.04676168	0.295209508	0.514332163	0.153979681
ENSMUSG00000032346	Ooep	67968	protein_coding	1.165099727	-0.154027658	0.999164295	0.154156488	0.87748635	1	0.154027658
ENSMUSG00000060548	Tnfrsf19	29820	protein_coding	33.41747318	0.154040064	0.251462382	-0.61257697	0.54015609	0.734530441	0.154040064
ENSMUSG00000059363	Fxn	14297	protein_coding	75.09941831	0.154062457	0.122623233	-1.256388806	0.208975038	0.412258246	0.154062457
ENSMUSG00000031503	Col4a2	12827	protein_coding	16.77000391	-0.154158132	0.579907342	0.265832351	0.790368326	0.895392916	0.154158132
ENSMUSG00000023473	Celsr3	107934	protein_coding	17.8054604	-0.154172415	0.543643359	0.283591093	0.776723763	0.888506311	0.154172415
ENSMUSG00000003309	Ap1m2	11768	protein_coding	1791.21643	0.154194122	0.081692783	-1.887487684	0.059094761	0.168985435	0.154194122
ENSMUSG00000025584	Pde8a	18584	protein_coding	93.91882526	-0.154196457	0.163905047	0.940766983	0.346824284	0.568538439	0.154196457
ENSMUSG00000055415	Atp10b	319767	protein_coding	3229.405372	0.154197449	0.076909475	-2.004921366	0.044971454	0.137605817	0.154197449
ENSMUSG00000001127	Araf	11836	protein_coding	904.9185017	0.154226666	0.067361428	-2.289539745	0.022048011	0.079188614	0.154226666
ENSMUSG00000079316	Rab9	56382	protein_coding	298.7499233	-0.154255467	0.078550484	1.963774884	0.049556204	0.14798755	0.154255467
ENSMUSG00000022948	Setd4	224440	protein_coding	108.4114842	-0.154258443	0.143306749	1.07642134	0.281738863	0.499562412	0.154258443
ENSMUSG00000051735	Rinl	320435	protein_coding	21.55371536	0.154276229	0.338549176	-0.455698139	0.648607061	0.809117465	0.154276229
ENSMUSG00000091586	Cyp4f17	208285	protein_coding	37.3747132	-0.154294215	0.186045109	0.829337657	0.406913368	0.627713038	0.154294215
ENSMUSG00000029502	Golga3	269682	protein_coding	1300.471166	-0.15429926	0.099568277	1.549682938	0.121217635	0.283960728	0.15429926

Supplementary Table 1

ensembl_gene_id	external_gene_name	entrezgene_id	gene_biotype	baseMean	log2FoldChange	lfcSE	stat	pvalue	padj	absLog2FC
ENSMUSG00000085498	Gm14023	100503468	lncRNA	5.630308155	-0.154339955	0.526683081	0.293041415	0.769490496	0.884482916	0.154339955
ENSMUSG00000009555	Cdk9	107951	protein_coding	428.490346	-0.15434096	0.064480013	2.393624825	0.016682804	0.063640522	0.15434096
ENSMUSG00000042419	Nfkbil1	18038	protein_coding	166.0682744	0.15434413	0.09213839	-1.675133773	0.093907988	0.235970255	0.15434413
ENSMUSG00000025453	Nnt	18115	protein_coding	8.919812186	-0.15437655	0.385834805	0.400110481	0.689075145	0.833960201	0.15437655
ENSMUSG00000075296	Aldh3b2	621603	protein_coding	94.73288813	0.154437771	0.125422208	-1.231343107	0.218194566	0.42426293	0.154437771
ENSMUSG00000020911	Krt19	16669	protein_coding	39271.83275	-0.154497562	0.11614446	1.330218953	0.183446141	0.378300667	0.154497562
ENSMUSG00000021716	Srek1ip1	67288	protein_coding	276.9479802	-0.154500631	0.094695688	1.631548739	0.102774584	0.25251889	0.154500631
ENSMUSG00000006154	Eps8l1	67425	protein_coding	66.3002116	0.15453759	0.227227101	-0.680101929	0.496439923	0.702502904	0.15453759
ENSMUSG00000042496	Prdm10	382066	protein_coding	245.7047692	0.15455135	0.079635148	-1.940742927	0.052289467	0.154446119	0.15455135
ENSMUSG00000067424	Zfp563	240068	protein_coding	97.85510103	-0.154586599	0.119609142	1.292431296	0.196207824	0.395770858	0.154586599
ENSMUSG00000027799	Nbea	26422	protein_coding	229.4425089	0.154617537	0.154150998	-1.003026502	0.315848074	0.536652594	0.154617537
ENSMUSG00000073448	Gm10509	100310809	protein_coding	25.46150153	-0.154620429	0.219652548	0.703931872	0.481475202	0.692354022	0.154620429
ENSMUSG00000061079	Zfp143	20841	protein_coding	233.345022	0.154727552	0.088459486	-1.74913466	0.080267745	0.21050366	0.154727552
ENSMUSG00000023143	Nagpa	27426	protein_coding	210.1540503	-0.154773928	0.105485575	1.467252071	0.142307497	0.31779121	0.154773928
ENSMUSG00000048763	Hoxb3	15410	protein_coding	45.84458906	-0.154816658	0.39893854	0.38807145	0.697963159	0.839976943	0.154816658
ENSMUSG00000099881	2810013P06Rik	100503178	lncRNA	159.4390802	-0.154842553	0.129250337	1.19800503	0.230915059	0.439526427	0.154842553
ENSMUSG00000003955	Fam162a	70186	protein_coding	1099.613046	-0.154857898	0.121701793	1.272437282	0.203217795	0.405453505	0.154857898
ENSMUSG00000002504	Slc9a3r2	65962	protein_coding	111.7672352	-0.154919656	0.143681027	1.078219294	0.280935904	0.498589523	0.154919656
ENSMUSG00000060152	Pop5	117109	protein_coding	223.7617528	-0.154920333	0.102210292	1.515701893	0.129594749	0.297320449	0.154920333
ENSMUSG00000025024	Smndc1	76479	protein_coding	269.6054969	-0.154920355	0.106586852	1.453465899	0.146094441	0.323442761	0.154920355
ENSMUSG00000088242	Gm25913	115485866	scaRNA	0.727869722	-0.154931699	1.556620252	0.099530826	0.920716814	1	0.154931699
ENSMUSG00000023039	Krt7	110310	protein_coding	2104.849289	0.15499578	0.088774204	-1.745955173	0.080818741	0.211344528	0.15499578
ENSMUSG00000027498	Cstf1	67337	protein_coding	311.233901	0.155044453	0.11352519	-1.365727317	0.172024567	0.362441146	0.155044453
ENSMUSG00000015083	C8g	69379	protein_coding	24.06824754	-0.15508757	0.207457481	0.747563157	0.454723695	0.670174662	0.15508757
ENSMUSG00000057778	Cyb5d2	192986	protein_coding	98.09467096	-0.155109308	0.103838961	1.49374865	0.135241328	0.306361493	0.155109308
ENSMUSG00000033068	Entpd6	12497	protein_coding	777.6490143	0.155121424	0.078163828	-1.984567904	0.047192566	0.142788208	0.155121424
ENSMUSG00000021366	Hivep1	110521	protein_coding	683.6867437	-0.155195792	0.10502362	1.477722754	0.139482002	0.313005269	0.155195792
ENSMUSG00000060216	Arrb2	216869	protein_coding	35.11956412	0.15525835	0.229871072	-0.675414911	0.499412194	0.704201299	0.15525835
ENSMUSG00000030768	Disp1	68897	protein_coding	113.2067798	0.155263497	0.108423633	-1.432007883	0.152141565	0.332776929	0.155263497
ENSMUSG00000028345	Tex10	269536	protein_coding	179.7706571	-0.155329035	0.111914859	1.387921458	0.165160963	0.352113109	0.155329035
ENSMUSG00000025907	Rb1cc1	12421	protein_coding	994.8854898	-0.155329564	0.090320246	1.719764624	0.085475235	0.220621763	0.155329564
ENSMUSG00000040524	Zfp609	214812	protein_coding	287.5600983	-0.155368447	0.11975672	1.297367254	0.194504854	0.393392916	0.155368447
ENSMUSG00000034807	Colgalt1	234407	protein_coding	610.3715325	-0.155427521	0.069221678	2.245359049	0.024745092	0.086686561	0.155427521
ENSMUSG00000064120	Mocs1	56738	protein_coding	300.4099617	-0.155465815	0.088742832	1.751868982	0.079796339	0.209433685	0.155465815
ENSMUSG00000020159	Gabrp	216643	protein_coding	3.185763747	0.155480949	1.123853342	-0.138346298	0.889966737	1	0.155480949
ENSMUSG00000036931	Nfkbid	243910	protein_coding	54.12428243	0.155487116	0.272366735	-0.570874102	0.568084987	0.754128254	0.155487116
ENSMUSG00000026035	Ppil3	70225	protein_coding	111.8224253	0.155530895	0.123059077	-1.263871781	0.206276052	0.408887615	0.155530895
ENSMUSG00000074283	Zfp109	56869	protein_coding	60.41686287	-0.155604184	0.168839833	0.921608258	0.356732949	0.579660329	0.155604184
ENSMUSG00000041258	Zfp236	329002	protein_coding	582.9771293	0.155611926	0.0694226	-2.24151683	0.024992617	0.087325531	0.155611926

ensembl_gene_id	external_gene_name	entrezgene_id	gene_biotype	baseMean	log2FoldChange	lfcSE	stat	pvalue	padj	absLog2FC
ENSMUSG00000085427	6430710C18Rik	76218	lncRNA	5.413965063	0.155634789	0.465524612	-0.334321291	0.738137126	0.866982505	0.155634789
ENSMUSG00000050855	Zfp940	233057	protein_coding	21.99464052	0.15563629	0.21558927	-0.721911115	0.470349127	0.683706314	0.15563629
ENSMUSG00000027524	Edn3	13616	protein_coding	80.22262962	0.155649413	0.285524838	-0.545134406	0.585661081	0.767483504	0.155649413
ENSMUSG00000037465	Klf10	21847	protein_coding	1369.147879	0.155675479	0.127524655	-1.220748082	0.222181417	0.429285587	0.155675479
ENSMUSG00000024812	Tjp2	21873	protein_coding	2205.954975	0.155704573	0.063052907	-2.469427351	0.013532949	0.053877321	0.155704573
ENSMUSG00000029787	Avl9	78937	protein_coding	1317.433956	-0.155790786	0.092708193	1.68044248	0.092871257	0.233934706	0.155790786
ENSMUSG00000039330	Tsga10ip	78306	protein_coding	2.800449909	-0.155817219	1.181200122	0.131914327	0.895052057	1	0.155817219
ENSMUSG00000027570	Col9a3	12841	protein_coding	9.50997761	-0.155841883	0.524394339	0.297184527	0.766325635	0.882690208	0.155841883
ENSMUSG00000039634	Zfp189	230162	protein_coding	23.24689143	-0.155915278	0.23022212	0.677238477	0.498254659	0.703443399	0.155915278
ENSMUSG00000048534	Jaml	270152	protein_coding	17.69351534	0.155980962	0.296653513	-0.525801838	0.599025897	0.776435029	0.155980962
ENSMUSG00000007039	Ddah2	51793	protein_coding	15.70547817	-0.156019503	0.376865745	0.413992266	0.678879771	0.827931507	0.156019503
ENSMUSG00000038305	Spats2l	67198	protein_coding	141.0291859	-0.156145043	0.223608518	0.698296489	0.484991794	0.695300529	0.156145043
ENSMUSG00000046314	Stxbp6	217517	protein_coding	271.2728788	-0.1561621	0.121278417	1.287633069	0.197873722	0.39815848	0.1561621
ENSMUSG00000027163	Commd9	76501	protein_coding	275.6485141	0.156172208	0.087117247	-1.792666921	0.073026199	0.196922705	0.156172208
ENSMUSG00000013593	Ndufs2	226646	protein_coding	3334.428724	-0.156221235	0.078420824	1.992088687	0.046361328	0.14084087	0.156221235
ENSMUSG00000073002	Vamp5	53620	protein_coding	10.28557819	0.156239383	0.413684869	-0.377677295	0.705670336	0.845753813	0.156239383
ENSMUSG00000040195	Nemp1	72243	protein_coding	122.6003624	0.156241582	0.157741451	-0.990491598	0.321933894	0.543156551	0.156241582
ENSMUSG00000033701	Acbd6	72482	protein_coding	211.0248237	-0.15628415	0.131353633	1.189796938	0.234126213	0.443634979	0.15628415
ENSMUSG00000004895	Prcc	94315	protein_coding	533.6840781	-0.156295462	0.057828923	2.70272129	0.006877439	0.031443528	0.156295462
ENSMUSG00000060073	Psma3	19167	protein_coding	810.4499901	-0.156337342	0.111260673	1.405144666	0.159978264	0.344285818	0.156337342
ENSMUSG00000038722	Bud31	231889	protein_coding	335.7754621	0.156416099	0.079699281	-1.962578547	0.049695166	0.148254918	0.156416099
ENSMUSG00000036292	Gramd1c	207798	protein_coding	129.1398443	-0.156451806	0.114847303	1.362259296	0.173116069	0.363688435	0.156451806
ENSMUSG00000046679	C87436	232196	protein_coding	142.7843888	-0.156473941	0.118682469	1.318425058	0.187361397	0.383501006	0.156473941
ENSMUSG00000003435	Supt5	20924	protein_coding	1100.792404	0.156500161	0.05171416	-3.026253585	0.002476045	0.01350377	0.156500161
ENSMUSG00000030621	Me3	109264	protein_coding	19.70898627	-0.15654281	0.449724386	0.348086105	0.727775519	0.859322531	0.15654281
ENSMUSG00000073787	Gm10575	100126795	lncRNA	14.11834696	-0.15656965	0.390479095	0.400968073	0.68844363	0.83386713	0.15656965
ENSMUSG00000003534	Ddr1	12305	protein_coding	1570.915281	-0.156590423	0.091780474	1.706140926	0.087981844	0.22515016	0.156590423
ENSMUSG00000029471	Camkk2	207565	protein_coding	53.09128985	0.156638143	0.209392487	-0.748059996	0.45442397	0.669912287	0.156638143
ENSMUSG00000030421	Uri1	19777	protein_coding	398.7418389	0.15667653	0.105361645	-1.487035719	0.137005377	0.309296897	0.15667653
ENSMUSG00000031984	2810004N23Rik	66523	protein_coding	253.3436657	-0.156677062	0.108562518	1.443196649	0.148965083	0.328001251	0.156677062
ENSMUSG00000042670	Immp1l	66541	protein_coding	174.4201784	-0.156679447	0.13272415	1.180489357	0.237805641	0.448045905	0.156679447
ENSMUSG00000023909	Paqr4	76498	protein_coding	2166.79389	0.156730201	0.059036683	-2.654793483	0.007935698	0.035388872	0.156730201
ENSMUSG00000051339	2900026A02Rik	243219	protein_coding	3618.16354	-0.156778446	0.176221497	0.889666974	0.373644732	0.595844868	0.156778446
ENSMUSG00000032590	Apeh	235606	protein_coding	806.4781105	-0.15677922	0.076116887	2.059716667	0.039425635	0.12467923	0.15677922
ENSMUSG00000003033	Ap1m1	11767	protein_coding	132.3115318	0.156787116	0.115840479	-1.353474341	0.175904142	0.367893	0.156787116
ENSMUSG00000019832	Rab32	67844	protein_coding	178.8260189	-0.156803125	0.227369878	0.689638953	0.490421265	0.69793377	0.156803125
ENSMUSG00000040600	Eps8l3	99662	protein_coding	2039.571743	-0.156825862	0.123991511	1.264811284	0.205938985	0.408576247	0.156825862
ENSMUSG00000029029	Wrap73	59002	protein_coding	155.0830297	0.156841378	0.107584023	-1.457850089	0.144881869	0.321929383	0.156841378
ENSMUSG00000000627	Sema4f	20355	protein_coding	3.814325651	0.156850784	0.856214303	-0.183191035	0.854648129	1	0.156850784

Supplementary Table 1

ensembl_gene_id	external_gene_name	entrezgene_id	gene_biotype	baseMean	log2FoldChange	lfcSE	stat	pvalue	padj	absLog2FC
ENSMUSG00000021054	Sgpp1	81535	protein_coding	411.2425374	-0.156853183	0.08955951	1.751385018	0.079879612	0.209618927	0.156853183
ENSMUSG00000029022	Miip	28010	protein_coding	199.2010524	0.156938057	0.092270918	-1.700839886	0.088973057	0.226849015	0.156938057
ENSMUSG00000032977	Fam207a	108707	protein_coding	339.8313313	0.156954947	0.069611326	-2.254732907	0.024150099	0.085053259	0.156954947
ENSMUSG00000034432	Cops8	108679	protein_coding	334.0243595	0.156975723	0.072739184	-2.158062744	0.030922954	0.103308737	0.156975723
ENSMUSG00000025413	Ttc4	72354	protein_coding	536.1758669	0.156981598	0.061452142	-2.554534191	0.010632999	0.044782795	0.156981598
ENSMUSG00000090231	Cfb	14962	protein_coding	103.6827595	-0.157026186	0.136547157	1.14997771	0.250153052	0.462497481	0.157026186
ENSMUSG00000022201	Zfr	22763	protein_coding	961.7960191	0.157028384	0.056305086	-2.788884555	0.005288991	0.02553125	0.157028384
ENSMUSG00000035311	Gnptab	432486	protein_coding	536.944865	-0.157036493	0.128951576	1.217794286	0.223302156	0.430343308	0.157036493
ENSMUSG00000021018	Polr2h	245841	protein_coding	233.6282871	-0.15707163	0.086261518	1.820877179	0.068625529	0.1881903	0.15707163
ENSMUSG00000040818	Dennd6a	211922	protein_coding	555.5204888	-0.157111962	0.115461874	1.360725894	0.173600329	0.364391266	0.157111962
ENSMUSG00000037400	Atp11b	76295	protein_coding	1942.866814	0.15712495	0.075172463	-2.090192927	0.036600473	0.11738594	0.15712495
ENSMUSG00000042350	Arel1	68497	protein_coding	724.3174308	-0.15719216	0.064693179	2.429810408	0.015106723	0.058719535	0.15719216
ENSMUSG00000045268	Zfp691	195522	protein_coding	78.58897589	-0.157236975	0.122978398	1.278573943	0.201047131	0.402386658	0.157236975
ENSMUSG00000090534	Gm4675	105245869	lncRNA	9.848144355	-0.15724788	0.358975946	0.43804573	0.661353133	0.816921981	0.15724788
ENSMUSG00000086859	Snhg20	76972	lncRNA	35.5765524	-0.157261829	0.18790288	0.836931444	0.402631104	0.624030976	0.157261829
ENSMUSG00000032942	Ucp3	22229	protein_coding	1.496369095	0.157333059	1.074386859	-0.146439858	0.883574164	1	0.157333059
ENSMUSG00000031621	Isx	71597	protein_coding	1237.892139	-0.157380836	0.206052815	0.763788815	0.444993103	0.662372535	0.157380836
ENSMUSG00000098905	Zfp953	629016	protein_coding	97.28190839	-0.157395496	0.129057674	1.219574866	0.222626079	0.429692271	0.157395496
ENSMUSG00000024777	Ppp2r5b	225849	protein_coding	412.7646232	-0.157397483	0.101835097	1.545611362	0.122198456	0.285308256	0.157397483
ENSMUSG00000060639	H4c9	319158	protein_coding	160.1689433	0.15740548	0.165439514	-0.95143824	0.341381956	0.563251236	0.15740548
ENSMUSG00000074748	Atxn7l3b	382423	protein_coding	1244.986204	-0.157415013	0.105812464	1.487679305	0.136835488	0.309078852	0.157415013
ENSMUSG00000015869	Prpsap1	67763	protein_coding	1118.333419	-0.157454797	0.119610711	1.316393788	0.188041906	0.384369853	0.157454797
ENSMUSG00000039828	Wdr70	545085	protein_coding	122.2934343	0.157462231	0.212930882	-0.739499267	0.459603885	0.673998628	0.157462231
ENSMUSG00000041842	Fhdc1	229474	protein_coding	250.7260641	0.157509124	0.235071002	-0.670049146	0.502826461	0.707478587	0.157509124
ENSMUSG00000023074	Mospd1	70380	protein_coding	169.6334941	-0.157534991	0.120777789	1.304337433	0.192118558	0.389807821	0.157534991
ENSMUSG00000029165	Agbl5	231093	protein_coding	131.7318227	0.157544674	0.100235193	-1.571750093	0.11600852	0.274998498	0.157544674
ENSMUSG00000029499	Pxmp2	19301	protein_coding	42.65069727	-0.157556578	0.175119179	0.899710577	0.368274293	0.5907413	0.157556578
ENSMUSG00000036989	Trim3	55992	protein_coding	78.227704	-0.157559259	0.17529618	0.898817415	0.368749923	0.591274514	0.157559259
ENSMUSG00000026811	St6galnac6	50935	protein_coding	7907.948593	0.157564674	0.090131697	-1.748160526	0.080436235	0.210845076	0.157564674
ENSMUSG00000059890	Ube4a	140630	protein_coding	583.7229615	0.157655864	0.074719339	-2.109974012	0.034860594	0.11310174	0.157655864
ENSMUSG00000026975	Dph7	67228	protein_coding	83.50354579	0.157685076	0.134076557	-1.176082384	0.239561957	0.449849479	0.157685076
ENSMUSG00000042763	Maneal	215090	protein_coding	5.648226952	-0.157834368	0.533359036	0.295925179	0.767287222	0.883364555	0.157834368
ENSMUSG00000032251	Irak1bp1	65099	protein_coding	2.927674926	0.157897695	0.773946867	-0.204016196	0.838340843	1	0.157897695
ENSMUSG00000039254	Pomt1	99011	protein_coding	322.9467453	0.157918568	0.087102852	-1.813012591	0.069829879	0.190674731	0.157918568
ENSMUSG00000037697	Ddhd1	114874	protein_coding	202.3166707	0.158004082	0.076124451	-2.075602249	0.037930753	0.120901073	0.158004082
ENSMUSG00000021661	Ankra2	68558	protein_coding	200.4555883	-0.158010356	0.095196153	1.659839716	0.096946702	0.24162056	0.158010356
ENSMUSG00000024346	Pfdn1	67199	protein_coding	480.4818061	0.158037351	0.06790117	-2.327461366	0.019940723	0.07322684	0.158037351
ENSMUSG00000028790	Khdrbs1	20218	protein_coding	842.778024	0.158088623	0.072387137	-2.183932521	0.028967207	0.098226787	0.158088623
ENSMUSG00000026349	Ccnt2	72949	protein_coding	716.2202884	-0.15810535	0.057389477	2.754953665	0.005870045	0.027715628	0.15810535

ensembl_gene_id	external_gene_name	entrezgene_id	gene_biotype	baseMean	log2FoldChange	lfcSE	stat	pvalue	padj	absLog2FC
ENSMUSG00000068923	Syt11	229521	protein_coding	32.70692022	-0.158125923	0.196710953	0.803849105	0.421484126	0.641215003	0.158125923
ENSMUSG00000032245	Cln6	76524	protein_coding	245.9019106	-0.158146758	0.127306944	1.24224769	0.214145189	0.419152975	0.158146758
ENSMUSG00000029640	Usp12	22217	protein_coding	959.0244582	0.158209676	0.075250122	-2.10245075	0.03551381	0.114725166	0.158209676
ENSMUSG00000041319	Thoc6	386612	protein_coding	138.859101	0.158395807	0.148490465	-1.066706923	0.2861042	0.50453867	0.158395807
ENSMUSG00000052406	Rexo4	227656	protein_coding	388.681795	0.158520777	0.092716777	-1.709731302	0.087315572	0.224110872	0.158520777
ENSMUSG00000024911	Fibp	58249	protein_coding	427.7657657	-0.158520963	0.061934155	2.559507955	0.010482046	0.044259909	0.158520963
ENSMUSG00000000374	Trappc10	216131	protein_coding	2033.573954	0.158524524	0.066362506	-2.388766383	0.016905048	0.064265646	0.158524524
ENSMUSG00000029202	Pds5a	71521	protein_coding	1575.933899	0.158525468	0.069652026	-2.275963477	0.022848196	0.081458356	0.158525468
ENSMUSG00000020372	Rack1	14694	protein_coding	11895.48733	-0.158530305	0.102615797	1.544891817	0.122372433	0.285472293	0.158530305
ENSMUSG00000024340	Btnl2	547431	protein_coding	36.40047298	-0.158570466	0.395006246	0.40143787	0.688097773	0.833786242	0.158570466
ENSMUSG00000053263	Gm12592	791413	lncRNA	3.418988032	0.158648128	0.815765754	-0.194477553	0.84580197	1	0.158648128
ENSMUSG00000026069	Il1rl1	17082	protein_coding	3.475580188	-0.15870246	1.023984497	0.154985218	0.876832971	1	0.15870246
ENSMUSG00000042460	C1galt1	94192	protein_coding	3304.404108	0.158770538	0.150044339	-1.058157474	0.289983655	0.508958453	0.158770538
ENSMUSG00000107167	B3galt9	102637973	protein_coding	0.902170874	-0.158824566	1.553972013	0.102205551	0.918593522	1	0.158824566
ENSMUSG00000029713	Gnb2	14693	protein_coding	3181.631548	0.158826515	0.049648944	-3.198990804	0.001379096	0.00832093	0.158826515
ENSMUSG00000024527	Afg3l2	69597	protein_coding	1571.543025	-0.1588558	0.05510094	2.882996181	0.003939122	0.01996653	0.1588558
ENSMUSG00000000823	Zfp512b	269401	protein_coding	244.5038747	-0.159002906	0.090112431	1.764494685	0.077648729	0.205759165	0.159002906
ENSMUSG00000030029	Lrig1	16206	protein_coding	1960.805077	0.159007014	0.144603707	-1.09960538	0.271504097	0.487190988	0.159007014
ENSMUSG00000015804	Med28	66999	protein_coding	493.0832596	-0.159020027	0.087687675	1.813482076	0.069757501	0.190566644	0.159020027
ENSMUSG00000020961	Ston2	108800	protein_coding	401.0276201	-0.15908998	0.155835463	1.020884313	0.307309252	0.527754626	0.15908998
ENSMUSG00000024544	Ldlrad4	52662	protein_coding	35.10476049	-0.15909421	0.226246143	0.703190816	0.481936842	0.692663621	0.15909421
ENSMUSG00000049504	Proser1	212127	protein_coding	85.42094046	0.159094406	0.221705519	-0.71759335	0.473008059	0.685318046	0.159094406
ENSMUSG00000025500	Lmntd2	72000	protein_coding	59.7741332	-0.159104902	0.218755556	0.727318222	0.467031038	0.680659303	0.159104902
ENSMUSG00000085028	Slc2a4rg-ps	329584	transcribed_unitary_pseudogene	50.7606642	-0.159129009	0.194089288	0.819875279	0.412287211	0.632329199	0.159129009
ENSMUSG00000042626	Shc1	20416	protein_coding	941.049274	0.159132153	0.059786981	-2.661652257	0.007775816	0.034799413	0.159132153
ENSMUSG00000025220	Oga	76055	protein_coding	1615.433346	-0.159182519	0.048414739	3.287893798	0.001009399	0.006402953	0.159182519
ENSMUSG00000029629	Phf14	75725	protein_coding	388.4857469	-0.15919458	0.094156977	1.690735887	0.090887257	0.230377922	0.15919458
ENSMUSG00000030729	Pgm2l1	70974	protein_coding	239.2441964	-0.159203345	0.135163793	1.177854966	0.238854431	0.449052273	0.159203345
ENSMUSG00000039616	Mocos	68591	protein_coding	138.9512822	-0.159224397	0.191844824	0.829964517	0.406558845	0.627409847	0.159224397
ENSMUSG00000091618	H60c	670558	protein_coding	34.13455698	0.159247408	0.286923381	-0.555017189	0.578882904	0.762395109	0.159247408
ENSMUSG00000036632	Alg5	66248	protein_coding	374.1002987	0.159304801	0.108747103	-1.464910751	0.142945269	0.318770942	0.159304801
ENSMUSG00000033392	Clasp2	76499	protein_coding	315.7409898	0.159335574	0.082577153	-1.929535816	0.053664378	0.157297398	0.159335574
ENSMUSG00000047832	Cdca4	71963	protein_coding	275.3491748	0.159339116	0.11024835	-1.445274393	0.148380828	0.326976379	0.159339116
ENSMUSG00000021175	Cdca7l	217946	protein_coding	125.7894856	0.159344422	0.137821383	-1.156166182	0.24761319	0.459757614	0.159344422
ENSMUSG00000032307	Ube2q2	109161	protein_coding	278.7101655	-0.159354973	0.117833347	1.352375843	0.176255113	0.368253661	0.159354973
ENSMUSG00000046618	Olfml2a	241327	protein_coding	2.356296725	-0.159395148	0.686807087	0.232081396	0.816474794	1	0.159395148
ENSMUSG00000037742	Eef1a1	13627	protein_coding	66955.30617	-0.159397411	0.110622645	1.440911224	0.149609765	0.328982111	0.159397411
ENSMUSG00000062075	Lmnb2	16907	protein_coding	860.6863236	-0.15948353	0.076367928	2.088357429	0.036765605	0.117801163	0.15948353
ENSMUSG00000020024	Cep83	77048	protein_coding	276.7792167	0.159512584	0.089960322	-1.773143768	0.076204887	0.202844537	0.159512584

ensembl_gene_id	external_gene_name	entrezgene_id	gene_biotype	baseMean	log2FoldChange	lfcSE	stat	pvalue	padj	absLog2FC
ENSMUSG00000031352	Hccs	15159	protein_coding	313.0555181	0.159664382	0.064467087	-2.476680565	0.013261053	0.053024211	0.159664382
ENSMUSG00000031200	Mtcp1	17763	protein_coding	36.33218065	0.159685863	0.21445454	-0.744614049	0.456505073	0.671491432	0.159685863
ENSMUSG00000052974	Cyp2f2	13107	protein_coding	152.3939504	-0.159703978	0.276439555	0.577717535	0.563454839	0.750868206	0.159703978
ENSMUSG00000052681	Rap1b	215449	protein_coding	1097.10265	0.159746657	0.075113827	-2.126727719	0.033442713	0.109621469	0.159746657
ENSMUSG00000032261	Sh3bgrl2	212531	protein_coding	2571.618076	-0.159759555	0.077234943	2.06848804	0.038594154	0.122566127	0.159759555
ENSMUSG00000041143	Tmco4	77056	protein_coding	849.9724127	-0.159765634	0.0957339	1.668851199	0.095146873	0.238311216	0.159765634
ENSMUSG00000106629	Gm40155	105244562	lncRNA	1.331891768	0.159777445	1.09231206	-0.146274541	0.883704663	1	0.159777445
ENSMUSG00000058240	Cryzl1	66609	protein_coding	285.1971739	-0.159841578	0.076765435	2.082207671	0.037323505	0.119334048	0.159841578
ENSMUSG00000033282	Rpgrip1l	244585	protein_coding	122.9498009	0.159861674	0.109483075	-1.460149662	0.144248946	0.320998127	0.159861674
ENSMUSG00000032314	Etfa	110842	protein_coding	1179.25782	-0.159964614	0.069130247	2.313959819	0.020669926	0.075235434	0.159964614
ENSMUSG00000002804	Nudt14	66174	protein_coding	276.8796533	-0.159965961	0.082635109	1.935811097	0.052890841	0.155751645	0.159965961
ENSMUSG00000041355	Ssr2	66256	protein_coding	1548.889367	0.16001605	0.100751257	-1.588228821	0.112234606	0.268614824	0.16001605
ENSMUSG00000079614	Seh1l	72124	protein_coding	504.4110429	0.160023847	0.107639795	-1.486660653	0.13710446	0.309427758	0.160023847
ENSMUSG00000045795	Whamm	434204	protein_coding	257.1419507	0.160037877	0.082887494	-1.930784358	0.053509726	0.157015607	0.160037877
ENSMUSG00000085154	C130046K22Rik	399609	lncRNA	6.315412206	-0.160043341	0.405131123	0.395040846	0.692812744	0.836797094	0.160043341
ENSMUSG00000072618	Gm10384	100038735	lncRNA	29.42477144	-0.160051253	0.246243092	0.649972562	0.515709945	0.716728293	0.160051253
ENSMUSG00000031758	Cdyl2	75796	protein_coding	28.44988265	0.160052384	0.289905594	-0.552084495	0.580890464	0.764064128	0.160052384
ENSMUSG00000064264	Zfp428	232969	protein_coding	21.03887605	0.160063876	0.254313138	-0.629396803	0.529089311	0.726903567	0.160063876
ENSMUSG00000008140	Emc10	69683	protein_coding	648.4352733	-0.160072055	0.064990048	2.463024122	0.013777065	0.054559819	0.160072055
ENSMUSG00000025064	Col17a1	12821	protein_coding	14.92945471	0.160136552	0.426195617	-0.375734864	0.707114016	0.846609212	0.160136552
ENSMUSG00000024511	Rab27b	80718	protein_coding	918.5472493	0.160137711	0.181918241	-0.880272975	0.378711449	0.601173216	0.160137711
ENSMUSG00000040738	Ints8	72656	protein_coding	390.6399349	0.160158267	0.089107672	-1.797356656	0.072279017	0.19557897	0.160158267
ENSMUSG00000041720	Pi4ka	224020	protein_coding	834.5102092	-0.16016862	0.067909514	2.358559373	0.018346025	0.068544401	0.16016862
ENSMUSG00000020741	Cluh	74148	protein_coding	2974.017835	-0.160190289	0.070440509	2.27412168	0.022958672	0.081727701	0.160190289
ENSMUSG00000048445	Ccdc57	71276	protein_coding	45.00227416	-0.160201647	0.208338734	0.768947971	0.441924198	0.659670632	0.160201647
ENSMUSG00000026301	Iqca	74918	protein_coding	2.41105814	0.160232371	2.184985957	-0.073333364	0.941540843	1	0.160232371
ENSMUSG00000025531	Chm	12662	protein_coding	297.9115274	0.16025776	0.071421349	-2.243835531	0.024842985	0.086937288	0.16025776
ENSMUSG00000041263	Rusc1	72296	protein_coding	95.96319436	-0.160342808	0.1023962	1.565905851	0.11737066	0.277378268	0.160342808
ENSMUSG00000035649	Zcchc7	319885	protein_coding	430.1043593	0.160343405	0.081207922	-1.974479839	0.048327208	0.145342471	0.160343405
ENSMUSG00000052605	Isoc2b	67441	protein_coding	157.8760174	-0.160359781	0.122852342	1.305305033	0.191789001	0.389522022	0.160359781
ENSMUSG00000072258	Taf1a	21339	protein_coding	87.24456913	-0.160384564	0.115248564	1.39164046	0.1640313	0.350247335	0.160384564
ENSMUSG00000047804	Akap10	56697	protein_coding	415.5328382	-0.160391194	0.060158435	2.666146396	0.007672627	0.034419117	0.160391194
ENSMUSG00000020720	Psmd12	66997	protein_coding	576.924462	0.160408311	0.08668766	-1.850416907	0.064253484	0.179449017	0.160408311
ENSMUSG00000022010	Tsc22d1	21807	protein_coding	4251.953711	-0.160414272	0.066738962	2.403607531	0.016234192	0.062245517	0.160414272
ENSMUSG00000040820	Hlcs	110948	protein_coding	175.1739812	-0.16042608	0.14279538	1.123468279	0.261238665	0.47491467	0.16042608
ENSMUSG00000024292	Cyp4f14	64385	protein_coding	1529.276432	0.160426508	0.184511347	-0.869466892	0.384591809	0.607010336	0.160426508
ENSMUSG00000033054	Npat	244879	protein_coding	302.0202261	0.160458388	0.097493467	-1.645837325	0.099797256	0.247269599	0.160458388
ENSMUSG00000048668	Rhno1	72440	protein_coding	120.8270483	0.160484055	0.140445617	-1.142677557	0.253172491	0.465629861	0.160484055
ENSMUSG00000022836	Mylk	107589	protein_coding	629.219247	-0.160506778	0.133076105	1.206127716	0.22776825	0.436353234	0.160506778

Supplementary Table 1

ensembl_gene_id	external_gene_name	entrezgene_id	gene_biotype	baseMean	log2FoldChange	lfcSE	stat	pvalue	padj	absLog2FC
ENSMUSG00000020644	Id2	15902	protein_coding	188.6474569	0.160518974	0.211332188	-0.759557621	0.447519059	0.664777345	0.160518974
ENSMUSG00000033862	Cdk10	234854	protein_coding	222.5807347	-0.16052879	0.079318035	2.023862413	0.04298431	0.132820779	0.16052879
ENSMUSG00000059486	Kbtbd2	210973	protein_coding	489.7357074	-0.160570809	0.086048685	1.866046052	0.062034923	0.174819917	0.160570809
ENSMUSG00000020832	Eral1	57837	protein_coding	184.0202816	-0.160608647	0.105990062	1.515317988	0.129691895	0.297380166	0.160608647
ENSMUSG00000040021	Lats1	16798	protein_coding	1105.37841	0.160613071	0.061544234	-2.609717598	0.009061699	0.039359523	0.160613071
ENSMUSG00000032026	Rexo2	104444	protein_coding	213.5023952	-0.16063478	0.116615846	1.377469582	0.168367113	0.357012648	0.16063478
ENSMUSG00000001018	Snapin	20615	protein_coding	185.5619404	-0.16065561	0.103971412	1.54519023	0.122300257	0.285383751	0.16065561
ENSMUSG00000066568	Lsm14a	67070	protein_coding	642.5193262	0.160683743	0.076987969	-2.087127964	0.036876569	0.118133785	0.160683743
ENSMUSG00000042302	Ehbp1	216565	protein_coding	139.8924183	0.160686382	0.128318085	-1.252250471	0.21047861	0.414545055	0.160686382
ENSMUSG00000024038	Ndufv3	78330	protein_coding	1468.095508	0.160761979	0.063943501	-2.514125383	0.0119328	0.048873577	0.160761979
ENSMUSG00000087679	Tmem250-ps	109299	protein_coding	674.5217882	-0.160792589	0.075928962	2.117671357	0.03420291	0.111471652	0.160792589
ENSMUSG00000062203	Gspt1	14852	protein_coding	950.1009512	0.160817617	0.069823749	-2.303193679	0.021267948	0.077038583	0.160817617
ENSMUSG00000034248	Slc25a37	67712	protein_coding	310.6465352	-0.160838064	0.131191319	1.225980994	0.220205827	0.426566456	0.160838064
ENSMUSG00000086213	A330040F15Rik	74333	lncRNA	8.306844335	-0.160864149	0.428552568	0.3753662	0.707388139	0.846814623	0.160864149
ENSMUSG00000026775	Yme1l1	27377	protein_coding	2053.474203	0.160885625	0.071712374	-2.243484853	0.024865566	0.086997871	0.160885625
ENSMUSG00000002625	Akap8l	54194	protein_coding	399.2981533	0.161001466	0.148897585	-1.081289975	0.279568146	0.496963225	0.161001466
ENSMUSG00000021476	Habp4	56541	protein_coding	153.820358	0.161058902	0.092298145	-1.74498526	0.080987435	0.211618117	0.161058902
ENSMUSG00000040891	Foxa3	15377	protein_coding	703.9279152	0.161096529	0.166791323	-0.965856775	0.334115854	0.556489079	0.161096529
ENSMUSG00000073436	Eme2	193838	protein_coding	103.9156813	0.161170053	0.130217429	-1.237699546	0.215827487	0.421098196	0.161170053
ENSMUSG00000039967	Zfp292	30046	protein_coding	958.8566734	-0.161193712	0.070786576	2.277179114	0.022775532	0.081322191	0.161193712
ENSMUSG00000054321	Taf4b	72504	protein_coding	128.3238018	0.161264525	0.156775516	-1.028633357	0.303651997	0.524146449	0.161264525
ENSMUSG00000026991	Pkp4	227937	protein_coding	1002.183333	0.161337103	0.061130904	-2.63920687	0.008310025	0.036683176	0.161337103
ENSMUSG00000020948	Klhl28	66689	protein_coding	152.2844911	-0.1613404	0.127096444	1.26943284	0.20428673	0.406554718	0.1613404
ENSMUSG00000028651	Ppie	56031	protein_coding	195.6697184	-0.161444766	0.14393565	1.121645443	0.262013222	0.475826228	0.161444766
ENSMUSG00000051034	Zfp11	22648	protein_coding	26.95791606	-0.161548138	0.204549719	0.789774431	0.429659514	0.648455238	0.161548138
ENSMUSG00000022427	Tomm22	223696	protein_coding	1057.55404	-0.161576683	0.089747938	1.800338677	0.071807177	0.194652614	0.161576683
ENSMUSG00000051285	Pcmtd1	319263	protein_coding	529.7252526	-0.16161984	0.111425345	1.450476456	0.1469257	0.324759105	0.16161984
ENSMUSG00000105552	Gm19710	100503469	lncRNA	1.797380846	-0.16164054	0.865593043	0.186739648	0.851864769	1	0.16164054
ENSMUSG00000074925	Ptar1	72351	protein_coding	447.0602808	0.161648619	0.11886474	-1.359937515	0.173849698	0.364658188	0.161648619
ENSMUSG00000087396	4933407K13Rik	74396	lncRNA	36.065512	0.16165878	0.236718646	-0.682915278	0.494660379	0.700763122	0.16165878
ENSMUSG00000058704	Memo1	76890	protein_coding	325.5931556	-0.161667423	0.080808021	2.000635853	0.045431647	0.138818348	0.161667423
ENSMUSG00000068036	Afdn	17356	protein_coding	2354.289793	-0.161791765	0.069072	2.342363965	0.019162018	0.070982855	0.161791765
ENSMUSG00000070002	Ell	13716	protein_coding	329.420306	0.161797163	0.159353995	-1.015331705	0.309947736	0.530962336	0.161797163
ENSMUSG00000022462	Slc38a2	67760	protein_coding	2294.750585	0.161838996	0.064422206	-2.512161657	0.011999411	0.049101931	0.161838996
ENSMUSG00000037242	Clic4	29876	protein_coding	651.1385301	-0.161839735	0.109108819	1.483287393	0.13799807	0.31052626	0.161839735
ENSMUSG00000038564	Ift172	67661	protein_coding	333.9554467	0.161843658	0.065017864	-2.489218333	0.012802432	0.051565697	0.161843658
ENSMUSG00000064179	Tnnt1	21955	protein_coding	22.64541217	-0.161913455	0.351756682	0.460299586	0.645301198	0.806814922	0.161913455
ENSMUSG00000027936	Crtc2	74343	protein_coding	132.298355	-0.161967883	0.125150933	1.294180381	0.195603122	0.394941012	0.161967883
ENSMUSG00000058192	Zfp846	244721	protein_coding	102.2629385	0.161972551	0.105424391	-1.536385927	0.124443756	0.289079222	0.161972551

Supplementary Table 1

ensembl_gene_id	external_gene_name	entrezgene_id	gene_biotype	baseMean	log2FoldChange	lfcSE	stat	pvalue	padj	absLog2FC
ENSMUSG00000002733	Plekha3	83435	protein_coding	460.7360407	-0.161996021	0.10966964	1.477127314	0.139641513	0.31327808	0.161996021
ENSMUSG00000097327	E030030I06Rik	319887	transcribed_unprocessed_pseudogene	60.82592792	0.162017959	0.163223106	-0.992616564	0.32089685	0.542047573	0.162017959
ENSMUSG00000053164	Gpr21	338346	protein_coding	11.69089603	-0.16202883	0.346977147	0.466972629	0.640519461	0.803690806	0.16202883
ENSMUSG00000020677	Ddx52	78394	protein_coding	346.8247014	0.162033713	0.086151102	-1.880808357	0.059997993	0.170740454	0.162033713
ENSMUSG00000034833	Tespa1	67596	protein_coding	3.540361985	0.16204845	0.885961639	-0.182906847	0.85487111	1	0.16204845
ENSMUSG00000021258	Ccnk	12454	protein_coding	130.6706647	0.162063427	0.152942512	-1.059636233	0.289310123	0.508154368	0.162063427
ENSMUSG00000022718	Dgcr8	94223	protein_coding	261.661673	0.162100157	0.071699865	-2.260815351	0.023770694	0.084039656	0.162100157
ENSMUSG00000045667	Smtnl2	276829	protein_coding	9.655294015	-0.162189666	0.369909499	0.438457694	0.661054532	0.816884691	0.162189666
ENSMUSG00000029534	St7	64213	protein_coding	119.0924106	0.162210006	0.131723091	-1.231447011	0.218155724	0.42426293	0.162210006
ENSMUSG00000040028	Elavl1	15568	protein_coding	834.5032088	0.162226244	0.084653439	-1.916357399	0.055319619	0.160666232	0.162226244
ENSMUSG00000033943	Mga	29808	protein_coding	756.0824431	-0.162263617	0.085105283	1.906622141	0.056569536	0.163634492	0.162263617
ENSMUSG00000052423	B4galt3	57370	protein_coding	227.8352097	0.162264635	0.090920062	-1.784695613	0.074310703	0.199366082	0.162264635
ENSMUSG00000003429	Rps11	27207	protein_coding	4184.715633	-0.162334347	0.082828072	1.959895266	0.050008033	0.148948255	0.162334347
ENSMUSG00000019790	Stxbp5	78808	protein_coding	474.7146937	0.162385172	0.057244799	-2.836679929	0.004558528	0.022511824	0.162385172
ENSMUSG00000022407	Adsl	11564	protein_coding	562.2937834	-0.162436534	0.086936277	1.868455145	0.061698657	0.174109982	0.162436534
ENSMUSG00000048186	Bend7	209645	protein_coding	90.55242041	0.162444936	0.122985515	-1.320846083	0.186552691	0.382367026	0.162444936
ENSMUSG00000026976	Pax8	18510	protein_coding	47.78504022	-0.162475332	0.186913348	0.869254837	0.384707759	0.607019295	0.162475332
ENSMUSG00000040720	Virma	66185	protein_coding	670.4472199	0.162479068	0.059720244	-2.720669864	0.006514979	0.030010701	0.162479068
ENSMUSG00000073889	Il11ra1	16157	protein_coding	99.82988062	-0.162502395	0.137559537	1.181324086	0.237474002	0.447829813	0.162502395
ENSMUSG00000113800	2210039B01Rik	73644	lncRNA	15.08915867	-0.162512005	0.297215216	0.54678225	0.584528339	0.766833571	0.162512005
ENSMUSG00000031551	Ido1	15930	protein_coding	9.16238824	-0.162518991	0.681204054	0.238576078	0.811434317	0.906704483	0.162518991
ENSMUSG00000023147	Get1	71446	protein_coding	99.65362463	0.162559758	0.144811243	-1.122563102	0.261623093	0.475244062	0.162559758
ENSMUSG00000021408	Ripk1	19766	protein_coding	637.6770698	-0.162564276	0.064351972	2.526173949	0.011531237	0.04769017	0.162564276
ENSMUSG00000038671	Arfrp1	76688	protein_coding	273.9720005	-0.162612948	0.075148436	2.163889996	0.030472793	0.102074585	0.162612948
ENSMUSG00000108948	Or2ag13	258591	protein_coding	1.548386771	-0.162619868	2.361640836	0.068858848	0.945101975	1	0.162619868
ENSMUSG00000021458	Aopep	72061	protein_coding	829.5946266	-0.162634152	0.079370135	2.049059795	0.040456268	0.127038375	0.162634152
ENSMUSG00000034063	4930590J08Rik	381798	protein_coding	6.112190554	0.162668215	0.646482295	-0.251620525	0.801334392	0.901569434	0.162668215
ENSMUSG00000026480	Ncf2	17970	protein_coding	325.3550459	0.162678084	0.098994957	-1.643296685	0.100321564	0.248345121	0.162678084
ENSMUSG00000019710	Mrpl24	67707	protein_coding	593.934937	-0.162773681	0.051843611	3.139705702	0.001691176	0.009868582	0.162773681
ENSMUSG00000032033	Barx2	12023	protein_coding	18.35335699	-0.162774023	0.249682756	0.651923366	0.514450611	0.716025064	0.162774023
ENSMUSG00000035578	Iqcg	69707	protein_coding	12.3706547	-0.16288473	0.382691791	0.425629015	0.670378194	0.822972458	0.16288473
ENSMUSG00000037419	Endod1	71946	protein_coding	4368.752447	-0.162888583	0.090437279	1.801122119	0.071683634	0.194381533	0.162888583
ENSMUSG00000026470	Stx6	58244	protein_coding	360.621276	-0.162888758	0.067215106	2.423395102	0.015376193	0.059550294	0.162888758
ENSMUSG00000002831	Plin4	57435	protein_coding	6.29293453	0.162894302	0.607460351	-0.268156271	0.788579034	0.894265208	0.162894302
ENSMUSG00000036792	Mbd5	109241	protein_coding	164.401558	-0.162907097	0.161027122	1.011674899	0.311693521	0.532495959	0.162907097
ENSMUSG00000016018	Mtrex	72198	protein_coding	554.9383702	0.162912161	0.087045589	-1.871572845	0.061265723	0.173362261	0.162912161
ENSMUSG00000026828	Galnt5	241391	protein_coding	624.0795487	0.162953911	0.23696064	-0.687683454	0.491652142	0.698491194	0.162953911
ENSMUSG00000021051	Ppp2r5e	26932	protein_coding	455.2702314	0.163016336	0.087431912	-1.864494692	0.062252266	0.175163388	0.163016336
ENSMUSG00000069208	Zfp825	235956	protein_coding	46.3728346	-0.163033519	0.188751037	0.863748994	0.387725793	0.610149013	0.163033519

ensembl_gene_id	external_gene_name	entrezgene_id	gene_biotype	baseMean	log2FoldChange	lfcSE	stat	pvalue	padj	absLog2FC
ENSMUSG00000029279	Brdt	114642	protein_coding	11.96621103	-0.163048896	0.435390096	0.374489217	0.708040377	0.847226925	0.163048896
ENSMUSG00000038732	Mboat1	218121	protein_coding	3081.649454	-0.163068279	0.11932381	1.366603017	0.171749771	0.362093159	0.163068279
ENSMUSG00000040265	Dnm3	103967	protein_coding	8.258163436	0.163119614	0.839293567	-0.194353467	0.845899123	0.924166034	0.163119614
ENSMUSG00000020634	Ubxn2a	217379	protein_coding	874.4050732	-0.163128163	0.072165027	2.260487804	0.023790993	0.084093418	0.163128163
ENSMUSG00000042901	Aida	108909	protein_coding	184.8736614	-0.163202695	0.115832824	1.408950335	0.158849854	0.34286322	0.163202695
ENSMUSG00000062127	Cttnbp2nl	80281	protein_coding	841.0084442	-0.163278354	0.085805662	1.902885561	0.057055479	0.164593053	0.163278354
ENSMUSG00000049950	Rpp38	227522	protein_coding	46.11038147	0.163293647	0.199167965	-0.819879074	0.412285047	0.632329199	0.163293647
ENSMUSG00000036054	Sugp2	234373	protein_coding	263.7561372	-0.163319813	0.096364108	1.694819951	0.090109582	0.228911821	0.163319813
ENSMUSG00000032041	Tirap	117149	protein_coding	226.842317	0.163330649	0.08474879	-1.927232818	0.053950622	0.157940182	0.163330649
ENSMUSG00000024754	Cemip2	83921	protein_coding	648.0135713	-0.163379362	0.091194767	1.79154317	0.073206173	0.197279169	0.163379362
ENSMUSG00000044627	Swi5	72931	protein_coding	832.2462993	0.163404725	0.086639158	-1.886037783	0.059289863	0.169396679	0.163404725
ENSMUSG00000036565	Ttyh3	78339	protein_coding	397.9657913	0.163405228	0.131269759	-1.244804814	0.213203506	0.417979787	0.163405228
ENSMUSG00000049800	Sertad2	58172	protein_coding	628.7468077	-0.163426084	0.062606988	2.610348933	0.009044991	0.039297286	0.163426084
ENSMUSG00000069769	Msi2	76626	protein_coding	1156.507155	0.163473931	0.136225368	-1.200025617	0.230129391	0.438743376	0.163473931
ENSMUSG00000040034	Nup43	69912	protein_coding	84.06453145	0.163557894	0.179023814	-0.913609703	0.360921966	0.583310369	0.163557894
ENSMUSG00000018433	Nol11	68979	protein_coding	266.0209135	0.163660072	0.138152512	-1.184633347	0.236162446	0.446273789	0.163660072
ENSMUSG00000070520	Nsmce3	66647	protein_coding	220.8028734	0.163662119	0.108956726	-1.502083669	0.133075502	0.302752759	0.163662119
ENSMUSG00000026946	Nmi	64685	protein_coding	278.1947461	-0.163685435	0.098727858	1.657945773	0.097328412	0.242315905	0.163685435
ENSMUSG00000060586	H2-Eb1	14969	protein_coding	21.48435743	0.163705279	0.304688299	-0.537287711	0.591068893	0.771308708	0.163705279
ENSMUSG00000022941	Ripply3	170765	protein_coding	7.430426374	-0.163713693	0.404113752	0.405117846	0.685390889	0.832307165	0.163713693
ENSMUSG00000021608	Lpcat1	210992	protein_coding	169.198609	-0.163725881	0.159662673	1.025448708	0.305151515	0.525252463	0.163725881
ENSMUSG00000044312	Neurog3	11925	protein_coding	8.649443589	0.163773686	0.420773802	-0.389220254	0.697113222	0.839403501	0.163773686
ENSMUSG00000033906	Zdhhc15	108672	protein_coding	83.1685708	-0.163786325	0.172394621	0.950066327	0.342078552	0.564006111	0.163786325
ENSMUSG00000038121	Fam210a	108654	protein_coding	569.088552	-0.163869172	0.105889031	1.547555681	0.121729309	0.28456734	0.163869172
ENSMUSG00000040498	Igsf23	70080	protein_coding	104.2160978	0.163905892	0.143082989	-1.145530248	0.251989573	0.464375257	0.163905892
ENSMUSG00000042099	Kank3	80880	protein_coding	30.70987712	0.163912064	0.195919623	-0.836629133	0.402801065	0.624177234	0.163912064
ENSMUSG00000034509	Mad2l1bp	66591	protein_coding	223.4193403	0.163928727	0.113494158	-1.444380309	0.148632026	0.327398864	0.163928727
ENSMUSG00000036309	Skp1	21402	protein_coding	1852.957205	-0.163957196	0.089616865	1.829535067	0.067319491	0.185502098	0.163957196
ENSMUSG00000022601	Zbtb11	271377	protein_coding	287.4767542	-0.164005067	0.07551742	2.171751462	0.029874415	0.100633238	0.164005067
ENSMUSG00000001249	Hpn	15451	protein_coding	78.97831495	-0.164014115	0.260941717	0.628546929	0.529645714	0.727184014	0.164014115
ENSMUSG00000031549	Ido2	209176	protein_coding	4.101948238	0.164039524	0.873708753	-0.187750808	0.851071999	1	0.164039524
ENSMUSG00000036775	Decr2	26378	protein_coding	655.9835127	-0.164182188	0.066506653	2.468658091	0.013562073	0.053943227	0.164182188
ENSMUSG00000027669	Gnb4	14696	protein_coding	16.22140488	0.164194263	0.349750521	-0.469461096	0.638740087	0.80301103	0.164194263
ENSMUSG00000030703	Gdpd3	68616	protein_coding	23.68018604	0.164217332	0.28325543	-0.579749987	0.562083229	0.750140815	0.164217332
ENSMUSG00000065551	Mir210	387206	miRNA	2.840240944	0.16422262	0.668081134	-0.245812389	0.805827466	1	0.16422262
ENSMUSG00000031024	Denn2b	76954	protein_coding	586.4428695	0.164227303	0.087293496	-1.88132347	0.05992793	0.1706035	0.164227303
ENSMUSG00000035597	Prpf39	328110	protein_coding	314.6916448	0.164261422	0.108661099	-1.511685626	0.130613857	0.298680373	0.164261422
ENSMUSG00000040913	Fbxw4	30838	protein_coding	105.6788354	0.164273921	0.169389846	-0.969797927	0.332147226	0.554606509	0.164273921
ENSMUSG00000006333	Rps9	76846	protein_coding	7216.915476	-0.164284977	0.090728327	1.81073521	0.07018185	0.191366503	0.164284977

ensembl_gene_id	external_gene_name	entrezgene_id	gene_biotype	baseMean	log2FoldChange	lfcSE	stat	pvalue	padj	absLog2FC
ENSMUSG00000028266	Lmo4	16911	protein_coding	272.8171628	-0.164320079	0.081236121	2.02274651	0.043099289	0.133085576	0.164320079
ENSMUSG00000042642	Flad1	319945	protein_coding	435.5378725	-0.164321052	0.088349254	1.859903117	0.062899234	0.176502626	0.164321052
ENSMUSG00000002107	Celf2	14007	protein_coding	44.27859336	0.164329055	0.290035671	-0.56658222	0.570998058	0.756475084	0.164329055
ENSMUSG00000041297	Cdk13	69562	protein_coding	1133.106161	0.164347696	0.055328378	-2.970405112	0.002974073	0.015751711	0.164347696
ENSMUSG00000042729	Wdr74	107071	protein_coding	250.5090517	0.164426664	0.093343109	-1.761529751	0.078148782	0.206686257	0.164426664
ENSMUSG00000028330	Ncbp1	433702	protein_coding	590.2276357	0.164431194	0.109110049	-1.50702154	0.131805136	0.300890309	0.164431194
ENSMUSG00000036430	Tbcc	72726	protein_coding	162.4293287	0.164448104	0.09819136	-1.674771627	0.093979048	0.236076969	0.164448104
ENSMUSG00000044328	Trp53i13	216964	protein_coding	56.19827722	-0.16445423	0.197153965	0.834141127	0.404201462	0.625349723	0.16445423
ENSMUSG00000030674	Qprt	67375	protein_coding	1.686399452	0.164470625	1.746121812	-0.094191954	0.924956676	1	0.164470625
ENSMUSG00000028649	Macf1	11426	protein_coding	2476.787166	0.164499244	0.103149465	-1.594765848	0.110764607	0.266215502	0.164499244
ENSMUSG00000075327	Zbtb2	381990	protein_coding	179.5453621	-0.164529238	0.108047002	1.522756166	0.12781972	0.294806545	0.164529238
ENSMUSG00000039745	Htatip2	53415	protein_coding	270.7881585	-0.16453561	0.09154316	1.7973556	0.072279185	0.19557897	0.16453561
ENSMUSG00000022861	Dgkg	110197	protein_coding	86.22307417	-0.164604129	0.219195543	0.750946513	0.452684846	0.668926246	0.164604129
ENSMUSG00000021713	Ppwd1	238831	protein_coding	166.0299303	0.164609604	0.111580598	-1.475252922	0.140144558	0.314193217	0.164609604
ENSMUSG00000032096	Arcn1	213827	protein_coding	3064.99834	0.164631389	0.088602435	-1.858091008	0.063156092	0.177042895	0.164631389
ENSMUSG00000085776	5430402O13Rik	73183	lncRNA	1.794236721	0.164647841	0.997758978	-0.165017649	0.86893009	1	0.164647841
ENSMUSG00000114755	Galr3	14429	protein_coding	2.472506609	0.164656311	0.700842951	-0.234940383	0.814255005	1	0.164656311
ENSMUSG00000003873	Bax	12028	protein_coding	266.6889685	0.164710168	0.086904308	-1.895304988	0.058052008	0.166725366	0.164710168
ENSMUSG00000029027	Dffb	13368	protein_coding	92.07855077	-0.164720326	0.12449581	1.323099363	0.185802336	0.381254943	0.164720326
ENSMUSG00000029865	Sval1	71578	protein_coding	1757.576689	0.164766271	0.532383246	-0.309488085	0.756950274	0.878075079	0.164766271
ENSMUSG00000003484	Cyp4f18	72054	protein_coding	1.294035413	0.164771898	1.217563119	-0.135329246	0.892351563	1	0.164771898
ENSMUSG00000001700	Gramd3	107022	protein_coding	853.8991258	-0.164779161	0.124128568	1.327487808	0.18434737	0.379371759	0.164779161
ENSMUSG00000078588	Ccdc24	381546	protein_coding	2.996058309	0.164782867	0.680513911	-0.242144745	0.808668008	1	0.164782867
ENSMUSG00000001062	Vps9d1	72325	protein_coding	209.6321519	-0.164787235	0.085843901	1.919614933	0.054906556	0.15977566	0.164787235
ENSMUSG00000054659	Pm20d2	242377	protein_coding	23.26128489	-0.164824177	0.214199434	0.76948932	0.441602882	0.659260011	0.164824177
ENSMUSG00000025645	Ccdc51	66658	protein_coding	206.0809152	-0.164825682	0.103059422	1.599326663	0.109748038	0.264619521	0.164825682
ENSMUSG00000036199	Ndufa13	67184	protein_coding	1742.216452	-0.16482818	0.073965214	2.228455387	0.025850163	0.089682688	0.16482818
ENSMUSG00000079429	Mroh2a	100040766	protein_coding	112.012606	0.164861989	0.255654876	-0.644861509	0.519016937	0.719334577	0.164861989
ENSMUSG00000036613	Eipr1	380752	protein_coding	289.9350197	0.164882076	0.081104469	-2.032959193	0.042056646	0.130795377	0.164882076
ENSMUSG00000061780	Cfd	11537	protein_coding	1.654985867	0.164946992	1.466594165	-0.112469418	0.910451217	1	0.164946992
ENSMUSG00000039264	Gimap3	83408	protein_coding	30.42232825	0.164951606	0.302326177	-0.545608082	0.585335367	0.767282764	0.164951606
ENSMUSG00000001138	Cnnm3	94218	protein_coding	279.2170946	0.164971308	0.081264286	-2.030059142	0.042350528	0.131338332	0.164971308
ENSMUSG00000039810	Zc3h10	103284	protein_coding	195.8625891	-0.164983355	0.087838711	1.878253359	0.060346514	0.171466333	0.164983355
ENSMUSG00000060716	Plekhh1	211945	protein_coding	1467.468619	-0.165005736	0.0836772	1.971931857	0.048617386	0.145896332	0.165005736
ENSMUSG00000069237	Fam8a1	97863	protein_coding	826.6915626	0.165026026	0.08702888	-1.896221415	0.057930773	0.166521981	0.165026026
ENSMUSG00000021930	Spryd7	66674	protein_coding	267.4386052	-0.165036259	0.096956798	1.702162837	0.088724847	0.226286043	0.165036259
ENSMUSG00000018848	Rars	104458	protein_coding	963.5441283	0.165102278	0.06688193	-2.468563326	0.013565665	0.053943227	0.165102278
ENSMUSG00000035139	Secisbp2	75420	protein_coding	262.447109	-0.165103024	0.079426525	2.078688761	0.037645967	0.120138597	0.165103024
ENSMUSG00000002028	Kmt2a	214162	protein_coding	595.769029	0.165118514	0.176043625	-0.937940893	0.348274787	0.570180413	0.165118514

ensembl_gene_id	external_gene_name	entrezgene_id	gene_biotype	baseMean	log2FoldChange	lfcSE	stat	pvalue	padj	absLog2FC
ENSMUSG00000031557	Plekha2	83436	protein_coding	852.5406775	-0.165133198	0.097402387	1.695371168	0.090005032	0.228807636	0.165133198
ENSMUSG00000020346	Mgat1	17308	protein_coding	970.1125763	-0.165244858	0.063135112	2.617321054	0.008862294	0.038640192	0.165244858
ENSMUSG00000055137	Sugct	192136	protein_coding	21.55472587	-0.165250186	0.237216115	0.696622932	0.486038801	0.695595302	0.165250186
ENSMUSG00000005150	Wdr83	67836	protein_coding	152.2971956	-0.165250811	0.105684911	1.563617817	0.117907348	0.278398906	0.165250811
ENSMUSG00000075704	Txnrd2	26462	protein_coding	128.4815588	-0.165251588	0.100786445	1.639621164	0.10108396	0.249608668	0.165251588
ENSMUSG00000042043	Tbca	21371	protein_coding	526.8622185	-0.165274441	0.097985087	1.686730567	0.091655172	0.231775376	0.165274441
ENSMUSG00000032845	Alpk2	225638	protein_coding	1.551064803	-0.165343922	2.136455917	0.077391685	0.938311956	1	0.165343922
ENSMUSG00000020803	Txndc17	52700	protein_coding	1040.514391	-0.165386368	0.067681222	2.443607869	0.014541222	0.056890248	0.165386368
ENSMUSG00000049323	Smcr8	237782	protein_coding	561.3732788	0.165390786	0.080062483	-2.065771386	0.038850071	0.123236664	0.165390786
ENSMUSG00000048826	Dact2	240025	protein_coding	320.0927784	-0.165416307	0.140920508	1.173827071	0.240464298	0.450945654	0.165416307
ENSMUSG00000034707	Gns	75612	protein_coding	777.9486858	-0.165467197	0.050073324	3.304497989	0.000951467	0.00610432	0.165467197
ENSMUSG00000021996	Esd	13885	protein_coding	1755.685558	-0.165486692	0.077261548	2.141902366	0.032201339	0.106567719	0.165486692
ENSMUSG00000022969	Il10rb	16155	protein_coding	484.1918311	-0.165515571	0.07070035	2.341085604	0.019227758	0.071194438	0.165515571
ENSMUSG00000054342	Kcnn4	16534	protein_coding	628.1862516	-0.165637367	0.153534663	1.07882718	0.280664776	0.498301136	0.165637367
ENSMUSG00000029472	Anapc5	59008	protein_coding	1808.196903	0.165695677	0.087393287	-1.895977162	0.057963064	0.166571051	0.165695677
ENSMUSG00000101013	A630072M18Rik	320770	lncRNA	2.912692288	0.165749891	0.822768775	-0.201453794	0.840343756	1	0.165749891
ENSMUSG00000026970	Rbms1	56878	protein_coding	214.5223176	-0.165763687	0.103663655	1.59905308	0.109808809	0.264727397	0.165763687
ENSMUSG00000102629	Gm29999	102631733	lncRNA	1.10648684	0.16577657	2.644859176	-0.062678789	0.950022288	1	0.16577657
ENSMUSG00000024298	Zfp871	208292	protein_coding	677.253412	-0.165835918	0.102275384	1.621464635	0.104918029	0.256152621	0.165835918
ENSMUSG00000036285	Noa1	56412	protein_coding	185.7815432	-0.165840565	0.119632864	1.386245879	0.165671837	0.352762472	0.165840565
ENSMUSG00000019900	Rfx6	320995	protein_coding	11.36528297	-0.165873451	0.39414067	0.420848352	0.673865821	0.825096143	0.165873451
ENSMUSG00000020621	Rdh14	105014	protein_coding	337.4347965	0.165881628	0.099871418	-1.66095197	0.096723093	0.241136045	0.165881628
ENSMUSG00000012211	Tex22	75671	protein_coding	1.772839404	0.165917784	1.113741863	-0.148973285	0.881574713	1	0.165917784
ENSMUSG00000051853	Arf3	11842	protein_coding	1469.931698	-0.165964079	0.04125137	4.023238013	5.74E-05	0.000528406	0.165964079
ENSMUSG00000005505	Kbtbd4	67136	protein_coding	74.85362703	-0.166016781	0.138720673	1.196770296	0.231396099	0.440188363	0.166016781
ENSMUSG00000027661	Slc2a10	170441	protein_coding	141.578363	-0.166050882	0.146442464	1.133898443	0.25683715	0.469910115	0.166050882
ENSMUSG00000068479	Mfap1a	67532	protein_coding	188.0579281	-0.166251927	0.125920371	1.320294134	0.186736833	0.382601993	0.166251927
ENSMUSG00000065273	Gm25128	115488831	snoRNA	4.719228525	-0.166263377	0.483197167	0.344090132	0.730778504	0.861267215	0.166263377
ENSMUSG00000072049	Vmn2r121	100038941	protein_coding	1.976995173	0.16630737	2.655015217	-0.062638952	0.950054011	1	0.16630737
ENSMUSG00000021809	Nudt13	67725	protein_coding	145.8437399	-0.166322551	0.092879191	1.790740742	0.073334908	0.197497175	0.166322551
ENSMUSG00000025533	Asl	109900	protein_coding	361.3716769	-0.1663472	0.084025347	1.979726412	0.047734279	0.143952499	0.1663472
ENSMUSG00000068959	Zfp619	70227	protein_coding	33.49358062	-0.166352933	0.210653029	0.789701119	0.429702337	0.64845799	0.166352933
ENSMUSG00000038398	Upf3a	67031	protein_coding	381.4934391	-0.166376949	0.083350307	1.996116815	0.045921209	0.139760938	0.166376949
ENSMUSG00000025198	Erlin1	226144	protein_coding	633.4024936	0.166389202	0.08664647	-1.920322923	0.054817123	0.159701725	0.166389202
ENSMUSG00000068794	Col28a1	213945	protein_coding	19.81307103	0.166441673	0.252264318	-0.659790785	0.509388098	0.712785998	0.166441673
ENSMUSG00000039703	Nploc4	217365	protein_coding	885.3897604	0.166452471	0.066892886	-2.488343419	0.012833974	0.051654946	0.166452471
ENSMUSG00000039640	Mrpl12	56282	protein_coding	884.0264894	-0.166458093	0.076018181	2.189714246	0.028544967	0.097034086	0.166458093
ENSMUSG00000047143	Dmrta2	242620	protein_coding	4.909099811	0.1664749	0.623877742	-0.26683898	0.78959314	0.894883064	0.1664749
ENSMUSG00000071659	Hnrnpul2	68693	protein_coding	2042.047083	0.166475988	0.05133705	-3.242803954	0.001183596	0.00731937	0.166475988

Supplementary Table 1

ensembl_gene_id	external_gene_name	entrezgene_id	gene_biotype	baseMean	log2FoldChange	lfcSE	stat	pvalue	padj	absLog2FC
ENSMUSG00000022682	Rrn3	106298	protein_coding	479.4440879	0.166506312	0.09898916	-1.682066111	0.092556021	0.233354218	0.166506312
ENSMUSG00000021211	Akr1c12	622402	protein_coding	1355.208157	-0.166607961	0.073795543	2.257696812	0.023964568	0.084507982	0.166607961
ENSMUSG00000024180	Pgap6	60455	protein_coding	1398.940373	-0.166614852	0.059034657	2.822322701	0.004767718	0.023369313	0.166614852
ENSMUSG00000034320	Slc26a2	13521	protein_coding	13764.11954	0.166637559	0.116545882	-1.429802197	0.152773798	0.33362953	0.166637559
ENSMUSG00000034259	Exosc4	109075	protein_coding	197.5294121	0.166658817	0.0934882	-1.78267222	0.074639676	0.199902629	0.166658817
ENSMUSG00000021497	Txndc15	69672	protein_coding	420.0435906	-0.166673964	0.073431965	2.269773992	0.023221299	0.082503558	0.166673964
ENSMUSG00000071112	Spx	319552	protein_coding	2.455548208	0.166723789	0.808225378	-0.206283783	0.836569245	1	0.166723789
ENSMUSG00000040455	Usp45	77593	protein_coding	323.547897	-0.166725135	0.093336267	1.786284582	0.074053192	0.198945226	0.166725135
ENSMUSG00000010021	Kif19a	286942	protein_coding	12.68471478	-0.16672846	0.545303421	0.305753556	0.759792293	0.879422681	0.16672846
ENSMUSG00000062762	Ei24	13663	protein_coding	1031.47018	-0.16675286	0.089109762	1.871319777	0.061300771	0.173431717	0.16675286
ENSMUSG00000021192	Golga5	27277	protein_coding	734.3687518	0.166800903	0.078134721	-2.134785919	0.032778501	0.108084245	0.166800903
ENSMUSG00000021012	Zc3h14	75553	protein_coding	787.8053597	0.166804714	0.069211501	-2.410072195	0.015949364	0.061381449	0.166804714
ENSMUSG00000000544	Gpa33	59290	protein_coding	6357.056546	-0.166807686	0.052328496	3.18770267	0.001434079	0.008569101	0.166807686
ENSMUSG00000047423	AI837181	107242	protein_coding	308.2879792	-0.166821661	0.068086265	2.450151442	0.014279614	0.056092661	0.166821661
ENSMUSG00000038167	Plekhg6	213522	protein_coding	317.2407175	-0.166865058	0.279903205	0.596152724	0.551073205	0.741922624	0.166865058
ENSMUSG00000044361	BC024139	271278	protein_coding	27.36639905	0.166866626	0.232532286	-0.717606269	0.473000091	0.685318046	0.166866626
ENSMUSG00000029364	Wsb2	59043	protein_coding	800.7578525	-0.16687334	0.086687956	1.924988734	0.054230768	0.158479366	0.16687334
ENSMUSG00000021357	Exoc2	66482	protein_coding	325.6037746	-0.166901082	0.110974634	1.503957041	0.132592428	0.302143144	0.166901082
ENSMUSG00000028292	Rars2	109093	protein_coding	441.1938409	-0.166949868	0.097765687	1.707653006	0.087700747	0.224854857	0.166949868
ENSMUSG00000029017	Pmpcb	73078	protein_coding	871.6992277	-0.166960047	0.084233507	1.982109657	0.047466969	0.143382024	0.166960047
ENSMUSG00000002963	Pnkp	59047	protein_coding	338.8132175	-0.167080175	0.072571193	2.302293356	0.021318634	0.077202358	0.167080175
ENSMUSG00000041849	Card6	239319	protein_coding	44.92230794	0.167096959	0.262112377	-0.637501214	0.523798419	0.722399123	0.167096959
ENSMUSG00000044456	Rin3	217835	protein_coding	52.8999575	-0.167127467	0.256651555	0.651184313	0.514927516	0.716272317	0.167127467
ENSMUSG00000013523	Bcas1	76960	protein_coding	1057.469802	0.167165719	0.188643344	-0.886146921	0.375538362	0.597748482	0.167165719
ENSMUSG00000063796	Slc22a8	19879	protein_coding	2.62233006	0.167173104	2.672967045	-0.062542149	0.950131097	1	0.167173104
ENSMUSG00000031293	Rs1	20147	protein_coding	2.9890196	0.167290115	1.386910386	-0.12062071	0.903991465	1	0.167290115
ENSMUSG00000021981	Cab39l	69008	protein_coding	465.6122326	-0.167341678	0.108182978	1.546839259	0.121902011	0.284857762	0.167341678
ENSMUSG00000034453	Polr3b	70428	protein_coding	351.5863137	-0.167405446	0.106475255	1.572247431	0.115893179	0.274894315	0.167405446
ENSMUSG00000041762	Gpr155	68526	protein_coding	8.743753689	0.167409017	0.392647423	-0.426359647	0.669845801	0.822722373	0.167409017
ENSMUSG00000032305	Fam219b	78323	protein_coding	152.1632095	-0.167428877	0.109537518	1.52850713	0.126386675	0.292373241	0.167428877
ENSMUSG00000026113	Inpp4a	269180	protein_coding	153.6491237	-0.167433332	0.136386829	1.227635642	0.219583776	0.425861007	0.167433332
ENSMUSG00000070985	Acnat1	230161	protein_coding	358.0610235	-0.167437031	0.138765608	1.206617649	0.227579427	0.436092673	0.167437031
ENSMUSG00000026810	Dpm2	13481	protein_coding	241.979861	0.167492718	0.104606786	-1.601164936	0.109340393	0.263984696	0.167492718
ENSMUSG00000032733	Snx33	235406	protein_coding	318.865961	-0.1675095	0.077401666	2.164158833	0.030452161	0.102046874	0.1675095
ENSMUSG00000055670	Zzef1	195018	protein_coding	2593.609642	0.167539874	0.090851951	-1.8440977	0.065168903	0.181269925	0.167539874
ENSMUSG00000008333	Snrpb2	20639	protein_coding	250.4870873	0.167545761	0.103466939	-1.619316879	0.105379101	0.257088266	0.167545761
ENSMUSG00000029125	Stx18	71116	protein_coding	231.6783941	0.167558	0.089925816	-1.863291414	0.062421278	0.175579114	0.167558
ENSMUSG00000039630	Hnrnpu	51810	protein_coding	4573.946112	0.167562522	0.052716113	-3.17858264	0.00147997	0.008791449	0.167562522
ENSMUSG00000041685	Fcho2	218503	protein_coding	633.9611959	-0.16756448	0.105754348	1.58446894	0.113087044	0.270067889	0.16756448

ensembl_gene_id	external_gene_name	entrezgene_id	gene_biotype	baseMean	log2FoldChange	lfcSE	stat	pvalue	padj	absLog2FC
ENSMUSG00000030243	Recql	19691	protein_coding	164.8688465	0.16765257	0.09381347	-1.787084198	0.073923881	0.198694494	0.16765257
ENSMUSG00000024507	Hsd17b4	15488	protein_coding	1030.408261	-0.167678014	0.067358481	2.489337819	0.012798129	0.051560944	0.167678014
ENSMUSG00000047746	Fbxo40	207215	protein_coding	1.064085804	-0.167710837	2.026577229	0.08275571	0.934045787	1	0.167710837
ENSMUSG00000028646	Rragc	54170	protein_coding	565.012456	0.167724267	0.094145414	-1.781544737	0.074823503	0.19999509	0.167724267
ENSMUSG00000053091	Lins1	72635	protein_coding	98.42505994	0.167724894	0.116795044	-1.43606174	0.150984774	0.331035921	0.167724894
ENSMUSG00000028270	Gbp2	14469	protein_coding	61.95497333	-0.167739672	0.366091102	0.458191064	0.646815179	0.807553177	0.167739672
ENSMUSG00000063929	Cyp4a32	100040843	protein_coding	1.644414415	-0.167749584	2.693693896	0.062274924	0.950343897	1	0.167749584
ENSMUSG00000024622	Hmgxb3	106894	protein_coding	530.3086374	-0.167755075	0.060668577	2.765106488	0.005690422	0.027104593	0.167755075
ENSMUSG00000020272	Stk10	20868	protein_coding	477.6252499	-0.167846688	0.254972233	0.658293987	0.510349241	0.71356425	0.167846688
ENSMUSG00000041644	Slc5a12	241612	protein_coding	4.957144405	0.167899258	1.706013127	-0.098416158	0.921601845	0.962213622	0.167899258
ENSMUSG00000071716	Apol7e	666348	protein_coding	4.857197178	0.167900568	0.664625671	-0.252624259	0.800558582	0.901189824	0.167900568
ENSMUSG00000022677	Cep20	66086	protein_coding	446.8079597	0.167934176	0.079031157	-2.124911007	0.033594039	0.109921135	0.167934176
ENSMUSG00000024726	Carnmt1	67383	protein_coding	92.29481802	-0.167934854	0.145900541	1.151022832	0.249722843	0.462014445	0.167934854
ENSMUSG00000036197	Gxylt1	223827	protein_coding	458.0819575	0.167976544	0.083422017	-2.013575661	0.044054104	0.1353506	0.167976544
ENSMUSG00000074652	Myh7b	668940	protein_coding	6.613884007	0.168046712	0.733457708	-0.229115749	0.818778953	0.908812719	0.168046712
ENSMUSG00000024747	Aldh1a7	26358	protein_coding	238.9203625	-0.16809526	0.116616975	1.441430466	0.149463108	0.328844638	0.16809526
ENSMUSG00000057113	Npm1	18148	protein_coding	2400.795357	-0.168248369	0.128890918	1.305354732	0.191772085	0.389522022	0.168248369
ENSMUSG00000061130	Ppm1b	19043	protein_coding	1080.938619	0.168252461	0.061381831	-2.741079205	0.006123774	0.028631939	0.168252461
ENSMUSG00000023991	Foxp4	74123	protein_coding	585.1177393	0.168391377	0.129090091	-1.304448513	0.192080704	0.389807821	0.168391377
ENSMUSG00000063851	Rnf183	76072	protein_coding	8.183677877	0.168398387	0.538407395	-0.312771312	0.754454412	0.876856731	0.168398387
ENSMUSG00000051998	Lax1	240754	protein_coding	4.405173518	-0.168415654	0.664209991	0.253557846	0.799837167	0.900866933	0.168415654
ENSMUSG00000015750	Aph1a	226548	protein_coding	740.4180466	-0.168437137	0.108172647	1.557113947	0.119443434	0.28110145	0.168437137
ENSMUSG00000036334	Igsf10	242050	protein_coding	2.216569948	-0.168458909	1.461314054	0.115279059	0.908223936	1	0.168458909
ENSMUSG00000075908	Gm23940	115489070	snRNA	1.682673142	-0.168485179	0.86624076	0.19450156	0.845783174	1	0.168485179
ENSMUSG00000031103	Elf4	56501	protein_coding	643.8904753	-0.168549501	0.132286996	1.27411995	0.202620909	0.404694914	0.168549501
ENSMUSG00000078765	U2af1l4	233073	protein_coding	4.85003259	-0.168661187	0.522285797	0.322928918	0.746749075	0.872440598	0.168661187
ENSMUSG00000095315	Gm10130	102633750	protein_coding	36.53737851	-0.168708817	0.226467613	0.74495781	0.456297225	0.671470886	0.168708817
ENSMUSG00000032854	Ugt8a	22239	protein_coding	115.0030754	0.168712269	0.223813831	-0.753806272	0.450965557	0.667636772	0.168712269
ENSMUSG00000052446	Zfp961	234413	protein_coding	173.4539545	0.168754977	0.106197593	-1.589065934	0.112045508	0.268218365	0.168754977
ENSMUSG00000061410	Zcchc14	142682	protein_coding	545.1655815	0.168756357	0.054826335	-3.078016359	0.002083835	0.01173204	0.168756357
ENSMUSG00000051730	Mettl5	75422	protein_coding	80.94092471	-0.168940978	0.119160032	1.417765469	0.156259249	0.338979766	0.168940978
ENSMUSG00000031570	Plpp5	71910	protein_coding	553.9112411	0.168945861	0.07081883	-2.385606489	0.017050985	0.06471615	0.168945861
ENSMUSG00000034263	Ints14	69882	protein_coding	358.0111263	0.168971093	0.089748282	-1.882722313	0.05973801	0.17026467	0.168971093
ENSMUSG00000026556	Vangl2	93840	protein_coding	30.59334461	0.168972015	0.231287971	-0.730569838	0.465041941	0.6785988	0.168972015
ENSMUSG00000062861	Zfp28	22690	protein_coding	28.12978736	-0.169053516	0.263954661	0.640464218	0.521870845	0.721306924	0.169053516
ENSMUSG00000027805	Pfn2	18645	protein_coding	13.13998437	0.169057923	0.33804728	-0.500101414	0.61700367	0.788881899	0.169057923
ENSMUSG00000027673	Ndufb5	66046	protein_coding	1201.668883	-0.169109173	0.120173344	1.407210346	0.159365025	0.343481339	0.169109173
ENSMUSG00000039633	Lonrf1	244421	protein_coding	59.50320072	0.169196489	0.176827313	-0.956845896	0.338645043	0.560303	0.169196489
ENSMUSG00000025127	Gcgr	14527	protein_coding	2.038236806	0.169203707	2.174010987	-0.077830198	0.937963124	1	0.169203707

ensembl_gene_id	external_gene_name	entrezgene_id	gene_biotype	baseMean	log2FoldChange	lfcSE	stat	pvalue	padj	absLog2FC
ENSMUSG00000056201	Cfl1	12631	protein_coding	3177.317836	-0.169244777	0.07783061	2.174527181	0.029665566	0.10006477	0.169244777
ENSMUSG00000117231	Gm41609	105246306	lncRNA	2.783411581	-0.169264519	0.842677912	0.200865024	0.840804117	1	0.169264519
ENSMUSG00000061577	Adgrg5	382045	protein_coding	6.589052167	-0.169297381	0.581688948	0.291044521	0.77101727	0.885199039	0.169297381
ENSMUSG00000004788	Eif2b2	217715	protein_coding	335.7621947	0.169346511	0.074274163	-2.28001912	0.022606555	0.080865411	0.169346511
ENSMUSG00000037971	1110032A03Rik	68721	protein_coding	211.5008061	-0.169369603	0.093896558	1.803789252	0.071264351	0.193498767	0.169369603
ENSMUSG00000059554	Ccdc28a	215814	protein_coding	161.9738904	-0.169433923	0.129021685	1.31322051	0.189108655	0.386024762	0.169433923
ENSMUSG00000030532	Hddc3	68695	protein_coding	24.7222715	-0.169440409	0.237840825	0.712410954	0.476210334	0.687965835	0.169440409
ENSMUSG00000027180	Fbxo3	57443	protein_coding	638.4196973	-0.169492921	0.101538651	1.669245344	0.095068767	0.23816226	0.169492921
ENSMUSG00000036611	Eepd1	67484	protein_coding	67.36514369	-0.169550877	0.188225527	0.90078577	0.367702235	0.590110273	0.169550877
ENSMUSG00000021532	Fastkd3	69577	protein_coding	228.9781356	0.169560687	0.081132264	-2.089929186	0.036624161	0.117439107	0.169560687
ENSMUSG00000038976	Ppp1r9b	217124	protein_coding	703.8971078	-0.169606394	0.105882374	1.601837849	0.109191471	0.263739645	0.169606394
ENSMUSG00000022474	Pmm1	29858	protein_coding	300.4413957	-0.1696416	0.125722719	1.349331296	0.177230578	0.369543657	0.1696416
ENSMUSG00000010476	Ebf3	13593	protein_coding	8.512643667	-0.169677051	0.546051933	0.310734274	0.75600264	0.877468906	0.169677051
ENSMUSG00000020864	Ankrd40	71452	protein_coding	591.0432317	-0.169695984	0.0640579	2.649103148	0.00807057	0.035875474	0.169695984
ENSMUSG00000042328	Hps4	192232	protein_coding	336.985424	0.169700712	0.060796886	-2.791273077	0.005250116	0.025395551	0.169700712
ENSMUSG00000000983	Wfdc18	14038	protein_coding	61.86872149	0.169779619	0.221107892	-0.767858702	0.442571135	0.660397553	0.169779619
ENSMUSG00000038910	Plcl2	224860	protein_coding	1060.775626	-0.16980924	0.091176828	1.862416629	0.062544387	0.175865487	0.16980924
ENSMUSG00000022325	Pop1	67724	protein_coding	102.3439424	0.169889177	0.120689334	-1.407656924	0.159232683	0.343330529	0.169889177
ENSMUSG00000028780	Sema3c	20348	protein_coding	1664.906088	-0.169891974	0.128039538	1.326871191	0.184551296	0.379678597	0.169891974
ENSMUSG00000049892	Rasd1	19416	protein_coding	411.3524952	0.169918052	0.180761121	-0.940014373	0.347210188	0.569001592	0.169918052
ENSMUSG00000056515	Rab31	106572	protein_coding	92.85626726	-0.169948066	0.144631822	1.175039241	0.239979017	0.450393623	0.169948066
ENSMUSG00000005262	Ufd1	22230	protein_coding	396.216614	0.169952975	0.058527706	-2.903803784	0.003686591	0.018918697	0.169952975
ENSMUSG00000024069	Slc30a6	210148	protein_coding	245.3608576	0.169962353	0.09578267	-1.774458292	0.075987375	0.20239605	0.169962353
ENSMUSG00000029433	Diablo	66593	protein_coding	65.1761842	-0.170000975	0.154718724	1.098774411	0.271866476	0.487629303	0.170000975
ENSMUSG00000066877	Nck2	17974	protein_coding	853.0925249	-0.170137254	0.078946269	2.155101903	0.031153861	0.103913322	0.170137254
ENSMUSG00000030678	Maz	17188	protein_coding	677.0743007	0.17013984	0.113286576	-1.501853499	0.133134948	0.302752759	0.17013984
ENSMUSG00000009863	Sdhb	67680	protein_coding	2465.321882	-0.170195002	0.083677173	2.033947808	0.041956858	0.130534204	0.170195002
ENSMUSG00000035258	Abi3bp	320712	protein_coding	8.075400673	0.170225729	0.611631218	-0.278314324	0.780771081	0.890384202	0.170225729
ENSMUSG00000053441	Adamts19	240322	protein_coding	2.363738757	-0.170290293	2.653636139	0.064172435	0.948832926	1	0.170290293
ENSMUSG00000030216	Wbp11	60321	protein_coding	560.6834918	0.170303702	0.080641149	-2.111870983	0.034697517	0.112749861	0.170303702
ENSMUSG00000032898	Fbxo21	231670	protein_coding	257.7404179	0.170309681	0.152725582	-1.115135259	0.264792464	0.479629566	0.170309681
ENSMUSG00000049148	Plcxd3	239318	protein_coding	2.655909859	-0.170329931	0.812474232	0.209643486	0.833945936	1	0.170329931
ENSMUSG00000022346	Myc	17869	protein_coding	267.7016921	0.170352867	0.13754758	-1.23850138	0.215530211	0.420617573	0.170352867
ENSMUSG00000031751	Amfr	23802	protein_coding	1974.139966	-0.170374538	0.078460778	2.171461225	0.029896325	0.100654009	0.170374538
ENSMUSG00000036850	Mrpl41	107733	protein_coding	238.8957244	-0.170399382	0.094681196	1.799717253	0.071905295	0.194854619	0.170399382
ENSMUSG00000029049	Morn1	76866	protein_coding	39.02538386	-0.170458897	0.16953786	1.005432632	0.314688574	0.535859673	0.170458897
ENSMUSG00000078784	Rbis	69126	protein_coding	238.5433821	-0.170471728	0.110047096	1.549079748	0.121362551	0.284132491	0.170471728
ENSMUSG00000026827	Gpd2	14571	protein_coding	685.0719151	-0.170491181	0.089117929	1.91309631	0.055735724	0.161590545	0.170491181
ENSMUSG00000041354	Rgl2	19732	protein_coding	394.967256	-0.170539866	0.073781456	2.311419081	0.020809718	0.075644218	0.170539866

ensembl_gene_id	external_gene_name	entrezgene_id	gene_biotype	baseMean	log2FoldChange	lfcSE	stat	pvalue	padj	absLog2FC
ENSMUSG00000026000	Lancl1	14768	protein_coding	241.1745976	0.170554406	0.122483777	-1.392465274	0.16378155	0.349988901	0.170554406
ENSMUSG00000071533	Pcnp	76302	protein_coding	450.8615899	-0.170590964	0.108607489	1.570710882	0.11624982	0.275430349	0.170590964
ENSMUSG00000031158	Timm17b	21855	protein_coding	315.7352061	0.170622167	0.11112171	-1.535453036	0.124672585	0.289447921	0.170622167
ENSMUSG00000020153	Ndufs7	75406	protein_coding	981.0932989	-0.170691191	0.075576235	2.258529948	0.02391264	0.08439695	0.170691191
ENSMUSG00000032599	Ip6k2	76500	protein_coding	197.2940681	-0.17071889	0.082880479	2.059820277	0.039415725	0.124671766	0.17071889
ENSMUSG00000045679	Pqlc3	217430	protein_coding	288.3912373	0.170729261	0.107160403	-1.59321219	0.111112598	0.26676599	0.170729261
ENSMUSG00000008682	Rpl10	110954	protein_coding	5582.379804	-0.170755077	0.139736081	1.221982723	0.22171416	0.42858336	0.170755077
ENSMUSG00000046318	Ccbe1	320924	protein_coding	4.398483674	0.170767771	1.902813687	-0.089744872	0.928489957	0.965886893	0.170767771
ENSMUSG00000024191	Bnip1	224630	protein_coding	218.323894	-0.170774652	0.096245484	1.774365352	0.076002737	0.202404322	0.170774652
ENSMUSG00000026098	Pms1	227099	protein_coding	61.22562543	0.170795797	0.151912662	-1.124302579	0.260884685	0.474424588	0.170795797
ENSMUSG00000084346	Cyp4a30b	435802	protein_coding	1.160799633	0.170815812	1.636217724	-0.10439675	0.916854502	1	0.170815812
ENSMUSG00000051113	Garin5a	75538	protein_coding	8.502407951	0.17090064	0.363896356	-0.469640978	0.638611543	0.80301103	0.17090064
ENSMUSG00000043154	Ppp2r3a	235542	protein_coding	201.0436454	0.171008971	0.094124972	-1.816828921	0.069243313	0.189412634	0.171008971
ENSMUSG00000023971	Rrp36	224823	protein_coding	296.5645507	-0.171013795	0.103026051	1.659908271	0.096932907	0.24162056	0.171013795
ENSMUSG00000020451	Limk2	16886	protein_coding	1933.541994	-0.17108866	0.094011356	1.819872263	0.06877846	0.188515765	0.17108866
ENSMUSG00000012640	Zfp715	69930	protein_coding	248.6556988	-0.17109319	0.088807972	1.92655216	0.054035466	0.158048473	0.17109319
ENSMUSG00000030154	Klrb1f	232408	protein_coding	1.919863929	-0.171172317	1.066216123	0.160541858	0.872454252	1	0.171172317
ENSMUSG00000037826	Ppm1k	243382	protein_coding	176.9474208	-0.171200662	0.15394875	1.112062693	0.266111196	0.481173797	0.171200662
ENSMUSG00000052915	Msl1	74026	protein_coding	687.8258428	-0.171252102	0.055707142	3.074149836	0.002111034	0.011857691	0.171252102
ENSMUSG00000036890	Gtdc1	227835	protein_coding	53.80791273	-0.171264319	0.149006115	1.14937779	0.250400234	0.462725114	0.171264319
ENSMUSG00000053291	Rab4b	19342	protein_coding	44.92491362	0.171328702	0.201335049	-0.850963126	0.394789836	0.617207172	0.171328702
ENSMUSG00000031906	Smpd3	58994	protein_coding	2464.821688	0.17136173	0.148186864	-1.156389475	0.247521885	0.459691453	0.17136173
ENSMUSG00000026659	Dusp12	80915	protein_coding	61.63445272	0.171532738	0.166851776	-1.028054613	0.303924139	0.524360158	0.171532738
ENSMUSG00000025894	Aasdhppt	67618	protein_coding	207.1647365	-0.171651483	0.094464014	1.817109778	0.069200306	0.189357722	0.171651483
ENSMUSG00000008496	Pou2f2	18987	protein_coding	7.88182033	-0.171683851	1.271015986	0.135076076	0.892551726	0.947202684	0.171683851
ENSMUSG00000037818	Abhd18	269423	protein_coding	102.6564891	-0.171703444	0.139051312	1.234820741	0.216897226	0.42248653	0.171703444
ENSMUSG00000062210	Tnfaip8	106869	protein_coding	429.1067703	-0.171705298	0.186819036	0.919099584	0.358043487	0.580790426	0.171705298
ENSMUSG00000024597	Slc12a2	20496	protein_coding	5013.733157	-0.171719424	0.168251959	1.020608767	0.307439834	0.527870807	0.171719424
ENSMUSG00000041225	Arhgap12	75415	protein_coding	1227.356828	-0.171733077	0.084715335	2.027178152	0.042644197	0.132001174	0.171733077
ENSMUSG00000022772	Senp5	320213	protein_coding	434.6488476	0.171735071	0.070936162	-2.420980576	0.015478704	0.059891125	0.171735071
ENSMUSG00000027536	Chmp4c	66371	protein_coding	2119.13127	-0.171736476	0.067389507	2.548415667	0.010821345	0.045275826	0.171736476
ENSMUSG00000028909	Ptpru	19273	protein_coding	69.21857558	-0.171761272	0.172469566	0.995893222	0.319302029	0.540704851	0.171761272
ENSMUSG00000033253	Szt2	230676	protein_coding	643.4119986	0.171821339	0.13306677	-1.291241523	0.196619941	0.396311693	0.171821339
ENSMUSG00000028708	Mknk1	17346	protein_coding	130.5007595	0.171824492	0.13138629	-1.307780993	0.190947603	0.388417088	0.171824492
ENSMUSG00000033157	Abhd10	213012	protein_coding	116.2981329	0.171858112	0.126844375	-1.354873733	0.17545779	0.36728482	0.171858112
ENSMUSG00000020282	Rhbdf1	13650	protein_coding	305.1985913	-0.171867201	0.162471134	1.057832225	0.290131938	0.509056399	0.171867201
ENSMUSG00000032279	Idh3a	67834	protein_coding	1747.102829	-0.171940485	0.090332934	1.903408617	0.056987248	0.164439526	0.171940485
ENSMUSG00000030541	Idh2	269951	protein_coding	1539.143062	-0.172061091	0.089631924	1.919640717	0.054903297	0.15977566	0.172061091
ENSMUSG00000079363	Gbp4	17472	protein_coding	7.214883495	0.172082969	0.651609091	-0.26408927	0.791711132	0.896267126	0.172082969

ensembl_gene_id	external_gene_name	entrezgene_id	gene_biotype	baseMean	log2FoldChange	lfcSE	stat	pvalue	padj	absLog2FC
ENSMUSG00000085282	Gm15663	327812	lncRNA	21.293689	-0.172102161	0.234630181	0.733503934	0.463251129	0.676807614	0.172102161
ENSMUSG00000069020	Urm1	68205	protein_coding	281.6857773	0.172140047	0.100467913	-1.713383323	0.086642042	0.222728314	0.172140047
ENSMUSG00000025575	Cant1	76025	protein_coding	3681.528215	0.172140087	0.075210636	-2.288773175	0.022092535	0.0793122	0.172140087
ENSMUSG00000033502	Cdc14a	229776	protein_coding	535.68606	-0.172175971	0.105572431	1.630880047	0.102915633	0.252796186	0.172175971
ENSMUSG00000025204	Ndufb8	67264	protein_coding	2001.561507	-0.172178342	0.088985338	1.934906874	0.053001724	0.155964091	0.172178342
ENSMUSG00000087963	Gm25394	115488237	snoRNA	33.30204228	0.172212435	0.247773179	-0.695040663	0.487029819	0.695624496	0.172212435
ENSMUSG00000037395	Rcor3	214742	protein_coding	211.9110384	-0.172223505	0.108110282	1.593035387	0.111152253	0.26676599	0.172223505
ENSMUSG00000064844	Gm26202	115488998	snoRNA	8.735059899	0.172226306	0.36857548	-0.467275537	0.640302756	0.803678477	0.172226306
ENSMUSG00000050854	Tmem125	230678	protein_coding	256.680974	0.172234233	0.110471484	-1.55908319	0.118976692	0.280242631	0.172234233
ENSMUSG00000026869	Psmd5	66998	protein_coding	659.7948577	0.17223714	0.055188689	-3.120877523	0.00180313	0.010407861	0.17223714
ENSMUSG00000038451	Spsb2	14794	protein_coding	174.5364503	-0.172239348	0.118616639	1.452067346	0.14648288	0.32409141	0.172239348
ENSMUSG00000018750	Zbtb4	75580	protein_coding	284.4891581	-0.172246549	0.119821755	1.437523179	0.150569391	0.330520129	0.172246549
ENSMUSG00000049091	Sephs2	20768	protein_coding	754.6929956	-0.172298804	0.082209972	2.095838255	0.036096542	0.11606275	0.172298804
ENSMUSG00000020736	Nt5c	50773	protein_coding	856.4839924	-0.172349181	0.133103642	1.294849475	0.195372161	0.394567184	0.172349181
ENSMUSG00000009828	Cilk1	56542	protein_coding	1327.982695	0.172358345	0.087948077	-1.959773884	0.050022225	0.148948255	0.172358345
ENSMUSG00000034889	Cactin	70312	protein_coding	353.1525777	0.172369045	0.08566289	-2.012178739	0.044201099	0.135701235	0.172369045
ENSMUSG00000046417	Lrrc75a	192976	protein_coding	651.9882889	0.172385865	0.083845277	-2.055999708	0.039782544	0.125530417	0.172385865
ENSMUSG00000020923	Ubtf	21429	protein_coding	1662.608608	0.17239468	0.052891124	-3.259425565	0.001116381	0.0069833	0.17239468
ENSMUSG00000022351	Sqle	20775	protein_coding	1321.294961	0.172412637	0.150028017	-1.149202927	0.250472314	0.462725114	0.172412637
ENSMUSG00000009076	Zmat5	67178	protein_coding	111.8835088	-0.172424996	0.115509578	1.49273332	0.135507008	0.30685209	0.172424996
ENSMUSG00000002221	Paxip1	55982	protein_coding	316.7921165	0.172452817	0.081634205	-2.112506848	0.034642999	0.112617025	0.172452817
ENSMUSG00000043384	Gprasp1	67298	protein_coding	107.6814591	-0.172508263	0.109043962	1.582006561	0.113648073	0.270742105	0.172508263
ENSMUSG00000030180	Kdm5a	214899	protein_coding	823.7274639	-0.172566555	0.058182384	2.965958846	0.003017409	0.015940335	0.172566555
ENSMUSG00000047747	Rnf150	330812	protein_coding	120.4593266	0.172581647	0.168521874	-1.024090482	0.305792539	0.526136486	0.172581647
ENSMUSG00000084946	Dlx1as	111970	lncRNA	2.05402942	0.172653526	0.997350318	-0.173112219	0.862563221	1	0.172653526
ENSMUSG00000032589	Bsn	12217	protein_coding	192.5502294	-0.172689663	0.115466346	1.495584379	0.134761996	0.305611042	0.172689663
ENSMUSG00000036943	Rab8b	235442	protein_coding	153.2613813	0.172700427	0.107015304	-1.613791864	0.10657258	0.259233994	0.172700427
ENSMUSG00000025579	Gaa	14387	protein_coding	541.2897674	-0.172721401	0.099854116	1.729737418	0.0836772	0.216896136	0.172721401
ENSMUSG00000059811	Atl2	56298	protein_coding	1039.986973	0.172760603	0.075080009	-2.301020006	0.0213905	0.077346775	0.172760603
ENSMUSG00000056268	Dennd1b	329260	protein_coding	989.3114626	-0.172816984	0.10269773	1.682773164	0.092419012	0.233151171	0.172816984
ENSMUSG00000022280	Rnf19a	30945	protein_coding	417.8323338	-0.172851916	0.1127859	1.532566708	0.12538265	0.290646979	0.172851916
ENSMUSG00000019505	Ubb	22187	protein_coding	3635.678153	0.172890592	0.088282694	-1.95837468	0.050186064	0.149272747	0.172890592
ENSMUSG00000027506	Tpd52	21985	protein_coding	4673.407281	0.172925983	0.099190481	-1.74337276	0.081268523	0.212151185	0.172925983
ENSMUSG00000030699	Tbx6	21389	protein_coding	12.06950303	-0.172933351	0.491770297	0.351654729	0.725097215	0.857652848	0.172933351
ENSMUSG00000050891	Tatdn1	69694	protein_coding	104.6384511	0.172936226	0.103235219	-1.675166935	0.093901483	0.235970255	0.172936226
ENSMUSG00000003476	Crhr2	12922	protein_coding	2.160563904	0.172974823	1.945892549	-0.088892279	0.92916752	1	0.172974823
ENSMUSG00000053113	Socs3	12702	protein_coding	283.7209276	-0.172990601	0.273391347	0.632758144	0.526891594	0.725332426	0.172990601
ENSMUSG00000048264	Dip2c	208440	protein_coding	239.8391302	-0.172997052	0.075560039	2.289530994	0.022048519	0.079188614	0.172997052
ENSMUSG00000027940	Tpm3	59069	protein_coding	3357.576382	0.173027899	0.093885989	-1.842957621	0.065335198	0.181579513	0.173027899

ensembl_gene_id	external_gene_name	entrezgene_id	gene_biotype	baseMean	log2FoldChange	lfcSE	stat	pvalue	padj	absLog2FC
ENSMUSG00000004328	Hif3a	53417	protein_coding	78.65091293	0.173199069	0.178754884	-0.968919368	0.332585421	0.555003602	0.173199069
ENSMUSG00000033940	Brk1	101314	protein_coding	786.2218737	-0.173205111	0.08538197	2.028591174	0.042499948	0.131725022	0.173205111
ENSMUSG00000036528	Ppfibp2	19024	protein_coding	361.0320146	-0.173324512	0.098381582	1.761757729	0.07811024	0.206650512	0.173324512
ENSMUSG00000033991	Ttc37	218343	protein_coding	511.8681114	0.173333367	0.105196352	-1.647712711	0.099411638	0.246388083	0.173333367
ENSMUSG00000071713	Csf2rb	12983	protein_coding	6.008248577	0.173376172	1.01070774	-0.171539373	0.863799674	0.932373279	0.173376172
ENSMUSG00000048120	Entpd1	12495	protein_coding	4.759266183	-0.173439466	0.756823361	0.229167696	0.81873858	0.908812719	0.173439466
ENSMUSG00000035151	Elmod2	244548	protein_coding	363.1655186	0.173510182	0.090851337	-1.909825303	0.05615571	0.162665392	0.173510182
ENSMUSG00000044155	Lsm8	76522	protein_coding	268.0913019	0.173554796	0.099312487	-1.747562687	0.080539781	0.210915627	0.173554796
ENSMUSG00000031007	Atp6ap2	70495	protein_coding	1766.570917	0.173647109	0.14545848	-1.193791585	0.232559504	0.441638415	0.173647109
ENSMUSG00000027317	Ppp1r14d	72112	protein_coding	1478.858046	0.173665569	0.078563777	-2.210504323	0.027070181	0.092985996	0.173665569
ENSMUSG00000021055	Esr2	13983	protein_coding	18.32718754	-0.173678646	0.240821673	0.721191927	0.470791438	0.683906563	0.173678646
ENSMUSG00000024091	Vapa	30960	protein_coding	1721.675562	0.173684274	0.059942849	-2.897497829	0.003761523	0.01920773	0.173684274
ENSMUSG00000114362	4933433G19Rik	71226	lncRNA	10.43578278	0.173695632	0.35369934	-0.491082714	0.623367947	0.793353766	0.173695632
ENSMUSG00000039382	Wdr45	54636	protein_coding	116.9800346	-0.173725973	0.130420935	1.332040543	0.182846866	0.377583238	0.173725973
ENSMUSG00000028854	Slc9a1	20544	protein_coding	610.5255289	0.173775387	0.15156205	-1.146562658	0.251562417	0.464012259	0.173775387
ENSMUSG00000037722	Gnpnat1	54342	protein_coding	82.27110402	0.173812327	0.189073566	-0.919284122	0.357946981	0.580790426	0.173812327
ENSMUSG00000033096	Apmap	71881	protein_coding	436.6915348	0.173820212	0.106441701	-1.633008579	0.102467192	0.252075246	0.173820212
ENSMUSG00000021510	Zfp729a	212281	protein_coding	168.8757126	-0.173845623	0.110404714	1.574621378	0.115343865	0.27387327	0.173845623
ENSMUSG00000049382	Krt8	16691	protein_coding	59862.67777	-0.173874505	0.125855999	1.381535294	0.167114434	0.355177317	0.173874505
ENSMUSG00000039660	Spout1	227695	protein_coding	214.2834508	0.173887909	0.102846509	-1.690751688	0.090884238	0.230377922	0.173887909
ENSMUSG00000034484	Snx2	67804	protein_coding	779.2736883	-0.17401915	0.078996711	2.202865765	0.027604211	0.09441921	0.17401915
ENSMUSG00000020638	Cmpk2	22169	protein_coding	139.6178052	0.174040396	0.233790292	-0.744429526	0.456616662	0.671509918	0.174040396
ENSMUSG00000030055	Rab43	69834	protein_coding	102.1552357	-0.174077476	0.197793284	0.880098011	0.378806217	0.601173216	0.174077476
ENSMUSG00000097766	5730420D15Rik	70523	lncRNA	5.08579696	0.17409334	0.643017031	-0.270744523	0.786587531	0.893302015	0.17409334
ENSMUSG00000005686	Ampd3	11717	protein_coding	628.5802577	-0.174133955	0.070770184	2.460555348	0.013872217	0.05487085	0.174133955
ENSMUSG00000025234	Arih1	23806	protein_coding	1216.622711	0.174156726	0.04751826	-3.665048464	0.000247292	0.001911596	0.174156726
ENSMUSG00000030616	Sytl2	83671	protein_coding	1121.749075	-0.17417268	0.188891985	0.922075548	0.35648917	0.579378227	0.17417268
ENSMUSG00000007589	Tinf2	28113	protein_coding	243.1878222	0.174223691	0.102064727	-1.706992175	0.087823507	0.224960042	0.174223691
ENSMUSG00000039983	Ccdc32	269336	protein_coding	449.0769836	-0.174238427	0.137381517	1.268281427	0.204697467	0.407126819	0.174238427
ENSMUSG00000026155	Smap1	98366	protein_coding	307.8278901	0.174260439	0.111308657	-1.565560518	0.117451539	0.277481361	0.174260439
ENSMUSG00000043219	Hoxa6	15403	protein_coding	23.88076203	0.174316355	0.24283036	-0.717852394	0.472848304	0.685206817	0.174316355
ENSMUSG00000024176	Sox8	20681	protein_coding	1.275494418	-0.17431704	1.716071656	0.101579115	0.919090758	1	0.17431704
ENSMUSG00000019851	Perp	64058	protein_coding	2729.870833	-0.17433226	0.102104959	1.707382881	0.08775091	0.224907418	0.17433226
ENSMUSG00000074342	I830077J02Rik	433638	protein_coding	1.909749086	0.174367958	1.369073078	-0.127362053	0.898653849	1	0.174367958
ENSMUSG00000035818	Plekhs1	226245	protein_coding	13.21684127	0.174394487	0.371005043	-0.470059614	0.638312427	0.802947244	0.174394487
ENSMUSG00000038486	Sv2a	64051	protein_coding	5.212951589	-0.1744047	1.018340905	0.171263571	0.864016523	0.932456625	0.1744047
ENSMUSG00000052942	Glis3	226075	protein_coding	161.4940293	-0.174481752	0.12956759	1.346646579	0.178094085	0.370548786	0.174481752
ENSMUSG00000001380	Hars	15115	protein_coding	495.6326869	0.174504142	0.084836514	-2.056946161	0.039691405	0.12530374	0.174504142
ENSMUSG00000027288	Zfp106	20402	protein_coding	2039.282062	0.174504857	0.073562149	-2.372209893	0.017682043	0.066621322	0.174504857

ensembl_gene_id	external_gene_name	entrezgene_id	gene_biotype	baseMean	log2FoldChange	lfcSE	stat	pvalue	padj	absLog2FC
ENSMUSG00000019856	Fam184a	75906	protein_coding	2.874458202	-0.174595324	0.800094593	0.218218352	0.827258987	1	0.174595324
ENSMUSG00000038903	Ccdc68	381175	protein_coding	311.4916174	-0.174607454	0.175725769	0.993636022	0.3204001	0.5416756	0.174607454
ENSMUSG00000008475	Arpc5	67771	protein_coding	2619.45069	-0.174626115	0.0953489	1.83144342	0.067034385	0.184994292	0.174626115
ENSMUSG00000035376	Hacd2	70757	protein_coding	202.325567	-0.174645406	0.108771827	1.605612517	0.108359074	0.262189266	0.174645406
ENSMUSG00000064542	Gm22980	115490044	snoRNA	3.830785792	0.174670336	0.573881101	-0.304366768	0.760848483	1	0.174670336
ENSMUSG00000031637	Lrp2bp	67620	protein_coding	5.122365411	-0.174683051	0.835776667	0.209006854	0.834442888	0.916826487	0.174683051
ENSMUSG00000096188	Cmtm4	97487	protein_coding	3384.260396	-0.174697363	0.085045423	2.054165371	0.039959689	0.125813975	0.174697363
ENSMUSG00000049232	Tigd2	68140	protein_coding	168.7531409	-0.17472415	0.108955718	1.603625339	0.108796662	0.263016846	0.17472415
ENSMUSG00000020315	Sptbn1	20742	protein_coding	9189.480466	-0.174854227	0.060696866	2.880778519	0.003966943	0.020064348	0.174854227
ENSMUSG00000091017	Garin4	619288	protein_coding	24.70148789	0.174859788	0.709341185	-0.24651013	0.805287364	0.904107385	0.174859788
ENSMUSG00000021213	Akr1c13	27384	protein_coding	2180.60445	-0.174915562	0.067080407	2.607550713	0.009119256	0.039567892	0.174915562
ENSMUSG00000029610	Aimp2	231872	protein_coding	139.1958718	0.174974742	0.101941942	-1.716415627	0.086085998	0.221678493	0.174974742
ENSMUSG00000030166	Rad52	19365	protein_coding	99.62807433	-0.175027508	0.122773876	1.425608715	0.153981319	0.33564513	0.175027508
ENSMUSG00000039715	Dync2i2	71820	protein_coding	97.84335668	0.175033866	0.126931668	-1.37896137	0.167906665	0.356436387	0.175033866
ENSMUSG00000054161	Fam83e	73813	protein_coding	4718.969309	-0.175057959	0.057945285	3.021090647	0.002518659	0.013704495	0.175057959
ENSMUSG00000031402	Mpp1	17524	protein_coding	269.9622816	-0.175122649	0.141292724	1.239431472	0.215185752	0.420143948	0.175122649
ENSMUSG00000092118	Fancf	100040608	protein_coding	31.0877655	0.175255043	0.246232335	-0.711746661	0.476621667	0.68833522	0.175255043
ENSMUSG00000071646	Mta2	23942	protein_coding	1403.700857	0.175316295	0.06685436	-2.622361438	0.008732277	0.038193919	0.175316295
ENSMUSG00000047641	Krt87	406219	protein_coding	43.40028126	0.175320934	0.184455273	-0.950479382	0.341868725	0.563829035	0.175320934
ENSMUSG00000000355	Mcts1	68995	protein_coding	188.2292551	0.175364306	0.094345287	-1.858750042	0.063062577	0.176840786	0.175364306
ENSMUSG00000024096	Ralbp1	19765	protein_coding	1173.059871	-0.175364417	0.067029018	2.61624623	0.008890242	0.038706422	0.175364417
ENSMUSG00000035215	Lsm7	66094	protein_coding	92.66807274	-0.175438969	0.139067402	1.261539134	0.20711467	0.409951417	0.175438969
ENSMUSG00000044147	Arf6	11845	protein_coding	2538.103893	0.175471746	0.092217963	-1.902793554	0.057067489	0.164593053	0.175471746
ENSMUSG00000026779	Mastl	67121	protein_coding	173.1964412	0.175477532	0.194743903	-0.901068171	0.367552075	0.590046157	0.175477532
ENSMUSG00000069743	Zfp820	75424	protein_coding	79.36585352	-0.175481835	0.130629054	1.343359917	0.179155459	0.372241225	0.175481835
ENSMUSG00000047045	Tmem164	209497	protein_coding	1808.606806	-0.175497181	0.082215237	2.13460652	0.032793164	0.108093077	0.175497181
ENSMUSG00000040287	Stac3	237611	protein_coding	8.352956818	0.17551007	0.55009147	-0.319056157	0.749683929	0.874258149	0.17551007
ENSMUSG00000069184	Zfp72	238722	protein_coding	40.58875079	-0.175520964	0.236215665	0.743053868	0.457449068	0.672242383	0.175520964
ENSMUSG00000024174	Pot1b	72836	protein_coding	57.92408862	-0.17554468	0.140034249	1.253583897	0.209993287	0.413822094	0.17554468
ENSMUSG00000019822	Smpd2	20598	protein_coding	230.9495383	-0.175555228	0.097905695	1.793105376	0.072956076	0.196830035	0.175555228
ENSMUSG00000036835	Psenen	66340	protein_coding	245.6149462	-0.175641356	0.121713298	1.443074501	0.148999485	0.328033263	0.175641356
ENSMUSG00000028467	Gba2	230101	protein_coding	167.5303187	-0.175653476	0.115928616	1.515186515	0.129725177	0.297415185	0.175653476
ENSMUSG00000050989	Selenon	74777	protein_coding	35.40701628	0.175703725	0.231130551	-0.76019256	0.447139492	0.664452584	0.175703725
ENSMUSG00000069835	Sat2	69215	protein_coding	21.08351167	-0.175730876	0.277203709	0.633941285	0.526119141	0.724510632	0.175730876
ENSMUSG00000035473	Galm	319625	protein_coding	674.3127596	-0.175734473	0.058819463	2.987692576	0.002810921	0.015076179	0.175734473
ENSMUSG00000028745	Capzb	12345	protein_coding	2498.718381	0.175741607	0.056278962	-3.122687447	0.001792079	0.010353962	0.175741607
ENSMUSG00000005763	Cd247	12503	protein_coding	6.68586058	-0.175791099	0.452154951	0.38878508	0.697435139	0.839586229	0.175791099
ENSMUSG00000031660	Brd7	26992	protein_coding	426.1900261	-0.175792955	0.069477879	2.530200353	0.011399741	0.0473047	0.175792955
ENSMUSG00000030727	Rabep2	70314	protein_coding	338.1891267	-0.17581703	0.097219867	1.808447547	0.070536873	0.192028673	0.17581703

ensembl_gene_id	external_gene_name	entrezgene_id	gene_biotype	baseMean	log2FoldChange	lfcSE	stat	pvalue	padj	absLog2FC
ENSMUSG00000097267	1700109K24Rik	74303	lncRNA	6.624655469	0.175827774	0.46441947	-0.378596904	0.704987221	0.845227165	0.175827774
ENSMUSG00000074576	Mocs3	69372	protein_coding	122.8511559	0.175842133	0.11339825	-1.550660031	0.120983178	0.283633759	0.175842133
ENSMUSG00000047735	Samd9l	209086	protein_coding	1545.603148	-0.175849829	0.139593481	1.259728085	0.20776747	0.410708835	0.175849829
ENSMUSG00000022305	Lrp12	239393	protein_coding	166.4805285	-0.175887926	0.148420756	1.185062867	0.235992592	0.446089228	0.175887926
ENSMUSG00000027820	Mme	17380	protein_coding	15.65207168	0.175899131	0.757405157	-0.232239151	0.816352271	0.908409285	0.175899131
ENSMUSG00000020516	Rps6kb1	72508	protein_coding	493.8829122	0.175919614	0.07479144	-2.352135692	0.018665964	0.069550933	0.175919614
ENSMUSG00000043162	Pyurf	66459	protein_coding	159.2522166	-0.17599588	0.093701377	1.878263546	0.060345121	0.171466333	0.17599588
ENSMUSG00000030307	Slc6a11	243616	protein_coding	2.04742851	0.176008836	2.687610432	-0.065488969	0.947784689	1	0.176008836
ENSMUSG00000026890	Lhx6	16874	protein_coding	1.882689748	0.176009083	2.697732885	-0.065243332	0.94798026	1	0.176009083
ENSMUSG00000094950	Vmn2r66	233437	protein_coding	1.650431378	0.176009164	2.725787284	-0.064571863	0.948514888	1	0.176009164
ENSMUSG00000086118	Gm14169	100504350	lncRNA	1.407836067	0.176009277	2.802444811	-0.062805618	0.949921293	1	0.176009277
ENSMUSG00000057447	Or4c11c	258898	protein_coding	1.589823595	0.176009793	2.734047731	-0.064377001	0.948670043	1	0.176009793
ENSMUSG00000100459	Gm5833	545363	lncRNA	1.589823595	0.176009793	2.734047731	-0.064377001	0.948670043	1	0.176009793
ENSMUSG00000023433	Cela3b	67868	protein_coding	1.050223206	0.176009995	2.834549745	-0.062094516	0.950487564	1	0.176009995
ENSMUSG00000031441	Atp11a	50770	protein_coding	806.0789567	0.176010103	0.178910495	-0.983788586	0.325219472	0.547052145	0.176010103
ENSMUSG00000087469	Gm16142	102633324	lncRNA	1.585696135	0.176010272	2.711261486	-0.064918221	0.948239113	1	0.176010272
ENSMUSG00000039717	Ralyl	76897	protein_coding	1.451269219	0.176010352	2.739785544	-0.064242383	0.94877723	1	0.176010352
ENSMUSG00000025776	Crispld1	83691	protein_coding	1.494702371	0.176010528	2.719971762	-0.064710425	0.948404563	1	0.176010528
ENSMUSG00000116210	Spn-ps	20738	transcribed_processed_pseudogene	1.494702371	0.176010528	2.719971762	-0.064710425	0.948404563	1	0.176010528
ENSMUSG00000045871	Slitrk6	239250	protein_coding	1.443014299	0.17601055	2.728879874	-0.064499193	0.948572749	1	0.17601055
ENSMUSG00000008658	Rbfox1	268859	protein_coding	1.399581147	0.176010904	2.723944528	-0.064616185	0.948479598	1	0.176010904
ENSMUSG00000073010	Gm5127	331493	protein_coding	1.076674987	0.17601115	2.781198248	-0.063286086	0.949538696	1	0.17601115
ENSMUSG00000053228	Ceacam3	384557	protein_coding	1.26515423	0.176011399	2.739116354	-0.06425846	0.948764429	1	0.176011399
ENSMUSG00000101874	Or2y1e	258460	protein_coding	1.217593618	0.176011683	2.738801253	-0.064265957	0.94875846	1	0.176011683
ENSMUSG00000063881	Or1e1c	258924	protein_coding	1.122472394	0.17601179	2.76309771	-0.063700892	0.949208394	1	0.17601179
ENSMUSG00000059361	Nrsn2	228777	protein_coding	1.126599854	0.176012105	2.749163318	-0.064023881	0.948951212	1	0.176012105
ENSMUSG00000022112	Gpc5	103978	protein_coding	1.079039242	0.176012346	2.753149854	-0.063931263	0.949024959	1	0.176012346
ENSMUSG00000041907	Gpr45	93690	protein_coding	1.079039242	0.176012346	2.753149854	-0.063931263	0.949024959	1	0.176012346
ENSMUSG00000095941	Tle7	102638837	protein_coding	1.079039242	0.176012346	2.753149854	-0.063931263	0.949024959	1	0.176012346
ENSMUSG00000097547	B230110C06Rik	77981	lncRNA	1.589699978	0.176016293	2.415464129	-0.072870589	0.941909098	1	0.176016293
ENSMUSG00000118259	Gm5095	328953	lncRNA	1.276122551	0.176017408	2.415767062	-0.072861912	0.941916003	1	0.176017408
ENSMUSG00000085820	4933406K04Rik	71033	lncRNA	1.672438822	0.176017572	2.329068894	-0.075574223	0.939757845	1	0.176017572
ENSMUSG00000066189	Cacng3	54376	protein_coding	1.451811383	0.176017891	2.339733829	-0.075229878	0.940031812	1	0.176017891
ENSMUSG00000004296	Il12b	16160	protein_coding	1.041341862	0.176018703	2.385074045	-0.0738001	0.941169447	1	0.176018703
ENSMUSG00000109528	Or4f15	258439	protein_coding	1.256775693	0.176018739	2.349692258	-0.074911401	0.940285205	1	0.176018739
ENSMUSG00000015365	Mov10l1	83456	protein_coding	1.449847305	0.176018776	2.298166862	-0.076590947	0.938948961	1	0.176018776
ENSMUSG00000086277	4930558K02Rik	75368	protein_coding	1.038952644	0.176019069	2.358529837	-0.074630843	0.940508434	1	0.176019069
ENSMUSG00000004894	Hapln2	73940	protein_coding	1.084310157	0.176019157	2.367716487	-0.074341315	0.940738803	1	0.176019157
ENSMUSG00000047976	Kcna1	16485	protein_coding	1.169909389	0.176019553	2.330297273	-0.075535235	0.939788864	1	0.176019553

ensembl_gene_id	external_gene_name	entrezgene_id	gene_biotype	baseMean	log2FoldChange	lfcSE	stat	pvalue	padj	absLog2FC
ENSMUSG00000053268	Dspp	666279	protein_coding	1.169909389	0.176019553	2.330297273	-0.075535235	0.939788864	1	0.176019553
ENSMUSG00000100798	Gm19589	100503197	lncRNA	1.112236534	0.176019694	2.340384841	-0.075209722	0.940047849	1	0.176019694
ENSMUSG00000110874	D030045P18Rik	320363	lncRNA	1.395082835	0.176019727	2.262947058	-0.077783405	0.938000348	1	0.176019727
ENSMUSG00000039323	Igfbp2	16008	protein_coding	1.098676867	0.176019762	2.319360953	-0.075891491	0.939505426	1	0.176019762
ENSMUSG00000004344	Gpx5	14780	protein_coding	1.031355012	0.176020317	2.327788968	-0.075616956	0.939723846	1	0.176020317
ENSMUSG00000052417	Or2t6	258387	protein_coding	1.031355012	0.176020317	2.327788968	-0.075616956	0.939723846	1	0.176020317
ENSMUSG00000036218	Pdzrn4	239618	protein_coding	0.9837944	0.176020424	2.336098839	-0.075348021	0.939937815	1	0.176020424
ENSMUSG00000066772	Obox3	246791	protein_coding	0.940191321	0.176021222	2.306916041	-0.076301529	0.939179209	1	0.176021222
ENSMUSG00000062314	Or9i1b	258151	protein_coding	1.410036582	0.17602362	2.019225106	-0.087173847	0.930533327	1	0.17602362
ENSMUSG00000050343	Or1ad6	258912	protein_coding	1.115065698	0.176024266	2.038717679	-0.086340678	0.931195603	1	0.176024266
ENSMUSG00000070460	Or5ae2	258410	protein_coding	1.080687237	0.176024513	2.031040429	-0.086667163	0.930936078	1	0.176024513
ENSMUSG00000035861	Tmprss11b	319875	protein_coding	1.126553544	0.176024601	2.023587208	-0.086986417	0.930682309	1	0.176024601
ENSMUSG00000069441	Dsg1a	13510	protein_coding	1.930068697	0.176025634	1.816178701	-0.096920878	0.922789229	1	0.176025634
ENSMUSG00000012396	Nanog	71950	protein_coding	0.93524569	0.176026289	1.959774512	-0.089819664	0.928430521	1	0.176026289
ENSMUSG00000031872	Bean1	65115	protein_coding	0.74251537	0.176026823	1.949037062	-0.090314764	0.928037088	1	0.176026823
ENSMUSG00000092734	Mir5100	100628625	miRNA	0.706042491	0.176029121	1.75855527	-0.100098714	0.920265956	1	0.176029121
ENSMUSG00000040151	Hs2st1	23908	protein_coding	251.7515126	0.176068944	0.08322577	-2.115558004	0.034382417	0.11194538	0.176068944
ENSMUSG00000011034	Slc5a1	20537	protein_coding	2802.657482	0.176083509	0.066054419	-2.665733985	0.007682045	0.034445098	0.176083509
ENSMUSG00000041958	Pigs	276846	protein_coding	577.6311717	-0.176091603	0.081694357	2.155492851	0.031123287	0.103865546	0.176091603
ENSMUSG00000052534	Pbx1	18514	protein_coding	504.7017705	-0.176102333	0.093205018	1.889408283	0.058837142	0.168423741	0.176102333
ENSMUSG00000056055	Sag	20215	protein_coding	4.432524784	-0.176151823	0.784185124	0.224630406	0.822266808	0.910171686	0.176151823
ENSMUSG00000015542	Nat9	66176	protein_coding	215.3127448	-0.176209193	0.076176809	2.313160585	0.020713812	0.07534535	0.176209193
ENSMUSG00000056888	Glipr1	73690	protein_coding	8.691910961	0.176218212	0.487029086	-0.361822768	0.717484477	0.853563561	0.176218212
ENSMUSG00000109737	Spem3	105243794	protein_coding	2.179110047	-0.176233663	1.939169271	0.090881011	0.927587139	1	0.176233663
ENSMUSG00000026627	Pacc1	66950	protein_coding	63.86419866	-0.176243354	0.136653615	1.289708685	0.197151823	0.397106786	0.176243354
ENSMUSG00000026960	Arl6ip6	65103	protein_coding	187.6321521	0.176281864	0.102328712	-1.722701879	0.08494245	0.219589797	0.176281864
ENSMUSG00000113634	Gm36756	102640764	lncRNA	7.91542274	0.176289553	0.418946771	-0.420792248	0.673906793	0.825096143	0.176289553
ENSMUSG00000041566	Tssk1	22114	protein_coding	5.999294809	-0.176367481	0.750284268	0.235067546	0.814156308	0.907710402	0.176367481
ENSMUSG00000029999	Tgfa	21802	protein_coding	485.9824924	-0.176390707	0.078979372	2.23337693	0.025524098	0.088763622	0.176390707
ENSMUSG00000025609	Mkln1	27418	protein_coding	909.5875918	-0.176537717	0.048250194	3.65879809	0.000253401	0.001953624	0.176537717
ENSMUSG00000040250	Ints13	71177	protein_coding	338.1239509	0.176621724	0.071626576	-2.465868593	0.013668147	0.0542586	0.176621724
ENSMUSG00000020890	Gucy2e	14919	protein_coding	2.712523321	-0.176643049	1.925958506	0.091716955	0.926922926	1	0.176643049
ENSMUSG00000040054	Baz2a	116848	protein_coding	1175.888387	-0.176648288	0.101658593	1.737662132	0.082270365	0.214258446	0.176648288
ENSMUSG00000023979	Guca1b	107477	protein_coding	3.974569906	-0.176707197	0.62150492	0.284321477	0.776164029	1	0.176707197
ENSMUSG00000022721	Trmt2a	15547	protein_coding	334.3868072	0.176715969	0.082390759	-2.144851828	0.031964696	0.106039571	0.176715969
ENSMUSG00000059820	Nkapd1	270156	protein_coding	187.6684085	-0.176817636	0.143393597	1.233092969	0.21754108	0.423388606	0.176817636
ENSMUSG00000007908	Hmgcll1	208982	protein_coding	5.474922205	0.176855391	0.496531792	-0.356181404	0.721704718	0.855825916	0.176855391
ENSMUSG00000115987	Vps28	66914	protein_coding	1209.643518	-0.176871092	0.06002995	2.946380786	0.003215163	0.016858279	0.176871092
ENSMUSG00000032908	Sgpp2	433323	protein_coding	1654.866879	0.176931631	0.057901389	-3.055740716	0.002245053	0.012437037	0.176931631

ensembl_gene_id	external_gene_name	entrezgene_id	gene_biotype	baseMean	log2FoldChange	lfcSE	stat	pvalue	padj	absLog2FC
ENSMUSG00000026259	Ngef	53972	protein_coding	581.7892318	-0.176961836	0.085376581	2.072721056	0.038198247	0.12161285	0.176961836
ENSMUSG00000031604	Msmo1	66234	protein_coding	1324.568999	-0.176986244	0.170933922	1.035407375	0.300478703	0.520523758	0.176986244
ENSMUSG00000025857	Dnaaf5	433956	protein_coding	149.7114021	0.177050252	0.118684135	-1.491776916	0.135757637	0.307109811	0.177050252
ENSMUSG00000020733	Slc9a3r1	26941	protein_coding	2277.753961	0.177061485	0.163569296	-1.082486076	0.279036599	0.496338905	0.177061485
ENSMUSG00000027963	Extl2	58193	protein_coding	107.8183022	0.177165959	0.124944052	-1.417962332	0.156201763	0.338919446	0.177165959
ENSMUSG00000039782	Cpeb2	231207	protein_coding	254.2731588	0.17720516	0.11259324	-1.573852568	0.115521538	0.274255704	0.17720516
ENSMUSG00000055720	Ubl7	69459	protein_coding	242.1836611	-0.177261468	0.096159755	1.843405988	0.065269756	0.18142817	0.177261468
ENSMUSG00000034867	Ankrd27	245886	protein_coding	429.6197494	-0.177370369	0.074619467	2.376998597	0.017454154	0.065958331	0.177370369
ENSMUSG00000037993	Dhx38	64340	protein_coding	647.649623	0.177393589	0.065749695	-2.698013876	0.006975453	0.031812382	0.177393589
ENSMUSG00000066892	Fbxl12	30843	protein_coding	77.113051	0.177416184	0.132289366	-1.341122044	0.179880831	0.37341949	0.177416184
ENSMUSG00000002393	Nr2f6	13864	protein_coding	770.7801627	0.177418388	0.101495268	-1.748045916	0.080456077	0.210863618	0.177418388
ENSMUSG00000028466	Creb3	12913	protein_coding	593.3087554	0.177456754	0.069704091	-2.54585852	0.010900936	0.045517455	0.177456754
ENSMUSG00000040648	Ppip5k2	227399	protein_coding	1272.718426	0.177458431	0.082622943	-2.147810586	0.031728806	0.10540525	0.177458431
ENSMUSG00000030533	Unc45a	101869	protein_coding	612.1239803	-0.177466907	0.081650738	2.173488095	0.029743601	0.100242005	0.177466907
ENSMUSG00000044030	Irf2bp1	272359	protein_coding	524.817632	0.177498107	0.078866795	-2.250606306	0.024410484	0.085787344	0.177498107
ENSMUSG00000025277	Abhd6	66082	protein_coding	530.0203297	0.177519614	0.096352151	-1.842404267	0.065416038	0.181743011	0.177519614
ENSMUSG00000037190	Cyb561d2	56368	protein_coding	110.509105	-0.177587151	0.110119792	1.612672422	0.106815695	0.259672367	0.177587151
ENSMUSG00000020827	Mink1	50932	protein_coding	812.6688476	-0.177741446	0.103614665	1.715408198	0.086270413	0.222084117	0.177741446
ENSMUSG00000068284	Usf3	207806	protein_coding	2385.964938	0.177766438	0.094923319	-1.872737272	0.061104674	0.173114185	0.177766438
ENSMUSG00000067629	Syngap1	240057	protein_coding	14.18256161	0.177793202	0.545599469	-0.325867623	0.744524513	0.871118268	0.177793202
ENSMUSG00000069601	Ank3	11735	protein_coding	1334.653517	-0.177828747	0.165945113	1.071611834	0.283894416	0.502167136	0.177828747
ENSMUSG00000041890	Git2	26431	protein_coding	240.6842119	-0.177893981	0.118749899	1.498055847	0.134118741	0.304528653	0.177893981
ENSMUSG00000030314	Atg7	74244	protein_coding	385.8506075	-0.177924221	0.090561059	1.964687941	0.049450366	0.147805132	0.177924221
ENSMUSG00000052566	Hook2	170833	protein_coding	1074.607049	-0.178026227	0.067377801	2.642208917	0.008236723	0.036437516	0.178026227
ENSMUSG00000034189	Hsdl1	72552	protein_coding	321.0364023	-0.178037972	0.110214533	1.615376551	0.106229175	0.258635202	0.178037972
ENSMUSG00000026698	Pigc	67292	protein_coding	270.8289083	0.178061439	0.10417096	-1.709319368	0.087391808	0.224271688	0.178061439
ENSMUSG00000036046	5031439G07Rik	223739	protein_coding	563.7955456	-0.178071831	0.058061658	3.066943622	0.002162596	0.01206932	0.178071831
ENSMUSG00000006019	Dhx34	71723	protein_coding	191.0802932	-0.17808645	0.098474559	1.808451347	0.070536283	0.192028673	0.17808645
ENSMUSG00000003378	Grik5	14809	protein_coding	42.42152807	-0.178097172	0.256820157	0.69347038	0.488014408	0.696186289	0.178097172
ENSMUSG00000057649	Brd9	105246	protein_coding	442.6543068	0.178158296	0.069785679	-2.552934899	0.010681946	0.044908772	0.178158296
ENSMUSG00000020100	Slc29a3	71279	protein_coding	96.12917847	0.178240058	0.118738325	-1.501116494	0.133325433	0.303102451	0.178240058
ENSMUSG00000030854	Ptpn5	19259	protein_coding	7.035586618	-0.178256312	0.947574355	0.188118549	0.850783721	0.927198731	0.178256312
ENSMUSG00000027784	Ppm1l	242083	protein_coding	169.6123724	-0.178304405	0.11519384	1.547864063	0.121655029	0.284460467	0.178304405
ENSMUSG00000057388	Mrpl18	67681	protein_coding	503.8007814	0.178445449	0.079041874	-2.257606494	0.023970203	0.084509808	0.178445449
ENSMUSG00000039450	Dcxr	67880	protein_coding	646.5563522	-0.178453314	0.113540794	1.571710986	0.116017593	0.274998498	0.178453314
ENSMUSG00000020182	Ddc	13195	protein_coding	823.9024085	-0.178487246	0.128082374	1.393534798	0.16345813	0.349520595	0.178487246
ENSMUSG00000021375	Kif13a	16553	protein_coding	1239.219767	-0.178578875	0.101432623	1.760566473	0.078311807	0.20691859	0.178578875
ENSMUSG00000040714	Klc3	232943	protein_coding	13.50388459	0.178600657	0.281557843	-0.634330249	0.52586532	0.724255209	0.178600657
ENSMUSG00000020770	Unk	217331	protein_coding	222.3298838	0.178602962	0.098175517	-1.819220993	0.068877721	0.188725185	0.178602962

ensembl_gene_id	external_gene_name	entrezgene_id	gene_biotype	baseMean	log2FoldChange	lfcSE	stat	pvalue	padj	absLog2FC
ENSMUSG00000085568	Gm14858	434825	lncRNA	1.276331499	-0.178627024	1.296808544	0.137743559	0.890443092	1	0.178627024
ENSMUSG00000053094	Tmem248	71667	protein_coding	652.640138	0.178727561	0.09310019	-1.919733589	0.054891559	0.15977566	0.178727561
ENSMUSG00000025163	Cd7	12516	protein_coding	49.3415542	0.178760942	0.320312873	-0.558082289	0.576788194	0.761222618	0.178760942
ENSMUSG00000046697	Enpp7	238011	protein_coding	13.01578833	0.1788072	1.175221264	-0.15214769	0.879070451	0.940340033	0.1788072
ENSMUSG00000031453	Rasa3	19414	protein_coding	402.5957482	0.178837624	0.128035113	-1.396785771	0.162478002	0.347924739	0.178837624
ENSMUSG00000036478	Btg1	12226	protein_coding	2195.753303	-0.178855532	0.111236736	1.60788187	0.107861053	0.261367195	0.178855532
ENSMUSG00000043964	Orai3	269999	protein_coding	166.6032916	-0.178856878	0.103534643	1.727507553	0.08407655	0.217760374	0.178856878
ENSMUSG00000023026	Dip2b	239667	protein_coding	618.9014202	-0.178912128	0.089961713	1.988758575	0.046727857	0.141589694	0.178912128
ENSMUSG00000026923	Notch1	18128	protein_coding	497.4295688	0.178926165	0.101032518	-1.770976005	0.076564692	0.203670961	0.178926165
ENSMUSG00000029439	Sfswap	231769	protein_coding	559.6159748	0.179010479	0.115509838	-1.549742277	0.121203387	0.283960728	0.179010479
ENSMUSG00000020817	Rabep1	54189	protein_coding	417.9536927	-0.179059982	0.100821353	1.776012498	0.075730857	0.201777892	0.179059982
ENSMUSG00000022664	Slc35a5	74102	protein_coding	273.0526953	0.179075475	0.091261387	-1.962226105	0.049736166	0.148337194	0.179075475
ENSMUSG00000000088	Cox5a	12858	protein_coding	2338.513058	-0.179095835	0.074500742	2.403946983	0.016219125	0.06221406	0.179095835
ENSMUSG00000011263	Exoc3l2	74463	protein_coding	3.34495287	0.179111933	0.782680426	-0.228844272	0.818989955	1	0.179111933
ENSMUSG00000073565	Prr16	71373	protein_coding	6.019642926	0.179212321	0.496134619	-0.361217125	0.717937141	0.853653903	0.179212321
ENSMUSG00000038160	Atg5	11793	protein_coding	508.4919772	0.179233654	0.078208081	-2.291753627	0.021919864	0.07888094	0.179233654
ENSMUSG00000020069	Hnrnph3	432467	protein_coding	167.0932923	-0.179254643	0.110462775	1.622760643	0.104640582	0.255796319	0.179254643
ENSMUSG00000089832	Shkbp1	192192	protein_coding	288.267888	0.179256691	0.09299245	-1.927647804	0.053898949	0.157816886	0.179256691
ENSMUSG00000030772	Dkk3	50781	protein_coding	1.169304703	0.179285966	1.3915633	-0.128837809	0.897485988	1	0.179285966
ENSMUSG00000028407	Smim27	100504309	protein_coding	52.88781394	-0.17930853	0.139784654	1.282748325	0.199580263	0.400664779	0.17930853
ENSMUSG00000029718	Pcolce	18542	protein_coding	43.02328535	0.179319592	0.242771058	-0.738636615	0.460127684	0.674460666	0.179319592
ENSMUSG00000023923	Tbc1d5	72238	protein_coding	265.4259748	-0.179325013	0.079112366	2.266712803	0.023407776	0.083023198	0.179325013
ENSMUSG00000071649	B3gat3	72727	protein_coding	354.4497509	-0.179335563	0.09890002	1.813301575	0.06978532	0.190611112	0.179335563
ENSMUSG00000027835	Pdcd10	56426	protein_coding	439.9407687	-0.179367932	0.101662218	1.764351946	0.077672743	0.205789777	0.179367932
ENSMUSG00000074781	Ube2n	93765	protein_coding	466.1934498	-0.179387276	0.109822013	1.633436417	0.102377243	0.251954286	0.179387276
ENSMUSG00000020154	Ptprb	19263	protein_coding	7.561740164	-0.17940021	0.801374404	0.22386566	0.822861837	0.910240646	0.17940021
ENSMUSG00000036932	Aifm1	26926	protein_coding	871.0550483	-0.179428751	0.093365406	1.921790506	0.054632123	0.159370233	0.179428751
ENSMUSG00000029370	Rassf6	73246	protein_coding	691.7237114	-0.179450175	0.12106309	1.482286431	0.138264097	0.310948291	0.179450175
ENSMUSG00000027774	Gfm1	28030	protein_coding	719.6378551	-0.179458292	0.08845762	2.028748827	0.04248388	0.13170242	0.179458292
ENSMUSG00000038119	Cdon	57810	protein_coding	297.7249513	0.17951289	0.098631654	-1.820033257	0.068753941	0.188479843	0.17951289
ENSMUSG00000032641	Gpr19	14760	protein_coding	45.98129182	0.179520976	0.208152438	-0.862449546	0.388440186	0.610923926	0.179520976
ENSMUSG00000030592	Ryr1	20190	protein_coding	16.45818891	0.179545151	1.632586463	-0.109975891	0.912428495	0.957266079	0.179545151
ENSMUSG00000028015	Ctso	229445	protein_coding	253.4276358	-0.179568632	0.099585596	1.803158678	0.071363298	0.193640076	0.179568632
ENSMUSG00000046449	Nexmif	245555	protein_coding	32.63272915	-0.179604336	0.309553663	0.580204201	0.561776921	0.750095737	0.179604336
ENSMUSG00000056445	Hoxaas2	105242657	lncRNA	13.63957841	-0.179620915	0.321249853	0.55913151	0.576071972	0.761121093	0.179620915
ENSMUSG00000070047	Fat1	14107	protein_coding	5200.872292	-0.179665939	0.129013605	1.392612343	0.163737048	0.349981053	0.179665939
ENSMUSG00000028463	Car9	230099	protein_coding	581.7876409	-0.179674971	0.066507664	2.701567931	0.006901338	0.031544062	0.179674971
ENSMUSG00000006464	Bbs1	52028	protein_coding	37.38226508	-0.179697031	0.185593987	0.968226575	0.332931225	0.555300096	0.179697031
ENSMUSG00000059921	Unc5c	22253	protein_coding	4.138213019	0.179822962	2.416937868	-0.074401152	0.940691192	1	0.179822962

ensembl_gene_id	external_gene_name	entrezgene_id	gene_biotype	baseMean	log2FoldChange	lfcSE	stat	pvalue	padj	absLog2FC
ENSMUSG00000024246	Thumpd2	72167	protein_coding	33.75852895	-0.179831394	0.213678278	0.841598855	0.40001253	0.622191478	0.179831394
ENSMUSG00000023980	Taf8	63856	protein_coding	236.5783648	0.179860769	0.089983744	-1.998814009	0.045628481	0.13918813	0.179860769
ENSMUSG00000026174	Cnot9	58184	protein_coding	442.4016042	0.179881314	0.091681479	-1.962024566	0.049759624	0.14838036	0.179881314
ENSMUSG00000049516	Spty2d1	101685	protein_coding	605.8779915	0.179886373	0.077761923	-2.313296363	0.020706351	0.075334803	0.179886373
ENSMUSG00000002326	Gmpr2	105446	protein_coding	46.96642887	-0.179953792	0.185009213	0.972674761	0.330714973	0.553055905	0.179953792
ENSMUSG00000084507	Gm25321	115487006	snRNA	1.0140295	-0.179979246	1.22276789	0.147190033	0.882982027	1	0.179979246
ENSMUSG00000001672	Marveld3	73608	protein_coding	707.9073144	-0.180056657	0.082976245	2.169978371	0.030008485	0.100990445	0.180056657
ENSMUSG00000008373	Prpf31	68988	protein_coding	382.5358855	0.180060287	0.085478213	-2.106505061	0.035160504	0.113873419	0.180060287
ENSMUSG00000039182	AW209491	105351	protein_coding	123.9720337	-0.180095485	0.105592142	1.705576586	0.088086942	0.225215631	0.180095485
ENSMUSG00000044734	Serpinb1a	66222	protein_coding	4021.194329	-0.180123596	0.115349254	1.56154972	0.118394102	0.279268705	0.180123596
ENSMUSG00000049755	Zfp672	319475	protein_coding	447.5346529	-0.180139095	0.078105021	2.306370242	0.021089953	0.076528123	0.180139095
ENSMUSG00000031684	Slc10a7	76775	protein_coding	396.4327941	0.180181697	0.082703095	-2.178657246	0.02935714	0.099121614	0.180181697
ENSMUSG00000022708	Zbtb20	56490	protein_coding	246.5632567	-0.180237396	0.156195123	1.153924609	0.248531079	0.460736668	0.180237396
ENSMUSG00000021838	Samd4	74480	protein_coding	8.802200767	0.180267765	0.585204844	-0.308042161	0.758050251	0.878750865	0.180267765
ENSMUSG00000031834	Pik3r2	18709	protein_coding	683.1170899	0.180341867	0.084649701	-2.130448953	0.033134566	0.10878414	0.180341867
ENSMUSG00000020884	Asgr1	11889	protein_coding	2.445511912	-0.180346818	1.055577157	0.170851384	0.864340625	1	0.180346818
ENSMUSG00000060904	Arl1	104303	protein_coding	2655.052022	0.180371935	0.09547337	-1.88923817	0.058859922	0.16845975	0.180371935
ENSMUSG00000024231	Cul2	71745	protein_coding	442.6391054	0.180377797	0.093210033	-1.935175767	0.05296873	0.155922569	0.180377797
ENSMUSG00000062376	Borcs7	66439	protein_coding	64.91878825	-0.180392135	0.168044669	1.073477284	0.283057026	0.501169049	0.180392135
ENSMUSG00000003752	Itpkc	233011	protein_coding	396.2151726	-0.180418862	0.160560022	1.123684835	0.261146752	0.474848873	0.180418862
ENSMUSG00000030423	Pop4	66161	protein_coding	169.8511522	0.18044093	0.113672976	-1.587368749	0.112429153	0.268968865	0.18044093
ENSMUSG00000036377	Cracd	320827	protein_coding	919.1218993	-0.180449318	0.113195888	1.594133158	0.110906214	0.266439513	0.180449318
ENSMUSG00000088254	Gm24289	115487307	scaRNA	3.429383626	0.180451529	0.723449655	-0.249432047	0.803026598	1	0.180451529
ENSMUSG00000021492	F12	58992	protein_coding	1.842344972	0.180456164	1.250173021	-0.144344951	0.885228084	1	0.180456164
ENSMUSG00000034424	Gcsh	68133	protein_coding	459.7913758	0.180474439	0.076134438	-2.370470496	0.017765462	0.066769512	0.180474439
ENSMUSG00000054920	Klhl5	71778	protein_coding	238.9642914	0.180503304	0.146745443	-1.230043675	0.21868075	0.424858107	0.180503304
ENSMUSG00000057751	Megf6	230971	protein_coding	10.81094352	-0.180511378	0.598593874	0.301559013	0.762988257	0.881056435	0.180511378
ENSMUSG00000020903	Stx8	55943	protein_coding	269.723962	-0.18055801	0.085137545	2.12078009	0.033940313	0.11076884	0.18055801
ENSMUSG00000034639	Setmar	74729	protein_coding	41.61054378	-0.180572251	0.211993507	0.851781988	0.394335105	0.616875898	0.180572251
ENSMUSG00000045973	Slc25a51	230125	protein_coding	906.7155624	-0.180649557	0.064610313	2.795986419	0.005174157	0.025072194	0.180649557
ENSMUSG00000047454	Gphn	268566	protein_coding	263.1613846	-0.180659188	0.108056756	1.671891645	0.094545691	0.236959711	0.180659188
ENSMUSG00000025016	Tm9sf3	107358	protein_coding	6794.225974	0.180676347	0.134044552	-1.347882803	0.17769608	0.370029503	0.180676347
ENSMUSG00000026843	Fubp3	320267	protein_coding	526.5798514	0.180704805	0.061674122	-2.929993971	0.003389686	0.017619538	0.180704805
ENSMUSG00000019929	Dcn	13179	protein_coding	4.849207902	-0.180725958	0.716848431	0.252111813	0.800954639	0.901449255	0.180725958
ENSMUSG00000052748	Swt1	66875	protein_coding	252.7922563	-0.180742139	0.080942481	2.232970081	0.025550917	0.08879374	0.180742139
ENSMUSG00000029469	Ift81	12589	protein_coding	48.80113871	-0.180814868	0.173444259	1.042495553	0.297181986	0.516704919	0.180814868
ENSMUSG00000005973	Rcn1	19672	protein_coding	81.38356448	0.180977815	0.146364824	-1.23648436	0.216278576	0.421530084	0.180977815
ENSMUSG00000041840	Haus1	225745	protein_coding	76.01081472	0.18108326	0.145118714	-1.247828449	0.212093888	0.41642117	0.18108326
ENSMUSG00000024617	Camk2a	12322	protein_coding	8.64747986	-0.181152585	0.566986888	0.319500483	0.749347025	0.874044129	0.181152585

ensembl_gene_id	external_gene_name	entrezgene_id	gene_biotype	baseMean	log2FoldChange	lfcSE	stat	pvalue	padj	absLog2FC
ENSMUSG00000026322	Htr4	15562	protein_coding	310.7280628	-0.181221918	0.191822399	0.944738046	0.344792628	0.566362983	0.181221918
ENSMUSG00000076333	Mir599	100316736	miRNA	0.532854639	0.181223808	2.079475567	-0.087148804	0.930553233	1	0.181223808
ENSMUSG00000021265	Slc25a29	214663	protein_coding	56.78914337	0.181243293	0.171616584	-1.056094279	0.290925144	0.509852258	0.181243293
ENSMUSG00000021140	Pcnx	54604	protein_coding	549.1348683	-0.181272851	0.107311735	1.689217409	0.091177774	0.230830869	0.181272851
ENSMUSG00000028837	Psmb2	26445	protein_coding	940.3981802	0.181310364	0.070415277	-2.574872534	0.010027703	0.042679767	0.181310364
ENSMUSG00000018770	Atp5g3	228033	protein_coding	4721.173918	0.181316147	0.083490253	-2.171704378	0.029877968	0.100633238	0.181316147
ENSMUSG00000034801	Sos2	20663	protein_coding	441.716427	-0.181338418	0.082032332	2.210572506	0.027065454	0.092985996	0.181338418
ENSMUSG00000002881	Nab1	17936	protein_coding	700.4551356	-0.181343356	0.101737473	1.782463729	0.074673641	0.199909758	0.181343356
ENSMUSG00000032024	Clmp	71566	protein_coding	62.2060426	0.181351451	0.276385218	-0.656154668	0.511724613	0.714354347	0.181351451
ENSMUSG00000018425	Dhx40	67487	protein_coding	405.7652694	0.181393691	0.095219848	-1.904998744	0.056780234	0.164036653	0.181393691
ENSMUSG00000014503	Pkd2l2	53871	protein_coding	74.42289108	-0.181425422	0.13378887	1.356057654	0.175080825	0.366681641	0.181425422
ENSMUSG00000048175	Asb8	78541	protein_coding	332.4202544	-0.181527179	0.065170937	2.785400807	0.005346159	0.025753241	0.181527179
ENSMUSG00000025860	Xiap	11798	protein_coding	1491.39162	-0.181686392	0.08520091	2.132446608	0.032970149	0.108526787	0.181686392
ENSMUSG00000022419	Deptor	97998	protein_coding	1478.966416	-0.181686584	0.137789308	1.318582598	0.187308694	0.383501006	0.181686584
ENSMUSG00000063317	Usp31	76179	protein_coding	194.499862	0.18168734	0.172119765	-1.05558673	0.291157066	0.510042197	0.18168734
ENSMUSG00000026102	Inpp1	16329	protein_coding	274.099415	0.181690787	0.094979222	-1.912952995	0.05575407	0.161615362	0.181690787
ENSMUSG00000030413	Pglyrp1	21946	protein_coding	1161.635649	-0.181753777	0.082570381	2.201198255	0.027721992	0.094754639	0.181753777
ENSMUSG00000043065	Spice1	212514	protein_coding	158.7177065	-0.181772024	0.093926433	1.935259523	0.052958457	0.15592012	0.181772024
ENSMUSG00000038344	Txlng	353170	protein_coding	230.7691194	-0.181808272	0.076492447	2.376813394	0.01746292	0.065976358	0.181808272
ENSMUSG00000040003	Magi2	50791	protein_coding	7.714947144	-0.181876693	0.638341734	0.284920574	0.775704993	0.88791795	0.181876693
ENSMUSG00000028692	Akr1a1	58810	protein_coding	5507.489697	0.181899603	0.062334999	-2.918097482	0.003521742	0.018197138	0.181899603
ENSMUSG00000040174	Alkbh3	69113	protein_coding	164.1506408	0.181911439	0.092740709	-1.9615058	0.049820049	0.14848011	0.181911439
ENSMUSG00000024592	C330018D20Rik	77422	protein_coding	167.2154287	-0.181911462	0.097188945	1.871729986	0.061243969	0.173330403	0.181911462
ENSMUSG00000017188	Coa3	52469	protein_coding	945.7892459	-0.181947395	0.07399172	2.459023729	0.013931541	0.055052756	0.181947395
ENSMUSG00000051669	AU021092	239691	protein_coding	1.627256968	-0.181971562	1.629419264	0.111678784	0.911078102	1	0.181971562
ENSMUSG00000018481	Appbp2	66884	protein_coding	768.7052454	0.181977521	0.090457685	-2.011741967	0.044247144	0.135792109	0.181977521
ENSMUSG00000034160	Ogt	108155	protein_coding	1068.10919	-0.181980338	0.100548201	1.809881579	0.070314153	0.191611869	0.181980338
ENSMUSG00000033335	Dnm2	13430	protein_coding	2864.847894	0.182026993	0.096016331	-1.89579201	0.057987553	0.166571051	0.182026993
ENSMUSG00000027351	Spred1	114715	protein_coding	456.7733831	0.182070272	0.127180411	-1.431590538	0.152261039	0.332817843	0.182070272
ENSMUSG00000044906	4930503L19Rik	269033	protein_coding	3.550454355	0.182093066	0.657380274	-0.276998069	0.781781589	1	0.182093066
ENSMUSG00000024099	Ndufv2	72900	protein_coding	1733.994081	-0.182096143	0.075129039	2.423778422	0.015359974	0.059503296	0.182096143
ENSMUSG00000020074	Ccar1	67500	protein_coding	878.7161724	0.182135619	0.047667168	-3.820986827	0.000132919	0.001108596	0.182135619
ENSMUSG00000040771	Oard1	106821	protein_coding	279.1676283	0.182139262	0.089789202	-2.028520787	0.042507124	0.131725022	0.182139262
ENSMUSG00000061887	Ssbp3	72475	protein_coding	901.4786314	0.182139263	0.098230255	-1.854207366	0.063709499	0.178321807	0.182139263
ENSMUSG00000027243	Harbi1	241547	protein_coding	151.0920785	0.182170754	0.115041124	-1.583527249	0.113301341	0.270127107	0.182170754
ENSMUSG00000078676	Casc3	192160	protein_coding	465.3674467	-0.18220477	0.052446734	3.474091853	0.000512586	0.003624129	0.18220477
ENSMUSG00000026317	Cln8	26889	protein_coding	207.7092447	0.182325815	0.080608133	-2.261878653	0.023704902	0.083878883	0.182325815
ENSMUSG00000026109	Tmeff2	56363	protein_coding	3.742430174	-0.182360696	0.996661534	0.18297154	0.854820349	1	0.182360696
ENSMUSG00000042208	Sanbr	71675	protein_coding	270.0824699	0.182438581	0.097245821	-1.87605574	0.060647626	0.172144191	0.182438581

ensembl_gene_id	external_gene_name	entrezgene_id	gene_biotype	baseMean	log2FoldChange	lfcSE	stat	pvalue	padj	absLog2FC
ENSMUSG00000028465	Tln1	21894	protein_coding	2012.792541	0.182438884	0.066643987	-2.737514561	0.006190538	0.028864834	0.182438884
ENSMUSG00000114245	Percc1	102643076	protein_coding	1.227521128	-0.182488975	0.99527473	0.183355378	0.854519185	1	0.182488975
ENSMUSG00000091537	Tma7	66167	protein_coding	681.0476596	-0.182515858	0.100212394	1.82129026	0.068562746	0.188104702	0.182515858
ENSMUSG00000000439	Mkrn2	67027	protein_coding	193.1944081	-0.182567695	0.085816311	2.127424175	0.033384855	0.109497021	0.182567695
ENSMUSG00000013698	Pea15a	18611	protein_coding	170.1746743	-0.18263175	0.090192694	2.024906254	0.042876991	0.132607819	0.18263175
ENSMUSG00000087861	Gm24440	115488686	snRNA	0.91515639	0.182675591	1.347566906	-0.13555957	0.892169469	1	0.182675591
ENSMUSG00000055436	Srsf11	69207	protein_coding	914.306711	0.182698537	0.063797759	-2.863714012	0.004187059	0.020927689	0.182698537
ENSMUSG00000024388	Myo7b	17922	protein_coding	2929.274068	-0.18276335	0.161970662	1.128373175	0.259162352	0.472489189	0.18276335
ENSMUSG00000054717	Hmgb2	97165	protein_coding	939.3975758	0.182776853	0.152218724	-1.20075145	0.22984763	0.438404222	0.182776853
ENSMUSG00000036503	Rnf13	24017	protein_coding	491.9927761	-0.1828391	0.093913847	1.946881172	0.051548982	0.152668561	0.1828391
ENSMUSG00000008090	Fgfrl1	116701	protein_coding	234.1431804	0.182856205	0.13582576	-1.34625571	0.178220064	0.37057407	0.182856205
ENSMUSG00000039206	Daglb	231871	protein_coding	603.2413283	0.182872779	0.133455865	-1.37028656	0.170597467	0.360481579	0.182872779
ENSMUSG00000018395	Kif3a	16568	protein_coding	93.52070602	0.182899557	0.13212344	-1.384308162	0.166264116	0.35368873	0.182899557
ENSMUSG00000002981	Clptm1	56457	protein_coding	2254.661046	-0.182915722	0.063640536	2.874201475	0.004050505	0.020374669	0.182915722
ENSMUSG00000020910	Adprm	66358	protein_coding	130.3003949	-0.182920612	0.115642275	1.581779772	0.113699854	0.270826395	0.182920612
ENSMUSG00000063626	Unc5d	210801	protein_coding	2.717044933	-0.182922171	1.586952384	0.115266326	0.908234028	1	0.182922171
ENSMUSG00000030168	Adipor2	68465	protein_coding	2421.492345	-0.182937003	0.074438022	2.457574733	0.01398787	0.055222494	0.182937003
ENSMUSG00000027935	Rab13	68328	protein_coding	109.1300243	-0.182980407	0.101094931	1.809985977	0.070297962	0.191599363	0.182980407
ENSMUSG00000042472	Zfp410	52708	protein_coding	351.0208031	0.182990857	0.071203589	-2.569966772	0.010170827	0.043155457	0.182990857
ENSMUSG00000046991	Wdr27	71682	protein_coding	14.43255473	-0.183024963	0.394040478	0.464482644	0.642301991	0.804778079	0.183024963
ENSMUSG00000016554	Eif3d	55944	protein_coding	945.4496071	0.183095674	0.08874507	-2.063164449	0.039097007	0.123758475	0.183095674
ENSMUSG00000027170	Eif3m	98221	protein_coding	875.4602911	-0.183104839	0.117682474	1.555922749	0.119726463	0.281607009	0.183104839
ENSMUSG00000086682	Gm16023	100502734	lncRNA	18.64020479	-0.183190557	0.256609243	0.713889161	0.47529572	0.687185565	0.183190557
ENSMUSG00000027977	Ndst3	83398	protein_coding	2.301911773	0.183203815	3.05671988	-0.059934774	0.952207584	1	0.183203815
ENSMUSG00000040112	Mrps35	232536	protein_coding	534.0297774	-0.183206939	0.081738483	2.241379231	0.025001521	0.087325531	0.183206939
ENSMUSG00000026004	Kansl1l	68691	protein_coding	127.0605047	-0.183283717	0.130440796	1.405110386	0.159988456	0.344285818	0.183283717
ENSMUSG00000025236	Adpgk	72141	protein_coding	207.0232119	0.183322101	0.101134874	-1.812649723	0.069885864	0.190791065	0.183322101
ENSMUSG00000037263	Aldh3b3	73458	protein_coding	3.420348808	-0.183329782	0.767923681	0.238734378	0.811311558	1	0.183329782
ENSMUSG00000029326	Enoph1	67870	protein_coding	366.0294546	-0.183332984	0.089076157	2.058160005	0.039574776	0.125007242	0.183332984
ENSMUSG00000029784	Ssmem1	75647	protein_coding	1.137673775	0.183333759	1.343011946	-0.136509403	0.891418591	1	0.183333759
ENSMUSG00000040007	Bahd1	228536	protein_coding	428.4519866	-0.183361829	0.067806555	2.704190299	0.006847106	0.031346224	0.183361829
ENSMUSG00000006169	Clint1	216705	protein_coding	3157.748375	-0.183368135	0.04585679	3.998712857	6.37E-05	0.000580752	0.183368135
ENSMUSG00000034758	Tle6	114606	protein_coding	53.9456524	-0.183413905	0.200046537	0.916856187	0.359218005	0.581866222	0.183413905
ENSMUSG00000029056	Pank4	269614	protein_coding	252.4608979	-0.183495459	0.08749781	2.097143439	0.03598088	0.115758474	0.183495459
ENSMUSG00000042712	Tceal9	22381	protein_coding	918.7143962	0.183540381	0.132064701	-1.38977622	0.164596841	0.351137093	0.183540381
ENSMUSG00000045665	Mfsd5	106073	protein_coding	270.658823	0.183575234	0.081084545	-2.263997838	0.023574248	0.083506033	0.183575234
ENSMUSG00000022386	Trmu	72026	protein_coding	133.1568378	-0.183610443	0.105626589	1.738297573	0.082158394	0.214101896	0.183610443
ENSMUSG00000026999	Nup35	69482	protein_coding	61.32594081	-0.183613766	0.186949342	0.982157859	0.326022085	0.548038346	0.183613766
ENSMUSG00000004394	Tmed4	103694	protein_coding	772.9101958	0.18362889	0.07971689	-2.303512966	0.021249998	0.076990451	0.18362889

ensembl_gene_id	external_gene_name	entrezgene_id	gene_biotype	baseMean	log2FoldChange	lfcSE	stat	pvalue	padj	absLog2FC
ENSMUSG00000021248	Tmed10	68581	protein_coding	1760.584824	0.183718112	0.100197214	-1.83356507	0.066718581	0.184376923	0.183718112
ENSMUSG00000017667	Zfp334	228876	protein_coding	48.04106586	0.183720105	0.174252135	-1.054334886	0.291729622	0.510557756	0.183720105
ENSMUSG00000020604	Arsg	74008	protein_coding	74.29437661	-0.183733766	0.160082468	1.147744458	0.251074072	0.463422066	0.183733766
ENSMUSG00000057101	Zfp180	210135	protein_coding	361.6833312	-0.183733857	0.075276007	2.440802391	0.014654671	0.057293381	0.183733857
ENSMUSG00000097251	5033417F24Rik	75995	lncRNA	7.891876247	-0.183758554	0.373857624	0.491520145	0.623058608	0.793281643	0.183758554
ENSMUSG00000041775	Mapk1ip1	69546	protein_coding	77.61654925	-0.183758932	0.145328057	1.264442227	0.206071345	0.408658758	0.183758932
ENSMUSG00000042320	Prox2	73422	protein_coding	11.99104817	-0.183759201	0.38025108	0.48325754	0.628912884	0.797440677	0.183759201
ENSMUSG00000024910	Ctsw	13041	protein_coding	27.82554063	0.183914416	0.364872434	-0.504051275	0.614225346	0.78673795	0.183914416
ENSMUSG00000022408	Fam83f	213956	protein_coding	416.4902136	0.183937295	0.070493794	-2.609269324	0.00907358	0.039390404	0.183937295
ENSMUSG00000001630	Stk38l	232533	protein_coding	769.8535882	-0.183941685	0.137573416	1.337043811	0.181208337	0.375235922	0.183941685
ENSMUSG00000025745	Hadha	97212	protein_coding	3844.630925	-0.183964884	0.062466902	2.944997724	0.00322957	0.016920408	0.183964884
ENSMUSG00000031465	Angpt2	11601	protein_coding	24.28725615	-0.184020724	0.248026717	0.74193912	0.458124223	0.672740084	0.184020724
ENSMUSG00000025359	Pmel	20431	protein_coding	32.6022608	-0.184035397	0.200225063	0.919142657	0.35802096	0.580790426	0.184035397
ENSMUSG00000036934	4921524J17Rik	66714	protein_coding	62.61869877	-0.184059112	0.137045335	1.343052736	0.179254898	0.37240098	0.184059112
ENSMUSG00000112654	4930455C13Rik	73990	lncRNA	3.587447036	-0.184100068	0.829299757	0.2219946	0.824318087	1	0.184100068
ENSMUSG00000045962	Wnk1	232341	protein_coding	1151.013674	0.184155949	0.111369839	-1.653553162	0.098218336	0.244200181	0.184155949
ENSMUSG00000022508	Bcl6	12053	protein_coding	66.67620448	-0.184164335	0.180110425	1.022507915	0.306540569	0.527079777	0.184164335
ENSMUSG00000037999	Arap2	212285	protein_coding	1180.123084	-0.184181658	0.099890881	1.843828539	0.065208132	0.181293038	0.184181658
ENSMUSG00000020620	Abca8b	27404	protein_coding	7.568748282	-0.184256913	0.754741789	0.244132385	0.807128292	0.904987729	0.184256913
ENSMUSG00000003541	Ier3	15937	protein_coding	209.1625551	0.184308874	0.235923337	-0.78122358	0.434671007	0.652915864	0.184308874
ENSMUSG00000029507	Pus1	56361	protein_coding	296.4712922	-0.184321559	0.123543182	1.491960598	0.135709475	0.30705241	0.184321559
ENSMUSG00000032380	Dapk2	13143	protein_coding	431.5321326	-0.184321716	0.071082365	2.593072349	0.009512276	0.040902139	0.184321716
ENSMUSG00000078919	Dpm1	13480	protein_coding	46.37977861	-0.184326674	0.16390169	1.124617289	0.260751244	0.474234146	0.184326674
ENSMUSG00000015488	Cacfd1	381356	protein_coding	406.3464197	0.184340247	0.078855763	-2.337688958	0.019403389	0.071716108	0.184340247
ENSMUSG00000041417	Pik3r1	18708	protein_coding	1878.950061	-0.184474289	0.059238869	3.114075136	0.001845225	0.010614744	0.184474289
ENSMUSG00000024642	Tle4	21888	protein_coding	786.2792254	-0.184505952	0.129525427	1.424476691	0.154308528	0.336218865	0.184505952
ENSMUSG00000029475	Kdm2b	30841	protein_coding	182.0046918	-0.184530422	0.092405265	1.996968691	0.045828584	0.139530465	0.184530422
ENSMUSG00000026447	Pik3c2b	240752	protein_coding	1164.099362	-0.184539354	0.113638993	1.623908738	0.104395287	0.255480702	0.184539354
ENSMUSG00000053070	Cfap300	234912	protein_coding	12.2928017	-0.184569957	0.301442122	0.612289868	0.540345992	0.734651013	0.184569957
ENSMUSG00000060678	H4c3	319155	protein_coding	2895.116891	0.18458529	0.113282319	-1.629427184	0.103222618	0.253324166	0.18458529
ENSMUSG00000026395	Ptprc	19264	protein_coding	142.514823	-0.184586818	0.177520574	1.039805212	0.298430407	0.518220794	0.184586818
ENSMUSG00000031875	Cmtm3	68119	protein_coding	9.840673046	-0.184614238	0.456052163	0.404809478	0.685617561	0.832521206	0.184614238
ENSMUSG00000024948	Map4k2	26412	protein_coding	135.571432	0.184618634	0.151670444	-1.217235404	0.223514663	0.430702585	0.184618634
ENSMUSG00000026348	Acmsd	266645	protein_coding	1.401756215	0.184639395	1.251163208	-0.147574188	0.882678827	1	0.184639395
ENSMUSG00000098286	Mir8091	102465884	miRNA	4.49435316	-0.184648679	0.524737761	0.351887538	0.724922605	0.857548131	0.184648679
ENSMUSG00000026269	Rnpepl1	108657	protein_coding	1077.073099	-0.184672665	0.060648875	3.044947888	0.002327206	0.012806226	0.184672665
ENSMUSG00000002679	Med6	69792	protein_coding	168.411221	0.184705161	0.109788652	-1.682370249	0.092497067	0.233277344	0.184705161
ENSMUSG00000001687	Ubl3	24109	protein_coding	1267.387206	-0.184735152	0.046314395	3.988719976	6.64E-05	0.000602437	0.184735152
ENSMUSG00000041119	Pde9a	18585	protein_coding	1788.703457	0.184802742	0.131580433	-1.404484974	0.160174483	0.344596328	0.184802742

Supplementary Table 1

ensembl_gene_id	external_gene_name	entrezgene_id	gene_biotype	baseMean	log2FoldChange	lfcSE	stat	pvalue	padj	absLog2FC
ENSMUSG00000117869	Snhg4	100503380	lncRNA	135.0134672	-0.18483802	0.382936166	0.482686244	0.629318532	0.79746811	0.18483802
ENSMUSG00000041716	Gm20604	100859931	protein_coding	49.8223088	0.184880188	0.156901581	-1.178319475	0.238669267	0.448874722	0.184880188
ENSMUSG00000026928	Card9	332579	protein_coding	3.633058164	-0.184908364	0.700085901	0.264122393	0.791685609	1	0.184908364
ENSMUSG00000031832	Taf1c	21341	protein_coding	208.4102031	0.184940097	0.075360982	-2.454056359	0.014125484	0.055659328	0.184940097
ENSMUSG00000037253	Mex3c	240396	protein_coding	685.2613902	0.18494024	0.068725817	-2.690986399	0.00712411	0.032409794	0.18494024
ENSMUSG00000004561	Mettl17	52535	protein_coding	196.8463147	-0.1849698	0.091315319	2.025616324	0.042804117	0.132446542	0.1849698
ENSMUSG00000020680	Taf15	70439	protein_coding	320.6616031	0.185038753	0.07877424	-2.348975402	0.01882515	0.070049242	0.185038753
ENSMUSG00000065399	Mir133a-1	387151	miRNA	0.557688373	-0.185079915	1.419747568	0.130361143	0.896280708	1	0.185079915
ENSMUSG00000090112	Shprh	268281	protein_coding	372.7825435	-0.185108346	0.080758148	2.292132121	0.02189802	0.078853883	0.185108346
ENSMUSG00000048732	Klhl11	217194	protein_coding	143.4568342	0.185147526	0.098748015	-1.874949335	0.060799694	0.172486883	0.185147526
ENSMUSG00000032698	Lmo2	16909	protein_coding	8.289993688	-0.185189524	0.423241925	0.437550048	0.661712489	0.816921981	0.185189524
ENSMUSG00000086914	Gm16124	102636154	lncRNA	5.016178277	-0.185302015	0.468544773	0.395484116	0.692485642	0.836513744	0.185302015
ENSMUSG00000060733	Ipmk	69718	protein_coding	2554.942005	-0.185342078	0.103222253	1.795563199	0.072564013	0.196079582	0.185342078
ENSMUSG00000044986	Tst	22117	protein_coding	2241.347036	-0.185352884	0.095115974	1.948704042	0.051330779	0.152129476	0.185352884
ENSMUSG00000030981	Mmp21	214766	protein_coding	2.821747315	0.18535423	0.712242278	-0.260240421	0.794678327	1	0.18535423
ENSMUSG00000028042	Zbtb7b	22724	protein_coding	3334.971975	0.185366685	0.10744199	-1.725272257	0.084478419	0.218595521	0.185366685
ENSMUSG00000029556	Hnf1a	21405	protein_coding	335.3414884	0.185506413	0.149424711	-1.241470782	0.214431885	0.419407924	0.185506413
ENSMUSG00000026405	C4bp	12269	protein_coding	98.58385734	-0.185603197	0.294387209	0.630473036	0.528385139	0.726359126	0.185603197
ENSMUSG00000010608	Rbm25	67039	protein_coding	1048.274841	0.185623132	0.066817884	-2.77804567	0.005468693	0.026243183	0.185623132
ENSMUSG00000060131	Atp8b4	241633	protein_coding	4.59029099	0.185715921	1.260224836	-0.147367292	0.882842121	0.942116626	0.185715921
ENSMUSG00000072647	Adam1a	280668	protein_coding	8.458561603	-0.185731514	0.427321219	0.43464145	0.663822703	0.818698142	0.185731514
ENSMUSG00000031862	Atp13a1	170759	protein_coding	1056.698855	0.185763476	0.059313999	-3.131865665	0.001736993	0.010082498	0.185763476
ENSMUSG00000026816	Gtf3c5	70239	protein_coding	256.8159073	0.185785782	0.087930839	-2.112862611	0.034612529	0.112562288	0.185785782
ENSMUSG00000025223	Ldb1	16825	protein_coding	405.1099837	-0.185808453	0.091940428	2.020965723	0.043283314	0.133504042	0.185808453
ENSMUSG00000020747	Tmem94	71947	protein_coding	1790.974787	-0.185816154	0.083624258	2.2220365	0.026280839	0.090782827	0.185816154
ENSMUSG00000002413	Braf	109880	protein_coding	384.4369258	0.185841084	0.105243009	-1.765828303	0.077424659	0.205363124	0.185841084
ENSMUSG00000032667	Pon2	330260	protein_coding	1995.336025	-0.185996689	0.081730077	2.275743462	0.022861368	0.081487727	0.185996689
ENSMUSG00000018733	Pex12	103737	protein_coding	89.96585362	0.18601982	0.116840128	-1.592088464	0.111364829	0.267113839	0.18601982
ENSMUSG00000021190	Lgmn	19141	protein_coding	394.593738	-0.186028111	0.152727478	1.218039572	0.223208935	0.430314308	0.186028111
ENSMUSG00000042055	Wdr11	207425	protein_coding	623.1331384	-0.186120503	0.08679278	2.14442381	0.031998944	0.106094619	0.186120503
ENSMUSG00000019087	Atp6ap1	54411	protein_coding	1134.369132	-0.186121318	0.073357592	2.53717869	0.01117499	0.046543198	0.186121318
ENSMUSG00000110444	Gm10033	378466	protein_coding	70.23930316	-0.186121636	0.147235167	1.264111283	0.206190087	0.408815356	0.186121636
ENSMUSG00000031816	Mthfsd	234814	protein_coding	247.5170257	0.186123325	0.086823949	-2.143686475	0.032058017	0.106242441	0.186123325
ENSMUSG00000023830	Igf2r	16004	protein_coding	2570.864696	-0.186127996	0.121249915	1.535077337	0.124764832	0.289621372	0.186127996
ENSMUSG00000055707	Klhl26	234378	protein_coding	164.0265144	-0.186132205	0.097769619	1.903783672	0.056938364	0.164355907	0.186132205
ENSMUSG00000036905	C1qb	12260	protein_coding	2.699318208	-0.186175041	0.669580984	0.278047085	0.780976215	1	0.186175041
ENSMUSG00000021737	Psmd6	66413	protein_coding	675.7804153	0.186264494	0.084358088	-2.208021753	0.027242757	0.093415051	0.186264494
ENSMUSG00000039501	Znfx1	98999	protein_coding	778.126088	-0.18629565	0.112694952	1.65309667	0.098311191	0.244320693	0.18629565
ENSMUSG00000029512	Ulk1	22241	protein_coding	256.1727649	-0.186322652	0.104019913	1.79122099	0.073257839	0.197386189	0.186322652

Supplementary Table 1

ensembl_gene_id	external_gene_name	entrezgene_id	gene_biotype	baseMean	log2FoldChange	lfcSE	stat	pvalue	padj	absLog2FC
ENSMUSG00000075376	Rc3h2	319817	protein_coding	840.6026989	0.186479356	0.068126745	-2.73724153	0.006195679	0.028870044	0.186479356
ENSMUSG00000078317	F8a	14070	protein_coding	216.0620502	0.186490528	0.107300215	-1.738025672	0.08220629	0.214159123	0.186490528
ENSMUSG00000083307	AA414768	245350	processed_pseudogene	14.36870771	-0.186502172	0.304135912	0.613219829	0.539730995	0.734194205	0.186502172
ENSMUSG00000109644	0610005C13Rik	71661	lncRNA	209.945329	0.186539086	0.230519809	-0.809210657	0.418393986	0.63839585	0.186539086
ENSMUSG00000027487	Cdk5rap1	66971	protein_coding	191.4592255	-0.186539481	0.260062569	0.717286927	0.473197072	0.685335967	0.186539481
ENSMUSG00000040006	Ginm1	215751	protein_coding	682.17493	-0.186622981	0.084750011	2.202040787	0.027662427	0.094598742	0.186622981
ENSMUSG00000039191	Rbpj	19664	protein_coding	211.9850773	-0.186624033	0.109032289	1.711640052	0.086963023	0.223449098	0.186624033
ENSMUSG00000029777	Gars	353172	protein_coding	2084.438496	0.186627435	0.067810204	-2.752202847	0.005919584	0.027890446	0.186627435
ENSMUSG00000041852	Tcf20	21411	protein_coding	2493.85197	-0.186628557	0.039288299	4.750232498	2.03E-06	2.65E-05	0.186628557
ENSMUSG00000022191	Drosha	14000	protein_coding	546.3963208	0.186696205	0.059470331	-3.139316736	0.001693423	0.009870396	0.186696205
ENSMUSG00000062382	Ftl1-ps1	434624	protein_coding	5.32222936	-0.186720451	0.572247057	0.326293423	0.744202364	0.870990647	0.186720451
ENSMUSG00000019806	Aig1	66253	protein_coding	614.0977316	-0.186724873	0.075101043	2.486315319	0.012907354	0.051861813	0.186724873
ENSMUSG00000038500	Prr3	75210	protein_coding	33.11220749	-0.186756041	0.198417452	0.941227897	0.346588083	0.56826406	0.186756041
ENSMUSG00000066900	Suds3	71954	protein_coding	1170.516927	-0.186783791	0.059536129	3.137318382	0.001705009	0.009910776	0.186783791
ENSMUSG00000027708	Dcun1d1	114893	protein_coding	381.3928971	-0.186902974	0.090311168	2.069544415	0.038495028	0.122282111	0.186902974
ENSMUSG00000001964	Emd	13726	protein_coding	272.3423534	0.186977924	0.095416675	-1.959593798	0.050043287	0.148984107	0.186977924
ENSMUSG00000050846	Zfp623	78834	protein_coding	187.0898312	-0.18700087	0.12317254	1.51820259	0.128963335	0.296522686	0.18700087
ENSMUSG00000028556	Dock7	67299	protein_coding	829.6434261	0.187003865	0.054277561	-3.445325531	0.000570372	0.003959279	0.187003865
ENSMUSG00000045948	Mrps12	24030	protein_coding	389.3644304	0.187004908	0.107978153	-1.731877258	0.083295419	0.216245959	0.187004908
ENSMUSG00000050945	Zfp438	240186	protein_coding	36.74386659	-0.187064701	0.208071271	0.899041467	0.368630574	0.591140542	0.187064701
ENSMUSG00000070000	Fcho1	74015	protein_coding	76.40664514	-0.187071256	0.219748081	0.851298701	0.394603446	0.617091033	0.187071256
ENSMUSG00000109771	Gm35315	102638847	protein_coding	22.3020258	0.18709842	0.322706232	-0.579779383	0.562063402	0.750140815	0.18709842
ENSMUSG00000037953	A4gnt	333424	protein_coding	173.2133307	-0.187126369	0.234121605	0.799269971	0.424133887	0.643957486	0.187126369
ENSMUSG00000015745	Plekho1	67220	protein_coding	16.23690695	0.187157055	0.283269502	-0.66070316	0.508802697	0.712306522	0.187157055
ENSMUSG00000027263	Tubgcp4	51885	protein_coding	321.4791305	-0.187165731	0.082015212	2.282085568	0.022484289	0.080560978	0.187165731
ENSMUSG00000020015	Cdk17	237459	protein_coding	156.3773392	-0.187181625	0.149875806	1.24891155	0.211697427	0.416065952	0.187181625
ENSMUSG00000007739	Cct4	12464	protein_coding	1730.542364	-0.187230009	0.090398411	2.071164831	0.038343395	0.12191044	0.187230009
ENSMUSG00000058886	Deaf1	54006	protein_coding	228.0635256	-0.187243465	0.109424005	1.711173573	0.087049076	0.223580206	0.187243465
ENSMUSG00000010797	Wnt2	22413	protein_coding	0.968317744	-0.187247226	2.272048037	0.08241341	0.934317973	1	0.187247226
ENSMUSG00000030730	Atp2a1	11937	protein_coding	6.891946206	0.187247558	0.668897827	-0.279934469	0.779527782	0.890072723	0.187247558
ENSMUSG00000014418	Hps5	246694	protein_coding	846.0389007	0.18727808	0.079765922	-2.347845742	0.01888234	0.070214581	0.18727808
ENSMUSG00000029290	Zfp326	54367	protein_coding	281.1072066	0.187318491	0.075080575	-2.494899542	0.012599279	0.050983705	0.187318491
ENSMUSG00000063334	Krr1	52705	protein_coding	344.0576366	0.187393365	0.105015073	-1.784442553	0.074351782	0.19938276	0.187393365
ENSMUSG00000037860	Aim2	383619	protein_coding	534.0264468	0.187437247	0.086667047	-2.162727971	0.030562108	0.102353003	0.187437247
ENSMUSG00000022514	Il1rap	16180	protein_coding	116.7767418	0.187461559	0.141965758	-1.32047024	0.186678066	0.382529046	0.187461559
ENSMUSG00000054676	1600014C10Rik	72244	protein_coding	114.3424494	0.187466507	0.116649527	-1.607091869	0.108034217	0.261594876	0.187466507
ENSMUSG00000036764	Dnajc12	30045	protein_coding	47.30278606	-0.187471245	0.177764844	1.054602476	0.291607171	0.510451645	0.187471245
ENSMUSG00000092103	Gm17039	102636903	lncRNA	4.362966152	-0.187484117	0.533535746	0.351399355	0.725288767	1	0.187484117
ENSMUSG00000035441	Myo1d	338367	protein_coding	4271.874136	0.187488349	0.063911373	-2.93356783	0.003350904	0.017451579	0.187488349

ensembl_gene_id	external_gene_name	entrezgene_id	gene_biotype	baseMean	log2FoldChange	lfcSE	stat	pvalue	padj	absLog2FC
ENSMUSG00000031778	Cx3cl1	20312	protein_coding	948.5127061	-0.187521295	0.079321854	2.36405587	0.018076086	0.067704352	0.187521295
ENSMUSG00000029430	Ran	19384	protein_coding	1403.930419	0.187539409	0.144939164	-1.293918112	0.195693708	0.394975053	0.187539409
ENSMUSG00000045868	Gvin1	74558	protein_coding	2.851248343	-0.187554569	0.848886321	0.220941915	0.82513766	1	0.187554569
ENSMUSG00000056592	Zfp658	210104	protein_coding	30.75279394	-0.187578712	0.206176921	0.909794906	0.362930682	0.585225632	0.187578712
ENSMUSG00000022312	Eif3h	68135	protein_coding	1724.331813	-0.187589884	0.090510133	2.072584331	0.038210981	0.121629941	0.187589884
ENSMUSG00000056228	Cars2	71941	protein_coding	259.8898063	0.187606873	0.070357102	-2.666495165	0.007664671	0.03440456	0.187606873
ENSMUSG00000097431	Gm26782	102640338	lncRNA	23.93572516	-0.187607246	0.208968872	0.897776037	0.369304963	0.591883639	0.187607246
ENSMUSG00000032834	Pwp2	110816	protein_coding	193.3288858	-0.187656143	0.110684252	1.695418636	0.089996033	0.228807636	0.187656143
ENSMUSG00000020235	Fzr1	56371	protein_coding	668.375646	0.187680457	0.06633203	-2.829409206	0.004663403	0.022933722	0.187680457
ENSMUSG00000038415	Foxq1	15220	protein_coding	44.28055085	-0.187766252	0.305065087	0.615495708	0.538227403	0.733377497	0.187766252
ENSMUSG00000004791	Pgf	18654	protein_coding	1.058952313	-0.187806878	1.782718398	0.105348594	0.916099205	1	0.187806878
ENSMUSG00000017485	Top2b	21974	protein_coding	1621.776954	-0.18782445	0.102655648	1.829655294	0.0673015	0.185483473	0.18782445
ENSMUSG00000026944	Abca2	11305	protein_coding	116.5822681	-0.18788942	0.102307219	1.836521625	0.066280547	0.1835581	0.18788942
ENSMUSG00000025873	Faf2	76577	protein_coding	898.1574473	0.187928364	0.081644846	-2.301778659	0.021347658	0.077259526	0.187928364
ENSMUSG00000015133	Lrrk1	233328	protein_coding	669.2644442	0.187937915	0.067558597	-2.781850488	0.005404993	0.025977317	0.187937915
ENSMUSG00000001995	Sipa1l2	244668	protein_coding	308.4540463	0.187938501	0.074714199	-2.515432169	0.011888655	0.048729637	0.187938501
ENSMUSG00000030357	Fkbp4	14228	protein_coding	3688.442181	0.18793986	0.117240869	-1.603023429	0.108929482	0.26324771	0.18793986
ENSMUSG00000032303	Chrna3	110834	protein_coding	2.478116299	0.188036499	1.30002003	-0.144641232	0.884994141	1	0.188036499
ENSMUSG00000022526	Zfp251	71591	protein_coding	133.4246832	-0.188040799	0.122298025	1.537562017	0.124155741	0.288572541	0.188040799
ENSMUSG00000035919	Bbs9	319845	protein_coding	59.77272503	0.188041025	0.141033784	-1.333304825	0.182431791	0.377007156	0.188041025
ENSMUSG00000074238	Ap1ar	211556	protein_coding	1105.642848	-0.188062766	0.07303533	2.574956068	0.010025282	0.042679767	0.188062766
ENSMUSG00000005043	Sgsh	27029	protein_coding	180.1868489	-0.188114615	0.134231279	1.401421608	0.161088034	0.345901869	0.188114615
ENSMUSG00000014773	Dll1	13388	protein_coding	154.8938433	-0.188128956	0.156771565	1.200019637	0.230131714	0.438743376	0.188128956
ENSMUSG00000001864	Aif1l	108897	protein_coding	1.638956795	0.188246416	1.521612894	-0.123715051	0.901540894	1	0.188246416
ENSMUSG00000046658	Zfp316	54201	protein_coding	124.7983666	-0.188254171	0.124042656	1.517656723	0.129100959	0.296643	0.188254171
ENSMUSG00000063838	Cdc42ep5	58804	protein_coding	1538.642253	0.188353623	0.120617997	-1.561571475	0.118388974	0.279268705	0.188353623
ENSMUSG00000039219	Arid4b	94246	protein_coding	605.0346967	-0.188426651	0.08131068	2.317366555	0.020483773	0.074722558	0.188426651
ENSMUSG00000042569	Dhrs7b	216820	protein_coding	515.7550502	-0.188452186	0.066246496	2.844711755	0.004445163	0.022069064	0.188452186
ENSMUSG00000028804	Csmd2	329942	protein_coding	7.580897611	0.188477542	2.293762919	-0.082169583	0.934511861	0.969075596	0.188477542
ENSMUSG00000031561	Tenm3	23965	protein_coding	5.592257253	-0.188518534	1.010978684	0.186471325	0.852075164	0.927743965	0.188518534
ENSMUSG00000036968	Cnpy4	66455	protein_coding	130.8694483	-0.18857758	0.120150191	1.569515448	0.116527883	0.275891248	0.18857758
ENSMUSG00000059534	Uqcr10	66152	protein_coding	2293.749251	-0.188600188	0.089702442	2.10250896	0.035508716	0.114725166	0.188600188
ENSMUSG00000030199	Etv6	14011	protein_coding	469.2241528	0.188609481	0.095531113	-1.974325175	0.04834478	0.145350463	0.188609481
ENSMUSG00000024697	Gna14	14675	protein_coding	1063.559601	0.188691319	0.190579342	-0.990093247	0.322128544	0.54337393	0.188691319
ENSMUSG00000015165	Hnrnpl	15388	protein_coding	2599.261171	0.188758882	0.054735807	-3.448544805	0.000563616	0.003917321	0.188758882
ENSMUSG00000034459	Ifit1	15957	protein_coding	217.7121121	0.188761527	0.403239312	-0.468112907	0.63970385	0.803534599	0.188761527
ENSMUSG00000033416	Gucd1	68778	protein_coding	1917.921619	-0.188828874	0.089519094	2.109369812	0.034912673	0.113248455	0.188828874
ENSMUSG00000047648	Fbxo30	71865	protein_coding	177.5646882	-0.188832511	0.121186864	1.558192894	0.119187529	0.280659184	0.188832511
ENSMUSG00000067610	Klri1	503550	protein_coding	0.857917052	0.188851216	1.192080529	-0.158421526	0.874124653	1	0.188851216

ensembl_gene_id	external_gene_name	entrezgene_id	gene_biotype	baseMean	log2FoldChange	lfcSE	stat	pvalue	padj	absLog2FC
ENSMUSG00000059493	Nhs	195727	protein_coding	18.62653022	-0.188858939	0.337158463	0.560148889	0.575377886	0.760569151	0.188858939
ENSMUSG00000033793	Atp6v1h	108664	protein_coding	609.229472	-0.188864273	0.116457955	1.621737841	0.104859493	0.256047562	0.188864273
ENSMUSG00000034640	Tiparp	99929	protein_coding	255.2296017	-0.188942668	0.1341922	1.408000372	0.15913096	0.343290486	0.188942668
ENSMUSG00000031729	Ist1	71955	protein_coding	1570.242977	-0.188976946	0.054249065	3.483506052	0.000494892	0.003519658	0.188976946
ENSMUSG00000022831	Hcls1	15163	protein_coding	19.4352479	-0.188991813	0.359969267	0.525022078	0.599567843	0.776544903	0.188991813
ENSMUSG00000034656	Cacna1a	12286	protein_coding	82.03336297	-0.189037328	0.219563935	0.860967117	0.389256154	0.611857618	0.189037328
ENSMUSG00000039103	Nexn	68810	protein_coding	13.66460469	0.189045893	0.309094166	-0.611612622	0.540794085	0.734913884	0.189045893
ENSMUSG00000056952	Tatdn2	381801	protein_coding	723.4105804	0.189079595	0.063360235	-2.984199698	0.002843211	0.015214774	0.189079595
ENSMUSG00000019979	Apaf1	11783	protein_coding	559.676502	0.189144672	0.10192296	-1.855761171	0.063487607	0.177826758	0.189144672
ENSMUSG00000029625	Cpsf4	54188	protein_coding	194.3935628	0.189225734	0.09124196	-2.073889409	0.038089583	0.121313669	0.189225734
ENSMUSG00000020402	Vdac1	22333	protein_coding	3343.433747	-0.189324535	0.082259117	2.30156294	0.021359832	0.077273602	0.189324535
ENSMUSG00000002083	Bbc3	170770	protein_coding	90.00110663	-0.189342567	0.216493564	0.874587509	0.381798387	0.604102573	0.189342567
ENSMUSG00000000682	Cd52	23833	protein_coding	21.02868964	-0.189347919	0.366581148	0.516523886	0.605488561	0.780450414	0.189347919
ENSMUSG00000045731	Pnoc	18155	protein_coding	1.042114838	0.189415146	1.804789568	-0.104951375	0.916414393	1	0.189415146
ENSMUSG00000070533	Wfdc8	277343	protein_coding	2.080660926	-0.189469803	1.148962646	0.164905103	0.869018676	1	0.189469803
ENSMUSG00000029998	Pcyox1	66881	protein_coding	946.4617921	-0.189487827	0.094179799	2.011979523	0.044222096	0.135740462	0.189487827
ENSMUSG00000058446	Znrf2	387524	protein_coding	962.0838925	-0.189580124	0.070595156	2.685455139	0.00724311	0.032806559	0.189580124
ENSMUSG00000002728	Naa20	67877	protein_coding	433.3014968	-0.189585912	0.083379775	2.273763777	0.022980193	0.081770074	0.189585912
ENSMUSG00000086541	Has2os	594843	lncRNA	7.161959022	-0.189594184	0.42309136	0.448116417	0.65406918	0.81231093	0.189594184
ENSMUSG00000054499	Dedd2	67379	protein_coding	570.1075613	-0.189698026	0.153526528	1.235604222	0.216605715	0.422068056	0.189698026
ENSMUSG00000020823	Sec14l1	74136	protein_coding	415.1477947	-0.189703806	0.090406793	2.098335758	0.035875496	0.115554504	0.189703806
ENSMUSG00000048280	Zfp738	408068	protein_coding	135.0589432	-0.189716893	0.098379829	1.928412505	0.053803838	0.157622241	0.189716893
ENSMUSG00000005534	Insr	16337	protein_coding	1538.706535	-0.189775812	0.074044684	2.562990349	0.010377493	0.043863303	0.189775812
ENSMUSG00000024045	Akap8	56399	protein_coding	871.8097951	0.189804282	0.049813337	-3.810310494	0.000138792	0.001150611	0.189804282
ENSMUSG00000033579	Fa2h	338521	protein_coding	1092.551901	0.189889826	0.210573025	-0.9017766	0.367175553	0.589852079	0.189889826
ENSMUSG00000059474	Mbtd1	103537	protein_coding	280.7835957	-0.189937069	0.082216839	2.310196674	0.020877268	0.075856371	0.189937069
ENSMUSG00000031171	Ftsj1	54632	protein_coding	166.0142181	0.189937435	0.093514517	-2.031101048	0.042244745	0.131182723	0.189937435
ENSMUSG00000022843	Clcn2	12724	protein_coding	3105.045655	-0.189943672	0.171857371	1.105240179	0.269055531	0.484112338	0.189943672
ENSMUSG00000029710	Ephb4	13846	protein_coding	1331.420506	-0.189944455	0.10182123	1.865470043	0.062115547	0.17492772	0.189944455
ENSMUSG00000010054	Tusc2	80385	protein_coding	423.6079197	0.189947267	0.082266444	-2.308927633	0.020947597	0.07607843	0.189947267
ENSMUSG00000045316	Fahd1	68636	protein_coding	539.5082848	-0.190106298	0.106299873	1.788396292	0.073712094	0.198286614	0.190106298
ENSMUSG00000022110	Sucla2	20916	protein_coding	1141.389884	-0.190163996	0.069266211	2.745407812	0.006043574	0.028321899	0.190163996
ENSMUSG00000020744	Slc25a19	67283	protein_coding	202.6596348	0.190168097	0.092154772	-2.063572973	0.039058222	0.12365941	0.190168097
ENSMUSG00000015697	Setdb1	84505	protein_coding	468.7945429	-0.19019734	0.084449308	2.252207203	0.024309181	0.08552233	0.19019734
ENSMUSG00000024665	Fads2	56473	protein_coding	2791.062787	-0.190202777	0.229778332	0.827766376	0.407802821	0.628507412	0.190202777
ENSMUSG00000024007	Ppil1	68816	protein_coding	351.6669474	0.190254354	0.128927436	-1.47567003	0.140032495	0.313984606	0.190254354
ENSMUSG00000017428	Psmd11	69077	protein_coding	606.4387418	0.1902565	0.073091408	-2.602994046	0.009241356	0.039961181	0.1902565
ENSMUSG00000073433	Arhgdig	14570	protein_coding	14.42961814	-0.190309147	0.269473816	0.706225005	0.480048223	0.691115487	0.190309147
ENSMUSG00000002332	Dhrs1	52585	protein_coding	370.8718201	-0.190312078	0.090498963	2.102919981	0.035472765	0.114659863	0.190312078

ensembl_gene_id	external_gene_name	entrezgene_id	gene_biotype	baseMean	log2FoldChange	lfcSE	stat	pvalue	padj	absLog2FC
ENSMUSG00000043668	Tox3	244579	protein_coding	189.0585882	-0.190359934	0.088635164	2.14767959	0.031739218	0.105418633	0.190359934
ENSMUSG00000061894	Zscan20	269585	protein_coding	93.18035012	-0.190382776	0.120212052	1.583724534	0.113256419	0.270127107	0.190382776
ENSMUSG00000094018	S100a2	628324	protein_coding	3.870576199	0.190383578	1.669073507	-0.114065425	0.90918593	1	0.190383578
ENSMUSG00000022614	Lmf2	105847	protein_coding	242.494835	0.190394311	0.102009294	-1.866440825	0.061979716	0.174694151	0.190394311
ENSMUSG00000084128	Esrp2	77411	protein_coding	884.4901349	-0.19040145	0.065175005	2.921387594	0.00348476	0.018045571	0.19040145
ENSMUSG00000052819	Best2	212989	protein_coding	341.6619233	0.190428732	0.515698106	-0.369263975	0.711930975	0.850157504	0.190428732
ENSMUSG00000036009	Mettl25	216292	protein_coding	58.43224581	0.190499946	0.160684409	-1.185553393	0.235798717	0.445943657	0.190499946
ENSMUSG00000036875	Dna2	327762	protein_coding	72.24268012	0.190515312	0.18348814	-1.03829769	0.299131484	0.51911069	0.190515312
ENSMUSG00000085637	1700048M11Rik	100503166	lncRNA	1.216277845	-0.190532002	1.056903872	0.180273728	0.856937679	1	0.190532002
ENSMUSG00000013921	Clip3	76686	protein_coding	4.350059724	0.190537368	1.03876095	-0.183427542	0.854462567	1	0.190537368
ENSMUSG00000046101	Mcmdc2	240697	protein_coding	4.659237809	-0.190560289	0.745644402	0.255564567	0.798287081	0.899929685	0.190560289
ENSMUSG00000001755	Coasy	71743	protein_coding	394.6218828	-0.190562338	0.100243494	1.900994577	0.057302723	0.165147848	0.190562338
ENSMUSG00000052920	Prkg1	19091	protein_coding	3.586128684	-0.190699139	1.230130667	0.155023482	0.876802805	1	0.190699139
ENSMUSG00000020777	Acox1	11430	protein_coding	3744.49281	-0.190702452	0.125620882	1.518079237	0.128994425	0.296522686	0.190702452
ENSMUSG00000026594	Ralgps2	78255	protein_coding	744.9811165	0.190710583	0.053488712	-3.565436085	0.000363252	0.002700918	0.190710583
ENSMUSG00000028282	Casp8ap2	26885	protein_coding	350.3451636	0.190731283	0.077917975	-2.447847028	0.014371266	0.056385621	0.190731283
ENSMUSG00000025781	Atp5c1	11949	protein_coding	4427.719366	-0.190761343	0.092095713	2.071337918	0.038327228	0.12190868	0.190761343
ENSMUSG00000020641	Rsad2	58185	protein_coding	87.61965689	-0.190771432	0.330945787	0.576443149	0.564315686	0.751419871	0.190771432
ENSMUSG00000025815	Dhtkd1	209692	protein_coding	3.520276031	-0.190784915	1.291852297	0.147683227	0.88259277	1	0.190784915
ENSMUSG00000020183	Cpm	70574	protein_coding	638.5859354	-0.190815389	0.119530264	1.596377205	0.110404602	0.265659566	0.190815389
ENSMUSG00000031986	Sprtn	244666	protein_coding	221.3593752	-0.190867477	0.090715188	2.104030011	0.035375829	0.114480981	0.190867477
ENSMUSG00000021114	Atp6v1d	73834	protein_coding	438.6902015	0.190984481	0.063100362	-3.026678048	0.002472572	0.013498197	0.190984481
ENSMUSG00000025477	Inpp5a	212111	protein_coding	400.2037013	-0.191026144	0.089868572	2.125616766	0.033535182	0.109773894	0.191026144
ENSMUSG00000097534	Gm16675	100503498	lncRNA	16.92282244	-0.191090598	0.281441487	0.678970965	0.497156259	0.702914252	0.191090598
ENSMUSG00000004270	Lpcat3	14792	protein_coding	897.3946128	0.191096024	0.071953325	-2.655833111	0.007911276	0.035329044	0.191096024
ENSMUSG00000016128	Stard13	243362	protein_coding	352.2275489	0.191110107	0.071181961	-2.684810944	0.007257084	0.032851834	0.191110107
ENSMUSG00000028057	Rit1	19769	protein_coding	397.7155226	-0.191267786	0.066263168	2.88648718	0.003895686	0.019776625	0.191267786
ENSMUSG00000006678	Pola1	18968	protein_coding	255.8829336	0.191282757	0.176984891	-1.0807858	0.279792406	0.49722072	0.191282757
ENSMUSG00000016477	E2f3	13557	protein_coding	224.892631	0.191383164	0.090278825	-2.119911986	0.034013468	0.110941817	0.191383164
ENSMUSG00000001823	Hoxd12	15432	protein_coding	10.22649461	-0.191406363	0.481016998	0.397920165	0.690689035	0.835178936	0.191406363
ENSMUSG00000000085	Scmh1	29871	protein_coding	298.5073388	0.191411215	0.075486479	-2.535701994	0.011222219	0.046704567	0.191411215
ENSMUSG00000050122	Vwa3b	70853	protein_coding	26.85601329	-0.191426123	0.310639202	0.616232986	0.537740761	0.733113936	0.191426123
ENSMUSG00000036986	Pml	18854	protein_coding	372.188564	-0.191435803	0.10572552	1.810686789	0.070189349	0.191366503	0.191435803
ENSMUSG00000032872	Cyb5r4	266690	protein_coding	748.5624766	-0.191438113	0.071238893	2.68726963	0.007203878	0.032709605	0.191438113
ENSMUSG00000030282	Cmas	12764	protein_coding	2483.538607	0.191449857	0.119857228	-1.597315915	0.110195305	0.265233241	0.191449857
ENSMUSG00000031295	Phka2	110094	protein_coding	197.6154133	0.191468665	0.108348921	-1.767148786	0.077203314	0.204907671	0.191468665
ENSMUSG00000024873	Cnih2	12794	protein_coding	13.44018519	0.191557042	0.319808978	-0.59897331	0.549190683	0.740233019	0.191557042
ENSMUSG00000073490	Ifi207	226691	protein_coding	1.292580119	0.191583003	1.476612139	-0.129744974	0.896768199	1	0.191583003
ENSMUSG00000097333	Zfp87	170763	protein_coding	104.7743611	-0.191598915	0.14563136	1.315643245	0.188293812	0.384741896	0.191598915

ensembl_gene_id	external_gene_name	entrezgene_id	gene_biotype	baseMean	log2FoldChange	lfcSE	stat	pvalue	padj	absLog2FC
ENSMUSG00000028917	Plekhm2	69582	protein_coding	238.7318635	-0.191657862	0.097175387	1.972288141	0.048576723	0.145821162	0.191657862
ENSMUSG00000035674	Ndufa3	66091	protein_coding	1093.480778	-0.191737499	0.071628408	2.676835982	0.007432101	0.033524642	0.191737499
ENSMUSG00000032207	Lipc	15450	protein_coding	6.567225643	0.191752716	0.528274303	-0.362979451	0.716620235	0.852920113	0.191752716
ENSMUSG00000051331	Cacna1c	12288	protein_coding	39.91222212	0.191757959	0.343298723	-0.558574636	0.576452054	0.761222618	0.191757959
ENSMUSG00000037174	Elf2	69257	protein_coding	706.4038061	-0.19177291	0.069792962	2.747739967	0.006000758	0.028168424	0.19177291
ENSMUSG00000030149	Klrk1	27007	protein_coding	5.244775589	-0.19180404	0.759847258	0.252424468	0.80071299	0.901300137	0.19180404
ENSMUSG00000028974	Dffa	13347	protein_coding	264.6029564	-0.191814187	0.075501326	2.540540637	0.011068123	0.046167968	0.191814187
ENSMUSG00000049971	Glt1d1	319804	protein_coding	210.6982253	-0.191863439	0.135244176	1.418644745	0.156002614	0.338620816	0.191863439
ENSMUSG00000021687	Scamp1	107767	protein_coding	1374.936727	-0.191938781	0.165419145	1.16031782	0.245919432	0.457693395	0.191938781
ENSMUSG00000033885	Pxk	218699	protein_coding	624.4310511	-0.191946335	0.070040153	2.7405185	0.006134233	0.028656498	0.191946335
ENSMUSG00000053768	Chchd3	66075	protein_coding	1832.004477	-0.191972113	0.054459614	3.525036238	0.000423425	0.003084446	0.191972113
ENSMUSG00000028064	Sema4a	20351	protein_coding	2099.128769	0.191979675	0.113214693	-1.695713424	0.089940165	0.228714488	0.191979675
ENSMUSG00000008604	Ubqln4	94232	protein_coding	562.8310387	-0.192071241	0.066626594	2.882801452	0.003941558	0.01996653	0.192071241
ENSMUSG00000038025	Phf2	18676	protein_coding	384.9751733	0.192078361	0.077027209	-2.493642997	0.012643964	0.051106386	0.192078361
ENSMUSG00000118379	Gm41790	105246509	lncRNA	1.497901698	-0.192081739	2.353914626	0.081600979	0.934964023	1	0.192081739
ENSMUSG00000025583	Rptor	74370	protein_coding	660.771738	0.192110089	0.076564349	-2.509132407	0.012102811	0.049435028	0.192110089
ENSMUSG00000034422	Parp14	547253	protein_coding	759.6205408	-0.192124859	0.187009877	1.027351401	0.304255027	0.524504007	0.192124859
ENSMUSG00000043923	Ccdc84	382073	protein_coding	119.4097834	0.192134521	0.150943734	-1.272888354	0.203057662	0.405231932	0.192134521
ENSMUSG00000104986	1600023N17Rik	69788	TEC	5.529444828	0.192147661	0.606386796	-0.316873095	0.751339898	0.875081807	0.192147661
ENSMUSG00000062168	Ppef1	237178	protein_coding	1.923626493	0.192174345	1.086906263	-0.176808573	0.859658755	1	0.192174345
ENSMUSG00000047507	Baiap3	545192	protein_coding	35.65977367	-0.192223821	0.225162165	0.853712798	0.393264141	0.615929821	0.192223821
ENSMUSG00000047656	Trpt1	107328	protein_coding	87.904714	-0.192234852	0.13199558	1.456373407	0.145289423	0.3224445	0.192234852
ENSMUSG00000020646	Mboat2	67216	protein_coding	105.7056611	0.192281998	0.195518895	-0.983444586	0.325388676	0.547140677	0.192281998
ENSMUSG00000065738	Gm24494	115489817	snoRNA	7.866320771	-0.192317492	0.366473279	0.52477903	0.599736811	0.776544903	0.192317492
ENSMUSG00000018595	Glra4	14657	protein_coding	1.553709676	0.192332033	1.169090136	-0.16451429	0.869326298	1	0.192332033
ENSMUSG00000047227	Gm527	217648	protein_coding	16.81453139	-0.192365418	0.290794053	0.661517715	0.508280357	0.71205818	0.192365418
ENSMUSG00000021108	Prkch	18755	protein_coding	120.2662438	0.192365602	0.142588648	-1.349094792	0.177306521	0.36956202	0.192365602
ENSMUSG00000052488	Cherp	27967	protein_coding	199.4633712	0.192384422	0.150886998	-1.275023194	0.202301032	0.404218578	0.192384422
ENSMUSG00000029632	Ndufa4	17992	protein_coding	1763.759497	0.192418305	0.078860644	-2.439978867	0.014688121	0.0573834	0.192418305
ENSMUSG00000031783	Polr2c	20021	protein_coding	398.5322317	-0.192422867	0.078224365	2.459884053	0.013898191	0.054947264	0.192422867
ENSMUSG00000032065	Tex12	66654	protein_coding	17.48556049	-0.192424504	0.336386056	0.572034723	0.567298451	0.753386941	0.192424504
ENSMUSG00000030603	Psmc4	23996	protein_coding	999.4915012	0.192471022	0.064116898	-3.001876707	0.002683208	0.014480556	0.192471022
ENSMUSG00000034117	Ptgdr2	14764	protein_coding	1.88932073	0.1925089	0.924521621	-0.208225417	0.835052964	1	0.1925089
ENSMUSG00000030157	Clec2d	93694	protein_coding	1093.154734	-0.192533784	0.166777723	1.154433461	0.248322503	0.460660281	0.192533784
ENSMUSG00000019143	Hars2	70791	protein_coding	284.2598119	-0.192603783	0.087082921	2.211728552	0.026985426	0.092763808	0.192603783
ENSMUSG00000033032	Afap1l1	106877	protein_coding	183.5250928	0.192613384	0.104605161	-1.841337302	0.065572145	0.1820236	0.192613384
ENSMUSG00000003644	Rps6ka1	20111	protein_coding	3461.438561	0.192634951	0.059175914	-3.255293226	0.001132753	0.007066978	0.192634951
ENSMUSG00000049721	Gal3st1	53897	protein_coding	569.4297558	-0.192647031	0.106308589	1.812149267	0.069963136	0.190901365	0.192647031
ENSMUSG00000021177	Tdp1	104884	protein_coding	64.55294037	0.192686022	0.164379743	-1.172200532	0.241116555	0.451758428	0.192686022

Supplementary Table 1

ensembl_gene_id	external_gene_name	entrezgene_id	gene_biotype	baseMean	log2FoldChange	lfcSE	stat	pvalue	padj	absLog2FC
ENSMUSG00000026495	Efcab2	68226	protein_coding	38.94513098	-0.192699685	0.195728122	0.984527326	0.324856302	0.546769252	0.192699685
ENSMUSG00000022594	Lynx1	23936	protein_coding	104.1813425	-0.192730023	0.154117127	1.250542535	0.211101427	0.415328565	0.192730023
ENSMUSG00000027184	Caprin1	53872	protein_coding	4318.415462	0.192759987	0.072497283	-2.658858102	0.007840598	0.035051335	0.192759987
ENSMUSG00000047181	Samd14	217125	protein_coding	29.98517964	-0.192843225	0.296143699	0.651181255	0.514929489	0.716272317	0.192843225
ENSMUSG00000005621	Zfp592	233410	protein_coding	529.6606298	-0.192936324	0.072996022	2.64310738	0.008214898	0.036352985	0.192936324
ENSMUSG00000042359	Osbpl6	99031	protein_coding	167.2964436	-0.192960778	0.110690831	1.743240853	0.081291552	0.212177761	0.192960778
ENSMUSG00000104301	Wdr49	213248	protein_coding	1.8590389	0.192962241	1.602042445	-0.120447646	0.90412855	1	0.192962241
ENSMUSG00000042498	Radx	102871	protein_coding	62.27479171	-0.193004684	0.168189794	1.147540995	0.2511581	0.463479905	0.193004684
ENSMUSG00000025006	Sorbs1	20411	protein_coding	209.8932557	0.193045869	0.265413004	-0.727341411	0.467016836	0.680659303	0.193045869
ENSMUSG00000018820	Zfyve27	319740	protein_coding	366.1668607	-0.193054713	0.060598969	3.185775544	0.001443666	0.008606748	0.193054713
ENSMUSG00000022228	Zscan26	432731	protein_coding	414.0563171	-0.193072506	0.084277391	2.290916975	0.021968216	0.079002436	0.193072506
ENSMUSG00000024208	Uqcc2	67267	protein_coding	348.4102897	-0.193097982	0.103341817	1.868536736	0.061687295	0.174107671	0.193097982
ENSMUSG00000018486	Wnt9b	22412	protein_coding	1.359347322	-0.193115726	2.346964842	0.082283178	0.934421531	1	0.193115726
ENSMUSG00000040653	Ppp1r14c	76142	protein_coding	18.84796126	-0.193120914	0.291138909	0.663329111	0.507119796	0.710854454	0.193120914
ENSMUSG00000078619	Smarcd2	83796	protein_coding	1069.429312	-0.193132644	0.081406468	2.372448394	0.017670631	0.066608721	0.193132644
ENSMUSG00000109511	Nup62	18226	protein_coding	372.9055563	0.193184584	0.072336609	-2.670633683	0.007570822	0.034020286	0.193184584
ENSMUSG00000029911	Ssbp1	381760	protein_coding	224.9552479	0.193253449	0.112552641	-1.717005007	0.085978257	0.22143558	0.193253449
ENSMUSG00000099481	Xndc1	102443350	protein_coding	34.74330764	-0.193320809	0.165031989	1.171414165	0.241432341	0.452093626	0.193320809
ENSMUSG00000025083	Afap1l2	226250	protein_coding	34.87886495	-0.193325161	0.348242089	0.55514588	0.578794883	0.762380457	0.193325161
ENSMUSG00000038383	Pigu	228812	protein_coding	326.6759704	0.193375873	0.084080992	-2.299876201	0.021455235	0.077538047	0.193375873
ENSMUSG00000034427	Myo15b	217328	protein_coding	8292.18235	0.193388031	0.227938809	-0.848420819	0.396203653	0.618597629	0.193388031
ENSMUSG00000020528	Prpsap2	212627	protein_coding	234.8889403	-0.193409315	0.112009464	1.726722978	0.084217427	0.21805685	0.193409315
ENSMUSG00000056131	Pgm3	109785	protein_coding	578.962675	0.193415723	0.111660101	-1.732182951	0.083240993	0.216206632	0.193415723
ENSMUSG00000022619	Mapk8ip2	60597	protein_coding	8.627449759	0.193441616	0.620983165	-0.311508631	0.755413982	0.877032235	0.193441616
ENSMUSG00000029009	Mthfr	17769	protein_coding	246.2972323	-0.193450985	0.089753218	2.15536545	0.031133248	0.103865546	0.193450985
ENSMUSG00000036555	Iqce	74239	protein_coding	136.8278952	0.193457944	0.11001291	-1.758502204	0.078662097	0.207566563	0.193457944
ENSMUSG00000018286	Psmb6	19175	protein_coding	1358.8076	0.193478242	0.070183504	-2.756748125	0.00583793	0.027610883	0.193478242
ENSMUSG00000034528	Hsd17b13	243168	protein_coding	197.3560681	0.193518789	0.16138439	-1.199117143	0.230482399	0.43912271	0.193518789
ENSMUSG00000008136	Fhl2	14200	protein_coding	168.2223578	-0.193584625	0.104636473	1.850068335	0.064303701	0.179528541	0.193584625
ENSMUSG00000000303	Cdh1	12550	protein_coding	11260.16188	-0.193595812	0.080099678	2.416936193	0.015651761	0.060376822	0.193595812
ENSMUSG00000037552	Plekhg2	101497	protein_coding	175.9535712	0.19364533	0.247837408	-0.781340199	0.434602433	0.652884901	0.19364533
ENSMUSG00000078606	Gvin2	100042856	protein_coding	4.896840877	-0.193682646	0.550244674	0.351993677	0.724843004	0.857515393	0.193682646
ENSMUSG00000047495	Dlgap2	244310	protein_coding	3.060931308	0.193720031	1.429644488	-0.135502241	0.892214793	1	0.193720031
ENSMUSG00000034800	Zfp661	72180	protein_coding	56.20341954	-0.193876613	0.142473134	1.360794184	0.17357874	0.364391266	0.193876613
ENSMUSG00000073643	Wdfy1	69368	protein_coding	794.0306957	-0.19389187	0.070550167	2.748283651	0.005990816	0.02813775	0.19389187
ENSMUSG00000020390	Ube2b	22210	protein_coding	613.771816	-0.193907755	0.073990478	2.620712289	0.008774628	0.038324307	0.193907755
ENSMUSG00000035199	Arl6ip5	65106	protein_coding	378.5860503	-0.193922194	0.070045751	2.768507597	0.005631367	0.026854288	0.193922194
ENSMUSG00000009291	Pttg1ip	108705	protein_coding	2348.08714	-0.193923967	0.086848984	2.232887	0.025556397	0.088794097	0.193923967
ENSMUSG00000037750	Fam222b	216971	protein_coding	101.658057	0.193948209	0.134700635	-1.439846278	0.149910895	0.329600388	0.193948209

ensembl_gene_id	external_gene_name	entrezgene_id	gene_biotype	baseMean	log2FoldChange	lfcSE	stat	pvalue	padj	absLog2FC
ENSMUSG00000031889	Phaf1	234678	protein_coding	177.7361938	-0.19396128	0.096217114	2.015870891	0.043813478	0.134811958	0.19396128
ENSMUSG00000038894	Irs2	384783	protein_coding	908.2452513	0.193978431	0.205954907	-0.941849036	0.346269936	0.568024408	0.193978431
ENSMUSG00000070304	Scn2b	72821	protein_coding	33.70106633	-0.194032456	0.248975144	0.779324604	0.435788525	0.653940181	0.194032456
ENSMUSG00000021109	Hif1a	15251	protein_coding	1298.655051	-0.194085906	0.170297866	1.139684902	0.254417599	0.467050404	0.194085906
ENSMUSG00000064330	Pde6h	78600	protein_coding	3.002431167	0.194109036	1.18538007	-0.163752573	0.869925929	1	0.194109036
ENSMUSG00000026245	Farsb	23874	protein_coding	693.3992454	0.194157525	0.104322003	-1.861136865	0.062724851	0.176210159	0.194157525
ENSMUSG00000038332	Sesn1	140742	protein_coding	131.7678533	-0.19428507	0.110074156	1.765038022	0.077557376	0.205616071	0.19428507
ENSMUSG00000036181	H1f2	50708	protein_coding	7024.998537	0.194288914	0.092726819	-2.095282862	0.036145855	0.116153464	0.194288914
ENSMUSG00000057234	Mettl15	76894	protein_coding	46.56581644	-0.194344923	0.165382686	1.175122546	0.239945692	0.450382263	0.194344923
ENSMUSG00000001924	Uba1	22201	protein_coding	4139.393078	0.194351526	0.070435884	-2.759268629	0.005793089	0.027443223	0.194351526
ENSMUSG00000018168	Ikzf3	22780	protein_coding	17.14691942	-0.194372978	0.429298942	0.452768361	0.650715548	0.81021764	0.194372978
ENSMUSG00000044950	Pwwp2a	70802	protein_coding	243.8137758	0.194429103	0.083696907	-2.323014186	0.02017839	0.073869638	0.194429103
ENSMUSG00000062154	Tex33	73376	protein_coding	1.878796304	-0.194459857	1.219563996	0.15945031	0.873314105	1	0.194459857
ENSMUSG00000025156	Gps1	209318	protein_coding	597.5506498	-0.194503214	0.134169466	1.449683147	0.147146897	0.32503797	0.194503214
ENSMUSG00000060510	Zfp266	77519	protein_coding	621.9637289	-0.194531265	0.134494313	1.446390266	0.14806777	0.326547965	0.194531265
ENSMUSG00000015850	Adamtsl4	229595	protein_coding	67.07242817	0.194568137	0.161879798	-1.201929697	0.229390767	0.438111017	0.194568137
ENSMUSG00000030706	Mrpl48	52443	protein_coding	318.1567863	-0.194570263	0.096917491	2.007586679	0.044687231	0.136786828	0.194570263
ENSMUSG00000030629	Zfand6	65098	protein_coding	898.159949	0.19457895	0.076676651	-2.537655836	0.011159767	0.046491522	0.19457895
ENSMUSG00000038742	Angptl6	70726	protein_coding	14.15671115	0.194579052	0.390201396	-0.498663137	0.618016718	0.789504764	0.194579052
ENSMUSG00000028826	Maco1	66146	protein_coding	555.8738028	0.194590276	0.119216002	-1.63224964	0.102626907	0.252289573	0.194590276
ENSMUSG00000057858	Fam204a	76539	protein_coding	162.0763627	0.194592319	0.10026662	-1.940748763	0.052288759	0.154446119	0.194592319
ENSMUSG00000028488	Sh3gl2	20404	protein_coding	159.573154	-0.194611615	0.149046845	1.305707716	0.191651973	0.3894353	0.194611615
ENSMUSG00000031896	Ctrl	109660	protein_coding	7.224315802	0.194619958	0.574306904	-0.338877971	0.73470166	0.864656729	0.194619958
ENSMUSG00000035390	Brsk1	381979	protein_coding	18.16368006	-0.194649664	0.314811239	0.618305957	0.536373676	0.732371878	0.194649664
ENSMUSG00000022847	Thpo	21832	protein_coding	13.50957582	0.194673451	0.318677514	-0.610879157	0.541279584	0.735271124	0.194673451
ENSMUSG00000026649	Cfap126	75472	protein_coding	9.379494128	-0.19472588	0.549294203	0.354501975	0.722962725	0.856549354	0.19472588
ENSMUSG00000024039	Cbs	12411	protein_coding	773.6441896	-0.194737851	0.125303054	1.55413491	0.12015224	0.28232699	0.194737851
ENSMUSG00000030793	Pycard	66824	protein_coding	1925.760933	0.194780653	0.10391052	-1.874503683	0.060861035	0.172631249	0.194780653
ENSMUSG00000069565	Dazap1	70248	protein_coding	901.1296809	0.194790937	0.083141687	-2.342879299	0.019135573	0.070900795	0.194790937
ENSMUSG00000079502	Cfap77	329375	protein_coding	4.211051705	-0.194792546	0.587224215	0.331717497	0.740102588	1	0.194792546
ENSMUSG00000020872	Tac4	93670	protein_coding	2.718418975	-0.194850801	0.771038285	0.252712226	0.800490599	1	0.194850801
ENSMUSG00000022016	Akap11	219181	protein_coding	797.8371958	-0.194913311	0.079270702	2.458831635	0.013938997	0.055069042	0.194913311
ENSMUSG00000045106	Ccdc73	211936	protein_coding	11.03500545	-0.194918901	0.357019063	0.545962166	0.585091944	0.767207271	0.194918901
ENSMUSG00000025269	Apex2	77622	protein_coding	80.45622748	0.194958757	0.133336569	-1.462155193	0.143698687	0.320032382	0.194958757
ENSMUSG00000004356	Utp20	70683	protein_coding	1602.983358	0.194962854	0.065097266	-2.994946888	0.002744926	0.014765379	0.194962854
ENSMUSG00000025893	Kbtbd3	69149	protein_coding	47.41092475	0.194964953	0.150648099	-1.294174667	0.195605095	0.394941012	0.194964953
ENSMUSG00000078153	Psme2b	621823	protein_coding	25.58499774	-0.195164037	0.198890939	0.981261576	0.326463766	0.548343875	0.195164037
ENSMUSG00000024889	Rce1	19671	protein_coding	315.7941362	-0.195168156	0.086258185	2.262604476	0.023660082	0.083756183	0.195168156
ENSMUSG00000040229	Gpr34	23890	protein_coding	5.042864139	0.195184478	0.656614153	-0.297259018	0.766268767	0.882690208	0.195184478

ensembl_gene_id	external_gene_name	entrezgene_id	gene_biotype	baseMean	log2FoldChange	lfcSE	stat	pvalue	padj	absLog2FC
ENSMUSG00000006782	Cnp	12799	protein_coding	412.7897524	0.19520105	0.119055735	-1.639577045	0.101093139	0.249608668	0.19520105
ENSMUSG00000037152	Ndufc1	66377	protein_coding	637.7277788	-0.195205326	0.082422357	2.368354082	0.017867427	0.067059702	0.195205326
ENSMUSG00000031246	Sh3bgrl	56726	protein_coding	1425.480609	-0.195221113	0.132705053	1.471090273	0.141266705	0.315936941	0.195221113
ENSMUSG00000018363	Smurf2	66313	protein_coding	758.3995272	-0.195260785	0.074464017	2.622216637	0.008735988	0.038193919	0.195260785
ENSMUSG00000036766	Dner	227325	protein_coding	4.500829553	-0.195273738	0.798125129	0.244665568	0.80671539	0.904906279	0.195273738
ENSMUSG00000024758	Rtn3	20168	protein_coding	1578.916992	-0.195281465	0.063763914	3.062570234	0.00219445	0.01219993	0.195281465
ENSMUSG00000029723	Spacdr	78829	protein_coding	940.9655193	-0.195376404	0.105780576	1.846996975	0.064747579	0.180584617	0.195376404
ENSMUSG00000096145	Vkorc1	27973	protein_coding	97.62373016	0.195388126	0.168377907	-1.160414271	0.245880179	0.457679163	0.195388126
ENSMUSG00000054909	Wbscr25	71304	lncRNA	15.82449563	-0.195397004	0.492394442	0.396830239	0.69149265	0.835783476	0.195397004
ENSMUSG00000018761	Mpdu1	24070	protein_coding	486.9664778	-0.195410741	0.08353078	2.339386043	0.019315463	0.071455098	0.195410741
ENSMUSG00000002546	Golga2	99412	protein_coding	1278.53553	0.195443386	0.046224763	-4.228110077	2.36E-05	0.000238903	0.195443386
ENSMUSG00000036822	Topors	106021	protein_coding	469.7404297	-0.195504927	0.060513117	3.230785924	0.001234504	0.007575843	0.195504927
ENSMUSG00000048485	Zbtb8b	215627	protein_coding	1.009513459	-0.195537689	1.614358968	0.121124045	0.903592784	1	0.195537689
ENSMUSG00000073791	Efcab7	230500	protein_coding	34.82606105	0.195567907	0.221545433	-0.88274402	0.377374592	0.599541641	0.195567907
ENSMUSG00000027804	Ppid	67738	protein_coding	302.1327699	-0.195615964	0.106014953	1.845173336	0.065012328	0.181090121	0.195615964
ENSMUSG00000035048	Anapc13	69010	protein_coding	382.3010268	-0.195633979	0.094031319	2.080519348	0.037477923	0.11971188	0.195633979
ENSMUSG00000007646	Rad51c	114714	protein_coding	23.02806926	0.195677826	0.306494801	-0.638437668	0.523188817	0.722040793	0.195677826
ENSMUSG00000002475	Abhd3	106861	protein_coding	89.38957869	-0.195707423	0.175862452	1.112843708	0.26577556	0.480830149	0.195707423
ENSMUSG00000000538	Tom1l2	216810	protein_coding	888.8988228	0.195770464	0.1336443	-1.464862059	0.142958556	0.318770942	0.195770464
ENSMUSG00000014303	Glis2	83396	protein_coding	17.53368092	0.195811174	0.360163812	-0.54367254	0.586666832	0.768287864	0.195811174
ENSMUSG00000037007	Zfp113	56314	protein_coding	168.0004753	-0.195834674	0.092117391	2.125925109	0.033509496	0.109753236	0.195834674
ENSMUSG00000034354	Mtmr3	74302	protein_coding	1072.548974	-0.195898851	0.066489062	2.946332011	0.00321567	0.016858279	0.195898851
ENSMUSG00000031831	Dnaaf1	68270	protein_coding	22.26647925	0.195906286	0.304768214	-0.642804193	0.520351162	0.720291626	0.195906286
ENSMUSG00000018822	Sfrp5	54612	protein_coding	3.040096984	-0.196012997	0.687497269	0.285110947	0.775559144	1	0.196012997
ENSMUSG00000050010	Shisa3	330096	protein_coding	4.052201081	0.19602882	0.620167103	-0.316090323	0.751933952	1	0.19602882
ENSMUSG00000012483	Rpa3	68240	protein_coding	159.3919843	0.196037525	0.138275978	-1.417726548	0.156270616	0.338979766	0.196037525
ENSMUSG00000033114	Slc35d2	70484	protein_coding	64.7439584	-0.196060195	0.141419652	1.386371636	0.165633453	0.352762472	0.196060195
ENSMUSG00000034064	Poglut1	224143	protein_coding	176.0436635	-0.196072645	0.135365794	1.448465223	0.147486985	0.325571458	0.196072645
ENSMUSG00000046312	Myorg	329828	protein_coding	239.9469705	-0.196114635	0.150058695	1.306919503	0.191240052	0.388837505	0.196114635
ENSMUSG00000022685	Parn	74108	protein_coding	253.5333977	0.196175208	0.098611562	-1.989373298	0.046660015	0.141461059	0.196175208
ENSMUSG00000041703	Zic5	65100	protein_coding	1.093696463	0.196198178	1.843358759	-0.106435156	0.915237101	1	0.196198178
ENSMUSG00000104585	4921511C10Rik	70924	lncRNA	6.866514195	0.196222695	0.466586269	-0.420549655	0.674083963	0.825190702	0.196222695
ENSMUSG00000024170	Telo2	71718	protein_coding	145.884298	0.196286987	0.130289578	-1.506544042	0.13192757	0.30108314	0.196286987
ENSMUSG00000001228	Uhrf1	18140	protein_coding	433.5344856	0.196287684	0.163124897	-1.203296903	0.228861447	0.437741278	0.196287684
ENSMUSG00000042462	Dctpp1	66422	protein_coding	223.1751078	-0.19629983	0.107157712	1.831877768	0.066969634	0.184932165	0.19629983
ENSMUSG00000026870	Cutal	77996	protein_coding	883.9593632	-0.196301507	0.128929637	1.522547584	0.127871932	0.294844608	0.196301507
ENSMUSG00000036214	Polr1has	76416	protein_coding	65.24610163	0.196308254	0.13635654	-1.439668781	0.14996113	0.329666946	0.196308254
ENSMUSG00000098602	Mir6946	102465571	miRNA	1.459373607	0.196329335	0.929913088	-0.211126543	0.832788529	1	0.196329335
ENSMUSG00000052698	Tln2	70549	protein_coding	856.7780176	-0.19636358	0.145687676	1.347839335	0.177710064	0.370029503	0.19636358

Supplementary Table 1

ensembl_gene_id	external_gene_name	entrezgene_id	gene_biotype	baseMean	log2FoldChange	lfcSE	stat	pvalue	padj	absLog2FC
ENSMUSG00000074649	BC029722	613262	protein_coding	57.02699517	-0.196394691	0.146823117	1.337627851	0.181017781	0.375025422	0.196394691
ENSMUSG00000029173	Sepsecs	211006	protein_coding	557.0187626	-0.196404464	0.083179401	2.361215162	0.018215159	0.068117105	0.196404464
ENSMUSG00000085633	Bloc1s6os	75915	lncRNA	2.241721581	0.196519823	0.819373448	-0.239841581	0.810453071	1	0.196519823
ENSMUSG00000061232	H2-K1	14972	protein_coding	6213.373324	-0.196530637	0.095923711	2.048822295	0.040479495	0.127087144	0.196530637
ENSMUSG00000053453	Thoc7	66231	protein_coding	333.9483129	-0.196636839	0.070087335	2.805597306	0.005022341	0.024436932	0.196636839
ENSMUSG00000032578	Cish	12700	protein_coding	90.64616293	-0.196682269	0.161085249	1.220982496	0.222092647	0.429214508	0.196682269
ENSMUSG00000027080	Med19	381379	protein_coding	252.6959852	0.196692533	0.08225912	-2.39113344	0.016796447	0.063995287	0.196692533
ENSMUSG00000037012	Hk1	15275	protein_coding	2447.478373	-0.196722697	0.070879392	2.775456896	0.005512421	0.026363195	0.196722697
ENSMUSG00000067722	BC003965	214489	protein_coding	292.6760521	-0.19675496	0.084011092	2.342011702	0.019180114	0.071033953	0.19675496
ENSMUSG00000025261	Huwe1	59026	protein_coding	3430.262596	0.196891648	0.082089074	-2.398512243	0.016461826	0.06297211	0.196891648
ENSMUSG00000022533	Atp13a3	224088	protein_coding	2257.360283	0.196902184	0.112178623	-1.755255854	0.079215554	0.208539082	0.196902184
ENSMUSG00000020652	Cenpo	52504	protein_coding	107.5297666	0.196912148	0.121728711	-1.617631092	0.105742124	0.25785531	0.196912148
ENSMUSG00000030353	Tead4	21679	protein_coding	47.00008369	0.196920407	0.162283794	-1.21343236	0.224964561	0.432209358	0.196920407
ENSMUSG00000082769	Gm15793	102640707	processed_pseudogene	4.956336054	0.196937996	0.509364562	-0.386634662	0.699026693	0.840583034	0.196937996
ENSMUSG00000024503	Spink1	20730	protein_coding	1764.852772	-0.196978643	0.370510721	0.531640873	0.594974751	0.773528957	0.196978643
ENSMUSG00000022235	Cmbl	69574	protein_coding	473.3796486	-0.197050982	0.097808043	2.014670529	0.043939182	0.135072906	0.197050982
ENSMUSG00000034850	Tmem127	69470	protein_coding	1269.330798	-0.197062849	0.057746398	3.412556529	0.000643566	0.004377202	0.197062849
ENSMUSG00000068205	Macrod2	72899	protein_coding	81.3838651	0.197100373	0.116189911	-1.696363914	0.089816983	0.228436418	0.197100373
ENSMUSG00000020599	Rgs9	19739	protein_coding	7.810350848	0.197148146	0.719499266	-0.274007431	0.784078911	0.891754761	0.197148146
ENSMUSG00000106864	Gtf3c2	71752	protein_coding	215.9801971	-0.197174105	0.077360721	2.548762503	0.010810589	0.045254367	0.197174105
ENSMUSG00000028876	Epha10	230735	protein_coding	3.266303825	0.19719824	1.41530966	-0.139332222	0.889187628	1	0.19719824
ENSMUSG00000038057	Dbil5	13168	protein_coding	2.730616878	-0.197261975	0.625043194	0.315597349	0.75230815	1	0.197261975
ENSMUSG00000032119	Hinfp	102423	protein_coding	306.9718023	0.19731082	0.092558322	-2.131745871	0.033027744	0.108584543	0.19731082
ENSMUSG00000056917	Sipa1	20469	protein_coding	52.73060474	-0.197311384	0.262876077	0.75058707	0.452901205	0.669100957	0.197311384
ENSMUSG00000079557	Marchf2	224703	protein_coding	543.5442313	-0.19731383	0.075755782	2.604604234	0.009198044	0.039805161	0.19731383
ENSMUSG00000030435	U2af2	22185	protein_coding	1400.742857	0.197333245	0.074103733	-2.662932582	0.007746293	0.034686086	0.197333245
ENSMUSG00000047242	Taf9b	407786	protein_coding	64.50351298	0.197398864	0.171746621	-1.149360978	0.250407164	0.462725114	0.197398864
ENSMUSG00000026637	Traf5	22033	protein_coding	30.98674677	-0.197412391	0.19492619	1.012754577	0.311177402	0.532085855	0.197412391
ENSMUSG00000012126	Ubxn11	67586	protein_coding	22.92696816	0.197454805	0.283251467	-0.697100731	0.485739757	0.695380179	0.197454805
ENSMUSG00000017679	Ttpal	76080	protein_coding	313.9083776	0.197481588	0.066253747	-2.980685563	0.002876039	0.01536058	0.197481588
ENSMUSG00000030966	Trim21	20821	protein_coding	150.1656992	-0.19751841	0.106614449	1.852642028	0.063933684	0.178888658	0.19751841
ENSMUSG00000042564	Fam227a	75729	protein_coding	13.61292002	-0.197538548	0.327921487	0.602395867	0.54691066	0.738667101	0.197538548
ENSMUSG00000048706	Lurap1l	52829	protein_coding	1670.505441	-0.197558065	0.070073743	2.81928803	0.004813031	0.023543362	0.197558065
ENSMUSG00000004937	Sgta	52551	protein_coding	936.5555878	0.197577559	0.061681431	-3.203193501	0.001359126	0.008215451	0.197577559
ENSMUSG00000061665	Cd2ap	12488	protein_coding	4697.422268	0.197604011	0.069684844	-2.835681341	0.004572805	0.022575574	0.197604011
ENSMUSG00000061118	Dnajc30	66114	protein_coding	125.03056	-0.197632001	0.100631963	1.96390883	0.049540666	0.147967906	0.197632001
ENSMUSG00000027775	Mfsd1	66868	protein_coding	711.259561	-0.197705104	0.090541677	2.183581215	0.028993035	0.098274011	0.197705104
ENSMUSG00000048720	Tbc1d12	209478	protein_coding	143.6882113	0.197749349	0.133319364	-1.483275516	0.138001225	0.31052626	0.197749349
ENSMUSG00000044719	E230025N22Rik	240216	protein_coding	61.15496917	0.197785107	0.158501125	-1.24784671	0.212087199	0.41642117	0.197785107

Supplementary Table 1

ensembl_gene_id	external_gene_name	entrezgene_id	gene_biotype	baseMean	log2FoldChange	lfcSE	stat	pvalue	padj	absLog2FC
ENSMUSG00000023764	Sfi1	78887	protein_coding	437.1792321	-0.197837409	0.270222646	0.732127423	0.464090798	0.677570103	0.197837409
ENSMUSG00000002781	Tmem143	70209	protein_coding	240.0918913	-0.197885238	0.109733683	1.803322673	0.071337554	0.193614755	0.197885238
ENSMUSG00000029422	Rsrc2	208606	protein_coding	851.8249423	-0.197909709	0.060120833	3.291865722	0.000995251	0.006340887	0.197909709
ENSMUSG00000053930	Shisa6	380702	protein_coding	3.31131204	-0.197927954	1.548913125	0.127785058	0.898319075	1	0.197927954
ENSMUSG00000087354	4930404I05Rik	67394	lncRNA	4.095154259	-0.197951695	0.60954229	0.324754653	0.745366767	1	0.197951695
ENSMUSG00000042197	Zfp451	98403	protein_coding	310.4047953	0.198000369	0.078580864	-2.519702098	0.011745419	0.048307661	0.198000369
ENSMUSG00000038473	Nos1ap	70729	protein_coding	65.64001598	-0.198054579	0.239083073	0.82839231	0.40744836	0.628254175	0.198054579
ENSMUSG00000027636	Sla2	77799	protein_coding	8.808779535	0.198112766	0.531224895	-0.372935771	0.709196248	0.84811839	0.198112766
ENSMUSG00000031242	Chmp1b2	67028	protein_coding	404.8938859	0.198115143	0.10005738	-1.980015299	0.04770181	0.143880855	0.198115143
ENSMUSG00000025372	Baiap2	108100	protein_coding	301.2198203	0.198193977	0.102522112	-1.933182746	0.053213689	0.156392751	0.198193977
ENSMUSG00000032023	Jhy	70989	protein_coding	2.07486104	0.198197081	1.106486704	-0.179122876	0.85784122	1	0.198197081
ENSMUSG00000040859	Bsdc1	100383	protein_coding	986.8975794	-0.198341418	0.080453709	2.46528619	0.013690385	0.054307717	0.198341418
ENSMUSG00000042363	Lgalsl	216551	protein_coding	566.7422814	0.19835771	0.088556161	-2.239908633	0.025096855	0.087584418	0.19835771
ENSMUSG00000041231	Ublcp1	79560	protein_coding	271.4871247	-0.198412402	0.093121803	2.130676129	0.033115833	0.108765884	0.198412402
ENSMUSG00000099689	Zfp383	73729	protein_coding	24.37610379	-0.198467244	0.241410954	0.822113665	0.411012209	0.631215067	0.198467244
ENSMUSG00000025508	Rplp2	67186	protein_coding	3825.499404	-0.198513168	0.112518573	1.764270227	0.077686494	0.205793193	0.198513168
ENSMUSG00000020541	Tom1l1	71943	protein_coding	856.1949994	-0.198516232	0.063236859	3.139248761	0.001693816	0.009870396	0.198516232
ENSMUSG00000057421	Las1l	76130	protein_coding	355.9559262	0.198523252	0.068103923	-2.915004665	0.003556832	0.018344012	0.198523252
ENSMUSG00000020163	Uqcr11	66594	protein_coding	2359.194875	-0.198526172	0.092135448	2.154720863	0.031183684	0.103991793	0.198526172
ENSMUSG00000038781	Stap2	106766	protein_coding	877.8323372	-0.198559053	0.097917655	2.027816656	0.042578964	0.131848679	0.198559053
ENSMUSG00000069516	Lyz2	17105	protein_coding	13.27462715	-0.198579455	0.545770669	0.363851459	0.715968934	0.852575063	0.198579455
ENSMUSG00000098923	Tmem185b	226351	protein_coding	151.3188015	-0.198619151	0.112801574	1.760783511	0.078275052	0.20691859	0.198619151
ENSMUSG00000117684	4930449E18Rik	78120	lncRNA	2.075727916	0.198660024	1.096271159	-0.181214312	0.856199361	1	0.198660024
ENSMUSG00000049606	Zfp644	52397	protein_coding	984.7164987	-0.198712684	0.080948132	2.454814947	0.014095713	0.055555275	0.198712684
ENSMUSG00000048897	Zfp710	209225	protein_coding	641.2033248	0.198715087	0.1006183	-1.974939815	0.048274978	0.1452832	0.198715087
ENSMUSG00000020251	Glt8d2	74782	protein_coding	4.840622786	0.198831088	0.523055783	-0.380133621	0.70384623	0.844104331	0.198831088
ENSMUSG00000021024	Psma6	26443	protein_coding	1067.799567	0.198852342	0.070547107	-2.818717158	0.004821598	0.023571307	0.198852342
ENSMUSG00000037656	Slc20a2	20516	protein_coding	232.252308	-0.198884607	0.112099304	1.774182353	0.076032992	0.202452247	0.198884607
ENSMUSG00000041199	Rpusd1	106707	protein_coding	60.83445706	0.198895912	0.139990027	-1.420786297	0.155378896	0.337710856	0.198895912
ENSMUSG00000059857	Ntng1	80883	protein_coding	2.561819837	-0.198904473	1.350431268	0.147289594	0.882903446	1	0.198904473
ENSMUSG00000029725	Ppp1r35	69871	protein_coding	98.05067474	-0.19891838	0.14115209	1.409248562	0.158761683	0.342763083	0.19891838
ENSMUSG00000060477	Irak2	108960	protein_coding	435.3691408	-0.198923691	0.168260769	1.182234525	0.237112657	0.447506105	0.198923691
ENSMUSG00000030869	Ndufab1	70316	protein_coding	704.447861	-0.198982872	0.101186715	1.966492062	0.049241798	0.147315047	0.198982872
ENSMUSG00000029404	Arl6ip4	65105	protein_coding	296.5077556	0.199044086	0.075432646	-2.638699488	0.008322472	0.036718488	0.199044086
ENSMUSG00000014243	Zswim7	69747	protein_coding	57.47589174	-0.199054215	0.144788669	1.374791382	0.169196131	0.358402373	0.199054215
ENSMUSG00000056718	Gm13199	791411	protein_coding	4.150639168	0.199122558	0.504533473	-0.394666694	0.693088886	1	0.199122558
ENSMUSG00000046879	Irgm1	15944	protein_coding	535.3159919	0.199169953	0.134320228	-1.4827994	0.138127716	0.310726208	0.199169953
ENSMUSG00000071359	Tbpl1	237336	protein_coding	222.3835769	0.199172937	0.115675826	-1.721819873	0.085102153	0.219864981	0.199172937
ENSMUSG00000075602	Ly6a	110454	protein_coding	828.0159047	-0.19919659	0.207860232	0.958319869	0.337901488	0.559633454	0.19919659

ensembl_gene_id	external_gene_name	entrezgene_id	gene_biotype	baseMean	log2FoldChange	lfcSE	stat	pvalue	padj	absLog2FC
ENSMUSG00000020375	Rufy1	216724	protein_coding	296.9477303	-0.199242355	0.078309298	2.544300085	0.010949697	0.045697068	0.199242355
ENSMUSG00000032396	Dis3l	213550	protein_coding	281.0596975	-0.19924656	0.107653327	1.850816555	0.064195949	0.179403047	0.19924656
ENSMUSG00000061353	Cxcl12	20315	protein_coding	2.454987812	0.199276268	1.758689324	-0.113309534	0.909785159	1	0.199276268
ENSMUSG00000025825	Iscu	66383	protein_coding	317.7030967	-0.199311961	0.079423941	2.509469546	0.012091264	0.049412308	0.199311961
ENSMUSG00000038354	Ankrd35	213121	protein_coding	27.10780235	0.19932657	0.250933355	-0.794340674	0.426997131	0.646395058	0.19932657
ENSMUSG00000071644	Eef1g	67160	protein_coding	4154.50887	-0.199441905	0.104663936	1.90554561	0.056709184	0.163952289	0.199441905
ENSMUSG00000058267	Mrps14	64659	protein_coding	380.3598086	0.199465172	0.121454869	-1.64229869	0.100528118	0.248744583	0.199465172
ENSMUSG00000023915	Tnfrsf21	94185	protein_coding	968.4059235	-0.199497964	0.085846749	2.323884915	0.020131663	0.073733764	0.199497964
ENSMUSG00000040249	Lrp1	16971	protein_coding	3759.472412	0.199530824	0.217885049	-0.91576189	0.359791796	0.582349713	0.199530824
ENSMUSG00000035239	Neu3	50877	protein_coding	50.201232	-0.199631346	0.261774812	0.762607161	0.445697713	0.662922836	0.199631346
ENSMUSG00000025794	Rpl14	67115	protein_coding	4128.125655	-0.199632438	0.120174145	1.661192911	0.096674709	0.241063164	0.199632438
ENSMUSG00000015575	Atp6v0e	11974	protein_coding	463.1379784	-0.199640393	0.074714139	2.672056388	0.007538798	0.03389483	0.199640393
ENSMUSG00000052763	Zfp212	232784	protein_coding	165.4047057	-0.199653195	0.10606354	1.882392332	0.059782766	0.170333437	0.199653195
ENSMUSG00000022575	Gsdmd	69146	protein_coding	1054.782998	-0.199658384	0.083392061	2.394213328	0.016656058	0.063582557	0.199658384
ENSMUSG00000060029	4930473A02Rik	321014	lncRNA	2.987681182	-0.199750992	0.583636816	0.342252214	0.732161094	1	0.199750992
ENSMUSG00000032415	Ube2cbp	70348	protein_coding	30.70457409	-0.19975783	0.224558706	0.889557269	0.373703659	0.595844868	0.19975783
ENSMUSG00000001143	Lman2l	214895	protein_coding	193.5999613	0.199794765	0.117897613	-1.694646398	0.09014252	0.228911821	0.199794765
ENSMUSG00000035640	Cbarp	100503659	protein_coding	29.96439395	-0.199842875	0.266010469	0.751259437	0.452496536	0.668802273	0.199842875
ENSMUSG00000028550	Atg4c	242557	protein_coding	164.1856513	0.199893855	0.104301184	-1.916506095	0.055300708	0.160639559	0.199893855
ENSMUSG00000025184	R3hcc1l	52013	protein_coding	263.5271315	-0.199963284	0.06907181	2.895005703	0.003791516	0.019336968	0.199963284
ENSMUSG00000066278	Vps37b	330192	protein_coding	287.6005844	0.200015287	0.151807455	-1.31755906	0.187651298	0.383856501	0.200015287
ENSMUSG00000066151	Fkbp15	338355	protein_coding	679.3133015	-0.200036453	0.063949844	3.128020954	0.001759876	0.010183111	0.200036453
ENSMUSG00000054519	Zfp867	237775	protein_coding	57.21362027	-0.200054711	0.1376547	1.453308253	0.146138187	0.323458788	0.200054711
ENSMUSG00000063952	Brpf3	268936	protein_coding	587.9393225	0.200108723	0.229036744	-0.873697031	0.382283268	0.604659935	0.200108723
ENSMUSG00000021326	Trim27	19720	protein_coding	564.9555826	0.200130512	0.078520804	-2.548757801	0.010810735	0.045254367	0.200130512
ENSMUSG00000024505	Dtwd2	68857	protein_coding	35.4864083	0.200135044	0.234263737	-0.854315081	0.392930433	0.615582315	0.200135044
ENSMUSG00000029076	Sdf4	20318	protein_coding	1957.333345	-0.200221638	0.067081963	2.98473136	0.002838275	0.015198206	0.200221638
ENSMUSG00000104768	Gm38412	329730	lncRNA	2.196713347	-0.200233358	2.118043488	0.094536944	0.924682637	1	0.200233358
ENSMUSG00000067786	Nnat	18111	protein_coding	62.92630572	0.200255877	0.43072697	-0.464925326	0.641984933	0.804573016	0.200255877
ENSMUSG00000050335	Lgals3	16854	protein_coding	15954.24501	-0.200282251	0.147350211	1.35922609	0.174074956	0.365037946	0.200282251
ENSMUSG00000092680	Snord55	100216533	snoRNA	14.03553992	0.200286192	0.346420725	-0.578158805	0.563156909	0.750770075	0.200286192
ENSMUSG00000026478	Lamc1	226519	protein_coding	141.173497	0.200293831	0.129916437	-1.541712781	0.123143405	0.286623	0.200293831
ENSMUSG00000000568	Hnrnpd	11991	protein_coding	590.0392508	0.200349854	0.058140263	-3.445974319	0.000569004	0.003951446	0.200349854
ENSMUSG00000044703	Phf11a	219131	protein_coding	1.942545018	0.200356946	1.046244087	-0.19150115	0.848132979	1	0.200356946
ENSMUSG00000041141	Pnma8a	71691	protein_coding	5.862582738	-0.200393557	0.443520477	0.451824814	0.651395192	0.81075823	0.200393557
ENSMUSG00000071691	Gm960	381196	protein_coding	5.3350233	0.200475922	0.776716942	-0.258106797	0.796324486	0.898810919	0.200475922
ENSMUSG00000022650	Retnlb	57263	protein_coding	1123.237453	0.200509549	0.248429862	-0.807107276	0.419604681	0.639652146	0.200509549
ENSMUSG00000030120	Mlf2	30853	protein_coding	2125.669826	0.200535082	0.065114518	-3.079729206	0.002071889	0.01168152	0.200535082
ENSMUSG00000031373	Car5b	56078	protein_coding	9.238668342	0.200616561	0.338382562	-0.592869088	0.553268767	0.743665995	0.200616561

ensembl_gene_id	external_gene_name	entrezgene_id	gene_biotype	baseMean	log2FoldChange	lfcSE	stat	pvalue	padj	absLog2FC
ENSMUSG00000035678	Tnfsf9	21950	protein_coding	4.07814195	-0.200619626	0.576230804	0.348158454	0.727721187	1	0.200619626
ENSMUSG00000062098	Btbd3	228662	protein_coding	259.4957261	-0.200627796	0.094095131	2.132180423	0.032992017	0.108571968	0.200627796
ENSMUSG00000078517	Emc1	230866	protein_coding	792.8036004	0.200644265	0.073528204	-2.728806815	0.006356393	0.02939498	0.200644265
ENSMUSG00000035914	Cd276	102657	protein_coding	56.98418587	0.20069789	0.170777661	-1.175199902	0.23991475	0.450375375	0.20069789
ENSMUSG00000002108	Nr1h3	22259	protein_coding	218.0999375	-0.200752426	0.135507427	1.481486517	0.138476975	0.311384636	0.200752426
ENSMUSG00000035878	Hykk	235386	protein_coding	97.48062837	-0.200756996	0.13662186	1.469435391	0.141714734	0.316724471	0.200756996
ENSMUSG00000025001	Hells	15201	protein_coding	223.7573138	0.200789495	0.192884389	-1.040983653	0.297883135	0.517488122	0.200789495
ENSMUSG00000004709	Cd244a	18106	protein_coding	12.94387094	-0.200797063	0.434797411	0.461817523	0.644212184	0.806110415	0.200797063
ENSMUSG00000026034	Clk1	12747	protein_coding	1186.570251	-0.200799445	0.131342906	1.528818353	0.126309481	0.292303079	0.200799445
ENSMUSG00000032059	Alg9	102580	protein_coding	533.0050753	0.200972237	0.089225932	-2.252397185	0.024297183	0.085498335	0.200972237
ENSMUSG00000047369	Dnah14	240960	protein_coding	10.13073476	-0.201009636	0.721335392	0.27866321	0.780503299	0.890186044	0.201009636
ENSMUSG00000044243	Bhlha9	320522	protein_coding	1.330308751	0.201121741	1.099606038	-0.182903453	0.854873773	1	0.201121741
ENSMUSG00000044098	Rsbn1	229675	protein_coding	256.6177166	-0.201151475	0.073503736	2.736615643	0.006207477	0.028892413	0.201151475
ENSMUSG00000072707	Or2t1	18330	protein_coding	1.793092102	0.201205015	2.135180054	-0.094233278	0.924923851	1	0.201205015
ENSMUSG00000026961	Lrrc26	227618	protein_coding	337.1660421	-0.201206019	0.158058537	1.272984192	0.203023651	0.405213026	0.201206019
ENSMUSG00000029314	Gpat3	231510	protein_coding	188.5665395	0.201242566	0.13794416	-1.458869773	0.144600954	0.321521497	0.201242566
ENSMUSG00000046410	Kcnk6	52150	protein_coding	725.0816454	0.201257376	0.106973356	-1.881378536	0.059920445	0.1706035	0.201257376
ENSMUSG00000086013	Gm15706	100039495	lncRNA	35.97571296	-0.20133178	0.177024785	1.137308427	0.255409373	0.468075728	0.20133178
ENSMUSG00000050954	Zfp169	67911	protein_coding	106.7852293	-0.201365141	0.153532716	1.311545486	0.189673537	0.386972505	0.201365141
ENSMUSG00000026785	Pkn3	263803	protein_coding	96.19740908	0.201393666	0.169275196	-1.189741152	0.234148146	0.443634979	0.201393666
ENSMUSG00000040620	Dhx33	216877	protein_coding	299.8533508	0.20141471	0.094496967	-2.131441001	0.033052828	0.108625296	0.20141471
ENSMUSG00000070877	Ldlrad1	546840	protein_coding	4.236338044	0.201435053	0.644377768	-0.312603977	0.754581556	1	0.201435053
ENSMUSG00000033918	Parl	381038	protein_coding	379.9914651	-0.201471274	0.082406686	2.444841355	0.014491587	0.056736385	0.201471274
ENSMUSG00000021290	Atp5mpl	70257	protein_coding	1582.998141	-0.201532603	0.069960412	2.880666348	0.003968355	0.020065343	0.201532603
ENSMUSG00000034334	Fam151b	73942	protein_coding	38.77052933	0.201544158	0.189571978	-1.063153745	0.287712235	0.506641059	0.201544158
ENSMUSG00000074643	Cpne1	266692	protein_coding	2.208897915	-0.201573836	0.779111198	0.258722806	0.795849122	1	0.201573836
ENSMUSG00000036928	Stag3	50878	protein_coding	4.12582339	0.201640609	1.235012796	-0.163270057	0.870305809	1	0.201640609
ENSMUSG00000039137	Whrn	73750	protein_coding	112.3632648	-0.201892257	0.204009312	0.989622756	0.322358543	0.54358318	0.201892257
ENSMUSG00000032308	Ulk3	71742	protein_coding	86.87394365	-0.20189601	0.154536154	1.30646457	0.19139462	0.389062992	0.20189601
ENSMUSG00000037761	Actr5	109275	protein_coding	128.2185048	-0.201962493	0.1117608	1.807095987	0.070747314	0.192474654	0.201962493
ENSMUSG00000053178	Mterf1b	208595	protein_coding	10.49258331	-0.202011851	0.340855383	0.592661467	0.553407734	0.743731716	0.202011851
ENSMUSG00000027296	Itpka	228550	protein_coding	147.4346566	-0.202044025	0.138832262	1.455310335	0.145583367	0.322749862	0.202044025
ENSMUSG00000022840	Adcy5	224129	protein_coding	59.20751456	-0.202046577	0.216742849	0.932194892	0.351235815	0.573211901	0.202046577
ENSMUSG00000035235	Trim13	66597	protein_coding	45.57385387	-0.202054945	0.173282462	1.166043828	0.243596726	0.454822267	0.202054945
ENSMUSG00000002763	Pex6	224824	protein_coding	720.4521626	-0.202078852	0.05326876	3.793571569	0.000148496	0.001218211	0.202078852
ENSMUSG00000029388	Eif2b1	209354	protein_coding	812.7800603	0.202082711	0.090049512	-2.244128894	0.024824109	0.086889644	0.202082711
ENSMUSG00000047293	Gpr15	71223	protein_coding	1.829130367	0.202108632	1.113161499	-0.181562722	0.855925906	1	0.202108632
ENSMUSG00000023022	Lima1	65970	protein_coding	4427.735788	-0.202111738	0.094372794	2.141631388	0.032223156	0.106618553	0.202111738
ENSMUSG00000006241	Ccdc159	67119	protein_coding	20.8570792	-0.2021179	0.296228182	0.682304767	0.49504626	0.701011313	0.2021179

ensembl_gene_id	external_gene_name	entrezgene_id	gene_biotype	baseMean	log2FoldChange	lfcSE	stat	pvalue	padj	absLog2FC
ENSMUSG00000020381	Mrnip	68067	protein_coding	19.23696217	0.202155634	0.286800972	-0.704863838	0.480894976	0.691768261	0.202155634
ENSMUSG00000016382	Pls3	102866	protein_coding	168.870552	-0.202167731	0.095110453	2.125610015	0.033535745	0.109773894	0.202167731
ENSMUSG00000089281	Scarna6	100217415	scaRNA	509.0355506	-0.202326323	0.165241769	1.224426027	0.220791556	0.427312825	0.202326323
ENSMUSG00000075054	Yae1d1	67008	protein_coding	144.0469453	-0.202396138	0.09288543	2.178986915	0.02933264	0.099059146	0.202396138
ENSMUSG00000036459	Wtip	101543	protein_coding	157.2762602	0.202427497	0.151742475	-1.334020006	0.1821973	0.376665775	0.202427497
ENSMUSG00000110344	Smim36	121811722	protein_coding	12.54430808	-0.202471634	0.324069583	0.624778271	0.532116594	0.728866518	0.202471634
ENSMUSG00000029147	Ppm1g	14208	protein_coding	994.6693396	0.202479927	0.087052615	-2.32594881	0.020021282	0.073420411	0.202479927
ENSMUSG00000058056	Palld	72333	protein_coding	235.5317194	0.202510826	0.170140494	-1.190256482	0.2339456	0.44350564	0.202510826
ENSMUSG00000107456	Gm10400	100093700	TEC	16.75350537	-0.202520675	0.32912214	0.61533592	0.5383329	0.733377497	0.202520675
ENSMUSG00000036155	Mgat5	107895	protein_coding	1894.759269	-0.202537969	0.075961378	2.666328274	0.007668477	0.034412292	0.202537969
ENSMUSG00000032458	Copb2	50797	protein_coding	3276.41685	0.202612087	0.097001844	-2.088744693	0.036730711	0.1177122	0.202612087
ENSMUSG00000043099	Hic1	15248	protein_coding	12.28360544	0.20262558	0.477495218	-0.424351015	0.67130984	0.823809965	0.20262558
ENSMUSG00000044912	Syt16	238266	protein_coding	3.122624443	-0.202640894	1.159310261	0.174794359	0.861241221	1	0.202640894
ENSMUSG00000080364	Gm25777	115488712	snoRNA	3.282207993	0.202643272	0.588156422	-0.344539759	0.730440401	1	0.202643272
ENSMUSG00000024294	Mib1	225164	protein_coding	1557.703235	0.202725377	0.076883533	-2.636785397	0.008369576	0.036886891	0.202725377
ENSMUSG00000023150	Ivns1abp	117198	protein_coding	4510.427662	-0.20276088	0.133246821	1.521693936	0.128085788	0.295198531	0.20276088
ENSMUSG00000033624	Pdpr	319518	protein_coding	550.70092	-0.202818708	0.084429014	2.402239449	0.016295038	0.062415953	0.202818708
ENSMUSG00000040562	Gstm2	14863	protein_coding	979.5791652	0.202850356	0.131370059	-1.544114067	0.122560701	0.285669361	0.202850356
ENSMUSG00000044763	Trmt10c	52575	protein_coding	228.6717956	-0.202917557	0.113853164	1.782274198	0.074704528	0.199909758	0.202917557
ENSMUSG00000077565	Gm23201	115486177	snoRNA	8.143552718	-0.202936087	0.398016655	0.509868329	0.610143711	0.783873755	0.202936087
ENSMUSG00000078862	Gm14326	628147	protein_coding	43.29275658	-0.202950656	0.228418457	0.888503751	0.374269839	0.596246587	0.202950656
ENSMUSG00000050270	Tmem220	338369	protein_coding	60.50280818	0.202993545	0.192466335	-1.054696372	0.291564212	0.510430551	0.202993545
ENSMUSG00000025577	Cbx2	12416	protein_coding	79.10411912	-0.203022728	0.149445008	1.358511271	0.174301509	0.365420225	0.203022728
ENSMUSG00000030701	Plekhb1	27276	protein_coding	18.75973865	0.203028532	0.365750646	-0.555100951	0.578825613	0.762380457	0.203028532
ENSMUSG00000029003	Mad2l2	71890	protein_coding	57.44301246	-0.203044015	0.139168849	1.458976023	0.144571707	0.321507788	0.203044015
ENSMUSG00000087516	Tbx3os1	100039542	lncRNA	2.116243267	0.203096321	1.902048111	-0.106777699	0.91496534	1	0.203096321
ENSMUSG00000078607	1810010H24Rik	69066	protein_coding	14.89573155	0.203162423	0.284330703	-0.714528612	0.47490037	0.686795739	0.203162423
ENSMUSG00000079334	Naa80	56441	protein_coding	115.4106227	-0.203232771	0.113678976	1.78777798	0.073811835	0.198490252	0.203232771
ENSMUSG00000013997	Nit1	27045	protein_coding	523.401006	-0.203279563	0.073924809	2.74981521	0.005962888	0.028062449	0.203279563
ENSMUSG00000042129	Rassf4	213391	protein_coding	219.1180433	0.203301273	0.178180461	-1.140985224	0.253876072	0.466508229	0.203301273
ENSMUSG00000006127	Inpp5k	19062	protein_coding	277.5798478	-0.203310899	0.084077018	2.418150703	0.015599615	0.06023867	0.203310899
ENSMUSG00000027272	Ubr1	22222	protein_coding	363.8184511	-0.20338824	0.08404987	2.419851933	0.015526828	0.060049189	0.20338824
ENSMUSG00000025225	Nfkb2	18034	protein_coding	615.784582	0.203444516	0.081542207	-2.494959643	0.012597146	0.050983705	0.203444516
ENSMUSG00000018339	Gpx3	14778	protein_coding	16.98333351	0.203462073	0.301866724	-0.67401292	0.500303099	0.704889121	0.203462073
ENSMUSG00000031958	Ldhd	52815	protein_coding	81.7865209	-0.203502518	0.117683694	1.729232926	0.083767416	0.217061839	0.203502518
ENSMUSG00000036805	Noxa1	241275	protein_coding	941.2347946	0.203528044	0.105661473	-1.926227575	0.054075964	0.158138918	0.203528044
ENSMUSG00000029111	Nelfa	24116	protein_coding	283.3223238	0.203579743	0.076111433	-2.674759028	0.007478298	0.033677833	0.203579743
ENSMUSG00000034126	Pomt2	217734	protein_coding	221.9372219	0.203694917	0.092763794	-2.195845037	0.028103037	0.095748618	0.203694917
ENSMUSG00000042506	Usp22	216825	protein_coding	963.9994378	0.203705068	0.073479484	-2.772271338	0.005566661	0.026568742	0.203705068

ensembl_gene_id	external_gene_name	entrezgene_id	gene_biotype	baseMean	log2FoldChange	lfcSE	stat	pvalue	padj	absLog2FC
ENSMUSG00000003848	Nob1	67619	protein_coding	149.9183417	-0.203709448	0.105621793	1.928668721	0.053772002	0.157556926	0.203709448
ENSMUSG00000027709	Mccc1	72039	protein_coding	270.0785606	-0.203724572	0.097715497	2.084874752	0.037080671	0.118695523	0.203724572
ENSMUSG00000028291	Akirin2	433693	protein_coding	272.7017353	0.20378194	0.089250417	-2.283260382	0.022415035	0.080365152	0.20378194
ENSMUSG00000074093	Svip	75744	protein_coding	222.007557	-0.203819874	0.091814363	2.219912745	0.026424691	0.091158836	0.203819874
ENSMUSG00000039521	Foxp3	20371	protein_coding	4.861232003	-0.203826683	0.777181058	0.262264091	0.793117845	0.897030894	0.203826683
ENSMUSG00000048807	Slc35e4	103710	protein_coding	149.4091779	-0.203865924	0.118595444	1.719002992	0.085613827	0.220841415	0.203865924
ENSMUSG00000038429	Usp5	22225	protein_coding	1226.077587	0.203903161	0.07582818	-2.689015624	0.007166306	0.032583807	0.203903161
ENSMUSG00000001415	Smg5	229512	protein_coding	939.8515114	0.203957351	0.054982425	-3.709500806	0.000207668	0.001633842	0.203957351
ENSMUSG00000001034	Mapk7	23939	protein_coding	179.2333901	0.203983351	0.107420847	-1.898917736	0.057575293	0.165730938	0.203983351
ENSMUSG00000061689	Dlgap4	228836	protein_coding	301.6139486	0.203990609	0.096651401	-2.110580989	0.034808343	0.112998816	0.203990609
ENSMUSG00000025049	Taf5	226182	protein_coding	105.172549	0.204040164	0.142106457	-1.435826135	0.151051821	0.331061353	0.204040164
ENSMUSG00000022208	Jph4	319984	protein_coding	7.959427254	0.204239736	1.006164699	-0.202988373	0.83914412	0.919794636	0.204239736
ENSMUSG00000027173	Depdc7	211896	protein_coding	7.89618765	-0.204311489	0.438370661	0.46607017	0.641165274	0.804106371	0.204311489
ENSMUSG00000034154	Ino80	68142	protein_coding	520.8437612	-0.204328061	0.078431857	2.605166675	0.009182958	0.039779225	0.204328061
ENSMUSG00000020577	Tspan13	66109	protein_coding	1332.714959	0.204359956	0.175940526	-1.161528615	0.245426993	0.457189099	0.204359956
ENSMUSG00000001052	Sec24b	99683	protein_coding	368.63899	0.204368377	0.114036679	-1.792128458	0.073112391	0.197090766	0.204368377
ENSMUSG00000047866	Lonp2	66887	protein_coding	1022.882594	-0.204405321	0.059563558	3.431717788	0.000599771	0.004126525	0.204405321
ENSMUSG00000084651	Mir1904	100316683	miRNA	0.675602507	0.204456635	1.435162427	-0.142462366	0.886714804	1	0.204456635
ENSMUSG00000044340	Phlpp1	98432	protein_coding	477.7477236	-0.20448194	0.077307457	2.645048065	0.008167931	0.036230249	0.20448194
ENSMUSG00000042473	Tbc1d8b	245638	protein_coding	560.6046263	0.204548559	0.108869242	-1.878846183	0.060265499	0.171354072	0.204548559
ENSMUSG00000010095	Slc3a2	17254	protein_coding	546.909011	0.204563725	0.070591325	-2.89785927	0.003757191	0.019203423	0.204563725
ENSMUSG00000004233	Wars2	70560	protein_coding	194.3922823	-0.204580529	0.10414034	1.964469577	0.049475661	0.147853975	0.204580529
ENSMUSG00000097397	Gm16861	100503704	lncRNA	11.07403314	-0.204589542	0.35407428	0.577815315	0.563388814	0.750868206	0.204589542
ENSMUSG00000022762	Ncam2	17968	protein_coding	2.610747217	0.20459884	1.017737255	-0.201033065	0.84067272	1	0.20459884
ENSMUSG00000034998	Foxn2	14236	protein_coding	295.7868371	-0.204606586	0.125810224	1.626311284	0.103883453	0.254529872	0.204606586
ENSMUSG00000004789	Dlst	78920	protein_coding	2117.436162	-0.204672331	0.046726582	4.380211867	1.19E-05	0.000130357	0.204672331
ENSMUSG00000017548	Suz12	52615	protein_coding	665.0752688	0.204687387	0.107501831	-1.904036287	0.056905458	0.16428964	0.204687387
ENSMUSG00000029093	Sorcs2	81840	protein_coding	212.3508389	0.204757325	0.17119009	-1.19608165	0.231664698	0.440496352	0.204757325
ENSMUSG00000034022	Cpsf1	94230	protein_coding	917.256618	0.204784486	0.070532388	-2.903410663	0.003691223	0.01893658	0.204784486
ENSMUSG00000026568	Mpc2	70456	protein_coding	804.5309891	-0.204788994	0.056848509	3.602363492	0.000315337	0.002381578	0.204788994
ENSMUSG00000039328	Rnf122	68867	protein_coding	32.63764557	-0.204811839	0.233212589	0.878219482	0.379824622	0.602075619	0.204811839
ENSMUSG00000061374	Fiz1	23877	protein_coding	290.306065	0.204891219	0.071454964	-2.86741755	0.004138366	0.020759714	0.204891219
ENSMUSG00000032360	Hcrtr2	387285	protein_coding	1.9351828	-0.204989019	1.574825439	0.130166185	0.896434948	1	0.204989019
ENSMUSG00000091264	Smim13	108934	protein_coding	144.036121	-0.204991241	0.107142709	1.913254233	0.055715514	0.161560315	0.204991241
ENSMUSG00000009670	Tex11	83558	protein_coding	1.238471737	0.20506807	1.441110905	-0.142298604	0.886844149	1	0.20506807
ENSMUSG00000028234	Rps20	67427	protein_coding	4198.758247	-0.205123907	0.102689612	1.997513696	0.045769408	0.139401698	0.205123907
ENSMUSG00000028228	Cpne3	70568	protein_coding	1555.022533	-0.205168325	0.10024275	2.046714856	0.04068609	0.127638694	0.205168325
ENSMUSG00000021038	Vipas39	104799	protein_coding	701.7430237	-0.20538029	0.068912603	2.980300845	0.002879654	0.015369945	0.20538029
ENSMUSG00000040841	Six5	20475	protein_coding	58.5653385	0.205409627	0.225229637	-0.91200088	0.361768254	0.584163582	0.205409627

ensembl_gene_id	external_gene_name	entrezgene_id	gene_biotype	baseMean	log2FoldChange	lfcSE	stat	pvalue	padj	absLog2FC
ENSMUSG00000005087	Cd44	12505	protein_coding	496.5265804	0.205437056	0.197758354	-1.038828712	0.298884405	0.518790947	0.205437056
ENSMUSG00000114649	3110006O06Rik	73093	lncRNA	2.548999809	0.205472176	1.544164987	-0.133063615	0.89414307	1	0.205472176
ENSMUSG00000059022	Kcp	333088	protein_coding	3.291207224	0.205546605	1.18587215	-0.173329481	0.862392452	1	0.205546605
ENSMUSG00000038816	Ctnnal1	54366	protein_coding	203.9524693	-0.205562702	0.153548315	1.338749313	0.180652297	0.374503142	0.205562702
ENSMUSG00000020525	Ppm1d	53892	protein_coding	147.3267132	-0.205594074	0.09706466	2.118114604	0.034165363	0.11137126	0.205594074
ENSMUSG00000020561	Polr1f	28071	protein_coding	196.3199756	0.20560435	0.132960488	-1.546356756	0.122018432	0.285089471	0.20560435
ENSMUSG00000024601	Isoc1	66307	protein_coding	531.4508305	-0.205626265	0.123465587	1.665454072	0.095822199	0.239504737	0.205626265
ENSMUSG00000005057	Sh2b2	23921	protein_coding	6.22385315	-0.205670149	0.900414004	0.228417314	0.81932183	0.908985828	0.205670149
ENSMUSG00000026398	Nr5a2	26424	protein_coding	1303.449784	-0.205762785	0.066631589	3.088066609	0.002014633	0.011401366	0.205762785
ENSMUSG00000032411	Tfdp2	211586	protein_coding	676.1181821	0.205773809	0.10066949	-2.044053365	0.040948275	0.128287829	0.205773809
ENSMUSG00000071256	Zfp213	449521	protein_coding	147.0426323	0.20578974	0.142522097	-1.443914622	0.148762993	0.327599956	0.20578974
ENSMUSG00000055900	Tmem69	230657	protein_coding	106.4279622	-0.205835852	0.118944097	1.730526006	0.083536339	0.216667049	0.205835852
ENSMUSG00000034345	Gtf2h5	66467	protein_coding	288.7923274	0.20588449	0.104112325	-1.977522732	0.047982574	0.144595661	0.20588449
ENSMUSG00000035873	Pawr	114774	protein_coding	543.7583012	0.205887907	0.079094431	-2.603064523	0.009239456	0.039961181	0.205887907
ENSMUSG00000025510	Cd151	12476	protein_coding	1394.274901	-0.205911825	0.054361451	3.787827968	0.00015197	0.001242392	0.205911825
ENSMUSG00000002803	Btbd6	399566	protein_coding	249.165376	-0.205913948	0.088075229	2.337932582	0.019390746	0.071685421	0.205913948
ENSMUSG00000023235	Ccl25	20300	protein_coding	71.47910292	-0.205960895	0.19122051	1.077085794	0.281441939	0.499165737	0.205960895
ENSMUSG00000031148	Gpkow	209416	protein_coding	243.2497155	-0.206048286	0.08312496	2.478777563	0.013183349	0.052790938	0.206048286
ENSMUSG00000049489	Ccnq	69109	protein_coding	126.4359471	-0.206116429	0.147150995	1.400713799	0.161299674	0.346267391	0.206116429
ENSMUSG00000030681	Mvp	78388	protein_coding	5262.323786	-0.206183586	0.084261715	2.446942676	0.014407375	0.056473628	0.206183586
ENSMUSG00000029312	Klhl8	246293	protein_coding	54.18650627	0.206305236	0.203276291	-1.01490063	0.310153198	0.531259196	0.206305236
ENSMUSG00000000690	Hoxb6	15414	protein_coding	195.9673662	-0.20637576	0.267268359	0.772166825	0.440015628	0.657892085	0.20637576
ENSMUSG00000028339	Col15a1	12819	protein_coding	25.42196414	-0.206398129	0.402884517	0.51230097	0.608440387	0.782626881	0.206398129
ENSMUSG00000034613	Ppm1h	319468	protein_coding	731.8127045	-0.20649176	0.04793075	4.308127074	1.65E-05	0.000173843	0.20649176
ENSMUSG00000041028	Ghitm	66092	protein_coding	3596.526255	-0.206507975	0.052841777	3.908043715	9.30E-05	0.000809147	0.206507975
ENSMUSG00000049285	Mblac1	330216	protein_coding	32.11125278	-0.206511157	0.197843177	1.043812378	0.296572204	0.515753304	0.206511157
ENSMUSG00000028619	Tceanc2	66526	protein_coding	202.2862231	-0.206523212	0.089578648	2.305495964	0.021138813	0.076654886	0.206523212
ENSMUSG00000072763	5430403G16Rik	77200	protein_coding	23.04182159	-0.206556857	0.233927067	0.882996823	0.377237988	0.599511678	0.206556857
ENSMUSG00000041530	Ago1	236511	protein_coding	454.6063499	-0.206617782	0.099950114	2.06720907	0.038714458	0.122877294	0.206617782
ENSMUSG00000004798	Ulk2	29869	protein_coding	240.2942813	0.206631235	0.091097359	-2.26824618	0.023314206	0.082744638	0.206631235
ENSMUSG00000112503	Gm18409	100417125	transcribed_processed_pseudogene	1.048005245	-0.206662656	1.473628462	0.140240679	0.888469832	1	0.206662656
ENSMUSG00000086896	Cyp4x1os	105244662	lncRNA	0.889061718	0.206677864	1.759107218	-0.117490203	0.906471607	1	0.206677864
ENSMUSG00000048490	Nrip1	268903	protein_coding	1634.877353	-0.206714916	0.065911164	3.136265597	0.001711142	0.009942925	0.206714916
ENSMUSG00000000308	Ckmt1	12716	protein_coding	20666.60126	-0.206748307	0.16326732	1.266317759	0.205399341	0.408031364	0.206748307
ENSMUSG00000038268	Ovca2	246257	protein_coding	99.58783479	-0.206751445	0.130381207	1.585745743	0.112796998	0.269506609	0.206751445
ENSMUSG00000051451	Crebzf	233490	protein_coding	554.0778256	0.206758492	0.08440822	-2.449506602	0.014305209	0.056179837	0.206758492
ENSMUSG00000093046	Mir3081	100526478	miRNA	3.10805031	0.206824022	0.738887574	-0.279912709	0.779544477	1	0.206824022
ENSMUSG00000050697	Prkaa1	105787	protein_coding	880.2944289	-0.206853265	0.079157611	2.613182284	0.008970344	0.039003757	0.206853265
ENSMUSG00000032382	Snx1	56440	protein_coding	1394.478593	-0.206881826	0.064282319	3.218331707	0.001289386	0.007860067	0.206881826

Supplementary Table 1

ensembl_gene_id	external_gene_name	entrezgene_id	gene_biotype	baseMean	log2FoldChange	lfcSE	stat	pvalue	padj	absLog2FC
ENSMUSG00000042428	Mgat3	17309	protein_coding	1908.2898	0.206885104	0.055161023	-3.750566847	0.000176435	0.001419217	0.206885104
ENSMUSG00000002109	Ddb2	107986	protein_coding	72.44921646	-0.206984326	0.125384914	1.650791314	0.098781194	0.24534105	0.206984326
ENSMUSG00000005677	Nr1i3	12355	protein_coding	10.5371726	0.206984762	0.37747649	-0.548338155	0.583459733	0.766020418	0.206984762
ENSMUSG00000031093	Dock11	75974	protein_coding	138.9536522	-0.207000826	0.139898449	1.479650614	0.138966512	0.312060508	0.207000826
ENSMUSG00000043311	D17H6S53E	114585	protein_coding	401.9062859	-0.207029614	0.060879493	3.400646187	0.000672268	0.00453877	0.207029614
ENSMUSG00000063488	Zkscan7	382118	protein_coding	170.4805137	-0.207092189	0.088349776	2.344003541	0.01907799	0.070735054	0.207092189
ENSMUSG00000049076	Acap2	78618	protein_coding	1072.740486	-0.207100257	0.096232255	2.152087738	0.031390445	0.104512461	0.207100257
ENSMUSG00000027601	Mtfr1	67472	protein_coding	519.3396278	-0.207294188	0.07238311	2.863847501	0.004185295	0.020925206	0.207294188
ENSMUSG00000117042	2700054A10Rik	72578	lncRNA	14.77129315	0.207315338	0.308781755	-0.671397628	0.501967256	0.706691156	0.207315338
ENSMUSG00000032028	Nxpe2	78252	protein_coding	1115.157147	0.207419392	0.401614609	-0.516463763	0.605530542	0.780450414	0.207419392
ENSMUSG00000000942	Hoxa4	15401	protein_coding	8.188936131	-0.207424078	0.371064091	0.558997983	0.576163097	0.761180591	0.207424078
ENSMUSG00000025503	Taldo1	21351	protein_coding	3564.379783	-0.207428904	0.05969615	3.474745087	0.000511339	0.003617932	0.207428904
ENSMUSG00000021278	Amn	93835	protein_coding	101.6828334	0.20743774	0.251504523	-0.824787316	0.409492349	0.630052795	0.20743774
ENSMUSG00000090641	Zfp712	78251	protein_coding	44.95270168	-0.207454343	0.159183107	1.303243458	0.192491658	0.390229222	0.207454343
ENSMUSG00000048373	Fgfbp1	14181	protein_coding	1346.403658	0.207457459	0.110272633	-1.881314088	0.059929206	0.1706035	0.207457459
ENSMUSG00000039850	Endov	338371	protein_coding	221.1012313	-0.207492062	0.099191462	2.09183388	0.036453378	0.117005071	0.207492062
ENSMUSG00000027722	Spata5	57815	protein_coding	204.3676331	-0.207697153	0.13012507	1.596134807	0.110458699	0.265751013	0.207697153
ENSMUSG00000006642	Tcf23	69852	protein_coding	51.30864262	0.207715901	0.18788101	-1.105571557	0.268912006	0.484040442	0.207715901
ENSMUSG00000025862	Stag2	20843	protein_coding	1553.464545	-0.207737079	0.082104067	2.530167959	0.011400794	0.0473047	0.207737079
ENSMUSG00000053985	Zfp14	243906	protein_coding	24.20739645	-0.207805155	0.211754851	0.98134779	0.326421263	0.548343875	0.207805155
ENSMUSG00000031226	Pbdc1	67683	protein_coding	241.2586578	0.207911922	0.131291648	-1.58358833	0.113287431	0.270127107	0.207911922
ENSMUSG00000019158	Tmem160	69094	protein_coding	147.6660645	-0.207931635	0.129004047	1.611822579	0.107000553	0.260075004	0.207931635
ENSMUSG00000035242	Oaz1	18245	protein_coding	1681.83226	-0.207967974	0.117436371	1.770899189	0.076577467	0.203672136	0.207967974
ENSMUSG00000032530	Lyzl4	69032	protein_coding	20.7651797	0.20800613	0.323121998	-0.643738683	0.519744901	0.719935187	0.20800613
ENSMUSG00000065304	Gm23245	115490273	snoRNA	6.466674523	0.208082124	0.437635612	-0.475468902	0.634452793	0.800714821	0.208082124
ENSMUSG00000039157	Fam102a	98952	protein_coding	1615.432752	-0.208098	0.124322102	1.673861666	0.09415779	0.236336338	0.208098
ENSMUSG00000021061	Sptb	20741	protein_coding	41.58560082	-0.208103374	0.306283905	0.679445999	0.496855312	0.702910017	0.208103374
ENSMUSG00000033187	BC016579	212998	protein_coding	416.7133934	0.208201138	0.090826076	-2.292305764	0.021888005	0.07883501	0.208201138
ENSMUSG00000017144	Rnd3	74194	protein_coding	708.3211325	-0.208203945	0.166694968	1.249011573	0.211660841	0.416065952	0.208203945
ENSMUSG00000031608	Galnt7	108150	protein_coding	3285.135978	0.208262201	0.163888461	-1.270755731	0.203815562	0.406008467	0.208262201
ENSMUSG00000030956	Fam53b	77938	protein_coding	330.0124419	-0.208314742	0.093804874	2.220724079	0.026369655	0.090987984	0.208314742
ENSMUSG00000000532	Acvr1b	11479	protein_coding	1416.073448	-0.208322326	0.077532493	2.686903489	0.007211779	0.03273648	0.208322326
ENSMUSG00000021072	Tmx1	72736	protein_coding	853.8944756	0.208391728	0.11998267	-1.736848554	0.082413906	0.214493541	0.208391728
ENSMUSG00000023284	Zfp605	675812	protein_coding	38.9507602	-0.208490169	0.172568346	1.208159976	0.226985734	0.435409735	0.208490169
ENSMUSG00000074165	Zfp788	67607	protein_coding	87.99735588	-0.208493813	0.173314167	1.202981939	0.22898331	0.437918979	0.208493813
ENSMUSG00000052131	Akr1b7	11997	protein_coding	40.79996727	-0.208541884	0.215640617	0.967080723	0.333503681	0.555918017	0.208541884
ENSMUSG00000098184	Gm27011	115486886	lncRNA	3.560601229	0.208577349	0.55378526	-0.376639402	0.706441602	1	0.208577349
ENSMUSG00000114613	Gm10732	100038690	lncRNA	11.55635613	0.208581517	0.325555715	-0.640693766	0.521721665	0.721161093	0.208581517
ENSMUSG00000042207	Kdm5b	75605	protein_coding	986.3470608	-0.20861463	0.0674074	3.094832747	0.001969239	0.011209933	0.20861463

Supplementary Table 1

ensembl_gene_id	external_gene_name	entrezgene_id	gene_biotype	baseMean	log2FoldChange	lfcSE	stat	pvalue	padj	absLog2FC
ENSMUSG00000034042	Gpbp1l1	77110	protein_coding	1823.904987	-0.20878117	0.065310306	3.196756853	0.00138982	0.008373401	0.20878117
ENSMUSG00000042807	Hecw2	329152	protein_coding	6.586202508	-0.208793391	0.871761039	0.239507596	0.810712007	0.906297546	0.208793391
ENSMUSG00000031808	Slc27a1	26457	protein_coding	329.5812887	-0.208807856	0.11456553	1.822606289	0.068363042	0.187813556	0.208807856
ENSMUSG00000032477	Cdc25a	12530	protein_coding	658.6607289	0.208944955	0.101475055	-2.059077034	0.039486859	0.124778744	0.208944955
ENSMUSG00000078681	Tm2d3	68634	protein_coding	278.0047087	-0.208957782	0.120441904	1.734925929	0.082753926	0.215212336	0.208957782
ENSMUSG00000072844	G530011O06Rik	654820	lncRNA	3.095660523	-0.20897905	0.739162714	0.282724014	0.777388406	1	0.20897905
ENSMUSG00000089214	Mir1931	100316689	miRNA	1.157888644	-0.208989211	1.184711852	0.176405098	0.8599757	1	0.208989211
ENSMUSG00000047989	Ino80c	225280	protein_coding	286.6083973	0.209001466	0.150165216	-1.39181011	0.163979908	0.350228168	0.209001466
ENSMUSG00000001761	Smo	319757	protein_coding	337.6317393	0.209003491	0.118642168	-1.761629067	0.07813199	0.206674944	0.209003491
ENSMUSG00000083674	Zfp133-ps	668917	transcribed_unitary_pseudogene	6.779029773	-0.209009054	0.464483465	0.449981688	0.652723644	0.811601896	0.209009054
ENSMUSG00000035929	H2-Q4	15015	protein_coding	209.9076726	0.20906416	0.135937674	-1.537941272	0.124062975	0.288478734	0.20906416
ENSMUSG00000072946	Ptgr2	77219	protein_coding	505.755306	-0.209076631	0.080199359	2.606961378	0.009134966	0.03960484	0.209076631
ENSMUSG00000058248	Kcnh1	16510	protein_coding	16.9023302	-0.209118626	0.46308753	0.451574729	0.651575379	0.810851996	0.209118626
ENSMUSG00000074466	Gm15417	545539	lncRNA	63.45001478	0.209146019	0.185254487	-1.128966015	0.25891217	0.472341459	0.209146019
ENSMUSG00000060862	Zbtb40	230848	protein_coding	294.9972238	0.209167938	0.122153017	-1.712343599	0.086833366	0.223150671	0.209167938
ENSMUSG00000002997	Prkar2b	19088	protein_coding	44.73563027	0.209205883	0.251444164	-0.832017253	0.405399206	0.62645534	0.209205883
ENSMUSG00000085208	Brip1os	74038	lncRNA	765.0448961	0.209234744	0.105427237	-1.984636513	0.047184927	0.142788208	0.209234744
ENSMUSG00000055762	Eef1d	66656	protein_coding	1426.872211	0.209293048	0.084879215	-2.465775017	0.013671718	0.054259732	0.209293048
ENSMUSG00000034853	Acot11	329910	protein_coding	931.1586209	-0.209320032	0.085198067	2.456863641	0.014015587	0.055305475	0.209320032
ENSMUSG00000089239	Gm22200	115489001	snoRNA	1.124106392	0.209359243	1.041889755	-0.200941839	0.840744052	1	0.209359243
ENSMUSG00000019868	Vta1	66201	protein_coding	361.4492776	0.209502614	0.085932254	-2.437997427	0.014768879	0.057630735	0.209502614
ENSMUSG00000022543	Dnaaf8	74684	transcribed_unitary_pseudogene	12.4420275	0.209532359	0.306993503	-0.682530273	0.494903707	0.70086955	0.209532359
ENSMUSG00000029276	Glmn	170823	protein_coding	46.26622113	0.209542592	0.203167292	-1.031379556	0.302362864	0.522795554	0.209542592
ENSMUSG00000038463	Olfml2b	320078	protein_coding	2.180191761	0.209544901	0.797627352	-0.262710275	0.792773897	1	0.209544901
ENSMUSG00000005225	Plekha8	231999	protein_coding	1176.084052	-0.209579068	0.053157538	3.942602978	8.06E-05	0.000714089	0.209579068
ENSMUSG00000038069	Cdkn2aip	70925	protein_coding	206.6587952	-0.209609606	0.094333383	2.222008794	0.026282711	0.090782827	0.209609606
ENSMUSG00000026277	Stk25	59041	protein_coding	2623.700028	-0.209617363	0.095933384	2.185030435	0.028886615	0.097993747	0.209617363
ENSMUSG00000057367	Birc2	11797	protein_coding	485.0603058	0.209677081	0.085089599	-2.464191667	0.013732266	0.054421559	0.209677081
ENSMUSG00000037001	Zfp39	22698	protein_coding	52.30052857	-0.209687226	0.175123286	1.197369181	0.231162691	0.439947064	0.209687226
ENSMUSG00000028825	Rhd	19746	protein_coding	1.008669881	0.209703173	1.25284208	-0.167381968	0.867069512	1	0.209703173
ENSMUSG00000006818	Sod2	20656	protein_coding	1145.691561	-0.209705163	0.070904511	2.957571525	0.003100728	0.01632826	0.209705163
ENSMUSG00000116220	A430088P11Rik	106059	lncRNA	21.26649393	-0.209774966	0.252639608	0.830332853	0.406350618	0.62720573	0.209774966
ENSMUSG00000029068	Ccnl2	56036	protein_coding	1269.798626	0.209822827	0.120409602	-1.742575545	0.081407785	0.212413992	0.209822827
ENSMUSG00000026600	Soat1	20652	protein_coding	760.0751491	-0.209833715	0.18154282	1.155835932	0.247748273	0.45990501	0.209833715
ENSMUSG00000030105	Arl8b	67166	protein_coding	956.0467507	0.209863442	0.059777538	-3.510740829	0.00044686	0.003226691	0.209863442
ENSMUSG00000039853	Trim14	74735	protein_coding	188.529233	-0.2099016	0.086238801	2.433957784	0.014934737	0.058209168	0.2099016
ENSMUSG00000029101	Rgs12	71729	protein_coding	68.43575291	0.209919485	0.166022954	-1.264400372	0.206086359	0.408658758	0.209919485
ENSMUSG00000037845	Fdxacb1	382137	protein_coding	49.29478893	-0.20993285	0.161689031	1.298374098	0.194158815	0.392885514	0.20993285
ENSMUSG00000049119	Fam110b	242297	protein_coding	15.78287163	-0.209956646	0.315047274	0.666429021	0.505136918	0.709218761	0.209956646

ensembl_gene_id	external_gene_name	entrezgene_id	gene_biotype	baseMean	log2FoldChange	lfcSE	stat	pvalue	padj	absLog2FC
ENSMUSG00000029104	Htt	15194	protein_coding	1133.20288	0.209984163	0.0617342	-3.401423551	0.000670359	0.004529584	0.209984163
ENSMUSG00000019779	Frk	14302	protein_coding	1425.054743	-0.210016851	0.082300245	2.551837499	0.010715648	0.045027536	0.210016851
ENSMUSG00000063052	Lrrc40	67144	protein_coding	315.6628322	0.210094154	0.11643566	-1.804379807	0.071171786	0.1933428	0.210094154
ENSMUSG00000100334	C230024C17Rik	329271	lncRNA	1.39172506	-0.210115282	1.514351122	0.138749382	0.889648194	1	0.210115282
ENSMUSG00000062949	Atp11c	320940	protein_coding	295.9548987	0.210178365	0.090030579	-2.334521983	0.019568406	0.072116193	0.210178365
ENSMUSG00000002365	Snx9	66616	protein_coding	973.715712	-0.210181756	0.076055473	2.763532281	0.005717944	0.027204299	0.210181756
ENSMUSG00000025188	Hps1	192236	protein_coding	224.0090569	-0.210219103	0.103884343	2.023587938	0.043012567	0.132867477	0.210219103
ENSMUSG00000030337	Vamp1	22317	protein_coding	124.9562162	0.210257245	0.186478231	-1.1275163	0.259524253	0.472954995	0.210257245
ENSMUSG00000030096	Slc6a6	21366	protein_coding	1820.535582	-0.210258143	0.081647583	2.575191273	0.010018467	0.042673448	0.210258143
ENSMUSG00000020122	Egfr	13649	protein_coding	657.5154683	-0.210287316	0.071400866	2.945164792	0.003227827	0.01691664	0.210287316
ENSMUSG00000071267	Zfp942	73233	protein_coding	227.9632678	-0.210371738	0.084936916	2.476799805	0.013256624	0.053020073	0.210371738
ENSMUSG00000060176	Kif27	75050	protein_coding	31.74037844	-0.210378264	0.295607036	0.711682195	0.476661596	0.68833522	0.210378264
ENSMUSG00000043639	Rbm20	73713	protein_coding	53.07914798	-0.210387907	0.152517045	1.379438652	0.167759549	0.356273302	0.210387907
ENSMUSG00000013539	Tango2	27883	protein_coding	198.6100624	0.210400062	0.088909099	-2.366462658	0.017958986	0.067342119	0.210400062
ENSMUSG00000040746	Rnf167	70510	protein_coding	583.0663972	-0.210534138	0.068537992	3.071787382	0.002127812	0.011930684	0.210534138
ENSMUSG00000075184	F930017D23Rik	414125	lncRNA	2.898767311	0.210604882	0.69374657	-0.303576106	0.761450858	1	0.210604882
ENSMUSG00000022584	Ly6c2	100041546	protein_coding	3.649709887	0.210619312	1.008171375	-0.208912212	0.83451677	1	0.210619312
ENSMUSG00000027744	Stoml3	229277	protein_coding	1.461318199	0.210626998	2.071065045	-0.101699847	0.918994924	1	0.210626998
ENSMUSG00000005533	Igf1r	16001	protein_coding	234.7836478	-0.210690577	0.103300397	2.039591158	0.041391063	0.129309879	0.210690577
ENSMUSG00000027624	Epb41l1	13821	protein_coding	977.6629875	-0.210715183	0.101028099	2.085708688	0.037005019	0.118476325	0.210715183
ENSMUSG00000002996	Hbp1	73389	protein_coding	471.840677	-0.210794757	0.140845612	1.49663702	0.134487732	0.305198626	0.210794757
ENSMUSG00000020877	Scrn2	217140	protein_coding	197.1588123	-0.210806363	0.08109543	2.599485117	0.009336373	0.040329809	0.210806363
ENSMUSG00000027589	Pcmtd2	245867	protein_coding	534.0745173	-0.210903127	0.120194446	1.754682803	0.079313579	0.208654479	0.210903127
ENSMUSG00000030861	Acadsb	66885	protein_coding	272.1895655	-0.21090754	0.132400578	1.592950294	0.111171342	0.26676599	0.21090754
ENSMUSG00000028706	Nsun4	72181	protein_coding	140.4189279	-0.210923407	0.106275067	1.984693244	0.047178611	0.142788208	0.210923407
ENSMUSG00000037343	Taf2	319944	protein_coding	449.6341745	0.210957105	0.063976885	-3.29739569	0.000975859	0.006239	0.210957105
ENSMUSG00000023017	Asic1	11419	protein_coding	47.06770427	-0.211041736	0.15937937	1.324147136	0.185454184	0.380783029	0.211041736
ENSMUSG00000052504	Epha3	13837	protein_coding	3.18078286	-0.211102461	0.959396272	0.220036774	0.825842515	1	0.211102461
ENSMUSG00000052293	Taf9	108143	protein_coding	1.378840637	-0.211122395	0.942960294	0.223893197	0.82284041	1	0.211122395
ENSMUSG00000028838	Extl1	56219	protein_coding	13.56566019	0.211144467	0.336976024	-0.626586025	0.530930631	0.728048657	0.211144467
ENSMUSG00000038302	Afg1l	215951	protein_coding	359.9376698	-0.211282593	0.082886691	2.5490533	0.010801579	0.045254367	0.211282593
ENSMUSG00000028145	Them4	75778	protein_coding	92.23787718	-0.211301966	0.131469807	1.607228078	0.108004345	0.261594876	0.211301966
ENSMUSG00000064247	Plcxd1	403178	protein_coding	13.3442995	-0.211312571	0.358294205	0.589773901	0.555342242	0.745058233	0.211312571
ENSMUSG00000023912	Slc25a27	74011	protein_coding	12.82463405	-0.211342791	0.328599704	0.643161843	0.52011909	0.72021186	0.211342791
ENSMUSG00000052889	Prkcb	18751	protein_coding	7.335619186	-0.211397181	0.719865681	0.293661979	0.76901621	0.884245488	0.211397181
ENSMUSG00000030555	Ttc23	67009	protein_coding	144.7492363	0.211430048	0.083982618	-2.517545334	0.011817576	0.048509931	0.211430048
ENSMUSG00000028699	Tspan1	66805	protein_coding	13305.48047	-0.211514293	0.085898349	2.462379016	0.013801873	0.05461877	0.211514293
ENSMUSG00000000959	Oxa1l	69089	protein_coding	630.3510781	-0.211525832	0.086292794	2.451257187	0.01423582	0.055973887	0.211525832
ENSMUSG00000036241	Ube2r2	67615	protein_coding	890.0589716	-0.211556805	0.08643015	2.447719983	0.014376334	0.056392107	0.211556805

ensembl_gene_id	external_gene_name	entrezgene_id	gene_biotype	baseMean	log2FoldChange	lfcSE	stat	pvalue	padj	absLog2FC
ENSMUSG00000003868	Ruvbl2	20174	protein_coding	598.1396622	0.211559989	0.098702512	-2.143410384	0.03208016	0.106287366	0.211559989
ENSMUSG00000035051	Dhx57	106794	protein_coding	287.1984503	0.211724449	0.06602234	-3.206860721	0.001341919	0.008129295	0.211724449
ENSMUSG00000030089	Slc41a3	71699	protein_coding	25.89017749	0.211791925	0.224817672	-0.942060843	0.346161491	0.567902926	0.211791925
ENSMUSG00000016496	Cd274	60533	protein_coding	22.9978959	-0.211807789	0.277357862	0.763662465	0.445068414	0.662372535	0.211807789
ENSMUSG00000045374	Wdr81	192652	protein_coding	406.8684636	-0.211839846	0.071659304	2.956208524	0.003114464	0.016390142	0.211839846
ENSMUSG00000003271	Sult2b1	54200	protein_coding	226.6600151	-0.211897405	0.175930304	1.204439485	0.228419758	0.437296996	0.211897405
ENSMUSG00000035778	Ggta1	14594	protein_coding	4.873030973	0.211898521	0.768892726	-0.275589187	0.782863616	0.891186777	0.211898521
ENSMUSG00000012535	Tnpo3	320938	protein_coding	983.6304492	0.211917761	0.080051234	-2.647276638	0.008114294	0.036011679	0.211917761
ENSMUSG00000037363	Letm2	270035	protein_coding	19.45939686	0.21192054	0.303186776	-0.698976858	0.484566493	0.695051774	0.21192054
ENSMUSG00000031430	Vsig1	78789	protein_coding	86.60410586	0.211923331	0.31028291	-0.683000334	0.494606631	0.700749306	0.211923331
ENSMUSG00000067873	Htatsf1	72459	protein_coding	417.1381847	0.211956075	0.090866115	-2.332619547	0.019668122	0.07241317	0.211956075
ENSMUSG00000064128	Cenpj	219103	protein_coding	67.83136536	0.211956891	0.132667062	-1.597660255	0.110118609	0.265192684	0.211956891
ENSMUSG00000071192	Wfikkn1	215001	protein_coding	17.86020514	0.212049342	0.312435699	-0.678697546	0.497329521	0.702918497	0.212049342
ENSMUSG00000039737	Prkrip1	66801	protein_coding	176.9186959	-0.212052646	0.096614089	2.194841842	0.028174945	0.095954019	0.212052646
ENSMUSG00000046329	Slc25a23	66972	protein_coding	125.473777	0.212075495	0.160730649	-1.319446516	0.187019879	0.383134386	0.212075495
ENSMUSG00000003929	Zfp81	224694	protein_coding	108.6522589	-0.212085531	0.143222999	1.480806383	0.138658175	0.311717339	0.212085531
ENSMUSG00000038538	Ubn2	320538	protein_coding	627.1263135	0.212090516	0.109233534	-1.941624587	0.052182564	0.154268325	0.212090516
ENSMUSG00000067787	Blcap	53619	protein_coding	342.739281	-0.21211707	0.098875176	2.145301567	0.031928743	0.105941585	0.21211707
ENSMUSG00000021185	Dglucy	217830	protein_coding	359.8030972	0.212248403	0.097953865	-2.166820087	0.030248575	0.101632751	0.212248403
ENSMUSG00000043008	Klhl6	239743	protein_coding	3.949150141	0.212325548	0.672263047	-0.315837007	0.752126228	1	0.212325548
ENSMUSG00000084883	Ccdc85c	668158	protein_coding	1333.350434	0.212380865	0.089594698	-2.370462431	0.017765849	0.066769512	0.212380865
ENSMUSG00000084845	Tmem240	381582	protein_coding	5.388108631	-0.212399623	1.043064255	0.20363043	0.838642311	0.919610832	0.212399623
ENSMUSG00000032235	Ice2	93697	protein_coding	179.3292818	-0.21243441	0.095013247	2.235839906	0.02536226	0.08834262	0.21243441
ENSMUSG00000021752	Kctd6	71393	protein_coding	83.97150941	-0.212458934	0.115639064	1.837259197	0.06617164	0.183322799	0.212458934
ENSMUSG00000031885	Cbfb	12400	protein_coding	534.0730638	0.212476646	0.077839125	-2.729689536	0.0063394	0.029324606	0.212476646
ENSMUSG00000019338	Zfp687	78266	protein_coding	474.6390035	0.21251164	0.066671121	-3.187461615	0.001435275	0.008569101	0.21251164
ENSMUSG00000030588	Yif1b	77254	protein_coding	294.9339213	0.212587654	0.121747496	-1.746135734	0.080787369	0.21130879	0.212587654
ENSMUSG00000021245	Mlh3	217716	protein_coding	168.8800542	0.212711077	0.115951642	-1.834480936	0.066582635	0.184147653	0.212711077
ENSMUSG00000051790	Nlgn2	216856	protein_coding	28.66968994	0.212713872	0.432255364	-0.492102329	0.622647006	0.793088519	0.212713872
ENSMUSG00000032295	Man2c1	73744	protein_coding	458.7438721	-0.212745211	0.061334851	3.468586085	0.000523205	0.003686094	0.212745211
ENSMUSG00000049439	Cyp20a1	77951	protein_coding	245.4383426	0.212746581	0.158775004	-1.339924899	0.180269762	0.373991816	0.212746581
ENSMUSG00000027253	Lrp4	228357	protein_coding	738.5799724	-0.212780098	0.079636378	2.671895739	0.007542408	0.03390183	0.212780098
ENSMUSG00000025742	Prps2	110639	protein_coding	498.215185	-0.212953285	0.149224826	1.427063381	0.153561626	0.334862894	0.212953285
ENSMUSG00000038604	Ripor1	75687	protein_coding	68.96785528	-0.212969173	0.21786414	0.977532021	0.328305833	0.55048001	0.212969173
ENSMUSG00000097091	4930526L06Rik	75138	lncRNA	0.603910236	-0.212971876	1.461890278	0.145682531	0.884172012	1	0.212971876
ENSMUSG00000051502	Ufsp1	70240	protein_coding	50.43454267	-0.21304229	0.216564089	0.983737848	0.325244426	0.547052145	0.21304229
ENSMUSG00000025487	Psmd13	23997	protein_coding	889.6202986	0.213076246	0.064213816	-3.318230573	0.000905897	0.005852887	0.213076246
ENSMUSG00000027376	Prom2	192212	protein_coding	3.749898875	0.213096806	1.591851693	-0.133867248	0.893507549	1	0.213096806
ENSMUSG00000057411	Antkmt	214917	protein_coding	188.3063755	-0.213123578	0.096661479	2.204845005	0.027464973	0.09404034	0.213123578

ensembl_gene_id	external_gene_name	entrezgene_id	gene_biotype	baseMean	log2FoldChange	lfcSE	stat	pvalue	padj	absLog2FC
ENSMUSG00000031517	Gpm6a	234267	protein_coding	0.805656837	0.213204618	1.395955444	-0.152730246	0.878611008	1	0.213204618
ENSMUSG00000055923	Aasdh	231326	protein_coding	105.6313234	-0.213207738	0.120369786	1.771272882	0.076515335	0.203572456	0.213207738
ENSMUSG00000020839	Tmigd1	66601	protein_coding	708.8029619	0.213235384	0.214829799	-0.99257824	0.320915533	0.542047573	0.213235384
ENSMUSG00000021918	Nek4	23955	protein_coding	218.3299937	0.213292478	0.103269435	-2.065397936	0.038885364	0.123253915	0.213292478
ENSMUSG00000029918	Mrps33	14548	protein_coding	77.91356926	-0.213295131	0.152516166	1.398508344	0.161960469	0.347172272	0.213295131
ENSMUSG00000020514	Mrpl22	216767	protein_coding	150.0328645	-0.213305022	0.112260936	1.900082344	0.057422314	0.165344954	0.213305022
ENSMUSG00000024875	Yif1a	68090	protein_coding	835.8305966	0.213337242	0.074227625	-2.874094938	0.004051871	0.020375337	0.213337242
ENSMUSG00000079470	Utp14b	195434	protein_coding	1360.485252	-0.213374858	0.148504379	1.436825359	0.150767623	0.330823349	0.213374858
ENSMUSG00000048109	Rbm15	229700	protein_coding	651.6564679	0.21341483	0.065857501	-3.240554637	0.001192974	0.00736753	0.21341483
ENSMUSG00000074656	Eif2s2	67204	protein_coding	2202.84368	-0.213416593	0.127505647	1.673781507	0.094173548	0.236336338	0.213416593
ENSMUSG00000038239	Hrc	15464	protein_coding	25.76938739	-0.213504228	0.445864944	0.478854036	0.632042472	0.799444652	0.213504228
ENSMUSG00000040471	Ggt6	71522	protein_coding	652.5119818	-0.213516817	0.098871835	2.159531249	0.030808976	0.102970942	0.213516817
ENSMUSG00000023961	Enpp4	224794	protein_coding	151.5026769	-0.213765396	0.128866477	1.658813075	0.097153465	0.241953298	0.213765396
ENSMUSG00000022749	Tbc1d23	67581	protein_coding	413.6418274	0.213775139	0.090985248	-2.34955823	0.018795704	0.069955433	0.213775139
ENSMUSG00000086424	Gm15569	102638208	lncRNA	2.105176772	-0.213777194	0.975651621	0.219112221	0.826562631	1	0.213777194
ENSMUSG00000024730	Ms4a8a	64381	protein_coding	4049.477522	-0.213784696	0.073199596	2.920572064	0.003493894	0.018083738	0.213784696
ENSMUSG00000041592	Sdk2	237979	protein_coding	7.301054416	-0.213800064	1.228968708	0.173967053	0.861891355	0.931842966	0.213800064
ENSMUSG00000002006	Pdzd4	245469	protein_coding	18.38897037	0.213813944	0.296602028	-0.720878228	0.47098444	0.684066582	0.213813944
ENSMUSG00000032252	Glce	93683	protein_coding	560.5823529	0.213895075	0.116489593	-1.836173247	0.066332038	0.183669899	0.213895075
ENSMUSG00000093806	Asmt	107626	protein_coding	1.859854988	0.213912542	1.460277445	-0.146487603	0.883536476	1	0.213912542
ENSMUSG00000005299	Letm1	56384	protein_coding	1739.600115	-0.213915551	0.074306798	2.878815353	0.00399172	0.020134166	0.213915551
ENSMUSG00000036073	Galt	14430	protein_coding	220.1748724	-0.213932089	0.073348426	2.916655474	0.003538063	0.018266571	0.213932089
ENSMUSG00000097814	Panct2	329217	lncRNA	24.59897379	0.213961224	0.265990882	-0.804393076	0.421169998	0.640973307	0.213961224
ENSMUSG00000025103	Btbd1	83962	protein_coding	652.2586915	-0.213985996	0.091028078	2.35076915	0.018734653	0.069759652	0.213985996
ENSMUSG00000027111	Itga6	16403	protein_coding	3278.155289	-0.214051926	0.071521013	2.99285364	0.002763823	0.014862184	0.214051926
ENSMUSG00000026389	Steap3	68428	protein_coding	343.2584034	-0.214164316	0.081311681	2.633869001	0.008441805	0.037106194	0.214164316
ENSMUSG00000020661	Dnmt3a	13435	protein_coding	667.1065112	-0.214170615	0.088050162	2.432370466	0.015000356	0.05840978	0.214170615
ENSMUSG00000041831	Sytl3	83672	protein_coding	41.66489984	-0.214174434	0.349820381	0.612241156	0.540378216	0.734651013	0.214174434
ENSMUSG00000036752	Tubb4b	227613	protein_coding	7018.308984	0.214218861	0.096797943	-2.213051798	0.026894075	0.09253023	0.214218861
ENSMUSG00000038482	Tfdp1	21781	protein_coding	658.5221146	0.214222661	0.121869238	-1.757807504	0.078780268	0.207790664	0.214222661
ENSMUSG00000037966	Ninj1	18081	protein_coding	194.1435797	-0.214267032	0.079806279	2.684839264	0.007256469	0.032851834	0.214267032
ENSMUSG00000040022	Rab11fip2	74998	protein_coding	76.04458807	-0.214303435	0.149259967	1.435773033	0.151066936	0.331061353	0.214303435
ENSMUSG00000059112	Or4c52	258790	protein_coding	1.084298549	-0.214345386	2.046639544	0.104730404	0.916589736	1	0.214345386
ENSMUSG00000064967	Gm24405	115487650	snRNA	1.174392592	0.214389654	1.45459771	-0.147387592	0.882826099	1	0.214389654
ENSMUSG00000066842	Hmcn1	545370	protein_coding	421.9518776	0.214412657	0.175013385	-1.225121477	0.220529454	0.426993013	0.214412657
ENSMUSG00000038524	Fchsd1	319262	protein_coding	94.75259267	-0.214466197	0.136054364	1.576327215	0.114950413	0.273253364	0.214466197
ENSMUSG00000010936	Vac14	234729	protein_coding	330.893306	0.21448811	0.068943544	-3.111068802	0.001864115	0.01071493	0.21448811
ENSMUSG00000040356	Skiv2l	108077	protein_coding	1132.095621	0.214503202	0.065785003	-3.26067022	0.001111492	0.006960631	0.214503202
ENSMUSG00000037847	Nmrk1	225994	protein_coding	48.1001834	0.214557823	0.168686233	-1.271934407	0.203396426	0.40571187	0.214557823

ensembl_gene_id	external_gene_name	entrezgene_id	gene_biotype	baseMean	log2FoldChange	lfcSE	stat	pvalue	padj	absLog2FC
ENSMUSG00000018476	Kdm6b	216850	protein_coding	397.2410629	0.214704055	0.275597517	-0.779049308	0.435950669	0.654124055	0.214704055
ENSMUSG00000095620	Csta2	76770	protein_coding	5.714403864	0.214851854	0.788240914	-0.272571304	0.785182773	0.892764273	0.214851854
ENSMUSG00000020918	Kat2a	14534	protein_coding	279.0384518	-0.214922585	0.107509367	1.999105666	0.045596922	0.139132959	0.214922585
ENSMUSG00000086382	Chrna1os	102633437	lncRNA	6.592769829	-0.214923332	0.375169999	0.572869187	0.566733269	0.753213832	0.214923332
ENSMUSG00000041961	Znrf3	407821	protein_coding	161.9108852	-0.214977172	0.128558501	1.672212803	0.094482367	0.236872902	0.214977172
ENSMUSG00000006576	Slc4a3	20536	protein_coding	13.22576273	-0.21498554	0.517232477	0.415645865	0.67766916	0.827306109	0.21498554
ENSMUSG00000055210	Foxd2	17301	protein_coding	714.9593691	0.214993114	0.100747611	-2.133977288	0.03284464	0.108241146	0.214993114
ENSMUSG00000039615	Stub1	56424	protein_coding	408.9604448	0.215018155	0.075987494	-2.82965187	0.004659868	0.022923163	0.215018155
ENSMUSG00000006342	Susd2	71733	protein_coding	5.764627995	0.215060266	0.756227432	-0.284385698	0.776114818	0.888226437	0.215060266
ENSMUSG00000026860	Sh3glb2	227700	protein_coding	726.4911253	-0.215090895	0.080765101	2.663166315	0.007740914	0.034680809	0.215090895
ENSMUSG00000029409	U90926	57425	lncRNA	0.856508635	0.215179619	1.46875409	-0.146504865	0.88352285	1	0.215179619
ENSMUSG00000018999	Slc35b4	58246	protein_coding	246.3249837	0.215243237	0.104770683	-2.054422395	0.039934827	0.125783662	0.215243237
ENSMUSG00000027993	Trim2	80890	protein_coding	4173.692062	-0.215274243	0.077343895	2.783338535	0.005380263	0.025873521	0.215274243
ENSMUSG00000070574	2310016G11Rik	69578	lncRNA	60.31334871	-0.215311655	0.255569014	0.842479498	0.399519611	0.621711048	0.215311655
ENSMUSG00000038641	Akr1d1	208665	protein_coding	0.955054165	-0.215330907	1.286974435	0.167315606	0.867121726	1	0.215330907
ENSMUSG00000085696	Hoxaas3	72628	lncRNA	128.6348398	0.215336826	0.127706324	-1.686187653	0.091759661	0.231983933	0.215336826
ENSMUSG00000007850	Hnrnph1	59013	protein_coding	1972.792708	0.215338231	0.049783095	-4.325529238	1.52E-05	0.000161703	0.215338231
ENSMUSG00000034573	Ptpn13	19249	protein_coding	329.5704706	-0.215384847	0.123225613	1.747890251	0.080483033	0.210900795	0.215384847
ENSMUSG00000006589	Aprt	11821	protein_coding	894.4897775	-0.215392005	0.073873065	2.915704204	0.003548868	0.018308655	0.215392005
ENSMUSG00000006281	Tep1	21745	protein_coding	2204.324474	-0.215507074	0.070136695	3.072672218	0.002121514	0.011900366	0.215507074
ENSMUSG00000027901	Dennd2d	72121	protein_coding	891.9642785	0.215604586	0.093316189	-2.310473557	0.020861951	0.075817398	0.215604586
ENSMUSG00000027655	Dhx35	71715	protein_coding	261.2147952	-0.215632743	0.111682108	1.9307725	0.053511193	0.157015607	0.215632743
ENSMUSG00000006611	Hfe	15216	protein_coding	105.6602328	-0.215655529	0.108352034	1.990322853	0.046555382	0.141278128	0.215655529
ENSMUSG00000043467	Zbtb37	240869	protein_coding	111.5949704	-0.21566214	0.104840557	2.057048783	0.039681534	0.12530374	0.21566214
ENSMUSG00000118346	Tmem179b	67706	protein_coding	176.9657056	-0.215694279	0.093151111	2.315530927	0.020583894	0.075004948	0.215694279
ENSMUSG00000110424	Zbed5-ps	75479	lncRNA	13.89530209	0.215717013	0.376528881	-0.572909606	0.5667059	0.753213832	0.215717013
ENSMUSG00000028337	Coro2a	107684	protein_coding	3492.778217	-0.215733137	0.125830513	1.714473953	0.086441717	0.222351793	0.215733137
ENSMUSG00000054662	Ano9	71345	protein_coding	835.1249309	-0.215758157	0.132585052	1.627318869	0.103669392	0.254141273	0.215758157
ENSMUSG00000063229	Ldha	16828	protein_coding	9330.667281	-0.215785753	0.074195024	2.908358816	0.003633312	0.018683911	0.215785753
ENSMUSG00000059208	Hnrnpm	76936	protein_coding	2163.964709	0.215788923	0.070333765	-3.06807012	0.002154461	0.012036117	0.215788923
ENSMUSG00000024937	Ehbp1l1	114601	protein_coding	727.847952	0.215798718	0.143644966	-1.502306165	0.133018057	0.302752759	0.215798718
ENSMUSG00000033039	Micall1	27008	protein_coding	145.5588945	-0.215816589	0.100339471	2.150864338	0.031486909	0.104749157	0.215816589
ENSMUSG00000046971	Pla2g4f	271844	protein_coding	124.4601281	0.215826651	0.199902969	-1.079657055	0.280294924	0.497933561	0.215826651
ENSMUSG00000027189	Trim44	80985	protein_coding	499.0788136	-0.215903311	0.131470251	1.642221783	0.10054405	0.248746733	0.215903311
ENSMUSG00000030203	Dusp16	70686	protein_coding	407.2800466	-0.215936183	0.118847906	1.816911976	0.069230593	0.189409212	0.215936183
ENSMUSG00000066150	Slc31a1	20529	protein_coding	1213.844122	-0.215956803	0.081811809	2.639677651	0.008298492	0.036642767	0.215956803
ENSMUSG00000029036	Atad3a	108888	protein_coding	685.3869675	-0.215973112	0.07922641	2.726024224	0.006410229	0.02958595	0.215973112
ENSMUSG00000034659	Tmem109	68539	protein_coding	329.1040426	-0.216030968	0.130840555	1.651100969	0.098717959	0.245220876	0.216030968
ENSMUSG00000001827	Folr1	14275	protein_coding	26.07124678	0.216040871	0.257832947	-0.837910259	0.402081106	0.623763963	0.216040871

Supplementary Table 1

ensembl_gene_id	external_gene_name	entrezgene_id	gene_biotype	baseMean	log2FoldChange	lfcSE	stat	pvalue	padj	absLog2FC
ENSMUSG00000028464	Tpm2	22004	protein_coding	43.54387531	-0.216059168	0.348126304	0.620634424	0.534840185	0.730731431	0.216059168
ENSMUSG00000007564	Ppp2r1a	51792	protein_coding	3157.046018	0.216107751	0.062370661	-3.464894326	0.00053044	0.00373044	0.216107751
ENSMUSG00000040505	Abcg5	27409	protein_coding	31.56531548	-0.216167954	0.282100645	0.766279546	0.443509986	0.660955754	0.216167954
ENSMUSG00000086401	Gm15559	100504049	lncRNA	4.930663608	-0.216214251	0.5587316	0.386973372	0.698775921	0.840342672	0.216214251
ENSMUSG00000028578	Caap1	67770	protein_coding	128.4473468	0.216230368	0.095287174	-2.269249456	0.02325316	0.082564172	0.216230368
ENSMUSG00000028284	Map3k7	26409	protein_coding	634.418284	0.216332754	0.063213145	-3.422274834	0.000620995	0.004253469	0.216332754
ENSMUSG00000041264	Uspl1	231915	protein_coding	303.0697174	0.216355551	0.102272489	-2.115481415	0.034388937	0.11194538	0.216355551
ENSMUSG00000009075	Cabp7	192650	protein_coding	1.646629071	0.21637376	1.426541108	-0.151677199	0.879441541	1	0.21637376
ENSMUSG00000044783	Hjurp	212427	protein_coding	1736.373403	0.216395705	0.073666933	-2.937487635	0.003308834	0.017269937	0.216395705
ENSMUSG00000028164	Manba	110173	protein_coding	357.5683004	-0.216431642	0.103871252	2.083652959	0.037191746	0.118981886	0.216431642
ENSMUSG00000041718	Alg13	67574	protein_coding	58.81036003	0.216474217	0.163069538	-1.327496348	0.184344547	0.379371759	0.216474217
ENSMUSG00000017802	Retreg3	67998	protein_coding	1053.464675	-0.216533853	0.087037998	2.487808279	0.0128533	0.051707527	0.216533853
ENSMUSG00000061411	Nol4l	329540	protein_coding	749.0827583	0.216547925	0.139526345	-1.552021772	0.120657014	0.283150481	0.216547925
ENSMUSG00000028140	Mrpl9	78523	protein_coding	697.381977	-0.21658563	0.091605863	2.364320622	0.018063172	0.06767935	0.21658563
ENSMUSG00000028434	Epb41l4b	54357	protein_coding	2776.812878	-0.216604453	0.093135928	2.325680942	0.020035578	0.073420411	0.216604453
ENSMUSG00000029246	Ppat	231327	protein_coding	250.3846128	-0.216638819	0.164733232	1.31508874	0.18848008	0.38502722	0.216638819
ENSMUSG00000038608	Dock10	210293	protein_coding	31.99631213	-0.216642107	0.482153888	0.449321496	0.653199753	0.811870577	0.216642107
ENSMUSG00000028907	Utp11	67205	protein_coding	333.9096094	0.216672073	0.110686065	-1.957537048	0.05028436	0.14945931	0.216672073
ENSMUSG00000003190	Bcl2l12	75736	protein_coding	82.0519419	0.216683866	0.135329072	-1.601162727	0.109340882	0.263984696	0.216683866
ENSMUSG00000026839	Upp2	76654	protein_coding	41.83729662	-0.216686317	0.238000018	0.910446642	0.36258701	0.584928009	0.216686317
ENSMUSG00000005951	Shpk	74637	protein_coding	231.206665	-0.216707482	0.118309948	1.83169282	0.066997199	0.184936371	0.216707482
ENSMUSG00000031925	Maml2	270118	protein_coding	4.791131102	-0.216729065	0.651353286	0.332736581	0.739333134	0.867757454	0.216729065
ENSMUSG00000042831	Alkbh6	233065	protein_coding	243.1301651	0.216907619	0.071414473	-3.037306149	0.002387029	0.013089272	0.216907619
ENSMUSG00000056476	Med12l	329650	protein_coding	27.39616749	-0.216913132	0.341033893	0.636045673	0.524746654	0.723223689	0.216913132
ENSMUSG00000074811	Hps6	20170	protein_coding	98.41573524	-0.217042573	0.142880716	1.519047352	0.128750577	0.29613919	0.217042573
ENSMUSG00000040659	Efhd2	27984	protein_coding	1267.412956	-0.21715897	0.082346826	2.637126174	0.008361173	0.036859691	0.21715897
ENSMUSG00000110008	Or51f23b	259117	protein_coding	1.810633158	0.217195352	1.09065503	-0.199142117	0.842151576	1	0.217195352
ENSMUSG00000049562	Ap5b1	381201	protein_coding	196.5784772	-0.21737057	0.104213253	2.08582464	0.036994511	0.118465648	0.21737057
ENSMUSG00000014077	Chp1	56398	protein_coding	3496.50742	-0.217386496	0.114499209	1.898585139	0.057619044	0.16577019	0.217386496
ENSMUSG00000037134	Prmt9	102182	protein_coding	211.7503919	0.217392491	0.097568921	-2.228091578	0.025874409	0.089747949	0.217392491
ENSMUSG00000019854	Reps1	19707	protein_coding	201.5044184	0.217424082	0.078988633	-2.752599618	0.005912416	0.027880534	0.217424082
ENSMUSG00000039835	Nhsl1	215819	protein_coding	1006.218923	0.217431567	0.107795218	-2.017079895	0.043687176	0.134498514	0.217431567
ENSMUSG00000020311	Erlec1	66753	protein_coding	259.4756912	-0.217433186	0.138822377	1.566269005	0.117285654	0.277248109	0.217433186
ENSMUSG00000020614	Fam20a	208659	protein_coding	4.145936921	-0.21744222	1.219529919	0.178300029	0.858487356	1	0.21744222
ENSMUSG00000115548	5430440P10Rik	71362	lncRNA	3.561693523	-0.217569946	0.637977079	0.341030976	0.733080263	1	0.217569946
ENSMUSG00000053693	Mast1	56527	protein_coding	12.05954678	0.21770641	0.446213831	-0.487897045	0.625622768	0.794916857	0.21770641
ENSMUSG00000029578	Wipi2	74781	protein_coding	957.8094773	-0.217728199	0.05042604	4.317773133	1.58E-05	0.000167167	0.217728199
ENSMUSG00000028410	Dnaja1	15502	protein_coding	1066.128986	-0.217816695	0.136983872	1.590090073	0.111814503	0.267881142	0.217816695
ENSMUSG00000003161	Sri	109552	protein_coding	4026.608217	-0.217829092	0.088053893	2.473815558	0.013367869	0.053374514	0.217829092

Supplementary Table 1

ensembl_gene_id	external_gene_name	entrezgene_id	gene_biotype	baseMean	log2FoldChange	lfcSE	stat	pvalue	padj	absLog2FC
ENSMUSG00000036438	Calm2	12314	protein_coding	4263.96488	-0.217836947	0.082771404	2.631789929	0.008493636	0.037314155	0.217836947
ENSMUSG00000027404	Snrpb	20638	protein_coding	1362.467341	0.217990473	0.086977584	-2.506283383	0.012200779	0.049728326	0.217990473
ENSMUSG00000041782	Lad1	16763	protein_coding	4728.11922	-0.218014625	0.094175617	2.314979514	0.020614053	0.07508171	0.218014625
ENSMUSG00000034342	Cbl	12402	protein_coding	792.8974228	-0.218044978	0.12933036	1.685953536	0.091804749	0.232062395	0.218044978
ENSMUSG00000043366	Or51e2	170639	protein_coding	12.00240214	-0.218159245	0.391910371	0.556655964	0.577762509	0.761709251	0.218159245
ENSMUSG00000059586	Nsmce2	68501	protein_coding	148.6638496	0.21832304	0.094519768	-2.309813532	0.02089848	0.075916739	0.21832304
ENSMUSG00000050565	Tor1aip2	240832	protein_coding	2805.636559	0.218441622	0.070592825	-3.094388459	0.001972191	0.011213534	0.218441622
ENSMUSG00000026154	Sdhaf4	68002	protein_coding	93.83073687	-0.218474652	0.168459887	1.296894211	0.194667589	0.393673838	0.218474652
ENSMUSG00000033379	Atp6v0b	114143	protein_coding	984.1342883	-0.218491762	0.069825413	3.129115227	0.001753335	0.010159502	0.218491762
ENSMUSG00000092618	Btnl6	624681	protein_coding	1292.519803	-0.218548376	0.166782221	1.310381732	0.190066731	0.387358014	0.218548376
ENSMUSG00000032409	Atr	245000	protein_coding	304.2579594	0.218553107	0.126126766	-1.732805128	0.083130311	0.215987092	0.218553107
ENSMUSG00000027944	Hax1	23897	protein_coding	213.1775652	-0.21860328	0.141447712	1.545470592	0.122232477	0.285320309	0.21860328
ENSMUSG00000033326	Kdm4a	230674	protein_coding	1061.135728	0.218674428	0.069699624	-3.137383187	0.001704632	0.009910776	0.218674428
ENSMUSG00000025967	Eef1b2	55949	protein_coding	3862.142575	-0.218693747	0.097295593	2.247725097	0.024593725	0.086284421	0.218693747
ENSMUSG00000025810	Nrp1	18186	protein_coding	92.72404439	0.218713719	0.181720158	-1.20357434	0.228754142	0.437734172	0.218713719
ENSMUSG00000039611	Pgap4	67063	protein_coding	752.4011917	-0.218721751	0.101349879	2.15808596	0.030921149	0.103308737	0.218721751
ENSMUSG00000021640	Naip1	17940	protein_coding	2834.582642	-0.218745696	0.109448184	1.998623352	0.045649122	0.139215069	0.218745696
ENSMUSG00000027016	Zfp385b	241494	protein_coding	33.96168312	0.218757334	0.31687151	-0.690366054	0.489964019	0.697703355	0.218757334
ENSMUSG00000039304	Tnfsf10	22035	protein_coding	111.9336686	-0.218770409	0.159592011	1.370810528	0.170434028	0.36023903	0.218770409
ENSMUSG00000018143	Mafk	17135	protein_coding	1161.232265	-0.218823186	0.164661919	1.328924056	0.183873026	0.37880229	0.218823186
ENSMUSG00000080538	Gm25541	115489638	snRNA	24.84958873	0.218858431	0.323571339	-0.676383861	0.498796961	0.703739933	0.218858431
ENSMUSG00000027203	Dut	110074	protein_coding	229.5167819	0.218862716	0.169797604	-1.28896233	0.197411183	0.397518385	0.218862716
ENSMUSG00000021271	Zfp839	72805	protein_coding	147.9405459	-0.218887605	0.098330809	2.226032777	0.026011986	0.090073507	0.218887605
ENSMUSG00000112627	4933412E12Rik	71086	lncRNA	6.971958774	0.218926199	0.453324301	-0.482935061	0.629141846	0.797446101	0.218926199
ENSMUSG00000027002	Nckap1	50884	protein_coding	2816.744636	0.218966373	0.094697636	-2.312268641	0.020762884	0.07549059	0.218966373
ENSMUSG00000036206	Sh3bp4	98402	protein_coding	225.6560132	-0.219025198	0.121937802	1.796204242	0.07246204	0.195920934	0.219025198
ENSMUSG00000097577	6230400D17Rik	76133	lncRNA	15.00562911	-0.219128842	0.339403231	0.645629805	0.51851913	0.719081703	0.219128842
ENSMUSG00000005566	Trim28	21849	protein_coding	1988.247209	0.219274124	0.09638145	-2.27506563	0.022901993	0.081579713	0.219274124
ENSMUSG00000045482	Trrap	100683	protein_coding	1001.348926	0.219275372	0.079273434	-2.766063755	0.005673744	0.027040753	0.219275372
ENSMUSG00000026504	Sdccag8	76816	protein_coding	111.8290881	0.219290487	0.11454545	-1.91444084	0.055563851	0.161179189	0.219290487
ENSMUSG00000046637	Ttc34	242800	protein_coding	1.751341168	0.219302825	1.469057886	-0.149281268	0.881331695	1	0.219302825
ENSMUSG00000040690	Col16a1	107581	protein_coding	315.9584279	0.219310753	0.112428648	-1.950666101	0.051096778	0.151546731	0.219310753
ENSMUSG00000090264	Eif4ebp3	108112	protein_coding	252.495357	-0.219422088	0.154828143	1.417197699	0.156425134	0.339181285	0.219422088
ENSMUSG00000002768	Mea1	17256	protein_coding	640.8441848	-0.219540193	0.068614035	3.199639713	0.001375995	0.008308292	0.219540193
ENSMUSG00000052713	Zfp608	269023	protein_coding	300.1532992	0.219622711	0.124421953	-1.765144388	0.077539502	0.205601692	0.219622711
ENSMUSG00000006315	Tmem147	69804	protein_coding	454.4500834	-0.219638218	0.110899012	1.980524577	0.047644615	0.143734593	0.219638218
ENSMUSG00000025572	Tmc6	217353	protein_coding	884.5412072	0.219645712	0.055003687	-3.993290678	6.52E-05	0.000592563	0.219645712
ENSMUSG00000065547	Mir199a-1	387194	miRNA	3.142845306	-0.219656101	0.617791972	0.355550268	0.722177394	1	0.219656101
ENSMUSG00000028137	Celf3	78784	protein_coding	47.78239214	0.219679176	0.319939786	-0.686626628	0.492318044	0.698946027	0.219679176

Supplementary Table 1

ensembl_gene_id	external_gene_name	entrezgene_id	gene_biotype	baseMean	log2FoldChange	lfcSE	stat	pvalue	padj	absLog2FC
ENSMUSG00000057672	Pkn1	320795	protein_coding	848.4685309	-0.219807619	0.057340961	3.833343837	0.000126413	0.001061844	0.219807619
ENSMUSG00000040247	Tbc1d10c	108995	protein_coding	5.877846684	-0.220036688	0.516547624	0.425975608	0.67012562	0.822845898	0.220036688
ENSMUSG00000040998	Npnt	114249	protein_coding	7.76835469	-0.220121672	0.714790075	0.307952893	0.758118177	0.878750865	0.220121672
ENSMUSG00000025504	Eps8l2	98845	protein_coding	1666.166582	-0.220130961	0.183395062	1.200310185	0.230018896	0.438629566	0.220130961
ENSMUSG00000074824	Rslcan18	432770	protein_coding	6.257869338	-0.220226288	0.706866813	0.311552734	0.75538046	0.877032235	0.220226288
ENSMUSG00000022205	Sub1	20024	protein_coding	699.4548439	0.220230803	0.095795815	-2.298960591	0.021507177	0.077700618	0.220230803
ENSMUSG00000039262	Prrc2b	227723	protein_coding	993.692088	0.220245947	0.071831553	-3.066144846	0.002168382	0.012093437	0.220245947
ENSMUSG00000020788	Atp2a3	53313	protein_coding	12336.37218	-0.220284116	0.116360365	1.893119843	0.058341934	0.167325234	0.220284116
ENSMUSG00000051457	Spn	20737	protein_coding	14.04256165	-0.220292071	0.399449067	0.551489762	0.581297983	0.764339772	0.220292071
ENSMUSG00000026491	Ahctf1	226747	protein_coding	931.7488091	0.220346822	0.081302912	-2.71019594	0.006724347	0.030863221	0.220346822
ENSMUSG00000037260	Hgsnat	52120	protein_coding	347.3907778	-0.220409555	0.112033809	1.967348586	0.049143037	0.147109787	0.220409555
ENSMUSG00000027676	Ccdc39	51938	protein_coding	4.635969341	0.220425022	0.700892959	-0.314491706	0.753147615	0.876249099	0.220425022
ENSMUSG00000038286	Bphl	68021	protein_coding	273.2504621	-0.220533272	0.096725649	2.279987507	0.022608429	0.080865411	0.220533272
ENSMUSG00000023070	Rgn	19733	protein_coding	18.46717262	-0.220579854	0.260743674	0.845964353	0.397572631	0.619446591	0.220579854
ENSMUSG00000053390	Zfp952	240067	protein_coding	120.6461288	-0.220580786	0.10346824	2.131869519	0.033017575	0.108572726	0.220580786
ENSMUSG00000026581	Sell	20343	protein_coding	4.805919455	0.220596102	0.954159002	-0.231194279	0.817163875	0.908714429	0.220596102
ENSMUSG00000002007	Srpk3	56504	protein_coding	7.043315339	0.220679545	0.450777867	-0.489552752	0.624450417	0.794172689	0.220679545
ENSMUSG00000038244	Mical2	320878	protein_coding	1316.770004	-0.22071757	0.085545757	2.580111233	0.00987685	0.042179026	0.22071757
ENSMUSG00000003808	Farsa	66590	protein_coding	374.6676012	0.220791324	0.090105896	-2.450353804	0.01427159	0.056074481	0.220791324
ENSMUSG00000034902	Pip5k1c	18717	protein_coding	511.4238602	0.220828368	0.061479035	-3.591929608	0.000328239	0.00246612	0.220828368
ENSMUSG00000030494	Rhpn2	52428	protein_coding	1541.871169	-0.220836277	0.059686703	3.699924213	0.000215664	0.001688702	0.220836277
ENSMUSG00000028273	Pdlim5	56376	protein_coding	1183.24845	-0.220838988	0.08085237	2.731385461	0.006306866	0.029215031	0.220838988
ENSMUSG00000011114	Tbrg1	21376	protein_coding	1495.700411	0.220848082	0.081098699	-2.723201284	0.006465265	0.029831627	0.220848082
ENSMUSG00000040943	Tet2	214133	protein_coding	486.3317079	-0.220848987	0.124218335	1.777909737	0.075418681	0.201270862	0.220848987
ENSMUSG00000058897	Col25a1	77018	protein_coding	12.03261193	0.220972527	0.497956698	-0.443758519	0.657217182	0.814655179	0.220972527
ENSMUSG00000003531	Dgcr6	13353	protein_coding	199.6056077	-0.220990277	0.080529211	2.744225043	0.006065394	0.028407451	0.220990277
ENSMUSG00000022216	Psme1	19186	protein_coding	1057.636013	-0.221013526	0.07589412	2.91212977	0.003589735	0.018490605	0.221013526
ENSMUSG00000036992	Nxt1	56488	protein_coding	119.091934	0.221015324	0.142232767	-1.553898789	0.120208561	0.282356022	0.221015324
ENSMUSG00000037058	Paip2	67869	protein_coding	779.1739832	-0.221062455	0.112267301	1.969072502	0.048944766	0.146772084	0.221062455
ENSMUSG00000041658	Rragb	245670	protein_coding	6.288948453	0.221143236	0.452672756	-0.488527824	0.625176023	0.794593769	0.221143236
ENSMUSG00000036469	Marchf1	72925	protein_coding	3.779489773	0.221182356	0.732218138	-0.30207167	0.762597428	1	0.221182356
ENSMUSG00000024369	Nelfe	27632	protein_coding	355.1625546	0.221210374	0.085633258	-2.583229672	0.009788014	0.041864611	0.221210374
ENSMUSG00000047205	Dusp18	75219	protein_coding	172.1605755	-0.22121872	0.105361551	2.099615263	0.035762699	0.115258628	0.22121872
ENSMUSG00000034168	Irf2bpl	238330	protein_coding	601.3125042	-0.221279471	0.110160237	2.008705466	0.044568379	0.136567098	0.221279471
ENSMUSG00000022096	Hr	15460	protein_coding	396.8626151	0.221279592	0.097306641	-2.274044084	0.022963336	0.081727701	0.221279592
ENSMUSG00000024787	Snx15	69024	protein_coding	325.4406259	-0.221301584	0.077484647	2.856070102	0.004289204	0.021386449	0.221301584
ENSMUSG00000017386	Traf4	22032	protein_coding	687.8439043	-0.221346285	0.079991985	2.767105799	0.00565564	0.02696225	0.221346285
ENSMUSG00000055560	Zfp459	328274	protein_coding	32.5358627	-0.221360056	0.194083668	1.140539326	0.254061679	0.466691767	0.221360056
ENSMUSG00000097610	A930012L18Rik	626275	lncRNA	3.483185116	-0.221377447	0.590141677	0.375125932	0.707566812	1	0.221377447

ensembl_gene_id	external_gene_name	entrezgene_id	gene_biotype	baseMean	log2FoldChange	lfcSE	stat	pvalue	padj	absLog2FC
ENSMUSG00000026019	Wdr12	57750	protein_coding	125.3659619	-0.221380281	0.148965262	1.486120175	0.137247337	0.309673797	0.221380281
ENSMUSG00000073437	D330041H03Rik	654822	lncRNA	19.76405829	-0.22138433	0.327822358	0.675317971	0.499473768	0.704201299	0.22138433
ENSMUSG00000062867	Impdh2	23918	protein_coding	933.9784893	-0.221384731	0.152581638	1.450926427	0.146800348	0.324596932	0.221384731
ENSMUSG00000037169	Mycn	18109	protein_coding	34.25547326	0.221398125	0.28378553	-0.78016002	0.435296692	0.653617891	0.221398125
ENSMUSG00000027599	Armc1	74252	protein_coding	403.9909623	0.221441095	0.094409252	-2.345544425	0.018999314	0.070538371	0.221441095
ENSMUSG00000040560	Wdr7	104082	protein_coding	393.0683621	0.221444842	0.059590539	-3.716107395	0.000202316	0.001597055	0.221444842
ENSMUSG00000075703	Selenoi	28042	protein_coding	1039.9155	0.221505477	0.062987669	-3.516648281	0.000437032	0.003173763	0.221505477
ENSMUSG00000053012	Krcc1	57896	protein_coding	1091.830338	0.221526346	0.080505161	-2.751703666	0.005928614	0.027917061	0.221526346
ENSMUSG00000022032	Scara5	71145	protein_coding	50.2361419	0.221602307	0.263159176	-0.842084666	0.399740564	0.621944194	0.221602307
ENSMUSG00000029168	Dpysl5	65254	protein_coding	4.672932231	0.221608535	1.192166816	-0.185887186	0.852533231	0.927761944	0.221608535
ENSMUSG00000041798	Gck	103988	protein_coding	7.057151893	-0.221623936	0.465624594	0.475971284	0.634094836	0.800507769	0.221623936
ENSMUSG00000048222	Mfap1b	100034361	protein_coding	112.2865801	-0.221626809	0.135937234	1.630361331	0.103025152	0.252989943	0.221626809
ENSMUSG00000032449	Slc25a36	192287	protein_coding	588.1384348	-0.221667028	0.124192365	1.784868397	0.074282666	0.199334407	0.221667028
ENSMUSG00000035279	Ssc5d	269855	protein_coding	7.049063205	0.221682627	0.961489464	-0.230561681	0.81765534	0.908714429	0.221682627
ENSMUSG00000058355	Abce1	24015	protein_coding	1267.479482	0.221760713	0.115670466	-1.91717662	0.055215497	0.160458367	0.221760713
ENSMUSG00000040121	Rep15	66532	protein_coding	1122.441361	0.221836125	0.345927463	-0.64127931	0.521341229	0.720922137	0.221836125
ENSMUSG00000109408	A930037H05Rik	78439	lncRNA	30.10461172	-0.221850432	0.275074455	0.806510485	0.419948565	0.63998633	0.221850432
ENSMUSG00000069844	Sco1	52892	protein_coding	679.2118065	-0.221886201	0.082199431	2.699364184	0.006947211	0.031711558	0.221886201
ENSMUSG00000020876	Snx11	74479	protein_coding	215.539809	0.221908695	0.093581306	-2.371293004	0.017725973	0.066683451	0.221908695
ENSMUSG00000032911	Cspg4	121021	protein_coding	28.10052929	0.221914528	0.414464957	-0.535424104	0.592356626	0.772317806	0.221914528
ENSMUSG00000039994	Timeless	21853	protein_coding	104.2854225	0.221951017	0.173521321	-1.279099397	0.200862056	0.402162462	0.221951017
ENSMUSG00000018697	Aatf	56321	protein_coding	260.0394028	-0.222078377	0.099280507	2.23687794	0.025294319	0.088198982	0.222078377
ENSMUSG00000016150	Tenm1	23963	protein_coding	3.780300254	-0.22214601	2.442525307	0.090949318	0.927532863	1	0.22214601
ENSMUSG00000022428	Cby1	73739	protein_coding	139.7407449	0.222237512	0.132097947	-1.682369158	0.092497278	0.233277344	0.222237512
ENSMUSG00000020687	Cdc27	217232	protein_coding	771.8710917	0.22226015	0.088145629	-2.521510726	0.011685211	0.048151079	0.22226015
ENSMUSG00000031578	Mak16	67920	protein_coding	149.6415882	0.222305524	0.116584503	-1.906818817	0.056544054	0.163589437	0.222305524
ENSMUSG00000022452	Smdt1	69029	protein_coding	1228.525411	0.22233244	0.085666732	-2.59531834	0.009450334	0.040715581	0.22233244
ENSMUSG00000068601	Gm10244	621427	lncRNA	2.768496189	-0.222342903	0.720244807	0.308704626	0.757546225	1	0.222342903
ENSMUSG00000057842	Zfp595	218314	protein_coding	81.36903636	-0.222379133	0.137070955	1.622365097	0.104725198	0.255833124	0.222379133
ENSMUSG00000020387	Jade2	76901	protein_coding	209.4295142	-0.222497193	0.135477269	1.642321215	0.100523453	0.248744583	0.222497193
ENSMUSG00000066880	Zfp617	170938	protein_coding	155.4006519	-0.222511243	0.099893956	2.227474527	0.025915576	0.089852998	0.222511243
ENSMUSG00000025743	Sdc3	20970	protein_coding	8.17590166	-0.222540145	0.522583052	0.425846465	0.670219727	0.822900265	0.222540145
ENSMUSG00000026707	Nsun6	74455	protein_coding	116.4349971	0.222618592	0.109586243	-2.031446514	0.04220972	0.131147943	0.222618592
ENSMUSG00000054893	Zfp667	384763	protein_coding	46.28666482	-0.222738361	0.196039179	1.136193094	0.25587576	0.468566456	0.222738361
ENSMUSG00000068011	Mkrn2os	70291	protein_coding	311.9619695	-0.222814579	0.098986862	2.250951031	0.024388639	0.085735413	0.222814579
ENSMUSG00000025722	Wdr73	71968	protein_coding	146.7822528	-0.222820253	0.146892608	1.516892208	0.129293901	0.296962376	0.222820253
ENSMUSG00000041323	Ak7	78801	protein_coding	8.392695435	-0.222882263	0.579966198	0.384302161	0.700754506	0.841825268	0.222882263
ENSMUSG00000039834	Zfp335	329559	protein_coding	392.2488115	0.222898704	0.112993995	-1.972659731	0.048534343	0.145806101	0.222898704
ENSMUSG00000032182	Yipf2	74766	protein_coding	200.8399887	0.222941913	0.1004413	-2.219623929	0.026444307	0.091207452	0.222941913

ensembl_gene_id	external_gene_name	entrezgene_id	gene_biotype	baseMean	log2FoldChange	lfcSE	stat	pvalue	padj	absLog2FC
ENSMUSG00000037570	Mcrs1	51812	protein_coding	478.1159824	-0.222960333	0.092554291	2.408968081	0.015997697	0.061495803	0.222960333
ENSMUSG00000029461	Fam168a	319604	protein_coding	176.4999734	-0.223162447	0.111063373	2.009325314	0.044502645	0.13639879	0.223162447
ENSMUSG00000018921	Pelp1	75273	protein_coding	229.9136434	0.223169938	0.102251628	-2.182556335	0.029068498	0.098469165	0.223169938
ENSMUSG00000033488	Cryzl2	226527	protein_coding	135.6442445	-0.223260569	0.156193133	1.42938786	0.152892786	0.333845229	0.223260569
ENSMUSG00000025082	Vwa2	240675	protein_coding	10.35239946	0.223264401	0.425229381	-0.525044627	0.599552168	0.776544903	0.223264401
ENSMUSG00000093674	Rpl41	67945	protein_coding	6863.15504	-0.223286823	0.103593275	2.15541814	0.031129128	0.103865546	0.223286823
ENSMUSG00000030751	Psma1	26440	protein_coding	1219.994108	0.223306131	0.095532104	-2.337498298	0.019413289	0.071736644	0.223306131
ENSMUSG00000020225	Tmbim4	68212	protein_coding	687.014301	-0.22334698	0.062869426	3.552553184	0.000381512	0.002813884	0.22334698
ENSMUSG00000034159	Mab21l4	71874	protein_coding	163.6459061	-0.223357072	0.184205088	1.212545618	0.225303592	0.432685604	0.223357072
ENSMUSG00000034724	Cnot6l	231464	protein_coding	478.9499914	-0.223361095	0.12976644	1.721254709	0.085204614	0.220060838	0.223361095
ENSMUSG00000031987	Egln1	112405	protein_coding	472.5763813	-0.223366085	0.070190438	3.18228654	0.001461172	0.008692083	0.223366085
ENSMUSG00000024145	Pigf	18701	protein_coding	160.1128867	-0.223394815	0.101648574	2.19771715	0.027969269	0.095391264	0.223394815
ENSMUSG00000034394	Lif	16878	protein_coding	56.94524545	0.223402994	0.210894745	-1.059310389	0.289458445	0.508294925	0.223402994
ENSMUSG00000034171	Faah	14073	protein_coding	315.9281172	-0.223426399	0.088674288	2.519630028	0.011747824	0.048307661	0.223426399
ENSMUSG00000094840	Muc3a	619309	protein_coding	380.8969121	0.223449498	0.325969611	-0.685491808	0.493033628	0.699601077	0.223449498
ENSMUSG00000037145	Lypd8l	78354	protein_coding	370.8912982	0.223458023	0.310323594	-0.720080673	0.471475326	0.684298079	0.223458023
ENSMUSG00000022184	Fbxo4	106052	protein_coding	149.7388075	0.223496202	0.139894282	-1.597607847	0.110130279	0.265192684	0.223496202
ENSMUSG00000027569	Mrgbp	73247	protein_coding	209.662514	-0.223547447	0.085420411	2.617026113	0.008869956	0.038648666	0.223547447
ENSMUSG00000032386	Trip4	56404	protein_coding	339.9809949	0.223567771	0.098361338	-2.272923237	0.023030806	0.081931007	0.223567771
ENSMUSG00000074129	Rpl13a	22121	protein_coding	332.2726482	0.223584741	0.082789186	-2.700651531	0.00692038	0.031622347	0.223584741
ENSMUSG00000029130	Rnf32	56874	protein_coding	97.16527528	-0.223588568	0.114584493	1.951298664	0.051021528	0.151405123	0.223588568
ENSMUSG00000046185	Zfp84	74352	protein_coding	311.2110946	-0.223644412	0.088913016	2.515316893	0.011892544	0.048732869	0.223644412
ENSMUSG00000115145	Gm35166	102638653	lncRNA	3.051455361	-0.223702538	0.620293002	0.360640113	0.718368499	1	0.223702538
ENSMUSG00000026413	Pkp1	18772	protein_coding	465.8069533	-0.22370453	0.146102073	1.531152338	0.125731747	0.291415309	0.22370453
ENSMUSG00000024456	Diaph1	13367	protein_coding	1261.55468	-0.223706951	0.078764137	2.840213323	0.004508337	0.022343376	0.223706951
ENSMUSG00000028969	Cdk5	12568	protein_coding	281.3475556	-0.223736017	0.088572268	2.526027875	0.011536033	0.047698058	0.223736017
ENSMUSG00000039680	Mrps6	121022	protein_coding	347.0422005	-0.223746531	0.116286561	1.924096206	0.054342526	0.158736855	0.223746531
ENSMUSG00000020472	Zkscan17	268417	protein_coding	185.4149093	0.223837065	0.143723609	-1.557413332	0.119372383	0.280974277	0.223837065
ENSMUSG00000043241	Upf2	326622	protein_coding	355.2781338	0.223855619	0.064266794	-3.483223693	0.000495414	0.003520597	0.223855619
ENSMUSG00000014609	Chrne	11448	protein_coding	1.793716851	0.223855923	1.342067757	-0.166799271	0.867527993	1	0.223855923
ENSMUSG00000052783	Grk4	14772	protein_coding	7.506368899	-0.223985171	0.634595736	0.352957258	0.724120484	0.857084875	0.223985171
ENSMUSG00000005510	Ndufs3	68349	protein_coding	918.9246752	-0.223989478	0.082126136	2.727383629	0.006383877	0.029489048	0.223989478
ENSMUSG00000004707	Ly9	17085	protein_coding	6.066840388	-0.224028974	0.673822875	0.332474574	0.739530936	0.867862447	0.224028974
ENSMUSG00000029817	Tra2a	101214	protein_coding	939.77074	0.224106756	0.063232651	-3.544161933	0.000393863	0.002893353	0.224106756
ENSMUSG00000029722	Agfg2	231801	protein_coding	319.7216833	-0.224152755	0.090699367	2.471381703	0.013459207	0.053648407	0.224152755
ENSMUSG00000070817	Vmn1r85	252909	protein_coding	2.27187974	0.224163717	1.946325357	-0.115172788	0.908308168	1	0.224163717
ENSMUSG00000097726	9530036O11Rik	654796	lncRNA	10.70244573	-0.224218508	0.32360781	0.692871127	0.488390431	0.696363286	0.224218508
ENSMUSG00000024906	Mus81	71711	protein_coding	154.9660529	0.224330484	0.096179671	-2.332410603	0.019679101	0.07241317	0.224330484
ENSMUSG00000074754	Smim26	228715	protein_coding	61.25202312	-0.224378272	0.137234517	1.634998806	0.102049298	0.251303624	0.224378272

ensembl_gene_id	external_gene_name	entrezgene_id	gene_biotype	baseMean	log2FoldChange	lfcSE	stat	pvalue	padj	absLog2FC
ENSMUSG00000017688	Hnf4g	30942	protein_coding	410.8536428	-0.224418672	0.099162653	2.263137045	0.023627243	0.083675806	0.224418672
ENSMUSG00000057835	Zfp119a	104349	protein_coding	49.92334411	-0.224433701	0.141466441	1.586480151	0.112630431	0.269245209	0.224433701
ENSMUSG00000042507	Mideas	238317	protein_coding	799.5124373	0.224464725	0.164987672	-1.360493925	0.173673674	0.364427834	0.224464725
ENSMUSG00000073424	Cyp4f15	106648	protein_coding	3.060354926	-0.224498508	1.002283105	0.223987122	0.822767324	1	0.224498508
ENSMUSG00000053977	Cd8a	12525	protein_coding	23.68407837	-0.224533596	0.38873887	0.57759492	0.563537637	0.750868206	0.224533596
ENSMUSG00000023904	Hcfc1r1	353502	protein_coding	442.2787874	-0.224546995	0.103536313	2.168775262	0.03009975	0.101276951	0.224546995
ENSMUSG00000051169	Rpusd3	101122	protein_coding	48.3547032	0.224566174	0.180339052	-1.245244282	0.21304197	0.417785903	0.224566174
ENSMUSG00000054836	Elp6	72341	protein_coding	70.45007565	0.224568303	0.13680469	-1.641524877	0.100688507	0.248992215	0.224568303
ENSMUSG00000074884	Serf2	378702	protein_coding	1164.597632	0.224570317	0.081833857	-2.744222572	0.00606544	0.028407451	0.224570317
ENSMUSG00000025279	Dnase1l3	13421	protein_coding	3.160079395	0.224572311	0.910608949	-0.246617729	0.805204082	1	0.224572311
ENSMUSG00000042312	S100a13	20196	protein_coding	324.5390215	-0.22461974	0.096073099	2.338008675	0.019386798	0.071685421	0.22461974
ENSMUSG00000032267	Usp28	235323	protein_coding	194.5454755	-0.224634691	0.118227869	1.900014712	0.057431189	0.165344954	0.224634691
ENSMUSG00000034212	Ankmy1	241158	protein_coding	3.868101144	0.224643501	0.78193047	-0.287293448	0.773887639	1	0.224643501
ENSMUSG00000016995	Matn4	17183	protein_coding	10.61125571	-0.224719481	0.420327739	0.534629195	0.592906291	0.772538316	0.224719481
ENSMUSG00000001983	Taco1	70207	protein_coding	154.9256379	-0.224732268	0.105018416	2.139931996	0.032360263	0.10695305	0.224732268
ENSMUSG00000050377	Il31ra	218624	protein_coding	1.865747881	-0.224764564	1.837923369	0.122292675	0.902667233	1	0.224764564
ENSMUSG00000020475	Pgam2	56012	protein_coding	21.83916482	-0.224779337	0.300437394	0.748173634	0.454355432	0.669875536	0.224779337
ENSMUSG00000054027	Nt5dc3	103466	protein_coding	78.47696497	-0.224800948	0.105307026	2.134719367	0.03278394	0.108084245	0.224800948
ENSMUSG00000062818	Vmn1r51	22296	protein_coding	2.514230485	0.224826849	0.992074245	-0.226623007	0.8207169	1	0.224826849
ENSMUSG00000027175	Tcp11l1	320554	protein_coding	5.274779665	0.224839263	0.61719339	-0.36429305	0.715639189	0.852489762	0.224839263
ENSMUSG00000020580	Rock2	19878	protein_coding	1847.285505	0.22484787	0.090742839	-2.477858006	0.013217373	0.05288871	0.22484787
ENSMUSG00000020605	Hs1bp3	58240	protein_coding	198.4345727	-0.224892319	0.079673856	2.82266142	0.004762684	0.023359359	0.224892319
ENSMUSG00000087586	Gm6938	629024	lncRNA	0.673731784	0.224912598	1.667038992	-0.134917419	0.892677167	1	0.224912598
ENSMUSG00000035900	Gramd4	223752	protein_coding	484.5163238	-0.224973736	0.075792629	2.968279888	0.002994715	0.015840719	0.224973736
ENSMUSG00000054469	Lclat1	225010	protein_coding	994.1073221	0.225036891	0.084104693	-2.675675798	0.007457875	0.0336207	0.225036891
ENSMUSG00000037214	Thap1	73754	protein_coding	94.68272896	0.225046941	0.120926805	-1.861017837	0.062741658	0.176210159	0.225046941
ENSMUSG00000029136	Rbks	71336	protein_coding	113.0762204	0.225052316	0.125547297	-1.792569992	0.073041708	0.19693237	0.225052316
ENSMUSG00000034275	Igsf9b	235086	protein_coding	330.2080785	0.225058802	0.120929227	-1.861078642	0.062733072	0.176210159	0.225058802
ENSMUSG00000078768	Zfp566	72556	protein_coding	11.79619746	-0.225104958	0.30109144	0.747629882	0.454683435	0.670174662	0.225104958
ENSMUSG00000003810	Mast2	17776	protein_coding	1239.948857	-0.225215945	0.065913335	3.41684947	0.000633503	0.004319434	0.225215945
ENSMUSG00000063116	Or3a1	258702	protein_coding	1.525866186	-0.225319151	2.105714513	0.107003656	0.91478608	1	0.225319151
ENSMUSG00000036560	Lgi4	243914	protein_coding	2.294198655	0.225349054	1.591081068	-0.141632666	0.887370163	1	0.225349054
ENSMUSG00000045252	Zfp574	232976	protein_coding	238.6608123	0.225399399	0.104619448	-2.1544694	0.031203379	0.104013109	0.225399399
ENSMUSG00000051989	Smim11	68936	protein_coding	172.4953979	-0.225466172	0.103086381	2.187157696	0.028731014	0.097546045	0.225466172
ENSMUSG00000056962	Jmjd6	107817	protein_coding	417.0530269	0.225515674	0.100821518	-2.236781193	0.025300644	0.088202415	0.225515674
ENSMUSG00000033356	Pus7l	78895	protein_coding	51.49750604	0.22551766	0.213893372	-1.054346181	0.291724453	0.510557756	0.22551766
ENSMUSG00000078612	Fyb2	242594	protein_coding	78.81859586	-0.225604322	0.222570269	1.013631886	0.310758437	0.531744361	0.225604322
ENSMUSG00000000724	Cryba1	12957	protein_coding	2.787825654	-0.225729281	1.200644918	0.188006694	0.850871404	1	0.225729281
ENSMUSG00000042453	Reln	19699	protein_coding	6.368356984	0.225747796	2.524471777	-0.089423775	0.92874513	0.966007248	0.225747796

ensembl_gene_id	external_gene_name	entrezgene_id	gene_biotype	baseMean	log2FoldChange	lfcSE	stat	pvalue	padj	absLog2FC
ENSMUSG00000021124	Vti1b	53612	protein_coding	653.885327	-0.225748829	0.068820748	3.280243736	0.001037174	0.0065599	0.225748829
ENSMUSG00000021709	Erbin	59079	protein_coding	5974.937996	-0.225750849	0.097169439	2.32327006	0.020164649	0.073835702	0.225750849
ENSMUSG00000032803	Cdv3	321022	protein_coding	1667.766374	0.225772383	0.090144424	-2.504562941	0.012260279	0.049857012	0.225772383
ENSMUSG00000035372	1810055G02Rik	72056	protein_coding	183.0355441	-0.225786796	0.120750496	1.869862268	0.061502947	0.173816344	0.225786796
ENSMUSG00000000168	Dlat	235339	protein_coding	1207.408457	-0.225787931	0.096900569	2.330099114	0.019800913	0.07274578	0.225787931
ENSMUSG00000026888	Grb14	50915	protein_coding	23.1997682	-0.225796331	0.216193003	1.04442016	0.29629104	0.515481484	0.225796331
ENSMUSG00000041153	Osgin2	209212	protein_coding	87.10794107	0.225968671	0.112898341	-2.001523406	0.045336014	0.138618577	0.225968671
ENSMUSG00000039001	Rps21	66481	protein_coding	2352.891104	-0.225974219	0.126818969	1.781864499	0.074771331	0.19999509	0.225974219
ENSMUSG00000041287	Sox15	20670	protein_coding	2.717478564	-0.22599297	0.900971707	0.250832483	0.801943626	1	0.22599297
ENSMUSG00000030560	Ctsc	13032	protein_coding	898.6581489	-0.22602808	0.09266861	2.439100786	0.014723861	0.057495825	0.22602808
ENSMUSG00000012429	Mplkip	66308	protein_coding	128.6959589	-0.226064428	0.09954788	2.270911521	0.023152335	0.082276233	0.226064428
ENSMUSG00000102106	2310043O21Rik	69679	lncRNA	4.06074612	0.226070465	0.508952242	-0.444187974	0.656906686	1	0.226070465
ENSMUSG00000002658	Gtf2f1	98053	protein_coding	692.6946203	0.22613214	0.050069119	-4.516399438	6.29E-06	7.37E-05	0.22613214
ENSMUSG00000024942	Capn1	12333	protein_coding	1613.044064	-0.226140874	0.064854904	3.486873924	0.000488702	0.003481631	0.226140874
ENSMUSG00000026385	Dbi	13167	protein_coding	2844.518478	0.226212963	0.074418115	-3.03975668	0.002367694	0.0129987	0.226212963
ENSMUSG00000039205	Ciz1	68379	protein_coding	442.6914575	-0.226227331	0.093233282	2.426465387	0.015246705	0.059173697	0.226227331
ENSMUSG00000042589	Cux2	13048	protein_coding	30.60182346	0.226316672	0.433719374	-0.521804386	0.601806536	0.77804505	0.226316672
ENSMUSG00000020946	Gosr2	56494	protein_coding	785.9501357	0.226318794	0.062104157	-3.644181064	0.000268245	0.002060368	0.226318794
ENSMUSG00000032456	Nmnat3	74080	protein_coding	136.0641211	-0.226351082	0.183695613	1.232207335	0.217871644	0.423784727	0.226351082
ENSMUSG00000034075	Zdhhc5	228136	protein_coding	2297.73727	0.226441342	0.044299928	-5.11155099	3.20E-07	4.87E-06	0.226441342
ENSMUSG00000030417	Pdcd5	56330	protein_coding	289.4570775	-0.226554546	0.094901754	2.387253505	0.016974782	0.064474124	0.226554546
ENSMUSG00000007880	Arid1a	93760	protein_coding	760.7269786	0.226572572	0.15048071	-1.505658579	0.132154842	0.3013667	0.226572572
ENSMUSG00000028399	Ptprd	19266	protein_coding	1362.932505	-0.226648828	0.070978489	3.193204489	0.001407033	0.00846073	0.226648828
ENSMUSG00000022378	Cyrib	223601	protein_coding	398.2074156	0.226658909	0.072586316	-3.122612092	0.001792538	0.010353962	0.226658909
ENSMUSG00000072964	Bhlhb9	70237	protein_coding	23.93651225	0.226666937	0.260558863	-0.869926027	0.384340831	0.606725963	0.226666937
ENSMUSG00000029012	Orc5	26429	protein_coding	150.6407756	-0.226684321	0.109006017	2.079557877	0.037566104	0.119923959	0.226684321
ENSMUSG00000024667	Tmem216	68642	protein_coding	44.27243977	0.226736725	0.164440265	-1.378839447	0.167944261	0.356436387	0.226736725
ENSMUSG00000037013	Ss18	268996	protein_coding	730.5029599	0.226801209	0.086290518	-2.628344508	0.008580157	0.037634187	0.226801209
ENSMUSG00000003308	Keap1	50868	protein_coding	613.2364637	0.226855078	0.068845719	-3.295122484	0.000983788	0.00627754	0.226855078
ENSMUSG00000002477	Snrpd1	20641	protein_coding	296.7412377	0.226856357	0.139418199	-1.627164593	0.103702145	0.254161061	0.226856357
ENSMUSG00000033966	Cdkl4	381113	protein_coding	3.291153594	-0.226887055	0.822916438	0.275710929	0.782770101	1	0.226887055
ENSMUSG00000063286	Gvin-ps7	668139	transcribed_unprocessed_pseudogene	20.01980945	0.22691042	0.324050713	-0.700231202	0.483782929	0.694229344	0.22691042
ENSMUSG00000026851	BC005624	227707	protein_coding	299.0272797	-0.226963208	0.063715938	3.562110415	0.000367886	0.002731683	0.226963208
ENSMUSG00000004837	Grap	71520	protein_coding	4.578400126	0.226975633	0.570853475	-0.397607518	0.690919518	0.835396465	0.226975633
ENSMUSG00000042595	Fam199x	245622	protein_coding	417.9875504	0.227059536	0.126834659	-1.790201016	0.073421602	0.197666177	0.227059536
ENSMUSG00000090093	Gm14399	100043761	protein_coding	24.67886116	-0.227065639	0.2886287	0.786704991	0.431454582	0.649798537	0.227065639
ENSMUSG00000060923	Acyp2	75572	protein_coding	37.0417996	-0.227130817	0.17143161	1.324906284	0.185202236	0.380591057	0.227130817
ENSMUSG00000058600	Rpl30	19946	protein_coding	1757.1322	-0.227138977	0.123396991	1.840717296	0.065662998	0.182183929	0.227138977
ENSMUSG00000025808	Ccdc7a	74703	protein_coding	4.431189896	-0.227145412	0.917023379	0.247698606	0.804367611	0.903566201	0.227145412

Supplementary Table 1

ensembl_gene_id	external_gene_name	entrezgene_id	gene_biotype	baseMean	log2FoldChange	lfcSE	stat	pvalue	padj	absLog2FC
ENSMUSG00000054428	Atpif1	11983	protein_coding	1706.029637	-0.227151539	0.083731448	2.712858123	0.006670566	0.030641927	0.227151539
ENSMUSG00000028633	Ctps	51797	protein_coding	198.1828158	0.227239417	0.145672182	-1.559936937	0.118774785	0.279846882	0.227239417
ENSMUSG00000027947	Il6ra	16194	protein_coding	33.64422472	-0.227370779	0.351879707	0.646160533	0.518175396	0.718665364	0.227370779
ENSMUSG00000012848	Rps5	20103	protein_coding	6368.247198	-0.227419325	0.089329366	2.545851777	0.010901147	0.045517455	0.227419325
ENSMUSG00000062312	Erbb2	13866	protein_coding	2558.146127	-0.227425714	0.115880438	1.962589353	0.049693909	0.148254918	0.227425714
ENSMUSG00000026509	Capn2	12334	protein_coding	921.9104565	-0.227436121	0.132280201	1.719351192	0.085550444	0.220716112	0.227436121
ENSMUSG00000023277	Twf2	23999	protein_coding	218.51959	0.22744504	0.095873969	-2.37233361	0.017676123	0.066614215	0.22744504
ENSMUSG00000053964	Lgals4	16855	protein_coding	42014.74337	-0.227481825	0.086724212	2.62304862	0.008714684	0.038133091	0.227481825
ENSMUSG00000110195	Pde2a	207728	protein_coding	24.30640153	-0.227492908	0.314584066	0.723154577	0.469584922	0.683166089	0.227492908
ENSMUSG00000026694	Eef1aknmt	71449	protein_coding	128.9449848	-0.227507447	0.131427828	1.731044711	0.08344379	0.216495012	0.227507447
ENSMUSG00000034192	Lsm3	67678	protein_coding	179.6825719	0.227690123	0.114939763	-1.980951741	0.047596686	0.143668739	0.227690123
ENSMUSG00000024892	Pcx	18563	protein_coding	1016.520746	-0.227728568	0.191595454	1.188590662	0.234600784	0.444186809	0.227728568
ENSMUSG00000029832	Nfe2l3	18025	protein_coding	36.71294383	-0.227731792	0.227901519	0.999255261	0.317671052	0.539052565	0.227731792
ENSMUSG00000030839	Sergef	27414	protein_coding	65.08503692	-0.227788337	0.132525156	1.718830932	0.085645161	0.220887739	0.227788337
ENSMUSG00000048546	Tob2	57259	protein_coding	415.3398682	-0.227833951	0.096002831	2.373200326	0.017634697	0.066534014	0.227833951
ENSMUSG00000031749	St3gal2	20444	protein_coding	13.27869836	-0.227901892	0.45444056	0.501499892	0.616019354	0.788172589	0.227901892
ENSMUSG00000118391	Gm19519	100503033	lncRNA	1.414797724	-0.227903792	1.105079269	0.206232981	0.836608926	1	0.227903792
ENSMUSG00000028357	Kif12	16552	protein_coding	275.7928326	0.227919154	0.121377951	-1.877764062	0.060413449	0.17162699	0.227919154
ENSMUSG00000022114	Spry2	24064	protein_coding	391.298366	-0.227944023	0.129775289	1.756451663	0.079011318	0.208221097	0.227944023
ENSMUSG00000041135	Ripk2	192656	protein_coding	154.047095	0.228061108	0.108864733	-2.094903475	0.036179574	0.116216589	0.228061108
ENSMUSG00000022518	4930562C15Rik	78809	protein_coding	10.73904534	0.228097867	0.546875522	-0.417092844	0.676610499	0.826873809	0.228097867
ENSMUSG00000030707	Coro1a	12721	protein_coding	49.78359922	0.228135668	0.324196964	-0.703694647	0.481622955	0.692385331	0.228135668
ENSMUSG00000032606	Nicn1	66257	protein_coding	90.31346324	-0.228141558	0.125597705	1.816446876	0.06930185	0.189541363	0.228141558
ENSMUSG00000020589	Cyria	76820	protein_coding	203.8355814	-0.228145019	0.118748494	1.921245581	0.054700754	0.159457768	0.228145019
ENSMUSG00000038304	Cd160	54215	protein_coding	19.22000425	0.228151048	0.341572247	-0.667943752	0.504169498	0.708596728	0.228151048
ENSMUSG00000038122	Tbc1d32	544696	protein_coding	325.5681595	-0.228263217	0.063965538	3.568534294	0.000358984	0.002672795	0.228263217
ENSMUSG00000031482	Slc25a15	18408	protein_coding	384.3245897	-0.228280049	0.101306974	2.253349806	0.024237102	0.085323278	0.228280049
ENSMUSG00000055567	Unc80	329178	protein_coding	11.29465575	0.228329761	0.637509202	-0.35815916	0.720224208	0.855204229	0.228329761
ENSMUSG00000039087	Rreb1	68750	protein_coding	1219.66146	0.228469704	0.087086843	-2.623469813	0.008703916	0.038096068	0.228469704
ENSMUSG00000038991	Txndc5	105245	protein_coding	4055.139002	0.228551931	0.137218973	-1.665600077	0.095793096	0.239468239	0.228551931
ENSMUSG00000021952	Xpo4	57258	protein_coding	342.4705488	0.228610613	0.096478358	-2.369553321	0.017809587	0.066903439	0.228610613
ENSMUSG00000024563	Smad2	17126	protein_coding	670.0783359	0.228635455	0.060365461	-3.787521099	0.000152158	0.001243312	0.228635455
ENSMUSG00000113722	Snhg10	69434	lncRNA	5.614346775	-0.228644344	0.581845446	0.392964053	0.694346032	0.837885199	0.228644344
ENSMUSG00000022336	Eif3e	16341	protein_coding	1335.356268	-0.228775714	0.11877771	1.92608288	0.054094026	0.158163729	0.228775714
ENSMUSG00000077733	Gm23608	115486143	snoRNA	1.305305294	-0.228812627	0.833708038	0.274451747	0.783737481	1	0.228812627
ENSMUSG00000015627	Gata5	14464	protein_coding	12.84395841	0.228823025	0.445902582	-0.513168201	0.607833668	0.782288135	0.228823025
ENSMUSG00000061589	Dot1l	208266	protein_coding	450.7040136	0.228836537	0.174415816	-1.312017123	0.189514357	0.386709513	0.228836537
ENSMUSG00000110916	2310003N18Rik	71873	lncRNA	0.678458349	-0.22892818	1.512890836	0.151318373	0.879724577	1	0.22892818
ENSMUSG00000050552	Lamtor4	66096	protein_coding	421.2642511	-0.228957998	0.087736057	2.609622612	0.009064216	0.039360099	0.228957998

ensembl_gene_id	external_gene_name	entrezgene_id	gene_biotype	baseMean	log2FoldChange	lfcSE	stat	pvalue	padj	absLog2FC
ENSMUSG00000030094	Xpc	22591	protein_coding	264.8076829	-0.228977192	0.105414167	2.172167153	0.029843057	0.100556672	0.228977192
ENSMUSG00000044279	Crb3	224912	protein_coding	837.9752525	-0.228990498	0.101362686	2.259120265	0.023875905	0.084321363	0.228990498
ENSMUSG00000032226	Gcnt3	72077	protein_coding	3813.256002	-0.229009389	0.143362309	1.597417001	0.110172785	0.265217693	0.229009389
ENSMUSG00000053062	Jam2	67374	protein_coding	4.589631191	0.229142376	0.7204438	-0.318057253	0.750441508	0.874764669	0.229142376
ENSMUSG00000028132	Tlcd4	99887	protein_coding	473.4181673	0.22916987	0.103049794	-2.223875103	0.026156849	0.090461612	0.22916987
ENSMUSG00000016256	Ctsz	64138	protein_coding	994.3734663	-0.22922222	0.054334432	4.21872858	2.46E-05	0.0002483	0.22922222
ENSMUSG00000038276	Asic3	171209	protein_coding	6.974158078	0.229247531	0.508995377	-0.450392168	0.652427691	0.8114957	0.229247531
ENSMUSG00000020180	Snrpd3	67332	protein_coding	232.3243346	0.229273077	0.102213443	-2.243081431	0.024891564	0.087070385	0.229273077
ENSMUSG00000045975	C2cd2	207781	protein_coding	211.7460594	-0.229414959	0.113223804	2.02620784	0.042743491	0.132283734	0.229414959
ENSMUSG00000034471	Caskin2	140721	protein_coding	1047.197357	-0.229423106	0.119052484	1.927075344	0.053970241	0.157957314	0.229423106
ENSMUSG00000064927	Gm22043	115489855	snRNA	2.181553871	0.229463926	0.783875279	-0.292730148	0.769728424	1	0.229463926
ENSMUSG00000052062	Pard3b	72823	protein_coding	496.2263229	-0.229512675	0.076124596	3.014960842	0.002570124	0.013943178	0.229512675
ENSMUSG00000089844	A530032D15Rik	381287	protein_coding	6.100045147	-0.229616486	0.789415049	0.290869153	0.771151394	0.885199039	0.229616486
ENSMUSG00000041959	S100a10	20194	protein_coding	3326.055497	-0.229622442	0.081427981	2.819945182	0.004803185	0.023502823	0.229622442
ENSMUSG00000045838	Ccdc9b	214239	protein_coding	208.7233072	0.229829549	0.252381474	-0.910643502	0.362483242	0.58491775	0.229829549
ENSMUSG00000020601	Trib2	217410	protein_coding	41.31984004	0.229835835	0.186498048	-1.232376624	0.217808429	0.423711673	0.229835835
ENSMUSG00000040430	Pitpnc1	71795	protein_coding	35.91477986	-0.229870922	0.230601906	0.996830106	0.318846982	0.540266681	0.229870922
ENSMUSG00000031954	Cfdp1	23837	protein_coding	608.0543459	0.229876885	0.100346181	-2.290838398	0.021972762	0.079002436	0.229876885
ENSMUSG00000008683	Rps15a	267019	protein_coding	2789.327745	-0.229899931	0.137257447	1.674954153	0.093943227	0.23602289	0.229899931
ENSMUSG00000050619	Zscan29	99334	protein_coding	334.144663	-0.230043323	0.080968017	2.841162869	0.004494935	0.022291098	0.230043323
ENSMUSG00000022002	Erich6b	75272	protein_coding	1.597328555	0.230063495	1.443578464	-0.159370274	0.873377158	1	0.230063495
ENSMUSG00000045259	Klhdc9	68874	protein_coding	7.866343854	-0.23019347	0.410426565	0.560863963	0.574890278	0.760164897	0.23019347
ENSMUSG00000040387	Klhl32	212390	protein_coding	2.265121357	-0.23024918	0.915902921	0.251390376	0.801512307	1	0.23024918
ENSMUSG00000027562	Car2	12349	protein_coding	12114.95619	-0.230348782	0.127117836	1.812088611	0.069972506	0.190901365	0.230348782
ENSMUSG00000044148	1810030O07Rik	69155	protein_coding	360.3167868	0.230442729	0.107462387	-2.144403587	0.032000563	0.106094619	0.230442729
ENSMUSG00000015776	Med22	20933	protein_coding	444.9595013	-0.230503783	0.121699059	1.894047378	0.058218721	0.167087933	0.230503783
ENSMUSG00000027957	Slc35a3	229782	protein_coding	4145.818085	0.230547823	0.140505518	-1.640845327	0.100829526	0.249266287	0.230547823
ENSMUSG00000022426	Josd1	74158	protein_coding	487.9580867	-0.230575542	0.073224764	3.148873793	0.00163901	0.009604899	0.230575542
ENSMUSG00000039178	Tbc1d19	67249	protein_coding	71.06338958	0.230634623	0.189260064	-1.218612198	0.222991417	0.429954629	0.230634623
ENSMUSG00000111243	Gm4668	100043817	lncRNA	2.238561892	0.230667468	0.806766536	-0.285916009	0.774942452	1	0.230667468
ENSMUSG00000029821	Gsdme	54722	protein_coding	267.2471653	-0.230765165	0.088169804	2.617281131	0.008863331	0.038640192	0.230765165
ENSMUSG00000048070	Pirt	193003	protein_coding	2.990424314	-0.230798898	1.189717029	0.193994784	0.846179966	1	0.230798898
ENSMUSG00000038717	Atp5l	27425	protein_coding	265.0681505	-0.230800596	0.109345613	2.110743998	0.034794322	0.112975508	0.230800596
ENSMUSG00000025747	Tyms	22171	protein_coding	367.597761	0.230809853	0.159133334	-1.450418	0.146941991	0.324759105	0.230809853
ENSMUSG00000001089	Luzp1	269593	protein_coding	1839.660607	-0.230859779	0.063045539	3.661794055	0.000250455	0.001933622	0.230859779
ENSMUSG00000029847	Slc23a4	243753	protein_coding	56.2727512	-0.230953648	0.167408781	1.379579057	0.167716289	0.356246496	0.230953648
ENSMUSG00000042138	Msantd2	235184	protein_coding	75.63600806	-0.231012867	0.128694262	1.795051807	0.072645446	0.196184284	0.231012867
ENSMUSG00000030316	Tamm41	68971	protein_coding	139.6288062	0.231052467	0.110270295	-2.095328284	0.03614182	0.116153464	0.231052467
ENSMUSG00000056698	Elmod3	232089	protein_coding	143.2504337	-0.23119745	0.104548945	2.211380037	0.027009531	0.092827346	0.23119745

Supplementary Table 1

ensembl_gene_id	external_gene_name	entrezgene_id	gene_biotype	baseMean	log2FoldChange	lfcSE	stat	pvalue	padj	absLog2FC
ENSMUSG00000037940	Inpp4b	234515	protein_coding	221.384742	0.231197738	0.236300343	-0.978406274	0.327873426	0.549974786	0.231197738
ENSMUSG00000096423	Gm24978	115489655	snRNA	1.217576751	-0.23121461	1.185887791	0.194971743	0.845415069	1	0.23121461
ENSMUSG00000041301	Cftr	12638	protein_coding	2781.028435	-0.231367937	0.128546728	1.799874184	0.071880507	0.194819414	0.231367937
ENSMUSG00000079497	Gm13420	433415	protein_coding	5.037716649	0.231388363	0.560588266	-0.412759911	0.679782526	0.828297243	0.231388363
ENSMUSG00000035354	Uvrag	78610	protein_coding	229.1592827	0.231406436	0.090547074	-2.555647857	0.010599032	0.044651126	0.231406436
ENSMUSG00000055862	Izumo4	71564	protein_coding	95.12427262	-0.231442821	0.127888403	1.809724859	0.070338466	0.1916312	0.231442821
ENSMUSG00000053870	Fpgt	75540	protein_coding	598.148702	-0.231500117	0.125707358	1.841579685	0.065536655	0.181986265	0.231500117
ENSMUSG00000069873	4930438A08Rik	73988	protein_coding	1.923551896	0.231526289	1.283721098	-0.1803556	0.856873409	1	0.231526289
ENSMUSG00000000317	Bcl6b	12029	protein_coding	5.791817074	0.231535838	0.539100476	-0.429485501	0.667569943	0.821350533	0.231535838
ENSMUSG00000041923	Nol4	319211	protein_coding	2.914386858	0.231618473	1.103053309	-0.209979401	0.83368375	1	0.231618473
ENSMUSG00000026634	Angel2	52477	protein_coding	567.0722012	-0.231708141	0.076800916	3.016997087	0.002552923	0.013872644	0.231708141
ENSMUSG00000024959	Bad	12015	protein_coding	446.6765485	0.231876788	0.075647375	-3.065232441	0.002175009	0.012122206	0.231876788
ENSMUSG00000061080	Lsamp	268890	protein_coding	6.458188463	-0.231944788	3.607792987	0.064289938	0.948739365	0.975316352	0.231944788
ENSMUSG00000050677	Ccdc96	66717	protein_coding	6.975297706	0.231984455	0.459439957	-0.504928776	0.613608862	0.786448489	0.231984455
ENSMUSG00000100860	2010009K17Rik	72340	lncRNA	1.386261216	0.232039283	1.030355683	-0.22520309	0.821821286	1	0.232039283
ENSMUSG00000040282	Cdin1	399568	protein_coding	56.24825096	0.232068636	0.196146753	-1.183137791	0.236754543	0.446932387	0.232068636
ENSMUSG00000054716	Zfp771	244216	protein_coding	116.0597467	0.232085628	0.138194085	-1.679417957	0.093070616	0.234222517	0.232085628
ENSMUSG00000110277	Gm45871	108168395	protein_coding	233.7345473	-0.232119309	0.134485016	1.725986405	0.084349859	0.21833128	0.232119309
ENSMUSG00000002017	Fam98a	72722	protein_coding	382.5689866	0.232142081	0.114855537	-2.021165778	0.043262607	0.133465102	0.232142081
ENSMUSG00000002885	Adgre5	26364	protein_coding	384.6948306	-0.232216876	0.20726835	1.120368233	0.262556876	0.476573341	0.232216876
ENSMUSG00000059355	Wdr83os	414077	protein_coding	155.5010708	-0.232254705	0.117202825	1.981647668	0.047518688	0.143502708	0.232254705
ENSMUSG00000112532	Gm36283	102640148	lncRNA	2.820260988	-0.232289089	1.043424806	0.222621781	0.823829883	1	0.232289089
ENSMUSG00000045411	2410002F23Rik	668661	protein_coding	320.7675763	-0.232308211	0.118144199	1.966310779	0.049262722	0.147350951	0.232308211
ENSMUSG00000047109	Cldn14	56173	protein_coding	24.45098626	0.232336736	0.22112845	-1.050686769	0.293402475	0.512561987	0.232336736
ENSMUSG00000022681	Ntan1	18203	protein_coding	270.3831182	-0.232338195	0.068901561	3.372030935	0.000746161	0.004948633	0.232338195
ENSMUSG00000032076	Cadm1	54725	protein_coding	15.9009785	-0.232359512	0.281904609	0.824248716	0.409798252	0.630288565	0.232359512
ENSMUSG00000021007	Spata7	104871	protein_coding	57.71625872	-0.232383858	0.143034575	1.624669127	0.104233079	0.255121545	0.232383858
ENSMUSG00000037108	Zcwpw1	381678	protein_coding	12.32763039	-0.232432788	0.328747552	0.70702515	0.479550849	0.690797515	0.232432788
ENSMUSG00000046862	Pramel12	242736	protein_coding	235.5849336	-0.232497429	0.097308392	2.389284467	0.016881226	0.064232353	0.232497429
ENSMUSG00000047678	Gpr82	319200	protein_coding	3.200362035	-0.232519271	0.572169093	0.406382088	0.684461879	1	0.232519271
ENSMUSG00000030725	Lipt2	67164	protein_coding	105.0548147	0.232612012	0.17070334	-1.362668192	0.172987107	0.363680341	0.232612012
ENSMUSG00000078942	Naip6	17952	protein_coding	1314.518957	-0.232613997	0.086751498	2.681383072	0.007331853	0.033126737	0.232613997
ENSMUSG00000039108	Lsm14b	241846	protein_coding	320.8934685	-0.232634373	0.079783209	2.915831235	0.003547423	0.018306921	0.232634373
ENSMUSG00000038515	Grtp1	66790	protein_coding	893.6135834	-0.232640647	0.063379391	3.670604008	0.000241978	0.001872551	0.232640647
ENSMUSG00000028381	Ugcg	22234	protein_coding	838.6370373	-0.232648656	0.110508464	2.105256436	0.035268992	0.114179991	0.232648656
ENSMUSG00000030263	Irag2	16970	protein_coding	284.0956152	-0.232653945	0.189373945	1.228542531	0.219243374	0.425500655	0.232653945
ENSMUSG00000024831	Ighmbp2	20589	protein_coding	159.7298818	0.232730988	0.099779684	-2.332448623	0.019677103	0.07241317	0.232730988
ENSMUSG00000024213	Nudt3	56409	protein_coding	564.2392665	-0.232733993	0.099954816	2.328391986	0.019891299	0.07306159	0.232733993
ENSMUSG00000115111	Gm4632	100043765	lncRNA	1.88818489	0.232779832	0.83499639	-0.278779447	0.780414089	1	0.232779832

ensembl_gene_id	external_gene_name	entrezgene_id	gene_biotype	baseMean	log2FoldChange	lfcSE	stat	pvalue	padj	absLog2FC
ENSMUSG00000020905	Usp43	216835	protein_coding	470.3969182	0.232810021	0.071208714	-3.269403531	0.001077745	0.006772404	0.232810021
ENSMUSG00000041528	Rnf123	84585	protein_coding	339.7735242	-0.232823964	0.073532275	3.166282605	0.001544007	0.009122624	0.232823964
ENSMUSG00000028555	Ttc39a	230603	protein_coding	1728.851015	0.232845173	0.153021555	-1.521649497	0.128096928	0.295198531	0.232845173
ENSMUSG00000095687	Rnaset2a	100037283	protein_coding	5.574443753	-0.232874097	0.530002816	0.439382755	0.66038422	0.816530773	0.232874097
ENSMUSG00000025195	Dnmbp	71972	protein_coding	2249.445197	-0.232977146	0.083955992	2.774991273	0.005520319	0.026393326	0.232977146
ENSMUSG00000066979	Bub3	12237	protein_coding	630.621801	0.233088273	0.104448244	-2.231615046	0.025640416	0.089020995	0.233088273
ENSMUSG00000030929	Eri2	71151	protein_coding	89.33175073	0.233097028	0.153950887	-1.514099938	0.130000494	0.297863506	0.233097028
ENSMUSG00000021506	Pitx1	18740	protein_coding	2.34029626	0.233122813	1.282403927	-0.181785791	0.855750836	1	0.233122813
ENSMUSG00000034401	Spata6	67946	protein_coding	66.68340662	-0.233148978	0.195205177	1.194379075	0.232329718	0.441303539	0.233148978
ENSMUSG00000033628	Pik3c3	225326	protein_coding	560.0652115	-0.233160622	0.113598983	2.05248865	0.040122197	0.126157266	0.233160622
ENSMUSG00000086968	4933431E20Rik	329735	lncRNA	274.3853539	-0.233198748	0.120602095	1.933621045	0.053159738	0.156261999	0.233198748
ENSMUSG00000035798	Zdhhc17	320150	protein_coding	281.9068463	-0.233233221	0.089914015	2.593958479	0.009487794	0.040813086	0.233233221
ENSMUSG00000018844	Fndc8	78919	protein_coding	4.011348827	0.233242093	0.531521742	-0.438819478	0.660792345	1	0.233242093
ENSMUSG00000040811	Eml2	72205	protein_coding	212.8136152	-0.23333828	0.108865803	2.143356981	0.032084445	0.106287366	0.23333828
ENSMUSG00000073802	Cdkn2b	12579	protein_coding	635.0130847	-0.233392585	0.12336317	1.891914614	0.058502358	0.167639761	0.233392585
ENSMUSG00000029442	Wdr66	269701	protein_coding	8.130333841	0.233397386	1.067462688	-0.21864688	0.826925131	0.912640622	0.233397386
ENSMUSG00000038422	Hdhd3	72748	protein_coding	232.0661039	0.23343732	0.13090396	-1.783271646	0.074542095	0.199738463	0.23343732
ENSMUSG00000061531	Tmem236	625286	protein_coding	1118.486781	-0.233612531	0.175641449	1.330053538	0.183500632	0.378318282	0.233612531
ENSMUSG00000001901	Kcnh6	192775	protein_coding	44.99816516	0.233619655	0.200379186	-1.165887831	0.2436598	0.454872025	0.233619655
ENSMUSG00000034855	Cxcl10	15945	protein_coding	9.926883353	0.2336812	0.659381701	-0.354394428	0.72304331	0.856549354	0.2336812
ENSMUSG00000020211	Sf3a2	20222	protein_coding	227.7736423	0.233720914	0.137121933	-1.704475055	0.088292372	0.22556625	0.233720914
ENSMUSG00000110507	8030455M16Rik	77171	lncRNA	1.410516161	-0.233724331	1.770689959	0.131996192	0.894987304	1	0.233724331
ENSMUSG00000026873	Phf19	74016	protein_coding	73.61687591	-0.233734691	0.192159728	1.216356277	0.223849232	0.430945001	0.233734691
ENSMUSG00000029535	Triap1	69076	protein_coding	258.9212069	-0.233787832	0.097976739	2.386156488	0.017025505	0.064634295	0.233787832
ENSMUSG00000020496	Rnf187	108660	protein_coding	828.8957294	0.23380682	0.079836688	-2.928563607	0.003405321	0.017689676	0.23380682
ENSMUSG00000019863	Qrsl1	76563	protein_coding	209.8659142	-0.233816149	0.100502106	2.326480092	0.019992953	0.073386004	0.233816149
ENSMUSG00000032298	Neil1	72774	protein_coding	99.8489168	-0.233821428	0.102617355	2.278575868	0.02269229	0.081077558	0.233821428
ENSMUSG00000025402	Nab2	17937	protein_coding	14.34941495	0.233872208	0.331594795	-0.705295172	0.480626564	0.691562872	0.233872208
ENSMUSG00000020178	Adora2a	11540	protein_coding	7.409289904	0.234010139	0.685187938	-0.341526939	0.732706928	0.862924134	0.234010139
ENSMUSG00000050530	Fam171a1	269233	protein_coding	241.499486	-0.234081723	0.103513366	2.26136712	0.023736533	0.083972816	0.234081723
ENSMUSG00000022554	Hgh1	59053	protein_coding	84.6234852	0.234111935	0.126155059	-1.855747491	0.063489558	0.177826758	0.234111935
ENSMUSG00000063888	Rpl7l1	66229	protein_coding	790.3374468	0.234200637	0.094109302	-2.488602417	0.012824629	0.051642509	0.234200637
ENSMUSG00000079197	Psme2	19188	protein_coding	1151.241627	-0.234254911	0.10178902	2.301377005	0.021370331	0.077290766	0.234254911
ENSMUSG00000041688	Amot	27494	protein_coding	546.022296	0.234256784	0.113153428	-2.070257954	0.038428195	0.122133027	0.234256784
ENSMUSG00000069206	Zfp874a	238692	protein_coding	118.1293685	-0.234266194	0.187610117	1.248686359	0.211779812	0.416114999	0.234266194
ENSMUSG00000041168	Lonp1	74142	protein_coding	983.0723049	-0.234269364	0.06612828	3.542650212	0.000396128	0.002907402	0.234269364
ENSMUSG00000046311	Zfp62	22720	protein_coding	343.9804255	-0.234280574	0.086278656	2.715394332	0.00661969	0.030425148	0.234280574
ENSMUSG00000053749	Gm9920	100503794	lncRNA	33.26333551	0.234288352	0.202430828	-1.157374864	0.247119239	0.459127576	0.234288352
ENSMUSG00000018909	Arrb1	109689	protein_coding	1246.253235	-0.234311667	0.093104304	2.516657726	0.011847386	0.048608132	0.234311667

ensembl_gene_id	external_gene_name	entrezgene_id	gene_biotype	baseMean	log2FoldChange	lfcSE	stat	pvalue	padj	absLog2FC
ENSMUSG00000005575	Ube2m	22192	protein_coding	798.3029637	0.234340819	0.104338106	-2.245975403	0.024705583	0.086566519	0.234340819
ENSMUSG00000030428	Ttyh1	57776	protein_coding	63.19619484	-0.234370351	0.214461727	1.092830664	0.274468156	0.490866888	0.234370351
ENSMUSG00000021368	Tbc1d7	67046	protein_coding	77.74681006	0.234372434	0.117914182	-1.987652627	0.046850122	0.141882067	0.234372434
ENSMUSG00000030325	Klrb1c	17059	protein_coding	4.579012026	0.234475128	0.585144761	-0.400713027	0.688631419	0.83386713	0.234475128
ENSMUSG00000036138	Acaa1a	113868	protein_coding	1023.735748	-0.234479419	0.084854751	2.763303348	0.005721956	0.027207712	0.234479419
ENSMUSG00000021411	Pxdc1	66895	protein_coding	26.7556612	0.234608125	0.307561494	-0.762800708	0.445582259	0.662854285	0.234608125
ENSMUSG00000107499	Ccdc142	243510	protein_coding	4.115128326	0.234636888	0.597148058	-0.392929166	0.694371799	1	0.234636888
ENSMUSG00000118401	Gpr52	620246	protein_coding	7.355030286	-0.234822031	0.44381556	0.529098239	0.596737303	0.774787312	0.234822031
ENSMUSG00000117391	4930500A05Rik	74972	lncRNA	0.816735893	0.234830986	1.925621837	-0.121950728	0.90293804	1	0.234830986
ENSMUSG00000059436	Max	17187	protein_coding	1020.829914	-0.234913992	0.107263346	2.190067714	0.028519326	0.096986852	0.234913992
ENSMUSG00000022523	Fgf12	14167	protein_coding	15.6365556	-0.235057345	0.373205876	0.629832914	0.528803909	0.726753308	0.235057345
ENSMUSG00000024043	Arhgap28	268970	protein_coding	3.377703861	0.235133483	0.895777832	-0.262490848	0.79294304	1	0.235133483
ENSMUSG00000041559	Fmod	14264	protein_coding	1.842474253	0.23518286	1.481824879	-0.158711642	0.873896065	1	0.23518286
ENSMUSG00000063268	Parp10	671535	protein_coding	403.0783102	-0.235210081	0.086042881	2.733637894	0.006263889	0.029064867	0.235210081
ENSMUSG00000045165	AI467606	101602	protein_coding	35.02286716	0.235264256	0.213002579	-1.104513651	0.269370385	0.484414956	0.235264256
ENSMUSG00000006586	Runx1t1	12395	protein_coding	9.716754044	-0.235306503	0.485323657	0.484844494	0.627786658	0.796376973	0.235306503
ENSMUSG00000020691	Mettl2	52686	protein_coding	152.3381851	-0.235331816	0.127981079	1.838801631	0.065944368	0.182841722	0.235331816
ENSMUSG00000031818	Cox4i1	12857	protein_coding	8281.545296	-0.235336612	0.087072262	2.702773615	0.006876356	0.031443528	0.235336612
ENSMUSG00000064901	Snora21	100302498	snoRNA	32.03401835	0.235401744	0.267122279	-0.881250879	0.378182046	0.600451717	0.235401744
ENSMUSG00000077575	Gm24888	115485611	snoRNA	2.840927562	0.235418516	0.630064163	-0.373642129	0.708670586	1	0.235418516
ENSMUSG00000020902	Ntn1	18208	protein_coding	5.303619582	0.23544405	0.81890679	-0.287510194	0.773721698	0.88672609	0.23544405
ENSMUSG00000118134	4930549G23Rik	75286	lncRNA	3.109585598	-0.235539032	0.62550717	0.376556885	0.706502934	1	0.235539032
ENSMUSG00000031642	Sh3rf1	59009	protein_coding	515.7326429	-0.235564031	0.094476447	2.493362511	0.012653958	0.051129793	0.235564031
ENSMUSG00000055692	Tmem191	224019	protein_coding	44.05203251	0.235586613	0.21004401	-1.121605957	0.262030018	0.475826228	0.235586613
ENSMUSG00000049047	Armcx3	71703	protein_coding	124.1914394	-0.235595253	0.19590508	1.202599001	0.229131534	0.438050261	0.235595253
ENSMUSG00000016637	Ift27	67042	protein_coding	92.1451884	0.235597043	0.133281883	-1.767659921	0.077117774	0.204779373	0.235597043
ENSMUSG00000116505	Gm10865	100038602	lncRNA	3.662639595	0.235641122	0.635656597	-0.370705068	0.710857215	1	0.235641122
ENSMUSG00000017418	Arl5b	75869	protein_coding	446.5651473	-0.235659297	0.112363025	2.097302896	0.035966771	0.11573563	0.235659297
ENSMUSG00000026972	Arrdc1	215705	protein_coding	738.0458655	-0.235693881	0.061816529	3.812797081	0.000137403	0.00114081	0.235693881
ENSMUSG00000020684	Rasl10b	276952	protein_coding	2.231998047	0.235705705	1.7805403	-0.132378753	0.894684719	1	0.235705705
ENSMUSG00000113902	Ndufb1	102631912	protein_coding	697.9415576	-0.235723633	0.097918702	2.407340255	0.016069191	0.061727521	0.235723633
ENSMUSG00000031376	Atp2b3	320707	protein_coding	4.479601901	0.235750183	1.439757319	-0.163743	0.869933465	0.935658236	0.235750183
ENSMUSG00000051316	Taf7	24074	protein_coding	176.5163238	-0.23582262	0.107465768	2.194397571	0.02820684	0.096042838	0.23582262
ENSMUSG00000038365	Fbxo25	66822	protein_coding	1012.350395	-0.235829337	0.102729456	2.295635011	0.021696759	0.078282779	0.235829337
ENSMUSG00000030800	Prss8	76560	protein_coding	736.1082687	0.235835327	0.068256983	-3.455109152	0.00055007	0.003837987	0.235835327
ENSMUSG00000030604	Zfp626	71163	protein_coding	150.4785	-0.235846754	0.104898516	2.248332606	0.024554989	0.086166827	0.235846754
ENSMUSG00000002343	Armc6	76813	protein_coding	109.92289	0.235861173	0.146971326	-1.604810809	0.108535446	0.262539089	0.235861173
ENSMUSG00000045282	Tmem86b	68255	protein_coding	29.79116338	-0.235874776	0.239701993	0.98403344	0.325099071	0.546957754	0.235874776
ENSMUSG00000036526	Card11	108723	protein_coding	40.71905824	0.235899471	0.21018941	-1.122318536	0.261727028	0.475380569	0.235899471

ensembl_gene_id	external_gene_name	entrezgene_id	gene_biotype	baseMean	log2FoldChange	lfcSE	stat	pvalue	padj	absLog2FC
ENSMUSG00000030763	Lcmt1	30949	protein_coding	217.115282	0.235939358	0.125194831	-1.884577472	0.059486906	0.169754064	0.235939358
ENSMUSG00000035283	Adrb1	11554	protein_coding	2.918723112	-0.236030828	1.323695363	0.178312046	0.858477919	1	0.236030828
ENSMUSG00000078129	Actl10	70362	protein_coding	0.827606995	0.23604865	1.336594099	-0.176604588	0.85981899	1	0.23604865
ENSMUSG00000035004	Igsf6	80719	protein_coding	7.223716171	-0.236072785	0.421537885	0.560027446	0.575460717	0.760617759	0.236072785
ENSMUSG00000015337	Endog	13804	protein_coding	139.4899574	0.236127773	0.13519711	-1.746544535	0.080716376	0.211290803	0.236127773
ENSMUSG00000050666	Vstm4	320736	protein_coding	1.121168604	-0.236172321	1.991821193	0.118571045	0.905615206	1	0.236172321
ENSMUSG00000015027	Galns	50917	protein_coding	320.9466154	-0.236173256	0.062046973	3.806362265	0.000141026	0.001167368	0.236173256
ENSMUSG00000038257	Glra3	110304	protein_coding	0.801418616	-0.236173773	2.199936324	0.107354822	0.914507494	1	0.236173773
ENSMUSG00000038024	Dennd4c	329877	protein_coding	1522.476698	-0.236175374	0.066126961	3.57154434	0.000354882	0.002648228	0.236175374
ENSMUSG00000019787	Trdn	76757	protein_coding	1.753672952	-0.236177031	2.186911291	0.107995707	0.913999098	1	0.236177031
ENSMUSG00000096029	Or56a3	259046	protein_coding	0.897527938	-0.236181457	2.67509266	0.088289075	0.929646922	1	0.236181457
ENSMUSG00000031645	F11	109821	protein_coding	0.992649162	-0.236181783	2.658179121	0.088850966	0.929200353	1	0.236181783
ENSMUSG00000069421	Or6c69b	258543	protein_coding	0.945212167	-0.236182559	2.719997668	0.086831897	0.930805133	1	0.236182559
ENSMUSG00000096069	Or13a28	18362	protein_coding	1.135454616	-0.236183625	2.714333134	0.0870135	0.930660782	1	0.236183625
ENSMUSG00000030647	Ndufc2	68197	protein_coding	977.234188	-0.236191772	0.08778758	2.690491885	0.007134677	0.032448928	0.236191772
ENSMUSG00000036480	Prss56	69453	protein_coding	1.088017621	-0.2361943	3.271757651	0.072191869	0.942449216	1	0.2361943
ENSMUSG00000096584	Or51aa5	258722	protein_coding	1.088017621	-0.2361943	3.271757651	0.072191869	0.942449216	1	0.2361943
ENSMUSG00000070547	Mrgprb1	233231	protein_coding	1.365126374	-0.23619505	3.207033373	0.073649078	0.941289619	1	0.23619505
ENSMUSG00000075478	Slitrk1	76965	protein_coding	1.365126374	-0.23619505	3.207033373	0.073649078	0.941289619	1	0.23619505
ENSMUSG00000058692	Or7g28	258279	protein_coding	1.229524778	-0.236195156	3.241237349	0.07287191	0.941908047	1	0.236195156
ENSMUSG00000075174	Or8k3b	258843	protein_coding	1.230575841	-0.236196306	3.310240875	0.071353208	0.943116649	1	0.236196306
ENSMUSG00000030871	Ears2	67417	protein_coding	242.9588444	0.23622927	0.131636108	-1.794562851	0.072723376	0.196362629	0.23622927
ENSMUSG00000032050	Rdx	19684	protein_coding	252.0668247	0.236292504	0.366139676	-0.645361645	0.518692853	0.719201828	0.236292504
ENSMUSG00000040260	Daam2	76441	protein_coding	10.98173043	0.236349465	0.501586989	-0.471203341	0.637495533	0.802408631	0.236349465
ENSMUSG00000037326	Capn15	50817	protein_coding	305.6705679	0.236378888	0.09625002	-2.455884032	0.01405385	0.055403505	0.236378888
ENSMUSG00000036534	Slc38a7	234595	protein_coding	141.3194117	-0.236455066	0.094663735	2.497842142	0.012495182	0.050636917	0.236455066
ENSMUSG00000023921	Mmut	17850	protein_coding	1024.766578	0.236487191	0.093506186	-2.52910743	0.011435302	0.047391714	0.236487191
ENSMUSG00000050473	Slc35d3	76157	protein_coding	4.196902302	0.236591341	0.775132965	-0.305226783	0.760193435	1	0.236591341
ENSMUSG00000038776	Ephx1	13849	protein_coding	190.471193	0.236594649	0.199187959	-1.187795936	0.234913816	0.444677528	0.236594649
ENSMUSG00000051550	Zfp579	68490	protein_coding	66.64285499	-0.236656787	0.189389311	1.249578374	0.211453607	0.415856243	0.236656787
ENSMUSG00000006587	Snai3	30927	protein_coding	10.15148274	0.236709171	0.391329645	-0.604884333	0.545255853	0.737095216	0.236709171
ENSMUSG00000115518	Gm10791	100038614	lncRNA	10.7694913	-0.23673454	0.361938631	0.654073701	0.513064324	0.714999514	0.23673454
ENSMUSG00000047197	Gjd3	353155	protein_coding	0.880193528	-0.236784909	1.871208629	0.126541159	0.899303571	1	0.236784909
ENSMUSG00000024371	C2	12263	protein_coding	17.94925614	-0.23678542	0.272209795	0.869863699	0.384374895	0.606725963	0.23678542
ENSMUSG00000033487	Fndc3a	319448	protein_coding	2135.944928	0.236790242	0.062866691	-3.766545341	0.000165522	0.001338605	0.236790242
ENSMUSG00000048232	Fbxo10	269529	protein_coding	49.58875382	0.236814524	0.186028046	-1.273004419	0.203016474	0.405213026	0.236814524
ENSMUSG00000042271	Nxt2	237082	protein_coding	187.7217339	-0.236894626	0.122250568	1.937779349	0.052650148	0.155233806	0.236894626
ENSMUSG00000020053	Igf1	16000	protein_coding	3.250390382	-0.236938311	1.645909347	0.14395587	0.885535318	1	0.236938311
ENSMUSG00000039395	Mreg	381269	protein_coding	10.68253615	-0.236941855	0.424310189	0.558416604	0.576559937	0.761222618	0.236941855

ensembl_gene_id	external_gene_name	entrezgene_id	gene_biotype	baseMean	log2FoldChange	lfcSE	stat	pvalue	padj	absLog2FC
ENSMUSG00000023827	Agpat4	68262	protein_coding	796.2489715	-0.236959181	0.109381724	2.166350764	0.030284393	0.101670354	0.236959181
ENSMUSG00000034768	Asb16	217217	protein_coding	11.72894317	0.237007156	0.342222159	-0.692553507	0.488589796	0.696497169	0.237007156
ENSMUSG00000089804	Gm16136	105245924	lncRNA	68.88767446	-0.237023563	0.138517659	1.711143292	0.087054664	0.223580206	0.237023563
ENSMUSG00000041936	Agrn	11603	protein_coding	850.8461615	-0.237027958	0.133976377	1.769177253	0.076864299	0.204314673	0.237027958
ENSMUSG00000021553	Slc28a3	114304	protein_coding	59.39375905	-0.237043566	0.24949127	0.950107659	0.342057552	0.564006111	0.237043566
ENSMUSG00000028078	Dclk2	70762	protein_coding	23.04028338	0.237078445	0.240427143	-0.986071881	0.324097841	0.545970799	0.237078445
ENSMUSG00000068246	Apol9b	71898	protein_coding	37.57521582	-0.237096316	0.49568827	0.478317383	0.632424326	0.799512805	0.237096316
ENSMUSG00000036427	Gpi1	14751	protein_coding	5563.1764	-0.23710081	0.072469142	3.271748538	0.001068846	0.006724161	0.23710081
ENSMUSG00000028179	Cth	107869	protein_coding	1076.870089	-0.237203918	0.083677301	2.834746298	0.004586209	0.022621462	0.237203918
ENSMUSG00000026103	Gls	14660	protein_coding	1160.410907	-0.237292381	0.056811821	4.176813507	2.96E-05	0.000292855	0.237292381
ENSMUSG00000002102	Psmc3	19182	protein_coding	1721.501858	0.237298826	0.076575208	-3.098898869	0.001942413	0.011080142	0.237298826
ENSMUSG00000047502	Mroh7	381538	protein_coding	9.26069389	0.237349209	0.689774207	-0.344096961	0.730773368	0.861267215	0.237349209
ENSMUSG00000118516	Cyp2d13	68444	protein_coding	102.2889808	-0.237349922	0.258223662	0.919164108	0.358009741	0.580790426	0.237349922
ENSMUSG00000000001	Gnai3	14679	protein_coding	2306.328636	0.237352357	0.069655391	-3.407523132	0.000655554	0.004448039	0.237352357
ENSMUSG00000019828	Grm1	14816	protein_coding	2.14702026	0.237379294	1.743389937	-0.13615961	0.891695104	1	0.237379294
ENSMUSG00000038520	Tbc1d17	233204	protein_coding	385.7286094	-0.237386142	0.070004971	3.390989812	0.000696407	0.00466172	0.237386142
ENSMUSG00000038665	Dgki	320127	protein_coding	21.67724023	-0.237398506	0.407995966	0.581864837	0.560657727	0.749146509	0.237398506
ENSMUSG00000003233	Dvl3	13544	protein_coding	271.0858033	0.237422529	0.169917088	-1.397284588	0.162328008	0.347734431	0.237422529
ENSMUSG00000033107	Rnf125	67664	protein_coding	65.34931151	-0.237530626	0.202808066	1.171208971	0.24151479	0.452145476	0.237530626
ENSMUSG00000062961	Ccdc177	380768	protein_coding	8.838276383	0.237598968	0.378468606	-0.627790427	0.530141238	0.727438372	0.237598968
ENSMUSG00000032355	Mlip	69642	protein_coding	5.825166649	-0.237604966	0.940555745	0.252621886	0.800560416	0.901189824	0.237604966
ENSMUSG00000031355	Arhgap6	11856	protein_coding	26.90622287	0.237610217	0.237225541	-1.00162156	0.316526404	0.537548029	0.237610217
ENSMUSG00000031774	Psme3ip1	102122	protein_coding	512.7235777	0.237611184	0.059796413	-3.973669497	7.08E-05	0.000636513	0.237611184
ENSMUSG00000025959	Klf7	93691	protein_coding	260.7094764	0.237652152	0.109691631	-2.166547705	0.030269359	0.101661214	0.237652152
ENSMUSG00000030654	Arl6ip1	54208	protein_coding	1892.634373	0.237727575	0.071951608	-3.303992531	0.000953184	0.006112962	0.237727575
ENSMUSG00000026655	Fam107b	66540	protein_coding	1637.815533	-0.237833673	0.132291527	1.797799735	0.072208749	0.195501402	0.237833673
ENSMUSG00000062526	Mppe1	225651	protein_coding	177.9951539	-0.237848838	0.132572745	1.794100578	0.072797117	0.196529604	0.237848838
ENSMUSG00000029644	Pdx1	18609	protein_coding	6.555628951	-0.237866903	0.400762879	0.59353527	0.552822986	0.743248294	0.237866903
ENSMUSG00000014725	Adam28	13522	protein_coding	3.115529418	-0.237939496	1.57082402	0.151474317	0.879601569	1	0.237939496
ENSMUSG00000090083	Rnf8	58230	protein_coding	97.79898766	-0.238007866	0.13876622	1.715171498	0.086313789	0.222126524	0.238007866
ENSMUSG00000053617	Sh3pxd2a	14218	protein_coding	232.1460335	-0.238033201	0.122862836	1.937389761	0.052697717	0.155346323	0.238033201
ENSMUSG00000087331	1810021B22Rik	69120	lncRNA	24.32364556	0.23804411	0.206131743	-1.154815395	0.248166031	0.460473465	0.23804411
ENSMUSG00000025733	Rhot2	214952	protein_coding	574.5055749	-0.238129694	0.107179043	2.22179344	0.026297268	0.090795126	0.238129694
ENSMUSG00000087579	Hectd2os	668215	lncRNA	1.219167261	0.238188536	1.47460953	-0.161526513	0.871678731	1	0.238188536
ENSMUSG00000018209	Stk4	58231	protein_coding	702.0451212	-0.238222266	0.08238673	2.891512572	0.003833923	0.019529117	0.238222266
ENSMUSG00000005397	Nid1	18073	protein_coding	6.687141421	0.238227582	0.442415979	-0.538469659	0.590252846	0.770846581	0.238227582
ENSMUSG00000049687	Pheta2	338368	protein_coding	9.243610834	0.238236169	0.388069215	-0.613901231	0.539280597	0.733766095	0.238236169
ENSMUSG00000027597	Ahcy	269378	protein_coding	1096.492587	-0.2382436	0.102042162	2.3347565	0.019556145	0.072087093	0.2382436
ENSMUSG00000064669	Gm24461	115489568	snRNA	1.314840088	0.238332654	1.074878906	-0.221729772	0.824524253	1	0.238332654

Supplementary Table 1

ensembl_gene_id	external_gene_name	entrezgene_id	gene_biotype	baseMean	log2FoldChange	lfcSE	stat	pvalue	padj	absLog2FC
ENSMUSG00000032883	Acsl3	74205	protein_coding	1871.102864	-0.238352532	0.097039812	2.45623449	0.014040151	0.055375938	0.238352532
ENSMUSG00000020737	Jpt1	15374	protein_coding	1983.96074	-0.238362701	0.075079003	3.174825077	0.001499269	0.008896486	0.238362701
ENSMUSG00000038172	Ttc39b	69863	protein_coding	904.7816593	-0.238384944	0.091748128	2.598254026	0.009369915	0.040464115	0.238384944
ENSMUSG00000037706	Cd81	12520	protein_coding	896.2942862	-0.238476279	0.176924706	1.347896988	0.177691517	0.370029503	0.238476279
ENSMUSG00000029426	Scarb2	12492	protein_coding	1905.683768	-0.23871833	0.055582477	4.294848729	1.75E-05	0.000182944	0.23871833
ENSMUSG00000031558	Slit2	20563	protein_coding	69.61443798	0.238760466	0.233891093	-1.020818979	0.307340211	0.527754626	0.238760466
ENSMUSG00000004567	Mcoln1	94178	protein_coding	317.3939204	-0.238786506	0.098955496	2.413069656	0.015818796	0.060935759	0.238786506
ENSMUSG00000006998	Psmd2	21762	protein_coding	2369.167352	0.238840555	0.083064431	-2.875364978	0.004035607	0.020312105	0.238840555
ENSMUSG00000049287	Iba57	216792	protein_coding	241.437919	-0.238882402	0.096504115	2.475359765	0.013310202	0.053169976	0.238882402
ENSMUSG00000089842	Pitpnm2os2	100047482	lncRNA	1.397514702	0.23893431	1.341022527	-0.178173226	0.858586936	1	0.23893431
ENSMUSG00000018995	Nars2	244141	protein_coding	189.8799629	-0.238945893	0.130590577	1.829733042	0.067289867	0.185483473	0.238945893
ENSMUSG00000031799	Tpm4	326618	protein_coding	1422.721371	-0.239012743	0.116277336	2.055540231	0.039826854	0.125611281	0.239012743
ENSMUSG00000074500	Zfp558	72230	protein_coding	21.25250594	0.239015042	0.268755716	-0.889339379	0.373820714	0.595844868	0.239015042
ENSMUSG00000052301	Doc2a	13446	protein_coding	1.387111745	0.239025907	1.855973745	-0.128787332	0.89752593	1	0.239025907
ENSMUSG00000070493	Chchd2	14004	protein_coding	2816.881232	-0.239091055	0.08563278	2.792050622	0.005237516	0.025356887	0.239091055
ENSMUSG00000097779	4833407H14Rik	73899	lncRNA	19.50218618	0.23915415	0.423896318	-0.564180768	0.572631115	0.758030479	0.23915415
ENSMUSG00000086379	1700026D11Rik	70371	lncRNA	1.484035425	0.239168176	2.077808118	-0.115105998	0.908361106	1	0.239168176
ENSMUSG00000035021	Baz1a	217578	protein_coding	421.7778791	0.239177704	0.097670614	-2.448819507	0.014332525	0.056246986	0.239177704
ENSMUSG00000028673	Fuca1	71665	protein_coding	3072.602607	-0.239184501	0.060468702	3.955509097	7.64E-05	0.000680996	0.239184501
ENSMUSG00000022993	Tex49	73863	protein_coding	21.05659747	-0.239267745	0.267673001	0.89388076	0.371385693	0.593868753	0.239267745
ENSMUSG00000001240	Ramp2	54409	protein_coding	1.922389149	0.239293354	0.875039139	-0.273465886	0.784495112	1	0.239293354
ENSMUSG00000106583	4930447N08Rik	71647	lncRNA	9.541758434	-0.239353748	0.329764413	0.72583256	0.467941424	0.681625181	0.239353748
ENSMUSG00000021609	Slc6a3	13162	protein_coding	1.204093892	-0.239410528	1.809667784	0.132295292	0.894750731	1	0.239410528
ENSMUSG00000031775	Pllp	67801	protein_coding	248.3067963	-0.239637798	0.124716505	1.921460178	0.054673718	0.159435243	0.239637798
ENSMUSG00000031349	Nsdhl	18194	protein_coding	385.614839	0.239649309	0.096317395	-2.488120745	0.012842012	0.051674706	0.239649309
ENSMUSG00000028419	Chmp5	76959	protein_coding	1351.553118	0.239679837	0.070377334	-3.40563964	0.000660092	0.004471192	0.239679837
ENSMUSG00000049823	Zbtb12	193736	protein_coding	36.77641783	0.239684847	0.188704359	-1.270160628	0.204027419	0.406185509	0.239684847
ENSMUSG00000072620	Slfn2	20556	protein_coding	16.46149834	0.239705966	0.324750378	-0.738123749	0.460439253	0.674684457	0.239705966
ENSMUSG00000019820	Utrn	22288	protein_coding	1940.570233	0.239819647	0.06630065	-3.617153781	0.00029786	0.002264671	0.239819647
ENSMUSG00000036646	Man1b1	227619	protein_coding	1612.718236	0.239836018	0.078122091	-3.07001533	0.002140478	0.011982322	0.239836018
ENSMUSG00000015942	Gtf2ird2	114674	protein_coding	106.4166042	0.239934506	0.128525978	-1.866817205	0.061927119	0.174605505	0.239934506
ENSMUSG00000026656	Fcgr2b	14130	protein_coding	2.208400069	0.239950506	1.282658142	-0.187072843	0.85160352	1	0.239950506
ENSMUSG00000003559	As3mt	57344	protein_coding	547.2438961	0.239962467	0.12050437	-1.991317556	0.046445987	0.141033318	0.239962467
ENSMUSG00000029828	4921507P07Rik	70821	protein_coding	8.6366949	-0.2399992	0.379013582	0.633220578	0.526589609	0.725037586	0.2399992
ENSMUSG00000040824	Snrpd2	107686	protein_coding	424.1903831	-0.240000183	0.099320449	2.416422657	0.015673856	0.060447934	0.240000183
ENSMUSG00000077450	Rab11b	19326	protein_coding	627.2813281	-0.240007918	0.085633353	2.802738752	0.00506707	0.024640043	0.240007918
ENSMUSG00000053819	Camk2d	108058	protein_coding	1244.302031	0.24003223	0.051854279	-4.628976276	3.67E-06	4.53E-05	0.24003223
ENSMUSG00000031826	Usp10	22224	protein_coding	503.1833669	0.24003568	0.090667998	-2.64741347	0.008111011	0.036011679	0.24003568
ENSMUSG00000035429	Ptprh	545902	protein_coding	743.855371	-0.240053651	0.301926944	0.79507197	0.426571638	0.64620651	0.240053651

ensembl_gene_id	external_gene_name	entrezgene_id	gene_biotype	baseMean	log2FoldChange	lfcSE	stat	pvalue	padj	absLog2FC
ENSMUSG00000020929	Eftud2	20624	protein_coding	685.7322169	0.240107607	0.095672619	-2.509679452	0.01208408	0.049395172	0.240107607
ENSMUSG00000027742	Cog6	67542	protein_coding	824.3721224	0.240121072	0.083396905	-2.879256381	0.003986141	0.020124469	0.240121072
ENSMUSG00000076123	Mir700	735285	miRNA	0.604723788	0.240182063	1.875238775	-0.12808079	0.898085038	1	0.240182063
ENSMUSG00000028104	Polr3gl	69870	protein_coding	106.0592122	-0.240248018	0.102390262	2.346395196	0.018955996	0.070425045	0.240248018
ENSMUSG00000022710	Usp7	252870	protein_coding	2146.651576	0.240249991	0.05470695	-4.391580763	1.13E-05	0.000124365	0.240249991
ENSMUSG00000054099	Slc25a40	319653	protein_coding	240.3767146	-0.24025578	0.0982439	2.445503278	0.014465013	0.056645774	0.24025578
ENSMUSG00000061517	Sox21	223227	protein_coding	61.43197519	0.240271925	0.179960825	-1.335134604	0.181832296	0.376052415	0.240271925
ENSMUSG00000033589	Reep4	72549	protein_coding	444.2415006	0.240281594	0.076836856	-3.127165873	0.001765003	0.010209199	0.240281594
ENSMUSG00000025505	Tmem80	71448	protein_coding	91.90616517	-0.240356148	0.124071977	1.937231542	0.052717046	0.155375567	0.240356148
ENSMUSG00000071657	Bscl2	14705	protein_coding	435.5047249	-0.240393397	0.087852322	2.736335167	0.006212771	0.028908905	0.240393397
ENSMUSG00000091931	Gon7	100233175	protein_coding	42.69745708	0.240432196	0.189056975	-1.271744646	0.203463862	0.405748366	0.240432196
ENSMUSG00000074738	Fndc10	230991	protein_coding	24.42888891	-0.240483407	0.21010249	1.14460046	0.252374702	0.464617944	0.240483407
ENSMUSG00000021474	Sfxn1	14057	protein_coding	632.0734894	-0.24048732	0.112160226	2.144140828	0.032021605	0.10614307	0.24048732
ENSMUSG00000051768	Xrcc1	22594	protein_coding	283.7842762	0.24057364	0.106605577	-2.256670315	0.024028683	0.084679828	0.24057364
ENSMUSG00000070803	Cited4	56222	protein_coding	105.8485021	0.240655552	0.127294749	-1.89053793	0.058686053	0.168040558	0.240655552
ENSMUSG00000054383	Pnma1	70481	protein_coding	5.437831497	0.240697832	0.800983479	-0.300502867	0.76379361	0.881554912	0.240697832
ENSMUSG00000039089	L3mbtl3	237339	protein_coding	269.3276332	0.240742129	0.086374825	-2.787179344	0.005316904	0.025643505	0.240742129
ENSMUSG00000076448	Mir763	791076	miRNA	0.741381929	-0.240744467	1.507491673	0.159698704	0.873118422	1	0.240744467
ENSMUSG00000021699	Pde4d	238871	protein_coding	306.7545952	-0.240803983	0.085600046	2.813129118	0.004906195	0.023928204	0.240803983
ENSMUSG00000021696	Elovl7	74559	protein_coding	1065.020211	-0.240859919	0.122657412	1.963680103	0.049567201	0.14799363	0.240859919
ENSMUSG00000012117	Dhdds	67422	protein_coding	339.3509756	0.240868698	0.080788645	-2.981467224	0.002868708	0.015327126	0.240868698
ENSMUSG00000033904	Ccp110	101565	protein_coding	57.23302247	0.240905139	0.173633617	-1.387433742	0.165309542	0.352338897	0.240905139
ENSMUSG00000054737	Zfp182	319535	protein_coding	112.1191758	-0.240942543	0.118091724	2.040299987	0.041320456	0.129138155	0.240942543
ENSMUSG00000030107	Usp18	24110	protein_coding	45.18966116	0.240998068	0.441855464	-0.545422854	0.585462726	0.767388797	0.240998068
ENSMUSG00000022199	Slc22a17	59049	protein_coding	16.48130905	0.241039502	0.296731238	-0.812315897	0.416610391	0.636776036	0.241039502
ENSMUSG00000037172	Dennd11	243780	protein_coding	721.412624	-0.241064563	0.204437997	1.179157331	0.238335533	0.448589299	0.241064563
ENSMUSG00000046567	4930430F08Rik	68281	protein_coding	35.73222637	0.241135254	0.210669059	-1.144616371	0.252368108	0.464617944	0.241135254
ENSMUSG00000063457	Rps15	20054	protein_coding	5145.310868	-0.241257334	0.091563215	2.634871799	0.008416907	0.037046055	0.241257334
ENSMUSG00000038745	Nlrp6	101613	protein_coding	3094.516356	-0.241266475	0.113114546	2.132939438	0.032929695	0.108456517	0.241266475
ENSMUSG00000074793	Hspa12b	72630	protein_coding	2.987102186	0.241436457	0.778400885	-0.310169813	0.756431825	1	0.241436457
ENSMUSG00000016194	Hsd11b1	15483	protein_coding	2.518097602	0.241461699	0.854051905	-0.282724853	0.777387764	1	0.241461699
ENSMUSG00000004849	Ap1s1	11769	protein_coding	764.783446	-0.241536477	0.079071653	3.054653184	0.002253209	0.012461318	0.241536477
ENSMUSG00000118087	4833438C02Rik	100503392	lncRNA	81.7076119	-0.241596158	0.172596417	1.39977505	0.161580692	0.346538122	0.241596158
ENSMUSG00000040857	Erf	13875	protein_coding	400.2094692	-0.241689696	0.095669602	2.526295622	0.011527244	0.047685597	0.241689696
ENSMUSG00000057147	Dph6	66632	protein_coding	61.75814032	0.241763558	0.15459733	-1.563827516	0.117858081	0.278322371	0.241763558
ENSMUSG00000031641	Cbr4	234309	protein_coding	218.2895902	-0.241766209	0.122265596	1.977385433	0.047998079	0.144615997	0.241766209
ENSMUSG00000026571	Dcaf6	74106	protein_coding	331.0316023	-0.241769659	0.079406853	3.044695145	0.002329163	0.012812723	0.241769659
ENSMUSG00000047749	Zc3hav1l	209032	protein_coding	23.11025002	0.241799636	0.236820955	-1.021022977	0.307243553	0.527698391	0.241799636
ENSMUSG00000030220	Arhgdib	11857	protein_coding	15.07776002	0.241825111	0.369817015	-0.653904771	0.51317316	0.715090842	0.241825111

ensembl_gene_id	external_gene_name	entrezgene_id	gene_biotype	baseMean	log2FoldChange	lfcSE	stat	pvalue	padj	absLog2FC
ENSMUSG00000038368	Focad	230393	protein_coding	230.8519469	0.24196797	0.094079071	-2.571963837	0.010112345	0.042953833	0.24196797
ENSMUSG00000041679	Fbxl9	234684	protein_coding	43.35937392	-0.241980938	0.156270415	1.548475685	0.121507811	0.284375933	0.241980938
ENSMUSG00000002486	Tchp	77832	protein_coding	87.65235191	0.241984867	0.158215648	-1.529462295	0.126149879	0.292089426	0.241984867
ENSMUSG00000004266	Ptpn6	15170	protein_coding	326.55576	-0.242190736	0.090211426	2.684701328	0.007259465	0.032853604	0.242190736
ENSMUSG00000033998	Kcnk1	16525	protein_coding	1080.223591	0.242280872	0.096596158	-2.508183315	0.012135369	0.049543507	0.242280872
ENSMUSG00000020022	Ndufa12	66414	protein_coding	879.6929161	-0.242329024	0.084272066	2.875555748	0.004033169	0.020306023	0.242329024
ENSMUSG00000025498	Irf7	54123	protein_coding	985.4363221	-0.242350224	0.397370524	0.609884753	0.541938153	0.7356816	0.242350224
ENSMUSG00000025421	Hdhd2	76987	protein_coding	117.6664947	-0.242431836	0.128654571	1.884362394	0.059515973	0.169807667	0.242431836
ENSMUSG00000028675	Pnrc2	52830	protein_coding	1129.71745	0.242479789	0.086329773	-2.808762042	0.004973239	0.024219425	0.242479789
ENSMUSG00000078816	Prkcg	18752	protein_coding	10.68703957	0.242566707	0.407803194	-0.594813161	0.551968369	0.742703956	0.242566707
ENSMUSG00000045983	Eif4g1	208643	protein_coding	6219.577783	0.242584003	0.061604052	-3.937792966	8.22E-05	0.000726214	0.242584003
ENSMUSG00000027223	Mapk8ip1	19099	protein_coding	26.68077339	-0.24260367	0.326393322	0.743286254	0.457308392	0.672184316	0.24260367
ENSMUSG00000021102	Glrx5	73046	protein_coding	614.0312147	0.242606887	0.089600733	-2.707643998	0.006776267	0.031092878	0.242606887
ENSMUSG00000023050	Map3k12	26404	protein_coding	32.96156865	-0.242652785	0.270168367	0.898153947	0.369103482	0.591783971	0.242652785
ENSMUSG00000003410	Elavl3	15571	protein_coding	5.832940472	-0.242670161	1.067000965	0.227431997	0.820087842	0.909231489	0.242670161
ENSMUSG00000026344	Lypd1	72585	protein_coding	3.760208786	-0.242850518	0.744509438	0.326188636	0.744281639	1	0.242850518
ENSMUSG00000024258	Polr2d	69241	protein_coding	137.2645973	0.242852057	0.109875185	-2.210253898	0.027087546	0.092992945	0.242852057
ENSMUSG00000025058	Tasl	71398	protein_coding	3.212031609	0.242938041	0.691143003	-0.351501846	0.725211888	1	0.242938041
ENSMUSG00000027035	Cers6	241447	protein_coding	2359.595539	-0.242953649	0.06252775	3.885533234	0.000102106	0.000875933	0.242953649
ENSMUSG00000012405	Rpl15	66480	protein_coding	2405.234837	-0.242956348	0.123357083	1.969537074	0.04889145	0.14663883	0.242956348
ENSMUSG00000038256	Bcl9	77578	protein_coding	182.4540923	0.243009828	0.191177308	-1.271122765	0.203684978	0.405918699	0.243009828
ENSMUSG00000021311	Mtr	238505	protein_coding	123.4979709	0.24301863	0.139714181	-1.739398458	0.081964699	0.213630847	0.24301863
ENSMUSG00000060771	Tsga10	211484	protein_coding	64.5137722	-0.243057407	0.159958678	1.519501229	0.128636379	0.295987343	0.243057407
ENSMUSG00000059772	Slx1b	75764	protein_coding	147.4670254	-0.243061274	0.099323796	2.447160542	0.014398669	0.0564529	0.243061274
ENSMUSG00000026761	Orc4	26428	protein_coding	331.7678693	0.243087854	0.067971426	-3.576324143	0.00034846	0.002603828	0.243087854
ENSMUSG00000021224	Numb	18222	protein_coding	736.5076042	0.243103679	0.074739489	-3.252680494	0.00114322	0.007110783	0.243103679
ENSMUSG00000033948	Zswim5	74464	protein_coding	165.9298045	-0.243145437	0.094730573	2.566705014	0.010266991	0.043464619	0.243145437
ENSMUSG00000020063	Sirt1	93759	protein_coding	244.4208574	-0.243166647	0.11435142	2.126485587	0.033462848	0.109658699	0.243166647
ENSMUSG00000021044	Adck1	72113	protein_coding	162.2766267	-0.243167255	0.096522188	2.519288676	0.01175922	0.048327356	0.243167255
ENSMUSG00000078435	AU041133	216177	protein_coding	51.34367716	-0.243200906	0.169816583	1.432138737	0.15210412	0.332739097	0.243200906
ENSMUSG00000050721	Plekho2	102595	protein_coding	13.89762513	-0.243238413	0.430528002	0.564976987	0.572089417	0.757617052	0.243238413
ENSMUSG00000070509	Rgma	244058	protein_coding	20.70429734	0.243266584	0.32232676	-0.754720409	0.450416758	0.667221236	0.243266584
ENSMUSG00000024074	Crim1	50766	protein_coding	698.7661528	-0.243320595	0.070400575	3.456230198	0.000547787	0.003829873	0.243320595
ENSMUSG00000022787	Wdr53	68980	protein_coding	89.41475805	-0.243326463	0.134744629	1.805834221	0.070944238	0.192819808	0.243326463
ENSMUSG00000024197	Plin3	66905	protein_coding	1359.701036	0.243331455	0.059351183	-4.099858549	4.13E-05	0.000396224	0.243331455
ENSMUSG00000085990	Gm16731	105247126	lncRNA	10.71589802	0.243424062	0.38560898	-0.631271768	0.527862843	0.726026078	0.243424062
ENSMUSG00000031536	Polb	18970	protein_coding	198.5747644	-0.243426479	0.091332648	2.665273423	0.007692575	0.034482949	0.243426479
ENSMUSG00000045867	Cradd	12905	protein_coding	76.37058878	-0.243428454	0.141278575	1.723038719	0.084881523	0.219498448	0.243428454
ENSMUSG00000034161	Scx	20289	protein_coding	6.346028324	0.243435777	0.522589169	-0.465826296	0.641339841	0.804182698	0.243435777

ensembl_gene_id	external_gene_name	entrezgene_id	gene_biotype	baseMean	log2FoldChange	lfcSE	stat	pvalue	padj	absLog2FC
ENSMUSG00000026766	Mmadhc	109129	protein_coding	635.5301556	0.24356335	0.103457044	-2.354246167	0.018560315	0.069204119	0.24356335
ENSMUSG00000020467	Efemp1	216616	protein_coding	0.715411271	-0.243586078	1.89008975	0.128875403	0.89745624	1	0.243586078
ENSMUSG00000064358	mt-Co3	17710	protein_coding	15.8555211	0.243654134	0.669048226	-0.364180226	0.715723432	0.852528618	0.243654134
ENSMUSG00000073639	Rab18	19330	protein_coding	816.7695485	0.243661458	0.071043913	-3.429730228	0.000604182	0.004152083	0.243661458
ENSMUSG00000065591	Gm22753	115487847	snRNA	1.910692349	-0.243669145	0.732450794	0.332676471	0.739378512	1	0.243669145
ENSMUSG00000017561	Crlf3	54394	protein_coding	125.2709568	-0.243676696	0.108656041	2.242642871	0.024919853	0.087113984	0.243676696
ENSMUSG00000069892	9930111J21Rik2	245240	protein_coding	2.618218965	-0.243742361	0.84666315	0.287885874	0.7734341	1	0.243742361
ENSMUSG00000020277	Pfkl	18641	protein_coding	3188.534636	-0.243780343	0.098268277	2.48076339	0.013110137	0.052535985	0.243780343
ENSMUSG00000070421	Or52n4	259051	protein_coding	1.341653401	-0.243790244	1.427930403	0.17072978	0.864436245	1	0.243790244
ENSMUSG00000037995	Igsf9	93842	protein_coding	1379.981352	0.243829632	0.154034453	-1.58295516	0.113431685	0.270265596	0.243829632
ENSMUSG00000019861	Gopc	94221	protein_coding	519.9334188	0.243930569	0.131526121	-1.854616915	0.063650951	0.178188134	0.243930569
ENSMUSG00000038967	Pdk2	18604	protein_coding	501.7473971	0.243998832	0.114868646	-2.124155199	0.033657168	0.110042513	0.243998832
ENSMUSG00000026430	Rassf5	54354	protein_coding	13.41573901	0.244073354	0.428956701	-0.568992985	0.5693609	0.755154277	0.244073354
ENSMUSG00000052407	Ccdc171	320226	protein_coding	58.80388543	0.244119833	0.178288432	-1.369241015	0.170923949	0.360812744	0.244119833
ENSMUSG00000038879	Nipal2	223473	protein_coding	428.927085	0.244148158	0.082100168	-2.973783908	0.002941521	0.015594313	0.244148158
ENSMUSG00000025495	Ptdss2	27388	protein_coding	270.9568346	-0.244170228	0.091441213	2.670242667	0.007579645	0.034050667	0.244170228
ENSMUSG00000022906	Parp9	80285	protein_coding	605.8744435	-0.244278254	0.133941542	1.823767673	0.068187203	0.187361642	0.244278254
ENSMUSG00000063200	Nol7	70078	protein_coding	512.561897	0.244279695	0.088081287	-2.773343834	0.005548346	0.026496643	0.244279695
ENSMUSG00000019715	Gle1	74412	protein_coding	295.1931402	0.244310141	0.124108939	-1.968513649	0.049008967	0.146868346	0.244310141
ENSMUSG00000025757	Hspa4l	18415	protein_coding	825.986033	-0.244330243	0.138449588	1.764759621	0.077604174	0.205674101	0.244330243
ENSMUSG00000081984	Dnajb3	15504	protein_coding	19.85369476	0.244415257	0.338341117	-0.722393008	0.470052884	0.683555242	0.244415257
ENSMUSG00000085004	5430427M07Rik	71349	lncRNA	49.53493919	-0.244464994	0.532751078	0.458872828	0.646325492	0.807226927	0.244464994
ENSMUSG00000020105	Lrig3	320398	protein_coding	1248.363228	-0.244646108	0.105506059	2.318787278	0.020406573	0.074572727	0.244646108
ENSMUSG00000006021	Kptn	70394	protein_coding	165.2797366	-0.244983635	0.096071807	2.55000548	0.010772123	0.045218819	0.244983635
ENSMUSG00000003437	Paf1	54624	protein_coding	445.4813817	0.244992368	0.079020291	-3.10037287	0.001932772	0.011044218	0.244992368
ENSMUSG00000025534	Gusb	110006	protein_coding	471.0964945	0.245089897	0.093151388	-2.631092264	0.008511093	0.037373936	0.245089897
ENSMUSG00000099254	Mir7030	102465623	miRNA	0.659985141	-0.245204787	1.592158797	0.154007746	0.877603628	1	0.245204787
ENSMUSG00000030286	Emc3	66087	protein_coding	780.1422895	0.245284029	0.076515745	-3.205667407	0.001347496	0.008157095	0.245284029
ENSMUSG00000030224	Strap	20901	protein_coding	1041.658248	0.245400228	0.076564459	-3.205145446	0.001349942	0.008168908	0.245400228
ENSMUSG00000049502	Dtx3l	209200	protein_coding	684.6200442	-0.245512516	0.149951522	1.637279259	0.101572133	0.250425106	0.245512516
ENSMUSG00000052456	Get3	56495	protein_coding	652.7423513	0.245551989	0.073484026	-3.341569623	0.000833061	0.005454866	0.245551989
ENSMUSG00000033059	Pygb	110078	protein_coding	3121.316241	0.245619338	0.111116359	-2.210469643	0.027072585	0.092985996	0.245619338
ENSMUSG00000016494	Cd34	12490	protein_coding	4.557053465	0.245630213	0.767993325	-0.319833786	0.749094334	0.874014884	0.245630213
ENSMUSG00000099706	Gm29536	102636764	lncRNA	1.081133225	-0.245695568	2.318270806	0.105982255	0.915596431	1	0.245695568
ENSMUSG00000019437	Tlcd1	68385	protein_coding	113.8579512	-0.245814151	0.138055797	1.780542043	0.074987295	0.200249061	0.245814151
ENSMUSG00000032656	Marchf3	320253	protein_coding	78.80990822	0.245864775	0.38669118	-0.635816866	0.524895793	0.723368866	0.245864775
ENSMUSG00000112925	Gm31763	102634101	lncRNA	4.773041758	-0.245868815	0.609450792	0.403426853	0.686634236	0.83310074	0.245868815
ENSMUSG00000030498	Gas2	14453	protein_coding	35.57208848	0.245869253	0.181708102	-1.353100112	0.17602365	0.368018849	0.245869253
ENSMUSG00000024824	Rad9a	19367	protein_coding	88.55185857	0.245919877	0.161165764	-1.525881619	0.127039349	0.293636687	0.245919877

Supplementary Table 1

ensembl_gene_id	external_gene_name	entrezgene_id	gene_biotype	baseMean	log2FoldChange	lfcSE	stat	pvalue	padj	absLog2FC
ENSMUSG00000028885	Smpdl3b	100340	protein_coding	71.3783588	0.24596875	0.170663737	-1.441247884	0.149514665	0.328900667	0.24596875
ENSMUSG00000087620	5330434G04Rik	100043213	lncRNA	1.951641558	-0.246013033	2.32173845	0.105960701	0.915613533	1	0.246013033
ENSMUSG00000057530	Ece1	230857	protein_coding	1815.649389	-0.246221209	0.061937563	3.975313171	7.03E-05	0.000632887	0.246221209
ENSMUSG00000016409	Nkap	67050	protein_coding	176.8909683	-0.246231382	0.116424777	2.114939686	0.034435088	0.112051438	0.246231382
ENSMUSG00000028737	Aldh4a1	212647	protein_coding	239.9479563	0.24623306	0.090014287	-2.735488629	0.006228774	0.028942592	0.24623306
ENSMUSG00000047409	Ctdspl	69274	protein_coding	1185.281631	0.246316386	0.082724495	-2.977550805	0.002905615	0.015463527	0.246316386
ENSMUSG00000051319	Mtln	67885	protein_coding	149.9519705	-0.246458558	0.107371665	2.295378015	0.02171147	0.078315179	0.246458558
ENSMUSG00000089671	Gm16537	102638434	lncRNA	1.234546564	0.246567514	1.094063827	-0.225368491	0.821692622	1	0.246567514
ENSMUSG00000025494	Sigirr	24058	protein_coding	350.0996862	-0.246650413	0.070886494	3.479512077	0.000502328	0.003561803	0.246650413
ENSMUSG00000027457	Snph	241727	protein_coding	77.98691047	-0.246688606	0.191672203	1.287033808	0.198082505	0.398335908	0.246688606
ENSMUSG00000020444	Guk1	14923	protein_coding	762.4364379	0.246729482	0.108250343	-2.279248953	0.022652271	0.08100219	0.246729482
ENSMUSG00000048707	Tprn	97031	protein_coding	1616.994224	0.246818892	0.115532697	-2.136355327	0.032650462	0.107730217	0.246818892
ENSMUSG00000022559	Fbxl6	30840	protein_coding	172.693566	-0.247000629	0.091306149	2.705191615	0.0068265	0.03127124	0.247000629
ENSMUSG00000003746	Man1a	17155	protein_coding	1001.314946	-0.247025764	0.066094094	3.737486192	0.000185869	0.001488577	0.247025764
ENSMUSG00000039126	Prune2	353211	protein_coding	26.25242973	0.247037023	0.383288263	-0.644520187	0.519238171	0.719474674	0.247037023
ENSMUSG00000025173	Wdr45b	66840	protein_coding	479.0900109	0.247043644	0.049718117	-4.96888582	6.73E-07	9.64E-06	0.247043644
ENSMUSG00000033703	Fcsk	234730	protein_coding	763.5356677	0.247056246	0.06308783	-3.916068196	9.00E-05	0.000786839	0.247056246
ENSMUSG00000046352	Gjb2	14619	protein_coding	152.3780895	0.247062026	0.134459487	-1.837445847	0.066144104	0.183317181	0.247062026
ENSMUSG00000076011	Mir652	723976	miRNA	3.178487793	-0.247075957	0.674056176	0.366550988	0.713953973	1	0.247075957
ENSMUSG00000065822	Snord15a	449630	snoRNA	103.7713765	-0.247107515	0.1686556	1.465160448	0.142877148	0.318675475	0.247107515
ENSMUSG00000071708	Sms	20603	protein_coding	101.7950746	-0.247142637	0.141033669	1.752366222	0.079710856	0.209275846	0.247142637
ENSMUSG00000026156	B3gat2	280645	protein_coding	18.16490968	-0.247168251	0.261636213	0.944701988	0.344811042	0.566362983	0.247168251
ENSMUSG00000035885	Cox8a	12868	protein_coding	5437.324101	-0.247177347	0.077097353	3.206041935	0.001345744	0.008149473	0.247177347
ENSMUSG00000020009	Ifngr1	15979	protein_coding	2390.429537	-0.247185188	0.089101855	2.774186763	0.00553399	0.026443376	0.247185188
ENSMUSG00000022580	Rhpn1	14787	protein_coding	1.353787863	0.247323032	1.813675407	-0.136365654	0.891532225	1	0.247323032
ENSMUSG00000048076	Arf1	11840	protein_coding	4975.350239	0.247394042	0.079806105	-3.099938802	0.001935606	0.011045129	0.247394042
ENSMUSG00000031728	Zfp821	75871	protein_coding	151.7814857	-0.247452553	0.140319855	1.763489228	0.077818012	0.205991262	0.247452553
ENSMUSG00000045327	6330549D23Rik	229613	transcribed_processed_pseudogene	11.52152403	-0.24760125	0.34914579	0.709162926	0.478223377	0.689848083	0.24760125
ENSMUSG00000025817	Nudt5	53893	protein_coding	319.054395	0.247611719	0.143077831	-1.730608565	0.083521603	0.216662858	0.247611719
ENSMUSG00000060679	Mrps9	69527	protein_coding	422.7652655	-0.247619051	0.109673547	2.25778282	0.023959203	0.084507107	0.247619051
ENSMUSG00000089984	Fbxo24	71176	protein_coding	17.26199983	-0.247817723	0.354580077	0.698904814	0.484611519	0.695055986	0.247817723
ENSMUSG00000037989	Wnk2	75607	protein_coding	256.1705839	0.247827227	0.205581431	-1.205494223	0.228012567	0.436620149	0.247827227
ENSMUSG00000034243	Golgb1	224139	protein_coding	3597.660359	0.247843936	0.061099857	-4.056375072	4.98E-05	0.000465269	0.247843936
ENSMUSG00000010554	Mettl16	67493	protein_coding	271.3846053	0.247894613	0.092488993	-2.680260701	0.007356484	0.033210765	0.247894613
ENSMUSG00000043614	Vps37d	194309	protein_coding	12.45044718	-0.247907228	0.362883657	0.683158976	0.494506392	0.700667401	0.247907228
ENSMUSG00000020792	Exoc7	53413	protein_coding	588.5834499	0.247980608	0.059079162	-4.197429316	2.70E-05	0.000269538	0.247980608
ENSMUSG00000068747	Sort1	20661	protein_coding	5526.846597	-0.248021799	0.08454647	2.933555917	0.003351033	0.017451579	0.248021799
ENSMUSG00000028184	Adgrl2	99633	protein_coding	321.675023	-0.248041887	0.096638744	2.566691954	0.010267378	0.043464619	0.248041887
ENSMUSG00000118100	Gm50340	115489003	lncRNA	9.651272823	-0.248159575	0.342517846	0.724515753	0.46874916	0.682320284	0.248159575

ensembl_gene_id	external_gene_name	entrezgene_id	gene_biotype	baseMean	log2FoldChange	lfcSE	stat	pvalue	padj	absLog2FC
ENSMUSG00000068882	Ssb	20823	protein_coding	937.8280708	0.248247857	0.095484526	-2.599875275	0.009325765	0.040294527	0.248247857
ENSMUSG00000024220	Zfp523	224656	protein_coding	226.19673	0.248274647	0.08090479	-3.068726189	0.002149735	0.012021919	0.248274647
ENSMUSG00000021660	Btf3	218490	protein_coding	1718.639353	-0.248312568	0.077373534	3.209270071	0.001330724	0.008079258	0.248312568
ENSMUSG00000006931	P3h4	66180	protein_coding	39.28415494	-0.248356801	0.189764237	1.308765049	0.190613949	0.388090095	0.248356801
ENSMUSG00000073423	Zfp414	328801	protein_coding	72.7554379	-0.24837521	0.134220816	1.850496937	0.064241959	0.179449017	0.24837521
ENSMUSG00000017057	Il13ra1	16164	protein_coding	1703.244687	0.248392818	0.176197739	-1.409738963	0.158616775	0.342539221	0.248392818
ENSMUSG00000079499	6530402F18Rik	76220	lncRNA	4.483674929	0.248432237	0.919328737	-0.270232211	0.786981613	0.893375436	0.248432237
ENSMUSG00000020434	4921536K21Rik	67430	protein_coding	32.17336813	0.24844281	0.204722762	-1.213557338	0.224916806	0.432209358	0.24844281
ENSMUSG00000028411	Aptx	66408	protein_coding	131.5270672	0.248454833	0.135430483	-1.834556205	0.066571473	0.184147622	0.248454833
ENSMUSG00000028035	Dnajb4	67035	protein_coding	167.629072	-0.248472362	0.126890362	1.958165758	0.050210566	0.149293713	0.248472362
ENSMUSG00000068327	Tlx2	21909	protein_coding	0.922438628	-0.24861591	1.586021037	0.156754484	0.875438346	1	0.24861591
ENSMUSG00000006784	Odad4	74407	protein_coding	19.41512155	-0.248659147	0.31146916	0.798342753	0.424671615	0.644224421	0.248659147
ENSMUSG00000097331	F420014N23Rik	100038591	lncRNA	9.366613986	-0.248663811	0.428022149	0.580960148	0.561267313	0.749718386	0.248663811
ENSMUSG00000036291	Ap5m1	74385	protein_coding	505.2603806	-0.248731664	0.107154012	2.321253855	0.020273147	0.074167202	0.248731664
ENSMUSG00000031167	Rbm3	19652	protein_coding	1182.615487	0.248781557	0.145705091	-1.707432154	0.087741758	0.224907418	0.248781557
ENSMUSG00000097743	Gm16973	100503823	lncRNA	19.28364219	-0.24879377	0.238610656	1.042676694	0.297098055	0.516613382	0.24879377
ENSMUSG00000045502	Hcar2	80885	protein_coding	5.368212514	-0.248815788	0.631702472	0.393881296	0.69366868	0.837421924	0.248815788
ENSMUSG00000022285	Ywhaz	22631	protein_coding	3328.027835	0.248837314	0.085268197	-2.918289849	0.00351957	0.018191607	0.248837314
ENSMUSG00000023952	Gtpbp2	56055	protein_coding	1661.783552	0.248852786	0.188851321	-1.317718005	0.187598065	0.383842701	0.248852786
ENSMUSG00000025068	Gsto1	14873	protein_coding	3875.776188	-0.248922437	0.076518839	3.253086957	0.001141586	0.007103295	0.248922437
ENSMUSG00000034744	Nagk	56174	protein_coding	456.1158945	-0.249019696	0.066510305	3.744076879	0.000181058	0.001453571	0.249019696
ENSMUSG00000020814	Mxra7	67622	protein_coding	26.94068279	0.249186016	0.209596704	-1.188883278	0.234485601	0.444019633	0.249186016
ENSMUSG00000027605	Acss2	60525	protein_coding	1611.045378	-0.249188588	0.179663659	1.386972689	0.165450091	0.352592954	0.249188588
ENSMUSG00000096948	Gm4221	100043089	lncRNA	3.317957483	0.249219268	0.743418377	-0.335234204	0.737448422	1	0.249219268
ENSMUSG00000035725	Prkx	19108	protein_coding	370.5476149	-0.249269734	0.111335099	2.238914199	0.025161499	0.087791464	0.249269734
ENSMUSG00000044052	Ccr10	12777	protein_coding	12.61034892	-0.2492891	0.447550391	0.557007892	0.577522037	0.761475268	0.2492891
ENSMUSG00000049807	Arhgap23	58996	protein_coding	12.31250878	0.249346818	0.566460572	-0.44018389	0.659803927	0.816323585	0.249346818
ENSMUSG00000030309	Caprin2	232560	protein_coding	74.70183851	-0.249437277	0.19937423	1.251100897	0.210897668	0.415010021	0.249437277
ENSMUSG00000033222	Ttf2	74044	protein_coding	192.6705253	0.249560693	0.136163871	-1.832796698	0.06683281	0.184592245	0.249560693
ENSMUSG00000025094	Slc18a2	214084	protein_coding	34.56931683	-0.249566938	0.223526464	1.116498391	0.264208858	0.478777869	0.249566938
ENSMUSG00000021620	Acot12	74156	protein_coding	2.701134488	-0.249603534	1.401988247	0.178035397	0.858695177	1	0.249603534
ENSMUSG00000096938	9530052E02Rik	619321	lncRNA	1.871774418	0.249615533	1.201820333	-0.207697878	0.835464875	1	0.249615533
ENSMUSG00000005983	1700037C18Rik	73261	protein_coding	40.11056603	-0.249616005	0.16694201	1.495225828	0.134855515	0.305727036	0.249616005
ENSMUSG00000031255	Sytl4	27359	protein_coding	284.36999	-0.249616128	0.123115079	2.02750248	0.042611051	0.131923303	0.249616128
ENSMUSG00000021816	Ppp3cb	19056	protein_coding	514.1811904	0.249753759	0.097245747	-2.568274369	0.010220622	0.043322217	0.249753759
ENSMUSG00000002731	Prkra	23992	protein_coding	346.1960507	-0.249812214	0.091152365	2.740600476	0.006132703	0.028656498	0.249812214
ENSMUSG00000032387	Rbpms2	71973	protein_coding	5.143771636	0.249823359	0.572189385	-0.436609565	0.662394525	0.817346435	0.249823359
ENSMUSG00000052609	Plekhg3	263406	protein_coding	699.7721007	-0.249850456	0.091269119	2.737513629	0.006190555	0.028864834	0.249850456
ENSMUSG00000028028	Alpk1	71481	protein_coding	428.1280956	0.249872765	0.077675713	-3.216871202	0.001295968	0.007888281	0.249872765

ensembl_gene_id	external_gene_name	entrezgene_id	gene_biotype	baseMean	log2FoldChange	lfcSE	stat	pvalue	padj	absLog2FC
ENSMUSG00000021460	Auh	11992	protein_coding	251.5255861	-0.249912331	0.098034413	2.549230664	0.010796087	0.045254367	0.249912331
ENSMUSG00000033543	Gtf2a2	235459	protein_coding	294.9137802	0.249974304	0.103425874	-2.416941665	0.015651525	0.060376822	0.249974304
ENSMUSG00000029121	Crmp1	12933	protein_coding	6.654970246	0.249984945	0.913484157	-0.273660953	0.784345188	0.891996173	0.249984945
ENSMUSG00000022742	Cpox	12892	protein_coding	324.4683896	0.250062777	0.109567839	-2.282264379	0.022473736	0.080540642	0.250062777
ENSMUSG00000034947	Tmem106a	217203	protein_coding	187.479996	-0.25019233	0.163698432	1.528373405	0.126419854	0.292394604	0.25019233
ENSMUSG00000051590	Map3k19	22625	protein_coding	3.338909151	-0.250205433	1.399154711	0.178826138	0.858074221	1	0.250205433
ENSMUSG00000046834	Krt1	16678	protein_coding	1.615613748	-0.250252317	1.719776255	0.145514462	0.884304698	1	0.250252317
ENSMUSG00000036466	Megf11	214058	protein_coding	40.56098549	-0.250300596	0.231871256	1.079480919	0.280373395	0.498019388	0.250300596
ENSMUSG00000028643	Svbp	69216	protein_coding	153.5661911	-0.250322238	0.130112432	1.923891786	0.054368149	0.158736855	0.250322238
ENSMUSG00000062794	Zfp599	235048	protein_coding	68.44548954	-0.250405266	0.157749628	1.587358839	0.112431396	0.268968865	0.250405266
ENSMUSG00000038005	Hpf1	72612	protein_coding	138.8724749	0.250466121	0.138441843	-1.809179329	0.070423148	0.191813931	0.250466121
ENSMUSG00000074655	Gm1527	385263	protein_coding	0.836582822	0.250471944	1.452043343	-0.17249619	0.863047457	1	0.250471944
ENSMUSG00000003623	Crot	74114	protein_coding	733.9077185	0.250493667	0.070186798	-3.568957042	0.000358405	0.002670749	0.250493667
ENSMUSG00000031562	Dctd	320685	protein_coding	60.59424156	0.25050746	0.267243273	-0.937376109	0.348565126	0.57042954	0.25050746
ENSMUSG00000020412	Ascc2	75452	protein_coding	689.2759561	-0.250608369	0.057379731	4.367541738	1.26E-05	0.000137056	0.250608369
ENSMUSG00000086290	Snhg12	100039864	lncRNA	126.9600314	-0.250630292	0.155874174	1.607901334	0.10785679	0.261367195	0.250630292
ENSMUSG00000040675	Mthfd1l	270685	protein_coding	199.5872377	0.250642946	0.128803258	-1.945936377	0.051662383	0.152917797	0.250642946
ENSMUSG00000027236	Eif3j1	78655	protein_coding	213.0253005	-0.250683139	0.117008466	2.142435915	0.03215842	0.106468355	0.250683139
ENSMUSG00000035842	Ddx11	320209	protein_coding	135.0800011	0.250841204	0.127053138	-1.974301518	0.048347468	0.145350463	0.250841204
ENSMUSG00000026799	Med27	68975	protein_coding	111.2291501	0.250842986	0.104752947	-2.394615083	0.016637821	0.063542317	0.250842986
ENSMUSG00000034158	Lrrc58	320184	protein_coding	913.0095054	0.250850083	0.100643865	-2.492452791	0.012686419	0.051198321	0.250850083
ENSMUSG00000068154	Insm1	53626	protein_coding	25.9646222	0.250862861	0.272850095	-0.919416434	0.357877797	0.580790426	0.250862861
ENSMUSG00000020458	Rtn4	68585	protein_coding	862.0291734	-0.250927107	0.071737119	3.497869861	0.00046899	0.003361502	0.250927107
ENSMUSG00000022702	Hira	15260	protein_coding	114.2780495	0.250933978	0.099556316	-2.520522948	0.01171806	0.048232278	0.250933978
ENSMUSG00000047888	Tnrc6b	213988	protein_coding	1188.607526	0.250983823	0.085022068	-2.951984446	0.003157388	0.016594879	0.250983823
ENSMUSG00000067071	Hes6	55927	protein_coding	593.9466114	-0.251026462	0.132003707	1.901662221	0.057215328	0.164953548	0.251026462
ENSMUSG00000032127	Vps11	71732	protein_coding	413.7603677	0.251036541	0.072359362	-3.469302854	0.000521811	0.003679413	0.251036541
ENSMUSG00000032044	Rpusd4	71989	protein_coding	158.3902666	-0.251072953	0.097417424	2.577290009	0.009957837	0.042442029	0.251072953
ENSMUSG00000026434	Nucks1	98415	protein_coding	1700.768538	0.251089478	0.143248294	-1.752827003	0.079631707	0.209167807	0.251089478
ENSMUSG00000035247	Hectd1	207304	protein_coding	3509.654419	0.251130333	0.058365032	-4.302753317	1.69E-05	0.000177661	0.251130333
ENSMUSG00000030884	Uqcrc2	67003	protein_coding	3910.210541	-0.251367085	0.082576743	3.044042135	0.002334224	0.012832018	0.251367085
ENSMUSG00000038526	Car14	23831	protein_coding	6.612092239	0.251478914	0.493751357	-0.509322984	0.61052585	0.784161879	0.251478914
ENSMUSG00000035530	Eif1	20918	protein_coding	1898.356646	-0.251489454	0.104322921	2.410682635	0.015922697	0.061293105	0.251489454
ENSMUSG00000079020	Slc45a4	106068	protein_coding	350.0184222	0.251495018	0.090143571	-2.789938495	0.005271806	0.025463175	0.251495018
ENSMUSG00000034300	Fam53c	66306	protein_coding	287.2285929	0.251501755	0.089492469	-2.810311954	0.00494935	0.024110197	0.251501755
ENSMUSG00000018559	Ctdnep1	67181	protein_coding	373.5761126	0.251516192	0.120272767	-2.091214813	0.036508812	0.117137463	0.251516192
ENSMUSG00000033022	Cdo1	12583	protein_coding	333.6885399	0.251526659	0.160890951	-1.563336275	0.11797352	0.278515327	0.251526659
ENSMUSG00000020265	Sumo3	20610	protein_coding	937.6505378	0.251624722	0.087147094	-2.887356441	0.003884938	0.019745882	0.251624722
ENSMUSG00000026207	Speg	11790	protein_coding	10.00927154	-0.251661373	1.50015844	0.167756529	0.866774823	0.933837244	0.251661373

Supplementary Table 1

ensembl_gene_id	external_gene_name	entrezgene_id	gene_biotype	baseMean	log2FoldChange	lfcSE	stat	pvalue	padj	absLog2FC
ENSMUSG00000084453	Gm24596	115486854	snRNA	0.602479715	0.251757321	1.681821878	-0.149693213	0.881006663	1	0.251757321
ENSMUSG00000050567	Maml1	103806	protein_coding	211.9123572	-0.251779992	0.121130257	2.07858877	0.037655164	0.120138597	0.251779992
ENSMUSG00000117698	BC037704	100502982	lncRNA	10.31888751	0.251818163	0.335022099	-0.751646426	0.452263716	0.668637691	0.251818163
ENSMUSG00000055053	Nfic	18029	protein_coding	140.0744978	-0.251842605	0.194324872	1.295987499	0.194979792	0.394112152	0.251842605
ENSMUSG00000021692	Dimt1	66254	protein_coding	52.25722425	-0.251879036	0.167380214	1.504831609	0.132367375	0.301713259	0.251879036
ENSMUSG00000015759	Cnih1	12793	protein_coding	539.9719113	0.251894288	0.084697798	-2.974035859	0.002939107	0.015586518	0.251894288
ENSMUSG00000020821	Kif1c	16562	protein_coding	3928.726624	-0.251947473	0.060799676	4.143894978	3.41E-05	0.000334053	0.251947473
ENSMUSG00000023883	Phf10	72057	protein_coding	350.7423977	0.251949314	0.082671741	-3.047586899	0.002306868	0.012715494	0.251949314
ENSMUSG00000022724	Riox2	67014	protein_coding	225.9234368	-0.251950313	0.096731279	2.604641609	0.009197041	0.039805161	0.251950313
ENSMUSG00000026037	Orc2	18393	protein_coding	175.949398	0.252050682	0.11295752	-2.231375851	0.025656243	0.08904733	0.252050682
ENSMUSG00000032177	Pde4a	18577	protein_coding	27.90905933	-0.25214508	0.231414456	1.089582234	0.275897212	0.492718348	0.25214508
ENSMUSG00000033955	Tnks1bp1	228140	protein_coding	1887.823527	-0.252188643	0.066116459	3.814309557	0.000136564	0.001134417	0.252188643
ENSMUSG00000074749	Kiz	228730	protein_coding	212.1644345	-0.252256733	0.116753663	2.160589445	0.030727067	0.102738771	0.252256733
ENSMUSG00000109398	Gm3854	100416477	protein_coding	1.691607249	-0.252267148	1.330659974	0.189580474	0.849637889	1	0.252267148
ENSMUSG00000025142	Aspscr1	68938	protein_coding	511.5567168	0.252295865	0.071594976	-3.523932546	0.000425192	0.003094592	0.252295865
ENSMUSG00000038187	Btbd10	68815	protein_coding	163.5340108	-0.252325877	0.104367264	2.41767262	0.015620123	0.060282943	0.252325877
ENSMUSG00000098557	Kctd12	239217	protein_coding	650.0495667	0.252332132	0.196788472	-1.282250579	0.199754759	0.40082018	0.252332132
ENSMUSG00000000876	Pxmp4	59038	protein_coding	403.3845128	0.252365948	0.07788878	-3.240080911	0.001194958	0.007373206	0.252365948
ENSMUSG00000030655	Smg1	233789	protein_coding	2521.196632	0.252378721	0.128856335	-1.958605457	0.05015901	0.149272747	0.252378721
ENSMUSG00000032744	Heyl	56198	protein_coding	2.761000628	0.252409205	1.270981508	-0.198593924	0.842580407	1	0.252409205
ENSMUSG00000075706	Gpx4	625249	protein_coding	812.5209727	-0.252477931	0.112964205	2.23502596	0.025415645	0.088476845	0.252477931
ENSMUSG00000096010	H4c16	320332	protein_coding	787.5414501	0.252478734	0.09996556	-2.525657186	0.011548211	0.047736459	0.252478734
ENSMUSG00000052331	Ankrd44	329154	protein_coding	204.3607902	-0.252516896	0.149789728	1.685809157	0.091832563	0.232097177	0.252516896
ENSMUSG00000054733	Msra	110265	protein_coding	429.1323605	-0.252537831	0.063160261	3.998365827	6.38E-05	0.000581283	0.252537831
ENSMUSG00000090266	Mettl23	74319	protein_coding	149.2489083	-0.252551919	0.106718616	2.366521685	0.017956122	0.067342119	0.252551919
ENSMUSG00000022106	Rcbtb2	105670	protein_coding	193.366014	0.252600335	0.105940494	-2.384360549	0.017108831	0.06492078	0.252600335
ENSMUSG00000026579	F5	14067	protein_coding	8.697328289	-0.252624748	0.577204903	0.437669095	0.661626176	0.816921981	0.252624748
ENSMUSG00000034560	Washc4	319277	protein_coding	441.4067678	0.252771906	0.080542719	-3.138358235	0.001698971	0.009886119	0.252771906
ENSMUSG00000036834	Plch1	269437	protein_coding	646.6568773	0.252789409	0.145672316	-1.735329098	0.082682531	0.215060531	0.252789409
ENSMUSG00000039646	Vasn	246154	protein_coding	76.91545062	-0.252852353	0.157257172	1.607890757	0.107859107	0.261367195	0.252852353
ENSMUSG00000055681	Cope	59042	protein_coding	1556.51221	0.252854648	0.083303393	-3.035346335	0.002402596	0.013164059	0.252854648
ENSMUSG00000025934	Gsta3	14859	protein_coding	189.8485891	-0.252868645	0.153829498	1.64382416	0.100212529	0.2481124	0.252868645
ENSMUSG00000025362	Rps26	27370	protein_coding	3506.955216	-0.252881204	0.097697445	2.588411646	0.009641968	0.041336308	0.252881204
ENSMUSG00000053111	Fank1	66930	protein_coding	7.633535255	-0.252881425	0.455751398	0.554867031	0.578985614	0.762469572	0.252881425
ENSMUSG00000022885	St6gal1	20440	protein_coding	1193.89072	-0.25290226	0.374397729	0.675490904	0.499363928	0.704201299	0.25290226
ENSMUSG00000021420	Fars2	69955	protein_coding	485.2328471	-0.252955912	0.066493129	3.804241375	0.000142239	0.001176235	0.252955912
ENSMUSG00000048701	Ccdc6	76551	protein_coding	1165.142705	0.253030164	0.081110473	-3.1195745	0.001811125	0.010450353	0.253030164
ENSMUSG00000001804	Dsg4	16769	protein_coding	3.1744412	-0.253049098	0.786703525	0.321657511	0.74771217	1	0.253049098
ENSMUSG00000061028	Clasrp	53609	protein_coding	401.1485904	0.253051485	0.155847095	-1.623716401	0.104436349	0.255505462	0.253051485

ensembl_gene_id	external_gene_name	entrezgene_id	gene_biotype	baseMean	log2FoldChange	lfcSE	stat	pvalue	padj	absLog2FC
ENSMUSG00000003382	Etv3	27049	protein_coding	857.9848066	0.253097732	0.097550754	-2.59452359	0.009472211	0.040777919	0.253097732
ENSMUSG00000064655	Gm25788	115490049	snoRNA	184.7197399	0.253147144	0.294571408	-0.859374457	0.390133957	0.612475705	0.253147144
ENSMUSG00000028495	Rps6	20104	protein_coding	2270.231933	-0.253173975	0.148848245	1.700886534	0.088964296	0.226849015	0.253173975
ENSMUSG00000024271	Elp2	58523	protein_coding	340.4827967	0.253220658	0.075653929	-3.347091983	0.000816641	0.00535797	0.253220658
ENSMUSG00000065990	Aurkaip1	66077	protein_coding	678.2502808	-0.253221126	0.066363437	3.815672273	0.000135813	0.001128742	0.253221126
ENSMUSG00000045414	Dipk2a	68861	protein_coding	338.3034945	0.253263853	0.119263364	-2.123567917	0.033706291	0.110135672	0.253263853
ENSMUSG00000090622	A930033H14Rik	320700	lncRNA	5.978900149	0.253347587	0.69273455	-0.365721022	0.714573258	0.851895956	0.253347587
ENSMUSG00000100475	Gm29543	105245595	lncRNA	0.786870164	0.253375255	1.20184436	-0.21082202	0.833026156	1	0.253375255
ENSMUSG00000028307	Aldob	230163	protein_coding	2097.32192	-0.253386872	0.079394035	3.191510196	0.001415311	0.008495983	0.253386872
ENSMUSG00000017639	Rab11fip4	268451	protein_coding	569.3544164	-0.253449716	0.136894776	1.851419931	0.064109164	0.179258167	0.253449716
ENSMUSG00000025380	Fscn2	238021	protein_coding	4.373327456	0.253543611	0.586810052	-0.432071009	0.665689804	1	0.253543611
ENSMUSG00000024891	Slc29a2	13340	protein_coding	17.8861044	-0.253548807	0.303886244	0.83435434	0.404081339	0.625281037	0.253548807
ENSMUSG00000039307	Hexdc	238023	protein_coding	160.6752596	-0.253560193	0.117269733	2.162196386	0.030603042	0.102448537	0.253560193
ENSMUSG00000004040	Stat3	20848	protein_coding	1727.539255	0.253605191	0.088931607	-2.851687955	0.004348777	0.021650799	0.253605191
ENSMUSG00000031285	Dcx	13193	protein_coding	4.36715505	-0.253638579	1.168565526	0.217051225	0.828168424	1	0.253638579
ENSMUSG00000021936	Mapk8	26419	protein_coding	316.8993019	0.253680061	0.085850857	-2.954892581	0.003127779	0.016452754	0.253680061
ENSMUSG00000026279	Thap4	67026	protein_coding	748.5009064	-0.25381308	0.068020636	3.731412926	0.000190409	0.001515379	0.25381308
ENSMUSG00000087060	Eldr	72735	lncRNA	39.8785474	0.253877432	0.233226252	-1.088545696	0.276354275	0.493321208	0.253877432
ENSMUSG00000021135	Slc10a1	20493	protein_coding	18.92924193	0.253935273	0.406514512	-0.624664718	0.532191134	0.728866518	0.253935273
ENSMUSG00000029401	Rilpl2	80291	protein_coding	134.6422837	0.25398574	0.132495404	-1.91694	0.055245555	0.160507581	0.25398574
ENSMUSG00000064811	Gm22358	115488216	snoRNA	3.280849832	-0.254010285	0.621996724	0.408378815	0.682995585	1	0.254010285
ENSMUSG00000031340	Gabre	14404	protein_coding	25.5460836	0.254027744	0.430006893	-0.590752725	0.554686115	0.744703672	0.254027744
ENSMUSG00000090115	Usp49	224836	protein_coding	114.43641	-0.254193928	0.142695203	1.781376827	0.074850911	0.200014231	0.254193928
ENSMUSG00000090290	Tarbp1	212728	protein_coding	179.320561	-0.254197095	0.135936575	1.869968368	0.061488211	0.173816344	0.254197095
ENSMUSG00000031703	Itfg1	71927	protein_coding	1079.121204	-0.254221983	0.090875221	2.797484081	0.005150231	0.024970906	0.254221983
ENSMUSG00000032180	Tmed1	17083	protein_coding	88.40913091	-0.254269882	0.143330083	1.774016149	0.076060479	0.202492786	0.254269882
ENSMUSG00000087281	Gm16015	115490183	lncRNA	12.26252466	-0.254339719	0.468122976	0.54331817	0.586910756	0.768390968	0.254339719
ENSMUSG00000034278	Dnajc17	69408	protein_coding	86.78381278	-0.254347653	0.115803726	2.196368472	0.02806558	0.095640734	0.254347653
ENSMUSG00000001103	Sebox	18292	protein_coding	0.670790068	-0.254355966	1.541807372	0.164972597	0.868965551	1	0.254355966
ENSMUSG00000023232	Serinc2	230779	protein_coding	1458.525435	-0.254431116	0.06261987	4.063105158	4.84E-05	0.000454104	0.254431116
ENSMUSG00000042106	Inka1	68176	protein_coding	3.382475087	-0.254445992	0.741089359	0.343340501	0.731342315	1	0.254445992
ENSMUSG00000015468	Notch4	18132	protein_coding	126.9360663	0.254500311	0.160160392	-1.589034017	0.112052713	0.268218365	0.254500311
ENSMUSG00000096433	Zfp994	240038	protein_coding	172.3467726	-0.254569546	0.10991919	2.315969999	0.020559907	0.074950618	0.254569546
ENSMUSG00000030613	Ccdc90b	66365	protein_coding	186.0625569	-0.254635434	0.148100405	1.719343272	0.085551885	0.220716112	0.254635434
ENSMUSG00000050520	Cldn8	54420	protein_coding	1534.067741	0.254750674	0.186831397	-1.363532457	0.172714763	0.363275924	0.254750674
ENSMUSG00000078945	Naip2	17948	protein_coding	1593.315335	-0.254757567	0.090973029	2.800363686	0.005104506	0.024790788	0.254757567
ENSMUSG00000036975	Tmem177	66343	protein_coding	113.0761886	-0.25484957	0.11594054	2.198105775	0.027941569	0.095316478	0.25484957
ENSMUSG00000058454	Dhcr7	13360	protein_coding	696.3686814	-0.254984669	0.087901179	2.900810551	0.003721988	0.0190648	0.254984669
ENSMUSG00000021611	Tert	21752	protein_coding	49.58289233	0.255003196	0.186153709	-1.369852883	0.17073283	0.360508479	0.255003196

ensembl_gene_id	external_gene_name	entrezgene_id	gene_biotype	baseMean	log2FoldChange	lfcSE	stat	pvalue	padj	absLog2FC
ENSMUSG00000037692	Ahdc1	230793	protein_coding	385.3053635	0.255020697	0.18501217	-1.378399577	0.168079954	0.356592544	0.255020697
ENSMUSG00000029729	Zkscan1	74570	protein_coding	1425.653857	-0.255064076	0.100852267	2.529086204	0.011435994	0.047391714	0.255064076
ENSMUSG00000037813	D630003M21Rik	228846	protein_coding	6.693502661	0.25515146	0.642736619	-0.396976697	0.691384645	0.835769466	0.25515146
ENSMUSG00000049600	Zbtb45	232879	protein_coding	132.1783258	0.255243938	0.111854963	-2.281918761	0.022494137	0.080578781	0.255243938
ENSMUSG00000027018	Hat1	107435	protein_coding	222.7543857	0.255247622	0.150262779	-1.698674969	0.089380442	0.227592128	0.255247622
ENSMUSG00000051703	Tmem198	319998	protein_coding	14.5698324	-0.255263435	0.300036042	0.850775905	0.394893849	0.617252913	0.255263435
ENSMUSG00000032724	Abtb2	99382	protein_coding	87.29706344	-0.255284593	0.217358261	1.174487651	0.240199755	0.450500711	0.255284593
ENSMUSG00000027305	Ndufaf1	69702	protein_coding	158.5594265	-0.255291736	0.115109329	2.217819695	0.026567129	0.091535482	0.255291736
ENSMUSG00000079317	Trappc2	66226	protein_coding	30.37040877	-0.255306066	0.175349559	1.45598351	0.145397178	0.322533618	0.255306066
ENSMUSG00000046204	Pnma2	239157	protein_coding	2.717920323	-0.255338328	1.382775038	0.184656449	0.853498507	1	0.255338328
ENSMUSG00000077274	Gm22786	115485822	snoRNA	12.57717166	0.255374521	0.506658556	-0.504036729	0.614235567	0.78673795	0.255374521
ENSMUSG00000030207	Fam234b	74525	protein_coding	4.864155826	0.255391215	0.877587817	-0.291014995	0.771039851	0.885199039	0.255391215
ENSMUSG00000022793	B4galt4	56375	protein_coding	711.5027562	0.25551756	0.11585446	-2.205504736	0.027418696	0.093901357	0.25551756
ENSMUSG00000028051	Hcn3	15168	protein_coding	13.98544794	0.255559828	0.40124681	-0.636914293	0.524180672	0.72280558	0.255559828
ENSMUSG00000031245	Hmgn5	50887	protein_coding	162.6336161	-0.255563637	0.142458116	1.793956322	0.072820141	0.19654843	0.255563637
ENSMUSG00000037266	Rsrp1	27981	protein_coding	1540.201249	-0.255588353	0.109983825	2.323872196	0.020132345	0.073733764	0.255588353
ENSMUSG00000053604	Rpia	19895	protein_coding	325.416866	-0.255635863	0.10285147	2.485485746	0.012937477	0.05194233	0.255635863
ENSMUSG00000028871	Rspo1	192199	protein_coding	1.815758265	0.255671986	1.554332181	-0.164489926	0.869345476	1	0.255671986
ENSMUSG00000022313	Utp23	78581	protein_coding	86.56827302	0.255690646	0.137206063	-1.8635521	0.06238463	0.175505924	0.255690646
ENSMUSG00000031201	Brcc3	210766	protein_coding	357.9422538	0.255694053	0.080216082	-3.187565968	0.001434757	0.008569101	0.255694053
ENSMUSG00000022241	Tars	110960	protein_coding	1260.137458	0.255698025	0.099536497	-2.568887133	0.010202568	0.043279016	0.255698025
ENSMUSG00000053716	Dusp7	235584	protein_coding	89.54766608	0.255776407	0.159330162	-1.605323209	0.108422694	0.262304772	0.255776407
ENSMUSG00000039031	Arhgap18	73910	protein_coding	571.3383387	-0.255776764	0.10995216	2.32625501	0.020004951	0.073397413	0.255776764
ENSMUSG00000022263	Trio	223435	protein_coding	1418.049079	0.255867475	0.165859156	-1.542679229	0.122908624	0.286211349	0.255867475
ENSMUSG00000015247	Nipsnap3b	66536	protein_coding	678.6299117	0.255923476	0.086687174	-2.952264609	0.003154525	0.016585107	0.255923476
ENSMUSG00000036333	Kidins220	77480	protein_coding	1448.29918	-0.255963493	0.064787509	3.950815491	7.79E-05	0.000692995	0.255963493
ENSMUSG00000064919	Gm22572	115489570	snRNA	1.032727485	-0.255965213	1.247502146	0.205182182	0.83742979	1	0.255965213
ENSMUSG00000031264	Btk	12229	protein_coding	9.019640469	0.25606685	0.489868078	-0.522726141	0.601164844	0.777615016	0.25606685
ENSMUSG00000036158	Prickle1	106042	protein_coding	16.73154585	-0.256081676	0.254138377	1.007646618	0.313624141	0.534874425	0.256081676
ENSMUSG00000086938	4930481A15Rik	74931	lncRNA	77.19805723	0.256211543	0.192620793	-1.3301344	0.183473993	0.378310591	0.256211543
ENSMUSG00000045257	Morn2	378462	protein_coding	40.18825948	0.256393602	0.189765885	-1.351104824	0.176661856	0.36877688	0.256393602
ENSMUSG00000047675	Rps8	20116	protein_coding	6731.473885	-0.256416246	0.115285524	2.224184252	0.02613605	0.090408616	0.256416246
ENSMUSG00000022814	Umps	22247	protein_coding	334.1984266	-0.256447647	0.102430506	2.503625689	0.012292801	0.049963897	0.256447647
ENSMUSG00000033350	Chst2	54371	protein_coding	2.288815945	0.256486385	1.819366736	-0.140975637	0.887889189	1	0.256486385
ENSMUSG00000039523	Cep104	230967	protein_coding	235.4705219	0.25672109	0.099087047	-2.590864269	0.009573524	0.041117602	0.25672109
ENSMUSG00000040234	Tm7sf3	67623	protein_coding	673.8590881	-0.256804676	0.067872483	3.783634585	0.000154555	0.001258539	0.256804676
ENSMUSG00000044551	9930012K11Rik	268759	protein_coding	23.43677691	-0.25680999	0.234863908	1.093441698	0.274199914	0.490619315	0.25680999
ENSMUSG00000097316	Gm10516	100038712	lncRNA	98.89545416	-0.25685421	0.131550383	1.952515871	0.050876989	0.151084803	0.25685421
ENSMUSG00000041939	Mvk	17855	protein_coding	369.0059602	0.256875626	0.122190612	-2.102253367	0.035531087	0.114758532	0.256875626

ensembl_gene_id	external_gene_name	entrezgene_id	gene_biotype	baseMean	log2FoldChange	lfcSE	stat	pvalue	padj	absLog2FC
ENSMUSG00000071655	Ubxn1	225896	protein_coding	888.1224897	-0.256979498	0.07230569	3.55407018	0.000379318	0.00280271	0.256979498
ENSMUSG00000034485	Uaca	72565	protein_coding	1233.980279	0.257203664	0.067735812	-3.797159238	0.000146364	0.001204311	0.257203664
ENSMUSG00000027878	Notch2	18129	protein_coding	194.3909633	0.257209039	0.095945254	-2.680789582	0.007344868	0.033167393	0.257209039
ENSMUSG00000027488	Snta1	20648	protein_coding	68.52561231	0.257212276	0.208881618	-1.231378226	0.218181437	0.42426293	0.257212276
ENSMUSG00000024120	Lrpprc	72416	protein_coding	996.1747949	-0.257255983	0.088662907	2.901506291	0.003713733	0.019034324	0.257255983
ENSMUSG00000037572	Wdhd1	218973	protein_coding	156.962421	0.257263831	0.134031033	-1.919434812	0.054929329	0.159785615	0.257263831
ENSMUSG00000038127	Ccdc50	67501	protein_coding	526.0414112	-0.257350533	0.068227537	3.771945258	0.00016198	0.001312529	0.257350533
ENSMUSG00000050777	Tmem37	170706	protein_coding	181.5634369	-0.257389828	0.195810775	1.314482454	0.188683899	0.385300595	0.257389828
ENSMUSG00000003037	Rab8a	17274	protein_coding	1489.743873	0.257437932	0.046007329	-5.59558525	2.20E-08	4.01E-07	0.257437932
ENSMUSG00000021575	Ahrr	11624	protein_coding	4.727294468	0.257478073	0.881576736	-0.292065412	0.770236611	0.884771487	0.257478073
ENSMUSG00000015314	Slamf6	30925	protein_coding	3.751085303	0.257506042	1.42367839	-0.180873745	0.856466677	1	0.257506042
ENSMUSG00000019892	Lrriq1	74978	protein_coding	1.927943457	-0.257543301	1.767529175	0.145708091	0.884151834	1	0.257543301
ENSMUSG00000051510	Mafg	17134	protein_coding	343.8226761	-0.257581208	0.109025414	2.362579496	0.018148249	0.067913025	0.257581208
ENSMUSG00000024887	Asah2	54447	protein_coding	288.8512722	0.257744841	0.145864039	-1.76702114	0.077224688	0.204931463	0.257744841
ENSMUSG00000071650	Ganab	14376	protein_coding	2872.642326	0.257787275	0.094062429	-2.740597674	0.006132755	0.028656498	0.257787275
ENSMUSG00000023336	Wfdc1	67866	protein_coding	1.182022123	0.257896853	1.41275044	-0.182549476	0.85515153	1	0.257896853
ENSMUSG00000086843	E030013I19Rik	338535	lncRNA	1.991781034	0.258010087	0.832555709	-0.309901288	0.756636023	1	0.258010087
ENSMUSG00000020309	Chac2	68044	protein_coding	110.5356076	0.258079314	0.115451263	-2.235396202	0.025391349	0.088425293	0.258079314
ENSMUSG00000003436	Dll3	13389	protein_coding	4.481916371	0.258346906	0.605313149	-0.426798768	0.669525904	0.822609935	0.258346906
ENSMUSG00000020268	Lyrm7	75530	protein_coding	61.6628164	0.258639057	0.168313714	-1.536648744	0.124379349	0.288970255	0.258639057
ENSMUSG00000047030	Spata2	263876	protein_coding	624.9711988	-0.258643938	0.061503977	4.205320519	2.61E-05	0.000261409	0.258643938
ENSMUSG00000023990	Tfeb	21425	protein_coding	235.6683713	-0.258696578	0.118629037	2.180718865	0.029204217	0.098824478	0.258696578
ENSMUSG00000039367	Sec24c	218811	protein_coding	1755.822346	0.258748702	0.051693129	-5.005475753	5.57E-07	8.14E-06	0.258748702
ENSMUSG00000061882	Ccdc62	208908	protein_coding	22.13431048	0.258752684	0.347504182	-0.74460308	0.456511706	0.671491432	0.258752684
ENSMUSG00000094410	Zbed6	667118	protein_coding	64.56103782	0.258761987	0.200813401	-1.288569315	0.197547857	0.397745099	0.258761987
ENSMUSG00000047603	Zfp235	56525	protein_coding	117.132934	-0.258769747	0.103096718	2.509970759	0.012074116	0.049378888	0.258769747
ENSMUSG00000021235	Coq6	217707	protein_coding	276.4942108	-0.258800349	0.077134527	3.355181657	0.000793129	0.005227463	0.258800349
ENSMUSG00000065430	Mir26a-2	723962	miRNA	0.861081691	0.258802322	1.135238467	-0.227971769	0.819668187	1	0.258802322
ENSMUSG00000017466	Timp2	21858	protein_coding	60.34412327	-0.258949718	0.202027208	1.281756659	0.199928022	0.400972961	0.258949718
ENSMUSG00000019558	Slc6a8	102857	protein_coding	3072.635941	-0.258954601	0.16785704	1.542709207	0.122901347	0.286211349	0.258954601
ENSMUSG00000092827	Mir3091	100526556	miRNA	2.286772159	-0.258984816	0.729480014	0.355026609	0.722569657	1	0.258984816
ENSMUSG00000027230	Creb3l1	26427	protein_coding	1394.048887	0.259004983	0.128947863	-2.008602362	0.044579321	0.136567098	0.259004983
ENSMUSG00000007892	Rplp1	56040	protein_coding	8039.370389	-0.259050386	0.096080903	2.696169358	0.007014199	0.031962608	0.259050386
ENSMUSG00000025777	Gdap1	14545	protein_coding	2.092761502	0.259092501	1.391288791	-0.186224818	0.852268463	1	0.259092501
ENSMUSG00000030037	Mrpl53	68499	protein_coding	0.731297528	-0.259346446	1.299964776	0.199502672	0.841869554	1	0.259346446
ENSMUSG00000043336	Filip1l	78749	protein_coding	368.3515593	-0.259406357	0.17658554	1.469012457	0.141829411	0.316830918	0.259406357
ENSMUSG00000020260	Pofut2	80294	protein_coding	460.8748154	0.25940684	0.072234985	-3.591152419	0.000329219	0.002472362	0.25940684
ENSMUSG00000059674	Cdh24	239096	protein_coding	30.03725878	0.25944757	0.201194552	-1.289535763	0.197211892	0.397165509	0.25944757
ENSMUSG00000032091	Tmprss4	214523	protein_coding	2320.444617	0.259533107	0.049101476	-5.285647811	1.25E-07	2.03E-06	0.259533107

ensembl_gene_id	external_gene_name	entrezgene_id	gene_biotype	baseMean	log2FoldChange	lfcSE	stat	pvalue	padj	absLog2FC
ENSMUSG00000030754	Copb1	70349	protein_coding	2438.932301	0.259569979	0.114013141	-2.276667207	0.022806106	0.081360993	0.259569979
ENSMUSG00000025241	Fyco1	17281	protein_coding	536.4247948	-0.259571049	0.10059322	2.580403031	0.009868507	0.042154299	0.259571049
ENSMUSG00000030208	Emp1	13730	protein_coding	7661.737637	-0.259580256	0.208905982	1.24256976	0.214026419	0.419019832	0.259580256
ENSMUSG00000029060	Mib2	76580	protein_coding	420.4859231	-0.259604599	0.104213355	2.491087621	0.012735271	0.051357818	0.259604599
ENSMUSG00000024827	Gldc	104174	protein_coding	5.387436725	0.25964698	0.653389937	-0.397384418	0.691084004	0.835534172	0.25964698
ENSMUSG00000052397	Ezr	22350	protein_coding	7506.532088	-0.259688605	0.103590056	2.50688739	0.012179951	0.049685066	0.259688605
ENSMUSG00000015355	Cd48	12506	protein_coding	5.659539645	-0.259689033	0.548007178	0.47387889	0.635586274	0.80133583	0.259689033
ENSMUSG00000039542	Ncam1	17967	protein_coding	4.960362913	0.259696345	1.196352399	-0.217073452	0.828151103	0.913297021	0.259696345
ENSMUSG00000075227	Znhit2	29805	protein_coding	194.5982238	0.259735632	0.131947076	-1.968483426	0.049012441	0.146868346	0.259735632
ENSMUSG00000032531	Amotl2	56332	protein_coding	89.01379768	-0.259789731	0.225418522	1.152477303	0.249124991	0.461264989	0.259789731
ENSMUSG00000075590	Nrbp2	223649	protein_coding	264.3590152	-0.259830427	0.099089597	2.622176637	0.008737014	0.038193919	0.259830427
ENSMUSG00000079559	Pou2af3	100502940	protein_coding	11.53395845	-0.259866375	0.373570535	0.695628672	0.486661406	0.695624496	0.259866375
ENSMUSG00000047910	Pcdhb16	93887	protein_coding	4.047278109	0.259885989	0.659539472	-0.3940416	0.693550326	1	0.259885989
ENSMUSG00000002741	Ykt6	56418	protein_coding	1279.16101	0.259913361	0.065640401	-3.95965529	7.51E-05	0.000670367	0.259913361
ENSMUSG00000020078	Vps26a	30930	protein_coding	626.502784	-0.259915418	0.057191473	4.544653324	5.50E-06	6.52E-05	0.259915418
ENSMUSG00000030882	Dnhd1	77505	protein_coding	13.24027291	0.259944861	0.538362611	-0.482843451	0.629206897	0.797446101	0.259944861
ENSMUSG00000032375	Aph1b	208117	protein_coding	182.8007955	0.260026006	0.108167879	-2.403911469	0.016220701	0.06221406	0.260026006
ENSMUSG00000022881	Rfc4	106344	protein_coding	117.3732331	0.260069865	0.166110899	-1.565639982	0.117432924	0.27747708	0.260069865
ENSMUSG00000003812	Dnase2a	13423	protein_coding	87.59658385	-0.260077381	0.166973851	1.557593474	0.119329647	0.280913726	0.260077381
ENSMUSG00000024135	Srbd1	78586	protein_coding	240.5399569	0.260098263	0.107562622	-2.418110089	0.015601356	0.06023867	0.260098263
ENSMUSG00000048832	Vps37c	107305	protein_coding	590.565986	0.260129223	0.104103309	-2.498760364	0.012462855	0.050530713	0.260129223
ENSMUSG00000026831	Pierce1	69327	protein_coding	0.536437832	0.260141527	1.289019517	-0.20181349	0.840062535	1	0.260141527
ENSMUSG00000021587	Pcsk1	18548	protein_coding	252.5357674	-0.260163536	0.171674065	1.515450431	0.129658374	0.297380166	0.260163536
ENSMUSG00000051329	Nup160	59015	protein_coding	366.4279562	0.260181646	0.106343377	-2.446618242	0.014420349	0.056508103	0.260181646
ENSMUSG00000054226	Tprkb	69786	protein_coding	157.9202734	-0.260202647	0.101312093	2.568327625	0.010219052	0.043322217	0.260202647
ENSMUSG00000045555	Mettl24	327747	protein_coding	15.40316303	0.260257892	0.285646743	-0.911118012	0.362233197	0.584685663	0.260257892
ENSMUSG00000098661	Mir7052	102465637	miRNA	0.75556368	0.260285738	1.291148032	-0.201592483	0.840235322	1	0.260285738
ENSMUSG00000056608	Chd9	109151	protein_coding	992.9852421	0.260324238	0.101715946	-2.559325741	0.010487542	0.044271797	0.260324238
ENSMUSG00000061536	Sec22c	215474	protein_coding	93.37995713	0.260350338	0.187203958	-1.390730945	0.16430703	0.35060942	0.260350338
ENSMUSG00000060636	Rpl35a	57808	protein_coding	2022.019922	-0.260361813	0.102250664	2.546309261	0.010886869	0.045492347	0.260361813
ENSMUSG00000027765	P2ry1	18441	protein_coding	287.0346013	-0.260364969	0.113912239	2.285662819	0.022273992	0.079911515	0.260364969
ENSMUSG00000084925	1810062O18Rik	75602	lncRNA	7.985126977	-0.2604238	0.414523323	0.628248847	0.529840935	0.727270652	0.2604238
ENSMUSG00000028703	Lrrc41	230654	protein_coding	418.144449	0.260442749	0.090093179	-2.890815396	0.003842438	0.019566455	0.260442749
ENSMUSG00000007030	Vwa7	27762	protein_coding	8.388714714	-0.260451487	0.554601526	0.469619132	0.638627153	0.80301103	0.260451487
ENSMUSG00000025069	Gsto2	68214	protein_coding	11.75948833	0.260488366	0.311421116	-0.836450558	0.40290148	0.624274258	0.260488366
ENSMUSG00000039345	Mettl22	239706	protein_coding	52.89631017	-0.26050645	0.185244574	1.406283834	0.159639859	0.343804463	0.26050645
ENSMUSG00000021250	Fos	14281	protein_coding	19884.91458	-0.260515864	0.162831376	1.599911953	0.109618117	0.264383465	0.260515864
ENSMUSG00000000305	Cdh4	12561	protein_coding	6.699020939	-0.26063473	0.859697978	0.30317011	0.761760227	0.880656876	0.26063473
ENSMUSG00000070287	Slc35g2	245020	protein_coding	68.03923766	-0.260637041	0.258181872	1.009509453	0.31273037	0.533791146	0.260637041

ensembl_gene_id	external_gene_name	entrezgene_id	gene_biotype	baseMean	log2FoldChange	lfcSE	stat	pvalue	padj	absLog2FC
ENSMUSG00000050212	Eva1b	230752	protein_coding	21.24225523	-0.260685031	0.2782236	0.936962324	0.348777938	0.570608171	0.260685031
ENSMUSG00000027809	Etfdh	66841	protein_coding	1270.387727	-0.260719489	0.069484232	3.752210854	0.000175282	0.001412003	0.260719489
ENSMUSG00000107388	Gm42788	105242579	lncRNA	0.768561446	-0.26072896	1.551990769	0.167996463	0.866586061	1	0.26072896
ENSMUSG00000023959	Clic5	224796	protein_coding	1981.182429	-0.260753968	0.17298097	1.507414185	0.131704525	0.30074233	0.260753968
ENSMUSG00000023942	Slc29a1	63959	protein_coding	192.4175495	0.260776268	0.163917486	-1.590899627	0.111632167	0.267599593	0.260776268
ENSMUSG00000022807	Osbpl11	106326	protein_coding	308.7592968	-0.260816368	0.115960091	2.249190779	0.024500361	0.08602548	0.260816368
ENSMUSG00000069806	Cacng7	81904	protein_coding	1.732613599	0.260864737	2.083864862	-0.125183135	0.900378568	1	0.260864737
ENSMUSG00000035992	Fnip1	216742	protein_coding	420.6990944	0.260892133	0.113926804	-2.289997814	0.022021443	0.079125787	0.260892133
ENSMUSG00000055370	Gm9968	791329	lncRNA	6.194056111	0.260895364	0.498033482	-0.523851052	0.600382142	0.776893175	0.260895364
ENSMUSG00000061859	Patj	12695	protein_coding	1448.16689	-0.260943477	0.066644199	3.915471716	9.02E-05	0.000787953	0.260943477
ENSMUSG00000065049	Gm24041	115488605	snRNA	1.122783556	-0.261046832	1.143269787	0.228333535	0.819386956	1	0.261046832
ENSMUSG00000027423	Snx5	69178	protein_coding	1241.541243	-0.261062845	0.090520746	2.884011181	0.003926448	0.019914424	0.261062845
ENSMUSG00000030007	Cct7	12468	protein_coding	1984.552697	0.261070971	0.088165404	-2.961149834	0.003064928	0.016170677	0.261070971
ENSMUSG00000087480	Gm15910	100504616	lncRNA	3.393473218	-0.261151365	0.823452958	0.317141813	0.751135999	1	0.261151365
ENSMUSG00000106617	Gm36266	108169076	lncRNA	30.17615316	-0.2611736	0.243927922	1.07069989	0.284304392	0.502676953	0.2611736
ENSMUSG00000040220	Gas8	104346	protein_coding	99.16321582	0.261236759	0.124916261	-2.09129506	0.036501622	0.117137153	0.261236759
ENSMUSG00000024130	Abca3	27410	protein_coding	944.5200439	-0.261263943	0.087824703	2.974834361	0.002931468	0.015558323	0.261263943
ENSMUSG00000057315	Arhgap24	231532	protein_coding	172.8827879	-0.261300781	0.174227958	1.499763776	0.133675604	0.303731277	0.261300781
ENSMUSG00000021963	Sap18	20220	protein_coding	447.7547265	-0.261338514	0.102525023	2.549021748	0.010802556	0.045254367	0.261338514
ENSMUSG00000014776	Nol3	78688	protein_coding	33.99063604	0.261357411	0.186135103	-1.404127468	0.160280896	0.34473544	0.261357411
ENSMUSG00000028070	Naxe	246703	protein_coding	592.0439133	-0.261362402	0.082039366	3.185816923	0.001443459	0.008606748	0.261362402
ENSMUSG00000037638	Zbtb42	382639	protein_coding	283.202064	-0.261364675	0.090542266	2.88665932	0.003893556	0.019774484	0.261364675
ENSMUSG00000000295	Hddc2	69692	protein_coding	137.1426641	0.261376926	0.124756153	-2.09510248	0.036161884	0.116182363	0.261376926
ENSMUSG00000054630	Ugt2b5	22238	protein_coding	504.7003075	0.261403209	0.147195617	-1.775889894	0.075751067	0.201799181	0.261403209
ENSMUSG00000041560	Nop53	68077	protein_coding	1627.534098	-0.261471086	0.078768226	3.319499494	0.00090179	0.005835484	0.261471086
ENSMUSG00000045763	Basp1	70350	protein_coding	3.034096066	-0.261471636	0.800154926	0.326776262	0.743837115	1	0.261471636
ENSMUSG00000018678	Sp2	78912	protein_coding	105.1233526	0.261572026	0.219836655	-1.18984719	0.234106458	0.443634979	0.261572026
ENSMUSG00000026566	Mpzl1	68481	protein_coding	243.5235323	0.26158711	0.116386556	-2.247571523	0.024603525	0.08629118	0.26158711
ENSMUSG00000029063	Nadk	192185	protein_coding	2370.977621	-0.261607421	0.051898485	5.040752602	4.64E-07	6.90E-06	0.261607421
ENSMUSG00000019189	Rnf145	74315	protein_coding	999.0539551	0.261634785	0.111395023	-2.348711612	0.018838491	0.070067307	0.261634785
ENSMUSG00000089712	Gm15889	115486954	lncRNA	9.739251029	-0.26169447	0.538155745	0.486280175	0.626768533	0.795699228	0.26169447
ENSMUSG00000055555	Ct55	75013	protein_coding	0.776276256	0.261695311	1.551387458	-0.168684689	0.866044663	1	0.261695311
ENSMUSG00000027466	Rbck1	24105	protein_coding	1055.365863	0.261733392	0.053042994	-4.934363142	8.04E-07	1.13E-05	0.261733392
ENSMUSG00000041000	Trim62	67525	protein_coding	70.56497415	-0.261775564	0.203615491	1.285636781	0.198569854	0.398975857	0.261775564
ENSMUSG00000037031	Tspan15	70423	protein_coding	1429.986491	-0.261794868	0.087214207	3.001745657	0.002684364	0.014482058	0.261794868
ENSMUSG00000029614	Rpl6	19988	protein_coding	3733.03617	-0.261878671	0.122023846	2.146127006	0.031862847	0.105765439	0.261878671
ENSMUSG00000028919	Arhgef19	213649	protein_coding	317.6013869	-0.261987632	0.148373318	1.76573279	0.077440689	0.205372657	0.261987632
ENSMUSG00000025245	Lztfl1	93730	protein_coding	228.6916818	0.262079705	0.092357158	-2.837676146	0.004544326	0.022468565	0.262079705
ENSMUSG00000062980	Cped1	214642	protein_coding	10.07539681	0.262126885	0.41049658	-0.638560461	0.52310891	0.722040793	0.262126885

ensembl_gene_id	external_gene_name	entrezgene_id	gene_biotype	baseMean	log2FoldChange	lfcSE	stat	pvalue	padj	absLog2FC
ENSMUSG00000006546	Cryba2	12958	protein_coding	3.815871008	0.262361024	0.691512268	-0.379401836	0.704389487	1	0.262361024
ENSMUSG00000074415	Mir100hg	73144	lncRNA	7.137061925	0.262373999	1.443822281	-0.18172181	0.855801049	0.929905153	0.262373999
ENSMUSG00000029661	Col1a2	12843	protein_coding	10.94207406	0.2624602	0.622488327	-0.421630718	0.673294581	0.824897384	0.2624602
ENSMUSG00000032086	Bace1	23821	protein_coding	253.2294733	-0.262601166	0.104804016	2.505640299	0.01222299	0.049790441	0.262601166
ENSMUSG00000026024	Als2	74018	protein_coding	197.8154299	0.262620974	0.145845134	-1.800683825	0.071752729	0.194536949	0.262620974
ENSMUSG00000032212	Sltm	66660	protein_coding	807.7801081	0.262658188	0.060259657	-4.358773354	1.31E-05	0.000142007	0.262658188
ENSMUSG00000045394	Epcam	17075	protein_coding	16955.67628	-0.262666805	0.069394517	3.78512331	0.000153632	0.001253501	0.262666805
ENSMUSG00000025395	Prim1	19075	protein_coding	133.539156	0.262696864	0.213492199	-1.230475236	0.218519195	0.424602824	0.262696864
ENSMUSG00000023572	Ccndbp1	17151	protein_coding	317.0115465	-0.262711944	0.092809665	2.830652874	0.004645311	0.022878813	0.262711944
ENSMUSG00000063810	Alms1	236266	protein_coding	176.1286211	-0.262768658	0.106026827	2.478322365	0.013200182	0.052845529	0.262768658
ENSMUSG00000071893	Vmn1r4	171194	protein_coding	3.164694368	-0.262808786	0.697330773	0.376878228	0.706264102	1	0.262808786
ENSMUSG00000114213	A430090L17Rik	319803	lncRNA	4.695104736	-0.262835114	0.523360255	0.502206868	0.615522012	0.787780401	0.262835114
ENSMUSG00000021189	Atxn3	110616	protein_coding	465.270507	-0.262869782	0.136700194	1.922965677	0.05448436	0.159023459	0.262869782
ENSMUSG00000024268	Celf4	108013	protein_coding	14.81588526	-0.262903676	0.567201105	0.463510514	0.64299848	0.805274537	0.262903676
ENSMUSG00000028040	Efna4	13639	protein_coding	90.08655391	0.262945115	0.142456005	-1.845798747	0.064921433	0.180947387	0.262945115
ENSMUSG00000029186	Pi4k2b	67073	protein_coding	1724.302847	0.263014818	0.065096458	-4.040385973	5.34E-05	0.000494524	0.263014818
ENSMUSG00000020485	Supt4a	20922	protein_coding	222.5152332	-0.263100487	0.08515012	3.08984281	0.002002625	0.011349122	0.263100487
ENSMUSG00000022151	Ttc33	67515	protein_coding	209.5925292	-0.263103331	0.108277999	2.429887259	0.01510352	0.058719535	0.263103331
ENSMUSG00000041607	Mbp	17196	protein_coding	862.2582654	-0.263165105	0.068299513	3.853103672	0.00011663	0.000987961	0.263165105
ENSMUSG00000031787	Katnb1	74187	protein_coding	253.710702	0.263184848	0.085940353	-3.062412943	0.002195603	0.01219993	0.263184848
ENSMUSG00000047407	Tgif1	21815	protein_coding	435.2934613	-0.263247305	0.100619414	2.61626753	0.008889688	0.038706422	0.263247305
ENSMUSG00000025002	Cyp2c55	72082	protein_coding	18703.26013	-0.263253524	0.171990115	1.530631708	0.12586044	0.291600149	0.263253524
ENSMUSG00000086003	B230206L02Rik	100039440	lncRNA	8.601336583	-0.263268727	0.441811352	0.595884932	0.5512521	0.742102974	0.263268727
ENSMUSG00000004317	Clcn5	12728	protein_coding	449.7042287	-0.263382488	0.102960636	2.55808917	0.01052491	0.044395495	0.263382488
ENSMUSG00000035934	Pknox2	208076	protein_coding	9.259520741	-0.263383919	0.959712111	0.274440549	0.783746085	0.891560436	0.263383919
ENSMUSG00000026963	Tmem210	78217	protein_coding	2.621367255	0.263396723	1.055450873	-0.249558487	0.802928805	1	0.263396723
ENSMUSG00000047246	H2bc6	319179	protein_coding	712.2944283	0.263444962	0.183028582	-1.439365145	0.150047094	0.329724247	0.263444962
ENSMUSG00000058793	Cds2	110911	protein_coding	1176.890767	-0.263544963	0.099277292	2.654634886	0.007939429	0.035388872	0.263544963
ENSMUSG00000011382	Dhdh	71755	protein_coding	17.14731111	-0.263562419	0.340706367	0.773576442	0.439181308	0.657120605	0.263562419
ENSMUSG00000025417	Pip4k2c	117150	protein_coding	962.4817936	-0.263571795	0.062565583	4.21272816	2.52E-05	0.00025468	0.263571795
ENSMUSG00000051223	Bzw1	66882	protein_coding	3147.795137	0.263647282	0.075787199	-3.478783814	0.000503695	0.003569964	0.263647282
ENSMUSG00000031119	Gpc4	14735	protein_coding	283.4190775	-0.263666824	0.140773504	1.872986159	0.061070296	0.173046478	0.263666824
ENSMUSG00000050379	Septin6	56526	protein_coding	74.54523466	0.263671249	0.14582667	-1.808114034	0.070588754	0.192129489	0.263671249
ENSMUSG00000097101	1810034E14Rik	66503	lncRNA	75.91789605	-0.263673776	0.174695273	1.509335496	0.131213068	0.299827398	0.263673776
ENSMUSG00000063953	Amd2	100041585	protein_coding	1.172139577	-0.263685449	0.970198381	0.271785084	0.785787275	1	0.263685449
ENSMUSG00000032306	Mpi	110119	protein_coding	1075.836843	0.263807306	0.071157688	-3.707361962	0.00020943	0.00164613	0.263807306
ENSMUSG00000087231	E230016M11Rik	320172	lncRNA	13.21269008	-0.263807569	0.375281231	0.702959667	0.482080885	0.69281035	0.263807569
ENSMUSG00000036810	Cnep1r1	382030	protein_coding	200.2360959	0.263808343	0.102832259	-2.565423966	0.01030498	0.043612621	0.263808343
ENSMUSG00000042423	Fbrs	14123	protein_coding	401.3916878	0.263815366	0.196583827	-1.341999339	0.17959621	0.372922396	0.263815366

ensembl_gene_id	external_gene_name	entrezgene_id	gene_biotype	baseMean	log2FoldChange	lfcSE	stat	pvalue	padj	absLog2FC
ENSMUSG00000006418	Rnf114	81018	protein_coding	1139.45537	0.263866543	0.059183197	-4.458470604	8.25E-06	9.45E-05	0.263866543
ENSMUSG00000040350	Trim7	94089	protein_coding	36.19661002	-0.264021182	0.26615097	0.991997819	0.32119859	0.542303804	0.264021182
ENSMUSG00000030086	Chchd6	66098	protein_coding	45.06712271	0.264055874	0.187387578	-1.409142893	0.15879292	0.342785135	0.264055874
ENSMUSG00000044792	Isca1	69046	protein_coding	521.4084149	-0.264072423	0.112122569	2.355212022	0.01851214	0.069055676	0.264072423
ENSMUSG00000037259	Dzank1	241688	protein_coding	3.197509579	-0.264109551	1.404283351	0.188074259	0.850818439	1	0.264109551
ENSMUSG00000071369	Map3k5	26408	protein_coding	695.4748417	0.264135492	0.057190462	-4.618523511	3.86E-06	4.75E-05	0.264135492
ENSMUSG00000059070	Rpl18	19899	protein_coding	4929.390872	-0.26416624	0.102589588	2.574980989	0.01002456	0.042679767	0.26416624
ENSMUSG00000096914	Galntl6	270049	protein_coding	91.41345196	0.26417453	0.116230621	-2.272847959	0.023035344	0.081931007	0.26417453
ENSMUSG00000028932	Psmc2	19181	protein_coding	791.196072	0.264309364	0.066273585	-3.988155534	6.66E-05	0.000602545	0.264309364
ENSMUSG00000087413	Gm11266	102635783	lncRNA	1.689110431	0.264322206	0.983360061	-0.268794937	0.78808749	1	0.264322206
ENSMUSG00000022994	Adcy6	11512	protein_coding	3021.129186	-0.26436138	0.059358279	4.453656444	8.44E-06	9.64E-05	0.26436138
ENSMUSG00000026309	Ilkap	67444	protein_coding	422.4847328	-0.264480017	0.066637821	3.968917564	7.22E-05	0.000647282	0.264480017
ENSMUSG00000049624	Slc17a5	235504	protein_coding	278.5013415	-0.264490006	0.096193902	2.749550642	0.005967704	0.028073652	0.264490006
ENSMUSG00000042275	Pelo	105083	protein_coding	47.63686693	0.264584206	0.177557333	-1.490133929	0.136189025	0.307874819	0.264584206
ENSMUSG00000035890	Rnf126	70294	protein_coding	363.4571646	0.26474536	0.091945776	-2.879364031	0.003984781	0.020123752	0.26474536
ENSMUSG00000079465	Col4a3	12828	protein_coding	7.222849078	0.264750771	0.742403423	-0.35661308	0.721381484	0.855640813	0.264750771
ENSMUSG00000026771	Spopl	76857	protein_coding	237.6256255	-0.264894251	0.090653885	2.922039697	0.003477472	0.01801348	0.264894251
ENSMUSG00000023088	Abcc1	17250	protein_coding	517.2947491	-0.264899968	0.09167938	2.8894171	0.003859568	0.019641571	0.264899968
ENSMUSG00000014361	Mertk	17289	protein_coding	554.3284877	-0.26490581	0.127891722	2.071328825	0.038328077	0.12190868	0.26490581
ENSMUSG00000113679	Gm10432	100038713	lncRNA	4.514224801	0.264907021	0.724572782	-0.365604433	0.714660266	0.851938182	0.264907021
ENSMUSG00000025762	Larp1b	214048	protein_coding	774.5338933	0.264943341	0.064123139	-4.131789971	3.60E-05	0.000350484	0.264943341
ENSMUSG00000029445	Hpd	15445	protein_coding	582.5544579	-0.264970043	0.271254802	0.976830791	0.328652929	0.550946551	0.264970043
ENSMUSG00000074358	Ccdc61	232933	protein_coding	232.0258526	0.264992986	0.07759522	-3.415068429	0.00063766	0.004340608	0.264992986
ENSMUSG00000058626	Capn11	268958	protein_coding	7.396333509	-0.265055527	1.376404334	0.192570977	0.847294977	0.924739227	0.265055527
ENSMUSG00000026547	Tagln2	21346	protein_coding	2280.863053	-0.265071697	0.066461407	3.988355192	6.65E-05	0.000602545	0.265071697
ENSMUSG00000031357	Syap1	67043	protein_coding	823.9303119	-0.265077278	0.069559782	3.810783631	0.000138527	0.001148988	0.265077278
ENSMUSG00000018427	Ypel2	77864	protein_coding	262.1144766	-0.265093265	0.179152625	1.479706286	0.138951647	0.312060508	0.265093265
ENSMUSG00000032867	Fbxw8	231672	protein_coding	681.6777526	0.265118971	0.067943424	-3.902054937	9.54E-05	0.000825955	0.265118971
ENSMUSG00000032344	Cgas	214763	protein_coding	47.42962671	0.265137297	0.217910429	-1.216726058	0.223708461	0.43083733	0.265137297
ENSMUSG00000004668	Abca13	268379	protein_coding	15.13685905	-0.265192401	0.552728005	0.479788248	0.63137797	0.799093792	0.265192401
ENSMUSG00000063760	Rnf217	268291	protein_coding	137.0850299	0.26525939	0.111842592	-2.371720704	0.017705469	0.066679159	0.26525939
ENSMUSG00000026662	Sephs1	109079	protein_coding	428.7277544	0.265279174	0.093782691	-2.828658165	0.00467436	0.022973921	0.265279174
ENSMUSG00000031849	Comp	12845	protein_coding	5.605842113	0.265283484	0.847434002	-0.313043238	0.754247813	0.876856731	0.265283484
ENSMUSG00000087380	2210408F21Rik	73652	lncRNA	3.195610584	-0.265341472	0.964286772	0.275168632	0.783186685	1	0.265341472
ENSMUSG00000068962	Zfp114	232966	protein_coding	2.436509382	-0.265394098	0.774630555	0.34260732	0.731893894	1	0.265394098
ENSMUSG00000050222	Il17d	239114	protein_coding	23.89819608	-0.265415575	0.268517268	0.988448812	0.322932892	0.544366934	0.265415575
ENSMUSG00000074272	Ceacam1	26365	protein_coding	22651.19201	-0.265434745	0.124723713	2.128181877	0.033322007	0.109351285	0.265434745
ENSMUSG00000020709	Adap2	216991	protein_coding	33.03879242	-0.265454152	0.252924238	1.049540185	0.29392957	0.513157091	0.265454152
ENSMUSG00000025165	Sectm1a	209588	protein_coding	51.33279591	-0.265479796	0.362193041	0.73297873	0.463571402	0.677051219	0.265479796

ensembl_gene_id	external_gene_name	entrezgene_id	gene_biotype	baseMean	log2FoldChange	lfcSE	stat	pvalue	padj	absLog2FC
ENSMUSG00000024772	Ehd1	13660	protein_coding	835.3329967	0.265508081	0.082512819	-3.217779784	0.00129187	0.007872301	0.265508081
ENSMUSG00000025555	Farp1	223254	protein_coding	604.8358411	0.265597705	0.078403953	-3.387555012	0.000705186	0.004714752	0.265597705
ENSMUSG00000079659	Tmem243	652925	protein_coding	193.5276001	-0.265605478	0.123466003	2.15124384	0.031456958	0.104670605	0.265605478
ENSMUSG00000091243	Vgll3	73569	protein_coding	2.785309681	0.265686819	1.260564125	-0.21076819	0.833068162	1	0.265686819
ENSMUSG00000097619	4833422M21Rik	75768	lncRNA	2.191453282	-0.265714533	1.66437426	0.159648307	0.873158123	1	0.265714533
ENSMUSG00000042816	Gpr151	240239	protein_coding	2.4240118	0.26575842	0.691737181	-0.384189873	0.700837723	1	0.26575842
ENSMUSG00000003824	Syce2	71846	protein_coding	37.6998944	-0.265847962	0.192236568	1.38292087	0.16668913	0.354364611	0.265847962
ENSMUSG00000020405	Fabp6	16204	protein_coding	45.72191873	0.265855022	0.332531703	-0.799487748	0.424007648	0.64392701	0.265855022
ENSMUSG00000029376	Mthfd2l	665563	protein_coding	97.0674133	0.265857032	0.112855681	-2.355725724	0.018486562	0.068975843	0.265857032
ENSMUSG00000031666	Rbl2	19651	protein_coding	432.6219393	-0.265960896	0.078218271	3.400240049	0.000673267	0.004543659	0.265960896
ENSMUSG00000045348	Nyap1	243300	protein_coding	102.4867385	-0.265979928	0.346183835	0.768319896	0.442297157	0.660108021	0.265979928
ENSMUSG00000047841	Fndc11	332713	protein_coding	1.608105901	0.266048492	1.136492655	-0.234096094	0.814910378	1	0.266048492
ENSMUSG00000029420	Rimbp2	231760	protein_coding	45.48543567	0.266077247	0.288027305	-0.923791745	0.355594754	0.578423233	0.266077247
ENSMUSG00000023908	Pkmyt1	268930	protein_coding	151.5662956	0.266111782	0.133169279	-1.998297079	0.045684462	0.139280674	0.266111782
ENSMUSG00000063320	1190007I07Rik	544717	protein_coding	77.31736265	-0.266304661	0.129598144	2.05484934	0.039893559	0.125701628	0.266304661
ENSMUSG00000090700	Cyp4f40	631304	protein_coding	1028.11333	0.266322418	0.081073543	-3.284948551	0.00102001	0.006461237	0.266322418
ENSMUSG00000020297	Nsg2	18197	protein_coding	6.354313957	0.266338809	0.565764245	-0.470759352	0.637812595	0.802698942	0.266338809
ENSMUSG00000029860	Zyx	22793	protein_coding	523.582034	-0.266370601	0.122243214	2.179021577	0.029330065	0.099059146	0.266370601
ENSMUSG00000079260	Tmppe	100504715	protein_coding	123.5686371	-0.266479309	0.112810042	2.362194915	0.018167088	0.067952726	0.266479309
ENSMUSG00000058435	Btnl4	632126	protein_coding	926.0097216	-0.266506715	0.113691532	2.344121071	0.019071979	0.070728647	0.266506715
ENSMUSG00000034723	Tmx4	52837	protein_coding	173.0669407	0.266527235	0.183051826	-1.456020628	0.145386917	0.322533618	0.266527235
ENSMUSG00000078552	Dcdc2b	100504491	protein_coding	10.43441428	-0.266568397	0.40282096	0.66175404	0.508128865	0.711906345	0.266568397
ENSMUSG00000022892	App	11820	protein_coding	3986.685218	-0.266707999	0.036856101	7.236468105	4.61E-13	1.44E-11	0.266707999
ENSMUSG00000024921	Smarca2	67155	protein_coding	449.4488855	-0.266761734	0.073003949	3.654072675	0.000258113	0.001988097	0.266761734
ENSMUSG00000005986	Ankrd13d	68423	protein_coding	8.670700268	-0.266779481	0.470576833	0.566920132	0.570768446	0.756249943	0.266779481
ENSMUSG00000028341	Nr4a3	18124	protein_coding	10.41121024	-0.266866655	0.597924767	0.446321461	0.655365056	0.813003196	0.266866655
ENSMUSG00000002985	Apoe	11816	protein_coding	35.46939338	0.266898909	0.338396645	-0.788716179	0.430277909	0.648871201	0.266898909
ENSMUSG00000014856	Tmem208	66320	protein_coding	425.2527982	-0.266920151	0.076868313	3.472434105	0.000515762	0.00364298	0.266920151
ENSMUSG00000099632	2900093K20Rik	108168734	lncRNA	11.31669735	0.266920753	0.328244774	-0.813175942	0.416117191	0.636395319	0.266920753
ENSMUSG00000102543	Pcdhgc5	93708	protein_coding	16.83475698	0.266933356	0.318924583	-0.836979558	0.402604059	0.624030976	0.266933356
ENSMUSG00000074785	Plxnc1	54712	protein_coding	6.289313702	-0.26706617	0.548224687	0.487147289	0.626153956	0.795381772	0.26706617
ENSMUSG00000047021	Cfap65	241116	protein_coding	8.537294073	0.267195197	0.726960902	-0.367550987	0.713208067	0.850882677	0.267195197
ENSMUSG00000025237	Parp6	67287	protein_coding	291.7437838	-0.267210669	0.092240714	2.896884221	0.003768888	0.019227501	0.267210669
ENSMUSG00000087006	Gm13889	620695	protein_coding	2.280246643	-0.267261024	1.069891093	0.249802084	0.802740408	1	0.267261024
ENSMUSG00000104346	Pcdhga3	93711	protein_coding	3.043964228	0.267338922	0.628034542	-0.425675507	0.670344312	1	0.267338922
ENSMUSG00000025780	Itih5	209378	protein_coding	2887.531755	-0.267475664	0.190001974	1.407752031	0.159204509	0.343330529	0.267475664
ENSMUSG00000089417	Gm22009	115489854	scaRNA	1584.437012	-0.267648297	0.092001192	2.909182933	0.003623748	0.018648354	0.267648297
ENSMUSG00000037270	Bltp1	229227	protein_coding	1384.478442	0.267672642	0.134688183	-1.987350609	0.046883557	0.14195729	0.267672642
ENSMUSG00000050605	Zfp61	22719	protein_coding	52.09649368	0.267673627	0.160755898	-1.66509366	0.095894071	0.239648108	0.267673627

ensembl_gene_id	external_gene_name	entrezgene_id	gene_biotype	baseMean	log2FoldChange	lfcSE	stat	pvalue	padj	absLog2FC
ENSMUSG00000015013	Trappc2l	59005	protein_coding	237.5010327	-0.267710744	0.088858216	3.012785509	0.002588618	0.014029681	0.267710744
ENSMUSG00000087543	Gm16576	100504191	lncRNA	21.12522806	-0.267773004	0.336662679	0.795374779	0.426395526	0.646068054	0.267773004
ENSMUSG00000041872	Il17f	257630	protein_coding	0.950061646	0.267850176	1.448789142	-0.184878647	0.85332422	1	0.267850176
ENSMUSG00000006057	Atp5g1	11951	protein_coding	3021.225711	0.267901041	0.10654568	-2.514424255	0.011922691	0.048844287	0.267901041
ENSMUSG00000033576	Apol6	71939	protein_coding	146.5409936	-0.267918649	0.218177923	1.227982398	0.219453576	0.425708487	0.267918649
ENSMUSG00000031530	Dusp4	319520	protein_coding	80.1937705	-0.267940005	0.177957933	1.505636753	0.132160448	0.3013667	0.267940005
ENSMUSG00000057572	Zbtb8os	67106	protein_coding	165.8178384	-0.267971371	0.10551565	2.539636262	0.011096781	0.046264135	0.267971371
ENSMUSG00000071660	Ttc9c	70387	protein_coding	485.0010832	0.26802445	0.082688907	-3.24135919	0.001189612	0.007349514	0.26802445
ENSMUSG00000031753	Cog4	102339	protein_coding	800.3621095	-0.268034234	0.060732061	4.413389428	1.02E-05	0.000113627	0.268034234
ENSMUSG00000062175	Tgif2	228839	protein_coding	4.888765697	-0.268092956	0.510789328	0.524860135	0.599680424	0.776544903	0.268092956
ENSMUSG00000046364	Rpl27a	26451	protein_coding	2556.229272	-0.268105641	0.114526258	2.340997129	0.019232315	0.071195349	0.268105641
ENSMUSG00000040557	Mettl27	79565	protein_coding	132.4037764	-0.268131828	0.110654717	2.423139611	0.015387012	0.059578222	0.268131828
ENSMUSG00000061518	Cox5b	12859	protein_coding	4041.931803	-0.268136749	0.096679269	2.773466856	0.005546249	0.026494289	0.268136749
ENSMUSG00000007653	Gabrb2	14401	protein_coding	2.216204121	-0.268155215	1.957931288	0.13695844	0.891063646	1	0.268155215
ENSMUSG00000041147	Brca2	12190	protein_coding	81.26192107	0.26816452	0.161079257	-1.664798592	0.095952945	0.239722683	0.26816452
ENSMUSG00000028577	Plaa	18786	protein_coding	735.5395576	0.268179658	0.076327031	-3.513560735	0.000442143	0.003202436	0.268179658
ENSMUSG00000041598	Cdc42ep4	56699	protein_coding	942.0719346	-0.26821291	0.054891147	4.886268981	1.03E-06	1.41E-05	0.26821291
ENSMUSG00000005949	Ctns	83429	protein_coding	185.7954293	-0.268215736	0.127557184	2.102709764	0.035491148	0.114696833	0.268215736
ENSMUSG00000027510	Rbm38	56190	protein_coding	106.1387205	-0.268231023	0.216069128	1.241412992	0.214453222	0.419407924	0.268231023
ENSMUSG00000047730	Fcgbp	215384	protein_coding	68572.87662	-0.268258989	0.28466448	0.942369026	0.346003739	0.567700522	0.268258989
ENSMUSG00000034173	Chchd2l	71970	protein_coding	7.410225192	-0.268472016	0.440341731	0.609690149	0.542067081	0.735735661	0.268472016
ENSMUSG00000018661	Cog1	16834	protein_coding	867.935164	0.268568629	0.056873216	-4.722233869	2.33E-06	2.99E-05	0.268568629
ENSMUSG00000071281	Zfp65	235907	protein_coding	158.954708	-0.268593678	0.153062248	1.754800293	0.079293473	0.208654479	0.268593678
ENSMUSG00000039826	Trub2	227682	protein_coding	135.8706827	-0.268647349	0.103199351	2.603188351	0.00923612	0.039959466	0.268647349
ENSMUSG00000039552	Rsph4a	212892	protein_coding	1.612324807	0.26865254	1.428357573	-0.188084934	0.850810071	1	0.26865254
ENSMUSG00000027546	Atp9a	11981	protein_coding	3528.267587	-0.268777541	0.094738126	2.837057818	0.004553137	0.022498653	0.268777541
ENSMUSG00000020092	Pald1	27355	protein_coding	19.59007254	0.26885565	0.292574456	-0.918930698	0.358131822	0.580790426	0.26885565
ENSMUSG00000040528	Milr1	380732	protein_coding	5.94045404	0.268882492	0.529012942	-0.508272049	0.611262569	0.784646895	0.268882492
ENSMUSG00000049659	Aftph	216549	protein_coding	1921.60378	0.268937693	0.074487594	-3.610503128	0.000305604	0.002315017	0.268937693
ENSMUSG00000007812	Zfp655	72611	protein_coding	413.3374332	-0.269107521	0.092439513	2.911174157	0.003600733	0.018535691	0.269107521
ENSMUSG00000045503	Sys1	66460	protein_coding	290.9878546	0.269108395	0.068191596	-3.946357158	7.93E-05	0.000704123	0.269108395
ENSMUSG00000037887	Dusp8	18218	protein_coding	253.5845112	0.269138306	0.201893988	-1.333067461	0.182509667	0.37700811	0.269138306
ENSMUSG00000026939	Tmem141	51875	protein_coding	83.16784697	-0.269155431	0.109300943	2.462517002	0.013796563	0.054614131	0.269155431
ENSMUSG00000032773	Chrm1	12669	protein_coding	84.04993598	-0.269243859	0.233399798	1.153573661	0.248675002	0.460883095	0.269243859
ENSMUSG00000017721	Pigt	78928	protein_coding	1145.179823	0.269246706	0.076270885	-3.530137427	0.000415344	0.003030927	0.269246706
ENSMUSG00000028469	Npr2	230103	protein_coding	3.451989686	-0.269251144	1.26395485	0.213022755	0.831309214	1	0.269251144
ENSMUSG00000023106	Denr	68184	protein_coding	384.1782947	0.269257583	0.077245904	-3.485719893	0.000490815	0.003492164	0.269257583
ENSMUSG00000038497	Tmco3	234076	protein_coding	986.1891214	-0.269259672	0.103691858	2.596729155	0.00941161	0.040601707	0.269259672
ENSMUSG00000028760	Eif4g3	230861	protein_coding	795.4265474	0.269472181	0.065082787	-4.140452407	3.47E-05	0.000338705	0.269472181

Supplementary Table 1

ensembl_gene_id	external_gene_name	entrezgene_id	gene_biotype	baseMean	log2FoldChange	lfcSE	stat	pvalue	padj	absLog2FC
ENSMUSG00000039518	Cdsn	386463	protein_coding	10.40607751	0.269495136	0.366524788	-0.735271242	0.462174319	0.675880563	0.269495136
ENSMUSG00000043448	Gjc2	118454	protein_coding	2.334076099	0.269525444	0.794067258	-0.339423948	0.73429038	1	0.269525444
ENSMUSG00000026640	Plxna2	18845	protein_coding	733.726721	-0.269610799	0.104878568	2.570694878	0.010149471	0.043086943	0.269610799
ENSMUSG00000001134	Uxt	22294	protein_coding	116.0806418	-0.269723869	0.123854135	2.177754246	0.029424338	0.099328192	0.269723869
ENSMUSG00000069135	Cep43	75296	protein_coding	183.3879932	0.26974494	0.110837894	-2.433688794	0.014945839	0.058238694	0.26974494
ENSMUSG00000035898	Uba6	231380	protein_coding	345.4347032	0.2697567	0.099776871	-2.703599512	0.006859291	0.031386624	0.2697567
ENSMUSG00000078630	Tomt	791260	protein_coding	6.135913503	-0.269841857	0.524067688	0.514898863	0.606623695	0.781418386	0.269841857
ENSMUSG00000089789	Rdh1	107605	protein_coding	31.85633339	0.269895299	0.212363719	-1.270910584	0.203760461	0.405947672	0.269895299
ENSMUSG00000062585	Cnr2	12802	protein_coding	6.75457184	0.269955169	0.532731652	-0.50673762	0.61233893	0.785464687	0.269955169
ENSMUSG00000035551	Igfbpl1	75426	protein_coding	1.739899678	0.270057249	1.12608331	-0.239819955	0.810469836	1	0.270057249
ENSMUSG00000020275	Rel	19696	protein_coding	74.92110834	-0.270230229	0.203210816	1.329802394	0.183583387	0.378441649	0.270230229
ENSMUSG00000045594	Glb1	12091	protein_coding	988.9602574	-0.27024638	0.102450057	2.637835334	0.008343709	0.036802352	0.27024638
ENSMUSG00000040296	Rigi	230073	protein_coding	246.5890796	-0.270275284	0.170368961	1.586411524	0.112645988	0.269245209	0.270275284
ENSMUSG00000019261	Map1s	270058	protein_coding	135.2637085	0.270300741	0.111379637	-2.426841653	0.015230902	0.059126261	0.270300741
ENSMUSG00000025227	Mfsd13a	75146	protein_coding	148.6336079	-0.27037843	0.10701931	2.526445281	0.011522334	0.047677228	0.27037843
ENSMUSG00000030084	Plxna1	18844	protein_coding	498.5922761	0.270395221	0.08820088	-3.065674881	0.002171793	0.012108371	0.270395221
ENSMUSG00000032193	Ldlr	16835	protein_coding	3066.898394	-0.270399951	0.144777728	1.86769025	0.061805259	0.174291673	0.270399951
ENSMUSG00000063882	Uqcrh	66576	protein_coding	3833.611837	-0.270433893	0.075799904	3.567733966	0.000360082	0.002678555	0.270433893
ENSMUSG00000030451	Herc2	15204	protein_coding	2191.557104	0.270520376	0.067722937	-3.994516333	6.48E-05	0.000589831	0.270520376
ENSMUSG00000021215	Net1	56349	protein_coding	2401.676144	-0.270537662	0.071263767	3.796286291	0.00014688	0.001207955	0.270537662
ENSMUSG00000015451	C4a	625018	lncRNA	8.486682681	-0.270547007	0.67528489	0.400641286	0.688684244	0.83386713	0.270547007
ENSMUSG00000015289	Lage3	66192	protein_coding	134.2868906	-0.270589834	0.113566016	2.382665555	0.017187802	0.06516055	0.270589834
ENSMUSG00000047759	Hs3st3a1	15478	protein_coding	12.77610813	0.270611999	0.319802337	-0.846185183	0.397449446	0.619371535	0.270611999
ENSMUSG00000032128	Robo3	19649	protein_coding	4.115989278	0.270623254	1.916879387	-0.141179072	0.887728478	1	0.270623254
ENSMUSG00000045251	Zfp688	69234	protein_coding	85.65081839	-0.270692285	0.108118509	2.503662761	0.012291513	0.049963897	0.270692285
ENSMUSG00000040681	Hmgn1	15312	protein_coding	707.7778371	0.270745173	0.123830694	-2.186414082	0.028785324	0.097670196	0.270745173
ENSMUSG00000026253	Chrng	11449	protein_coding	2.318033037	-0.270894699	1.632050941	0.165984218	0.86816937	1	0.270894699
ENSMUSG00000022039	Adam2	11495	protein_coding	1.57713395	-0.270985641	1.836832216	0.147528793	0.882714654	1	0.270985641
ENSMUSG00000023938	Aars2	224805	protein_coding	271.9829597	-0.27101562	0.085112212	3.184215424	0.001451469	0.008643912	0.27101562
ENSMUSG00000027834	Serpini1	20713	protein_coding	5.89140624	-0.27104583	0.582882419	0.465009445	0.641924693	0.804573016	0.27104583
ENSMUSG00000042770	Hebp1	15199	protein_coding	4.489521221	-0.271063955	0.561258309	0.482957581	0.629125855	0.797446101	0.271063955
ENSMUSG00000024402	Lta	16992	protein_coding	2.009876145	0.271370248	1.218240328	-0.222755922	0.823725475	1	0.271370248
ENSMUSG00000022863	Btg3	12228	protein_coding	75.74599489	-0.271385934	0.232278772	1.168363047	0.242660354	0.453724307	0.271385934
ENSMUSG00000009739	Pou6f1	19009	protein_coding	27.73103172	-0.271471073	0.267896795	1.013341997	0.310896834	0.531926058	0.271471073
ENSMUSG00000036120	Rfxank	19727	protein_coding	186.5110694	-0.271474988	0.084176716	3.225060318	0.001259461	0.007703947	0.271474988
ENSMUSG00000055531	Cpsf6	432508	protein_coding	925.5996912	0.271602087	0.064575567	-4.205957476	2.60E-05	0.000260832	0.271602087
ENSMUSG00000112481	Gm29684	628062	lncRNA	1.107287656	-0.271615637	1.318152018	0.2060579	0.836745684	1	0.271615637
ENSMUSG00000030761	Myo7a	17921	protein_coding	290.7957043	0.271646505	0.159159411	-1.706757415	0.087867151	0.225018236	0.271646505
ENSMUSG00000022999	Lmbr1l	74775	protein_coding	208.2188384	-0.271720106	0.14058925	1.932723204	0.053270305	0.156531285	0.271720106

Supplementary Table 1

ensembl_gene_id	external_gene_name	entrezgene_id	gene_biotype	baseMean	log2FoldChange	lfcSE	stat	pvalue	padj	absLog2FC
ENSMUSG00000051314	Ffar2	233079	protein_coding	249.7548097	0.271798773	0.192046834	-1.415273387	0.156988355	0.340132673	0.271798773
ENSMUSG00000038400	Pmepa1	65112	protein_coding	98.37356302	-0.271823642	0.166378383	1.633767787	0.102307619	0.251864642	0.271823642
ENSMUSG00000050957	Insl6	27356	protein_coding	35.66475697	-0.271833052	0.214776288	1.265656719	0.20563601	0.408255959	0.271833052
ENSMUSG00000066152	Slc31a2	20530	protein_coding	143.044918	-0.271947285	0.122161078	2.226136916	0.026005012	0.090068533	0.271947285
ENSMUSG00000032492	Pth1r	19228	protein_coding	1.780535617	0.272001368	1.692807558	-0.16068062	0.872344954	1	0.272001368
ENSMUSG00000022130	Tgds	76355	protein_coding	178.4023564	0.272027242	0.106143782	-2.562818441	0.010382633	0.043873796	0.272027242
ENSMUSG00000031367	Ap1s2	108012	protein_coding	23.04839775	0.272061674	0.283750331	-0.958806542	0.337656213	0.559563944	0.272061674
ENSMUSG00000034765	Dusp5	240672	protein_coding	309.9442989	-0.272105109	0.37331392	0.728890872	0.466068419	0.679736277	0.272105109
ENSMUSG00000036123	Slc9a3	105243	protein_coding	4235.618702	-0.272136944	0.201440574	1.350953976	0.176710176	0.368831124	0.272136944
ENSMUSG00000052155	Acvr2a	11480	protein_coding	556.1834559	0.272170651	0.076700461	-3.548487815	0.00038745	0.002851314	0.272170651
ENSMUSG00000018001	Cyth3	19159	protein_coding	195.2166562	-0.272185123	0.088666939	3.069747606	0.002142397	0.011989004	0.272185123
ENSMUSG00000060012	Kif13b	16554	protein_coding	2807.153215	-0.272240509	0.088896276	3.062451212	0.002195323	0.01219993	0.272240509
ENSMUSG00000047655	Vmn1r74	171240	protein_coding	0.990263078	-0.272243953	2.653927822	0.102581521	0.918295109	1	0.272243953
ENSMUSG00000051615	Rap2a	76108	protein_coding	930.4860802	0.272261631	0.072667284	-3.746687884	0.000179185	0.001439931	0.272261631
ENSMUSG00000038351	Sgsm2	97761	protein_coding	370.3515703	-0.2723031	0.08888544	3.063528741	0.002187432	0.012175009	0.2723031
ENSMUSG00000020941	Map3k14	53859	protein_coding	88.13626945	-0.272340137	0.136838598	1.99022894	0.046565722	0.141279869	0.272340137
ENSMUSG00000020532	Acaca	107476	protein_coding	919.7559771	0.272408031	0.098951601	-2.752942121	0.005906234	0.027859338	0.272408031
ENSMUSG00000001123	Lgals9	16859	protein_coding	821.7128348	0.272414692	0.185675278	-1.467156505	0.142333486	0.317806272	0.272414692
ENSMUSG00000035456	Prdm8	77630	protein_coding	1.496919971	-0.272415437	2.656245923	0.102556557	0.918314923	1	0.272415437
ENSMUSG00000041733	Coq5	52064	protein_coding	557.3082598	-0.272448239	0.063701891	4.276925449	1.89E-05	0.000196193	0.272448239
ENSMUSG00000042613	Pbxip1	229534	protein_coding	981.2944105	-0.272464731	0.085332107	3.192991953	0.001408069	0.008461735	0.272464731
ENSMUSG00000036904	Fzd8	14370	protein_coding	291.9464418	0.272491763	0.116642906	-2.336119458	0.019485016	0.071937304	0.272491763
ENSMUSG00000025591	Tma16	66282	protein_coding	82.92596611	-0.272534271	0.159250028	1.711360905	0.08701451	0.22354661	0.272534271
ENSMUSG00000026925	Inpp5e	64436	protein_coding	230.521519	-0.272545729	0.110285278	2.471279344	0.013463061	0.053650817	0.272545729
ENSMUSG00000086433	Rsf1os1	75448	lncRNA	1.353482348	-0.272557988	1.264771391	0.215499805	0.829377664	1	0.272557988
ENSMUSG00000022040	Ephx2	13850	protein_coding	300.8501988	-0.272598269	0.10154382	2.684538259	0.007263007	0.03286063	0.272598269
ENSMUSG00000056313	Tcim	69068	protein_coding	1698.250943	-0.272622025	0.125404713	2.173937632	0.029709819	0.10016904	0.272622025
ENSMUSG00000052229	Gpr17	574402	protein_coding	7.245093141	-0.272645162	0.693539802	0.393121146	0.694230007	0.837885199	0.272645162
ENSMUSG00000021127	Zfp36l1	12192	protein_coding	297.4017026	-0.272718646	0.127033564	2.146823537	0.031807333	0.105623626	0.272718646
ENSMUSG00000026238	Ptma	19231	protein_coding	4956.985153	0.272740592	0.155347687	-1.755678481	0.079143323	0.208481869	0.272740592
ENSMUSG00000032702	Kank1	107351	protein_coding	534.3326967	0.272754803	0.129198444	-2.111130702	0.034761079	0.112911971	0.272754803
ENSMUSG00000074647	Fam83c	71405	protein_coding	164.3508341	-0.272812866	0.122079728	2.234710632	0.025436352	0.088524117	0.272812866
ENSMUSG00000053774	Ubxn7	224111	protein_coding	402.4362548	-0.272814798	0.098006388	2.783643033	0.005375216	0.025856776	0.272814798
ENSMUSG00000019948	Actr6	67019	protein_coding	44.43404449	0.272896673	0.193864771	-1.407665103	0.15923026	0.343330529	0.272896673
ENSMUSG00000055976	Cldn23	71908	protein_coding	1184.808521	-0.272932919	0.218255632	1.250519478	0.211109845	0.415328565	0.272932919
ENSMUSG00000024766	Lipo3	381236	protein_coding	208.5524654	-0.27300293	0.170966085	1.596825063	0.110304707	0.265457875	0.27300293
ENSMUSG00000026548	Slamf9	98365	protein_coding	6.239719076	0.273004265	0.521396277	-0.523602253	0.600555214	0.777056241	0.273004265
ENSMUSG00000026489	Coq8a	67426	protein_coding	566.0013298	-0.2730084	0.165126563	1.653328183	0.09826409	0.244240395	0.2730084
ENSMUSG00000073468	Sft2d1	106489	protein_coding	205.0871782	-0.273123548	0.125778299	2.171467976	0.029895815	0.100654009	0.273123548

ensembl_gene_id	external_gene_name	entrezgene_id	gene_biotype	baseMean	log2FoldChange	lfcSE	stat	pvalue	padj	absLog2FC
ENSMUSG00000096370	Gm21992	102902673	protein_coding	49.54391665	0.273166941	0.160233811	-1.704802121	0.088231336	0.225445669	0.273166941
ENSMUSG00000030651	Art2b	11872	protein_coding	3.093952423	-0.273349773	0.814348482	0.33566683	0.737122122	1	0.273349773
ENSMUSG00000055198	Gm12830	433746	lncRNA	7.278838361	-0.273391961	0.387135327	0.706192234	0.480068599	0.691115487	0.273391961
ENSMUSG00000013701	Timm23	53600	protein_coding	357.4352556	-0.273500589	0.168598265	1.622202865	0.104759918	0.255880091	0.273500589
ENSMUSG00000103749	Pcdhgb5	93702	protein_coding	11.52110366	-0.273523263	0.323047538	0.846696635	0.397164235	0.619083741	0.273523263
ENSMUSG00000024558	Mapk4	225724	protein_coding	3.433521055	0.27354868	0.867809541	-0.315217414	0.752596584	1	0.27354868
ENSMUSG00000001349	Cnn1	12797	protein_coding	1.730209806	-0.273727896	1.201032647	0.227910454	0.819715855	1	0.273727896
ENSMUSG00000078653	Cntd1	68107	protein_coding	18.59462968	0.273752144	0.271899201	-1.006814817	0.314023773	0.5351691	0.273752144
ENSMUSG00000030987	Stim1	20866	protein_coding	1706.451417	-0.273772599	0.120327016	2.275238004	0.022891656	0.081578077	0.273772599
ENSMUSG00000029175	Slc35f6	74919	protein_coding	586.7981472	0.273821121	0.084910108	-3.224835372	0.001260451	0.007703947	0.273821121
ENSMUSG00000022972	Cfap298	68001	protein_coding	69.82455986	-0.273910794	0.164890261	1.661170233	0.096679262	0.241063164	0.273910794
ENSMUSG00000022865	Cxadr	13052	protein_coding	2541.696885	-0.274014119	0.076120023	3.59976401	0.000318506	0.002397361	0.274014119
ENSMUSG00000001627	Ifrd1	15982	protein_coding	1018.895549	-0.274226544	0.23326798	1.17558588	0.239760401	0.450136796	0.274226544
ENSMUSG00000035342	Lzts2	226154	protein_coding	857.8108083	-0.274238837	0.067865663	4.040907069	5.32E-05	0.000493703	0.274238837
ENSMUSG00000007476	Lrrc8a	241296	protein_coding	170.6372748	-0.274350294	0.150951381	1.817474555	0.069144481	0.189251307	0.274350294
ENSMUSG00000054302	Eapp	66266	protein_coding	331.9961671	-0.274406334	0.084219187	3.258240119	0.001121055	0.007009884	0.274406334
ENSMUSG00000027876	Reg4	67709	protein_coding	1841.309659	0.27444871	0.514558916	-0.533366932	0.593779604	0.772952576	0.27444871
ENSMUSG00000030695	Aldoa	11674	protein_coding	12023.19891	-0.27447424	0.073567265	3.730928994	0.000190775	0.001517562	0.27447424
ENSMUSG00000031434	Morc4	75746	protein_coding	103.8819292	0.274486581	0.172529689	-1.590952741	0.111620212	0.267599593	0.274486581
ENSMUSG00000016179	Camk1g	215303	protein_coding	7.676924393	0.274591236	0.605268814	-0.453668237	0.650067631	0.809917796	0.274591236
ENSMUSG00000075467	Dnlz	52838	protein_coding	263.0263576	-0.274625287	0.090150371	3.046302367	0.002316747	0.012761429	0.274625287
ENSMUSG00000071262	Zfp957	105590	protein_coding	1.695652921	-0.274639868	1.510760892	0.181789104	0.855748236	1	0.274639868
ENSMUSG00000030031	Kbtbd8	243574	protein_coding	139.0976541	0.274713722	0.140175064	-1.95979024	0.050020312	0.148948255	0.274713722
ENSMUSG00000047635	Mtrfr	72650	protein_coding	76.02140049	-0.274846038	0.1443833	1.903586061	0.056964116	0.164401504	0.274846038
ENSMUSG00000020546	Stxbp4	20913	protein_coding	35.31941154	0.274848767	0.173347473	-1.585536622	0.112844463	0.269566536	0.274848767
ENSMUSG00000052712	BC004004	80748	protein_coding	505.3211445	-0.2748639	0.063078876	4.357463493	1.32E-05	0.000142578	0.2748639
ENSMUSG00000030691	Fchsd2	207278	protein_coding	675.5212477	-0.27488335	0.098330428	2.795506487	0.005181846	0.025095735	0.27488335
ENSMUSG00000028609	Magoh	17149	protein_coding	349.6860809	0.27492277	0.10991512	-2.501227944	0.012376349	0.050254003	0.27492277
ENSMUSG00000065725	Gm26165	115486130	snoRNA	49.02067933	0.274930224	0.235660161	-1.166638534	0.243356375	0.454613934	0.274930224
ENSMUSG00000041654	Slc39a11	69806	protein_coding	1622.606275	0.274952073	0.100512099	-2.735512202	0.006228328	0.028942592	0.274952073
ENSMUSG00000040680	Kremen2	73016	protein_coding	118.2973199	-0.274967325	0.15014895	1.831297017	0.067056223	0.185023636	0.274967325
ENSMUSG00000021768	Dusp13	27389	protein_coding	5.501553555	-0.275016412	0.535452523	0.513614933	0.607521237	0.782086507	0.275016412
ENSMUSG00000043572	Pars2	230577	protein_coding	97.60868005	-0.275026553	0.145272995	1.893170534	0.058335195	0.167325234	0.275026553
ENSMUSG00000031706	Rfx1	19724	protein_coding	378.1262583	0.275064467	0.124847758	-2.203199086	0.02758072	0.094358402	0.275064467
ENSMUSG00000026031	Cflar	12633	protein_coding	924.7004247	0.275200917	0.084076959	-3.273202568	0.001063362	0.006694122	0.275200917
ENSMUSG00000026335	Pam	18484	protein_coding	2699.569679	-0.275263022	0.164458158	1.673757178	0.094178331	0.236336338	0.275263022
ENSMUSG00000005980	Dnase1	13419	protein_coding	12.87564026	0.275285559	0.348028812	-0.790984967	0.428952764	0.647921517	0.275285559
ENSMUSG00000026339	Ccdc93	70829	protein_coding	1048.404304	-0.27529202	0.101803289	2.70415644	0.006847804	0.031346224	0.27529202
ENSMUSG00000035069	Oma1	67013	protein_coding	555.8675535	-0.27533947	0.091183332	3.019625018	0.002530878	0.013766443	0.27533947

ensembl_gene_id	external_gene_name	entrezgene_id	gene_biotype	baseMean	log2FoldChange	lfcSE	stat	pvalue	padj	absLog2FC
ENSMUSG00000062519	Zfp398	272347	protein_coding	207.320157	-0.27536192	0.087230874	3.156702513	0.00159564	0.009377369	0.27536192
ENSMUSG00000034135	Sik3	70661	protein_coding	287.7969799	-0.275365548	0.103681707	2.655873985	0.007910317	0.035329044	0.275365548
ENSMUSG00000031410	Nxf7	170722	protein_coding	7.918472339	-0.275393423	0.463591955	0.594042713	0.552483545	0.743094418	0.275393423
ENSMUSG00000038388	Pals2	56524	protein_coding	725.2380489	0.275434678	0.135665557	-2.030247649	0.042331373	0.131303585	0.275434678
ENSMUSG00000028568	Btf3l4	70533	protein_coding	148.7525809	-0.275445414	0.101548839	2.712442769	0.006678931	0.030671822	0.275445414
ENSMUSG00000022105	Rb1	19645	protein_coding	559.4823068	-0.275463893	0.080875434	3.406026783	0.000659157	0.004469372	0.275463893
ENSMUSG00000037126	Psd	73728	protein_coding	81.2862406	0.275475078	0.164027495	-1.679444521	0.093065443	0.234222517	0.275475078
ENSMUSG00000024539	Ptpn2	19255	protein_coding	417.2228819	0.275575619	0.07437968	-3.704985279	0.000211403	0.001657697	0.275575619
ENSMUSG00000035773	Kiss1r	114229	protein_coding	18.25819984	-0.275686987	0.293545727	0.939161981	0.347647586	0.569548832	0.275686987
ENSMUSG00000038156	Spon1	233744	protein_coding	16.94585536	0.27578355	0.412218097	-0.669023394	0.503480555	0.708014489	0.27578355
ENSMUSG00000037280	Galnt6	207839	protein_coding	1620.472235	-0.275804738	0.092414377	2.984435418	0.002841022	0.015207984	0.275804738
ENSMUSG00000027176	Cstf3	228410	protein_coding	297.3904968	0.275812519	0.100206156	-2.752450854	0.005915102	0.027885242	0.275812519
ENSMUSG00000022973	Synj1	104015	protein_coding	316.4321615	-0.27582191	0.147757654	1.866718258	0.061940943	0.174614668	0.27582191
ENSMUSG00000096740	Lbhd1	102308570	protein_coding	58.36055564	0.275878877	0.151780956	-1.81761193	0.069123467	0.189251307	0.275878877
ENSMUSG00000015087	Rabl6	227624	protein_coding	763.9880864	0.275901557	0.066893308	-4.124501633	3.72E-05	0.000359858	0.275901557
ENSMUSG00000037221	Mospd3	68929	protein_coding	62.60449672	0.275952199	0.246518876	-1.119395819	0.262971314	0.477220689	0.275952199
ENSMUSG00000028945	Rheb	19744	protein_coding	584.45135	0.276014289	0.054666249	-5.049080407	4.44E-07	6.62E-06	0.276014289
ENSMUSG00000030739	Myh14	71960	protein_coding	11567.04495	0.276036414	0.165080265	-1.672134551	0.094497793	0.236875615	0.276036414
ENSMUSG00000025507	Pidd1	57913	protein_coding	56.29588947	0.276041931	0.173558396	-1.590484449	0.111725648	0.267745952	0.276041931
ENSMUSG00000038930	Rccd1	269955	protein_coding	68.7241451	-0.276067487	0.177679236	1.553740853	0.120246244	0.282387155	0.276067487
ENSMUSG00000041078	Grid1	14803	protein_coding	3.455580334	0.276184852	1.337119771	-0.206552067	0.836359698	1	0.276184852
ENSMUSG00000022687	Boc	117606	protein_coding	881.4940742	-0.276228171	0.121926139	2.265536939	0.02347975	0.083216624	0.276228171
ENSMUSG00000108171	Gm43915	215458	lncRNA	9.634940761	0.276235261	0.610535459	-0.452447531	0.650946612	0.810260975	0.276235261
ENSMUSG00000085929	Gm13421	105244102	lncRNA	16.19569016	0.276239497	0.304515816	-0.90714335	0.364330996	0.586981666	0.276239497
ENSMUSG00000031170	Slc38a5	209837	protein_coding	2.979517742	0.276288938	0.72733978	-0.379862268	0.704047658	1	0.276288938
ENSMUSG00000021676	Iqgap2	544963	protein_coding	6303.936767	0.276303098	0.093727826	-2.947930298	0.003199092	0.016787354	0.276303098
ENSMUSG00000040359	Ufl1	67490	protein_coding	382.3544299	0.27631276	0.121238866	-2.279077416	0.022662464	0.081006045	0.27631276
ENSMUSG00000041426	Hibch	227095	protein_coding	324.855701	-0.276357358	0.095973328	2.879522516	0.003982779	0.020119794	0.276357358
ENSMUSG00000085499	Gm15713	626693	lncRNA	2.878286837	0.276430578	0.677241503	-0.40817135	0.68314788	1	0.276430578
ENSMUSG00000100622	Gm20379	115488195	protein_coding	2.475493123	-0.276435203	0.816168811	0.338698562	0.734836824	1	0.276435203
ENSMUSG00000021577	Sdha	66945	protein_coding	5115.202895	-0.276543195	0.069316252	3.989586674	6.62E-05	0.00060057	0.276543195
ENSMUSG00000025486	Sirt3	64384	protein_coding	172.8727581	-0.276546223	0.130071809	2.126104218	0.033494583	0.109726154	0.276546223
ENSMUSG00000050144	Slc25a44	229517	protein_coding	567.9009961	0.276599484	0.095107087	-2.908295203	0.003634051	0.018683911	0.276599484
ENSMUSG00000072612	Gm10382	639281	lncRNA	238.8920579	0.276737404	0.234352979	-1.180857205	0.237659455	0.447999484	0.276737404
ENSMUSG00000022711	Pmm2	54128	protein_coding	2269.755702	0.276747727	0.050922905	-5.434641426	5.49E-08	9.36E-07	0.276747727
ENSMUSG00000040412	Elapor1	229722	protein_coding	3228.096386	-0.27679775	0.173757495	1.593011857	0.111157531	0.26676599	0.27679775
ENSMUSG00000022037	Clu	12759	protein_coding	46.70961116	-0.27685908	0.30879098	0.896590567	0.369937434	0.59260115	0.27685908
ENSMUSG00000035329	Fbxo33	70611	protein_coding	329.3806769	0.276874185	0.077868148	-3.555679584	0.000377003	0.002788738	0.276874185
ENSMUSG00000027309	Dnaaf9	228602	protein_coding	208.9466692	-0.277009716	0.158366785	1.749165499	0.080262416	0.21050366	0.277009716

Supplementary Table 1

ensembl_gene_id	external_gene_name	entrezgene_id	gene_biotype	baseMean	log2FoldChange	lfcSE	stat	pvalue	padj	absLog2FC
ENSMUSG00000098176	Ccdc166	223648	protein_coding	74.12393305	-0.277208705	0.164229931	1.68793047	0.091424577	0.231384745	0.277208705
ENSMUSG00000093803	Ppp2r3d	19054	protein_coding	295.97457	-0.277265644	0.122669521	2.260265158	0.023804799	0.084106216	0.277265644
ENSMUSG00000033159	Cnppd1	69171	protein_coding	673.9541981	-0.277268821	0.082814081	3.348087895	0.000813712	0.005342998	0.277268821
ENSMUSG00000060002	Chpt1	212862	protein_coding	1068.314852	-0.277308752	0.173792168	1.595634346	0.110570455	0.265929452	0.277308752
ENSMUSG00000027333	Smox	228608	protein_coding	246.4206691	-0.277338309	0.120225671	2.306814391	0.02106517	0.076471798	0.277338309
ENSMUSG00000032661	Oas3	246727	protein_coding	131.7518807	-0.277440447	0.524076319	0.529389397	0.596535352	0.774662172	0.277440447
ENSMUSG00000029646	Cdx2	12591	protein_coding	3237.993453	0.277472726	0.1369935	-2.025444465	0.042821746	0.132476266	0.277472726
ENSMUSG00000048878	Hexim1	192231	protein_coding	862.9329806	0.27747629	0.190897605	-1.453534679	0.146075358	0.323442761	0.27747629
ENSMUSG00000056234	Ncoa4	27057	protein_coding	639.7812737	-0.277497911	0.083837279	3.309958448	0.000933098	0.006002799	0.277497911
ENSMUSG00000073779	Lrp8os2	619295	lncRNA	4.06116636	0.277540126	0.848443327	-0.32711687	0.743579493	1	0.277540126
ENSMUSG00000031939	Taf1d	75316	protein_coding	1162.213486	-0.277595716	0.107062421	2.592839895	0.009518707	0.040914089	0.277595716
ENSMUSG00000029521	Chek2	50883	protein_coding	184.2800168	0.277651595	0.17351533	-1.600155992	0.109563982	0.264291499	0.277651595
ENSMUSG00000066571	Garre1	233103	protein_coding	215.5443069	-0.277687105	0.09582988	2.897708983	0.003758992	0.019206682	0.277687105
ENSMUSG00000054128	H2-T3	15043	protein_coding	556.5987613	-0.277804604	0.180604062	1.53819688	0.124000484	0.28837403	0.277804604
ENSMUSG00000042351	Grap2	17444	protein_coding	24.59776655	0.277958622	0.228821519	-1.214739867	0.224465322	0.431627891	0.277958622
ENSMUSG00000061136	Prpf40a	56194	protein_coding	1314.840574	0.278095914	0.071051163	-3.914023388	9.08E-05	0.000792278	0.278095914
ENSMUSG00000035297	Cops4	26891	protein_coding	846.6458988	0.278097043	0.090274244	-3.080580144	0.002065977	0.01165615	0.278097043
ENSMUSG00000015748	Prpf3	70767	protein_coding	196.1107853	0.278151339	0.09707284	-2.865387873	0.004164987	0.020855246	0.278151339
ENSMUSG00000019210	Atp6v1e1	11973	protein_coding	1086.52366	-0.278196959	0.062968898	4.418005854	9.96E-06	0.000111756	0.278196959
ENSMUSG00000024608	Rps14	20044	protein_coding	6383.655202	-0.278269296	0.091094027	3.054747973	0.002252497	0.012461318	0.278269296
ENSMUSG00000025207	Sema4g	26456	protein_coding	3469.686647	0.278343287	0.112868055	-2.466094484	0.01365953	0.054237431	0.278343287
ENSMUSG00000039197	Adk	11534	protein_coding	1766.617388	-0.278367492	0.044937996	6.194479459	5.85E-10	1.32E-08	0.278367492
ENSMUSG00000118376	Gm9926	100503565	lncRNA	118.3821918	-0.278392762	0.117221425	2.374930702	0.017552245	0.066267555	0.278392762
ENSMUSG00000080059	Rps19-ps3	277692	processed_pseudogene	7.008002651	-0.278420033	0.399966346	0.696108649	0.486360791	0.695624496	0.278420033
ENSMUSG00000029253	Cenpc1	12617	protein_coding	254.4039697	0.278451186	0.103687314	-2.685489441	0.007242366	0.032806559	0.278451186
ENSMUSG00000047654	Tssk6	83984	protein_coding	15.09213172	-0.278521165	0.269707467	1.032678734	0.301754269	0.522023104	0.278521165
ENSMUSG00000031590	Frg1	14300	protein_coding	512.2861752	-0.278632783	0.117950197	2.362291792	0.01816234	0.06795036	0.278632783
ENSMUSG00000072109	A530040E14Rik	621875	lncRNA	3.832151378	0.278647845	1.825451062	-0.152646023	0.878677429	1	0.278647845
ENSMUSG00000078861	Zfp931	353208	protein_coding	46.57902727	-0.278672379	0.144100304	1.933877805	0.053128154	0.156196961	0.278672379
ENSMUSG00000022701	Ccdc191	212153	protein_coding	92.31026356	-0.27871388	0.132626129	2.101500522	0.035597052	0.114868009	0.27871388
ENSMUSG00000030047	Arhgap25	232201	protein_coding	7.560596836	0.278733219	0.536975262	-0.519080187	0.603704825	0.779177919	0.278733219
ENSMUSG00000056145	AI504432	229694	lncRNA	4.115888	0.278775442	1.000013494	-0.278771681	0.780420049	1	0.278775442
ENSMUSG00000001911	Nfix	18032	protein_coding	252.0123995	-0.278852007	0.08478384	3.288975898	0.001005526	0.006384236	0.278852007
ENSMUSG00000027695	Pld1	18805	protein_coding	1605.020211	-0.278904581	0.073786418	3.779890506	0.000156897	0.00127605	0.278904581
ENSMUSG00000025221	Kcnip2	80906	protein_coding	5.615970765	-0.279023329	0.797089209	0.350052824	0.726299054	0.858315628	0.279023329
ENSMUSG00000012519	Mlkl	74568	protein_coding	228.5532404	-0.279087006	0.092746572	3.00913554	0.002619922	0.014180725	0.279087006
ENSMUSG00000021217	Tshz3	243931	protein_coding	1.301668583	-0.279093086	1.885054265	0.14805573	0.882298787	1	0.279093086
ENSMUSG00000024421	Lama3	16774	protein_coding	624.249901	-0.279122031	0.428743946	0.651022675	0.51503185	0.716272317	0.279122031
ENSMUSG00000060336	Zfp937	245174	protein_coding	33.18158986	-0.279157085	0.206328575	1.352973452	0.176064112	0.368018849	0.279157085

ensembl_gene_id	external_gene_name	entrezgene_id	gene_biotype	baseMean	log2FoldChange	lfcSE	stat	pvalue	padj	absLog2FC
ENSMUSG00000060703	Cd302	66205	protein_coding	34.81159356	0.279381909	0.184636009	-1.51314963	0.130241655	0.298143983	0.279381909
ENSMUSG00000038729	Pakap	677884	protein_coding	25.52320714	-0.279435872	0.30704444	0.910082827	0.36277883	0.585166009	0.279435872
ENSMUSG00000098490	Mir7089	102465660	miRNA	0.684017244	-0.27945767	1.520190873	0.183830646	0.854146314	1	0.27945767
ENSMUSG00000064293	Cntn4	269784	protein_coding	3.540275142	0.279475958	0.958951346	-0.291439142	0.770715482	1	0.279475958
ENSMUSG00000000385	Tmprss2	50528	protein_coding	5690.980347	-0.279479413	0.152569275	1.831819763	0.066978278	0.184932165	0.279479413
ENSMUSG00000014704	Hoxa2	15399	protein_coding	19.06172562	0.279495057	0.232493424	-1.202163283	0.229300272	0.438111017	0.279495057
ENSMUSG00000070283	Ndufaf3	66706	protein_coding	255.9193059	0.279528977	0.090598004	-3.085376767	0.002032944	0.011497276	0.279528977
ENSMUSG00000086596	Susd5	382111	protein_coding	1.505291913	-0.279559256	2.295075609	0.121808299	0.903050841	1	0.279559256
ENSMUSG00000034647	Ankrd12	106585	protein_coding	279.9576617	-0.279579555	0.098556843	2.836734074	0.004557756	0.022511824	0.279579555
ENSMUSG00000053182	Cd200l1	208166	protein_coding	117.0043917	-0.279580111	0.162537291	1.720098257	0.085414583	0.220499674	0.279580111
ENSMUSG00000026158	Ogfrl1	70155	protein_coding	35.20765603	-0.27963571	0.189070271	1.479004117	0.139139214	0.312363375	0.27963571
ENSMUSG00000031994	Adamts8	30806	protein_coding	2.933012214	0.279682305	1.408612659	-0.198551605	0.842613513	1	0.279682305
ENSMUSG00000026576	Atp1b1	11931	protein_coding	9681.290842	-0.279694768	0.097385517	2.872036576	0.004078358	0.020502282	0.279694768
ENSMUSG00000045639	Zfp629	320683	protein_coding	268.9488206	-0.279701255	0.108750882	2.5719447	0.010112904	0.042953833	0.279701255
ENSMUSG00000033166	Dis3	72662	protein_coding	232.1353528	0.2797226	0.146112329	-1.914435299	0.055564559	0.161179189	0.2797226
ENSMUSG00000049350	Zg16	69036	protein_coding	28919.21281	0.279760853	0.345492335	-0.809745469	0.418086479	0.638103523	0.279760853
ENSMUSG00000005089	Slc1a2	20511	protein_coding	12.82068931	0.279765404	0.559349707	-0.500161886	0.616961093	0.788881899	0.279765404
ENSMUSG00000026721	Rabgap1l	29809	protein_coding	987.4648096	-0.27986703	0.078873284	3.548312125	0.000387708	0.002851946	0.27986703
ENSMUSG00000080626	Mir1192	100316672	miRNA	3.899898349	-0.279976511	0.576581712	0.485579936	0.627265025	1	0.279976511
ENSMUSG00000031221	Igbp1	18518	protein_coding	783.1075684	-0.279996079	0.09131013	3.06642951	0.002166319	0.01208601	0.279996079
ENSMUSG00000027133	Nop10	66181	protein_coding	331.2885307	-0.280081829	0.1124728	2.490218329	0.012766464	0.05145848	0.280081829
ENSMUSG00000044244	Il20rb	213208	protein_coding	15.23688356	-0.280188043	0.340102817	0.823833348	0.410034256	0.630476965	0.280188043
ENSMUSG00000001289	Pfdn5	56612	protein_coding	932.5534379	-0.280222629	0.069851679	4.011680661	6.03E-05	0.000552495	0.280222629
ENSMUSG00000035572	Dcaf10	242418	protein_coding	398.5828702	0.280249385	0.094101393	-2.978164028	0.002899808	0.015447555	0.280249385
ENSMUSG00000042202	Slc35e2	320541	protein_coding	378.1969699	0.28028161	0.061870255	-4.530151171	5.89E-06	6.93E-05	0.28028161
ENSMUSG00000061315	Naca	17938	protein_coding	2724.967511	-0.280372855	0.094192597	2.976591187	0.002914724	0.015492034	0.280372855
ENSMUSG00000046994	Mars2	212679	protein_coding	265.0592007	-0.280389402	0.124291903	2.255894354	0.024077248	0.084814777	0.280389402
ENSMUSG00000020288	Ahsa2	268390	protein_coding	161.0010325	-0.280466921	0.10575009	2.652167211	0.007997692	0.035589839	0.280466921
ENSMUSG00000026553	Copa	12847	protein_coding	4823.750438	0.280497255	0.087727116	-3.19738376	0.001386803	0.008358271	0.280497255
ENSMUSG00000025224	Gbf1	107338	protein_coding	1315.261492	0.280540841	0.06878846	-4.078312566	4.54E-05	0.000429797	0.280540841
ENSMUSG00000046330	Rpl37a	19981	protein_coding	3648.62809	-0.280688302	0.107285466	2.616275176	0.008889488	0.038706422	0.280688302
ENSMUSG00000060470	Adgrg3	54672	protein_coding	4.949994743	-0.280719598	0.869884707	0.322708971	0.746915658	0.872440598	0.280719598
ENSMUSG00000025209	Twnk	226153	protein_coding	212.5054192	-0.28074595	0.08285856	3.388255231	0.000703388	0.004704636	0.28074595
ENSMUSG00000096458	Moap1	64113	protein_coding	4.181102908	0.280776623	0.542389368	-0.517666162	0.604691211	1	0.280776623
ENSMUSG00000042903	Foxo4	54601	protein_coding	386.5439698	-0.28081082	0.083126326	3.378121404	0.000729829	0.004853962	0.28081082
ENSMUSG00000021418	Rpp40	208366	protein_coding	36.85646396	-0.280876633	0.253488675	1.108044108	0.267842767	0.483088189	0.280876633
ENSMUSG00000032184	Lysmd2	70082	protein_coding	22.47833881	0.280890067	0.247546381	-1.13469672	0.256502412	0.469401688	0.280890067
ENSMUSG00000030789	Itgax	16411	protein_coding	11.1584588	0.280930756	0.646069412	-0.434830609	0.663685385	0.818589921	0.280930756
ENSMUSG00000026248	Mrpl44	69163	protein_coding	364.33407	0.280955266	0.097487729	-2.881955177	0.00395216	0.020007959	0.280955266

Supplementary Table 1

ensembl_gene_id	external_gene_name	entrezgene_id	gene_biotype	baseMean	log2FoldChange	lfcSE	stat	pvalue	padj	absLog2FC
ENSMUSG00000063275	Hacd1	30963	protein_coding	231.4904676	-0.280984393	0.131016376	2.144650924	0.031980767	0.10607158	0.280984393
ENSMUSG00000061778	Mospd2	76763	protein_coding	253.2571572	0.281019103	0.093796674	-2.99604552	0.002735056	0.014731479	0.281019103
ENSMUSG00000024019	Cmtr1	74157	protein_coding	72.71332969	0.281108642	0.18788546	-1.496170285	0.134609286	0.305377683	0.281108642
ENSMUSG00000071506	Tmem139	109218	protein_coding	371.9740942	-0.28112956	0.145564757	1.931302369	0.053445671	0.156906987	0.28112956
ENSMUSG00000039754	Alkbh4	72041	protein_coding	99.0284082	-0.281198232	0.125455289	2.241421901	0.024998759	0.087325531	0.281198232
ENSMUSG00000021710	Nln	75805	protein_coding	538.6498595	-0.281269231	0.071377114	3.940608076	8.13E-05	0.000718512	0.281269231
ENSMUSG00000040263	Klhdc4	234825	protein_coding	329.5586398	0.281296752	0.102869934	-2.734489481	0.006247709	0.02899794	0.281296752
ENSMUSG00000007867	Ift43	76411	protein_coding	184.4509084	-0.281369883	0.112708795	2.496432364	0.012544959	0.050788785	0.281369883
ENSMUSG00000032202	Rab27a	11891	protein_coding	560.1514198	0.281396592	0.159216726	-1.767380848	0.077164468	0.20483749	0.281396592
ENSMUSG00000041881	Ndufa7	66416	protein_coding	1366.383817	-0.281451382	0.091714487	3.068777808	0.002149364	0.012021919	0.281451382
ENSMUSG00000034813	Grip1	74053	protein_coding	31.94376817	-0.281466573	0.242867987	1.158928257	0.24648543	0.458287111	0.281466573
ENSMUSG00000048007	Timm8a1	30058	protein_coding	43.08266745	-0.281470582	0.183534927	1.533607725	0.125126187	0.290256194	0.281470582
ENSMUSG00000020916	Krt36	16673	protein_coding	30.28762411	0.281476524	0.429585316	-0.655228458	0.512320674	0.714747285	0.281476524
ENSMUSG00000046111	Cep295	319675	protein_coding	197.6715514	0.281560125	0.117943893	-2.387237841	0.016975505	0.064474124	0.281560125
ENSMUSG00000033917	Gde1	56209	protein_coding	529.3451247	-0.281578786	0.150974159	1.865079345	0.062170283	0.175022172	0.281578786
ENSMUSG00000048440	Cyp4f16	70101	protein_coding	440.5954303	-0.28158371	0.093043282	3.026373355	0.002475065	0.013502881	0.28158371
ENSMUSG00000079445	B3gnt7	227327	protein_coding	4128.456134	-0.281607624	0.421168303	0.66863442	0.50372871	0.708204999	0.281607624
ENSMUSG00000055407	Map6	17760	protein_coding	10.37842152	0.281686991	0.511951446	-0.550222083	0.582167059	0.76507296	0.281686991
ENSMUSG00000053886	Sh2d4a	72281	protein_coding	1138.950122	-0.281759647	0.123846823	2.275065593	0.022901995	0.081579713	0.281759647
ENSMUSG00000023988	Bysl	53414	protein_coding	222.8126808	0.28176839	0.121643382	-2.316347872	0.020539282	0.074891965	0.28176839
ENSMUSG00000056050	Mia3	338366	protein_coding	3166.743659	0.281786686	0.148092753	-1.902771605	0.057070354	0.164593053	0.281786686
ENSMUSG00000030341	Tnfrsf1a	21937	protein_coding	424.2590791	0.281813301	0.091663913	-3.074419264	0.002109128	0.011855051	0.281813301
ENSMUSG00000022876	Samsn1	67742	protein_coding	3.382096871	-0.281832727	0.727270683	0.387521088	0.698370476	1	0.281832727
ENSMUSG00000046861	Hectd3	76608	protein_coding	1273.321477	-0.281874681	0.094624752	2.978868378	0.00289315	0.015417065	0.281874681
ENSMUSG00000039470	Zdhhc2	70546	protein_coding	216.7699618	0.281966631	0.188930674	-1.492434369	0.135585311	0.306972281	0.281966631
ENSMUSG00000026638	Irf6	54139	protein_coding	2224.719441	-0.282009491	0.0645995	4.365505748	1.27E-05	0.000137975	0.282009491
ENSMUSG00000073607	Gm10548	633395	transcribed_processed_pseudogene	9.560768231	0.282034638	0.422367364	-0.667747233	0.504294954	0.708596728	0.282034638
ENSMUSG00000073557	Ppp1r12b	329251	protein_coding	144.1516595	0.282114342	0.135592508	-2.080604209	0.037470148	0.119710201	0.282114342
ENSMUSG00000025427	Rnf165	225743	protein_coding	3.741195933	-0.282264374	2.012589419	0.140249358	0.888462975	1	0.282264374
ENSMUSG00000020954	Strn3	94186	protein_coding	672.8073073	0.282326006	0.055455563	-5.09103125	3.56E-07	5.40E-06	0.282326006
ENSMUSG00000041697	Cox6a1	12861	protein_coding	5145.288174	-0.282346546	0.080470331	3.508703697	0.000450296	0.003244412	0.282346546
ENSMUSG00000018927	Ccl6	20305	protein_coding	2732.501412	-0.282361431	0.369957898	0.763225851	0.445328714	0.662536791	0.282361431
ENSMUSG00000031545	Gpat4	102247	protein_coding	1143.704641	0.282416461	0.072356274	-3.903137139	9.50E-05	0.000823133	0.282416461
ENSMUSG00000005338	Cadm3	94332	protein_coding	5.235101377	0.282420525	3.175992912	-0.088923538	0.929142677	0.966128972	0.282420525
ENSMUSG00000068735	Trp53i11	277414	protein_coding	2342.180673	0.28242601	0.120700587	-2.339889282	0.019289457	0.071374882	0.28242601
ENSMUSG00000047517	Dmbt1	12945	protein_coding	91353.67156	-0.282472945	0.11062084	2.553523783	0.0106639	0.044864238	0.282472945
ENSMUSG00000049550	Clip1	56430	protein_coding	670.1303144	-0.2825768	0.073663868	3.836029876	0.000125039	0.001052445	0.2825768
ENSMUSG00000026854	Usp20	74270	protein_coding	214.7805317	0.282617256	0.098466714	-2.870180622	0.004102374	0.020610466	0.282617256
ENSMUSG00000022540	Rogdi	66049	protein_coding	89.18914941	0.282622183	0.146673416	-1.926880758	0.053994492	0.157984593	0.282622183

ensembl_gene_id	external_gene_name	entrezgene_id	gene_biotype	baseMean	log2FoldChange	lfcSE	stat	pvalue	padj	absLog2FC
ENSMUSG00000023057	Fabp2	14079	protein_coding	3502.633321	0.282729487	0.124798239	-2.265492601	0.023482468	0.083216624	0.282729487
ENSMUSG00000074733	Zfp950	414758	protein_coding	205.47405	-0.282847741	0.132202129	2.139509725	0.032394409	0.107013657	0.282847741
ENSMUSG00000039741	Bahcc1	268515	protein_coding	246.3683421	0.282853021	0.167370312	-1.689983231	0.091031163	0.230625736	0.282853021
ENSMUSG00000037225	Fgf2	14173	protein_coding	9.458392223	0.282893298	0.448374648	-0.630930627	0.528085885	0.72618955	0.282893298
ENSMUSG00000028085	Gatb	229487	protein_coding	183.5377499	-0.282902604	0.089681469	3.154526881	0.001607586	0.009444208	0.282902604
ENSMUSG00000027959	Sass6	72776	protein_coding	62.01024492	0.282904583	0.197447065	-1.4328123	0.151911485	0.33244981	0.282904583
ENSMUSG00000030317	Timp4	110595	protein_coding	32.10263439	0.282948793	0.330848128	-0.85522259	0.392427932	0.615086833	0.282948793
ENSMUSG00000073427	Gm4924	237412	protein_coding	36.67678443	-0.282994823	0.197798337	1.430723977	0.152509339	0.333254034	0.282994823
ENSMUSG00000059401	Mamld1	333639	protein_coding	13.40253352	-0.283044729	0.299872341	0.943884079	0.345228888	0.566884219	0.283044729
ENSMUSG00000097032	4930539J05Rik	319587	lncRNA	12.7014376	0.283100423	0.546780447	-0.517758864	0.604626523	0.779942456	0.283100423
ENSMUSG00000005981	Trap1	68015	protein_coding	1169.531432	-0.283101573	0.088802173	3.188002754	0.001432592	0.008569101	0.283101573
ENSMUSG00000070604	Vsig10l	75690	protein_coding	26.74741685	0.28318488	0.329497938	-0.859443559	0.390095847	0.612475705	0.28318488
ENSMUSG00000078656	Vps25	28084	protein_coding	9.242983358	-0.28321466	0.359720769	0.787318066	0.431095697	0.649494967	0.28321466
ENSMUSG00000051403	Ppp1r37	232947	protein_coding	1312.181862	-0.283306873	0.048578057	5.831992643	5.48E-09	1.10E-07	0.283306873
ENSMUSG00000073676	Hspe1	15528	protein_coding	809.5418139	-0.283415942	0.131874781	2.149129187	0.031624161	0.105142217	0.283415942
ENSMUSG00000027883	Gpsm2	76123	protein_coding	730.145371	0.283680531	0.099151244	-2.861088974	0.004221885	0.021082618	0.283680531
ENSMUSG00000022257	Laptm4b	114128	protein_coding	1023.171683	0.283792302	0.086845295	-3.267791318	0.001083903	0.006808508	0.283792302
ENSMUSG00000056121	Fez2	225020	protein_coding	272.8114918	-0.283814893	0.104281639	2.72161902	0.006496299	0.029941356	0.283814893
ENSMUSG00000057522	Spop	20747	protein_coding	723.7392424	-0.283830657	0.101477096	2.79699232	0.005158076	0.025001604	0.283830657
ENSMUSG00000040111	Gramd1b	235283	protein_coding	167.6937489	-0.283870707	0.183388799	1.547917369	0.121642192	0.284460467	0.283870707
ENSMUSG00000085862	Gm13483	100502624	lncRNA	1.41208787	-0.283888703	1.069404888	0.265464191	0.790651892	1	0.283888703
ENSMUSG00000109865	Hspa14	50497	protein_coding	342.3548531	0.283899858	0.103401832	-2.745597935	0.006040074	0.028320777	0.283899858
ENSMUSG00000001016	Ilf2	67781	protein_coding	599.0691827	0.283957736	0.101753554	-2.79064196	0.005260363	0.025430219	0.283957736
ENSMUSG00000030218	Mgp	17313	protein_coding	1.922316784	-0.284041886	1.324702686	0.214419348	0.830220055	1	0.284041886
ENSMUSG00000020804	Aanat	11298	protein_coding	3.012966882	-0.284077796	0.955967431	0.29716263	0.766342351	1	0.284077796
ENSMUSG00000022288	4930447A16Rik	74892	lncRNA	2.073562015	-0.284105303	0.82794337	0.343145815	0.731488766	1	0.284105303
ENSMUSG00000042606	Hirip3	233876	protein_coding	123.1190782	0.284166499	0.168678105	-1.684667365	0.092052766	0.232532135	0.284166499
ENSMUSG00000086607	4930511M06Rik	75084	lncRNA	2.971304886	-0.284180066	0.876040502	0.32439147	0.745641677	1	0.284180066
ENSMUSG00000065395	Mir193a	387188	miRNA	1.018051733	-0.28424178	1.5345271	0.185230863	0.853047962	1	0.28424178
ENSMUSG00000025137	Pcyt2	68671	protein_coding	743.2073929	-0.284246121	0.063339605	4.487652261	7.20E-06	8.35E-05	0.284246121
ENSMUSG00000079003	Samd1	666704	protein_coding	188.4374237	-0.284322042	0.140445293	2.024432681	0.042925652	0.132673445	0.284322042
ENSMUSG00000019841	Rev3l	19714	protein_coding	621.4562572	0.284323627	0.078840862	-3.606297811	0.000310597	0.002350684	0.284323627
ENSMUSG00000108034	4930557K07Rik	78202	lncRNA	4.469464205	0.284410908	0.560939788	-0.507025734	0.612136763	0.785395579	0.284410908
ENSMUSG00000059810	Rgs3	50780	protein_coding	280.2856093	0.284539229	0.077835603	-3.655643654	0.000256538	0.001976883	0.284539229
ENSMUSG00000058331	Zfp85	22746	protein_coding	26.8020056	-0.284588989	0.234051785	1.215923176	0.224014188	0.431111794	0.284588989
ENSMUSG00000029088	Kcnip4	80334	protein_coding	5.908667535	-0.284707939	0.519274575	0.548280144	0.583499559	0.766020418	0.284707939
ENSMUSG00000021040	Slirp	380773	protein_coding	119.7636619	0.284732985	0.144903311	-1.964986055	0.049415852	0.147728706	0.284732985
ENSMUSG00000026475	Rgs16	19734	protein_coding	21.46153405	-0.284755502	0.315012217	0.903950659	0.366021565	0.588671613	0.284755502
ENSMUSG00000030138	Bms1	213895	protein_coding	521.3168353	0.284760069	0.069649483	-4.088473529	4.34E-05	0.000413537	0.284760069

ensembl_gene_id	external_gene_name	entrezgene_id	gene_biotype	baseMean	log2FoldChange	lfcSE	stat	pvalue	padj	absLog2FC
ENSMUSG00000021371	Mcur1	76137	protein_coding	608.4284643	-0.284784087	0.071755874	3.968791257	7.22E-05	0.000647282	0.284784087
ENSMUSG00000005610	Eif4g2	13690	protein_coding	7380.913851	0.284799863	0.073981729	-3.849597309	0.000118312	0.001000926	0.284799863
ENSMUSG00000024835	Coro1b	23789	protein_coding	2944.85137	-0.284825042	0.060709439	4.69161055	2.71E-06	3.41E-05	0.284825042
ENSMUSG00000045382	Cxcr4	12767	protein_coding	10.65614432	-0.284864223	0.376620189	0.756370029	0.449427372	0.666174493	0.284864223
ENSMUSG00000021363	Mak	17152	protein_coding	2.626865201	0.284915268	1.141935749	-0.249502013	0.802972484	1	0.284915268
ENSMUSG00000055633	Zfp580	68992	protein_coding	1.908987423	-0.284976369	1.152651964	0.2472354	0.80472605	1	0.284976369
ENSMUSG00000026393	Nek7	59125	protein_coding	658.2849535	0.285002625	0.103872562	-2.74377199	0.006073771	0.028422287	0.285002625
ENSMUSG00000075600	Zc3h3	223642	protein_coding	79.22584204	-0.285047502	0.194905016	1.462494435	0.143605768	0.319894352	0.285047502
ENSMUSG00000018008	Cyth4	72318	protein_coding	15.43700199	0.285123282	0.524878167	-0.543218027	0.586979696	0.768401324	0.285123282
ENSMUSG00000040728	Esrp1	207920	protein_coding	3237.534697	0.285134628	0.055160869	-5.169146753	2.35E-07	3.65E-06	0.285134628
ENSMUSG00000030407	Qpctl	67369	protein_coding	166.8552056	0.285163315	0.103477033	-2.755812627	0.005854653	0.02767136	0.285163315
ENSMUSG00000028199	Cryz	12972	protein_coding	172.5381559	-0.285283978	0.117140355	2.435403058	0.01487521	0.058004537	0.285283978
ENSMUSG00000037278	Tmem97	69071	protein_coding	569.0764841	0.285292363	0.152697101	-1.86835481	0.061712632	0.174119665	0.285292363
ENSMUSG00000005355	Casp14	12365	protein_coding	34.26789104	-0.285329079	0.367964504	0.775425552	0.438088241	0.6563215	0.285329079
ENSMUSG00000020085	Aifm2	71361	protein_coding	141.5467454	-0.285332756	0.140083632	2.036874341	0.041662636	0.12986349	0.285332756
ENSMUSG00000069305	H4c18	319161	protein_coding	875.018648	0.28536644	0.159162559	-1.792924424	0.072985009	0.196850041	0.28536644
ENSMUSG00000047721	Bola2	66162	protein_coding	299.6729182	-0.285371928	0.077532447	3.680677422	0.000232615	0.001809424	0.285371928
ENSMUSG00000029319	Coq2	71883	protein_coding	502.7051177	-0.285377935	0.112543108	2.535721109	0.011221606	0.046704567	0.285377935
ENSMUSG00000034981	Parm1	231440	protein_coding	1887.908261	-0.285379801	0.082192936	3.472072137	0.000516458	0.003646336	0.285379801
ENSMUSG00000031068	Glrx3	30926	protein_coding	565.4202544	-0.285526646	0.116449725	2.451930608	0.014209206	0.055893077	0.285526646
ENSMUSG00000097011	Gm4651	100043793	lncRNA	13.96596615	-0.285588798	0.296490137	0.963232036	0.335431091	0.557777472	0.285588798
ENSMUSG00000046748	Tmem45a2	69457	protein_coding	0.728947028	0.285604521	1.875190965	-0.152306899	0.878944884	1	0.285604521
ENSMUSG00000063439	B9d2	232987	protein_coding	18.67206116	-0.285615074	0.241021936	1.185016928	0.236010754	0.446089228	0.285615074
ENSMUSG00000028931	Kcnab2	16498	protein_coding	12.20204014	0.285639032	0.657864542	-0.434191256	0.664149562	0.818817302	0.285639032
ENSMUSG00000084745	Gm25290	115490269	snoRNA	1.18978069	-0.285658439	1.042505645	0.274011408	0.784075855	1	0.285658439
ENSMUSG00000025578	Cbx8	30951	protein_coding	119.7410823	0.285718204	0.118556155	-2.409982044	0.015953306	0.061382313	0.285718204
ENSMUSG00000028426	Rad23b	19359	protein_coding	1907.320276	0.285798149	0.07685457	-3.718687765	0.00020026	0.00158386	0.285798149
ENSMUSG00000053835	H2-T24	15042	protein_coding	18.68947736	0.285833508	0.306567616	-0.932366935	0.351146927	0.573211901	0.285833508
ENSMUSG00000033184	Tmed7	66676	protein_coding	2271.935811	0.285926541	0.091183477	-3.135727557	0.001714284	0.009957679	0.285926541
ENSMUSG00000062822	4833420G17Rik	67392	protein_coding	279.3079547	-0.285964596	0.092184173	3.102100786	0.001921525	0.010995173	0.285964596
ENSMUSG00000033405	Nudt15	214254	protein_coding	21.08043286	-0.285999816	0.295349353	0.968344142	0.332872526	0.555258272	0.285999816
ENSMUSG00000070737	Tmem35b	100039968	protein_coding	92.9752356	-0.286090244	0.148461571	1.927032306	0.053975604	0.157957314	0.286090244
ENSMUSG00000035295	Wdr38	76646	protein_coding	5.034171088	0.286175955	0.690399908	-0.414507522	0.67850246	0.827715862	0.286175955
ENSMUSG00000038371	Sbf2	319934	protein_coding	953.5920327	0.286197659	0.068528793	-4.176312559	2.96E-05	0.000293324	0.286197659
ENSMUSG00000041189	Chrnb1	11443	protein_coding	18.34427008	-0.286301706	0.294769554	0.97127299	0.331412355	0.553829552	0.286301706
ENSMUSG00000056468	5730596B20Rik	77580	TEC	19.35143286	-0.286303893	0.287703966	0.995133635	0.319671274	0.54094184	0.286303893
ENSMUSG00000039713	Plekhg5	269608	protein_coding	121.7499048	-0.286316801	0.108777763	2.632126208	0.008485234	0.03728716	0.286316801
ENSMUSG00000018398	Septin8	20362	protein_coding	503.7809586	-0.286377749	0.075289369	3.80369437	0.000142554	0.001178247	0.286377749
ENSMUSG00000006527	Sfmbt1	54650	protein_coding	174.2451667	0.286395276	0.119607583	-2.394457525	0.016644971	0.063554926	0.286395276

Supplementary Table 1

ensembl_gene_id	external_gene_name	entrezgene_id	gene_biotype	baseMean	log2FoldChange	lfcSE	stat	pvalue	padj	absLog2FC
ENSMUSG00000028124	Gclm	14630	protein_coding	872.249796	-0.28639703	0.081993863	3.492908126	0.000477791	0.003410008	0.28639703
ENSMUSG00000062991	Nrg1	211323	protein_coding	5.382176543	0.286410199	1.220141509	-0.234735231	0.814414241	0.907710402	0.286410199
ENSMUSG00000032727	Mier3	218613	protein_coding	948.3770307	-0.286453238	0.124148783	2.30733827	0.02103597	0.076382586	0.286453238
ENSMUSG00000027999	Pla2g12a	66350	protein_coding	90.50164269	-0.286505298	0.155628341	1.840958374	0.065627659	0.182116478	0.286505298
ENSMUSG00000042331	Specc1	432572	protein_coding	650.3511003	-0.286519446	0.097603638	2.935540637	0.00332967	0.017356745	0.286519446
ENSMUSG00000070372	Capza1	12340	protein_coding	789.169861	-0.286551824	0.108097008	2.650876553	0.008028318	0.035697265	0.286551824
ENSMUSG00000093989	Rnasek	52898	protein_coding	4.596643476	-0.286568966	0.488679953	0.58641441	0.557597063	0.746748674	0.286568966
ENSMUSG00000021814	Anxa7	11750	protein_coding	2004.027689	-0.286596447	0.117277576	2.443744645	0.014535711	0.056882163	0.286596447
ENSMUSG00000097852	4933405D12Rik	74057	lncRNA	7.163167024	0.286672409	0.405096245	-0.707664938	0.479153356	0.690541222	0.286672409
ENSMUSG00000029397	Rchy1	68098	protein_coding	400.1650067	0.286672591	0.07296797	-3.928745608	8.54E-05	0.000751269	0.286672591
ENSMUSG00000093445	Lrch4	231798	protein_coding	5.323629597	0.286712022	0.698135636	-0.410682404	0.681305431	0.829503357	0.286712022
ENSMUSG00000036923	Stox1	216021	protein_coding	5.85741463	0.286718592	0.448465678	-0.639332296	0.522606783	0.721840784	0.286718592
ENSMUSG00000087029	Gm14133	100503289	lncRNA	0.785192368	-0.286739242	1.304156744	0.219865628	0.825975806	1	0.286739242
ENSMUSG00000039246	Lyplal1	226791	protein_coding	93.09813448	0.286750292	0.136015601	-2.108216186	0.035012294	0.113460169	0.286750292
ENSMUSG00000020657	Dnajc27	217378	protein_coding	19.95841801	0.286769818	0.277715429	-1.032603119	0.301789668	0.522023104	0.286769818
ENSMUSG00000022236	Ropn1l	252967	protein_coding	2.435551349	0.286805303	0.740249285	-0.387444214	0.698427376	1	0.286805303
ENSMUSG00000017550	Atad5	237877	protein_coding	118.6480393	0.286812775	0.127262411	-2.253711636	0.024214315	0.085261236	0.286812775
ENSMUSG00000078995	Zfp456	408065	protein_coding	35.85525306	-0.286878508	0.211006589	1.359571328	0.173965616	0.36485499	0.286878508
ENSMUSG00000018965	Ywhah	22629	protein_coding	1404.141201	-0.286909327	0.107189898	2.676645218	0.007436333	0.033534573	0.286909327
ENSMUSG00000085024	C230035I16Rik	320842	lncRNA	3.163590988	-0.286911267	0.615739785	0.465961878	0.641242788	1	0.286911267
ENSMUSG00000031480	Thsd1	56229	protein_coding	3.374912589	0.286961766	0.853984513	-0.336026897	0.736850584	1	0.286961766
ENSMUSG00000040268	Plekha1	101476	protein_coding	634.9130397	-0.286997142	0.099838477	2.874614581	0.00404521	0.020354233	0.286997142
ENSMUSG00000071654	Uqcc3	107197	protein_coding	209.430851	-0.287059784	0.110298485	2.602572328	0.00925273	0.039999889	0.287059784
ENSMUSG00000021679	S100z	268686	protein_coding	2.623236859	-0.287136009	0.610466687	0.470354919	0.638101467	1	0.287136009
ENSMUSG00000032528	Vipr1	22354	protein_coding	3656.004552	-0.287141306	0.092171698	3.115287139	0.001837659	0.010584969	0.287141306
ENSMUSG00000033458	Fan1	330554	protein_coding	102.4799543	-0.287151679	0.119651284	2.399904706	0.01639934	0.062762157	0.287151679
ENSMUSG00000033313	Fbxl8	50788	protein_coding	140.8687522	-0.287241085	0.112335759	2.556987085	0.010558314	0.044513657	0.287241085
ENSMUSG00000050295	Foxc1	17300	protein_coding	24.96779171	0.287296977	0.345484607	-0.831576782	0.405647872	0.626589558	0.287296977
ENSMUSG00000019996	Map7	17761	protein_coding	2168.028734	0.287382664	0.069031923	-4.163040132	3.14E-05	0.000309798	0.287382664
ENSMUSG00000044641	Pard6b	58220	protein_coding	457.4460441	-0.287388739	0.185198708	1.551785875	0.120713467	0.283202471	0.287388739
ENSMUSG00000064672	Gm22806	115488214	snoRNA	3.327123334	-0.287395315	0.623290344	0.461093803	0.644731307	1	0.287395315
ENSMUSG00000039033	Tasp1	75812	protein_coding	160.6486602	0.287493419	0.095389201	-3.013899006	0.002579136	0.013987474	0.287493419
ENSMUSG00000032763	Ilvbl	216136	protein_coding	616.2683379	-0.28755931	0.064869504	4.432888968	9.30E-06	0.000105168	0.28755931
ENSMUSG00000021256	Vash1	238328	protein_coding	2.886258572	-0.28766464	0.958627913	0.300079557	0.764116472	1	0.28766464
ENSMUSG00000059602	Syn3	27204	protein_coding	25.02034235	-0.287697579	0.2792033	1.030423279	0.30281135	0.5233341	0.287697579
ENSMUSG00000028300	C9orf72	73205	protein_coding	158.0226828	0.28771223	0.12004144	-2.396774239	0.016540111	0.063213005	0.28771223
ENSMUSG00000020131	Pcsk4	18551	protein_coding	30.82095813	-0.287755825	0.283355301	1.015530059	0.309853225	0.530888475	0.287755825
ENSMUSG00000086690	4933406J10Rik	77800	lncRNA	2.568998568	-0.287779256	0.749735614	0.383840984	0.701096309	1	0.287779256
ENSMUSG00000039914	Coq10a	210582	protein_coding	130.2269776	-0.287802162	0.115380521	2.494373924	0.012617954	0.051034263	0.287802162

Supplementary Table 1

ensembl_gene_id	external_gene_name	entrezgene_id	gene_biotype	baseMean	log2FoldChange	lfcSE	stat	pvalue	padj	absLog2FC
ENSMUSG00000022217	Emc9	85308	protein_coding	49.39440565	-0.287824563	0.172029351	1.673113113	0.094305031	0.236536038	0.287824563
ENSMUSG00000024403	Atp6v1g2	66237	protein_coding	14.33366429	0.287888831	0.351960471	-0.817957851	0.413381255	0.633557127	0.287888831
ENSMUSG00000071855	Ccdc112	240261	protein_coding	33.21168901	0.287904226	0.206038507	-1.397332132	0.162313718	0.347734431	0.287904226
ENSMUSG00000029545	Acads	11409	protein_coding	3593.541056	-0.288006537	0.068498856	4.204545209	2.62E-05	0.000261952	0.288006537
ENSMUSG00000026750	Psmb7	19177	protein_coding	742.9782924	0.28808445	0.078007105	-3.693053962	0.000221577	0.001731719	0.28808445
ENSMUSG00000030516	Tjp1	21872	protein_coding	1349.560291	-0.288189771	0.093130557	3.094470607	0.001971645	0.011213534	0.288189771
ENSMUSG00000062328	Rpl17	319195	protein_coding	5286.330961	-0.28821999	0.111329176	2.588898984	0.009628333	0.041288574	0.28821999
ENSMUSG00000019970	Sgk1	20393	protein_coding	2154.152609	-0.288281038	0.238878604	1.206809791	0.227505405	0.436052025	0.288281038
ENSMUSG00000028393	Alad	17025	protein_coding	471.1127707	-0.288543872	0.102399401	2.817827724	0.004834974	0.023622712	0.288543872
ENSMUSG00000038712	Mindy1	75007	protein_coding	1077.118146	-0.288582934	0.077334547	3.731617319	0.000190254	0.00151488	0.288582934
ENSMUSG00000032579	Hemk1	69536	protein_coding	97.12007879	-0.288632816	0.112382161	2.568315228	0.010219418	0.043322217	0.288632816
ENSMUSG00000045467	Ttll13	269954	protein_coding	9.734603502	0.288635389	0.488318569	-0.591080102	0.554466752	0.744666516	0.288635389
ENSMUSG00000076430	Hus1b	210554	protein_coding	1.022839979	0.28870106	1.236236422	-0.233532239	0.815348138	1	0.28870106
ENSMUSG00000022131	Gpr180	58245	protein_coding	344.6795673	0.288715599	0.121836781	-2.36969161	0.017802928	0.06689364	0.288715599
ENSMUSG00000116665	E130310I04Rik	278725	lncRNA	4.695095975	0.288730807	0.738772736	-0.390824936	0.695926642	0.838607402	0.288730807
ENSMUSG00000002944	Cd36	12491	protein_coding	2.337678092	-0.288752939	1.31348983	0.219836448	0.825998532	1	0.288752939
ENSMUSG00000021178	Psmc1	19179	protein_coding	1068.429891	0.288825077	0.083256487	-3.469099999	0.000522205	0.003680621	0.288825077
ENSMUSG00000025481	Urah	76974	protein_coding	141.0284378	-0.288881082	0.118383968	2.440204421	0.014678953	0.057361152	0.288881082
ENSMUSG00000039018	Mtg1	212508	protein_coding	106.0851846	-0.288900697	0.134230235	2.152277364	0.031375516	0.104504894	0.288900697
ENSMUSG00000074865	Zfp934	77117	protein_coding	28.0031055	-0.289105972	0.261677657	1.104817185	0.269238813	0.484231077	0.289105972
ENSMUSG00000096169	Olfr1564	100043474	protein_coding	1.993834474	-0.289116031	2.136508922	0.135321705	0.892357524	1	0.289116031
ENSMUSG00000031938	4931406C07Rik	70984	protein_coding	1752.950469	-0.289151623	0.153164314	1.887852432	0.059045764	0.168933082	0.289151623
ENSMUSG00000049106	Dcaf5	320808	protein_coding	606.9068177	0.289167671	0.059121973	-4.89103552	1.00E-06	1.39E-05	0.289167671
ENSMUSG00000029699	Ssc4d	109267	protein_coding	48.95522193	0.289184993	0.254447754	-1.136520126	0.255738947	0.468471767	0.289184993
ENSMUSG00000015711	Prune1	229589	protein_coding	218.8802271	-0.289216849	0.086204948	3.354991274	0.000793675	0.005227463	0.289216849
ENSMUSG00000049922	Slc35c1	228368	protein_coding	2384.218455	0.289253496	0.081023874	-3.569978581	0.00035701	0.002661702	0.289253496
ENSMUSG00000001909	Trmt1	212528	protein_coding	468.3596643	-0.289310082	0.09134763	3.167132889	0.0015395	0.009099247	0.289310082
ENSMUSG00000023330	Dtwd1	69185	protein_coding	49.72229384	0.289313759	0.168188929	-1.720171247	0.085401318	0.220499674	0.289313759
ENSMUSG00000038213	Tapbpl	213233	protein_coding	416.4343254	0.289407673	0.093465775	-3.096402651	0.001958842	0.011158438	0.289407673
ENSMUSG00000009772	Nuak2	74137	protein_coding	523.9410574	0.289517925	0.117853552	-2.456590571	0.014026244	0.055334304	0.289517925
ENSMUSG00000032012	Nectin1	58235	protein_coding	143.238199	0.289543878	0.117260186	-2.469242879	0.013539928	0.053892113	0.289543878
ENSMUSG00000097712	Gm10532	100038353	lncRNA	2.843628676	0.289559025	0.855949992	-0.338289653	0.735144921	1	0.289559025
ENSMUSG00000055373	Fut9	14348	protein_coding	196.7253644	0.289703156	0.415579334	-0.697106744	0.485735994	0.695380179	0.289703156
ENSMUSG00000030103	Bhlhe40	20893	protein_coding	1843.336453	-0.289909527	0.114980197	2.521386591	0.011689335	0.048151079	0.289909527
ENSMUSG00000048938	Nr1h5	381463	protein_coding	1.913324202	-0.289913592	2.11424801	0.137123739	0.890932989	1	0.289913592
ENSMUSG00000035509	Fbxl21	213311	protein_coding	5.516442851	-0.290007858	0.49680251	0.583748777	0.559389345	0.747996408	0.290007858
ENSMUSG00000022562	Oplah	75475	protein_coding	420.6415778	-0.290026714	0.086211749	3.364120535	0.00076788	0.005084511	0.290026714
ENSMUSG00000024215	Spdef	30051	protein_coding	1556.002787	0.290029529	0.172297199	-1.683309602	0.092315173	0.23299599	0.290029529
ENSMUSG00000043020	Dnai3	242253	protein_coding	8.097700296	-0.29010248	0.386019621	0.751522627	0.452338189	0.668687928	0.29010248

ensembl_gene_id	external_gene_name	entrezgene_id	gene_biotype	baseMean	log2FoldChange	lfcSE	stat	pvalue	padj	absLog2FC
ENSMUSG00000048661	Lemd3	380664	protein_coding	340.302	0.290225818	0.080940003	-3.585690743	0.000336187	0.00251811	0.290225818
ENSMUSG00000026979	Psd4	215632	protein_coding	1069.882507	-0.290240298	0.089207567	3.253538996	0.001139771	0.007094676	0.290240298
ENSMUSG00000039158	Akna	100182	protein_coding	91.87337162	0.290265388	0.124724732	-2.327248049	0.019952067	0.073252209	0.290265388
ENSMUSG00000095098	Ccdc85b	240514	protein_coding	164.3266939	-0.290324241	0.131901325	2.201071454	0.027730966	0.094754639	0.290324241
ENSMUSG00000073096	Lrrc61	243371	protein_coding	60.0999971	-0.290378518	0.162409034	1.787945596	0.073784786	0.198449829	0.290378518
ENSMUSG00000033294	Noc4l	100608	protein_coding	181.419763	0.290382613	0.10692519	-2.715754945	0.006612484	0.030408957	0.290382613
ENSMUSG00000014504	Srp19	66384	protein_coding	293.9973561	0.290424732	0.0915403	-3.172643441	0.001510579	0.008943393	0.290424732
ENSMUSG00000091625	Lsm5	66373	protein_coding	73.59632009	0.290448374	0.162962783	-1.782298805	0.074700518	0.199909758	0.290448374
ENSMUSG00000034575	Tent4a	210106	protein_coding	293.2991915	0.290473902	0.072002822	-4.03420161	5.48E-05	0.000506309	0.290473902
ENSMUSG00000021886	Gpr65	14744	protein_coding	2.342899374	-0.290563612	0.845215072	0.343774764	0.731015681	1	0.290563612
ENSMUSG00000071379	Hpcal1	53602	protein_coding	770.7716787	-0.290656035	0.071328745	4.074879427	4.60E-05	0.000434691	0.290656035
ENSMUSG00000036622	Atp13a2	74772	protein_coding	447.2218301	0.290707555	0.075448871	-3.853040504	0.00011666	0.000987961	0.290707555
ENSMUSG00000097069	Gm16998	100502920	lncRNA	1.57763972	0.290797749	1.065457172	-0.272932368	0.784905205	1	0.290797749
ENSMUSG00000037601	Nme1	18102	protein_coding	304.499851	0.290819314	0.137739897	-2.111365836	0.034740879	0.112868558	0.290819314
ENSMUSG00000111329	A830035O19Rik	100101457	lncRNA	9.89619027	-0.290867214	0.36047287	0.806904589	0.419721455	0.6397711	0.290867214
ENSMUSG00000055026	Gabrg3	14407	protein_coding	3.321163435	-0.290931807	1.099226336	0.264669611	0.791263989	1	0.290931807
ENSMUSG00000066613	Zfp932	69504	protein_coding	70.80768165	-0.290937998	0.162984124	1.785069553	0.074250036	0.199311621	0.290937998
ENSMUSG00000038298	Pdzk1	59020	protein_coding	3.874629485	0.290971077	1.039543364	-0.279902779	0.779552095	1	0.290971077
ENSMUSG00000038102	Trappc11	320714	protein_coding	870.6608411	0.291088313	0.068044505	-4.27791068	1.89E-05	0.000195572	0.291088313
ENSMUSG00000033880	Lgals3bp	19039	protein_coding	2070.14766	0.291101277	0.173909062	-1.673870665	0.094156021	0.236336338	0.291101277
ENSMUSG00000062488	Ifit3b	667370	protein_coding	19.52741256	-0.291152679	0.518977063	0.561012615	0.574788937	0.760117839	0.291152679
ENSMUSG00000114203	Gm35279	102638802	lncRNA	2.119244396	0.291226016	0.859251629	-0.338929839	0.734662585	1	0.291226016
ENSMUSG00000030077	Chl1	12661	protein_coding	3.355168515	-0.291337049	1.181158164	0.246653715	0.80517623	1	0.291337049
ENSMUSG00000050503	Fbxl22	74165	protein_coding	23.12027733	0.291363506	0.254860078	-1.143229287	0.252943405	0.465324602	0.291363506
ENSMUSG00000079293	Clec7a	56644	protein_coding	1.192262421	-0.291376147	1.447254141	0.201330326	0.840440292	1	0.291376147
ENSMUSG00000050840	Cdh20	23836	protein_coding	1.807692416	0.291403192	1.76176707	-0.165403927	0.868626063	1	0.291403192
ENSMUSG00000041453	Rpl21	19933	protein_coding	4333.555043	-0.291555507	0.116663847	2.499107606	0.01245065	0.050506026	0.291555507
ENSMUSG00000031374	Zfp92	22754	protein_coding	4.108695213	0.291564351	0.787878207	-0.370062719	0.711335759	1	0.291564351
ENSMUSG00000046675	Tmem251	320351	protein_coding	113.3849813	0.291622427	0.102334245	-2.849705184	0.004375977	0.021779652	0.291622427
ENSMUSG00000070563	Spaca4	69363	protein_coding	116.5352562	-0.291677068	0.19660808	1.483545675	0.137929491	0.310491768	0.291677068
ENSMUSG00000029823	Luc7l2	192196	protein_coding	1391.947578	0.291707299	0.088245763	-3.30562385	0.000947652	0.00608931	0.291707299
ENSMUSG00000021824	Ap3m1	55946	protein_coding	793.6076594	0.291772179	0.08554874	-3.410595856	0.000648211	0.004405167	0.291772179
ENSMUSG00000020392	Cdkn2aipnl	52626	protein_coding	412.7546711	0.29181261	0.118556843	-2.461372976	0.01384064	0.054759062	0.29181261
ENSMUSG00000118462	Lhb	100040019	protein_coding	2.032214403	0.29181549	1.621095076	-0.180011336	0.857143669	1	0.29181549
ENSMUSG00000060063	Alox5ap	11690	protein_coding	30.17882555	-0.291859223	0.228799712	1.275610102	0.202093381	0.404039964	0.291859223
ENSMUSG00000058729	Lin9	72568	protein_coding	106.8479264	0.29187458	0.136943569	-2.131349293	0.033060377	0.108626953	0.29187458
ENSMUSG00000055980	Irs1	16367	protein_coding	92.9877376	0.29199639	0.235127894	-1.241861975	0.214287492	0.419287439	0.29199639
ENSMUSG00000030235	Slco1c1	58807	protein_coding	3.800668815	-0.292004448	1.021944274	0.285734218	0.775081695	1	0.292004448
ENSMUSG00000030664	Sox6os	68469	lncRNA	1.598836572	0.292023816	0.901504125	-0.323929539	0.745991379	1	0.292023816

ensembl_gene_id	external_gene_name	entrezgene_id	gene_biotype	baseMean	log2FoldChange	lfcSE	stat	pvalue	padj	absLog2FC
ENSMUSG00000050947	Amigo1	229715	protein_coding	141.1691325	-0.29209991	0.125595748	2.325714958	0.020033762	0.073420411	0.29209991
ENSMUSG00000042688	Mapk6	50772	protein_coding	1132.244422	-0.292107672	0.096586065	3.02432523	0.002491884	0.013581179	0.292107672
ENSMUSG00000078937	Cpt1b	12895	protein_coding	6.768835035	0.292127614	0.473279037	-0.617241821	0.537075235	0.732754497	0.292127614
ENSMUSG00000073402	Gm8909	667977	protein_coding	49.97551737	-0.292162539	0.377322226	0.774305138	0.438750366	0.656832884	0.292162539
ENSMUSG00000031347	Cetn2	26370	protein_coding	170.2792819	0.292231568	0.141164995	-2.070141882	0.03843906	0.122144051	0.292231568
ENSMUSG00000043460	Elfn2	207393	protein_coding	26.88350363	0.292239761	0.284521671	-1.027126545	0.30436088	0.52454765	0.292239761
ENSMUSG00000028194	Ddah1	69219	protein_coding	759.2474552	0.292301735	0.093117873	-3.13905082	0.00169496	0.009870825	0.292301735
ENSMUSG00000028630	Dyrk2	69181	protein_coding	1274.801428	-0.29240713	0.1160697	2.5192374	0.011760933	0.048327356	0.29240713
ENSMUSG00000031934	Panx1	55991	protein_coding	266.6679967	-0.292412637	0.098220107	2.977115838	0.00290974	0.015480494	0.292412637
ENSMUSG00000059323	Tonsl	72749	protein_coding	268.3818435	0.292495762	0.117978681	-2.479225575	0.013166801	0.05273746	0.292495762
ENSMUSG00000008601	Rab25	53868	protein_coding	1413.978257	-0.292548452	0.078802909	3.71240675	0.000205298	0.001618275	0.292548452
ENSMUSG00000029193	Cckar	12425	protein_coding	2.051617315	-0.292593854	2.156048651	0.135708373	0.892051828	1	0.292593854
ENSMUSG00000040729	Cep126	234915	protein_coding	37.94135907	0.292751161	0.190386621	-1.537666667	0.124130138	0.288572541	0.292751161
ENSMUSG00000046338	Gpat2	215456	protein_coding	10.11522933	-0.292755171	0.34682433	0.844102174	0.398612313	0.620539568	0.292755171
ENSMUSG00000061244	Exoc5	105504	protein_coding	872.4276925	0.292799482	0.059882301	-4.889583014	1.01E-06	1.39E-05	0.292799482
ENSMUSG00000020553	Pctp	18559	protein_coding	238.7040467	-0.292808614	0.116229703	2.519223636	0.011761393	0.048327356	0.292808614
ENSMUSG00000073481	Mtarc2	67247	protein_coding	940.4328762	-0.292849032	0.072890863	4.017637036	5.88E-05	0.000540217	0.292849032
ENSMUSG00000053877	Srcap	100043597	protein_coding	127.2978072	0.292921331	0.194155218	-1.508696674	0.131376316	0.300075862	0.292921331
ENSMUSG00000028391	Wdr31	71354	protein_coding	44.82824573	0.29299872	0.165764894	-1.76755592	0.077135173	0.204792643	0.29299872
ENSMUSG00000020739	Nup85	445007	protein_coding	347.8979032	0.29314291	0.142656614	-2.054884811	0.039890132	0.125701628	0.29314291
ENSMUSG00000037907	Ankrd13b	268445	protein_coding	88.36566004	-0.29322344	0.156645277	1.871894545	0.061221194	0.17331619	0.29322344
ENSMUSG00000021057	Akap5	238276	protein_coding	5.422027834	0.293337524	0.812429298	-0.361062218	0.718052936	0.85370239	0.293337524
ENSMUSG00000048495	Tyw5	68736	protein_coding	51.57329318	0.2933697	0.146890163	-1.997204541	0.045802968	0.139478188	0.2933697
ENSMUSG00000005410	Mcm5	17218	protein_coding	524.3027787	0.2934684	0.178967057	-1.639790054	0.101048827	0.249608668	0.2934684
ENSMUSG00000036306	Lzts1	211134	protein_coding	5.077591883	0.293670084	0.558075198	-0.526219558	0.598735667	0.776186935	0.293670084
ENSMUSG00000055061	4931431C16Rik	74364	lncRNA	2.502415758	-0.293816311	0.964381228	0.304668219	0.760618857	1	0.293816311
ENSMUSG00000016534	Lamp2	16784	protein_coding	1377.733359	-0.293822425	0.079195002	3.710113235	0.000207167	0.001631449	0.293822425
ENSMUSG00000078580	E430018J23Rik	101604	protein_coding	123.2827398	-0.293836313	0.099551918	2.951588676	0.003161438	0.016610875	0.293836313
ENSMUSG00000041879	Ipo9	226432	protein_coding	445.2200558	0.293867801	0.133507564	-2.201132229	0.027726664	0.094754639	0.293867801
ENSMUSG00000074623	Gm826	329554	protein_coding	3.220125009	-0.293874231	0.819649188	0.358536597	0.719941785	1	0.293874231
ENSMUSG00000031594	Fgl1	234199	protein_coding	12.74139141	-0.293897	0.280358461	1.048290102	0.294504968	0.513672903	0.293897
ENSMUSG00000019194	Scn1b	20266	protein_coding	4.66818688	0.294104738	0.733696701	-0.400853293	0.68852814	0.83386713	0.294104738
ENSMUSG00000016526	Dyrk3	226419	protein_coding	12.89876351	0.294104743	0.293616188	-1.001663925	0.316505936	0.537548029	0.294104743
ENSMUSG00000058546	Rpl23a	268449	protein_coding	1833.197884	-0.294126466	0.117294751	2.50758421	0.012155961	0.04960305	0.294126466
ENSMUSG00000038690	Atp5j2	57423	protein_coding	2721.202412	-0.294175284	0.08132283	3.61737636	0.000297604	0.002263768	0.294175284
ENSMUSG00000056459	Zbtb25	109929	protein_coding	25.48307226	0.294242402	0.215801233	-1.363488048	0.172728749	0.363275924	0.294242402
ENSMUSG00000063316	Rpl27	19942	protein_coding	1155.504046	-0.294252395	0.124191205	2.369349713	0.017819395	0.066917689	0.294252395
ENSMUSG00000037390	Muc3	666339	protein_coding	8415.002334	-0.294300607	0.223582281	1.31629665	0.188074495	0.384388887	0.294300607
ENSMUSG00000059439	Bcas3	192197	protein_coding	218.4451014	-0.294337255	0.145144835	2.02788652	0.042571831	0.131848679	0.294337255

ensembl_gene_id	external_gene_name	entrezgene_id	gene_biotype	baseMean	log2FoldChange	lfcSE	stat	pvalue	padj	absLog2FC
ENSMUSG00000021262	Evl	14026	protein_coding	80.31801567	-0.294346368	0.207994146	1.415166598	0.157019656	0.340132673	0.294346368
ENSMUSG00000031682	1700011L22Rik	67687	protein_coding	0.682712175	-0.294356578	1.472861608	0.199853521	0.841595142	1	0.294356578
ENSMUSG00000096472	Cdkn2d	12581	protein_coding	78.13233696	0.29436986	0.177592134	-1.657561365	0.097406033	0.242436056	0.29436986
ENSMUSG00000034071	Zfp551	619331	protein_coding	32.42119753	-0.294387204	0.211315704	1.393115601	0.163584836	0.349700969	0.294387204
ENSMUSG00000032068	Plet1	76509	protein_coding	802.1542491	-0.294412439	0.2425028	1.214057897	0.224725616	0.432027803	0.294412439
ENSMUSG00000031711	Zfp330	30932	protein_coding	425.6325122	0.294574089	0.087968969	-3.348613632	0.00081217	0.005334992	0.294574089
ENSMUSG00000107476	Zfp862-ps	58894	transcribed_unitary_pseudogene	88.99500903	-0.294682865	0.145757822	2.021729343	0.043204321	0.133360029	0.294682865
ENSMUSG00000090291	Lrrc10b	278795	protein_coding	2.293005535	0.294798275	0.830208707	-0.355089356	0.72252265	1	0.294798275
ENSMUSG00000001942	Siae	22619	protein_coding	317.4246897	-0.294850006	0.098432459	2.995455029	0.002740357	0.014751683	0.294850006
ENSMUSG00000034353	Ramp1	51801	protein_coding	567.6754566	-0.29497883	0.283324294	1.041134967	0.297812913	0.517476912	0.29497883
ENSMUSG00000030155	Clec2e	232409	protein_coding	297.2433641	-0.295085333	0.291339203	1.012858312	0.311127843	0.532085855	0.295085333
ENSMUSG00000043716	Rpl7	19989	protein_coding	8169.21816	-0.295241172	0.11942709	2.472145754	0.013430475	0.053546803	0.295241172
ENSMUSG00000004267	Eno2	13807	protein_coding	4.609587994	0.295268979	1.320899878	-0.22353623	0.823118189	0.910297568	0.295268979
ENSMUSG00000027257	Pacsin3	80708	protein_coding	77.98923128	-0.295277783	0.135020916	2.186904014	0.028749532	0.097588853	0.295277783
ENSMUSG00000018727	Cpsf4l	52670	protein_coding	4.995080223	0.295406404	0.558284132	-0.529132724	0.596713382	0.774787312	0.295406404
ENSMUSG00000021798	Ldb3	24131	protein_coding	146.4849421	0.295446893	0.171622154	-1.72149624	0.085160814	0.219982118	0.295446893
ENSMUSG00000001281	Itgb7	16421	protein_coding	23.40675992	0.295506007	0.419969874	-0.703636201	0.481659361	0.692385331	0.295506007
ENSMUSG00000042073	Abhd14b	76491	protein_coding	323.1566715	-0.295575166	0.089867447	3.289012599	0.001005395	0.006384236	0.295575166
ENSMUSG00000062157	Ifnlr1	242700	protein_coding	166.4272197	-0.295675139	0.1004065	2.944780864	0.003231835	0.016926901	0.295675139
ENSMUSG00000010492	Uckl1os	100271841	lncRNA	23.56098448	-0.295680621	0.302546531	0.97730627	0.32841755	0.55060786	0.295680621
ENSMUSG00000014294	Ndufa2	17991	protein_coding	1087.156071	0.295704783	0.091163448	-3.243677034	0.001179975	0.007309115	0.295704783
ENSMUSG00000088252	Snord13	100217422	snoRNA	122.0335719	0.295717737	0.237385065	-1.245730172	0.212863473	0.417634239	0.295717737
ENSMUSG00000028251	Tstd3	77032	protein_coding	78.52046811	-0.295888761	0.164546773	1.798204579	0.072144594	0.195389167	0.295888761
ENSMUSG00000044548	Dact1	59036	protein_coding	2.2809594	-0.295941968	0.958334127	0.308808754	0.75746701	1	0.295941968
ENSMUSG00000049281	Scn3b	235281	protein_coding	2.765669134	0.296056832	0.788995611	-0.375232546	0.707487527	1	0.296056832
ENSMUSG00000085272	Sbk3	381835	protein_coding	1.997001449	0.296063347	1.971555758	-0.150167372	0.880632567	1	0.296063347
ENSMUSG00000015127	Unkl	74154	protein_coding	58.65172907	-0.296071246	0.217341947	1.362237024	0.173123095	0.363688435	0.296071246
ENSMUSG00000027829	Ccnl1	56706	protein_coding	1116.24025	-0.296099288	0.135707426	2.181894518	0.029117319	0.098614315	0.296099288
ENSMUSG00000031639	Tlr3	142980	protein_coding	314.0775447	0.296175953	0.07562197	-3.916533167	8.98E-05	0.00078574	0.296175953
ENSMUSG00000067367	Lyar	17089	protein_coding	194.2987943	0.296220836	0.113361364	-2.613066961	0.008973372	0.03900665	0.296220836
ENSMUSG00000047141	Zfp654	72020	protein_coding	341.9919037	0.296238497	0.114124541	-2.59574755	0.009438538	0.040685989	0.296238497
ENSMUSG00000055214	Pld5	319455	protein_coding	4.237035004	0.296267717	0.916480482	-0.323266805	0.746493192	1	0.296267717
ENSMUSG00000030409	Dmpk	13400	protein_coding	55.95799308	0.296340018	0.195965945	-1.51220161	0.130482582	0.298488621	0.296340018
ENSMUSG00000028367	Txn1	22166	protein_coding	6987.792315	-0.296362763	0.087306858	3.394495825	0.000687551	0.004614708	0.296362763
ENSMUSG00000009376	Met	17295	protein_coding	848.4251323	-0.29638196	0.087755395	3.377364539	0.00073184	0.004863751	0.29638196
ENSMUSG00000026062	Slc9a2	226999	protein_coding	3888.855354	-0.296567909	0.127273248	2.330166887	0.019797332	0.07274578	0.296567909
ENSMUSG00000063931	Pepd	18624	protein_coding	554.9297218	0.296618873	0.069849743	-4.246527783	2.17E-05	0.00022214	0.296618873
ENSMUSG00000031754	Nudt21	68219	protein_coding	227.7820049	-0.296673119	0.145517902	2.038739673	0.041476016	0.129452831	0.296673119
ENSMUSG00000099988	1700054A03Rik	78457	lncRNA	6.004438827	-0.29683787	0.617033201	0.481072768	0.630464782	0.79831751	0.29683787

ensembl_gene_id	external_gene_name	entrezgene_id	gene_biotype	baseMean	log2FoldChange	lfcSE	stat	pvalue	padj	absLog2FC
ENSMUSG00000032047	Acat1	110446	protein_coding	1973.464263	-0.296849264	0.117120776	2.534556831	0.011258966	0.046798532	0.296849264
ENSMUSG00000063767	S100a7a	381493	protein_coding	2.069222869	0.296859154	0.847567951	-0.350248206	0.726152432	1	0.296859154
ENSMUSG00000047642	D930020B18Rik	216393	protein_coding	21.42500458	0.296861095	0.359427841	-0.825926825	0.408845603	0.629409554	0.296861095
ENSMUSG00000039637	Coro7	78885	protein_coding	291.0859764	0.296941956	0.124671296	-2.381798908	0.017228303	0.065269142	0.296941956
ENSMUSG00000047428	Dlk2	106565	protein_coding	2.994804949	0.297077112	1.074290021	-0.276533437	0.782138381	1	0.297077112
ENSMUSG00000086358	Gm13270	105244084	lncRNA	6.265431712	0.297078903	0.519607023	-0.571737658	0.567499718	0.753472451	0.297078903
ENSMUSG00000098973	Mir6236	102465959	miRNA	33850.15109	0.297110487	0.720734547	-0.412232892	0.680168731	0.828402273	0.297110487
ENSMUSG00000021257	Angel1	68737	protein_coding	59.47326982	-0.297158992	0.143473595	2.071175478	0.0383424	0.12191044	0.297158992
ENSMUSG00000085396	Firre	103012	lncRNA	687.0072759	0.297161967	0.146224942	-2.032224884	0.042130895	0.130976957	0.297161967
ENSMUSG00000026433	Rab29	226422	protein_coding	120.5681597	-0.297217402	0.137740596	2.157805395	0.030942965	0.103335112	0.297217402
ENSMUSG00000042210	Abhd14a	68644	protein_coding	114.0873029	-0.297398973	0.174323508	1.706017599	0.088004803	0.22515016	0.297398973
ENSMUSG00000022031	Elp3	74195	protein_coding	600.7050092	0.297469239	0.107081239	-2.777977185	0.005469846	0.026243183	0.297469239
ENSMUSG00000031782	Coq9	67914	protein_coding	1052.411883	-0.297584653	0.069496561	4.282005447	1.85E-05	0.000192981	0.297584653
ENSMUSG00000028980	H6pd	100198	protein_coding	441.7330111	0.29761894	0.123528875	-2.40930666	0.015982862	0.061453081	0.29761894
ENSMUSG00000036390	Gadd45a	13197	protein_coding	395.2938373	0.297635228	0.196495949	-1.514714326	0.129844765	0.297648036	0.297635228
ENSMUSG00000031386	Hcfc1	15161	protein_coding	1346.196222	0.297638565	0.076506413	-3.890374038	0.00010009	0.000861774	0.297638565
ENSMUSG00000027800	Tm4sf1	17112	protein_coding	23.38318779	-0.2976762	0.252460884	1.179098304	0.238359034	0.448589299	0.2976762
ENSMUSG00000040711	Sh3pxd2b	268396	protein_coding	98.90978726	-0.297692568	0.134756503	2.209114666	0.027166666	0.093231573	0.297692568
ENSMUSG00000026134	Prim2	19076	protein_coding	96.62734088	0.297815765	0.17469238	-1.704801122	0.088231522	0.225445669	0.297815765
ENSMUSG00000087406	E130215H24Rik	109344	lncRNA	8.711785259	0.297899637	0.368355923	-0.808727694	0.418671795	0.638701711	0.297899637
ENSMUSG00000021645	Smn1	20595	protein_coding	86.90196801	0.297911938	0.133835706	-2.2259526	0.026017357	0.090073507	0.297911938
ENSMUSG00000034285	Nipsnap1	18082	protein_coding	592.7549703	-0.297979209	0.092632225	3.216798573	0.001296296	0.007888281	0.297979209
ENSMUSG00000073764	Gm12888	545677	protein_coding	0.631497094	0.297991747	1.50908882	-0.197464684	0.843463915	1	0.297991747
ENSMUSG00000039155	Cdh26	381409	protein_coding	21.34531719	0.298036901	0.242209107	-1.2304942	0.218512098	0.424602824	0.298036901
ENSMUSG00000110754	4930405J17Rik	73811	lncRNA	12.87500499	-0.298121852	0.43216107	0.689839674	0.490295016	0.69793377	0.298121852
ENSMUSG00000028998	Tomm7	66169	protein_coding	1052.543416	-0.298167841	0.070484895	4.230237409	2.33E-05	0.000236946	0.298167841
ENSMUSG00000070570	Slc17a7	72961	protein_coding	2.930631261	-0.298210033	1.981825654	0.150472385	0.880391936	1	0.298210033
ENSMUSG00000002808	Epdr1	105298	protein_coding	12.35386077	0.298334027	0.46006166	-0.64846531	0.51668404	0.717449153	0.298334027
ENSMUSG00000114953	4930519K11Rik	78019	lncRNA	3.677895781	-0.298419904	0.54923255	0.5433398	0.586895866	1	0.298419904
ENSMUSG00000059866	Tnip2	231130	protein_coding	149.1086606	0.298459575	0.100612111	-2.966437851	0.003012712	0.015925715	0.298459575
ENSMUSG00000045930	Clec14a	66864	protein_coding	296.1161789	-0.298587518	0.163936103	1.821365227	0.068551357	0.188104702	0.298587518
ENSMUSG00000072244	Trim6	94088	protein_coding	102.3608969	-0.298646566	0.137850172	2.166457693	0.030276229	0.101663614	0.298646566
ENSMUSG00000048612	Myof	226101	protein_coding	692.9328978	0.298748849	0.093881553	-3.182189045	0.001461664	0.008692083	0.298748849
ENSMUSG00000041625	Ggact	223267	protein_coding	470.4285392	-0.298770069	0.06702156	4.457820288	8.28E-06	9.48E-05	0.298770069
ENSMUSG00000041540	Sox5	20678	protein_coding	8.254653456	0.298798081	0.545689065	-0.547561057	0.583993335	0.766436139	0.298798081
ENSMUSG00000027217	Tspan18	241556	protein_coding	7.936200751	-0.298806777	0.668470534	0.44700067	0.654874576	0.812734264	0.298806777
ENSMUSG00000058254	Tspan7	21912	protein_coding	613.4151732	-0.298894797	0.070841535	4.219202732	2.45E-05	0.00024793	0.298894797
ENSMUSG00000033287	Kctd17	72844	protein_coding	247.4441389	-0.298910656	0.15532482	1.924423003	0.054301583	0.158658236	0.298910656
ENSMUSG00000040896	Kcnd3	56543	protein_coding	10.70243785	-0.298957849	0.61602265	0.485303339	0.627461187	0.796333696	0.298957849

ensembl_gene_id	external_gene_name	entrezgene_id	gene_biotype	baseMean	log2FoldChange	lfcSE	stat	pvalue	padj	absLog2FC
ENSMUSG00000033706	Smyd5	232187	protein_coding	156.2788817	0.298996428	0.116162198	-2.573956357	0.010054295	0.042770901	0.298996428
ENSMUSG00000038255	Neurod2	18013	protein_coding	1.524772059	0.299073516	1.214382313	-0.246276245	0.805468397	1	0.299073516
ENSMUSG00000005237	Dnah2	327954	protein_coding	27.61773084	0.299090581	0.611290544	-0.489277291	0.624645396	0.794347303	0.299090581
ENSMUSG00000020674	Pxdn	69675	protein_coding	7.880933473	-0.299132342	0.660816195	0.452671022	0.650785649	0.810243834	0.299132342
ENSMUSG00000050310	Rictor	78757	protein_coding	605.4360134	-0.299151228	0.068120978	4.391469948	1.13E-05	0.000124365	0.299151228
ENSMUSG00000027168	Pax6	18508	protein_coding	12.89517148	-0.299231118	0.634986985	0.471239765	0.637469525	0.802408631	0.299231118
ENSMUSG00000050931	Sgms2	74442	protein_coding	835.6117263	-0.299281368	0.110084147	2.718660011	0.006554694	0.030176812	0.299281368
ENSMUSG00000078307	AI593442	330941	protein_coding	2.288712613	0.299323804	2.412708601	-0.124061316	0.901266727	1	0.299323804
ENSMUSG00000007613	Tgfbr1	21812	protein_coding	569.0541805	0.299333795	0.105058445	-2.849212131	0.004382765	0.021806863	0.299333795
ENSMUSG00000072774	Zfp951	626391	protein_coding	19.36301331	-0.29940898	0.356708408	0.839366197	0.401263847	0.622900809	0.29940898
ENSMUSG00000097191	Mm2pr	433966	lncRNA	0.991943658	-0.299410194	1.289060567	0.232270075	0.816328254	1	0.299410194
ENSMUSG00000036672	Cenpt	320394	protein_coding	91.97220197	0.299419714	0.126079381	-2.374850766	0.017556047	0.066267555	0.299419714
ENSMUSG00000024124	Prss30	30943	protein_coding	1663.590572	-0.299606637	0.213399445	1.403971022	0.160327479	0.344790726	0.299606637
ENSMUSG00000038000	Acd	497652	protein_coding	120.2061509	-0.299636472	0.101212664	2.960464231	0.003071758	0.016201536	0.299636472
ENSMUSG00000006494	Pdk1	228026	protein_coding	713.1242666	-0.299776199	0.134654272	2.226265792	0.025996383	0.090057536	0.299776199
ENSMUSG00000014313	Cox6c	12864	protein_coding	3353.840418	-0.299830428	0.109262551	2.744128031	0.006067187	0.028407578	0.299830428
ENSMUSG00000061455	Stx17	67727	protein_coding	454.674596	-0.299840374	0.090978996	3.295709848	0.000981734	0.006266853	0.299840374
ENSMUSG00000039492	Ccdc27	381580	protein_coding	1.224253683	-0.29986656	1.684759944	0.177987708	0.858732629	1	0.29986656
ENSMUSG00000036411	9530077C05Rik	68283	protein_coding	6.861403604	-0.299962366	0.433412948	0.692093689	0.488878495	0.696638145	0.299962366
ENSMUSG00000035399	Oser1	66680	protein_coding	128.8691533	-0.300040219	0.151235422	1.983928197	0.047263845	0.142911457	0.300040219
ENSMUSG00000047875	Gpr157	269604	protein_coding	22.81360963	-0.30009567	0.224117706	1.339009201	0.180567678	0.374374718	0.30009567
ENSMUSG00000004610	Etfb	110826	protein_coding	463.9506483	0.3001368	0.078917353	-3.803178759	0.000142851	0.001180112	0.3001368
ENSMUSG00000091183	Gm5141	380850	protein_coding	16.82076323	-0.3001414	0.302239664	0.993057614	0.320681878	0.541897747	0.3001414
ENSMUSG00000015126	Tsr3	68327	protein_coding	89.01849926	0.300167798	0.124066893	-2.419402889	0.015546011	0.060109302	0.300167798
ENSMUSG00000028412	Slc44a1	100434	protein_coding	4697.03714	-0.300187531	0.097648495	3.074164446	0.00211093	0.011857691	0.300187531
ENSMUSG00000041817	Fam169a	320557	protein_coding	5.941815034	0.30026026	0.451015131	-0.665743207	0.505575251	0.709532566	0.30026026
ENSMUSG00000041445	Mmrn2	105450	protein_coding	3.396282476	0.300269039	0.713657791	-0.420746529	0.673940181	1	0.300269039
ENSMUSG00000060938	Rpl26	19941	protein_coding	4155.911559	-0.300292945	0.112381813	2.672077767	0.007538318	0.03389483	0.300292945
ENSMUSG00000070709	Zfp974	73430	protein_coding	78.22390138	-0.300516691	0.141024737	2.130950197	0.033093245	0.108713319	0.300516691
ENSMUSG00000031452	1700029H14Rik	66501	protein_coding	3.810759855	0.300557464	0.570440484	-0.52688663	0.598272319	1	0.300557464
ENSMUSG00000004383	Large1	16795	protein_coding	159.1880848	-0.300581186	0.125341295	2.398101805	0.016480284	0.063028118	0.300581186
ENSMUSG00000032413	Rasa2	114713	protein_coding	609.0438241	-0.300635324	0.061337536	4.901327075	9.52E-07	1.32E-05	0.300635324
ENSMUSG00000053329	Gatd3a	28295	protein_coding	538.2661955	-0.300643179	0.083057	3.61972116	0.000294921	0.002244387	0.300643179
ENSMUSG00000107355	AI839979	100740	lncRNA	3.431780723	0.300773371	0.582703218	-0.516169058	0.605736339	1	0.300773371
ENSMUSG00000074227	Spint2	20733	protein_coding	6804.875222	-0.300778371	0.078528459	3.830183029	0.000128048	0.001074484	0.300778371
ENSMUSG00000039879	Heca	380629	protein_coding	736.4708239	-0.300806345	0.090609119	3.319824187	0.000900742	0.005830986	0.300806345
ENSMUSG00000051527	Usp29	57775	protein_coding	5.201539669	0.301110256	1.44786313	-0.20796873	0.835253384	0.91717364	0.301110256
ENSMUSG00000035875	AI182371	98870	protein_coding	8.157668239	-0.301134933	0.486688943	0.618742088	0.536086278	0.732131062	0.301134933
ENSMUSG00000026196	Bard1	12021	protein_coding	67.6023253	0.301161035	0.163308558	-1.844122798	0.065165246	0.181269925	0.301161035

ensembl_gene_id	external_gene_name	entrezgene_id	gene_biotype	baseMean	log2FoldChange	lfcSE	stat	pvalue	padj	absLog2FC
ENSMUSG00000024525	Impa2	114663	protein_coding	243.7186004	-0.301170544	0.116435345	2.586590382	0.009693075	0.041512301	0.301170544
ENSMUSG00000027953	Slc50a1	19729	protein_coding	417.2787955	-0.301172524	0.161442068	1.865514532	0.062109317	0.17492772	0.301172524
ENSMUSG00000038046	Mrm3	67390	protein_coding	86.39727467	-0.301197364	0.124731888	2.41475832	0.015745653	0.060682336	0.301197364
ENSMUSG00000054889	Dsp	109620	protein_coding	8770.832075	-0.301353385	0.100354313	3.002894196	0.002674254	0.014436949	0.301353385
ENSMUSG00000023007	Prpf40b	54614	protein_coding	130.2856517	-0.301362929	0.200398552	1.503817899	0.132628261	0.302183099	0.301362929
ENSMUSG00000000743	Chmp1a	234852	protein_coding	2211.975879	-0.301485374	0.084884122	3.55172873	0.000382709	0.00281989	0.301485374
ENSMUSG00000026042	Col5a2	12832	protein_coding	4.684123938	0.301595154	1.03789276	-0.290584119	0.771369407	0.885225461	0.301595154
ENSMUSG00000078713	Tomm5	68512	protein_coding	380.7257603	-0.301605186	0.109758332	2.747902414	0.005997786	0.028162478	0.301605186
ENSMUSG00000050824	Sstr5	20609	protein_coding	6.727346483	-0.301664632	0.464149034	0.64993054	0.51573709	0.716728293	0.301664632
ENSMUSG00000040624	Plekhg1	213783	protein_coding	242.8810111	-0.301791238	0.10294432	2.931596786	0.003372243	0.01753991	0.301791238
ENSMUSG00000054850	Smim10l2a	320237	protein_coding	31.80226361	0.301878233	0.279095736	-1.081629684	0.279417109	0.496825672	0.301878233
ENSMUSG00000068922	Msto1	229524	protein_coding	111.980296	-0.301880571	0.111984127	2.695744296	0.007023156	0.031968135	0.301880571
ENSMUSG00000032358	Fam83b	208994	protein_coding	483.0777262	-0.301958417	0.081276832	3.71518436	0.000203056	0.001601366	0.301958417
ENSMUSG00000022178	Ajuba	16475	protein_coding	61.63960021	0.301972293	0.153797898	-1.96343576	0.049595562	0.148051539	0.301972293
ENSMUSG00000024302	Dtna	13527	protein_coding	5.066544218	0.302033553	1.322524846	-0.228376468	0.819353582	0.908985828	0.302033553
ENSMUSG00000032120	C2cd2l	71764	protein_coding	709.8284277	0.30217044	0.120730196	-2.502857194	0.012319524	0.050060192	0.30217044
ENSMUSG00000031990	Jam3	83964	protein_coding	1.664061895	0.302182297	0.930924879	-0.324604384	0.745480508	1	0.302182297
ENSMUSG00000040658	Dnph1	381101	protein_coding	35.51102689	-0.302354623	0.211196947	1.431624026	0.15225145	0.332817843	0.302354623
ENSMUSG00000027452	Acss1	68738	protein_coding	1861.529848	0.302446943	0.088547376	-3.415651104	0.000636297	0.004334906	0.302446943
ENSMUSG00000027660	Skil	20482	protein_coding	1389.429241	0.302487539	0.115647151	-2.61560735	0.008906892	0.038748264	0.302487539
ENSMUSG00000023087	Noct	12457	protein_coding	411.9515873	-0.302504131	0.221853448	1.363531349	0.172715111	0.363275924	0.302504131
ENSMUSG00000039990	Edrf1	214764	protein_coding	346.7716783	0.30260664	0.08634719	-3.504533745	0.000457408	0.003288476	0.30260664
ENSMUSG00000049521	Cdc42ep1	104445	protein_coding	263.8802214	-0.302608899	0.128511652	2.354719549	0.01853669	0.069131639	0.302608899
ENSMUSG00000030750	Nsmce1	67711	protein_coding	218.4240825	0.302643268	0.098221732	-3.081225124	0.002061507	0.011638882	0.302643268
ENSMUSG00000001666	Ddt	13202	protein_coding	237.2367162	0.302655211	0.09780965	-3.094328739	0.001972588	0.011213534	0.302655211
ENSMUSG00000022829	Stxbp5l	207227	protein_coding	5.531905107	-0.302663943	0.772536509	0.391779469	0.695221166	0.838326567	0.302663943
ENSMUSG00000001054	Rmnd5b	66089	protein_coding	220.2758414	-0.302707093	0.107229871	2.822973585	0.004758049	0.02334356	0.302707093
ENSMUSG00000037251	Pomk	74653	protein_coding	147.5273132	-0.302711636	0.123447295	2.452152843	0.014200433	0.05587466	0.302711636
ENSMUSG00000021823	Vcl	22330	protein_coding	768.1632426	-0.302733326	0.078682503	3.847530435	0.000119314	0.001007326	0.302733326
ENSMUSG00000074513	Arfip1	99889	protein_coding	285.2762268	0.302735177	0.092759423	-3.263659559	0.001099832	0.006900696	0.302735177
ENSMUSG00000091705	H2-Q2	15013	protein_coding	1602.710067	0.302765899	0.148818892	-2.034458762	0.041905362	0.130398559	0.302765899
ENSMUSG00000039270	Megf9	230316	protein_coding	196.9933914	0.302775524	0.148549572	-2.038212027	0.041528733	0.129592876	0.302775524
ENSMUSG00000038195	Rilp	280408	protein_coding	156.3817357	-0.302840535	0.134540697	2.25092141	0.024390515	0.085735413	0.302840535
ENSMUSG00000026411	Tmem9	66241	protein_coding	113.0458281	-0.302877609	0.166187235	1.822508265	0.068377901	0.18782313	0.302877609
ENSMUSG00000048473	Sult6b2	330440	protein_coding	1.645984505	0.302994137	1.423901236	-0.21279154	0.831489563	1	0.302994137
ENSMUSG00000030279	C2cd5	74741	protein_coding	371.2575392	0.303018298	0.068969054	-4.393540005	1.12E-05	0.000123434	0.303018298
ENSMUSG00000026611	Spata17	74717	protein_coding	15.664591	-0.303048782	0.355402023	0.852692899	0.39382963	0.616464692	0.303048782
ENSMUSG00000010086	Rnf112	22671	protein_coding	2.874758228	0.303102066	0.844882606	-0.35875051	0.719781739	1	0.303102066
ENSMUSG00000025529	Zfp711	245595	protein_coding	28.45476812	0.303189728	0.236978775	-1.279396132	0.200757595	0.402050804	0.303189728

Supplementary Table 1

ensembl_gene_id	external_gene_name	entrezgene_id	gene_biotype	baseMean	log2FoldChange	lfcSE	stat	pvalue	padj	absLog2FC
ENSMUSG00000050199	Lgr4	107515	protein_coding	3417.034626	-0.303244413	0.113599952	2.669406167	0.00759855	0.034126314	0.303244413
ENSMUSG00000064141	Zfp69	381549	protein_coding	22.26014415	0.303302672	0.271147593	-1.118588842	0.263315586	0.477583041	0.303302672
ENSMUSG00000080932	Gm10224	100043805	processed_pseudogene	2.305757843	-0.303326585	0.841593908	0.360419179	0.718533686	1	0.303326585
ENSMUSG00000031779	Ccl22	20299	protein_coding	2.328077985	0.303356684	0.978022146	-0.310173635	0.756428919	1	0.303356684
ENSMUSG00000020273	Papolg	216578	protein_coding	204.2903423	-0.303362071	0.075223518	4.032808878	5.51E-05	0.000509035	0.303362071
ENSMUSG00000039585	Myo9a	270163	protein_coding	228.7526898	-0.303402546	0.126911956	2.390653773	0.016818405	0.064054228	0.303402546
ENSMUSG00000027011	Ube2e3	22193	protein_coding	341.2589165	0.303438357	0.095613694	-3.173586796	0.001505679	0.0089185	0.303438357
ENSMUSG00000085236	2610206C17Rik	72495	lncRNA	4.203006931	0.303483748	0.697651177	-0.435007864	0.663556719	1	0.303483748
ENSMUSG00000039067	Psmd7	17463	protein_coding	696.3359626	0.303489312	0.066440436	-4.567840488	4.93E-06	5.89E-05	0.303489312
ENSMUSG00000018907	Alox12e	11685	protein_coding	1.522378619	-0.303511924	2.073163792	0.146400359	0.883605344	1	0.303511924
ENSMUSG00000025980	Hspd1	15510	protein_coding	2851.908288	-0.303515744	0.141621772	2.143143241	0.032101598	0.106322863	0.303515744
ENSMUSG00000034936	Arl4d	80981	protein_coding	47.03801556	0.303662853	0.313165677	-0.969655602	0.332218188	0.554612255	0.303662853
ENSMUSG00000074622	Mafb	16658	protein_coding	154.7516673	0.303673614	0.286824964	-1.058741924	0.289717329	0.508545065	0.303673614
ENSMUSG00000063362	Alg11	207958	protein_coding	363.3534138	0.303740767	0.116594799	-2.605097045	0.009184824	0.039779225	0.303740767
ENSMUSG00000027412	Lpin3	64899	protein_coding	670.2651064	0.303753467	0.110186731	-2.756715478	0.005838513	0.027610883	0.303753467
ENSMUSG00000002015	Bcap31	27061	protein_coding	1447.602573	-0.303757459	0.090750836	3.347158805	0.000816444	0.00535797	0.303757459
ENSMUSG00000067567	Hdac8	70315	protein_coding	86.36797598	-0.303783708	0.125055822	2.429184844	0.015132815	0.058800778	0.303783708
ENSMUSG00000054052	Rdh19	216453	protein_coding	6.291819702	-0.303788079	0.501650618	0.605577005	0.544795676	0.736895388	0.303788079
ENSMUSG00000078350	Smim1	68859	protein_coding	8.871264425	0.303887818	0.367296832	-0.827363023	0.408031332	0.628624818	0.303887818
ENSMUSG00000009293	Ube2g2	22213	protein_coding	345.7386962	0.30391006	0.095414914	-3.185142098	0.001446829	0.008622498	0.30391006
ENSMUSG00000045062	Pcdhb7	93878	protein_coding	1.848662838	0.303946872	0.820171449	-0.370589432	0.710943355	1	0.303946872
ENSMUSG00000022314	Rad21	19357	protein_coding	1818.859854	0.303948725	0.099194453	-3.06417059	0.002182744	0.012153013	0.303948725
ENSMUSG00000036273	Lrrk2	66725	protein_coding	272.1380098	0.30395797	0.268459254	-1.132231299	0.257537205	0.470773677	0.30395797
ENSMUSG00000020087	Tysnd1	71767	protein_coding	389.3471629	0.303997092	0.09332747	-3.257316335	0.00112471	0.007027417	0.303997092
ENSMUSG00000092080	Vmn2r16	384220	protein_coding	1.882770371	-0.304010144	2.046527197	0.148549281	0.881909297	1	0.304010144
ENSMUSG00000035469	Rcbtb1	71330	protein_coding	680.5054487	0.304323442	0.117303543	-2.594324396	0.009477702	0.040790921	0.304323442
ENSMUSG00000042340	Ctf1	13019	protein_coding	20.75261484	-0.304457004	0.281407525	1.081907826	0.279293487	0.496688848	0.304457004
ENSMUSG00000051579	Tceal8	66684	protein_coding	233.4139916	0.304484462	0.146367508	-2.080273593	0.037500445	0.119750527	0.304484462
ENSMUSG00000006711	D130043K22Rik	210108	protein_coding	21.99730057	-0.30456319	0.298110294	1.021646003	0.306948476	0.527501415	0.30456319
ENSMUSG00000000440	Pparg	19016	protein_coding	1086.922247	-0.304584627	0.130342265	2.336806311	0.019449258	0.071853476	0.304584627
ENSMUSG00000026650	Meig1	104362	protein_coding	1.002649023	-0.304593136	1.156025873	0.263482975	0.792178344	1	0.304593136
ENSMUSG00000070315	4930581F22Rik	78934	lncRNA	49.22519084	-0.304772382	0.211963262	1.437854746	0.150475272	0.330401362	0.304772382
ENSMUSG00000022683	Pla2g10	26565	protein_coding	683.6162985	-0.304825594	0.149477248	2.03927753	0.041422337	0.129358637	0.304825594
ENSMUSG00000025357	Dgka	13139	protein_coding	1389.302797	-0.304870513	0.118196788	2.579346848	0.009898734	0.042239714	0.304870513
ENSMUSG00000027954	Efna1	13636	protein_coding	921.8314469	-0.304931311	0.137245557	2.221793689	0.026297251	0.090795126	0.304931311
ENSMUSG00000040957	Cables1	63955	protein_coding	375.6738166	-0.304936894	0.163068394	1.869993854	0.061484671	0.173816344	0.304936894
ENSMUSG00000039903	Eva1c	70967	protein_coding	10.21877705	-0.304980647	0.567343575	0.537559004	0.590881539	0.771232643	0.304980647
ENSMUSG00000033595	Lgi3	213469	protein_coding	1.733234576	-0.305007438	1.834323986	0.166277844	0.867938301	1	0.305007438
ENSMUSG00000073295	Nudt11	58242	protein_coding	17.38907589	0.305079298	0.291252086	-1.047475065	0.294880527	0.514056479	0.305079298

ensembl_gene_id	external_gene_name	entrezgene_id	gene_biotype	baseMean	log2FoldChange	lfcSE	stat	pvalue	padj	absLog2FC
ENSMUSG00000034761	Map4k5	399510	protein_coding	325.3416142	-0.305091141	0.076518043	3.987179107	6.69E-05	0.000603709	0.305091141
ENSMUSG00000071753	Cdr1os	331424	lncRNA	4.466318064	-0.305096388	3.14706773	0.096946241	0.922769086	0.962580298	0.305096388
ENSMUSG00000034993	Vat1	26949	protein_coding	533.601369	-0.305172211	0.100845574	3.026133896	0.002477026	0.013504657	0.305172211
ENSMUSG00000033174	Mgll	23945	protein_coding	788.4843876	0.305269977	0.213994853	-1.426529532	0.153715549	0.335154287	0.305269977
ENSMUSG00000022102	Dok2	13449	protein_coding	6.723452598	-0.305369322	0.58172254	0.524939814	0.599625031	0.776544903	0.305369322
ENSMUSG00000028125	Abca4	11304	protein_coding	4.108984253	0.305404623	1.701354723	-0.17950673	0.857539833	1	0.305404623
ENSMUSG00000079049	Serpinb1c	380839	protein_coding	2.149348383	-0.305508397	0.833859729	0.366378644	0.714082553	1	0.305508397
ENSMUSG00000027087	Itgav	16410	protein_coding	468.3685802	0.305510796	0.115701727	-2.640503336	0.008278298	0.036592026	0.305510796
ENSMUSG00000022226	Mcpt2	17225	protein_coding	3.958337632	-0.305526885	0.637949286	0.478920334	0.631995304	1	0.305526885
ENSMUSG00000038080	Kdm1b	218214	protein_coding	380.6362165	-0.3057676	0.111696474	2.737486577	0.006191065	0.028864834	0.3057676
ENSMUSG00000020805	Slc13a5	237831	protein_coding	1.940004603	0.305779722	1.522286502	-0.200868707	0.840801237	1	0.305779722
ENSMUSG00000025525	Apool	68117	protein_coding	317.6691265	-0.305893605	0.094164448	3.248504223	0.001160135	0.007207851	0.305893605
ENSMUSG00000040209	Zfp704	170753	protein_coding	823.5977352	0.30593439	0.124087545	-2.465472182	0.01368328	0.054292572	0.30593439
ENSMUSG00000057182	Scn3a	20269	protein_coding	28.12957521	-0.305998471	0.29306679	1.044125372	0.296427389	0.515610058	0.305998471
ENSMUSG00000118506	Cfap141	73545	protein_coding	4.221437255	0.306024165	0.57119495	-0.535761329	0.592123512	1	0.306024165
ENSMUSG00000031488	Rab11fip1	75767	protein_coding	2327.12458	-0.306073352	0.071518793	4.279621321	1.87E-05	0.000194686	0.306073352
ENSMUSG00000037434	Slc30a1	22782	protein_coding	764.8918148	0.306142437	0.108049927	-2.833342384	0.004606402	0.022714279	0.306142437
ENSMUSG00000023755	Rhebl1	69159	protein_coding	12.40027194	-0.306151179	0.356000323	0.859974444	0.38980313	0.612367665	0.306151179
ENSMUSG00000032479	Map4	17758	protein_coding	1117.13714	0.306155515	0.098840187	-3.097480132	0.001951735	0.011125629	0.306155515
ENSMUSG00000026773	Pfkfb3	170768	protein_coding	34.22727905	0.306242944	0.2180027	-1.404766753	0.160090649	0.344460843	0.306242944
ENSMUSG00000041491	Cep78	208518	protein_coding	90.23261706	0.30624331	0.180767061	-1.69413226	0.090240153	0.229089299	0.30624331
ENSMUSG00000024949	Sf1	22668	protein_coding	1365.569028	0.306321978	0.058692444	-5.219104148	1.80E-07	2.84E-06	0.306321978
ENSMUSG00000030226	Lmo3	109593	protein_coding	1.722639271	-0.30632302	2.391605225	0.128082602	0.898083604	1	0.30632302
ENSMUSG00000027297	Ltk	17005	protein_coding	14.15298958	-0.306331579	0.356600676	0.859032526	0.390322571	0.612497263	0.306331579
ENSMUSG00000061397	Krt79	223917	protein_coding	2.77448124	-0.306562632	0.811386716	0.37782555	0.705560192	1	0.306562632
ENSMUSG00000115338	Pnp	18950	protein_coding	271.3796399	-0.306579144	0.1049789	2.920388224	0.003495956	0.018083738	0.306579144
ENSMUSG00000026094	Stk17b	98267	protein_coding	774.4068002	-0.306580081	0.1621503	1.890715472	0.058662336	0.168010721	0.306580081
ENSMUSG00000028821	Syf2	68592	protein_coding	485.1075575	-0.306714575	0.084605615	3.625227194	0.000288708	0.002203196	0.306714575
ENSMUSG00000030020	Prickle2	243548	protein_coding	4.074078195	-0.306764784	1.027283496	0.298617456	0.765231945	1	0.306764784
ENSMUSG00000067995	Gtf2f2	68705	protein_coding	239.0662523	0.306771585	0.089102415	-3.442909887	0.000575491	0.003986434	0.306771585
ENSMUSG00000035762	Tmem161b	72745	protein_coding	250.5641697	0.306867181	0.087371329	-3.512218309	0.000444383	0.003214427	0.306867181
ENSMUSG00000074923	Pak6	214230	protein_coding	27.54455496	-0.306907482	0.223982013	1.370232719	0.170614268	0.360481579	0.306907482
ENSMUSG00000004151	Etv1	14009	protein_coding	10.7289061	-0.306928895	0.424755427	0.722601466	0.469924767	0.683489308	0.306928895
ENSMUSG00000028551	Cdkn2c	12580	protein_coding	138.779042	0.306931123	0.17800503	-1.724283427	0.08465669	0.219013029	0.306931123
ENSMUSG00000039478	Micu3	78506	protein_coding	16.50012273	0.306968967	0.305494536	-1.004826374	0.314980462	0.535990818	0.306968967
ENSMUSG00000025377	Tepsin	78777	protein_coding	376.1950276	0.306980166	0.087863032	-3.493849	0.00047611	0.003403673	0.306980166
ENSMUSG00000059713	Rcan3	53902	protein_coding	459.7019171	0.30703113	0.107079838	-2.867310383	0.004139768	0.02076044	0.30703113
ENSMUSG00000028004	Npy2r	18167	protein_coding	5.856195652	-0.307039448	0.51280471	0.598745373	0.549342695	0.740342018	0.307039448
ENSMUSG00000038059	Smim3	106878	protein_coding	55.97877006	0.30705683	0.255527499	-1.201658649	0.229495808	0.438111017	0.30705683

Supplementary Table 1

ensembl_gene_id	external_gene_name	entrezgene_id	gene_biotype	baseMean	log2FoldChange	lfcSE	stat	pvalue	padj	absLog2FC
ENSMUSG00000038301	Snx10	71982	protein_coding	122.4360142	-0.307094572	0.13997321	2.19395248	0.028238826	0.096124511	0.307094572
ENSMUSG00000001555	Fkbp10	14230	protein_coding	2.103093283	-0.307135473	1.063052967	0.288918315	0.772643887	1	0.307135473
ENSMUSG00000110605	Gm32856	102635552	lncRNA	25.11593868	0.307195774	0.220613695	-1.392460131	0.163783107	0.349988901	0.307195774
ENSMUSG00000003518	Dusp3	72349	protein_coding	322.3325746	-0.307201474	0.101144142	3.037264143	0.002387362	0.013089272	0.307201474
ENSMUSG00000031432	Prps1	19139	protein_coding	169.877847	0.307344335	0.1628163	-1.887675468	0.059069532	0.168971808	0.307344335
ENSMUSG00000028271	Gtf2b	229906	protein_coding	440.8867106	0.307405453	0.09300424	-3.305284296	0.000948801	0.006091952	0.307405453
ENSMUSG00000078763	Slfn1	20555	protein_coding	1.283975828	-0.30743757	1.479256954	0.207832432	0.835359809	1	0.30743757
ENSMUSG00000023345	Poc1a	70235	protein_coding	177.5815267	0.307441905	0.109812229	-2.799705535	0.005114924	0.024814295	0.307441905
ENSMUSG00000097368	Gm10390	100503427	lncRNA	12.5643057	-0.30761217	0.489236991	0.628759019	0.529506833	0.727166502	0.30761217
ENSMUSG00000113204	Gm46430	108168101	lncRNA	76.64037619	-0.307653642	0.186483675	1.649761794	0.098991666	0.245826823	0.307653642
ENSMUSG00000032198	Dock6	319899	protein_coding	356.1381211	0.307697431	0.198559452	-1.549648869	0.121225817	0.283960728	0.307697431
ENSMUSG00000022673	Mcm4	17217	protein_coding	334.979159	0.307698572	0.158725789	-1.938554366	0.052555625	0.154982783	0.307698572
ENSMUSG00000028936	Rpl22	19934	protein_coding	2181.722287	-0.30771121	0.108740271	2.829781523	0.00465798	0.022923163	0.30771121
ENSMUSG00000078921	Tgtp2	100039796	protein_coding	4.428257961	0.307740669	0.60482844	-0.508806546	0.610887831	0.784402584	0.307740669
ENSMUSG00000002458	Rgs19	56470	protein_coding	35.29536985	0.307855181	0.27593296	-1.115688323	0.264555571	0.479304239	0.307855181
ENSMUSG00000041779	Tram2	170829	protein_coding	22.89650644	0.307991391	0.315613695	-0.975849259	0.32913917	0.551537722	0.307991391
ENSMUSG00000027985	Lef1	16842	protein_coding	4.714976867	-0.308015273	0.97882764	0.314677744	0.753006344	0.8762082	0.308015273
ENSMUSG00000037300	Ttc13	234875	protein_coding	607.8674656	0.308060764	0.077733917	-3.963016084	7.40E-05	0.000661879	0.308060764
ENSMUSG00000084771	A230072E10Rik	331547	lncRNA	7.412120672	-0.308074459	0.403421799	0.763653475	0.445073773	0.662372535	0.308074459
ENSMUSG00000059970	Hspa2	15512	protein_coding	25.19448088	0.308259224	0.277656646	-1.110217343	0.266905381	0.48202925	0.308259224
ENSMUSG00000034557	Zfyve9	230597	protein_coding	415.1646789	-0.308385659	0.070958448	4.346003435	1.39E-05	0.000149154	0.308385659
ENSMUSG00000025648	Pfkfb4	270198	protein_coding	355.1117951	-0.308385675	0.13935447	2.212958614	0.026900499	0.09253023	0.308385675
ENSMUSG00000009585	Apobec3	80287	protein_coding	157.4644568	-0.308439635	0.205648923	1.499835884	0.133656919	0.303730613	0.308439635
ENSMUSG00000020258	Glyctk	235582	protein_coding	13.20524941	-0.308471618	0.278877598	1.106118315	0.268675312	0.483691716	0.308471618
ENSMUSG00000041859	Mcm3	17215	protein_coding	342.6520783	0.308704048	0.176161689	-1.75239038	0.079706705	0.209275846	0.308704048
ENSMUSG00000042046	Dstyk	213452	protein_coding	180.4671145	-0.3087111	0.091180658	3.385708194	0.000709948	0.004741185	0.3087111
ENSMUSG00000034591	Slc41a2	338365	protein_coding	283.8871807	0.30876327	0.165821388	-1.862023195	0.062599821	0.175901555	0.30876327
ENSMUSG00000085286	Ube4bos3	74310	lncRNA	3.963826637	0.308788257	0.814985005	-0.378888268	0.704770836	1	0.308788257
ENSMUSG00000036550	Cnot1	234594	protein_coding	3622.800164	0.308936937	0.064629434	-4.780127549	1.75E-06	2.29E-05	0.308936937
ENSMUSG00000023966	Rsph9	75564	protein_coding	10.98454688	-0.308983274	0.365850768	0.844560955	0.398356017	0.620277218	0.308983274
ENSMUSG00000024800	Rpp30	54364	protein_coding	74.03605533	0.309020721	0.14520619	-2.128151159	0.033324553	0.109351285	0.309020721
ENSMUSG00000043430	Psapl1	76943	protein_coding	3.595908902	-0.309243197	0.847804703	0.364757587	0.715292368	1	0.309243197
ENSMUSG00000024429	Gnl1	14670	protein_coding	397.2212928	-0.309436149	0.090853967	3.405862824	0.000659553	0.004469372	0.309436149
ENSMUSG00000031493	Ggn	243897	protein_coding	6.607679439	0.30944396	0.692874319	-0.446609077	0.655157341	0.812818663	0.30944396
ENSMUSG00000117284	Gm7072	631624	protein_coding	285.2379513	-0.309444036	0.126739554	2.441574296	0.014623379	0.05718458	0.309444036
ENSMUSG00000035776	Cd99l2	171486	protein_coding	284.7507908	-0.309623051	0.105490749	2.93507301	0.003334692	0.01737744	0.309623051
ENSMUSG00000047844	Bex4	406217	protein_coding	25.95833606	0.309644738	0.274337332	-1.128700695	0.259024116	0.472341459	0.309644738
ENSMUSG00000059878	Zfp422	67255	protein_coding	276.6760462	-0.309660704	0.091735778	3.375571804	0.000736625	0.004889317	0.309660704
ENSMUSG00000030867	Plk1	18817	protein_coding	655.5858751	0.309719941	0.14209232	-2.17970922	0.029279023	0.09897928	0.309719941

ensembl_gene_id	external_gene_name	entrezgene_id	gene_biotype	baseMean	log2FoldChange	lfcSE	stat	pvalue	padj	absLog2FC
ENSMUSG00000028874	Fgr	14191	protein_coding	19.58799669	0.309742593	0.307381439	-1.007681511	0.313607384	0.534874425	0.309742593
ENSMUSG00000027533	Fabp5	16592	protein_coding	41.15700856	-0.309765689	0.228650551	1.354755924	0.175495334	0.367316849	0.309765689
ENSMUSG00000050064	Zfp697	242109	protein_coding	57.85552946	-0.309785995	0.170030115	1.821947802	0.068462906	0.187931588	0.309785995
ENSMUSG00000088712	Gm22728	115490275	scaRNA	2.013300495	-0.309796186	0.81337812	0.380875977	0.703295279	1	0.309796186
ENSMUSG00000034210	Efcab14	230648	protein_coding	763.9784164	-0.309869821	0.085869889	3.608596949	0.000307857	0.002331022	0.309869821
ENSMUSG00000032688	Malt1	240354	protein_coding	364.5250924	0.309882321	0.080016037	-3.872752672	0.000107613	0.000919359	0.309882321
ENSMUSG00000025366	Esyt1	23943	protein_coding	584.9950417	0.309896286	0.103941387	-2.981452299	0.002868847	0.015327126	0.309896286
ENSMUSG00000034614	Pik3ip1	216505	protein_coding	27.30474844	-0.309987369	0.222710534	1.39188463	0.163957337	0.350225257	0.309987369
ENSMUSG00000051499	Zfp786	330301	protein_coding	21.28906884	-0.309988077	0.246636995	1.256859609	0.208804477	0.412069446	0.309988077
ENSMUSG00000003657	Calb2	12308	protein_coding	1.966699971	-0.310003909	2.088874513	0.148407148	0.882021459	1	0.310003909
ENSMUSG00000078963	Hsbp1l1	66255	protein_coding	33.39149787	0.310073751	0.208836437	-1.484768441	0.137605175	0.31001526	0.310073751
ENSMUSG00000037418	Best1	24115	protein_coding	17.48140812	0.310283159	0.667368472	-0.464935298	0.641977792	0.804573016	0.310283159
ENSMUSG00000002012	Pnck	93843	protein_coding	11.04187126	0.310589711	0.632155131	-0.491318817	0.623200973	0.793304622	0.310589711
ENSMUSG00000045100	Slc25a26	67582	protein_coding	74.84497139	-0.310610865	0.157196675	1.975937885	0.048161813	0.145029937	0.310610865
ENSMUSG00000048617	Rtbdn	234542	protein_coding	3.096937161	0.310700085	0.891771311	-0.348407805	0.727533942	1	0.310700085
ENSMUSG00000085333	1700030A11Rik	74179	lncRNA	20.83685856	-0.310736049	0.256752886	1.210253383	0.22618168	0.433917781	0.310736049
ENSMUSG00000021373	Cap2	67252	protein_coding	10.77192565	0.310901922	0.413950107	-0.751061339	0.452615741	0.668918592	0.310901922
ENSMUSG00000014353	Tmem87b	72477	protein_coding	1112.306228	-0.310902744	0.078397156	3.965740105	7.32E-05	0.000654908	0.310902744
ENSMUSG00000095432	Zfp748	212276	protein_coding	175.2934566	-0.310935421	0.135198922	2.299836531	0.021457483	0.077538047	0.310935421
ENSMUSG00000061286	Exosc5	27998	protein_coding	200.2961834	-0.31094203	0.108104173	2.876318474	0.004023435	0.020269375	0.31094203
ENSMUSG00000033249	Hsf4	26386	protein_coding	9.314780543	-0.310948473	0.366173751	0.849182858	0.39577955	0.618169251	0.310948473
ENSMUSG00000028062	Lamtor2	83409	protein_coding	490.8462374	-0.311051292	0.079975253	3.889344262	0.000100515	0.000864538	0.311051292
ENSMUSG00000026198	Abcb6	74104	protein_coding	65.67808401	-0.311077401	0.150156632	2.07168606	0.038294728	0.121849545	0.311077401
ENSMUSG00000052117	D630039A03Rik	242484	protein_coding	493.4986947	-0.311266161	0.204253152	1.523923415	0.127527844	0.294503539	0.311266161
ENSMUSG00000112343	Sfta3-ps	104798	lncRNA	2.400106364	0.311361547	2.37939655	-0.130857358	0.895888148	1	0.311361547
ENSMUSG00000047230	Cldn2	12738	protein_coding	2145.946905	0.311386101	0.213743712	-1.456819939	0.14516609	0.322338142	0.311386101
ENSMUSG00000040272	Accs	329470	protein_coding	48.1644694	-0.311444374	0.202805163	1.535682669	0.124616228	0.289400593	0.311444374
ENSMUSG00000030301	Ccdc91	67015	protein_coding	418.351977	-0.311548838	0.099638191	3.126801427	0.001767192	0.010218283	0.311548838
ENSMUSG00000097170	Gm16982	100036523	lncRNA	9.653436583	-0.311560839	0.378363101	0.823444035	0.410255531	0.630552252	0.311560839
ENSMUSG00000021198	Unc79	217843	protein_coding	24.84916131	-0.311658921	0.321194246	0.970312901	0.331890548	0.554292124	0.311658921
ENSMUSG00000050332	Amer1	72345	protein_coding	146.4391598	-0.311739941	0.130164329	2.394972143	0.016621628	0.063495157	0.311739941
ENSMUSG00000030446	Zfp273	212569	protein_coding	47.29763679	-0.311783975	0.16080382	1.938909005	0.052512419	0.154934252	0.311783975
ENSMUSG00000011257	Pabpc4	230721	protein_coding	720.5894427	-0.311887456	0.101329003	3.077968277	0.002084171	0.01173204	0.311887456
ENSMUSG00000095440	Fignl2	668225	protein_coding	39.49388611	-0.312150931	0.27696934	1.127023414	0.259732582	0.473163139	0.312150931
ENSMUSG00000072972	Adam4	11498	protein_coding	12.0033713	0.312326449	0.322493431	-0.968473831	0.332807782	0.555206356	0.312326449
ENSMUSG00000019188	H13	14950	protein_coding	1292.964796	0.312398462	0.085042653	-3.673432703	0.000239314	0.001854541	0.312398462
ENSMUSG00000023110	Prmt5	27374	protein_coding	485.3655788	0.312504328	0.124398094	-2.512131164	0.012000448	0.049101931	0.312504328
ENSMUSG00000026380	Tfcp2l1	81879	protein_coding	6841.680259	-0.312557686	0.102254369	3.056668265	0.002238118	0.01240278	0.312557686
ENSMUSG00000036502	Tmem255a	245386	protein_coding	4.159201623	0.312567196	0.805662118	-0.387963129	0.698043319	1	0.312567196

ensembl_gene_id	external_gene_name	entrezgene_id	gene_biotype	baseMean	log2FoldChange	lfcSE	stat	pvalue	padj	absLog2FC
ENSMUSG00000031360	Ctps2	55936	protein_coding	278.3985948	-0.312668638	0.119473295	2.617058791	0.008869106	0.038648666	0.312668638
ENSMUSG00000028318	Polr1e	64424	protein_coding	102.2777721	0.312674405	0.126724215	-2.467361154	0.0136113	0.054110978	0.312674405
ENSMUSG00000019080	Mfsd3	69572	protein_coding	50.29048862	-0.312790291	0.160309386	1.951166417	0.051037252	0.151424575	0.312790291
ENSMUSG00000028999	Rint1	72772	protein_coding	328.9620044	0.312852097	0.093853571	-3.333406422	0.000857895	0.005597504	0.312852097
ENSMUSG00000079110	Capn3	12335	protein_coding	8.826591099	0.312889127	0.574192926	-0.544919858	0.585808638	0.767554399	0.312889127
ENSMUSG00000059142	Zfp945	240041	protein_coding	109.4464929	-0.312918986	0.165060185	1.895787192	0.05798819	0.166571051	0.312918986
ENSMUSG00000054978	Kbtbd13	74492	protein_coding	32.69638902	0.312956657	0.214351098	-1.460018912	0.144284876	0.321034822	0.312956657
ENSMUSG00000000184	Ccnd2	12444	protein_coding	2225.177164	-0.313018104	0.168398539	1.858793468	0.063056419	0.176840786	0.313018104
ENSMUSG00000029830	Svopl	320590	protein_coding	3.701174892	0.313177278	1.102798485	-0.283984139	0.776422536	1	0.313177278
ENSMUSG00000021708	Rasgrf2	19418	protein_coding	122.4727549	0.313194295	0.140496722	-2.229192898	0.025801073	0.089531187	0.313194295
ENSMUSG00000017376	Nlk	18099	protein_coding	206.0390343	0.313197746	0.105116092	-2.97954139	0.002886802	0.015398144	0.313197746
ENSMUSG00000018570	2810408A11Rik	70419	protein_coding	59.26404825	-0.313259287	0.206362243	1.518006793	0.129012686	0.296522686	0.313259287
ENSMUSG00000020629	Adi1	104923	protein_coding	170.5638355	-0.313278699	0.137324888	2.281295859	0.022530946	0.080642929	0.313278699
ENSMUSG00000042826	Fgf11	14166	protein_coding	38.68027627	0.313298631	0.296870283	-1.055338473	0.291270552	0.510066755	0.313298631
ENSMUSG00000020495	Smg8	74133	protein_coding	189.2580013	0.313318037	0.097005057	-3.229914485	0.001238272	0.007596149	0.313318037
ENSMUSG00000027684	Mecom	14013	protein_coding	1993.493236	-0.313331381	0.098807697	3.171123215	0.001518507	0.0089816	0.313331381
ENSMUSG00000031490	Eif4ebp1	13685	protein_coding	172.3207177	0.313514057	0.113568312	-2.760576884	0.005769938	0.027372809	0.313514057
ENSMUSG00000029547	Ints1	68510	protein_coding	966.4975283	0.313551424	0.078254774	-4.006802483	6.15E-05	0.000562777	0.313551424
ENSMUSG00000026525	Opn3	13603	protein_coding	12.31463748	0.313553905	0.289627381	-1.0826114	0.278980945	0.496293367	0.313553905
ENSMUSG00000021916	Glt8d1	76485	protein_coding	96.95076436	0.313578353	0.133718409	-2.345064943	0.019023766	0.070613276	0.313578353
ENSMUSG00000039639	Kcne1	16509	protein_coding	1.678192659	0.313603331	1.819368007	-0.172369378	0.863147145	1	0.313603331
ENSMUSG00000117602	9130209A04Rik	77668	lncRNA	30.89964646	-0.313612729	0.360731001	0.869381141	0.384638695	0.607019295	0.313612729
ENSMUSG00000031520	Vegfc	22341	protein_coding	46.43083456	0.31363143	0.175893803	-1.783072652	0.074574477	0.199760409	0.31363143
ENSMUSG00000042737	Dpm3	68563	protein_coding	227.3283986	-0.313758526	0.096868278	3.239022451	0.001199401	0.007393398	0.313758526
ENSMUSG00000034522	Zfp395	380912	protein_coding	117.6602315	-0.313822958	0.136693479	2.295815132	0.021686454	0.078262696	0.313822958
ENSMUSG00000052337	Immt	76614	protein_coding	3455.618749	0.313946739	0.064640777	-4.856790959	1.19E-06	1.62E-05	0.313946739
ENSMUSG00000026003	Acadl	11363	protein_coding	1224.472796	-0.314209249	0.083201437	3.776488255	0.000159055	0.00129073	0.314209249
ENSMUSG00000098269	Mir8094	102465886	miRNA	1.4427443	0.31428815	0.985263064	-0.318989071	0.7497348	1	0.31428815
ENSMUSG00000050973	Gdpgp1	269952	protein_coding	154.439607	-0.314341631	0.119083636	2.63967108	0.008298653	0.036642767	0.314341631
ENSMUSG00000043432	Leng9	243813	protein_coding	662.0572498	0.314348943	0.104220977	-3.016177276	0.002559836	0.013891924	0.314348943
ENSMUSG00000110949	Nudt8	66387	protein_coding	2.264023428	0.314357973	0.654203804	-0.48051994	0.630857728	1	0.314357973
ENSMUSG00000023073	Slc10a2	20494	protein_coding	884.0612118	-0.314369667	0.289385225	1.086336275	0.277330244	0.494297255	0.314369667
ENSMUSG00000002661	Alkbh7	66400	protein_coding	180.1184416	-0.31439002	0.114957934	2.734826624	0.006241314	0.028986639	0.31439002
ENSMUSG00000037679	Inf2	70435	protein_coding	199.1891956	-0.314456481	0.16779994	1.873996379	0.060930924	0.172799808	0.314456481
ENSMUSG00000021360	Gcnt2	14538	protein_coding	118.27705	-0.314471705	0.299356521	1.050492251	0.293491852	0.512663892	0.314471705
ENSMUSG00000034686	Prr7	432763	protein_coding	6.982176276	-0.314512755	0.455711336	0.690157848	0.490094928	0.697829595	0.314512755
ENSMUSG00000047155	Cyp4x1	81906	protein_coding	5.900181616	-0.314601322	0.741687331	0.424169739	0.671442028	0.823849409	0.314601322
ENSMUSG00000064823	Snord82	80828	snoRNA	9.073406557	0.314654047	0.408154369	-0.770919217	0.440754816	0.658639492	0.314654047
ENSMUSG00000017868	Sgk2	27219	protein_coding	623.6600271	0.314714149	0.25421722	-1.23797337	0.215725935	0.420949793	0.314714149

Supplementary Table 1

ensembl_gene_id	external_gene_name	entrezgene_id	gene_biotype	baseMean	log2FoldChange	lfcSE	stat	pvalue	padj	absLog2FC
ENSMUSG00000030960	Eef1akmt2	72096	protein_coding	136.4301531	0.314774741	0.097070494	-3.242743781	0.001183846	0.00731937	0.314774741
ENSMUSG00000044430	Klk12	69511	protein_coding	1.742873645	0.314797527	2.719977863	-0.115735327	0.907862308	1	0.314797527
ENSMUSG00000028803	Nipal3	74552	protein_coding	1283.69388	0.314799236	0.054895266	-5.734542482	9.78E-09	1.88E-07	0.314799236
ENSMUSG00000037432	Fer1l5	100534273	protein_coding	40.33816458	0.314838993	0.246930281	-1.275011681	0.202305108	0.404218578	0.314838993
ENSMUSG00000047044	D030056L22Rik	225995	protein_coding	166.9608603	0.314871008	0.146328908	-2.151803177	0.03141286	0.104544934	0.314871008
ENSMUSG00000031107	Rbmx2	209003	protein_coding	42.03037357	0.314873842	0.200356923	-1.571564571	0.116051568	0.275039552	0.314873842
ENSMUSG00000117406	Ntn3	18209	protein_coding	3.962712511	-0.314894761	0.861978746	0.365316155	0.714875421	1	0.314894761
ENSMUSG00000074825	Itpripl1	73338	protein_coding	50.56958869	0.314913388	0.149688473	-2.103791842	0.035396609	0.114525783	0.314913388
ENSMUSG00000075028	Prdm11	100042784	protein_coding	23.62410191	0.314915263	0.294088508	-1.070817982	0.28425128	0.502676953	0.314915263
ENSMUSG00000086598	Btbd18	100270744	protein_coding	2.442160673	-0.314996763	0.91753819	0.343306433	0.731367942	1	0.314996763
ENSMUSG00000020431	Adcy1	432530	protein_coding	7.674253062	-0.315069784	0.694159196	0.453886927	0.649910213	0.809917796	0.315069784
ENSMUSG00000024248	Cox7a2l	20463	protein_coding	1933.402661	-0.315219251	0.108787199	2.897576677	0.003760578	0.01920773	0.315219251
ENSMUSG00000071068	Treml2	328833	protein_coding	5.409347774	0.315226323	1.07792604	-0.292437803	0.769951908	0.884582288	0.315226323
ENSMUSG00000079083	Jrkl	77532	protein_coding	131.1730597	-0.315356696	0.107762854	2.926395182	0.00342915	0.017779903	0.315356696
ENSMUSG00000066704	Cyp2b19	13090	protein_coding	3.382391211	-0.31541726	1.812520559	0.174021342	0.86184869	1	0.31541726
ENSMUSG00000024682	Cblif	14603	protein_coding	0.769826449	-0.315448324	1.689041225	0.186761767	0.851847425	1	0.315448324
ENSMUSG00000048647	Exd1	241624	protein_coding	26.95258218	0.315472773	0.238187404	-1.324472946	0.185346022	0.380697042	0.315472773
ENSMUSG00000061650	Med9	192191	protein_coding	231.0019456	0.315497312	0.110776675	-2.848048214	0.004398826	0.021880184	0.315497312
ENSMUSG00000073155	1810058I24Rik	67705	protein_coding	117.1544906	-0.3155147	0.131520963	2.398968905	0.016441311	0.062908204	0.3155147
ENSMUSG00000025289	Prdx4	53381	protein_coding	241.571296	0.315604037	0.173179624	-1.822408605	0.06839301	0.187833389	0.315604037
ENSMUSG00000086446	Prkag2os1	71161	lncRNA	5.27118103	0.315642962	0.536397215	-0.588450038	0.55623026	0.745631404	0.315642962
ENSMUSG00000027076	Timm10	30059	protein_coding	166.0803308	-0.315644232	0.116129736	2.718031075	0.006567167	0.030217385	0.315644232
ENSMUSG00000038751	Ptk6	20459	protein_coding	345.569062	0.315779698	0.227786422	-1.386297285	0.165656145	0.352762472	0.315779698
ENSMUSG00000027071	P2rx3	228139	protein_coding	6.661052192	0.315866511	0.505051049	-0.625415019	0.531698708	0.728669914	0.315866511
ENSMUSG00000027186	Elf5	13711	protein_coding	3.146927862	-0.315896703	1.563945062	0.201987084	0.839926821	1	0.315896703
ENSMUSG00000021226	Acot2	171210	protein_coding	85.51475551	-0.316007831	0.132337438	2.387894424	0.016945209	0.064388674	0.316007831
ENSMUSG00000033538	Casp4	12363	protein_coding	271.2059602	0.316025709	0.199216237	-1.586345136	0.112661039	0.269245209	0.316025709
ENSMUSG00000041124	Msantd4	78100	protein_coding	518.0671926	0.316058857	0.058261324	-5.424848515	5.80E-08	9.81E-07	0.316058857
ENSMUSG00000047150	1700001C19Rik	75462	protein_coding	5.270014231	-0.316074301	0.61046837	0.517757047	0.60462779	0.779942456	0.316074301
ENSMUSG00000048550	Thnsl1	208967	protein_coding	97.15261463	-0.316326043	0.114704765	2.757741084	0.005820228	0.027548077	0.316326043
ENSMUSG00000061983	Rps12	20042	protein_coding	5463.873793	-0.316378833	0.105269572	3.005415786	0.00265218	0.014342673	0.316378833
ENSMUSG00000025644	Gm7628	115487215	lncRNA	3.168498646	0.316423756	0.82794387	-0.382180202	0.702327706	1	0.316423756
ENSMUSG00000028865	Cd164l2	69655	protein_coding	7.834879444	-0.316493183	0.514402495	0.615263701	0.538380585	0.733377497	0.316493183
ENSMUSG00000094483	Purb	19291	protein_coding	2923.262862	0.316513189	0.110871746	-2.854768705	0.004306818	0.021461314	0.316513189
ENSMUSG00000019845	Tube1	71924	protein_coding	45.88777051	0.316607861	0.193836406	-1.633376657	0.102389803	0.251954286	0.316607861
ENSMUSG00000026278	Bok	51800	protein_coding	162.0690791	-0.316684385	0.110024969	2.8782956	0.003998303	0.020161214	0.316684385
ENSMUSG00000022455	Wbp2nl	74716	protein_coding	4.600310878	-0.316758321	0.541070792	0.585428609	0.558259557	0.747162726	0.316758321
ENSMUSG00000085795	Zfp703	353310	protein_coding	169.6751107	0.316840867	0.226203897	-1.400687038	0.16130768	0.346267391	0.316840867
ENSMUSG00000036944	Tmem71	213068	protein_coding	5.249241061	0.316843357	0.539401509	-0.587397979	0.556936451	0.746166522	0.316843357

ensembl_gene_id	external_gene_name	entrezgene_id	gene_biotype	baseMean	log2FoldChange	lfcSE	stat	pvalue	padj	absLog2FC
ENSMUSG00000022240	Ctnnd2	18163	protein_coding	6.590599064	0.316860209	0.749718085	-0.422639143	0.672558563	0.824518194	0.316860209
ENSMUSG00000088695	Gm22334	115486131	snoRNA	0.958428519	0.316869955	1.604605319	-0.197475324	0.84345559	1	0.316869955
ENSMUSG00000075470	Alg10b	380959	protein_coding	798.3295329	0.316904107	0.100480258	-3.153894235	0.001611075	0.009451256	0.316904107
ENSMUSG00000049090	Zadh2	225791	protein_coding	435.9917053	-0.316944001	0.084800174	3.737539502	0.00018583	0.001488577	0.316944001
ENSMUSG00000022550	Adck5	268822	protein_coding	775.8290033	-0.317158277	0.095654076	3.315679677	0.000914206	0.00589734	0.317158277
ENSMUSG00000033781	Asb13	142688	protein_coding	167.0950045	-0.31717792	0.114871452	2.761155307	0.005759728	0.027332226	0.31717792
ENSMUSG00000037894	H2az1	51788	protein_coding	1235.160633	0.317482039	0.152579953	-2.080758532	0.037456014	0.119688198	0.317482039
ENSMUSG00000037977	6430571L13Rik	235599	protein_coding	3.575455706	0.317483112	0.6580804	-0.482438182	0.629494703	1	0.317483112
ENSMUSG00000020042	Btbd11	74007	protein_coding	19.4818469	0.317565868	0.350383244	-0.906338626	0.364756647	0.587266381	0.317565868
ENSMUSG00000025329	Padi1	18599	protein_coding	0.80691154	0.317630035	1.445676598	-0.219710297	0.826096784	1	0.317630035
ENSMUSG00000021850	ccdc198	67082	protein_coding	82.96260562	-0.317645901	0.152697557	2.080229093	0.037504525	0.119750527	0.317645901
ENSMUSG00000055737	Ghr	14600	protein_coding	185.503506	-0.317715381	0.144467207	2.199221462	0.027862179	0.095110269	0.317715381
ENSMUSG00000097842	9330104G04Rik	320950	lncRNA	6.875550342	0.317761913	0.486625351	-0.652990873	0.513762163	0.715609679	0.317761913
ENSMUSG00000051695	Pcbp1	23983	protein_coding	1361.272616	0.317810273	0.083967136	-3.784936444	0.000153748	0.001253823	0.317810273
ENSMUSG00000042540	Acot5	217698	protein_coding	2.77379508	-0.317817493	0.743642841	0.427379214	0.669103144	1	0.317817493
ENSMUSG00000029394	Cdk2ap1	13445	protein_coding	146.016263	-0.317892845	0.188964572	1.68228807	0.092512993	0.233281352	0.317892845
ENSMUSG00000044442	N6amt1	67768	protein_coding	100.5326031	-0.317973672	0.115126597	2.761947985	0.005745763	0.027285164	0.317973672
ENSMUSG00000060036	Rpl3	27367	protein_coding	6307.52096	-0.318056981	0.128615927	2.472920643	0.01340139	0.053482492	0.318056981
ENSMUSG00000052214	Opa3	403187	protein_coding	954.6977226	0.318188124	0.056636111	-5.618113876	1.93E-08	3.55E-07	0.318188124
ENSMUSG00000023781	Hes7	84653	protein_coding	2.332024242	0.318205469	0.798904598	-0.398302213	0.69040743	1	0.318205469
ENSMUSG00000039942	Ptger4	19219	protein_coding	439.0006206	-0.318260173	0.115774951	2.748955365	0.005978553	0.028104133	0.318260173
ENSMUSG00000100733	4932411K12Rik	71174	lncRNA	1.005729404	-0.318431978	1.731783574	0.183875158	0.854111394	1	0.318431978
ENSMUSG00000040432	Ltb4r2	57260	protein_coding	10.93280527	0.318565136	0.479228019	-0.664746474	0.506212664	0.710135685	0.318565136
ENSMUSG00000028420	Tmem38b	52076	protein_coding	117.0611175	-0.318597051	0.137437484	2.31812343	0.020442614	0.074638365	0.318597051
ENSMUSG00000026788	Zbtb43	71834	protein_coding	449.4241456	-0.318631918	0.072078343	4.420633263	9.84E-06	0.000110481	0.318631918
ENSMUSG00000031563	Wwc2	52357	protein_coding	272.4394587	-0.318651869	0.112288443	2.837797545	0.004542599	0.022466748	0.318651869
ENSMUSG00000070034	Sp110	109032	protein_coding	41.33612642	-0.318664831	0.729721555	0.436693734	0.662333474	0.817346435	0.318664831
ENSMUSG00000045045	Lrfn4	225875	protein_coding	323.2355971	-0.318739351	0.106941141	2.980511989	0.00287767	0.015364319	0.318739351
ENSMUSG00000028223	Decr1	67460	protein_coding	1011.458846	-0.318755893	0.103481447	3.080319237	0.002067788	0.011662382	0.318755893
ENSMUSG00000029098	Acox3	80911	protein_coding	438.1112908	-0.318775112	0.074871636	4.257621863	2.07E-05	0.00021219	0.318775112
ENSMUSG00000032366	Tpm1	22003	protein_coding	2775.032069	-0.31878597	0.063740356	5.001320793	5.69E-07	8.30E-06	0.31878597
ENSMUSG00000089917	Uckl1	68556	protein_coding	405.8772084	-0.318791195	0.066727396	4.777515913	1.77E-06	2.32E-05	0.318791195
ENSMUSG00000041632	Mrps27	218506	protein_coding	277.6087768	0.318859296	0.099888147	-3.192163479	0.001412114	0.008482957	0.318859296
ENSMUSG00000014599	Csf1	12977	protein_coding	11.97825786	0.318879481	0.58567435	-0.544465505	0.58612118	0.767773712	0.318879481
ENSMUSG00000025429	Pstpip2	19201	protein_coding	130.8540548	-0.318972698	0.112716017	2.829879073	0.00465656	0.022923163	0.318972698
ENSMUSG00000009687	Fxyd5	18301	protein_coding	10.3317659	0.318983918	0.703318245	-0.453541367	0.650158963	0.809917796	0.318983918
ENSMUSG00000071604	Entrep1	381217	protein_coding	542.1517923	0.319008233	0.114928701	-2.775705541	0.005508207	0.026350675	0.319008233
ENSMUSG00000060244	Alyref2	56009	protein_coding	26.67509324	-0.319019698	0.197553066	1.614855713	0.106341945	0.258825479	0.319019698
ENSMUSG00000075524	4930407I10Rik	328573	protein_coding	2.919992514	0.31932635	2.371393359	-0.134657689	0.892882528	1	0.31932635

ensembl_gene_id	external_gene_name	entrezgene_id	gene_biotype	baseMean	log2FoldChange	lfcSE	stat	pvalue	padj	absLog2FC
ENSMUSG00000045404	Kcnk13	217826	protein_coding	1.011936034	0.319385866	1.620814049	-0.19705275	0.843786258	1	0.319385866
ENSMUSG00000039163	Cmc1	67899	protein_coding	202.4417651	-0.319493567	0.110770996	2.884270958	0.00392321	0.019904115	0.319493567
ENSMUSG00000028520	4921539E11Rik	70941	protein_coding	1.392657679	-0.319545924	1.646079373	0.194125465	0.846077642	1	0.319545924
ENSMUSG00000057137	Tmem140	68487	protein_coding	42.23697594	-0.319803166	0.405531766	0.78860201	0.430344656	0.648871201	0.319803166
ENSMUSG00000052751	Repin1	58887	protein_coding	226.623741	-0.319923343	0.180044016	1.776917385	0.075581834	0.20154342	0.319923343
ENSMUSG00000079677	Fdx2	68165	protein_coding	1.145932549	-0.319990589	1.147122146	0.278950755	0.780282617	1	0.319990589
ENSMUSG00000094500	Smim18	72632	protein_coding	1.07568703	-0.320127289	1.138746055	0.281122633	0.778616342	1	0.320127289
ENSMUSG00000007338	Mrpl49	18120	protein_coding	371.4628082	-0.320212182	0.077614738	4.125662086	3.70E-05	0.000358259	0.320212182
ENSMUSG00000031320	Rps4x	20102	protein_coding	6713.270678	-0.3202655	0.128575431	2.490876341	0.012742846	0.051375821	0.3202655
ENSMUSG00000029530	Ccr9	12769	protein_coding	10.49062579	-0.320306317	0.575861349	0.556221245	0.578059617	0.76182422	0.320306317
ENSMUSG00000029456	Acad10	71985	protein_coding	195.5872024	-0.320425756	0.106856204	2.998663106	0.00271167	0.014624596	0.320425756
ENSMUSG00000012422	Tmem167	66074	protein_coding	680.2606148	0.320463659	0.102914454	-3.113883867	0.001846422	0.010616924	0.320463659
ENSMUSG00000025977	Boll	75388	protein_coding	2.980627632	-0.320578684	0.738419087	0.434141925	0.664185382	1	0.320578684
ENSMUSG00000039128	Cdc123	98828	protein_coding	632.4841308	0.320629917	0.093316214	-3.435950772	0.000590478	0.004076571	0.320629917
ENSMUSG00000093606	B130034C11Rik	414095	lncRNA	9.188327546	0.32063232	0.340120781	-0.942701354	0.345833681	0.567534275	0.32063232
ENSMUSG00000017715	Pgs1	74451	protein_coding	551.9821958	0.320663934	0.093428374	-3.432190023	0.000598728	0.004121541	0.320663934
ENSMUSG00000035811	Ugt2b35	243085	protein_coding	498.452295	0.320715253	0.125032455	-2.565056041	0.010315914	0.043636528	0.320715253
ENSMUSG00000085810	Gm16325	100286843	lncRNA	1.354338746	0.320731476	0.935968905	-0.342673218	0.731844313	1	0.320731476
ENSMUSG00000006777	Krt23	94179	protein_coding	116.0193685	0.320807655	0.157552603	-2.036193937	0.041730885	0.130002609	0.320807655
ENSMUSG00000021208	Ifi27l2b	217845	protein_coding	1312.821643	-0.320873157	0.175733944	1.82590312	0.067864856	0.18675558	0.320873157
ENSMUSG00000052221	Ppp1r36	210762	protein_coding	1.650178958	-0.321008244	1.524124651	0.210618104	0.833185284	1	0.321008244
ENSMUSG00000040354	Mars1	216443	protein_coding	479.9886973	0.321042605	0.123170311	-2.606493422	0.009147458	0.039648589	0.321042605
ENSMUSG00000033429	Mcee	73724	protein_coding	311.2222834	-0.32104658	0.102276102	3.139018544	0.001695147	0.009870825	0.32104658
ENSMUSG00000040570	Rundc3b	242819	protein_coding	314.9085561	-0.321051475	0.084623797	3.793867525	0.000148319	0.001217364	0.321051475
ENSMUSG00000087175	Gm15133	622070	lncRNA	14.67817255	-0.321075954	0.389002375	0.825383018	0.409154173	0.629730716	0.321075954
ENSMUSG00000116848	4930542D17Rik	75211	lncRNA	1.924505704	0.321126796	2.372065424	-0.135378558	0.892312577	1	0.321126796
ENSMUSG00000077689	Gm22499	115485942	snoRNA	0.519056765	0.32125799	1.52620921	-0.210494071	0.833282079	1	0.32125799
ENSMUSG00000051705	Senp8	71599	protein_coding	75.12571387	-0.32128484	0.161121171	1.994057254	0.046145798	0.140315174	0.32128484
ENSMUSG00000038893	Fam117a	215512	protein_coding	79.03766668	-0.321491147	0.175189819	1.835101771	0.066490612	0.184012672	0.321491147
ENSMUSG00000029438	Bcl7a	77045	protein_coding	102.4724472	-0.321494965	0.142327741	2.258835572	0.023893615	0.084361596	0.321494965
ENSMUSG00000090862	Rps13	68052	protein_coding	2231.644036	-0.321575563	0.131667913	2.442322923	0.014593087	0.057079639	0.321575563
ENSMUSG00000003464	Pex19	19298	protein_coding	809.3313464	-0.32159642	0.057380563	5.604622951	2.09E-08	3.83E-07	0.32159642
ENSMUSG00000051212	Gpr183	321019	protein_coding	13.7658368	0.321644595	0.367302079	-0.875695002	0.381195863	0.603611898	0.321644595
ENSMUSG00000035559	Mpv17l2	234384	protein_coding	391.3319125	0.321776233	0.091684304	-3.509610896	0.000448763	0.003236188	0.321776233
ENSMUSG00000025816	Sec61a2	57743	protein_coding	115.167796	0.321779516	0.13339201	-2.412284777	0.015852894	0.061038631	0.321779516
ENSMUSG00000036880	Acaa2	52538	protein_coding	1533.572137	-0.321901941	0.067863225	4.74339293	2.10E-06	2.73E-05	0.321901941
ENSMUSG00000027160	Ccdc34	68201	protein_coding	238.2078044	0.322103617	0.142349176	-2.262771204	0.023649797	0.083737724	0.322103617
ENSMUSG00000000093	Tbx2	21385	protein_coding	2.79133405	0.322110222	0.827415446	-0.389296844	0.697056571	1	0.322110222
ENSMUSG00000010051	Hyal1	15586	protein_coding	24.0130484	0.322510783	0.276498455	-1.166410797	0.243448395	0.454734397	0.322510783

Supplementary Table 1

ensembl_gene_id	external_gene_name	entrezgene_id	gene_biotype	baseMean	log2FoldChange	lfcSE	stat	pvalue	padj	absLog2FC
ENSMUSG00000021520	Uqcrb	67530	protein_coding	2463.672785	-0.322614932	0.092620161	3.48320417	0.00049545	0.003520597	0.322614932
ENSMUSG00000052749	Trim30b	244183	protein_coding	3.599797518	0.322614971	0.906399401	-0.355930257	0.721892796	1	0.322614971
ENSMUSG00000037341	Slc9a7	236727	protein_coding	11.5650912	0.322690791	0.452345266	-0.71337276	0.475615125	0.687587163	0.322690791
ENSMUSG00000115852	Gm52969	102633938	transcribed_processed_pseudogene	2.145470673	0.322707696	1.40427879	-0.229803154	0.818244733	1	0.322707696
ENSMUSG00000020120	Plek	56193	protein_coding	37.29012428	0.322841297	0.215664374	-1.496961649	0.134403237	0.305048799	0.322841297
ENSMUSG00000051455	Meioc	268491	protein_coding	2.684008742	0.322939255	0.732457581	-0.440898236	0.659286671	1	0.322939255
ENSMUSG00000042079	Hnrnpf	98758	protein_coding	1023.73977	-0.323087454	0.135227702	2.38921057	0.016884622	0.064232353	0.323087454
ENSMUSG00000007458	M6pr	17113	protein_coding	2615.400493	0.32317392	0.097880359	-3.301723877	0.000960926	0.006157833	0.32317392
ENSMUSG00000088408	Gm25881	115489577	snRNA	2.223350512	-0.323191781	0.852775576	0.378988083	0.704696713	1	0.323191781
ENSMUSG00000022197	Pdzd2	68070	protein_coding	38.6756161	-0.323250083	0.255475197	1.265289495	0.205767571	0.408369867	0.323250083
ENSMUSG00000029086	Prom1	19126	protein_coding	4922.35339	-0.323409648	0.112854723	2.865716557	0.004160666	0.020846249	0.323409648
ENSMUSG00000038188	Scarf1	380713	protein_coding	44.7877686	-0.323531022	0.215387212	1.502090207	0.133073814	0.302752759	0.323531022
ENSMUSG00000088208	Gm23751	115490492	scaRNA	2.297367509	-0.323640972	0.772148666	0.419143341	0.675111377	1	0.323640972
ENSMUSG00000005503	Evx1	14028	protein_coding	27.69509638	0.323648812	0.23908886	-1.353675832	0.175839822	0.367865096	0.323648812
ENSMUSG00000003282	Plag1	56711	protein_coding	35.53715859	-0.323655344	0.185014924	1.749347224	0.080231018	0.21047419	0.323655344
ENSMUSG00000008398	Elk3	13713	protein_coding	23.61902344	-0.323663063	0.216829458	1.492707985	0.135513642	0.30685209	0.323663063
ENSMUSG00000001819	Hoxd13	15433	protein_coding	129.721534	-0.323667915	0.427426394	0.757248311	0.448901112	0.665664739	0.323667915
ENSMUSG00000038700	Hoxb5	15413	protein_coding	46.14178143	-0.323697613	0.364563208	0.887905322	0.374591682	0.596644197	0.323697613
ENSMUSG00000034685	Fam171a2	217219	protein_coding	8.583221428	0.323716638	0.493014382	-0.656606885	0.51143372	0.71423395	0.323716638
ENSMUSG00000029363	Rfc5	72151	protein_coding	268.1711963	0.324005288	0.156350729	-2.072297905	0.038237668	0.121691433	0.324005288
ENSMUSG00000037669	Ldah	68832	protein_coding	666.2664129	0.324012713	0.083058863	-3.901001077	9.58E-05	0.000829125	0.324012713
ENSMUSG00000019866	Crybg1	11630	protein_coding	1724.301617	0.324050898	0.094809447	-3.417917798	0.000631022	0.0043132	0.324050898
ENSMUSG00000074183	Gsta1	14857	protein_coding	88.64165165	0.32407895	0.213745341	-1.516191882	0.12947084	0.297203427	0.32407895
ENSMUSG00000070639	Lrrc8b	433926	protein_coding	318.6946558	0.324090969	0.081689877	-3.967333291	7.27E-05	0.0006509	0.324090969
ENSMUSG00000083041	Gmfg-ps	100040018	processed_pseudogene	1.089915481	0.324116066	1.259546478	-0.257327595	0.796925892	1	0.324116066
ENSMUSG00000094622	Gm3055	100040944	protein_coding	5.085343695	-0.324257985	0.476953063	0.67985303	0.496597525	0.702605511	0.324257985
ENSMUSG00000032085	Tagln	21345	protein_coding	16.70482724	0.324298969	0.292976583	-1.106910887	0.268332458	0.483440174	0.324298969
ENSMUSG00000027107	Chrna1	11435	protein_coding	20.41056025	-0.324317872	0.299507175	1.082838406	0.278880155	0.496167516	0.324317872
ENSMUSG00000029598	Plbd2	71772	protein_coding	261.1084495	-0.324324551	0.07545721	4.298125402	1.72E-05	0.000180604	0.324324551
ENSMUSG00000076437	Selenoh	72657	protein_coding	169.8483574	0.324374005	0.154787214	-2.095612401	0.036116588	0.116104602	0.324374005
ENSMUSG00000029368	Alb	11657	protein_coding	13.29974312	0.324413195	0.39894782	-0.813171995	0.416119453	0.636395319	0.324413195
ENSMUSG00000098347	Mir6339	102466628	miRNA	3.41881392	0.324463581	0.70447594	-0.460574396	0.645103985	1	0.324463581
ENSMUSG00000068129	Cst7	13011	protein_coding	3.316451659	0.324560635	0.66698986	-0.486605051	0.626538244	1	0.324560635
ENSMUSG00000027618	Nfs1	18041	protein_coding	472.7081416	-0.324572356	0.079273142	4.094354614	4.23E-05	0.000404346	0.324572356
ENSMUSG00000010601	Apol7a	75761	protein_coding	116.0037701	-0.324595026	0.362472479	0.895502541	0.370518508	0.593022866	0.324595026
ENSMUSG00000021982	Cdadc1	71891	protein_coding	210.291062	-0.324596698	0.100891099	3.217297672	0.001294043	0.007882634	0.324596698
ENSMUSG00000018166	Erbb3	13867	protein_coding	4674.942508	-0.324708628	0.110593873	2.936045359	0.003324257	0.017334001	0.324708628
ENSMUSG00000006219	Fblim1	74202	protein_coding	674.5872437	-0.324754597	0.073899448	4.394546981	1.11E-05	0.000122946	0.324754597
ENSMUSG00000053714	4732471J01Rik	654804	lncRNA	51.94766484	0.324823822	0.23822289	-1.363529013	0.172715847	0.363275924	0.324823822

Supplementary Table 1

ensembl_gene_id	external_gene_name	entrezgene_id	gene_biotype	baseMean	log2FoldChange	lfcSE	stat	pvalue	padj	absLog2FC
ENSMUSG00000071415	Rpl23	65019	protein_coding	4564.948076	-0.324891988	0.123531273	2.63003836	0.008537523	0.03747705	0.324891988
ENSMUSG00000000751	Rpa1	68275	protein_coding	536.3567917	0.324892458	0.145487395	-2.233131322	0.025540286	0.088775474	0.324892458
ENSMUSG00000038225	Primpol	408022	protein_coding	101.3645654	-0.324924498	0.167787573	1.936523027	0.052803675	0.155547608	0.324924498
ENSMUSG00000049488	Tmem67	329795	protein_coding	62.76575439	0.324970124	0.162949731	-1.994296777	0.046119631	0.140291806	0.324970124
ENSMUSG00000028073	Pear1	73182	protein_coding	56.42051547	0.324986066	0.188119561	-1.72755063	0.084068821	0.217760374	0.324986066
ENSMUSG00000027955	Gask1b	68659	protein_coding	11.59797536	0.325040201	0.39115002	-0.830986028	0.405981523	0.626929013	0.325040201
ENSMUSG00000004730	Adgre1	13733	protein_coding	2.567413694	-0.325065846	0.953161254	0.341039719	0.733073681	1	0.325065846
ENSMUSG00000018239	Zcchc10	67966	protein_coding	17.82974131	-0.325203919	0.310321896	1.047956728	0.294658544	0.513807046	0.325203919
ENSMUSG00000004446	Bid	12122	protein_coding	130.8285758	-0.32523774	0.111432126	2.918707127	0.003514863	0.018172963	0.32523774
ENSMUSG00000032265	Tent5a	212943	protein_coding	1498.066844	-0.325252827	0.093534066	3.477372918	0.000506353	0.003584189	0.325252827
ENSMUSG00000062006	Rpl34	68436	protein_coding	2381.690994	-0.325253138	0.133832097	2.430307414	0.015086021	0.058674176	0.325253138
ENSMUSG00000065476	Mir30b	387226	miRNA	1.570088225	-0.325306091	1.196960565	0.271776783	0.785793658	1	0.325306091
ENSMUSG00000040938	Slc16a11	216867	protein_coding	33.00610827	-0.325417119	0.21937323	1.483394848	0.137969535	0.31052626	0.325417119
ENSMUSG00000029235	Pdcl2	79455	protein_coding	0.70998615	0.325422102	1.431476783	-0.227333133	0.820164711	1	0.325422102
ENSMUSG00000091996	Smim35	408059	protein_coding	9.909708889	0.325534315	0.435073604	-0.748228145	0.454322557	0.669875536	0.325534315
ENSMUSG00000010066	Cacna2d2	56808	protein_coding	87.11161187	-0.325569244	0.171937957	1.893527461	0.05828776	0.167227948	0.325569244
ENSMUSG00000092032	Vmn2r59	628444	protein_coding	3.14997079	-0.325572464	1.972285767	0.165073677	0.868885991	1	0.325572464
ENSMUSG00000001418	Glmp	56700	protein_coding	664.7783026	-0.325575765	0.062999502	5.167910155	2.37E-07	3.66E-06	0.325575765
ENSMUSG00000031389	Arhgap4	171207	protein_coding	41.4516654	-0.325671029	0.221302509	1.4716102	0.141126169	0.31570815	0.325671029
ENSMUSG00000029030	Tprgl	67808	protein_coding	835.8385533	-0.325689518	0.069481319	4.687440092	2.77E-06	3.47E-05	0.325689518
ENSMUSG00000004637	Wwox	80707	protein_coding	128.5253158	0.325700174	0.098070851	-3.321070119	0.00089673	0.005807294	0.325700174
ENSMUSG00000063296	Tmem117	320709	protein_coding	295.5126244	-0.325766143	0.151490187	2.150410859	0.03152273	0.104847204	0.325766143
ENSMUSG00000031303	Map3k15	270672	protein_coding	37.62904205	0.325822745	0.213512227	-1.526014455	0.127006265	0.293627531	0.325822745
ENSMUSG00000023995	Tspo2	70026	protein_coding	4.02871342	-0.325849092	0.655032594	0.497454775	0.618868387	1	0.325849092
ENSMUSG00000037705	Tecta	21683	protein_coding	2.324789347	-0.325928564	1.869870719	0.1743054	0.861625455	1	0.325928564
ENSMUSG00000073542	Cep76	225659	protein_coding	90.12638527	0.326024802	0.146340183	-2.227855642	0.025890143	0.089783667	0.326024802
ENSMUSG00000026172	Bcs1l	66821	protein_coding	138.7936149	-0.326025129	0.114238599	2.853896423	0.004318661	0.021513836	0.326025129
ENSMUSG00000088990	Gm22767	115490051	scaRNA	10.02024492	0.32603211	0.405552629	-0.803920593	0.421442836	0.641211258	0.32603211
ENSMUSG00000040549	Ckap5	75786	protein_coding	1113.770644	0.326096937	0.105139847	-3.101554239	0.001925076	0.011004052	0.326096937
ENSMUSG00000025257	Ribc1	66611	protein_coding	26.55848979	-0.326099701	0.230654636	1.413800764	0.157420409	0.340684159	0.326099701
ENSMUSG00000047793	Sned1	208777	protein_coding	234.9429766	-0.326181896	0.1194318	2.731114302	0.006312058	0.029222685	0.326181896
ENSMUSG00000029651	Mtus2	77521	protein_coding	44.12555704	0.326498441	0.350858994	-0.930568824	0.352076648	0.574354812	0.326498441
ENSMUSG00000008822	Acyp1	66204	protein_coding	74.6327845	-0.326530646	0.139320234	2.343741725	0.019091387	0.070752953	0.326530646
ENSMUSG00000022705	Drd3	13490	protein_coding	7.844231049	-0.326577474	0.472497573	0.691172806	0.489456951	0.697221779	0.326577474
ENSMUSG00000035165	Kcne3	57442	protein_coding	1342.14672	-0.326633126	0.20204281	1.616653051	0.105953193	0.258145624	0.326633126
ENSMUSG00000042622	Maff	17133	protein_coding	420.9130001	-0.32672142	0.241501175	1.352877147	0.176094881	0.368018849	0.32672142
ENSMUSG00000084950	Gm5577	434064	lncRNA	1.896004816	0.326917305	0.989876564	-0.330260678	0.741203002	1	0.326917305
ENSMUSG00000026361	Cdc73	214498	protein_coding	440.7482187	0.326925199	0.094122327	-3.473407517	0.000513894	0.003631345	0.326925199
ENSMUSG00000030188	Magohb	66441	protein_coding	101.0553448	0.326968244	0.166087307	-1.96865281	0.048992974	0.146863309	0.326968244

Supplementary Table 1

ensembl_gene_id	external_gene_name	entrezgene_id	gene_biotype	baseMean	log2FoldChange	lfcSE	stat	pvalue	padj	absLog2FC
ENSMUSG00000024587	Nars	70223	protein_coding	2300.34225	0.326983013	0.076362224	-4.28199959	1.85E-05	0.000192981	0.326983013
ENSMUSG00000028549	Itgb3bp	67733	protein_coding	56.14732233	0.326999157	0.16104839	-2.030440403	0.042311793	0.131292175	0.326999157
ENSMUSG00000020719	Ddx5	13207	protein_coding	7846.135244	-0.327018404	0.05674902	5.762538348	8.29E-09	1.62E-07	0.327018404
ENSMUSG00000085763	Smc2os	72648	lncRNA	1.406289038	-0.327097333	0.959652674	0.340849707	0.733216728	1	0.327097333
ENSMUSG00000036057	Ptpn23	104831	protein_coding	289.5156694	0.327184347	0.185615477	-1.762699708	0.07795115	0.206262665	0.327184347
ENSMUSG00000078789	Dph1	116905	protein_coding	115.3273093	-0.32731663	0.118587363	2.760130786	0.005777823	0.027402344	0.32731663
ENSMUSG00000006235	Epor	13857	protein_coding	2.289641013	-0.327321048	1.196851489	0.273485099	0.784480345	1	0.327321048
ENSMUSG00000031512	Tex29	75528	protein_coding	2.126293241	0.327326124	1.363299762	-0.240098424	0.810253956	1	0.327326124
ENSMUSG00000098188	Sowahc	268301	protein_coding	840.2308738	-0.327467688	0.093488639	3.502753829	0.000460475	0.003307101	0.327467688
ENSMUSG00000095961	Vmn2r102	224572	protein_coding	4.263653931	0.327555553	1.774015776	-0.184640722	0.853510844	1	0.327555553
ENSMUSG00000037321	Tap1	21354	protein_coding	242.7449895	-0.327598158	0.21044266	1.556709832	0.119539393	0.281287196	0.327598158
ENSMUSG00000049881	2810025M15Rik	69953	transcribed_processed_pseudogene	74.3116588	0.327619451	0.15575536	-2.103423287	0.035428785	0.114584988	0.327619451
ENSMUSG00000108621	Gm33989	102637087	lncRNA	232.8901115	-0.327623624	0.130692624	2.50682567	0.012182078	0.049685066	0.327623624
ENSMUSG00000021578	Ccdc127	67433	protein_coding	356.8246997	0.327624074	0.101377532	-3.23172272	0.001230464	0.007553859	0.327624074
ENSMUSG00000069678	Pcgf1	69837	protein_coding	62.65418776	-0.327637655	0.133051072	2.46249542	0.013797394	0.054614131	0.327637655
ENSMUSG00000029131	Dnajb6	23950	protein_coding	368.91685	-0.327659776	0.083130289	3.941520944	8.10E-05	0.000716166	0.327659776
ENSMUSG00000084678	Gm26020	115489260	snRNA	2.543665007	0.327672183	0.7873799	-0.416155129	0.677296492	1	0.327672183
ENSMUSG00000060279	Ap2a1	11771	protein_coding	788.8169379	0.327674585	0.074205576	-4.415767714	1.01E-05	0.000112689	0.327674585
ENSMUSG00000079103	Tgm7	640543	protein_coding	4.428075409	0.32771691	0.779631635	-0.420348399	0.674230958	0.825194181	0.32771691
ENSMUSG00000021701	Plk2	20620	protein_coding	435.9368009	-0.327721486	0.169725922	1.930886462	0.053497095	0.157015607	0.327721486
ENSMUSG00000025856	Pdgfa	18590	protein_coding	439.1293052	-0.327748176	0.109629352	2.989602414	0.002793408	0.014996859	0.327748176
ENSMUSG00000045662	Henmt1	66715	protein_coding	2.175776258	0.327789703	0.939544492	-0.348881512	0.727178266	1	0.327789703
ENSMUSG00000038886	Man2a2	140481	protein_coding	95.40982354	0.327855301	0.146635243	-2.235856095	0.025361199	0.08834262	0.327855301
ENSMUSG00000020017	Hal	15109	protein_coding	3.016966196	0.327868004	1.266596139	-0.258857574	0.795745134	1	0.327868004
ENSMUSG00000019865	Nmbr	18101	protein_coding	1.104007993	0.327877305	1.466522755	-0.223574645	0.823088294	1	0.327877305
ENSMUSG00000043531	Sorcs1	58178	protein_coding	2.456876349	-0.327877411	1.823736955	0.179783279	0.857322711	1	0.327877411
ENSMUSG00000021003	Galc	14420	protein_coding	61.20589836	0.327963839	0.146854	-2.233264597	0.025531501	0.088763622	0.327963839
ENSMUSG00000003847	Nfat5	54446	protein_coding	364.0062262	-0.328119322	0.181871537	1.804126849	0.071211424	0.193418659	0.328119322
ENSMUSG00000091086	Rpl6l	432502	processed_pseudogene	4.569947312	0.328164506	0.51919138	-0.63206848	0.527342131	0.725771143	0.328164506
ENSMUSG00000028759	Hp1bp3	15441	protein_coding	1682.846115	0.328268874	0.056297725	-5.830943903	5.51E-09	1.10E-07	0.328268874
ENSMUSG00000042035	Igsf3	78908	protein_coding	477.6689561	-0.328278663	0.131318429	2.499867419	0.012423979	0.050422608	0.328278663
ENSMUSG00000062064	Slc2a7	435818	protein_coding	2.815142809	0.328343916	1.166249306	-0.281538359	0.778297511	1	0.328343916
ENSMUSG00000021702	Thbs4	21828	protein_coding	2318.485996	-0.328465371	0.236151228	1.390911128	0.164252378	0.350538094	0.328465371
ENSMUSG00000098866	Mir6539	102465310	miRNA	1.029048646	-0.32847835	1.304722171	0.251761147	0.80122569	1	0.32847835
ENSMUSG00000091754	Gm3636	100041151	protein_coding	2.323818863	-0.328495342	0.751823312	0.436931573	0.662160974	1	0.328495342
ENSMUSG00000008028	1700008O03Rik	69349	protein_coding	3.494263005	0.328557138	1.668194871	-0.196953692	0.843863777	1	0.328557138
ENSMUSG00000065750	Gm23346	115490470	snoRNA	5.467598206	0.328608324	0.499962992	-0.657265297	0.511010346	0.713884177	0.328608324
ENSMUSG00000042747	Krtcap2	66059	protein_coding	597.6373589	0.328621987	0.119399964	-2.75227878	0.005918212	0.027890446	0.328621987
ENSMUSG00000028484	Psip1	101739	protein_coding	215.3461363	0.328638088	0.149550121	-2.197511354	0.027983947	0.095392863	0.328638088

Supplementary Table 1

ensembl_gene_id	external_gene_name	entrezgene_id	gene_biotype	baseMean	log2FoldChange	lfcSE	stat	pvalue	padj	absLog2FC
ENSMUSG00000033285	Wdr3	269470	protein_coding	328.9247399	0.328640846	0.095191602	-3.452414284	0.000555594	0.003866447	0.328640846
ENSMUSG00000040283	Btnl9	237754	protein_coding	5.49571421	0.328688612	0.726325428	-0.452536287	0.650882686	0.810260975	0.328688612
ENSMUSG00000065669	Gm22581	115489818	snoRNA	2.830239605	-0.328745788	0.647565576	0.507664089	0.611688937	1	0.328745788
ENSMUSG00000007034	Slc44a4	70129	protein_coding	4978.067283	-0.32878964	0.06454245	5.094161109	3.50E-07	5.32E-06	0.32878964
ENSMUSG00000060803	Gstp1	14870	protein_coding	1513.103542	-0.328816551	0.102464998	3.209062203	0.001331687	0.00808213	0.328816551
ENSMUSG00000022195	6030458C11Rik	77877	protein_coding	410.9536593	0.328846767	0.080276491	-4.096426766	4.20E-05	0.000401209	0.328846767
ENSMUSG00000090015	Gm15446	100642166	protein_coding	68.55705781	-0.328891661	0.173473447	1.895919326	0.057970713	0.166571051	0.328891661
ENSMUSG00000019813	Cep57l1	103268	protein_coding	42.6276725	0.328901725	0.18992797	-1.731718213	0.083323746	0.216285499	0.328901725
ENSMUSG00000055553	Kxd1	75620	protein_coding	467.1456214	-0.328984117	0.07107467	4.628711166	3.68E-06	4.53E-05	0.328984117
ENSMUSG00000105352	C030018K13Rik	77382	lncRNA	1.22926799	0.329042794	1.490185899	-0.220806541	0.82524307	1	0.329042794
ENSMUSG00000022668	Gtpbp8	66067	protein_coding	246.2040834	-0.329058442	0.099409999	3.310114133	0.000932579	0.006001799	0.329058442
ENSMUSG00000029833	Trim24	21848	protein_coding	775.2969861	0.329125603	0.053321555	-6.172468192	6.72E-10	1.51E-08	0.329125603
ENSMUSG00000021831	Ero1a	50527	protein_coding	565.6525988	0.329188136	0.077612936	-4.241408123	2.22E-05	0.000226707	0.329188136
ENSMUSG00000075254	Heg1	77446	protein_coding	116.7881646	0.329208603	0.171118918	-1.923858602	0.05437231	0.158736855	0.329208603
ENSMUSG00000039062	Anpep	16790	protein_coding	146.7071497	-0.329218342	0.598181182	0.550365594	0.582068642	0.765030035	0.329218342
ENSMUSG00000015305	Sash1	70097	protein_coding	949.8679935	-0.329252812	0.13037675	2.525395158	0.011556826	0.047749183	0.329252812
ENSMUSG00000038201	Kcna7	16495	protein_coding	1.221273544	-0.329275573	1.153266835	0.285515514	0.77524922	1	0.329275573
ENSMUSG00000020151	Ptprr	19279	protein_coding	247.6263121	-0.329284976	0.264378012	1.245508178	0.212945011	0.417696153	0.329284976
ENSMUSG00000063972	Nr6a1	14536	protein_coding	126.0547618	-0.329288658	0.201267982	1.636070748	0.101824781	0.250862963	0.329288658
ENSMUSG00000035228	Ccdc106	232821	protein_coding	3.381885486	0.329518308	0.739039501	-0.445873743	0.65568845	1	0.329518308
ENSMUSG00000028763	Hspg2	15530	protein_coding	494.711838	-0.329543414	0.113415268	2.905635376	0.003665083	0.018819941	0.329543414
ENSMUSG00000024121	Atp6v0c	11984	protein_coding	601.5625673	-0.329553712	0.103875967	3.172569393	0.001510964	0.008943393	0.329553712
ENSMUSG00000027612	Mmp24	17391	protein_coding	7.278261621	-0.329554093	0.531525683	0.620015371	0.535247667	0.731167175	0.329554093
ENSMUSG00000031881	Cdh16	12556	protein_coding	2.1285986	0.329555822	1.535221205	-0.214663412	0.830029751	1	0.329555822
ENSMUSG00000001542	Ell2	192657	protein_coding	698.8523013	0.329599082	0.077084209	-4.275831432	1.90E-05	0.000197035	0.329599082
ENSMUSG00000031959	Wdr59	319481	protein_coding	231.2036618	0.329600991	0.127306751	-2.589029959	0.009624672	0.041288574	0.329600991
ENSMUSG00000027086	Fastkd1	320720	protein_coding	316.3305267	-0.329615412	0.098733692	3.338428918	0.000842536	0.005507949	0.329615412
ENSMUSG00000059040	Eno1b	433182	protein_coding	2.408980379	0.329946079	0.703218397	-0.469194322	0.638930744	1	0.329946079
ENSMUSG00000099301	Mir3473e	102466677	miRNA	0.594424915	0.329994827	1.242514481	-0.265586303	0.790557836	1	0.329994827
ENSMUSG00000046793	Gpr61	229714	protein_coding	2.296110606	-0.330123715	0.875349322	0.377133685	0.706074259	1	0.330123715
ENSMUSG00000034035	Ccdc17	622665	protein_coding	46.84070774	-0.330149738	0.168294744	1.96173529	0.049793311	0.148427209	0.330149738
ENSMUSG00000021556	Golm1	105348	protein_coding	8577.456403	0.330271326	0.104551668	-3.158929276	0.001583499	0.009312644	0.330271326
ENSMUSG00000040522	Tlr8	170744	protein_coding	1.775747267	-0.330409503	1.258193437	0.262606284	0.792854056	1	0.330409503
ENSMUSG00000033233	Trim45	229644	protein_coding	45.29145739	0.33043343	0.170428112	-1.938843452	0.052520403	0.154934252	0.33043343
ENSMUSG00000020362	Cnot6	104625	protein_coding	755.08091	0.330454269	0.094030407	-3.51433413	0.000440858	0.003195929	0.330454269
ENSMUSG00000010358	Ifi35	70110	protein_coding	265.0714238	-0.330489449	0.124837479	2.647357596	0.008112352	0.036011679	0.330489449
ENSMUSG00000090236	Car15	80733	protein_coding	7.294955998	-0.330494451	0.46712957	0.7075006	0.47925544	0.690564248	0.330494451
ENSMUSG00000032841	Prr5l	72446	protein_coding	55.0448305	-0.330690124	0.23621456	1.399956562	0.161526326	0.346538122	0.330690124
ENSMUSG00000095115	Itpripl2	319622	protein_coding	94.39265214	-0.330692082	0.213201596	1.551076957	0.120883243	0.283485892	0.330692082

ensembl_gene_id	external_gene_name	entrezgene_id	gene_biotype	baseMean	log2FoldChange	lfcSE	stat	pvalue	padj	absLog2FC
ENSMUSG00000028571	Cyp2j13	230459	protein_coding	8.492776229	0.330788697	0.472129075	-0.700631913	0.483532757	0.693930647	0.330788697
ENSMUSG00000042487	Leo1	235497	protein_coding	394.0237129	0.330835398	0.097273956	-3.401068585	0.00067123	0.004533615	0.330835398
ENSMUSG00000086837	Gm16618	100504291	lncRNA	3.673615392	-0.330850672	0.561497349	0.589229268	0.555707486	1	0.330850672
ENSMUSG00000029622	Arpc1b	11867	protein_coding	3650.446721	-0.330978232	0.078687442	4.206239555	2.60E-05	0.000260665	0.330978232
ENSMUSG00000084804	1700095J12Rik	73563	lncRNA	1.564889482	-0.331038669	1.175832962	0.281535456	0.778299737	1	0.331038669
ENSMUSG00000003153	Slc2a3	20527	protein_coding	4.796960898	-0.331108635	0.825275573	0.401209784	0.688265678	0.833786242	0.331108635
ENSMUSG00000053600	Zfp472	224691	protein_coding	102.1422363	-0.331236943	0.146742278	2.257270004	0.023991209	0.084565809	0.331236943
ENSMUSG00000085645	Hoxb5os	75395	lncRNA	21.29178218	-0.331280186	0.419317511	0.790046152	0.429500816	0.64833423	0.331280186
ENSMUSG00000062101	Zfp119b	240120	protein_coding	35.81965475	-0.331328164	0.189470677	1.748704179	0.080342167	0.210665378	0.331328164
ENSMUSG00000040520	Manea	242362	protein_coding	284.3061845	0.331373519	0.088156516	-3.758922583	0.000170647	0.001378698	0.331373519
ENSMUSG00000041845	Rhod	11854	protein_coding	109.1867582	0.331375315	0.24222359	-1.3680555	0.171294705	0.361410598	0.331375315
ENSMUSG00000052395	Rft1	328370	protein_coding	229.0351121	0.331404405	0.103691954	-3.196047448	0.001393242	0.008390955	0.331404405
ENSMUSG00000033111	3830406C13Rik	218734	protein_coding	177.0094226	-0.331464336	0.117407019	2.823207167	0.004754584	0.023333491	0.331464336
ENSMUSG00000000957	Mmp14	17387	protein_coding	630.9506752	0.331557817	0.090794655	-3.651732771	0.000260477	0.002004434	0.331557817
ENSMUSG00000061731	Ext1	14042	protein_coding	473.9098162	0.331589994	0.070072002	-4.732132424	2.22E-06	2.87E-05	0.331589994
ENSMUSG00000067818	Myl9	98932	protein_coding	1.156438844	-0.331605523	1.276788566	0.259718431	0.795080975	1	0.331605523
ENSMUSG00000046269	Usp27x	54651	protein_coding	25.25459994	-0.331697895	0.227477789	1.458155086	0.144797801	0.321915843	0.331697895
ENSMUSG00000017677	Wsb1	78889	protein_coding	1261.365348	-0.331841446	0.140290405	2.36538947	0.018011119	0.067522273	0.331841446
ENSMUSG00000035198	Tubg1	103733	protein_coding	189.6115995	0.33189879	0.1420511	-2.336474626	0.019466519	0.07190116	0.33189879
ENSMUSG00000030123	Plxnd1	67784	protein_coding	25.74337841	-0.331986626	0.263819154	1.258387122	0.208251787	0.41130117	0.331986626
ENSMUSG00000074277	Phldb3	232970	protein_coding	78.7771785	-0.33203871	0.188247675	1.763839631	0.077758983	0.205917797	0.33203871
ENSMUSG00000002699	Lcp2	16822	protein_coding	10.50200992	0.332057008	0.56150781	-0.591366678	0.554274762	0.744656458	0.332057008
ENSMUSG00000025076	Casp7	12369	protein_coding	1841.89003	-0.332100012	0.105295755	3.153973414	0.001610638	0.009451256	0.332100012
ENSMUSG00000039084	Chad	12643	protein_coding	28.69203514	-0.332171844	0.280751351	1.183153147	0.236748458	0.446932387	0.332171844
ENSMUSG00000020743	Mif4gd	69674	protein_coding	414.5996211	-0.332206987	0.07963419	4.171662774	3.02E-05	0.000298838	0.332206987
ENSMUSG00000095789	Nupr1l	69034	protein_coding	6.9537229	0.33221243	0.430870945	-0.77102537	0.440691894	0.658605063	0.33221243
ENSMUSG00000068335	Dok1	13448	protein_coding	152.5507721	-0.332319378	0.143496066	2.315877971	0.020564932	0.074952393	0.332319378
ENSMUSG00000019699	Akt3	23797	protein_coding	8.768642076	-0.332320684	0.418065038	0.794901879	0.426670581	0.646247636	0.332320684
ENSMUSG00000074639	Rdh16f2	216454	protein_coding	5.810058366	-0.332348626	0.516836231	0.643044364	0.520195314	0.720257015	0.332348626
ENSMUSG00000071724	Smpd5	100503915	protein_coding	4.69529745	-0.332413846	0.481890411	0.68981212	0.490312346	0.69793377	0.332413846
ENSMUSG00000021193	Pitrm1	69617	protein_coding	558.3400146	0.332451084	0.088027051	-3.776692301	0.000158925	0.001290307	0.332451084
ENSMUSG00000003269	Cyth2	19158	protein_coding	703.7946589	0.33249873	0.077350241	-4.298612744	1.72E-05	0.000180437	0.33249873
ENSMUSG00000039851	4932438H23Rik	74387	protein_coding	12.17160751	0.332536195	0.367609448	-0.904590991	0.36568211	0.588354867	0.332536195
ENSMUSG00000052516	Robo2	268902	protein_coding	350.9332882	0.332566963	0.250443849	-1.327910288	0.184207746	0.379255294	0.332566963
ENSMUSG00000032418	Me1	17436	protein_coding	222.9460377	0.332633958	0.195071809	-1.705187238	0.088159509	0.225331394	0.332633958
ENSMUSG00000026189	Pecr	111175	protein_coding	83.54268441	0.332637573	0.174550312	-1.905683059	0.056691339	0.163933457	0.332637573
ENSMUSG00000046854	Pip5kl1	227733	protein_coding	3.133264272	0.332773483	0.815490132	-0.408065616	0.683225503	1	0.332773483
ENSMUSG00000053317	Sec61b	66212	protein_coding	1085.581679	0.33286972	0.110346065	-3.016598002	0.002556286	0.013886349	0.33286972
ENSMUSG00000109228	Fam81b	238726	protein_coding	1.329190379	-0.332893795	1.626703972	0.204643131	0.837850954	1	0.332893795

ensembl_gene_id	external_gene_name	entrezgene_id	gene_biotype	baseMean	log2FoldChange	lfcSE	stat	pvalue	padj	absLog2FC
ENSMUSG00000052560	Cpne8	66871	protein_coding	241.8838955	0.332953162	0.086142333	-3.865151417	0.00011102	0.000944559	0.332953162
ENSMUSG00000056116	H2-T22	15039	protein_coding	394.3735863	-0.332971258	0.093440939	3.563440856	0.000366025	0.002719091	0.332971258
ENSMUSG00000055235	Wdr86	269633	protein_coding	4.348801381	-0.333074247	0.953645163	0.349264338	0.726890869	1	0.333074247
ENSMUSG00000029630	Cyp3a25	56388	protein_coding	29.63697323	0.333080698	0.404778226	-0.822872074	0.410580745	0.630787089	0.333080698
ENSMUSG00000028698	Pik3r3	18710	protein_coding	291.1591737	0.333096947	0.125134828	-2.661904373	0.007769995	0.034782786	0.333096947
ENSMUSG00000031936	Hephl1	244698	protein_coding	3.481866073	0.333149048	1.286888984	-0.2588794	0.795728293	1	0.333149048
ENSMUSG00000049604	Hoxb13	15408	protein_coding	2359.510884	-0.333371281	0.332142507	1.00369954	0.315523459	0.53655334	0.333371281
ENSMUSG00000020393	Kremen1	84035	protein_coding	301.2583722	-0.333388009	0.096809142	3.443765769	0.000573672	0.003977173	0.333388009
ENSMUSG00000046667	Rbm12b1	72397	protein_coding	15.19406535	0.333403821	0.29034956	-1.148284231	0.250851247	0.463099872	0.333403821
ENSMUSG00000006373	Pgrmc1	53328	protein_coding	1302.994746	-0.333444916	0.067663507	4.927987509	8.31E-07	1.17E-05	0.333444916
ENSMUSG00000056209	Npm3	18150	protein_coding	237.1279803	-0.333495105	0.148390072	2.247421948	0.024613074	0.086297303	0.333495105
ENSMUSG00000035298	Klhl35	72184	protein_coding	5.938956	0.333499315	0.575046726	-0.57995168	0.561947203	0.750140815	0.333499315
ENSMUSG00000092738	Mir5135	100628604	miRNA	2.482308048	-0.333547335	0.729598012	0.457165905	0.647551803	1	0.333547335
ENSMUSG00000022940	Pigp	56176	protein_coding	184.2591153	-0.333577769	0.096473053	3.457729994	0.000544747	0.00381346	0.333577769
ENSMUSG00000038762	Abcf1	224742	protein_coding	1382.801041	0.333644807	0.059848058	-5.574864394	2.48E-08	4.48E-07	0.333644807
ENSMUSG00000032507	Fbxl2	72179	protein_coding	7.835455882	-0.333708396	0.485744478	0.687003995	0.492080211	0.698788598	0.333708396
ENSMUSG00000036091	Hyal3	109685	protein_coding	7.110948342	-0.333784804	0.438149638	0.761805501	0.446176096	0.663438867	0.333784804
ENSMUSG00000042414	Prdm14	383491	protein_coding	14.70294844	0.333819272	0.339355411	-0.983686312	0.325269772	0.547052145	0.333819272
ENSMUSG00000056972	Magel2	27385	protein_coding	1.515053078	0.333895151	2.110792113	-0.158184763	0.87431121	1	0.333895151
ENSMUSG00000039753	Fbxl5	242960	protein_coding	1205.800414	0.334136804	0.06637875	-5.033791781	4.81E-07	7.13E-06	0.334136804
ENSMUSG00000060376	Bckdha	12039	protein_coding	169.4368071	-0.334150474	0.127069132	2.62967463	0.008546662	0.037507197	0.334150474
ENSMUSG00000070815	Vmn1r87	171261	protein_coding	1.173573317	-0.334180396	2.3171638	0.144219583	0.885327078	1	0.334180396
ENSMUSG00000026149	Tm4sf20	66261	protein_coding	1618.159119	-0.334186696	0.092870939	3.598399022	0.000320182	0.002408878	0.334186696
ENSMUSG00000033792	Atp7a	11977	protein_coding	432.2208069	-0.334192883	0.084758759	3.942871333	8.05E-05	0.000713673	0.334192883
ENSMUSG00000044968	Napepld	242864	protein_coding	137.9597204	-0.334211493	0.141820793	2.356576119	0.018444287	0.068863768	0.334211493
ENSMUSG00000066438	Plekhd1	217682	protein_coding	48.08359622	-0.334251802	0.22594843	1.479327832	0.139052718	0.312211636	0.334251802
ENSMUSG00000022893	Adamts1	11504	protein_coding	2.318913282	-0.334274532	1.477382879	0.226261274	0.820998217	1	0.334274532
ENSMUSG00000026492	Tfb2m	15278	protein_coding	135.2763403	-0.334314597	0.112543333	2.970541106	0.002972756	0.015750587	0.334314597
ENSMUSG00000041954	Tnfrsf18	21936	protein_coding	21.85718948	0.334393219	0.22035625	-1.517511846	0.129137505	0.296685693	0.334393219
ENSMUSG00000018920	Cxcl16	66102	protein_coding	40.22026556	-0.334500512	0.498202785	0.671414377	0.501956589	0.706691156	0.334500512
ENSMUSG00000077391	Gm24336	115489055	snoRNA	363.4043518	-0.334516564	0.095524588	3.501889629	0.000461971	0.00331551	0.334516564
ENSMUSG00000018341	Il12rb2	16162	protein_coding	3.664138448	-0.33455936	0.687675117	0.486507874	0.626607124	1	0.33455936
ENSMUSG00000037295	Ldlrap1	100017	protein_coding	231.033187	-0.33460773	0.095077057	3.519332011	0.000432635	0.003147371	0.33460773
ENSMUSG00000029381	Shroom3	27428	protein_coding	3014.014056	-0.334654093	0.102650173	3.260141534	0.001113566	0.006968334	0.334654093
ENSMUSG00000068739	Sars	20226	protein_coding	1417.324866	0.334904228	0.068358184	-4.899255816	9.62E-07	1.33E-05	0.334904228
ENSMUSG00000023008	Fmnl3	22379	protein_coding	31.43141714	0.334971772	0.296771187	-1.128720664	0.259015689	0.472341459	0.334971772
ENSMUSG00000040782	Cop1	26374	protein_coding	873.1296702	-0.335020882	0.085413128	3.922358171	8.77E-05	0.000768199	0.335020882
ENSMUSG00000026199	Ankzf1	52231	protein_coding	297.8663641	-0.335042185	0.063587768	5.268972217	1.37E-07	2.21E-06	0.335042185
ENSMUSG00000096146	Kcnj11	16514	protein_coding	6.783093094	-0.335156614	0.489942534	0.684073315	0.493928871	0.700180271	0.335156614

Supplementary Table 1

ensembl_gene_id	external_gene_name	entrezgene_id	gene_biotype	baseMean	log2FoldChange	lfcSE	stat	pvalue	padj	absLog2FC
ENSMUSG00000032553	Srprb	20818	protein_coding	501.809713	0.33524281	0.105611619	-3.174298558	0.001501991	0.00890944	0.33524281
ENSMUSG00000037188	Grhl3	230824	protein_coding	13.73789308	-0.335277211	0.298005188	1.125071724	0.260558642	0.474068167	0.335277211
ENSMUSG00000029705	Cux1	13047	protein_coding	1411.515856	-0.335426486	0.052946516	6.335194682	2.37E-10	5.67E-09	0.335426486
ENSMUSG00000075225	Ccdc162	75973	protein_coding	11.62163564	-0.335436347	0.566802627	0.591804503	0.553981508	0.744442234	0.335436347
ENSMUSG00000026197	Zfand2b	68818	protein_coding	288.1580147	-0.335504095	0.07492246	4.478017638	7.53E-06	8.70E-05	0.335504095
ENSMUSG00000054702	Ap1s3	252903	protein_coding	142.206841	-0.335531869	0.164364158	2.04139317	0.041211763	0.128871625	0.335531869
ENSMUSG00000089313	Gm23182	115485729	snoRNA	2.782626561	-0.33561454	0.637488919	0.526463331	0.598566323	1	0.33561454
ENSMUSG00000063480	Snu13	20826	protein_coding	298.3020006	-0.335847375	0.142524792	2.356413714	0.018452354	0.068863768	0.335847375
ENSMUSG00000021774	Ube2e1	22194	protein_coding	216.2063816	0.335850655	0.094869491	-3.540133413	0.000399925	0.002930061	0.335850655
ENSMUSG00000040631	Dok4	114255	protein_coding	1217.418812	-0.335872293	0.100494971	3.342180101	0.000831231	0.005445042	0.335872293
ENSMUSG00000030450	Oca2	18431	protein_coding	2.882194114	-0.335976907	2.416464055	0.139036584	0.88942124	1	0.335976907
ENSMUSG00000055170	Ifng	15978	protein_coding	0.949112482	-0.336066571	1.515460347	0.221758736	0.824501704	1	0.336066571
ENSMUSG00000018974	Sart3	53890	protein_coding	533.4700765	0.336166906	0.068037858	-4.9408802	7.78E-07	1.10E-05	0.336166906
ENSMUSG00000035757	Selenoo	223776	protein_coding	286.3331158	-0.336194924	0.071056889	4.731348735	2.23E-06	2.88E-05	0.336194924
ENSMUSG00000031969	Acad8	66948	protein_coding	485.7879196	-0.336297867	0.097153954	3.461494379	0.000537185	0.003770114	0.336297867
ENSMUSG00000026469	Xpr1	19775	protein_coding	1297.393655	-0.336364815	0.091784998	3.664703637	0.000247625	0.001912667	0.336364815
ENSMUSG00000067724	Gbx1	231044	protein_coding	1.699974173	0.336367624	1.220512587	-0.275595375	0.782858863	1	0.336367624
ENSMUSG00000032280	Tle3	21887	protein_coding	582.368779	-0.336389075	0.152959881	2.19919807	0.027863842	0.095110269	0.336389075
ENSMUSG00000028684	Urod	22275	protein_coding	294.0694349	-0.336395793	0.09576872	3.512585257	0.00044377	0.003211398	0.336395793
ENSMUSG00000026674	Ddr2	18214	protein_coding	3.979259346	0.336472226	1.256766259	-0.267728565	0.788908261	1	0.336472226
ENSMUSG00000073414	Mpig6b	106722	protein_coding	7.692916513	-0.336646781	0.713745897	0.471661949	0.637168101	0.802306417	0.336646781
ENSMUSG00000064558	Gm24620	115488217	snoRNA	11.14152052	0.336675332	0.58854619	-0.572045725	0.567290997	0.753386941	0.336675332
ENSMUSG00000035697	Arhgap45	70719	protein_coding	41.68005492	0.336769938	0.366366412	-0.919216191	0.357982504	0.580790426	0.336769938
ENSMUSG00000007080	Pole	18973	protein_coding	212.2240194	0.336781049	0.175190842	-1.922366743	0.054559627	0.15918687	0.336781049
ENSMUSG00000021139	Gm20498	105940408	protein_coding	7.797719984	0.336817056	0.366196517	-0.919771325	0.357692271	0.580678813	0.336817056
ENSMUSG00000034653	Ythdc2	240255	protein_coding	433.0618497	0.336823707	0.080499545	-4.184169097	2.86E-05	0.000284557	0.336823707
ENSMUSG00000031791	Tmem38a	74166	protein_coding	48.41541246	-0.336916754	0.166501975	2.02350004	0.043021619	0.132870586	0.336916754
ENSMUSG00000048677	Tpcn2	233979	protein_coding	166.4817481	0.336971253	0.148725677	-2.265723439	0.023468322	0.083204714	0.336971253
ENSMUSG00000053289	Ddx10	77591	protein_coding	382.8725877	0.33702096	0.102966209	-3.273121944	0.001063666	0.006694122	0.33702096
ENSMUSG00000068547	Clca4a	99663	protein_coding	1035.136583	0.337174935	0.351081757	-0.960388653	0.336859647	0.559233675	0.337174935
ENSMUSG00000009030	Pdcl	67466	protein_coding	650.2719161	0.337183529	0.112220075	-3.004663185	0.002658751	0.014369954	0.337183529
ENSMUSG00000060981	H4c8	69386	protein_coding	3119.732364	0.3372335	0.115819263	-2.911722029	0.003594424	0.018508983	0.3372335
ENSMUSG00000021690	Jmy	57748	protein_coding	300.8625783	-0.337502282	0.127628913	2.644403007	0.008183516	0.036261245	0.337502282
ENSMUSG00000063179	Pstk	214580	protein_coding	177.878734	0.337633179	0.100995103	-3.343064843	0.000828585	0.005429865	0.337633179
ENSMUSG00000097281	Gm26685	102635083	lncRNA	11.31956123	-0.337792839	0.310594766	1.087567712	0.276785991	0.493664959	0.337792839
ENSMUSG00000002147	Stat6	20852	protein_coding	1081.217133	-0.337793239	0.051786505	6.522804276	6.90E-11	1.77E-09	0.337793239
ENSMUSG00000030245	Golt1b	66964	protein_coding	376.7172749	0.337998042	0.127298249	-2.655166469	0.007926928	0.035366953	0.337998042
ENSMUSG00000020592	Sdc1	20969	protein_coding	3487.253821	-0.338069504	0.073237891	4.61604638	3.91E-06	4.80E-05	0.338069504
ENSMUSG00000092985	Mir378b	100628571	miRNA	1.796434961	0.338174944	0.902565447	-0.374681908	0.707897049	1	0.338174944

Supplementary Table 1

ensembl_gene_id	external_gene_name	entrezgene_id	gene_biotype	baseMean	log2FoldChange	lfcSE	stat	pvalue	padj	absLog2FC
ENSMUSG00000048234	Rnf149	67702	protein_coding	507.0911776	-0.338211148	0.09999565	3.382258611	0.000718924	0.004789552	0.338211148
ENSMUSG00000000884	Gnb1l	13972	protein_coding	35.88478301	0.338238164	0.175245938	-1.930077052	0.053597291	0.157212375	0.338238164
ENSMUSG00000028334	Nans	94181	protein_coding	6530.111198	0.338246564	0.125471955	-2.695794158	0.007022105	0.031968135	0.338246564
ENSMUSG00000051098	Mblac2	72852	protein_coding	43.7218868	-0.338477617	0.164548563	2.057007432	0.039685511	0.12530374	0.338477617
ENSMUSG00000074452	Pate2	330921	protein_coding	3.708818975	0.338608699	0.942236876	-0.359366851	0.71932067	1	0.338608699
ENSMUSG00000047710	Champ1	101994	protein_coding	246.4927137	-0.338611481	0.110889835	3.053584502	0.00226125	0.012501603	0.338611481
ENSMUSG00000037649	H2-DMa	14998	protein_coding	32.232851	0.33865056	0.239306045	-1.415135837	0.157028672	0.340132673	0.33865056
ENSMUSG00000096210	H1f0	14958	protein_coding	4148.075524	0.338662798	0.120348859	-2.814009225	0.004892782	0.023883952	0.338662798
ENSMUSG00000021917	Spcs1	69019	protein_coding	807.8661316	0.338678296	0.099783902	-3.394117585	0.000688501	0.00461816	0.338678296
ENSMUSG00000017221	Psmd3	22123	protein_coding	1250.797615	0.338721871	0.06070996	-5.579345953	2.41E-08	4.37E-07	0.338721871
ENSMUSG00000047828	A730018C14Rik	100504733	lncRNA	1.447388305	-0.338774122	2.675537406	0.126619094	0.899241884	1	0.338774122
ENSMUSG00000033186	Mzt1	76789	protein_coding	476.2058652	0.338796292	0.103338296	-3.278516329	0.001043543	0.00659513	0.338796292
ENSMUSG00000033760	Rbm4b	66704	protein_coding	107.5134477	0.338826785	0.134101767	-2.526639231	0.011515973	0.047662852	0.338826785
ENSMUSG00000040269	Mrps28	66230	protein_coding	260.2369146	0.339056804	0.087302327	-3.88370868	0.000102875	0.000882077	0.339056804
ENSMUSG00000000869	Il4	16189	protein_coding	3.584584434	0.339143864	0.674329082	-0.502935248	0.615009798	1	0.339143864
ENSMUSG00000066838	Zfp772	232855	protein_coding	61.82415854	-0.339146786	0.180518134	1.878740808	0.060279893	0.171365493	0.339146786
ENSMUSG00000072974	Gm4787	214321	protein_coding	8.275664938	-0.339154334	0.377123311	0.899319465	0.368482522	0.591017907	0.339154334
ENSMUSG00000036825	Ssx2ip	99167	protein_coding	923.5946227	0.339158903	0.068004568	-4.987295884	6.12E-07	8.86E-06	0.339158903
ENSMUSG00000051177	Plcb1	18795	protein_coding	80.02445606	-0.339167147	0.180776698	1.876166286	0.06063245	0.172130699	0.339167147
ENSMUSG00000042523	Dnal1	105000	protein_coding	18.91950664	0.339192921	0.271850569	-1.24771827	0.212134248	0.4164509	0.339192921
ENSMUSG00000052146	Rps10	67097	protein_coding	130.037676	0.339197626	0.210275326	-1.613111877	0.106720204	0.259543427	0.339197626
ENSMUSG00000023963	Cyp39a1	56050	protein_coding	170.4036972	-0.33929377	0.138053123	2.457704423	0.013982821	0.055215758	0.33929377
ENSMUSG00000039220	Ppp1r10	52040	protein_coding	1053.556411	0.339313513	0.108591239	-3.124685894	0.00177995	0.010288448	0.339313513
ENSMUSG00000058183	Mmel1	27390	protein_coding	2.424476123	0.3393232	1.052176063	-0.322496597	0.747076515	1	0.3393232
ENSMUSG00000061751	Kalrn	545156	protein_coding	530.7705397	-0.339402219	0.15015521	2.260342612	0.023799995	0.084106216	0.339402219
ENSMUSG00000068957	4930589L23Rik	75879	lncRNA	2.403820046	-0.339570143	1.397830447	0.24292656	0.808062289	1	0.339570143
ENSMUSG00000026737	Pip4k2a	18718	protein_coding	23.60369808	0.339574524	0.282749417	-1.20097338	0.229761528	0.438341251	0.339574524
ENSMUSG00000043110	Lrrn4	320974	protein_coding	2.419293682	0.339620203	1.313829126	-0.258496479	0.796023766	1	0.339620203
ENSMUSG00000113733	Gm34785	102638153	lncRNA	1.591771574	-0.33984417	2.377925107	0.142916263	0.886356314	1	0.33984417
ENSMUSG00000011832	Evi5l	213027	protein_coding	201.4260437	0.339960502	0.101795091	-3.339655184	0.000838825	0.005488253	0.339960502
ENSMUSG00000044505	Lingo4	320747	protein_coding	6.151755563	0.340146623	0.632686701	-0.537622527	0.590837675	0.771232643	0.340146623
ENSMUSG00000042041	2010003K11Rik	69861	protein_coding	229.4280833	-0.340261596	0.219657216	1.549057218	0.121367966	0.284132491	0.340261596
ENSMUSG00000051557	Pusl1	433813	protein_coding	64.19289146	-0.340270623	0.175873199	1.934749726	0.053021015	0.15599306	0.340270623
ENSMUSG00000000214	Th	21823	protein_coding	1.329020868	-0.340409452	1.221681322	0.278640138	0.780521007	1	0.340409452
ENSMUSG00000039145	Camk1d	227541	protein_coding	1227.512325	-0.340423449	0.147032131	2.315299697	0.020596536	0.075034459	0.340423449
ENSMUSG00000002409	Dyrk1b	13549	protein_coding	140.5312619	-0.340468081	0.20146846	1.689932417	0.091040885	0.230625736	0.340468081
ENSMUSG00000064612	Gm26457	115487641	snoRNA	0.934973304	-0.340483625	1.557490309	0.218610429	0.826953528	1	0.340483625
ENSMUSG00000079652	Garin1a	245884	protein_coding	2.770867153	0.340578967	0.745923202	-0.456587174	0.6479678	1	0.340578967
ENSMUSG00000052392	Acot4	171282	protein_coding	101.6846364	0.340652983	0.197252849	-1.726986381	0.084170109	0.21796851	0.340652983

Supplementary Table 1

ensembl_gene_id	external_gene_name	entrezgene_id	gene_biotype	baseMean	log2FoldChange	lfcSE	stat	pvalue	padj	absLog2FC
ENSMUSG00000034858	Atosa	235493	protein_coding	161.3943623	-0.340728366	0.115580763	2.947967774	0.003198704	0.016787354	0.340728366
ENSMUSG00000073684	Faap20	67513	protein_coding	140.0149796	-0.340909081	0.097074283	3.511837242	0.00044502	0.003216222	0.340909081
ENSMUSG00000029597	Sds	231691	protein_coding	1.006901766	0.341000126	1.200162472	-0.284128303	0.776312058	1	0.341000126
ENSMUSG00000026414	Tnnt2	21956	protein_coding	41.98883802	-0.341055711	0.360028994	0.947300682	0.343485583	0.565422739	0.341055711
ENSMUSG00000085933	Tmem61	329909	protein_coding	2.067779304	0.341075155	1.638752617	-0.20813096	0.835126714	1	0.341075155
ENSMUSG00000105814	Mir703	735265	miRNA	13.81022801	-0.34109014	0.495670164	0.68813934	0.491365039	0.698491194	0.34109014
ENSMUSG00000063354	Slc39a4	72027	protein_coding	2083.852109	0.341123246	0.139934015	-2.437743585	0.014779253	0.057657592	0.341123246
ENSMUSG00000087166	L1td1	381591	protein_coding	2.178468644	0.341128873	2.39350521	-0.14252272	0.886667134	1	0.341128873
ENSMUSG00000104158	Becn2	226720	protein_coding	1.225701694	-0.341282537	2.338677206	0.145929731	0.883976861	1	0.341282537
ENSMUSG00000046794	Ppp1r3b	244416	protein_coding	451.9678914	-0.341336207	0.107541917	3.17398293	0.001503625	0.008912731	0.341336207
ENSMUSG00000040705	A930016O22Rik	100503495	lncRNA	1.77064334	0.341357266	1.464428698	-0.233099274	0.815684319	1	0.341357266
ENSMUSG00000029311	Hsd17b11	114664	protein_coding	830.6145914	-0.341410768	0.086219807	3.959771884	7.50E-05	0.000670367	0.341410768
ENSMUSG00000074194	Zfp791	244556	protein_coding	47.43435594	-0.341474808	0.263592543	1.295464596	0.195160007	0.394331623	0.341474808
ENSMUSG00000067529	Or4d11	258675	protein_coding	1.654415108	-0.341483741	1.627978525	0.209759364	0.83385549	1	0.341483741
ENSMUSG00000003549	Ercc1	13870	protein_coding	136.5481908	0.341558297	0.123775763	-2.759492556	0.005789121	0.027440166	0.341558297
ENSMUSG00000050368	Hoxd10	15430	protein_coding	6.855629936	-0.341565452	0.432779325	0.789236991	0.429973506	0.648691985	0.341565452
ENSMUSG00000038900	Rpl12	269261	protein_coding	4875.85955	-0.341605305	0.13552416	2.520622931	0.011714731	0.04823163	0.341605305
ENSMUSG00000036103	Colec12	140792	protein_coding	3.103964521	0.341821474	1.479239283	-0.23107923	0.817253251	1	0.341821474
ENSMUSG00000094649	Gm7102	633057	protein_coding	0.582982676	0.341900338	1.478778636	-0.231204542	0.817155901	1	0.341900338
ENSMUSG00000050860	Phospho1	237928	protein_coding	31.20995311	0.341914631	0.443124604	-0.771599293	0.440351793	0.658275506	0.341914631
ENSMUSG00000037166	Ppp1r14a	68458	protein_coding	13.99927368	-0.341939703	0.511142778	0.668971015	0.503513967	0.708014489	0.341939703
ENSMUSG00000045678	Or5b124	258628	protein_coding	3.236212819	-0.341953918	2.649750935	0.129051343	0.897317022	1	0.341953918
ENSMUSG00000030748	Il4ra	16190	protein_coding	541.6680457	0.341956837	0.118771324	-2.879119514	0.003987872	0.020127052	0.341956837
ENSMUSG00000028247	Coq3	230027	protein_coding	126.4632877	-0.342101144	0.119189481	2.870229317	0.004101742	0.020610466	0.342101144
ENSMUSG00000009281	Rarres2	71660	protein_coding	0.849920138	0.342162104	1.428486129	-0.239527775	0.810696362	1	0.342162104
ENSMUSG00000078877	Gm14295	628147	protein_coding	27.23764193	-0.342200973	0.301511054	1.134953323	0.256394876	0.469256898	0.342200973
ENSMUSG00000046761	Fam83h	105732	protein_coding	1594.695384	-0.342203447	0.095002121	3.60206113	0.000315704	0.002381698	0.342203447
ENSMUSG00000022912	Pros1	19128	protein_coding	97.90920003	0.342300552	0.237308523	-1.442428397	0.149181558	0.328390328	0.342300552
ENSMUSG00000041841	Rpl37	67281	protein_coding	2977.514515	-0.342301202	0.109921648	3.114047205	0.0018454	0.010614744	0.342301202
ENSMUSG00000044122	Proca1	216974	protein_coding	6.958356059	-0.342376118	0.631867613	0.541847865	0.587923314	0.769209762	0.342376118
ENSMUSG00000025352	Gdf11	14561	protein_coding	15.09307297	0.342422059	0.351008435	-0.975537978	0.329293473	0.551684327	0.342422059
ENSMUSG00000027890	Gstm4	14865	protein_coding	134.3776161	0.342614347	0.132481623	-2.586127344	0.009706106	0.041557335	0.342614347
ENSMUSG00000058022	Adtrp	109254	protein_coding	345.6876245	-0.342629448	0.111994395	3.059344615	0.002218218	0.01230489	0.342629448
ENSMUSG00000018322	Tomm34	67145	protein_coding	264.8394164	-0.342639135	0.083791626	4.089181116	4.33E-05	0.000412515	0.342639135
ENSMUSG00000018500	Adora2b	11541	protein_coding	15.76454196	-0.342686723	0.359490704	0.953256146	0.340460305	0.561998621	0.342686723
ENSMUSG00000057132	Rpgrip1	77945	protein_coding	13.17024536	-0.342701509	0.512287199	0.66896364	0.503518673	0.708014489	0.342701509
ENSMUSG00000087385	Frg2f1	433752	protein_coding	39.3366598	-0.342762545	0.17953134	1.909207295	0.056235356	0.162838973	0.342762545
ENSMUSG00000030122	Ptms	69202	protein_coding	1857.836468	0.342824453	0.061814801	-5.545992946	2.92E-08	5.22E-07	0.342824453
ENSMUSG00000055541	Lair1	52855	protein_coding	2.705883775	-0.342862773	1.28821576	0.26615322	0.790121209	1	0.342862773

ensembl_gene_id	external_gene_name	entrezgene_id	gene_biotype	baseMean	log2FoldChange	lfcSE	stat	pvalue	padj	absLog2FC
ENSMUSG00000023206	Il15ra	16169	protein_coding	92.138871	-0.342916562	0.107476298	3.190624989	0.001419654	0.008514609	0.342916562
ENSMUSG00000029195	Klb	83379	protein_coding	104.4256924	-0.342947575	0.244287997	1.403865842	0.160358803	0.344813186	0.342947575
ENSMUSG00000000411	Tssk3	58864	protein_coding	2.158301263	-0.343101692	0.770106421	0.445525038	0.65594037	1	0.343101692
ENSMUSG00000054604	Cggbp1	106143	protein_coding	1354.661379	0.343139345	0.090175499	-3.805239217	0.000141667	0.00117209	0.343139345
ENSMUSG00000051413	Plagl2	54711	protein_coding	456.363815	0.343181875	0.067508084	-5.083567126	3.70E-07	5.60E-06	0.343181875
ENSMUSG00000023186	Vwa5a	67776	protein_coding	844.5287409	-0.343184019	0.110030997	3.118975817	0.001814809	0.010467953	0.343184019
ENSMUSG00000022799	Arhgap31	12549	protein_coding	124.0547977	0.343203079	0.113600645	-3.021136717	0.002518276	0.013704495	0.343203079
ENSMUSG00000022415	Syngr1	20972	protein_coding	24.14276917	-0.343314016	0.321821272	1.066784721	0.286069059	0.50453867	0.343314016
ENSMUSG00000054277	Arfgap3	66251	protein_coding	1678.476631	-0.343491191	0.079742615	4.307498427	1.65E-05	0.000174227	0.343491191
ENSMUSG00000028238	Atp6v0d2	242341	protein_coding	1.057275941	-0.343551436	1.799260992	0.190940302	0.848572365	1	0.343551436
ENSMUSG00000026601	Axdnd1	77352	protein_coding	5.687297155	0.343584538	0.723297344	-0.4750253	0.634768941	0.800822518	0.343584538
ENSMUSG00000020227	Irak3	73914	protein_coding	54.87642455	0.343635098	0.214929384	-1.598827908	0.109858846	0.264732086	0.343635098
ENSMUSG00000015605	Srf	20807	protein_coding	603.7535988	0.343718029	0.113525273	-3.027678514	0.002464401	0.013458042	0.343718029
ENSMUSG00000018932	Map2k3	26397	protein_coding	664.2537028	0.343745927	0.110618968	-3.107477262	0.001886915	0.010834671	0.343745927
ENSMUSG00000035150	Eif2s3x	26905	protein_coding	673.3378471	-0.34378638	0.123793281	2.777100473	0.005484622	0.026283536	0.34378638
ENSMUSG00000000126	Wnt9a	216795	protein_coding	2.57348249	-0.343805938	0.86540762	0.3972763	0.691163722	1	0.343805938
ENSMUSG00000026887	Mrrf	67871	protein_coding	166.884311	-0.343927164	0.126826136	2.711800392	0.006691888	0.030722778	0.343927164
ENSMUSG00000031757	Mt4	17752	protein_coding	1.146383389	0.343940545	1.425877599	-0.241213233	0.809389855	1	0.343940545
ENSMUSG00000078779	Zfp59	22717	protein_coding	63.68863395	-0.344005867	0.205123802	1.677064598	0.093529851	0.235306513	0.344005867
ENSMUSG00000062270	Morf4l1	21761	protein_coding	707.8509806	-0.344036446	0.120047212	2.865842855	0.004159006	0.020844259	0.344036446
ENSMUSG00000065546	Mir196a-1	387191	miRNA	38.601088	-0.34404645	0.245149561	1.403414505	0.16049327	0.345012479	0.34404645
ENSMUSG00000054385	Ceacam2	26367	protein_coding	114.0129266	-0.344088807	0.251936562	1.365775592	0.172009409	0.362441146	0.344088807
ENSMUSG00000053004	Hrh1	15465	protein_coding	103.4545315	-0.344127542	0.178311567	1.929922708	0.053616415	0.157212621	0.344127542
ENSMUSG00000021624	Cd180	17079	protein_coding	2.095844052	0.344131581	0.838290692	-0.410515809	0.681427609	1	0.344131581
ENSMUSG00000065616	Mir375	723900	miRNA	1.286860372	-0.344142716	1.073068562	0.320708972	0.748430947	1	0.344142716
ENSMUSG00000022219	Cideb	12684	protein_coding	73.52983364	-0.34416287	0.195163869	1.763455872	0.077823634	0.205991262	0.34416287
ENSMUSG00000001768	Rin2	74030	protein_coding	1003.603423	-0.344221389	0.07060838	4.875078402	1.09E-06	1.49E-05	0.344221389
ENSMUSG00000032402	Smad3	17127	protein_coding	827.9745455	0.344354164	0.07050594	-4.884044747	1.04E-06	1.43E-05	0.344354164
ENSMUSG00000066735	Vkorc1l1	69568	protein_coding	504.7301261	0.344433126	0.083297413	-4.134979863	3.55E-05	0.000346059	0.344433126
ENSMUSG00000054046	Klk13	626834	protein_coding	2.100461105	-0.344554053	2.368073076	0.145499755	0.884316309	1	0.344554053
ENSMUSG00000016427	Ndufa1	54405	protein_coding	1017.648649	-0.344610636	0.101259369	3.403246933	0.000665901	0.004503147	0.344610636
ENSMUSG00000014547	Wdfy2	268752	protein_coding	210.5125414	0.344699425	0.135910461	-2.536224378	0.011205491	0.046658467	0.344699425
ENSMUSG00000061798	Or4c106	258455	protein_coding	1.312567999	-0.344702227	2.369486238	0.145475513	0.884335448	1	0.344702227
ENSMUSG00000089133	Gm24771	115485992	snoRNA	1.343199634	0.344713418	0.971710887	-0.354748951	0.722777675	1	0.344713418
ENSMUSG00000052833	Sae1	56459	protein_coding	886.8062332	0.344821331	0.112385442	-3.068202828	0.002153504	0.012034844	0.344821331
ENSMUSG00000069520	Tmem19	67226	protein_coding	484.8328188	0.344831042	0.078280476	-4.405070851	1.06E-05	0.000117654	0.344831042
ENSMUSG00000030433	Sbk2	381836	protein_coding	1.363890202	-0.344845787	1.516577459	0.227384223	0.820124987	1	0.344845787
ENSMUSG00000097583	6430590A07Rik	619719	lncRNA	22.56253102	0.345129368	0.32468834	-1.062955842	0.287801977	0.506745053	0.345129368
ENSMUSG00000066009	Zfp987	626316	protein_coding	1.805231828	-0.345208357	0.752500752	0.458748189	0.646415005	1	0.345208357

ensembl_gene_id	external_gene_name	entrezgene_id	gene_biotype	baseMean	log2FoldChange	lfcSE	stat	pvalue	padj	absLog2FC
ENSMUSG00000020538	Srebf1	20787	protein_coding	1439.499973	0.345332054	0.103770711	-3.327837415	0.000875229	0.005694853	0.345332054
ENSMUSG00000078776	9530053A07Rik	319482	protein_coding	11.59892865	0.345348693	0.66031766	-0.523003872	0.600971559	0.777473116	0.345348693
ENSMUSG00000029878	Dbpht2	386753	lncRNA	5.929207797	-0.345383519	0.589611443	0.585781573	0.558022308	0.747120423	0.345383519
ENSMUSG00000029859	Epha1	13835	protein_coding	388.2805688	-0.345421699	0.136924834	2.522710366	0.011645427	0.048018124	0.345421699
ENSMUSG00000010721	Lmbr1	56873	protein_coding	225.8401383	-0.345495763	0.104599381	3.303038323	0.000956433	0.00613142	0.345495763
ENSMUSG00000009013	Dynll1	56455	protein_coding	1292.205892	0.345499564	0.077532941	-4.456164801	8.34E-06	9.54E-05	0.345499564
ENSMUSG00000022758	P2rx6	18440	protein_coding	4.838238797	-0.345541711	0.55434741	0.623330613	0.533067284	0.729516295	0.345541711
ENSMUSG00000027603	Ggt7	207182	protein_coding	3.844706428	0.345580011	0.782031211	-0.441900536	0.658561183	1	0.345580011
ENSMUSG00000027406	Idh3b	170718	protein_coding	2601.791602	-0.345681295	0.065753554	5.257226054	1.46E-07	2.34E-06	0.345681295
ENSMUSG00000049154	Fam183b	75429	protein_coding	4.200643105	0.345803344	0.545384042	-0.634054752	0.526045091	1	0.345803344
ENSMUSG00000042105	Inpp5f	101490	protein_coding	134.7144716	-0.345844839	0.127935821	2.703268214	0.006866132	0.031409224	0.345844839
ENSMUSG00000074653	Lrrc31	320352	protein_coding	185.2377497	-0.345849376	0.200857326	1.721865876	0.085093818	0.219864981	0.345849376
ENSMUSG00000057948	Unc13d	70450	protein_coding	291.6363164	-0.345943387	0.13648276	2.534703921	0.01125424	0.046796074	0.345943387
ENSMUSG00000068893	Sprr2a2	100303744	protein_coding	182.1677889	-0.345943638	0.409571839	0.844647032	0.398307941	0.620277218	0.345943638
ENSMUSG00000017314	Mpp2	50997	protein_coding	17.23928318	0.346097785	0.371762617	-0.930964464	0.351871947	0.574134308	0.346097785
ENSMUSG00000020668	Kif3c	16570	protein_coding	22.92728246	-0.346237137	0.272494041	1.270622784	0.203862878	0.406017465	0.346237137
ENSMUSG00000038807	Rap1gap2	380711	protein_coding	1551.29128	-0.346263225	0.066217791	5.229157023	1.70E-07	2.70E-06	0.346263225
ENSMUSG00000096935	1700113A16Rik	76642	lncRNA	10.59052926	0.346336151	0.342892634	-1.01004255	0.312474905	0.533520531	0.346336151
ENSMUSG00000003123	Lipe	16890	protein_coding	79.3060354	-0.346352363	0.154880557	2.236254636	0.025335096	0.088285245	0.346352363
ENSMUSG00000058925	Ccdc192	75444	protein_coding	1.70323952	0.346384551	0.892298343	-0.38819365	0.697872732	1	0.346384551
ENSMUSG00000030499	Kctd15	233107	protein_coding	18.03618501	-0.346411048	0.326697327	1.06034246	0.288988829	0.507895701	0.346411048
ENSMUSG00000096856	Gm10778	100233208	protein_coding	3.883453264	0.346414964	0.686712695	-0.504453997	0.613942382	1	0.346414964
ENSMUSG00000020460	Rps27a	78294	protein_coding	3153.000897	-0.346554033	0.145650413	2.379354967	0.017342966	0.065598201	0.346554033
ENSMUSG00000030056	Isy1	57905	protein_coding	168.3930389	0.346894072	0.131014055	-2.647762267	0.008102648	0.036008378	0.346894072
ENSMUSG00000052105	Mtcl1	68617	protein_coding	87.22911153	0.346912171	0.165198912	-2.099966437	0.035731794	0.115181503	0.346912171
ENSMUSG00000051627	H1f4	50709	protein_coding	4849.101536	0.346923182	0.093491578	-3.710742612	0.000206652	0.001628174	0.346923182
ENSMUSG00000078521	Aunip	69885	protein_coding	28.67691774	0.346931407	0.26583519	-1.305062011	0.191871733	0.38964213	0.346931407
ENSMUSG00000025544	Tm9sf2	68059	protein_coding	7385.485686	0.346963685	0.108843963	-3.187716383	0.001434011	0.008569101	0.346963685
ENSMUSG00000020354	Sgcd	24052	protein_coding	2.776965027	0.34705364	1.400212559	-0.247857825	0.804244412	1	0.34705364
ENSMUSG00000050017	Pitpnb	56305	protein_coding	335.9596429	0.347152647	0.085699774	-4.050800007	5.10E-05	0.000475421	0.347152647
ENSMUSG00000038685	Rtel1	269400	protein_coding	278.208045	0.347158925	0.100513188	-3.453864429	0.000552615	0.003848961	0.347158925
ENSMUSG00000042333	Tnfrsf14	230979	protein_coding	138.008373	-0.347184124	0.132099183	2.628207962	0.008583602	0.037639301	0.347184124
ENSMUSG00000014852	Adamts13	279028	protein_coding	7.838655263	-0.347194811	0.551921806	0.629065219	0.52930636	0.727020313	0.347194811
ENSMUSG00000025260	Hsd17b10	15108	protein_coding	612.3846672	-0.347278546	0.085996502	4.038286885	5.38E-05	0.00049841	0.347278546
ENSMUSG00000024978	Gpam	14732	protein_coding	437.2591239	-0.347281619	0.141891633	2.447513009	0.014384593	0.056411107	0.347281619
ENSMUSG00000028159	Dapp1	26377	protein_coding	116.7102726	0.347509934	0.189820633	-1.830727929	0.067141162	0.185177119	0.347509934
ENSMUSG00000050921	P2ry10	78826	protein_coding	13.35735642	-0.34766872	0.534073041	0.650975979	0.515061993	0.716272317	0.34766872
ENSMUSG00000058402	Zfp420	233058	protein_coding	35.18450881	-0.347673551	0.201889331	1.722099673	0.085051464	0.219837202	0.347673551
ENSMUSG00000020205	Phlda1	21664	protein_coding	110.58274	0.347692886	0.189220351	-1.837502592	0.066135734	0.183317181	0.347692886

Supplementary Table 1

ensembl_gene_id	external_gene_name	entrezgene_id	gene_biotype	baseMean	log2FoldChange	lfcSE	stat	pvalue	padj	absLog2FC
ENSMUSG00000029378	Areg	11839	protein_coding	1037.869365	-0.34775534	0.3442481	1.010188118	0.312405171	0.533456622	0.34775534
ENSMUSG00000039509	Nup133	234865	protein_coding	286.7905266	0.347759456	0.14382998	-2.417850966	0.01561247	0.060267491	0.347759456
ENSMUSG00000054932	Afp	11576	protein_coding	1.534981418	0.347832427	1.154261437	-0.301346312	0.763150429	1	0.347832427
ENSMUSG00000049517	Rps23	66475	protein_coding	3336.722903	-0.347859962	0.120790424	2.879863745	0.003978471	0.020104181	0.347859962
ENSMUSG00000024260	Sap130	269003	protein_coding	416.7302454	0.347871366	0.102401646	-3.397126702	0.000680974	0.004584431	0.347871366
ENSMUSG00000051228	Nyx	236690	protein_coding	5.662617772	-0.347889985	0.639880631	0.543679505	0.586662038	0.768287864	0.347889985
ENSMUSG00000020400	Tnip1	57783	protein_coding	988.7857202	-0.347907761	0.136490116	2.548959375	0.010804489	0.045254367	0.347907761
ENSMUSG00000095193	Gm20939	100044193	lncRNA	25.21277573	-0.347960265	0.229580616	1.515634339	0.129611839	0.297320449	0.347960265
ENSMUSG00000059900	Tmem40	94346	protein_coding	14.40508409	0.347977558	0.320349197	-1.086244516	0.277370827	0.494297255	0.347977558
ENSMUSG00000023947	Nfkbie	18037	protein_coding	32.46302274	-0.348082347	0.199107674	1.74821161	0.080427392	0.210845076	0.348082347
ENSMUSG00000026980	Ly75	17076	protein_coding	425.4134548	-0.348131063	0.084297797	4.129776534	3.63E-05	0.000352943	0.348131063
ENSMUSG00000005514	Por	18984	protein_coding	1218.840006	-0.348132454	0.140071893	2.485384096	0.012941172	0.05194233	0.348132454
ENSMUSG00000047344	Lancl3	236285	protein_coding	57.65016083	-0.3481577	0.18805857	1.851325895	0.064122683	0.179265615	0.3481577
ENSMUSG00000038437	Mllt6	246198	protein_coding	810.1890366	-0.348178642	0.09775389	3.5617881	0.000368338	0.00273381	0.348178642
ENSMUSG00000027894	Slc6a17	229706	protein_coding	3.647026552	-0.348197811	2.328118831	0.149561872	0.881110291	1	0.348197811
ENSMUSG00000015839	Nfe2l2	18024	protein_coding	2656.824541	-0.348219031	0.074199649	4.693001049	2.69E-06	3.39E-05	0.348219031
ENSMUSG00000019734	Tmc4	353499	protein_coding	2303.699576	-0.34823279	0.055662743	6.25611985	3.95E-10	9.15E-09	0.34823279
ENSMUSG00000026163	Sphkap	77629	protein_coding	1.966832869	-0.348241529	2.058507315	0.169171868	0.865661457	1	0.348241529
ENSMUSG00000041617	Ccdc74a	72315	protein_coding	11.9913792	0.348495088	0.393349812	-0.885967343	0.375635125	0.597844879	0.348495088
ENSMUSG00000040950	Mgl2	216864	protein_coding	1.160401234	0.348574296	2.294876222	-0.151892417	0.879271789	1	0.348574296
ENSMUSG00000024613	Tcof1	21453	protein_coding	468.9502343	0.34861853	0.108087224	-3.225344456	0.001258212	0.007701301	0.34861853
ENSMUSG00000025925	Terf1	21749	protein_coding	136.6388503	0.348717967	0.153001631	-2.279178103	0.02265648	0.08100219	0.348717967
ENSMUSG00000079037	Prnp	19122	protein_coding	1078.07028	-0.348750755	0.11397079	3.060001213	0.002213361	0.012282071	0.348750755
ENSMUSG00000020358	Hnrnpab	15384	protein_coding	2395.196954	0.348964589	0.067385854	-5.178603034	2.24E-07	3.48E-06	0.348964589
ENSMUSG00000029298	Gbp9	236573	protein_coding	8.819260975	0.349006463	0.440495341	-0.79230455	0.428183119	0.647232589	0.349006463
ENSMUSG00000063049	Ing2	69260	protein_coding	99.71774672	-0.349014454	0.10402774	3.355013338	0.000793612	0.005227463	0.349014454
ENSMUSG00000039795	Zfand1	66361	protein_coding	206.4562184	-0.349057424	0.120327373	2.9008979	0.003720951	0.0190648	0.349057424
ENSMUSG00000028914	Casp9	12371	protein_coding	234.146135	-0.34910763	0.082585003	4.227252149	2.37E-05	0.000239668	0.34910763
ENSMUSG00000040429	Mterf1a	545725	protein_coding	21.44467532	-0.349142498	0.266841716	1.30842547	0.190729038	0.388133006	0.349142498
ENSMUSG00000022416	Cacna1i	239556	protein_coding	4.06962951	-0.349183109	1.646788165	0.212038874	0.832076707	1	0.349183109
ENSMUSG00000026090	Cracdl	72097	protein_coding	761.7755874	-0.349353152	0.091466819	3.819452317	0.000133748	0.001112705	0.349353152
ENSMUSG00000050708	Ftl1	14325	protein_coding	5272.320298	-0.349361102	0.105276705	3.318503395	0.000905012	0.005849461	0.349361102
ENSMUSG00000028081	Rps3a1	20091	protein_coding	4645.301009	-0.349629833	0.144933349	2.412349089	0.015850098	0.061038631	0.349629833
ENSMUSG00000002718	Cse1l	110750	protein_coding	626.2203008	0.349680453	0.143269797	-2.44071298	0.0146583	0.057294003	0.349680453
ENSMUSG00000022763	Aifm3	72168	protein_coding	20.69217114	-0.349786271	0.282863205	1.236591629	0.216238729	0.421530084	0.349786271
ENSMUSG00000085007	Smim43	100503068	protein_coding	8.810297549	0.349816756	0.517282506	-0.676258624	0.498876458	0.703779756	0.349816756
ENSMUSG00000095217	H2bc15	319187	protein_coding	847.0901938	0.349882429	0.196051351	-1.784646865	0.074318615	0.199366082	0.349882429
ENSMUSG00000049164	Zfp518a	72672	protein_coding	479.3053875	-0.349947335	0.156816512	2.231571987	0.025643265	0.089020995	0.349947335
ENSMUSG00000025381	Cnpy2	56530	protein_coding	706.6510287	-0.349978797	0.105061963	3.331165594	0.000864831	0.005638305	0.349978797

ensembl_gene_id	external_gene_name	entrezgene_id	gene_biotype	baseMean	log2FoldChange	lfcSE	stat	pvalue	padj	absLog2FC
ENSMUSG00000066441	Rdh11	17252	protein_coding	395.9058985	0.350066971	0.147545642	-2.372601227	0.017663322	0.066596371	0.350066971
ENSMUSG00000070691	Runx3	12399	protein_coding	7.421216286	0.350216221	0.593055036	-0.590529039	0.554836023	0.744703672	0.350216221
ENSMUSG00000033256	Shf	435684	protein_coding	137.9026778	0.350254661	0.123415594	-2.838009763	0.004539579	0.022458542	0.350254661
ENSMUSG00000039462	Col10a1	12813	protein_coding	7.345775253	-0.350292002	0.482140291	0.726535427	0.467510598	0.681117777	0.350292002
ENSMUSG00000063430	Wscd2	320916	protein_coding	5.152776439	-0.350323427	1.260560326	0.277910878	0.781080773	0.890541867	0.350323427
ENSMUSG00000017774	Myo1c	17913	protein_coding	1170.482851	-0.350337015	0.051734747	6.771793387	1.27E-11	3.52E-10	0.350337015
ENSMUSG00000019891	Dcbld1	66686	protein_coding	415.0273793	0.350342809	0.082767185	-4.232870908	2.31E-05	0.000234331	0.350342809
ENSMUSG00000090693	Trim43a	547109	protein_coding	1.779807132	0.350361804	0.894704007	-0.39159521	0.695357328	1	0.350361804
ENSMUSG00000079481	Nhsl2	100042480	protein_coding	6.267534532	0.350535327	1.635786648	-0.214291593	0.830319674	0.914187552	0.350535327
ENSMUSG00000049676	Catsperg1	320225	protein_coding	7.624021209	0.350574887	0.712358375	-0.492132751	0.622625501	0.793088519	0.350574887
ENSMUSG00000028518	Prkaa2	108079	protein_coding	1740.606832	-0.350623494	0.085763116	4.088278408	4.35E-05	0.000413646	0.350623494
ENSMUSG00000037474	Dtl	76843	protein_coding	135.947748	0.35063343	0.178248191	-1.967107925	0.04917077	0.147129207	0.35063343
ENSMUSG00000019890	Nts	67405	protein_coding	89.28142259	-0.350852711	0.334099712	1.05014371	0.293652045	0.512889463	0.350852711
ENSMUSG00000020108	Ddit4	74747	protein_coding	752.6388956	0.35097501	0.130022226	-2.699346261	0.006947585	0.031711558	0.35097501
ENSMUSG00000085175	Gm11423	100503272	lncRNA	18.27416697	-0.350985805	0.275127432	1.275720865	0.20205421	0.404039964	0.350985805
ENSMUSG00000087497	2810001G20Rik	66456	lncRNA	41.41646092	-0.351042011	0.18320395	1.916126865	0.05534895	0.16072315	0.351042011
ENSMUSG00000018574	Acadvl	11370	protein_coding	1406.102522	-0.351134618	0.070334929	4.992322045	5.97E-07	8.65E-06	0.351134618
ENSMUSG00000031788	Kifc3	16582	protein_coding	367.6169216	0.351166145	0.116153124	-3.023303485	0.002500314	0.013622626	0.351166145
ENSMUSG00000022604	Cep97	74201	protein_coding	56.35596095	-0.351197761	0.155331166	2.260961342	0.023761651	0.084025676	0.351197761
ENSMUSG00000020072	Pbld2	67307	protein_coding	210.9804743	-0.351264371	0.102775506	3.417782948	0.000631334	0.004313552	0.351264371
ENSMUSG00000034177	Rnf43	207742	protein_coding	463.4665767	-0.351375734	0.08621873	4.075399108	4.59E-05	0.000434218	0.351375734
ENSMUSG00000024454	Hdac3	15183	protein_coding	785.6234261	0.351510112	0.063302053	-5.552902234	2.81E-08	5.04E-07	0.351510112
ENSMUSG00000084466	Gm22960	115488256	snRNA	0.599327296	0.35151934	1.59696346	-0.220117334	0.825779775	1	0.35151934
ENSMUSG00000031903	Pla2g15	192654	protein_coding	93.47732099	0.351539495	0.113150362	-3.106834915	0.00189102	0.010850695	0.351539495
ENSMUSG00000059291	Rpl11	67025	protein_coding	3009.443102	-0.351586576	0.13374213	2.628839364	0.008567682	0.037589453	0.351586576
ENSMUSG00000106514	Mir6976	102466778	miRNA	3.210492038	-0.351664531	0.641923075	0.547829709	0.583808837	1	0.351664531
ENSMUSG00000029344	Tpst2	22022	protein_coding	192.7116517	0.351725731	0.119787993	-2.936235274	0.003322222	0.017328863	0.351725731
ENSMUSG00000074259	Gramd2	546134	protein_coding	38.52623928	0.351761653	0.21859483	-1.609194752	0.107573764	0.260900739	0.351761653
ENSMUSG00000058975	Kcnc1	16502	protein_coding	7.782633571	-0.351774051	0.730682535	0.481432132	0.630209404	0.798287804	0.351774051
ENSMUSG00000021496	Pcbd2	72562	protein_coding	113.7070219	-0.351825851	0.153340523	2.29440884	0.021767026	0.078433486	0.351825851
ENSMUSG00000039579	Grin3a	242443	protein_coding	141.1328394	-0.351916155	0.320002265	1.099730201	0.271449692	0.487146296	0.351916155
ENSMUSG00000018042	Cyb5r3	109754	protein_coding	1270.979313	0.35195601	0.046046088	-7.643559485	2.11E-14	7.33E-13	0.35195601
ENSMUSG00000028165	Cisd2	67006	protein_coding	659.9207995	0.352007401	0.061350241	-5.737669422	9.60E-09	1.85E-07	0.352007401
ENSMUSG00000036985	Zdhhc9	208884	protein_coding	364.3501193	0.352015358	0.102761314	-3.425563041	0.000613527	0.004211046	0.352015358
ENSMUSG00000024654	Asrgl1	66514	protein_coding	66.06263678	0.35203249	0.187502427	-1.877482309	0.06045202	0.171677499	0.35203249
ENSMUSG00000024944	Arl2	56327	protein_coding	153.5096912	-0.352060368	0.096878099	3.634055298	0.000279001	0.002138015	0.352060368
ENSMUSG00000026942	Traf2	22030	protein_coding	312.8508368	0.352063563	0.090414923	-3.89386565	9.87E-05	0.000850344	0.352063563
ENSMUSG00000044254	Pcsk9	100102	protein_coding	265.1200426	-0.352114598	0.227839966	1.545447027	0.122238173	0.285320309	0.352114598
ENSMUSG00000028822	Tmem50a	71817	protein_coding	488.2967619	-0.35235745	0.09286083	3.794468032	0.00014796	0.00121563	0.35235745

ensembl_gene_id	external_gene_name	entrezgene_id	gene_biotype	baseMean	log2FoldChange	lfcSE	stat	pvalue	padj	absLog2FC
ENSMUSG00000086629	2810403D21Rik	69964	lncRNA	28.32632591	-0.352359755	0.238714332	1.476072892	0.139924325	0.313784669	0.352359755
ENSMUSG00000085887	Arhgap27os3	653030	lncRNA	24.75717895	-0.352467833	0.363237073	0.970352036	0.331871048	0.554292124	0.352467833
ENSMUSG00000078485	Plekhn1	231002	protein_coding	185.1140361	-0.352485367	0.131424862	2.682029576	0.007317699	0.033071834	0.352485367
ENSMUSG00000038570	Saxo2	330577	protein_coding	6.896763813	0.352494171	0.43275679	-0.814531809	0.415340357	0.635615852	0.352494171
ENSMUSG00000042677	Zc3h12a	230738	protein_coding	402.8412792	0.35254461	0.210748398	-1.672822256	0.094362292	0.236643719	0.35254461
ENSMUSG00000041995	Zbed3	72114	protein_coding	115.1686903	0.352558459	0.161145305	-2.187829542	0.028682021	0.097419765	0.352558459
ENSMUSG00000027246	Ell3	269344	protein_coding	5.357924613	-0.352696046	0.4282429	0.823588777	0.410173255	0.630552252	0.352696046
ENSMUSG00000063146	Clip2	269713	protein_coding	1318.705423	0.352747826	0.076573963	-4.606628829	4.09E-06	5.00E-05	0.352747826
ENSMUSG00000053907	Mat2a	232087	protein_coding	2633.791637	-0.352889003	0.108744626	3.245116693	0.001174025	0.0072831	0.352889003
ENSMUSG00000020806	Rhbdf2	217344	protein_coding	209.2401804	-0.353076302	0.102183144	3.455328237	0.000549623	0.003837834	0.353076302
ENSMUSG00000034799	Unc13a	382018	protein_coding	33.43981098	-0.353127858	0.417929059	0.84494689	0.398140493	0.62019321	0.353127858
ENSMUSG00000091475	Cerox1	72834	lncRNA	1.385414542	-0.353138341	1.154471662	0.305887406	0.759690376	1	0.353138341
ENSMUSG00000055401	Fbxo6	50762	protein_coding	187.1528825	-0.353158595	0.115446101	3.059077727	0.002220195	0.012311721	0.353158595
ENSMUSG00000047787	Flrt1	396184	protein_coding	11.9978969	-0.353208784	0.469246768	0.752714367	0.451621575	0.668047177	0.353208784
ENSMUSG00000038224	Serpinf2	18816	protein_coding	11.66815033	0.353320148	0.395291081	-0.893822716	0.371416753	0.593868753	0.353320148
ENSMUSG00000027195	Hsd17b12	56348	protein_coding	1137.909397	0.353370118	0.102855859	-3.43558571	0.000591274	0.004080362	0.353370118
ENSMUSG00000034472	Rasd2	75141	protein_coding	261.8393969	-0.353387711	0.283730115	1.24550653	0.212945617	0.417696153	0.353387711
ENSMUSG00000047631	Apof	103161	protein_coding	1.956177607	-0.353400241	1.429002684	0.247305512	0.804671793	1	0.353400241
ENSMUSG00000034584	Exph5	320051	protein_coding	896.2302551	-0.35349995	0.104516646	3.382235876	0.000718984	0.004789552	0.35349995
ENSMUSG00000048758	Rpl29	19944	protein_coding	2303.426833	-0.353580718	0.142267081	2.485330517	0.01294312	0.05194233	0.353580718
ENSMUSG00000030486	Zfp108	54678	protein_coding	29.63454191	-0.353808633	0.225532773	1.568768159	0.11670197	0.276145055	0.353808633
ENSMUSG00000075569	Rsph10b	18861	protein_coding	2.161217055	0.353951842	0.776347504	-0.455919341	0.648447981	1	0.353951842
ENSMUSG00000036327	Qsox2	227638	protein_coding	374.595413	0.353977425	0.172259551	-2.054907392	0.03988795	0.125701628	0.353977425
ENSMUSG00000032359	Ctsh	13036	protein_coding	479.914797	-0.35403182	0.086190528	4.107549018	4.00E-05	0.000384378	0.35403182
ENSMUSG00000030720	Cln3	12752	protein_coding	261.8821675	-0.354088317	0.089558796	3.953696697	7.70E-05	0.000685437	0.354088317
ENSMUSG00000024583	Txnl1	53382	protein_coding	874.4860714	0.354098756	0.117148031	-3.022660764	0.00250563	0.013647086	0.354098756
ENSMUSG00000029267	Mtf2	17765	protein_coding	393.7188048	0.354109098	0.08146585	-4.346718279	1.38E-05	0.00014896	0.354109098
ENSMUSG00000021990	Spata13	219140	protein_coding	698.7758846	-0.35418316	0.105488405	3.357555359	0.00078635	0.005188088	0.35418316
ENSMUSG00000033105	Lss	16987	protein_coding	755.4421813	0.354211154	0.103449781	-3.423991347	0.000617086	0.004230202	0.354211154
ENSMUSG00000029410	Ppef2	19023	protein_coding	3.116932454	-0.354363248	0.831069037	0.426394478	0.669820425	1	0.354363248
ENSMUSG00000025499	Hras	15461	protein_coding	760.4029002	0.354954551	0.05916076	-5.999830802	1.98E-09	4.21E-08	0.354954551
ENSMUSG00000069808	Tlcd3a	116972	protein_coding	494.2554124	0.355033847	0.114156666	-3.110057966	0.001870506	0.010747927	0.355033847
ENSMUSG00000085436	Zfp335os	629777	lncRNA	14.15310467	0.355035605	0.291761199	-1.216870528	0.223653481	0.43083733	0.355035605
ENSMUSG00000000740	Rpl13	270106	protein_coding	4237.277656	-0.355193453	0.121144275	2.931987106	0.003368007	0.017528923	0.355193453
ENSMUSG00000029028	Lrrc47	72946	protein_coding	381.8416015	0.355201264	0.067641278	-5.251250057	1.51E-07	2.42E-06	0.355201264
ENSMUSG00000031732	Phlpp2	244650	protein_coding	585.2810748	-0.355212281	0.174469735	2.035953583	0.041755016	0.130053252	0.355212281
ENSMUSG00000016239	Lonrf3	74365	protein_coding	93.15501068	0.355292817	0.154705366	-2.296577207	0.0216429	0.078139671	0.355292817
ENSMUSG00000052837	Junb	16477	protein_coding	5504.789745	-0.355331694	0.163468503	2.173701276	0.029727577	0.100208451	0.355331694
ENSMUSG00000099825	1810012K16Rik	69108	lncRNA	3.483418035	-0.355476897	0.606229919	0.586373067	0.557624839	1	0.355476897

Supplementary Table 1

ensembl_gene_id	external_gene_name	entrezgene_id	gene_biotype	baseMean	log2FoldChange	lfcSE	stat	pvalue	padj	absLog2FC
ENSMUSG00000087408	Cers1	14559	protein_coding	2.17285123	0.355499576	1.390554731	-0.255653063	0.798218741	1	0.355499576
ENSMUSG00000087440	4930577N17Rik	67746	TEC	14.82732485	0.355535808	0.274756783	-1.294001935	0.195664752	0.394964884	0.355535808
ENSMUSG00000029761	Cald1	109624	protein_coding	19.29350427	0.355634978	0.315841282	-1.1259927	0.260168609	0.473905185	0.355634978
ENSMUSG00000112963	Gm6093	619715	lncRNA	2.169034596	-0.355759482	2.368965223	0.150175055	0.880626506	1	0.355759482
ENSMUSG00000028573	Fggy	75578	protein_coding	101.5934836	-0.355782765	0.131570647	2.704119606	0.006848563	0.031346224	0.355782765
ENSMUSG00000050022	Amz1	231842	protein_coding	5.562665818	0.355813514	0.635018079	-0.560320289	0.575260991	0.760475505	0.355813514
ENSMUSG00000029769	Ccdc136	232664	protein_coding	5.522273823	-0.356143431	0.658511382	0.540831093	0.588624009	0.769577776	0.356143431
ENSMUSG00000043644	0610009L18Rik	66838	lncRNA	27.30046995	-0.356237644	0.217930367	1.63463977	0.102124586	0.251451529	0.356237644
ENSMUSG00000031161	Hdac6	15185	protein_coding	229.6286234	0.35627635	0.091972354	-3.87373308	0.000107181	0.000916141	0.35627635
ENSMUSG00000058706	0610030E20Rik	68364	protein_coding	218.4395863	-0.356318573	0.095664175	3.724681413	0.000195562	0.001548948	0.356318573
ENSMUSG00000116632	Magef1	76222	transcribed_processed_pseudogene	25.90277464	-0.356357595	0.203913255	1.747594071	0.080534343	0.210915627	0.356357595
ENSMUSG00000027224	Duoxa1	213696	protein_coding	14.95224597	0.356549314	0.420518885	-0.847879434	0.396505119	0.618855346	0.356549314
ENSMUSG00000001576	Ergic1	67458	protein_coding	1411.913263	0.356610932	0.122734849	-2.90553935	0.003666207	0.018819941	0.356610932
ENSMUSG00000028457	Atp8b5	320571	protein_coding	24.79013147	-0.356622989	0.279555212	1.275679987	0.202068666	0.404039964	0.356622989
ENSMUSG00000005899	Smpd4	77626	protein_coding	305.4861794	0.356660698	0.068279876	-5.223511175	1.76E-07	2.77E-06	0.356660698
ENSMUSG00000039105	Atp6v1g1	66290	protein_coding	704.216962	-0.356669561	0.105904786	3.36783233	0.000757617	0.005018564	0.356669561
ENSMUSG00000023072	Cep89	72140	protein_coding	248.1485723	0.356809937	0.079150913	-4.507969921	6.55E-06	7.64E-05	0.356809937
ENSMUSG00000026049	Tex30	75623	protein_coding	66.76533749	0.356877088	0.170538859	-2.092643808	0.036380963	0.116818048	0.356877088
ENSMUSG00000031508	Ankrd10	102334	protein_coding	614.3969833	-0.356986066	0.103725324	3.441648131	0.000578182	0.004001262	0.356986066
ENSMUSG00000049897	Stkld1	279029	protein_coding	5.319311173	-0.357102687	0.562094579	0.635307118	0.525228132	0.723645701	0.357102687
ENSMUSG00000024232	Bambi	68010	protein_coding	65.10629957	0.35714635	0.209557901	-1.704284824	0.088327889	0.225622081	0.35714635
ENSMUSG00000041966	Dcaf17	75763	protein_coding	223.9542386	-0.357287771	0.099695053	3.583806418	0.000338623	0.002533766	0.357287771
ENSMUSG00000115816	Gm34589	102637893	lncRNA	3.243340397	0.357325267	0.982931235	-0.36353028	0.716208798	1	0.357325267
ENSMUSG00000034480	Diaph2	54004	protein_coding	190.4627504	-0.357378827	0.090291432	3.958059128	7.56E-05	0.000674132	0.357378827
ENSMUSG00000043398	Gpr135	238252	protein_coding	2.617281806	0.357475585	0.638747313	-0.559651019	0.575717498	1	0.357475585
ENSMUSG00000033610	Pank1	75735	protein_coding	409.5749029	-0.357483776	0.08536185	4.187863494	2.82E-05	0.000280811	0.357483776
ENSMUSG00000026921	Egfl7	353156	protein_coding	19.85732057	0.357536595	0.407527653	-0.877330882	0.380306943	0.602493175	0.357536595
ENSMUSG00000006498	Ptbp1	19205	protein_coding	2524.212974	0.357607024	0.062889004	-5.686320392	1.30E-08	2.45E-07	0.357607024
ENSMUSG00000109450	Gm39043	105242985	lncRNA	1.275980282	0.357821229	2.319062956	-0.154295608	0.87737666	1	0.357821229
ENSMUSG00000006920	Ezh1	14055	protein_coding	570.4435737	-0.357921085	0.066691086	5.366850469	8.01E-08	1.34E-06	0.357921085
ENSMUSG00000051985	Igfn1	226438	protein_coding	7.095364019	0.357977556	0.830585848	-0.430994047	0.666472699	0.820495762	0.357977556
ENSMUSG00000067430	Zfp763	73451	protein_coding	67.86384842	-0.358057571	0.149898926	2.388660024	0.016909942	0.064269456	0.358057571
ENSMUSG00000073164	2410018L13Rik	69732	lncRNA	3.10327556	0.358068425	0.651225324	-0.549837993	0.582430497	1	0.358068425
ENSMUSG00000046324	Ermp1	226090	protein_coding	1489.593293	-0.358088825	0.080755928	4.434211008	9.24E-06	0.000104668	0.358088825
ENSMUSG00000026288	Inpp5d	16331	protein_coding	73.40425278	-0.358100587	0.210805483	1.698725199	0.089370973	0.227592128	0.358100587
ENSMUSG00000034919	Ttc22	230576	protein_coding	1043.399703	0.358113362	0.146034134	-2.452257921	0.014196287	0.055871658	0.358113362
ENSMUSG00000010911	Apip	56369	protein_coding	83.67163471	0.35824535	0.129711695	-2.761858512	0.005747338	0.027285164	0.35824535
ENSMUSG00000027078	Ube2l6	56791	protein_coding	74.12809324	-0.358318851	0.23088581	1.551931021	0.120678729	0.283161202	0.358318851
ENSMUSG00000029627	Zkscan14	67235	protein_coding	95.98420759	-0.358433428	0.141985361	2.524439318	0.011588301	0.047830343	0.358433428

ensembl_gene_id	external_gene_name	entrezgene_id	gene_biotype	baseMean	log2FoldChange	lfcSE	stat	pvalue	padj	absLog2FC
ENSMUSG00000087648	E130018N17Rik	77017	lncRNA	0.844759267	-0.358477698	1.342347163	0.267052897	0.789428434	1	0.358477698
ENSMUSG00000040502	Marchf9	216438	protein_coding	51.29466239	-0.35859327	0.185640807	1.931651104	0.053402584	0.156808369	0.35859327
ENSMUSG00000070524	Fcrlb	435653	protein_coding	0.994003205	0.358622093	2.264973191	-0.158333924	0.874193678	1	0.358622093
ENSMUSG00000019055	Plod1	18822	protein_coding	165.1631335	0.35875691	0.147993586	-2.424138234	0.015344763	0.059503296	0.35875691
ENSMUSG00000036580	Spg20	229285	protein_coding	158.4447611	0.358771191	0.120418547	-2.979368206	0.002888435	0.015401876	0.358771191
ENSMUSG00000097494	4933406C10Rik	74076	lncRNA	30.36306031	0.358776799	0.207411249	-1.729784676	0.083668753	0.216896136	0.358776799
ENSMUSG00000026114	Cnga3	12790	protein_coding	3.137280409	-0.358820897	0.665247915	0.539379212	0.589625222	1	0.358820897
ENSMUSG00000025348	Itga7	16404	protein_coding	377.5038996	-0.358826737	0.148693962	2.413189701	0.015813587	0.060929904	0.358826737
ENSMUSG00000057863	Rpl36	54217	protein_coding	2298.916531	-0.358833261	0.112037063	3.202808538	0.001360944	0.00822343	0.358833261
ENSMUSG00000030058	Copg1	54161	protein_coding	2091.569461	0.358847273	0.078979336	-4.543559025	5.53E-06	6.55E-05	0.358847273
ENSMUSG00000018566	Slc2a4	20528	protein_coding	15.29549454	0.358858365	0.31230306	-1.149070921	0.250526738	0.462773887	0.358858365
ENSMUSG00000025532	Crcp	12909	protein_coding	197.0150354	0.35887156	0.079219909	-4.53006783	5.90E-06	6.93E-05	0.35887156
ENSMUSG00000036002	Atosb	230088	protein_coding	600.7783592	0.358925418	0.135114687	-2.656450057	0.007896815	0.035293098	0.358925418
ENSMUSG00000064778	Snord59a	115487514	snoRNA	2.021526508	0.358975826	0.751793938	-0.477492312	0.633011594	1	0.358975826
ENSMUSG00000041020	Map7d2	78283	protein_coding	2.588392455	0.358995413	1.280999718	-0.280246286	0.779288558	1	0.358995413
ENSMUSG00000020034	Tcp11l2	216198	protein_coding	78.71791407	-0.359002722	0.204298233	1.757248294	0.078875497	0.207979566	0.359002722
ENSMUSG00000074059	Fbxw18	546161	protein_coding	1.252245074	0.35907496	2.667387635	-0.134616715	0.892914925	1	0.35907496
ENSMUSG00000035615	Frmpd1	666060	protein_coding	1.906504513	-0.359207925	1.608180963	0.223362876	0.823253095	1	0.359207925
ENSMUSG00000031697	Orc6	56452	protein_coding	119.7567386	0.35952199	0.114319286	-3.144893598	0.001661472	0.009712409	0.35952199
ENSMUSG00000017929	B4galt5	56336	protein_coding	714.5709613	-0.359573837	0.088716675	4.053058099	5.06E-05	0.000471385	0.359573837
ENSMUSG00000046085	4931422A03Rik	70986	lncRNA	5.853868128	0.359586301	0.676458076	-0.53157219	0.59502233	0.773528957	0.359586301
ENSMUSG00000014747	Ankrd53	75305	protein_coding	2.561874909	-0.359625845	0.874077946	0.411434525	0.680753942	1	0.359625845
ENSMUSG00000019256	Ahr	11622	protein_coding	181.1430776	-0.359646522	0.099316273	3.621224524	0.000293212	0.002234472	0.359646522
ENSMUSG00000036925	Mucl2	20770	protein_coding	1.481793215	0.359735414	2.680059852	-0.134226634	0.893223365	1	0.359735414
ENSMUSG00000075334	Rprm	67874	protein_coding	19.60200025	0.359977983	0.357690542	-1.006395028	0.314225584	0.535446743	0.359977983
ENSMUSG00000091144	Phf11c	628705	protein_coding	20.37515397	0.36014316	0.296258774	-1.215637111	0.224123191	0.431271309	0.36014316
ENSMUSG00000020462	Cfap36	216618	protein_coding	146.5046556	0.360215577	0.108256678	-3.32742132	0.000876537	0.005701118	0.360215577
ENSMUSG00000038692	Hoxb4	15412	protein_coding	45.96910916	-0.360280872	0.323781259	1.11272923	0.265824738	0.480866438	0.360280872
ENSMUSG00000034951	Cog7	233824	protein_coding	530.4384339	0.360318651	0.081349318	-4.429276876	9.45E-06	0.000106652	0.360318651
ENSMUSG00000030177	Ccdc77	67200	protein_coding	117.1861639	0.3603478	0.130866366	-2.753555476	0.005895178	0.027823083	0.3603478
ENSMUSG00000028603	Scp2	20280	protein_coding	1506.66314	-0.360442928	0.114031884	3.160896022	0.001572846	0.009273111	0.360442928
ENSMUSG00000079071	Slc28a2b	381417	protein_coding	0.822665944	0.360457426	2.153715791	-0.167365363	0.867082577	1	0.360457426
ENSMUSG00000022391	Rangap1	19387	protein_coding	1534.704299	0.360585148	0.118204151	-3.050528626	0.002284389	0.012608423	0.360585148
ENSMUSG00000022877	Hrg	94175	protein_coding	4.617059125	-0.360585956	0.732289728	0.492408868	0.622430332	0.792979237	0.360585956
ENSMUSG00000073805	Insyn2a	627214	protein_coding	25.00545805	-0.360720444	0.247623526	1.456729297	0.145191119	0.322338142	0.360720444
ENSMUSG00000091337	Eid1	58521	protein_coding	154.0341746	-0.360838848	0.2057783	1.753532069	0.07951072	0.208982974	0.360838848
ENSMUSG00000032849	Abcc4	239273	protein_coding	191.7466898	0.361022037	0.175332388	-2.059072154	0.039487327	0.124778744	0.361022037
ENSMUSG00000031853	Map3k21	234878	protein_coding	999.7690053	-0.361139119	0.047159683	7.657793648	1.89E-14	6.60E-13	0.361139119
ENSMUSG00000079339	Ifit1bl1	667373	protein_coding	893.4063939	-0.361249044	0.341061612	1.059189984	0.289513266	0.508294925	0.361249044

ensembl_gene_id	external_gene_name	entrezgene_id	gene_biotype	baseMean	log2FoldChange	lfcSE	stat	pvalue	padj	absLog2FC
ENSMUSG00000068966	Zbtb34	241311	protein_coding	69.53147174	-0.361287847	0.189373077	1.907809986	0.05641578	0.163304151	0.361287847
ENSMUSG00000022686	B3gnt5	108105	protein_coding	1168.683129	0.361301907	0.349214656	-1.034612666	0.300849836	0.520943084	0.361301907
ENSMUSG00000053965	Pde5a	242202	protein_coding	1301.162266	-0.361306066	0.065472405	5.518448062	3.42E-08	6.04E-07	0.361306066
ENSMUSG00000074240	Cib3	234421	protein_coding	0.741128	-0.361309263	1.51163196	0.239019333	0.811090592	1	0.361309263
ENSMUSG00000031533	Mrps31	57312	protein_coding	380.45752	-0.361377994	0.080727024	4.476543004	7.59E-06	8.75E-05	0.361377994
ENSMUSG00000028041	Adam15	11490	protein_coding	595.1730466	-0.361492351	0.091641555	3.944633518	7.99E-05	0.000708826	0.361492351
ENSMUSG00000032561	Acpp	56318	protein_coding	391.2246115	-0.361533542	0.273779712	1.320527148	0.186659078	0.382529046	0.361533542
ENSMUSG00000046574	Prr12	233210	protein_coding	120.5012842	0.361542477	0.294027806	-1.229620021	0.218839429	0.425016386	0.361542477
ENSMUSG00000047810	Ccdc88b	78317	protein_coding	35.3234608	0.361701309	0.308681275	-1.171763037	0.241292207	0.451933708	0.361701309
ENSMUSG00000085347	Hk1os	70162	lncRNA	6.535562295	0.361765089	0.473754574	-0.763612868	0.445097979	0.662372535	0.361765089
ENSMUSG00000020290	Xpo1	103573	protein_coding	1209.672737	0.361798521	0.108846106	-3.323945472	0.000887535	0.005758032	0.361798521
ENSMUSG00000057098	Ebf1	13591	protein_coding	5.203127784	0.36191929	1.976591579	-0.183102718	0.854717423	0.929155653	0.36191929
ENSMUSG00000006362	Cbfa2t3	12398	protein_coding	181.5269668	-0.361963822	0.104721596	3.456439117	0.000547363	0.003828526	0.361963822
ENSMUSG00000038679	Trps1	83925	protein_coding	4.203120742	0.362109116	1.003784831	-0.360743762	0.718291007	1	0.362109116
ENSMUSG00000034429	Zfp707	69020	protein_coding	127.8979956	-0.362190521	0.132394388	2.735693915	0.00622489	0.02894083	0.362190521
ENSMUSG00000037851	Iars	105148	protein_coding	678.9028413	0.362209272	0.132181722	-2.740237209	0.006139486	0.028672926	0.362209272
ENSMUSG00000043372	Hexim2	71059	protein_coding	47.16847377	0.362209931	0.204808444	-1.768530264	0.076972297	0.204491718	0.362209931
ENSMUSG00000039016	Timm8b	30057	protein_coding	441.1677435	-0.362225916	0.113637357	3.187560189	0.001434786	0.008569101	0.362225916
ENSMUSG00000044768	Macir	52392	protein_coding	647.2872067	-0.362357196	0.097165884	3.729263614	0.00019204	0.001525421	0.362357196
ENSMUSG00000021835	Bmp4	12159	protein_coding	1.105873731	0.362360113	1.751121076	-0.206930359	0.836064247	1	0.362360113
ENSMUSG00000025226	Fbxl15	68431	protein_coding	64.48056532	-0.362447163	0.181380565	1.99826902	0.045687502	0.139280674	0.362447163
ENSMUSG00000023868	Pde10a	23984	protein_coding	6.331258319	-0.362535489	1.181699505	0.306791606	0.759001998	0.878912418	0.362535489
ENSMUSG00000043313	Pcdhb19	93890	protein_coding	4.481621139	0.362702124	0.628095351	-0.577463475	0.563626405	0.750925894	0.362702124
ENSMUSG00000031785	Adgrg1	14766	protein_coding	622.7651661	0.36274867	0.084794794	-4.277959216	1.89E-05	0.000195572	0.36274867
ENSMUSG00000040097	Flywch1	224613	protein_coding	316.3528538	0.362769133	0.079472286	-4.564725029	5.00E-06	5.97E-05	0.362769133
ENSMUSG00000008438	Adam21	56622	protein_coding	1.630940061	0.362818828	1.352317505	-0.268294115	0.788472936	1	0.362818828
ENSMUSG00000025314	Ptprj	19271	protein_coding	1476.531566	-0.363008651	0.07595705	4.779130475	1.76E-06	2.30E-05	0.363008651
ENSMUSG00000091311	Spata31d1b	238662	protein_coding	1.756207656	-0.363032201	2.251319296	0.161253093	0.871894066	1	0.363032201
ENSMUSG00000029096	Htra3	78558	protein_coding	2.119687911	0.363145202	1.102119217	-0.329497205	0.741779906	1	0.363145202
ENSMUSG00000024878	Cbwd1	226043	protein_coding	143.1361766	0.363233685	0.112501389	-3.228704013	0.001243525	0.007622708	0.363233685
ENSMUSG00000027330	Cdc25b	12531	protein_coding	261.2853911	0.363292342	0.132090113	-2.750337124	0.005953398	0.028025774	0.363292342
ENSMUSG00000005267	Zfp287	170740	protein_coding	34.20938363	0.36330209	0.216813243	-1.675645292	0.093807692	0.235825882	0.36330209
ENSMUSG00000022797	Tfrc	22042	protein_coding	3689.141464	0.363321044	0.196994821	-1.844317743	0.065136847	0.181269925	0.363321044
ENSMUSG00000057378	Ryr3	20192	protein_coding	15.04678787	-0.363326489	0.738881634	0.491724889	0.622913841	0.793281643	0.363326489
ENSMUSG00000060572	Mfap2	17150	protein_coding	2.426237999	0.363339231	0.941275789	-0.386007199	0.699491337	1	0.363339231
ENSMUSG00000006301	Tmbim1	69660	protein_coding	1959.161183	-0.363354537	0.059147964	6.143145311	8.09E-10	1.80E-08	0.363354537
ENSMUSG00000038623	Tm6sf1	107769	protein_coding	3.430445202	-0.36339921	1.009453447	0.359996006	0.71885012	1	0.36339921
ENSMUSG00000102252	Snrpn	20646	protein_coding	90.11721924	-0.363446474	0.148189803	2.452574112	0.014183816	0.055849197	0.363446474
ENSMUSG00000021868	Ppif	105675	protein_coding	1024.298652	-0.36377097	0.140157994	2.595435038	0.009447126	0.040712379	0.36377097

Supplementary Table 1

ensembl_gene_id	external_gene_name	entrezgene_id	gene_biotype	baseMean	log2FoldChange	lfcSE	stat	pvalue	padj	absLog2FC
ENSMUSG00000110682	A530010L16Rik	382036	lncRNA	1.051893037	-0.363817815	2.267273745	0.160464882	0.872514883	1	0.363817815
ENSMUSG00000037583	Nr0b2	23957	protein_coding	0.740836427	0.363824673	1.485811428	-0.244865981	0.806560203	1	0.363824673
ENSMUSG00000017009	Sdc4	20971	protein_coding	4041.24277	-0.363882613	0.093014715	3.912097293	9.15E-05	0.000797335	0.363882613
ENSMUSG00000086315	Arl4aos	100043606	lncRNA	4.968866543	-0.363956028	0.561746993	0.64790027	0.517049456	0.717711842	0.363956028
ENSMUSG00000026012	Cd28	12487	protein_coding	3.38776774	0.36396969	0.848792151	-0.428808972	0.668062249	1	0.36396969
ENSMUSG00000015335	Zdhhc12	66220	protein_coding	197.5562389	0.364046426	0.1306517	-2.786388746	0.005329891	0.025698634	0.364046426
ENSMUSG00000035161	Ints6	18130	protein_coding	595.3029205	0.364096697	0.102687873	-3.545664014	0.000391625	0.002879422	0.364096697
ENSMUSG00000020723	Cacng4	54377	protein_coding	3.811869888	-0.364151483	1.016625298	0.358196361	0.72019637	1	0.364151483
ENSMUSG00000036620	Mgat4b	103534	protein_coding	3186.738883	0.364188809	0.059358472	-6.135414147	8.49E-10	1.89E-08	0.364188809
ENSMUSG00000026009	Icos	54167	protein_coding	1.315116046	0.364274993	1.966191913	-0.185269297	0.853017819	1	0.364274993
ENSMUSG00000032398	Snapc5	330959	protein_coding	324.9787641	-0.364277342	0.083580947	4.358377779	1.31E-05	0.00014217	0.364277342
ENSMUSG00000062647	Rpl7a	27176	protein_coding	7851.891397	-0.364298193	0.10429048	3.493110704	0.000477429	0.003410008	0.364298193
ENSMUSG00000111748	Gm34885	102638284	lncRNA	2.608768835	0.364340214	0.762232615	-0.477990848	0.632656719	1	0.364340214
ENSMUSG00000000125	Wnt3	22415	protein_coding	1.172382201	-0.364404492	1.143027307	0.318806463	0.749873277	1	0.364404492
ENSMUSG00000030523	Trpm1	17364	protein_coding	5.896013806	0.364507488	1.67771845	-0.217263802	0.828002765	0.913297021	0.364507488
ENSMUSG00000031010	Usp9x	22284	protein_coding	3161.038092	0.364525237	0.078142459	-4.664880523	3.09E-06	3.85E-05	0.364525237
ENSMUSG00000097133	Gm26628	102632638	lncRNA	5.672486284	0.364569232	0.548442973	-0.664734986	0.506220013	0.710135685	0.364569232
ENSMUSG00000031139	Mcf2	109904	protein_coding	0.968284731	-0.364627328	2.621964826	0.139066445	0.889397643	1	0.364627328
ENSMUSG00000042857	Gm9776	328309	lncRNA	27.59171516	-0.364629863	0.216445742	1.684624794	0.092060985	0.232532135	0.364629863
ENSMUSG00000033498	Strc	140476	protein_coding	28.88549158	0.364666112	0.331178106	-1.101117815	0.270845385	0.48643183	0.364666112
ENSMUSG00000059898	Dsc3	13507	protein_coding	1.427791439	-0.364774476	1.228632038	0.296894811	0.766546818	1	0.364774476
ENSMUSG00000062209	Erbb4	13869	protein_coding	4.333702883	0.364785995	1.432303337	-0.254684874	0.7989665	1	0.364785995
ENSMUSG00000023067	Cdkn1a	12575	protein_coding	1579.839772	0.364809767	0.18528591	-1.968901828	0.048964366	0.1468042	0.364809767
ENSMUSG00000044934	Zfp367	238673	protein_coding	177.0728838	0.364902485	0.195170644	-1.869658666	0.061531233	0.173816344	0.364902485
ENSMUSG00000034120	Srsf2	20382	protein_coding	1667.331754	0.365132936	0.061188521	-5.967343666	2.41E-09	5.09E-08	0.365132936
ENSMUSG00000002257	Def6	23853	protein_coding	14.67321342	0.3652394	0.343825125	-1.062282461	0.288107474	0.506958639	0.3652394
ENSMUSG00000022339	Ebag9	55960	protein_coding	287.3136681	0.365378522	0.097733376	-3.738523504	0.000185104	0.001483889	0.365378522
ENSMUSG00000047221	Fam185a	330050	protein_coding	168.8650543	0.365414807	0.111247814	-3.284692019	0.001020939	0.006462165	0.365414807
ENSMUSG00000029560	Snx8	231834	protein_coding	73.45353405	0.365497185	0.168543682	-2.168560587	0.03011606	0.101311187	0.365497185
ENSMUSG00000052934	Fbxo31	76454	protein_coding	214.671038	-0.365511835	0.107045586	3.41454372	0.000638889	0.004347186	0.365511835
ENSMUSG00000109179	Gm35339	102638882	protein_coding	28.11538796	0.365572437	0.313089902	-1.167627682	0.242956979	0.454124681	0.365572437
ENSMUSG00000064853	Gm23442	115488838	snoRNA	350.4057424	0.365572869	0.231760576	-1.577372975	0.11470973	0.272838324	0.365572869
ENSMUSG00000049686	Orai1	109305	protein_coding	286.5680725	0.365691698	0.092889116	-3.936862727	8.26E-05	0.000728644	0.365691698
ENSMUSG00000040495	Chrm4	12672	protein_coding	2.673958511	-0.365734776	0.655603209	0.557859954	0.576940019	1	0.365734776
ENSMUSG00000103472	Pcdhga7	93715	protein_coding	11.91914712	-0.365748765	0.324318727	1.127744822	0.259427702	0.47292075	0.365748765
ENSMUSG00000022914	Brwd1	93871	protein_coding	1106.817086	0.365872768	0.097925156	-3.736249029	0.000186786	0.00149302	0.365872768
ENSMUSG00000048478	Spata33	320869	protein_coding	5.392142092	-0.36587796	0.570220392	0.641643064	0.521104965	0.720791305	0.36587796
ENSMUSG00000034327	Kctd9	105440	protein_coding	228.9717852	0.366007939	0.106464764	-3.437831702	0.000586392	0.004051748	0.366007939
ENSMUSG00000071866	Ppia	268373	protein_coding	3724.795969	-0.366009675	0.163663011	2.236361618	0.025328093	0.08827947	0.366009675

Supplementary Table 1

ensembl_gene_id	external_gene_name	entrezgene_id	gene_biotype	baseMean	log2FoldChange	lfcSE	stat	pvalue	padj	absLog2FC
ENSMUSG00000023010	Tmbim6	110213	protein_coding	14146.59156	0.366027588	0.06307834	-5.802746031	6.52E-09	1.29E-07	0.366027588
ENSMUSG00000037926	Ssh2	237860	protein_coding	711.7469507	0.366342818	0.075216096	-4.87053755	1.11E-06	1.52E-05	0.366342818
ENSMUSG00000001661	Hoxc6	15425	protein_coding	1.269656671	-0.366378233	2.029044672	0.180566864	0.856707567	1	0.366378233
ENSMUSG00000116802	Gm5165	102640673	lncRNA	71.85713028	-0.366557633	0.172566918	2.124147766	0.033657789	0.110042513	0.366557633
ENSMUSG00000075829	Gm23692	115488709	snRNA	0.519997961	0.366672309	1.61681408	-0.226786935	0.820589424	1	0.366672309
ENSMUSG00000027315	Spint1	20732	protein_coding	3021.392747	-0.366739034	0.087580548	4.187448474	2.82E-05	0.000281155	0.366739034
ENSMUSG00000025323	Sp4	20688	protein_coding	103.5391442	-0.366818087	0.114674161	3.198785883	0.001380076	0.008323805	0.366818087
ENSMUSG00000074698	Csnk2a1	12995	protein_coding	1229.347599	-0.366927986	0.073689068	4.979408701	6.38E-07	9.17E-06	0.366927986
ENSMUSG00000043251	Exoc3l	277978	protein_coding	6.966016804	0.367049993	0.417452993	-0.879260659	0.379259962	0.601649715	0.367049993
ENSMUSG00000024055	Cyp4f13	170716	protein_coding	413.6088856	-0.367096703	0.107482161	3.415419812	0.000636838	0.0043368	0.367096703
ENSMUSG00000109293	Dcst2	329702	protein_coding	25.33108856	0.367150158	0.224149456	-1.637970328	0.101427884	0.25018373	0.367150158
ENSMUSG00000027298	Tyro3	22174	protein_coding	170.4327221	-0.367174751	0.144073774	2.548519001	0.010818139	0.045273886	0.367174751
ENSMUSG00000023122	Sult1c2	69083	protein_coding	471.216222	-0.367192166	0.192683799	1.905672235	0.056692744	0.163933457	0.367192166
ENSMUSG00000037752	Xkr8	381560	protein_coding	16.67130374	-0.367334546	0.287229144	1.278890229	0.200935714	0.402261169	0.367334546
ENSMUSG00000029716	Tfr2	50765	protein_coding	2.559339327	-0.367345285	1.66136062	0.221111107	0.825005922	1	0.367345285
ENSMUSG00000025134	Alyref	21681	protein_coding	697.1903988	0.367359453	0.098313218	-3.736623197	0.000186508	0.001492245	0.367359453
ENSMUSG00000036403	Cep135	381644	protein_coding	124.1916632	0.367392087	0.120449351	-3.050179053	0.00228705	0.012618891	0.367392087
ENSMUSG00000035948	Acss3	380660	protein_coding	3.727997968	-0.367394129	0.644600509	0.569956312	0.56870733	1	0.367394129
ENSMUSG00000025384	Faap100	71885	protein_coding	291.8385743	-0.36743241	0.09789374	3.753380051	0.000174466	0.001406803	0.36743241
ENSMUSG00000001156	Mxd1	17119	protein_coding	5861.726798	0.367444627	0.19670543	-1.867994323	0.061762862	0.174214948	0.367444627
ENSMUSG00000034445	Cyb561a3	225912	protein_coding	249.1868227	-0.367473755	0.072721326	5.05317733	4.35E-07	6.49E-06	0.367473755
ENSMUSG00000039199	Zdhhc1	70796	protein_coding	130.9788502	-0.367497545	0.122601793	2.99748915	0.002722135	0.014666671	0.367497545
ENSMUSG00000093904	Tomm20	67952	protein_coding	558.8886389	-0.367567977	0.122207752	3.007730464	0.002632065	0.014241781	0.367567977
ENSMUSG00000044296	Zfp879	214779	protein_coding	7.462595675	-0.367741001	0.464991331	0.790855607	0.429028256	0.647976278	0.367741001
ENSMUSG00000103677	Pcdhga4	93712	protein_coding	2.704708252	-0.367741167	0.778095429	0.472617052	0.636486415	1	0.367741167
ENSMUSG00000000738	Spg7	234847	protein_coding	1010.419472	-0.367742173	0.073770032	4.984980517	6.20E-07	8.95E-06	0.367742173
ENSMUSG00000031458	Coprs	66423	protein_coding	121.3252242	-0.367783028	0.120801231	3.044530466	0.002330438	0.01281547	0.367783028
ENSMUSG00000068299	Nat8f4	75541	protein_coding	160.5825172	-0.367813191	0.152723422	2.408361371	0.016024311	0.061569443	0.367813191
ENSMUSG00000030350	Prmt8	381813	protein_coding	1.273069236	-0.367817315	2.658809964	0.138339077	0.889972444	1	0.367817315
ENSMUSG00000096773	Or52e8b	258147	protein_coding	1.502617377	-0.367846978	2.659121938	0.138334002	0.889976454	1	0.367846978
ENSMUSG00000020062	Slc5a8	216225	protein_coding	4559.823663	-0.367856439	0.140637397	2.615637421	0.008906107	0.038748264	0.367856439
ENSMUSG00000067847	Romo1	67067	protein_coding	309.8356556	-0.367896222	0.119314367	3.083419295	0.002046366	0.01156526	0.367896222
ENSMUSG00000036743	Psma8	73677	protein_coding	1.471404427	0.367904381	2.33176238	-0.157779534	0.874630526	1	0.367904381
ENSMUSG00000023044	Csad	246277	protein_coding	326.2438401	-0.367923171	0.15318852	2.401767254	0.016316086	0.062458007	0.367923171
ENSMUSG00000030304	Ergic2	67456	protein_coding	364.9244456	0.367950831	0.071863809	-5.120113114	3.05E-07	4.67E-06	0.367950831
ENSMUSG00000030471	Zdhhc13	243983	protein_coding	1632.912806	0.367966118	0.090495239	-4.06613789	4.78E-05	0.000448493	0.367966118
ENSMUSG00000071528	Atp5md	66477	protein_coding	1154.171185	-0.367979712	0.112337175	3.275671755	0.00105411	0.006649187	0.367979712
ENSMUSG00000028012	Rrh	20132	protein_coding	4.132266736	0.367981946	0.574903239	-0.640076314	0.522122987	1	0.367981946
ENSMUSG00000026546	Cfap45	71870	protein_coding	40.70866607	-0.368028588	0.257640177	1.428459615	0.153159611	0.334228588	0.368028588

Supplementary Table 1

ensembl_gene_id	external_gene_name	entrezgene_id	gene_biotype	baseMean	log2FoldChange	lfcSE	stat	pvalue	padj	absLog2FC
ENSMUSG00000041229	Phf8	320595	protein_coding	467.4915633	0.368073409	0.06611664	-5.567031386	2.59E-08	4.67E-07	0.368073409
ENSMUSG00000069805	Fbp1	14121	protein_coding	26.62847414	-0.368086939	0.217223304	1.694509441	0.090168519	0.228942639	0.368086939
ENSMUSG00000034115	Scn11a	24046	protein_coding	3.837939344	-0.368125398	1.047068046	0.35157734	0.725155262	1	0.368125398
ENSMUSG00000009927	Rps25	75617	protein_coding	3272.024089	-0.368139364	0.115065479	3.199390192	0.001377186	0.008312447	0.368139364
ENSMUSG00000033629	Hacd3	57874	protein_coding	759.2491393	-0.368146147	0.123933001	2.970525565	0.002972907	0.015750587	0.368146147
ENSMUSG00000001504	Irx2	16372	protein_coding	1.441664084	0.368177229	1.88620038	-0.195195183	0.84524015	1	0.368177229
ENSMUSG00000026200	Glb1l	74577	protein_coding	25.44663093	-0.368204191	0.209882474	1.754335105	0.079373104	0.208654479	0.368204191
ENSMUSG00000067928	Zfp760	240034	protein_coding	62.40520674	-0.368220635	0.222695626	1.653470443	0.098235157	0.244205235	0.368220635
ENSMUSG00000078787	Cyp2t4	384724	protein_coding	3.678384508	-0.368228148	1.111441589	0.331306793	0.740412761	1	0.368228148
ENSMUSG00000040274	Cdk6	12571	protein_coding	535.6265	-0.368289224	0.116145483	3.17093024	0.001519516	0.008984351	0.368289224
ENSMUSG00000022180	Slc7a8	50934	protein_coding	55.9297678	-0.368383034	0.258779413	1.423540726	0.154579465	0.336504783	0.368383034
ENSMUSG00000089204	Mir1927	100316784	miRNA	0.808328913	-0.36877076	1.748635983	0.210890525	0.832972698	1	0.36877076
ENSMUSG00000050100	Hmx2	15372	protein_coding	10.75714585	-0.368930291	0.316634168	1.165162601	0.243953181	0.4551483	0.368930291
ENSMUSG00000040148	Hmx3	15373	protein_coding	12.40317648	-0.368933831	0.302540631	1.219452178	0.222672616	0.429717907	0.368933831
ENSMUSG00000018379	Srsf1	110809	protein_coding	2515.858567	0.368957906	0.108774255	-3.39195984	0.000693946	0.004647131	0.368957906
ENSMUSG00000062937	Mtap	66902	protein_coding	82.54539445	0.36896313	0.182225751	-2.024758456	0.042892173	0.132619611	0.36896313
ENSMUSG00000054766	Set	56086	protein_coding	1779.18401	0.369019146	0.114717119	-3.21677488	0.001296403	0.007888281	0.369019146
ENSMUSG00000024749	Tmc1	13409	protein_coding	14.0006371	-0.369036731	0.438890114	0.840840838	0.400437106	0.622382905	0.369036731
ENSMUSG00000032892	Rangrf	57785	protein_coding	2.125793818	0.369043629	0.813242203	-0.453793012	0.649977814	1	0.369043629
ENSMUSG00000074361	C5ar2	319430	protein_coding	1.769504278	-0.369051302	1.869579998	0.197397973	0.843516116	1	0.369051302
ENSMUSG00000025626	Phf6	70998	protein_coding	296.0560183	0.36911082	0.135928285	-2.715482065	0.006617936	0.030425148	0.36911082
ENSMUSG00000045273	Cenph	26886	protein_coding	39.56665013	0.369132197	0.246489262	-1.497558939	0.134247882	0.304738078	0.369132197
ENSMUSG00000052040	Klf13	50794	protein_coding	917.6883872	-0.369223238	0.056918221	6.486907586	8.76E-11	2.22E-09	0.369223238
ENSMUSG00000034187	Nsf	18195	protein_coding	783.9279586	0.369241946	0.081275126	-4.543111353	5.54E-06	6.56E-05	0.369241946
ENSMUSG00000005628	Tmod4	50874	protein_coding	5.86270183	-0.369326391	0.531367993	0.695048245	0.487025067	0.695624496	0.369326391
ENSMUSG00000054381	Zfp747	269997	protein_coding	147.4618541	-0.369334696	0.129835267	2.844640781	0.004446153	0.022069064	0.369334696
ENSMUSG00000024401	Tnf	21926	protein_coding	47.22344395	0.36948065	0.420448166	-0.878778124	0.37952159	0.601768559	0.36948065
ENSMUSG00000015652	Steap1	70358	protein_coding	51.08661632	0.369644266	0.193773656	-1.907608465	0.05644184	0.163322332	0.369644266
ENSMUSG00000033825	Tpsb2	17229	protein_coding	3.467266505	0.369668109	0.753357757	-0.490693971	0.623642911	1	0.369668109
ENSMUSG00000062257	Opcml	330908	protein_coding	4.802744365	0.36974358	1.565622677	-0.236163914	0.81330548	0.907311132	0.36974358
ENSMUSG00000049321	Zfp2	22678	protein_coding	42.21486268	-0.369762654	0.188267796	1.964024975	0.049527196	0.147954434	0.369762654
ENSMUSG00000028756	Pink1	68943	protein_coding	1489.90727	0.369800761	0.099017631	-3.7346961	0.000187942	0.00150081	0.369800761
ENSMUSG00000096714	Or2ag17	258180	protein_coding	0.872725862	0.369830886	1.764694139	-0.20957223	0.834001556	1	0.369830886
ENSMUSG00000045092	S1pr1	13609	protein_coding	6.011860627	0.369972398	0.605371024	-0.611149828	0.541100395	0.73508818	0.369972398
ENSMUSG00000030683	Sez6l2	233878	protein_coding	27.99294349	-0.369985957	0.264012314	1.401396591	0.161095511	0.345901869	0.369985957
ENSMUSG00000079434	Neu2	23956	protein_coding	16.31354835	0.370063176	0.250968392	-1.474540973	0.140335994	0.314536999	0.370063176
ENSMUSG00000059248	Septin9	53860	protein_coding	740.4849849	0.370223421	0.083736222	-4.421305512	9.81E-06	0.000110288	0.370223421
ENSMUSG00000029385	Ccng2	12452	protein_coding	1000.418281	-0.370365461	0.193335658	1.915660384	0.055408338	0.16083904	0.370365461
ENSMUSG00000024158	Hagh	14651	protein_coding	797.0307647	0.370381466	0.072889247	-5.081428058	3.75E-07	5.65E-06	0.370381466

ensembl_gene_id	external_gene_name	entrezgene_id	gene_biotype	baseMean	log2FoldChange	lfcSE	stat	pvalue	padj	absLog2FC
ENSMUSG00000036591	Arhgap21	71435	protein_coding	1686.590094	0.370448132	0.113511831	-3.263520012	0.001100374	0.006901473	0.370448132
ENSMUSG00000042116	Vwa1	246228	protein_coding	10.96385472	-0.37065897	0.483683886	0.766324826	0.44348305	0.660955754	0.37065897
ENSMUSG00000023036	Pcdhgc4	93707	protein_coding	86.62277068	0.370757756	0.204137766	-1.816213448	0.069337636	0.189607836	0.370757756
ENSMUSG00000005483	Dnajb1	81489	protein_coding	1720.22231	-0.370763676	0.159091259	2.330509409	0.019779243	0.072698514	0.370763676
ENSMUSG00000040940	Arhgef1	16801	protein_coding	593.8326909	0.370837352	0.097544805	-3.801712998	0.000143699	0.001185931	0.370837352
ENSMUSG00000003762	Coq8b	76889	protein_coding	314.1990708	0.370866081	0.070687338	-5.246570182	1.55E-07	2.47E-06	0.370866081
ENSMUSG00000034205	Loxl2	94352	protein_coding	1.062377492	-0.37109947	1.201372248	0.308896323	0.757400394	1	0.37109947
ENSMUSG00000020437	Myo1g	246177	protein_coding	17.74860056	0.371154811	0.487840951	-0.760811101	0.446769903	0.664082645	0.371154811
ENSMUSG00000035033	Tbr1	21375	protein_coding	2.546540648	0.371169709	1.200389528	-0.30920772	0.757163522	1	0.371169709
ENSMUSG00000039699	Batf2	74481	protein_coding	205.5196637	-0.37119916	0.147987152	2.508320191	0.012130669	0.049536564	0.37119916
ENSMUSG00000022818	Cyp2ab1	224044	protein_coding	2.028871797	-0.37122336	1.571428895	0.236232999	0.813251875	1	0.37122336
ENSMUSG00000025855	Prkar1b	19085	protein_coding	7.065192715	-0.371254677	0.53210083	0.6977149	0.485355508	0.695380179	0.371254677
ENSMUSG00000000416	Cttnbp2	30785	protein_coding	160.6270681	0.371307988	0.21622968	-1.717192512	0.085944003	0.221416423	0.371307988
ENSMUSG00000032397	Tipin	66131	protein_coding	107.4275607	0.371349067	0.135537224	-2.739830851	0.006147081	0.028700282	0.371349067
ENSMUSG00000011884	Gltp	56356	protein_coding	674.0650229	0.371393315	0.12548038	-2.959772008	0.003078668	0.016227616	0.371393315
ENSMUSG00000046876	Atxn1	20238	protein_coding	640.277287	-0.371395024	0.131639611	2.821301439	0.004782924	0.023423844	0.371395024
ENSMUSG00000043831	Lysmd4	75099	protein_coding	185.3594674	0.371468471	0.098139069	-3.785123256	0.000153632	0.001253501	0.371468471
ENSMUSG00000037921	Ddx60	234311	protein_coding	430.6013194	0.371573418	0.460017507	-0.807737557	0.419241678	0.639216792	0.371573418
ENSMUSG00000019301	Hsd17b1	15485	protein_coding	1.49276165	0.371606949	1.117658864	-0.332486916	0.739521618	1	0.371606949
ENSMUSG00000030275	Etnk1	75320	protein_coding	1162.066988	0.371642322	0.093481773	-3.97555919	7.02E-05	0.000632578	0.371642322
ENSMUSG00000039943	Plcb4	18798	protein_coding	331.2181571	0.371680148	0.155444693	-2.391076472	0.016799053	0.063995287	0.371680148
ENSMUSG00000047649	Cd3eap	70333	protein_coding	76.4126091	-0.371685767	0.15842803	2.346085896	0.018971735	0.070451816	0.371685767
ENSMUSG00000000555	Itga5	16402	protein_coding	100.4401499	-0.37179649	0.145559821	2.554252186	0.010641615	0.044806045	0.37179649
ENSMUSG00000045636	Mtus1	102103	protein_coding	2496.660997	-0.371801121	0.074644841	4.980935277	6.33E-07	9.12E-06	0.371801121
ENSMUSG00000029798	Herc6	67138	protein_coding	40.89739116	-0.371965456	0.328571401	1.132068874	0.25760548	0.470794255	0.371965456
ENSMUSG00000022422	Dscc1	72107	protein_coding	22.69344562	0.371988643	0.272961513	-1.362787883	0.172949371	0.363647302	0.371988643
ENSMUSG00000111952	Gm4489	100043513	lncRNA	1.161039685	-0.372010254	1.225124108	0.303651076	0.761393735	1	0.372010254
ENSMUSG00000020481	Ankrd36	76389	protein_coding	2.461475965	-0.372021377	1.760917887	0.211265602	0.832680023	1	0.372021377
ENSMUSG00000054905	Stfa3	20863	protein_coding	1.043837783	-0.372038226	2.649742664	0.140405418	0.888339678	1	0.372038226
ENSMUSG00000075273	Ttc30b	72421	protein_coding	127.8890125	-0.372067206	0.133757295	2.781659158	0.005408181	0.025985072	0.372067206
ENSMUSG00000049690	Nckap5	210356	protein_coding	58.39141168	0.372072339	0.406741877	-0.914762804	0.360316166	0.582711741	0.372072339
ENSMUSG00000024140	Epas1	13819	protein_coding	1165.044084	-0.372131699	0.140558481	2.64752219	0.008108404	0.036011679	0.372131699
ENSMUSG00000092183	4930515G01Rik	67642	lncRNA	12.02847996	-0.372178165	0.324520184	1.146856755	0.251440828	0.463839794	0.372178165
ENSMUSG00000062169	Cnih4	98417	protein_coding	456.8286678	-0.37219464	0.095291967	3.905834376	9.39E-05	0.00081486	0.37219464
ENSMUSG00000068732	Tmem167b	67495	protein_coding	821.5397099	0.372219332	0.102494734	-3.631594678	0.000281675	0.002154013	0.372219332
ENSMUSG00000034175	Rhbdd3	279766	protein_coding	98.31341374	-0.37223745	0.114345363	3.255378604	0.001132413	0.007066978	0.37223745
ENSMUSG00000051037	Zfp455	218311	protein_coding	15.90003902	-0.372242241	0.317887843	1.170986086	0.24160437	0.45221065	0.372242241
ENSMUSG00000028654	Mycl	16918	protein_coding	167.6538569	-0.372279038	0.125151748	2.974621165	0.002933506	0.01556181	0.372279038
ENSMUSG00000079184	Mphosph8	75339	protein_coding	164.9043928	0.372326902	0.114707607	-3.24587804	0.00117089	0.00726647	0.372326902

ensembl_gene_id	external_gene_name	entrezgene_id	gene_biotype	baseMean	log2FoldChange	lfcSE	stat	pvalue	padj	absLog2FC
ENSMUSG00000026820	Ptges2	96979	protein_coding	458.0624826	-0.372578599	0.09200872	4.049383576	5.14E-05	0.000477499	0.372578599
ENSMUSG00000057329	Bcl2	12043	protein_coding	209.3342526	0.372820247	0.151302415	-2.464073347	0.0137368	0.054426466	0.372820247
ENSMUSG00000029618	Ocm	18261	protein_coding	1.786016222	0.372875438	1.453064465	-0.256613142	0.797477429	1	0.372875438
ENSMUSG00000078650	G6pc	14377	protein_coding	1.75634793	0.372897139	1.678752245	-0.22212756	0.824214584	1	0.372897139
ENSMUSG00000048001	Hes5	15208	protein_coding	1.411274838	-0.373139596	1.118157902	0.333709215	0.738598995	1	0.373139596
ENSMUSG00000065536	Mir98	723947	miRNA	0.753702534	-0.373143504	1.231512038	0.302996229	0.761892735	1	0.373143504
ENSMUSG00000055760	Gemin6	67242	protein_coding	57.89797195	0.373210074	0.22553855	-1.654750705	0.097975077	0.243645564	0.373210074
ENSMUSG00000035067	Xkr6	219149	protein_coding	32.65467725	-0.373239946	0.233156626	1.600812093	0.109418544	0.264120665	0.373239946
ENSMUSG00000021048	Mthfd1	108156	protein_coding	858.9911121	-0.373247267	0.139095285	2.683392672	0.007287936	0.032955362	0.373247267
ENSMUSG00000014778	Fhod1	234686	protein_coding	328.4245009	0.373247786	0.136618582	-2.732042599	0.0062943	0.029171357	0.373247786
ENSMUSG00000030781	Slc5a2	246787	protein_coding	2.995941002	0.373301133	0.931483376	-0.400759845	0.688596946	1	0.373301133
ENSMUSG00000055254	Ntrk2	18212	protein_coding	114.625146	0.373409077	0.200158367	-1.865568164	0.062101807	0.17492772	0.373409077
ENSMUSG00000027429	Sec23b	27054	protein_coding	1005.87877	0.373494526	0.098526936	-3.79078596	0.000150171	0.001229514	0.373494526
ENSMUSG00000028138	Adh5	11532	protein_coding	1220.414968	-0.373553052	0.105015628	3.557118665	0.000374945	0.002777855	0.373553052
ENSMUSG00000039531	Zup1	72580	protein_coding	167.9050686	-0.373556329	0.088066233	4.241765726	2.22E-05	0.000226486	0.373556329
ENSMUSG00000085418	Grip1os1	78105	lncRNA	1.268973061	-0.373557935	1.289362977	0.289722865	0.772028263	1	0.373557935
ENSMUSG00000097328	Tnfsf12	21944	protein_coding	4.70156422	-0.373626051	0.58904357	0.634292726	0.525889803	0.724255209	0.373626051
ENSMUSG00000055430	Nap1l5	58243	protein_coding	2.125323568	-0.373632923	0.94842236	0.393952039	0.693616449	1	0.373632923
ENSMUSG00000021466	Ptch1	19206	protein_coding	229.2515766	-0.373668009	0.079028668	4.728258962	2.26E-06	2.91E-05	0.373668009
ENSMUSG00000049760	Micos13	224904	protein_coding	902.386781	-0.373741273	0.093710245	3.988264829	6.66E-05	0.000602545	0.373741273
ENSMUSG00000041346	Wrap53	216853	protein_coding	187.9854117	0.373764104	0.084173784	-4.440386123	8.98E-06	0.000102059	0.373764104
ENSMUSG00000055865	Tafa3	329731	protein_coding	6.648731599	-0.373855918	0.605056635	0.617885825	0.536650604	0.732538277	0.373855918
ENSMUSG00000025511	Tspan4	64540	protein_coding	18.81446361	0.373866554	0.305452837	-1.223974731	0.22096176	0.427441699	0.373866554
ENSMUSG00000001229	Dpp9	224897	protein_coding	685.4643441	0.373874438	0.088654674	-4.217199374	2.47E-05	0.000249836	0.373874438
ENSMUSG00000029273	Sult1d1	53315	protein_coding	3732.462429	-0.373921239	0.089222347	4.190892197	2.78E-05	0.000277255	0.373921239
ENSMUSG00000074398	Gm15441	100038464	lncRNA	8.893832201	-0.373956043	0.422734998	0.884611031	0.376366455	0.598604992	0.373956043
ENSMUSG00000074796	Slc4a11	269356	protein_coding	5.207805302	0.374024715	0.748385872	-0.499775221	0.61723336	0.789053391	0.374024715
ENSMUSG00000021493	Pdlim7	67399	protein_coding	64.55576322	0.374051371	0.204926157	-1.825298325	0.067956023	0.186913018	0.374051371
ENSMUSG00000059939	9430015G10Rik	230996	protein_coding	109.4381657	0.374077802	0.12204811	-3.065002819	0.002176679	0.012123334	0.374077802
ENSMUSG00000037286	Stag1	20842	protein_coding	690.5960594	0.374090899	0.08821937	-4.240462129	2.23E-05	0.000227524	0.374090899
ENSMUSG00000026564	Dusp27	240892	protein_coding	5.17840358	-0.374105603	0.670383597	0.558047071	0.576812242	0.761222618	0.374105603
ENSMUSG00000038208	Pgap3	320655	protein_coding	369.6452711	0.374125464	0.091903572	-4.07084792	4.68E-05	0.000440521	0.374125464
ENSMUSG00000013091	Tmem190	78052	protein_coding	10.58430065	-0.374299028	0.3229984	1.158826261	0.246527011	0.458307673	0.374299028
ENSMUSG00000059834	Sclt1	67161	protein_coding	145.8573364	0.374385841	0.112931937	-3.315145827	0.000915953	0.005906307	0.374385841
ENSMUSG00000021700	Rab3c	67295	protein_coding	22.98476268	-0.374487517	0.281713851	1.329318797	0.183742815	0.378653073	0.374487517
ENSMUSG00000001348	Acp5	11433	protein_coding	1653.527593	-0.374886724	0.104908207	3.573473751	0.000352277	0.002629971	0.374886724
ENSMUSG00000051444	Bbs12	241950	protein_coding	8.872638281	0.374953451	0.341696653	-1.097328428	0.272497848	0.488337435	0.374953451
ENSMUSG00000065490	Mir30c-1	387227	miRNA	1.398686512	0.375042826	1.246356438	-0.300911373	0.763482078	1	0.375042826
ENSMUSG00000065436	Mir342	723909	miRNA	3.395535395	-0.375045051	0.569903321	0.658085394	0.510483261	1	0.375045051

ensembl_gene_id	external_gene_name	entrezgene_id	gene_biotype	baseMean	log2FoldChange	lfcSE	stat	pvalue	padj	absLog2FC
ENSMUSG00000022018	Rgcc	66214	protein_coding	79.70284167	0.37511982	0.185658518	-2.020482682	0.043333345	0.133608449	0.37511982
ENSMUSG00000040139	9430038I01Rik	77252	protein_coding	109.2211386	-0.375357306	0.151262263	2.481499998	0.013083071	0.052452983	0.375357306
ENSMUSG00000027285	Haus2	66296	protein_coding	138.7119214	-0.375386336	0.129980104	2.888029201	0.003876639	0.019710226	0.375386336
ENSMUSG00000057322	Rpl38	67671	protein_coding	2246.914901	-0.37548697	0.126006205	2.979908574	0.002883344	0.015384668	0.37548697
ENSMUSG00000021638	Ocln	18260	protein_coding	1379.949565	-0.3755925	0.074200392	5.06186681	4.15E-07	6.21E-06	0.3755925
ENSMUSG00000026229	Psmd1	70247	protein_coding	1326.404854	0.375603187	0.068911414	-5.45052213	5.02E-08	8.60E-07	0.375603187
ENSMUSG00000030054	Gp9	54368	protein_coding	2.530686409	-0.375622641	0.764433732	0.491373713	0.623162153	1	0.375622641
ENSMUSG00000089707	Slain1os	70898	lncRNA	3.171450805	0.375676415	0.888393677	-0.422871554	0.672388978	1	0.375676415
ENSMUSG00000097325	Gm16897	320400	lncRNA	7.538915236	0.375705714	0.454097139	-0.8273686	0.408028172	0.628624818	0.375705714
ENSMUSG00000038615	Nfe2l1	18023	protein_coding	2849.939657	0.375839699	0.057943929	-6.486265376	8.80E-11	2.23E-09	0.375839699
ENSMUSG00000035397	Klf16	118445	protein_coding	202.8614781	-0.376046997	0.118442715	3.174927191	0.001498741	0.008896486	0.376046997
ENSMUSG00000053559	Smagp	207818	protein_coding	1223.022636	-0.376066808	0.097039943	3.875381596	0.000106458	0.000910903	0.376066808
ENSMUSG00000027671	Actl6a	56456	protein_coding	452.6510068	0.376070265	0.073918632	-5.087624757	3.63E-07	5.49E-06	0.376070265
ENSMUSG00000061787	Rps17	20068	protein_coding	3196.137274	-0.376092653	0.116262273	3.234864097	0.001217006	0.007482994	0.376092653
ENSMUSG00000025255	Zfhx4	80892	protein_coding	4.582601074	0.376118448	1.886350043	-0.19938953	0.84195805	0.921777727	0.376118448
ENSMUSG00000021427	Ssr1	107513	protein_coding	2266.144613	0.376163407	0.130572857	-2.88086985	0.003965794	0.020064348	0.376163407
ENSMUSG00000045996	Polr2k	17749	protein_coding	77.79481917	-0.376330824	0.148558332	2.533219234	0.011302024	0.046953868	0.376330824
ENSMUSG00000019945	Cabcoco1	73287	protein_coding	37.44826747	0.37634742	0.175693988	-2.142062024	0.032188491	0.106546551	0.37634742
ENSMUSG00000041935	Rimoc1	106064	protein_coding	292.1502941	-0.376486691	0.103431028	3.639978254	0.000272661	0.002092344	0.376486691
ENSMUSG00000035840	Lysmd3	80289	protein_coding	459.2087043	0.376538697	0.07956471	-4.732483762	2.22E-06	2.87E-05	0.376538697
ENSMUSG00000075707	Dio3	107585	protein_coding	3.051772979	-0.37657571	0.712201224	0.528749035	0.596979556	1	0.37657571
ENSMUSG00000060093	H4c1	326619	protein_coding	637.2264932	0.376576473	0.153121543	-2.45933045	0.013919643	0.055018905	0.376576473
ENSMUSG00000031843	Mphosph6	68533	protein_coding	160.6538595	0.376626745	0.105813463	-3.55934619	0.000371779	0.002758114	0.376626745
ENSMUSG00000024900	Cpt1a	12894	protein_coding	4499.121888	-0.376751668	0.084946953	4.435140436	9.20E-06	0.000104289	0.376751668
ENSMUSG00000025927	Tfap2b	21419	protein_coding	3.62961883	0.376800034	2.059913728	-0.182920298	0.854860556	1	0.376800034
ENSMUSG00000078664	Sprr2a1	20755	protein_coding	294.2020933	0.37693391	0.804671417	-0.468432085	0.639475628	0.803370142	0.37693391
ENSMUSG00000025287	Acot9	56360	protein_coding	188.7606751	-0.376979036	0.123375271	3.055547778	0.002246498	0.012440868	0.376979036
ENSMUSG00000037563	Rps16	20055	protein_coding	1139.973889	-0.377137828	0.164682046	2.290096816	0.022015705	0.079122383	0.377137828
ENSMUSG00000025290	Rps24	20088	protein_coding	5629.019648	-0.377192592	0.116965399	3.224821997	0.00126051	0.007703947	0.377192592
ENSMUSG00000061099	Gapdhs	14447	protein_coding	5.151040313	-0.377192899	0.491891332	0.766821601	0.443187592	0.660786267	0.377192899
ENSMUSG00000038774	Ascc3	77987	protein_coding	1103.257027	0.377199131	0.074879672	-5.037403603	4.72E-07	7.00E-06	0.377199131
ENSMUSG00000071723	Gspt2	14853	protein_coding	43.09054626	0.377217138	0.200809982	-1.878478024	0.060315801	0.171438061	0.377217138
ENSMUSG00000069922	Ces3a	382053	protein_coding	2.176648266	-0.377287152	1.077019666	0.350306651	0.726108574	1	0.377287152
ENSMUSG00000087739	Gm24644	115489635	scaRNA	1.932033912	0.377386716	1.145756963	-0.329377633	0.741870272	1	0.377386716
ENSMUSG00000070298	Trcg1	541610	protein_coding	3.866956192	-0.377466739	0.973235512	0.387847273	0.69812906	1	0.377466739
ENSMUSG00000006930	Hap1	15114	protein_coding	38.76364469	-0.377551306	0.216389066	1.744779962	0.081023178	0.21167802	0.377551306
ENSMUSG00000051076	Vtcn1	242122	protein_coding	13.35244912	0.377622921	0.400567203	-0.94272052	0.345823875	0.567534275	0.377622921
ENSMUSG00000015968	Cacna1d	12289	protein_coding	28.85793307	0.377686796	0.321026924	-1.176495702	0.239396849	0.449607593	0.377686796
ENSMUSG00000035877	Zhx3	320799	protein_coding	215.7609004	0.377713306	0.119553424	-3.15936837	0.001581115	0.009308568	0.377713306

ensembl_gene_id	external_gene_name	entrezgene_id	gene_biotype	baseMean	log2FoldChange	lfcSE	stat	pvalue	padj	absLog2FC
ENSMUSG00000031842	Pde4c	110385	protein_coding	9.776557292	0.377980687	0.586249824	-0.64474337	0.519093506	0.719334577	0.377980687
ENSMUSG00000097787	2700046G09Rik	67188	lncRNA	17.5155948	-0.378028814	0.240925417	1.569069877	0.116631658	0.276057789	0.378028814
ENSMUSG00000032313	Tmem266	244886	protein_coding	14.33247724	0.378029251	0.368535563	-1.025760575	0.305004454	0.525108794	0.378029251
ENSMUSG00000037816	Fbxw17	109082	protein_coding	75.89895135	-0.378182632	0.157429512	2.402234675	0.016295251	0.062415953	0.378182632
ENSMUSG00000036639	Nudt1	17766	protein_coding	160.5664415	-0.378430372	0.098696265	3.834292738	0.000125926	0.001058292	0.378430372
ENSMUSG00000077438	Gm22595	115485710	snoRNA	0.730776855	-0.378459916	1.489460187	0.254091999	0.799424485	1	0.378459916
ENSMUSG00000092622	Khdc3	66991	protein_coding	3.129958707	-0.378473653	0.766704677	0.493636813	0.621562697	1	0.378473653
ENSMUSG00000015966	Il17rb	50905	protein_coding	36.0296325	-0.378481184	0.249323386	1.518033228	0.129006022	0.296522686	0.378481184
ENSMUSG00000022332	Khdrbs3	13992	protein_coding	268.632847	0.378545026	0.139813057	-2.7075084	0.006779036	0.031096942	0.378545026
ENSMUSG00000031647	Mfap3l	71306	protein_coding	590.2475571	-0.378614591	0.164197892	2.305843192	0.021119396	0.076601296	0.378614591
ENSMUSG00000039824	Myl6b	216459	protein_coding	16.93961804	0.378683869	0.299186978	-1.265709731	0.205617023	0.408255959	0.378683869
ENSMUSG00000063576	Klhdc3	71765	protein_coding	267.4209456	-0.378738331	0.097553446	3.882367527	0.000103444	0.000886497	0.378738331
ENSMUSG00000028310	Ppp3r2	19059	protein_coding	1.752935216	-0.378748481	1.085534825	0.348904957	0.727160664	1	0.378748481
ENSMUSG00000036242	Armh4	67419	protein_coding	84.72228682	-0.378771392	0.150029639	2.524643757	0.011581563	0.047814481	0.378771392
ENSMUSG00000038489	Polr2l	66491	protein_coding	86.75773656	-0.378845599	0.140021463	2.705625196	0.006817594	0.031247781	0.378845599
ENSMUSG00000052911	Lamb2	16779	protein_coding	375.1405986	0.37886112	0.105326576	-3.597013544	0.000321892	0.002420638	0.37886112
ENSMUSG00000004952	Rasa4	54153	protein_coding	375.3183499	-0.378967558	0.141730493	2.673860437	0.007498365	0.033749795	0.378967558
ENSMUSG00000097276	4930525G20Rik	320163	lncRNA	6.211962058	0.378981075	0.422978338	-0.895982232	0.370262253	0.592811812	0.378981075
ENSMUSG00000040596	Pogk	71592	protein_coding	257.8635811	-0.37902932	0.122024047	3.106185446	0.001895178	0.010863233	0.37902932
ENSMUSG00000043102	Qrfp	227717	protein_coding	5.230202856	-0.379042677	0.685540395	0.552910784	0.580324503	0.76356297	0.379042677
ENSMUSG00000055546	Timd4	276891	protein_coding	1.338644369	0.379136957	1.839733768	-0.206082513	0.836726459	1	0.379136957
ENSMUSG00000090799	Klhl33	546611	protein_coding	4.15706893	-0.379301387	0.711252039	0.533286889	0.593835002	1	0.379301387
ENSMUSG00000021662	Arhgef28	110596	protein_coding	1121.941498	-0.379374636	0.072565351	5.228041064	1.71E-07	2.72E-06	0.379374636
ENSMUSG00000055866	Per2	18627	protein_coding	345.4918205	0.379523539	0.286779907	-1.323396548	0.185703539	0.381194313	0.379523539
ENSMUSG00000042628	Zfyve1	217695	protein_coding	642.7604009	-0.379644665	0.131932786	2.877561197	0.004007622	0.02019882	0.379644665
ENSMUSG00000037005	Xpnpep2	170745	protein_coding	74.70777283	-0.379691094	0.135822566	2.795493456	0.005182055	0.025095735	0.379691094
ENSMUSG00000067586	S1pr3	13610	protein_coding	1.599838458	-0.379699641	1.374355979	0.276274594	0.782337168	1	0.379699641
ENSMUSG00000027879	Sec22b	20333	protein_coding	645.962207	0.379798282	0.105808163	-3.58949886	0.000331314	0.002485836	0.379798282
ENSMUSG00000049353	Rd3	74023	protein_coding	3.396721554	-0.379881399	1.011779904	0.375458533	0.70731948	1	0.379881399
ENSMUSG00000037239	Spred3	101809	protein_coding	11.74235666	0.379994896	0.575545498	-0.660234329	0.509103466	0.712606767	0.379994896
ENSMUSG00000064380	Gm26448	115487256	snoRNA	825.4893378	0.380136693	0.201515819	-1.886386363	0.059242909	0.169321114	0.380136693
ENSMUSG00000027220	Syt13	80976	protein_coding	18.72321956	-0.380168546	0.357973222	1.06200275	0.288234437	0.507128009	0.380168546
ENSMUSG00000024966	Stip1	20867	protein_coding	1193.273994	0.380276057	0.123343578	-3.083063295	0.002048816	0.011575143	0.380276057
ENSMUSG00000015950	Ncf1	17969	protein_coding	11.45428723	-0.380322031	0.39141973	0.971647573	0.331225907	0.553630732	0.380322031
ENSMUSG00000049848	Ceacam19	319930	protein_coding	2.070724396	0.380383504	1.286223431	-0.295736724	0.767431149	1	0.380383504
ENSMUSG00000043872	Zmym1	68310	protein_coding	57.05730672	0.380697276	0.192995832	-1.972567347	0.048544876	0.14581122	0.380697276
ENSMUSG00000030823	9130019O22Rik	78921	protein_coding	63.45172627	-0.380721855	0.187437	2.031199037	0.042234808	0.131176532	0.380721855
ENSMUSG00000063458	Lrmda	76633	protein_coding	2.528547714	-0.380737312	1.18224839	0.322045109	0.747418521	1	0.380737312
ENSMUSG00000074881	Mageb3	17147	protein_coding	1.566394407	0.38075342	1.504223655	-0.253122877	0.800173261	1	0.38075342

ensembl_gene_id	external_gene_name	entrezgene_id	gene_biotype	baseMean	log2FoldChange	lfcSE	stat	pvalue	padj	absLog2FC
ENSMUSG00000020111	Micu1	216001	protein_coding	1306.66792	-0.380986705	0.039482782	9.649439313	4.94E-22	2.80E-20	0.380986705
ENSMUSG00000020952	Scfd1	76983	protein_coding	709.6162205	0.381004577	0.08297506	-4.591796361	4.39E-06	5.32E-05	0.381004577
ENSMUSG00000117232	1600002D24Rik	69776	lncRNA	3.217013469	0.381136985	0.759816226	-0.501617327	0.615936728	1	0.381136985
ENSMUSG00000034566	Atp5h	71679	protein_coding	2426.365602	-0.381146027	0.09980534	3.818894117	0.000134051	0.001114664	0.381146027
ENSMUSG00000044286	Or5au1	258420	protein_coding	1.249069549	0.381223364	1.585650225	-0.240420843	0.810004022	1	0.381223364
ENSMUSG00000064442	Gm26225	115488410	snoRNA	9.978634917	0.381321403	0.459008415	-0.830750353	0.406114674	0.627075992	0.381321403
ENSMUSG00000022139	Mbnl2	105559	protein_coding	2544.778113	0.381326426	0.06728056	-5.667705918	1.45E-08	2.71E-07	0.381326426
ENSMUSG00000037979	Ccdc92	215707	protein_coding	19.1628266	0.381332092	0.303208832	-1.257654961	0.208516567	0.411599641	0.381332092
ENSMUSG00000044991	Shld1	73747	protein_coding	53.74655213	-0.381415277	0.187198374	2.037492465	0.041600716	0.129751344	0.381415277
ENSMUSG00000034958	Atcay	16467	protein_coding	2.941082851	-0.381451324	1.27844374	0.298371616	0.76541955	1	0.381451324
ENSMUSG00000060519	Tor3a	30935	protein_coding	348.1300018	-0.38152458	0.115040379	3.316440567	0.00091172	0.005884119	0.38152458
ENSMUSG00000048351	Coa7	69893	protein_coding	186.763013	-0.381601728	0.113015962	3.376529483	0.000734065	0.004876247	0.381601728
ENSMUSG00000041506	Rrp9	27966	protein_coding	244.9337626	0.381768866	0.127028269	-3.005385098	0.002652448	0.014342673	0.381768866
ENSMUSG00000028527	Ak4	11639	protein_coding	91.52010506	0.381909911	0.32044991	-1.191792849	0.233342481	0.442718029	0.381909911
ENSMUSG00000036596	Cpz	242939	protein_coding	1.717281649	-0.382021156	2.490834039	0.153370778	0.878105888	1	0.382021156
ENSMUSG00000025754	Agbl1	244071	protein_coding	1.122583772	-0.3820576	2.479767014	0.154069958	0.877554575	1	0.3820576
ENSMUSG00000031633	Slc25a4	11739	protein_coding	263.6299243	0.382132798	0.078610014	-4.861121099	1.17E-06	1.59E-05	0.382132798
ENSMUSG00000037060	Cavin3	109042	protein_coding	10.27469055	-0.38214037	0.349541294	1.093262445	0.274278587	0.490623547	0.38214037
ENSMUSG00000066258	Trim12a	76681	protein_coding	159.5066008	-0.382146648	0.1619307	2.359939457	0.018277918	0.068336322	0.382146648
ENSMUSG00000016541	Atxn10	54138	protein_coding	258.6014449	0.382164347	0.110874003	-3.44683456	0.000567196	0.003940543	0.382164347
ENSMUSG00000036168	Ccdc38	237465	protein_coding	4.491768956	0.382238788	0.691698164	-0.552609227	0.580531023	0.763773846	0.382238788
ENSMUSG00000038587	Akap12	83397	protein_coding	5.595848448	0.382244655	0.677381119	-0.564297769	0.572551499	0.757985847	0.382244655
ENSMUSG00000032116	Stt3a	16430	protein_coding	2485.978908	0.382411951	0.11908674	-3.21120514	0.001321795	0.008036866	0.382411951
ENSMUSG00000037443	Cep85	70012	protein_coding	408.4721543	0.38245962	0.076474148	-5.001162217	5.70E-07	8.30E-06	0.38245962
ENSMUSG00000022546	Gpt	76282	protein_coding	680.4671163	-0.38247666	0.215237893	1.776994998	0.075569063	0.201541909	0.38247666
ENSMUSG00000065904	Gm26109	115488999	snoRNA	0.754857076	-0.382581941	1.353214105	0.282720923	0.777390776	1	0.382581941
ENSMUSG00000027871	Hsd3b1	15492	protein_coding	2.257820628	-0.382698444	0.953672425	0.4012892	0.688207214	1	0.382698444
ENSMUSG00000024235	Map3k8	26410	protein_coding	15.29321221	0.382719754	0.324956805	-1.177755775	0.238893984	0.449071742	0.382719754
ENSMUSG00000075415	Fnbp1	14269	protein_coding	183.9259945	-0.382803717	0.097963238	3.907626215	9.32E-05	0.000810119	0.382803717
ENSMUSG00000105834	4930592C13Rik	381498	lncRNA	1.056006653	-0.382912941	1.121910207	0.341304445	0.732874402	1	0.382912941
ENSMUSG00000005732	Ranbp1	19385	protein_coding	413.205177	0.382948762	0.109038081	-3.512064406	0.00044464	0.003214881	0.382948762
ENSMUSG00000004665	Cnn2	12798	protein_coding	138.0961622	0.3829495	0.159979219	-2.393745268	0.016677327	0.063634329	0.3829495
ENSMUSG00000019359	Gdpd2	71584	protein_coding	29.60188966	-0.38295664	0.373006462	1.026675616	0.304573235	0.524764095	0.38295664
ENSMUSG00000038058	Nod1	107607	protein_coding	196.9956085	0.382983384	0.123294192	-3.106256478	0.001894723	0.010863233	0.382983384
ENSMUSG00000059895	Ptp4a3	19245	protein_coding	117.9206965	-0.383030525	0.149003971	2.57060616	0.010152071	0.043086943	0.383030525
ENSMUSG00000032840	Spring1	76792	protein_coding	177.4431317	0.383093724	0.112709222	-3.398956347	0.000676435	0.004563174	0.383093724
ENSMUSG00000026956	Uap1l1	227620	protein_coding	212.6560169	-0.383141486	0.118161482	3.242524387	0.001184758	0.007322266	0.383141486
ENSMUSG00000025871	4833439L19Rik	97820	protein_coding	2349.102617	0.383177946	0.059558701	-6.433618276	1.25E-10	3.05E-09	0.383177946
ENSMUSG00000073457	Smok2b	236574	protein_coding	3.695090602	0.383184039	2.057832128	-0.186207628	0.852281942	1	0.383184039

ensembl_gene_id	external_gene_name	entrezgene_id	gene_biotype	baseMean	log2FoldChange	lfcSE	stat	pvalue	padj	absLog2FC
ENSMUSG00000066516	Klk1b21	16616	protein_coding	0.786071127	0.383197788	1.637126203	-0.234067348	0.814932695	1	0.383197788
ENSMUSG00000031504	Rab20	19332	protein_coding	253.5450897	0.383266046	0.095983222	-3.993052511	6.52E-05	0.000592833	0.383266046
ENSMUSG00000047342	Zfp286	192651	protein_coding	14.03382548	0.383312771	0.365356504	-1.049147248	0.294110353	0.513201434	0.383312771
ENSMUSG00000047153	Khnyn	219094	protein_coding	1081.231787	-0.383377027	0.094174934	4.07090308	4.68E-05	0.000440521	0.383377027
ENSMUSG00000015094	Npdc1	18146	protein_coding	398.5012754	-0.38344183	0.076411788	5.018097868	5.22E-07	7.68E-06	0.38344183
ENSMUSG00000105975	Gm9831	101056032	lncRNA	5.394376818	-0.383445196	0.716892354	0.534871371	0.592738806	0.772538316	0.383445196
ENSMUSG00000004654	Ghrhr	14602	protein_coding	4.029737241	0.383483404	1.136266867	-0.337494135	0.735744432	1	0.383483404
ENSMUSG00000007107	Atp1a4	27222	protein_coding	4.296479189	0.38360083	1.229868679	-0.311903894	0.755113563	1	0.38360083
ENSMUSG00000023066	Rttn	246102	protein_coding	180.7592191	0.383631875	0.114310613	-3.356047741	0.000790649	0.005214369	0.383631875
ENSMUSG00000089069	Gm22840	115486335	snoRNA	0.727132154	0.383646685	1.214429351	-0.315906961	0.752073128	1	0.383646685
ENSMUSG00000032089	Il10ra	16154	protein_coding	1.919824091	-0.383728671	0.983784269	0.390053676	0.696496856	1	0.383728671
ENSMUSG00000027637	Rab5if	67388	protein_coding	367.1865554	0.38374015	0.112233984	-3.419108345	0.000628267	0.00429793	0.38374015
ENSMUSG00000024393	Prrc2a	53761	protein_coding	2315.334918	0.383742871	0.129746363	-2.957638765	0.003100052	0.01632826	0.383742871
ENSMUSG00000039652	Cpeb3	208922	protein_coding	51.67375434	-0.383744492	0.144249513	2.660282755	0.007807508	0.034924193	0.383744492
ENSMUSG00000026696	Vamp4	53330	protein_coding	211.4178536	-0.383776367	0.106536133	3.602311768	0.0003154	0.002381578	0.383776367
ENSMUSG00000036022	Fam122b	78755	protein_coding	46.83030279	0.383779122	0.190100648	-2.018820689	0.04350586	0.134003711	0.383779122
ENSMUSG00000067103	AY702103	446210	lncRNA	1.111252454	-0.383808552	2.100607098	0.182713156	0.855023093	1	0.383808552
ENSMUSG00000031919	Tmed6	66269	protein_coding	21.0134008	-0.383887951	0.326796522	1.174700233	0.240114666	0.450443439	0.383887951
ENSMUSG00000051956	Rnf133	386611	protein_coding	4.363465835	0.383896985	0.576066805	-0.666410531	0.505148733	1	0.383896985
ENSMUSG00000070730	Rmdn3	67809	protein_coding	664.779333	-0.383922202	0.096524104	3.977474926	6.97E-05	0.000627847	0.383922202
ENSMUSG00000026628	Atf3	11910	protein_coding	6886.389736	-0.384005668	0.353594844	1.086004716	0.277476906	0.494416662	0.384005668
ENSMUSG00000031556	Tm2d2	69742	protein_coding	1019.523434	0.384084225	0.10297649	-3.729824388	0.000191613	0.001522763	0.384084225
ENSMUSG00000029863	Casp2	12366	protein_coding	565.4762914	-0.384119068	0.091784759	4.184998359	2.85E-05	0.000283691	0.384119068
ENSMUSG00000072591	Fzd10os	319616	lncRNA	1.560035394	0.384365898	1.729498521	-0.222241241	0.824126091	1	0.384365898
ENSMUSG00000030785	Cox6a2	12862	protein_coding	1.847168346	-0.384401541	0.713507419	0.538749185	0.59005993	1	0.384401541
ENSMUSG00000006476	Nsmf	56876	protein_coding	599.9405758	-0.384559589	0.09144909	4.205176785	2.61E-05	0.000261416	0.384559589
ENSMUSG00000069307	H2bc23	665596	protein_coding	0.801861309	-0.384562364	1.394339497	0.275802532	0.782699739	1	0.384562364
ENSMUSG00000028277	Ube2j1	56228	protein_coding	834.3883723	0.384676223	0.064944956	-5.923111588	3.16E-09	6.55E-08	0.384676223
ENSMUSG00000036718	Micall2	231830	protein_coding	427.634514	0.38471319	0.138150112	-2.784747584	0.005356941	0.025783888	0.38471319
ENSMUSG00000037499	Nenf	66208	protein_coding	203.0310228	-0.384776578	0.097404523	3.950294758	7.81E-05	0.000694131	0.384776578
ENSMUSG00000068040	Tm9sf4	99237	protein_coding	2496.352728	0.384829086	0.116422094	-3.305464391	0.000948192	0.006090406	0.384829086
ENSMUSG00000039372	Marchf4	381270	protein_coding	4.129041232	0.384868275	0.914648693	-0.420782621	0.673913823	1	0.384868275
ENSMUSG00000022292	Rrm2b	382985	protein_coding	66.94917364	-0.384871865	0.178121258	2.160729543	0.030716237	0.102723358	0.384871865
ENSMUSG00000073705	Cenps	69928	protein_coding	26.49058505	0.384907365	0.256152404	-1.502649825	0.132929368	0.30270362	0.384907365
ENSMUSG00000031147	Magix	54634	protein_coding	3.321987036	-0.384947457	0.80545818	0.47792358	0.632704598	1	0.384947457
ENSMUSG00000036402	Gng12	14701	protein_coding	2481.492076	-0.384948004	0.081296882	4.735089424	2.19E-06	2.83E-05	0.384948004
ENSMUSG00000079555	Haus3	231123	protein_coding	89.54648382	0.385115226	0.136627244	-2.818729381	0.004821415	0.023571307	0.385115226
ENSMUSG00000023052	Npff	54615	protein_coding	2.818483738	0.385130425	0.629986915	-0.611330832	0.540980584	1	0.385130425
ENSMUSG00000065130	Gm24457	115489766	snRNA	1.779350454	-0.38521708	0.872111233	0.441706362	0.658701705	1	0.38521708

Supplementary Table 1

ensembl_gene_id	external_gene_name	entrezgene_id	gene_biotype	baseMean	log2FoldChange	lfcSE	stat	pvalue	padj	absLog2FC
ENSMUSG00000038633	Degs1	13244	protein_coding	215.0721421	0.385305024	0.134819272	-2.857937278	0.004264047	0.021273857	0.385305024
ENSMUSG00000028600	Podn	242608	protein_coding	5.028462797	-0.385359027	1.06049421	0.363376833	0.716323405	0.852797822	0.385359027
ENSMUSG00000025876	Unc5a	107448	protein_coding	63.18040595	0.385385077	0.141003066	-2.733168074	0.006272831	0.029098184	0.385385077
ENSMUSG00000085451	Gm40614	105245120	lncRNA	9.086184668	-0.385425974	0.351516555	1.096466068	0.272874865	0.488906968	0.385425974
ENSMUSG00000101026	Ly6g6g	78725	protein_coding	1.003024077	0.38554842	1.748097254	-0.220553187	0.825440355	1	0.38554842
ENSMUSG00000033126	Ybey	216119	protein_coding	69.69656848	-0.385696573	0.150350121	2.565322664	0.01030799	0.04361418	0.385696573
ENSMUSG00000042808	Gpx2	14776	protein_coding	4073.146887	0.385701532	0.160508827	-2.402992657	0.016261514	0.062321337	0.385701532
ENSMUSG00000030041	M1ap	110958	protein_coding	6.640345389	0.385757696	0.435150148	-0.886493314	0.375351756	0.597566653	0.385757696
ENSMUSG00000025403	Shmt2	108037	protein_coding	215.8646248	0.385823059	0.114835092	-3.359801015	0.000779986	0.005158468	0.385823059
ENSMUSG00000073633	Fbxo36	66153	protein_coding	66.24697476	0.385850604	0.143006086	-2.698141137	0.006972787	0.031809007	0.385850604
ENSMUSG00000021236	Entpd5	12499	protein_coding	3252.950939	-0.385866626	0.088590697	4.355611124	1.33E-05	0.000143449	0.385866626
ENSMUSG00000039891	Txlnb	378431	protein_coding	2.799877452	0.385879457	0.900421089	-0.428554441	0.668247507	1	0.385879457
ENSMUSG00000070306	Ccdc153	270150	protein_coding	1.184555884	-0.385949568	1.333362693	0.289455802	0.772232598	1	0.385949568
ENSMUSG00000048163	Selplg	20345	protein_coding	23.53220069	0.386026488	0.384422123	-1.004173446	0.315295019	0.53622883	0.386026488
ENSMUSG00000070564	Ntn5	243967	protein_coding	9.69480135	0.386193364	0.449216201	-0.859704889	0.389951739	0.612426345	0.386193364
ENSMUSG00000087026	A230103J11Rik	320466	lncRNA	10.9929108	-0.386234676	0.325718992	1.185791082	0.235704814	0.445817123	0.386234676
ENSMUSG00000024855	Pacs1	107975	protein_coding	89.41355998	-0.386273991	0.120925402	3.194316374	0.001401624	0.008435285	0.386273991
ENSMUSG00000024483	Ankhd1	108857	protein_coding	1052.071487	0.386290581	0.061523395	-6.278759139	3.41E-10	8.01E-09	0.386290581
ENSMUSG00000036995	Asap3	230837	protein_coding	29.04400561	0.386404717	0.327560533	-1.179643694	0.238141957	0.448564665	0.386404717
ENSMUSG00000072889	Nfxl1	100978	protein_coding	225.8046805	0.386626195	0.089599404	-4.315053221	1.60E-05	0.000169022	0.386626195
ENSMUSG00000001473	Tubb6	67951	protein_coding	53.71543349	-0.386666957	0.146655624	2.636564125	0.008375037	0.03690111	0.386666957
ENSMUSG00000104529	Rbakdn	100042605	lncRNA	2.236369447	-0.386701882	0.919339361	0.420630181	0.674025151	1	0.386701882
ENSMUSG00000026479	Lamc2	16782	protein_coding	704.8006561	-0.386721083	0.078790702	4.908207105	9.19E-07	1.28E-05	0.386721083
ENSMUSG00000021947	Cryl1	68631	protein_coding	1152.351243	-0.386799961	0.07759668	4.984748825	6.20E-07	8.96E-06	0.386799961
ENSMUSG00000087022	9130024F11Rik	329160	lncRNA	170.759575	0.386843586	0.127823108	-3.026397909	0.002474864	0.013502881	0.386843586
ENSMUSG00000040258	Nxph4	104080	protein_coding	1.087769733	-0.386998962	1.721435343	0.224811791	0.822125693	1	0.386998962
ENSMUSG00000067455	H4c11	319159	protein_coding	705.9345883	0.387032223	0.195813455	-1.976535386	0.048094172	0.144852664	0.387032223
ENSMUSG00000056687	Gm11696	100036768	lncRNA	26.90192985	0.387050916	0.282066164	-1.372199028	0.170001488	0.359584034	0.387050916
ENSMUSG00000054598	9130230L23Rik	231253	lncRNA	53.79332513	-0.387092689	0.22275769	1.737729857	0.082258425	0.214258446	0.387092689
ENSMUSG00000079435	Rpl36a	19982	protein_coding	1793.777072	-0.38710609	0.117315328	3.299705984	0.000967862	0.006195065	0.38710609
ENSMUSG00000070808	Bicra	243842	protein_coding	128.2067668	0.387114297	0.287208862	-1.347849418	0.17770682	0.370029503	0.387114297
ENSMUSG00000031950	Gabarapl2	93739	protein_coding	425.2433339	-0.387235582	0.103245286	3.750636919	0.000176386	0.001419217	0.387235582
ENSMUSG00000109901	Chmp1b	67064	protein_coding	681.1050592	-0.387320338	0.08024207	4.826898649	1.39E-06	1.86E-05	0.387320338
ENSMUSG00000018698	Lhx1	16869	protein_coding	1.331511612	0.387521988	2.118247508	-0.182944621	0.854841471	1	0.387521988
ENSMUSG00000020027	Socs2	216233	protein_coding	321.5386418	-0.387527001	0.097642312	3.96884293	7.22E-05	0.000647282	0.387527001
ENSMUSG00000024922	Ovol1	18426	protein_coding	306.2852745	-0.387611879	0.137341476	2.822249271	0.004768809	0.023369313	0.387611879
ENSMUSG00000022421	Nptxr	73340	protein_coding	8.411736894	0.387679682	0.620891342	-0.624392153	0.532370077	0.72898503	0.387679682
ENSMUSG00000017756	Slc12a7	20499	protein_coding	1183.374339	-0.387753409	0.073716462	5.260065404	1.44E-07	2.32E-06	0.387753409
ENSMUSG00000002668	Dennd1c	70785	protein_coding	272.7072676	-0.387759958	0.099024807	3.915786043	9.01E-05	0.000787343	0.387759958

ensembl_gene_id	external_gene_name	entrezgene_id	gene_biotype	baseMean	log2FoldChange	lfcSE	stat	pvalue	padj	absLog2FC
ENSMUSG00000052727	Map1b	17755	protein_coding	42.71415155	-0.387782735	0.311907188	1.243263219	0.213770853	0.418744149	0.387782735
ENSMUSG00000032392	Parp16	214424	protein_coding	186.73551	0.387835003	0.109076537	-3.555622632	0.000377085	0.002788738	0.387835003
ENSMUSG00000027692	Tnik	665113	protein_coding	365.3945849	-0.387879843	0.100209806	3.870677533	0.000108533	0.000926263	0.387879843
ENSMUSG00000060739	Nsa2	59050	protein_coding	566.5537046	-0.387886614	0.131181242	2.956875608	0.003107735	0.016359939	0.387886614
ENSMUSG00000021877	Arf4	11843	protein_coding	2789.773457	0.388066535	0.073110356	-5.307955775	1.11E-07	1.82E-06	0.388066535
ENSMUSG00000078546	Zfp995	70081	protein_coding	111.1440899	-0.388099301	0.17631079	2.201222631	0.027720267	0.094754639	0.388099301
ENSMUSG00000026623	Lpgat1	226856	protein_coding	800.0147537	0.388119087	0.088891826	-4.366195476	1.26E-05	0.000137721	0.388119087
ENSMUSG00000034667	Xpot	73192	protein_coding	638.5925855	0.388129961	0.114846955	-3.379540724	0.00072607	0.004832861	0.388129961
ENSMUSG00000066894	Vsig10	231668	protein_coding	1899.947731	-0.388271857	0.066448301	5.843217237	5.12E-09	1.03E-07	0.388271857
ENSMUSG00000063564	Col23a1	237759	protein_coding	9.456731132	0.388336566	0.523162132	-0.742287223	0.457913331	0.67261003	0.388336566
ENSMUSG00000020088	Sar1a	20224	protein_coding	1404.553693	0.388365974	0.112391803	-3.455465275	0.000549344	0.003837834	0.388365974
ENSMUSG00000032526	Ss18l2	26901	protein_coding	187.3489857	0.388395202	0.111592726	-3.480470605	0.000500534	0.003550608	0.388395202
ENSMUSG00000033717	Adra2a	11551	protein_coding	552.388221	0.388700367	0.242821773	-1.60076406	0.109429187	0.264120665	0.388700367
ENSMUSG00000018931	Natd1	24083	protein_coding	106.8937824	-0.388812583	0.144723462	2.686589844	0.007218553	0.032744159	0.388812583
ENSMUSG00000034066	Farp2	227377	protein_coding	476.1827756	-0.38895656	0.083576804	4.653881704	3.26E-06	4.04E-05	0.38895656
ENSMUSG00000022812	Gsk3b	56637	protein_coding	1126.072129	0.388999268	0.084913198	-4.581140241	4.62E-06	5.57E-05	0.388999268
ENSMUSG00000030323	Ift122	81896	protein_coding	105.5201337	0.389173285	0.106798539	-3.64399448	0.000268439	0.002060904	0.389173285
ENSMUSG00000091558	Gm17227	102639888	lncRNA	4.062402064	-0.389228912	0.554646721	0.701760052	0.482828816	1	0.389228912
ENSMUSG00000018659	Pnpo	103711	protein_coding	1138.468085	0.389248968	0.11808125	-3.296450271	0.000979149	0.006255193	0.389248968
ENSMUSG00000031196	F8	14069	protein_coding	10.72845721	0.389275851	0.390314855	-0.997338036	0.318600457	0.540015186	0.389275851
ENSMUSG00000024553	Galr1	14427	protein_coding	3.610916737	-0.38935857	0.733400229	0.530895075	0.595491491	1	0.38935857
ENSMUSG00000044258	Ctla2a	13024	protein_coding	1.448729426	-0.389518394	1.160548834	0.33563292	0.737147696	1	0.389518394
ENSMUSG00000036246	Gmip	78816	protein_coding	172.2782648	-0.389721487	0.110566495	3.524770213	0.00042385	0.003086183	0.389721487
ENSMUSG00000016356	Col20a1	73368	protein_coding	62.19146546	0.389751308	0.293204314	-1.329282309	0.183754848	0.378653073	0.389751308
ENSMUSG00000037353	Letmd1	68614	protein_coding	244.4812048	-0.389822192	0.080541564	4.840012713	1.30E-06	1.75E-05	0.389822192
ENSMUSG00000020395	Itk	16428	protein_coding	12.31732638	-0.389887912	0.545323213	0.714966652	0.47462965	0.686643367	0.389887912
ENSMUSG00000044707	Ccnjl	380694	protein_coding	65.62020252	0.389902964	0.19819781	-1.967241536	0.049155372	0.147109787	0.389902964
ENSMUSG00000078994	Zfp429	72807	protein_coding	74.66629445	-0.389923986	0.151562938	2.5726869	0.010091245	0.042894936	0.389923986
ENSMUSG00000030787	Lyve1	114332	protein_coding	28.7049331	0.389983214	0.231644889	-1.683539043	0.092270788	0.232919564	0.389983214
ENSMUSG00000004880	Lbr	98386	protein_coding	1027.576695	0.390024479	0.117361731	-3.323267957	0.000889694	0.005763989	0.390024479
ENSMUSG00000084780	Gm15350	100504249	lncRNA	1.221605614	0.390062999	1.141318143	-0.341765354	0.732527485	1	0.390062999
ENSMUSG00000009207	Lnpk	69605	protein_coding	193.8150024	0.390262478	0.12347151	-3.160749202	0.001573639	0.009274475	0.390262478
ENSMUSG00000040666	Sh3bgr	50795	protein_coding	40.73821556	-0.390281466	0.307251356	1.270235127	0.204000889	0.406181658	0.390281466
ENSMUSG00000022564	Grina	66168	protein_coding	402.5585633	-0.39028532	0.130580149	2.988856455	0.002800237	0.015028634	0.39028532
ENSMUSG00000026463	Atp2b4	381290	protein_coding	17.70396357	0.390564948	0.642557059	-0.607829208	0.543300755	0.736456016	0.390564948
ENSMUSG00000054206	Gzmm	16904	protein_coding	7.588896483	0.390635187	0.585312655	-0.667395764	0.504519371	0.708596728	0.390635187
ENSMUSG00000046691	Chtf8	214987	protein_coding	62.69309374	0.390639056	0.144350303	-2.706187996	0.00680605	0.03120353	0.390639056
ENSMUSG00000031447	Lamp1	16783	protein_coding	3846.132265	-0.390796402	0.080582994	4.849613819	1.24E-06	1.67E-05	0.390796402
ENSMUSG00000065523	Mir192	387187	miRNA	27.09477451	0.390934888	0.447262552	-0.8740613	0.382084871	0.604440039	0.390934888

Supplementary Table 1

ensembl_gene_id	external_gene_name	entrezgene_id	gene_biotype	baseMean	log2FoldChange	lfcSE	stat	pvalue	padj	absLog2FC
ENSMUSG00000025969	Nrp2	18187	protein_coding	1215.760381	-0.390953957	0.148151475	2.638879949	0.008318043	0.036708756	0.390953957
ENSMUSG00000018672	Copz2	56358	protein_coding	73.90551393	-0.391064135	0.185454896	2.108675175	0.034972629	0.113383099	0.391064135
ENSMUSG00000030890	Ilk	16202	protein_coding	15.38558968	0.391098554	0.300905588	-1.29973842	0.193690638	0.392130341	0.391098554
ENSMUSG00000022074	Tnfrsf10b	21933	protein_coding	89.99753697	-0.391308826	0.178856209	2.187840318	0.028681236	0.097419765	0.391308826
ENSMUSG00000028995	Hycc1	84652	protein_coding	269.7259587	0.391327861	0.120963796	-3.235082527	0.001216076	0.007482219	0.391327861
ENSMUSG00000051518	Rps19bp1	66538	protein_coding	152.5398663	0.391424511	0.110166533	-3.553025608	0.000380827	0.002810091	0.391424511
ENSMUSG00000065118	Gm23297	115486827	snoRNA	12.66185364	-0.391507903	0.502112682	0.779721199	0.435554998	0.653742472	0.391507903
ENSMUSG00000026228	Htr2b	15559	protein_coding	8.606103259	-0.391574078	0.452305553	0.865729097	0.38663874	0.609134912	0.391574078
ENSMUSG00000005493	Msh4	55993	protein_coding	3.241025313	0.391689404	0.756716222	-0.517617295	0.604725312	1	0.391689404
ENSMUSG00000029072	Tas1r3	83771	protein_coding	49.30427049	0.391801048	0.250809429	-1.562146404	0.118253503	0.27902128	0.391801048
ENSMUSG00000007891	Ctsd	13033	protein_coding	1540.757913	-0.391853446	0.079157037	4.950329892	7.41E-07	1.05E-05	0.391853446
ENSMUSG00000055319	Sec23ip	207352	protein_coding	645.6758765	0.391986317	0.081512624	-4.808903169	1.52E-06	2.01E-05	0.391986317
ENSMUSG00000021431	Snrnp48	67797	protein_coding	322.6115111	0.392071961	0.099540368	-3.938823718	8.19E-05	0.000723489	0.392071961
ENSMUSG00000022802	Lmln	239833	protein_coding	83.15391701	-0.392090767	0.141866302	2.763804802	0.005713171	0.027189423	0.392090767
ENSMUSG00000028753	Vwa5b1	75718	protein_coding	4.544532348	0.392115453	1.522195644	-0.257598591	0.796716717	0.899007849	0.392115453
ENSMUSG00000075324	Fign	60344	protein_coding	5.923438168	0.392175428	0.782841842	-0.500963806	0.616396596	0.788502504	0.392175428
ENSMUSG00000026687	Aldh9a1	56752	protein_coding	836.1810411	0.392184019	0.063027514	-6.222425624	4.90E-10	1.12E-08	0.392184019
ENSMUSG00000042345	Ubash3a	328795	protein_coding	4.77214624	0.392543432	1.263957414	-0.310566976	0.756129836	0.87755486	0.392543432
ENSMUSG00000048865	Arhgap30	226652	protein_coding	18.79270464	0.392554268	0.438601153	-0.895014218	0.370779488	0.593065878	0.392554268
ENSMUSG00000032536	Trak1	67095	protein_coding	3127.364489	-0.3927503	0.10601994	3.704494656	0.000211813	0.00166012	0.3927503
ENSMUSG00000001588	Acap1	216859	protein_coding	16.76449914	-0.392755286	0.409298644	0.959581205	0.337266032	0.559393767	0.392755286
ENSMUSG00000034201	Gas2l1	78926	protein_coding	506.8465825	-0.392831179	0.102747744	3.823258427	0.0001317	0.001100095	0.392831179
ENSMUSG00000025790	Slco3a1	108116	protein_coding	13.99898509	-0.392969933	0.470714021	0.834837959	0.40380895	0.625062193	0.392969933
ENSMUSG00000025091	Pnliprp2	18947	protein_coding	13.2403378	0.393024111	0.372285408	-1.055706462	0.291102344	0.510000436	0.393024111
ENSMUSG00000033102	Cdc14b	218294	protein_coding	218.8864918	-0.393038146	0.13545179	2.901682916	0.00371164	0.019029502	0.393038146
ENSMUSG00000049871	Nlrc3	268857	protein_coding	12.70991135	0.393284643	0.545784599	-0.72058582	0.471164381	0.684193826	0.393284643
ENSMUSG00000041216	Clvs1	74438	protein_coding	1.752887874	0.39347572	2.687419417	-0.14641396	0.883594607	1	0.39347572
ENSMUSG00000027074	Slc43a3	58207	protein_coding	14.50789535	-0.393513263	0.444729071	0.884838183	0.376243912	0.59846773	0.393513263
ENSMUSG00000029503	P2rx2	231602	protein_coding	5.218769846	-0.393575688	0.502701632	0.782921047	0.433673477	0.652196761	0.393575688
ENSMUSG00000024487	Yipf5	67180	protein_coding	475.6935054	0.393592081	0.086035433	-4.574767245	4.77E-06	5.72E-05	0.393592081
ENSMUSG00000030336	Cd27	21940	protein_coding	8.417064767	0.393661995	0.539395972	-0.72982005	0.465500187	0.679207466	0.393661995
ENSMUSG00000100714	2010308F09Rik	72122	lncRNA	1.331520012	-0.393686544	1.048813567	0.375363703	0.707389996	1	0.393686544
ENSMUSG00000003418	St8sia6	241230	protein_coding	1.585898502	-0.393803154	1.689778673	0.233050139	0.815722473	1	0.393803154
ENSMUSG00000068263	Efcc1	58229	protein_coding	2.602393823	-0.393824995	1.636575174	0.240639722	0.809834361	1	0.393824995
ENSMUSG00000030859	Fam24a	68223	protein_coding	0.931062152	0.393942932	1.701076255	-0.231584523	0.81686073	1	0.393942932
ENSMUSG00000032518	Rpsa	16785	protein_coding	8056.599601	-0.393985027	0.13897556	2.834923111	0.004583672	0.022615701	0.393985027
ENSMUSG00000053054	Adh6a	69117	protein_coding	6.280655339	0.394074675	0.851996872	-0.462530659	0.643700821	0.805858188	0.394074675
ENSMUSG00000021939	Ctsb	13030	protein_coding	3572.910923	-0.394125291	0.054920568	7.17627844	7.16E-13	2.22E-11	0.394125291
ENSMUSG00000021259	Cyp46a1	13116	protein_coding	2.350557374	-0.394156911	1.927824999	0.20445679	0.837996555	1	0.394156911

ensembl_gene_id	external_gene_name	entrezgene_id	gene_biotype	baseMean	log2FoldChange	lfcSE	stat	pvalue	padj	absLog2FC
ENSMUSG00000009633	G0s2	14373	protein_coding	3.144475123	-0.394168435	0.644114379	0.611954101	0.540568126	1	0.394168435
ENSMUSG00000065687	Gm23734	115487646	snoRNA	11.81589892	-0.394285306	0.323498734	1.218815608	0.222914186	0.429896633	0.394285306
ENSMUSG00000011427	Zfp790	233056	protein_coding	225.1513943	0.394346085	0.08115215	-4.85934243	1.18E-06	1.60E-05	0.394346085
ENSMUSG00000023175	Bsg	12215	protein_coding	7166.135774	-0.394371247	0.066694741	5.913078628	3.36E-09	6.93E-08	0.394371247
ENSMUSG00000029674	Limk1	16885	protein_coding	46.89637463	0.394482481	0.176731714	-2.232097867	0.025608495	0.088956393	0.394482481
ENSMUSG00000024968	Rcor2	104383	protein_coding	12.40995403	0.394508453	0.3986003	-0.98973446	0.322303927	0.54358318	0.394508453
ENSMUSG00000036615	Rfxap	170767	protein_coding	126.9790956	0.394555323	0.101156461	-3.900446087	9.60E-05	0.000830593	0.394555323
ENSMUSG00000021733	Slc4a7	218756	protein_coding	2889.599175	0.394831665	0.158388243	-2.492809175	0.012673694	0.051167007	0.394831665
ENSMUSG00000028648	Ndufs5	595136	protein_coding	243.5734784	-0.394890355	0.160788832	2.455956355	0.014051022	0.055403505	0.394890355
ENSMUSG00000012123	Crybg2	230806	protein_coding	680.9024377	-0.394969245	0.20026544	1.97222868	0.048583507	0.145821162	0.394969245
ENSMUSG00000093314	Mir5136	100628605	miRNA	0.565156851	0.395151452	1.294375891	-0.305283384	0.76015033	1	0.395151452
ENSMUSG00000059562	Ccdc154	207209	protein_coding	3.770811276	0.39518159	0.949198899	-0.416331699	0.6771673	1	0.39518159
ENSMUSG00000027454	Gins1	69270	protein_coding	34.82824187	0.395348533	0.216258375	-1.828130505	0.067529968	0.186051032	0.395348533
ENSMUSG00000028896	Rcc1	100088	protein_coding	487.3965379	0.395533607	0.087614832	-4.514459472	6.35E-06	7.43E-05	0.395533607
ENSMUSG00000038079	Tmem237	381259	protein_coding	16.12294454	0.395606089	0.284716584	-1.389473289	0.164688878	0.351288056	0.395606089
ENSMUSG00000060560	Ces4a	234677	protein_coding	1.678301082	-0.395697268	2.386219263	0.16582603	0.868293861	1	0.395697268
ENSMUSG00000024097	Srsf7	225027	protein_coding	1167.810585	0.395706161	0.080913553	-4.890480621	1.01E-06	1.39E-05	0.395706161
ENSMUSG00000059456	Ptk2b	19229	protein_coding	609.1502107	-0.395746131	0.123374805	3.207673804	0.001338132	0.00811528	0.395746131
ENSMUSG00000021388	Aspn	66695	protein_coding	2.907484934	0.39575539	0.598512719	-0.661231378	0.508463941	1	0.39575539
ENSMUSG00000020420	Zfp607a	545938	protein_coding	58.66899731	-0.395795275	0.143248429	2.762999067	0.005727293	0.027217415	0.395795275
ENSMUSG00000007836	Hnrnpa0	77134	protein_coding	1506.71077	0.395797622	0.086070405	-4.5985333	4.25E-06	5.17E-05	0.395797622
ENSMUSG00000001175	Calm1	12313	protein_coding	15731.1525	0.395861048	0.065376796	-6.055069588	1.40E-09	3.04E-08	0.395861048
ENSMUSG00000026311	Asb1	65247	protein_coding	58.04103891	0.39592655	0.172493776	-2.295309189	0.021715411	0.078315179	0.39592655
ENSMUSG00000073158	9030624G23Rik	66808	protein_coding	5.512831898	-0.395966082	0.526778571	0.751674619	0.452246757	0.668637691	0.395966082
ENSMUSG00000014873	Surf2	20931	protein_coding	137.0924415	-0.396054205	0.092460816	4.283481621	1.84E-05	0.000192306	0.396054205
ENSMUSG00000068227	Il2rb	16185	protein_coding	62.39576062	0.396071968	0.36170581	-1.095011351	0.273511663	0.489835334	0.396071968
ENSMUSG00000050079	Rspry1	67610	protein_coding	540.8335613	0.396072281	0.057802138	-6.852208159	7.27E-12	2.06E-10	0.396072281
ENSMUSG00000071984	Fndc1	68655	protein_coding	5.816281746	0.396148735	1.313975189	-0.301488748	0.763041829	0.881056689	0.396148735
ENSMUSG00000018411	Mapt	17762	protein_coding	85.62385355	0.396353214	0.144871169	-2.735901265	0.006220969	0.028930746	0.396353214
ENSMUSG00000096377	Mir5123	100628614	miRNA	0.995281493	-0.396396717	1.121037778	0.353598001	0.723640173	1	0.396396717
ENSMUSG00000062444	Ap3b2	11775	protein_coding	7.511840001	0.396420627	0.762939063	-0.519596711	0.603344691	0.779139367	0.396420627
ENSMUSG00000065874	Gm25683	115486588	snoRNA	0.974049656	-0.396427707	1.117231813	0.354830307	0.722716722	1	0.396427707
ENSMUSG00000034949	Zfr2	103406	protein_coding	76.864209	0.396459958	0.283278589	-1.399540852	0.161650857	0.346643585	0.396459958
ENSMUSG00000042579	4632404H12Rik	74034	lncRNA	51.42270263	-0.396497277	0.176968657	2.240494358	0.025058847	0.087507252	0.396497277
ENSMUSG00000030972	Acsm5	272428	protein_coding	1.725627493	-0.396527332	1.859917811	0.213196158	0.831173965	1	0.396527332
ENSMUSG00000017311	Pyy	217212	protein_coding	345.769881	-0.396566613	0.204263447	1.941446785	0.052204108	0.154304393	0.396566613
ENSMUSG00000020364	Zfp354a	21408	protein_coding	12.25051881	-0.396613925	0.336137231	1.179916677	0.238033356	0.448423779	0.396613925
ENSMUSG00000028863	Meaf6	70088	protein_coding	150.1787806	-0.396618848	0.092651128	4.280777349	1.86E-05	0.000193921	0.396618848
ENSMUSG00000033953	Ppp3r1	19058	protein_coding	749.7106257	0.396721043	0.06642645	-5.972335476	2.34E-09	4.95E-08	0.396721043

ensembl_gene_id	external_gene_name	entrezgene_id	gene_biotype	baseMean	log2FoldChange	lfcSE	stat	pvalue	padj	absLog2FC
ENSMUSG00000060798	Intu	380614	protein_coding	112.3812884	0.396808192	0.136836407	-2.899872935	0.00373314	0.019107412	0.396808192
ENSMUSG00000111769	Gm36278	102640141	lncRNA	0.768378386	0.396939462	1.472507019	-0.26956711	0.787493306	1	0.396939462
ENSMUSG00000049233	Apoo-ps	621156	processed_pseudogene	6.901797808	0.397031003	0.478765289	-0.829281094	0.406945366	0.627713038	0.397031003
ENSMUSG00000092274	Neat1	66961	lncRNA	13206.44079	0.397126026	0.22271522	-1.783111302	0.074568187	0.199760409	0.397126026
ENSMUSG00000085315	A430018G15Rik	115489452	lncRNA	7.886794312	-0.397192569	0.400034878	0.992894847	0.320761201	0.541897747	0.397192569
ENSMUSG00000024524	Gnal	14680	protein_coding	47.16907468	-0.397322709	0.291119363	1.364810314	0.172312676	0.36280925	0.397322709
ENSMUSG00000027902	Chil6	229688	protein_coding	8.715010541	0.397339685	0.407415855	-0.975268097	0.329427291	0.551796561	0.397339685
ENSMUSG00000035208	Slfn8	276950	protein_coding	3.124181101	0.397544269	0.952009709	-0.417584259	0.676251107	1	0.397544269
ENSMUSG00000032577	Mapkapk3	102626	protein_coding	987.4317593	-0.39763091	0.115487936	3.4430515	0.00057519	0.003986019	0.39763091
ENSMUSG00000027750	Postn	50706	protein_coding	3.863682583	0.397649441	0.920341255	-0.432067387	0.665692437	1	0.397649441
ENSMUSG00000001166	Oas1c	114643	protein_coding	34.15534306	-0.397880752	0.228307651	1.742739462	0.081379135	0.212372794	0.397880752
ENSMUSG00000052798	Nup107	103468	protein_coding	214.6455589	0.397887859	0.148399479	-2.681194456	0.007335987	0.03313635	0.397887859
ENSMUSG00000024222	Fkbp5	14229	protein_coding	2708.065445	-0.398096238	0.146194793	2.723053473	0.006468159	0.02983307	0.398096238
ENSMUSG00000109946	Brd3os	100504116	protein_coding	2.39458305	0.398122754	0.804121596	-0.495102676	0.620527645	1	0.398122754
ENSMUSG00000003949	Hlf	217082	protein_coding	2.558689088	0.398200479	1.559909591	-0.255271511	0.798513402	1	0.398200479
ENSMUSG00000028949	Smarcd3	66993	protein_coding	8.194857215	0.398521749	0.420480794	-0.947776343	0.343243326	0.565170522	0.398521749
ENSMUSG00000037216	Lipt1	623661	protein_coding	31.60448138	-0.398561132	0.217195303	1.835035692	0.066500401	0.184012672	0.398561132
ENSMUSG00000026135	Zfp142	77264	protein_coding	156.6495373	0.39861677	0.114516158	-3.480877972	0.000499773	0.00354826	0.39861677
ENSMUSG00000020186	Csrp2	13008	protein_coding	1896.143937	-0.39862706	0.096284991	4.140074749	3.47E-05	0.000339062	0.39862706
ENSMUSG00000034825	Nrip3	78593	protein_coding	7.838650674	-0.398691668	0.575733752	0.69249313	0.488627699	0.696497169	0.398691668
ENSMUSG00000030259	Rassf8	71323	protein_coding	20.17374288	0.398712208	0.286622098	-1.391072812	0.164203348	0.350538094	0.398712208
ENSMUSG00000060538	Tmem219	68742	protein_coding	186.8231772	-0.399006382	0.114608411	3.48147556	0.000498659	0.003541875	0.399006382
ENSMUSG00000039958	Etfbkmt	320204	protein_coding	176.4759036	-0.399071296	0.128862899	3.096867293	0.001955774	0.011144808	0.399071296
ENSMUSG00000038598	Shoc1	100155	protein_coding	12.30177602	-0.399128144	0.393732486	1.013703868	0.310724078	0.531740666	0.399128144
ENSMUSG00000024594	Prrc1	73137	protein_coding	686.8998891	0.399186394	0.074909565	-5.328910847	9.88E-08	1.63E-06	0.399186394
ENSMUSG00000028860	Sytl1	269589	protein_coding	706.0781379	-0.399213284	0.160230023	2.491501137	0.012720456	0.05132314	0.399213284
ENSMUSG00000048537	Phldb1	102693	protein_coding	1117.032136	-0.399220091	0.080169139	4.979722848	6.37E-07	9.17E-06	0.399220091
ENSMUSG00000026097	Ormdl1	227102	protein_coding	188.8633996	0.399332938	0.148641555	-2.686549784	0.007219419	0.032744159	0.399332938
ENSMUSG00000035781	R3hdm4	109284	protein_coding	1349.272026	-0.399492336	0.071942555	5.552935037	2.81E-08	5.04E-07	0.399492336
ENSMUSG00000022516	Nudt16l1	66911	protein_coding	148.0807693	0.399513685	0.114358981	-3.49350512	0.000476724	0.003406585	0.399513685
ENSMUSG00000029524	Sirt4	75387	protein_coding	58.31240879	-0.39954807	0.154085408	2.593029895	0.00951345	0.040902139	0.39954807
ENSMUSG00000030834	Abcc6	27421	protein_coding	142.6910011	0.399563256	0.183795	-2.173961511	0.029708026	0.10016904	0.399563256
ENSMUSG00000086915	Gm16364	100504626	lncRNA	5.700380337	0.399576048	0.48242543	-0.828264894	0.4075205	0.628274069	0.399576048
ENSMUSG00000025089	Gfra1	14585	protein_coding	2.936660048	-0.399578926	0.949262761	0.42093606	0.673801773	1	0.399578926
ENSMUSG00000031712	Il15	16168	protein_coding	142.1420713	-0.399597964	0.142675484	2.800747209	0.005098444	0.024770728	0.399597964
ENSMUSG00000018819	Lsp1	16985	protein_coding	19.08222659	0.399657582	0.446441339	-0.895207381	0.37067624	0.593065878	0.399657582
ENSMUSG00000058396	Gpr182	11536	protein_coding	7.586931324	0.399749373	0.535648915	-0.746289895	0.455492315	0.670888341	0.399749373
ENSMUSG00000019768	Esr1	13982	protein_coding	34.42371914	-0.399840883	0.313326483	1.276115826	0.201914578	0.403926995	0.399840883
ENSMUSG00000041375	Ccdc9	243846	protein_coding	676.9065328	-0.399862762	0.07069329	5.656304296	1.55E-08	2.89E-07	0.399862762

ensembl_gene_id	external_gene_name	entrezgene_id	gene_biotype	baseMean	log2FoldChange	lfcSE	stat	pvalue	padj	absLog2FC
ENSMUSG00000112023	Lilrb4b	14727	protein_coding	2.329151448	0.399868314	1.047460365	-0.381750305	0.702646583	1	0.399868314
ENSMUSG00000026179	Pnkd	56695	protein_coding	226.3418164	0.399989846	0.114731487	-3.486312741	0.000489728	0.003485935	0.399989846
ENSMUSG00000053846	Lipg	16891	protein_coding	700.2904616	-0.400148083	0.179962587	2.223507063	0.026181628	0.09052835	0.400148083
ENSMUSG00000050304	Slc25a2	83885	protein_coding	4.025802316	-0.400246036	0.571937745	0.699806998	0.484047844	1	0.400246036
ENSMUSG00000043760	Pkhd1	241035	protein_coding	160.5985171	0.400502636	0.112221665	-3.568853104	0.000358547	0.002670749	0.400502636
ENSMUSG00000022227	Mcpt1	17224	protein_coding	9.685281371	-0.400502756	0.546720843	0.732554394	0.463830256	0.677309474	0.400502756
ENSMUSG00000048992	Prss32	69814	protein_coding	4928.401481	-0.400520895	0.087428884	4.581104977	4.63E-06	5.57E-05	0.400520895
ENSMUSG00000025497	Cdhr5	72040	protein_coding	6413.323795	-0.400697645	0.131953302	3.036662503	0.002392131	0.013111071	0.400697645
ENSMUSG00000020681	Ace	11421	protein_coding	249.1719677	-0.400844034	0.216991635	1.847278742	0.064706752	0.18053173	0.400844034
ENSMUSG00000084861	1700095J07Rik	115490402	lncRNA	1.412887162	0.400988745	1.69079589	-0.237159758	0.812532854	1	0.400988745
ENSMUSG00000023832	Acat2	110460	protein_coding	335.9198837	0.401062355	0.137008272	-2.927285695	0.003419346	0.017740208	0.401062355
ENSMUSG00000049928	Glp2r	93896	protein_coding	5.298122	0.401155393	0.951747635	-0.421493448	0.673394794	0.824897384	0.401155393
ENSMUSG00000020846	Rflnb	76566	protein_coding	1347.030903	-0.401277487	0.116579285	3.442099409	0.000577218	0.003996721	0.401277487
ENSMUSG00000054405	Dnajc8	68598	protein_coding	449.7354076	-0.401302135	0.072999804	5.49730426	3.86E-08	6.75E-07	0.401302135
ENSMUSG00000063698	Sfxn4	94281	protein_coding	4.221702561	0.401390554	0.642797416	-0.624443322	0.532336481	1	0.401390554
ENSMUSG00000044786	Zfp36	22695	protein_coding	3799.777969	-0.401545645	0.195745304	2.051367958	0.040231127	0.126475696	0.401545645
ENSMUSG00000022748	Cmss1	66497	protein_coding	63.71296895	0.40157833	0.174808521	-2.297246885	0.02160469	0.078035837	0.40157833
ENSMUSG00000068551	Zfp467	68910	protein_coding	323.7323645	-0.401751524	0.103751791	3.872237022	0.000107841	0.000920831	0.401751524
ENSMUSG00000002233	Rhoc	11853	protein_coding	1016.064078	-0.401960489	0.187011002	2.149394876	0.031603111	0.105093391	0.401960489
ENSMUSG00000028680	Plk3	12795	protein_coding	1078.890588	-0.401992116	0.202011085	1.989950775	0.046596358	0.141320893	0.401992116
ENSMUSG00000051396	Gm45902	119065974	protein_coding	51.59804981	0.402198045	0.175880354	-2.286770723	0.022209209	0.07969641	0.402198045
ENSMUSG00000022098	Bmp1	12153	protein_coding	53.69668115	0.402348706	0.29627527	-1.35802325	0.174456307	0.365696631	0.402348706
ENSMUSG00000079355	Ackr4	252837	protein_coding	45.57932078	-0.40239054	0.191480541	2.10146962	0.035599762	0.114868009	0.40239054
ENSMUSG00000097587	4930578M01Rik	75951	lncRNA	4.731075024	0.402695269	0.62702278	-0.642233874	0.520721342	0.720610174	0.402695269
ENSMUSG00000032113	Chek1	12649	protein_coding	63.50856503	0.402730711	0.259647628	-1.551066398	0.120885773	0.283485892	0.402730711
ENSMUSG00000094995	Pate3	100312956	protein_coding	0.952377064	0.402746905	1.990264018	-0.202358532	0.839636444	1	0.402746905
ENSMUSG00000030366	Ceacam12	67315	protein_coding	18.90920295	-0.402780086	0.324916651	1.239641258	0.215108113	0.420139706	0.402780086
ENSMUSG00000040613	Apobec1	11810	protein_coding	369.0642877	0.402832415	0.205086996	-1.964202616	0.0495066	0.147919664	0.402832415
ENSMUSG00000021822	Plau	18792	protein_coding	1.567206475	0.402841882	2.041983928	-0.197279654	0.8436087	1	0.402841882
ENSMUSG00000087172	Gm12153	102638460	lncRNA	1.257814786	0.402967328	1.826519097	-0.220620375	0.825388035	1	0.402967328
ENSMUSG00000062054	Iah1	67732	protein_coding	297.1767974	-0.403023626	0.091980062	4.381641159	1.18E-05	0.000129591	0.403023626
ENSMUSG00000020190	Mknk2	17347	protein_coding	2461.109709	-0.403256502	0.075297813	5.355487573	8.53E-08	1.42E-06	0.403256502
ENSMUSG00000032292	Nr2e3	23958	protein_coding	50.96211015	0.403281118	0.214150567	-1.883166236	0.059677843	0.170151917	0.403281118
ENSMUSG00000002058	Unc119	22248	protein_coding	267.3049594	-0.403285452	0.199680738	2.019651253	0.043419575	0.133824348	0.403285452
ENSMUSG00000086503	Xist	213742	lncRNA	4057.971602	-0.403302884	0.143454529	2.811363898	0.004933196	0.024045689	0.403302884
ENSMUSG00000036853	Mcoln3	171166	protein_coding	9.452455448	0.403349639	0.5053395	-0.798175561	0.424768618	0.64425321	0.403349639
ENSMUSG00000064280	Ccdc146	75172	protein_coding	3.487828517	0.403355433	0.917983417	-0.43939294	0.660376841	1	0.403355433
ENSMUSG00000027752	Exosc8	69639	protein_coding	113.4935711	0.403362635	0.173966607	-2.318621033	0.020415594	0.074589184	0.403362635
ENSMUSG00000028188	Spata1	70951	protein_coding	15.60441947	-0.403372461	0.302367068	1.334048921	0.182187824	0.376665775	0.403372461

ensembl_gene_id	external_gene_name	entrezgene_id	gene_biotype	baseMean	log2FoldChange	lfcSE	stat	pvalue	padj	absLog2FC
ENSMUSG00000093289	Snord92	100217462	snoRNA	0.752580739	0.403406466	1.668387279	-0.241794259	0.808939588	1	0.403406466
ENSMUSG00000051650	B3gnt2	53625	protein_coding	77.41885872	0.403989351	0.263517403	-1.533065162	0.1252598	0.290432101	0.403989351
ENSMUSG00000041481	Serpina3g	20715	protein_coding	5.025363542	-0.404039213	0.625249616	0.646204657	0.518146824	0.718665364	0.404039213
ENSMUSG00000004285	Atp6v1f	66144	protein_coding	524.0618	-0.404148447	0.065963659	6.126834846	8.96E-10	1.98E-08	0.404148447
ENSMUSG00000019487	Trip10	106628	protein_coding	568.5023952	-0.404301997	0.10760129	3.757408476	0.000171682	0.001385242	0.404301997
ENSMUSG00000027997	Casp6	12368	protein_coding	250.4280422	-0.404337168	0.091093162	4.438721411	9.05E-06	0.00010271	0.404337168
ENSMUSG00000061482	H4c4	319156	protein_coding	3470.831971	0.404400776	0.129853507	-3.11428459	0.001843915	0.010614744	0.404400776
ENSMUSG00000000127	Fer	14158	protein_coding	219.4842831	0.404584724	0.093407587	-4.331390403	1.48E-05	0.000157966	0.404584724
ENSMUSG00000084941	Gm11944	100302648	lncRNA	3.453327496	-0.404594709	0.56658222	0.714097081	0.475167151	1	0.404594709
ENSMUSG00000001168	Oas1h	246729	protein_coding	5.133349268	0.404649722	0.699438257	-0.5785353	0.562902772	0.750688555	0.404649722
ENSMUSG00000043019	Edem3	66967	protein_coding	2164.056912	0.404739527	0.103494596	-3.910731031	9.20E-05	0.00080104	0.404739527
ENSMUSG00000054091	1810037I17Rik	67704	protein_coding	844.9011446	-0.40483404	0.086108759	4.701426967	2.58E-06	3.27E-05	0.40483404
ENSMUSG00000033177	Tmprss7	208171	protein_coding	20.49299184	0.40487886	0.301953397	-1.340865395	0.179964159	0.373498571	0.40487886
ENSMUSG00000020649	Rrm2	20135	protein_coding	183.589983	0.404879921	0.21057928	-1.922695912	0.05451825	0.159094255	0.404879921
ENSMUSG00000061576	Dpp6	13483	protein_coding	4.914617198	0.404882578	2.088390358	-0.193873035	0.846275298	0.924222269	0.404882578
ENSMUSG00000028013	Ppa2	74776	protein_coding	868.1982255	-0.405004667	0.074379137	5.44513804	5.18E-08	8.85E-07	0.405004667
ENSMUSG00000046753	Ccdc66	320234	protein_coding	151.0990289	0.405024683	0.090948786	-4.453326978	8.45E-06	9.65E-05	0.405024683
ENSMUSG00000063652	Slc22a21	56517	protein_coding	119.7352017	-0.405131841	0.165308836	2.450757318	0.014255603	0.056038331	0.405131841
ENSMUSG00000031428	Zcchc18	66995	protein_coding	3.211898331	-0.405268059	0.717345937	0.564954839	0.572104482	1	0.405268059
ENSMUSG00000036304	Zdhhc23	332175	protein_coding	234.1547085	-0.405300215	0.12323	3.288973598	0.001005535	0.006384236	0.405300215
ENSMUSG00000021773	Comtd1	69156	protein_coding	76.67169163	-0.405307043	0.153474049	2.640883233	0.008269021	0.036560808	0.405307043
ENSMUSG00000058470	Gm8369	666926	protein_coding	3.362067935	0.405436363	1.133674183	-0.35763041	0.720619917	1	0.405436363
ENSMUSG00000058740	Kcnt1	227632	protein_coding	6.90951613	0.405521822	1.095738023	-0.370090125	0.711315339	0.849668102	0.405521822
ENSMUSG00000061477	Rps7	20115	protein_coding	3952.441881	-0.405628197	0.133526485	3.037810799	0.002383035	0.013074234	0.405628197
ENSMUSG00000054715	Zscan22	232878	protein_coding	84.03098568	0.40564216	0.145803556	-2.78211431	0.005400601	0.025963765	0.40564216
ENSMUSG00000028167	Bdh2	69772	protein_coding	2.589970441	0.405653735	1.05613847	-0.38409143	0.700910683	1	0.405653735
ENSMUSG00000029334	Prkg2	19092	protein_coding	608.8810466	-0.405721418	0.184087739	2.203956769	0.027527385	0.0942345	0.405721418
ENSMUSG00000021567	Nkd2	72293	protein_coding	5.314085686	0.405792032	0.773224867	-0.524804684	0.599718975	0.776544903	0.405792032
ENSMUSG00000045980	Tmem104	320534	protein_coding	101.6701797	0.405795093	0.15950395	-2.544106866	0.010955756	0.045710802	0.405795093
ENSMUSG00000034282	Evpl	14027	protein_coding	920.6237005	-0.405824624	0.146163571	2.776510051	0.005494594	0.026309923	0.405824624
ENSMUSG00000032498	Mlh1	17350	protein_coding	255.2824509	0.405876855	0.080714162	-5.028570535	4.94E-07	7.31E-06	0.405876855
ENSMUSG00000078444	Gm10941	100169878	lncRNA	1.812385002	-0.405931714	0.754178395	0.538243626	0.590408865	1	0.405931714
ENSMUSG00000090213	Peds1	407243	protein_coding	553.679367	0.406011041	0.114949217	-3.532090524	0.000412288	0.00301129	0.406011041
ENSMUSG00000025129	Ppp1r27	68701	protein_coding	3.31974573	0.406077082	0.893836284	-0.45430812	0.649607073	1	0.406077082
ENSMUSG00000063015	Ccni	12453	protein_coding	2077.841335	-0.406089434	0.08931016	4.546956725	5.44E-06	6.46E-05	0.406089434
ENSMUSG00000027200	Sema6d	214968	protein_coding	690.2369689	0.406164491	0.148026232	-2.743868337	0.006071989	0.028422001	0.406164491
ENSMUSG00000027746	Ufm1	67890	protein_coding	733.3893813	0.406175366	0.084569504	-4.80285858	1.56E-06	2.07E-05	0.406175366
ENSMUSG00000029171	Pgm2	66681	protein_coding	897.7788634	-0.40619376	0.058245407	6.973833322	3.08E-12	9.01E-11	0.40619376
ENSMUSG00000037016	Frem2	242022	protein_coding	25.77171125	0.406228867	0.30508991	-1.331505414	0.183022765	0.377733062	0.406228867

ensembl_gene_id	external_gene_name	entrezgene_id	gene_biotype	baseMean	log2FoldChange	lfcSE	stat	pvalue	padj	absLog2FC
ENSMUSG00000037593	Rskr	216976	protein_coding	7.20426931	0.40631249	0.561792683	-0.723242759	0.469530753	0.683156904	0.40631249
ENSMUSG00000109032	Gm7972	666190	transcribed_unprocessed_pseudogene	5.979304134	0.406331812	0.55075975	-0.737765988	0.460656665	0.674823398	0.406331812
ENSMUSG00000051256	Jagn1	67767	protein_coding	314.6824037	0.406474007	0.087309228	-4.655567513	3.23E-06	4.01E-05	0.406474007
ENSMUSG00000031996	Aplp2	11804	protein_coding	2189.793415	-0.406533732	0.106169768	3.829091287	0.000128617	0.001078713	0.406533732
ENSMUSG00000038020	Rapgefl1	268480	protein_coding	875.0866052	-0.406588354	0.123029918	3.30479253	0.000950467	0.006100279	0.406588354
ENSMUSG00000027356	Fermt1	241639	protein_coding	2353.9896	0.406625197	0.078035554	-5.210768346	1.88E-07	2.96E-06	0.406625197
ENSMUSG00000017607	Tns4	217169	protein_coding	1735.160878	-0.406723963	0.198294357	2.051112136	0.040256028	0.12652989	0.406723963
ENSMUSG00000036098	Myrf	225908	protein_coding	8.283474487	0.406730402	0.900810977	-0.451515815	0.65161783	0.810851996	0.406730402
ENSMUSG00000037681	Esyt3	272636	protein_coding	6.672380396	-0.406733526	0.813012391	0.500279615	0.616878206	0.788881899	0.406733526
ENSMUSG00000069814	Ccdc92b	432582	protein_coding	20.71704413	0.407043986	0.306561016	-1.327774784	0.18425252	0.379300188	0.407043986
ENSMUSG00000053205	Styx	56291	protein_coding	232.9961024	-0.407172245	0.15788648	2.578892409	0.009911765	0.042284395	0.407172245
ENSMUSG00000005320	Fgfr4	14186	protein_coding	273.682636	0.407225331	0.081289309	-5.009580394	5.45E-07	7.99E-06	0.407225331
ENSMUSG00000009621	Vav2	22325	protein_coding	243.4076303	-0.407274156	0.199697567	2.039454768	0.041404661	0.129327894	0.407274156
ENSMUSG00000046242	Nme9	69797	protein_coding	4.869880917	-0.407314113	0.660506806	0.616669062	0.537453031	0.732906636	0.407314113
ENSMUSG00000030806	Stx1b	56216	protein_coding	4.475847153	-0.407314907	1.372814384	0.29670064	0.766695068	0.882867468	0.407314907
ENSMUSG00000056076	Eif3b	27979	protein_coding	1597.762711	0.407328055	0.072653084	-5.60647988	2.06E-08	3.79E-07	0.407328055
ENSMUSG00000041343	Ankrd42	73845	protein_coding	30.36852708	0.40734954	0.196270962	-2.075444762	0.037945333	0.120924206	0.40734954
ENSMUSG00000079396	Gm3411	100861615	protein_coding	2.839144112	0.407412464	0.826911838	-0.492691537	0.622230559	1	0.407412464
ENSMUSG00000032870	Smap2	69780	protein_coding	298.0693265	0.407493644	0.118567541	-3.436806058	0.000588617	0.00406542	0.407493644
ENSMUSG00000033669	Zfp7	223669	protein_coding	255.1219643	-0.407567321	0.123962596	3.287825	0.001009646	0.006402953	0.407567321
ENSMUSG00000056598	Drc3	74665	protein_coding	26.71722158	0.407597803	0.221643544	-1.838978914	0.065918288	0.182830803	0.407597803
ENSMUSG00000029413	Naaa	67111	protein_coding	193.3425136	-0.407670322	0.152513273	2.673015371	0.007517281	0.033825714	0.407670322
ENSMUSG00000058756	Thra	21833	protein_coding	528.8281767	-0.407737595	0.110869363	3.677639923	0.000235402	0.001828517	0.407737595
ENSMUSG00000026728	Vim	22352	protein_coding	72.31679523	0.407864367	0.157756916	-2.585397691	0.009726673	0.0416346	0.407864367
ENSMUSG00000040997	Abhd4	105501	protein_coding	591.9989339	-0.407917768	0.063376493	6.436420676	1.22E-10	3.00E-09	0.407917768
ENSMUSG00000044252	Osbpl1a	64291	protein_coding	121.5594616	-0.407951259	0.091662485	4.450580411	8.56E-06	9.76E-05	0.407951259
ENSMUSG00000048206	Dnajb8	56691	protein_coding	1.294606934	-0.407956891	2.296413759	0.177649559	0.858998201	1	0.407956891
ENSMUSG00000044712	Slc38a6	625098	protein_coding	57.12879755	0.407959031	0.149298562	-2.732504764	0.006285476	0.029148653	0.407959031
ENSMUSG00000111709	Gm3776	100042295	protein_coding	7.982764519	0.407966089	0.480516653	-0.849015505	0.395872665	0.618256212	0.407966089
ENSMUSG00000038578	Susd1	634731	protein_coding	19.89475593	0.408038979	0.375816737	-1.085739242	0.277594374	0.494519255	0.408038979
ENSMUSG00000059277	R74862	97423	lncRNA	7.770301938	0.408302088	0.619909911	-0.658647459	0.51012218	0.713367573	0.408302088
ENSMUSG00000079299	Klrb1	100043861	protein_coding	5.286382301	-0.408377706	0.57141929	0.714672594	0.474811376	0.686785939	0.408377706
ENSMUSG00000020738	Sumo2	170930	protein_coding	126.5173315	-0.408389726	0.22888763	1.784236773	0.074385199	0.19938276	0.408389726
ENSMUSG00000077526	Gm25379	115485641	snoRNA	2.571234655	0.408473279	0.620636136	-0.658152588	0.510440087	1	0.408473279
ENSMUSG00000029228	Lnx1	16924	protein_coding	310.3765878	-0.408532995	0.159151975	2.566936378	0.010260144	0.043456274	0.408532995
ENSMUSG00000063410	Stk24	223255	protein_coding	866.4352967	0.408569297	0.063328102	-6.45162709	1.11E-10	2.72E-09	0.408569297
ENSMUSG00000055480	Zfp458	238690	protein_coding	36.92975604	-0.408588786	0.180840306	2.259390033	0.023859134	0.084280157	0.408588786
ENSMUSG00000097207	6030443J06Rik	320719	lncRNA	16.13184403	-0.408634798	0.37558665	1.087990741	0.276599195	0.493582047	0.408634798
ENSMUSG00000032939	Nup93	71805	protein_coding	312.7117105	0.408779329	0.108404648	-3.770865313	0.000162682	0.00131693	0.408779329

Supplementary Table 1

ensembl_gene_id	external_gene_name	entrezgene_id	gene_biotype	baseMean	log2FoldChange	lfcSE	stat	pvalue	padj	absLog2FC
ENSMUSG00000073758	Sh3d21	66938	protein_coding	538.27075	-0.408800062	0.204380557	2.000190565	0.04547969	0.13890357	0.408800062
ENSMUSG00000025466	Fuom	69064	protein_coding	289.2664188	-0.408809043	0.101795932	4.015966385	5.92E-05	0.000543756	0.408809043
ENSMUSG00000022149	C9	12279	protein_coding	6.463641515	0.408835041	0.461864913	-0.885183155	0.376057856	0.598287036	0.408835041
ENSMUSG00000097571	Jpx	70252	lncRNA	26.25287773	-0.408847483	0.2877082	1.421049114	0.155302482	0.337678102	0.408847483
ENSMUSG00000025272	Tro	56191	protein_coding	2.658784496	-0.408967407	1.125940048	0.363223075	0.716438251	1	0.408967407
ENSMUSG00000002871	Tpra1	24100	protein_coding	320.8771257	-0.409121075	0.057208934	7.151349418	8.59E-13	2.62E-11	0.409121075
ENSMUSG00000026959	Grin1	14810	protein_coding	35.56391231	0.409139368	0.243016723	-1.683585236	0.092261854	0.232919564	0.409139368
ENSMUSG00000055485	Soga1	320706	protein_coding	107.1175943	0.40920219	0.288661801	-1.417583444	0.156312417	0.339025906	0.40920219
ENSMUSG00000051483	Cbr1	12408	protein_coding	2514.504006	-0.409425473	0.107979608	3.791692528	0.000149624	0.001225641	0.409425473
ENSMUSG00000034593	Myo5a	17918	protein_coding	22.87853839	0.409445697	0.281208895	-1.456019719	0.145387168	0.322533618	0.409445697
ENSMUSG00000058317	Ube2e2	218793	protein_coding	82.19895282	0.409561494	0.124686859	-3.284720607	0.001020835	0.006462165	0.409561494
ENSMUSG00000019864	Rtn4ip1	170728	protein_coding	226.3461736	-0.409615093	0.09171209	4.466315114	7.96E-06	9.14E-05	0.409615093
ENSMUSG00000022479	Vdr	22337	protein_coding	3216.802767	-0.409628891	0.086778244	4.720410019	2.35E-06	3.01E-05	0.409628891
ENSMUSG00000024712	Rfk	54391	protein_coding	4689.976775	-0.409658522	0.085233761	4.806294112	1.54E-06	2.04E-05	0.409658522
ENSMUSG00000078570	1110065P20Rik	68920	protein_coding	88.30916753	0.40975405	0.147609837	-2.775926453	0.005504466	0.026340408	0.40975405
ENSMUSG00000049908	Gja8	14616	protein_coding	3.122585891	0.409802034	1.00832342	-0.406419236	0.684434588	1	0.409802034
ENSMUSG00000042246	Tmc7	209760	protein_coding	107.6433223	-0.409809071	0.211170953	1.940650765	0.052300652	0.154451532	0.409809071
ENSMUSG00000036585	Fgf1	14164	protein_coding	433.1556149	0.409908226	0.165732343	-2.473314615	0.013386624	0.053436476	0.409908226
ENSMUSG00000031133	Arhgef6	73341	protein_coding	9.141096346	0.410173008	0.573271993	-0.715494586	0.474303485	0.686291751	0.410173008
ENSMUSG00000107121	1110006O24Rik	66123	lncRNA	6.582179506	0.410185365	0.566136321	-0.724534621	0.468737581	0.682320284	0.410185365
ENSMUSG00000084989	Crocc2	381284	protein_coding	6.341726365	0.410272984	0.566464978	-0.72426893	0.468900648	0.682480636	0.410272984
ENSMUSG00000041548	Hspb8	80888	protein_coding	3.511719659	-0.410420414	1.065871536	0.385056173	0.700195798	1	0.410420414
ENSMUSG00000025736	Jmjd8	72106	protein_coding	247.7908566	0.410494182	0.108652279	-3.778054022	0.000158059	0.001283905	0.410494182
ENSMUSG00000097637	4933417D19Rik	71186	lncRNA	4.587657589	-0.41064561	0.522979733	0.785203678	0.43233416	0.650767142	0.41064561
ENSMUSG00000075553	Gm5464	432870	lncRNA	3.988332135	-0.410709652	0.59286303	0.69275639	0.488462445	1	0.410709652
ENSMUSG00000036095	Dgkb	217480	protein_coding	3.538448214	0.410747998	2.041793829	-0.201170163	0.840565521	1	0.410747998
ENSMUSG00000015599	Ttbk1	106763	protein_coding	7.701822479	0.410928984	1.335143342	-0.307778926	0.758250558	0.878808271	0.410928984
ENSMUSG00000061878	Sphk1	20698	protein_coding	3.218030528	-0.410966841	0.87838688	0.467865414	0.639880838	1	0.410966841
ENSMUSG00000001288	Rarg	19411	protein_coding	135.8272374	-0.411080453	0.155729658	2.639705617	0.008297807	0.036642767	0.411080453
ENSMUSG00000041774	Ydjc	69101	protein_coding	343.0547874	0.411269552	0.09573773	-4.295793867	1.74E-05	0.000182282	0.411269552
ENSMUSG00000036412	Arsi	545260	protein_coding	1.347292927	0.411292248	1.663868634	-0.247190337	0.804760923	1	0.411292248
ENSMUSG00000029103	Lrpap1	16976	protein_coding	567.94931	-0.411619941	0.082169266	5.009414835	5.46E-07	7.99E-06	0.411619941
ENSMUSG00000063172	Hspb11	72938	protein_coding	108.0959343	-0.411689647	0.140572439	2.928665463	0.003404206	0.017689445	0.411689647
ENSMUSG00000037805	Rpl10a	19896	protein_coding	2970.473524	-0.411722483	0.126527478	3.254016349	0.001137857	0.007085436	0.411722483
ENSMUSG00000068748	Ptprz1	19283	protein_coding	3.162340113	0.411786625	2.415064787	-0.170507486	0.864611048	1	0.411786625
ENSMUSG00000100164	2610306M01Rik	67170	lncRNA	31.91827091	-0.411824729	0.216635835	1.901000028	0.057302009	0.165147848	0.411824729
ENSMUSG00000049764	Zfp280b	64453	protein_coding	228.0917877	0.411904259	0.091822578	-4.485871232	7.26E-06	8.40E-05	0.411904259
ENSMUSG00000062590	Armc9	78795	protein_coding	82.94303056	0.412267579	0.165268941	-2.494525445	0.012612568	0.051024976	0.412267579
ENSMUSG00000031026	Trim66	330627	protein_coding	5.312342899	-0.412327019	1.490681665	0.276602999	0.782084962	0.890758364	0.412327019

ensembl_gene_id	external_gene_name	entrezgene_id	gene_biotype	baseMean	log2FoldChange	lfcSE	stat	pvalue	padj	absLog2FC
ENSMUSG00000030602	Pak4	70584	protein_coding	1462.60188	0.412332765	0.062335032	-6.614783918	3.72E-11	9.85E-10	0.412332765
ENSMUSG00000064127	Med14	26896	protein_coding	562.5313857	0.412353565	0.101655414	-4.056385674	4.98E-05	0.000465269	0.412353565
ENSMUSG00000039168	Dap	223453	protein_coding	490.0122428	0.412401096	0.079331371	-5.198461722	2.01E-07	3.15E-06	0.412401096
ENSMUSG00000034918	Cdhr2	268663	protein_coding	7592.048941	-0.412417106	0.126379394	3.26332556	0.001101129	0.006903589	0.412417106
ENSMUSG00000037072	Selenof	93684	protein_coding	2022.303617	0.412440072	0.121187083	-3.403333597	0.000665689	0.004503147	0.412440072
ENSMUSG00000021180	Rps6ka5	73086	protein_coding	44.9060221	0.412441705	0.198025491	-2.082770772	0.037272123	0.119192842	0.412441705
ENSMUSG00000020422	Tns3	319939	protein_coding	1689.31868	-0.412545758	0.070033731	5.890672302	3.85E-09	7.89E-08	0.412545758
ENSMUSG00000031767	Nudt7	67528	protein_coding	217.7972619	-0.412576771	0.088569625	4.658219702	3.19E-06	3.96E-05	0.412576771
ENSMUSG00000089715	Cbx6	494448	protein_coding	82.99351455	0.412682315	0.219098283	-1.88354883	0.059626029	0.170033542	0.412682315
ENSMUSG00000031828	Klhl36	234796	protein_coding	68.32190722	-0.41268764	0.126764307	3.255550793	0.001131727	0.00706591	0.41268764
ENSMUSG00000029162	Khk	16548	protein_coding	134.4899403	-0.412748484	0.127711538	3.231880938	0.001229783	0.007552486	0.412748484
ENSMUSG00000033565	Rbfox2	93686	protein_coding	361.2651183	0.412781419	0.071794693	-5.749469794	8.95E-09	1.74E-07	0.412781419
ENSMUSG00000030704	Rab6a	19346	protein_coding	1432.979333	0.412851889	0.06305605	-6.547379499	5.86E-11	1.52E-09	0.412851889
ENSMUSG00000062833	Sval3	387564	protein_coding	4.790788896	0.4128569	0.687174612	-0.600803483	0.547970884	0.739252608	0.4128569
ENSMUSG00000082361	Btc	12223	protein_coding	63.08619396	-0.412942233	0.186326413	2.216230258	0.026675737	0.091871348	0.412942233
ENSMUSG00000014301	Pam16	66449	protein_coding	87.09081804	-0.413026042	0.137173338	3.010979014	0.002604068	0.014104161	0.413026042
ENSMUSG00000024304	Cdh2	12558	protein_coding	6.464159667	0.413066099	0.719828952	-0.573839241	0.566076592	0.752646336	0.413066099
ENSMUSG00000099354	1700124L16Rik	67934	lncRNA	0.685650798	-0.413123186	1.39877487	0.295346445	0.76772924	1	0.413123186
ENSMUSG00000054753	AU018091	245128	protein_coding	2.392429008	0.413282614	2.1753595	-0.189983593	0.849321987	1	0.413282614
ENSMUSG00000029736	Nobox	18291	protein_coding	0.833255486	0.413340268	1.692946202	-0.244154402	0.80711124	1	0.413340268
ENSMUSG00000000296	Tpd52l1	21987	protein_coding	78.33771218	0.413547683	0.190720977	-2.168338735	0.030132923	0.101319853	0.413547683
ENSMUSG00000042426	Dhx29	218629	protein_coding	249.0460981	0.413667927	0.120111257	-3.444039626	0.000573092	0.003974816	0.413667927
ENSMUSG00000097113	Gm19705	100503460	lncRNA	2.616431859	0.413701185	1.006424355	-0.411060387	0.681028256	1	0.413701185
ENSMUSG00000019773	Fbxo5	67141	protein_coding	97.9753439	0.413797233	0.241405237	-1.714118708	0.086506927	0.222484875	0.413797233
ENSMUSG00000027854	Sike1	66641	protein_coding	585.5447606	-0.413898966	0.130931177	3.161194878	0.001571233	0.00926691	0.413898966
ENSMUSG00000005374	Tbl2	27368	protein_coding	379.9095726	0.413921531	0.112253463	-3.687383161	0.000226572	0.001766577	0.413921531
ENSMUSG00000020101	Vsir	74048	protein_coding	16.64102361	0.414086236	0.437578318	-0.946313424	0.343988749	0.56579982	0.414086236
ENSMUSG00000031805	Jak3	16453	protein_coding	181.3281232	0.414112329	0.12827471	-3.228324037	0.001245178	0.00763001	0.414112329
ENSMUSG00000061039	Or8b53	258783	protein_coding	3.838933201	-0.414137856	0.800215186	0.517533112	0.60478406	1	0.414137856
ENSMUSG00000117797	2900057B20Rik	73009	lncRNA	5.78138608	0.414138901	0.43057094	-0.961836628	0.336131676	0.558663295	0.414138901
ENSMUSG00000032555	Topbp1	235559	protein_coding	694.566745	0.414152072	0.146765096	-2.821870341	0.004774448	0.02338927	0.414152072
ENSMUSG00000086287	Gm15972	100502834	lncRNA	13.3700495	-0.414267303	0.317792167	1.303579336	0.19237705	0.390140563	0.414267303
ENSMUSG00000021482	Prxl2c	66129	protein_coding	448.7733581	-0.414276826	0.129844445	3.190562569	0.001419961	0.008514609	0.414276826
ENSMUSG00000024339	Tap2	21355	protein_coding	217.4976814	-0.414388914	0.092837232	4.463606945	8.06E-06	9.24E-05	0.414388914
ENSMUSG00000002324	Rec8	56739	protein_coding	48.38913692	0.414504439	0.333245067	-1.243842684	0.213557467	0.418548305	0.414504439
ENSMUSG00000004032	Gstm5	14866	protein_coding	346.8949509	0.414606999	0.122686725	-3.379395768	0.000726453	0.004833462	0.414606999
ENSMUSG00000041477	Dcp1b	319618	protein_coding	148.6414247	-0.41463763	0.163986875	2.528480592	0.011455742	0.047449241	0.41463763
ENSMUSG00000029541	Cyp2w1	545817	protein_coding	86.99935248	0.414683309	0.288060409	-1.439570644	0.14998891	0.329684129	0.414683309
ENSMUSG00000038831	Ralgps1	241308	protein_coding	161.0108101	0.414733361	0.105608655	-3.92707737	8.60E-05	0.000754888	0.414733361

ensembl_gene_id	external_gene_name	entrezgene_id	gene_biotype	baseMean	log2FoldChange	lfcSE	stat	pvalue	padj	absLog2FC
ENSMUSG00000036887	C1qa	12259	protein_coding	5.829170395	0.414858017	0.515574654	-0.804651691	0.421020704	0.640805153	0.414858017
ENSMUSG00000022483	Col2a1	12824	protein_coding	8.296944246	0.414867241	1.33311189	-0.311202116	0.755646975	0.877241064	0.414867241
ENSMUSG00000006720	Zfp184	193452	protein_coding	14.12924868	0.415103248	0.311607123	-1.332136583	0.182815311	0.377565289	0.415103248
ENSMUSG00000106193	Mir7090	102465661	miRNA	0.821431154	-0.415127297	1.133423718	0.366259582	0.714171386	1	0.415127297
ENSMUSG00000026708	Cenpl	70454	protein_coding	90.95224322	0.415274284	0.176226256	-2.356483614	0.018448882	0.068863768	0.415274284
ENSMUSG00000032637	Atxn2l	233871	protein_coding	676.6525257	0.415279316	0.161402603	-2.57294064	0.01008385	0.042874537	0.415279316
ENSMUSG00000055067	Smyd3	69726	protein_coding	100.4989981	-0.415305598	0.132629476	3.131322012	0.001740212	0.010094213	0.415305598
ENSMUSG00000016481	Cr1l	12946	protein_coding	753.9549658	-0.41540833	0.095546314	4.347716977	1.38E-05	0.00014838	0.41540833
ENSMUSG00000029163	Emilin1	100952	protein_coding	32.71842405	0.415574175	0.220588394	-1.883934905	0.059573781	0.169937908	0.415574175
ENSMUSG00000030083	Abtb1	80283	protein_coding	260.8181473	-0.415717512	0.11156283	3.726308427	0.000194305	0.001541925	0.415717512
ENSMUSG00000038612	Mcl1	17210	protein_coding	4154.089562	-0.415719009	0.083076759	5.004034981	5.61E-07	8.20E-06	0.415719009
ENSMUSG00000034209	Rasl10a	75668	protein_coding	3.444711318	-0.415752372	0.583815028	0.712130302	0.476384092	1	0.415752372
ENSMUSG00000063730	Hsd3b2	15493	protein_coding	341.619233	-0.415919087	0.169175627	2.458504773	0.013951692	0.055106014	0.415919087
ENSMUSG00000078970	Dnaaf10	103784	protein_coding	241.0445353	0.41601399	0.126122728	-3.298485501	0.000972079	0.00621724	0.41601399
ENSMUSG00000042262	Ccr8	12776	protein_coding	0.835391382	-0.416086163	1.616693917	0.257368546	0.796894282	1	0.416086163
ENSMUSG00000003863	Ppfia3	76787	protein_coding	353.5353585	-0.416127215	0.237463575	1.752383352	0.079707912	0.209275846	0.416127215
ENSMUSG00000090667	Mdfic2	330390	protein_coding	1.486721491	0.416245099	1.575926929	-0.264127157	0.791681939	1	0.416245099
ENSMUSG00000071573	Rnls	67795	protein_coding	35.01362822	-0.416341212	0.199323735	2.088768864	0.036728534	0.1177122	0.416341212
ENSMUSG00000040795	Iqcc	230767	protein_coding	93.52777409	-0.416386795	0.112882264	3.688682179	0.000225419	0.001759245	0.416386795
ENSMUSG00000100826	Snhg14	16353	lncRNA	30.58017426	-0.416559237	0.288474313	1.444008073	0.148736704	0.327585748	0.416559237
ENSMUSG00000008855	Hdac5	15184	protein_coding	507.4996003	-0.416716365	0.112610238	3.700519353	0.000215159	0.001685546	0.416716365
ENSMUSG00000034438	Gbp8	76074	protein_coding	7.810724718	-0.416906976	0.525741826	0.792988031	0.427784796	0.647057325	0.416906976
ENSMUSG00000061451	Tmem151a	381199	protein_coding	15.93672208	0.417005729	0.321931193	-1.295325642	0.195207917	0.394331932	0.417005729
ENSMUSG00000034112	Atp2c2	69047	protein_coding	745.9400414	-0.417013785	0.257750979	1.617894092	0.105685424	0.257759428	0.417013785
ENSMUSG00000040433	Zbtb38	245007	protein_coding	689.081318	-0.417106968	0.089030744	4.684976781	2.80E-06	3.51E-05	0.417106968
ENSMUSG00000025933	Tmem14a	75712	protein_coding	29.27309587	-0.417110704	0.244784543	1.703991188	0.088382734	0.22572726	0.417110704
ENSMUSG00000055305	Zfp93	22755	protein_coding	70.12331837	-0.417169818	0.151596063	2.751851261	0.005925943	0.027912442	0.417169818
ENSMUSG00000029156	Sgcb	24051	protein_coding	28.94618125	-0.417222293	0.181778015	2.295229665	0.021719966	0.078315179	0.417222293
ENSMUSG00000053931	Cnn3	71994	protein_coding	189.0305939	-0.417245677	0.148287236	2.813766644	0.004896476	0.023887859	0.417245677
ENSMUSG00000060675	Plaat3	225845	protein_coding	503.1832793	-0.417444726	0.107384193	3.887394532	0.000101326	0.000870602	0.417444726
ENSMUSG00000048498	Cd300e	217306	protein_coding	0.750245638	-0.41753209	1.675542718	0.249192149	0.803212152	1	0.41753209
ENSMUSG00000048481	Mypop	232934	protein_coding	20.6380703	-0.417565205	0.306592419	1.361955416	0.173211956	0.363760214	0.417565205
ENSMUSG00000050994	Adgb	215772	protein_coding	6.186712567	0.417919196	0.955372219	-0.437441228	0.66179139	0.816925028	0.417919196
ENSMUSG00000062204	Or2y8	211472	protein_coding	1.526547279	-0.418026196	1.494334917	0.279740633	0.779676502	1	0.418026196
ENSMUSG00000057880	Abat	268860	protein_coding	3251.617225	-0.418034489	0.142943452	2.924474557	0.003450383	0.017884376	0.418034489
ENSMUSG00000041650	Pcca	110821	protein_coding	479.6104837	-0.418037183	0.091030756	4.592263099	4.38E-06	5.31E-05	0.418037183
ENSMUSG00000030411	Nova2	384569	protein_coding	5.986011185	0.418197266	0.874027329	-0.478471613	0.632314574	0.799512805	0.418197266
ENSMUSG00000021303	Gng4	14706	protein_coding	9.516874143	-0.418210736	0.42917396	0.974455058	0.329830643	0.552046615	0.418210736
ENSMUSG00000056643	Chst13	71797	protein_coding	2.458583394	0.418272136	0.771890281	-0.541880299	0.587900969	1	0.418272136

ensembl_gene_id	external_gene_name	entrezgene_id	gene_biotype	baseMean	log2FoldChange	lfcSE	stat	pvalue	padj	absLog2FC
ENSMUSG00000020423	Btg2	12227	protein_coding	6560.98182	-0.418378903	0.16128749	2.593994755	0.009486793	0.040813086	0.418378903
ENSMUSG00000010142	Tnfrsf13b	57916	protein_coding	2.288464675	0.418415659	1.051021896	-0.398103655	0.69055378	1	0.418415659
ENSMUSG00000043556	Fbxl7	448987	protein_coding	2.043911232	-0.41852882	3.094800825	0.135236108	0.892425199	1	0.41852882
ENSMUSG00000039431	Mtmr7	54384	protein_coding	255.3294178	0.418618779	0.096109949	-4.355623752	1.33E-05	0.000143449	0.418618779
ENSMUSG00000036086	Zranb3	226409	protein_coding	67.09382733	0.418656907	0.17788128	-2.353574845	0.018593864	0.069313561	0.418656907
ENSMUSG00000068015	Lrch1	380916	protein_coding	319.0378765	-0.418659108	0.088729375	4.718382276	2.38E-06	3.04E-05	0.418659108
ENSMUSG00000024854	Pold4	69745	protein_coding	156.599412	0.418883845	0.118253425	-3.542255504	0.000396721	0.002910462	0.418883845
ENSMUSG00000029468	P2rx7	18439	protein_coding	41.83642883	-0.419010578	0.234605307	1.786023442	0.074095463	0.198998609	0.419010578
ENSMUSG00000021767	Kat6b	54169	protein_coding	592.6017423	0.41901964	0.092616078	-4.524264573	6.06E-06	7.11E-05	0.41901964
ENSMUSG00000027875	Hmgcs2	15360	protein_coding	13878.88844	-0.41916398	0.126489755	3.313817638	0.000920315	0.005929802	0.41916398
ENSMUSG00000030209	Grin2b	14812	protein_coding	12.16565868	-0.419194825	0.778328852	0.538583176	0.590174499	0.770846581	0.419194825
ENSMUSG00000026853	Crat	12908	protein_coding	636.1319498	-0.419260572	0.06375544	6.576075248	4.83E-11	1.27E-09	0.419260572
ENSMUSG00000100210	H3c7	260423	protein_coding	1190.312132	0.419485542	0.196292553	-2.137042575	0.032594529	0.107588657	0.419485542
ENSMUSG00000020471	Pold2	18972	protein_coding	445.3870034	0.419496795	0.115349389	-3.636749166	0.000276101	0.002116771	0.419496795
ENSMUSG00000020886	Dlg4	13385	protein_coding	14.10521162	0.419656632	0.764481736	-0.548942653	0.583044806	0.765756487	0.419656632
ENSMUSG00000021754	Map3k1	26401	protein_coding	544.2573616	-0.419675227	0.065505315	6.40673546	1.49E-10	3.61E-09	0.419675227
ENSMUSG00000051439	Cd14	12475	protein_coding	378.8724501	0.419793558	0.218200702	-1.923887292	0.054368712	0.158736855	0.419793558
ENSMUSG00000024313	Mep1b	17288	protein_coding	1092.926499	-0.419936155	0.159042706	2.640398708	0.008280854	0.036593531	0.419936155
ENSMUSG00000010755	Cars	27267	protein_coding	358.041771	0.419942097	0.074504199	-5.636488994	1.74E-08	3.22E-07	0.419942097
ENSMUSG00000026027	Stradb	227154	protein_coding	173.9445148	-0.419942398	0.112745963	3.724677912	0.000195565	0.001548948	0.419942398
ENSMUSG00000027950	Chrnb2	11444	protein_coding	35.67048712	-0.419950478	0.172708636	2.431554602	0.015034182	0.058500114	0.419950478
ENSMUSG00000038980	Rbbp8nl	271887	protein_coding	53.67869189	0.419971073	0.181439858	-2.314657204	0.0206317	0.075129413	0.419971073
ENSMUSG00000028772	Zcchc17	619605	protein_coding	380.6274014	0.420082396	0.077705023	-5.406116337	6.44E-08	1.09E-06	0.420082396
ENSMUSG00000032289	Thsd4	207596	protein_coding	414.198872	-0.420332611	0.150984236	2.783950317	0.005370126	0.025839819	0.420332611
ENSMUSG00000037526	Atg14	100504663	protein_coding	152.2864395	-0.42045472	0.121457427	3.461745643	0.000536684	0.003768198	0.42045472
ENSMUSG00000047880	Cxcr5	12145	protein_coding	5.137772515	0.420498878	0.646141822	-0.650784184	0.515185811	0.716335759	0.420498878
ENSMUSG00000005198	Polr2a	20020	protein_coding	583.2022804	0.420697282	0.3025741	-1.390394227	0.164409198	0.350782106	0.420697282
ENSMUSG00000050002	Idnk	75731	protein_coding	257.5339971	-0.420714233	0.07420308	5.669767767	1.43E-08	2.69E-07	0.420714233
ENSMUSG00000087273	Gm13203	115489366	lncRNA	5.95722193	-0.42073824	0.548983427	0.766395158	0.443441213	0.660955754	0.42073824
ENSMUSG00000020461	Clhc1	73324	protein_coding	1.993614873	0.42111257	1.002016435	-0.420265133	0.674291778	1	0.42111257
ENSMUSG00000045094	Arhgef37	328967	protein_coding	117.6867656	-0.421130106	0.135843806	3.100105327	0.001934518	0.011044625	0.421130106
ENSMUSG00000100583	Gm29443	102634559	lncRNA	1.214411167	-0.421171803	1.853718199	0.227203791	0.82026528	1	0.421171803
ENSMUSG00000015316	Slamf1	27218	protein_coding	0.858326258	-0.421269889	1.859354727	0.226567789	0.820759842	1	0.421269889
ENSMUSG00000037325	Bbs7	71492	protein_coding	78.30475049	0.42131402	0.135778432	-3.102952467	0.001916004	0.01096738	0.42131402
ENSMUSG00000045246	Kcng4	66733	protein_coding	1.00490653	-0.421410452	1.867371819	0.22567035	0.821457821	1	0.421410452
ENSMUSG00000089125	Gm26403	115488986	snRNA	1.693491278	-0.421417963	0.899822807	0.468334387	0.639545481	1	0.421417963
ENSMUSG00000112941	Gm48623	12890	lncRNA	3.777080459	-0.421612106	0.809882957	0.520583996	0.6026566	1	0.421612106
ENSMUSG00000027286	Lrrc57	66606	protein_coding	84.87838517	-0.421615767	0.128323947	3.285557976	0.001017806	0.00644975	0.421615767
ENSMUSG00000019873	Reep3	28193	protein_coding	876.4242171	-0.421705063	0.071666058	5.88430666	4.00E-09	8.18E-08	0.421705063

ensembl_gene_id	external_gene_name	entrezgene_id	gene_biotype	baseMean	log2FoldChange	lfcSE	stat	pvalue	padj	absLog2FC
ENSMUSG00000033713	Foxn3	71375	protein_coding	850.1754611	-0.421755796	0.091750823	4.596752191	4.29E-06	5.21E-05	0.421755796
ENSMUSG00000063142	Kcnma1	16531	protein_coding	198.847557	-0.421793642	0.216038398	1.952401272	0.050890582	0.151098	0.421793642
ENSMUSG00000064326	Siva1	30954	protein_coding	92.09133519	-0.421875945	0.137342372	3.071710058	0.002128364	0.011930684	0.421875945
ENSMUSG00000027962	Vcam1	22329	protein_coding	6.427941917	-0.421985447	0.686537449	0.614657581	0.538780879	0.733468492	0.421985447
ENSMUSG00000055912	Tmem150a	232086	protein_coding	8.42677998	-0.42212035	0.49168472	0.858518341	0.390606308	0.612813278	0.42212035
ENSMUSG00000071711	Mpst	246221	protein_coding	2582.223104	0.422374913	0.17906956	-2.358719788	0.018338097	0.068530286	0.422374913
ENSMUSG00000056738	A730036I17Rik	329513	lncRNA	2.437069142	-0.422516346	0.99650065	0.424000071	0.671565762	1	0.422516346
ENSMUSG00000020135	Apc2	23805	protein_coding	15.61390409	0.422626105	0.532864901	-0.793120552	0.42770759	0.647057325	0.422626105
ENSMUSG00000035024	Ncapd3	78658	protein_coding	389.2009247	0.422683285	0.10983479	-3.848355189	0.000118914	0.001004984	0.422683285
ENSMUSG00000047881	Rell1	100532	protein_coding	653.5286486	0.422717325	0.073243184	-5.771422032	7.86E-09	1.54E-07	0.422717325
ENSMUSG00000027861	Casq2	12373	protein_coding	1.614685553	-0.422827309	1.72494433	0.245125192	0.806359498	1	0.422827309
ENSMUSG00000021703	Serinc5	218442	protein_coding	1040.890113	-0.423007546	0.136715717	3.094066697	0.001974331	0.011219581	0.423007546
ENSMUSG00000032294	Pkm	18746	protein_coding	7768.212112	-0.423037532	0.103991276	4.06800983	4.74E-05	0.000445412	0.423037532
ENSMUSG00000062997	Rpl35	66489	protein_coding	1045.800546	-0.423060183	0.137574484	3.075135526	0.002104069	0.011831403	0.423060183
ENSMUSG00000042371	Slc5a10	109342	protein_coding	5.357167305	0.423119598	0.797136874	-0.530799178	0.59555795	0.773864504	0.423119598
ENSMUSG00000086241	4930483K19Rik	74953	lncRNA	3.193818796	-0.423302653	0.673170819	0.628819076	0.52946751	1	0.423302653
ENSMUSG00000000934	Top1mt	72960	protein_coding	69.74926484	-0.423332295	0.153976646	2.749327936	0.005971761	0.028080198	0.423332295
ENSMUSG00000021036	Sptlc2	20773	protein_coding	3490.325014	0.423421325	0.053607084	-7.898607685	2.82E-15	1.05E-13	0.423421325
ENSMUSG00000030852	Tacc2	57752	protein_coding	652.5990578	-0.423441562	0.166071035	2.549761692	0.010779658	0.045227455	0.423441562
ENSMUSG00000024498	Tcerg1	56070	protein_coding	511.0436432	0.423452835	0.087398022	-4.845107787	1.27E-06	1.71E-05	0.423452835
ENSMUSG00000034108	Ccs	12460	protein_coding	440.7266292	-0.423458229	0.08565375	4.943837606	7.66E-07	1.09E-05	0.423458229
ENSMUSG00000038541	Srd5a2	94224	protein_coding	33.3534861	-0.423570324	0.341457435	1.24047767	0.214798769	0.419775423	0.423570324
ENSMUSG00000064147	Rab44	442827	protein_coding	3.886678246	-0.423579577	1.195623944	0.35427492	0.723132862	1	0.423579577
ENSMUSG00000069833	Ahnak	66395	protein_coding	15650.19897	-0.423686964	0.139804322	3.030571292	0.002440916	0.013347443	0.423686964
ENSMUSG00000060579	Fhit	14198	protein_coding	69.54354836	0.423700373	0.147897082	-2.864832538	0.004172298	0.020879192	0.423700373
ENSMUSG00000036136	Fam110c	104943	protein_coding	435.6957207	-0.423701253	0.097742115	4.334889358	1.46E-05	0.000155877	0.423701253
ENSMUSG00000068744	Psrc1	56742	protein_coding	44.07955277	0.423807377	0.205278413	-2.064549167	0.038965677	0.123437405	0.423807377
ENSMUSG00000025978	Rftn2	74013	protein_coding	49.10049141	-0.423819683	0.175884957	2.40964145	0.015968205	0.061425328	0.423819683
ENSMUSG00000050410	Tcf19	106795	protein_coding	143.3826344	0.424011127	0.208655389	-2.032112041	0.042142315	0.130987801	0.424011127
ENSMUSG00000034107	Ano7	404545	protein_coding	430.9897189	-0.424018368	0.130709414	3.243977265	0.001178732	0.007304155	0.424018368
ENSMUSG00000020286	1700093K21Rik	67358	protein_coding	0.778796039	-0.424199072	2.049897089	0.206936765	0.836059244	1	0.424199072
ENSMUSG00000050612	Txndc2	213272	protein_coding	0.807100321	-0.424310931	2.058222134	0.2061541	0.836670541	1	0.424310931
ENSMUSG00000001520	Nrip2	60345	protein_coding	8.123189256	0.424324004	0.41643322	-1.018948499	0.308227417	0.528838189	0.424324004
ENSMUSG00000017830	Dhx58	80861	protein_coding	104.0843335	-0.424373158	0.206649329	2.053590782	0.040015315	0.125929956	0.424373158
ENSMUSG00000087141	Plcxd2	433022	protein_coding	213.4427945	0.424377379	0.11143597	-3.808262075	0.000139947	0.001159019	0.424377379
ENSMUSG00000045903	Npas4	225872	protein_coding	1.95850209	0.424507006	1.468043082	-0.289165224	0.772454943	1	0.424507006
ENSMUSG00000039842	Mcph1	244329	protein_coding	162.6017771	0.424533018	0.108972375	-3.895785692	9.79E-05	0.000844958	0.424533018
ENSMUSG00000070489	4930527J03Rik	75164	pseudogene	1.638540011	-0.424564057	0.870362873	0.487801204	0.625690659	1	0.424564057
ENSMUSG00000035849	Krt222	268481	protein_coding	8.810325165	-0.424577185	0.439287723	0.966512751	0.33378767	0.556110732	0.424577185

Supplementary Table 1

ensembl_gene_id	external_gene_name	entrezgene_id	gene_biotype	baseMean	log2FoldChange	lfcSE	stat	pvalue	padj	absLog2FC
ENSMUSG00000046408	1700067K01Rik	73453	protein_coding	3.970932652	-0.42467318	0.733686831	0.578820775	0.562710112	1	0.42467318
ENSMUSG00000024187	Fam234a	106581	protein_coding	3133.495001	-0.424675033	0.061051344	6.95603086	3.50E-12	1.02E-10	0.424675033
ENSMUSG00000024565	Sall3	20689	protein_coding	1.97652087	-0.42467932	2.086225832	0.203563446	0.838694661	1	0.42467932
ENSMUSG00000027863	Cd2	12481	protein_coding	2.814823708	-0.424758546	0.841185783	0.504952121	0.613592465	1	0.424758546
ENSMUSG00000054793	Cadm4	260299	protein_coding	8.976264229	0.424916982	0.474719057	-0.895091477	0.37073819	0.593065878	0.424916982
ENSMUSG00000013643	Lypd8	70163	protein_coding	50651.70691	-0.424951336	0.101626552	4.181499091	2.90E-05	0.000287573	0.424951336
ENSMUSG00000038259	Gdf5	14563	protein_coding	1.079146846	-0.425057561	2.116749004	0.200806784	0.840849658	1	0.425057561
ENSMUSG00000032604	Qars	97541	protein_coding	2147.676628	0.425082825	0.070418913	-6.036486621	1.58E-09	3.40E-08	0.425082825
ENSMUSG00000036678	Aaas	223921	protein_coding	167.7605063	0.425184094	0.140347426	-3.029511173	0.002449498	0.013385512	0.425184094
ENSMUSG00000036882	Arhgap33	233071	protein_coding	29.67500558	0.425186007	0.353699448	-1.202111029	0.229320514	0.438111017	0.425186007
ENSMUSG00000044667	Plppr4	229791	protein_coding	1.619479293	-0.42524228	2.131793987	0.199476254	0.841890216	1	0.42524228
ENSMUSG00000046049	Rp1l1	271209	protein_coding	1.710789745	-0.425289985	2.135870801	0.199117842	0.842170565	1	0.425289985
ENSMUSG00000069041	Slc25a31	73333	protein_coding	0.823246668	0.425301347	1.372281726	-0.309922765	0.75661969	1	0.425301347
ENSMUSG00000059114	Lrrc74a	627607	protein_coding	1.429863731	-0.425304125	2.137037686	0.199015735	0.842250436	1	0.425304125
ENSMUSG00000029074	Ttll10	330010	protein_coding	186.7450912	-0.425335724	0.209286215	2.032316003	0.042121675	0.130972952	0.425335724
ENSMUSG00000018593	Sparc	20692	protein_coding	6.325948919	0.425387635	0.676686072	-0.628633649	0.529588926	0.727166502	0.425387635
ENSMUSG00000022432	Smc1b	140557	protein_coding	1.771050683	-0.425391833	2.144296372	0.198382947	0.842745459	1	0.425391833
ENSMUSG00000043259	Fam13c	71721	protein_coding	2.035142422	-0.425416316	2.146331835	0.198206218	0.842883723	1	0.425416316
ENSMUSG00000096916	Zfp850	100043772	protein_coding	58.13774274	0.425442114	0.142285798	-2.990053252	0.002789288	0.014979611	0.425442114
ENSMUSG00000038770	Kpna7	381686	protein_coding	1.520058982	-0.42545685	2.149902108	0.197895917	0.843126499	1	0.42545685
ENSMUSG00000040767	Snrnp25	78372	protein_coding	147.0721174	-0.425458636	0.137170018	3.101688272	0.001924205	0.01100288	0.425458636
ENSMUSG00000054708	Ankrd24	70615	protein_coding	112.410448	-0.42546107	0.145874844	2.91661713	0.003538498	0.018266571	0.42546107
ENSMUSG00000022269	Marchf11	211147	protein_coding	0.978956374	-0.425475504	2.151729647	0.197736507	0.843251225	1	0.425475504
ENSMUSG00000031928	Mre11a	17535	protein_coding	274.1701846	0.42552679	0.098397801	-4.32455589	1.53E-05	0.000162315	0.42552679
ENSMUSG00000024973	Plaat5	66727	protein_coding	3.856383712	0.425595983	0.628307128	-0.677369337	0.498171648	1	0.425595983
ENSMUSG00000034220	Gpc1	14733	protein_coding	35.96399926	-0.425637148	0.197303714	2.157268804	0.030984726	0.103453653	0.425637148
ENSMUSG00000042961	Egflam	268780	protein_coding	5.89378942	0.425700475	1.05603688	-0.403111371	0.686866296	0.83324102	0.425700475
ENSMUSG00000070883	Ccdc173	75051	protein_coding	8.128672288	0.425730796	0.370452829	-1.149217288	0.250466394	0.462725114	0.425730796
ENSMUSG00000060126	Tpt1	22070	protein_coding	8051.526135	-0.425796484	0.097175452	4.381728856	1.18E-05	0.000129591	0.425796484
ENSMUSG00000062093	Gm10110	328451	transcribed_processed_pseudogene	6.210441477	0.425796703	0.495509066	-0.85931163	0.39016861	0.612475705	0.425796703
ENSMUSG00000061533	Cep128	75216	protein_coding	106.2754082	0.425902809	0.178287828	-2.388849614	0.016901219	0.064265646	0.425902809
ENSMUSG00000002057	Foxn1	15218	protein_coding	21.24176094	0.426083349	0.36793535	-1.158038632	0.246848272	0.458698364	0.426083349
ENSMUSG00000021182	Ccdc88c	68339	protein_coding	1296.866558	0.426123328	0.077437168	-5.502826879	3.74E-08	6.56E-07	0.426123328
ENSMUSG00000029922	Mkrn1	54484	protein_coding	2460.244198	-0.42612796	0.070361348	6.0562791	1.39E-09	3.02E-08	0.42612796
ENSMUSG00000087165	2010001A14Rik	76516	lncRNA	54.9024863	-0.426299491	0.153033997	2.785652209	0.005342015	0.025742061	0.426299491
ENSMUSG00000020919	Stat5b	20851	protein_coding	281.305574	-0.426376201	0.071972895	5.924121889	3.14E-09	6.51E-08	0.426376201
ENSMUSG00000005824	Tnfsf14	50930	protein_coding	2.183785361	0.426423076	0.991325072	-0.430154637	0.667083158	1	0.426423076
ENSMUSG00000016206	H2-M3	14991	protein_coding	60.70927599	0.426503751	0.164276453	-2.596256149	0.009424578	0.040647029	0.426503751
ENSMUSG00000048148	Nwd1	319555	protein_coding	4.261356306	0.426548366	1.56029956	-0.273375944	0.784564242	1	0.426548366

Supplementary Table 1

ensembl_gene_id	external_gene_name	entrezgene_id	gene_biotype	baseMean	log2FoldChange	lfcSE	stat	pvalue	padj	absLog2FC
ENSMUSG00000024194	Cuta	67675	protein_coding	415.1820319	0.426591251	0.095328637	-4.474953853	7.64E-06	8.81E-05	0.426591251
ENSMUSG00000095253	Zfp799	240064	protein_coding	161.8967245	-0.426699732	0.148015478	2.8828048	0.003941516	0.01996653	0.426699732
ENSMUSG00000066113	Adamtsl1	77739	protein_coding	4.451857525	0.426709736	1.609615805	-0.265100364	0.790932147	0.89578585	0.426709736
ENSMUSG00000033276	Stk36	269209	protein_coding	9.827059859	0.426763775	0.396129862	-1.077333006	0.281331522	0.499023496	0.426763775
ENSMUSG00000067889	Sptbn2	20743	protein_coding	223.3785657	-0.42676438	0.16364717	2.607832323	0.009111758	0.039545746	0.42676438
ENSMUSG00000019278	Dpep1	13479	protein_coding	3476.834646	-0.426998924	0.147296142	2.898914509	0.00374457	0.019150767	0.426998924
ENSMUSG00000025911	Adhfe1	76187	protein_coding	17.42564255	-0.427057615	0.308193512	1.385680098	0.165844608	0.353023699	0.427057615
ENSMUSG00000038884	Shfl	319278	protein_coding	25.32259633	-0.427097759	0.225698884	1.892334387	0.058446442	0.167508599	0.427097759
ENSMUSG00000029851	Tcaf2	232748	protein_coding	295.52622	-0.427202432	0.163622684	2.610899792	0.009030435	0.03924437	0.427202432
ENSMUSG00000048583	Igf2	16002	protein_coding	1.886294065	-0.427325	1.752262815	0.243870381	0.807331209	1	0.427325
ENSMUSG00000115149	9330188P03Rik	380930	lncRNA	15.26499233	-0.427361405	0.263774404	1.620177693	0.105194112	0.256689693	0.427361405
ENSMUSG00000044770	Scml4	268297	protein_coding	607.8272101	-0.427429495	0.16901251	2.528981411	0.011439409	0.047393475	0.427429495
ENSMUSG00000040329	Il7	16196	protein_coding	104.7828703	0.427540654	0.167388881	-2.554175954	0.010643945	0.044806045	0.427540654
ENSMUSG00000074261	Erich4	632778	protein_coding	54.29682545	0.427615698	0.240700271	-1.776548469	0.075642561	0.20160769	0.427615698
ENSMUSG00000041313	Slc7a1	11987	protein_coding	485.5017483	0.427665761	0.104250346	-4.10229584	4.09E-05	0.000392527	0.427665761
ENSMUSG00000032999	Nlrp4f	97895	protein_coding	1.118845089	-0.427671646	2.366524379	0.180717194	0.856589562	1	0.427671646
ENSMUSG00000016493	Cd46	17221	protein_coding	23.22949885	-0.42768496	0.251055612	1.703546704	0.088465806	0.22586956	0.42768496
ENSMUSG00000028938	Galntl5	67909	protein_coding	0.937598424	-0.427686945	2.368391335	0.180581198	0.856696315	1	0.427686945
ENSMUSG00000083193	4930595D18Rik	78062	lncRNA	2.917893146	-0.42780435	0.781597905	0.547345824	0.584141167	1	0.42780435
ENSMUSG00000073198	Bnip3l-ps	100043324	transcribed_processed_pseudogene	3.019667564	0.427812265	0.60398664	-0.708314119	0.478750211	1	0.427812265
ENSMUSG00000069727	Zfp975	434179	protein_coding	54.56090511	-0.427890543	0.173924457	2.460209165	0.013885606	0.054910657	0.427890543
ENSMUSG00000099564	Gm28729	102635744	protein_coding	1.383043982	-0.4278909	2.391412243	0.178928121	0.857994141	1	0.4278909
ENSMUSG00000020811	Wscd1	216881	protein_coding	3.935572492	0.427904967	0.620930011	-0.689135586	0.490737948	1	0.427904967
ENSMUSG00000026914	Psmd14	59029	protein_coding	783.175018	0.427909313	0.075439083	-5.672249672	1.41E-08	2.65E-07	0.427909313
ENSMUSG00000035805	Mlc1	170790	protein_coding	1.013459738	-0.427944429	2.39786038	0.178469286	0.85835444	1	0.427944429
ENSMUSG00000026473	Glul	14645	protein_coding	6370.506637	-0.427968029	0.173724128	2.463492174	0.01375909	0.054501706	0.427968029
ENSMUSG00000045942	BC049762	193286	protein_coding	2.677712198	-0.427971386	0.742624386	0.576295897	0.564415196	1	0.427971386
ENSMUSG00000116976	4930500H12Rik	74991	lncRNA	1.515106644	-0.427974407	2.400990927	0.178249073	0.858527372	1	0.427974407
ENSMUSG00000050808	Muc15	269328	protein_coding	1.196928993	-0.428002642	2.404511493	0.177999832	0.858723108	1	0.428002642
ENSMUSG00000038055	Dexi	58239	protein_coding	157.884108	0.428048998	0.126100357	-3.394510587	0.000687514	0.004614708	0.428048998
ENSMUSG00000058093	Zfp729b	100416706	protein_coding	116.107383	-0.428051879	0.194277208	2.20330467	0.027573282	0.094352503	0.428051879
ENSMUSG00000114961	A930002C04Rik	319467	lncRNA	0.871395148	-0.428080566	2.414007611	0.177331904	0.859247692	1	0.428080566
ENSMUSG00000040536	Necab1	69352	protein_coding	1.715040822	-0.428129123	2.41916303	0.176974068	0.859528759	1	0.428129123
ENSMUSG00000087410	2310065F04Rik	74184	lncRNA	1.485175994	-0.428142042	2.420830284	0.176857521	0.859620306	1	0.428142042
ENSMUSG00000022820	Ndufb4	68194	protein_coding	279.5903331	-0.4281476	0.110670151	3.868681799	0.000109425	0.000932912	0.4281476
ENSMUSG00000075090	Or4a15	258975	protein_coding	1.0263964	-0.42816378	2.423775049	0.176651616	0.859782048	1	0.42816378
ENSMUSG00000064010	Dppa1	347708	protein_coding	1.117073477	-0.428165315	2.42387617	0.17664488	0.859787339	1	0.428165315
ENSMUSG00000032035	Ets1	23871	protein_coding	58.69123534	0.42819345	0.261317699	-1.638593373	0.101297973	0.249900617	0.42819345
ENSMUSG00000074965	Or4k35	258391	protein_coding	1.073640325	-0.428204989	2.428702309	0.176310199	0.86005025	1	0.428204989

Supplementary Table 1

ensembl_gene_id	external_gene_name	entrezgene_id	gene_biotype	baseMean	log2FoldChange	lfcSE	stat	pvalue	padj	absLog2FC
ENSMUSG00000099696	2900052N01Rik	73040	lncRNA	1.580613906	-0.428229571	2.43132314	0.176130258	0.86019161	1	0.428229571
ENSMUSG00000057564	Or4e1	57270	protein_coding	1.703709504	-0.428252262	2.434070474	0.175940782	0.860340466	1	0.428252262
ENSMUSG00000071322	Tcp10a	21461	protein_coding	1.430728211	-0.428252751	2.434244458	0.175928408	0.860350187	1	0.428252751
ENSMUSG00000103567	Pcdhga5	93713	protein_coding	8.765933606	0.428260669	0.383596042	-1.116436621	0.264235285	0.478777869	0.428260669
ENSMUSG00000073971	Or52m1	258322	protein_coding	1.200439207	-0.42827397	2.436996049	0.175738475	0.860499407	1	0.42827397
ENSMUSG00000043331	Or10d5	258825	protein_coding	1.201180071	-0.428274842	2.437101932	0.175731198	0.860505124	1	0.428274842
ENSMUSG00000024306	Ccdc178	70950	protein_coding	2.452871333	-0.428279948	2.077035203	0.206197732	0.836636459	1	0.428279948
ENSMUSG00000025932	Eya1	14048	protein_coding	1.194688356	-0.4284176	2.454632218	0.174534334	0.86144555	1	0.4284176
ENSMUSG00000050334	C130071C03Rik	320203	lncRNA	1.343861907	-0.42850014	2.464949766	0.173837271	0.861993352	1	0.42850014
ENSMUSG00000016386	Mpped2	77015	protein_coding	0.924071318	-0.428553829	2.472131722	0.173353962	0.86237321	1	0.428553829
ENSMUSG00000085336	Gm11732	102637448	lncRNA	0.984131332	-0.428556401	2.472317665	0.173341965	0.86238264	1	0.428556401
ENSMUSG00000067780	Pi15	94227	protein_coding	1.258658764	-0.428599756	2.477603638	0.172989638	0.862659573	1	0.428599756
ENSMUSG00000038760	Trhr	22045	protein_coding	1.295250233	-0.428634943	2.482104673	0.172690116	0.862895014	1	0.428634943
ENSMUSG00000015668	Pdzd11	72621	protein_coding	383.3642497	0.428648805	0.085459225	-5.01582839	5.28E-07	7.76E-06	0.428648805
ENSMUSG00000037033	Clca3b	229927	protein_coding	3844.558357	0.428701723	0.188300123	-2.276693805	0.022804517	0.081360993	0.428701723
ENSMUSG00000115727	4930432J09Rik	73877	lncRNA	1.44618699	-0.428755724	2.497692154	0.171660756	0.86370424	1	0.428755724
ENSMUSG00000063687	Pcdhb5	93876	protein_coding	3.031933483	-0.42875702	0.67002971	0.639907475	0.522232754	1	0.42875702
ENSMUSG00000069804	Gm10277	791303	protein_coding	32.06453063	-0.428995458	0.303425016	1.413843403	0.157407887	0.340684159	0.428995458
ENSMUSG00000025040	Fundc1	72018	protein_coding	256.9252873	0.429016933	0.08759206	-4.897897534	9.69E-07	1.34E-05	0.429016933
ENSMUSG00000045087	S1pr5	94226	protein_coding	1.278137616	-0.429027311	1.834587051	0.233854976	0.815097568	1	0.429027311
ENSMUSG00000064600	Gm25636	115486823	snoRNA	8.03350012	-0.429177642	0.413844441	1.037050639	0.299712258	0.519697021	0.429177642
ENSMUSG00000028459	Cd72	12517	protein_coding	6.847993452	0.429537533	0.603152148	-0.712154527	0.476369092	0.688134988	0.429537533
ENSMUSG00000033685	Ucp2	22228	protein_coding	2733.747347	-0.429541233	0.06947815	6.182393075	6.31E-10	1.42E-08	0.429541233
ENSMUSG00000036553	Sh3tc1	231147	protein_coding	567.6742219	0.42975634	0.144466183	-2.974788491	0.002931906	0.015558323	0.42975634
ENSMUSG00000047843	Bri3	55950	protein_coding	228.2942448	-0.429840708	0.102056171	4.211805147	2.53E-05	0.000254944	0.429840708
ENSMUSG00000029073	Cptp	79554	protein_coding	400.4141575	0.430032689	0.074798244	-5.74923511	8.96E-09	1.74E-07	0.430032689
ENSMUSG00000041219	Arhgap11a	228482	protein_coding	429.601323	0.430087556	0.128259667	-3.353256462	0.000798667	0.005252561	0.430087556
ENSMUSG00000041567	Serpina12	68054	protein_coding	1.158044699	-0.430202891	2.623980958	0.163950462	0.869770141	1	0.430202891
ENSMUSG00000025318	Jph3	57340	protein_coding	5.334700026	0.430262557	0.615855485	-0.698642081	0.484775739	0.695226137	0.430262557
ENSMUSG00000000579	Dynlt1c	100040563	protein_coding	19.2245882	-0.430344196	0.313222424	1.373925246	0.169464891	0.358787591	0.430344196
ENSMUSG00000033863	Klf9	16601	protein_coding	999.2982016	-0.430382996	0.132462776	3.24908634	0.001157763	0.007195824	0.430382996
ENSMUSG00000065728	Gm26175	115490468	snoRNA	5.111862217	0.430388218	0.477563669	-0.901216416	0.367473265	0.590046157	0.430388218
ENSMUSG00000022124	Fbxl3	50789	protein_coding	1137.411194	-0.430407229	0.097510202	4.413971242	1.01E-05	0.000113436	0.430407229
ENSMUSG00000094338	H2bc13	319185	protein_coding	398.5385631	0.430525053	0.204839225	-2.101770567	0.035573379	0.114836207	0.430525053
ENSMUSG00000073900	Or2ag20	257902	protein_coding	1.616966817	-0.430808918	2.816833125	0.152940873	0.878444903	1	0.430808918
ENSMUSG00000022586	Ly6i	57248	protein_coding	0.791931008	0.430878747	1.933419247	-0.222858414	0.823645703	1	0.430878747
ENSMUSG00000040935	Padi6	242726	protein_coding	1.387418677	-0.430905498	2.835192463	0.151984567	0.879199108	1	0.430905498
ENSMUSG00000060303	Or5b97	258698	protein_coding	1.387418677	-0.430905498	2.835192463	0.151984567	0.879199108	1	0.430905498
ENSMUSG00000061584	Lyg2	332427	protein_coding	1.530100513	-0.430943803	2.842269904	0.151619592	0.87948698	1	0.430943803

Supplementary Table 1

ensembl_gene_id	external_gene_name	entrezgene_id	gene_biotype	baseMean	log2FoldChange	lfcSE	stat	pvalue	padj	absLog2FC
ENSMUSG00000035560	Wdr20rt	70948	protein_coding	1.343985524	-0.43094792	2.843308224	0.151565671	0.879529511	1	0.43094792
ENSMUSG00000040133	Gpr176	381413	protein_coding	1.25299176	-0.431021368	2.857547532	0.150836115	0.880104996	1	0.431021368
ENSMUSG00000073209	Klf14	619665	protein_coding	1.25299176	-0.431021368	2.857547532	0.150836115	0.880104996	1	0.431021368
ENSMUSG00000030638	Sh3gl3	20408	protein_coding	1.066876772	-0.431065384	2.866438772	0.150383601	0.880461979	1	0.431065384
ENSMUSG00000050926	Dcaf12l2	245403	protein_coding	1.173979019	-0.431072745	2.867518758	0.150329529	0.880504637	1	0.431072745
ENSMUSG00000052085	Dock8	76088	protein_coding	321.690361	-0.431159217	0.089137987	4.836986248	1.32E-06	1.77E-05	0.431159217
ENSMUSG00000034387	Ssu2	243612	protein_coding	1.016951905	-0.431220179	2.897002335	0.148850477	0.881671619	1	0.431220179
ENSMUSG00000021179	Nrde2	217827	protein_coding	307.5056019	0.431224004	0.102378769	-4.21204522	2.53E-05	0.000254828	0.431224004
ENSMUSG00000010538	Tsacc	76927	protein_coding	12.34153434	-0.431236851	0.28543273	1.510817805	0.130834877	0.299128777	0.431236851
ENSMUSG00000038042	Ptpdc1	218232	protein_coding	8.971125172	0.431246988	0.514009428	-0.838986532	0.401476867	0.62300216	0.431246988
ENSMUSG00000049097	Ankrd34a	545554	protein_coding	1.204794997	0.431293603	1.79916423	-0.239718863	0.810548211	1	0.431293603
ENSMUSG00000075093	Or4c1	258446	protein_coding	0.971755547	-0.431308886	2.915317934	0.147945746	0.882385586	1	0.431308886
ENSMUSG00000009905	Kdsr	70750	protein_coding	350.0899859	-0.4314308	0.125096911	3.448772618	0.000563141	0.003915665	0.4314308
ENSMUSG00000061111	Mcrip1	192173	protein_coding	85.78828509	-0.431594617	0.152832882	2.823964395	0.004743365	0.023285356	0.431594617
ENSMUSG00000072761	Gm6712	100862203	lncRNA	32.81463177	-0.43160334	0.189044061	2.283083313	0.022425461	0.080385081	0.43160334
ENSMUSG00000056215	Lrguk	74354	protein_coding	5.444960781	0.43168399	0.979999046	-0.440494296	0.659579142	0.8161622	0.43168399
ENSMUSG00000026459	Myog	17928	protein_coding	1.357843179	-0.431800025	1.502308173	0.2874244	0.773787381	1	0.431800025
ENSMUSG00000031930	Wwp2	66894	protein_coding	1498.28454	-0.431805071	0.111101598	3.886578391	0.000101667	0.000872625	0.431805071
ENSMUSG00000026443	Lrrn2	16980	protein_coding	0.840252914	0.431950242	1.902827205	-0.22700445	0.820420282	1	0.431950242
ENSMUSG00000014329	Bicc1	83675	protein_coding	9.452400303	-0.43205503	0.460504835	0.9382204	0.348131157	0.570058297	0.43205503
ENSMUSG00000035202	Lars2	102436	protein_coding	11475.44353	-0.432136069	0.563011242	0.767544299	0.442757965	0.660497294	0.432136069
ENSMUSG00000031886	Ces2e	234673	protein_coding	2622.98925	-0.432214419	0.132957883	3.250761891	0.001150962	0.007156245	0.432214419
ENSMUSG00000118292	Gm10549	433171	lncRNA	3.413631735	-0.432515945	0.6577194	0.657599494	0.51079552	1	0.432515945
ENSMUSG00000020519	Sap30l	50724	protein_coding	356.9772235	0.432530532	0.134854993	-3.207374998	0.001339523	0.008117753	0.432530532
ENSMUSG00000085440	Sorbs2os	319940	lncRNA	6.670762508	0.432574454	0.490195349	-0.882453199	0.377531778	0.599649878	0.432574454
ENSMUSG00000019467	Arhgef25	52666	protein_coding	3.310672309	0.432704668	1.114051032	-0.388406506	0.697715231	1	0.432704668
ENSMUSG00000078253	Krtap16-1	100504183	protein_coding	2.227888009	0.432831223	2.211732655	-0.195697804	0.844846704	1	0.432831223
ENSMUSG00000019986	Ahi1	52906	protein_coding	22.2274704	0.432934872	0.289768905	-1.494069461	0.135157465	0.30625554	0.432934872
ENSMUSG00000035783	Acta2	11475	protein_coding	2.37141596	-0.43305208	0.811992069	0.533320578	0.593811685	1	0.43305208
ENSMUSG00000016756	Cmah	12763	protein_coding	608.2699785	-0.433267392	0.252111382	1.718555463	0.085695347	0.220951697	0.433267392
ENSMUSG00000058101	1700084J12Rik	73486	transcribed_processed_pseudogene	2.911161007	0.43329874	0.663160237	-0.653384681	0.513508312	1	0.43329874
ENSMUSG00000015656	Hspa8	15481	protein_coding	14797.55801	-0.433498722	0.136699526	3.171179403	0.001518213	0.0089816	0.433498722
ENSMUSG00000038393	Txnip	56338	protein_coding	9188.776409	-0.43362476	0.110952658	3.908196226	9.30E-05	0.000809062	0.43362476
ENSMUSG00000024729	Ms4a20	69369	protein_coding	1.967640148	-0.43374342	1.708121792	0.253930031	0.799549613	1	0.43374342
ENSMUSG00000036840	Siah1a	20437	protein_coding	153.7875753	-0.433743598	0.102148392	4.24621072	2.17E-05	0.000222179	0.433743598
ENSMUSG00000033170	Card10	105844	protein_coding	420.8433466	-0.433993238	0.076626476	5.66375043	1.48E-08	2.77E-07	0.433993238
ENSMUSG00000006445	Epha2	13836	protein_coding	738.358647	0.434031298	0.282321519	-1.537365269	0.124203886	0.288643819	0.434031298
ENSMUSG00000096606	Tpbgl	100503386	protein_coding	3.205554922	-0.434164969	0.671061847	0.646982049	0.517643562	1	0.434164969
ENSMUSG00000035325	Sec31a	69162	protein_coding	2489.776038	0.434269707	0.050256479	-8.641069117	5.57E-18	2.57E-16	0.434269707

ensembl_gene_id	external_gene_name	entrezgene_id	gene_biotype	baseMean	log2FoldChange	lfcSE	stat	pvalue	padj	absLog2FC
ENSMUSG00000028933	Xrcc2	57434	protein_coding	40.86995964	0.434435715	0.241653861	-1.797760292	0.072215002	0.195501402	0.434435715
ENSMUSG00000038759	Nup205	70699	protein_coding	702.0150011	0.434540331	0.121096983	-3.588366293	0.000332757	0.00249439	0.434540331
ENSMUSG00000043913	Ccdc60	269693	protein_coding	3.338921294	0.434549439	1.971247295	-0.220443899	0.82552546	1	0.434549439
ENSMUSG00000036500	Akp3	11648	protein_coding	4.015933001	0.434551839	1.330754564	-0.326545443	0.744011713	1	0.434551839
ENSMUSG00000032714	Syde1	71709	protein_coding	10.83176034	0.434587946	0.402958339	-1.078493492	0.280813585	0.498479581	0.434587946
ENSMUSG00000113918	Gm6566	625237	lncRNA	6.227928959	-0.434599514	0.471798395	0.921155136	0.356969438	0.579930477	0.434599514
ENSMUSG00000038763	Alpk3	116904	protein_coding	4.048740204	0.434669836	1.311641531	-0.331393773	0.740347068	1	0.434669836
ENSMUSG00000093176	Mir5119	100628613	miRNA	0.63535501	0.43472486	1.623565259	-0.2677594	0.788884524	1	0.43472486
ENSMUSG00000038845	Phb	18673	protein_coding	662.893273	-0.434729246	0.117277171	3.706853107	0.000209851	0.001648655	0.434729246
ENSMUSG00000049670	Morn4	226123	protein_coding	2.968266116	-0.434804829	0.6251333	0.695539382	0.48671734	1	0.434804829
ENSMUSG00000096299	Gm21814	436100	lncRNA	8.893010522	-0.434812763	0.432295132	1.00582387	0.314500303	0.535752227	0.434812763
ENSMUSG00000062464	Cyp4f37	677156	protein_coding	1.919440296	-0.434832899	1.412725199	0.307797227	0.758236631	1	0.434832899
ENSMUSG00000034990	Otoa	246190	protein_coding	3.970591941	0.434836182	0.953111106	-0.456228218	0.648225876	1	0.434836182
ENSMUSG00000049314	Fbxw13	211305	protein_coding	1.485909581	-0.434890619	1.718351247	0.253085986	0.800201768	1	0.434890619
ENSMUSG00000006800	Sulf2	72043	protein_coding	285.7922779	0.43490427	0.191443313	-2.271713036	0.023103848	0.082121599	0.43490427
ENSMUSG00000024669	Cd5	12507	protein_coding	4.4947527	0.434960568	0.757904145	-0.57389918	0.566036028	0.752646336	0.434960568
ENSMUSG00000111422	1700027J07Rik	69443	lncRNA	1.70197375	0.435032375	1.799182593	-0.241794455	0.808939436	1	0.435032375
ENSMUSG00000098134	Rnf113a2	66381	protein_coding	152.886509	0.43508864	0.114434352	-3.802080687	0.000143486	0.001184764	0.43508864
ENSMUSG00000060508	Nlrp9b	243874	protein_coding	192.4738956	0.435134402	0.37166245	-1.170778491	0.241687826	0.452315589	0.435134402
ENSMUSG00000015962	1700016C15Rik	69428	protein_coding	1.380578869	-0.435212109	1.017793982	0.427603343	0.668939932	1	0.435212109
ENSMUSG00000035637	Grhpr	76238	protein_coding	283.1358386	0.435256048	0.102377062	-4.251499695	2.12E-05	0.000217399	0.435256048
ENSMUSG00000092009	Myh15	667772	protein_coding	1.841525791	0.435301111	2.134261025	-0.203958703	0.838385771	1	0.435301111
ENSMUSG00000050627	Gpd1l	333433	protein_coding	2079.998984	0.435313399	0.063196944	-6.888203371	5.65E-12	1.61E-10	0.435313399
ENSMUSG00000031910	Has3	15118	protein_coding	13.89680433	-0.43553238	0.379711967	1.147007251	0.251378624	0.463828671	0.43553238
ENSMUSG00000032014	Oaf	102644	protein_coding	173.248456	-0.43557327	0.144320757	3.018091641	0.00254372	0.013831738	0.43557327
ENSMUSG00000047798	Cd300lf	246746	protein_coding	32.18037812	-0.435617234	0.345206928	1.261901772	0.206984134	0.409799304	0.435617234
ENSMUSG00000037053	Azgp1	12007	protein_coding	53.43271107	-0.435776376	0.222591111	1.957743839	0.050260078	0.14941403	0.435776376
ENSMUSG00000009575	Cbx5	12419	protein_coding	1496.984328	0.435816628	0.149086248	-2.9232517	0.003463964	0.017949136	0.435816628
ENSMUSG00000002274	Metrn	70083	protein_coding	27.78141983	0.435866018	0.259649131	-1.678673124	0.093215767	0.23455206	0.435866018
ENSMUSG00000031812	Map1lc3b	67443	protein_coding	994.8523245	-0.435938881	0.083256852	5.236072136	1.64E-07	2.61E-06	0.435938881
ENSMUSG00000040478	Prdm13	230025	protein_coding	1.413184018	0.435947839	1.879743994	-0.23191873	0.816601137	1	0.435947839
ENSMUSG00000071658	Gng3	14704	protein_coding	3.229612148	0.435993152	0.747085521	-0.583592025	0.559494827	1	0.435993152
ENSMUSG00000027340	Slc23a2	54338	protein_coding	801.0425191	-0.43599524	0.103508569	4.21216566	2.53E-05	0.000254828	0.43599524
ENSMUSG00000064360	mt-Nd3	17718	protein_coding	4.026843207	0.436025201	0.680983224	-0.640287727	0.521985558	1	0.436025201
ENSMUSG00000024953	Prdx5	54683	protein_coding	1732.303799	-0.436094008	0.078502753	5.555142834	2.77E-08	4.98E-07	0.436094008
ENSMUSG00000023861	Mpc1	55951	protein_coding	434.7616214	-0.436137889	0.095262088	4.578294435	4.69E-06	5.63E-05	0.436137889
ENSMUSG00000013155	Enkd1	102124	protein_coding	27.1461322	0.436191956	0.234903666	-1.856897184	0.063325783	0.177458338	0.436191956
ENSMUSG00000044461	Shisa2	219134	protein_coding	610.8043837	-0.436254972	0.120226475	3.628609862	0.000284951	0.002177552	0.436254972
ENSMUSG00000055945	Prr18	320111	protein_coding	4.268634207	-0.436286151	0.70953394	0.614891164	0.538626598	1	0.436286151

ensembl_gene_id	external_gene_name	entrezgene_id	gene_biotype	baseMean	log2FoldChange	lfcSE	stat	pvalue	padj	absLog2FC
ENSMUSG00000070305	Mpzl3	319742	protein_coding	357.2052248	-0.436384536	0.140839161	3.098460206	0.001945291	0.011092726	0.436384536
ENSMUSG00000030161	Gabarapl1	57436	protein_coding	255.7253235	-0.436466443	0.106863653	4.084330193	4.42E-05	0.000420016	0.436466443
ENSMUSG00000028186	Uox	22262	protein_coding	19.31647302	0.436497435	0.228130313	-1.91336885	0.055700849	0.161546158	0.436497435
ENSMUSG00000060212	Pcnx2	270109	protein_coding	5.294769774	0.436674395	0.796622211	-0.548157444	0.5835838	0.766020418	0.436674395
ENSMUSG00000037020	Wdr62	233064	protein_coding	79.06598951	0.436692213	0.19410204	-2.249807431	0.024461172	0.085928908	0.436692213
ENSMUSG00000086468	Etaa1os	102633673	lncRNA	6.248461861	-0.437075845	0.446326264	0.979274313	0.327444459	0.549590181	0.437075845
ENSMUSG00000002984	Tomm40	53333	protein_coding	811.1435228	0.437179665	0.091219634	-4.792604889	1.65E-06	2.17E-05	0.437179665
ENSMUSG00000039298	Cdk5rap2	214444	protein_coding	316.7777503	0.437215006	0.094078302	-4.647352241	3.36E-06	4.16E-05	0.437215006
ENSMUSG00000035435	Abca17	381072	protein_coding	3.488875326	0.437312016	1.727339182	-0.2531709	0.800136152	1	0.437312016
ENSMUSG00000073755	5730409E04Rik	230757	protein_coding	8.76238218	-0.437312687	0.422939338	1.033984422	0.301143446	0.521287512	0.437312687
ENSMUSG00000097615	Gm2061	100039138	lncRNA	113.1899271	0.437466598	0.259603616	-1.685132913	0.091962931	0.232355545	0.437466598
ENSMUSG00000030894	Tpp1	12751	protein_coding	337.2842054	-0.437643545	0.06663193	6.568075457	5.10E-11	1.34E-09	0.437643545
ENSMUSG00000089960	Ugt1a1	394436	protein_coding	285.5276743	-0.437672443	0.188781348	2.318409355	0.020427084	0.07460396	0.437672443
ENSMUSG00000078496	Zfp982	195531	protein_coding	3.38353899	-0.437790437	0.745000556	0.587637732	0.556775479	1	0.437790437
ENSMUSG00000066621	Tecpr1	70381	protein_coding	865.4918131	0.437856356	0.057842989	-7.569739483	3.74E-14	1.29E-12	0.437856356
ENSMUSG00000019303	Psmc3ip	19183	protein_coding	21.58298918	0.437865057	0.24938446	-1.755783248	0.079125426	0.208467978	0.437865057
ENSMUSG00000025991	Cps1	227231	protein_coding	338.1852598	0.437874025	0.198474906	-2.20619339	0.027370462	0.093755613	0.437874025
ENSMUSG00000030173	Klra5	16636	protein_coding	5.718169354	-0.437938073	0.624446932	0.701321523	0.483102385	0.693738201	0.437938073
ENSMUSG00000083012	Fam220a	67238	protein_coding	5.207960021	0.438174238	0.507103159	-0.864073179	0.387547692	0.61004314	0.438174238
ENSMUSG00000056222	Spock1	20745	protein_coding	4.680521767	-0.438238364	0.832422168	0.526461669	0.598567478	0.776186935	0.438238364
ENSMUSG00000031627	Irf2	16363	protein_coding	289.3605141	-0.438265742	0.149676324	2.928089965	0.003410513	0.017711075	0.438265742
ENSMUSG00000030060	Hmces	232210	protein_coding	112.9710063	-0.438433935	0.12174911	3.601126416	0.000316841	0.002388097	0.438433935
ENSMUSG00000075318	Scn2a	110876	protein_coding	3.251113992	-0.438456361	2.442427295	0.179516648	0.857532047	1	0.438456361
ENSMUSG00000017007	Rbpjl	19668	protein_coding	3.570849102	0.438554823	0.99317598	-0.441568093	0.658801777	1	0.438554823
ENSMUSG00000053769	Lysmd1	217779	protein_coding	17.57136594	-0.43875895	0.246934016	1.776826691	0.075596759	0.201550674	0.43875895
ENSMUSG00000005124	Ccn4	22402	protein_coding	2.07521022	0.438863489	1.564212618	-0.280565112	0.779043978	1	0.438863489
ENSMUSG00000057103	Nat8f1	66116	protein_coding	14.9047493	-0.438998381	0.322660175	1.360559543	0.173652924	0.364427834	0.438998381
ENSMUSG00000000706	Btn1a1	12231	protein_coding	64.98481613	0.439057655	0.193261291	-2.271834425	0.023096513	0.0821132	0.439057655
ENSMUSG00000017453	Pipox	19193	protein_coding	56.69456944	0.439060457	0.184522758	-2.379437973	0.017339061	0.065598201	0.439060457
ENSMUSG00000092819	Gm23639	115489002	snoRNA	70.69377676	0.439076428	0.297948351	-1.473666249	0.140571474	0.314808425	0.439076428
ENSMUSG00000017832	Hspb9	75482	protein_coding	1.614354407	0.439175598	0.861465125	-0.509800786	0.610191035	1	0.439175598
ENSMUSG00000023931	Efhb	211482	protein_coding	18.16969602	-0.439327912	0.289669768	1.516650893	0.129354849	0.297019741	0.439327912
ENSMUSG00000026388	3110009E18Rik	73103	protein_coding	11.91757192	-0.439583656	0.301298722	1.458962897	0.14457532	0.321507788	0.439583656
ENSMUSG00000024143	Rhoq	104215	protein_coding	399.7375403	0.439630616	0.122468651	-3.589739999	0.000331008	0.002484666	0.439630616
ENSMUSG00000053192	Mllt11	56772	protein_coding	69.20473161	-0.439631178	0.153456726	2.864854412	0.00417201	0.020879192	0.439631178
ENSMUSG00000044724	Gpr152	269053	protein_coding	1.913782085	0.439992976	1.21291006	-0.362758122	0.716785578	1	0.439992976
ENSMUSG00000028702	Rad54l	19366	protein_coding	84.62725531	0.440134448	0.215172015	-2.045500421	0.040805548	0.127940538	0.440134448
ENSMUSG00000027984	Hadh	15107	protein_coding	3226.117383	-0.440178053	0.091731581	4.798544293	1.60E-06	2.11E-05	0.440178053
ENSMUSG00000032353	Tmed3	66111	protein_coding	1299.249991	0.440179002	0.170020115	-2.588981909	0.009626015	0.041288574	0.440179002

ensembl_gene_id	external_gene_name	entrezgene_id	gene_biotype	baseMean	log2FoldChange	lfcSE	stat	pvalue	padj	absLog2FC
ENSMUSG00000026748	Plxdc2	67448	protein_coding	1.330671848	0.440264514	2.128354374	-0.206856771	0.836121718	1	0.440264514
ENSMUSG00000070495	Ctcfl	664799	protein_coding	11.14434052	-0.440272828	0.360911906	1.219890007	0.222506575	0.429533455	0.440272828
ENSMUSG00000060791	Gmfg	63986	protein_coding	179.9138322	0.440410638	0.11158623	-3.946818849	7.92E-05	0.000703145	0.440410638
ENSMUSG00000026825	Dnm1	13429	protein_coding	47.49953848	0.440519662	0.366054853	-1.203425273	0.228811793	0.437741278	0.440519662
ENSMUSG00000042010	Acacb	100705	protein_coding	418.6456114	0.440976235	0.207942412	-2.120665187	0.033949988	0.110778523	0.440976235
ENSMUSG00000028211	Trp53inp1	60599	protein_coding	782.0176475	-0.441026455	0.146209856	3.01639347	0.002558011	0.013886585	0.441026455
ENSMUSG00000031451	Gas6	14456	protein_coding	4193.708382	-0.441076375	0.117964695	3.739054097	0.000184714	0.0014822	0.441076375
ENSMUSG00000031150	Ccdc120	54648	protein_coding	136.7964492	-0.441077938	0.187099231	2.357454578	0.018400706	0.068717608	0.441077938
ENSMUSG00000070081	Mir505	751545	miRNA	0.507643072	-0.441096104	1.491859967	0.295668571	0.767483201	1	0.441096104
ENSMUSG00000028521	Slc35d1	242585	protein_coding	503.6798964	-0.441140831	0.226348543	1.948944863	0.05130201	0.152100788	0.441140831
ENSMUSG00000065653	Gm23105	115487521	snoRNA	138.5365793	0.441162266	0.25358685	-1.739689048	0.081913633	0.213539052	0.441162266
ENSMUSG00000022265	Ank	11732	protein_coding	835.0839124	-0.441193401	0.099220599	4.446590773	8.72E-06	9.92E-05	0.441193401
ENSMUSG00000038323	1700066M21Rik	73467	protein_coding	94.34043391	0.441218886	0.199824006	-2.208037435	0.027241664	0.093415051	0.441218886
ENSMUSG00000027555	Car13	71934	protein_coding	541.9713108	0.441239847	0.080026914	-5.513643185	3.51E-08	6.19E-07	0.441239847
ENSMUSG00000049551	Fzd9	14371	protein_coding	26.4801668	-0.441241007	0.260937219	1.69098532	0.090839606	0.230331489	0.441241007
ENSMUSG00000000340	Dbt	13171	protein_coding	396.8491352	-0.441389494	0.115535458	3.820381206	0.000133246	0.0011102	0.441389494
ENSMUSG00000079084	Ccdc82	66396	protein_coding	146.1379082	-0.441393526	0.130739204	3.376137467	0.000735112	0.004881237	0.441393526
ENSMUSG00000007591	Tssk4	71099	protein_coding	21.97588492	0.441470647	0.249425015	-1.769953372	0.076734909	0.204058016	0.441470647
ENSMUSG00000038836	Agbl3	76223	protein_coding	15.74774053	0.441682726	0.332498874	-1.328373598	0.184054717	0.379085971	0.441682726
ENSMUSG00000033544	Angptl1	72713	protein_coding	7.539300965	0.441696484	0.453450058	-0.974079672	0.330016982	0.552168231	0.441696484
ENSMUSG00000056073	Grik2	14806	protein_coding	3.108925784	-0.441698759	2.400522119	0.18400112	0.854012576	1	0.441698759
ENSMUSG00000008668	Rps18	20084	protein_coding	5419.960139	-0.441755199	0.118829387	3.717558516	0.000201157	0.001589432	0.441755199
ENSMUSG00000022900	Ildr1	106347	protein_coding	3769.495805	0.441977427	0.103217514	-4.282000328	1.85E-05	0.000192981	0.441977427
ENSMUSG00000102047	2010010A06Rik	100503117	transcribed_processed_pseudogene	18.57494253	-0.441984319	0.485773084	0.909857573	0.362897627	0.585225632	0.441984319
ENSMUSG00000045775	Slc16a5	217316	protein_coding	1424.148791	0.442114644	0.182758047	-2.419125459	0.015557873	0.060127011	0.442114644
ENSMUSG00000010044	Zmynd10	114602	protein_coding	3.300740004	0.44218702	0.954294101	-0.46336556	0.643102361	1	0.44218702
ENSMUSG00000054414	Slc30a7	66500	protein_coding	930.3959601	0.442439938	0.102665212	-4.309540969	1.64E-05	0.000172846	0.442439938
ENSMUSG00000039058	Ak5	229949	protein_coding	3.849746196	-0.442447584	0.740218428	0.597725707	0.550022969	1	0.442447584
ENSMUSG00000040710	St8sia4	20452	protein_coding	14.39143101	0.442553511	0.600320585	-0.737195296	0.461003594	0.674995696	0.442553511
ENSMUSG00000049751	Rpl36al	66483	protein_coding	241.3721747	0.442724948	0.167496693	-2.643186196	0.008212986	0.036352985	0.442724948
ENSMUSG00000067235	H2-Q10	15007	protein_coding	50.19584864	-0.442751685	0.222238756	1.992234353	0.046345351	0.140818237	0.442751685
ENSMUSG00000052821	Cysltr1	58861	protein_coding	4.094267078	0.442780331	0.808485926	-0.547666096	0.583921196	1	0.442780331
ENSMUSG00000023935	Spats1	71020	protein_coding	7.424574327	0.44279073	0.399080864	-1.109526341	0.267203188	0.482454733	0.44279073
ENSMUSG00000045128	Rpl18a	76808	protein_coding	4357.431313	-0.442819122	0.125361688	3.532332161	0.000411912	0.003009871	0.442819122
ENSMUSG00000022783	Spag6l	50525	protein_coding	3.941934199	-0.442895326	0.655219782	0.675949258	0.499072862	1	0.442895326
ENSMUSG00000027634	Ndrg3	29812	protein_coding	621.5140633	-0.442987737	0.092051589	4.812385559	1.49E-06	1.98E-05	0.442987737
ENSMUSG00000021095	Gsc	14836	protein_coding	0.733220376	0.442989588	1.563733826	-0.283289637	0.776954821	1	0.442989588
ENSMUSG00000040813	Tex264	21767	protein_coding	602.1598606	-0.443043409	0.064680794	6.849690319	7.40E-12	2.09E-10	0.443043409
ENSMUSG00000027358	Bmp2	12156	protein_coding	291.5161615	-0.44310701	0.239584812	1.849478713	0.064388719	0.179735514	0.44310701

ensembl_gene_id	external_gene_name	entrezgene_id	gene_biotype	baseMean	log2FoldChange	lfcSE	stat	pvalue	padj	absLog2FC
ENSMUSG00000028778	Hcrtr1	230777	protein_coding	2.785054448	0.443131966	0.876812312	-0.505389762	0.613285108	1	0.443131966
ENSMUSG00000022475	Hdac7	56233	protein_coding	83.55447829	0.44317491	0.179430888	-2.469891975	0.013515386	0.053833353	0.44317491
ENSMUSG00000024462	Gabbr1	54393	protein_coding	82.78885852	0.443238017	0.22071991	-2.008146961	0.044627677	0.136655194	0.443238017
ENSMUSG00000059991	Nptx2	53324	protein_coding	13.61438182	0.443274809	0.434132844	-1.021057988	0.307226966	0.527698391	0.443274809
ENSMUSG00000112280	A830082N09Rik	414093	lncRNA	5.301018894	0.443357974	0.582161879	-0.761571636	0.446315709	0.663584952	0.443357974
ENSMUSG00000032062	2310030G06Rik	66952	protein_coding	209.0092851	0.44338908	0.098652938	-4.49443362	6.98E-06	8.11E-05	0.44338908
ENSMUSG00000017404	Rpl19	19921	protein_coding	4196.104562	-0.443413852	0.128105608	3.461314907	0.000537544	0.003771026	0.443413852
ENSMUSG00000070544	Top1	21969	protein_coding	1458.307412	0.443452829	0.059058362	-7.50872205	5.97E-14	2.01E-12	0.443452829
ENSMUSG00000063445	Nmral1	67824	protein_coding	75.90380393	0.443454409	0.190133355	-2.332333582	0.019683149	0.07241317	0.443454409
ENSMUSG00000055421	Pcdh9	211712	protein_coding	1.523347713	0.443608746	2.37420918	-0.186844845	0.851782285	1	0.443608746
ENSMUSG00000044628	Rnf208	68846	protein_coding	8.11183613	-0.443641531	0.515058537	0.861341963	0.389049731	0.611707822	0.443641531
ENSMUSG00000095562	Gm55594	170942	lncRNA	29.69464996	-0.443715301	0.874819145	0.507208037	0.612008856	0.785292483	0.443715301
ENSMUSG00000030775	Trat1	77647	protein_coding	4.158100137	-0.444044077	0.814595254	0.545110071	0.585677816	1	0.444044077
ENSMUSG00000045912	C2cd4c	237397	protein_coding	29.14695195	-0.444098878	0.223834838	1.984047172	0.047250581	0.142911457	0.444098878
ENSMUSG00000063873	Slc24a3	94249	protein_coding	41.99309544	-0.444154527	0.226805831	1.958302946	0.050194475	0.149272747	0.444154527
ENSMUSG00000006456	Rbm14	56275	protein_coding	494.4200081	0.444162972	0.066475768	-6.6815771	2.36E-11	6.36E-10	0.444162972
ENSMUSG00000049804	Armcx4	100503043	protein_coding	12.02141982	0.444340431	0.532326883	-0.834713491	0.403879044	0.625085148	0.444340431
ENSMUSG00000031391	L1cam	16728	protein_coding	1325.043731	-0.444510066	0.093620934	4.747977262	2.05E-06	2.67E-05	0.444510066
ENSMUSG00000042942	Greb1l	381157	protein_coding	103.9857606	-0.444671902	0.139473255	3.188223443	0.001431499	0.008568238	0.444671902
ENSMUSG00000031538	Plat	18791	protein_coding	10.20670937	0.444688975	0.639750493	-0.69509751	0.486994195	0.695624496	0.444688975
ENSMUSG00000028820	Sfpq	71514	protein_coding	2146.021868	0.445087799	0.062204855	-7.155193876	8.36E-13	2.56E-11	0.445087799
ENSMUSG00000029464	Gpn3	68080	protein_coding	199.6008751	0.445115208	0.113765429	-3.912570038	9.13E-05	0.000796221	0.445115208
ENSMUSG00000091813	Ces2h	436059	protein_coding	3.747487908	0.445177391	0.690452576	-0.64476172	0.519081613	1	0.445177391
ENSMUSG00000099146	0610031O16Rik	68369	lncRNA	2.883680877	0.445215205	0.846881922	-0.525711074	0.599088968	1	0.445215205
ENSMUSG00000030298	Sec13	110379	protein_coding	1710.738724	0.44522746	0.070077479	-6.353360135	2.11E-10	5.06E-09	0.44522746
ENSMUSG00000110062	B430319F04Rik	619306	TEC	20.31040183	0.445266052	0.28928253	-1.53920823	0.123753469	0.287840111	0.445266052
ENSMUSG00000020838	Slc6a4	15567	protein_coding	157.7763391	-0.445366262	0.311801098	1.428366562	0.153186378	0.334228588	0.445366262
ENSMUSG00000024992	Pde6c	110855	protein_coding	1.60711077	-0.445516611	1.564678537	0.284733637	0.775848218	1	0.445516611
ENSMUSG00000050910	Cdr2l	237988	protein_coding	119.5561089	0.445594217	0.181362677	-2.456923468	0.014013254	0.055305475	0.445594217
ENSMUSG00000085287	4833418N02Rik	74597	lncRNA	51.11624493	0.445836499	0.227943841	-1.955905001	0.050476346	0.14994898	0.445836499
ENSMUSG00000028066	Pmf1	67037	protein_coding	216.2148066	0.445849232	0.101891739	-4.375715196	1.21E-05	0.000132912	0.445849232
ENSMUSG00000028688	Toe1	68276	protein_coding	165.311386	0.445860824	0.105582605	-4.222862512	2.41E-05	0.000244236	0.445860824
ENSMUSG00000058145	Adamts17	233332	protein_coding	70.90786864	0.445894914	0.179188384	-2.488414167	0.012831421	0.051654946	0.445894914
ENSMUSG00000035828	Pim3	223775	protein_coding	1138.718354	-0.445952152	0.130779458	3.409955654	0.000649734	0.004413704	0.445952152
ENSMUSG00000028018	Gstcd	67553	protein_coding	134.8455573	0.44597737	0.146585819	-3.042431886	0.002346749	0.012896578	0.44597737
ENSMUSG00000029209	Gnpda2	67980	protein_coding	61.99082544	-0.446021702	0.144624574	3.083996663	0.002042399	0.01154679	0.446021702
ENSMUSG00000104667	Gm4961	242914	lncRNA	6.723290657	0.446026964	0.526367663	-0.847367716	0.396790193	0.618855346	0.446026964
ENSMUSG00000105496	Mir7004	102466784	miRNA	0.710300434	-0.446045312	1.184992124	0.376412048	0.70661059	1	0.446045312
ENSMUSG00000034111	Tmed8	382620	protein_coding	71.76214383	-0.446089143	0.154760981	2.882439353	0.003946091	0.019983363	0.446089143

ensembl_gene_id	external_gene_name	entrezgene_id	gene_biotype	baseMean	log2FoldChange	lfcSE	stat	pvalue	padj	absLog2FC
ENSMUSG00000116396	Gm20556	328576	lncRNA	1.743750709	-0.446138843	0.948192549	0.470515027	0.637987101	1	0.446138843
ENSMUSG00000061838	Suclg2	20917	protein_coding	2478.300946	-0.44636377	0.104558569	4.269030965	1.96E-05	0.000202378	0.44636377
ENSMUSG00000020834	Dhrs13	70451	protein_coding	63.80426074	-0.4464199	0.176448125	2.530034824	0.011405121	0.047310767	0.4464199
ENSMUSG00000019975	Ikbip	67454	protein_coding	168.2562871	0.446698242	0.129359894	-3.453143222	0.000554095	0.003857639	0.446698242
ENSMUSG00000029189	Sel1l3	231238	protein_coding	2624.965694	0.446737562	0.081715918	-5.466958867	4.58E-08	7.90E-07	0.446737562
ENSMUSG00000059409	Ppp2r5d	21770	protein_coding	838.4114419	0.446748508	0.072817242	-6.135202236	8.51E-10	1.89E-08	0.446748508
ENSMUSG00000064065	Ipcef1	320495	protein_coding	15.15853716	0.447123242	0.352874744	-1.267087684	0.205123939	0.407579707	0.447123242
ENSMUSG00000018865	Sult4a1	29859	protein_coding	9.336353813	0.447445005	0.442221385	-1.01181223	0.311627841	0.532495959	0.447445005
ENSMUSG00000026787	Gad2	14417	protein_coding	25.80302935	-0.447506512	0.27156914	1.647854801	0.09938247	0.246352764	0.447506512
ENSMUSG00000024691	Fam111a	107373	protein_coding	143.2875634	0.447590662	0.251582861	-1.779098385	0.075223633	0.200815241	0.447590662
ENSMUSG00000024411	Aqp4	11829	protein_coding	14478.62701	0.447625109	0.190695821	-2.347325217	0.018908742	0.07028111	0.447625109
ENSMUSG00000054942	Miga1	215708	protein_coding	125.807203	-0.447827378	0.109682022	4.082960623	4.45E-05	0.000421772	0.447827378
ENSMUSG00000046808	Atp10d	231287	protein_coding	9.185001778	-0.4478678	0.463515	0.966242302	0.33392295	0.556279996	0.4478678
ENSMUSG00000042155	Klhl23	277396	protein_coding	54.73108486	0.44787949	0.254513182	-1.759749679	0.078450258	0.207184962	0.44787949
ENSMUSG00000023926	Rhag	19743	protein_coding	0.947043583	0.448018642	1.114554425	-0.401971077	0.687705312	1	0.448018642
ENSMUSG00000074646	6430550D23Rik	320095	protein_coding	12.34203345	-0.448086357	0.430162653	1.041667272	0.297565968	0.51726362	0.448086357
ENSMUSG00000038047	Haus6	230376	protein_coding	141.6992519	0.448176509	0.14513634	-3.087968934	0.002015295	0.011401366	0.448176509
ENSMUSG00000022451	Twf1	19230	protein_coding	1843.717767	0.448198056	0.072213454	-6.206572744	5.42E-10	1.23E-08	0.448198056
ENSMUSG00000030978	Rrm1	20133	protein_coding	673.3457049	0.448203667	0.191504613	-2.340432742	0.019261407	0.071287064	0.448203667
ENSMUSG00000000394	Gcg	14526	protein_coding	415.3484431	-0.448330828	0.131236124	3.416215096	0.000634981	0.004327722	0.448330828
ENSMUSG00000014164	Klhl3	100503085	protein_coding	26.06305303	-0.448341885	0.251666107	1.781494899	0.074831637	0.19999509	0.448341885
ENSMUSG00000034708	Grn	14824	protein_coding	871.9073227	-0.448419247	0.061425098	7.300260974	2.87E-13	9.10E-12	0.448419247
ENSMUSG00000035000	Dpp4	13482	protein_coding	758.5165014	0.448495547	0.101393308	-4.423324948	9.72E-06	0.000109485	0.448495547
ENSMUSG00000050623	Catsperz	67077	protein_coding	5.305472626	0.448574867	0.44054907	-1.018217713	0.308574504	0.529213766	0.448574867
ENSMUSG00000049115	Agtr1a	11607	protein_coding	4.486803755	0.448584026	0.624800589	-0.717963513	0.472779785	0.685206817	0.448584026
ENSMUSG00000003545	Fosb	14282	protein_coding	5081.997123	-0.448713739	0.117286808	3.82578184	0.000130358	0.001090541	0.448713739
ENSMUSG00000021390	Ogn	18295	protein_coding	4.382227197	0.448714766	0.527533824	-0.850589565	0.394997388	1	0.448714766
ENSMUSG00000029217	Tec	21682	protein_coding	168.2900672	-0.448841359	0.100872712	4.449581547	8.60E-06	9.80E-05	0.448841359
ENSMUSG00000021118	Plek2	27260	protein_coding	406.2310013	-0.448957168	0.092625318	4.847024334	1.25E-06	1.69E-05	0.448957168
ENSMUSG00000012609	Ttll5	320244	protein_coding	248.2222762	0.449024949	0.084451559	-5.316952728	1.06E-07	1.74E-06	0.449024949
ENSMUSG00000036782	Klhl13	67455	protein_coding	36.68910002	0.449052102	0.283476292	-1.584090502	0.113173125	0.270127107	0.449052102
ENSMUSG00000012076	Brms1l	52592	protein_coding	83.69881485	0.449066429	0.180541502	-2.487330743	0.012870568	0.051764384	0.449066429
ENSMUSG00000043895	S1pr2	14739	protein_coding	27.16675731	0.449113823	0.206535542	-2.174511078	0.029666774	0.10006477	0.449113823
ENSMUSG00000030493	Faap24	101831	protein_coding	83.12339132	0.449223967	0.128538453	-3.494860538	0.00047431	0.003393738	0.449223967
ENSMUSG00000055197	Fev	260298	protein_coding	3.814250231	-0.44954904	0.719535603	0.624776645	0.532117661	1	0.44954904
ENSMUSG00000055240	Zfp101	22643	protein_coding	121.6451827	0.449612786	0.165410746	-2.71815947	0.006564619	0.03021408	0.449612786
ENSMUSG00000091648	C2cd4d	271944	protein_coding	4.150391142	0.449623403	0.611000114	-0.735881047	0.461803092	1	0.449623403
ENSMUSG00000018068	Ints2	70422	protein_coding	325.6084509	0.449655412	0.121819539	-3.691160011	0.000223234	0.001743841	0.449655412
ENSMUSG00000040964	Arhgef10l	72754	protein_coding	799.219105	0.449780034	0.066299006	-6.784114296	1.17E-11	3.25E-10	0.449780034

ensembl_gene_id	external_gene_name	entrezgene_id	gene_biotype	baseMean	log2FoldChange	lfcSE	stat	pvalue	padj	absLog2FC
ENSMUSG00000079427	Mthfsl	100039707	protein_coding	55.76990498	-0.449799404	0.145608533	3.089100578	0.002007635	0.011373612	0.449799404
ENSMUSG00000022780	Meltf	30060	protein_coding	1.886135024	-0.449908038	1.199274405	0.375150205	0.707548761	1	0.449908038
ENSMUSG00000078941	Ak6	108143	protein_coding	131.639024	0.449935957	0.110430286	-4.074389114	4.61E-05	0.00043511	0.449935957
ENSMUSG00000040812	Agbl2	271813	protein_coding	35.95918759	-0.450271445	0.354880552	1.268797184	0.20451341	0.406858746	0.450271445
ENSMUSG00000051736	Fam229b	66337	protein_coding	5.091200518	0.450307274	0.482642716	-0.933003356	0.350818235	0.572963936	0.450307274
ENSMUSG00000044037	Als2cl	235633	protein_coding	455.3478948	-0.450449635	0.171204284	2.631065207	0.00851177	0.037373936	0.450449635
ENSMUSG00000079580	Tmem217	71138	protein_coding	3.863646088	0.450702474	0.706020037	-0.638370655	0.523232428	1	0.450702474
ENSMUSG00000032000	Birc3	11796	protein_coding	485.6661365	-0.450965353	0.103404968	4.361157504	1.29E-05	0.000140653	0.450965353
ENSMUSG00000038128	Camk4	12326	protein_coding	4.076186762	0.451089194	0.664461584	-0.678879268	0.497214363	1	0.451089194
ENSMUSG00000073650	Catip	241112	protein_coding	2.263683746	0.451112105	1.508792966	-0.298988738	0.764948639	1	0.451112105
ENSMUSG00000032527	Pccb	66904	protein_coding	2502.654338	0.451131835	0.107833508	-4.183596013	2.87E-05	0.000285104	0.451131835
ENSMUSG00000039131	Gipc2	54120	protein_coding	1710.843042	-0.451146109	0.061112426	7.382231994	1.56E-13	5.08E-12	0.451146109
ENSMUSG00000021376	Tpmt	22017	protein_coding	145.7373507	-0.451275875	0.095740994	4.713507327	2.43E-06	3.10E-05	0.451275875
ENSMUSG00000041460	Cacna2d4	319734	protein_coding	12.89654523	0.451567643	0.647614113	-0.69727888	0.485628283	0.695380179	0.451567643
ENSMUSG00000005142	Man2b1	17159	protein_coding	1359.027933	-0.451616444	0.128334779	3.519049537	0.000433096	0.003149337	0.451616444
ENSMUSG00000090565	4930405O22Rik	414079	lncRNA	6.020608153	-0.451755639	0.479820388	0.941509885	0.346443626	0.568175062	0.451755639
ENSMUSG00000064307	Lrrc51	69358	protein_coding	37.54127648	-0.45176234	0.19156642	2.358254336	0.018361108	0.068585239	0.45176234
ENSMUSG00000100680	1810044D09Rik	69798	lncRNA	12.67821067	-0.451855516	0.320399448	1.410288062	0.158454642	0.342262172	0.451855516
ENSMUSG00000029607	Ankrd61	66729	protein_coding	11.12867942	-0.451867836	0.455774973	0.991427486	0.321476884	0.542471509	0.451867836
ENSMUSG00000031385	Plxnb3	140571	protein_coding	4.225033661	0.451929709	1.514466372	-0.298408547	0.765391367	1	0.451929709
ENSMUSG00000070498	Tmem132b	208151	protein_coding	4.920391046	0.451948092	1.007307355	-0.448669505	0.653670087	0.81212066	0.451948092
ENSMUSG00000026639	Lamb3	16780	protein_coding	3251.876467	0.452145529	0.153752807	-2.940730252	0.003274395	0.01711111	0.452145529
ENSMUSG00000038630	Zkscan16	100041581	protein_coding	1.072622222	0.452167249	1.784841166	-0.253337528	0.800007399	1	0.452167249
ENSMUSG00000070568	Slc6a21	76713	protein_coding	2.3357246	-0.45224324	1.772390476	0.255160048	0.798599487	1	0.45224324
ENSMUSG00000027654	Fam83d	71878	protein_coding	164.8221123	0.452283151	0.159749782	-2.831197291	0.004637411	0.022846718	0.452283151
ENSMUSG00000011589	Fsd1	240121	protein_coding	7.001741867	-0.452358353	0.61540664	0.735056017	0.46230538	0.675942878	0.452358353
ENSMUSG00000005672	Kit	16590	protein_coding	617.1475563	-0.452503741	0.232208998	1.948691673	0.051332257	0.152129476	0.452503741
ENSMUSG00000033705	Stard9	668880	protein_coding	11.87644145	0.452526152	0.70831004	-0.638881459	0.52290005	0.722022907	0.452526152
ENSMUSG00000084669	Gm23206	115488234	snRNA	2.878228843	-0.452575428	0.741123946	0.610660917	0.541424085	1	0.452575428
ENSMUSG00000114590	5930438M14Rik	328324	lncRNA	1.053439962	-0.452618558	1.378899454	0.328246238	0.74272549	1	0.452618558
ENSMUSG00000044813	Shb	230126	protein_coding	340.6828314	0.452790123	0.107863966	-4.197788564	2.70E-05	0.000269273	0.452790123
ENSMUSG00000018925	Heatr9	629303	protein_coding	2.647235069	0.452901766	1.073988543	-0.421700742	0.673243462	1	0.452901766
ENSMUSG00000022012	Enox1	239188	protein_coding	6.268478808	-0.453112849	0.747931416	0.605821389	0.544633365	0.736796542	0.453112849
ENSMUSG00000056155	Nanos3	244551	protein_coding	0.995244453	0.453308281	1.663730324	-0.272464999	0.7852645	1	0.453308281
ENSMUSG00000008153	Clstn3	232370	protein_coding	5.733117623	0.453366472	1.390823417	-0.32596983	0.744447182	0.871097624	0.453366472
ENSMUSG00000019961	Tmpo	21917	protein_coding	1693.729055	0.453574005	0.140215271	-3.234840275	0.001217108	0.007482994	0.453574005
ENSMUSG00000080727	C920021L13Rik	100042889	lncRNA	7.519670238	-0.453741391	0.473741766	0.957782116	0.338172639	0.559860817	0.453741391
ENSMUSG00000026815	Gfi1b	14582	protein_coding	8.037330404	-0.453853575	0.414504525	1.094930329	0.273547161	0.489845783	0.453853575
ENSMUSG00000027698	Nceh1	320024	protein_coding	730.4143298	-0.45390039	0.119461343	3.79955875	0.000144954	0.001195092	0.45390039

ensembl_gene_id	external_gene_name	entrezgene_id	gene_biotype	baseMean	log2FoldChange	lfcSE	stat	pvalue	padj	absLog2FC
ENSMUSG00000014786	Slc9a5	277973	protein_coding	56.98383599	0.454128697	0.203965719	-2.226495215	0.025981029	0.09002323	0.454128697
ENSMUSG00000030760	Acer3	66190	protein_coding	274.052669	-0.454192757	0.092869284	4.890667173	1.00E-06	1.39E-05	0.454192757
ENSMUSG00000038274	Fau	14109	protein_coding	3570.908476	-0.454229761	0.093544207	4.855776508	1.20E-06	1.62E-05	0.454229761
ENSMUSG00000047419	Cmya5	76469	protein_coding	3.455408347	0.45430362	1.18958425	-0.381901172	0.702534671	1	0.45430362
ENSMUSG00000017390	Aldoc	11676	protein_coding	153.143405	-0.45437845	0.183628293	2.474446839	0.013344268	0.053293166	0.45437845
ENSMUSG00000019843	Fyn	14360	protein_coding	31.69116201	0.454397269	0.275647626	-1.64847155	0.099255944	0.246223924	0.454397269
ENSMUSG00000020866	Cacna1g	12291	protein_coding	7.075889621	0.45463428	1.135955808	-0.400221801	0.688993158	0.833930977	0.45463428
ENSMUSG00000043439	Epop	103551	protein_coding	38.94792219	0.45475179	0.428890573	-1.060297937	0.289009078	0.507895701	0.45475179
ENSMUSG00000028943	Espn	56226	protein_coding	362.5170049	-0.454883364	0.23358103	1.947432821	0.051482866	0.1525001	0.454883364
ENSMUSG00000094584	Ms4a18	76002	protein_coding	9.715614168	-0.454909834	0.395992945	1.148782673	0.250645606	0.462889905	0.454909834
ENSMUSG00000004347	Pde1c	18575	protein_coding	17.56158493	0.454936704	0.672609406	-0.676375768	0.498802098	0.703739933	0.454936704
ENSMUSG00000040270	Bach2	12014	protein_coding	37.29151923	-0.455048061	0.255939996	1.777948222	0.075412359	0.201270862	0.455048061
ENSMUSG00000063894	Zkscan8	93681	protein_coding	187.2335421	-0.455062337	0.153283676	2.968759297	0.002990047	0.015825683	0.455062337
ENSMUSG00000056501	Cebpb	12608	protein_coding	151.7238841	0.455458921	0.251218274	-1.813000755	0.069831705	0.190674731	0.455458921
ENSMUSG00000026712	Mrc1	17533	protein_coding	11.60092243	0.455507691	0.409924963	-1.111197735	0.266483246	0.481477497	0.455507691
ENSMUSG00000062545	Tlr12	384059	protein_coding	2.555266575	0.455574448	1.016191082	-0.448315731	0.653925349	1	0.455574448
ENSMUSG00000046215	Rprml	104582	protein_coding	2.0241664	0.455747102	0.799778587	-0.56984159	0.568785144	1	0.455747102
ENSMUSG00000033805	Ephx4	384214	protein_coding	53.59836069	-0.455749266	0.169902638	2.6824143	0.007309288	0.033042863	0.455749266
ENSMUSG00000031618	Nr3c2	110784	protein_coding	2264.670624	-0.455786415	0.094962865	4.799627877	1.59E-06	2.10E-05	0.455786415
ENSMUSG00000032717	Mdfi	17240	protein_coding	7.98383472	0.455927113	0.501159805	-0.909743975	0.362957547	0.585225632	0.455927113
ENSMUSG00000073680	Tmem88b	320587	protein_coding	2.585082203	0.45595923	1.057909174	-0.431000355	0.666468112	1	0.45595923
ENSMUSG00000037852	Cpe	12876	protein_coding	60.54527734	-0.456128968	0.193868004	2.352781058	0.018633602	0.069446017	0.456128968
ENSMUSG00000020149	Rab1a	19324	protein_coding	3547.676157	0.456192869	0.068623614	-6.64775345	2.98E-11	7.94E-10	0.456192869
ENSMUSG00000038670	Mybpc2	233199	protein_coding	4.359735534	-0.456204822	1.13155128	0.403167607	0.686824928	1	0.456204822
ENSMUSG00000044921	Rassf9	237504	protein_coding	87.23444602	-0.456358219	0.211071144	2.162106153	0.030609995	0.102451045	0.456358219
ENSMUSG00000028063	Lmna	16905	protein_coding	5558.575528	-0.456558786	0.083707878	5.454191342	4.92E-08	8.45E-07	0.456558786
ENSMUSG00000026255	Efhd1	98363	protein_coding	4.653546524	0.456594202	0.55159799	-0.827766253	0.40780289	0.628507412	0.456594202
ENSMUSG00000076436	Oxct2a	64059	protein_coding	6.396688263	-0.456607455	0.532618174	0.857288537	0.391285442	0.613645564	0.456607455
ENSMUSG00000027824	Vmn2r1	56544	protein_coding	1.151493856	-0.456743966	1.985518595	0.230037617	0.818062539	1	0.456743966
ENSMUSG00000050555	Hyls1	76832	protein_coding	80.23776213	0.456807046	0.138744845	-3.292425352	0.000993273	0.006330723	0.456807046
ENSMUSG00000029553	Tfec	21426	protein_coding	0.883982192	0.456878508	1.953914082	-0.233827328	0.815119033	1	0.456878508
ENSMUSG00000031146	Plp2	18824	protein_coding	816.3280395	-0.456933864	0.119953858	3.809246901	0.000139391	0.001154992	0.456933864
ENSMUSG00000087593	Gm16174	102638408	lncRNA	14.16951093	0.456981812	0.292861043	-1.56040492	0.118664223	0.279706106	0.456981812
ENSMUSG00000073529	F830208F22Rik	619308	lncRNA	5.545066673	-0.456991537	0.695795003	0.656790483	0.511315644	0.71414691	0.456991537
ENSMUSG00000069270	H2ac6	319164	protein_coding	287.5560934	0.457078711	0.14936921	-3.060059783	0.002212928	0.012282071	0.457078711
ENSMUSG00000074673	Ttll9	74711	protein_coding	2.64675703	-0.457094242	1.32198849	0.345762649	0.729521098	1	0.457094242
ENSMUSG00000030554	Synm	233335	protein_coding	6.048099546	0.457195294	0.664317764	-0.688217776	0.491315652	0.698491194	0.457195294
ENSMUSG00000029581	Fscn1	14086	protein_coding	3.301436769	0.457604946	0.801353136	-0.571040313	0.567972317	1	0.457604946
ENSMUSG00000062760	Shisal1	72301	protein_coding	6.666743207	0.457634652	0.691055048	-0.662226047	0.507826364	0.711603273	0.457634652

ensembl_gene_id	external_gene_name	entrezgene_id	gene_biotype	baseMean	log2FoldChange	lfcSE	stat	pvalue	padj	absLog2FC
ENSMUSG00000005881	Ergic3	66366	protein_coding	1201.49326	-0.457682286	0.072376529	6.323628566	2.55E-10	6.08E-09	0.457682286
ENSMUSG00000056305	Usp39	28035	protein_coding	310.9536195	0.458124786	0.087779968	-5.219012877	1.80E-07	2.84E-06	0.458124786
ENSMUSG00000044250	Pced1b	239647	protein_coding	2.731902687	0.458130668	0.870285682	-0.526414116	0.59860051	1	0.458130668
ENSMUSG00000035699	Slc51a	106407	protein_coding	562.9237994	-0.458183881	0.276894457	1.654723919	0.097980513	0.243645564	0.458183881
ENSMUSG00000030432	Rpl28	19943	protein_coding	3355.339182	-0.458245854	0.123128253	3.721695422	0.00019789	0.00156603	0.458245854
ENSMUSG00000032311	Nrg4	83961	protein_coding	14.44962026	-0.458523111	0.335769405	1.365589313	0.172067903	0.362486203	0.458523111
ENSMUSG00000037428	Vgf	381677	protein_coding	6.244944824	-0.458907835	0.878519106	0.522365229	0.601416061	0.777725809	0.458907835
ENSMUSG00000004187	Kifc2	16581	protein_coding	135.2386902	-0.459052278	0.261011333	1.758744623	0.078620894	0.207507849	0.459052278
ENSMUSG00000005952	Trpv1	193034	protein_coding	7.557979413	-0.459110636	0.477997125	0.960488278	0.336809528	0.559233675	0.459110636
ENSMUSG00000042115	Klhdc8a	213417	protein_coding	102.3951251	-0.45916776	0.148493182	3.092180751	0.001986918	0.011271717	0.45916776
ENSMUSG00000058794	Nfe2	18022	protein_coding	7.285971883	0.459223805	0.645341388	-0.711598254	0.476713588	0.68833522	0.459223805
ENSMUSG00000017778	Cox7c	12867	protein_coding	1621.738254	-0.459314199	0.111264576	4.128126096	3.66E-05	0.00035465	0.459314199
ENSMUSG00000101581	C430002N11Rik	319707	lncRNA	5.035726409	-0.45938012	0.589126154	0.779765278	0.435529047	0.653742472	0.45938012
ENSMUSG00000031227	Magee1	107528	protein_coding	11.24022182	0.459395617	0.315385199	-1.456617553	0.14522198	0.322338142	0.459395617
ENSMUSG00000055884	Fancm	104806	protein_coding	119.961835	0.459563448	0.118972804	-3.862760516	0.000112113	0.000952873	0.459563448
ENSMUSG00000025409	Mbd6	110962	protein_coding	197.1092124	0.45957216	0.25338527	-1.813728792	0.06971949	0.190494318	0.45957216
ENSMUSG00000022369	Mtbp	105837	protein_coding	78.31923301	0.459655699	0.155361934	-2.958612103	0.003090278	0.016283616	0.459655699
ENSMUSG00000041571	Selenow	20364	protein_coding	464.0144297	-0.459776682	0.064884179	7.086113908	1.38E-12	4.11E-11	0.459776682
ENSMUSG00000025791	Pgm1	72157	protein_coding	1251.022536	0.459862032	0.111805004	-4.11307202	3.90E-05	0.000376608	0.459862032
ENSMUSG00000042784	Muc1	17829	protein_coding	89.57783023	-0.459865791	0.222649925	2.06542082	0.0388832	0.123253915	0.459865791
ENSMUSG00000035478	Mbd3	17192	protein_coding	457.1232174	0.460005642	0.091659642	-5.018627944	5.20E-07	7.67E-06	0.460005642
ENSMUSG00000024810	Il33	77125	protein_coding	8.907093825	0.460083997	0.71444519	-0.643973819	0.519592411	0.719844721	0.460083997
ENSMUSG00000044788	Fads6	328035	protein_coding	15.90813112	-0.460118197	0.347140987	1.325450507	0.185021775	0.38040956	0.460118197
ENSMUSG00000063558	Aox1	11761	protein_coding	2.739077259	-0.460212891	1.407880861	0.326883406	0.743756072	1	0.460212891
ENSMUSG00000035580	Kcnh8	211468	protein_coding	10.37192118	-0.460373834	0.450862559	1.02109573	0.307209086	0.527698391	0.460373834
ENSMUSG00000057914	Cacnb2	12296	protein_coding	5.299847693	0.460428863	0.889443723	-0.517659354	0.604695962	0.779964063	0.460428863
ENSMUSG00000056429	Tgoln1	22134	protein_coding	11969.85452	-0.460470368	0.102624595	4.486939704	7.23E-06	8.37E-05	0.460470368
ENSMUSG00000025597	Klhl4	237010	protein_coding	0.9043219	-0.460517273	2.160109019	0.213191681	0.831177456	1	0.460517273
ENSMUSG00000031075	Ano1	101772	protein_coding	1838.864703	-0.460563075	0.189410359	2.431562225	0.015033866	0.058500114	0.460563075
ENSMUSG00000062078	Qki	19317	protein_coding	9.394283861	0.461382129	0.435341929	-1.059815511	0.289228539	0.508065109	0.461382129
ENSMUSG00000061273	Mmgt1	236792	protein_coding	503.5561078	0.461414286	0.120625825	-3.825169986	0.000130682	0.001092699	0.461414286
ENSMUSG00000074519	Zfp971	626848	protein_coding	77.87987359	-0.461612222	0.158435208	2.913570969	0.003573206	0.018411209	0.461612222
ENSMUSG00000020818	Mfsd11	69900	protein_coding	264.7616294	0.461795869	0.077378901	-5.967981731	2.40E-09	5.07E-08	0.461795869
ENSMUSG00000070871	Ccnyl1	227210	protein_coding	134.6604215	0.46199229	0.10737518	-4.302598524	1.69E-05	0.000177672	0.46199229
ENSMUSG00000074136	4930513N10Rik	319960	lncRNA	26.40119587	0.462029547	0.322264546	-1.433696484	0.151658896	0.331941022	0.462029547
ENSMUSG00000117916	9630028I04Rik	319720	lncRNA	1.85538576	0.462083447	1.746348493	-0.264599791	0.791317781	1	0.462083447
ENSMUSG00000021565	Slc6a19	74338	protein_coding	24.93142059	-0.462087883	0.515134121	0.897024414	0.36970589	0.592404722	0.462087883
ENSMUSG00000052135	Foxo6	329934	protein_coding	20.1092518	-0.462381178	0.363201816	1.273069564	0.202993358	0.405213026	0.462381178
ENSMUSG00000047053	Teddm1a	240819	protein_coding	1.054587016	0.462645232	2.266044493	-0.204164231	0.838225162	1	0.462645232

Supplementary Table 1

ensembl_gene_id	external_gene_name	entrezgene_id	gene_biotype	baseMean	log2FoldChange	lfcSE	stat	pvalue	padj	absLog2FC
ENSMUSG00000029033	Acap3	140500	protein_coding	133.9596917	-0.462733605	0.120594794	3.837094377	0.000124499	0.00104843	0.462733605
ENSMUSG00000067860	Zic3	22773	protein_coding	1.206198641	0.46280127	2.277154727	-0.203236637	0.838950078	1	0.46280127
ENSMUSG00000099826	Scgb2b10	624584	unprocessed_pseudogene	1.292615626	0.462857951	1.224906896	-0.377871945	0.705525725	1	0.462857951
ENSMUSG00000049013	Prss48	368202	protein_coding	10.35613046	-0.462909157	0.44116386	1.049290749	0.294044322	0.513159947	0.462909157
ENSMUSG00000032952	Ap4b1	67489	protein_coding	46.85776379	0.46291648	0.157864793	-2.932360471	0.00336396	0.01751338	0.46291648
ENSMUSG00000031714	Gab1	14388	protein_coding	332.626056	-0.462958407	0.09330694	4.961671736	6.99E-07	9.98E-06	0.462958407
ENSMUSG00000031398	Plxna3	18846	protein_coding	55.62183707	-0.462968882	0.164177078	2.819936184	0.00480332	0.023502823	0.462968882
ENSMUSG00000025351	Cd63	12512	protein_coding	2203.851664	-0.463254931	0.074101251	6.251647928	4.06E-10	9.39E-09	0.463254931
ENSMUSG00000048029	Eno4	226265	protein_coding	3.25085477	0.463692611	0.848533594	-0.546463468	0.584747393	1	0.463692611
ENSMUSG00000020689	Itgb3	16416	protein_coding	3.206184111	0.463708688	1.024566992	-0.452589915	0.650844062	1	0.463708688
ENSMUSG00000100153	Ppp1ccb	434233	transcribed_processed_pseudogene	9.960299267	0.463773596	0.366476395	-1.265493774	0.205694379	0.408322752	0.463773596
ENSMUSG00000028889	Yrdc	230734	protein_coding	278.6296466	0.463784071	0.098563978	-4.705411476	2.53E-06	3.22E-05	0.463784071
ENSMUSG00000091670	Vmn2r105	627743	protein_coding	2.683204595	0.463887774	2.730137059	-0.169913731	0.865077982	1	0.463887774
ENSMUSG00000109714	Gm35021	102638463	lncRNA	1.473126396	0.463900576	1.826772404	-0.25394547	0.799537686	1	0.463900576
ENSMUSG00000029759	Pon3	269823	protein_coding	647.7494364	-0.464141254	0.076556625	6.062718323	1.34E-09	2.91E-08	0.464141254
ENSMUSG00000020181	Nav3	260315	protein_coding	4.621563981	0.464176267	1.935627895	-0.23980656	0.810480221	0.906179443	0.464176267
ENSMUSG00000051486	Pcdhb11	93882	protein_coding	2.100825372	-0.46423241	0.916891424	0.506311214	0.612638192	1	0.46423241
ENSMUSG00000022935	Grik1	14805	protein_coding	2.310974618	0.464255028	2.745473404	-0.169098352	0.865719281	1	0.464255028
ENSMUSG00000057719	Sh3rf2	269016	protein_coding	218.0298426	-0.464331038	0.163966377	2.8318674	0.004627704	0.022812506	0.464331038
ENSMUSG00000024299	Adamts10	224697	protein_coding	36.67201618	0.464415301	0.196430179	-2.36427673	0.018065313	0.06767935	0.464415301
ENSMUSG00000000732	Icosl	50723	protein_coding	64.11463815	-0.464489028	0.188488994	2.464276663	0.013729009	0.054421559	0.464489028
ENSMUSG00000027881	Prpf38b	66921	protein_coding	921.0098777	0.464541548	0.063574881	-7.306998265	2.73E-13	8.69E-12	0.464541548
ENSMUSG00000024502	Jakmip2	76217	protein_coding	4.085529913	0.464637386	1.341658748	-0.346315624	0.729105528	1	0.464637386
ENSMUSG00000044807	Zfp354c	30944	protein_coding	3.627428433	0.464730089	0.628778843	-0.739099437	0.459846619	1	0.464730089
ENSMUSG00000026274	Pask	269224	protein_coding	88.13422769	0.46487801	0.145695569	-3.190749127	0.001419044	0.008514609	0.46487801
ENSMUSG00000047238	Mageh1	75625	protein_coding	64.92819832	0.465195928	0.178418155	-2.607335162	0.009124999	0.039582411	0.465195928
ENSMUSG00000109372	Gm19410	100502846	protein_coding	14.21064954	0.465267349	0.51643945	-0.900913647	0.367634235	0.590110273	0.465267349
ENSMUSG00000069633	Pex11g	69129	protein_coding	132.0350097	0.465341194	0.10193752	-4.564964834	5.00E-06	5.97E-05	0.465341194
ENSMUSG00000049538	Adamts16	271127	protein_coding	12.24699685	-0.465456359	0.370440254	1.256495085	0.208936527	0.412258246	0.465456359
ENSMUSG00000043456	Zfp536	243937	protein_coding	4.945321792	0.465475275	2.143822795	-0.217123951	0.828111749	0.913297021	0.465475275
ENSMUSG00000073408	Mucl3	268949	protein_coding	1.03076368	-0.465534602	2.170611155	0.214471671	0.830179256	1	0.465534602
ENSMUSG00000029166	Mapre3	100732	protein_coding	117.5871664	0.465640739	0.373410057	-1.24699571	0.212399071	0.416871672	0.465640739
ENSMUSG00000048827	Pkd1l3	244646	protein_coding	17.51327116	-0.465668024	0.34230304	1.360396987	0.173704332	0.364445856	0.465668024
ENSMUSG00000029149	Krtcap3	69815	protein_coding	623.5015703	-0.465836225	0.085802141	5.429191128	5.66E-08	9.61E-07	0.465836225
ENSMUSG00000075520	Malrd1	102635496	protein_coding	307.4158627	-0.465886769	0.220056926	2.117119323	0.034249722	0.111571269	0.465886769
ENSMUSG00000012428	Steap4	117167	protein_coding	65.51503263	0.465989625	0.264957494	-1.758733519	0.078622781	0.207507849	0.465989625
ENSMUSG00000034842	Art3	109979	protein_coding	3.547614589	0.466068923	0.870394929	-0.535468334	0.592326049	1	0.466068923
ENSMUSG00000022194	Pabpn1	12050	protein_coding	285.5957835	0.466081556	0.185736517	-2.509369533	0.012094688	0.049414074	0.466081556
ENSMUSG00000044646	Zbtb7c	207259	protein_coding	378.7141027	0.466165452	0.128933521	-3.615548922	0.000299712	0.002276086	0.466165452

ensembl_gene_id	external_gene_name	entrezgene_id	gene_biotype	baseMean	log2FoldChange	lfcSE	stat	pvalue	padj	absLog2FC
ENSMUSG00000028614	Ndc1	72787	protein_coding	267.2450282	0.466266324	0.182619342	-2.553214353	0.010673379	0.04488418	0.466266324
ENSMUSG00000090220	Pitpnm2os1	75927	lncRNA	0.711202118	-0.466445507	1.653849914	0.282036177	0.777915771	1	0.466445507
ENSMUSG00000035183	Slc24a5	317750	protein_coding	5.438212479	0.466547101	0.537835822	-0.867452636	0.385694053	0.607820554	0.466547101
ENSMUSG00000096795	Zfp433	73610	protein_coding	7.878656976	-0.466646119	0.422292054	1.105031729	0.269145841	0.484159903	0.466646119
ENSMUSG00000020328	Nudcd2	52653	protein_coding	307.0368417	0.466846025	0.113821322	-4.101569185	4.10E-05	0.000393534	0.466846025
ENSMUSG00000047938	4930483J18Rik	67638	lncRNA	0.927086421	0.46693282	1.609942913	-0.290030669	0.771792775	1	0.46693282
ENSMUSG00000028841	Cnksr1	194231	protein_coding	537.1333268	0.466984204	0.133391962	-3.500842158	0.00046379	0.003327122	0.466984204
ENSMUSG00000039934	Gsap	212167	protein_coding	41.62402427	0.467064606	0.285894761	-1.633694175	0.102323082	0.251865163	0.467064606
ENSMUSG00000026407	Cacna1s	12292	protein_coding	3.050140602	0.467157861	2.21281001	-0.211115215	0.832797368	1	0.467157861
ENSMUSG00000021591	Glrx	93692	protein_coding	693.7739106	-0.467243866	0.083393457	5.602884027	2.11E-08	3.86E-07	0.467243866
ENSMUSG00000032532	Cck	12424	protein_coding	14.94979161	-0.467267735	0.378579261	1.234266592	0.21710358	0.422731291	0.467267735
ENSMUSG00000004263	Atn1	13498	protein_coding	20.89237574	0.467294103	0.483409504	-0.966663044	0.333712508	0.556097714	0.467294103
ENSMUSG00000037944	Ccr7	12775	protein_coding	2.80737558	0.467307482	0.919880942	-0.508008657	0.611447272	1	0.467307482
ENSMUSG00000033021	Gmppa	69080	protein_coding	877.7133015	0.467421653	0.073473519	-6.361770371	1.99E-10	4.80E-09	0.467421653
ENSMUSG00000022070	Bora	77744	protein_coding	192.3632293	0.467734146	0.107111422	-4.366799896	1.26E-05	0.000137432	0.467734146
ENSMUSG00000048997	Atxn7l2	72522	protein_coding	45.31718395	-0.467747712	0.207150235	2.258011976	0.023944913	0.084474747	0.467747712
ENSMUSG00000074088	Snrnp40	66585	protein_coding	469.0111552	0.467791684	0.071604935	-6.532953143	6.45E-11	1.66E-09	0.467791684
ENSMUSG00000026068	Il18rap	16174	protein_coding	4.677070469	0.467890195	0.804333389	-0.581711765	0.560760846	0.749223673	0.467890195
ENSMUSG00000061769	Klra6	16637	protein_coding	1.447507332	-0.467966747	1.239059719	0.377678929	0.705669123	1	0.467966747
ENSMUSG00000045757	Zfp764	233893	protein_coding	79.89149714	-0.46798189	0.156434754	2.991546815	0.00277568	0.014921086	0.46798189
ENSMUSG00000093626	Ly6l	102637705	protein_coding	0.841356297	-0.468603076	2.138869838	0.219089104	0.826580639	1	0.468603076
ENSMUSG00000039682	Lap3	66988	protein_coding	956.4867207	0.468610868	0.087853181	-5.334022767	9.61E-08	1.59E-06	0.468610868
ENSMUSG00000025075	Habp2	226243	protein_coding	2.916101105	-0.468630298	0.633609673	0.739619861	0.459530687	1	0.468630298
ENSMUSG00000036216	Leap2	259301	protein_coding	2.078990679	-0.468677653	0.771463923	0.607517266	0.543507688	1	0.468677653
ENSMUSG00000038146	Notch3	18131	protein_coding	6.442227996	-0.469118693	0.910107261	0.515454291	0.606235603	0.781162199	0.469118693
ENSMUSG00000037364	Srrt	83701	protein_coding	1172.271533	0.469244548	0.065796176	-7.131790527	9.91E-13	3.00E-11	0.469244548
ENSMUSG00000066224	Arid3c	550619	protein_coding	1.317453713	-0.469258782	1.84893786	0.25379911	0.799650761	1	0.469258782
ENSMUSG00000035045	Zc3h12b	547176	protein_coding	5.290560113	0.469294466	0.606149426	-0.774222405	0.438799281	0.656846567	0.469294466
ENSMUSG00000017764	Zswim1	71971	protein_coding	117.9526382	0.469334349	0.133521737	-3.51504077	0.000439686	0.003190048	0.469334349
ENSMUSG00000107877	4933427D14Rik	74477	protein_coding	6.482535297	-0.469439722	0.704879763	0.66598553	0.50542035	0.709429427	0.469439722
ENSMUSG00000058952	Cfi	12630	protein_coding	3.165295345	0.469450627	0.634859873	-0.739455504	0.459630449	1	0.469450627
ENSMUSG00000101930	Gm5441	432713	lncRNA	2.836536298	0.469637098	1.01447057	-0.46293812	0.643408723	1	0.469637098
ENSMUSG00000026688	Mgst3	66447	protein_coding	5649.409416	-0.469680091	0.093948616	4.999329508	5.75E-07	8.36E-06	0.469680091
ENSMUSG00000029648	Flt1	14254	protein_coding	6.389910573	0.469821249	1.617721542	-0.290421582	0.771493735	0.885228942	0.469821249
ENSMUSG00000025193	Cutc	66388	protein_coding	117.4187564	-0.469842636	0.172302668	2.726844814	0.00639431	0.029520727	0.469842636
ENSMUSG00000037674	Rfx7	319758	protein_coding	460.230867	0.469885054	0.099976746	-4.699943455	2.60E-06	3.29E-05	0.469885054
ENSMUSG00000027544	Nfatc2	18019	protein_coding	59.31645313	-0.469996099	0.200521914	2.343864023	0.019085128	0.070745635	0.469996099
ENSMUSG00000037490	Slc2a12	353169	protein_coding	4.662177629	-0.470034754	0.762728199	0.616254591	0.537726504	0.733113936	0.470034754
ENSMUSG00000025347	Mettl7b	71664	protein_coding	3232.229152	-0.470090749	0.156798386	2.998058593	0.002717054	0.014644071	0.470090749

Supplementary Table 1

ensembl_gene_id	external_gene_name	entrezgene_id	gene_biotype	baseMean	log2FoldChange	lfcSE	stat	pvalue	padj	absLog2FC
ENSMUSG00000097768	2310043M15Rik	102633000	lncRNA	6.888303282	-0.47015899	0.490108588	0.959295555	0.337409874	0.559438733	0.47015899
ENSMUSG00000041476	Smpx	66106	protein_coding	74.92847309	0.470258404	0.209492738	-2.244748003	0.024784314	0.086787144	0.470258404
ENSMUSG00000050108	Bpifc	270757	protein_coding	1.892381843	0.470303085	1.541031407	-0.305187216	0.760223568	1	0.470303085
ENSMUSG00000057286	St6galnac2	20446	protein_coding	490.9551484	-0.470468499	0.118226555	3.979380922	6.91E-05	0.000623516	0.470468499
ENSMUSG00000020847	Rph3al	380714	protein_coding	335.1317162	0.470589922	0.166743455	-2.822239239	0.004768959	0.023369313	0.470589922
ENSMUSG00000038126	Mphosph9	269702	protein_coding	158.4800113	0.470719327	0.147298306	-3.19568731	0.001394982	0.008398372	0.470719327
ENSMUSG00000025480	Syce1	74075	protein_coding	1.05413698	0.470792025	1.479181207	-0.318278803	0.750273461	1	0.470792025
ENSMUSG00000022519	Srl	106393	protein_coding	101.2181997	0.470860619	0.149357371	-3.15257705	0.001618361	0.00949063	0.470860619
ENSMUSG00000007029	Vars	22321	protein_coding	1309.767714	0.470933205	0.124573818	-3.780354593	0.000156605	0.001274607	0.470933205
ENSMUSG00000021874	4933413J09Rik	71106	lncRNA	2.500832805	-0.471011559	1.012885108	0.465019729	0.641917328	1	0.471011559
ENSMUSG00000094282	Cfap73	546886	protein_coding	0.697544318	-0.47102371	2.209119698	0.213217831	0.831157061	1	0.47102371
ENSMUSG00000047945	Marcksl1	17357	protein_coding	186.1303169	0.471033818	0.159859421	-2.946550261	0.003213402	0.01685709	0.471033818
ENSMUSG00000066964	Tmem211	333048	protein_coding	2.046128449	0.471132947	1.44435147	-0.326189959	0.744280638	1	0.471132947
ENSMUSG00000029776	Hibadh	58875	protein_coding	742.1563521	-0.471201004	0.105207247	4.478788478	7.51E-06	8.68E-05	0.471201004
ENSMUSG00000090841	Myl6	17904	protein_coding	5529.678099	-0.471235529	0.083466523	5.645802787	1.64E-08	3.06E-07	0.471235529
ENSMUSG00000037492	Zmat4	320158	protein_coding	13.51560238	-0.471481987	0.505673073	0.932384998	0.351137595	0.573211901	0.471481987
ENSMUSG00000102752	Gm7694	665574	protein_coding	69.77448562	-0.471555955	0.146337205	3.222392791	0.001271247	0.007756249	0.471555955
ENSMUSG00000032348	Gsta4	14860	protein_coding	473.1813114	-0.471573847	0.121957312	3.866712369	0.000110312	0.00093902	0.471573847
ENSMUSG00000070732	Rbm44	329207	protein_coding	1.538714407	-0.471634295	1.062702971	0.44380632	0.657182619	1	0.471634295
ENSMUSG00000024818	Slc25a45	107375	protein_coding	23.5128208	-0.471782667	0.217916582	2.164969103	0.030390051	0.101900774	0.471782667
ENSMUSG00000058586	Serhl	68607	protein_coding	448.8863745	-0.471869945	0.101530985	4.647546225	3.36E-06	4.16E-05	0.471869945
ENSMUSG00000031662	Snx20	71607	protein_coding	170.0368053	0.471883486	0.149375038	-3.159051816	0.001582833	0.009312046	0.471883486
ENSMUSG00000071719	Nalf2	620592	protein_coding	2.786751286	-0.471954244	1.248217088	0.378102694	0.705354307	1	0.471954244
ENSMUSG00000033065	Pfkm	18642	protein_coding	127.3495707	-0.471978446	0.128443704	3.674593862	0.000238228	0.001847862	0.471978446
ENSMUSG00000041353	Tmem29	382245	protein_coding	99.35241978	0.47227642	0.131754316	-3.58452333	0.000337694	0.002527962	0.47227642
ENSMUSG00000004609	Cd33	12489	protein_coding	5.682651899	-0.472446099	0.979825699	0.482173614	0.629682619	0.797713998	0.472446099
ENSMUSG00000020376	Rnf130	59044	protein_coding	648.0970419	-0.472623409	0.116038754	4.072978997	4.64E-05	0.000437255	0.472623409
ENSMUSG00000061046	Haghl	68977	protein_coding	72.90591381	-0.472633771	0.151683567	3.115919413	0.001833724	0.010565985	0.472633771
ENSMUSG00000029436	Mmp17	23948	protein_coding	7.614402339	0.472811361	0.508517527	-0.929783804	0.352483034	0.574847405	0.472811361
ENSMUSG00000044139	Prss53	330657	protein_coding	3.736389824	0.472823971	0.5551043	-0.85177501	0.394338979	1	0.472823971
ENSMUSG00000067599	Klra7	16638	protein_coding	4.907183715	0.472829546	0.67336036	-0.702193913	0.482558241	0.693375689	0.472829546
ENSMUSG00000029864	Gstk1	76263	protein_coding	567.3508664	-0.472879579	0.098720541	4.790082917	1.67E-06	2.19E-05	0.472879579
ENSMUSG00000034362	Csta1	209294	protein_coding	20.03341865	0.473400164	0.320401895	-1.477519861	0.139536338	0.313084659	0.473400164
ENSMUSG00000047115	Fam221a	231946	protein_coding	55.49319286	-0.473422634	0.157899937	2.998244598	0.002715396	0.014639914	0.473422634
ENSMUSG00000039873	Neurl2	415115	protein_coding	3.637728304	-0.473489351	0.579770401	0.816684242	0.414108899	1	0.473489351
ENSMUSG00000020534	Shmt1	20425	protein_coding	1066.360831	0.473524223	0.130357801	-3.632496245	0.000280693	0.002147988	0.473524223
ENSMUSG00000022132	Cldn10	58187	protein_coding	3.200519201	0.47352596	0.685545931	-0.690728277	0.489736316	1	0.47352596
ENSMUSG00000044702	Palb2	233826	protein_coding	56.87086493	0.473754177	0.149892972	-3.160616345	0.001574357	0.009275396	0.473754177
ENSMUSG00000034324	Tmem132c	208213	protein_coding	25.26704998	-0.473793114	0.290137044	1.632997664	0.102469488	0.252075246	0.473793114

ensembl_gene_id	external_gene_name	entrezgene_id	gene_biotype	baseMean	log2FoldChange	lfcSE	stat	pvalue	padj	absLog2FC
ENSMUSG00000061119	Prcp	72461	protein_coding	563.4440398	-0.473807839	0.089804116	5.276014738	1.32E-07	2.14E-06	0.473807839
ENSMUSG00000067613	Krt83	100126226	protein_coding	3.543466219	0.473813813	0.623047273	-0.76047811	0.446968849	1	0.473813813
ENSMUSG00000049555	Tmie	20776	protein_coding	15.95387722	-0.473873471	0.381283613	1.242837232	0.213927819	0.418926125	0.473873471
ENSMUSG00000027761	Aadac	67758	protein_coding	752.610648	-0.473954241	0.163453103	2.899634395	0.003735981	0.019112763	0.473954241
ENSMUSG00000003680	Taf6l	225895	protein_coding	108.4813491	0.474185589	0.151566757	-3.128559317	0.001756656	0.010174535	0.474185589
ENSMUSG00000038523	1700003F12Rik	75480	protein_coding	0.699402081	-0.474188315	1.524629972	0.311018623	0.755786464	1	0.474188315
ENSMUSG00000086693	A730081D07Rik	102633880	lncRNA	1.405513142	-0.474390274	1.140741681	0.415861261	0.677511528	1	0.474390274
ENSMUSG00000013974	Mcemp1	69189	protein_coding	2.406692044	0.474411821	1.919164666	-0.247197038	0.804755738	1	0.474411821
ENSMUSG00000039887	Alg14	66789	protein_coding	201.2565418	0.474511873	0.124229614	-3.81963572	0.000133649	0.001112439	0.474511873
ENSMUSG00000031157	Pqbp1	54633	protein_coding	332.8393723	0.474591074	0.084986586	-5.584305647	2.35E-08	4.27E-07	0.474591074
ENSMUSG00000022148	Fyb	23880	protein_coding	70.13702095	-0.474597277	0.213346109	2.224541521	0.026112032	0.090344458	0.474597277
ENSMUSG00000027132	Katnbl1	72425	protein_coding	134.382643	0.474629343	0.117830272	-4.028076451	5.62E-05	0.000518229	0.474629343
ENSMUSG00000026308	Klhl30	70788	protein_coding	1.4756976	0.474833706	1.431423844	-0.331721249	0.740099754	1	0.474833706
ENSMUSG00000047766	Lrrc49	102747	protein_coding	100.5995998	0.475097122	0.144883597	-3.279164321	0.00104115	0.006582523	0.475097122
ENSMUSG00000035513	Ntng2	171171	protein_coding	2.852251825	0.475135774	1.8115756	-0.262277641	0.793107399	1	0.475135774
ENSMUSG00000059842	Zfp341	228807	protein_coding	42.38202689	-0.475230041	0.160081791	2.968670198	0.002990914	0.015825683	0.475230041
ENSMUSG00000026173	Plcd4	18802	protein_coding	7.50826548	-0.475356803	0.578577424	0.821595837	0.41130696	0.631550268	0.475356803
ENSMUSG00000013033	Adgrl1	330814	protein_coding	50.08510284	0.475357012	0.26616321	-1.785960622	0.074105634	0.198998609	0.475357012
ENSMUSG00000029161	Cgref1	68567	protein_coding	1315.163731	0.475403327	0.085140966	-5.583720112	2.35E-08	4.28E-07	0.475403327
ENSMUSG00000037617	Spag1	26942	protein_coding	35.07199557	0.475652166	0.252730611	-1.882052053	0.059828948	0.170406218	0.475652166
ENSMUSG00000044055	Otos	260301	protein_coding	0.922942345	0.475764025	2.268129417	-0.209760528	0.833854581	1	0.475764025
ENSMUSG00000016344	Ppdpf	66496	protein_coding	517.0560215	-0.475829602	0.114483547	4.156314301	3.23E-05	0.000318106	0.475829602
ENSMUSG00000118061	Rbfaos	105246506	lncRNA	2.20174845	0.475835412	1.076903266	-0.441855297	0.658593921	1	0.475835412
ENSMUSG00000042510	AA986860	212439	protein_coding	454.4262282	0.475896206	0.159858881	-2.976976972	0.002911059	0.01548252	0.475896206
ENSMUSG00000042842	Serpinb6b	20708	protein_coding	5.428352434	0.47606318	0.549101548	-0.866985682	0.385949855	0.608165639	0.47606318
ENSMUSG00000028133	Rwdd3	66568	protein_coding	25.72703948	0.476073202	0.255906568	-1.860339913	0.06283745	0.176389198	0.476073202
ENSMUSG00000116810	Gm32624	102635233	lncRNA	1.002291532	0.476172055	2.290609601	-0.207880057	0.835322621	1	0.476172055
ENSMUSG00000056758	Hmga2	15364	protein_coding	105.837822	-0.476212359	0.123093659	3.868699354	0.000109417	0.000932912	0.476212359
ENSMUSG00000029369	Afm	280662	protein_coding	39.12336394	0.476386138	0.234885162	-2.028166162	0.042543292	0.131812369	0.476386138
ENSMUSG00000033055	Ankrd54	223690	protein_coding	209.9735642	0.476415029	0.082080035	-5.804274222	6.46E-09	1.28E-07	0.476415029
ENSMUSG00000040537	Adam22	11496	protein_coding	38.13222131	-0.476431975	0.263267721	1.809686254	0.070344455	0.1916312	0.476431975
ENSMUSG00000070425	Xntrpc	102443351	protein_coding	1.896769625	0.476760364	0.969013883	-0.492005711	0.622715307	1	0.476760364
ENSMUSG00000031104	Rab33a	19337	protein_coding	3.338804102	-0.476841013	1.029349749	0.463244892	0.643188842	1	0.476841013
ENSMUSG00000106445	Gm21190	100861755	protein_coding	2.574209659	-0.477079216	0.989027572	0.482372009	0.629541702	1	0.477079216
ENSMUSG00000093772	4931403E22Rik	70942	lncRNA	1.654459326	-0.477217294	0.950420129	0.502111939	0.615588782	1	0.477217294
ENSMUSG00000041420	Meis3	17537	protein_coding	98.54348924	-0.47727088	0.175273871	2.72300074	0.006469191	0.02983307	0.47727088
ENSMUSG00000022488	Nckap1l	105855	protein_coding	15.47486199	0.477491643	0.357525411	-1.335546028	0.181697701	0.375915289	0.477491643
ENSMUSG00000086638	4930405A21Rik	74846	lncRNA	2.991033092	0.477511936	0.605997196	-0.787977138	0.430710079	1	0.477511936
ENSMUSG00000117226	Gm31235	102633401	lncRNA	27.35772577	-0.477528797	0.352422669	1.35498888	0.1754211	0.36725457	0.477528797

ensembl_gene_id	external_gene_name	entrezgene_id	gene_biotype	baseMean	log2FoldChange	lfcSE	stat	pvalue	padj	absLog2FC
ENSMUSG00000070044	Fam149a	212326	protein_coding	107.2583727	0.477555724	0.136750706	-3.492162769	0.000479126	0.003417675	0.477555724
ENSMUSG00000048960	Prex2	109294	protein_coding	5.246609321	0.477672308	1.076826871	-0.443592485	0.65733724	0.814655179	0.477672308
ENSMUSG00000020696	Rffl	67338	protein_coding	291.271079	-0.477713772	0.100889704	4.735010161	2.19E-06	2.83E-05	0.477713772
ENSMUSG00000004933	Matk	17179	protein_coding	101.7852543	-0.47775195	0.220470668	2.16696377	0.030237617	0.101616606	0.47775195
ENSMUSG00000020609	Apob	238055	protein_coding	954.8512561	-0.477753101	0.216150705	2.21027778	0.02708589	0.092992945	0.477753101
ENSMUSG00000028750	Pla2g2c	18781	protein_coding	48.28758472	0.477786207	0.174571124	-2.736914303	0.006201845	0.028888694	0.477786207
ENSMUSG00000071042	Rasgrp3	240168	protein_coding	3.002817608	-0.477833185	1.482943929	0.322219321	0.747286549	1	0.477833185
ENSMUSG00000027523	Gnas	14683	protein_coding	257.8998902	0.478133337	0.23056712	-2.073727331	0.038104641	0.121338228	0.478133337
ENSMUSG00000031197	Vbp1	22327	protein_coding	393.2624129	0.47823885	0.132074961	-3.620965283	0.000293506	0.002235681	0.47823885
ENSMUSG00000101304	Plet1os	69631	lncRNA	22.52912879	-0.478255816	0.368241909	1.298754445	0.194028213	0.392717478	0.478255816
ENSMUSG00000026575	Nme7	171567	protein_coding	126.9604432	0.478366609	0.125008356	-3.826677062	0.000129885	0.001087135	0.478366609
ENSMUSG00000034786	Gpsm3	106512	protein_coding	6.403999712	0.478485687	0.579144155	-0.826194451	0.408693796	0.629272248	0.478485687
ENSMUSG00000000792	Slc5a5	114479	protein_coding	6.212422916	0.478489045	0.529059428	-0.90441455	0.365775626	0.588390676	0.478489045
ENSMUSG00000091712	Sec14l5	665119	protein_coding	1.645349379	0.478491789	1.629939457	-0.293564149	0.769090974	1	0.478491789
ENSMUSG00000020988	L2hgdh	217666	protein_coding	346.3587161	0.478767778	0.097826875	-4.89403118	9.88E-07	1.37E-05	0.478767778
ENSMUSG00000028743	Akr7a5	110198	protein_coding	512.7153375	-0.47877837	0.107518716	4.452977017	8.47E-06	9.66E-05	0.47877837
ENSMUSG00000031073	Fgf15	14170	protein_coding	1.388515282	0.47895882	2.053376696	-0.23325424	0.81556399	1	0.47895882
ENSMUSG00000039701	Usp53	99526	protein_coding	1356.181103	-0.478967936	0.118930232	4.027301777	5.64E-05	0.000519649	0.478967936
ENSMUSG00000113186	A330076C08Rik	100503592	lncRNA	2.605007127	-0.479451038	1.954438008	0.245314016	0.8062133	1	0.479451038
ENSMUSG00000039738	Slx4	52864	protein_coding	145.4503025	0.479459139	0.105653697	-4.538025214	5.68E-06	6.71E-05	0.479459139
ENSMUSG00000022844	Pdia5	72599	protein_coding	805.3038839	0.479466259	0.186996644	-2.564036706	0.01034626	0.043750335	0.479466259
ENSMUSG00000049313	Sorl1	20660	protein_coding	2222.236355	-0.479479594	0.094714411	5.062372128	4.14E-07	6.20E-06	0.479479594
ENSMUSG00000031628	Casp3	12367	protein_coding	719.6075695	-0.479510148	0.075739043	6.331082714	2.43E-10	5.80E-09	0.479510148
ENSMUSG00000014959	Gorasp2	70231	protein_coding	1426.67588	0.479547706	0.090302778	-5.310442434	1.09E-07	1.79E-06	0.479547706
ENSMUSG00000057766	Ankrd29	225187	protein_coding	7.423794955	-0.479647614	0.456377999	1.050987591	0.293264289	0.512561987	0.479647614
ENSMUSG00000061474	Mrps36	66128	protein_coding	234.5395077	0.479652838	0.135871477	-3.530195215	0.000415253	0.003030927	0.479652838
ENSMUSG00000024590	Lmnb1	16906	protein_coding	653.4535611	0.479803974	0.120608372	-3.978197918	6.94E-05	0.000626283	0.479803974
ENSMUSG00000024190	Dusp1	19252	protein_coding	2915.3634	-0.479814061	0.182811725	2.624635055	0.008674188	0.037986091	0.479814061
ENSMUSG00000052396	Mogat2	233549	protein_coding	54.99598574	0.47997555	0.334143303	-1.436436243	0.150878246	0.330978129	0.47997555
ENSMUSG00000033213	AA467197	433470	protein_coding	1523.986115	-0.48004957	0.118577752	4.048394928	5.16E-05	0.000479251	0.48004957
ENSMUSG00000024349	Sting1	72512	protein_coding	74.88788078	0.48014115	0.212636612	-2.258036117	0.023943408	0.084474747	0.48014115
ENSMUSG00000065560	Mir148b	724064	miRNA	0.492069034	-0.480228289	1.808031506	0.265608363	0.790540844	1	0.480228289
ENSMUSG00000000365	Rnf17	30054	protein_coding	3.619223913	0.480237868	1.193597199	-0.402345002	0.687430139	1	0.480237868
ENSMUSG00000040852	Plekhh2	213556	protein_coding	37.65888765	0.48027151	0.203173325	-2.363851214	0.018086075	0.067726402	0.48027151
ENSMUSG00000020718	Polg2	50776	protein_coding	139.369675	-0.480325534	0.132208122	3.633101557	0.000280035	0.002143947	0.480325534
ENSMUSG00000035351	Nup37	69736	protein_coding	137.4359806	0.480403289	0.172320748	-2.787843573	0.005306016	0.025598465	0.480403289
ENSMUSG00000002265	Peg3	18616	protein_coding	45.93140291	-0.480404892	0.232770656	2.063855038	0.039031463	0.123598385	0.480404892
ENSMUSG00000069867	Pabpn1l	382035	protein_coding	0.653230925	-0.480472962	1.611291574	0.298191197	0.76555724	1	0.480472962
ENSMUSG00000029554	Mad1l1	17120	protein_coding	243.9778441	0.480524586	0.093599069	-5.133860772	2.84E-07	4.36E-06	0.480524586

ensembl_gene_id	external_gene_name	entrezgene_id	gene_biotype	baseMean	log2FoldChange	lfcSE	stat	pvalue	padj	absLog2FC
ENSMUSG00000025582	Nptx1	18164	protein_coding	6.89709185	-0.480659273	0.629062846	0.764087843	0.444814896	0.662290428	0.480659273
ENSMUSG00000070167	Snora57	118027464	snoRNA	328.7843409	0.480731066	0.338042465	-1.422102593	0.15499647	0.337101502	0.480731066
ENSMUSG00000027332	Ivd	56357	protein_coding	1044.549836	0.48106977	0.084556211	-5.689348709	1.28E-08	2.42E-07	0.48106977
ENSMUSG00000050966	Lin28a	83557	protein_coding	1.768529121	-0.481120496	1.924648259	0.249978402	0.802604051	1	0.481120496
ENSMUSG00000065315	Gm26064	115488251	snRNA	0.732790944	0.481223191	1.60003383	-0.300758135	0.763598934	1	0.481223191
ENSMUSG00000071202	Ccdc78	381077	protein_coding	3.802585954	0.481294859	0.634230414	-0.758864362	0.447933699	1	0.481294859
ENSMUSG00000023216	Epb42	13828	protein_coding	2.858335945	0.481298027	1.09869857	-0.43806194	0.661341383	1	0.481298027
ENSMUSG00000020895	Tmem107	66910	protein_coding	82.35769612	0.481310817	0.210307009	-2.288610445	0.022101997	0.079328922	0.481310817
ENSMUSG00000049001	Ndnf	68169	protein_coding	28.73696684	0.481339897	0.272142614	-1.768704612	0.076943182	0.204447258	0.481339897
ENSMUSG00000027364	Usp50	75083	protein_coding	3.753573894	-0.481346922	0.760428959	0.632993939	0.526737601	1	0.481346922
ENSMUSG00000062124	Defb45	433490	protein_coding	17.4900985	-0.481370283	0.451125503	1.067042941	0.285952446	0.504402765	0.481370283
ENSMUSG00000102742	Pcdhga11	93723	protein_coding	2.676218197	0.481396978	0.740827576	-0.649809744	0.515815124	1	0.481396978
ENSMUSG00000049109	Themis	210757	protein_coding	4.37431913	0.481545056	0.889796094	-0.541185851	0.588379489	1	0.481545056
ENSMUSG00000078502	Zfp268	433801	protein_coding	23.52489724	-0.481557533	0.302265882	1.593158744	0.111124584	0.26676599	0.481557533
ENSMUSG00000021514	Zfp369	170936	protein_coding	189.8545434	-0.481573082	0.098764872	4.875955115	1.08E-06	1.48E-05	0.481573082
ENSMUSG00000029446	Psph	100678	protein_coding	80.84162608	0.481605765	0.138805234	-3.469651334	0.000521134	0.003676212	0.481605765
ENSMUSG00000027359	Slc27a2	26458	protein_coding	62.16010087	0.481636847	0.155970455	-3.088000537	0.002015081	0.011401366	0.481636847
ENSMUSG00000028212	Ccne2	12448	protein_coding	98.33362052	0.481776503	0.159655153	-3.01760697	0.002547791	0.013849316	0.481776503
ENSMUSG00000042660	Wdr55	67936	protein_coding	183.8828718	-0.48195423	0.120649732	3.994656454	6.48E-05	0.000589808	0.48195423
ENSMUSG00000026880	Stom	13830	protein_coding	409.2167108	-0.482105248	0.205640535	2.344407671	0.019057328	0.070692483	0.482105248
ENSMUSG00000091184	Gm17059	102640356	lncRNA	2.299271622	-0.482209316	0.79072597	0.609831135	0.541973675	1	0.482209316
ENSMUSG00000027339	Rassf2	215653	protein_coding	12.39395348	0.482290007	0.483892961	-0.996687379	0.318916278	0.5402916	0.482290007
ENSMUSG00000057123	Gja5	14613	protein_coding	4.18170667	0.482520714	0.971187189	-0.496835954	0.619304739	1	0.482520714
ENSMUSG00000046434	Hnrnpa1	15382	protein_coding	1534.742962	-0.48266035	0.103080275	4.682373537	2.84E-06	3.55E-05	0.48266035
ENSMUSG00000038521	C1s1	50908	protein_coding	1.781254396	0.482696432	1.120191405	-0.430905317	0.666537217	1	0.482696432
ENSMUSG00000022621	Rabl2	68708	protein_coding	57.10279319	0.482708549	0.133085941	-3.627043882	0.000286685	0.00218877	0.482708549
ENSMUSG00000050157	Gm867	333670	protein_coding	3.275827834	-0.483007423	0.794946766	0.607597193	0.543454663	1	0.483007423
ENSMUSG00000100738	2010106C02Rik	72104	lncRNA	6.433518297	-0.483116358	0.523936706	0.922089161	0.35648207	0.579378227	0.483116358
ENSMUSG00000048385	Scrt1	170729	protein_coding	1.876992989	-0.483231204	1.51272964	0.319443205	0.749390453	1	0.483231204
ENSMUSG00000064210	Ano6	105722	protein_coding	366.868342	0.483262743	0.094502495	-5.113756427	3.16E-07	4.82E-06	0.483262743
ENSMUSG00000060224	Pyroxd2	74580	protein_coding	93.14470477	-0.48340776	0.12043487	4.013852151	5.97E-05	0.000548043	0.48340776
ENSMUSG00000037772	Mrpl23	19935	protein_coding	82.66838866	-0.483635253	0.169971732	2.845386387	0.004435758	0.022037337	0.483635253
ENSMUSG00000047854	Stx19	68159	protein_coding	26.75244992	0.483800063	0.255575921	-1.89297983	0.058360552	0.167349566	0.483800063
ENSMUSG00000017697	Ada	11486	protein_coding	97.23261115	0.483890981	0.133396241	-3.62747088	0.000286211	0.002186165	0.483890981
ENSMUSG00000087881	Gm22442	115487906	scaRNA	559.6408806	-0.4839547	0.122259443	3.958423901	7.54E-05	0.000673467	0.4839547
ENSMUSG00000089371	Mir1938	100316693	miRNA	44.54097687	-0.483955685	0.301242915	1.606529684	0.108157578	0.261778433	0.483955685
ENSMUSG00000109324	Prmt1	15469	protein_coding	320.0149496	0.484223374	0.091352192	-5.300621309	1.15E-07	1.88E-06	0.484223374
ENSMUSG00000087113	Gm11714	108167930	lncRNA	0.587287972	-0.484460529	1.798650965	0.269346604	0.787662972	1	0.484460529
ENSMUSG00000022753	Tmem30c	71027	protein_coding	2.492230077	0.484464144	1.511035571	-0.320617299	0.748500426	1	0.484464144

Supplementary Table 1

ensembl_gene_id	external_gene_name	entrezgene_id	gene_biotype	baseMean	log2FoldChange	lfcSE	stat	pvalue	padj	absLog2FC
ENSMUSG00000025396	Hsd17b6	27400	protein_coding	0.961759712	0.484485058	1.816476746	-0.266716906	0.789687136	1	0.484485058
ENSMUSG00000117328	4930474M22Rik	74917	lncRNA	1.54601673	0.484557718	1.105204393	-0.438432675	0.661072665	1	0.484557718
ENSMUSG00000086847	Tbx3os2	433944	lncRNA	1.960580594	-0.48465604	1.493028233	0.324612776	0.745474156	1	0.48465604
ENSMUSG00000028497	Hacd4	66775	protein_coding	31.23029906	-0.484771825	0.194948443	2.486666818	0.01289461	0.05182755	0.484771825
ENSMUSG00000108622	Gm36864	102640913	protein_coding	5.782901445	0.484884374	0.49056611	-0.988418002	0.322947974	0.544366934	0.484884374
ENSMUSG00000090946	Ccdc71l	72123	protein_coding	1240.427268	0.484978735	0.149819092	-3.237095681	0.001207529	0.007435174	0.484978735
ENSMUSG00000049184	Purg	75029	protein_coding	16.54499121	-0.485040841	0.346739066	1.398864126	0.161853732	0.347033567	0.485040841
ENSMUSG00000063856	Gpx1	14775	protein_coding	2230.788943	-0.485087889	0.096727535	5.014992769	5.30E-07	7.79E-06	0.485087889
ENSMUSG00000014837	4931428F04Rik	74356	protein_coding	30.51017272	0.485110742	0.244524813	-1.98389168	0.047267917	0.142911457	0.485110742
ENSMUSG00000074028	Slc22a13	102570	protein_coding	1.088063369	0.485131108	1.825286708	-0.26578351	0.790405944	1	0.485131108
ENSMUSG00000042485	Mustn1	66175	protein_coding	3.311059811	0.485344954	0.657576593	-0.738081251	0.460465076	1	0.485344954
ENSMUSG00000028093	Acp6	66659	protein_coding	369.3779006	-0.485462568	0.080826034	6.006264876	1.90E-09	4.05E-08	0.485462568
ENSMUSG00000048216	Gpr85	64450	protein_coding	6.226617011	0.485671811	0.440665407	-1.102132828	0.27040393	0.485744642	0.485671811
ENSMUSG00000071656	Lrrn4cl	68852	protein_coding	1.568358496	0.485921008	1.08083425	-0.44957958	0.653013615	1	0.485921008
ENSMUSG00000030718	Ppme1	72590	protein_coding	597.5768195	0.485979681	0.063275271	-7.680404565	1.59E-14	5.57E-13	0.485979681
ENSMUSG00000025665	Rps6ka6	67071	protein_coding	48.88601925	0.486045934	0.195527473	-2.485819137	0.012925364	0.051921541	0.486045934
ENSMUSG00000024308	Tapbp	21356	protein_coding	1700.502227	-0.486059838	0.060563259	8.025655301	1.01E-15	3.93E-14	0.486059838
ENSMUSG00000048911	Rnf24	51902	protein_coding	50.98819505	0.486061153	0.266053546	-1.826929807	0.067710324	0.186423523	0.486061153
ENSMUSG00000027459	Fam110a	73847	protein_coding	126.560225	0.486156588	0.110142205	-4.413899178	1.02E-05	0.000113436	0.486156588
ENSMUSG00000001521	Tulp3	22158	protein_coding	27.00002826	0.486286036	0.266252338	-1.826410386	0.067788469	0.186607563	0.486286036
ENSMUSG00000021260	Hhipl1	214305	protein_coding	2.820235089	0.486330355	2.125476706	-0.228810014	0.819016583	1	0.486330355
ENSMUSG00000021200	Asb2	65256	protein_coding	12.23886726	0.486525386	0.4842377	-1.004724302	0.315029623	0.535990818	0.486525386
ENSMUSG00000095362	Gm14325	329575	protein_coding	45.55378092	-0.486725087	0.272479136	1.786283874	0.074053307	0.198945226	0.486725087
ENSMUSG00000048796	Cyb561d1	72023	protein_coding	275.3524968	-0.486933506	0.105552564	4.613184996	3.97E-06	4.86E-05	0.486933506
ENSMUSG00000020432	Tcn2	21452	protein_coding	1024.033092	-0.486957032	0.084343689	5.773485095	7.76E-09	1.52E-07	0.486957032
ENSMUSG00000031342	Gpm6b	14758	protein_coding	6.914623785	-0.486996575	0.632443708	0.770023591	0.441285899	0.659125086	0.486996575
ENSMUSG00000019899	Lama2	16773	protein_coding	3.657374594	-0.487244144	1.290928544	0.377436959	0.705848905	1	0.487244144
ENSMUSG00000050822	Slc29a4	243328	protein_coding	13.26677078	-0.487267894	0.373573129	1.304344064	0.192116298	0.389807821	0.487267894
ENSMUSG00000032754	Slc8b1	170756	protein_coding	522.0735575	-0.487322681	0.137148921	3.553237447	0.000380521	0.002809084	0.487322681
ENSMUSG00000079018	Ly6c1	17067	protein_coding	13.48075454	0.48746065	0.486478428	-1.002019046	0.316334394	0.537332461	0.48746065
ENSMUSG00000019763	Rmnd1	66084	protein_coding	213.6411049	-0.487500825	0.093907547	5.191284858	2.09E-07	3.25E-06	0.487500825
ENSMUSG00000063455	D630045J12Rik	330286	protein_coding	20.76930276	0.487558809	0.386132985	-1.262670705	0.206707546	0.409464624	0.487558809
ENSMUSG00000029765	Plxna4	243743	protein_coding	5.089643079	0.487563046	1.557665067	-0.313008911	0.754273892	0.876856731	0.487563046
ENSMUSG00000074912	Gm14207	100043609	lncRNA	32.50199648	-0.487587963	0.210683402	2.314315981	0.020650396	0.075180916	0.487587963
ENSMUSG00000023032	Slc4a8	59033	protein_coding	31.15778632	0.487591769	0.336037729	-1.451003047	0.146779012	0.324596932	0.487591769
ENSMUSG00000055850	Rnf181	66510	protein_coding	833.3842525	0.487697626	0.074239391	-6.569256809	5.06E-11	1.33E-09	0.487697626
ENSMUSG00000117924	Tmem223	66836	protein_coding	127.5750152	-0.487772079	0.240202118	2.030673515	0.042288125	0.13128129	0.487772079
ENSMUSG00000028047	Thbs3	21827	protein_coding	13.71837464	-0.487785561	0.351896311	1.386162758	0.165697211	0.352762472	0.487785561
ENSMUSG00000103897	Pcdhga8	93716	protein_coding	4.337395447	-0.487802845	0.552950631	0.882181551	0.377678637	1	0.487802845

ensembl_gene_id	external_gene_name	entrezgene_id	gene_biotype	baseMean	log2FoldChange	lfcSE	stat	pvalue	padj	absLog2FC
ENSMUSG00000028671	Gale	74246	protein_coding	3837.840522	0.487860425	0.082262863	-5.930506265	3.02E-09	6.28E-08	0.487860425
ENSMUSG00000113035	5830428M24Rik	76062	lncRNA	19.02143589	-0.487921157	0.255449173	1.910051816	0.056126542	0.162609424	0.487921157
ENSMUSG00000030352	Tspan9	109246	protein_coding	109.7059206	0.487955868	0.16868713	-2.892668032	0.003819848	0.019469435	0.487955868
ENSMUSG00000026023	Cdk15	271697	protein_coding	1.255966403	0.488097818	1.700515199	-0.287029377	0.774089827	1	0.488097818
ENSMUSG00000032009	Sesn3	75747	protein_coding	389.4746678	0.48840629	0.093971356	-5.197395387	2.02E-07	3.16E-06	0.48840629
ENSMUSG00000028289	Epha7	13841	protein_coding	18.53793495	0.488552136	0.354313763	-1.378868637	0.16793526	0.356436387	0.488552136
ENSMUSG00000040339	Fam102b	329739	protein_coding	916.8670228	0.488600574	0.089521805	-5.457894601	4.82E-08	8.28E-07	0.488600574
ENSMUSG00000089774	Slc5a3	53881	protein_coding	1060.367175	-0.488684188	0.133902133	3.649562395	0.000262687	0.002018623	0.488684188
ENSMUSG00000032013	Trim29	72169	protein_coding	7.933615581	-0.488965175	0.428908465	1.140022207	0.25427705	0.466951438	0.488965175
ENSMUSG00000030512	Snrpa1	68981	protein_coding	237.7426247	0.48901604	0.11865661	-4.12127096	3.77E-05	0.000364515	0.48901604
ENSMUSG00000026616	Cr2	12902	protein_coding	1.96562298	-0.489086351	1.093897848	0.447104226	0.654799808	1	0.489086351
ENSMUSG00000033967	Rnf225	381845	protein_coding	7.824438053	0.489095891	0.57714751	-0.847436543	0.396751843	0.618855346	0.489095891
ENSMUSG00000097115	1810019N24Rik	69086	lncRNA	6.072506162	0.489097189	0.520031533	-0.940514485	0.346953723	0.568637732	0.489097189
ENSMUSG00000027811	4930579G24Rik	75939	protein_coding	20.06394072	0.48915868	0.302992847	-1.614423194	0.106435664	0.259015408	0.48915868
ENSMUSG00000026409	Pfkfb2	18640	protein_coding	370.7094317	0.489187412	0.060204988	-8.125363506	4.46E-16	1.79E-14	0.489187412
ENSMUSG00000079235	Ccdc13	100502861	protein_coding	3.5687713	0.489531188	0.820206968	-0.596838612	0.550615136	1	0.489531188
ENSMUSG00000032902	Slc16a1	20501	protein_coding	4504.265076	-0.489836068	0.141781663	3.454861911	0.000550575	0.003837987	0.489836068
ENSMUSG00000042750	Bex2	12069	protein_coding	26.82692195	0.489982418	0.224695553	-2.180650271	0.029209294	0.098824478	0.489982418
ENSMUSG00000050105	Fam110d	72690	protein_coding	1.072019769	-0.490009715	1.214099302	0.403599371	0.686507348	1	0.490009715
ENSMUSG00000033029	1700088E04Rik	27660	protein_coding	11.493256	-0.490215107	0.429200072	1.142159891	0.253387564	0.465869766	0.490215107
ENSMUSG00000043753	Dmrta1	242523	protein_coding	8.914909272	0.490367274	0.461830074	-1.061791559	0.288330323	0.50718864	0.490367274
ENSMUSG00000030329	Pianp	319352	protein_coding	3.96666673	-0.490419903	0.989710114	0.495518735	0.620234001	1	0.490419903
ENSMUSG00000090840	Tex54	74307	protein_coding	2.966724422	0.490433924	0.767002937	-0.639415966	0.522552365	1	0.490433924
ENSMUSG00000031506	Ptpn7	320139	protein_coding	8.531459135	0.490440165	0.768926293	-0.637824677	0.523587814	0.722169631	0.490440165
ENSMUSG00000029810	Tmem176b	65963	protein_coding	636.9505517	0.490539803	0.107296192	-4.571828639	4.83E-06	5.79E-05	0.490539803
ENSMUSG00000021464	Ror2	26564	protein_coding	100.8057168	0.4905476	0.151402864	-3.240015333	0.001195233	0.007373206	0.4905476
ENSMUSG00000005803	Sqor	59010	protein_coding	5734.612306	-0.490602079	0.082239021	5.96556325	2.44E-09	5.14E-08	0.490602079
ENSMUSG00000098922	Mir8106	102465896	miRNA	0.82024253	0.490629303	1.231069304	-0.398539141	0.690232812	1	0.490629303
ENSMUSG00000051984	Sec31b	240667	protein_coding	14.85243918	-0.490653356	0.376285026	1.303940691	0.192253805	0.389986406	0.490653356
ENSMUSG00000049811	Fam161a	73873	protein_coding	83.1265319	-0.490754942	0.182653534	2.686807803	0.007213845	0.03273686	0.490754942
ENSMUSG00000022747	St3gal6	54613	protein_coding	1031.774233	-0.490825459	0.261068993	1.880060338	0.060099855	0.170971405	0.490825459
ENSMUSG00000024254	Abcg8	67470	protein_coding	21.95050198	-0.490842949	0.391853799	1.252617557	0.210344922	0.414416136	0.490842949
ENSMUSG00000073293	Nudt10	102954	protein_coding	17.08424415	0.49095158	0.268911452	-1.825699781	0.067895496	0.186808768	0.49095158
ENSMUSG00000062826	Ces2f	71903	protein_coding	4.964370409	-0.490990165	0.62113169	0.790476759	0.42924939	0.648250976	0.490990165
ENSMUSG00000042757	Tmem108	81907	protein_coding	7.748625609	0.49102474	0.891932155	-0.550518038	0.581964108	0.765030035	0.49102474
ENSMUSG00000031273	Col4a6	94216	protein_coding	4.291506031	0.491208113	1.301083334	-0.37753778	0.705773994	1	0.491208113
ENSMUSG00000002289	Angptl4	57875	protein_coding	24.40689371	-0.49129389	0.425166142	1.1555339	0.247871859	0.460059583	0.49129389
ENSMUSG00000030693	Klk10	69540	protein_coding	1.574461976	0.491312364	1.364086994	-0.360176709	0.718714991	1	0.491312364
ENSMUSG00000044162	Tnip3	414084	protein_coding	335.0642948	0.49133867	0.241406994	-2.035312492	0.04181944	0.130180251	0.49133867

ensembl_gene_id	external_gene_name	entrezgene_id	gene_biotype	baseMean	log2FoldChange	lfcSE	stat	pvalue	padj	absLog2FC
ENSMUSG00000026511	Srp9	27058	protein_coding	595.5305796	0.491421245	0.081637236	-6.019572286	1.75E-09	3.74E-08	0.491421245
ENSMUSG00000056531	Ccdc18	73254	protein_coding	74.67031896	0.491464478	0.178383444	-2.755101406	0.005867395	0.027715628	0.491464478
ENSMUSG00000053318	Slamf8	74748	protein_coding	1.667832329	0.491643796	1.460769454	-0.336564949	0.736444883	1	0.491643796
ENSMUSG00000062901	Klhl24	75785	protein_coding	777.3567233	-0.491702114	0.218608381	2.249237248	0.024497406	0.08602548	0.491702114
ENSMUSG00000070972	Dnajc25	72429	protein_coding	110.4155126	0.491775293	0.118271064	-4.158035585	3.21E-05	0.000316096	0.491775293
ENSMUSG00000078813	Leng1	69757	protein_coding	211.3720974	0.491847093	0.10414543	-4.722694908	2.33E-06	2.98E-05	0.491847093
ENSMUSG00000083011	Gm12816	433745	processed_pseudogene	0.733314262	0.492109242	1.87727104	-0.262140752	0.793212929	1	0.492109242
ENSMUSG00000033730	Egr3	13655	protein_coding	7.584895595	0.492161909	0.625336452	-0.787035375	0.431261158	0.649597228	0.492161909
ENSMUSG00000049598	Vsig8	240916	protein_coding	70.22733458	0.492267962	0.616727892	-0.798193122	0.424758428	0.64425321	0.492267962
ENSMUSG00000006307	Kmt2b	75410	protein_coding	673.4690644	0.49232009	0.199119716	-2.472482878	0.013417814	0.05352217	0.49232009
ENSMUSG00000096687	Mfsd4b4	213332	protein_coding	26.24642192	0.492484066	0.232843189	-2.115088995	0.034422363	0.112032105	0.492484066
ENSMUSG00000111761	9230112J17Rik	100504561	lncRNA	8.442196576	-0.49250319	0.419734514	1.173368341	0.240648127	0.451187917	0.49250319
ENSMUSG00000021884	Hacl1	56794	protein_coding	15.14609076	-0.492670701	0.361638386	1.362329664	0.17309387	0.363688435	0.492670701
ENSMUSG00000032827	Ppp1r9a	243725	protein_coding	539.3770863	-0.492674421	0.097992935	5.027652464	4.97E-07	7.34E-06	0.492674421
ENSMUSG00000089308	Gm24837	115490071	snoRNA	1.428546032	0.492951877	1.14968795	-0.428770152	0.668090503	1	0.492951877
ENSMUSG00000037621	Atoh8	71093	protein_coding	25.32528985	0.493048074	0.260674218	-1.89143398	0.058566436	0.167794262	0.493048074
ENSMUSG00000062591	Tubb4a	22153	protein_coding	2.866452807	0.493257067	1.109467845	-0.44458888	0.656616885	1	0.493257067
ENSMUSG00000037849	Ifi206	102639543	protein_coding	2.404894845	-0.493258781	0.750573158	0.657176154	0.511067657	1	0.493258781
ENSMUSG00000058063	Trim31	224762	protein_coding	1046.417811	0.493320339	0.075622932	-6.523422521	6.87E-11	1.76E-09	0.493320339
ENSMUSG00000015790	Surf1	20930	protein_coding	608.4397266	-0.493373869	0.061400355	8.035358559	9.33E-16	3.66E-14	0.493373869
ENSMUSG00000002835	Chaf1a	27221	protein_coding	170.3902762	0.493400382	0.140680216	-3.507247831	0.000452767	0.003259372	0.493400382
ENSMUSG00000026830	Ermn	77767	protein_coding	8.039979152	0.493474007	0.38478242	-1.282475449	0.199675912	0.40075936	0.493474007
ENSMUSG00000090272	Mndal	100040462	protein_coding	6.047281846	-0.493502402	0.613860073	0.803933051	0.421435641	0.641211258	0.493502402
ENSMUSG00000021115	Vrk1	22367	protein_coding	203.4709154	0.493533219	0.14599914	-3.380384431	0.000723845	0.004819991	0.493533219
ENSMUSG00000057143	Trim12c	319236	protein_coding	217.9759074	-0.493748641	0.126781963	3.894470715	9.84E-05	0.000848668	0.493748641
ENSMUSG00000038037	Socs1	12703	protein_coding	42.65635143	0.493836298	0.330552534	-1.493972206	0.135182884	0.306271113	0.493836298
ENSMUSG00000029602	Rasal1	19415	protein_coding	5.245952773	-0.493846566	0.70967343	0.695878619	0.486504848	0.695624496	0.493846566
ENSMUSG00000042848	Vmn1r226	171225	protein_coding	0.89118345	0.493953082	1.956466402	-0.252472049	0.800676216	1	0.493953082
ENSMUSG00000025145	Lrrc45	217366	protein_coding	471.9088699	0.494027343	0.089415061	-5.525102127	3.29E-08	5.82E-07	0.494027343
ENSMUSG00000027713	1810062G17Rik	72282	protein_coding	10.34280748	0.494039467	0.351601065	-1.405113679	0.159987477	0.344285818	0.494039467
ENSMUSG00000002489	Tiam1	21844	protein_coding	219.6785982	0.494135786	0.154320695	-3.202005962	0.001364742	0.00824336	0.494135786
ENSMUSG00000049649	Gpr3	14748	protein_coding	3.915725966	-0.494400093	0.830151434	0.595554103	0.551473145	1	0.494400093
ENSMUSG00000036231	Agr3	403205	protein_coding	13.4058293	-0.494478272	0.29406428	1.681531234	0.092659775	0.233580144	0.494478272
ENSMUSG00000053367	Epp13	627821	protein_coding	2.23436834	0.494518344	2.409977282	-0.205196268	0.837418785	1	0.494518344
ENSMUSG00000057596	Trim30d	209387	protein_coding	228.6143693	-0.494571577	0.17055255	2.899819298	0.003733778	0.019107412	0.494571577
ENSMUSG00000097084	Foxl1	14241	protein_coding	0.980516654	0.49463165	1.967522297	-0.251398244	0.801506226	1	0.49463165
ENSMUSG00000019312	Grb7	14786	protein_coding	750.9292366	-0.494795458	0.105278029	4.699892893	2.60E-06	3.29E-05	0.494795458
ENSMUSG00000029094	Afap1	70292	protein_coding	161.0428446	0.494842644	0.11216687	-4.411664918	1.03E-05	0.000114459	0.494842644
ENSMUSG00000002020	Ltbp2	16997	protein_coding	6.976178037	-0.494914792	0.817507374	0.605394897	0.544916641	0.736964526	0.494914792

Supplementary Table 1

ensembl_gene_id	external_gene_name	entrezgene_id	gene_biotype	baseMean	log2FoldChange	lfcSE	stat	pvalue	padj	absLog2FC
ENSMUSG00000030577	Cd22	12483	protein_coding	3.064228491	0.494931743	1.329231282	-0.372344339	0.70963649	1	0.494931743
ENSMUSG00000074671	Tspyl3	241732	protein_coding	6.001366393	0.495405745	0.46706647	-1.060675036	0.288837609	0.507809662	0.495405745
ENSMUSG00000085457	1110046J04Rik	68808	lncRNA	5.467816483	-0.495462571	0.50068463	0.989570164	0.322384259	0.54358318	0.495462571
ENSMUSG00000097429	Gm26520	105244630	lncRNA	1.309208534	-0.495588692	1.147208415	0.431995342	0.665744799	1	0.495588692
ENSMUSG00000027399	Il1a	16175	protein_coding	2.196080359	0.495667151	1.0403055	-0.476463068	0.633744512	1	0.495667151
ENSMUSG00000059060	Rad51b	19363	protein_coding	25.72174688	0.495773749	0.258415262	-1.91851575	0.055045647	0.160039404	0.495773749
ENSMUSG00000030472	Ceacam18	72431	protein_coding	72.69657171	-0.495887862	0.16165436	3.067581119	0.002157989	0.012051751	0.495887862
ENSMUSG00000064288	H4c12	319160	protein_coding	969.0656754	0.495966229	0.178587736	-2.77715726	0.005483664	0.026283536	0.495966229
ENSMUSG00000061414	Cracr2a	381812	protein_coding	733.2993923	-0.496050193	0.178892727	2.772891897	0.005556057	0.026525797	0.496050193
ENSMUSG00000025647	Shisa5	66940	protein_coding	1952.372672	-0.496146153	0.076101757	6.519509874	7.05E-11	1.80E-09	0.496146153
ENSMUSG00000042632	Pla2g6	53357	protein_coding	153.4175473	0.49619889	0.149508036	-3.318877736	0.0009038	0.005843912	0.49619889
ENSMUSG00000114284	9630015K15Rik	78569	lncRNA	1.849747	-0.496377971	1.427331044	0.347766535	0.728015524	1	0.496377971
ENSMUSG00000030287	Itpr2	16439	protein_coding	743.7250978	-0.496423785	0.094180808	5.270965433	1.36E-07	2.19E-06	0.496423785
ENSMUSG00000032607	Amt	434437	protein_coding	213.9268687	-0.496436176	0.119198579	4.164782681	3.12E-05	0.000307626	0.496436176
ENSMUSG00000040606	Kazn	71529	protein_coding	121.9965725	-0.496478459	0.14235934	3.487501841	0.000487556	0.003474964	0.496478459
ENSMUSG00000034336	Ina	226180	protein_coding	1.602556247	-0.496494529	1.184372224	0.419204806	0.67506646	1	0.496494529
ENSMUSG00000000632	Sez6	20370	protein_coding	2.45980291	0.496646017	1.517420108	-0.32729632	0.743443776	1	0.496646017
ENSMUSG00000023243	Kcnk5	16529	protein_coding	1110.066698	-0.496678342	0.11260101	4.410958157	1.03E-05	0.000114755	0.496678342
ENSMUSG00000010110	Stx5a	56389	protein_coding	993.4831042	0.496848208	0.05557355	-8.940371921	3.88E-19	1.89E-17	0.496848208
ENSMUSG00000037254	Itih2	16425	protein_coding	109.3908525	0.496957881	0.416872588	-1.19210976	0.233218211	0.442685693	0.496957881
ENSMUSG00000074220	Zfp382	233060	protein_coding	3.172095675	0.496961002	0.577999326	-0.859795125	0.389901987	1	0.496961002
ENSMUSG00000033533	Acsm1	117147	protein_coding	1.026291417	0.4970555	2.008279129	-0.247503195	0.804518819	1	0.4970555
ENSMUSG00000026879	Gsn	227753	protein_coding	2169.49228	-0.497172228	0.197087669	2.522594293	0.011649271	0.048021984	0.497172228
ENSMUSG00000073792	Alg6	320438	protein_coding	248.7222711	0.497206612	0.1495832	-3.323946882	0.000887531	0.005758032	0.497206612
ENSMUSG00000051855	Mest	17294	protein_coding	8.163757029	-0.497244424	0.422616268	1.176586095	0.239360751	0.449590928	0.497244424
ENSMUSG00000078157	Fem1al	216622	protein_coding	25.28884062	-0.497317549	0.305442701	1.628186061	0.103485439	0.253780598	0.497317549
ENSMUSG00000054679	Srsf12	272009	protein_coding	1.45040971	0.49745976	1.298308031	-0.383160043	0.701601103	1	0.49745976
ENSMUSG00000097415	AU020206	108167440	lncRNA	81.84781693	0.497592225	0.178336217	-2.790191657	0.005267685	0.025458165	0.497592225
ENSMUSG00000028024	Enpep	13809	protein_coding	77.36476061	-0.497725747	0.407823647	1.220443568	0.22229677	0.429396526	0.497725747
ENSMUSG00000031461	Myom2	17930	protein_coding	4.692608166	0.497745364	1.797217612	-0.276953309	0.781815959	0.890653197	0.497745364
ENSMUSG00000049744	Arhgap15	76117	protein_coding	5.454381744	0.497782971	0.790888131	-0.629397448	0.529088888	0.726903567	0.497782971
ENSMUSG00000112366	C730027H18Rik	319572	lncRNA	1.115101584	0.497794706	2.02113682	-0.246294413	0.805454335	1	0.497794706
ENSMUSG00000117322	6330415G19Rik	320020	lncRNA	6.163317022	0.497820361	0.498193865	-0.999250283	0.317673463	0.539052565	0.497820361
ENSMUSG00000029092	D5Ertd615e	52401	lncRNA	1.138702293	0.49787755	2.022597619	-0.246157489	0.805560322	1	0.49787755
ENSMUSG00000029993	Nfu1	56748	protein_coding	252.8494328	-0.497880726	0.095976973	5.18750186	2.13E-07	3.32E-06	0.497880726
ENSMUSG00000089665	Fcor	100503924	protein_coding	1.728574944	0.497883308	0.926792021	-0.537211474	0.591121547	1	0.497883308
ENSMUSG00000031354	Amelx	11704	protein_coding	1.205089304	0.497897697	1.735718188	-0.286853995	0.774224117	1	0.497897697
ENSMUSG00000066072	Cyp4a10	13117	protein_coding	1.16223802	0.497988766	2.024547027	-0.245975401	0.805701275	1	0.497988766
[truncated: 800,862 more chars]
